# ALDOC promotes non-small cell lung cancer through affecting MYC-mediated UBE2N transcription and regulating Wnt/β-catenin pathway

**DOI:** 10.18632/aging.205038

**Published:** 2023-09-18

**Authors:** Bin Shang, Fengjuan Lu, Shujuan Jiang, Mengmeng Xing, Xinyu Mao, Guanghai Yang, Hao Zhang

**Affiliations:** 1 Department of Thoracic Surgery, Shandong Provincial Hospital Affiliated to Shandong First Medical University, Jinan 250021, Shandong Province, China; 2 Department of Respiratory and Critical Care Medicine, Shandong Provincial Hospital Affiliated to Shandong First Medical University, Jinan 250021, Shandong Province, China; 3 Department of Thoracic Surgery, Union Hospital, Tongji Medical College, Huazhong University of Science and Technology, Wuhan 430022, Hubei Province, China

**Keywords:** NSCLC, ALDOC, UBE2N, transcription, Wnt/β-catenin pathway

## Abstract

Despite advancements in therapeutic options, the overall prognosis for non-small cell lung cancer (NSCLC) remains poor. Therefore, it is crucial to further explore the etiology and targets for novel treatments to effectively manage NSCLC. In this study, immunohistochemistry was used to analyze the expression of aldolase, fructose-bisphosphate C (ALDOC) protein in tumor tissues and adjacent non-malignant tissues from 79 NSCLC patients. Our findings revealed that ALDOC was overexpressed in NSCLC tissues. ALDOC expression was associated with lymph node metastasis, lymphatic metastasis and pathological stage. In addition, Kaplan-Meier analysis showed that higher ALDOC levels were indicative of a poorer prognosis. Additionally, we observed elevated ALDOC mRNA levels in NSCLC cell lines relative to normal cells. To investigate the functional roles of ALDOC, we infected cells with small interfering RNA against ALDOC, which led to attenuated proliferation and migration, as well as ameliorated apoptosis. Furthermore, through our investigations, we discovered that ubiquitin-conjugating enzyme E2N (UBE2N) acts as a downstream factor of ALDOC. ALDOC promoted NSCLC through affecting MYC-mediated UBE2N transcription and regulating the Wnt pathway. More importantly, we found that downregulation of UBE2N or the use of Wnt pathway inhibitor could reverse the promoting effects of ALDOC elevation on NSCLC development *in vitro* and *in vivo*. Based on these findings, our study highlights the potential of ALDOC as a future therapeutic target for NSCLC.

## INTRODUCTION

Lung cancer continues to be the leading cause of cancer-related mortality worldwide. Although the 5-year survival rate for patients with non-small cell lung cancer (NSCLC) is 15%, significant progress has been achieved in the treatment of this disease through targeted therapies that address specific molecular alterations. These advancements primarily focus on targeting mutations in EGFR, BRAF, and MET, as well as translocations involving ALK, ROS1, RET, and NTRK1/2 [1–3]. Treatment with EGFR tyrosine kinase inhibitors (TKIs) has shown improved outcomes in patients with EGFR mutation. However, the long-term effectiveness of these treatments is limited due to the development of resistance [4–6]. Therefore, it is of great significance to investigate the underlying pathogenesis of lung cancer and find more appropriate therapeutic targets.

Glycolysis plays a crucial role in regulating cancer cell behavior. Aldolase, an enzyme involved in glycolysis, is essential for glucose-consuming cells. Apart from its glycolytic function, aldolase also has non-glycolytic roles, including interactions with vacuolar-H+-ATPase and other molecules [7–9]. Aldolase presents in three isoforms: ALDOA, ALDOB, and ALDOC, which are frequently expressed in various tissues such as muscle and liver [10, 11]. ALDOC, also known as zebrin II, is characterized by specific expression in distinct subpopulations of cerebellar Purkinje cells and has been extensively studied in relation to cerebellar compartmentalization [12, 13]. Notably, the expression patterns of ALDOC have been implicated in human malignancies [14]. For instance, overexpression of ALDOC was associated with spheroid formation in colorectal cancer [15]. Additionally, the enrichment of ALDOC has been shown to predict a favorable prognosis in glioblastomas [16]. However, the specific roles of ALDOC in lung cancer and the underlying mechanisms governing its function in cancer remain poorly understood.

Here, we sought to investigate the functional roles and underlying mechanism of ALDOC in lung cancer. Through our analysis, we identified three genes that were regulated by ALDOC. Among these genes, we focused our attention on UBE2N due to its significant association with ALDOC and its known involvement in the development of various human cancers. We also elucidated the mechanism through which ALDOC regulates UBE2N. Overall, our findings highlight the potential of ALDOC as a promising therapeutic target for lung cancer. Further research in this area could lead to the development of novel treatment strategies for improving patient outcomes in lung cancer.

## RESULTS

### ALDOC is strongly expressed in NSCLC

**Figure 1:**
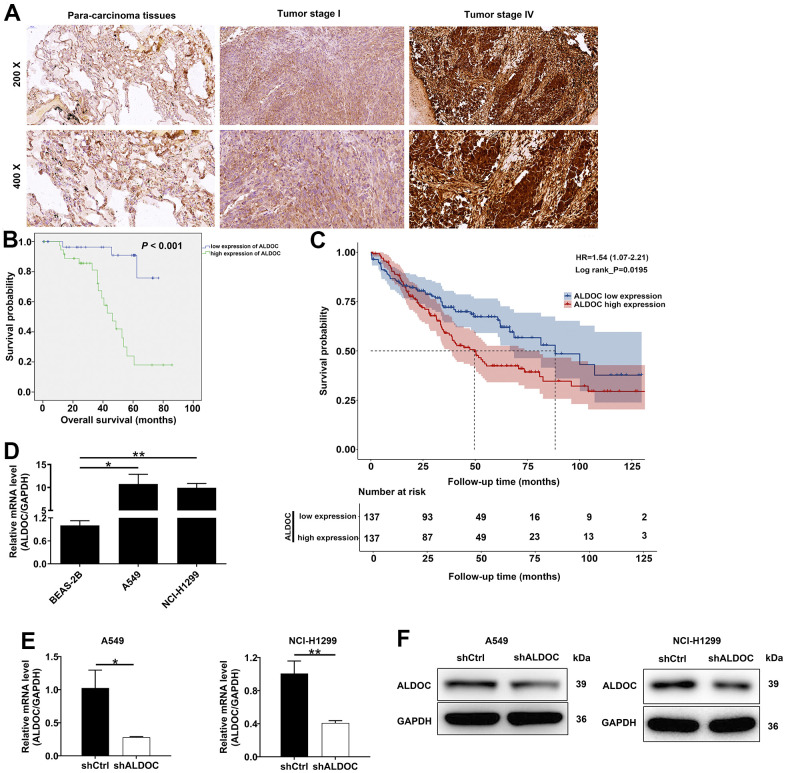
**ALDOC is strongly expressed in NSCLC.** (**A**) ALDOC protein patterns were detected in NSCLC tissues compared with normal samples through IHC staining. (**B**) Kaplan-Meier analysis revealed the relationship between ALDOC level and patients’ overall survival. (**C**) Association between high ALDOC expression and poorer survival outcomes in patients with NSCLC using GEO database. (**D**) ALDOC mRNA levels were quantified in BEAS-2B, A549 and NCI-H1299 cells. (**E**, **F**) After infecting shALDOC and shCtrl, ALDOC mRNA and protein levels in A549 and NCI-H1299 cells were evaluated through qRT-PCR (**E**) and western blot experiments (**F**). ** *P* < 0.01; *P < 0.05.

First, we analyzed ALDOC expression in NSCLC tissues and their adjacent non-malignant tissues using IHC staining. The results indicated that ALDOC expression was significantly increased in tumor tissues; not only that, ALDOC expression was higher in metastatic NSCLC as compared to earlier stages (*P* < 0.001, Figure 1A and Table 1). Notably, ALDOC expression showed a significant positive correlation with lymph node metastasis, lymphatic metastasis, and pathological stage (Tables 2, 3). Additionally, patients with high ALDOC levels exhibited decreased survival probabilities, implying a poor prognosis (Figure 1B). To further investigate the relationship between ALDOC and pathological parameters, we expanded our analysis to larger cohorts, such as the TCGA and GEO databases. The results from these analyses showed a significant association between high ALDOC expression and poorer survival outcomes in patients with NSCLC (Figure 1C). Furthermore, we observed that high ALDOC expression was linked to advanced stage disease, as well as pathologic T, pathologic_N and pathologic_M parameters (Supplementary Table 3). Further analysis of ALDOC expression in cell lines showed elevated levels of ALDOC in both NSCLC cell lines (A549 and NCI-H1299) compared to the normal cell line BEAS-2B (Figure 1D). Taken together, these observations demonstrated that ALDOC was significantly upregulated in NSCLC, suggesting a potential role for ALDOC in its development.

**Table 1:** Expression patterns of ALDOC in lung cancer tissues and para-carcinoma tissues revealed in immunohistochemistry analysis.

**ALDOC expression**	**Tumor tissue**		**Para-carcinoma tissue**		***P* value**
	**Cases**	**Percentage**	**Cases**	**Percentage**	
Low	41	51.9%	82	92.1%	< 0.001
High	38	48.1%	7	7.9%	

**Table 2:** Relationship between ALDOC expression and tumor characteristics in patients with lung cancer.

**Features**	**No. of patients**	**ALDOC expression**		***P* value**
		**Low**	**High**	
All patients	79	41	38	
Age (years)				0.223
< 62	38	17	21	
≥ 62	41	24	17	
Gender				0.843
Male	49	25	24	
Female	30	16	14	
Tumor size				0.441
≤ 3cm	41	23	18	
> 3cm	38	18	20	
Lymph node metastasis				0.031
No	37	24	13	
Yes	42	17	25	
Stage				0.008
I	28	21	7	
II	32	12	20	
III	13	7	6	
IV	6	1	5	
Tumor infiltrate (T)				0.363
T1	41	23	18	
T2	20	11	9	
T3	6	1	5	
T4	12	6	6	
lymphatic metastasis (N)				0.035
N0	37	24	13	
N1	34	14	20	
N2	8	3	5	
Metastasis (M)				0.074
M0	73	40	33	
M1	6	1	5	

**Table 3:** Relationship between ALDOC expression with lymph node metastasis, lymphatic metastasis and stage in patients with lung cancer.

		**ALDOC**
Lymph node metastasis	Spearman correlation	0.244
	Signification (double-tailed)	0.031
	N	79
lymphatic metastasis (N)	Spearman correlation	0.239
	Signification (double-tailed)	0.034
	N	79
Stage	Spearman correlation	0.302
	Signification (double-tailed)	0.007
	N	79

### ALDOC regulates proliferation, migration and apoptosis of NSCLC cells

**Figure 2:**
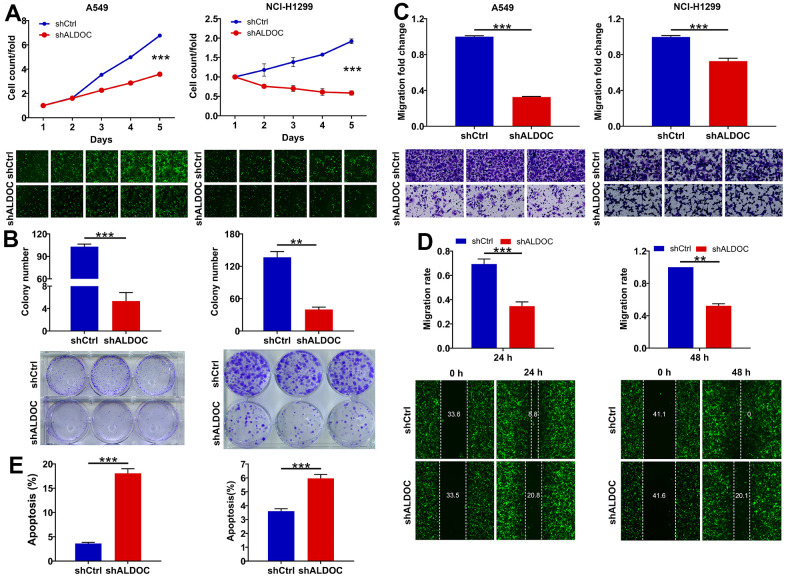
**ALDOC controls proliferation, migration and apoptosis of NSCLC cells.** (**A**, **B**) CCK8 assay (**A**) and colony formation assay (**B**) were performed to assess the alterations in cell proliferation upon knocking down ALDOC in A549 and NCI-H1299 cells. (**C**, **D**) After silencing ALDOC in A549 and NCI-H1299 cells, the abilities to migrate were analyzed through transwell chambers assay (**C**) and wound-healing assay (**D**). (**E**) After silencing ALDOC in A549 and NCI-H1299 cells, the changes in cell apoptosis were tested through flow cytometry detection. *** *P* < 0.001; **P < 0.01.

To further investigate the biological functions of ALDOC in NSCLC, we examined the effects of ALDOC silencing on NSCLC cell phenotypes. By using shRNA targeting ALDOC in A549 and NCI-H1299 cells, we successfully achieved a significant reduction in ALDOC mRNA and protein expression (Figure 1E, 1F). Subsequently, we evaluated the impacts of ALDOC downregulation on cell behaviors. We observed a substantial decrease in cell proliferation upon ALDOC knockdown (*P* < 0.001, Figure 2A). Additionally, ALDOC depletion led to a significant reduction in colony formation ability *in vitro* (*P* < 0.001, Figure 2B). Besides, transwell chamber assays showed a marked decrease in cell migration in A549 and NCI-H1299 cells following ALDOC inhibition, as evidenced by the images and quantification of three independent experiments (*P* < 0.001, Figure 2C). This result was further validated by a wound-healing assay (Figure 2D). Furthermore, flow cytometry analysis revealed that silencing ALDOC promoted apoptosis in both A549 and NCI-H1299 cells (*P* < 0.001, Figure 2E). Collectively, these data provide compelling evidence that ALDOC downregulation suppresses cell viability and migration while enhancing apoptosis in NSCLC cells.

### UBE2N is a downstream target of ALDOC

**Figure 3:**
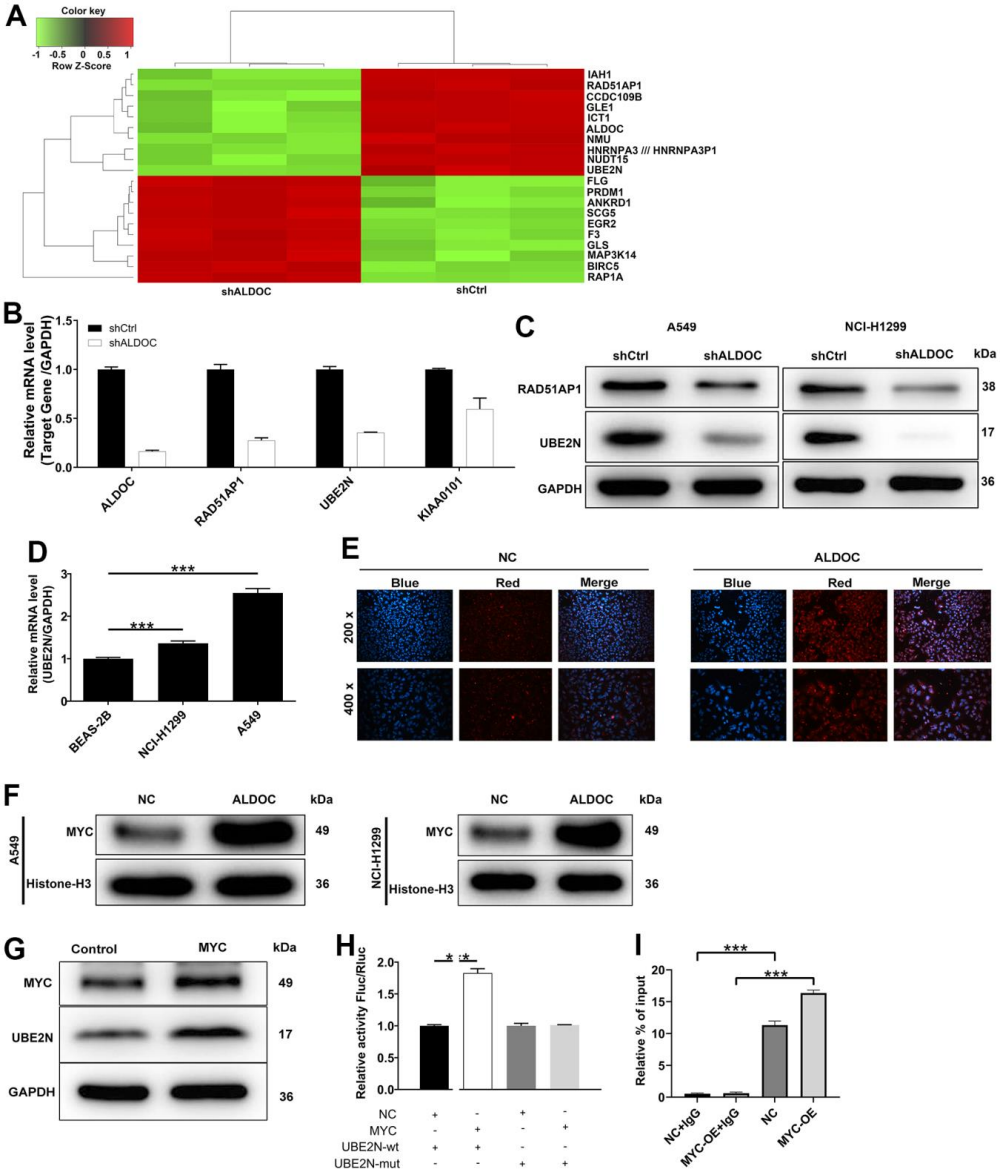
**UBE2N is a downstream target of ALDOC.** (**A**) The heat map of the top 20 DEGs in shALDOC-infected-A549 cells. (**B**) RAD51AP1, UBE2N and KIAA0101 mRNA levels were detected in A549 cells in response to ALDOC knockdown. (**C**) RAD51AP1 and UBE2N protein levels were detected in A549 and NCI-H1299 cells in response to ALDOC knockdown. (**D**) UBE2N mRNA levels were quantified in BEAS-2B, A549 and NCI-H1299 cells. (**E**) Immunofluorescence experiments showed the interaction between ALDOC and MYC. (**F**) Validation of increased MYC levels in the nuclear fraction of NSCLC cell lines after ALDOC overexpression using western blot analysis. (**G**) MYC and UBE2N protein expression were tested after overexpressing MYC in A549 cells. (**H**) Dual luciferase reporter experiment demonstrated that MYC could increase the activity of luciferase in UBE2N-WT group not in UBE2N-MUT group. (**I**) The transcriptional regulation of UBE2N by MYC was confirmed by ChIP-qPCR assay. *** *P* < 0.001.

To uncover the mediators of ALDOC’s effects on lung cancer development, we performed a gene expression analysis using the genechip primeview human patharray™ in A549 cells after shALDOC and shCtrl infection. Statistical analysis identified a total of 4182 differentially expressed genes (DEGs) upon ALDOC knockdown, with 2398 upregulated and 1884 downregulated genes. The heat map displaying the top 20 DEGs was shown in Figure 3A. Among them, we selected those that (i) were downregulated by shALDOC, (ii) contained logFC ≥ 2, and (iii) were related to NSCLC patients’ prognosis (Supplementary Figure 1). After applying these stringent filters, three genes RAD51AP1, UBE2N and KIAA0101, reached the threshold. Subsequent analysis confirmed a clear reduction in UBE2N mRNA and protein levels upon ALDOC knockdown (Figure 3B, 3C). Further examination of UBE2N expression in NSCLC cell lines revealed its upregulation compared to normal cell lines (Figure 3D). These data implied that UBE2N is a downstream target of ALDOC. Next, we investigated the mechanism underlying the interaction between ALDOC and UBE2N. Analysis of the HumanTFDB (hust.edu.cn) website identified MYC as the transcription factor of UBE2N. Immunofluorescence experiments showed that ALDOC promoted MYC translocation into the nucleus (Figure 3E), indicating an interaction between ALDOC and MYC. Western blot analysis confirmed increased MYC levels in the nuclear fraction of NSCLC cell lines following ALDOC overexpression (Figure 3F). Also, MYC overexpression led to an upregulation of UBE2N protein level (Figure 3G). To validate the hypothesis that ALDOC regulates UBE2N expression through MYC, we performed a dual-luciferase assay using a luciferase reporter construct with wild-type (WT) or mutated (MUT) UBE2N promoter. The results showed that MYC significantly increased luciferase expression in the UBE2N-WT group compared to the negative control (NC) group (*P* < 0.001), indicating their binding interaction. However, in the UBE2N-MUT group, MYC failed to upregulate luciferase expression (Figure 3H). Furthermore, a ChIP-qPCR assay confirmed that MYC overexpression enhanced the transcriptional regulation of UBE2N (Figure 3I). Collectively, our findings demonstrated that ALDOC could transcriptionally activate UBE2N by interacting with MYC.

### ALDOC mediates NSCLC development via acting on UBE2N

**Figure 4:**
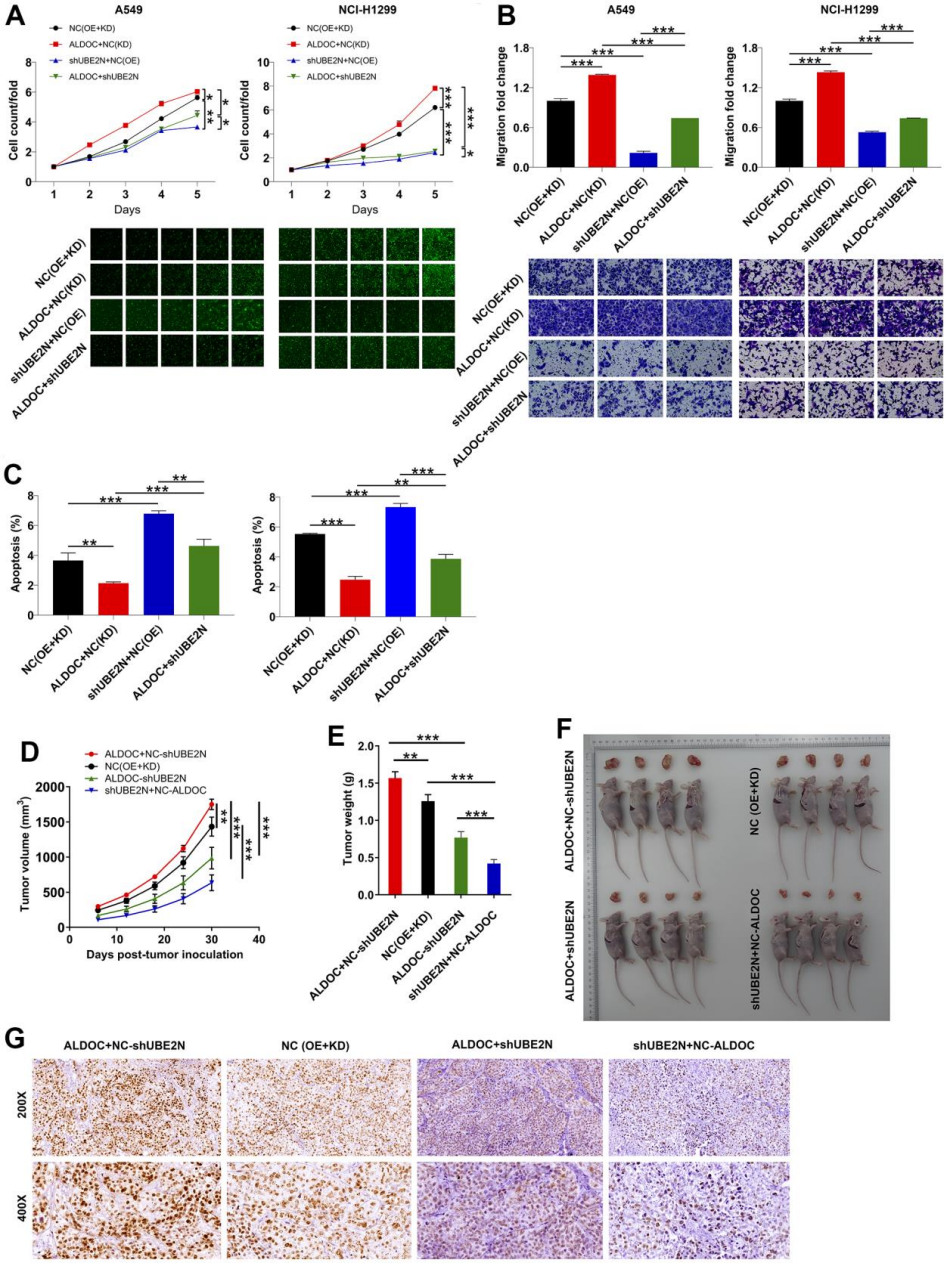
**ALDOC mediates NSCLC development via acting on UBE2N.** (**A**–**C**) After infecting indicated lentiviral particles in A549 and NCI-H1299 cells, the changes in cell proliferation (**A**), migration (**B**) and apoptosis (**C**) were evaluated via CCK8, transwell and flow cytometry assays. (**D**, **E**) The volume (**D**) and weight (**E**) of tumor from xenograft models were monitored. (**F**) The tumor were harvested and photographed. (**G**) Ki67 was stained using IHC staining. NC (OE+KD): Control; NC (KD)+ALDOC: ALDOC overexpression; NC (OE)+shUBE2N: UBE2N downregulation; ALDOC+shUBE2N: ALDOC overexpression and UBE2N downregulation. *** *P* < 0.001; ** *P* < 0.01; *P < 0.05.

In this section, we aimed to examine the effects of ALDOC and UBE2N on NSCLC progression both *in vitro* and *in vivo*. To achieve this, we established cell models of A549 and NCI-H1299 cells with merely overexpressing ALDOC, merely silencing UBE2N, and simultaneously silencing UBE2N and overexpressing ALDOC. In agreement to the above data, forced ALDOC expression enhanced the proliferation and migration of A549 and NCI-H1299 cell lines, while arresting cell apoptosis. Furthermore, silencing UBE2N achieved the same effects on lung cancer cells as knocking down ALDOC. More notably, UBE2N knockdown inhibited the proliferation and migration of ALDOC-overexpressing A549 and NCI-H1299 cells. Then, flow cytometry experiments showed that UBE2N inhibition accelerated cell apoptosis in cells overexpressing ALDOC (Figure 4A–4C).

Additionally, we generated xenograft tumor models by subcutaneously injecting the aforementioned cell models into nude mice. Tumor length and width were measured at regular intervals to calculate tumor volume. Consistent with our expectations, forced expression of ALDOC led to increased tumor volume and weight. Furthermore, increased expression of Ki67, as verified by IHC staining, indicated enhanced tumor proliferation. Conversely, depletion of UBE2N impaired the growth of xenografts and reversed the malignant phenotypes of ALDOC-overexpressing A375 cells (Figure 4D–4G). These data indicated that ALDOC and UBE2N contribute to the development of NSCLC, both *in vitro* and *in vivo*.

### ALDOC regulates NSCLC through Wnt/β-catenin pathway

**Figure 5:**
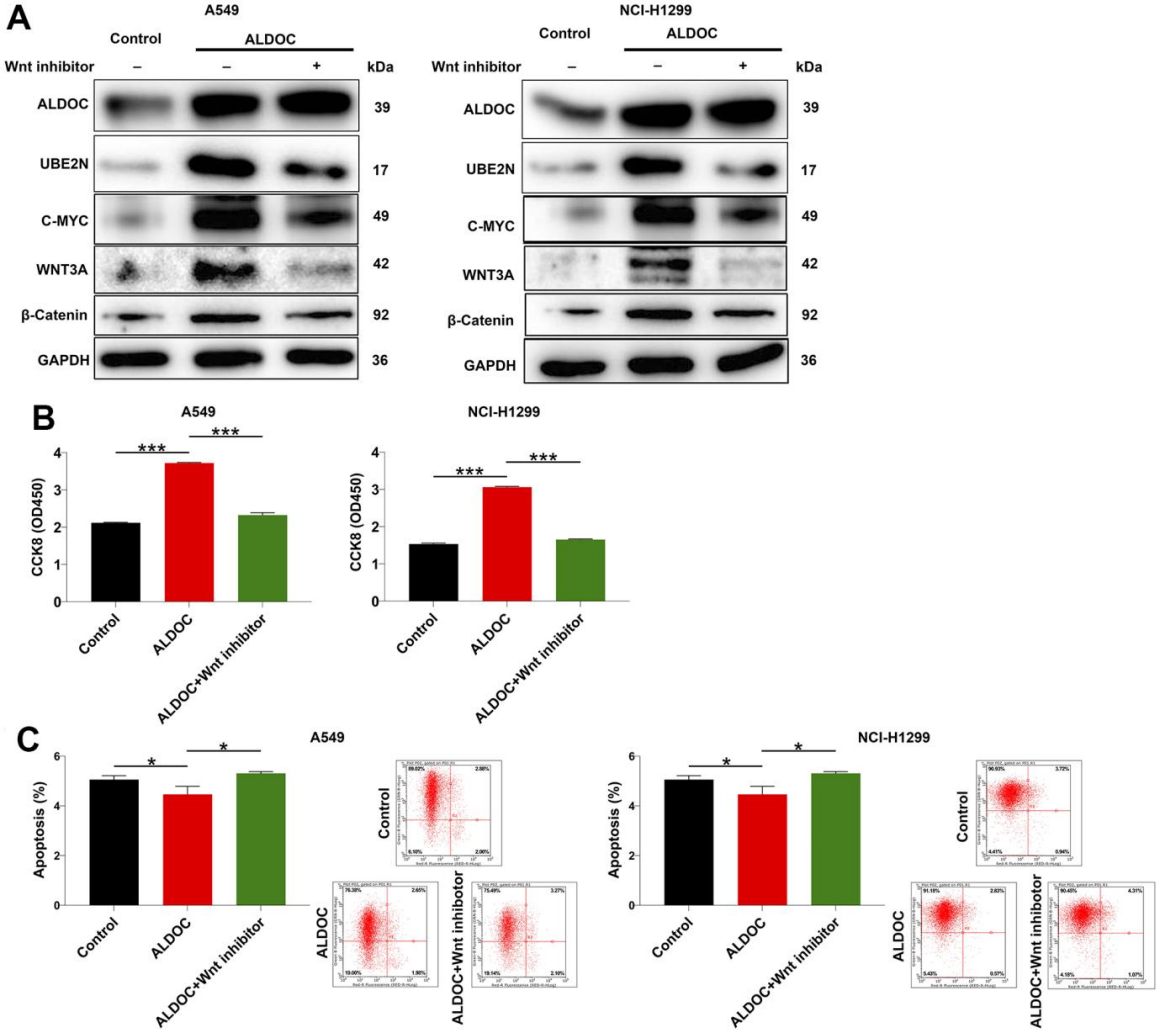
**ALDOC regulates NSCLC through Wnt/β-catenin pathway.** (**A**) After Wnt inhibitor treatment, the levels of ALDOC, UBE2N, c-MYC, WNT3A and β-catenin in ALDOC-overexpressing A549 and NCI-H1299 cells were investigated through western blot analysis. (**B**, **C**) After Wnt inhibitor treatment in ALDOC-overexpressing A549 and NCI-H1299 cells, the changes in cell proliferation (**B**) and apoptosis (**C**) were evaluated via CCK8 and flow cytometry assays. *** *P* < 0.001; *P < 0.05.

Finally, we sought to gain insights on the downstream pathway involved in ALDOC-induced NSCLC. Previous studies have shown that EPB41 suppresses the Wnt/β-catenin signaling in NSCLC by sponging ALDOC [17]. Additionally, the Wnt/β-catenin pathway has been implicated in regulating cell proliferation and apoptosis, playing a crucial role in cancer initiation and progression [18]. Based on these findings, we hypothesized that ALDOC might regulate NSCLC cell events through the Wnt/β-catenin pathway. To test this hypothesis, we performed western blot analysis in ALDOC-overexpressing A549 and NCI-H1299 cells and observed an increase in ALDOC, UBE2N, as well as c-MYC, WNT3A and β-catenin, which are key components of the Wnt/β-catenin pathway. Importantly, treatment with a Wnt inhibitor led to the inhibition of these proteins excluding ALDOC (Figure 5A). Then, we performed a CCK8 assay to assess the effects of Wnt inhibitor on cell proliferation. The results showed that Wnt inhibitor disrupted the proliferative potential of the cells (Figure 5B). Additionally, it accelerated cell apoptosis (Figure 5C). These data demonstrated that ALDOC regulates NSCLC through Wnt/β-catenin pathway.

## DISCUSSION

ALDOC is a metabolic enzyme that primarily functions in glycolysis, the metabolic pathway that converts glucose into energy. In recent years, there has been emerging evidence suggesting that metabolic enzymes can also have non-metabolic functions, including the regulation of gene expression and signaling pathways. For example, several metabolic enzymes have been implicated in tumorigenesis and progression of cancer, where they play multifaceted roles beyond their canonical metabolic functions [14, 19, 20]. In the case of ALDOC, it has been shown to interact with various signaling molecules and transcription factors, suggesting its involvement in cellular processes beyond glycolysis [21, 22]. In our study, we observed significant alterations in the expression levels of ALDOC in NSCLC cell lines and patient samples. Importantly, these alterations were associated with changes in the expression of UBE2N and the activity of Wnt/β-catenin pathway.

UBE2N, an ubiquitin-conjugating enzyme, plays a central role in ubiquitin-mediated cellular activities, such as signal transduction. UBE2N has dual roles in cells. On one hand, UBE2N is engaged in various human biological processes, including protein degradation, cell cycle regulation, apoptosis, and DNA repair [23–25]. On the other hand, its abnormality have been implicated in diseases and may contribute to the development of cancers [26, 27]. Interestingly, recent studies have focused on the functional significance of UBE2N in human cancers. For instance, Dikshit A et al. reported that UBE2N is highly expressed in malignant melanoma and promotes melanoma growth via MEK/FRA1/SOX10 signaling [28]. Another study from Zhu et al. illustrated that UBE2N regulates the sensitivity of ovarian cancer cells to paclitaxel through the Fos/P53 Axis [29]. In this study, we found that UBE2N was increased in lung cancer cell lines. Silencing UBE2N achieved the same effects on lung cancer cells as knocking down ALDOC. More notably, UBE2N knockdown inhibited the proliferation and migration of ALDOC-overexpressing A549 and NCI-H1299 cells. At the same time, UBE2N inhibition accelerated cell apoptosis in ALDOC-overexpressing cells. Thus, we concluded that ALDOC regulates the proliferation, migration, and apoptosis of lung cancer cells via targeting UBE2N.

The Wnt/β-catenin pathway is highly conserved and plays a crucial role in various cellular processes, including embryonic development, proliferation, and differentiation. The Wnt family is implicated in normal physiological activities such as organ formation, stem cell renewal, and cell survival [30]. In humans, the Wnt family consists of cysteine-rich glycoproteins that serve as ligands for up to 15 receptors and co-receptors [31]. These ligands induce intracellular signal transduction pathways, including the Wnt/β-catenin dependent or canonical pathway, as well as the β-catenin-independent or non-canonical pathway [32]. Aberrations in the Wnt/β-catenin pathway are implicated in numerous human malignancies. Accumulating evidence has shown that dysregulation of Wnt/β-catenin signaling promotes the development and progression of human cancers, including head and neck cancer [33], liver cancer [34], prostate cancer [35], and ovarian cancer [18]. In lung cancer, Wnt signaling is particularly important in NSCLC cell lines and may influence tumorigenesis, prognosis and treatment resistance [36]. Consequently, targeting the Wnt signaling pathway has become a major focus in the study of solid malignancies [37]. Here, we found that the overexpression of ALDOC led to an increase in UBE2N, C-MYC, WNT3A, and β-catenin expression, and these effects were diminished upon treatment with a Wnt inhibitor. Moreover, the Wnt inhibitor mitigated cell viability and promoted cell apoptosis. These findings indicated that ALDOC influences NSCLC cell behaviors through the Wnt/β-catenin pathway.

Our findings indicated a potential correlation between ALDOC, UBE2N, and the Wnt/β-catenin signaling pathway in NSCLC. The specific mechanism by which ALDOC alteration leads to changes in transcriptional regulation of UBE2N was investigated. ALDOC could transcriptionally activate UBE2N by interacting with MYC. However, the binding sites between ALDOC and MYC and their functional relevance remains to be investigated. ALDOC is a metabolic enzyme. The altered expression of ALDOC has been linked to various metabolic pathways, including glycolysis and pentose phosphate pathway (PPP) metabolism, in cancer cells [38]. Moreover, research has demonstrated the crosstalk between metabolic alterations and transcriptional regulation in cancer. For example, ERG-rearrangements possibly induce metabolic changes in prostate cancer by increasing glucose uptake through activation of major metabolic signaling molecules such as neuropeptide Y (NPY) [39]. We hypothesize that the altered expression of ALDOC in NSCLC could disrupt glucose metabolism and other metabolic processes, resulting in changes in the transcriptional profile of NSCLC cells and patient samples. Moreover, ALDOC alteration might lead to the transcriptional changes in NSCLC via Wnt/β-catenin signaling pathway. The correlation between UBE2N and the Wnt pathway in the context of NSCLC has not been determined. Several plausible explanations can be proposed based on existing knowledge. It has been reported that overexpression of Evi/Wls is sufficient to enhance downstream Wnt signaling in glioma, making it a crucial regulator of glioma tumorigenesis [40]. Evi/Wls is constantly produced in Wnt-secreting cells and immediately degraded in the absence of lipid-modified Wnts [41]. The degradation of Evi/Wls relies on its ubiquitination by the ER membrane-associated E3 ubiquitin ligase CGRRF1, as well as the E2 ubiquitin-conjugating enzymes UBE2J2, UBE2K, and UBE2N, at multiple amino acid positions [42]. Based on these observations, we hypothesize that Wnt ligands may competitively bind to Evi/Wls to activate Wnt signaling transduction, which in turn inhibits UBE2N-mediated Evi/Wls ubiquitination and promotes the progression of tumors. In our study, we propose that when ALDOC-overexpressed NSCLC cells are treated with a Wnt pathway inhibitor, the binding of Wnt ligand to Evi/Wls is blocked. Consequently, Evi/Wls becomes susceptible to ubiquitination, resulting in a reduction in UBE2N levels. However, further investigation is needed to validate this mechanism.

In conclusion, our work confirmed the role of ALDOC as a promoter in NSCLC, and its interaction with UBE2N regulates the development and progression of NSCLC, suggesting ALDOC as a potential therapeutic target for NSCLC.

## MATERIALS AND METHODS

### Tissue collection

Human lung cancer samples, including tumor tissues and adjacent non-malignant tissues, were collected from a total of 79 patients who underwent primary lung cancer tumor resection at Shandong Provincial Hospital. Informed consent was obtained from all patients and detailed clinicopathological data were obtained and summarized.

### Bioinformatics analysis

RNA-seq data of lung adenocarcinoma (LUAD) cohorts were downloaded from The Cancer Genome Atlas (TCGA) database (https://tcga-data.nci.nih.gov/tcga/). Gene Expression Omnibus (GEO) dataset GSE41271 (https://www.ncbi.nlm.nih.gov/geo/query/acc.cgi?acc=GSE41271) was employed.

### Immunohistochemistry (IHC)

Paraffin sections (4 μm thick) were initially incubated in an oven at 60° C for 30 min. Following dehydration and rehydration, antigen retrieval was performed by adding citric acid buffer and heating at 120° C for 20 min. To block endogenous peroxidase activity, 3% H_2_O_2_ was applied for 10 min. Subsequently, the sections were incubated with the primary antibody overnight at 4° C. Afterward, the secondary antibody was added and incubated for 2 h at room temperature. The slides were then stained with DAB for 5 min and counterstained with hematoxylin (Baso DiagnosticsInc., Zhuhai, China) for 10 – 15 s. Microscopic images were captured and viewed using ImageScope and CaseViewer. All slides were assessed by three independent pathologists in a randomized manner. Staining scores were categorized as follows: 1 (1%-24%), 2 (25%-49%), 3 (50%-74%) and 4 (75%-100%). The staining intensity was scored from 0 (no signal color) to 3 (light yellow, brown, and dark brown). IHC results were defined based on staining scores and intensity scores, specifically classified as negative (0), positive (1-4), ++ positive (5-8) and +++ positive (9-12). Further information regarding the antibodies used in this study was provided in Supplementary Table 1.

### Cell lines and cell culture

Human NSCLC cell lines A549 and NCI-H1299, as well as normal cell line BEAS-2B were purchased from American type culture collection (ATCC). A549 cells were cultured in F12K medium supplemented with 10% fetal bovine serum (FBS), while NCI-H1299 and BEAS-2B cells were cultured in RPMI-1640 medium supplemented with 10% FBS. All cell lines were maintained in a 37° C incubator containing 5% CO_2_.

### Lentivirus RNAi construction and infection

The corresponding RNAi target sequences for ALDOC and UBE2N, as well as the ALDOC overexpression sequence, were designed. These sequences were then ligated to the linearized vector BR-V-108 through the restriction sites at both ends. The resulting constructs were then transferred into prepared DH5α *E. coli* competent cells. Positive clones were identified by PCR, and the plasmids were extracted by the Endofree Maxi plasmid kit. The qualified plasmids were transfected into 293T cells, and the cells were harvested 48-72 h after infection. Finally, the cells were cultured for additional 72 h at 37° C, and the infection efficiency was evaluated.

### RNA extraction and quantitative real-time PCR (qRT-PCR)

A549 and NCI-H1299 cells were collected, and total RNA was extracted with TRIzol reagent (Sigma, St. Louis, MO, USA) following the manufacturer’s instruction. Then, cDNA was synthesized using the Promega M-MLV Kit (Promega, Madison, WI, USA). qRT-PCR assay was conducted using the SYBR Green Mastermixs Kit (Vazyme, Nanjing, Jiangsu, China). GAPDH was chosen as an internal control, and the relative expression of mRNA was determined using the 2^-ΔΔCt^ method. The primer sequences utilized in qRT-PCR were provided in Supplementary Table 2.

### Western blot assay

The total cellular proteins were extracted and quantified using the BCA protein assay kit (Thermo Fisher Scientific, USA, Cat. # A53227). After that, the proteins were resolved in 10% SDS-PAGE and subsequently transferred onto PVDF membranes for Western blot assay. The membranes were then blocked and incubated with primary antibodies followed by secondary antibodies at room temperature for 2 h. For detection, the ECL+plusTM Western blotting system kit was used, and X-ray imaging was performed. The antibodies employed in western blot analysis were listed in Supplementary Table 1.

### CCK8 assay

A549 and NCI-H1299 cell lines after being infected were resuspended and counted. A total of 100 μL cell suspension at a density of 3000 cells/well was added to a 96-well plate, with three replicates set for each group. The cells were then placed in an incubator. Starting from the second day, 10 μL of CCK-8 reagent was added into each well 2~4 h before the culture was terminated. After a 4-h incubation, the 96-well plate was placed on a shaker and oscillated for 2-5 min, and the OD value was measured at 450 nm using a microplate reader for 5 consecutive days. The experiment was repeated three times.

### Colony formation assay

A549 and NCI-H1299 cells infected with the indicated lentivirus were collected, trypsinized, resuspended before being seeded into 6-well plates at a density of 1200 cells per well. The cells were then cultured in their respective growth medium, with a medium change performed every 72 h. Cell clones were visualized and captured using an Olympus fluorescence microscope. To fix the cells, 1 mL of 4% paraformaldehyde was added and allowed to incubate for 30-60 min. Subsequently, the cells were attained with 500 μL of Giemsa for 10-20 min at room temperature. After washing and air-drying, the cells were photographed using a digital camera (XDS-100; Caikang Optical Instrument Co., Ltd., Shanghai, China), and colonies (defined as clones containing > 50 cells) were counted. The experiment was repeated three times.

### Wound-healing assay

A549 and NCI-H1299 cells infected with lentiviruses were inoculated into 96-well plates. Images (50× magnification) were obtained at 0 h, 24 h or 48 h after creating scratches using a Cellomics scanner (cat. no. ArrayScan VT1; Thermo Fisher Scientific, USA). Cell migration was quantified as the ratio of cell migration distance to the width of the initial “0 h” scratch area at different time points.

### Transwell assay

To initiate the migration assay, the upper chamber of a transwell plate was incubated with 100 μL serum-free medium for 1-2 h. A549 and NCI-H1299 cell lines, previously infected with the indicated lentiviruses, were diluted and transferred into each chamber. Simultaneously, 600 μL of medium containing 30% FBS was added to the lower chamber. The upper chamber was then placed onto the lower chamber and incubated for 40 h. Following the incubation, 400 μL of Giemsa staining solution was added to visualize the cells. Finally, the cells were dissolved in 10% acetic acid and the optical density at 570 nm (OD570) was detected. The experiment was repeated three times to assess the migration ability of the cells.

### Detection of cell apoptosis and cell cycle by fluorescence activated cells sorting (FACS)

After infection with the indicated lentiviruses, A549 and NCI-H1299 cell lines were inoculated in a 6-well plate with 2 mL of culture medium per well. When the cells reached 85% confluence, the cell suspension was centrifuged and the supernatants were discarded. The cells were then washed with pre-cooled D-Hanks (pH=7.2~7.4) at 4° C. Next, 10 μL of Annexin V-APC (eBioscience, San Diego, CA, USA) was added for staining in the dark. The level of cell apoptosis was measured and the apoptotic rate was analyzed using FACSCalibur (BD Biosciences, San Jose, CA, USA).

### PrimeView human gene expression array

Total RNA was extracted using previously described methods. The quality and integrity of RNA were determined using the Nanodrop 2000 (Thermo Fisher Scientific, Waltham, MA, USA). Gene expression array was performed with Affymetrix human GeneChip PrimeView according to the manufacturer’s instructions, and the data were scanned using the Affymetrix Scanner 3000 (Affymetrix, Santa Clara, CA, USA). Statistical significance of the raw data was completed using a Welch t-test with the Benjamini-Hochberg FDR correction method (|Fold Change| ≥ 1.3 and FDR < 0.05 were considered significant). Significant difference analysis and functional analysis were performed using Ingenuity Pathway Analysis (IPA) (Qiagen, Hilden, Germany), with a |Z - score| > 0 indicating significance.

### Immunofluorescence (IF) experiment

ALDOC-overexpressing A549 cells were fixed with 4% paraformaldehyde and blocked with serum for 30 min. After that, the slides were incubated with the primary antibody MYC overnight at 4° C, followed by incubation with the secondary antibody at room temperature for 2 h. Finally, the nuclei were stained with DAPI (Abcam, Cat. ab104139) and images were captured. Detailed information about the antibodies used can be found in Supplementary Table 1.

### Dual-luciferase assay

UBE2N wild type (UBE2N-WT) and mutant (UBE2N-MUT) luciferase plasmids were generated using a promoter region fragment of UBE2N. A549 cells were subsequently infected with c-MYC overexpression plasmid in conjunction with UBE2N-WT or UBE2N-MUT plasmids. Firefly luciferase activity and the Renilla luciferase signal were measured using the Promega (Madison, WI, USA) dual luciferase system (Cat. E2940). The sequences of both UBE2N-WT and UBE2N-MUT DNA in the luciferase reporter assay were listed in “Supplementary Information Files 1–3”.

### Chromatin immunoprecipitation (ChIP)-qPCR assay

A549 cells overexpressing MYC were cross-linked with formaldehyde, and then lysed in SDS buffer. The lysates were sonicated to mechanically shear the DNA into fragments. Protein–DNA complexes were precipitated using 5 μg of control normal rabbit IgG (CST, Cat. 13987S), 2 μg Histone H3 (D2B12) XP^®^ Rabbit mAb (CST, Cat. 4620), and anti-MYC antibody (1:50, Sanying, Wuhan, Cat 10828-1-AP). The DNA fragment was eluted and subsequently detected by qPCR. Primers for ChIP-qPCR amplification were presented in Supplementary Table 2.

### The construction of nude mouse tumor formation model

The animal experimental procedures were approved by the Animal Care and Welfare Committee of Shandong Provincial Hospital (Approval No. 2022-158). A total of 1 × 10^7^ A549 cells expressing the indicated lentivirus were subcutaneously injected into four-week-old female BALB-c nude mice obtained from Shanghai Weitong Lihua Animal Research Co., Ltd. (Shanghai, China) to construct xenograft models (4 mice/group). The length (L) and width (W) of the tumors were measured and used to calculate tumor volume (calculated as π/6×L×W^2^, where L represents the longest dimension and W represents the dimension perpendicular to length). After 30 days, the mice were euthanized, and the tumors were excised, weighed, photographed, frozen in liquid nitrogen, and stored at −80° C.

### Statistical analysis

All data were analyzed using GraphPad Prism 6 (San Diego, CA, USA) and SPSS version 24.0. Data were presented as the mean ± standard deviation (SD). The association between ALDOC expression and the clinicopathological characteristics of patients was assessed using the Mann-Whitney U test and Spearman correlation analysis. Student’s t-test was used for comparisons between two groups, while one-way ANOVA was used for comparisons among multiple groups. Statistical significance was set at *P* < 0.05.

### Data availability statement

All data generated or analysed during this study are available from the corresponding author on reasonable request.

## Supplementary Material

Supplementary Figure 1

Supplementary Tables 1-3

Supplementary Information File 1

Supplementary Information File 2

Supplementary Information File 3
