# Supplementary material for: ALDOC promotes non-small cell lung cancer through affecting MYC-mediated UBE2N transcription and regulating Wnt/β-catenin pathway
Source: Aging (Albany NY). 2023 Sep 18;15(18):9614–32. doi: 10.18632/aging.205038 (PMC10564444; doi:10.18632/aging.205038)
Supplement: Supplementary Figure 1 [file aging-15-205038-s001.pdf]

SUPPLEMENTARY FIGURE

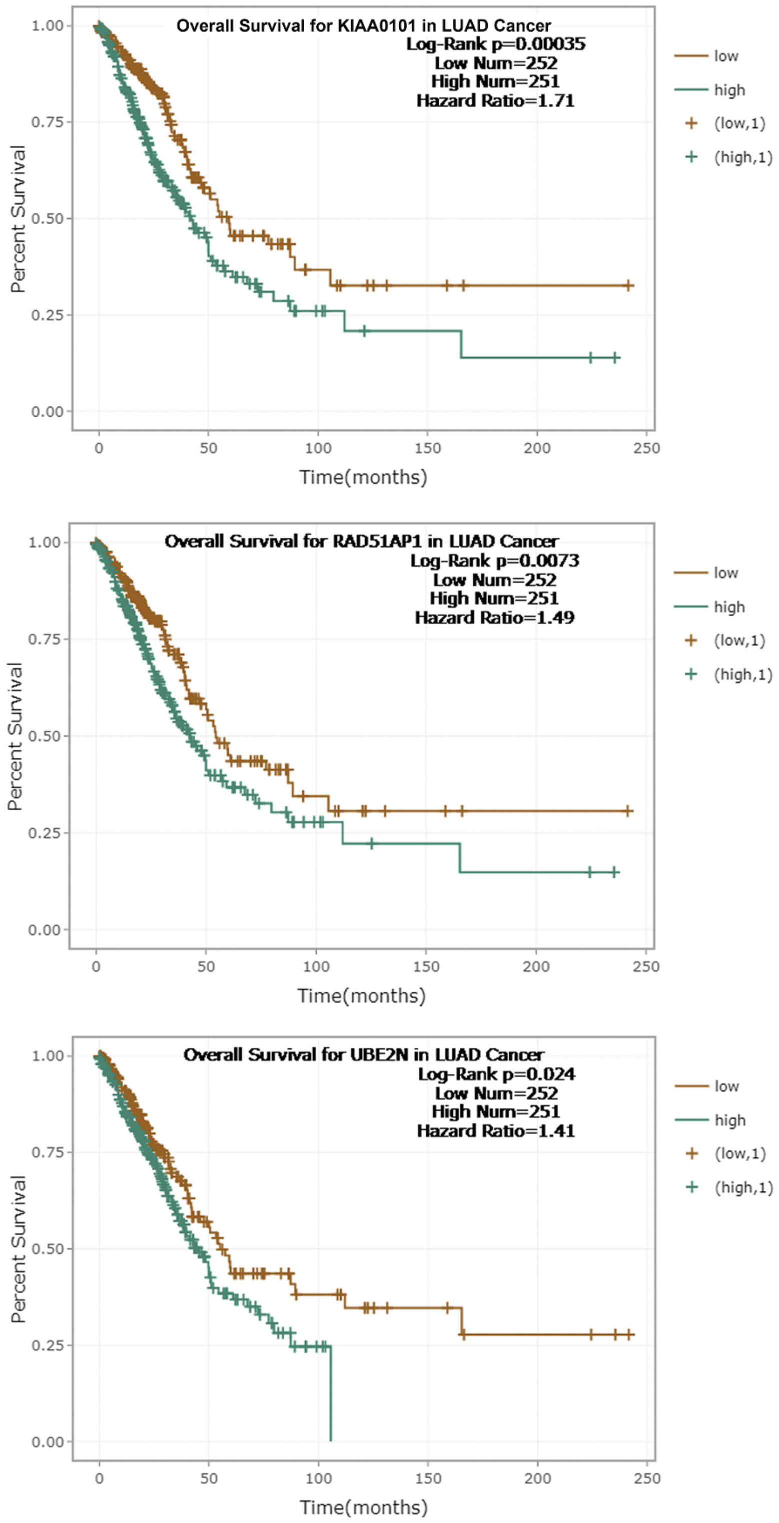

Supplementary Figure 1. The relationship between KIAA0101, UBE2N and RAD51AP1 expression with NSCLC patients' prognosis.
