# Supplementary material for: ALDOC promotes non-small cell lung cancer through affecting MYC-mediated UBE2N transcription and regulating Wnt/β-catenin pathway
Source: Aging (Albany NY). 2023 Sep 18;15(18):9614–32. doi: 10.18632/aging.205038 (PMC10564444; doi:10.18632/aging.205038)
Supplement: Supplementary Tables 1-3 [file aging-15-205038-s002.pdf]

SUPPLEMENTARY TABLES

Supplementary Table 1. Antibodies used in Western blotting, IHC and IF.

Primary antibodies	Dilution in WB	Source species	Company	Catalog no.
ALDOC	1:5000	Rabbit	Proteintech	14884-1-AP
RAD51AP1	1:1000	Rabbit	Abcam	ab101321
UBE2N	1:1000	Rabbit	Abcam	ab109286
C-MYC	1:1000	Rabbit	Proteintech	10828-1-AP
WNT3A	1:1000	Rabbit	CST	2721S
β-Catenin	1:2000	Mouse	Proteintech	66379-1-Ig
GAPDH	1:30000	Mouse	Proteintech	60004-1-lg
Secondary antibody	Dilution		Company	Catalog No.
Goat Anti-Rabbit	1:3000		Beyotime	A0208
Goat Anti- Mouse	1:3000		Beyotime	A0216

Primary antibodies	Dilution in IHC and IF	Source species	Company	Catalog no.
ALDOC	1:200	Rabbit	Sanying, Wuhan	14844-1-AP
Ki67	1:100	Rabbit	Abcam	ab16667
MYC	1:200	Rabbit	Sanying, Wuhan	10828-1-AP
Secondary antibody	Dilution		Company	Catalog No.
Goat Anti-Rabbit	1/400		Abcam	ab97080
Alexa Fluor® 594 Goat Anti-Rabbit (H+L)	1/200		Yisheng	33112ES60

Supplementary Table 2. Primers used in qPCR and ChIP-qPCR.

Gene	Forward primer sequence (5′-3′)	Reverse primer sequence (5′-3′)
ALDOC	TACCCCAGAGGAGATTGCCAT	GCCTCTTCTTCGCTCTGACC
UBE2N	ATCCGCACAGTTCTGCTATCG	TATGGCTTGGGCTTCGTTG
UBE2N(ChIP-qPCR)	ACACTCCCAACACAATAGCACTC	GAGAAGCCAAGGTTCAGAGGTAAT
RAD51AP1	GGAAGATGATGTTGGTGGTGTT	GTGCAAAGTCTGGTTCAGTGTC
KIAA0101	GGATAGTTTTCGGGTCCTTGT	AGCAGCCACCACTTTTCTGTA
GAPDH	TGACTTCAACAGCGACACCCA	CACCCTGTTGCTGTAGCCAAA

Supplementary Table 3. Correlation between the expression of ALDOC and clinicopathological parameters based on RNA-seq data of lung adenocarcinoma (LUAD) cohorts from TCGA database.

		ALDOC expression		No.	*P* value
		Low	High		
Stage	Stage_I	143	131	274	< 0.001
	Stage_II	54	67	121	
	Stage_III	43	41	84	
	Stage_IV	12	14	26	
Tumor infiltrate (T)	T1	84	84	168	< 0.001
	T2	136	140	276	
	T3	26	21	47	
	T4	8	11	19	
lymphatic metastasis (N)	N0	172	158	330	< 0.001
	N1	39	56	95	
	N2	37	37	74	
	N3	0	2	2	
Metastasis (M)	M0	179	165	344	< 0.001
	M1	11	14	25	
	M2	0	0	0	
	M3	0	0	0	
