# Supplementary material for: ALDOC promotes non-small cell lung cancer through affecting MYC-mediated UBE2N transcription and regulating Wnt/β-catenin pathway
Source: Aging (Albany NY). 2023 Sep 18;15(18):9614–32. doi: 10.18632/aging.205038 (PMC10564444; doi:10.18632/aging.205038)

**Supplementary Information File 1. Immunofluorescence data_MYC_ALDOC and MYC_NC.**

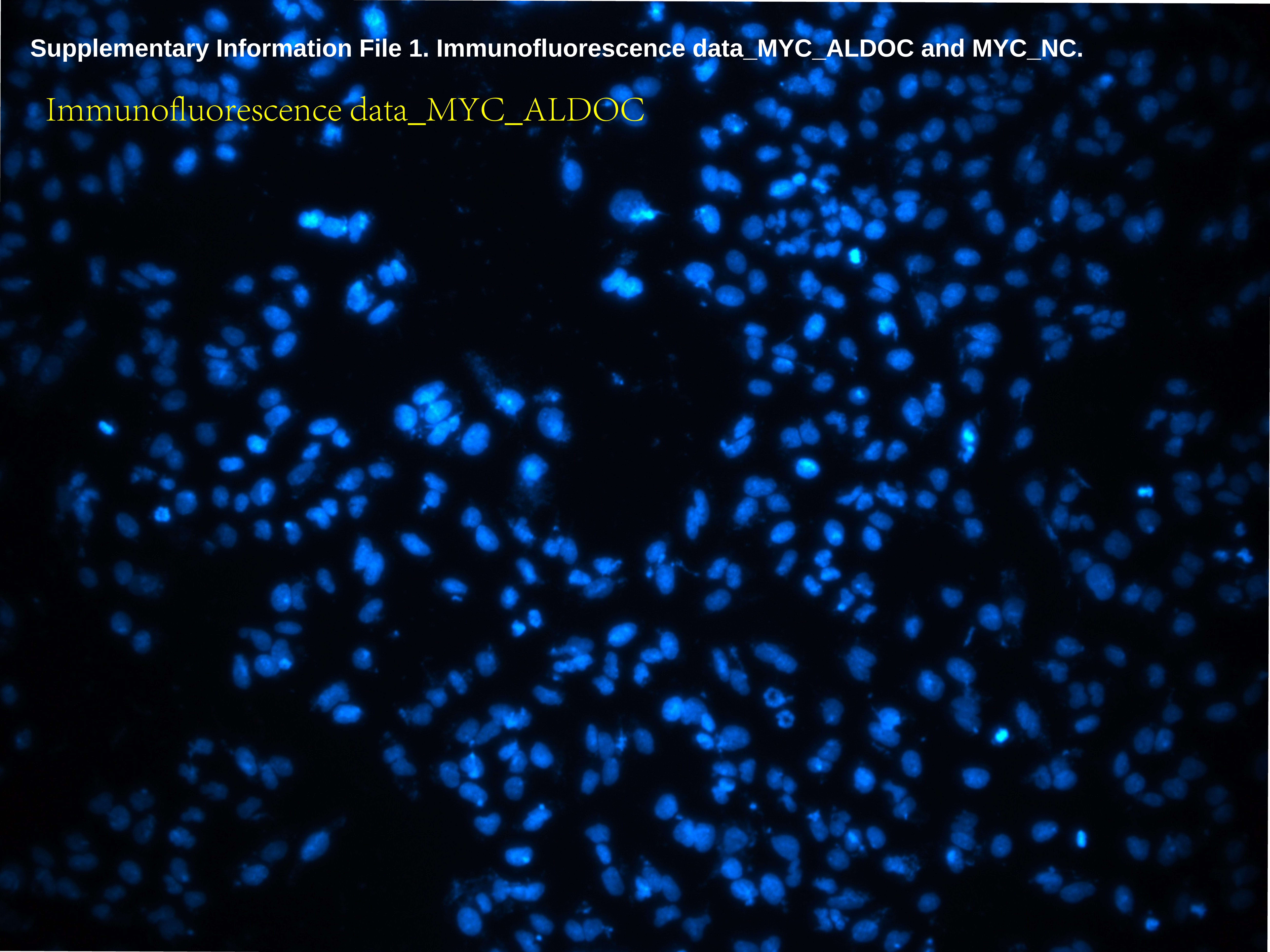

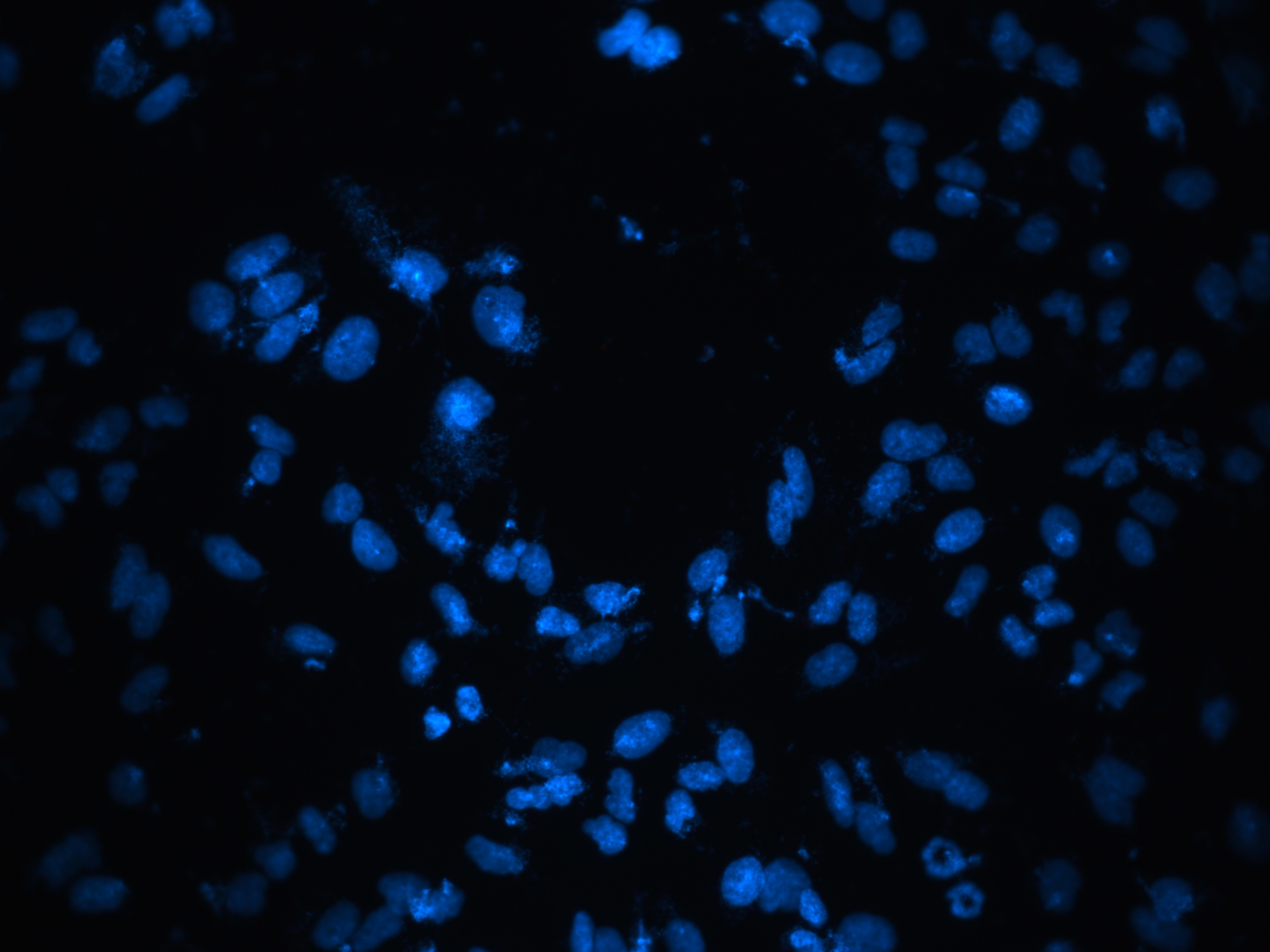

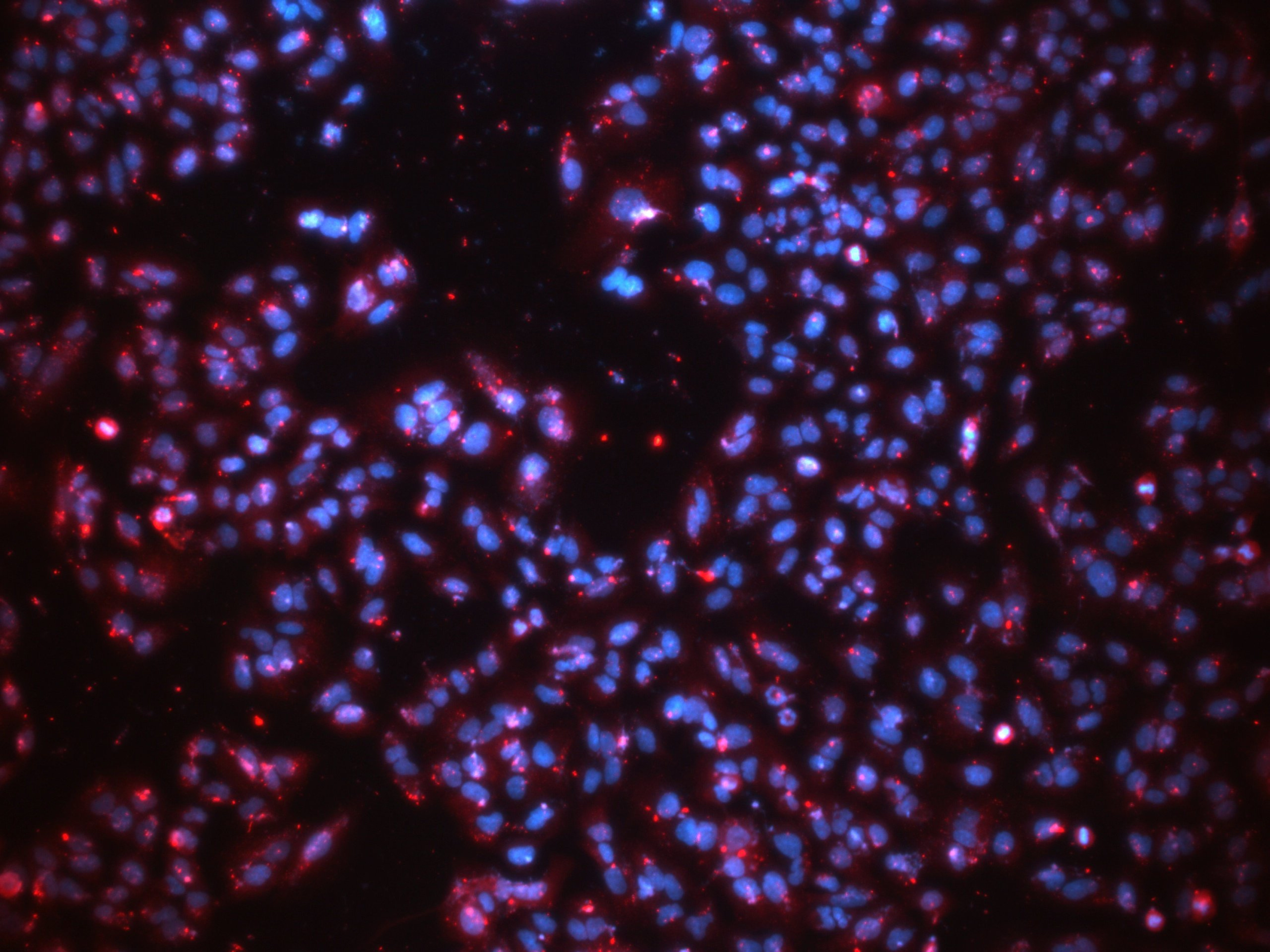

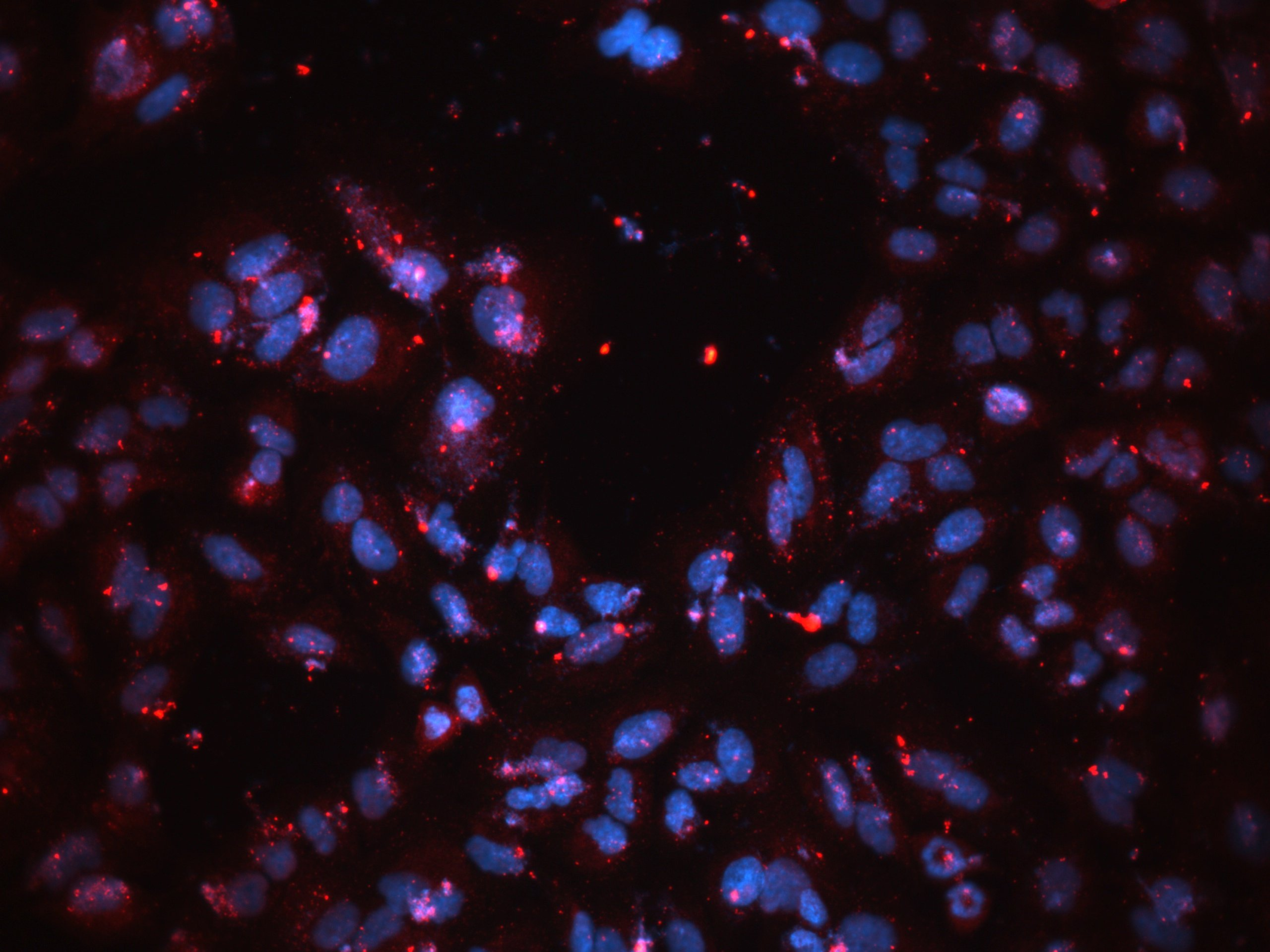

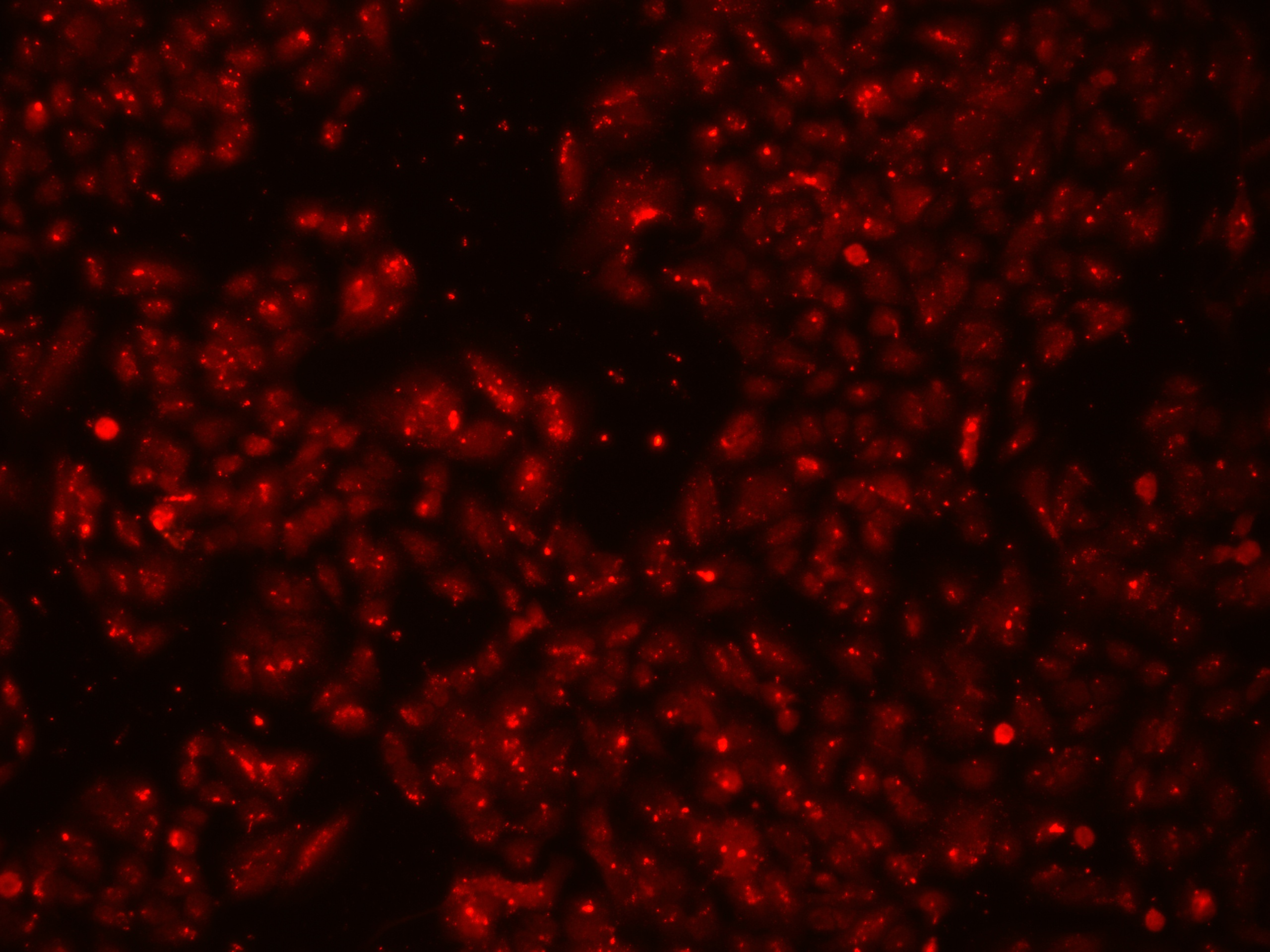

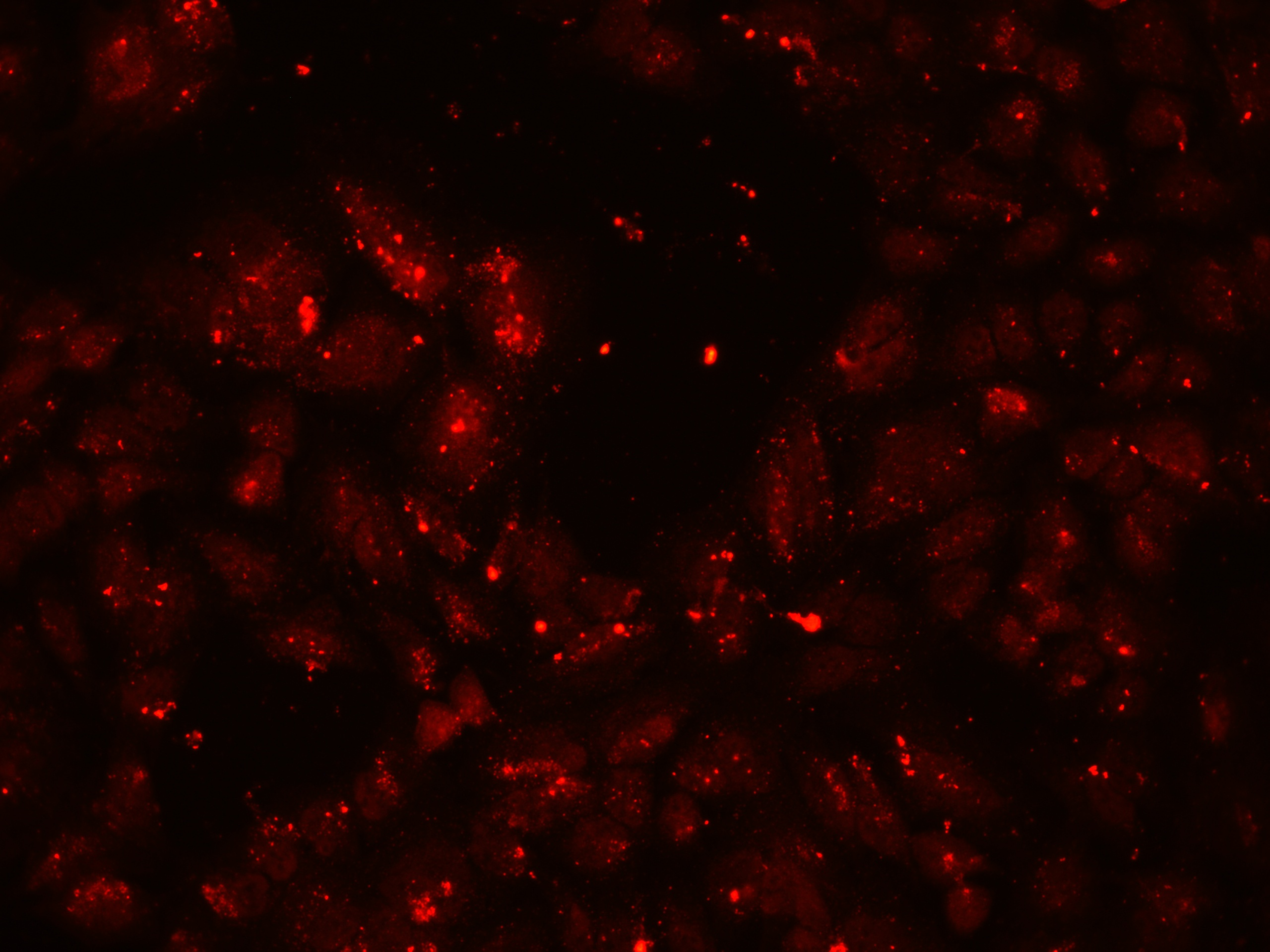

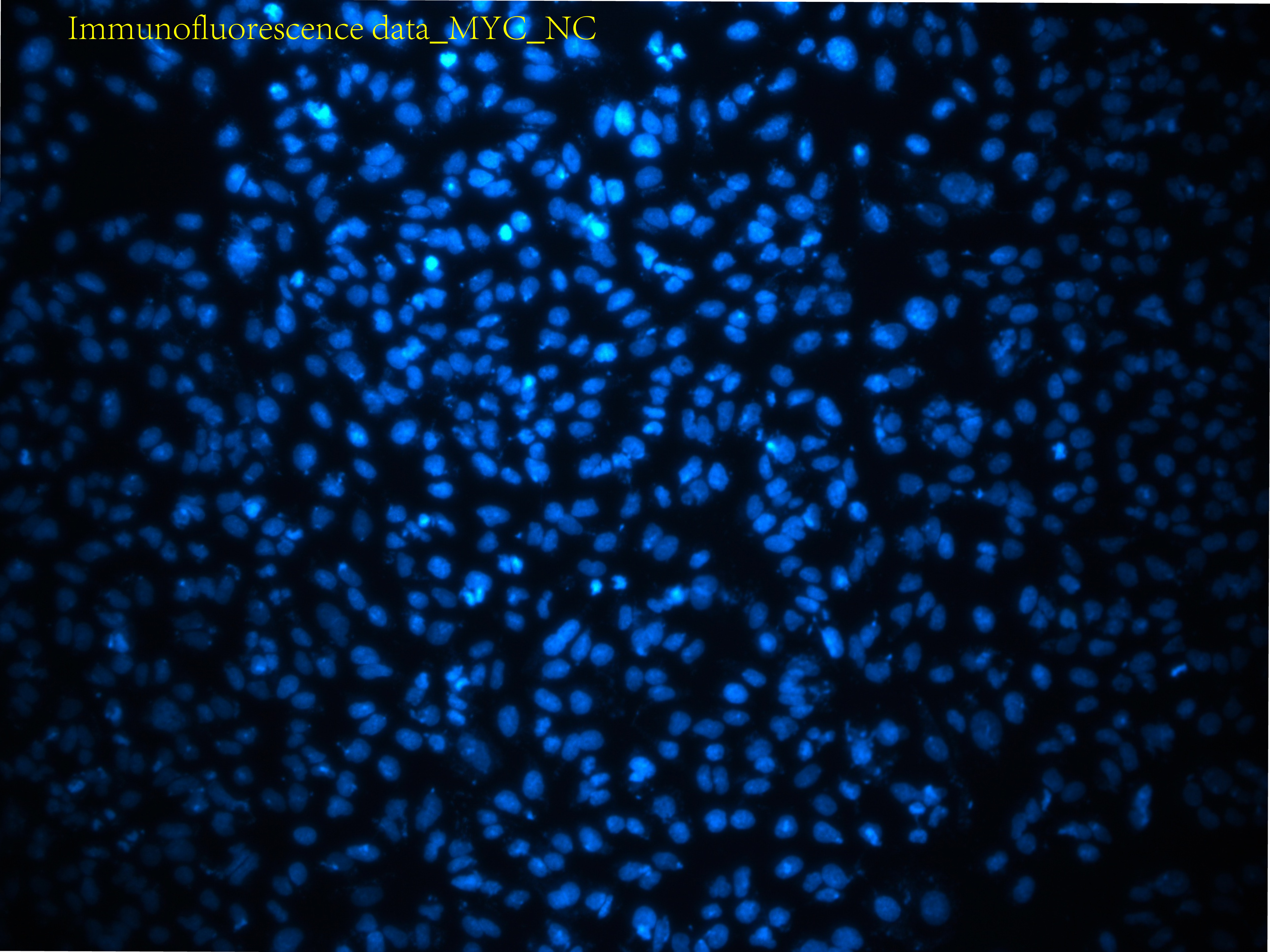
Immunofluorescence data_MYC_NC

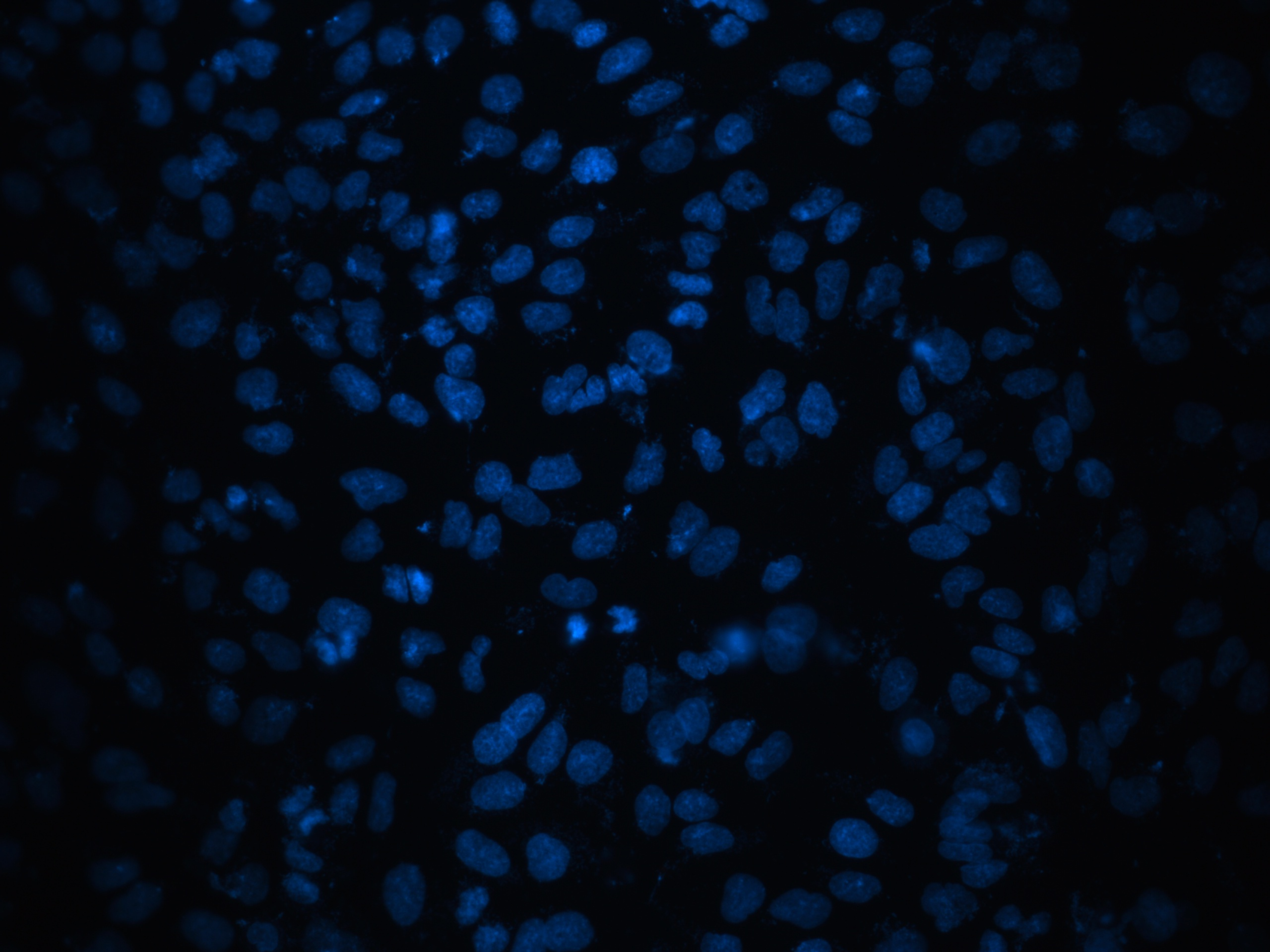

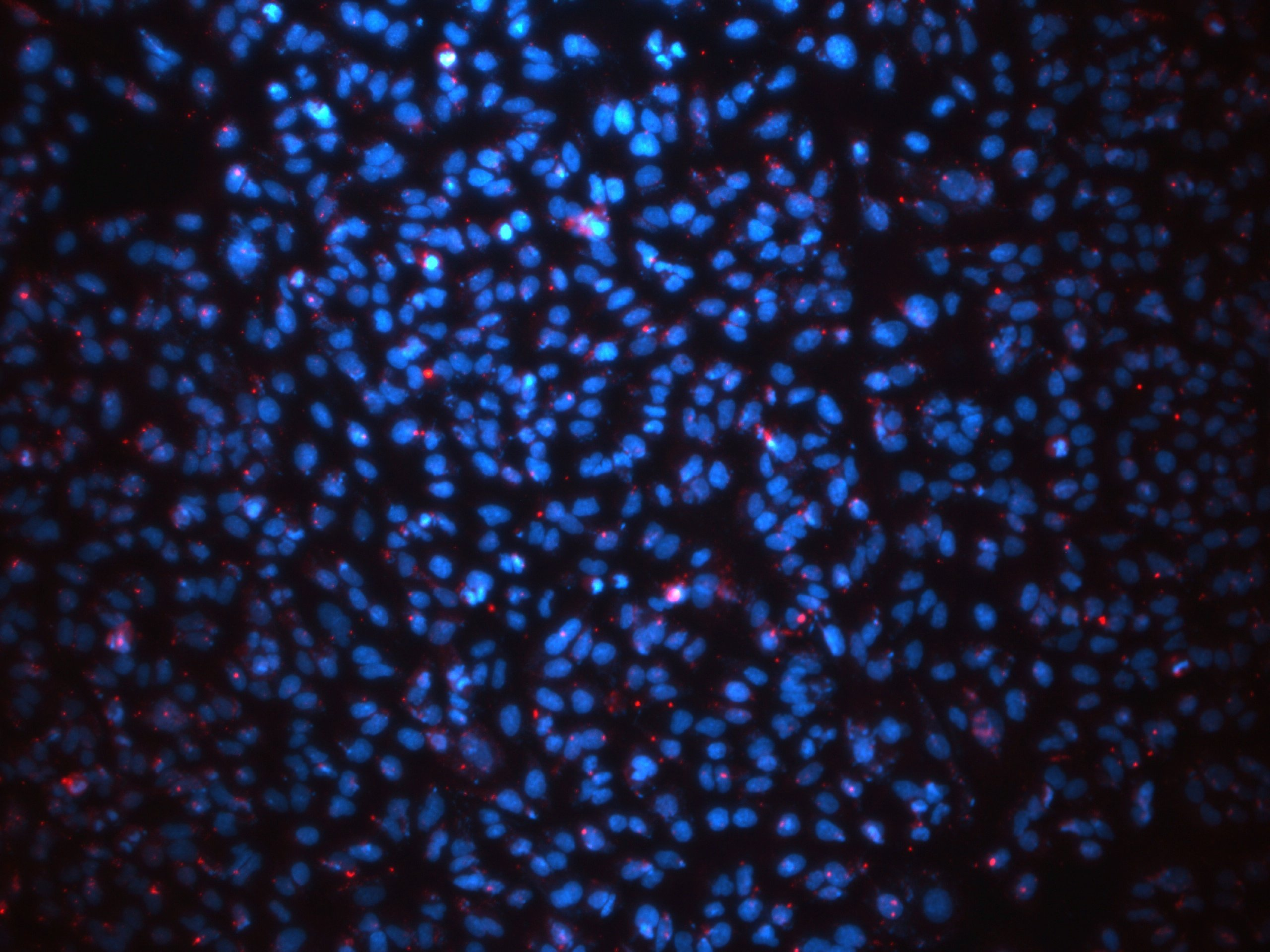

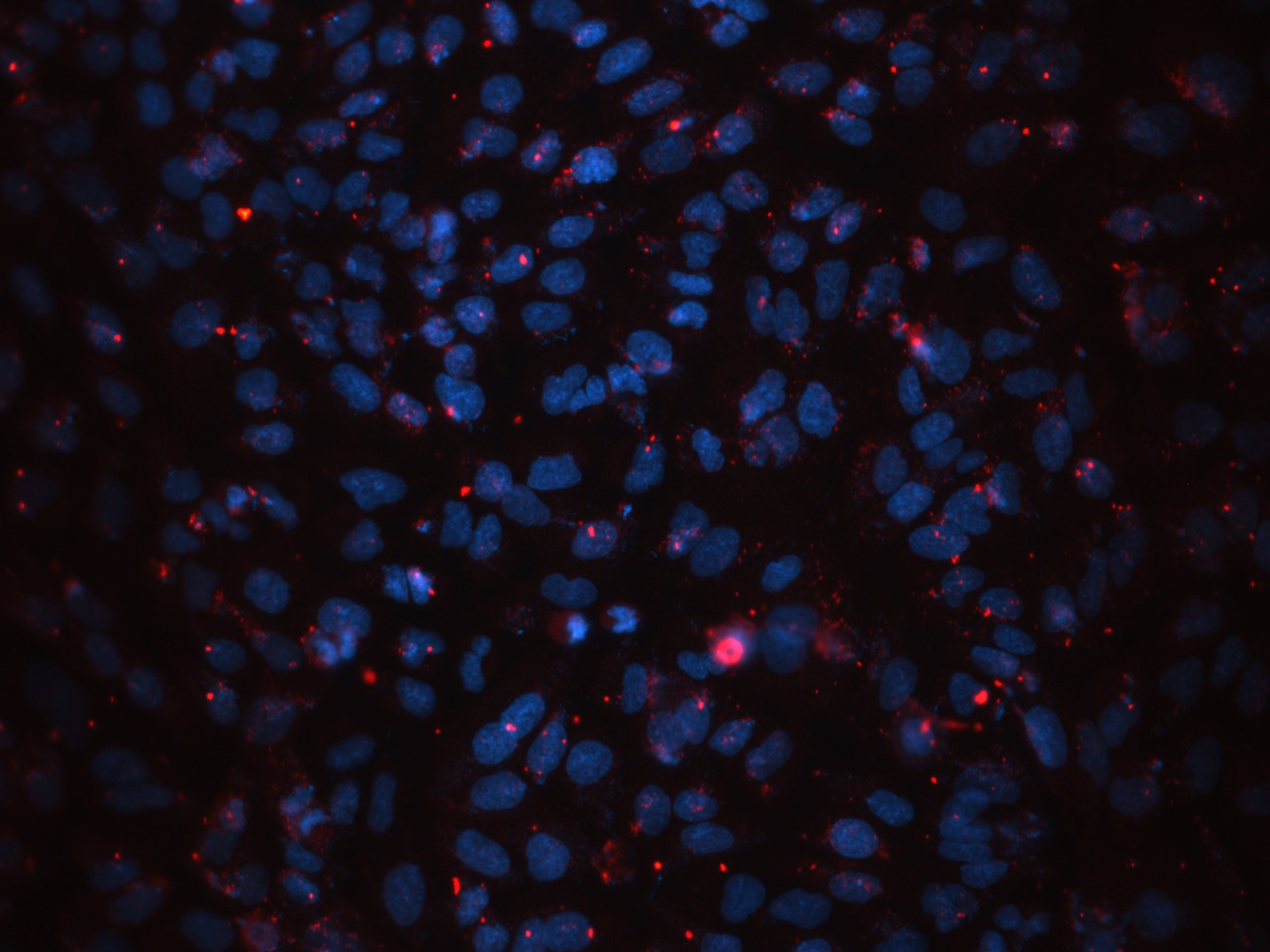

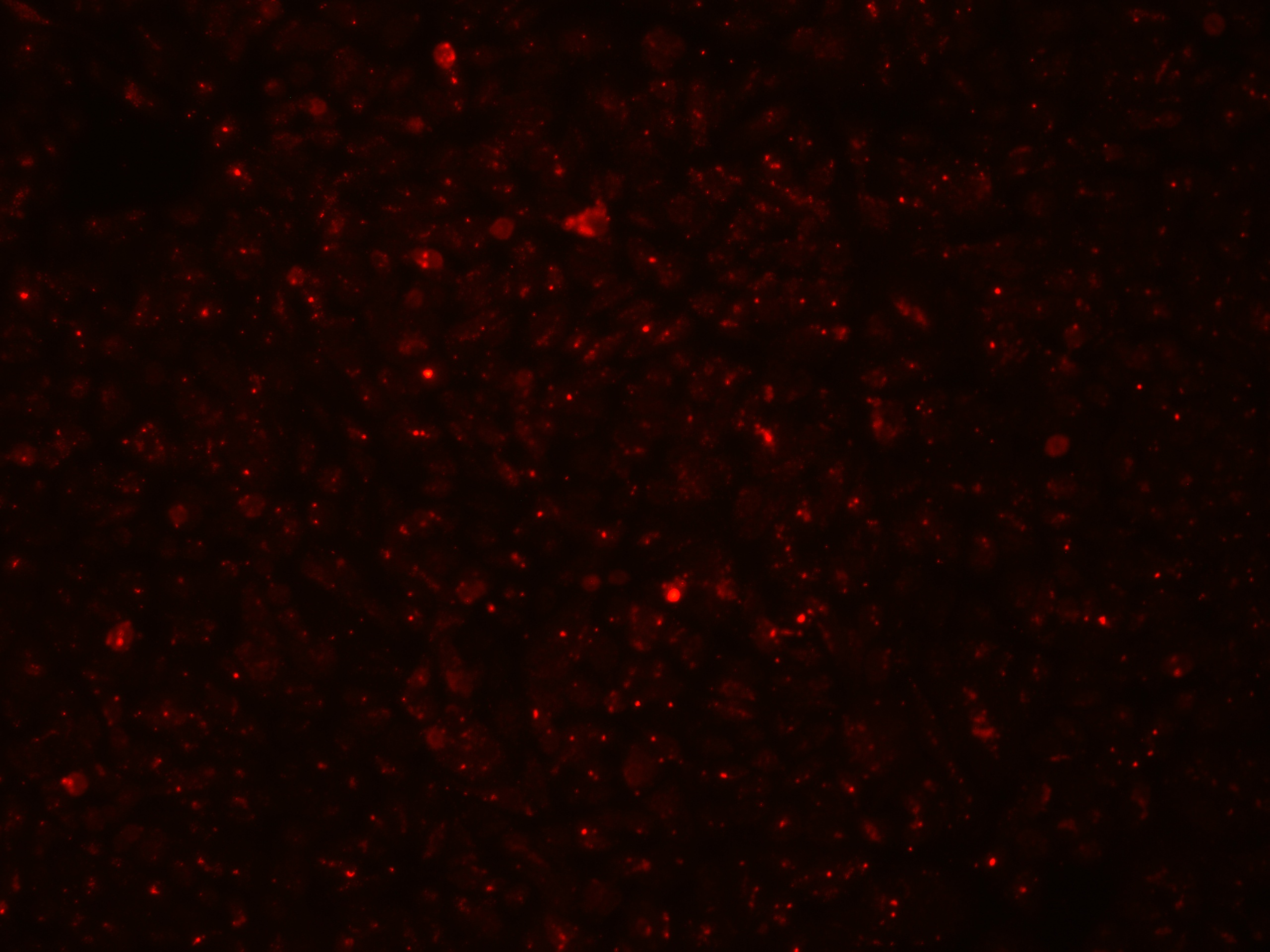

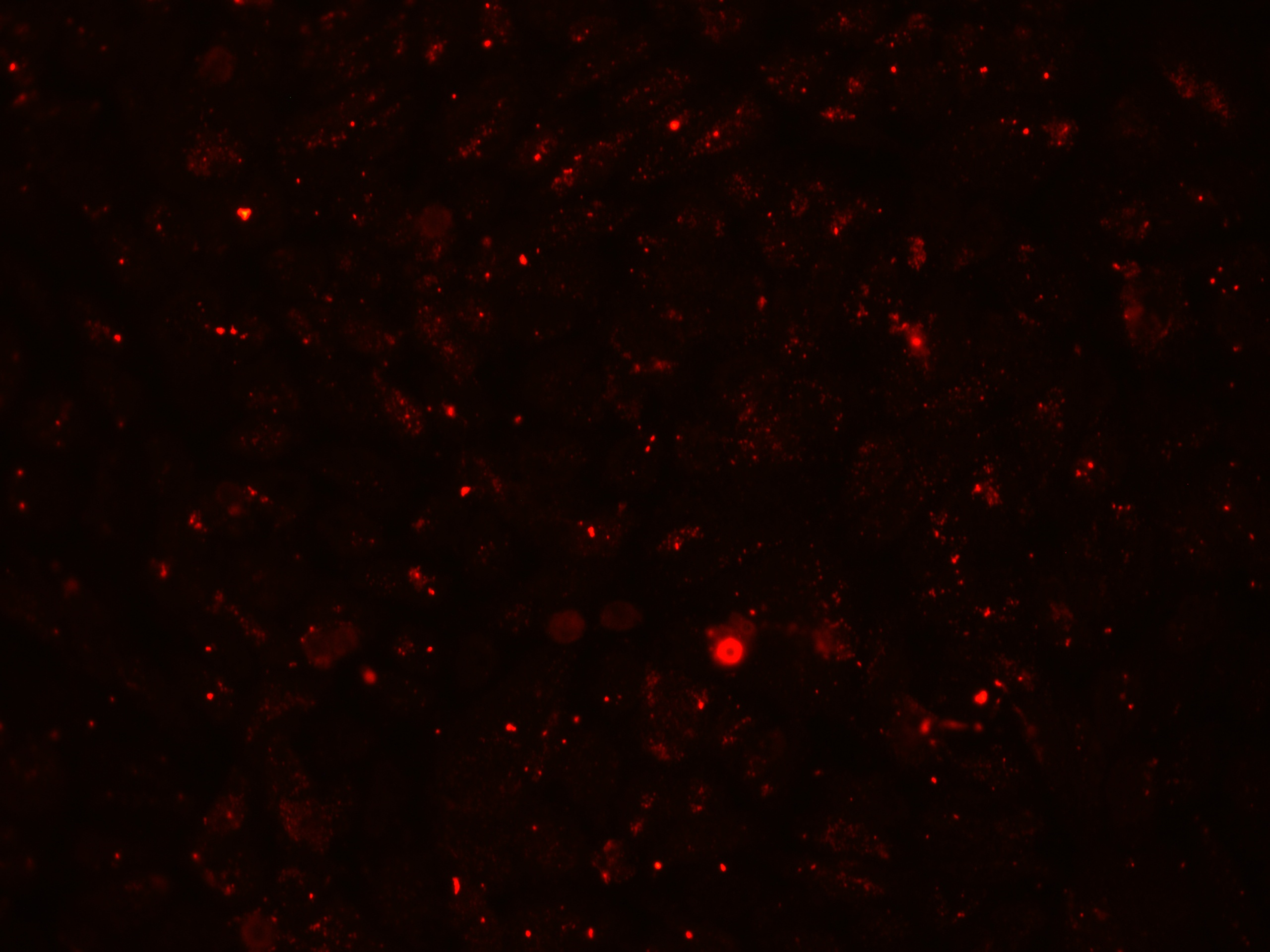

Supplement: Supplementary Information File 1 [file aging-15-205038-s003.pdf]
