# Supplementary material for: ALDOC promotes non-small cell lung cancer through affecting MYC-mediated UBE2N transcription and regulating Wnt/β-catenin pathway
Source: Aging (Albany NY). 2023 Sep 18;15(18):9614–32. doi: 10.18632/aging.205038 (PMC10564444; doi:10.18632/aging.205038)
Supplement: Supplementary Information File 3 [file aging-15-205038-s005.docx]

**Supplementary Information File 3. The sequences of UBE2N-WT and UBE2N-MUT DNA in the luciferase reporter assay.**

UBE2N WT

ccacgaggccatgtgatttccacggctgggtcaaaaaaggcgatttagtgtccatttcagcttgctagaacacttactcttggagcccagccaccatgcaatggggaagcccaagatgccccagggagaggcccacttggagtgaaactaacagccagcaccaacttgcccgccttgtgaaccatcatggaagccaatcttctggttgcacaggctgatgcagcacaagcagagtgtgccttcctgccaagccctgcccagactgcagacctacacactcccaacacaatagcactctgcctttctgcctaccagcatgaattatcctgggtcctctataaaatattctgaaaagtatctttgtccagtttcatttacttttgcagattcttctagtcacagaaccacctattgcagacttctggttatttgaaattctcattattacctctgaaccttggcttctcatctaaaacgttctgttaataatagagactacttcatctgtttcataattgaatgatgtattaaatttattaaccatttagcatcgtgccagcacaaaatatttcttattgttcttagtaggtttcttgtattcagagttccagtttctttagtgcactattttttctctctcatagctcaatgccatttcacaggccatttcctatgcttggcatactcttccctacaaggtcaggtaggttcttactcatcattgaggacttcacttatttaagaaggcttccctgagttctcaagcagttaatgcctctctgctaaaatagcaccttggaggaacctgtattacagacagcccttaacacattggattgtaactaattgtttactggtctctcttctccattggaatctaagttcctggaaggcacggtcagtcttgtcttcatttattcaccatctactgtctgttagtgtgctaggaaacactgtggttaagtagttaagggccaggtcttggcggatctgcctggggacaaaacagctccttggaaaaggttaactatctaagcctcatttgtaaaatggaaacaacacctcctcacgattgctgtgaagatgaagtaaagtgcttgcaaacagtccatcacacagcaaaccatccgtcatcaggataaaacgcttccccaacaattcaattttcgggcacccccactctaattctctcataggacctcgcactctataggtaatctctgctttacggattactctgggttctttaaacctctccttggtcattcgtcctgttaacaattctgagtctcaacctttaggtctaggcgttcagaaccattcttcccgcctccaactcgctccaaacccttttccttcagcctaccctgggcctaggggcgtgtcagaggtccgatttatacaatttaaacatcaaaactcccccaacagcgtgtgagtaactggagagacccccagtcagagctttgccaccgagtttcggtcttgccctccctttgaggaaggagcaagacgatgagccccacctcagtcgttcactgcagagccagcagggaacgcgcgcaatgcaggctgagctgaaagaacgaacgcgtgagctgaaagaacgaacgcgtgaatgaatgaatgggatacgtcgtaggccaggcctctgttacgtcgtccaccggcgcgggcgtggccacggttaagagagacgcgcgcgcagtcgcgcgcgggtcgtgccgtaccaccgtcgcgggcaggctcggccacgagcgccagagccccgcgcctcccctcgcggcctgtcccaagtccctgccccgcaacagagcgtcacttccgccatccccggcagcggttggggcggggcgcacgggggagggggccaggtcggagggaagcccgcccgtgcccgagcccgcgcccgagcagggactacatttcccgaggggcctcggcggcggctgcggcgacgggcgcggcaacgtcccccggaagtggagcccgggacttccactcgtgcgtgaggcgagaggagccggagacgagaccagaggccgaactcgggttctgacaagatggccgggctgccccgcaggatcatcaaggtaaccgc

UBE2N MUT

attaatccacgaggccatgtgatttccacggctgggtcaaaaaaggcgatttagtgtccatttcagcttgctagaacacttactcttggagcccagccaccatgcaatggggaagcccaagatgccccagggagaggcccacttggagtgaaactaacagccagcaccaacttgcccgccttgtgaaccatcatggaagccaatcttctggttgcacaggctgatgcagcacaagcagagtgtgccttcctgccaagccctgcccagactgcagacctacacactcccaacacaatagcactctgcctttctgcctaccagcatgaattatcctgggtcctctataaaatattctgaaaagtatctttgtccagtttcatttacttttgcagattcttctagtcacagaaccacctattgcagacttctggttatttgaaattctcattattacctctgaaccttggcttctcatctaaaacgttctgttaataatagagactacttcatctgtttcataattgaatgatgtattaaatttattaaccatttagcatcgtgccagcacaaaatatttcttattgttcttagtaggtttcttgtattcagagttccagtttctttagtgcactattttttctctctcatagctcaatgccatttcacaggccatttcctatgcttggcatactcttccctacaaggtcaggtaggttcttactcatcattgaggacttcacttatttaagaaggcttccctgagttctcaagcagttaatgcctctctgctaaaatagcaccttggaggaacctgtattacagacagcccttaacacattggattgtaactaattgtttactggtctctcttctccattggaatctaagttcctggaaggcacggtcagtcttgtcttcatttattcaccatctactgtctgttagtgtgctaggaaacactgtggttaagtagttaagggccaggtcttggcggatctgcctggggacaaaacagctccttggaaaaggttaactatctaagcctcatttgtaaaatggaaacaacacctcctcacgattgctgtgaagatgaagtaaagtgcttgcaaacagtccatcacacagcaaaccatccgtcatcaggataaaacgcttccccaacaattcaattttcgggcacccccactctaattctctcataggacctcgcactctataggtaatctctgctttacggattactctgggttctttaaacctctccttggtcattcgtcctgttaacaattctgagtctcaacctttaggtctaggcgttcagaaccattcttcccgcctccaactcgctccaaacccttttccttcagcctaccctgggcctaggggcgtgtcagaggtccgatttatacaatttaaacatcaaaactcccccaacagcgtgtgagtaactggagagacccccagtcagagctttgccaccgagtttcggtcttgccctccctttgaggaaggagcaagacgatgagccccacctcagtcgttcactgcagagccagcagggaacgcgcgcaatgcaggctgagctgaaagaacgaacgcgtgagctgaaagaacgaacgcgtgaatgaatgaatgggatacgtcgtaggccaggcctctgttacgtcgtccaccggcgcgggcgtggccacggttaagagagacgcgcgcgcagtcgcgcgcgggtcgtgccgtaccaccgtcgcgggcaggctcaattgtagatatcagagccccgcgcctcccctcgcggcctgtcccaagtccctgccccgcaacagagcgtcacttccgccatccccggcagcggttggggcggggcgcacgggggagggggccaggtcggagggaagcccgcccgtgcccgagcccgcgcccgagcagggactacatttcccgaggggcctcggcggcggctgcggcgacgggcgcggcaacgtcccccggaagtggagcccgggacttccactcgtgcgtgaggcgagaggagccggagacgagaccagaggccgaactcgggttctgacaagatggccgggctgccccgcaggatcatcaaggtaaccgcaagctt
