# Supplementary material for: ALDOC promotes non-small cell lung cancer through affecting MYC-mediated UBE2N transcription and regulating Wnt/β-catenin pathway
Source: Aging (Albany NY). 2023 Sep 18;15(18):9614–32. doi: 10.18632/aging.205038 (PMC10564444; doi:10.18632/aging.205038)
Supplement: Supplementary Information File 2 [file aging-15-205038-s004.pdf]

Supplementary Information File 2. Gene chip analysis.

Gene chip analysis_All_probe_FDR_FC

ProbeID	Genesymbc	logFC	P.Value	adj.P.Val
11762533_	RAP1A	2.560714	1.57E-11	4.66E-07
11722728_	EGR2	2.805736	1.94E-11	4.66E-07
11721291_	F3	2.286221	2.86E-11	4.66E-07
11744683_	FKBP4	-2.00882	5.81E-11	5.84E-07
11732191_	RAD51AP1	-2.34297	6.62E-11	5.84E-07
11728696_	DUT	-1.87905	8.46E-11	5.84E-07
11718482_	UBE2N	-2.05145	1.08E-10	5.84E-07
11719292_	ITGB1BP1	-1.98742	1.21E-10	5.84E-07
11717144_	LANCL1	-1.71481	1.43E-10	5.84E-07
11716468_	ISCA1	-1.78934	1.46E-10	5.84E-07
11743190_	RAD51AP1	-2.30509	1.60E-10	5.84E-07
11728376_	NMU	-1.86591	1.63E-10	5.84E-07
11729129_	CPA4	1.88552	1.70E-10	5.84E-07
11722850_	BIRC3	2.213271	2.07E-10	5.84E-07
11721138_	LSM6	-2.02355	2.07E-10	5.84E-07
11723199_	HNRNPA3 /	-2.45409	2.11E-10	5.84E-07
11729128_	CPA4	1.696111	2.27E-10	5.84E-07
11726583_	CKAP4	-1.66332	2.32E-10	5.84E-07
11720825_	JAG1	1.691126	2.36E-10	5.84E-07
11748367_	GLS	2.002125	2.64E-10	5.84E-07
11727523_	ZNF267	1.982918	2.85E-10	5.84E-07
11727522_	ZNF267	2.015084	2.86E-10	5.84E-07
11742698_	SCG5	2.436254	2.89E-10	5.84E-07
11727181_	SKIL	1.631591	2.90E-10	5.84E-07
11734549_	TGFBI	1.590836	3.10E-10	5.84E-07
11727131_	CPD	-1.86291	3.11E-10	5.84E-07
11736404_	IAH1	-2.43885	3.42E-10	6.02E-07
11721137_	LSM6	-1.81302	3.46E-10	6.02E-07
11715743_	RCC2	-1.46826	3.57E-10	6.02E-07
11728377_	NMU	-2.09142	3.72E-10	6.06E-07
11717142_	LANCL1	-1.45388	3.93E-10	6.20E-07
11747886_	RAD51AP1	-1.94129	4.11E-10	6.28E-07
11758564_	NUDT15	-2.71292	4.40E-10	6.43E-07
11722851_	BIRC3	2.055566	4.52E-10	6.43E-07
11736240_	AAED1	-1.59724	4.83E-10	6.43E-07
11738800_	BRWD3	1.514104	4.96E-10	6.43E-07
11718483_	UBE2N	-2.13635	4.98E-10	6.43E-07
11727111_	FNBP1	-1.7203	5.00E-10	6.43E-07
11722852_	BIRC3	1.980963	5.13E-10	6.43E-07
11718397_	JUN	1.604618	5.84E-10	7.06E-07
11754869_	ZNF267	1.925751	6.08E-10	7.06E-07
11723246_	SFRP1	-1.38705	6.21E-10	7.06E-07
11715441_	CTGF	1.464066	6.32E-10	7.06E-07
11721314_	ESYT2	-1.60356	6.64E-10	7.06E-07
11720293_	LIN28B	1.830698	6.74E-10	7.06E-07
11732997_	MCM6	-1.49277	6.79E-10	7.06E-07
11738629_	PXYLP1	-1.34024	6.96E-10	7.06E-07
11726218_	PRDM1	2.177436	7.10E-10	7.06E-07
11727023_	TMEM64	1.473207	7.10E-10	7.06E-07
11754392_	FAM208A	-2.06598	7.38E-10	7.06E-07

11719698_HSPB11	-1.59826	7.72E-10	7.06E-07
11757863_TMEM261	-1.69424	8.02E-10	7.06E-07
11716286_VAMP3	-1.68183	8.08E-10	7.06E-07
11747517_FAM45A //	-1.53299	8.13E-10	7.06E-07
11743290_GALNT1	-1.69803	8.19E-10	7.06E-07
11754659_TPD52	-1.7446	8.22E-10	7.06E-07
11754082_LOC100128	-1.41945	8.34E-10	7.06E-07
11721290_F3	2.371637	8.38E-10	7.06E-07
11717900_ASF1B	-1.53539	8.57E-10	7.10E-07
11720926_POLR2G	-1.8429	8.86E-10	7.22E-07
11720893_SOS1	-1.32808	9.02E-10	7.23E-07
11756600_TPD52	-1.75324	9.23E-10	7.26E-07
11743582_EEF1E1	-1.48121	9.44E-10	7.26E-07
11719242_CAMK2N1	-1.65666	9.57E-10	7.26E-07
11758138_MDC1	-1.92731	9.88E-10	7.26E-07
11755242_SKIL	1.659653	9.90E-10	7.26E-07
11745250_TMEM40	1.642201	1.02E-09	7.26E-07
11743816_S1PR1	1.574513	1.04E-09	7.26E-07
11716890_TMEM261	-1.67221	1.04E-09	7.26E-07
11730012_KBTBD8	1.491232	1.05E-09	7.26E-07
11725054_HNMT	1.49482	1.06E-09	7.26E-07
11739535_FN1	1.425989	1.07E-09	7.26E-07
11715967_NEDD8	-1.40787	1.09E-09	7.32E-07
11715440_CTGF	1.408882	1.13E-09	7.44E-07
11749460_FN1	1.486691	1.15E-09	7.44E-07
11717481_FKBP10	-1.5071	1.16E-09	7.44E-07
11731919_TMPO	-1.28971	1.20E-09	7.56E-07
11738892_TPM1	1.484563	1.21E-09	7.56E-07
11755733_F3	1.557632	1.25E-09	7.65E-07
11745205_TPD52	-1.78977	1.28E-09	7.65E-07
11728294_ICT1	-2.74337	1.29E-09	7.65E-07
11727152_PRICKLE1	1.395967	1.30E-09	7.65E-07
11751629_ASF1B	-1.63621	1.30E-09	7.65E-07
11715649_TXNRD1	-1.32833	1.32E-09	7.67E-07
11716210_CITED2	1.381797	1.33E-09	7.67E-07
11758139_SDC1	-1.42937	1.39E-09	7.89E-07
11716854_IMPAD1	-1.55509	1.43E-09	8.01E-07
11716291_HNRNPA0	-1.41591	1.48E-09	8.21E-07
11743151_GLS	2.230645	1.54E-09	8.45E-07
11746931_INHBA	1.339773	1.59E-09	8.61E-07
11719273_UBE2D3	-1.44519	1.63E-09	8.64E-07
11741553_SKIL	1.681688	1.63E-09	8.64E-07
11729643_TPD52	-1.78489	1.65E-09	8.64E-07
11715493_CYR61	1.294509	1.67E-09	8.64E-07
11758201_IMPAD1	-1.70444	1.70E-09	8.64E-07
11757472_NEDD8	-1.41674	1.76E-09	8.64E-07
11754334_EGR1	1.24662	1.77E-09	8.64E-07
11719272_UBE2D3	-1.40285	1.78E-09	8.64E-07
11717143_LANCL1	-1.99671	1.78E-09	8.64E-07
11716875_C14orf142	-1.68807	1.80E-09	8.64E-07
11741409_RASAL2	1.370064	1.81E-09	8.64E-07

11718859_NUDCD3	-1.30287	1.81E-09	8.64E-07
11716066_SLC37A4	-1.37108	1.82E-09	8.64E-07
11719738_DCP2	1.256685	1.86E-09	8.72E-07
11725999_FAS	1.410714	1.87E-09	8.72E-07
11746100_ZCCHC17	-1.32097	1.92E-09	8.83E-07
11728388_TSPAN2	2.077624	1.94E-09	8.83E-07
11724682_TGFBR2	1.252381	1.97E-09	8.83E-07
11715435_TIMP3	-1.82034	1.97E-09	8.83E-07
11715862_CDIPT	-1.4334	2.00E-09	8.83E-07
11742309_TPM1	1.425303	2.01E-09	8.83E-07
11715968_LOC100128	-1.53268	2.07E-09	9.04E-07
11718856_NUDCD3	-1.8247	2.09E-09	9.04E-07
11757835_CPEB4	1.194847	2.12E-09	9.09E-07
11748534_DDAH1	1.202538	2.15E-09	9.09E-07
11739536_FN1	1.522518	2.16E-09	9.09E-07
11739973_NUAK1	1.273029	2.19E-09	9.15E-07
11728464_ZNF562	1.234157	2.27E-09	9.22E-07
11751122_SDC2	1.264935	2.30E-09	9.22E-07
11745837_FN1	1.468364	2.30E-09	9.22E-07
11741015_CDCA8	-1.25974	2.33E-09	9.22E-07
11722188_MTDH	-1.52273	2.33E-09	9.22E-07
11718400_PNP	-1.39573	2.34E-09	9.22E-07
11725585_PI15	1.602631	2.37E-09	9.22E-07
11743618_ITGA2	1.17013	2.37E-09	9.22E-07
11751032_SLC37A4	-1.32214	2.38E-09	9.22E-07
11721365_IREB2	1.283582	2.42E-09	9.31E-07
11716058_EFTUD2	-1.15262	2.44E-09	9.31E-07
11718394_JUN	1.807433	2.49E-09	9.45E-07
11749444_CPA4	1.659507	2.53E-09	9.45E-07
11719062_SESN1	1.204481	2.55E-09	9.45E-07
11718939_TNFAIP3	1.352158	2.56E-09	9.45E-07
11716509_AKR1A1	-1.56912	2.57E-09	9.45E-07
11715424_BCAP31	-1.23835	2.62E-09	9.54E-07
11746594_MCM2	-1.18464	2.67E-09	9.68E-07
11757597_YKT6	-1.27342	2.70E-09	9.71E-07
11723752_MEOX2	-1.29207	2.74E-09	9.77E-07
11756504_LOC728715	-1.2035	2.88E-09	1.02E-06
11733456_SP4	-1.37495	2.95E-09	1.04E-06
11736249_KIAA0101	-2.08538	2.99E-09	1.04E-06
11755871_TNRC6A	1.138819	3.01E-09	1.04E-06
11732833_C9orf135	1.429245	3.05E-09	1.04E-06
11734735_FAR1	-1.82599	3.06E-09	1.04E-06
11729641_TPD52	-1.67319	3.07E-09	1.04E-06
11750416_TXNRD1	-1.41606	3.12E-09	1.05E-06
11746744_TBC1D4	1.148723	3.13E-09	1.05E-06
11722999_UBE2D3	-1.16665	3.16E-09	1.05E-06
11750805_LIMCH1	1.219247	3.17E-09	1.05E-06
11749965_ZCCHC17	-1.21687	3.23E-09	1.06E-06
11726597_DUT	-1.81677	3.26E-09	1.06E-06
11721803_BUB3	-1.31157	3.28E-09	1.06E-06
11724951_CPEB2	1.347656	3.33E-09	1.07E-06

11725385_UGCG	1.274149	3.38E-09	1.07E-06
11725335_MZT1	-1.09645	3.38E-09	1.07E-06
11715423_BCAP31	-1.2857	3.46E-09	1.08E-06
11735016_NEDD4	1.184825	3.46E-09	1.08E-06
11741535_TMEM2	1.208098	3.48E-09	1.08E-06
11733030_AIDA	-1.37495	3.51E-09	1.08E-06
11725752_FBXW7	1.12302	3.52E-09	1.08E-06
11723200_HNRNPA3	-1.14543	3.60E-09	1.10E-06
11731496_INHBA	1.492949	3.61E-09	1.10E-06
11758221_ARRDC3	1.317271	3.76E-09	1.12E-06
11715742_RCC2	-1.42642	3.77E-09	1.12E-06
11755475_MTDH	-1.1743	3.78E-09	1.12E-06
11728479_CCDC181	1.168326	3.78E-09	1.12E-06
11718747_PCGF2	-1.15431	3.81E-09	1.12E-06
11716753_GNS	-1.36042	3.83E-09	1.12E-06
11727586_NUDT12	-1.18286	3.84E-09	1.12E-06
11755928_MMP1	1.556457	3.87E-09	1.12E-06
11718913_PDZD2	-1.26781	3.92E-09	1.13E-06
11755315_RNPEP	-1.18903	3.94E-09	1.13E-06
11757792_NUDCD3	-1.26828	3.97E-09	1.13E-06
11725155_FJX1	1.099549	4.05E-09	1.14E-06
11716064_SLC37A4	-1.41901	4.11E-09	1.15E-06
11753877_RRM2B	1.193835	4.22E-09	1.17E-06
11741536_TMEM2	1.105286	4.22E-09	1.17E-06
11730774_TMEM14A	-1.10077	4.24E-09	1.17E-06
11715422_BCAP31	-1.23642	4.30E-09	1.18E-06
11721428_TBX3	1.253106	4.38E-09	1.20E-06
11719634_KLF4	1.600992	4.43E-09	1.20E-06
11719525_DICER1	1.065973	4.51E-09	1.20E-06
11719739_DCP2	1.229366	4.54E-09	1.20E-06
11744519_POLE2	-1.1375	4.54E-09	1.20E-06
11733671_EYA1	-1.25716	4.54E-09	1.20E-06
11747500_AP1M1	-1.18684	4.55E-09	1.20E-06
11718761_WLS	-1.18656	4.61E-09	1.21E-06
11729996_SLFN12	1.036762	4.64E-09	1.21E-06
11756746_AKIP1 ///	1.498723	4.66E-09	1.21E-06
11726013_AJUBA	1.044404	4.71E-09	1.22E-06
11726217_PRDM1	1.494225	4.73E-09	1.22E-06
11754176_IL24	1.108943	4.77E-09	1.22E-06
11742942_UBE2D3	-1.33583	4.84E-09	1.23E-06
11716065_SLC37A4	-1.2522	4.88E-09	1.23E-06
11716417_UNG	-1.14636	4.89E-09	1.23E-06
11758478_CDCA7	-1.39805	4.92E-09	1.23E-06
11732927_KLRC1	1.146818	4.94E-09	1.23E-06
11755137_CCDC109B	-2.85304	5.02E-09	1.24E-06
11728362_KLHL28	1.314049	5.17E-09	1.25E-06
11741105_FHL2	1.062448	5.19E-09	1.25E-06
11734681_ATG4B	-1.20183	5.22E-09	1.25E-06
11754063_ASF1B	-1.38095	5.23E-09	1.25E-06
11736279_MUC7	1.349366	5.24E-09	1.25E-06
11745006_SDC2	1.023397	5.26E-09	1.25E-06

11720421_AFF4	1.477324	5.29E-09	1.25E-06
11715650_TXNRD1	-1.42105	5.31E-09	1.25E-06
11736013_GLE1	-2.25634	5.32E-09	1.25E-06
11741191_BCAP31	-1.21218	5.33E-09	1.25E-06
11717428_GNB1	-1.15239	5.34E-09	1.25E-06
11747751_SLC2A14 /	1.049646	5.35E-09	1.25E-06
11720799_RAB8B	1.031037	5.38E-09	1.25E-06
11756633_LIMCH1	1.250031	5.41E-09	1.25E-06
11746889_CKAP4	-1.42137	5.42E-09	1.25E-06
11725998_FAS	1.324483	5.44E-09	1.25E-06
11751154_ALDOC	-2.09317	5.47E-09	1.25E-06
11721647_CTNND1 //	-1.23713	5.54E-09	1.26E-06
11723247_SFRP1	-1.4532	5.59E-09	1.26E-06
11756207_PSME3	-1.173	5.63E-09	1.26E-06
11757451_FKBP10	-1.38028	5.63E-09	1.26E-06
11744985_FNDC3A	1.08729	5.66E-09	1.26E-06
11736729_RIMS2	1.800628	5.72E-09	1.27E-06
11729320_SOCS5	1.005724	5.72E-09	1.27E-06
11717658_CD109	-1.19402	5.78E-09	1.27E-06
11753244_BDNF	1.252654	5.81E-09	1.27E-06
11737807_ABCB9	1.059731	5.84E-09	1.27E-06
11721516_SUDS3	-1.12141	5.85E-09	1.27E-06
11719697_HSPB11	-1.59606	5.85E-09	1.27E-06
11740430_FAM208A	-1.87361	5.89E-09	1.27E-06
11716949_TM9SF3	-1.17454	5.91E-09	1.27E-06
11717754_SEC14L1	1.172693	6.01E-09	1.27E-06
11734006_IRAK1	-1.29992	6.05E-09	1.27E-06
11743197_TLR4	1.238289	6.06E-09	1.27E-06
11741116_FLG	2.12643	6.06E-09	1.27E-06
11726727_NABP1	1.261278	6.07E-09	1.27E-06
11724461_ALDOC	-2.05563	6.13E-09	1.28E-06
11736429_STYX	-1.03868	6.14E-09	1.28E-06
11724162_GXYLT1	1.298406	6.17E-09	1.28E-06
11722970_CREB5	1.448751	6.31E-09	1.29E-06
11742993_PSAT1	-1.06813	6.32E-09	1.29E-06
11757718_MTDH	-1.20957	6.35E-09	1.29E-06
11723095_CYLD	1.250768	6.38E-09	1.29E-06
11722422_PTRHD1	-1.08438	6.40E-09	1.29E-06
11716633_TMEM50A	-1.81101	6.40E-09	1.29E-06
11745361_CRNDE	-1.33543	6.43E-09	1.29E-06
11720796_NUP58	-1.33637	6.47E-09	1.30E-06
11743617_ITGA2	1.176164	6.57E-09	1.31E-06
11748382_SMURF2	1.049981	6.60E-09	1.31E-06
11718250_TBC1D13	-1.30133	6.61E-09	1.31E-06
11716226_LIMA1	1.009432	6.67E-09	1.31E-06
11718395_JUN	1.501369	6.75E-09	1.33E-06
11751115_GDA	1.37844	6.80E-09	1.33E-06
11746798_SDC2	1.254496	6.84E-09	1.33E-06
11715460_DHCR24	-1.18701	6.84E-09	1.33E-06
11734395_KLRC3	1.528837	6.89E-09	1.33E-06
11718773_SMARCE1	-1.1474	6.95E-09	1.34E-06

11728358_FAM13B	1.022867	7.07E-09	1.36E-06
11757679_SMAD5	-1.07808	7.22E-09	1.36E-06
11735765_RAB30	1.204431	7.22E-09	1.36E-06
11753695_UBE2N	-1.64673	7.23E-09	1.36E-06
11723198_HNRNPA3 /	-1.24845	7.25E-09	1.36E-06
11747079_ALDOC	-1.92141	7.25E-09	1.36E-06
11752545_PDLIM5	1.029046	7.29E-09	1.37E-06
11755494_SLC37A4	-1.36527	7.35E-09	1.37E-06
11755762_TMEM2	1.221994	7.36E-09	1.37E-06
11719162_GPT2	-1.3541	7.45E-09	1.38E-06
11717546_MPRIP	-1.09344	7.46E-09	1.38E-06
11732192_RAD51AP1	-1.91509	7.58E-09	1.39E-06
11756203_KIAA1143	-1.07493	7.66E-09	1.40E-06
11722971_CREB5	1.339072	7.68E-09	1.40E-06
11719368_MAPKAPK2	-1.45042	7.73E-09	1.40E-06
11719009_PAPOLA	-1.08228	7.78E-09	1.41E-06
11731445_ANKRD1	2.121436	7.88E-09	1.42E-06
11741023_SMAD5	-1.12908	7.91E-09	1.42E-06
11757924_SIPA1L2	1.110624	8.01E-09	1.43E-06
11745007_SDC2	1.195398	8.03E-09	1.43E-06
11733997_SERPINB7	1.283482	8.08E-09	1.44E-06
11755094_MEX3D	-1.58483	8.16E-09	1.44E-06
11743640_FNDC3A	1.044654	8.18E-09	1.44E-06
11751214_RGS7	1.230603	8.19E-09	1.44E-06
11720367_TMEM2	1.171012	8.25E-09	1.44E-06
11748678_F2R	1.15484	8.27E-09	1.44E-06
11749964_ZCCHC17	-1.19067	8.29E-09	1.44E-06
11715442_CTGF	1.192167	8.39E-09	1.45E-06
11739534_FN1	1.480188	8.47E-09	1.46E-06
11736289_PTPRG	1.081618	8.73E-09	1.50E-06
11739367_ASPH	-1.01647	8.85E-09	1.52E-06
11739524_MPRIP	-1.145	9.00E-09	1.54E-06
11747210_SRI	-1.23283	9.03E-09	1.54E-06
11733187_IL7R	1.324521	9.05E-09	1.54E-06
11725399_MLLT1	-1.22383	9.10E-09	1.54E-06
11751647_IL7R	1.346633	9.16E-09	1.54E-06
11735174_IL32	1.206563	9.16E-09	1.54E-06
11726533_CCDC148	1.125962	9.18E-09	1.54E-06
11718396_JUN	1.305371	9.25E-09	1.54E-06
11725418_LPCAT2	1.090987	9.27E-09	1.54E-06
11739798_LY6K	-1.64638	9.46E-09	1.57E-06
11749167_PXYLP1	-1.07792	9.55E-09	1.58E-06
11717860_EGR1	1.59349	9.67E-09	1.59E-06
11752081_EPT1	1.059938	9.70E-09	1.59E-06
11744021_CHD6	-1.07831	9.83E-09	1.61E-06
11728285_RBMS2	-1.09223	9.95E-09	1.62E-06
11755387_SACM1L	-1.18623	1.02E-08	1.64E-06
11763263_SRSF11	-0.95516	1.02E-08	1.64E-06
11743620_WHAMM	1.052752	1.02E-08	1.64E-06
11747314_PDP1	1.026887	1.02E-08	1.64E-06
11740354_EFTUD2	-1.15491	1.02E-08	1.64E-06

11754081_NEDD8	-1.41048	1.03E-08	1.65E-06
11744835_CBS /// C	-1.03463	1.04E-08	1.65E-06
11751520_NCBP2	-1.1002	1.04E-08	1.65E-06
11717901_ZCCHC17	-1.25715	1.05E-08	1.66E-06
11741897_MMP1	1.786453	1.06E-08	1.66E-06
11747208_MYSM1	1.153593	1.06E-08	1.67E-06
11757650_FN1	1.230318	1.07E-08	1.67E-06
11755281_TGFBI	1.326932	1.07E-08	1.67E-06
11758219_RRM2	-1.26332	1.08E-08	1.67E-06
11729319_SOCS5	1.068798	1.08E-08	1.67E-06
11726289_GRAMD3	1.194749	1.08E-08	1.67E-06
11748024_CLK4	1.136486	1.09E-08	1.69E-06
11757735_DNAJB9	1.0997	1.10E-08	1.69E-06
11728863_GINS1	-1.01129	1.10E-08	1.69E-06
11722424_NEDD4L	0.922539	1.13E-08	1.72E-06
11743055_PLAA	-1.2163	1.13E-08	1.72E-06
11758659_TBCA	-0.95343	1.14E-08	1.72E-06
11758329_SDHD	-1.01572	1.14E-08	1.73E-06
11734550_TGFBI	1.330522	1.15E-08	1.73E-06
11715690_CD164	1.003711	1.16E-08	1.75E-06
11758499_DTL	-0.95741	1.18E-08	1.76E-06
11736555_SLC2A14 /	1.489045	1.19E-08	1.78E-06
11718503_GPCPD1	0.911564	1.20E-08	1.78E-06
11753421_CITED2	1.351726	1.21E-08	1.80E-06
11728799_IMMP2L	1.096187	1.24E-08	1.83E-06
11717844_ETNK1	0.985116	1.25E-08	1.85E-06
11734548_TGFBI	1.324458	1.27E-08	1.87E-06
11724902_MAP3K14	2.131354	1.27E-08	1.87E-06
11717925_YAP1	0.955398	1.27E-08	1.87E-06
11724635_YOD1	1.172924	1.28E-08	1.87E-06
11722930_GART	-0.94555	1.31E-08	1.91E-06
11717399_TIPARP	0.939704	1.33E-08	1.93E-06
11748938_SLC37A4	-1.15123	1.34E-08	1.93E-06
11718914_PDZD2	-1.00525	1.34E-08	1.93E-06
11758842_THBS1	1.085016	1.34E-08	1.93E-06
11745912_PELI1	1.101371	1.35E-08	1.93E-06
11747323_NCBP2	-1.03481	1.36E-08	1.95E-06
11718285_MRPL45	-1.06001	1.38E-08	1.95E-06
11757365_MRPL45	-1.06001	1.38E-08	1.95E-06
11729451_PCLO	1.109097	1.38E-08	1.96E-06
11725722_ATAD2	-1.06149	1.41E-08	1.99E-06
11718183_CDCA5	-0.99564	1.41E-08	1.99E-06
11724218_GPATCH2	1.22186	1.42E-08	1.99E-06
11751230_PSAT1	-1.14679	1.42E-08	1.99E-06
11734657_SLC2A14 /	1.426013	1.44E-08	2.00E-06
11718772_SMARCE1	-1.15897	1.44E-08	2.00E-06
11751287_MMP19	1.476038	1.45E-08	2.00E-06
11716031_ARF3	-1.35197	1.45E-08	2.00E-06
11742939_UBE2D3	-1.04426	1.46E-08	2.00E-06
11720768_METTL9	-1.29076	1.46E-08	2.00E-06
11739734_CPEB4	1.254328	1.46E-08	2.00E-06

11716917_DDAH1	1.126727	1.46E-08	2.00E-06
11742961_FAM43A	0.941903	1.47E-08	2.00E-06
11741317_NHLH2	1.144577	1.49E-08	2.03E-06
11758795_MRPL45	-0.9203	1.50E-08	2.03E-06
11716781_TBL1XR1	-1.06582	1.50E-08	2.03E-06
11721142_MKI67	-0.97229	1.51E-08	2.03E-06
11724963_FBXO5	-0.97187	1.51E-08	2.03E-06
11738542_SERPINB7	1.012355	1.51E-08	2.03E-06
11744763_ASNS	-0.93429	1.52E-08	2.03E-06
11721093_THBS1	0.963783	1.52E-08	2.03E-06
11740173_SOCS5	0.921341	1.52E-08	2.03E-06
11760482_NDUFS7	-0.99823	1.53E-08	2.03E-06
11734226_LIMCH1	1.229477	1.54E-08	2.04E-06
11756403_KCTD10	-1.02549	1.55E-08	2.05E-06
11717903_IER3IP1	-0.87732	1.56E-08	2.06E-06
11750804_LIMCH1	1.285462	1.57E-08	2.06E-06
11715484_MCL1	1.123623	1.57E-08	2.06E-06
11724357_MYO5A	0.92019	1.58E-08	2.06E-06
11728936_KIAA1549L	-1.13948	1.59E-08	2.06E-06
11716365_ASNS	-0.9364	1.59E-08	2.06E-06
11716630_ANGPTL4	1.394684	1.61E-08	2.08E-06
11723821_SMURF2	0.890195	1.63E-08	2.10E-06
11727880_CHAF1A	-0.92267	1.65E-08	2.12E-06
11739328_CENPH	-1.17033	1.65E-08	2.12E-06
11718675_CTNNAL1 /	0.953206	1.66E-08	2.13E-06
11746586_NDUFS7	-1.04333	1.67E-08	2.14E-06
11719531_MMP19	1.647633	1.69E-08	2.16E-06
11729132_PSME3	-1.18409	1.71E-08	2.17E-06
11753752_RRM2B	1.191991	1.74E-08	2.20E-06
11745871_PAPOLA	-1.01793	1.74E-08	2.20E-06
11718724_ARRDC3	1.323833	1.75E-08	2.20E-06
11736194_RRM2B	1.266278	1.75E-08	2.20E-06
11728390_TSPAN2	2.058193	1.75E-08	2.20E-06
11716483_GRINA	-0.97584	1.76E-08	2.21E-06
11719926_RBM12	-1.15036	1.77E-08	2.21E-06
11745803_TPM1	0.988051	1.78E-08	2.21E-06
11744338_COPS7A	-1.12318	1.79E-08	2.22E-06
11720075_SLC35F5	0.975612	1.80E-08	2.22E-06
11749905_CYR61	1.02014	1.80E-08	2.22E-06
200011_PMARF3	-1.2731	1.80E-08	2.22E-06
11743444_RHOB	1.064641	1.81E-08	2.22E-06
11720570_JADE2	-0.94977	1.81E-08	2.22E-06
11748213_TSC22D2	0.892633	1.83E-08	2.24E-06
11728296_RNMT	1.081597	1.84E-08	2.25E-06
11733126_PRIM1	-1.00147	1.85E-08	2.25E-06
11722244_TMEM27	1.361277	1.86E-08	2.25E-06
11725077_RIT1	0.919395	1.86E-08	2.25E-06
11757504_DDAH1	0.975184	1.86E-08	2.25E-06
11730731_DNAJB9	1.188017	1.86E-08	2.25E-06
11734458_FAM160B1	1.730976	1.87E-08	2.25E-06
11717843_ETNK1	0.940586	1.88E-08	2.25E-06

11717817_TNRC6A	1.024992	1.89E-08	2.26E-06
11734659_FOS	1.278136	1.90E-08	2.27E-06
11747091_TMPO	-0.98308	1.90E-08	2.27E-06
11716059_EFTUD2	-0.9819	1.91E-08	2.27E-06
11758254_SRI	-1.37923	1.91E-08	2.27E-06
11717842_ETNK1	1.018458	1.92E-08	2.27E-06
11749240_GRAMD3	1.245897	1.94E-08	2.29E-06
11740238_SULF2	1.035723	1.96E-08	2.30E-06
11731848_KLRC1 ///	1.442204	1.97E-08	2.31E-06
11716285_VAMP3	-1.66124	1.98E-08	2.32E-06
11726191_ZNF45	0.894089	1.99E-08	2.32E-06
11731321_GLE1	-1.44709	2.00E-08	2.32E-06
11715331_DSN1	-0.99546	2.00E-08	2.32E-06
11755541_NUP58	-1.20656	2.00E-08	2.32E-06
11741252_RCC1	-0.94571	2.01E-08	2.33E-06
11726515_CLK4	1.028135	2.03E-08	2.34E-06
11727440_LOXL4	1.097632	2.04E-08	2.35E-06
11728714_THOC6	-0.91467	2.04E-08	2.35E-06
11716384_CCL2	1.258282	2.05E-08	2.35E-06
11739335_SCYL2	0.846999	2.06E-08	2.36E-06
11755850_ZBTB10	0.995717	2.08E-08	2.37E-06
11745893_MTHFD2	-0.87037	2.09E-08	2.37E-06
11716728_TPM1	0.869452	2.09E-08	2.37E-06
11745289_LIMA1	0.853427	2.10E-08	2.38E-06
11751822_MCM6	-1.37298	2.11E-08	2.38E-06
11743815_S1PR1	1.411802	2.12E-08	2.39E-06
11732240_SLITRK6	0.924962	2.13E-08	2.39E-06
11726726_NABP1	0.953479	2.14E-08	2.39E-06
11744003_MTHFD2	-0.91799	2.14E-08	2.39E-06
11755581_LATS2	1.045941	2.14E-08	2.39E-06
11749349_POLR2C	-0.96881	2.15E-08	2.39E-06
11729250_B3GALT2	1.704898	2.15E-08	2.39E-06
11723062_NDUFS7	-0.9469	2.17E-08	2.41E-06
11721091_THBS1	0.954428	2.18E-08	2.41E-06
11748015_VDAC3	-0.87876	2.19E-08	2.43E-06
11718247_KIAA0430	-1.09164	2.22E-08	2.44E-06
11718719_FOCAD	-0.94904	2.22E-08	2.44E-06
11725375_HAPLN1	0.878046	2.23E-08	2.44E-06
11724741_HMGN2	-0.90692	2.23E-08	2.44E-06
11731834_IL24	0.919617	2.23E-08	2.44E-06
11746797_SDC2	1.315075	2.24E-08	2.45E-06
11763170_FOSL2	0.83965	2.25E-08	2.45E-06
11745211_ZNF33B	1.028141	2.26E-08	2.45E-06
11748793_ASNS	-0.88231	2.26E-08	2.45E-06
11722230_PDP1	0.875071	2.26E-08	2.45E-06
11716364_ASNS	-0.93624	2.27E-08	2.45E-06
11738893_TPM1	0.8606	2.28E-08	2.45E-06
11742940_UBE2D3	-1.02437	2.28E-08	2.45E-06
11720780_KIAA1324	1.473427	2.29E-08	2.45E-06
11752770_SMARCE1	-1.11519	2.29E-08	2.45E-06
11743135_CEBPD	1.140837	2.35E-08	2.51E-06

11725148_C9orf40	-1.14268	2.37E-08	2.52E-06
11721517_SUDS3	-1.10654	2.37E-08	2.52E-06
11720795_NUP58	-1.34441	2.38E-08	2.52E-06
11732651_ZNF571	1.234574	2.40E-08	2.54E-06
11718774_SMARCE1	-0.85104	2.42E-08	2.55E-06
11732312_ANKRD13C	0.872399	2.42E-08	2.55E-06
11717000_GUCD1	-1.04824	2.45E-08	2.57E-06
11758584_STYX	-0.94221	2.46E-08	2.58E-06
11726536_CYP1A1	1.155746	2.48E-08	2.59E-06
11736014_GLE1	-1.8898	2.48E-08	2.59E-06
11749332_EBP	-0.8316	2.52E-08	2.63E-06
11757047_ATF4	-0.8126	2.54E-08	2.64E-06
11715689_CD164	0.953542	2.55E-08	2.64E-06
11741357_FNDC3A	0.908864	2.55E-08	2.64E-06
11758303_ZCCHC17	-1.14468	2.56E-08	2.64E-06
11737718_EEF1E1 //	-0.96732	2.56E-08	2.64E-06
11723282_CEACAM1	0.851714	2.62E-08	2.69E-06
11750321_F2RL2	1.224693	2.66E-08	2.73E-06
11731351_NF1	0.975645	2.68E-08	2.75E-06
11715691_ZFP36	1.029986	2.71E-08	2.77E-06
11745543_AJUBA	0.88605	2.71E-08	2.77E-06
11737591_MYSM1	0.89104	2.77E-08	2.82E-06
11726619_C1GALT1C1	-0.79931	2.78E-08	2.82E-06
11724462_ALDOC	-1.72543	2.81E-08	2.85E-06
11739081_DNAJB4	0.910705	2.82E-08	2.85E-06
11742890_MND1	-1.01792	2.82E-08	2.85E-06
11728569_GSTCD	-1.11541	2.83E-08	2.85E-06
11743682_KLF10	1.202342	2.84E-08	2.85E-06
11759291_PSMD12	-0.97257	2.84E-08	2.85E-06
11722605_CDC37L1	1.058229	2.85E-08	2.85E-06
11725211_PLCXD1	-0.96779	2.85E-08	2.85E-06
11733061_PELI1	0.871993	2.87E-08	2.87E-06
11754510_VDAC3	-0.90416	2.88E-08	2.87E-06
11719699_CDC45	-1.00309	2.89E-08	2.87E-06
11716136_COX7A2 //	-1.33362	2.90E-08	2.88E-06
11757430_TMED5	0.852585	2.91E-08	2.88E-06
11754075_SMARCE1	-0.82625	2.95E-08	2.91E-06
11743808_DHCR7	-1.11543	2.97E-08	2.93E-06
11753980_PXYLP1	-1.28628	2.98E-08	2.93E-06
11720157_GDA	0.936446	2.99E-08	2.93E-06
11724949_WDR35	0.900969	2.99E-08	2.93E-06
11726193_ 4-Mar	0.852631	3.04E-08	2.96E-06
11729170_DUSP10	0.80749	3.05E-08	2.96E-06
11723709_CRY1	0.997313	3.05E-08	2.96E-06
11732419_TYW5	1.13982	3.05E-08	2.96E-06
11753951_PSPC1	0.869322	3.06E-08	2.96E-06
11757584_FNDC3A	0.927254	3.07E-08	2.97E-06
11725126_TMEM147	-0.88309	3.11E-08	3.00E-06
11749158_PPP2R1A	-0.81216	3.11E-08	3.00E-06
11726883_ATAD2B	1.095555	3.11E-08	3.00E-06
11751338_DHCR24	-0.91667	3.14E-08	3.02E-06

11719614_LARP4	-1.09913	3.16E-08	3.03E-06
11761349_TPM1	0.979443	3.18E-08	3.03E-06
11749620_FAM45A //	-1.25764	3.18E-08	3.03E-06
11749139_SEMA3C	0.945301	3.22E-08	3.07E-06
11758571_NEDD4L	0.8344	3.23E-08	3.07E-06
11725751_FBXW7	1.007685	3.23E-08	3.07E-06
11744946_AIDA	-1.34008	3.24E-08	3.07E-06
11758649_FBXW11	-0.9584	3.27E-08	3.08E-06
11717039_GADD45A	0.872746	3.28E-08	3.08E-06
11731889_EDA2R	1.083311	3.29E-08	3.08E-06
11747253_UNG	-0.88763	3.29E-08	3.08E-06
11751962_GPR37	1.23251	3.29E-08	3.08E-06
11722606_CDC37L1	0.886983	3.29E-08	3.08E-06
11744914_EID1	-1.02521	3.30E-08	3.08E-06
11720672_PPM1A	-1.04767	3.31E-08	3.08E-06
11751993_JAG1	1.706518	3.32E-08	3.09E-06
11747092_TMPO	-0.92274	3.34E-08	3.10E-06
11744002_MTHFD2	-0.84301	3.35E-08	3.10E-06
11738808_OSBPL10	0.787027	3.35E-08	3.10E-06
11719174_NT5E	0.958512	3.37E-08	3.11E-06
11758403_RAPH1	1.075754	3.38E-08	3.11E-06
11722062_REEP3	0.958738	3.38E-08	3.11E-06
11747670_WDR73	0.863724	3.39E-08	3.11E-06
11745130_HMGB3	-1.17726	3.41E-08	3.13E-06
11736889_EPHX3	1.027795	3.44E-08	3.15E-06
11755408_FAM199X	0.784992	3.45E-08	3.16E-06
11751306_FBXO28	0.852652	3.47E-08	3.16E-06
11755127_TMF1	0.788721	3.48E-08	3.16E-06
11758876_CREBRF	0.940018	3.49E-08	3.16E-06
11742991_PSAT1	-1.07397	3.50E-08	3.16E-06
11747430_ZNF260	1.08892	3.50E-08	3.16E-06
11723245_SFRP1	-1.36886	3.50E-08	3.16E-06
11733068_KLF10	1.199073	3.54E-08	3.18E-06
11727219_DSE	0.896461	3.55E-08	3.18E-06
11731770_DSE	0.896461	3.55E-08	3.18E-06
11740656_HMGA2	0.834934	3.56E-08	3.18E-06
11723192_UHRF1	-0.85701	3.56E-08	3.18E-06
11741547_NEDD4L	0.905696	3.57E-08	3.18E-06
11725419_LPCAT2	0.796444	3.57E-08	3.18E-06
11760258_KIAA1324	1.514912	3.59E-08	3.19E-06
11763585_TMPO	-1.29629	3.59E-08	3.19E-06
11727447_ST6GALNAC	1.269367	3.59E-08	3.19E-06
11732533_LMNB1	-1.14449	3.62E-08	3.20E-06
11744889_LMNB1	-1.14449	3.62E-08	3.20E-06
11722796_MAPK6	0.91536	3.63E-08	3.20E-06
11728792_EEF2K ///	-0.90434	3.64E-08	3.20E-06
11719881_OSTM1	1.195785	3.65E-08	3.21E-06
11731599_FOSL2	1.370653	3.69E-08	3.24E-06
11731918_TMPO	-0.9802	3.73E-08	3.26E-06
11715991_MCM2	-1.17322	3.73E-08	3.26E-06
11758396_POLR3G	-0.99836	3.74E-08	3.26E-06

11756773_FRMD5	1.170287	3.75E-08	3.27E-06
11752940_EGR1	1.192781	3.76E-08	3.27E-06
11729704_ZNF561	0.849067	3.77E-08	3.27E-06
11751272_ZNF562	1.115315	3.77E-08	3.27E-06
11719643_CDS1	1.22515	3.79E-08	3.27E-06
11739366_ASPH	-0.83033	3.80E-08	3.27E-06
11729450_PCLO	0.875042	3.80E-08	3.27E-06
11719615_LARP4	-1.05472	3.81E-08	3.27E-06
11718631_SERTAD1	0.896049	3.82E-08	3.27E-06
11755700_TIPARP	0.953025	3.82E-08	3.27E-06
11742941_UBE2D3	-1.01087	3.82E-08	3.27E-06
11731621_ZNF91	1.535146	3.84E-08	3.27E-06
11717959_TCF3	-0.8806	3.85E-08	3.27E-06
11732408_LMBRD2	1.088938	3.85E-08	3.27E-06
11719137_THOC1	-0.875	3.85E-08	3.27E-06
11745868_KIF2C	-0.83557	3.85E-08	3.27E-06
11741106_FHL2	1.064084	3.86E-08	3.27E-06
11745870_MTHFD2	-1.00254	3.86E-08	3.27E-06
11723365_COPS7A	-0.94809	3.89E-08	3.28E-06
11755050_PLCB4	0.865589	3.92E-08	3.30E-06
11720800_RAB8B	1.145198	3.92E-08	3.30E-06
11739369_PPM1D	0.871842	3.95E-08	3.31E-06
11722242_TSPAN12	0.850058	3.96E-08	3.32E-06
11721477_TTI1	-0.75384	3.97E-08	3.32E-06
11757989_ANKRD46	0.917917	3.98E-08	3.32E-06
11747280_DHFR	-0.95299	4.03E-08	3.36E-06
11736382_ZNF697	1.122436	4.05E-08	3.37E-06
11733188_INCENP	-1.14893	4.09E-08	3.39E-06
11752382_S1PR1	1.294161	4.09E-08	3.39E-06
11749088_PTPRK	0.902075	4.09E-08	3.39E-06
11755211_DICER1	0.933744	4.10E-08	3.39E-06
11743498_BMP2	0.935534	4.11E-08	3.39E-06
11758330_SDHD	-0.89445	4.12E-08	3.39E-06
11746580_ZNF140	0.852956	4.13E-08	3.39E-06
11753763_CDKN3	-0.81283	4.13E-08	3.39E-06
11727736_AKAP7	-1.44219	4.17E-08	3.41E-06
11742669_HIST2H4A	0.851367	4.17E-08	3.41E-06
11754575_AHNAK	-0.88765	4.17E-08	3.41E-06
11728052_FAM126B	0.829392	4.19E-08	3.41E-06
11754933_TSC22D2	0.933669	4.20E-08	3.41E-06
11716372_VPS26B	-0.83976	4.20E-08	3.41E-06
11755807_DNA2	-0.9726	4.20E-08	3.41E-06
11736367_MCM10	-0.9546	4.22E-08	3.42E-06
11716514_ADD1	-0.76629	4.22E-08	3.42E-06
11762266_IL7R	1.197025	4.28E-08	3.46E-06
11733811_FASTKD2	-0.89315	4.29E-08	3.46E-06
11760466_ATP11B	1.23195	4.30E-08	3.46E-06
11716203_MGP	1.545092	4.33E-08	3.47E-06
11731622_ZNF91	1.20309	4.33E-08	3.47E-06
11727879_CHAF1A	-0.96364	4.34E-08	3.48E-06
11752173_TMEM2	1.091955	4.36E-08	3.48E-06

11758108_EFEMP1	1.388333	4.37E-08	3.49E-06
11756134_FHL2	0.937101	4.38E-08	3.49E-06
11730052_SNX16	0.88218	4.38E-08	3.49E-06
11746378_TAB3	1.03613	4.41E-08	3.51E-06
11734858_ERO1B	0.760545	4.45E-08	3.53E-06
11719898_HBEGF	0.885659	4.50E-08	3.56E-06
11726516_CLK4	1.075228	4.53E-08	3.58E-06
11718998_DHFR	-1.16739	4.54E-08	3.58E-06
11733799_SLC31A2	0.887101	4.55E-08	3.58E-06
11730735_PPP1R8	-0.78341	4.56E-08	3.58E-06
11715524_COTL1	-0.86667	4.56E-08	3.58E-06
11725198_IL1A	1.384836	4.57E-08	3.58E-06
11728861_DCAF17	1.059709	4.57E-08	3.58E-06
11730998_ZNF654	0.771805	4.60E-08	3.60E-06
11745100_CDH19	0.922907	4.61E-08	3.60E-06
11728076_HDAC9	0.915373	4.66E-08	3.62E-06
11726840_AVL9	0.984884	4.66E-08	3.62E-06
11744848_PRR16	1.340806	4.67E-08	3.62E-06
11722601_ACAT2	-0.77229	4.68E-08	3.62E-06
11762135_PTPRK	1.25314	4.68E-08	3.62E-06
11736244_HIST1H2AC	1.12649	4.68E-08	3.62E-06
11753442_TMEM64	1.565734	4.71E-08	3.63E-06
11731503_TBC1D4	0.865345	4.72E-08	3.63E-06
11717859_EBP	-0.8999	4.73E-08	3.63E-06
11728050_FAM126B	0.920564	4.73E-08	3.63E-06
11728463_ZNF562	1.296589	4.74E-08	3.63E-06
11720082_CBX6	-0.79118	4.74E-08	3.63E-06
11754178_RRM2B	1.111318	4.75E-08	3.63E-06
11738203_IMPDH1	-0.75569	4.75E-08	3.63E-06
11718165_RBL2	-0.98253	4.78E-08	3.64E-06
11746018_GRAMD3	1.135655	4.78E-08	3.64E-06
11730876_FANCI	-0.84135	4.79E-08	3.64E-06
11741356_FNDC3A	1.069806	4.81E-08	3.65E-06
11756590_KMO	1.814892	4.85E-08	3.67E-06
11736596_GINS4	-1.02277	4.86E-08	3.67E-06
11739470_TMEM87B	0.983153	4.87E-08	3.68E-06
11723441_EPHA4	1.099622	4.88E-08	3.68E-06
11716562_AIMP2	-1.02859	4.89E-08	3.68E-06
11721473_HCCS	0.895074	4.92E-08	3.68E-06
11747404_ZNF586	0.938875	4.93E-08	3.68E-06
11725951_RIOK3	0.780293	4.93E-08	3.68E-06
11748815_RRM2B	1.047714	4.94E-08	3.68E-06
11722189_MTDH	-1.06046	4.94E-08	3.68E-06
11717163_CDC20	-0.83016	4.94E-08	3.68E-06
11758793_CCDC109B	-2.14067	4.95E-08	3.68E-06
11755569_TMEM64	1.41237	4.95E-08	3.68E-06
11739469_TMEM87B	0.950069	4.97E-08	3.69E-06
11756182_PSAT1	-1.04174	4.97E-08	3.69E-06
11742308_TPM1	0.830454	4.99E-08	3.69E-06
11726131_PKP2	0.893293	4.99E-08	3.69E-06
11749599_SEMA3C	0.883113	5.02E-08	3.70E-06

11717041_MTMR4	-1.00074	5.02E-08	3.70E-06
11718912_PDZD2	-0.98233	5.05E-08	3.70E-06
11751110_FBXL2	0.860294	5.06E-08	3.70E-06
11739733_CPEB4	1.110243	5.06E-08	3.70E-06
11726097_BAZ2B	0.963056	5.06E-08	3.70E-06
11742763_FAM214A	0.957772	5.06E-08	3.70E-06
11748732_PAPOLA	-0.97933	5.16E-08	3.77E-06
11727817_UBASH3B	0.849949	5.21E-08	3.80E-06
11740126_HIPK3	1.297123	5.22E-08	3.80E-06
11732313_ANKRD13C	0.821291	5.24E-08	3.81E-06
11726617_CDK1	-0.77143	5.26E-08	3.82E-06
11753420_RRM2B	1.095644	5.27E-08	3.82E-06
11720247_CEP55	-0.76241	5.30E-08	3.83E-06
11758770_HSPA4	-1.58876	5.30E-08	3.83E-06
11715615_BTG2	0.79696	5.30E-08	3.83E-06
11720186_MAD2L1	-0.8898	5.37E-08	3.84E-06
11722843_ENAH	-1.12792	5.37E-08	3.84E-06
11720626_HNRNPDL	-1.09364	5.37E-08	3.84E-06
11758498_DNM3	2.008691	5.38E-08	3.84E-06
11758248_SDHD	-0.93247	5.38E-08	3.84E-06
11758311_SDHD	-0.93247	5.38E-08	3.84E-06
11763834_PPP1R1C	0.796987	5.38E-08	3.84E-06
11720231_TES	1.008385	5.44E-08	3.88E-06
11716262_AP2B1	0.807502	5.50E-08	3.91E-06
11720823_JAG1	1.194996	5.50E-08	3.91E-06
11746183_RNF6	0.804743	5.52E-08	3.92E-06
11733955_SLC22A15	0.909832	5.54E-08	3.93E-06
11744598_CDCA8	-1.0981	5.60E-08	3.96E-06
11744789_CENPU	-0.79288	5.60E-08	3.96E-06
11736597_GINS4	-0.99149	5.61E-08	3.96E-06
11758515_MTDH	-1.13056	5.63E-08	3.97E-06
11722831_CCNY	-0.89459	5.65E-08	3.98E-06
11748761_KLF5	0.937221	5.68E-08	3.99E-06
11722868_RAD1	-0.81751	5.70E-08	4.01E-06
11747489_CLIP4	1.103416	5.75E-08	4.04E-06
11724516_GPD1L	-0.94157	5.77E-08	4.04E-06
11726006_SLC30A1	0.858761	5.78E-08	4.04E-06
11749228_TMEM30A	0.748892	5.81E-08	4.05E-06
11724992_GTPBP3	-0.79229	5.84E-08	4.07E-06
11716144_PDCD6IP	-1.27571	5.86E-08	4.08E-06
11751952_MYO19	-0.86947	5.87E-08	4.08E-06
11727448_ST6GALNAC	1.290284	5.89E-08	4.09E-06
11757325_CIB1	-0.95754	5.92E-08	4.11E-06
11758060_PCGF5	0.940532	5.94E-08	4.11E-06
11758629_SPTBN1	-0.86881	5.98E-08	4.13E-06
11749201_IQCJ-SCHI	0.83421	5.98E-08	4.13E-06
11736010_RSL1D1	0.743622	6.00E-08	4.14E-06
11719224_COQ10A	-0.98965	6.04E-08	4.16E-06
11752670_FADS1	-0.81502	6.05E-08	4.16E-06
11753831_CDKN3	-0.88521	6.07E-08	4.17E-06
11724021_HIST1H2BD	1.243147	6.12E-08	4.19E-06

11726288_GRAMD3	1.131322	6.13E-08	4.20E-06
11756666_MCM2	-1.00735	6.16E-08	4.21E-06
11743191_NTM	0.921893	6.20E-08	4.22E-06
11721097_TMX1	-1.12998	6.21E-08	4.22E-06
11722190_MTDH	-1.06924	6.21E-08	4.22E-06
11747288_WDR73	0.741851	6.22E-08	4.22E-06
11741159_EYA1	-1.1757	6.22E-08	4.22E-06
11739166_LYPD1	0.843332	6.23E-08	4.22E-06
11726302_DTL	-0.82824	6.27E-08	4.24E-06
11751237_DUSP10	0.813094	6.27E-08	4.24E-06
11724903_MAP3K14	1.243023	6.29E-08	4.24E-06
11723725_USP32	0.842581	6.29E-08	4.24E-06
11744888_LMNB1	-1.03555	6.32E-08	4.24E-06
11724345_ITGA4	0.818259	6.32E-08	4.24E-06
11719792_GPRC5A	0.820324	6.32E-08	4.24E-06
11744286_CBS /// C	-1.03396	6.33E-08	4.25E-06
11729916_ARL5B	1.048718	6.42E-08	4.30E-06
11724612_DYRK2	0.830598	6.45E-08	4.31E-06
11756138_HK2	-0.998	6.46E-08	4.31E-06
11718956_PRKAB2	0.983209	6.49E-08	4.33E-06
11748927_PTPRG	0.984637	6.52E-08	4.33E-06
11746154_PCGF2	-0.98898	6.52E-08	4.33E-06
11721092_THBS1	0.777119	6.53E-08	4.33E-06
11737441_ST7L	0.918071	6.54E-08	4.34E-06
11736021_FKBP4	-1.0471	6.57E-08	4.35E-06
11726247_PARP1	-0.79936	6.59E-08	4.36E-06
11740663_GXYLT1	1.188246	6.60E-08	4.36E-06
11745256_CPEB4	0.953043	6.63E-08	4.37E-06
11733060_PELI1	1.063236	6.69E-08	4.41E-06
11743841_PLCXD2	1.190414	6.75E-08	4.44E-06
11748659_EFEMP1	1.377658	6.75E-08	4.44E-06
11721644_ARL1	-0.77612	6.76E-08	4.44E-06
11726927_EPHX3	1.036245	6.82E-08	4.45E-06
11722964_AMMECR1	0.855155	6.82E-08	4.45E-06
11715812_MLLT6	-0.96881	6.82E-08	4.45E-06
11724246_TMEM127	-0.74699	6.82E-08	4.45E-06
11724037_PTGS2	1.340572	6.82E-08	4.45E-06
11758683_JADE2	-1.022	6.83E-08	4.45E-06
11721800_NAA50	-0.92474	6.84E-08	4.45E-06
11737335_ZNF283	1.00876	6.88E-08	4.46E-06
11722454_QKI	-0.84448	6.89E-08	4.46E-06
11719344_ATF3	0.842422	6.89E-08	4.46E-06
11745738_AIDA	-0.95646	6.90E-08	4.46E-06
11715347_HBB	1.619768	6.91E-08	4.46E-06
11720361_PHIP	0.951542	6.98E-08	4.50E-06
11732993_RGS4	0.960799	6.99E-08	4.50E-06
11739026_HMGB1	-0.76772	7.06E-08	4.54E-06
11748362_ABCC3	1.075634	7.09E-08	4.55E-06
11728300_CCNE2	-0.76542	7.13E-08	4.57E-06
11716878_B4GALT5	-0.71842	7.13E-08	4.57E-06
11716563_AIMP2	-1.13779	7.17E-08	4.59E-06

11743822_WHSC1	-0.89559	7.20E-08	4.60E-06
11733877_LMO7	0.899303	7.22E-08	4.61E-06
11763310_MIR21 ///	0.72984	7.23E-08	4.61E-06
11741222_FAM198B	0.930095	7.24E-08	4.61E-06
11740885_OSBPL3	0.827776	7.25E-08	4.61E-06
11723281_CREBZF	0.850188	7.27E-08	4.61E-06
11756409_BLM	-0.75211	7.32E-08	4.64E-06
11739025_HMGB1	-0.77266	7.35E-08	4.65E-06
11728156_LACC1	0.876558	7.37E-08	4.66E-06
11731369_ZNF442	0.945564	7.38E-08	4.66E-06
11719791_GPRC5A	0.755927	7.41E-08	4.67E-06
11756839_ZC3HAV1	0.815329	7.42E-08	4.67E-06
11742992_PSAT1	-1.06287	7.45E-08	4.69E-06
11747400_COPS7A	-1.10217	7.51E-08	4.71E-06
11722937_MB21D2	0.803116	7.51E-08	4.71E-06
11724727_EIF5	-1.00048	7.52E-08	4.71E-06
11722555_HADH	-0.71049	7.58E-08	4.74E-06
11749172_NEXN	0.84935	7.58E-08	4.74E-06
11730999_ZNF654	0.930514	7.59E-08	4.74E-06
11719642_CDS1	1.214724	7.63E-08	4.75E-06
11758061_LINC00472	1.411086	7.64E-08	4.75E-06
11744788_TMEM68	-1.19856	7.71E-08	4.79E-06
11731806_CHRM3	1.040894	7.72E-08	4.79E-06
11741140_NEDD4L	0.818226	7.72E-08	4.79E-06
11719571_RCHY1	1.156946	7.74E-08	4.80E-06
11721738_LATS2	1.155622	7.79E-08	4.82E-06
11725376_HAPLN1	0.808804	7.81E-08	4.83E-06
11718349_TUSC2	0.812451	7.84E-08	4.83E-06
11732652_ZNF571	1.144758	7.84E-08	4.83E-06
11745181_NAA15	-0.72653	7.84E-08	4.83E-06
11758915_YY1	-1.01409	7.86E-08	4.83E-06
11731306_CNST	0.766833	7.87E-08	4.83E-06
11736427_CLIP4	0.943048	7.91E-08	4.85E-06
11735128_TSC22D2	0.93532	7.92E-08	4.85E-06
11723112_CCDC84	0.695194	7.93E-08	4.85E-06
11733157_CCDC50	-0.72511	7.94E-08	4.85E-06
11718106_IPO5	-0.88597	7.96E-08	4.86E-06
11728128_APTX	-0.74571	7.99E-08	4.86E-06
11736603_GLG1	-0.80298	7.99E-08	4.86E-06
11743470_WDFY3	1.060438	7.99E-08	4.86E-06
11749755_ZNF791	0.809645	8.01E-08	4.86E-06
11757515_ITGAV	-0.82652	8.03E-08	4.87E-06
11717283_ASAP1	0.782181	8.06E-08	4.87E-06
11741396_GGT1 ///	1.257613	8.06E-08	4.87E-06
11749796_ERGIC1	-0.80692	8.07E-08	4.87E-06
11759124_FBXL17	-1.11442	8.07E-08	4.87E-06
11724545_HCFC2	0.924039	8.09E-08	4.87E-06
11722803_GNAI1	0.824293	8.10E-08	4.88E-06
11736477_ELL2	1.02877	8.16E-08	4.90E-06
11740054_NCBP2	-0.91585	8.19E-08	4.91E-06
11749205_PXYLP1	-0.7657	8.21E-08	4.92E-06

11758645_EIF4EBP2	-0.95113	8.25E-08	4.94E-06
11755450_ZNF608	0.909088	8.26E-08	4.94E-06
11757800_NFIB	-0.72947	8.28E-08	4.94E-06
11753558_MCM7	-0.78943	8.28E-08	4.94E-06
11723668_SEMA3C	0.686221	8.34E-08	4.97E-06
11721143_MKI67	-0.79183	8.36E-08	4.98E-06
11757970_DICER1	0.847718	8.39E-08	4.99E-06
11740685_NAPEPLD	1.215592	8.40E-08	4.99E-06
11740172_SOCS5	1.088982	8.55E-08	5.07E-06
11758388_LHX8	0.841877	8.56E-08	5.07E-06
11716155_B4GALT1	-0.7999	8.61E-08	5.09E-06
11744402_BLZF1	0.705073	8.62E-08	5.09E-06
11729169_DUSP10	0.820621	8.68E-08	5.13E-06
11715845_PTPRF	-0.74854	8.73E-08	5.14E-06
11719373_DYNLT3	-0.8274	8.74E-08	5.14E-06
11721854_NCAPH	-0.92369	8.75E-08	5.14E-06
11724274_PTPRK	0.86468	8.83E-08	5.19E-06
11717609_MEN1	-0.8623	8.85E-08	5.19E-06
11724273_PTPRK	1.031507	8.85E-08	5.19E-06
11746020_THUMPD2	0.886625	8.93E-08	5.23E-06
11724695_ZNF227	0.761373	9.00E-08	5.26E-06
11734631_BAGE2 ///	0.744333	9.01E-08	5.26E-06
11728129_APTX	-0.94967	9.03E-08	5.26E-06
11739370_PPM1D	0.895748	9.04E-08	5.26E-06
11754596_PAICS	-0.70539	9.08E-08	5.28E-06
11754235_PTPRF	-0.74064	9.12E-08	5.30E-06
11749023_SLC16A4	1.277855	9.14E-08	5.30E-06
11729717_SMAD5	-0.9437	9.14E-08	5.30E-06
11738426_ADGRF1	0.749447	9.15E-08	5.30E-06
11721448_ETF1	-0.80003	9.16E-08	5.30E-06
11756342_GDA	0.727036	9.19E-08	5.31E-06
11734821_APBB2	0.729561	9.24E-08	5.33E-06
11746463_IL6	1.705973	9.26E-08	5.33E-06
11751260_SFRP1	-1.18912	9.30E-08	5.35E-06
11722098_RICTOR	0.768259	9.31E-08	5.35E-06
11719928_KDM5B	0.772771	9.33E-08	5.36E-06
11753992_FKBP10	-0.85687	9.34E-08	5.36E-06
11721493_SULF2	0.987291	9.35E-08	5.36E-06
11756046_MDM2	0.751943	9.37E-08	5.36E-06
11728864_GINS1	-0.82512	9.37E-08	5.36E-06
11718293_CDKN2A	0.993984	9.40E-08	5.36E-06
11737149_ETNK1	0.939038	9.41E-08	5.37E-06
11730875_FANCI	-1.32094	9.44E-08	5.37E-06
11736426_CLIP4	0.811561	9.46E-08	5.37E-06
11752739_SLC2A14 /	1.006373	9.47E-08	5.37E-06
11736478_ELL2	0.965348	9.47E-08	5.37E-06
11757874_PFDN1	-0.76632	9.48E-08	5.37E-06
11757415_SLC5A3	-0.74229	9.50E-08	5.37E-06
11745571_CENPA	-0.79247	9.51E-08	5.37E-06
11754296_AEN	0.811704	9.52E-08	5.37E-06
11748014_VDAC3	-0.80206	9.52E-08	5.37E-06

11758151_RNASEH2A	-0.89559	9.57E-08	5.39E-06
11730296_TLR3	1.234569	9.57E-08	5.39E-06
11744468_SYNCRIP	-0.8623	9.58E-08	5.39E-06
11741479_PSAT1	-0.90706	9.61E-08	5.40E-06
11752683_CCDC181	1.017655	9.63E-08	5.40E-06
11721799_NAA50	-0.94764	9.64E-08	5.40E-06
11751258_DEK	-0.73481	9.65E-08	5.40E-06
11741315_PLSCR4	1.35747	9.67E-08	5.41E-06
11752361_NEXN	0.912151	9.72E-08	5.43E-06
11724515_GPD1L	-1.02847	9.73E-08	5.43E-06
11722371_TRIM22	1.436686	9.95E-08	5.54E-06
11742832_ASPM	-0.74208	9.96E-08	5.55E-06
11745050_ALCAM	0.70243	9.98E-08	5.55E-06
11720382_STX6	0.879829	1.00E-07	5.58E-06
11745872_PAPOLA	-0.97081	1.01E-07	5.61E-06
11758228_AMMECR1	0.847732	1.02E-07	5.61E-06
11736278_ZNF136	0.818134	1.02E-07	5.61E-06
11723666_SEMA3C	0.927203	1.02E-07	5.61E-06
11721874_IFIT2	0.767553	1.02E-07	5.61E-06
11717472_PRKCA	1.021586	1.02E-07	5.61E-06
11758336_AKT3	0.728427	1.02E-07	5.61E-06
11750839_ZNF561	0.826134	1.02E-07	5.62E-06
11744184_SIPA1L2	1.009861	1.03E-07	5.64E-06
11717352_RPRD1A	-0.88771	1.03E-07	5.64E-06
11717450_NEK7	0.816275	1.03E-07	5.64E-06
11715683_KRT7	1.21819	1.03E-07	5.67E-06
11726433_OSBPL3	0.806395	1.04E-07	5.67E-06
11749721_ZFP30	0.988552	1.04E-07	5.69E-06
11739919_ZNF195	0.751633	1.05E-07	5.71E-06
11752905_MCM7	-1.05215	1.06E-07	5.76E-06
11730961_TPM1	1.491381	1.06E-07	5.76E-06
11715799_PRRC2B	-0.92006	1.06E-07	5.77E-06
11746882_ZNF155	0.855813	1.06E-07	5.77E-06
11758094_NFATC2IP	-0.82959	1.06E-07	5.78E-06
11749806_SAV1	0.860331	1.07E-07	5.78E-06
11719863_TTLL12	-0.87634	1.07E-07	5.79E-06
11734718_FAM45A //	-1.46829	1.07E-07	5.79E-06
11728730_DSCC1	-0.79438	1.07E-07	5.79E-06
11750257_EEA1	1.142218	1.07E-07	5.79E-06
11743796_APITD1 //	-0.80753	1.07E-07	5.79E-06
11746094_MPP4	0.733428	1.08E-07	5.82E-06
11752869_CEBPD	1.120469	1.08E-07	5.82E-06
11756569_NUSAP1	-0.71957	1.08E-07	5.82E-06
11746283_KRTAP2-3	1.33942	1.09E-07	5.87E-06
11746002_CASP4	0.733935	1.10E-07	5.90E-06
11759556_BLZF1	0.805679	1.10E-07	5.90E-06
11756128_NAA50	-0.8423	1.11E-07	5.92E-06
11715487_MCL1	0.922419	1.11E-07	5.92E-06
11726668_MFSD8	0.841169	1.12E-07	5.96E-06
11752656_CBS /// C	-0.87981	1.12E-07	5.96E-06
11730049_CA13	0.937805	1.12E-07	5.97E-06

11759342_FMN1	-0.79803	1.12E-07	5.97E-06
11747279_DHFR	-0.7356	1.13E-07	6.00E-06
11734587_BAGE2	0.781783	1.13E-07	6.00E-06
11715512_NDFIP1	-0.82606	1.14E-07	6.02E-06
11736193_RRM2B	0.900631	1.14E-07	6.02E-06
11748435_CAP2	0.74505	1.14E-07	6.02E-06
11745843_CDK1	-0.81374	1.15E-07	6.06E-06
11756949_PPP2R1A	-0.71935	1.15E-07	6.06E-06
11746732_GRAMD3	0.881153	1.15E-07	6.06E-06
11745129_NOCT	1.144651	1.15E-07	6.06E-06
11725364_GAS7	-0.72101	1.15E-07	6.07E-06
11723972_APTX	-0.97586	1.15E-07	6.07E-06
11724901_IQCJ-SCHI	0.69184	1.16E-07	6.07E-06
11753788_CDKN3	-0.7798	1.16E-07	6.07E-06
11721256_CCND1	0.823197	1.17E-07	6.11E-06
11730112_DEPDC1	-0.66832	1.17E-07	6.14E-06
11715741_RCC2	-0.66561	1.17E-07	6.14E-06
11724095_PDE4B	0.740756	1.18E-07	6.15E-06
11731368_ZNF442	0.785254	1.18E-07	6.17E-06
11724610_E2F5	0.708556	1.19E-07	6.20E-06
11718374_WDR5	-0.66259	1.19E-07	6.20E-06
11731750_DUSP9	-1.03309	1.19E-07	6.21E-06
11749867_DHFR	-0.89607	1.19E-07	6.21E-06
11723599_PHLPP2	0.929372	1.19E-07	6.21E-06
11725188_ADGRG6	0.951559	1.20E-07	6.23E-06
11726882_ATAD2B	1.276676	1.20E-07	6.23E-06
11745443_DCAF16	-0.88566	1.20E-07	6.23E-06
11716765_IQGAP1	-0.80735	1.20E-07	6.23E-06
11724836_NCEH1	1.154974	1.21E-07	6.23E-06
11735323_ZNF441	1.079458	1.21E-07	6.23E-06
11725442_FOXM1	-0.6761	1.21E-07	6.25E-06
11717744_ 6-Mar	-1.01995	1.22E-07	6.27E-06
11749176_RIOK3	1.07912	1.22E-07	6.27E-06
11721736_TNFRSF19	-1.24193	1.22E-07	6.27E-06
11720433_FLRT2	0.875329	1.22E-07	6.27E-06
11739561_GDAP1	0.738514	1.22E-07	6.27E-06
11726610_MTF2	0.754721	1.23E-07	6.31E-06
11748563_REEP5	0.745782	1.24E-07	6.33E-06
11718399_TGM2	-0.8627	1.24E-07	6.34E-06
11728729_DSCC1	-0.93263	1.24E-07	6.34E-06
11746009_ACAT2	-0.77685	1.24E-07	6.34E-06
11731566_MDM2	0.776491	1.25E-07	6.35E-06
11733933_TMEM68	-1.15759	1.25E-07	6.36E-06
11729201_C6orf120	0.891614	1.25E-07	6.36E-06
11720218_MAP3K3	-1.02686	1.25E-07	6.36E-06
11751701_TMX3	0.699467	1.26E-07	6.40E-06
11741706_GJA5	0.770143	1.27E-07	6.42E-06
11725334_MZT1	-1.02341	1.27E-07	6.42E-06
11739261_ACSL3	-0.92933	1.28E-07	6.47E-06
11746223_JMJD1C	0.788264	1.28E-07	6.48E-06
11718857_NUDCD3	-1.10788	1.28E-07	6.48E-06

11753836_BANF1	-0.7339	1.28E-07	6.48E-06
11740934_NUSAP1	-0.8351	1.29E-07	6.48E-06
11736131_DCBLD2	0.647341	1.29E-07	6.51E-06
11764156_TMEM41B	-0.72302	1.30E-07	6.52E-06
11754972_BAZ2A	-0.89472	1.30E-07	6.52E-06
11725950_RIOK3	0.711968	1.30E-07	6.53E-06
11758137_SPOPL	-2.06986	1.30E-07	6.53E-06
11718723_ARRDC3	1.293597	1.31E-07	6.55E-06
11750120_FAM19A2	1.330388	1.31E-07	6.55E-06
11728587_PARD6B	1.05063	1.32E-07	6.57E-06
11718999_DHFR	-1.09819	1.32E-07	6.57E-06
11716183_ALCAM	0.710613	1.32E-07	6.58E-06
11752965_GRINA	-1.05439	1.32E-07	6.59E-06
11737620_PRR16	1.372558	1.33E-07	6.59E-06
11739336_SCYL2	0.740613	1.33E-07	6.59E-06
11754937_GMNN	-0.96387	1.33E-07	6.60E-06
11733779_DUSP3	-0.67547	1.33E-07	6.60E-06
11722913_USP13	-0.86583	1.33E-07	6.60E-06
11733029_AIDA	-0.88057	1.33E-07	6.60E-06
11721255_CCND1	0.653089	1.34E-07	6.61E-06
11755402_ZNF195	0.710635	1.34E-07	6.61E-06
11747348_PSAT1	-0.82497	1.34E-07	6.62E-06
11746454_USP15	0.694666	1.34E-07	6.63E-06
11727102_IGF2BP1	0.772539	1.35E-07	6.64E-06
11755681_HMGB1	-0.77345	1.35E-07	6.65E-06
11748774_CTSV	-1.03491	1.35E-07	6.65E-06
11747349_PSAT1	-0.97272	1.36E-07	6.67E-06
11726701_AFTPH	0.7258	1.36E-07	6.67E-06
11757696_PSMB6	-0.81709	1.37E-07	6.69E-06
11756714_PPP2R2A	-0.86381	1.37E-07	6.70E-06
11757785_HMGB1	-0.72457	1.37E-07	6.70E-06
11727314_ABCC5	0.827312	1.37E-07	6.70E-06
11725441_FOXM1	-0.77206	1.37E-07	6.70E-06
11740303_APITD1	-0.71933	1.37E-07	6.70E-06
11757017_RNASEH2A	-0.85048	1.38E-07	6.71E-06
11751153_ALDOC	-0.99086	1.38E-07	6.72E-06
11732534_RGS7	0.76512	1.39E-07	6.73E-06
11758619_LIF	0.778338	1.39E-07	6.73E-06
11752939_PGK1	-0.70456	1.39E-07	6.73E-06
11748385_KIAA0430	-0.73082	1.40E-07	6.77E-06
11732311_ANKRD13C	0.886533	1.40E-07	6.77E-06
11746473_CDIPT	-0.89881	1.40E-07	6.78E-06
11743007_NFKBIE	0.807027	1.40E-07	6.78E-06
11746019_THUMPD2	0.928389	1.40E-07	6.78E-06
11746207_OSBPL10	0.711274	1.41E-07	6.78E-06
11743698_EDEM3	0.696254	1.41E-07	6.80E-06
11716515_ADD1	-0.67386	1.41E-07	6.80E-06
11737041_PHLDB2	0.70355	1.42E-07	6.80E-06
11740540_REV3L	0.71883	1.42E-07	6.80E-06
11758046_RAD1	-0.80268	1.42E-07	6.80E-06
11715329_SLC6A15	-1.03451	1.42E-07	6.80E-06

11732493_TRMT10B	0.6994	1.42E-07	6.80E-06
11718594_CCNL1	0.739465	1.42E-07	6.80E-06
11757039_XIAP	0.999035	1.43E-07	6.84E-06
11744185_SIPA1L2	0.703953	1.43E-07	6.84E-06
11715579_VKORC1	-0.77365	1.45E-07	6.89E-06
11745892_MTHFD2	-0.8769	1.45E-07	6.89E-06
11717480_FKBP10	-0.99755	1.45E-07	6.89E-06
11739435_FN1	0.981283	1.45E-07	6.92E-06
11754206_HIVEP2	1.170123	1.46E-07	6.95E-06
11743733_RCAN1	-0.67044	1.46E-07	6.95E-06
11747725_STXBP6	1.114723	1.46E-07	6.95E-06
11748071_SRPR	-0.68968	1.47E-07	6.98E-06
11754515_SNX13	0.738027	1.47E-07	6.98E-06
11743136_CEBPD	1.031282	1.48E-07	7.00E-06
11723928_PNMA2	-1.01063	1.48E-07	7.01E-06
11735129_SPC24	-0.76624	1.48E-07	7.01E-06
11731567_MDM2	0.736942	1.49E-07	7.01E-06
11721645_ARL1	-0.82631	1.49E-07	7.02E-06
11724351_SMC6	-0.66644	1.50E-07	7.04E-06
11749490_MSH2	-0.67691	1.50E-07	7.06E-06
11758122_RLF	0.656676	1.50E-07	7.06E-06
11751335_MKRN1	-0.71644	1.50E-07	7.06E-06
11730821_CDKN3	-0.77775	1.51E-07	7.07E-06
11732712_FST	-0.81652	1.51E-07	7.07E-06
11735832_WHSC1	-0.84739	1.51E-07	7.07E-06
11746647_NSD1	0.83956	1.51E-07	7.07E-06
11727256_ZNF20 ///	0.824177	1.51E-07	7.07E-06
11732756_ELAVL2	1.193512	1.51E-07	7.07E-06
11719970_PACSIN3	-1.17956	1.52E-07	7.09E-06
11716551_HES1	1.237302	1.52E-07	7.09E-06
11722692_LOC105369	-0.77578	1.53E-07	7.12E-06
11758476_BNC2	-0.81088	1.54E-07	7.14E-06
11731841_B4GALT6	-0.73699	1.54E-07	7.14E-06
11720867_PLAC8	1.090994	1.55E-07	7.21E-06
11719416_MSH2	-0.88482	1.56E-07	7.22E-06
11763694_GLS	0.8629	1.56E-07	7.22E-06
11720380_IMPDH1	-0.79001	1.56E-07	7.22E-06
11744465_PPP1R15A	0.763096	1.56E-07	7.22E-06
11756899_NUDT21	-0.67004	1.56E-07	7.22E-06
11722434_MAD2L2	-0.6947	1.57E-07	7.22E-06
11725863_FAM13B	0.792496	1.57E-07	7.22E-06
11751370_RAB8B	1.139826	1.57E-07	7.22E-06
11751446_RMND5A	-0.90834	1.58E-07	7.26E-06
11759029_BRI3BP //	-1.19118	1.58E-07	7.28E-06
11722880_PHF19	-0.7689	1.58E-07	7.28E-06
11718874_ATP2A2	-0.80484	1.59E-07	7.30E-06
11736447_TMPO	-0.79624	1.59E-07	7.31E-06
11755207_NT5E	0.876284	1.60E-07	7.32E-06
11731569_PAWR	0.748732	1.60E-07	7.32E-06
11739853_SNX16	0.828233	1.60E-07	7.32E-06
11741011_SACM1L	-0.74497	1.62E-07	7.42E-06

11722922_FAM122B	-1.11028	1.63E-07	7.43E-06
11750302_PLK1	-0.73763	1.63E-07	7.43E-06
11741628_ST7L	0.926375	1.63E-07	7.43E-06
11755401_ZNF195	0.717298	1.63E-07	7.43E-06
11747730_DOCK4	0.757189	1.63E-07	7.43E-06
11736556_SLC2A14 /	0.929655	1.64E-07	7.43E-06
11720053_EMG1	-0.63585	1.64E-07	7.44E-06
11723868_SYNJ1	0.714866	1.65E-07	7.46E-06
11723641_FUCA1	0.936294	1.65E-07	7.46E-06
11759730_OVOS ///	-1.05931	1.65E-07	7.46E-06
11733562_KLHDC10	0.889112	1.65E-07	7.46E-06
11721337_WDR26	0.658173	1.66E-07	7.49E-06
11716217_PRKAB1	0.725451	1.66E-07	7.49E-06
11717689_RNF19A	0.938883	1.66E-07	7.49E-06
11758408_ZNF540	0.975093	1.66E-07	7.50E-06
11738935_KLRC3	1.833703	1.67E-07	7.50E-06
11757828_ATG5	-1.06369	1.67E-07	7.50E-06
11759982_BAGE2	0.760166	1.67E-07	7.51E-06
11752798_IREB2	0.94164	1.68E-07	7.53E-06
11758106_NRM	-0.80098	1.68E-07	7.54E-06
11723338_PDGFC	0.823141	1.68E-07	7.54E-06
11750967_ZNF586	0.813832	1.68E-07	7.54E-06
11733816_RPL22L1	-0.6603	1.70E-07	7.59E-06
11745183_NAA15	-0.6955	1.70E-07	7.61E-06
11742735_NUSAP1	-0.80679	1.71E-07	7.61E-06
11725984_FBXO28	0.707972	1.71E-07	7.62E-06
11740614_TMEM30A	0.729588	1.71E-07	7.62E-06
11732320_ZNF92	0.748376	1.71E-07	7.62E-06
11732131_IDS	-0.81884	1.71E-07	7.62E-06
11758970_PVRL3	0.727919	1.72E-07	7.63E-06
11729210_ORC2	-0.74899	1.72E-07	7.63E-06
11738516_ZCCHC6	0.747635	1.72E-07	7.63E-06
11752120_SUCO	-0.6946	1.72E-07	7.64E-06
11731751_RNF213	0.693593	1.73E-07	7.64E-06
11745209_SAMD4A	0.816942	1.73E-07	7.64E-06
11745826_CCNL1	0.794526	1.73E-07	7.64E-06
11722231_PDP1	0.743326	1.73E-07	7.65E-06
11748808_ARID3B	1.173994	1.74E-07	7.66E-06
11724032_FST	-1.04777	1.76E-07	7.75E-06
11759151_SPC24	-0.9782	1.76E-07	7.75E-06
11756884_C15orf52	0.649875	1.76E-07	7.75E-06
11719985_BLOC1S2	0.618353	1.78E-07	7.81E-06
11755241_AASS	0.714557	1.78E-07	7.81E-06
11739545_NEDD1	-0.79667	1.79E-07	7.83E-06
11728744_C4orf46	-1.02542	1.79E-07	7.83E-06
11717639_SDC4	-0.78237	1.79E-07	7.83E-06
11746666_FAM45A //	-1.45845	1.79E-07	7.83E-06
11718794_ZBED1	-0.70574	1.79E-07	7.83E-06
11731494_ITCH	0.720744	1.80E-07	7.85E-06
11715465_IGFBP4	-1.07707	1.80E-07	7.86E-06
11721384_ATG2B	0.800028	1.80E-07	7.86E-06

11721099_C3AR1	1.304435	1.80E-07	7.86E-06
11721593_CHST1	-1.28854	1.82E-07	7.93E-06
11741727_NEDD1	-0.66054	1.83E-07	7.93E-06
11753880_CDKN3	-1.02445	1.83E-07	7.93E-06
11715826_HMG20B	-0.82717	1.83E-07	7.93E-06
11717392_FN3KRP	-0.79513	1.83E-07	7.93E-06
11740390_RBM47	-0.68431	1.83E-07	7.93E-06
11758347_DDIT3	0.854093	1.83E-07	7.94E-06
11716395_ADGRG1	0.822749	1.84E-07	7.94E-06
11744285_CBS /// C	-0.7637	1.84E-07	7.94E-06
11726012_AJUBA	1.0388	1.84E-07	7.94E-06
11724229_RBMS1	0.780481	1.85E-07	8.00E-06
11763683_ARL6IP4	-0.65405	1.86E-07	8.01E-06
11752840_ELL2	0.939283	1.86E-07	8.01E-06
11743692_MCM4	-0.71051	1.88E-07	8.07E-06
11721842_GLIPR1	0.915468	1.88E-07	8.07E-06
11752535_CCNL1	0.666897	1.88E-07	8.08E-06
11720798_RAB8B	1.02698	1.89E-07	8.09E-06
11725698_XIAP	0.980042	1.89E-07	8.10E-06
11716848_PREX1	-1.13983	1.89E-07	8.10E-06
11732393_WWC2	0.63948	1.89E-07	8.10E-06
11744617_RPRD1A	-0.8984	1.90E-07	8.11E-06
11717862_EGR1	0.992986	1.90E-07	8.11E-06
11744662_HMG20B	-0.68487	1.91E-07	8.14E-06
11719155_DRAM1	0.769722	1.91E-07	8.15E-06
11749389_LANCL1	-1.2036	1.91E-07	8.15E-06
11749024_SLC16A4	0.961191	1.92E-07	8.18E-06
11754248_NAPEPLD	0.90242	1.93E-07	8.23E-06
11752090_PPM1D	0.89941	1.94E-07	8.24E-06
11743352_COBLL1	0.690404	1.95E-07	8.26E-06
11749986_CAMK2G	-0.92174	1.95E-07	8.26E-06
11758428_DCP2	1.290954	1.95E-07	8.26E-06
11747402_COPS7A	-0.98111	1.96E-07	8.28E-06
11732348_TCF20	-0.63886	1.98E-07	8.36E-06
11718947_MPP5	0.675131	1.98E-07	8.36E-06
11723706_ABHD5	0.686699	1.98E-07	8.36E-06
11737967_C15orf54	1.17424	1.98E-07	8.36E-06
11759205_DOCK5	-0.71363	1.98E-07	8.36E-06
11729194_MYNN	0.685489	1.98E-07	8.36E-06
11749454_ADGRF1	0.806004	1.99E-07	8.36E-06
11722883_CREBRF	0.82411	1.99E-07	8.36E-06
11720331_SELM	0.753544	1.99E-07	8.37E-06
11739137_RNF26	-1.04692	1.99E-07	8.37E-06
11757631_VGLL3	0.953312	2.00E-07	8.37E-06
11751878_JMJD1C	0.82864	2.00E-07	8.37E-06
11757626_SLC16A4	0.728697	2.00E-07	8.37E-06
11742099_CD164	0.727314	2.00E-07	8.39E-06
11735902_BDNF	1.172624	2.02E-07	8.43E-06
11722455_ZNF655	0.745975	2.02E-07	8.43E-06
11742948_MARCKS	-0.95854	2.04E-07	8.50E-06
11739526_LPP	0.628753	2.04E-07	8.51E-06

11716185_ALCAM	0.70764	2.05E-07	8.52E-06
11743528_RALBP1	-0.62396	2.05E-07	8.54E-06
11746608_RALBP1	-0.62396	2.05E-07	8.54E-06
11735042_C1orf52	0.635305	2.05E-07	8.54E-06
11745069_TRAPPC13	-0.62468	2.06E-07	8.54E-06
11726039_C3orf38	0.650547	2.06E-07	8.54E-06
11718105_IPO5	-0.704	2.07E-07	8.56E-06
11738327_HNMT	1.243063	2.07E-07	8.56E-06
11718957_PRKAB2	0.985242	2.07E-07	8.56E-06
11726105_PPP2R3A	-0.63423	2.08E-07	8.56E-06
11717717_FNDC3B	0.633574	2.08E-07	8.56E-06
11717777_USP15	0.760822	2.08E-07	8.56E-06
11734586_BAGE2 ///	0.760515	2.08E-07	8.56E-06
11724851_STXBP6	1.155217	2.08E-07	8.56E-06
11740140_RASGRP3	1.369061	2.09E-07	8.60E-06
11759780_ANKRD13C	1.119938	2.09E-07	8.60E-06
11753764_TNFRSF21	-0.61339	2.09E-07	8.60E-06
11753859_SNHG12	0.725296	2.10E-07	8.60E-06
11721209_EXO1	-0.67433	2.10E-07	8.60E-06
11748713_ASPM	-0.67043	2.10E-07	8.61E-06
11744681_NT5E	0.707423	2.10E-07	8.61E-06
11755695_FUCA1	0.882879	2.10E-07	8.61E-06
11724230_RBMS1	0.961484	2.11E-07	8.62E-06
11754177_RRM2B	1.085797	2.11E-07	8.62E-06
11733695_UBE2C	-0.64249	2.11E-07	8.63E-06
11716766_IQGAP1	-0.69776	2.12E-07	8.63E-06
11745452_TRMT13	0.694065	2.12E-07	8.63E-06
11724204_SNRPD1	-1.04042	2.12E-07	8.63E-06
11741642_ZFX /// Z	0.789139	2.12E-07	8.63E-06
11745561_ZFX /// Z	0.789139	2.12E-07	8.63E-06
11743513_GINS2	-0.83002	2.14E-07	8.68E-06
11743732_RCAN1	-0.68264	2.14E-07	8.68E-06
11747740_PPP2R1A	-0.73303	2.14E-07	8.68E-06
11725964_CAV2	0.681611	2.14E-07	8.69E-06
11752256_RBM15B	-0.72353	2.16E-07	8.74E-06
11753243_BAGE ///	0.686763	2.16E-07	8.75E-06
11758261_CEP55	-0.73022	2.17E-07	8.75E-06
11746010_ACAT2	-0.6588	2.17E-07	8.75E-06
11739560_GDAP1	0.816567	2.17E-07	8.76E-06
11745204_TPD52	-1.52863	2.18E-07	8.79E-06
11751329_FGFR1OP	0.632126	2.19E-07	8.82E-06
11716039_BABAM1	-0.69209	2.19E-07	8.83E-06
11720550_NCAPD3	-0.76262	2.19E-07	8.83E-06
11751187_ZNF485	0.83239	2.21E-07	8.86E-06
11748877_WRB	-0.69128	2.21E-07	8.86E-06
11749564_EFEMP1	1.209026	2.21E-07	8.86E-06
11715762_AP1M1	-1.03113	2.21E-07	8.86E-06
11718217_RAB22A	-0.87778	2.22E-07	8.86E-06
11757884_MSRB3	0.650365	2.22E-07	8.86E-06
11724217_GPATCH2	0.907213	2.22E-07	8.86E-06
11723829_ANO1	-1.08525	2.22E-07	8.86E-06

11716010_DYNC1LI2	-0.72098	2.22E-07	8.86E-06
11742432_NBEAL1	0.835695	2.22E-07	8.86E-06
11725189_ADGRG6	0.980665	2.23E-07	8.88E-06
11727153_LRRC1	0.81084	2.24E-07	8.92E-06
11753856_CKS2	-0.70239	2.24E-07	8.92E-06
11720163_VEGFC	0.680581	2.24E-07	8.92E-06
11749208_ELL2	0.76732	2.25E-07	8.95E-06
11729623_KIF2C	-0.84484	2.25E-07	8.95E-06
11731845_IGSF3	-0.90144	2.26E-07	8.95E-06
11758136_SLC35G2	0.654075	2.26E-07	8.96E-06
11716337_INSIG1	-1.03015	2.26E-07	8.97E-06
11747230_BUB1	-0.85758	2.27E-07	8.97E-06
11743457_ERGIC1	-0.83229	2.27E-07	8.97E-06
11716169_CCDC28A	-0.79031	2.27E-07	8.97E-06
11747047_MAP3K7CL	1.105299	2.27E-07	8.97E-06
11728745_C4orf46	-1.38944	2.29E-07	9.04E-06
11716151_PMEPA1	0.852692	2.29E-07	9.04E-06
11762865_CREB5	1.752915	2.29E-07	9.04E-06
11715973_MTFR1L	-0.79494	2.30E-07	9.04E-06
11727236_C18orf25	1.01599	2.30E-07	9.05E-06
11723556_DCAF16	-1.1263	2.30E-07	9.05E-06
11728694_LOC100996	0.711866	2.31E-07	9.05E-06
11741284_RFC5	-0.77985	2.31E-07	9.07E-06
11758044_KIAA0753	0.94531	2.32E-07	9.08E-06
11717924_YAP1	0.696431	2.32E-07	9.10E-06
11750674_STAT3	0.717009	2.33E-07	9.10E-06
11754259_FDFT1	-0.64672	2.33E-07	9.11E-06
11742640_RDM1	-0.97836	2.34E-07	9.16E-06
11726700_AFTPH	0.719343	2.35E-07	9.18E-06
11733857_EGLN1	0.758103	2.36E-07	9.22E-06
11743274_RSRC2	0.624325	2.37E-07	9.23E-06
11732241_SLITRK6	0.798175	2.37E-07	9.23E-06
11755561_PPP2R1A	-0.7325	2.37E-07	9.23E-06
11743196_TLR4	0.783541	2.38E-07	9.24E-06
11727536_CCNF	-0.79475	2.38E-07	9.25E-06
11717739_PCYOX1	-0.6915	2.38E-07	9.25E-06
11736257_PRKCE	1.021814	2.40E-07	9.29E-06
11758198_DDIT3	0.860144	2.40E-07	9.30E-06
11725410_NAV1	-0.84624	2.40E-07	9.31E-06
11749473_MEF2D	0.685368	2.40E-07	9.31E-06
11722241_SAV1	0.72508	2.41E-07	9.32E-06
11748529_CASP4	0.755932	2.41E-07	9.32E-06
11717638_SDC4	-0.76768	2.42E-07	9.34E-06
11724122_SLC35G2	0.719678	2.42E-07	9.34E-06
11721656_CISD2	-0.60168	2.42E-07	9.34E-06
11732370_CUX1	-0.70746	2.43E-07	9.35E-06
11727805_UBE2W	-0.63408	2.43E-07	9.36E-06
11717745_ 6-Mar	-0.91464	2.44E-07	9.37E-06
11755707_YAF2	0.683949	2.44E-07	9.37E-06
11758436_SEC62	-0.84465	2.45E-07	9.40E-06
11723939_CCNB1	-0.65108	2.45E-07	9.40E-06

11715437_TIMP3	-0.68959	2.45E-07	9.40E-06
11739196_PCGF3	-0.80581	2.45E-07	9.40E-06
11744369_EPB41L4A-	0.742441	2.46E-07	9.41E-06
11752058_DNA2	-0.78407	2.46E-07	9.41E-06
11721065_ADCY9	-1.10179	2.46E-07	9.42E-06
11724308_MED30	-0.61492	2.48E-07	9.45E-06
11757975_GFPT1	0.634913	2.48E-07	9.45E-06
11734015_SMAD2	-0.79631	2.48E-07	9.45E-06
11745021_MYC	0.64764	2.48E-07	9.45E-06
11743650_DEK	-0.69208	2.48E-07	9.45E-06
11747987_ZNF791	0.692279	2.50E-07	9.50E-06
11758375_RNF20	0.647316	2.52E-07	9.57E-06
11744188_ZNF420	0.744543	2.52E-07	9.59E-06
11718398_TGM2	-0.87143	2.53E-07	9.61E-06
11748785_FOXRED1	-0.82599	2.54E-07	9.61E-06
11718591_SDF2L1	-0.7078	2.54E-07	9.61E-06
11759715_LRCH1	0.623022	2.54E-07	9.61E-06
11749574_RPA2	-0.62869	2.54E-07	9.63E-06
11764030_CEBPD	0.730833	2.55E-07	9.64E-06
11715915_CD44	-0.6455	2.56E-07	9.64E-06
11718112_SF3A3	-0.68394	2.56E-07	9.64E-06
11742227_CLDN22	0.612671	2.56E-07	9.64E-06
11715363_A2M	1.578177	2.56E-07	9.64E-06
11716885_BANF1	-0.70099	2.56E-07	9.64E-06
11745950_PARP1	-0.75776	2.56E-07	9.65E-06
11718636_JMJD1C	0.623266	2.57E-07	9.65E-06
11758262_CCNJL	0.61727	2.57E-07	9.65E-06
11720052_EMG1	-0.62665	2.57E-07	9.65E-06
11753810_CCL5	0.703822	2.57E-07	9.65E-06
11758442_ARID4A	0.751634	2.57E-07	9.65E-06
11736054_CELF1	0.630136	2.59E-07	9.68E-06
11755420_KDM4B	0.79095	2.59E-07	9.69E-06
11734136_MTBP	-0.8113	2.60E-07	9.71E-06
11748766_FBXW7	0.944849	2.60E-07	9.73E-06
11731407_IFIT3	0.788126	2.62E-07	9.77E-06
11719063_SESN1	0.971861	2.62E-07	9.78E-06
11716849_PREX1	-1.21003	2.63E-07	9.79E-06
11719741_DCP2	0.979693	2.65E-07	9.85E-06
11750186_ALDH1A3	-0.65167	2.65E-07	9.86E-06
11720185_MAD2L1	-0.74208	2.65E-07	9.86E-06
11718464_NEURL1B	-1.36334	2.65E-07	9.86E-06
11748434_PARP2	-0.70321	2.65E-07	9.86E-06
11726141_FMN2	0.65168	2.66E-07	9.86E-06
11734824_SPRED1	-1.06536	2.67E-07	9.87E-06
11723435_TTK	-0.71686	2.67E-07	9.87E-06
11735694_PRKACB	0.918436	2.67E-07	9.87E-06
11754109_BIRC5	-0.84761	2.67E-07	9.87E-06
11742046_PCMTD2	-0.63825	2.67E-07	9.87E-06
11719959_SGPL1	-0.80524	2.67E-07	9.87E-06
11717268_CAMK2G	-0.96605	2.67E-07	9.87E-06
11719154_DRAM1	0.739748	2.68E-07	9.87E-06

11752557_CREBRF	0.874271	2.68E-07	9.87E-06
11731183_TFPI	0.661516	2.69E-07	9.90E-06
11759440_GLS	1.355355	2.69E-07	9.90E-06
11757492_RRAS2	0.69147	2.70E-07	9.91E-06
11753689_MDM2	0.665154	2.70E-07	9.91E-06
11763528_SLC12A4	0.724996	2.70E-07	9.91E-06
11756885_ATAD2	-0.63021	2.70E-07	9.92E-06
11752458_PKM	0.974324	2.71E-07	9.93E-06
11721608_RAPGEF2	0.686804	2.71E-07	9.93E-06
11744077_ARPC1B	-0.73023	2.71E-07	9.93E-06
11747588_HDAC9	0.778307	2.72E-07	9.95E-06
11756853_CLK4	1.127551	2.72E-07	9.95E-06
11753665_EGLN1	0.641777	2.73E-07	9.96E-06
11726608_MTF2	0.675804	2.73E-07	9.96E-06
11722786_TIMP2	-0.80532	2.73E-07	9.97E-06
11736527_SMC2	-0.64326	2.74E-07	9.98E-06
11730264_FAM83D	-0.72685	2.75E-07	1.00E-05
11744828_CLK1	0.916858	2.75E-07	1.00E-05
11753632_ATF3	0.739317	2.76E-07	1.00E-05
11741856_ZNF658 //	0.702844	2.76E-07	1.00E-05
11743604_RRM1	-0.65875	2.76E-07	1.00E-05
11744297_RMI2	-0.69793	2.77E-07	1.00E-05
11741297_SMARCA1	-0.62343	2.77E-07	1.00E-05
11745469_INPP4B	0.715503	2.77E-07	1.00E-05
11752734_ZNF138	0.933162	2.77E-07	1.00E-05
11732078_EVA1A	0.687025	2.77E-07	1.00E-05
11744896_GPSM3	-0.92671	2.78E-07	1.00E-05
11759031_ABCC2	0.631884	2.78E-07	1.00E-05
11731303_DUSP10	0.752702	2.78E-07	1.00E-05
11747401_COPS7A	-1.03316	2.78E-07	1.00E-05
11737043_PHLDB2	0.638079	2.79E-07	1.00E-05
11750484_MRE11A	-0.85532	2.79E-07	1.00E-05
11733077_PPP1R12A	0.619876	2.79E-07	1.00E-05
11718677_CTNNAL1 /	0.758705	2.79E-07	1.00E-05
11755522_LPXN	0.716444	2.80E-07	1.00E-05
11731465_CTSC	-0.79684	2.80E-07	1.00E-05
11736745_ZBTB20	0.841266	2.80E-07	1.00E-05
11734743_DCTN5	0.655156	2.81E-07	1.01E-05
11727916_CENPA	-0.84613	2.81E-07	1.01E-05
11731525_TRIP13	-0.63415	2.83E-07	1.01E-05
11755645_ZNF529	0.688528	2.83E-07	1.01E-05
11756174_SCD	-0.6337	2.83E-07	1.01E-05
11744572_KLF5	0.627839	2.83E-07	1.01E-05
11744721_MSH2	-0.85385	2.83E-07	1.01E-05
11742399_LOC105369	-0.72376	2.83E-07	1.01E-05
11720427_ILVBL	-0.58398	2.84E-07	1.01E-05
11717353_EAPP	-1.01959	2.85E-07	1.01E-05
11754108_RBM14 ///	0.676416	2.87E-07	1.02E-05
11750667_RRM1	-0.67009	2.87E-07	1.02E-05
11721682_RBMX2	-0.66394	2.88E-07	1.02E-05
11732246_HNRNPC	-0.76611	2.88E-07	1.02E-05

11718637_JMJD1C	0.673371	2.88E-07	1.02E-05
11749032_PTBP3	0.662306	2.89E-07	1.02E-05
11758948_SLC30A1	0.661388	2.89E-07	1.02E-05
11741296_SMARCA1	-0.67283	2.90E-07	1.03E-05
11758537_TRIM25	-0.75747	2.90E-07	1.03E-05
11721145_MKI67	-0.7376	2.91E-07	1.03E-05
11737880_GALNT2	0.604089	2.91E-07	1.03E-05
11728705_COPG2	-0.87813	2.91E-07	1.03E-05
11743298_CNOT6	0.75042	2.93E-07	1.03E-05
11728478_CCDC181	1.067259	2.93E-07	1.03E-05
11734799_RLIM	0.767242	2.94E-07	1.03E-05
11741198_ZNF138	0.726611	2.94E-07	1.04E-05
11754416_FBXO28	0.627798	2.95E-07	1.04E-05
11722305_ARHGAP23	0.664835	2.95E-07	1.04E-05
11743272_RSRC2	0.60139	2.96E-07	1.04E-05
11739139_RNF26	-0.82991	2.97E-07	1.04E-05
11718299_CCDC25	-0.69284	2.98E-07	1.05E-05
11744466_AK6 /// T	-0.69844	2.98E-07	1.05E-05
11722069_SKA1	-0.93433	2.99E-07	1.05E-05
11722872_RFC4	-0.95103	2.99E-07	1.05E-05
11743412_OTUD1	0.809036	3.00E-07	1.05E-05
11759510_MCM4	-0.62807	3.00E-07	1.05E-05
11722251_NEK2	-0.65526	3.01E-07	1.05E-05
11736328_ZNF322	0.797326	3.01E-07	1.05E-05
11756626_ZWINT	-0.61271	3.02E-07	1.05E-05
11724882_TUFT1	0.759006	3.02E-07	1.05E-05
11720836_RIN2	0.62382	3.03E-07	1.06E-05
11736229_CRIM1	0.710431	3.04E-07	1.06E-05
11718965_NUFIP2	-0.78831	3.04E-07	1.06E-05
11735003_ST7L	0.866402	3.06E-07	1.06E-05
11758101_EIF4EBP2	-0.81477	3.06E-07	1.06E-05
11731497_ZNF134	0.778126	3.06E-07	1.06E-05
11751165_RBMS2	0.788073	3.06E-07	1.06E-05
11725413_TRIM14	-0.63461	3.06E-07	1.06E-05
11722645_ZNHIT6	0.621287	3.07E-07	1.06E-05
11740097_TBX3	0.778739	3.08E-07	1.07E-05
11722802_GNAI1	-0.64827	3.09E-07	1.07E-05
11756577_PEA15	0.657963	3.09E-07	1.07E-05
11740048_ZNF461	0.7581	3.10E-07	1.07E-05
11754080_CDIPT	-1.31929	3.10E-07	1.07E-05
11719899_C11orf96	-0.74115	3.12E-07	1.08E-05
11749798_GNAI1	0.794558	3.12E-07	1.08E-05
11757065_BANF1	-0.62751	3.14E-07	1.08E-05
11716184_ALCAM	0.620679	3.14E-07	1.08E-05
11731775_ADGRE1	0.593883	3.15E-07	1.08E-05
11736025_MED28	0.797011	3.16E-07	1.08E-05
11743609_FAM3C	0.719361	3.16E-07	1.08E-05
11744752_GTF3C2	-0.65924	3.16E-07	1.08E-05
11753978_MTFR1L	-0.60381	3.16E-07	1.08E-05
11743064_CDC6	-0.609	3.16E-07	1.08E-05
11744664_ADAMTS12	0.877354	3.17E-07	1.08E-05

11726291_LRRC17	1.28047	3.17E-07	1.08E-05
11746985_TBX3	1.06522	3.17E-07	1.08E-05
11745819_H1F0	-0.63265	3.17E-07	1.08E-05
11753858_SNHG12	0.6351	3.18E-07	1.08E-05
11718509_UBE2L3	-0.5889	3.19E-07	1.09E-05
11751643_EGR1	0.664524	3.19E-07	1.09E-05
11732206_NAP1L1	-0.7722	3.19E-07	1.09E-05
11751178_ATP6V1A	0.66308	3.20E-07	1.09E-05
11720498_CDC42EP3	-0.77485	3.21E-07	1.09E-05
11728647_NUCKS1	-0.99361	3.22E-07	1.10E-05
11722279_PHC3	0.735165	3.23E-07	1.10E-05
11721601_ZBTB18	0.713232	3.23E-07	1.10E-05
11725616_PPP1CB	-0.91228	3.25E-07	1.10E-05
11753563_MDM2	0.679067	3.26E-07	1.11E-05
11754470_CHTF18	-0.65603	3.26E-07	1.11E-05
11745424_SMC2	-0.90466	3.27E-07	1.11E-05
11748858_BABAM1	-0.74798	3.27E-07	1.11E-05
11739403_KMT5B	0.64612	3.28E-07	1.11E-05
11723337_PDGFC	0.781629	3.28E-07	1.11E-05
11743295_CENPF	-0.87817	3.29E-07	1.11E-05
11751813_TMEM107	-0.73527	3.29E-07	1.11E-05
11725082_CALB2	0.858912	3.29E-07	1.11E-05
11749457_FCHO2	0.805243	3.30E-07	1.11E-05
11722452_QKI	-0.61865	3.30E-07	1.11E-05
11719849_UBE2S	-0.63403	3.30E-07	1.11E-05
11726498_LIF	0.671426	3.31E-07	1.11E-05
11743651_DEK	-0.61459	3.31E-07	1.11E-05
11729517_ZNF644	0.635994	3.32E-07	1.11E-05
11761742_IMMP2L	1.289019	3.33E-07	1.12E-05
11720155_ZC3H7A	0.677325	3.33E-07	1.12E-05
11725937_LGALS3	0.67019	3.33E-07	1.12E-05
11750719_ZNF45	0.858114	3.33E-07	1.12E-05
11718797_PAQR4	-0.67354	3.34E-07	1.12E-05
11719880_OSTM1	1.102461	3.34E-07	1.12E-05
11743189_RGL1	0.847207	3.35E-07	1.12E-05
11759296_MAP3K2	0.999171	3.35E-07	1.12E-05
11748976_STRADA	-0.75041	3.35E-07	1.12E-05
11742816_FAM13A	0.681386	3.37E-07	1.12E-05
11724948_CITED4	0.683764	3.37E-07	1.12E-05
11716779_EIF1AX	-0.62666	3.37E-07	1.12E-05
11720932_DLST	-0.65868	3.39E-07	1.13E-05
11756530_ST7L	0.86748	3.39E-07	1.13E-05
11748762_KLF5	0.817815	3.39E-07	1.13E-05
11740190_DICER1	0.978771	3.39E-07	1.13E-05
11716984_PNKD	-0.64861	3.40E-07	1.13E-05
11716727_PLK1	-0.68901	3.41E-07	1.13E-05
11745621_APTX	-0.88217	3.41E-07	1.13E-05
11751180_ADAM10	0.712075	3.42E-07	1.13E-05
11720827_CENPW	-0.72987	3.44E-07	1.14E-05
11752187_NAPEPLD	1.100519	3.44E-07	1.14E-05
11718705_IGF1R	-0.91002	3.45E-07	1.14E-05

11739324_WDR4	0.963626	3.47E-07	1.14E-05
11722300_ETS1	0.630672	3.47E-07	1.14E-05
11723830_ANO1	-1.03609	3.47E-07	1.14E-05
11749908_SYT11	0.752997	3.47E-07	1.14E-05
11726504_ERCC6-PGE	0.961803	3.47E-07	1.14E-05
11721232_VCPKMT	0.742099	3.49E-07	1.15E-05
11739080_DNAJB4	0.880617	3.49E-07	1.15E-05
11746379_TAB3	0.615068	3.49E-07	1.15E-05
11744148_MRPL12	-0.69873	3.49E-07	1.15E-05
11746119_SYNCRIP	-0.6825	3.49E-07	1.15E-05
11758963_PAFAH1B2	-0.66163	3.50E-07	1.15E-05
11756903_HHAT	0.919216	3.50E-07	1.15E-05
11722240_SAV1	0.763823	3.50E-07	1.15E-05
11720714_NIPA1	-1.21203	3.51E-07	1.15E-05
11729837_ZCCHC2	0.734008	3.52E-07	1.15E-05
11716092_CKS1B	-0.67402	3.52E-07	1.15E-05
11750446_ATG4B	-0.76267	3.53E-07	1.15E-05
11756696_ZNF567	0.721636	3.53E-07	1.15E-05
11733076_PPP1R12A	0.598444	3.54E-07	1.15E-05
11732060_LEMD1	0.607954	3.54E-07	1.15E-05
11739285_ARL6IP6	-0.72944	3.54E-07	1.15E-05
11720859_ABHD3	-1.08607	3.54E-07	1.15E-05
11715885_TMEM248	-0.69834	3.55E-07	1.15E-05
11741732_ANP32E	-0.75479	3.56E-07	1.16E-05
11717909_IGFBP6	-0.7179	3.56E-07	1.16E-05
11722804_PTBP2	0.614489	3.58E-07	1.16E-05
11754360_RRM2	-0.57909	3.58E-07	1.16E-05
11751697_NAA50	-0.67039	3.58E-07	1.16E-05
11757434_C1QBP	-0.58692	3.59E-07	1.17E-05
11756665_CD164	0.618031	3.59E-07	1.17E-05
11755039_FBXL2	0.670252	3.60E-07	1.17E-05
11724593_NUP37	-0.60812	3.61E-07	1.17E-05
11724468_RPA3	-0.66725	3.63E-07	1.17E-05
11724203_SNRPD1	-1.04448	3.63E-07	1.17E-05
11735268_SLC41A2	0.982619	3.64E-07	1.17E-05
11747125_RIOK3	0.892923	3.65E-07	1.18E-05
11730896_MAPKAPK2	-1.21672	3.66E-07	1.18E-05
11730364_HRH1	1.0006	3.66E-07	1.18E-05
11726743_F2RL2	1.117586	3.67E-07	1.18E-05
11749193_UBE2L3	-0.64235	3.68E-07	1.18E-05
11724752_TMF1	0.683492	3.68E-07	1.18E-05
11755984_TRIML2	0.896803	3.68E-07	1.18E-05
11718215_DDA1	0.587372	3.68E-07	1.18E-05
11715940_KHDRBS1	-0.5643	3.69E-07	1.18E-05
11726038_C3orf38	0.631741	3.69E-07	1.18E-05
11758181_HMGB1	-0.65304	3.69E-07	1.18E-05
11742997_MCM3	-0.58631	3.70E-07	1.19E-05
11737442_ST7L	1.018815	3.72E-07	1.19E-05
11718735_PCMTD1	0.573893	3.73E-07	1.19E-05
11752877_TUBB	-0.59749	3.74E-07	1.19E-05
11723782_AGPS	-1.17977	3.74E-07	1.20E-05

11725640_NSL1	-0.57145	3.74E-07	1.20E-05
11729852_ZMYM5	0.705577	3.75E-07	1.20E-05
11728110_GRIP1	1.024709	3.75E-07	1.20E-05
11721231_VCPKMT	0.752943	3.77E-07	1.20E-05
11735146_PPP1R8	-0.86656	3.78E-07	1.20E-05
11753085_PGK1	-0.79738	3.79E-07	1.21E-05
11746856_SERPINE1	0.69963	3.79E-07	1.21E-05
11718517_AK6 /// T	-0.74453	3.79E-07	1.21E-05
11758208_KLF2	0.696949	3.80E-07	1.21E-05
11724643_SPOPL	-0.8378	3.81E-07	1.21E-05
11727453_COPRS	-0.62773	3.82E-07	1.21E-05
11751226_DLST	-0.64483	3.83E-07	1.21E-05
11718013_IRS2	-0.84833	3.83E-07	1.21E-05
11734010_TRAPPC13	-0.61373	3.83E-07	1.21E-05
11729223_GATA3	0.883973	3.83E-07	1.21E-05
11753735_TMSB4X	-0.61766	3.83E-07	1.21E-05
11718375_WDR5	-0.63493	3.84E-07	1.21E-05
11718873_ATP2A2	-0.78268	3.85E-07	1.22E-05
11721195_RRP1B	-0.83409	3.85E-07	1.22E-05
11753904_CDC42EP2	0.624625	3.86E-07	1.22E-05
11745800_CCP110	0.759831	3.87E-07	1.22E-05
11719511_PHC1	-0.78252	3.87E-07	1.22E-05
11721966_HMBOX1	0.867937	3.88E-07	1.22E-05
11726603_DARS2	-0.58145	3.88E-07	1.22E-05
11752700_ATP11B	0.698644	3.88E-07	1.22E-05
11746584_MTF2	0.617192	3.89E-07	1.22E-05
11750831_ZNF571	0.935011	3.89E-07	1.22E-05
11720203_AURKB	-0.65277	3.89E-07	1.22E-05
11715675_CCNG1	0.594753	3.89E-07	1.22E-05
11756155_NHS	0.812326	3.90E-07	1.22E-05
11744735_TMEM200A	1.323521	3.91E-07	1.22E-05
11747964_SLC4A7	0.735893	3.91E-07	1.22E-05
11736528_SMC2	-0.95517	3.92E-07	1.23E-05
11718673_TMEM245	0.636828	3.93E-07	1.23E-05
11740000_CPEB3	0.96889	3.93E-07	1.23E-05
11726671_DCLRE1B	-0.74363	3.93E-07	1.23E-05
11763238_CTCFL ///	-0.63859	3.94E-07	1.23E-05
11716339_INSIG1	-0.68683	3.94E-07	1.23E-05
11754302_ATP2A2	-0.77557	3.94E-07	1.23E-05
11722285_SLC30A7	0.711265	3.95E-07	1.23E-05
11740686_NAPEPLD	0.962927	3.95E-07	1.23E-05
11751923_ERBB2IP	0.639254	3.95E-07	1.23E-05
11718879_SMC1A	-0.629	3.96E-07	1.23E-05
11749291_FOS	1.033013	3.96E-07	1.23E-05
11728676_MED14	-0.67197	3.96E-07	1.23E-05
11748901_EDA2R	0.86282	3.96E-07	1.23E-05
11723177_FRAS1	0.920604	3.98E-07	1.23E-05
11759287_DNAJB4	0.810242	3.98E-07	1.23E-05
11728762_LOC100506	0.776191	3.98E-07	1.23E-05
11716703_PRKACB	0.636904	3.98E-07	1.23E-05
11717215_ATP9A	-0.88413	3.99E-07	1.23E-05

11725508_SLC7A6	0.973411	4.00E-07	1.24E-05
11755748_PLAC8	1.262131	4.00E-07	1.24E-05
11758569_HIST2H2BF	1.289553	4.01E-07	1.24E-05
11734200_ZNF844	0.898029	4.02E-07	1.24E-05
11727675_MOCOS	-0.56242	4.02E-07	1.24E-05
11748136_MAD2L1	-0.73158	4.02E-07	1.24E-05
11746373_RRN3 ///	0.613001	4.03E-07	1.24E-05
11731648_NTM	0.755681	4.03E-07	1.24E-05
11733302_ZNF790	0.824333	4.04E-07	1.24E-05
11758773_HACD2	-0.85922	4.04E-07	1.24E-05
11751123_CDC45	-0.77644	4.05E-07	1.24E-05
11755772_FOXO1	0.964529	4.05E-07	1.24E-05
11760660_HIST2H2BC	1.581778	4.05E-07	1.24E-05
11742100_ZNF195	0.706371	4.05E-07	1.24E-05
11758463_SCYL2	0.741205	4.06E-07	1.24E-05
11736737_RASA2	0.712064	4.07E-07	1.25E-05
11752768_MTFR1L	-0.76478	4.09E-07	1.25E-05
11719779_CD164	0.5788	4.10E-07	1.25E-05
11723840_RMI1	-0.83453	4.10E-07	1.25E-05
11728061_ZNF608	0.822161	4.11E-07	1.26E-05
11727666_TWIST1	-0.65149	4.12E-07	1.26E-05
11760343_ZNF726	-0.80648	4.13E-07	1.26E-05
11754966_AIF1L	-0.62449	4.13E-07	1.26E-05
11724510_PMAIP1	0.678898	4.13E-07	1.26E-05
11739426_GPD2	-0.57572	4.13E-07	1.26E-05
11730741_AKIRIN1	0.555461	4.13E-07	1.26E-05
11715252_RSRC1	-0.71866	4.14E-07	1.26E-05
11737336_ZNF283	1.095933	4.14E-07	1.26E-05
11753301_CBR1	-0.61847	4.14E-07	1.26E-05
11755923_ARHGEF28	0.677232	4.15E-07	1.26E-05
11732013_ZNF419	0.745836	4.16E-07	1.26E-05
11716332_RPL28	0.594121	4.17E-07	1.26E-05
11736027_ADAM10	0.71808	4.17E-07	1.26E-05
11726667_MFSD8	0.658048	4.18E-07	1.26E-05
11729603_MCM7	-0.60973	4.18E-07	1.27E-05
11740938_TGIF1	0.607695	4.19E-07	1.27E-05
11727815_UBASH3B	0.70713	4.20E-07	1.27E-05
11764091_YY1	-0.75237	4.20E-07	1.27E-05
11745180_ZNF24	0.784828	4.20E-07	1.27E-05
11744058_DDOST	-0.68862	4.21E-07	1.27E-05
11742714_TBRG1	-0.94413	4.22E-07	1.27E-05
11744103_TLCD1	-0.92433	4.22E-07	1.27E-05
11722900_RARA	0.800396	4.22E-07	1.27E-05
11716760_CASP4	0.742234	4.23E-07	1.27E-05
11716492_SIVA1	-0.59323	4.24E-07	1.27E-05
11744020_NUP210	-0.6225	4.24E-07	1.27E-05
11727487_HAUS6	-0.70857	4.24E-07	1.27E-05
11717233_MKLN1	0.805135	4.25E-07	1.27E-05
11751438_ST6GALNAC	0.798567	4.26E-07	1.28E-05
11749528_CDCA7	-0.95102	4.26E-07	1.28E-05
11746008_NEXN	0.782565	4.28E-07	1.28E-05

11737992_GPR37	1.170325	4.29E-07	1.28E-05
11726740_EARS2	-0.72786	4.29E-07	1.28E-05
11717833_TEX10	0.56733	4.29E-07	1.28E-05
11743502_GTF3C2	-0.57049	4.30E-07	1.28E-05
11733898_TROVE2	0.735122	4.30E-07	1.28E-05
11725559_ZNF112	0.946059	4.31E-07	1.29E-05
11747816_GDAP1	0.604618	4.31E-07	1.29E-05
11758444_DHTKD1	-0.83733	4.31E-07	1.29E-05
11759343_FMN1	-0.76823	4.32E-07	1.29E-05
11739226_NF1	0.652768	4.34E-07	1.29E-05
11758456_GFPT1	0.689855	4.34E-07	1.29E-05
11741306_SAMD8	0.851587	4.34E-07	1.29E-05
11731221_MICU3	1.314856	4.34E-07	1.29E-05
11722936_MB21D2	1.01291	4.35E-07	1.29E-05
11721882_TUBGCP3	-0.65018	4.35E-07	1.29E-05
11747231_SHC4	0.720335	4.36E-07	1.29E-05
11750929_FRS2	0.71498	4.36E-07	1.29E-05
11728281_RBMS2	-0.56115	4.38E-07	1.30E-05
11725128_GNG4	-0.74502	4.38E-07	1.30E-05
11725449_C2orf49	0.634461	4.38E-07	1.30E-05
11723724_USP32	0.683568	4.38E-07	1.30E-05
11739427_GPD2	-0.61531	4.39E-07	1.30E-05
11745790_TMEM135	0.730445	4.41E-07	1.30E-05
11733702_UBE2C	-0.54715	4.42E-07	1.31E-05
11721446_SNX4	0.702961	4.43E-07	1.31E-05
11727489_KIF11	-0.61136	4.44E-07	1.31E-05
11725074_FAM126A	0.581458	4.44E-07	1.31E-05
11758864_SRP19	-0.71667	4.45E-07	1.31E-05
11720782_CDK2	-0.65969	4.45E-07	1.31E-05
11732936_TBC1D2B	-0.55822	4.45E-07	1.31E-05
11722572_CCNA2	-0.63034	4.46E-07	1.31E-05
11744796_DAB2	0.556392	4.46E-07	1.31E-05
11758279_CORO1C	-0.6145	4.46E-07	1.31E-05
11718180_FERMT2	0.699472	4.47E-07	1.31E-05
11740111_COASY	-0.66595	4.49E-07	1.32E-05
11729915_ARL5B	0.90773	4.50E-07	1.32E-05
11744932_CREBRF	0.736301	4.51E-07	1.32E-05
11756551_KLHL28	1.286286	4.51E-07	1.32E-05
11743172_SLCO4A1	-0.71957	4.53E-07	1.32E-05
11743273_RSRC2	0.565119	4.53E-07	1.32E-05
11759422_ZNF195	0.727592	4.53E-07	1.32E-05
11734359_RNASEL	0.633995	4.53E-07	1.32E-05
11730023_PILRB ///	0.739999	4.55E-07	1.33E-05
11723950_PBK	-0.66519	4.55E-07	1.33E-05
11723680_CNOT4	0.678019	4.55E-07	1.33E-05
11746351_REV3L	0.593551	4.56E-07	1.33E-05
11747713_PRC1	-0.75765	4.56E-07	1.33E-05
11743597_ZNF140	0.792303	4.56E-07	1.33E-05
11745906_UMAD1	-0.95287	4.58E-07	1.33E-05
11757851_ABL1	-0.62839	4.58E-07	1.33E-05
11759175_MED28	0.658878	4.60E-07	1.34E-05

11741122_LYPD1	0.89587	4.60E-07	1.34E-05
11745464_CDKN3	-0.78576	4.60E-07	1.34E-05
11755324_NRXN3	0.950704	4.61E-07	1.34E-05
11751203_ZNHIT6	0.728593	4.62E-07	1.34E-05
11722369_TRIM22	0.933401	4.62E-07	1.34E-05
11744529_PELI1	1.096295	4.63E-07	1.34E-05
11759028_BRI3BP //	-1.12972	4.63E-07	1.34E-05
11736998_NIPAL1	0.987166	4.64E-07	1.34E-05
11743626_LGR4	0.703829	4.64E-07	1.34E-05
11718095_RNF38	0.668703	4.65E-07	1.34E-05
11715975_MTFR1L	-0.6277	4.65E-07	1.34E-05
11751206_AURKA ///	-0.66085	4.66E-07	1.35E-05
11751896_COL12A1	1.134754	4.67E-07	1.35E-05
11726127_ABCB9	0.948254	4.68E-07	1.35E-05
11748631_REEP5	-0.58212	4.68E-07	1.35E-05
11732996_HSP90AA1	-0.89534	4.69E-07	1.35E-05
11719585_CNNM3	-0.87209	4.69E-07	1.35E-05
11725749_GAL	-0.65229	4.70E-07	1.35E-05
11755747_PLAC8	0.79273	4.71E-07	1.35E-05
11740253_ZWINT	-0.59843	4.72E-07	1.36E-05
11716249_TMED9	-0.65186	4.73E-07	1.36E-05
11725862_FAM13B	0.975436	4.76E-07	1.36E-05
11738845_NTM	0.70919	4.76E-07	1.36E-05
11758441_HOXA1	0.880837	4.76E-07	1.36E-05
11753688_MDM2	0.557404	4.77E-07	1.36E-05
11757796_NCS1	-0.71036	4.77E-07	1.36E-05
11717688_RNF19A	0.701982	4.77E-07	1.37E-05
11722182_ATP6V1C1	-0.87788	4.78E-07	1.37E-05
11718676_TMEM245	0.862698	4.78E-07	1.37E-05
11758306_CNOT6	0.732875	4.79E-07	1.37E-05
11739857_TENM2	0.882501	4.80E-07	1.37E-05
11737765_C3orf17	0.576676	4.81E-07	1.37E-05
11748174_MCM3	-0.54381	4.82E-07	1.37E-05
11751018_PPM1D	0.824094	4.82E-07	1.37E-05
11736028_ADAM10	0.650875	4.82E-07	1.37E-05
11756971_PKM	0.979853	4.82E-07	1.37E-05
11726880_XIRP2	1.252929	4.82E-07	1.37E-05
11754889_PRIMPOL	-0.60078	4.82E-07	1.37E-05
11758686_CD164	0.566837	4.82E-07	1.37E-05
11723244_CDK6	0.567491	4.84E-07	1.37E-05
11726870_SETBP1	0.82381	4.84E-07	1.37E-05
11732421_ZNF234	0.742315	4.84E-07	1.37E-05
11753949_ARL6IP4	-0.68645	4.85E-07	1.37E-05
11729458_FANCI	-1.03868	4.86E-07	1.37E-05
11727147_ZNF564	0.862954	4.86E-07	1.37E-05
11721208_IFFO2	-0.66801	4.86E-07	1.37E-05
11748783_UHRF1	-0.81023	4.86E-07	1.37E-05
11742812_RPS6KA3	0.600063	4.87E-07	1.37E-05
11750879_CREB5	1.362503	4.87E-07	1.37E-05
11724420_ZC3H6	-0.72005	4.87E-07	1.37E-05
11747739_PPP2R1A	-0.5882	4.87E-07	1.37E-05

11751804_SPC24	-0.73458	4.89E-07	1.38E-05
11743343_RMND5A	-1.25632	4.90E-07	1.38E-05
11719474_IMP4	-0.63728	4.90E-07	1.38E-05
11719382_PPIH	-0.63541	4.90E-07	1.38E-05
11738052_MRGPRX3	0.740744	4.91E-07	1.38E-05
11726335_CRLS1	-0.6777	4.93E-07	1.38E-05
11755908_IGSF3	-0.77867	4.94E-07	1.39E-05
11763714_SSBP2	0.563106	4.94E-07	1.39E-05
11758521_SPIRE1	-0.89429	4.95E-07	1.39E-05
11743864_ATP2B1	0.665056	4.95E-07	1.39E-05
11750542_NOC3L	0.583683	4.96E-07	1.39E-05
11753086_PGK1	-0.70872	4.97E-07	1.39E-05
11730011_KBTBD8	1.190231	4.98E-07	1.39E-05
11752681_TMEM135	0.550741	4.98E-07	1.39E-05
11751466_C3orf38	0.654676	4.99E-07	1.39E-05
11724346_IFIH1	0.899819	4.99E-07	1.39E-05
11744261_SLC25A11	-0.63167	4.99E-07	1.39E-05
11748408_SUCO	-0.77319	5.00E-07	1.40E-05
11720679_CKLF ///	-0.61132	5.00E-07	1.40E-05
11743652_DEK	-0.65487	5.01E-07	1.40E-05
11734890_IL32	1.082115	5.01E-07	1.40E-05
11748775_UPP1	0.665722	5.02E-07	1.40E-05
11733376_DLX1	-0.67637	5.03E-07	1.40E-05
11757902_DYNC1LI2	-0.67642	5.04E-07	1.40E-05
11751755_KIF22	-0.5571	5.05E-07	1.40E-05
11723555_DCAF16	-1.08184	5.06E-07	1.41E-05
11715974_MTFR1L	-0.74532	5.06E-07	1.41E-05
11721242_FDFT1	-0.63873	5.07E-07	1.41E-05
11748365_KIAA0753	0.81346	5.07E-07	1.41E-05
11730368_ZNF557	0.718785	5.07E-07	1.41E-05
11757852_AP1G1	0.600324	5.08E-07	1.41E-05
11758892_NDC1	-0.57533	5.08E-07	1.41E-05
11723667_SEMA3C	0.791944	5.09E-07	1.41E-05
11717622_SUCO	-0.59835	5.10E-07	1.41E-05
11748942_TNRC6A	0.800184	5.10E-07	1.41E-05
11717931_TBK1	0.610595	5.12E-07	1.41E-05
11717496_IMPA2	-0.65244	5.13E-07	1.42E-05
11741911_GBP3	1.354474	5.14E-07	1.42E-05
11728322_RAD54L	-0.7181	5.15E-07	1.42E-05
11750838_ZNF561	0.760999	5.15E-07	1.42E-05
11756069_CDKN3	-0.79624	5.15E-07	1.42E-05
11720770_PPIF	0.613768	5.15E-07	1.42E-05
11717117_PDXP ///	-0.7912	5.15E-07	1.42E-05
11719383_PPIH	-0.63632	5.15E-07	1.42E-05
11721731_TMX3	0.680734	5.15E-07	1.42E-05
11726142_FMN2	0.702249	5.16E-07	1.42E-05
11748171_C1orf52	0.579838	5.17E-07	1.42E-05
11751810_GINS4	-0.96235	5.17E-07	1.42E-05
11724388_ZNF721	0.913515	5.18E-07	1.42E-05
11752387_ETS1	0.91224	5.19E-07	1.42E-05
11739635_MED13	0.843011	5.19E-07	1.42E-05

11721207_FAM160B1	0.8735	5.22E-07	1.43E-05
11726618_C1GALT1C1	-0.73834	5.23E-07	1.43E-05
11721114_DERA	-0.59519	5.23E-07	1.43E-05
11748314_CDC20	-0.89363	5.25E-07	1.43E-05
11734734_FAR1	-0.62978	5.25E-07	1.43E-05
11757447_CYTH3	0.586732	5.27E-07	1.44E-05
11732857_THAP1	0.623725	5.27E-07	1.44E-05
11721305_PHLDA1	0.529681	5.28E-07	1.44E-05
11728488_NRXN3	0.747753	5.28E-07	1.44E-05
11747142_PHC3	0.76196	5.28E-07	1.44E-05
11733138_ZNF586	0.902879	5.29E-07	1.44E-05
11733093_FOXRED2	-0.66328	5.29E-07	1.44E-05
11718559_NOL4L	0.670327	5.29E-07	1.44E-05
11729267_WWC1	0.995157	5.30E-07	1.44E-05
11732821_MAP2K4	0.762276	5.32E-07	1.45E-05
11757942_UTRN	0.602947	5.34E-07	1.45E-05
11726248_PARP1	-0.72518	5.35E-07	1.45E-05
11721257_CCND1	0.609093	5.35E-07	1.45E-05
11746496_YAP1	0.640432	5.35E-07	1.45E-05
11722571_CCNA2	-0.57049	5.38E-07	1.46E-05
11725471_MAN2A1	0.556793	5.38E-07	1.46E-05
11731077_LONRF1	0.721671	5.38E-07	1.46E-05
11731652_C20orf24	-0.56799	5.39E-07	1.46E-05
11754687_ZWILCH	-0.56674	5.40E-07	1.46E-05
11731053_FNIP1	0.838272	5.41E-07	1.46E-05
11719740_DCP2	1.907338	5.43E-07	1.46E-05
11749400_TRAPPC4	-0.63084	5.43E-07	1.46E-05
11756170_CXorf38	0.580767	5.43E-07	1.46E-05
11749013_ZBED1	-0.77923	5.43E-07	1.46E-05
11716358_PRC1	-0.59533	5.43E-07	1.46E-05
11723602_MYO19	-0.62013	5.45E-07	1.47E-05
11743527_RALBP1	-0.63872	5.47E-07	1.47E-05
11720835_CDC25C	-1.01581	5.48E-07	1.47E-05
11729736_JAKMIP2	0.604852	5.48E-07	1.47E-05
11720971_TOP2A	-0.56797	5.50E-07	1.48E-05
11741354_PON2	0.560589	5.50E-07	1.48E-05
11744729_GOLGA4	0.571417	5.52E-07	1.48E-05
11732823_MAP2K4 //	0.857693	5.53E-07	1.48E-05
11747296_ARMC8	0.615914	5.53E-07	1.48E-05
11720704_TPD52L1	0.604422	5.53E-07	1.48E-05
11721483_CCDC85B	-0.96408	5.54E-07	1.48E-05
11756141_KDELR2	-0.59256	5.55E-07	1.48E-05
11740192_TMEM107	-0.58386	5.56E-07	1.49E-05
11729232_KLF3	0.58206	5.57E-07	1.49E-05
11758529_CENPA	-0.65181	5.58E-07	1.49E-05
11728283_RBMS2	0.700856	5.58E-07	1.49E-05
11744793_DLGAP5	-0.74036	5.59E-07	1.49E-05
11748491_KHDRBS1	-0.63834	5.59E-07	1.49E-05
11724509_PMAIP1	0.59422	5.62E-07	1.50E-05
11721104_POLDIP3	-0.70784	5.63E-07	1.50E-05
11749666_ITCH	0.652816	5.63E-07	1.50E-05

11716985_PNKD	-0.77837	5.63E-07	1.50E-05
11736419_ZNF800	0.734256	5.63E-07	1.50E-05
11717861_EGR1	0.836389	5.64E-07	1.50E-05
11754516_CD109	0.709555	5.66E-07	1.50E-05
11754663_COPS7A	-0.7647	5.66E-07	1.50E-05
11733796_RCAN1	-0.62317	5.66E-07	1.50E-05
11720206_CLDN12	0.644875	5.68E-07	1.51E-05
11756925_AGO3	0.718787	5.69E-07	1.51E-05
11733765_IFNB1	1.319853	5.69E-07	1.51E-05
11736277_ZNF136	0.843347	5.71E-07	1.51E-05
11740529_UBE2K	-0.67819	5.71E-07	1.51E-05
11755368_MSH6	-0.52826	5.73E-07	1.51E-05
11719774_C5orf24	0.743994	5.73E-07	1.51E-05
11741208_CDK2	-0.60876	5.73E-07	1.51E-05
11729689_LYRM7	-0.5537	5.74E-07	1.51E-05
11753515_IL32	1.128184	5.74E-07	1.51E-05
11724397_PRRG1	0.602883	5.74E-07	1.51E-05
11731755_SYTL2	0.780082	5.74E-07	1.51E-05
11740790_ELK4	0.65835	5.75E-07	1.52E-05
11728203_PHGDH	-0.62774	5.76E-07	1.52E-05
11756465_RND3	0.549888	5.76E-07	1.52E-05
11729914_ARL5B	1.082724	5.76E-07	1.52E-05
11728757_NHLRC3	0.624408	5.77E-07	1.52E-05
11753427_RND3	0.625228	5.78E-07	1.52E-05
11740105_TMEM17	-0.9158	5.78E-07	1.52E-05
11754906_PTBP2	0.597359	5.79E-07	1.52E-05
11733056_PTBP3	0.763594	5.80E-07	1.52E-05
11741947_NF1	0.782449	5.81E-07	1.52E-05
11755786_FGFR3	-0.69663	5.81E-07	1.52E-05
11720970_TOP2A	-0.59866	5.82E-07	1.53E-05
11759258_HIST2H2AC	-0.93252	5.83E-07	1.53E-05
11759347_TXK	1.266624	5.84E-07	1.53E-05
11750101_ALDH1A3	-0.57234	5.85E-07	1.53E-05
11731498_ZNF134	0.675213	5.86E-07	1.53E-05
11731887_KIF14	-0.59969	5.86E-07	1.53E-05
11752680_TMEM135	0.708395	5.86E-07	1.53E-05
11725793_PTGER4	1.142831	5.86E-07	1.53E-05
11756368_KIF22	-0.58541	5.88E-07	1.53E-05
11725569_KLHL15	0.575238	5.88E-07	1.53E-05
11746423_RNFT1	0.582059	5.92E-07	1.54E-05
11721307_PHLDA1	0.633062	5.92E-07	1.54E-05
11723010_KIF20A	-0.69306	5.93E-07	1.54E-05
11719487_LAMB3	1.066992	5.93E-07	1.54E-05
11748094_FAM3C	0.727185	5.95E-07	1.55E-05
11715221_NSUN5	-0.60439	5.95E-07	1.55E-05
11725326_CAMK2D	-0.69761	5.96E-07	1.55E-05
11728103_ZNF416	0.800614	5.97E-07	1.55E-05
11739978_AGO3	0.722708	5.98E-07	1.55E-05
11751609_ADPRM	0.572177	5.99E-07	1.55E-05
11748004_SLC16A4	0.994887	5.99E-07	1.55E-05
11722826_NCAPG	-0.61403	5.99E-07	1.55E-05

11747720_BIRC5	-0.6415	6.00E-07	1.55E-05
11720986_YRDC	0.678643	6.00E-07	1.55E-05
11724328_UBE2C	-0.60318	6.00E-07	1.55E-05
11721551_SRCAP	-0.61835	6.01E-07	1.55E-05
11763575_PAFAH1B2	-0.64352	6.01E-07	1.55E-05
11727545_PANK3	0.78802	6.02E-07	1.56E-05
11756456_C10orf2	-0.6899	6.03E-07	1.56E-05
11726392_GIMAP2	1.003606	6.04E-07	1.56E-05
11748263_DCAF17	1.086082	6.04E-07	1.56E-05
11758371_KIF15	-0.67605	6.04E-07	1.56E-05
11762631_ZFC3H1	0.988619	6.06E-07	1.56E-05
11718128_TK1	-0.65929	6.07E-07	1.56E-05
11739317_MAP3K2	0.780714	6.08E-07	1.56E-05
11720264_ASCC3	0.615254	6.08E-07	1.56E-05
11729711_MARS2	0.676505	6.09E-07	1.56E-05
11717009_MCFD2	0.617462	6.09E-07	1.56E-05
11721447_SNX4	0.584892	6.12E-07	1.57E-05
11721215_TMEM106B	-1.19012	6.13E-07	1.57E-05
11729384_CCNY	-0.80618	6.14E-07	1.58E-05
11744454_PARD3	0.572686	6.15E-07	1.58E-05
11753993_FKBP10	-0.8615	6.15E-07	1.58E-05
11720297_PLEKHF2	-0.61335	6.17E-07	1.58E-05
11752632_SMC1A	-0.56914	6.18E-07	1.58E-05
11764151_TTC17	-0.54767	6.21E-07	1.59E-05
11716325_CLK1	0.974544	6.21E-07	1.59E-05
11718484_H1F0	-0.59632	6.22E-07	1.59E-05
11742186_TMOD2	0.792125	6.22E-07	1.59E-05
11723354_CHD7	0.63659	6.22E-07	1.59E-05
11739007_CAPZA1	-0.61398	6.25E-07	1.59E-05
11719732_TIGAR	0.621628	6.25E-07	1.60E-05
11748733_KCTD7 ///	0.599787	6.26E-07	1.60E-05
11739920_ZNF195	0.694642	6.27E-07	1.60E-05
11748878_WRB	-0.56595	6.28E-07	1.60E-05
11733172_ARHGEF39	-0.70649	6.32E-07	1.61E-05
11721250_LOC150776	-0.68282	6.32E-07	1.61E-05
11719680_TNFRSF10B	-0.63124	6.32E-07	1.61E-05
11756032_CBR3	-0.66733	6.33E-07	1.61E-05
11758936_HNRNPR	-0.79974	6.34E-07	1.61E-05
11743608_FAM3C	0.787979	6.34E-07	1.61E-05
11751906_PTPRM	0.780319	6.34E-07	1.61E-05
11715473_SRPR	-0.65454	6.34E-07	1.61E-05
11720602_SYT11	0.594235	6.36E-07	1.61E-05
11726282_BCR /// B	-0.89913	6.37E-07	1.61E-05
11749094_DSE	0.731181	6.37E-07	1.61E-05
11740287_NEDD1	-0.77009	6.37E-07	1.61E-05
11726796_NEXN	0.714628	6.38E-07	1.61E-05
11749478_MCM3	-0.66141	6.38E-07	1.61E-05
11757224_C4orf27 /	-0.57485	6.39E-07	1.61E-05
11731268_NUP98	0.905448	6.39E-07	1.61E-05
11715796_LUM	0.84582	6.40E-07	1.62E-05
11734811_EGR2	0.668423	6.41E-07	1.62E-05

11727197_PCDHA1 //	0.65341	6.42E-07	1.62E-05
11718386_ZHX2	0.819777	6.44E-07	1.62E-05
11741209_LOC145783	-1.18017	6.44E-07	1.62E-05
11754858_ZNF383	1.125698	6.45E-07	1.62E-05
11754142_LOC728026	-0.71829	6.46E-07	1.62E-05
11722690_SPTBN1	-0.76315	6.48E-07	1.63E-05
11740366_SHMT1	-0.54089	6.49E-07	1.63E-05
11756586_ARL6IP4	-0.63353	6.49E-07	1.63E-05
11750840_TGFBR2	0.794854	6.50E-07	1.63E-05
11744479_RAB18	0.567844	6.51E-07	1.63E-05
11750740_ST3GAL5	0.644316	6.51E-07	1.63E-05
11736047_ANTXR2	0.73515	6.52E-07	1.64E-05
11723565_CENPA	-0.80674	6.54E-07	1.64E-05
11722832_CCNY	-0.71473	6.54E-07	1.64E-05
11756183_SLC25A11	-0.58458	6.55E-07	1.64E-05
11736394_IL32	0.957932	6.56E-07	1.64E-05
11758316_SRPR	-0.60884	6.57E-07	1.64E-05
11755251_FADS1	-0.92421	6.59E-07	1.65E-05
11754461_UCP2	-0.57024	6.60E-07	1.65E-05
11738250_CHEK1	-0.52716	6.61E-07	1.65E-05
11754183_HMGB3	-0.74257	6.61E-07	1.65E-05
11715240_ZNF184	0.664003	6.61E-07	1.65E-05
11726037_C3orf38	0.592692	6.62E-07	1.65E-05
11743901_NARS	-0.52669	6.65E-07	1.66E-05
11743503_GTF3C2	-0.64387	6.65E-07	1.66E-05
11736589_DET1	-0.73529	6.68E-07	1.66E-05
11735748_ZNF317	0.65673	6.68E-07	1.66E-05
11756082_MID1	0.581568	6.69E-07	1.66E-05
11723209_KBTBD6	-0.6592	6.69E-07	1.66E-05
11745789_TMEM135	0.55956	6.69E-07	1.66E-05
11732155_OGT	0.573335	6.70E-07	1.66E-05
11726126_ABCB9	0.75906	6.70E-07	1.66E-05
11744918_C19orf12	0.526166	6.71E-07	1.66E-05
11756747_RBMS2	0.619206	6.72E-07	1.67E-05
11730288_ARHGAP5	-1.29745	6.76E-07	1.68E-05
11751389_FEN1	-0.61844	6.77E-07	1.68E-05
11756808_ZNF225	0.877931	6.77E-07	1.68E-05
11740987_NFX1	0.579759	6.79E-07	1.68E-05
11721248_GNG2	-0.56361	6.79E-07	1.68E-05
11720512_CARHSP1	-0.63617	6.79E-07	1.68E-05
11728597_ZNF222	0.844072	6.80E-07	1.68E-05
11725379_POGLUT1	0.56214	6.80E-07	1.68E-05
11754483_PXDC1	-0.5408	6.80E-07	1.68E-05
11743536_VRK1	-0.77517	6.81E-07	1.68E-05
11722708_CCSER2	0.641932	6.82E-07	1.68E-05
11746041_TRA2B	-0.60036	6.85E-07	1.69E-05
11729062_PDGFA	0.741345	6.85E-07	1.69E-05
11758200_CKS1B	-0.6554	6.87E-07	1.69E-05
11729840_ZCCHC2	0.854665	6.87E-07	1.69E-05
11748436_CAP2	0.690735	6.90E-07	1.70E-05
11745717_HMGB1	-0.63285	6.90E-07	1.70E-05

11725403_TM4SF18	0.655824	6.90E-07	1.70E-05
11738703_ITGB3BP	-0.58612	6.91E-07	1.70E-05
11735584_BRCA1	-0.75733	6.92E-07	1.70E-05
11718940_TNFAIP3	1.363743	6.93E-07	1.70E-05
11724202_SNRPD1	-0.5779	6.93E-07	1.70E-05
11745411_TMX3	0.66093	6.94E-07	1.70E-05
11720277_OLFML2A	-0.80905	6.96E-07	1.70E-05
11743963_CNST	0.738434	6.98E-07	1.71E-05
11719598_CDCA3	-0.77048	6.98E-07	1.71E-05
11739385_ROCK2	0.557738	6.99E-07	1.71E-05
11723381_DPY19L4	0.734821	6.99E-07	1.71E-05
11740857_AGO3	0.65312	6.99E-07	1.71E-05
11743169_LPXN	0.681799	7.00E-07	1.71E-05
11750797_ZNF317	0.717012	7.02E-07	1.71E-05
11722904_TBC1D9	0.878556	7.02E-07	1.71E-05
11718674_CTNNAL1 /	0.909275	7.03E-07	1.71E-05
11741815_TAB3	0.570203	7.03E-07	1.71E-05
11740406_LHX4	0.663091	7.03E-07	1.71E-05
11743530_CENPK	-0.57448	7.04E-07	1.71E-05
11749031_PTBP3	0.749726	7.05E-07	1.71E-05
11728153_HIST1H2BD	1.174697	7.05E-07	1.71E-05
11722036_SND1	-0.69344	7.05E-07	1.72E-05
11725842_PIF1	-0.75597	7.08E-07	1.72E-05
11738060_HIST1H4H	1.418698	7.08E-07	1.72E-05
11739209_PRKAA1	0.613401	7.08E-07	1.72E-05
11715362_ATP6V0E1	0.530592	7.09E-07	1.72E-05
11717715_FNDC3B	0.601488	7.11E-07	1.72E-05
11750103_HADH	-0.51143	7.12E-07	1.72E-05
11726648_ZHX1	0.570214	7.12E-07	1.72E-05
11722627_KIAA1033	0.698322	7.12E-07	1.72E-05
11720892_SOS1	-0.95956	7.13E-07	1.73E-05
11719896_FAM91A1 /	0.586832	7.14E-07	1.73E-05
11720659_CDK9	-0.65325	7.16E-07	1.73E-05
11747743_MTF2	0.624073	7.18E-07	1.73E-05
11726458_GSPT2	0.557287	7.18E-07	1.74E-05
11725161_DDIAS	-0.56414	7.19E-07	1.74E-05
11725788_CENPN	-0.66626	7.19E-07	1.74E-05
11743653_DEK	-0.56604	7.20E-07	1.74E-05
11722370_TRIM22	1.639501	7.23E-07	1.74E-05
11717486_HARS2	-0.6064	7.24E-07	1.74E-05
11749744_TBL1XR1	0.598031	7.25E-07	1.75E-05
11751907_PTPRM	0.620477	7.26E-07	1.75E-05
11740066_ERCC6	1.054279	7.27E-07	1.75E-05
11757995_EDEM3	0.628558	7.27E-07	1.75E-05
11744661_HMG20B	-0.66613	7.28E-07	1.75E-05
11763331_VPS36	0.654555	7.29E-07	1.75E-05
11717407_RPP25L	-1.24775	7.29E-07	1.75E-05
11755469_MCM7	-0.60189	7.30E-07	1.75E-05
11731318_WBSCR27	-0.82632	7.32E-07	1.76E-05
11739957_BCL2L1	0.578892	7.32E-07	1.76E-05
11742806_TEX261	-0.87966	7.33E-07	1.76E-05

11719238_EFTUD1	0.591261	7.34E-07	1.76E-05
11757431_BLOC1S2	0.545538	7.35E-07	1.76E-05
11758164_FEN1	-0.60638	7.36E-07	1.76E-05
11745322_FDFT1	-0.61293	7.36E-07	1.76E-05
11752665_C1orf112	-0.57231	7.36E-07	1.76E-05
11723578_SMIM13	0.951836	7.36E-07	1.76E-05
11744925_TUBGCP3	-0.75231	7.39E-07	1.76E-05
11731838_SPTLC1	0.847484	7.39E-07	1.76E-05
11719343_ATF3	0.704491	7.39E-07	1.76E-05
11718958_PRKAB2	0.692257	7.39E-07	1.76E-05
11726602_DARS2	-0.56179	7.39E-07	1.76E-05
11732258_SH3RF1	0.663697	7.40E-07	1.76E-05
11717818_TNRC6A	0.658599	7.40E-07	1.76E-05
11759521_SBNO1	0.642368	7.40E-07	1.76E-05
11715436_TIMP3	-0.57351	7.46E-07	1.77E-05
11734028_FOXM1	-0.64453	7.48E-07	1.78E-05
11731319_WBSCR27	-0.6022	7.48E-07	1.78E-05
11736250_KIAA0101	-0.53335	7.48E-07	1.78E-05
11754812_NPAS2	0.609128	7.50E-07	1.78E-05
11743504_BCCIP	-0.79504	7.51E-07	1.78E-05
11733873_PGK1	-0.65807	7.51E-07	1.78E-05
11755819_DDX58	0.822937	7.54E-07	1.79E-05
11757721_CSRNP1	1.027852	7.54E-07	1.79E-05
11716311_LSM3	-0.51228	7.54E-07	1.79E-05
11751388_FEN1	-0.60301	7.57E-07	1.79E-05
11718707_IGF1R	0.636863	7.57E-07	1.79E-05
11741092_GPD2	-0.57601	7.59E-07	1.80E-05
11721362_MAP4K3	0.768718	7.60E-07	1.80E-05
11726590_ELOVL4	0.73647	7.61E-07	1.80E-05
11750976_TEX10	0.569462	7.61E-07	1.80E-05
11725298_DXO /// T	-0.64109	7.62E-07	1.80E-05
11731842_B4GALT6	-0.70516	7.63E-07	1.80E-05
11748000_C3orf17	0.547069	7.63E-07	1.80E-05
11742639_RDM1	-0.81484	7.65E-07	1.80E-05
11724431_C3orf52	1.051631	7.66E-07	1.80E-05
11756358_PLK3	0.679369	7.66E-07	1.80E-05
11733464_XDH	0.644215	7.67E-07	1.81E-05
11746433_CLDN1	1.033887	7.68E-07	1.81E-05
11736850_FSD1L	0.784222	7.69E-07	1.81E-05
11724228_RBMS1	0.628892	7.69E-07	1.81E-05
11725803_C21orf91	0.937217	7.70E-07	1.81E-05
11718198_LHFP	0.634711	7.72E-07	1.81E-05
11721218_MSRB3	0.748768	7.72E-07	1.81E-05
11750400_GNE	0.654879	7.74E-07	1.82E-05
11723451_FTO	-0.61427	7.75E-07	1.82E-05
11762402_ZMYND8	-0.53729	7.75E-07	1.82E-05
11748017_PECR	-0.57422	7.80E-07	1.83E-05
11720221_GFPT1	0.566706	7.80E-07	1.83E-05
11758872_CDC37L1	0.869949	7.81E-07	1.83E-05
11731942_PRDM1	1.2716	7.81E-07	1.83E-05
11730300_FAM19A2	1.208469	7.81E-07	1.83E-05

11755902_TRPV2	0.606604	7.82E-07	1.83E-05
11739550_MTMR2	0.548157	7.82E-07	1.83E-05
11728673_KIF22	-0.59373	7.82E-07	1.83E-05
11734299_INTS6	0.981334	7.87E-07	1.84E-05
11745049_CDC45	-0.71767	7.87E-07	1.84E-05
11756374_TPST1	0.574439	7.88E-07	1.84E-05
11726607_MTF2	0.655856	7.89E-07	1.84E-05
11763293_ZSCAN9	0.878813	7.90E-07	1.84E-05
11743373_KNSTRN	-0.64215	7.91E-07	1.84E-05
11753517_YWHAE	-0.54335	7.92E-07	1.84E-05
11743367_PLCB4	0.802665	7.92E-07	1.84E-05
11729020_ZNF804A	0.865929	7.94E-07	1.85E-05
11733897_TROVE2	0.621537	7.95E-07	1.85E-05
11716726_PLK1	-0.76267	7.97E-07	1.85E-05
11739408_KMT2C	0.753145	7.97E-07	1.85E-05
11743271_RSRC2	0.660576	7.97E-07	1.85E-05
11748275_FDFT1	-0.61072	7.98E-07	1.85E-05
11719158_RAPH1	1.002064	7.99E-07	1.85E-05
11732133_IDS	0.809714	8.00E-07	1.85E-05
11719347_ARHGAP19	-0.60198	8.01E-07	1.85E-05
11740806_CEP85L	0.610381	8.01E-07	1.85E-05
11718216_DDA1	0.577882	8.02E-07	1.86E-05
11749950_C3orf17	0.565641	8.02E-07	1.86E-05
11748826_PACSIN3	-0.84384	8.03E-07	1.86E-05
11730758_IL6ST	1.046682	8.04E-07	1.86E-05
11724134_SLC12A2	0.647855	8.05E-07	1.86E-05
11718620_CHRDL1	-0.69814	8.06E-07	1.86E-05
11760825_MYSM1	0.950333	8.08E-07	1.86E-05
11739989_CPPED1	0.604519	8.09E-07	1.87E-05
11746462_LMO7	0.867074	8.09E-07	1.87E-05
11716482_GRINA	-0.83847	8.10E-07	1.87E-05
11740998_DGKA	0.681816	8.13E-07	1.87E-05
11724744_C8orf46	0.932843	8.16E-07	1.88E-05
11733058_PTBP3	0.774515	8.16E-07	1.88E-05
11744215_RNF149	0.647341	8.18E-07	1.88E-05
11720230_TES	0.713856	8.22E-07	1.89E-05
11717932_TBK1	0.611259	8.23E-07	1.89E-05
11718127_TK1	-0.61896	8.23E-07	1.89E-05
11740681_TGIF1	0.581215	8.26E-07	1.90E-05
11754110_BIRC5	-0.65208	8.26E-07	1.90E-05
11723399_ALPK2	0.647631	8.26E-07	1.90E-05
11744692_HP1BP3	-0.51717	8.31E-07	1.90E-05
11728022_TMEM45A	-0.56763	8.34E-07	1.91E-05
11748081_FLOT1	-0.51447	8.34E-07	1.91E-05
11739283_TRIM23	0.576643	8.35E-07	1.91E-05
11726725_NABP1	0.969699	8.35E-07	1.91E-05
11746471_MAP4K4	0.517722	8.40E-07	1.92E-05
11718926_ARID5B	0.593132	8.41E-07	1.92E-05
11725837_C1orf112	-0.64044	8.42E-07	1.92E-05
11736519_CXorf38	0.518071	8.45E-07	1.93E-05
11721217_TMEM106B	-0.83368	8.45E-07	1.93E-05

11747442_NAPB	0.76757	8.45E-07	1.93E-05
11724094_COL4A5	0.567427	8.50E-07	1.94E-05
11730691_ALS2	0.682974	8.50E-07	1.94E-05
11719096_CYB5B	-0.98677	8.53E-07	1.94E-05
11743085_KDM6A	0.602077	8.55E-07	1.95E-05
11757280_DEK	-0.68049	8.58E-07	1.95E-05
11745635_TNIK	1.019911	8.60E-07	1.96E-05
11724732_TUBB	-0.54038	8.62E-07	1.96E-05
11721588_TRAF1	0.849065	8.63E-07	1.96E-05
11717267_CAMK2G	-0.61845	8.64E-07	1.96E-05
11722737_APPL1	-0.62132	8.65E-07	1.96E-05
11755120_PSME4	0.580694	8.65E-07	1.96E-05
11722367_DLGAP5	-0.72265	8.65E-07	1.96E-05
11758421_PTEN	0.617378	8.66E-07	1.96E-05
11739402_KMT5B	0.643619	8.66E-07	1.96E-05
11754566_RNF217	0.959157	8.66E-07	1.96E-05
11724953_PDIK1L	-0.95999	8.68E-07	1.97E-05
11722882_CREBRF	0.691062	8.70E-07	1.97E-05
11735324_FAM72A //	-0.55279	8.73E-07	1.98E-05
11722005_NASP	-0.56699	8.75E-07	1.98E-05
11748660_SPG20	0.50868	8.75E-07	1.98E-05
11751412_ARL1	-0.6055	8.78E-07	1.98E-05
11756106_SETBP1	0.598881	8.81E-07	1.99E-05
11733797_RCAN1	-0.61422	8.81E-07	1.99E-05
11763185_RPL7L1	-0.61117	8.82E-07	1.99E-05
11746788_PRR16	0.779583	8.85E-07	2.00E-05
11727924_PURA	-0.67528	8.87E-07	2.00E-05
11742688_HIST2H2AA	0.869414	8.89E-07	2.00E-05
11729737_RCCD1	-0.64624	8.90E-07	2.00E-05
11755641_PGAP1	0.676174	8.91E-07	2.00E-05
11732111_TNFRSF21	-0.6282	8.91E-07	2.00E-05
11752184_ZNF608	0.745886	8.91E-07	2.00E-05
11720781_CDK2	-0.54179	8.93E-07	2.01E-05
11759536_TTC17	-0.66989	8.95E-07	2.01E-05
11731538_ADGRF1	0.704153	8.95E-07	2.01E-05
11749195_DDOST	-0.66863	9.00E-07	2.02E-05
11724788_KIF3A	0.901439	9.00E-07	2.02E-05
11724679_TMEM131	0.538909	9.01E-07	2.02E-05
11753661_ID1	-0.56335	9.02E-07	2.02E-05
11733839_ABHD2	0.750206	9.02E-07	2.02E-05
11764010_TMPO	-0.90019	9.06E-07	2.03E-05
11759826_GPNMB	0.704768	9.10E-07	2.04E-05
11757005_FUCA1	0.996978	9.10E-07	2.04E-05
11734772_SYNRG	0.523979	9.10E-07	2.04E-05
11751081_NMD3	0.558692	9.11E-07	2.04E-05
11723798_CSTF3	-0.53638	9.12E-07	2.04E-05
11726611_MAFF	0.871881	9.12E-07	2.04E-05
11725003_C8orf58	0.641259	9.12E-07	2.04E-05
11729741_CD68	-0.71843	9.13E-07	2.04E-05
11752461_FMNL2	0.547395	9.13E-07	2.04E-05
11721643_ARL1	-0.80963	9.14E-07	2.04E-05

11757092_LSM3	-0.53249	9.14E-07	2.04E-05
11749307_CCNG1	0.593724	9.14E-07	2.04E-05
11754746_ZNF561	0.568264	9.18E-07	2.04E-05
11749494_GTF2H1	0.550255	9.20E-07	2.05E-05
11753820_MDM2	0.721455	9.20E-07	2.05E-05
11718943_AURKA	-0.6343	9.21E-07	2.05E-05
11723701_ABHD5	1.149304	9.22E-07	2.05E-05
11721881_TUBGCP3	-0.51983	9.24E-07	2.05E-05
11747163_CEP85L	0.587353	9.25E-07	2.05E-05
11716852_IMPAD1	-0.55011	9.26E-07	2.05E-05
11751569_SKA1	-0.71598	9.27E-07	2.06E-05
11733037_DPH3	0.626179	9.33E-07	2.07E-05
11738026_DMD	0.84769	9.33E-07	2.07E-05
11759299_ARMC8	0.737828	9.33E-07	2.07E-05
11730403_MRE11A	-0.59057	9.34E-07	2.07E-05
11748801_FDFT1	-0.63859	9.36E-07	2.07E-05
11758603_PANK3	0.797355	9.36E-07	2.07E-05
11739304_CAMK4	0.602734	9.36E-07	2.07E-05
11722143_TRANK1	0.522848	9.40E-07	2.08E-05
11749956_TBC1D31	-0.55363	9.40E-07	2.08E-05
11718088_TNKS	-0.56767	9.40E-07	2.08E-05
11745114_ADGRE2	0.671933	9.41E-07	2.08E-05
11754124_COTL1	-0.50492	9.43E-07	2.08E-05
11722022_CDK5R1	0.646921	9.45E-07	2.08E-05
11752488_CDH19	0.75589	9.46E-07	2.08E-05
11731897_PTHLH	1.117356	9.46E-07	2.08E-05
11748687_CSNK1A1	0.501359	9.46E-07	2.08E-05
11754236_PTPRF	-0.53024	9.49E-07	2.09E-05
11725791_MRE11A	-0.9679	9.49E-07	2.09E-05
11753851_ATP6V0E1	0.532057	9.50E-07	2.09E-05
11728257_RAD18	-0.75671	9.52E-07	2.09E-05
11742758_GTF2H1	0.609208	9.53E-07	2.10E-05
11736582_TRIM36	0.659253	9.54E-07	2.10E-05
11715740_RCC2	-0.7022	9.54E-07	2.10E-05
11748738_SEMA3E	1.29992	9.60E-07	2.11E-05
11744474_IDI1	-0.5816	9.60E-07	2.11E-05
11718718_TOM1L2	-0.64273	9.63E-07	2.11E-05
11716347_EFCAB14	0.557923	9.68E-07	2.12E-05
11729215_PIGX	-0.57968	9.69E-07	2.12E-05
11745470_INPP4B	0.663417	9.70E-07	2.12E-05
11726082_CCNT2	0.663652	9.70E-07	2.12E-05
11757298_BRD3	-0.65827	9.72E-07	2.12E-05
11728152_HIST1H2BD	1.103143	9.72E-07	2.12E-05
11718704_IGF1R	0.712493	9.74E-07	2.13E-05
11734848_ERBB2IP	0.545618	9.78E-07	2.14E-05
11744409_KLHL18	0.649373	9.78E-07	2.14E-05
11715697_NOLC1	-0.60758	9.79E-07	2.14E-05
11742750_GSPT1	-0.57603	9.79E-07	2.14E-05
11722575_AP1G1	0.536955	9.80E-07	2.14E-05
11756354_RRP8	0.498198	9.80E-07	2.14E-05
11731403_ATP6V1G1	-0.49449	9.81E-07	2.14E-05

11752555_FMR1	0.526196	9.81E-07	2.14E-05
11723838_GATA6	0.915352	9.83E-07	2.14E-05
11723677_CKAP2	-0.55208	9.87E-07	2.15E-05
11745838_MRS2	0.543578	9.87E-07	2.15E-05
11739079_ANP32A	-0.58889	9.88E-07	2.15E-05
11717521_PTTG1 ///	-0.53303	9.93E-07	2.16E-05
11720728_UBR1	0.611075	9.94E-07	2.16E-05
11722610_CDC42SE1	0.572099	9.95E-07	2.16E-05
11730382_SNX18	0.665816	9.97E-07	2.16E-05
11755161_PLK2	0.487089	9.99E-07	2.17E-05
11728728_ADRB2	1.356087	9.99E-07	2.17E-05
11716851_IMPAD1	-0.55015	1.00E-06	2.17E-05
11716095_KLF6	0.59353	1.00E-06	2.17E-05
11729525_CIZ1	-0.50319	1.00E-06	2.17E-05
11726362_ATXN3	-0.68753	1.00E-06	2.17E-05
11756336_ZNF23	0.680048	1.00E-06	2.17E-05
11722531_CALD1	0.563215	1.00E-06	2.17E-05
11757821_LDB1	0.598737	1.01E-06	2.17E-05
11739146_MKNK2	-0.59337	1.01E-06	2.17E-05
11717616_OXSR1	0.560655	1.01E-06	2.17E-05
11716616_REEP5	0.510209	1.01E-06	2.17E-05
11718218_RAB22A	0.598129	1.01E-06	2.17E-05
11729239_CSRP2	-0.6328	1.01E-06	2.17E-05
11732362_ZNF44	0.655837	1.01E-06	2.18E-05
11740369_PDLIM5	0.537281	1.01E-06	2.18E-05
11727022_TMEM64	0.989748	1.01E-06	2.18E-05
11751524_TMED5	0.566852	1.01E-06	2.18E-05
11743068_SIAH2	-0.52692	1.01E-06	2.18E-05
11733107_COMMD2	0.547602	1.01E-06	2.18E-05
11718160_KLF13	-0.76104	1.02E-06	2.18E-05
11751473_NAPG	0.759652	1.02E-06	2.18E-05
11745597_MICAL3	0.744554	1.02E-06	2.18E-05
11746559_CAP2	0.718967	1.02E-06	2.19E-05
11754231_TMEM185B	-0.72692	1.02E-06	2.19E-05
11730436_TAF1D	0.603402	1.02E-06	2.19E-05
11743603_RRM1	-0.64061	1.02E-06	2.19E-05
11742906_DNAJC1	0.64212	1.02E-06	2.19E-05
11750035_REEP5	0.508209	1.02E-06	2.19E-05
11736479_FAM72A //	-0.59808	1.02E-06	2.19E-05
11744827_CLK1	0.839851	1.02E-06	2.19E-05
11723180_ITSN2	0.543781	1.03E-06	2.19E-05
11745724_MALAT1	-0.5812	1.03E-06	2.20E-05
11743865_ATP2B1	0.660749	1.03E-06	2.20E-05
11763073_BIRC5	-0.60513	1.03E-06	2.20E-05
11729355_ZNF200	0.53387	1.03E-06	2.20E-05
11745098_SLC44A5	0.799219	1.03E-06	2.20E-05
11757558_LONRF1	0.719531	1.03E-06	2.20E-05
11720153_NRIP1	0.765179	1.03E-06	2.20E-05
11740881_IL15	1.038871	1.03E-06	2.20E-05
11721873_IFIT2	0.633135	1.03E-06	2.20E-05
11761515_ND1	0.489389	1.03E-06	2.20E-05

11739467_PPP4R1	0.51663	1.03E-06	2.20E-05
11733038_DPH3	0.538727	1.03E-06	2.20E-05
11740791_ELK4	0.935424	1.03E-06	2.20E-05
11744426_KIAA0101	-0.53741	1.04E-06	2.20E-05
11722522_SUSD1	1.066805	1.04E-06	2.20E-05
11718404_RFC3	-0.52428	1.04E-06	2.21E-05
11717452_NEK7	0.535471	1.04E-06	2.21E-05
11755300_SAPCD2	-1.13099	1.04E-06	2.21E-05
11754044_CLSPN	-0.71132	1.04E-06	2.21E-05
11721769_GNE	0.549165	1.04E-06	2.21E-05
11744778_DCAF8	0.819434	1.04E-06	2.21E-05
11727768_BNC2	-0.50104	1.04E-06	2.21E-05
11734413_TRIM23	0.547102	1.04E-06	2.21E-05
11725816_CPT1C	-0.58974	1.05E-06	2.22E-05
11724172_FCHO2	0.543499	1.05E-06	2.22E-05
11745297_ITGB3BP	-0.53539	1.05E-06	2.22E-05
11721775_ASPH	0.552386	1.05E-06	2.23E-05
11722902_TRMT13	0.594325	1.05E-06	2.23E-05
11746124_MAP3K7CL	1.07251	1.05E-06	2.23E-05
11731076_LONRF1	0.782044	1.06E-06	2.23E-05
11717341_PTGFRN	0.551346	1.06E-06	2.23E-05
11752492_ACVR2A	0.991615	1.06E-06	2.23E-05
11719365_SPATS2L	-0.65197	1.06E-06	2.23E-05
11723416_CNN2	0.707078	1.06E-06	2.23E-05
11749019_MYNN	0.514421	1.06E-06	2.24E-05
11738181_CNTNAP3 /	0.683324	1.07E-06	2.25E-05
11750974_PDGFA	0.620872	1.07E-06	2.25E-05
11720844_N4BP1	0.543344	1.07E-06	2.25E-05
11733931_PARP2	-0.70497	1.07E-06	2.25E-05
11753279_SPAG5	-0.6155	1.08E-06	2.26E-05
11720115_STK26	-0.59217	1.08E-06	2.27E-05
11733942_TBCEL	0.612875	1.08E-06	2.27E-05
11750607_SNX13	0.689839	1.08E-06	2.27E-05
11718003_ZMYM3	-0.59161	1.08E-06	2.27E-05
11722747_HOOK3	-0.69859	1.08E-06	2.28E-05
11720261_TRIM33	0.506938	1.08E-06	2.28E-05
11761642_TPM1	0.750958	1.09E-06	2.28E-05
11736020_OPTN	0.559734	1.09E-06	2.28E-05
11748214_TSC22D2	0.777219	1.09E-06	2.28E-05
11721144_MKI67	-0.71829	1.09E-06	2.28E-05
11739250_NUP58	-0.53954	1.09E-06	2.29E-05
11747866_RBL2	-0.61522	1.10E-06	2.29E-05
11732811_RIC1	0.720148	1.10E-06	2.30E-05
11716063_TNC	0.509982	1.10E-06	2.30E-05
11739546_NEDD1	-0.74365	1.10E-06	2.30E-05
11745846_TMEM128	-0.71804	1.10E-06	2.30E-05
11722686_LIFR	1.153101	1.11E-06	2.31E-05
11723179_ITSN2	0.663508	1.11E-06	2.31E-05
200040_PMKHDRBS1	-0.53795	1.11E-06	2.31E-05
11725336_RLIM	0.516915	1.11E-06	2.31E-05
11730515_SGOL1	-0.60699	1.11E-06	2.31E-05

11758315_PER2	1.029533	1.11E-06	2.32E-05
11726759_PRMT3	-0.71628	1.11E-06	2.32E-05
11722682_IRAK1 ///	-0.82061	1.11E-06	2.32E-05
11716062_TNC	0.590299	1.11E-06	2.32E-05
11735991_LARS	-0.77993	1.11E-06	2.32E-05
11739562_GDAP1	0.559951	1.12E-06	2.32E-05
11740420_SPAG1	0.659455	1.12E-06	2.32E-05
11722884_CREBRF	0.735066	1.12E-06	2.32E-05
11743693_MCM4	-0.57883	1.12E-06	2.32E-05
11757581_MT1X	-0.62948	1.12E-06	2.32E-05
11729271_ASB1	0.528602	1.12E-06	2.32E-05
11720930_DLST	-0.49779	1.12E-06	2.32E-05
11746316_AARS	-0.4888	1.12E-06	2.32E-05
11739576_HPS5	0.660095	1.12E-06	2.33E-05
11716330_DNAJB6	0.489097	1.13E-06	2.33E-05
11744969_PAPLN	0.695821	1.13E-06	2.34E-05
11729185_PNO1	0.526514	1.13E-06	2.34E-05
11730319_TNFRSF11B	0.651942	1.13E-06	2.34E-05
11726249_PARP1	-0.65888	1.13E-06	2.34E-05
11734305_LACTB	0.671777	1.14E-06	2.35E-05
11717698_ARIH1	0.596229	1.14E-06	2.35E-05
11736730_RIMS2	1.514413	1.14E-06	2.36E-05
11743296_CENPF	-0.58009	1.14E-06	2.36E-05
11719994_NLK	0.695018	1.14E-06	2.36E-05
11753793_KIAA0101	-0.54051	1.14E-06	2.36E-05
11735806_SSX2 ///	-0.52175	1.14E-06	2.36E-05
11747096_KCNK1	-0.63481	1.15E-06	2.36E-05
11747183_CACUL1	-0.71609	1.15E-06	2.36E-05
11757933_BTG2	0.726236	1.15E-06	2.36E-05
11720148_SH3D19	0.540319	1.15E-06	2.36E-05
11757534_FAM198B	1.276551	1.15E-06	2.36E-05
11750102_ALDH1A3	-0.53805	1.15E-06	2.37E-05
11723186_FGD6	0.665812	1.15E-06	2.37E-05
11726387_ZFP82	0.623492	1.15E-06	2.37E-05
11736741_MKI67	-0.5557	1.16E-06	2.37E-05
11719099_ISOC1	-0.72752	1.16E-06	2.37E-05
11748956_IFT88	0.623182	1.16E-06	2.37E-05
11739360_AKAP10	0.666041	1.16E-06	2.38E-05
11739730_CSNK1G1	0.584113	1.16E-06	2.38E-05
11729146_HNRNPD	-0.64306	1.16E-06	2.38E-05
11751495_YAF2	0.636231	1.16E-06	2.38E-05
11732824_MAP2K4	0.785509	1.17E-06	2.39E-05
11724248_TMEM127	-0.58434	1.17E-06	2.39E-05
11747297_STRADA	-0.65796	1.17E-06	2.39E-05
11759023_PPP2R5E	-0.5649	1.17E-06	2.39E-05
11717435_CDCA7L	-0.58903	1.17E-06	2.39E-05
11717965_KRAS	-0.62205	1.17E-06	2.40E-05
11744483_POC1B	0.535513	1.17E-06	2.40E-05
11717529_SLC25A20	0.497577	1.18E-06	2.40E-05
11733864_BIRC5	-0.50015	1.18E-06	2.41E-05
11743253_CALD1	0.494297	1.18E-06	2.42E-05

11755513_ZNF136	0.727965	1.19E-06	2.42E-05
11757849_PNMA2	-1.48003	1.19E-06	2.42E-05
11716179_C5orf15	0.49767	1.19E-06	2.42E-05
11757664_MKRN1	-0.54233	1.19E-06	2.42E-05
11757824_RGL1	0.834167	1.19E-06	2.42E-05
11733696_UBE2C	-0.53775	1.19E-06	2.42E-05
11724532_HAUS4	-0.82331	1.19E-06	2.43E-05
11719136_THOC1	-0.76295	1.20E-06	2.43E-05
11747302_ZNF337	0.568157	1.20E-06	2.44E-05
11725682_MRS2	0.675213	1.20E-06	2.44E-05
11761804_TSC22D2	0.845	1.20E-06	2.44E-05
11724026_TCF19	-0.86167	1.21E-06	2.45E-05
11744924_ZNF451	0.554392	1.21E-06	2.46E-05
11715703_ITGA5	0.616174	1.21E-06	2.46E-05
11748837_RAB30	0.727918	1.21E-06	2.46E-05
11739939_ATP8A2	0.542499	1.21E-06	2.46E-05
11716494_MAP4	-0.63511	1.21E-06	2.46E-05
11729705_ZNF561	0.925129	1.22E-06	2.46E-05
11734600_PSIP1	-0.60051	1.22E-06	2.46E-05
11758126_EAF1	0.614523	1.22E-06	2.47E-05
11717550_BTBD10	0.561285	1.22E-06	2.47E-05
11716550_HES1	0.959093	1.22E-06	2.47E-05
11724011_SIKE1	-0.68373	1.22E-06	2.47E-05
11754106_EFNA1	0.689315	1.23E-06	2.48E-05
11758249_FDPS	-0.56058	1.23E-06	2.48E-05
11729574_ENC1	0.521551	1.23E-06	2.49E-05
11723470_LPGAT1	0.535661	1.23E-06	2.49E-05
11746806_C6orf120	0.658438	1.23E-06	2.49E-05
11721668_CDT1	-0.92786	1.24E-06	2.49E-05
11726703_BCL10	0.601599	1.24E-06	2.49E-05
11716341_EFNA1	0.648591	1.24E-06	2.49E-05
11730563_STRADA	-0.6076	1.24E-06	2.50E-05
11715978_C6orf48	0.491707	1.24E-06	2.50E-05
11758902_ZNF641	0.68375	1.24E-06	2.50E-05
11722260_EDEM1	0.668003	1.25E-06	2.51E-05
11725320_CRK	0.543203	1.25E-06	2.51E-05
11744288_ZNF622	0.565326	1.25E-06	2.51E-05
11720029_LDLR	-0.59274	1.25E-06	2.51E-05
11754368_FBN1	0.592143	1.25E-06	2.51E-05
11749852_FOS	0.725882	1.25E-06	2.51E-05
11758638_MKL2	-0.49868	1.26E-06	2.52E-05
11722684_MECP2	-0.66586	1.26E-06	2.52E-05
11741056_ADORA2B	-0.66547	1.26E-06	2.52E-05
11740119_SMC2	-0.52955	1.26E-06	2.52E-05
11747085_DYNC1LI2	-0.71593	1.26E-06	2.52E-05
11725064_TCOF1	-0.87238	1.26E-06	2.52E-05
11753264_C3orf17	0.591504	1.26E-06	2.53E-05
11754877_MAP2K4 //	0.695389	1.26E-06	2.53E-05
11718403_RFC3	-0.54637	1.27E-06	2.53E-05
11748694_RAB5A	0.480105	1.27E-06	2.53E-05
11725733_NFYA	-0.71951	1.27E-06	2.54E-05

11729750_LPAR1	0.586652	1.27E-06	2.54E-05
11755778_ROBO4	-0.7945	1.27E-06	2.54E-05
11748205_ADGRE1	0.578695	1.27E-06	2.54E-05
11744391_PAPOLA	-0.65445	1.28E-06	2.54E-05
11743927_SEC24A	0.62892	1.28E-06	2.55E-05
11723589_RC3H1	0.543447	1.29E-06	2.56E-05
11749589_CTSS	0.984982	1.29E-06	2.56E-05
11718100_CD59	0.4848	1.29E-06	2.57E-05
11729549_ADGRG6	0.838066	1.29E-06	2.57E-05
11733823_AGPAT3	-0.68143	1.29E-06	2.57E-05
11718918_PAK4	-0.68709	1.29E-06	2.57E-05
11727878_CHAF1A	-0.69944	1.29E-06	2.57E-05
11716103_PCNA	-0.4927	1.29E-06	2.57E-05
11747383_BIVM	0.564794	1.29E-06	2.57E-05
11722252_NEK2	-0.52972	1.29E-06	2.57E-05
11746394_NRCAM	0.836592	1.30E-06	2.57E-05
11755818_FYCO1	-0.59175	1.30E-06	2.57E-05
11759557_BLZF1	0.808056	1.30E-06	2.58E-05
11716655_TRAF4	0.491392	1.31E-06	2.59E-05
11726605_MTF2	0.533056	1.31E-06	2.59E-05
11722408_GALNT7	0.570081	1.31E-06	2.59E-05
11755007_LOC105376	0.586932	1.31E-06	2.59E-05
11728758_CHIC2	0.526625	1.31E-06	2.60E-05
11755563_MOK	0.482581	1.31E-06	2.60E-05
11759847_ALCAM	1.008373	1.32E-06	2.60E-05
11716008_RBM5	0.491487	1.32E-06	2.61E-05
11733751_BDNF	0.736557	1.32E-06	2.61E-05
11720744_CROT	0.589857	1.32E-06	2.61E-05
11731915_ZNF607	0.545807	1.32E-06	2.61E-05
11721172_TRIM32	0.532565	1.32E-06	2.61E-05
11723147_NAV3	0.516893	1.32E-06	2.61E-05
11741292_ZFP30	0.894107	1.32E-06	2.61E-05
11739286_KMT2A	0.47726	1.32E-06	2.61E-05
11747411_HAUS7 ///	-0.5523	1.33E-06	2.61E-05
11736444_RBAK	0.554429	1.33E-06	2.62E-05
11755405_KIAA0226L	0.864167	1.34E-06	2.63E-05
11715938_KHDRBS1	-0.46667	1.34E-06	2.63E-05
11740120_SMC2	-0.84512	1.34E-06	2.64E-05
11746733_GRAMD3	0.916147	1.34E-06	2.64E-05
11716615_REEP5	-0.55945	1.34E-06	2.64E-05
11739383_IDI1	-0.59822	1.34E-06	2.64E-05
11751076_ZNF140	0.790626	1.35E-06	2.64E-05
11729351_MMP8	0.588078	1.35E-06	2.65E-05
11751818_PTGES3	-0.50978	1.35E-06	2.65E-05
11749756_ZNF791	0.785573	1.35E-06	2.65E-05
11722002_HNRNPU	-0.79931	1.36E-06	2.66E-05
11739384_ROCK2	0.73927	1.36E-06	2.66E-05
11758964_PAFAH1B2	-0.50378	1.36E-06	2.66E-05
11718528_TOR3A	-0.96563	1.36E-06	2.66E-05
11757991_ANKRD12	0.77512	1.36E-06	2.66E-05
11755873_ACSS1	-0.52096	1.36E-06	2.66E-05

11739863_TTF2	-0.59336	1.36E-06	2.66E-05
11716782_TBL1XR1	0.541834	1.36E-06	2.67E-05
11730409_ABCC2	0.593457	1.36E-06	2.67E-05
11754793_RRP12	0.546105	1.36E-06	2.67E-05
11725564_MANEA	-0.69528	1.37E-06	2.67E-05
11748457_TRIM37	0.524366	1.37E-06	2.67E-05
11755286_FAM200A	0.497416	1.37E-06	2.67E-05
11717390_LSM2	-0.76262	1.37E-06	2.67E-05
11746145_SECISBP2L	0.740089	1.37E-06	2.67E-05
11724952_CPEB2	0.700692	1.37E-06	2.67E-05
11724645_SPOPL	-0.96405	1.37E-06	2.67E-05
11757767_NAV1	-0.75462	1.37E-06	2.67E-05
11729653_SMAD2	-0.50874	1.37E-06	2.67E-05
11724350_PPP1R14C	-0.64071	1.37E-06	2.67E-05
11724057_AADAT	0.564489	1.38E-06	2.68E-05
11723105_CMPK2	-0.86614	1.38E-06	2.68E-05
11732901_APOBEC3A	-0.61753	1.38E-06	2.69E-05
11755691_DLC1	0.563153	1.38E-06	2.69E-05
11728301_CCNE2	-0.62796	1.38E-06	2.69E-05
11734397_ZMYM5	0.693436	1.39E-06	2.69E-05
11720268_IBTK	0.499116	1.39E-06	2.69E-05
11754974_NT5C2	0.524596	1.39E-06	2.70E-05
11748688_CSNK1A1	0.497845	1.39E-06	2.70E-05
11722284_NAA35	0.572444	1.39E-06	2.70E-05
11732661_STIL	-0.64593	1.39E-06	2.70E-05
11757568_ATG4B	-0.75784	1.40E-06	2.70E-05
11719046_SNRK	0.56558	1.40E-06	2.70E-05
11724905_MAP4K5	0.530862	1.40E-06	2.70E-05
11754644_CNN2	0.497648	1.40E-06	2.70E-05
11730361_CCDC126	0.482557	1.40E-06	2.70E-05
11759599_PNISR	0.602193	1.40E-06	2.70E-05
11734793_SNAPC1	0.623234	1.40E-06	2.71E-05
11718181_FERMT2	0.563323	1.40E-06	2.71E-05
11724073_LOC102724	-0.6834	1.40E-06	2.71E-05
11726395_FGD4	1.03243	1.40E-06	2.71E-05
11724358_MYO5A	-0.87868	1.40E-06	2.71E-05
11745301_DNAJC6	0.614709	1.40E-06	2.71E-05
11724366_PPIP5K1	-0.83301	1.41E-06	2.71E-05
11739006_CAPZA1	-0.47692	1.41E-06	2.71E-05
11718157_PTX3	0.846301	1.41E-06	2.71E-05
11736133_PHF19	-0.56397	1.41E-06	2.71E-05
11717240_RBBP4	-0.55247	1.42E-06	2.72E-05
11723443_EPHA4	0.80096	1.42E-06	2.72E-05
11725079_RIT1	0.547216	1.42E-06	2.73E-05
11725681_MRS2	0.778551	1.42E-06	2.73E-05
11729585_SLC25A6	-0.47099	1.42E-06	2.74E-05
11759561_PPP4R2	-0.61837	1.43E-06	2.74E-05
11751295_HARS2	-0.53088	1.43E-06	2.74E-05
11717803_NTN4	1.135606	1.43E-06	2.74E-05
11746956_ABHD2	0.627263	1.43E-06	2.74E-05
11756330_DDX58	0.564801	1.43E-06	2.74E-05

11720976_WIPF2	-0.48125	1.43E-06	2.74E-05
11763387_CDK8	-0.55915	1.44E-06	2.75E-05
11744903_C16orf59	-0.6189	1.44E-06	2.75E-05
11719320_GOLGA4	0.499226	1.44E-06	2.76E-05
11754920_VOPP1	-0.56894	1.44E-06	2.76E-05
11754462_RSPRY1	0.486904	1.44E-06	2.76E-05
11718123_AIMP1	-0.6344	1.45E-06	2.77E-05
11726259_RNF141	-0.53305	1.45E-06	2.77E-05
11746633_POLA2	-0.53814	1.45E-06	2.77E-05
11746232_KIDINS220	0.479026	1.45E-06	2.77E-05
11719122_DPCD	0.561989	1.45E-06	2.77E-05
11756194_TROAP	-0.65288	1.45E-06	2.77E-05
11715306_AREG	1.342219	1.45E-06	2.77E-05
11748201_ZNF644	0.650891	1.46E-06	2.78E-05
11715536_IDH2	-0.67713	1.46E-06	2.78E-05
11743490_RSRC2	0.556302	1.46E-06	2.78E-05
11759868_CDH19	0.848524	1.46E-06	2.78E-05
11760465_ATP11B	0.804068	1.46E-06	2.78E-05
11734728_FAM98B	-0.46617	1.46E-06	2.78E-05
11744071_LSG1	0.469125	1.46E-06	2.78E-05
11740986_CD99L2	-0.54211	1.46E-06	2.78E-05
11722183_ATP6V1C1	-0.81452	1.46E-06	2.78E-05
11723304_TRAPPC1	-0.60744	1.46E-06	2.78E-05
11739806_RASAL2	1.07003	1.47E-06	2.78E-05
11739189_AKT3	0.532154	1.47E-06	2.78E-05
11719318_GOLGA4	0.51375	1.47E-06	2.79E-05
11762245_RMND1	0.668895	1.47E-06	2.79E-05
11745453_TRMT13	0.601679	1.47E-06	2.79E-05
11749091_KDM6A	0.463286	1.47E-06	2.79E-05
11745877_HDGF	-0.50241	1.47E-06	2.79E-05
11743694_H2AFV	-0.54272	1.47E-06	2.79E-05
11730299_FAM19A2	1.016591	1.47E-06	2.79E-05
11733904_RRP15	-0.67977	1.48E-06	2.79E-05
11736705_HPS5	0.55821	1.48E-06	2.80E-05
11721864_SLC37A3	0.597792	1.48E-06	2.80E-05
11744728_GOLGA4	0.517422	1.48E-06	2.80E-05
11753915_SEL1L3	-0.73523	1.48E-06	2.80E-05
11717022_TMEM222	-0.522	1.48E-06	2.80E-05
11737545_DGKH	1.035184	1.49E-06	2.80E-05
11716638_COL4A1	0.515406	1.49E-06	2.80E-05
11740133_CALD1	0.561065	1.49E-06	2.80E-05
11717805_CASP3	0.537387	1.49E-06	2.80E-05
11761516_HNRNPM	0.597214	1.49E-06	2.80E-05
11733196_KIAA1586	0.627479	1.49E-06	2.80E-05
11729655_SMAD2	-0.8089	1.49E-06	2.80E-05
11718951_MPP5	0.689263	1.49E-06	2.81E-05
11732247_HNRNPC	-0.68	1.50E-06	2.81E-05
11729898_ZBED8	-0.94257	1.50E-06	2.83E-05
11745175_MKRN1	-0.55646	1.50E-06	2.83E-05
11728783_ING3	0.696925	1.51E-06	2.84E-05
11721730_TMX3	-0.68328	1.51E-06	2.85E-05

11727789_USP1	-0.51992	1.52E-06	2.85E-05
11739066_MAZ	-0.70813	1.52E-06	2.85E-05
11746167_ZBTB26	0.778943	1.52E-06	2.85E-05
11738156_ZBTB26	0.631379	1.52E-06	2.85E-05
11733274_ZNF485	0.890178	1.52E-06	2.86E-05
11723805_AKAP12	-0.49488	1.52E-06	2.86E-05
11720368_TMEM2	0.853738	1.53E-06	2.86E-05
11723998_SPC25	-1.13324	1.53E-06	2.86E-05
11750732_NFATC2IP	-1.00252	1.53E-06	2.86E-05
11753492_NUP37	-0.5919	1.53E-06	2.86E-05
11722710_CBX2	-0.73954	1.53E-06	2.87E-05
11750916_FAM73A	0.585232	1.53E-06	2.87E-05
11744511_ITGB3BP	-0.55898	1.53E-06	2.87E-05
11744165_PPME1	0.568443	1.54E-06	2.87E-05
11731418_RFC5	-0.62053	1.54E-06	2.87E-05
11719000_DHFR	-0.75995	1.54E-06	2.87E-05
11753254_SGOL2	-0.58975	1.54E-06	2.87E-05
11743056_PLAA	-0.52183	1.54E-06	2.88E-05
11723614_DYRK1A	0.680632	1.55E-06	2.90E-05
11720190_AP3S2 ///	-0.68326	1.56E-06	2.91E-05
11747350_BTBD10	0.598186	1.56E-06	2.91E-05
11742657_HIST2H2AA	0.831552	1.56E-06	2.91E-05
11733102_POLD3	-0.63014	1.56E-06	2.91E-05
11715992_MCM2	-0.76588	1.57E-06	2.92E-05
11727234_EPG5	0.715813	1.57E-06	2.92E-05
11716793_CCNB2	-0.60122	1.57E-06	2.92E-05
11730399_ARID3A	0.565923	1.57E-06	2.92E-05
11725712_PPIA	-0.5368	1.57E-06	2.92E-05
11749546_SLC39A6	-0.55083	1.57E-06	2.92E-05
11750799_IL31RA	0.904462	1.57E-06	2.92E-05
11743114_DHX15	-0.48225	1.58E-06	2.93E-05
11722379_GNG11	0.479138	1.58E-06	2.93E-05
11739603_BIRC5	-0.51115	1.58E-06	2.93E-05
11739074_RPL7L1	-0.49671	1.58E-06	2.93E-05
11740128_ZNF623	0.618422	1.58E-06	2.93E-05
11759613_GPALPP1	-0.61661	1.59E-06	2.94E-05
11717436_CDCA7L	-0.69885	1.59E-06	2.94E-05
11751373_PDGFC	0.866069	1.59E-06	2.95E-05
11735717_ZNF226	0.702321	1.59E-06	2.95E-05
11730865_VKORC1L1	0.455145	1.60E-06	2.95E-05
11722063_REEP3	0.836394	1.60E-06	2.96E-05
11731203_TOR1AIP2	0.763565	1.60E-06	2.96E-05
11720030_SCG2	0.52739	1.60E-06	2.96E-05
11758043_IER2	0.646209	1.60E-06	2.96E-05
11749687_MAP4K3	0.835864	1.61E-06	2.97E-05
11730848_ABHD13	0.648901	1.61E-06	2.97E-05
11722125_CCDC174	0.556439	1.61E-06	2.97E-05
11746815_SLC25A13	0.537485	1.61E-06	2.98E-05
11756338_DHRS11	-0.4495	1.61E-06	2.98E-05
11750824_PRC1	-0.60033	1.61E-06	2.98E-05
11743021_ZNF195	0.653442	1.62E-06	2.98E-05

11736170_MTMR2	0.517573	1.62E-06	2.98E-05
11719573_TRUB1	-0.55949	1.62E-06	2.98E-05
11763627_MRPL12	-0.49255	1.62E-06	2.98E-05
11744370_OBSL1	-0.5929	1.62E-06	2.99E-05
11728965_ZNF468	0.507544	1.62E-06	2.99E-05
11745873_PAPOLA	-0.72552	1.62E-06	2.99E-05
11729696_C2orf69	0.650617	1.62E-06	2.99E-05
11715937_KHDRBS1	-0.68029	1.63E-06	2.99E-05
11739177_ABHD17B	0.545183	1.63E-06	2.99E-05
11726369_BACH1 ///	0.509665	1.63E-06	2.99E-05
11735904_IFNL1	0.790951	1.63E-06	2.99E-05
11752031_KIAA0753	0.716452	1.64E-06	3.01E-05
11751737_TMEM234	0.588153	1.64E-06	3.01E-05
11720384_STX6	0.60544	1.64E-06	3.01E-05
11747943_E2F8	-0.54707	1.64E-06	3.02E-05
11721085_TRIM37	0.502256	1.65E-06	3.02E-05
11728751_OSBP	0.638212	1.65E-06	3.02E-05
11727543_OIP5	-0.62658	1.65E-06	3.02E-05
11717541_WRNIP1	-0.64507	1.65E-06	3.02E-05
11723869_ASB7	-0.49575	1.65E-06	3.02E-05
11758527_ITPR2	0.543756	1.65E-06	3.02E-05
11718093_BIVM	0.655111	1.65E-06	3.02E-05
11733494_N4BP2L2	0.757633	1.66E-06	3.03E-05
11720680_DGKA	0.570158	1.66E-06	3.03E-05
11715988_ATP5G1	-0.50681	1.66E-06	3.03E-05
11727628_ALKBH8	0.613927	1.66E-06	3.03E-05
11731697_ALG10 ///	-0.45419	1.66E-06	3.03E-05
11720561_PARP2	-0.57146	1.66E-06	3.04E-05
11751270_PI15	0.705477	1.67E-06	3.04E-05
11718188_FOXJ2	-0.51607	1.67E-06	3.04E-05
11743012_TAB2	0.566015	1.67E-06	3.04E-05
11746846_NAPEPLD	0.87519	1.67E-06	3.04E-05
11729679_PCDHB14	0.590093	1.67E-06	3.05E-05
11753996_HSPE1	-0.46017	1.67E-06	3.05E-05
11756745_FAM214A	0.847026	1.68E-06	3.05E-05
11718084_LYN	0.519362	1.68E-06	3.05E-05
11722713_XPA	0.499339	1.68E-06	3.05E-05
11740378_AFTPH	0.647329	1.68E-06	3.05E-05
11724454_SLC22A4	0.528469	1.68E-06	3.05E-05
11753907_POFUT2	0.517188	1.68E-06	3.05E-05
11745845_NME7	0.572691	1.68E-06	3.06E-05
11749048_PSMD3	-0.49436	1.69E-06	3.07E-05
11757804_CIZ1	-0.52547	1.69E-06	3.07E-05
11747262_RRAS2	0.614614	1.69E-06	3.07E-05
11746634_CKAP2	-0.53774	1.70E-06	3.07E-05
11744272_NCAPD2	-0.61612	1.70E-06	3.07E-05
11717218_STK10	0.538111	1.70E-06	3.09E-05
11731074_PGGT1B	0.49287	1.70E-06	3.09E-05
11719961_CTBP1	-0.54014	1.70E-06	3.09E-05
11744197_MYL12B	0.477811	1.71E-06	3.09E-05
11749970_GINS1	-0.89106	1.71E-06	3.09E-05

11722321_HHEX	0.548944	1.71E-06	3.09E-05
11743934_C1GALT1C1	-0.81951	1.71E-06	3.09E-05
11751886_ERBB2IP	0.487392	1.71E-06	3.09E-05
11749616_IGFBP4	-1.17264	1.71E-06	3.09E-05
11752407_EMX1 ///	-0.64173	1.72E-06	3.10E-05
11730169_SERINC3	0.506639	1.72E-06	3.11E-05
11721156_USP38	0.604328	1.72E-06	3.11E-05
11749373_MTMR10	0.718524	1.73E-06	3.11E-05
11742785_SETX	0.525408	1.73E-06	3.11E-05
11758222_SLC20A1	-0.45351	1.73E-06	3.11E-05
11722782_FGFBP2	-0.63729	1.73E-06	3.11E-05
11718640_SPR	-0.51564	1.73E-06	3.11E-05
11743270_RSRC2	0.590556	1.73E-06	3.11E-05
11738447_NFAT5	0.539097	1.73E-06	3.12E-05
11754031_CKS1B	-0.60777	1.74E-06	3.13E-05
11741223_FAM198B	1.408044	1.74E-06	3.13E-05
11763403_CELF1	0.496314	1.74E-06	3.13E-05
11739361_AKAP10	0.576962	1.74E-06	3.13E-05
11744429_STX10	-0.45441	1.75E-06	3.14E-05
11722253_NEK2	-0.48424	1.75E-06	3.14E-05
11716596_SLC44A1	-0.64985	1.75E-06	3.14E-05
11724289_TMEM256	-0.4885	1.75E-06	3.14E-05
11727765_BNC2	-0.55512	1.76E-06	3.15E-05
11718563_ANKRD10	0.633809	1.76E-06	3.15E-05
11739117_EFR3A	1.01315	1.76E-06	3.16E-05
11744823_GPR89A //	0.546291	1.76E-06	3.16E-05
11759324_CEP85L	0.564609	1.76E-06	3.16E-05
11717800_ATF5	-0.54682	1.77E-06	3.16E-05
11715916_CD44	-0.61941	1.77E-06	3.16E-05
11739219_CACUL1	-0.70214	1.77E-06	3.16E-05
11716221_EIF2S1	-0.54615	1.77E-06	3.16E-05
11755519_TGS1	-0.55771	1.77E-06	3.16E-05
11743592_TRIM4	0.576291	1.77E-06	3.16E-05
11728303_TGFA	0.578118	1.77E-06	3.17E-05
11732058_CDC7	-0.524	1.78E-06	3.18E-05
11725065_TCOF1	-0.5318	1.78E-06	3.18E-05
11746421_TRMT13	0.569574	1.78E-06	3.18E-05
11717471_PRKCA	0.827936	1.78E-06	3.18E-05
11758840_WIPF2	-0.74671	1.79E-06	3.19E-05
11729838_ZCCHC2	0.614968	1.79E-06	3.19E-05
11750664_CFAP69	0.763336	1.80E-06	3.20E-05
11731443_ARHGEF12	0.643301	1.80E-06	3.20E-05
11733031_MTFR1L	-0.60561	1.80E-06	3.20E-05
11721816_FCMR	0.916128	1.80E-06	3.20E-05
11753840_SNRPF	-0.46083	1.80E-06	3.20E-05
11746337_AP1S2	0.697006	1.80E-06	3.20E-05
11719123_TIMELESS	-0.76712	1.80E-06	3.21E-05
11734004_TMEM192 /	0.496704	1.81E-06	3.21E-05
11721511_NUS1	-1.11976	1.81E-06	3.21E-05
11750598_TPX2	-0.48997	1.81E-06	3.21E-05
11755254_METTL16	-0.49797	1.81E-06	3.21E-05

11751911_CDC42BPA	0.68811	1.81E-06	3.21E-05
11735999_ZNF791	0.816308	1.81E-06	3.22E-05
11720668_KAT2B	0.676412	1.81E-06	3.22E-05
11745271_OPTN	0.526284	1.81E-06	3.22E-05
11730278_PUS3	0.471304	1.82E-06	3.22E-05
11721667_CDT1	-0.79018	1.82E-06	3.22E-05
11732774_ZNF860	0.985517	1.82E-06	3.22E-05
11717540_RYBP	0.576753	1.82E-06	3.22E-05
11729688_LYRM7	-0.56288	1.82E-06	3.22E-05
11739243_NEMF	0.479616	1.82E-06	3.22E-05
11740652_LOC100996	0.475654	1.82E-06	3.23E-05
11725711_PPIA	-0.48673	1.83E-06	3.23E-05
11760140_CDK17	0.700196	1.83E-06	3.23E-05
11716055_STMN3	0.831578	1.83E-06	3.23E-05
11746506_SPP1	0.481797	1.83E-06	3.23E-05
11752293_USP32 ///	0.792371	1.83E-06	3.24E-05
11747443_PRPF3	0.543372	1.84E-06	3.24E-05
11729608_ZNF394	0.566872	1.84E-06	3.24E-05
11742944_CORO1A	-0.73347	1.84E-06	3.24E-05
11759016_MAN1A2	0.482992	1.84E-06	3.24E-05
11718166_RBL2	-0.6873	1.84E-06	3.25E-05
11730897_WAPL	0.493144	1.85E-06	3.25E-05
11733374_ZNF23	0.664064	1.86E-06	3.27E-05
11730831_KBTBD7	-0.61032	1.86E-06	3.27E-05
11720165_ASXL1	-0.62197	1.86E-06	3.27E-05
11727873_NHEJ1	-0.58708	1.86E-06	3.27E-05
11722644_ZNHIT6	0.689362	1.86E-06	3.27E-05
11715537_IDH2	-0.72832	1.87E-06	3.28E-05
11756857_JAK2	0.572478	1.87E-06	3.28E-05
11744674_VEZT	0.561758	1.87E-06	3.29E-05
11725660_DPP8	0.490824	1.87E-06	3.29E-05
11725057_NEDD9	0.610467	1.88E-06	3.29E-05
11752951_RPL15	-0.45746	1.88E-06	3.30E-05
11723957_LSM5	-0.93165	1.88E-06	3.30E-05
11763730_ARL6IP4	-0.63675	1.88E-06	3.30E-05
11737157_TMEM241	-0.53998	1.88E-06	3.30E-05
11757312_SERPINE1	1.26718	1.89E-06	3.30E-05
11742864_RLF	0.609351	1.89E-06	3.31E-05
11754859_KIAA0226L	0.820484	1.90E-06	3.32E-05
11755235_FAR2	0.615162	1.90E-06	3.33E-05
11752290_ROR1	1.566004	1.90E-06	3.33E-05
11749681_EXOC5	0.494394	1.90E-06	3.33E-05
11748149_FNBP1	0.621964	1.90E-06	3.33E-05
11746938_NAA30	0.633819	1.91E-06	3.33E-05
11757943_NFAT5	0.485472	1.91E-06	3.33E-05
11743616_USP47	0.497336	1.91E-06	3.33E-05
11716053_LAMC1	0.554594	1.91E-06	3.33E-05
11717755_SEC14L1	0.70988	1.91E-06	3.33E-05
11716338_INSIG1	-0.89598	1.91E-06	3.33E-05
11739960_VPS36	0.665706	1.91E-06	3.33E-05
11736759_SDHC	-0.52952	1.91E-06	3.33E-05

11732822_MAP2K4	0.658951	1.91E-06	3.33E-05
11733876_SMC4	-0.63549	1.91E-06	3.33E-05
11757793_TFPI	0.686546	1.92E-06	3.34E-05
11745708_RDM1	-1.06475	1.92E-06	3.34E-05
11724343_ITGA4	0.539402	1.92E-06	3.34E-05
11749028_PAPD4	0.486776	1.92E-06	3.34E-05
11719604_PTPN9	-0.49412	1.92E-06	3.34E-05
11736670_MCM4	-0.51759	1.92E-06	3.34E-05
11740782_RNF128	0.665724	1.92E-06	3.34E-05
11724160_GXYLT1	0.632058	1.92E-06	3.34E-05
11743713_ARID4A	0.68374	1.92E-06	3.34E-05
11744057_DDOST	-0.52979	1.92E-06	3.34E-05
11758583_TMEM154	-0.49436	1.92E-06	3.34E-05
11735300_FANCB	-0.73461	1.93E-06	3.34E-05
11736083_GTSE1	-0.56074	1.93E-06	3.34E-05
11753284_HAUS7	-0.55943	1.93E-06	3.35E-05
11742848_ADPRM	0.69065	1.93E-06	3.35E-05
11763599_GPATCH2L	0.658645	1.93E-06	3.35E-05
11718876_DNER	0.790547	1.93E-06	3.35E-05
11751330_CCND1	0.571879	1.94E-06	3.35E-05
11755639_CDC42BPA	0.526471	1.94E-06	3.35E-05
11715361_ATP6V0E1	0.522311	1.94E-06	3.35E-05
11716009_DYNC1LI2	-0.62443	1.94E-06	3.35E-05
11728852_TUB	-0.82421	1.94E-06	3.36E-05
11717060_DECR1	0.537407	1.95E-06	3.36E-05
11728691_MKRN1	-0.54861	1.95E-06	3.36E-05
11728385_SIX5	-0.64407	1.95E-06	3.36E-05
11744210_RBM14-RBM	0.498161	1.95E-06	3.36E-05
11754666_CORO1A	-0.55608	1.95E-06	3.36E-05
11717020_TMEM222	-0.55826	1.95E-06	3.36E-05
11754320_SMC4	-0.48222	1.95E-06	3.36E-05
11733181_ETV1	-0.81211	1.95E-06	3.37E-05
11742232_FAM3C	0.67751	1.96E-06	3.37E-05
11727499_ZRANB1	0.509389	1.96E-06	3.37E-05
11721681_RBMX2	-0.61604	1.96E-06	3.37E-05
11726252_SLC1A3	1.388772	1.96E-06	3.37E-05
11736310_CSNK1A1	0.439845	1.96E-06	3.37E-05
11729718_SMAD5	-0.99105	1.96E-06	3.37E-05
11725683_MRS2	0.503438	1.96E-06	3.37E-05
11740698_AKNAD1	0.900849	1.96E-06	3.37E-05
11753494_RAB18	0.564348	1.97E-06	3.38E-05
11723678_CKAP2	-0.70279	1.97E-06	3.38E-05
11726636_KAT6A	-0.48916	1.97E-06	3.38E-05
11720304_RTN3	-0.47696	1.97E-06	3.38E-05
11758203_MID1	0.532679	1.97E-06	3.39E-05
11744586_GPATCH2	0.725489	1.98E-06	3.39E-05
11751932_STIL	-0.54774	1.98E-06	3.39E-05
11719546_GOLGA1	0.471442	1.98E-06	3.40E-05
11721387_LACTB	0.505958	1.98E-06	3.40E-05
11720755_STAT3	0.548056	1.99E-06	3.40E-05
11729063_PDGFA	0.674547	1.99E-06	3.40E-05

11717423_RBM8A	-0.44359	1.99E-06	3.40E-05
11747840_NSMCE4A	-0.46345	2.00E-06	3.42E-05
11718182_CDCA5	-0.87488	2.00E-06	3.42E-05
11741571_ZNF682	0.513344	2.00E-06	3.42E-05
11727441_LOXL4	0.951256	2.00E-06	3.42E-05
11716193_SETD2	0.463814	2.00E-06	3.42E-05
11746942_DYNC1LI2	-0.69258	2.00E-06	3.42E-05
11719457_EXOC5	0.439818	2.00E-06	3.42E-05
11748338_SLC31A2	0.54566	2.01E-06	3.43E-05
11723259_ZNF24	0.81338	2.01E-06	3.43E-05
11747922_RBFOX2	0.476184	2.01E-06	3.43E-05
11750034_REEP5	0.549918	2.02E-06	3.44E-05
11719924_RBM12	-0.72514	2.02E-06	3.44E-05
11752598_ZDHHC20	0.623792	2.02E-06	3.44E-05
11763708_MOK	0.459664	2.02E-06	3.44E-05
11721563_MELK	-0.53073	2.03E-06	3.45E-05
11755986_EGFLAM	0.611496	2.03E-06	3.45E-05
11725614_PPP1CB	-0.49776	2.04E-06	3.46E-05
11763298_ERVK3-1 /	0.48399	2.04E-06	3.46E-05
11761149_C5orf45	0.544564	2.04E-06	3.47E-05
11724570_APPBP2	0.643807	2.04E-06	3.47E-05
11723835_C2orf42	0.53853	2.04E-06	3.47E-05
11731005_MIS18BP1	-0.79695	2.05E-06	3.48E-05
11730742_AKIRIN1	0.560846	2.05E-06	3.48E-05
11717682_GNA13	0.716953	2.05E-06	3.48E-05
11717196_MEST	-0.68701	2.05E-06	3.48E-05
11751011_SHMT1	-0.51078	2.05E-06	3.48E-05
11763171_FOSL2	0.98213	2.06E-06	3.49E-05
11740007_POLR3G	-0.69907	2.06E-06	3.49E-05
11758477_ANKRD13C	0.773252	2.06E-06	3.49E-05
11724344_ITGA4	0.838409	2.06E-06	3.49E-05
11736932_ZNF506	0.578042	2.06E-06	3.49E-05
11720327_POLR2C	-0.80991	2.06E-06	3.49E-05
11718680_ADGRE5	0.47365	2.07E-06	3.51E-05
11747522_ZSCAN26	0.585906	2.07E-06	3.51E-05
11752190_ZDHHC9	0.54497	2.07E-06	3.51E-05
11750887_BIRC2	0.455548	2.07E-06	3.51E-05
11739085_PCID2	0.572824	2.08E-06	3.51E-05
11729716_CBR1	-0.44815	2.08E-06	3.52E-05
11763712_PPP1R1C	0.770392	2.08E-06	3.52E-05
11750441_LMNB1	-0.67022	2.09E-06	3.52E-05
11739224_NF1	0.746722	2.09E-06	3.52E-05
11734997_SKI	0.722232	2.09E-06	3.52E-05
11727380_H2AFV	-0.48356	2.09E-06	3.52E-05
11725678_LEPROT	-0.503	2.09E-06	3.52E-05
11725321_CRK	0.534733	2.09E-06	3.52E-05
11744602_TMEM39A	-0.64532	2.09E-06	3.52E-05
11757459_WIPF1	-0.59669	2.09E-06	3.53E-05
11751494_YAF2	0.707132	2.10E-06	3.53E-05
11725781_ZC3H8	0.648039	2.10E-06	3.53E-05
11723884_SOWAHC	0.609402	2.10E-06	3.53E-05

11755456_AP2S1	-0.49568	2.10E-06	3.54E-05
11744274_NDC80	-0.74244	2.12E-06	3.56E-05
11743046_FLOT1	-0.54312	2.12E-06	3.56E-05
11716611_AP2S1	-0.47302	2.12E-06	3.56E-05
11727970_SLC14A1	0.734467	2.12E-06	3.57E-05
11739190_AKT3	0.51547	2.13E-06	3.57E-05
11735063_ITGB3	0.57856	2.13E-06	3.57E-05
11739805_RASAL2	0.499358	2.13E-06	3.57E-05
11744738_LOC145783	-1.46007	2.13E-06	3.57E-05
11736736_ZNF621	0.479666	2.13E-06	3.57E-05
11727737_AKAP7	-1.20557	2.13E-06	3.57E-05
11717314_HAUS1	-0.51913	2.13E-06	3.57E-05
11750741_TAB2	0.529201	2.14E-06	3.58E-05
11750154_RGS3	0.539705	2.14E-06	3.58E-05
11753986_CBLB	0.755029	2.14E-06	3.58E-05
11737795_KIAA1524	-0.63053	2.14E-06	3.58E-05
11747384_BIVM	0.557472	2.14E-06	3.58E-05
11739008_CAPZA1	-0.51536	2.14E-06	3.58E-05
11727188_WNT5A	0.470334	2.15E-06	3.59E-05
11746800_HACD3	-0.47526	2.15E-06	3.59E-05
11758116_APPBP2	-0.64678	2.15E-06	3.59E-05
11745671_FMN2	0.62329	2.15E-06	3.59E-05
11749260_MAGEA4	-0.54786	2.16E-06	3.60E-05
11721737_TNFRSF19	-0.76298	2.16E-06	3.60E-05
11746395_NRCAM	0.811363	2.16E-06	3.60E-05
11722637_ANKRD28	-0.47407	2.16E-06	3.60E-05
11747001_ZNF615	0.489384	2.16E-06	3.60E-05
11744162_DUSP4	0.466005	2.16E-06	3.60E-05
11755686_CPOX	-0.54391	2.16E-06	3.60E-05
11750196_ANXA4	0.506617	2.17E-06	3.60E-05
11721648_GPATCH4	0.487334	2.17E-06	3.60E-05
11728101_ZNF367	0.545297	2.17E-06	3.60E-05
11721609_PABPC1L	0.53056	2.17E-06	3.61E-05
11720647_BUB1B	-0.74309	2.17E-06	3.61E-05
11745241_VEZT	0.54134	2.17E-06	3.61E-05
11745254_POLR3C	0.493999	2.18E-06	3.61E-05
11726124_TCN1	0.991216	2.18E-06	3.61E-05
11744005_PHLDA2	0.448111	2.18E-06	3.62E-05
11747458_MOK	0.555934	2.18E-06	3.62E-05
11754680_MAP3K2	0.531404	2.19E-06	3.63E-05
11716007_RBM5	0.54798	2.19E-06	3.63E-05
11760246_OGT	0.465945	2.19E-06	3.63E-05
11743729_CCDC47	-0.5368	2.20E-06	3.64E-05
11720423_AFF4	1.031586	2.20E-06	3.64E-05
11744595_APTX	-0.5909	2.20E-06	3.64E-05
11718005_CDC25B	-0.51187	2.20E-06	3.65E-05
11758050_RGS3	0.466515	2.20E-06	3.65E-05
11724028_HIC2	0.603379	2.21E-06	3.65E-05
11743737_NR3C1	-0.49608	2.21E-06	3.65E-05
11719184_ECD	0.45175	2.21E-06	3.66E-05
11724038_PTGS2	0.770306	2.21E-06	3.66E-05

11717676_CYFIP2	0.660985	2.22E-06	3.66E-05
11760057_MICAL3	0.669201	2.22E-06	3.67E-05
11759779_ZNF800	1.099422	2.22E-06	3.67E-05
11756943_MAP2K4	0.604418	2.22E-06	3.67E-05
11723267_KPNA6	0.611293	2.22E-06	3.67E-05
11742744_STX10	-0.45407	2.22E-06	3.67E-05
11726270_NKIRAS2	0.501218	2.23E-06	3.67E-05
11720258_TRAPPC6B	0.650537	2.23E-06	3.67E-05
11723947_ACSL5	1.027265	2.23E-06	3.67E-05
11755109_CYFIP2	0.667413	2.23E-06	3.67E-05
11719980_FMNL2	0.453479	2.23E-06	3.67E-05
11726591_ELOVL4	0.699509	2.23E-06	3.67E-05
11718911_PDZD2	-0.94923	2.23E-06	3.67E-05
11746638_DHRS11	-0.53289	2.24E-06	3.68E-05
11753263_C3orf17	0.576217	2.24E-06	3.68E-05
11728731_DSCC1	-0.93451	2.24E-06	3.68E-05
11745459_FADS1	-0.48428	2.24E-06	3.68E-05
11742905_DNAJC1	0.641095	2.26E-06	3.71E-05
11717504_CANT1	-0.43291	2.26E-06	3.71E-05
11755061_SLTM	0.472848	2.26E-06	3.71E-05
11750794_TINAGL1	0.633518	2.26E-06	3.71E-05
11750442_LMNB1 ///	-1.08037	2.26E-06	3.71E-05
11751549_CD46	0.440527	2.26E-06	3.71E-05
11759601_PNISR	0.477084	2.27E-06	3.72E-05
11722120_KNTC1	-0.61638	2.27E-06	3.72E-05
11717892_PTP4A1	0.519796	2.27E-06	3.72E-05
11720931_DLST	-0.64601	2.27E-06	3.72E-05
11718120_ATP6V1A	0.542901	2.27E-06	3.72E-05
11731741_ZNF224	0.540813	2.28E-06	3.72E-05
11730232_TIAL1	0.513471	2.28E-06	3.73E-05
11757075_RPL15	-0.54995	2.28E-06	3.73E-05
11720721_RRAS2	0.682672	2.29E-06	3.74E-05
11727490_KIF11	-0.63368	2.29E-06	3.74E-05
11747298_S100A16	0.485025	2.29E-06	3.74E-05
11723197_HNRNPA3	-0.95286	2.29E-06	3.74E-05
11754676_CENPE	-0.61119	2.29E-06	3.74E-05
11724079_E2F2	-0.51284	2.30E-06	3.75E-05
11717394_ARIH2	-0.61343	2.30E-06	3.76E-05
11722694_ZMYM4	0.510206	2.30E-06	3.76E-05
11740170_ARHGAP42	1.174878	2.30E-06	3.76E-05
11717866_PSME4	0.499079	2.31E-06	3.76E-05
11738082_POC1B	0.720002	2.31E-06	3.76E-05
11736758_SDHC	-0.52429	2.31E-06	3.77E-05
11745300_DNAJC6	0.489057	2.32E-06	3.77E-05
11741575_DEK	-0.70344	2.32E-06	3.77E-05
11743924_GJA5	0.708558	2.32E-06	3.77E-05
11752930_GBP1	1.092794	2.32E-06	3.77E-05
11753631_ATF3	0.603727	2.33E-06	3.78E-05
11751381_C8orf46	1.161569	2.34E-06	3.80E-05
11718809_PRKCH	0.557915	2.34E-06	3.80E-05
11750580_ARHGEF12	0.857404	2.34E-06	3.80E-05

11715614_BTG2	0.571097	2.34E-06	3.80E-05
11740032_DUSP14	0.478214	2.34E-06	3.81E-05
11732998_MCM6	-0.96142	2.35E-06	3.81E-05
11741032_HMMR	-0.50378	2.35E-06	3.81E-05
11720319_SPAG5	-0.52753	2.35E-06	3.82E-05
11720630_SNRNP25	-0.53955	2.35E-06	3.82E-05
11755047_NPLOC4	-0.55635	2.35E-06	3.82E-05
11757428_SF3B5	-0.56827	2.36E-06	3.82E-05
11743381_PRPF40A	-0.70773	2.36E-06	3.82E-05
11720443_BAZ1A	0.43752	2.36E-06	3.82E-05
11733075_PPP1R12A	0.587314	2.36E-06	3.82E-05
11743674_KDM6B	0.760262	2.36E-06	3.82E-05
11719408_HIPK2	0.529752	2.36E-06	3.82E-05
11726441_ERCC6L	-0.72844	2.36E-06	3.82E-05
11736647_CRK	0.500827	2.36E-06	3.82E-05
11720154_NRIP1	0.770818	2.36E-06	3.82E-05
11715850_C6orf48	0.479275	2.36E-06	3.82E-05
11743939_CCDC90B	0.44598	2.37E-06	3.83E-05
11721735_DHTKD1	-0.84389	2.37E-06	3.83E-05
11760037_GLS	0.442168	2.37E-06	3.83E-05
11758175_SLC36A1	0.660915	2.38E-06	3.83E-05
11725182_NEK6	0.642158	2.38E-06	3.83E-05
11729858_H6PD	-0.55393	2.38E-06	3.84E-05
11717046_C11orf95	-0.47426	2.38E-06	3.84E-05
11731888_KIF14	-0.51981	2.38E-06	3.84E-05
11717915_BIVM-ERCC	0.463447	2.38E-06	3.84E-05
11741141_NEDD4L	0.689204	2.38E-06	3.84E-05
11721649_GPATCH4	0.484022	2.38E-06	3.84E-05
11731145_SYNE1	0.77459	2.39E-06	3.85E-05
11716171_LMNB2	-0.47686	2.39E-06	3.85E-05
11760109_TMEM41B	-0.738	2.39E-06	3.85E-05
11736464_ZNF773	0.818078	2.39E-06	3.85E-05
11731738_PGAP1	0.617615	2.40E-06	3.85E-05
11736637_DENND4A	0.654949	2.40E-06	3.85E-05
11750648_RPS6KB1	0.52971	2.40E-06	3.85E-05
11726797_NEXN	0.552466	2.40E-06	3.86E-05
11756910_FANCD2	-0.4433	2.40E-06	3.86E-05
11735833_CEMIP	-0.49731	2.41E-06	3.86E-05
11754992_CHD1	0.571384	2.41E-06	3.86E-05
11718600_TM2D2	-0.55053	2.41E-06	3.86E-05
11749304_HNRNPD	-0.44572	2.41E-06	3.86E-05
11725259_PPM1B	0.631806	2.41E-06	3.86E-05
11716218_PRKAB1	0.476866	2.42E-06	3.87E-05
11749814_NEK2	-0.49605	2.42E-06	3.87E-05
11721610_KIAA0753	0.759201	2.42E-06	3.88E-05
11718501_UBE2Q1	0.443374	2.42E-06	3.88E-05
11717538_RYBP	0.490338	2.43E-06	3.88E-05
11723509_ZNF609	0.541743	2.43E-06	3.88E-05
11715728_ITGB1	-0.52031	2.43E-06	3.89E-05
11726379_WHSC1	-0.57562	2.44E-06	3.90E-05
11727790_SPP1	0.488496	2.45E-06	3.91E-05

11720252_C20orf194	0.729144	2.45E-06	3.91E-05
11755981_HBEGF	0.911791	2.45E-06	3.92E-05
11741492_FAM111B	-0.84566	2.46E-06	3.92E-05
11740067_ERCC6	0.563136	2.46E-06	3.92E-05
11742984_EGFLAM	0.788987	2.46E-06	3.93E-05
11751294_ST6GALNAC	1.183955	2.46E-06	3.93E-05
11724686_IFNAR2	0.523696	2.47E-06	3.94E-05
11748771_YTHDF3	0.504227	2.47E-06	3.94E-05
11737760_RND3	0.501665	2.47E-06	3.94E-05
11732403_OGFRL1	0.820924	2.48E-06	3.94E-05
11740118_SMC2	-0.59381	2.48E-06	3.94E-05
11739216_RSAD2	1.007799	2.48E-06	3.94E-05
11759509_MCM4	-0.63152	2.48E-06	3.94E-05
11721021_MTMR3	0.471667	2.48E-06	3.94E-05
11742933_PSIP1	-0.43831	2.49E-06	3.95E-05
11731526_TRIP13	-0.52041	2.49E-06	3.96E-05
11759551_OBSL1	-0.63114	2.49E-06	3.96E-05
11731201_FIGN	0.53064	2.50E-06	3.97E-05
11759038_IL6ST	0.529329	2.50E-06	3.97E-05
11739003_HDGF	-0.49945	2.50E-06	3.97E-05
11737479_PON2	0.536795	2.50E-06	3.97E-05
11740929_BAGE ///	0.743317	2.50E-06	3.97E-05
11718574_SERBP1	-0.52843	2.51E-06	3.98E-05
11753809_LSM3	-0.47592	2.51E-06	3.98E-05
11747080_TARS2	-0.58265	2.51E-06	3.98E-05
11736052_EEF1A1	-0.55082	2.51E-06	3.98E-05
11747367_RAB18	0.510898	2.52E-06	3.99E-05
11718071_NCOR2	-0.43628	2.53E-06	4.00E-05
11728935_KIAA1549L	-0.65397	2.53E-06	4.00E-05
11756360_H2AFZ	-0.44659	2.53E-06	4.00E-05
11741556_AKIRIN1	0.541479	2.54E-06	4.01E-05
11750738_PAK4	-0.57324	2.54E-06	4.01E-05
11746650_ACER3	-0.77962	2.54E-06	4.02E-05
11756477_TTLL11	0.923146	2.55E-06	4.03E-05
11747219_ARID4B	0.738348	2.56E-06	4.04E-05
11743770_MAB21L1 /	0.655489	2.56E-06	4.04E-05
11748882_C1orf50	0.526577	2.56E-06	4.04E-05
11716346_EFCAB14	0.500219	2.56E-06	4.04E-05
11737161_CCNT2	0.889534	2.56E-06	4.04E-05
11747673_SACM1L	-0.51918	2.57E-06	4.05E-05
11753379_MAPK1	0.554379	2.57E-06	4.05E-05
11727130_CPD	0.528646	2.57E-06	4.05E-05
11738092_PDE9A	0.646696	2.57E-06	4.05E-05
11754184_ALDH1A3	-0.468	2.57E-06	4.05E-05
11728159_ATP6V0A4	0.568629	2.57E-06	4.05E-05
11744216_C15orf57	0.503796	2.58E-06	4.05E-05
11760158_HMGB1	-0.52331	2.58E-06	4.06E-05
11744573_INO80	0.5922	2.58E-06	4.06E-05
11730171_AKAP8L	0.641798	2.58E-06	4.06E-05
11735718_GSTCD	-0.66429	2.58E-06	4.06E-05
11724812_ZNF346	0.502109	2.58E-06	4.06E-05

11735804_NAT6	-0.54199	2.59E-06	4.06E-05
11726961_SGTB	0.751514	2.59E-06	4.06E-05
11717863_DUSP5	0.647323	2.59E-06	4.06E-05
11743277_UTRN	0.749433	2.59E-06	4.06E-05
11761125_SNX5	0.655962	2.59E-06	4.06E-05
11747742_MTF2	0.481876	2.59E-06	4.06E-05
11718248_KIAA0430	-0.58588	2.59E-06	4.06E-05
11755897_FN1	0.64386	2.59E-06	4.06E-05
11751040_RAB18	0.493339	2.59E-06	4.07E-05
11717986_APOC1	-0.52457	2.60E-06	4.07E-05
11744365_ZFAS1	0.436679	2.60E-06	4.08E-05
11755843_PCNXL2	0.592483	2.61E-06	4.09E-05
11743340_SMARCA2	-0.48038	2.61E-06	4.09E-05
11734817_APBB2	0.621334	2.62E-06	4.10E-05
11733884_DEPDC1	-0.46817	2.62E-06	4.10E-05
11739552_CREB5	1.146385	2.62E-06	4.10E-05
11735281_BTRC	0.523122	2.63E-06	4.10E-05
11723713_E2F1	-0.62139	2.63E-06	4.10E-05
11731991_TROAP	-0.6905	2.63E-06	4.10E-05
11716445_COMMD4 //	-0.51866	2.63E-06	4.10E-05
11720051_SPOCK1	0.562989	2.63E-06	4.10E-05
11729609_ZNF394	0.549556	2.63E-06	4.11E-05
11723038_SRSF3	-0.4962	2.63E-06	4.11E-05
11736565_PTPN14	0.666038	2.64E-06	4.11E-05
11745940_PSD3	0.485504	2.64E-06	4.11E-05
11727163_ACVR2A	1.060883	2.64E-06	4.11E-05
11745767_MMP2	0.473838	2.64E-06	4.11E-05
11720198_RNF6	0.556837	2.65E-06	4.12E-05
11743602_RRM1	-0.48975	2.65E-06	4.12E-05
11723241_CDK6	0.483803	2.65E-06	4.12E-05
11731595_STAM2	-0.55874	2.66E-06	4.13E-05
11724399_DDX60L	0.63174	2.66E-06	4.13E-05
11724922_VPS13B	0.583934	2.66E-06	4.13E-05
11728957_E2F8	-0.92349	2.66E-06	4.14E-05
11737042_PHLDB2	0.605394	2.67E-06	4.15E-05
11716636_AARS	-0.45918	2.67E-06	4.15E-05
11722199_ADAM12	0.688824	2.67E-06	4.15E-05
11720929_DLST	-0.66413	2.68E-06	4.15E-05
11759340_DENND1B	0.749467	2.68E-06	4.16E-05
11739943_SEPSECS	0.45029	2.68E-06	4.16E-05
11731113_PEMT	-0.55241	2.68E-06	4.16E-05
11739228_FBXW11	-0.44612	2.68E-06	4.16E-05
11724292_TRIT1	0.442538	2.68E-06	4.16E-05
11721288_WWOX	0.553422	2.68E-06	4.16E-05
11741186_HIST1H3D	0.926532	2.69E-06	4.16E-05
11747839_LPAR1	0.744102	2.69E-06	4.17E-05
11719882_OSTM1	0.508893	2.69E-06	4.17E-05
11732690_C9orf85	0.62841	2.70E-06	4.17E-05
11719813_SLC48A1	0.55871	2.70E-06	4.17E-05
11724291_TRIT1	0.427824	2.70E-06	4.18E-05
11725097_RABL3	-1.18837	2.70E-06	4.18E-05

11726319_WWP1	0.440396	2.71E-06	4.19E-05
11721171_TRIM32	0.536685	2.71E-06	4.19E-05
11726104_PPP2R3A	-0.6581	2.71E-06	4.19E-05
11749608_RNF11	0.591903	2.72E-06	4.20E-05
11741225_CASC5	-0.67591	2.72E-06	4.20E-05
11719837_LGALS8	0.465874	2.72E-06	4.20E-05
11746691_ARID4B	0.682669	2.72E-06	4.20E-05
11749401_TRIT1	0.493258	2.73E-06	4.21E-05
11716710_ADM	0.418429	2.73E-06	4.22E-05
11733028_AIDA	-0.71039	2.74E-06	4.23E-05
11760222_CASP4	0.576237	2.74E-06	4.23E-05
11715466_ARHGDIB	0.486082	2.75E-06	4.23E-05
11716138_TMEM30A	0.474213	2.75E-06	4.23E-05
11736000_ZNF791	0.632395	2.75E-06	4.23E-05
11727686_KCTD18	-0.72972	2.75E-06	4.24E-05
11745150_LOC100506	0.71989	2.76E-06	4.25E-05
11744675_FAM208A	0.447341	2.76E-06	4.25E-05
11736001_DCN	0.805352	2.76E-06	4.25E-05
11732448_EXOG	0.522998	2.77E-06	4.25E-05
11747652_CDC25B	-0.47692	2.77E-06	4.26E-05
11715864_HACD3	-0.4353	2.78E-06	4.26E-05
11746352_SLMAP	0.531688	2.78E-06	4.27E-05
11727037_MAVS	-0.47835	2.78E-06	4.27E-05
11737611_ZDHHC21	0.7705	2.80E-06	4.29E-05
11721914_MDM4	0.563453	2.80E-06	4.29E-05
11741643_ZFX	0.728132	2.80E-06	4.29E-05
11716404_EIF4EBP2	-0.50511	2.80E-06	4.29E-05
11760530_AGO3	0.79822	2.80E-06	4.29E-05
11732205_NAP1L1	-0.50793	2.80E-06	4.29E-05
11733616_HNMT	0.669318	2.80E-06	4.29E-05
11724036_PTGS2	0.755089	2.81E-06	4.29E-05
11758125_DEPDC1B	-0.45372	2.81E-06	4.30E-05
11719338_SLC23A2	0.612167	2.82E-06	4.32E-05
11738121_SNX18	0.720253	2.83E-06	4.32E-05
11758536_KCTD15	-0.46135	2.83E-06	4.33E-05
11737793_IDH3A	-0.54773	2.83E-06	4.33E-05
11742025_NMNAT2	0.49387	2.84E-06	4.34E-05
11747969_RGL1	0.835031	2.84E-06	4.34E-05
11744306_LRR1	-0.58017	2.84E-06	4.34E-05
11732171_GRSF1	-0.90966	2.85E-06	4.35E-05
11746974_LDLR	-0.54765	2.86E-06	4.36E-05
11752281_RBM5	0.59472	2.86E-06	4.36E-05
11744603_GTPBP6	-0.45366	2.86E-06	4.36E-05
11749073_TNFRSF19	-0.74112	2.86E-06	4.36E-05
11716621_TNPO1	0.464017	2.86E-06	4.37E-05
11758286_POLI	-0.62269	2.87E-06	4.37E-05
11753760_CDK2AP2	-0.47659	2.87E-06	4.37E-05
11732713_FST	-0.80139	2.87E-06	4.38E-05
11719800_C6orf62	-0.55806	2.88E-06	4.38E-05
11718422_CCNB1IP1	0.437512	2.88E-06	4.39E-05
11740719_PRMT3	-0.55625	2.88E-06	4.39E-05

11750066_NR2F1-AS1	0.649109	2.89E-06	4.39E-05
11735062_ITGB3	0.717398	2.89E-06	4.40E-05
11726042_STK17B	0.795261	2.89E-06	4.40E-05
11717286_HLTF	-0.49184	2.90E-06	4.40E-05
11722801_SS18	0.464847	2.90E-06	4.40E-05
11719140_NFX1	0.640839	2.91E-06	4.41E-05
11744232_RFC5	-0.62597	2.92E-06	4.43E-05
11720646_SPTLC2	0.589804	2.92E-06	4.43E-05
11719971_PACSIN3	-0.80356	2.92E-06	4.43E-05
11722533_CALD1	0.526592	2.93E-06	4.44E-05
11744797_DAB2	0.635384	2.93E-06	4.44E-05
11716388_BAZ1B	-0.45641	2.93E-06	4.44E-05
11737040_PHLDB2	0.472653	2.93E-06	4.44E-05
11721098_TMX1	-0.41885	2.93E-06	4.44E-05
11728802_PAPD5	0.511846	2.94E-06	4.45E-05
11719934_TKT	-1.59986	2.94E-06	4.45E-05
11754581_CERS6	-0.55639	2.94E-06	4.45E-05
11727439_RALGAPB	0.429752	2.94E-06	4.46E-05
11757413_NDUFS3	-0.50542	2.95E-06	4.46E-05
11718122_ATP6V1A	0.608618	2.95E-06	4.47E-05
11744412_TMEM131	0.527415	2.96E-06	4.47E-05
11723439_SRPX2	0.592942	2.96E-06	4.47E-05
11723971_APTX	-0.6485	2.96E-06	4.47E-05
11733000_ICAM1	0.474032	2.97E-06	4.49E-05
11735486_HOXC8	0.503128	2.97E-06	4.49E-05
11759631_TRIO	0.585933	2.98E-06	4.50E-05
11742886_BAG5	-0.43682	2.98E-06	4.50E-05
11740122_ZNF419	0.676767	2.99E-06	4.51E-05
11754422_CARS2	0.429384	2.99E-06	4.51E-05
11722529_PTPN21	0.643442	2.99E-06	4.51E-05
11726364_OASL	0.475096	2.99E-06	4.51E-05
11744462_PCID2	0.520007	2.99E-06	4.51E-05
11739227_FBXW11	-0.47123	3.00E-06	4.51E-05
11755652_MBLAC2	-0.89544	3.00E-06	4.51E-05
11755071_HMCN1	0.598543	3.00E-06	4.52E-05
11718755_MTMR10	0.559101	3.01E-06	4.53E-05
11759139_FAM98B	-0.4787	3.01E-06	4.53E-05
11756695_PCNXL4	0.526207	3.01E-06	4.53E-05
11764173_TMEM183A	-0.59488	3.01E-06	4.53E-05
11740714_SLC1A5	-0.44442	3.01E-06	4.53E-05
11722141_RELB	0.579995	3.02E-06	4.53E-05
11758933_TM4SF18	0.623965	3.02E-06	4.53E-05
11740485_ABL1	-0.58976	3.02E-06	4.54E-05
11734804_KIF20B	-0.75121	3.03E-06	4.55E-05
11755671_SYTL2	0.730189	3.03E-06	4.55E-05
11725193_TTPAL	-0.54569	3.03E-06	4.55E-05
11716304_ABHD14B	-0.48737	3.04E-06	4.56E-05
11746032_RALGPS2	0.505444	3.04E-06	4.56E-05
11754705_MRPL43	0.498023	3.05E-06	4.57E-05
11725220_SLK	0.515264	3.05E-06	4.57E-05
11722518_SMC5	0.774327	3.05E-06	4.57E-05

11744998_MPZL1	0.62504	3.06E-06	4.57E-05
11721721_MSH5 ///	-0.52027	3.06E-06	4.57E-05
11737577_LRRC7	1.055793	3.06E-06	4.58E-05
11721666_CDT1	-0.70598	3.06E-06	4.58E-05
11722442_NUF2	-0.74308	3.06E-06	4.58E-05
11723723_SLAIN2	0.484533	3.06E-06	4.58E-05
11731337_ZNF398	0.548505	3.07E-06	4.58E-05
11733822_AGPAT3	-0.41736	3.07E-06	4.58E-05
11717118_P4HA2	0.493045	3.07E-06	4.58E-05
11748773_CTSV	-0.88412	3.07E-06	4.58E-05
11763716_ARL6IP4	-0.61415	3.07E-06	4.58E-05
11742737_RAB9A	0.496223	3.07E-06	4.58E-05
11741095_CORO2A	-0.54482	3.07E-06	4.58E-05
11728978_CASP8	0.59219	3.07E-06	4.58E-05
11752335_ZNF12	0.474759	3.08E-06	4.59E-05
11727293_ALG10B	-1.40639	3.08E-06	4.59E-05
11739483_CCDC88A	-0.44405	3.08E-06	4.59E-05
11717282_ASAP1	0.474557	3.08E-06	4.59E-05
11754333_ACAD8	0.600233	3.09E-06	4.60E-05
11728510_FKTN	0.696303	3.09E-06	4.61E-05
11729495_FAM133A	-0.91444	3.10E-06	4.61E-05
11725514_SMAD7	0.698282	3.10E-06	4.61E-05
11732276_CCL5	0.694845	3.10E-06	4.61E-05
11749196_WDR46	-0.48058	3.10E-06	4.61E-05
11715900_COASY	-0.58787	3.11E-06	4.62E-05
11757283_DCN	0.853492	3.11E-06	4.62E-05
11718846_SH3GLB1	0.442663	3.11E-06	4.62E-05
11727353_CENPM	-0.55796	3.11E-06	4.62E-05
11759525_GADD45B	0.602636	3.11E-06	4.62E-05
11739711_PHACTR2	0.686769	3.11E-06	4.62E-05
11756705_NAA25	0.499315	3.12E-06	4.63E-05
11721216_TMEM106B	-0.90739	3.13E-06	4.64E-05
11747340_ABCB9	0.842452	3.13E-06	4.64E-05
11754058_SPARC	0.451704	3.13E-06	4.65E-05
11715359_TIMP1	0.440372	3.14E-06	4.65E-05
11729947_TRMT10A	-0.63326	3.14E-06	4.65E-05
11729261_ZNF644	0.511643	3.14E-06	4.65E-05
11726584_ARMC1	-0.49181	3.15E-06	4.66E-05
11738637_CTBP1	-0.49164	3.15E-06	4.66E-05
11724990_CLTCL1	0.712274	3.15E-06	4.66E-05
11756678_FAM64A	-0.54356	3.15E-06	4.66E-05
11739888_CEP85L	0.856324	3.15E-06	4.66E-05
11716865_SLC1A5	-0.46703	3.15E-06	4.66E-05
11756187_RPA2	-0.41608	3.16E-06	4.67E-05
11733352_NF2	0.478323	3.16E-06	4.67E-05
11735341_TANC1	0.418787	3.16E-06	4.67E-05
11741226_EYA1	-0.7813	3.16E-06	4.67E-05
11756159_SEC22C	-0.49921	3.16E-06	4.67E-05
11757056_LOC728026	-0.44051	3.17E-06	4.67E-05
11733080_POT1	-1.0756	3.17E-06	4.68E-05
11757614_OPA1	-0.51277	3.17E-06	4.68E-05

11727784_TPM4	-0.4263	3.17E-06	4.68E-05
11715984_ADNP	0.415565	3.18E-06	4.68E-05
11736413_NHLH2	1.042614	3.18E-06	4.68E-05
11739305_WRB	-0.55169	3.18E-06	4.68E-05
11754257_C15orf57	0.603097	3.18E-06	4.68E-05
11743054_CHORDC1	0.562621	3.19E-06	4.69E-05
11732395_NHLRC2	0.493615	3.19E-06	4.69E-05
11715605_SF3B5	-0.53947	3.20E-06	4.70E-05
11721283_CDS2	0.531722	3.20E-06	4.70E-05
11718258_ANKRD27	-0.43291	3.20E-06	4.70E-05
11735242_ZC3H12C	0.652649	3.21E-06	4.72E-05
11746114_ZNF35	0.886525	3.21E-06	4.72E-05
11756224_ZDHHC9	0.582177	3.22E-06	4.73E-05
11753582_PTTG3P	-0.46685	3.22E-06	4.73E-05
11749692_ACTN2	0.69406	3.22E-06	4.73E-05
11720972_TOP2A	-0.52626	3.22E-06	4.73E-05
11749493_GTF2H1	0.572691	3.23E-06	4.74E-05
11723116_RAD51L3-R	0.473211	3.23E-06	4.74E-05
11741470_SLTM	-0.54854	3.24E-06	4.75E-05
11719839_CASP3	0.520024	3.24E-06	4.76E-05
11746809_ZNF544	0.546884	3.25E-06	4.76E-05
11729633_YBX3	0.432715	3.25E-06	4.76E-05
11720124_SRD5A3	-0.50262	3.26E-06	4.77E-05
11743417_P3H2	1.048931	3.26E-06	4.78E-05
11741368_MTFR1L	-0.61421	3.26E-06	4.78E-05
11751245_CNN2	0.461982	3.26E-06	4.78E-05
11718980_YKT6	-0.63967	3.27E-06	4.79E-05
11750961_PAK4	-0.46919	3.28E-06	4.80E-05
11754654_KCTD2	-0.70507	3.29E-06	4.80E-05
11719836_LGALS8	0.472044	3.29E-06	4.80E-05
11763852_ELOVL4	0.661488	3.29E-06	4.80E-05
11758857_RAB2A	-0.70248	3.29E-06	4.80E-05
11733360_SLC14A1	0.821206	3.29E-06	4.80E-05
11716884_BANF1	-0.67577	3.29E-06	4.80E-05
11731992_TROAP	-0.72979	3.29E-06	4.80E-05
11754773_WWC2	0.518463	3.29E-06	4.80E-05
11748689_GNA13	0.475697	3.29E-06	4.80E-05
11716323_ICMT	-0.44375	3.30E-06	4.80E-05
11733969_SHMT1	-0.46364	3.30E-06	4.81E-05
11717628_BIRC2	0.477866	3.30E-06	4.81E-05
11734828_SPRED1	0.529846	3.30E-06	4.81E-05
11746042_TRA2B	-0.57862	3.30E-06	4.81E-05
11728274_PMP22	0.444816	3.31E-06	4.81E-05
11739134_COL5A2	0.527265	3.31E-06	4.81E-05
11734823_APBB2	0.743001	3.31E-06	4.81E-05
11733292_PHF20L1	0.49336	3.31E-06	4.82E-05
11721513_NDEL1	0.442958	3.31E-06	4.82E-05
11720066_GPHN	0.414655	3.31E-06	4.82E-05
11747662_PTGES3	-0.43145	3.32E-06	4.82E-05
11758140_CPSF6	-0.57959	3.32E-06	4.82E-05
11722180_DR1	0.460863	3.32E-06	4.82E-05

11722736_APPL1	-0.60469	3.32E-06	4.82E-05
11728958_E2F8	-0.92185	3.32E-06	4.82E-05
11715561_SCD	-0.46907	3.33E-06	4.83E-05
11741608_RRP12	0.577689	3.33E-06	4.83E-05
11727937_MSANTD3	0.601956	3.33E-06	4.83E-05
11732222_TVP23B //	0.446594	3.33E-06	4.83E-05
11739958_BCL2L1	0.456912	3.33E-06	4.83E-05
11743510_UFL1	-0.55989	3.35E-06	4.85E-05
11718125_AIMP1	-0.44345	3.35E-06	4.85E-05
11731594_STAM2	-0.56711	3.35E-06	4.85E-05
11737127_ITGA1	0.586146	3.35E-06	4.85E-05
11755900_EXT1	0.471878	3.35E-06	4.85E-05
11726606_MTF2	0.550671	3.36E-06	4.85E-05
11720541_LGALSL	0.625858	3.36E-06	4.86E-05
11747726_STXBP6	0.593665	3.36E-06	4.86E-05
11748950_BAZ2B	0.60053	3.37E-06	4.86E-05
11745822_SRSF6	0.479825	3.37E-06	4.87E-05
11753283_HAUS7 ///	-0.64586	3.37E-06	4.87E-05
11729316_ISOC2	-0.5799	3.38E-06	4.88E-05
11756505_USP32 ///	0.735412	3.38E-06	4.88E-05
11732091_HMG20A	-0.45264	3.38E-06	4.88E-05
11724482_SHOC2	0.441741	3.38E-06	4.88E-05
11725765_COL11A1	0.926612	3.39E-06	4.89E-05
11717764_MGLL	0.486947	3.39E-06	4.89E-05
11751474_ALCAM	0.524359	3.39E-06	4.89E-05
11763385_RPL7L1	-0.44975	3.39E-06	4.89E-05
11757117_RPS2	-0.60243	3.40E-06	4.90E-05
11739763_GINS3	-0.62243	3.40E-06	4.90E-05
11739975_ZNF117	0.759721	3.40E-06	4.90E-05
11763332_VPS36	0.66302	3.40E-06	4.90E-05
11736053_EEF1A1	-0.56557	3.40E-06	4.90E-05
11754911_NEXN	0.826532	3.41E-06	4.90E-05
11732902_APOBEC3B	-0.54639	3.41E-06	4.91E-05
11717272_COL5A1	0.663781	3.42E-06	4.92E-05
11748543_TXNIP	-0.4301	3.42E-06	4.92E-05
11744398_LIG1	-0.46247	3.43E-06	4.92E-05
11753819_MDM2	0.775	3.43E-06	4.93E-05
11747606_NCAPD2	-0.66836	3.43E-06	4.93E-05
11726634_KAT6A	-0.59928	3.43E-06	4.93E-05
11724605_HS6ST2	-0.53784	3.44E-06	4.93E-05
11755753_TENM2	0.518602	3.44E-06	4.93E-05
11737480_PON2	0.494487	3.44E-06	4.93E-05
11752280_RBM5	0.606021	3.44E-06	4.93E-05
11728555_ATP6V1E2	-0.59806	3.44E-06	4.93E-05
11755151_ARNTL	0.750012	3.44E-06	4.93E-05
11723201_HNRNPA3	-0.83085	3.45E-06	4.94E-05
11723299_RPS6KB1	0.469711	3.45E-06	4.95E-05
11757755_ZNF3	0.48698	3.45E-06	4.95E-05
11727155_TRIOBP	0.425772	3.46E-06	4.95E-05
11729199_C6orf120	0.736973	3.46E-06	4.95E-05
11726130_PKP2	0.635243	3.46E-06	4.95E-05

11757285_DCTN5	0.624051	3.46E-06	4.95E-05
11733313_PCDHA1 //	0.613115	3.47E-06	4.96E-05
11756742_AGPAT5	0.533403	3.47E-06	4.96E-05
11731722_ALDH3B1	0.582835	3.49E-06	4.98E-05
11752036_TMEM123	-0.48597	3.49E-06	4.98E-05
11757978_FAM103A1	0.486665	3.49E-06	4.98E-05
11727199_PCDHA1 //	0.694576	3.49E-06	4.99E-05
11741054_LAMP2	-0.47336	3.49E-06	4.99E-05
11716874_WDR46	-0.52148	3.49E-06	4.99E-05
11739410_KMT2C	0.477735	3.50E-06	4.99E-05
11740746_OPA1	-0.57646	3.51E-06	5.00E-05
11726670_DCLRE1B	-0.73733	3.51E-06	5.00E-05
11721063_ARFGEF2	0.734115	3.51E-06	5.01E-05
11755092_GPR180	0.488115	3.52E-06	5.01E-05
11756347_PRR11	-0.59498	3.52E-06	5.01E-05
11726853_TPTE	0.675993	3.52E-06	5.01E-05
11716977_NSFL1C	-0.61363	3.53E-06	5.03E-05
11759300_AKAP10	0.557682	3.54E-06	5.03E-05
11728384_SNAPC4	0.455885	3.54E-06	5.03E-05
11725486_CCDC77	-0.47252	3.55E-06	5.04E-05
11754154_CYP1A1	0.846997	3.55E-06	5.04E-05
11718222_GSPT1	-0.7218	3.55E-06	5.05E-05
11749306_CCNG1	0.629058	3.55E-06	5.05E-05
11754047_RDM1	-1.05842	3.55E-06	5.05E-05
11743483_RNASEH2B	-0.52516	3.56E-06	5.05E-05
11746799_HACD3	-0.40308	3.56E-06	5.06E-05
11747335_CEP70	-0.60842	3.57E-06	5.06E-05
11725412_TRIM14	-0.47	3.58E-06	5.08E-05
11731649_NTM	0.591557	3.58E-06	5.08E-05
11739182_MTRF1L	0.4761	3.58E-06	5.08E-05
11724448_DNMBP	0.526739	3.59E-06	5.08E-05
11739303_DLC1	0.465692	3.59E-06	5.09E-05
11722095_KIF21B	-0.56082	3.59E-06	5.09E-05
11720409_CYP1B1	0.705378	3.60E-06	5.09E-05
11726885_C15orf41	-0.55437	3.60E-06	5.10E-05
11728600_FAM24B	0.666315	3.61E-06	5.10E-05
11715360_TIMP1	0.437305	3.61E-06	5.11E-05
11757889_RPL15	-0.46339	3.61E-06	5.11E-05
11736327_ZNF670	0.541002	3.61E-06	5.11E-05
11721509_LOC105370	-0.43518	3.61E-06	5.11E-05
11733568_ZNF433	0.509697	3.62E-06	5.11E-05
11726595_DUT	-0.47936	3.62E-06	5.12E-05
11751321_CRIM1	0.602242	3.63E-06	5.13E-05
11724818_SH3KBP1	-0.73102	3.63E-06	5.13E-05
11724820_NEK6	0.601018	3.63E-06	5.13E-05
11755434_PRKACB	0.753511	3.64E-06	5.13E-05
11745109_TMTC1	0.842275	3.64E-06	5.14E-05
11754059_SPARC	0.486836	3.65E-06	5.16E-05
11716642_DTYMK	-0.56919	3.66E-06	5.16E-05
11723799_CSTF3	-0.55902	3.66E-06	5.16E-05
11734957_PCNXL4	0.55889	3.66E-06	5.16E-05

11743326_CCDC50	-0.65759	3.66E-06	5.16E-05
11718325_SESN2	0.559983	3.66E-06	5.16E-05
11717627_BIRC2	0.418345	3.67E-06	5.17E-05
11748274_FDFT1	-0.59567	3.67E-06	5.17E-05
11721538_COL4A3BP	-0.54521	3.68E-06	5.18E-05
11726514_CLK4	0.836092	3.69E-06	5.19E-05
11733383_AFAP1	-0.49129	3.69E-06	5.19E-05
11717109_PCM1	-0.44489	3.69E-06	5.19E-05
11748248_CKAP2	-0.50573	3.69E-06	5.19E-05
11734841_RAD51	-0.46619	3.70E-06	5.20E-05
11749096_NEMF	0.508304	3.70E-06	5.20E-05
11747645_ADAM17	0.482897	3.71E-06	5.21E-05
11736192_RRM2B	0.498675	3.71E-06	5.21E-05
11716301_ITGAV	0.535429	3.71E-06	5.22E-05
11724096_PDE4B	0.484318	3.73E-06	5.23E-05
11749530_RALBP1	-0.58324	3.73E-06	5.23E-05
11722366_HIRIP3	-0.4637	3.73E-06	5.24E-05
11715519_MLEC	-0.54611	3.74E-06	5.24E-05
11724562_RHOF	-0.63527	3.74E-06	5.24E-05
11752155_KDM4C	0.428321	3.74E-06	5.24E-05
11757290_COL5A2	0.41665	3.74E-06	5.24E-05
11720535_MMAB	-0.53713	3.75E-06	5.26E-05
11729354_ZNF200	0.551405	3.75E-06	5.26E-05
11735238_SEC22C	0.586999	3.75E-06	5.26E-05
11731891_ZNF658 //	0.800808	3.76E-06	5.26E-05
11727484_TPR	-0.46796	3.77E-06	5.27E-05
11745992_ATG5	0.435225	3.77E-06	5.27E-05
11722806_RAB35	-0.48528	3.77E-06	5.27E-05
11749895_BCAT1	0.475202	3.77E-06	5.27E-05
11736706_HPS5	0.661822	3.77E-06	5.27E-05
11738147_LRIG3	0.455827	3.78E-06	5.28E-05
11747518_TLR4	0.883995	3.78E-06	5.28E-05
11745978_SMG1 ///	0.694421	3.78E-06	5.28E-05
11764248_LDLRAD3	-0.45409	3.78E-06	5.28E-05
11731517_MFAP3	0.448706	3.79E-06	5.28E-05
11718126_AIMP1	-0.45052	3.79E-06	5.29E-05
11737174_HMMR	-0.48014	3.79E-06	5.29E-05
11731031_ZNF8	0.427351	3.79E-06	5.29E-05
11723358_SCAF11	-0.42025	3.79E-06	5.29E-05
11725409_NAV1	-0.56268	3.80E-06	5.29E-05
11718962_NUFIP2	0.457726	3.80E-06	5.29E-05
11757829_HACD3	-0.4317	3.80E-06	5.29E-05
11726678_AFAP1L2	0.408271	3.80E-06	5.29E-05
11720182_SMG8	-0.50174	3.80E-06	5.29E-05
11747734_KIAA0895	0.791005	3.80E-06	5.29E-05
11747762_MAGOH	-0.49121	3.81E-06	5.30E-05
11722846_UBE2R2	-0.48443	3.83E-06	5.32E-05
11733103_POLD3	-0.53333	3.83E-06	5.33E-05
11756373_UMPS	-0.45772	3.83E-06	5.33E-05
11726378_WHSC1	-0.57877	3.83E-06	5.33E-05
11743311_PILRB ///	0.582987	3.84E-06	5.33E-05

11733932_TMEM68	-0.70241	3.84E-06	5.33E-05
11744591_TTC8	0.528068	3.84E-06	5.33E-05
11719319_GOLGA4	0.493493	3.84E-06	5.33E-05
11724238_CYB5R4	0.479126	3.84E-06	5.33E-05
11719168_MOAP1	0.488415	3.84E-06	5.33E-05
11717038_RNF13	0.503814	3.85E-06	5.34E-05
11719418_ANKRA2	0.503309	3.85E-06	5.34E-05
11749776_TFAP2C	-0.45423	3.85E-06	5.34E-05
11733259_TAOK3	0.487554	3.87E-06	5.36E-05
11723597_MAP2K7	-0.54762	3.87E-06	5.36E-05
11715463_IGFBP4	-0.68081	3.88E-06	5.38E-05
11719775_C5orf24	0.76301	3.88E-06	5.38E-05
11755395_REST	0.757103	3.88E-06	5.38E-05
11741816_TAB3	0.459629	3.89E-06	5.38E-05
11747688_ZNF623	0.495984	3.89E-06	5.38E-05
11753214_PIBF1	0.497394	3.89E-06	5.38E-05
11726292_WDR44	0.408267	3.89E-06	5.38E-05
11756796_UBXN7	0.536782	3.89E-06	5.38E-05
11746320_SERPINH1	-0.44574	3.89E-06	5.38E-05
11748519_SEC22C	-0.46744	3.90E-06	5.38E-05
11717718_FNDC3B	0.453182	3.90E-06	5.38E-05
11736810_RPL27A	0.662123	3.90E-06	5.38E-05
11728284_RBMS2	0.59572	3.90E-06	5.38E-05
11721837_MYLK	0.50379	3.91E-06	5.40E-05
11725882_RNFT2	-0.56378	3.92E-06	5.40E-05
11716393_ADH5 ///	-0.52858	3.92E-06	5.41E-05
11734912_ZNF443 //	0.642159	3.92E-06	5.41E-05
11733929_ARMC8	0.526436	3.92E-06	5.41E-05
11715806_SEPW1	0.425431	3.93E-06	5.41E-05
11754456_GOLGB1	0.484498	3.94E-06	5.43E-05
11752101_EIF2S1	-0.52786	3.94E-06	5.43E-05
11736612_RIC1	0.58306	3.95E-06	5.43E-05
11758089_HMMR	-0.48335	3.95E-06	5.43E-05
11731246_SERTAD4	0.476101	3.95E-06	5.43E-05
11727796_NDFIP2	0.596533	3.95E-06	5.43E-05
11719763_VPS13C	0.490411	3.96E-06	5.44E-05
11735614_EGR4	0.972983	3.97E-06	5.46E-05
11752207_PRDM1	0.975416	3.97E-06	5.46E-05
11733874_SMC4	-0.50844	3.97E-06	5.46E-05
11722019_CEP85	-0.56748	3.97E-06	5.46E-05
11717271_PRAME	-0.45995	3.98E-06	5.46E-05
11743926_SEC24A	0.50673	3.98E-06	5.46E-05
11730022_PILRB ///	0.48908	3.99E-06	5.48E-05
11739002_HDGF	-0.44754	4.00E-06	5.48E-05
11748264_DCAF17	1.010361	4.00E-06	5.48E-05
11725232_ATP8B2	-0.46822	4.00E-06	5.49E-05
11756601_FAM8A1	0.648016	4.01E-06	5.50E-05
11759063_SIK2	-0.50196	4.02E-06	5.50E-05
11717553_DERL2	0.409519	4.02E-06	5.50E-05
11716626_KIF3B	0.480881	4.02E-06	5.51E-05
11721339_NCOA1	0.459582	4.02E-06	5.51E-05

11729642_TPD52	-0.88459	4.03E-06	5.52E-05
11753965_MSL3P1	-1.1004	4.04E-06	5.52E-05
11730331_BRI3BP	-0.54353	4.04E-06	5.52E-05
11764036_KPNB1	-0.53841	4.04E-06	5.52E-05
11747114_PLK4	-0.57762	4.04E-06	5.53E-05
11754089_NKIRAS2	0.433582	4.04E-06	5.53E-05
11743923_TCFL5	-0.48178	4.04E-06	5.53E-05
11721205_ANK3	0.554869	4.05E-06	5.53E-05
11723783_AGPS	0.478336	4.05E-06	5.53E-05
11721206_ANK3	0.451119	4.05E-06	5.53E-05
11751724_ZNF542P	0.592533	4.05E-06	5.53E-05
11752781_TANK	0.581525	4.05E-06	5.53E-05
11724171_FCHO2	0.555457	4.06E-06	5.53E-05
11723560_SMCO4	-0.52939	4.06E-06	5.53E-05
11719359_ALDH5A1	-0.96678	4.06E-06	5.54E-05
11742219_CD46	0.477522	4.06E-06	5.54E-05
11757625_CD200	-0.75606	4.06E-06	5.54E-05
11755049_PIK3C3	0.441623	4.07E-06	5.54E-05
11721641_CAP2	0.699937	4.07E-06	5.54E-05
11749319_PGAP1	0.520185	4.07E-06	5.54E-05
11751097_SNX10	-0.4034	4.08E-06	5.55E-05
11715746_MINOS1-NB	-0.46554	4.09E-06	5.57E-05
11722094_PPIC	0.462674	4.09E-06	5.57E-05
11756763_HINT2	-0.54125	4.10E-06	5.57E-05
11758551_ZNF264	0.529286	4.11E-06	5.59E-05
11747967_SNX13	0.468324	4.12E-06	5.60E-05
11758344_SDCBP	0.402298	4.12E-06	5.60E-05
11732249_RCOR3	0.503909	4.12E-06	5.60E-05
11732294_ZNF566	0.573632	4.13E-06	5.61E-05
11729738_RCCD1	-0.61668	4.13E-06	5.61E-05
11743882_STRN3	0.496788	4.13E-06	5.61E-05
11725885_CNEP1R1	0.596834	4.14E-06	5.61E-05
11733919_SERTAD2	0.447917	4.15E-06	5.63E-05
11720410_CYP1B1	0.714386	4.15E-06	5.63E-05
11717426_CHMP3 ///	-0.53365	4.15E-06	5.63E-05
11729158_UBE2T	-0.55261	4.15E-06	5.63E-05
11731987_DNAJC9	-0.59679	4.16E-06	5.63E-05
11754588_RBM5	0.557887	4.16E-06	5.63E-05
11739809_TMEM156	1.496244	4.16E-06	5.64E-05
11758514_SERBP1	-0.66806	4.16E-06	5.64E-05
11757029_NDUFA9	-0.55945	4.17E-06	5.64E-05
11746533_KIAA1217	0.426438	4.17E-06	5.65E-05
11720103_PAK1	-0.4279	4.17E-06	5.65E-05
11716392_ADH5	-0.63085	4.19E-06	5.66E-05
11733664_ARHGAP11A	-0.50283	4.19E-06	5.67E-05
11717706_VPS13D	-0.75965	4.19E-06	5.67E-05
11746843_ABI2	0.503936	4.20E-06	5.67E-05
11720000_SYNJ2BP	-0.5716	4.20E-06	5.67E-05
11718481_CFI	0.858805	4.20E-06	5.67E-05
11753415_ARL6IP4	-0.5636	4.20E-06	5.68E-05
11729305_ATAD1	0.415538	4.20E-06	5.68E-05

11722735_APPL1	-0.44464	4.21E-06	5.68E-05
11756160_CTNNAL1	0.442589	4.21E-06	5.68E-05
11760295_EBNA1BP2	0.72319	4.21E-06	5.68E-05
11722918_KCNS3	-0.78245	4.21E-06	5.68E-05
11725798_USP46	0.556544	4.22E-06	5.69E-05
11750010_WHSC1	-0.52727	4.22E-06	5.69E-05
11716639_COL4A1	0.750099	4.22E-06	5.69E-05
11741269_CCDC77	-0.45897	4.23E-06	5.69E-05
11720841_SMNDC1	0.402141	4.23E-06	5.69E-05
11715994_ID2	0.461059	4.23E-06	5.69E-05
11756490_BCLAF1	-0.39137	4.23E-06	5.69E-05
11739783_CDON	-0.44998	4.23E-06	5.70E-05
11739837_KDM5A	0.397797	4.24E-06	5.71E-05
11743546_MYO1B	0.440558	4.24E-06	5.71E-05
11729353_LNPEP	0.7617	4.25E-06	5.71E-05
11728738_TBC1D20	0.40695	4.25E-06	5.71E-05
11758590_CYBRD1	0.434944	4.25E-06	5.71E-05
11718787_NRGN	-0.46338	4.26E-06	5.72E-05
11723916_EIF2S2	-0.55873	4.26E-06	5.72E-05
11738844_NTM	0.733571	4.26E-06	5.72E-05
11757810_TMED10	-0.42561	4.27E-06	5.73E-05
11756625_FAM156A /	-0.39825	4.28E-06	5.74E-05
11755364_MAMLD1	-0.5927	4.28E-06	5.74E-05
11744533_MTRF1L	0.436344	4.29E-06	5.76E-05
11745911_RUFY2	0.766129	4.31E-06	5.78E-05
11758213_AKIRIN1	0.408923	4.32E-06	5.79E-05
11721797_SUMO3	-0.58486	4.33E-06	5.80E-05
11757563_PPP1R35	-0.4744	4.33E-06	5.81E-05
11750612_ZNF112	0.947539	4.34E-06	5.81E-05
11739004_HDGF	-0.59687	4.35E-06	5.82E-05
11722326_ZBTB34	0.755692	4.35E-06	5.83E-05
11725374_HAPLN1	0.583509	4.36E-06	5.84E-05
11742910_ANKRD50	0.437179	4.37E-06	5.84E-05
11736044_MYH10	-0.4534	4.37E-06	5.84E-05
11721883_TUBGCP3	-0.54988	4.37E-06	5.84E-05
11752334_ZNF12	0.458009	4.37E-06	5.84E-05
11728348_RPL39L	-0.54094	4.37E-06	5.84E-05
11723747_FAM64A	-0.67979	4.37E-06	5.84E-05
11719700_FARP1	-0.55186	4.38E-06	5.85E-05
11751164_CPPED1	0.534292	4.38E-06	5.85E-05
11718728_ZNF655	0.505631	4.39E-06	5.87E-05
11749441_WDR41	0.428474	4.40E-06	5.87E-05
11729231_KLF3	0.432053	4.40E-06	5.87E-05
11746215_NAP1L4	-0.43932	4.40E-06	5.87E-05
11741309_STK26	-0.52395	4.40E-06	5.87E-05
11724740_HMGN2	-0.42147	4.40E-06	5.87E-05
11736465_ZNF773	0.781375	4.41E-06	5.87E-05
11749192_UBE2L3	-0.6381	4.41E-06	5.87E-05
11721672_NXT1	-0.47391	4.41E-06	5.88E-05
11748255_RBL2	-0.57755	4.41E-06	5.88E-05
11743680_PTCH1	-0.51815	4.42E-06	5.88E-05

11737559_AJAP1	0.574126	4.42E-06	5.88E-05
11741661_ARMC8	0.608599	4.42E-06	5.88E-05
11717266_CAMK2G	-0.61291	4.43E-06	5.89E-05
11755777_RECK	0.628488	4.43E-06	5.89E-05
11719701_FARP1	-0.53153	4.44E-06	5.90E-05
11754004_ATP6V1G1	-0.48753	4.44E-06	5.90E-05
11733492_SLC44A5	0.776759	4.44E-06	5.90E-05
11731625_CCDC186	0.524222	4.45E-06	5.91E-05
11727438_RALGAPB	-0.40203	4.45E-06	5.91E-05
11716595_SLC44A1	-0.49531	4.46E-06	5.92E-05
11733875_SMC4	-0.61697	4.46E-06	5.92E-05
11716866_SLC1A5	-0.47483	4.46E-06	5.92E-05
11755756_NHSL1	-0.47156	4.46E-06	5.92E-05
11720537_MMAB	-0.55147	4.48E-06	5.94E-05
11728954_KITLG	0.864992	4.48E-06	5.94E-05
11723561_SMCO4	-0.87466	4.48E-06	5.94E-05
11727262_ENTPD7	0.653275	4.48E-06	5.94E-05
11734002_ACTN2	0.771496	4.48E-06	5.94E-05
11746771_CCDC130	0.59656	4.49E-06	5.95E-05
11719838_CASP3	0.462825	4.50E-06	5.95E-05
11719362_SLF2	-0.51458	4.50E-06	5.95E-05
11746195_LOC100996	0.42464	4.50E-06	5.96E-05
11719331_KIAA0907	0.489797	4.52E-06	5.98E-05
11746005_SPACA6	0.568138	4.52E-06	5.98E-05
11759191_NIPAL2	0.45479	4.52E-06	5.98E-05
11753782_TANK	0.503456	4.52E-06	5.98E-05
11745233_ZNF582	0.920623	4.53E-06	5.99E-05
11728167_ZNF264	0.572133	4.54E-06	5.99E-05
11718475_IDH1	-0.4631	4.54E-06	5.99E-05
11744916_CAST	0.440516	4.54E-06	5.99E-05
11724429_ZNF337	0.664831	4.54E-06	5.99E-05
11718514_TSPAN14	0.507183	4.54E-06	6.00E-05
11741037_GPR89A //	0.462959	4.55E-06	6.00E-05
11745203_RMI1	-0.65702	4.55E-06	6.00E-05
11717656_SPRED2	0.423693	4.55E-06	6.00E-05
11717289_YTHDF3	0.446707	4.55E-06	6.00E-05
11720508_FBN1	0.641087	4.55E-06	6.00E-05
11716873_WDR46	-0.52616	4.55E-06	6.00E-05
11744131_SECISBP2L	0.650569	4.55E-06	6.00E-05
11741960_ADAM9	0.426956	4.56E-06	6.00E-05
11727848_GNA12	-0.52076	4.56E-06	6.00E-05
11724100_MAP3K6	-0.6768	4.56E-06	6.00E-05
11748128_SLC25A32	0.450994	4.56E-06	6.00E-05
11716833_TM7SF3	0.439211	4.56E-06	6.00E-05
11719835_LGALS8	0.465035	4.57E-06	6.01E-05
11754245_SDCBP	0.409872	4.57E-06	6.01E-05
11720107_KIF2A	0.488577	4.57E-06	6.01E-05
11727869_GALK2	0.449978	4.57E-06	6.01E-05
11755489_EXOC7	-0.70734	4.57E-06	6.01E-05
11723243_CDK6	0.429618	4.57E-06	6.01E-05
11719316_PIBF1	0.480775	4.58E-06	6.01E-05

11716195_ID1	-0.4941	4.58E-06	6.01E-05
11728867_PCK2	-0.67327	4.58E-06	6.01E-05
11731548_TMEM67	0.699362	4.58E-06	6.01E-05
11758085_CEP44	-0.68979	4.59E-06	6.02E-05
11722500_LYSMD3	0.456377	4.60E-06	6.03E-05
11739456_SGMS1	0.413741	4.61E-06	6.04E-05
11722324_ZNF84	0.566236	4.61E-06	6.04E-05
11716348_EFCAB14	0.615458	4.61E-06	6.04E-05
11756784_NCAPG2	-0.56374	4.61E-06	6.04E-05
11727969_SLC14A1	0.698726	4.61E-06	6.04E-05
11754186_ST7L	0.951595	4.62E-06	6.04E-05
11751517_PRKAA1	0.673653	4.62E-06	6.05E-05
11730290_ARHGAP5	-0.58994	4.62E-06	6.05E-05
11722584_CAST	0.507713	4.63E-06	6.05E-05
11740518_LGALS8	0.434733	4.64E-06	6.07E-05
11734706_NMD3	0.481098	4.64E-06	6.07E-05
11759882_EPS15L1	0.70042	4.65E-06	6.07E-05
11723123_SH3PXD2B	-0.76336	4.65E-06	6.07E-05
11718824_IGIP	-0.82608	4.65E-06	6.08E-05
11722175_SF3B3	-0.53025	4.66E-06	6.08E-05
11717763_MGLL	0.496058	4.67E-06	6.09E-05
11725902_TMEM135	0.470437	4.67E-06	6.09E-05
11749449_HNRNPH2 /	-0.51624	4.67E-06	6.10E-05
11720756_STAT3	0.575236	4.67E-06	6.10E-05
11726002_KRCC1	0.593672	4.68E-06	6.10E-05
11756868_DLX1	-0.56659	4.68E-06	6.10E-05
11731549_TMEM67	0.680039	4.68E-06	6.10E-05
11720771_PPIF	0.582023	4.68E-06	6.10E-05
11763731_VPS36	0.520828	4.69E-06	6.11E-05
11747814_IDH2	-0.61934	4.70E-06	6.12E-05
11715844_PTPRF	-0.56086	4.71E-06	6.13E-05
11733079_POT1	-0.43888	4.71E-06	6.13E-05
11720669_KAT2B	0.548252	4.71E-06	6.13E-05
11718135_PRPS2	-0.39925	4.71E-06	6.13E-05
11726768_SMPX	0.604172	4.73E-06	6.16E-05
11755735_GTPBP6	-0.4729	4.75E-06	6.17E-05
11718927_ARID5B	0.542531	4.75E-06	6.17E-05
11743699_EDEM3	0.426299	4.75E-06	6.17E-05
11761864_TMOD3	0.473504	4.75E-06	6.17E-05
11732977_GON4L	0.466351	4.75E-06	6.17E-05
11759491_MRPL35	-0.56521	4.75E-06	6.17E-05
11723733_OTUD6B	0.446419	4.76E-06	6.18E-05
11751224_MAPK1	0.416452	4.76E-06	6.18E-05
11719960_CTBP1	-0.49251	4.77E-06	6.19E-05
11730394_ZNF552	0.939967	4.77E-06	6.19E-05
11727132_CPD	0.535433	4.78E-06	6.20E-05
11752694_PRR11	-0.55671	4.80E-06	6.22E-05
11722325_ZNF84	-0.47641	4.80E-06	6.22E-05
11757316_FLOT1	-0.47442	4.80E-06	6.22E-05
11756393_CORO1A	-0.6066	4.80E-06	6.22E-05
11740049_ZNF461	0.856489	4.80E-06	6.22E-05

11744032_NACC2	0.787757	4.81E-06	6.23E-05
11730840_ZMYND8	-0.54525	4.82E-06	6.23E-05
11744301_TTC17	-0.52948	4.82E-06	6.23E-05
11735178_C4orf33	-0.47415	4.82E-06	6.23E-05
11718177_CMTM4	-0.52659	4.82E-06	6.24E-05
11716186_ALCAM	0.426723	4.82E-06	6.24E-05
11745018_WLS	-0.42282	4.82E-06	6.24E-05
11755857_RNPS1	-0.41223	4.83E-06	6.24E-05
11756717_UBE2K	0.440647	4.83E-06	6.24E-05
11725462_MAU2	-0.46057	4.83E-06	6.25E-05
11741501_FAM133B /	0.49223	4.84E-06	6.25E-05
11719769_RNF111	0.560381	4.85E-06	6.26E-05
11752999_GALNT2	0.514558	4.86E-06	6.27E-05
11724306_EAF1	0.788652	4.86E-06	6.27E-05
11763977_SNRPF	-0.41076	4.86E-06	6.27E-05
11734684_UMPS	-0.49016	4.87E-06	6.28E-05
11756282_MAZ	-0.45196	4.87E-06	6.29E-05
11729765_ZNF331	-0.39951	4.88E-06	6.30E-05
11726635_KAT6A	-0.43677	4.89E-06	6.30E-05
11754375_SNX10	-0.49539	4.90E-06	6.31E-05
11723442_EPHA4	0.770356	4.90E-06	6.31E-05
11738644_STAMBPL1	0.602185	4.90E-06	6.32E-05
11750960_AASS	0.704204	4.91E-06	6.32E-05
11754377_ASB13	-0.47054	4.92E-06	6.33E-05
11739391_ATE1	0.479924	4.92E-06	6.33E-05
11754497_VIPAS39	0.460636	4.92E-06	6.33E-05
11715939_KHDRBS1	-0.61305	4.92E-06	6.33E-05
11743047_FLOT1	-0.5547	4.92E-06	6.33E-05
11744581_KHDRBS3	0.532374	4.93E-06	6.33E-05
11748947_UBTF	-0.44338	4.93E-06	6.33E-05
11758490_SPG20	0.438294	4.93E-06	6.34E-05
11715586_MAPRE1	0.468757	4.94E-06	6.35E-05
11718280_TRIAP1	0.445043	4.95E-06	6.35E-05
11716196_ID1	-0.50326	4.95E-06	6.36E-05
11736026_ADAM10	0.557595	4.96E-06	6.36E-05
11726005_PDE4B	0.797279	4.97E-06	6.37E-05
11757351_TAP1	0.515423	4.97E-06	6.37E-05
11743886_ATXN1L	-0.47231	4.98E-06	6.38E-05
11745143_SYNE2	-0.55801	4.98E-06	6.39E-05
11722352_ACTA2	0.430677	4.99E-06	6.40E-05
11736501_SS18	-0.52725	5.00E-06	6.40E-05
11748403_CNN2	0.586698	5.00E-06	6.40E-05
11758610_HDLBP	-0.70688	5.00E-06	6.40E-05
11736710_DENND4A	0.462095	5.01E-06	6.41E-05
11737647_CRACR2A	-0.66507	5.01E-06	6.41E-05
11725636_MOB1A	0.407172	5.01E-06	6.41E-05
11754092_ATG4B	-0.486	5.01E-06	6.41E-05
11758446_TSC22D1	0.434995	5.02E-06	6.43E-05
11722628_KIAA1033	0.476121	5.03E-06	6.43E-05
11733032_RBM18	0.519306	5.03E-06	6.43E-05
11759548_SREBF2	-0.60418	5.03E-06	6.43E-05

200096_PMATP6V0E1	0.461736	5.03E-06	6.43E-05
11740499_CBX5	0.461853	5.03E-06	6.43E-05
11720595_CD46	0.424445	5.04E-06	6.43E-05
11741673_TMEM161B	0.465172	5.04E-06	6.44E-05
11748603_CDKN1B	0.467365	5.05E-06	6.45E-05
11754363_CARM1	-0.4108	5.07E-06	6.47E-05
11715388_CDKN1A	0.441157	5.08E-06	6.48E-05
11733446_MMS22L	-0.72237	5.08E-06	6.48E-05
11748675_MCM5	-0.52138	5.09E-06	6.49E-05
11718538_FAHD2A	-0.45655	5.10E-06	6.50E-05
11730901_MBOAT2	-0.4999	5.10E-06	6.50E-05
11724483_SHOC2	0.450601	5.11E-06	6.52E-05
11755715_CD46	0.444789	5.12E-06	6.52E-05
11716214_PRPF3	0.569777	5.12E-06	6.52E-05
11753366_PTGES3	-0.44969	5.13E-06	6.52E-05
11743100_ENO2	-0.54506	5.13E-06	6.53E-05
11743742_ATP11A	-0.48362	5.13E-06	6.53E-05
11755252_AMIGO2	0.455852	5.13E-06	6.53E-05
11731110_ADAM17	0.571315	5.13E-06	6.53E-05
11758777_RBM8A	-0.70192	5.16E-06	6.56E-05
11721243_LAP3	-0.3887	5.16E-06	6.56E-05
11716012_TNFAIP1	-0.46825	5.16E-06	6.56E-05
11756053_LPP	0.735727	5.17E-06	6.57E-05
11752748_MRS2	0.58225	5.18E-06	6.57E-05
11718099_IFT20	0.453025	5.18E-06	6.57E-05
11719089_PCK2	-0.4792	5.18E-06	6.57E-05
11736761_CARD16	0.867789	5.18E-06	6.58E-05
11740248_SPAG1	0.627795	5.20E-06	6.59E-05
11725841_ZSCAN16	0.632617	5.20E-06	6.60E-05
11725521_GLUL	-0.48647	5.21E-06	6.60E-05
11737169_STRN3	0.5257	5.21E-06	6.61E-05
11725813_MAST3	-0.60919	5.21E-06	6.61E-05
11752327_ZBTB18	0.609024	5.23E-06	6.63E-05
11752237_SENP5	0.635176	5.24E-06	6.64E-05
11729676_CDK17	0.406214	5.24E-06	6.64E-05
11715710_CTSC	-0.56166	5.24E-06	6.64E-05
11723777_BRD3	-0.55976	5.24E-06	6.64E-05
11740587_ADNP	0.415261	5.25E-06	6.64E-05
11716622_CDK2AP2	-0.4596	5.25E-06	6.64E-05
11729792_MAN1A2	0.710467	5.25E-06	6.64E-05
11758572_HELLS	-0.43376	5.25E-06	6.64E-05
11739802_VCPIP1	0.542006	5.26E-06	6.64E-05
11733059_PTBP3	0.711647	5.26E-06	6.65E-05
11756005_COMMD4	-0.44166	5.26E-06	6.65E-05
11743472_ZNF226	0.512325	5.27E-06	6.65E-05
11746886_RAD52	0.4452	5.27E-06	6.65E-05
11734362_NFX1	0.534334	5.27E-06	6.65E-05
11720599_SUB1	-0.71483	5.27E-06	6.66E-05
11719284_UBR7	-0.56007	5.28E-06	6.67E-05
11743339_MFSD11	0.534022	5.29E-06	6.67E-05
11735576_LETM2	0.760564	5.30E-06	6.68E-05

11724170_FCHO2	0.657625	5.31E-06	6.70E-05
11749469_PPP4R3A	0.436089	5.31E-06	6.70E-05
11729238_CSRP2	-0.63732	5.32E-06	6.70E-05
11733078_PPP1R12A	0.535677	5.32E-06	6.70E-05
11717801_ATF5	-0.57626	5.32E-06	6.70E-05
11721948_RAB3GAP2	0.484182	5.32E-06	6.70E-05
11748393_SLC37A3	0.620971	5.33E-06	6.71E-05
11758170_DIAPH2	1.060706	5.33E-06	6.71E-05
11715704_ITGA5	0.546566	5.33E-06	6.71E-05
11726455_MYO5B	1.116445	5.34E-06	6.72E-05
11750681_CREBRF	0.722358	5.34E-06	6.72E-05
11757473_NSUN5	-0.54437	5.34E-06	6.72E-05
11752384_COL6A1	0.418601	5.35E-06	6.73E-05
11716759_CASP4	0.68112	5.37E-06	6.75E-05
11748960_DDX3X	0.445381	5.38E-06	6.76E-05
11725748_MXD3	-0.53683	5.38E-06	6.76E-05
11727180_PHTF1	0.460721	5.38E-06	6.76E-05
11716120_WDR34	-0.452	5.38E-06	6.76E-05
11745202_RMI1	-0.69832	5.39E-06	6.76E-05
11729614_H2AFZ	-0.39918	5.39E-06	6.76E-05
11755910_UBXN7	0.477754	5.40E-06	6.77E-05
11755245_C3orf17	0.498776	5.41E-06	6.78E-05
11743024_ATP7A	0.54285	5.41E-06	6.78E-05
11753336_NIF3L1	-0.49686	5.41E-06	6.78E-05
11724488_AKR1B10	0.538307	5.42E-06	6.79E-05
11742239_DEPDC1B	-0.5045	5.42E-06	6.79E-05
11756163_MRPS34	-0.43415	5.43E-06	6.80E-05
11724587_SYT1	0.697985	5.43E-06	6.80E-05
11724285_FRMD4A	-0.44643	5.43E-06	6.80E-05
11754137_QKI	-0.62868	5.43E-06	6.80E-05
11747324_NCBP2	-0.42464	5.44E-06	6.81E-05
11757156_SNHG12 //	-0.45432	5.44E-06	6.81E-05
11717098_RPA2	-0.473	5.45E-06	6.82E-05
11724915_TMTC3	0.609546	5.45E-06	6.82E-05
11716283_PAPD7	-0.42977	5.46E-06	6.82E-05
11743092_THEM4	-0.47	5.46E-06	6.82E-05
11736305_MIR22HG	0.410166	5.46E-06	6.82E-05
11760911_NSUN5P1 /	0.405238	5.46E-06	6.82E-05
11758974_H2AFV	-0.43891	5.46E-06	6.82E-05
11757973_NMT2	0.418689	5.47E-06	6.83E-05
11731194_SEMA3D	0.885478	5.47E-06	6.83E-05
11716744_TJP2	0.39335	5.48E-06	6.83E-05
11727992_STX11	1.400774	5.48E-06	6.83E-05
11728660_HN1L	-0.87243	5.48E-06	6.83E-05
11722179_DR1	0.442115	5.48E-06	6.83E-05
11724838_NCEH1	0.732446	5.49E-06	6.84E-05
11715842_MAPK1	0.490936	5.49E-06	6.85E-05
11743761_SNIP1	0.505587	5.50E-06	6.85E-05
11753780_NEK2	-0.51305	5.50E-06	6.85E-05
11759196_METTL10	-1.24872	5.52E-06	6.87E-05
11745467_TMED5	0.472971	5.54E-06	6.90E-05

11740148_ZNF429	0.971848	5.54E-06	6.90E-05
11734137_MTBP	-0.58018	5.54E-06	6.90E-05
11739409_BAGE2 ///	0.499735	5.55E-06	6.90E-05
11718678_PSEN1	0.507702	5.55E-06	6.90E-05
11758072_TMEM161B	0.438106	5.56E-06	6.91E-05
11729229_KLF3	0.6499	5.56E-06	6.91E-05
11739953_ZNF14	0.74895	5.56E-06	6.91E-05
11732960_EIF2D	-0.46057	5.56E-06	6.91E-05
11733149_DDX58	0.665521	5.57E-06	6.92E-05
11760464_MED6	0.537921	5.57E-06	6.92E-05
11759405_STX10	-0.44976	5.57E-06	6.92E-05
11726819_TMEM201	-0.61814	5.57E-06	6.92E-05
11722609_CDC42SE1	0.428131	5.58E-06	6.92E-05
11739040_GTF2H1	0.416434	5.59E-06	6.93E-05
11736366_MCM10	-0.7662	5.59E-06	6.93E-05
11726462_ZCCHC10	0.414604	5.59E-06	6.93E-05
11748082_FLOT1	-0.60557	5.59E-06	6.93E-05
11720222_GFPT1	0.613769	5.60E-06	6.93E-05
11743679_PTCH1	-0.5358	5.60E-06	6.93E-05
11744634_ZC3H7A	0.561894	5.60E-06	6.93E-05
11755664_WDR44	0.415571	5.60E-06	6.93E-05
11744841_TIPRL	-0.49229	5.60E-06	6.93E-05
11761824_NSAP11	0.62247	5.60E-06	6.93E-05
11761565_CEP70	-0.43414	5.60E-06	6.93E-05
11725255_IL15	0.567411	5.61E-06	6.93E-05
11746793_ZNF410	0.418863	5.61E-06	6.93E-05
11756275_PDLIM1	0.476775	5.62E-06	6.94E-05
11755694_AGFG1	0.422561	5.62E-06	6.95E-05
11757786_EXOC5	0.383824	5.64E-06	6.97E-05
11750423_SEPSECS	0.52459	5.65E-06	6.98E-05
11747801_HDAC9	1.04374	5.65E-06	6.98E-05
11722126_CCDC174	0.468804	5.66E-06	6.99E-05
11756111_ARAF	-0.39483	5.66E-06	6.99E-05
11752899_SEC62	0.42474	5.67E-06	7.00E-05
11729584_SLC25A6	-0.48161	5.67E-06	7.00E-05
11719407_HIPK2	0.46158	5.67E-06	7.00E-05
11730500_STRN	0.573694	5.67E-06	7.00E-05
11727389_TRPV2	0.600338	5.68E-06	7.00E-05
11741091_ARNTL	0.4862	5.68E-06	7.00E-05
11743018_NOC3L	0.479823	5.68E-06	7.00E-05
11758562_C1GALT1	0.403366	5.68E-06	7.00E-05
11726596_DUT	-0.46126	5.69E-06	7.01E-05
11720205_CLDN12	0.541542	5.70E-06	7.02E-05
11717619_EPHA2	0.493163	5.71E-06	7.02E-05
11758432_CPEB1	0.551694	5.72E-06	7.04E-05
11749934_HSP90AA4F	-0.38688	5.72E-06	7.04E-05
11754604_SPP1	0.518316	5.73E-06	7.05E-05
11721880_ARID4B	0.484259	5.73E-06	7.05E-05
11726265_ME1	-0.53282	5.74E-06	7.05E-05
11730886_FAM63B	0.386459	5.74E-06	7.06E-05
11723702_ABHD5	0.621821	5.75E-06	7.06E-05

11747493_TMEM128	-0.40833	5.75E-06	7.07E-05
11750554_NEDD4	0.546648	5.76E-06	7.07E-05
11732103_HIST1H3H	0.592648	5.76E-06	7.07E-05
11724921_VPS13B	0.42588	5.76E-06	7.07E-05
11726801_EBF1	0.743928	5.77E-06	7.08E-05
11759667_TTC17	0.536219	5.78E-06	7.09E-05
11759550_ZFAS1	-0.70105	5.79E-06	7.10E-05
11715840_SDHC	-0.66245	5.80E-06	7.11E-05
11728956_ZSWIM4	0.856867	5.80E-06	7.11E-05
11721925_BCL9	-0.47092	5.81E-06	7.12E-05
11724335_USP8	0.385874	5.81E-06	7.12E-05
11717716_FNDC3B	0.494299	5.82E-06	7.13E-05
11754315_TMSB4X	-0.6197	5.83E-06	7.14E-05
11724245_TMEM127	-0.60607	5.83E-06	7.14E-05
11756256_FANCG	-0.69846	5.83E-06	7.14E-05
11752167_SOCS4	0.879698	5.84E-06	7.14E-05
11731237_ZDBF2	-0.91955	5.84E-06	7.14E-05
11721001_HEXIM1	-0.46649	5.84E-06	7.14E-05
11753647_DR1	0.462965	5.84E-06	7.15E-05
11737944_SPP1	0.563414	5.84E-06	7.15E-05
11720802_BIN3	-0.39392	5.85E-06	7.15E-05
11749790_CASP8	0.65906	5.85E-06	7.15E-05
11752439_MIA3	0.460011	5.85E-06	7.15E-05
200060_PM RNPS1	-0.38882	5.86E-06	7.15E-05
11741188_SLC30A6	0.519543	5.86E-06	7.15E-05
11749045_TAPT1	0.663388	5.86E-06	7.15E-05
11721278_SUV39H1	-0.65607	5.86E-06	7.15E-05
11745766_PRRC2A	-0.39618	5.86E-06	7.15E-05
11755347_SAR1B	0.409897	5.87E-06	7.15E-05
11754437_KIAA0754	0.447723	5.87E-06	7.15E-05
11731101_MFI2	-0.4354	5.87E-06	7.16E-05
11747048_ZDHHC20	0.653924	5.87E-06	7.16E-05
11718811_PRKCH	0.598236	5.88E-06	7.17E-05
11727174_EPC2	0.514967	5.88E-06	7.17E-05
11750593_TGM2	-0.51824	5.89E-06	7.17E-05
11750627_HSPA4L	0.486466	5.90E-06	7.19E-05
11717537_VEGFB	-0.43796	5.91E-06	7.19E-05
11717014_SNRPF	-0.41794	5.91E-06	7.19E-05
11754907_RNF38	0.690353	5.91E-06	7.20E-05
11740132_DENND1B	0.538423	5.92E-06	7.20E-05
11736338_TXLNG	0.46468	5.92E-06	7.20E-05
11723797_ZDHHC12	-0.42298	5.92E-06	7.20E-05
11717985_PITPNB	0.43475	5.93E-06	7.21E-05
11718107_IPO5	-0.42517	5.93E-06	7.21E-05
11756711_CAST	0.460946	5.94E-06	7.21E-05
11754153_CASP8	0.532817	5.96E-06	7.24E-05
11747342_TTF2	-0.47125	5.96E-06	7.24E-05
11755150_CCL3P1 //	0.661097	5.97E-06	7.25E-05
11716958_AAAS	-0.47208	5.98E-06	7.25E-05
11743372_PTGES3	-0.42088	5.98E-06	7.26E-05
11724197_STC1	0.84001	5.98E-06	7.26E-05

11725710_PPIA	-0.47087	5.99E-06	7.26E-05
11752978_DR1	0.386095	5.99E-06	7.26E-05
11721753_ALG13	0.491616	5.99E-06	7.26E-05
11739433_ATP11B	-0.55643	6.00E-06	7.27E-05
11763368_ZGRF1	-0.70882	6.00E-06	7.27E-05
11744059_UQCRC2	-0.43828	6.01E-06	7.28E-05
11734017_UBQLN1	-0.41027	6.02E-06	7.29E-05
11741607_RRP12	0.597022	6.02E-06	7.29E-05
11716371_VPS26B	-0.58455	6.02E-06	7.29E-05
11742759_TRIP11	0.465834	6.03E-06	7.30E-05
11718178_CMTM4	-0.67069	6.03E-06	7.30E-05
11751451_TRIML2	0.903259	6.04E-06	7.30E-05
11746113_SYNE1	0.616933	6.04E-06	7.30E-05
11724837_NCEH1	0.862833	6.04E-06	7.30E-05
11754928_MYOF	0.490149	6.06E-06	7.32E-05
11746036_CBR1	-0.49317	6.06E-06	7.33E-05
11751777_ARMC1	-0.40536	6.06E-06	7.33E-05
11722949_CDRT4 ///	0.400624	6.07E-06	7.33E-05
11747942_ZEB2	0.707234	6.07E-06	7.33E-05
11734752_ARHGAP24	0.613452	6.08E-06	7.33E-05
11715600_PPP1R11	-0.648	6.08E-06	7.33E-05
11716963_PPM1F	-0.4534	6.08E-06	7.34E-05
11728071_NCAPG2	-0.71527	6.09E-06	7.35E-05
11746512_MTX3	0.652141	6.10E-06	7.36E-05
11715394_CD81	-0.40672	6.11E-06	7.37E-05
11749245_CXCL11	1.020751	6.12E-06	7.38E-05
11720993_SOX2	-0.53545	6.12E-06	7.38E-05
11756196_MTHFD1L	-0.55871	6.13E-06	7.38E-05
11758962_PAFAH1B2	-0.46368	6.14E-06	7.39E-05
11743576_CPE	0.4465	6.14E-06	7.40E-05
11753302_CBR1	-0.45208	6.15E-06	7.40E-05
11747233_KBTBD7	-0.53226	6.15E-06	7.41E-05
11725565_MANEA	-0.68297	6.16E-06	7.41E-05
11757675_C16orf87	0.505422	6.16E-06	7.41E-05
11763803_TMEM67	0.813714	6.17E-06	7.42E-05
11754313_ARL4C	0.47784	6.17E-06	7.42E-05
11716350_HSPE1 ///	-0.57509	6.19E-06	7.45E-05
11719662_FAM188A	0.543939	6.20E-06	7.45E-05
11716991_AREL1	0.430139	6.21E-06	7.46E-05
11739407_KMT2C	0.577861	6.22E-06	7.47E-05
11721998_PARP14	0.800574	6.22E-06	7.47E-05
11721632_TMEM97	-0.4349	6.22E-06	7.47E-05
11723587_RC3H1	0.422949	6.22E-06	7.47E-05
11732572_ZNF582	0.583607	6.23E-06	7.47E-05
11751364_SLC47A1	-0.91346	6.23E-06	7.47E-05
11722879_PPP2R2B	0.53142	6.23E-06	7.48E-05
11755077_ZFP69B	0.53031	6.23E-06	7.48E-05
11739218_CACUL1	-0.61531	6.24E-06	7.48E-05
11755583_FAM161A	-0.46021	6.24E-06	7.48E-05
11752040_PHC3	0.948275	6.24E-06	7.48E-05
11716257_RERE	0.560466	6.24E-06	7.48E-05

11723092_FNIP2	0.565417	6.25E-06	7.49E-05
11722933_CHML	0.666562	6.25E-06	7.49E-05
11738144_SSX2IP	-0.6665	6.25E-06	7.49E-05
11756205_DCAF6	0.384132	6.26E-06	7.49E-05
11760221_CASP4	0.882466	6.26E-06	7.49E-05
11751469_NF1	0.50007	6.27E-06	7.50E-05
11751742_MED6	0.396787	6.28E-06	7.51E-05
11739781_TGM2	-0.63905	6.28E-06	7.51E-05
11747540_ADAM9	0.390957	6.28E-06	7.51E-05
11745000_TXNRD2	0.484238	6.28E-06	7.51E-05
11725078_RIT1	0.645563	6.28E-06	7.51E-05
11731679_FAM20C	-0.69852	6.29E-06	7.52E-05
11730923_ADAMTS4	-0.51807	6.29E-06	7.52E-05
11743251_MMP2	0.428463	6.31E-06	7.53E-05
11726334_CRLS1	-0.45566	6.31E-06	7.53E-05
11751687_EDEM1	0.573167	6.31E-06	7.53E-05
11739235_PDE12	0.563208	6.31E-06	7.53E-05
11725149_C9orf40	-0.5902	6.33E-06	7.55E-05
11739455_SGMS1	0.466953	6.33E-06	7.55E-05
11717515_HSD17B10	0.445118	6.33E-06	7.55E-05
11725822_JPH1	-0.41647	6.35E-06	7.57E-05
11738125_SLC25A24	0.458558	6.35E-06	7.57E-05
11754541_CEP95	0.424358	6.36E-06	7.57E-05
11748104_CDC25C	-0.74868	6.36E-06	7.57E-05
11721294_GIT2	-0.60577	6.36E-06	7.58E-05
11742685_LAMP2 ///	0.480808	6.39E-06	7.61E-05
11717297_LRPPRC	-0.6354	6.39E-06	7.61E-05
11718942_LSM14B	-0.45382	6.41E-06	7.63E-05
11722163_PLK2	0.432518	6.42E-06	7.64E-05
11725890_ABCC6 ///	-1.10497	6.42E-06	7.64E-05
11719633_KLF4	0.65683	6.44E-06	7.65E-05
11745283_RBFOX2	0.402715	6.44E-06	7.66E-05
11733803_CPPED1	0.439197	6.44E-06	7.66E-05
11723720_SLAIN2	-0.58693	6.45E-06	7.66E-05
11763456_TACC3	-0.41605	6.45E-06	7.67E-05
11751859_PDCD1LG2	0.459261	6.46E-06	7.67E-05
11739281_TRIM23	0.556951	6.46E-06	7.67E-05
11728766_KLHL5	0.460623	6.47E-06	7.68E-05
11728427_IKBIP	0.588113	6.47E-06	7.68E-05
11735058_ARNT2	-0.53026	6.48E-06	7.69E-05
11717350_NCAPH2	-0.60501	6.49E-06	7.70E-05
11726494_SENP8	-0.39458	6.50E-06	7.71E-05
11735758_SH3D19	0.517954	6.51E-06	7.71E-05
11733906_THAP1	0.569065	6.51E-06	7.72E-05
11751658_ATP11B	0.596603	6.52E-06	7.73E-05
11728538_MCC	0.648419	6.52E-06	7.73E-05
11716610_AP2S1	-0.42922	6.53E-06	7.73E-05
11744146_RBM3	0.424382	6.53E-06	7.73E-05
11754125_COTL1	-0.59591	6.54E-06	7.74E-05
11727535_CCNF	-0.6773	6.56E-06	7.76E-05
11747708_CCSER2	0.699975	6.56E-06	7.77E-05

11721625_GLUL	-0.55412	6.59E-06	7.80E-05
11744480_RAB18	0.457402	6.59E-06	7.80E-05
11756869_PDZD2	-0.72081	6.60E-06	7.80E-05
11747440_YKT6	-0.48567	6.60E-06	7.80E-05
11721454_SERPINA1	0.78905	6.60E-06	7.80E-05
11727121_MYCBP2	0.414321	6.60E-06	7.80E-05
11722931_GART	-0.51164	6.61E-06	7.80E-05
11729173_SKA2	-0.3976	6.61E-06	7.80E-05
11739379_FAM198B	0.852318	6.61E-06	7.80E-05
11733946_RAP2B	0.386576	6.62E-06	7.82E-05
11724884_HAPLN3	-0.51771	6.62E-06	7.82E-05
11753418_PGGT1B	0.527649	6.63E-06	7.82E-05
11717091_CDKN1B	0.503252	6.63E-06	7.82E-05
11763164_DNAJC9	-0.57208	6.66E-06	7.85E-05
11749477_EDEM3	0.587052	6.66E-06	7.86E-05
11726587_TARS2	-0.66398	6.68E-06	7.87E-05
11721313_ESYT2	0.534056	6.68E-06	7.87E-05
11741554_CASP8	0.642627	6.70E-06	7.89E-05
11726081_CCNT2	0.452282	6.70E-06	7.89E-05
11731222_SHOX2	-0.69268	6.70E-06	7.90E-05
11740489_MAPK9	0.73376	6.71E-06	7.90E-05
11719471_DCP1A	-0.40198	6.72E-06	7.91E-05
11726615_CDH2	0.408399	6.72E-06	7.91E-05
11737210_HDGF	-0.40805	6.72E-06	7.91E-05
11749740_USP3	0.43559	6.73E-06	7.91E-05
11727495_TCEB3	0.399954	6.73E-06	7.91E-05
11735103_HDLBP	-0.52899	6.73E-06	7.91E-05
11719613_LARP4	0.46217	6.74E-06	7.92E-05
11756406_ADM	0.418384	6.74E-06	7.92E-05
11723583_RACGAP1	-0.44285	6.74E-06	7.92E-05
11715545_TMED10	-0.46216	6.74E-06	7.92E-05
11745124_BDH1	-0.71591	6.75E-06	7.92E-05
11746090_SLK	0.535465	6.77E-06	7.95E-05
11727833_WDR43	0.402848	6.77E-06	7.95E-05
11739696_AFF3	0.848352	6.78E-06	7.96E-05
11716725_ADCY6	-0.48258	6.79E-06	7.97E-05
11752350_EOGT	0.652997	6.80E-06	7.97E-05
11744566_DDX49	0.43945	6.80E-06	7.97E-05
11716767_IQGAP1	-0.46946	6.81E-06	7.98E-05
11755903_N4BP2L1	-0.40808	6.82E-06	7.99E-05
11758886_RHBDD1	-0.54384	6.82E-06	7.99E-05
11720259_TRIM33	-0.4274	6.82E-06	7.99E-05
11718324_SESN2	0.545782	6.82E-06	7.99E-05
11751067_AKIRIN1	0.563196	6.82E-06	7.99E-05
11717727_HN1	0.521228	6.83E-06	7.99E-05
11749412_PARP14	0.764165	6.83E-06	7.99E-05
11725849_SENP7	0.440826	6.83E-06	7.99E-05
11745833_ECT2	-0.42841	6.83E-06	7.99E-05
11720320_AK2	-0.4358	6.85E-06	8.01E-05
11744528_GON4L ///	0.387779	6.86E-06	8.02E-05
11717958_PATL1	0.526305	6.87E-06	8.03E-05

11729171_NIFK	0.429229	6.87E-06	8.03E-05
11720411_CYP1B1	1.050988	6.89E-06	8.05E-05
11758308_HSP90AA1	-0.38619	6.89E-06	8.05E-05
11749691_HELLS	-0.39724	6.89E-06	8.05E-05
11757052_TWF2	-0.44679	6.90E-06	8.05E-05
11716656_TRAF4	0.475825	6.90E-06	8.06E-05
11754005_ATP6V1G1	-0.43377	6.90E-06	8.06E-05
11720312_XRCC5	-0.37515	6.91E-06	8.06E-05
11733798_DDX5	-0.59241	6.92E-06	8.07E-05
11732976_GON4L	0.641017	6.93E-06	8.08E-05
11741631_C3orf58	-0.52588	6.93E-06	8.08E-05
11748476_DUSP3	-0.57936	6.94E-06	8.09E-05
11741126_SLFN11	-0.52956	6.95E-06	8.10E-05
11754967_CBX3	-0.38296	6.96E-06	8.10E-05
11718096_MEF2A	0.491164	6.97E-06	8.12E-05
11760877_CEP70	-0.49523	6.97E-06	8.12E-05
11720619_C19orf25	-0.46396	6.98E-06	8.12E-05
11719127_CGRRF1	0.460977	6.98E-06	8.12E-05
11756233_AKIRIN2	-0.44511	6.98E-06	8.12E-05
11734213_NDUFS3 //	-0.44683	6.99E-06	8.13E-05
11757907_HSPA1A //	-0.38612	6.99E-06	8.13E-05
11722912_USP13	-0.41934	6.99E-06	8.13E-05
11733092_FOXRED2	-0.60435	7.00E-06	8.13E-05
11749789_TP53I3	0.443179	7.00E-06	8.14E-05
11728735_ZBTB38	0.422353	7.01E-06	8.14E-05
11724385_HYLS1	-0.43134	7.01E-06	8.14E-05
11763294_ZSCAN9	0.570339	7.01E-06	8.14E-05
11742193_CNR1	0.564844	7.01E-06	8.14E-05
11717765_MGLL	0.462852	7.01E-06	8.14E-05
11739005_CAPZA1	-0.55127	7.03E-06	8.16E-05
11717902_IER3IP1	-0.41707	7.04E-06	8.16E-05
11732870_CASP1	0.945631	7.04E-06	8.16E-05
11743544_CEP57	-0.47447	7.04E-06	8.17E-05
11728094_MAP3K1	0.536394	7.05E-06	8.18E-05
11723242_CDK6	0.601222	7.06E-06	8.18E-05
200027_PMNARS	-0.48786	7.06E-06	8.18E-05
11757322_HAUS7	-0.58113	7.06E-06	8.18E-05
11723414_CNN2	0.726333	7.08E-06	8.20E-05
11750175_SVIL ///	0.55169	7.08E-06	8.20E-05
11736239_ABCA5	0.640938	7.10E-06	8.22E-05
11751635_PRKAB1	0.495818	7.10E-06	8.22E-05
11716705_TRIM44	0.408184	7.10E-06	8.22E-05
11746928_ENC1	0.52607	7.10E-06	8.22E-05
11731338_ZNF398	0.495432	7.12E-06	8.24E-05
11727194_ZNF202	0.484998	7.12E-06	8.24E-05
11763560_NF1	0.530216	7.13E-06	8.24E-05
11757937_BIRC2	0.385925	7.13E-06	8.24E-05
11722789_RAB5A	0.43223	7.14E-06	8.25E-05
11727178_MTHFR	0.446804	7.14E-06	8.25E-05
11755361_DET1	-0.74184	7.14E-06	8.25E-05
11727521_INTS6	0.454751	7.16E-06	8.26E-05

11725392_MAK16	0.432969	7.16E-06	8.27E-05
11727412_CLN8	0.472503	7.17E-06	8.28E-05
11735319_RBMS3	0.491879	7.18E-06	8.28E-05
11730745_AKIRIN1	0.444688	7.18E-06	8.29E-05
11744315_SGK223	0.603444	7.19E-06	8.29E-05
11741393_HMBOX1	0.509475	7.19E-06	8.29E-05
11760619_ABI2	0.417971	7.19E-06	8.29E-05
11756887_TNS2	-0.37858	7.20E-06	8.30E-05
11731109_ADAM17	0.439158	7.21E-06	8.31E-05
11715739_PLPP3	0.453083	7.23E-06	8.33E-05
11732204_NAP1L1	-0.56733	7.24E-06	8.34E-05
11731435_SLC7A6OS	0.48811	7.25E-06	8.34E-05
11725110_NDE1	-0.39067	7.25E-06	8.34E-05
11744000_NFKBIA	0.439356	7.25E-06	8.34E-05
11717052_SSR1	-0.41016	7.25E-06	8.35E-05
11749549_PIGX	-0.62313	7.26E-06	8.36E-05
11725210_NFXL1	0.398753	7.27E-06	8.36E-05
11725302_DDX19A	0.415413	7.27E-06	8.36E-05
11722688_LIFR	0.993228	7.28E-06	8.37E-05
11738027_DMD	0.709888	7.28E-06	8.37E-05
11718638_ELP6	-0.39895	7.28E-06	8.37E-05
11716127_BAG1	0.409747	7.29E-06	8.38E-05
11756635_PLCB4	0.861954	7.30E-06	8.38E-05
11741484_SCRN1	0.425661	7.30E-06	8.39E-05
11718297_TACC3	-0.465	7.32E-06	8.40E-05
11739411_KMT2C	0.489475	7.32E-06	8.40E-05
11730754_AGPAT5	0.424711	7.32E-06	8.40E-05
11743594_FMR1	0.448448	7.32E-06	8.40E-05
11761986_ITSN1	-0.40851	7.33E-06	8.41E-05
11737250_IL1RAPL1	0.598733	7.34E-06	8.42E-05
11727579_CSTF3	-0.43045	7.35E-06	8.42E-05
11719374_EDEM2	-0.46524	7.36E-06	8.43E-05
11717967_TYW1	-0.46743	7.36E-06	8.43E-05
11737790_PTRH2	0.380374	7.37E-06	8.44E-05
11745679_CTBP1	-0.4266	7.38E-06	8.45E-05
11722385_UBTF	-0.74567	7.39E-06	8.46E-05
11737197_KIF4A ///	-0.62053	7.39E-06	8.46E-05
11745236_DPYD	0.404334	7.39E-06	8.46E-05
11725538_FAM199X	0.763176	7.39E-06	8.46E-05
11737747_GMFB	-0.50903	7.40E-06	8.46E-05
11733074_PPP1R12A	0.41265	7.40E-06	8.47E-05
11720601_SYT11	0.555252	7.40E-06	8.47E-05
11718610_TP53INP1	0.608393	7.41E-06	8.47E-05
11718260_NOTCH1	-0.44255	7.41E-06	8.47E-05
11748045_LDLR	-0.4681	7.42E-06	8.48E-05
11729545_DZANK1	0.535298	7.42E-06	8.48E-05
11736834_TM2D2	-0.55924	7.43E-06	8.48E-05
11755032_ASAP1	0.405327	7.43E-06	8.48E-05
11759019_MATR3	-0.39447	7.44E-06	8.49E-05
11724380_NHSL1	-0.38798	7.45E-06	8.50E-05
11739979_AKAP2 ///	0.446925	7.46E-06	8.52E-05

11756543_NXF1	0.407251	7.47E-06	8.52E-05
11732389_DYNC2LI1	0.413343	7.47E-06	8.52E-05
11716733_IRF1	1.017819	7.49E-06	8.54E-05
11717174_ABL1	-0.65885	7.50E-06	8.55E-05
11754234_KLHDC10	0.760306	7.52E-06	8.56E-05
11727774_ARL13B	0.407192	7.52E-06	8.56E-05
11746224_SAYSD1	-0.50348	7.52E-06	8.56E-05
11729664_STK38L	-0.98778	7.52E-06	8.57E-05
11720485_SENP2	-0.56753	7.53E-06	8.57E-05
11736437_DEDD	0.407364	7.53E-06	8.58E-05
11717687_JAK1	0.415449	7.54E-06	8.58E-05
11730289_ARHGAP5	-0.41795	7.55E-06	8.59E-05
11722548_HIVEP1	0.438703	7.55E-06	8.59E-05
11729151_PIGM	-0.45678	7.56E-06	8.60E-05
11735464_ABHD17B	0.436547	7.57E-06	8.61E-05
11733104_POLD3	-0.5409	7.58E-06	8.61E-05
11728494_ZNF197	0.580687	7.58E-06	8.61E-05
11727103_IGF2BP1	0.415841	7.59E-06	8.62E-05
11752765_TXNIP	-0.4751	7.60E-06	8.63E-05
11723454_RIOK2	0.387181	7.60E-06	8.63E-05
11723356_CHD7	0.412746	7.64E-06	8.67E-05
11721804_BUB3	-0.41487	7.64E-06	8.67E-05
11736342_EGR3	0.631582	7.65E-06	8.68E-05
11730575_ZNF470	0.632664	7.65E-06	8.68E-05
11723431_ZFP91	0.376515	7.67E-06	8.70E-05
11721642_CAP2	0.730423	7.67E-06	8.70E-05
11716114_POR	-0.39574	7.67E-06	8.70E-05
11754293_STK40	0.606085	7.67E-06	8.70E-05
11753913_SSX2IP	-0.53014	7.68E-06	8.71E-05
11741283_AKAP7	-1.09071	7.68E-06	8.71E-05
11751750_NXF1	0.490248	7.69E-06	8.71E-05
11725080_RNFT1	0.539551	7.69E-06	8.71E-05
11748702_ANXA4	0.399243	7.70E-06	8.71E-05
11753334_BAG1	0.44153	7.70E-06	8.71E-05
11732293_MTMR2	0.435983	7.70E-06	8.71E-05
11723686_FAM46A	0.501744	7.70E-06	8.72E-05
11736663_ZNF354B	0.669688	7.71E-06	8.73E-05
11717561_DTX3L	0.520321	7.72E-06	8.73E-05
11729617_UBE2J2	0.410617	7.72E-06	8.73E-05
11728372_DNAJC16	0.521427	7.72E-06	8.73E-05
11758512_EIF4E	-0.49643	7.72E-06	8.73E-05
11743025_ATP7A	0.607985	7.73E-06	8.73E-05
11755929_THOP1	-0.45023	7.73E-06	8.73E-05
11740083_AGMO	-1.08685	7.74E-06	8.74E-05
11753332_HNRNPL	-0.43036	7.75E-06	8.75E-05
11726156_POLA1	-0.41461	7.75E-06	8.75E-05
11747988_SLC39A10	0.414706	7.75E-06	8.75E-05
11758123_RELL2	0.523869	7.76E-06	8.76E-05
11744687_ATG4B	-0.41943	7.76E-06	8.76E-05
11745284_RBFOX2	0.437461	7.77E-06	8.77E-05
11732220_ECI1	-0.53322	7.78E-06	8.77E-05

11745856_RNF145	0.499797	7.78E-06	8.77E-05
11728313_ZNF148	-0.82745	7.78E-06	8.77E-05
11720028_LDLR	-0.56314	7.78E-06	8.77E-05
11746418_VIT	0.500656	7.79E-06	8.78E-05
11727150_PRICKLE1	1.061527	7.80E-06	8.78E-05
11739270_PAK2	-0.42831	7.80E-06	8.78E-05
11755490_EXOC7	-0.56661	7.81E-06	8.79E-05
11724602_ERAP1	0.462044	7.81E-06	8.79E-05
11723440_EPHA4	0.744422	7.81E-06	8.79E-05
11754133_INPP4B	0.5335	7.82E-06	8.80E-05
11749682_EXOC5	0.453334	7.83E-06	8.80E-05
11750166_EED	-0.40432	7.83E-06	8.80E-05
11724425_FAM98A	0.387986	7.84E-06	8.81E-05
11750634_LOC100996	0.458115	7.84E-06	8.81E-05
11737369_HOXC5	-0.47354	7.84E-06	8.81E-05
11751550_CD46	0.471313	7.84E-06	8.81E-05
11757917_CNOT6L	-0.52014	7.85E-06	8.82E-05
11718706_IGF1R	-0.67889	7.86E-06	8.83E-05
11740123_ZNF419	0.680698	7.86E-06	8.83E-05
11751369_PKD2	0.505228	7.87E-06	8.83E-05
11729638_FAM134C	-0.49561	7.87E-06	8.83E-05
11725576_CDC14A	0.734542	7.87E-06	8.84E-05
11739212_ARL14EP	0.486644	7.88E-06	8.84E-05
11724604_HS6ST2	-0.46641	7.88E-06	8.84E-05
11753491_ERP29	-0.68418	7.90E-06	8.85E-05
11721774_ASPH	0.577431	7.90E-06	8.85E-05
11759439_PRKAR1B	-0.63644	7.91E-06	8.86E-05
11758676_RHOQ	0.510581	7.91E-06	8.87E-05
11718823_EDRF1	0.390884	7.92E-06	8.87E-05
11749356_DCUN1D4	0.524276	7.92E-06	8.88E-05
11750105_HNRNPAB	-0.37805	7.94E-06	8.89E-05
11729684_THAP10	-0.45036	7.94E-06	8.89E-05
11757479_TMEM141	-0.59049	7.95E-06	8.90E-05
11725923_ASAP2	0.42129	7.96E-06	8.91E-05
11737794_FGFR1OP	0.608806	7.97E-06	8.91E-05
11742220_CD46	0.415827	7.97E-06	8.92E-05
11748291_RTFDC1	-0.46264	8.02E-06	8.96E-05
11717190_TXNIP	-0.49407	8.02E-06	8.97E-05
11732242_PLK4	-0.45177	8.02E-06	8.97E-05
11737527_DYRK1A	0.482741	8.03E-06	8.97E-05
11752822_ACTR1A	-0.38873	8.03E-06	8.97E-05
11750812_FLI1	0.577857	8.03E-06	8.97E-05
11739923_ZNF37A	0.384007	8.04E-06	8.97E-05
11736238_ABCA5	0.763019	8.04E-06	8.98E-05
11741765_METTL20	-0.45896	8.05E-06	8.98E-05
11743774_LZTFL1	0.448195	8.05E-06	8.98E-05
11720660_CDK9	-0.51205	8.06E-06	8.99E-05
11727287_C5orf34	-0.45042	8.06E-06	8.99E-05
11754675_CLIP4	0.489432	8.06E-06	8.99E-05
11722226_SDHAF3	-0.70249	8.07E-06	8.99E-05
11732373_TSEN34	-0.37929	8.07E-06	8.99E-05

11754702_ZNF493	0.564024	8.08E-06	9.00E-05
11717963_KRAS	-0.38786	8.08E-06	9.00E-05
11718249_TBC1D13	-0.52851	8.09E-06	9.01E-05
11756402_MAGOHB	-0.44697	8.09E-06	9.01E-05
11743673_KDM6B	0.711005	8.10E-06	9.01E-05
11726192_TFAP2C	-0.40266	8.10E-06	9.01E-05
11720784_ACTL6A	-0.39378	8.10E-06	9.01E-05
11721840_GLIPR1	0.782463	8.10E-06	9.01E-05
11751133_ATP6V1B2	0.486973	8.12E-06	9.03E-05
11751613_C20orf24	-0.37569	8.12E-06	9.03E-05
11725651_DCAF5	0.458337	8.12E-06	9.03E-05
11756641_ZNF256	0.686273	8.13E-06	9.03E-05
11733294_PHF20L1	0.630167	8.14E-06	9.04E-05
11760590_OGFRL1	0.801612	8.14E-06	9.04E-05
11746692_BAZ1A	0.464973	8.14E-06	9.04E-05
11717769_CTR9	0.45189	8.15E-06	9.04E-05
11754101_TAB2	0.505363	8.15E-06	9.04E-05
11728389_TSPAN2	1.813947	8.15E-06	9.04E-05
11754983_DAZAP1	-0.42332	8.15E-06	9.04E-05
11759290_AKT3	0.434287	8.15E-06	9.04E-05
11717897_PTP4A1	0.511649	8.15E-06	9.04E-05
11757526_C19orf60	-0.44195	8.17E-06	9.06E-05
11756962_HDAC6	-0.69383	8.17E-06	9.06E-05
11751310_SNIP1	0.517108	8.17E-06	9.06E-05
11739642_STXBP5	0.613339	8.18E-06	9.06E-05
11739965_GPR39	1.038702	8.18E-06	9.06E-05
11727060_MRPS36	-0.50077	8.19E-06	9.07E-05
11750244_MGLL	0.461729	8.19E-06	9.07E-05
11752050_WDR44	0.439125	8.20E-06	9.07E-05
11760405_TRMT13	0.623032	8.22E-06	9.10E-05
11721074_LAS1L	-0.46991	8.22E-06	9.10E-05
11744816_RAPH1	0.528483	8.22E-06	9.10E-05
11717424_CHMP3 ///	-0.49133	8.23E-06	9.11E-05
11745754_SQSTM1	0.405854	8.23E-06	9.11E-05
11747082_NSMCE4A	-0.44061	8.24E-06	9.11E-05
11747795_R3HCC1L	0.502266	8.24E-06	9.11E-05
11740237_KAT6A	-0.39265	8.25E-06	9.11E-05
11744635_ZC3H7A	0.520155	8.25E-06	9.12E-05
11733220_MRPL11	-0.3728	8.26E-06	9.13E-05
11724318_LRIF1	0.442329	8.26E-06	9.13E-05
11718424_ACD	-0.4251	8.27E-06	9.13E-05
11755279_HLA-DPB1	-0.46767	8.28E-06	9.14E-05
11741445_PTGES2	-0.56311	8.28E-06	9.14E-05
11739840_INPP4B	-0.68338	8.29E-06	9.14E-05
11721856_PIGK	-0.60864	8.29E-06	9.14E-05
11728692_LOC100996	0.516722	8.30E-06	9.16E-05
11742745_PLIN2	-0.71691	8.31E-06	9.16E-05
11758190_SDHD	-0.71975	8.31E-06	9.16E-05
11719554_CTSS	0.466525	8.31E-06	9.16E-05
11719802_ELF1	0.399003	8.32E-06	9.17E-05
11716676_ZNF106	0.400953	8.32E-06	9.17E-05

11759533_FIP1L1	-0.68113	8.33E-06	9.17E-05
11734181_GAN	0.924674	8.33E-06	9.17E-05
11739082_NFYC	-0.39703	8.33E-06	9.17E-05
11757812_INPP5A	-0.47217	8.34E-06	9.17E-05
11730390_SEMA3A	0.379393	8.34E-06	9.17E-05
11752931_GBP1	0.858548	8.35E-06	9.18E-05
11718960_NUFIP2	-0.39002	8.37E-06	9.20E-05
11725212_PLCXD1	-0.40802	8.38E-06	9.21E-05
11758031_BHLHE41	0.668647	8.39E-06	9.23E-05
11748033_KIAA0895	0.787734	8.39E-06	9.23E-05
11760121_LONP2	-0.41485	8.40E-06	9.24E-05
11728288_KRT15	0.750461	8.41E-06	9.24E-05
11719840_PHLDA3	0.44884	8.42E-06	9.25E-05
11739451_FRMD6	0.430548	8.43E-06	9.25E-05
11750245_MGLL	0.514367	8.43E-06	9.25E-05
11733200_CCDC14	-0.38291	8.43E-06	9.25E-05
11756761_SQSTM1	0.363595	8.43E-06	9.26E-05
11734207_ZNF155	0.537606	8.44E-06	9.26E-05
11746837_PLK2	0.404356	8.44E-06	9.26E-05
11753808_LSM3	-0.51264	8.45E-06	9.27E-05
11757843_PEA15	0.35535	8.46E-06	9.28E-05
11741362_ZNF26	0.692862	8.47E-06	9.28E-05
11726640_MIB1	-0.76996	8.47E-06	9.28E-05
11758434_FBXW2	-0.55971	8.47E-06	9.28E-05
11735495_C11orf63	0.786141	8.47E-06	9.28E-05
11743486_NAA25	0.372641	8.48E-06	9.29E-05
11761466_INTS4	0.528036	8.50E-06	9.31E-05
11745781_ZNF540	1.521099	8.52E-06	9.33E-05
11747836_RPS6KB1	0.439372	8.56E-06	9.37E-05
11722814_RANBP2 //	0.399187	8.56E-06	9.37E-05
11720839_SMNDC1	0.382112	8.57E-06	9.38E-05
11743891_RUFY2	0.750296	8.58E-06	9.39E-05
11739191_AKT3	0.451063	8.60E-06	9.41E-05
11725224_ZSCAN9	0.543434	8.61E-06	9.41E-05
11725142_TGS1	-0.55764	8.61E-06	9.41E-05
11746437_FZD3	0.449789	8.61E-06	9.41E-05
11757284_MYDGF	-0.39709	8.62E-06	9.42E-05
11743013_RNF115	0.416234	8.62E-06	9.42E-05
11741051_IMPDH1	-0.7117	8.63E-06	9.42E-05
11755051_USP25	0.603575	8.63E-06	9.43E-05
11746242_ARNTL2	0.49895	8.64E-06	9.43E-05
11743532_RUFY3	0.415432	8.66E-06	9.44E-05
11729200_C6orf120	0.479279	8.66E-06	9.44E-05
11755942_C19orf54	-0.42173	8.66E-06	9.45E-05
11719036_UVRAG	-0.49001	8.67E-06	9.45E-05
11754620_PKP4	0.527568	8.67E-06	9.45E-05
11753173_ZNF410	0.42734	8.67E-06	9.46E-05
11716093_KLF6	0.522744	8.69E-06	9.47E-05
11752445_NF1	0.513643	8.70E-06	9.48E-05
11728131_EXOC8	0.425955	8.70E-06	9.48E-05
11724782_HERC4	0.410063	8.70E-06	9.48E-05

11739135_COL5A2	0.47835	8.71E-06	9.48E-05
11754330_IRF2	0.453337	8.72E-06	9.49E-05
11751356_SDCBP	0.390256	8.74E-06	9.51E-05
11753990_USP47	0.494491	8.75E-06	9.52E-05
11716574_KDM3A	0.3711	8.76E-06	9.53E-05
11728081_BARX1	-0.41575	8.76E-06	9.53E-05
11729502_ASCC3	0.499914	8.77E-06	9.53E-05
11748544_TXNIP	-0.42273	8.77E-06	9.53E-05
11722409_GALNT7	0.504633	8.77E-06	9.53E-05
11758567_IKBIP	0.429995	8.78E-06	9.54E-05
11717655_SPRED2	0.421377	8.78E-06	9.54E-05
11732456_ 1-Mar	0.739817	8.78E-06	9.54E-05
11727607_OGG1	-0.37489	8.78E-06	9.54E-05
11747553_MCCC1	0.441464	8.79E-06	9.54E-05
11716287_RABAC1	-0.40604	8.79E-06	9.54E-05
11719992_NLK	0.597895	8.79E-06	9.55E-05
11720081_CBX6	-0.40594	8.80E-06	9.55E-05
11751895_DOCK4	0.471114	8.80E-06	9.55E-05
11723580_SMIM13	0.453901	8.80E-06	9.55E-05
11728234_CLDN1	1.058542	8.81E-06	9.55E-05
11755872_ATP11B	0.515232	8.82E-06	9.56E-05
11724941_ZNF343	0.401	8.82E-06	9.56E-05
11734068_SSR3	0.404523	8.83E-06	9.57E-05
11757888_DNAAF5	-0.37448	8.85E-06	9.59E-05
11743977_MYL12A	0.370356	8.85E-06	9.59E-05
11740195_ZNF626	0.414384	8.86E-06	9.60E-05
11750147_TRIM4	0.537277	8.88E-06	9.61E-05
11750465_NFYC	-0.45437	8.88E-06	9.61E-05
11716577_SDC1	-0.46379	8.89E-06	9.63E-05
11749780_BCAT1	0.446119	8.90E-06	9.63E-05
11722566_NT5C2	0.489188	8.90E-06	9.63E-05
11733869_HLA-DOA	-0.48418	8.91E-06	9.64E-05
11717419_DNAL1	0.669789	8.92E-06	9.64E-05
11733381_OSCP1	0.398597	8.92E-06	9.64E-05
11743630_RPL26L1	-0.38134	8.92E-06	9.64E-05
11759663_SP100	0.606298	8.92E-06	9.64E-05
11723312_PXMP2	-0.42096	8.93E-06	9.64E-05
11745328_LRPPRC	0.460555	8.93E-06	9.64E-05
11745997_AP1G1	0.689674	8.96E-06	9.67E-05
11716117_PDIA4	-0.44303	8.96E-06	9.67E-05
11754592_ATF5	-0.56598	8.97E-06	9.68E-05
11762366_RBPJ	-0.52343	8.99E-06	9.70E-05
11719391_PRKDC	-0.38394	9.00E-06	9.71E-05
11721822_FSD1	-0.41742	9.00E-06	9.71E-05
11717966_TYW1 ///	-0.3757	9.01E-06	9.72E-05
11747369_C1orf112	-0.56034	9.02E-06	9.72E-05
11747357_CIAPIN1	-0.43693	9.03E-06	9.73E-05
11755947_CMBL	0.465515	9.03E-06	9.73E-05
11720975_WIPF2	-0.4083	9.03E-06	9.73E-05
11727847_GNA12	-0.48115	9.04E-06	9.74E-05
11721918_BCL7C	-0.42845	9.05E-06	9.75E-05

11763847_LINC01119	0.619801	9.06E-06	9.75E-05
11727208_DDHD1	0.675971	9.06E-06	9.75E-05
11728565_FAM210A	0.370571	9.06E-06	9.75E-05
11715568_CD9	-0.39308	9.06E-06	9.75E-05
11763503_NF1	0.933106	9.08E-06	9.76E-05
11735542_CDC14A	0.772131	9.08E-06	9.77E-05
11723771_MASTL	-0.42906	9.09E-06	9.77E-05
11751328_FGFR1OP	0.645181	9.10E-06	9.78E-05
11743709_NAA15	-0.55866	9.10E-06	9.78E-05
11736333_PHF20	-0.39744	9.11E-06	9.79E-05
11722542_HIVEP2	0.843534	9.12E-06	9.80E-05
11730478_RNF125	-0.49805	9.13E-06	9.81E-05
11728053_INPP5B	0.435355	9.13E-06	9.81E-05
11723557_DCAF16	-0.77851	9.15E-06	9.82E-05
11749421_CEP85	-0.56724	9.15E-06	9.83E-05
11757373_APOC1	-0.65695	9.17E-06	9.84E-05
11749475_ELMO1	-0.51167	9.22E-06	9.89E-05
11755123_LPCAT3	-0.47387	9.22E-06	9.89E-05
11733218_TFAP4	-0.42243	9.23E-06	9.90E-05
11746174_IDH1	-0.44355	9.26E-06	9.93E-05
11745127_TNNT1	-0.39474	9.26E-06	9.93E-05
11743904_PDLIM1	0.439186	9.26E-06	9.93E-05
11752754_SDC1	-0.58927	9.27E-06	9.93E-05
11721734_GCH1	-0.73252	9.31E-06	9.97E-05
11717125_SH3PXD2A	-0.37794	9.31E-06	9.98E-05
11725463_MAU2	-0.44566	9.32E-06	9.98E-05
11758020_EIF5	0.356438	9.33E-06	9.99E-05
11754568_FOXRED1	-0.48806	9.33E-06	9.99E-05
11759503_FAM103A1	0.370961	9.35E-06	0.0001
11739432_ATP11B	-0.58143	9.35E-06	0.0001
11739311_HUS1	0.757589	9.37E-06	0.0001
11715546_CBX1	-0.4151	9.37E-06	0.0001
11720504_RAB6B	-0.41616	9.39E-06	0.0001
11729613_H2AFZ	-0.37006	9.39E-06	0.0001
11727941_EME1	-0.46971	9.40E-06	0.0001
11739977_GPR180	0.489086	9.40E-06	0.0001
11763770_TMEM67	0.858587	9.40E-06	0.0001
11743940_CCDC90B	0.41291	9.40E-06	0.0001
11749451_DERA	-0.57325	9.41E-06	0.0001
11727826_NDUFAF7	0.444706	9.42E-06	0.000101
11721699_PMS1	-0.41459	9.44E-06	0.000101
11749000_MSH6	-0.47743	9.44E-06	0.000101
11730054_SNX16	0.661193	9.44E-06	0.000101
11719584_CNNM3	-0.91209	9.45E-06	0.000101
11730546_EFCAB11	-0.39465	9.47E-06	0.000101
11726342_DNAJB14 /	0.409827	9.47E-06	0.000101
11743523_KDM5A	0.393118	9.49E-06	0.000101
11751223_MAPK1	0.393825	9.49E-06	0.000101
11722049_DUSP6	0.398365	9.49E-06	0.000101
11754751_BAG1	0.433627	9.50E-06	0.000101
11743909_ALG14	-0.45171	9.50E-06	0.000101

11743978_MYL12A	0.359233	9.50E-06	0.000101
11718000_MPLKIP	-0.42137	9.51E-06	0.000101
11723596_MAP2K7	-0.39772	9.53E-06	0.000101
11727528_PHF20L1	0.477733	9.54E-06	0.000102
11751553_DEPDC7	0.415233	9.56E-06	0.000102
11718147_ATG5	0.442907	9.56E-06	0.000102
11726806_PTPMT1	-0.43295	9.57E-06	0.000102
11747154_FXYD6 ///	-0.68218	9.58E-06	0.000102
11718210_PYCR1	-0.37634	9.59E-06	0.000102
11754135_H2AFZ	-0.41068	9.60E-06	0.000102
11717562_DTX3L	0.588009	9.62E-06	0.000102
11756307_MTMR10	0.73209	9.63E-06	0.000102
11722432_C1orf27	0.539108	9.63E-06	0.000102
11746867_GRIK2	0.690755	9.64E-06	0.000102
11757671_FAM200B	0.385846	9.64E-06	0.000102
11758328_NCOA3	0.69354	9.67E-06	0.000103
11751689_GNE	0.597934	9.67E-06	0.000103
11754006_LAPTM4A	-0.38121	9.69E-06	0.000103
11754767_RPL7L1	-0.47517	9.71E-06	0.000103
11745355_PHLDB3	0.439618	9.72E-06	0.000103
11723584_RACGAP1	-0.37412	9.73E-06	0.000103
11761734_HSP90AA4F	-0.38465	9.73E-06	0.000103
11722106_PCNX	0.594011	9.74E-06	0.000103
11734465_HIST1H1A	-1.11758	9.75E-06	0.000103
11724169_ZNF322	0.483119	9.76E-06	0.000103
11726601_ZMPSTE24	0.465851	9.76E-06	0.000103
11751025_GALNT11	-0.42735	9.76E-06	0.000103
11760881_TTF2	-0.48198	9.78E-06	0.000104
11744416_SALL2	-0.38378	9.78E-06	0.000104
11743019_ZSCAN29	0.660048	9.80E-06	0.000104
11717589_EXOSC8	-0.38417	9.83E-06	0.000104
11731519_MFAP3	0.565556	9.86E-06	0.000104
11750790_UBE4A	0.443451	9.87E-06	0.000104
11741101_ZNF655	0.451286	9.89E-06	0.000105
11753749_ANP32A	-0.40156	9.90E-06	0.000105
11720804_PAGR1	-0.4206	9.90E-06	0.000105
11755587_TRIM22	1.281796	9.90E-06	0.000105
11744885_SPAG9	0.476638	9.91E-06	0.000105
11726385_MT1X	-0.52846	9.92E-06	0.000105
11719842_PHLDA3	0.471739	9.92E-06	0.000105
11744132_SECISBP2L	0.470324	9.94E-06	0.000105
11733130_ZSCAN26	0.512686	9.95E-06	0.000105
11718085_LYN	0.569257	9.96E-06	0.000105
11733303_ZNF790	0.879114	9.97E-06	0.000105
11738871_MAPK1	0.428945	9.98E-06	0.000105
11732303_CREB1	-0.3929	9.99E-06	0.000105
11733057_PTBP3	-0.38172	9.99E-06	0.000105
11740052_CENPP	-0.59041	1.00E-05	0.000106
11743160_HJURP	-0.37343	1.00E-05	0.000106
11742082_LCTL	0.397345	1.00E-05	0.000106
11727125_PVRL3	0.396396	1.01E-05	0.000106

11735850_MRGPRX4	0.791853	1.01E-05	0.000106
11754198_LINC00341	-0.7576	1.01E-05	0.000106
11729698_PPP2R4	-0.48108	1.01E-05	0.000106
11746548_CNN2	0.512203	1.01E-05	0.000106
11739655_EGFLAM	0.763325	1.01E-05	0.000106
200037_PMCBX3	-0.39621	1.01E-05	0.000106
11725056_NEDD9	0.498176	1.01E-05	0.000106
11751762_INTS6	0.376546	1.01E-05	0.000106
11744372_PRPSAP1	-0.37688	1.01E-05	0.000106
11742811_RPS6KA3	-0.39286	1.01E-05	0.000106
11728360_BCDIN3D	0.450557	1.02E-05	0.000107
11719498_ZPR1	0.504091	1.02E-05	0.000107
11756527_SNX10	-0.46165	1.02E-05	0.000107
11751805_TYMS	-0.38971	1.02E-05	0.000107
11718858_NUDCD3	-0.69048	1.02E-05	0.000107
11748074_PREB	-0.44907	1.02E-05	0.000107
11745249_TMEM40	0.741317	1.02E-05	0.000107
11725657_ZUFSP	0.388469	1.02E-05	0.000107
11722211_NASP	-0.59879	1.02E-05	0.000107
11761935_HNRNPLL	0.58789	1.02E-05	0.000107
11749042_DHCR24	-0.69845	1.02E-05	0.000107
11755882_SLC35B3	0.503315	1.03E-05	0.000107
11717945_FNBP4	0.410255	1.03E-05	0.000108
11716073_ATP6V1B2	0.428824	1.03E-05	0.000108
11732701_CSGALNACT	0.53656	1.03E-05	0.000108
11755445_ACTR8	0.459403	1.03E-05	0.000108
11746248_MANSC1	0.71501	1.03E-05	0.000108
11747338_ZNF544	0.564029	1.03E-05	0.000108
11731440_IFT57	0.359124	1.03E-05	0.000108
11762068_FCF1	-0.43489	1.03E-05	0.000108
11739173_SNAP23	0.424188	1.03E-05	0.000108
11751922_LRP6	0.71925	1.04E-05	0.000108
11739933_FAM111B	-0.88807	1.04E-05	0.000108
11755388_LOC441155	0.472312	1.04E-05	0.000108
11759131_HIST1H2AG	1.108971	1.04E-05	0.000109
11730837_WDR55	0.473383	1.04E-05	0.000109
11741155_LIMS1	0.370612	1.04E-05	0.000109
11752049_WDR44	0.427774	1.04E-05	0.000109
11727426_CANX	-0.52847	1.04E-05	0.000109
11757306_CD59	0.412099	1.04E-05	0.000109
11739793_LRIF1	0.423115	1.04E-05	0.000109
11746234_ZNF106	0.494028	1.04E-05	0.000109
11720694_HENMT1	-0.48637	1.04E-05	0.000109
11725028_PSPC1	-0.38588	1.05E-05	0.000109
11746311_ZNF326	-0.38752	1.05E-05	0.000109
11722453_QKI	-0.72898	1.05E-05	0.000109
11715518_MLEC	-0.45859	1.05E-05	0.000109
11719746_NF2	0.578342	1.05E-05	0.000109
11731054_FNIP1	0.525117	1.05E-05	0.000109
11746225_GAS6-AS1	0.503817	1.05E-05	0.000109
11746427_WDR66	0.957632	1.05E-05	0.000109

11720615_TUBG2	0.431083	1.05E-05	0.000109
11753091_AGPAT3	-0.45092	1.05E-05	0.000109
11722616_UBLCP1	0.377442	1.05E-05	0.00011
11750424_FOXP1	0.450657	1.05E-05	0.00011
11753546_SNRPB2	-0.39721	1.05E-05	0.00011
11739554_GAS7	-0.5994	1.05E-05	0.00011
11743065_CDC6	-0.41716	1.06E-05	0.00011
11724519_GUF1	0.489526	1.06E-05	0.00011
11733164_TTC29	0.562316	1.06E-05	0.00011
11759902_HACD4	-0.52457	1.06E-05	0.00011
11726024_EDN1	0.999449	1.06E-05	0.00011
11740257_CXorf23	0.679481	1.06E-05	0.00011
11725380_POGLUT1	0.515057	1.06E-05	0.00011
11722730_APPL1	-0.49682	1.06E-05	0.00011
11733106_COMMD2	0.46313	1.06E-05	0.00011
11741367_SDCBP	0.387839	1.06E-05	0.00011
11754913_TRPS1	0.393711	1.06E-05	0.00011
11715569_CD9	-0.36336	1.06E-05	0.00011
11757354_FBXO25	0.452278	1.06E-05	0.00011
11734546_SDCBP	0.412464	1.06E-05	0.00011
11753655_PHLPP2	0.60295	1.06E-05	0.00011
11762649_UBA6	1.012167	1.06E-05	0.00011
11747864_CPEB1	0.728803	1.06E-05	0.00011
11747783_AHCY	-0.35885	1.06E-05	0.00011
11718819_TSC1	0.459744	1.07E-05	0.000111
11756353_CLVS1	-0.54355	1.07E-05	0.000111
11716572_RPA1	-0.39854	1.07E-05	0.000111
11730969_THAP2	0.830944	1.07E-05	0.000111
11732489_NPHP3	0.521041	1.07E-05	0.000111
11718726_ZNF655	0.607214	1.07E-05	0.000111
11735329_CD47	-0.52071	1.07E-05	0.000111
11729632_YBX3	0.459033	1.07E-05	0.000111
11721026_OCEL1	-0.52127	1.07E-05	0.000111
11717451_NEK7	0.49349	1.07E-05	0.000111
11729182_NUAK2	0.477403	1.07E-05	0.000111
11730220_SPG20	0.451872	1.08E-05	0.000111
11715894_NGRN	0.415758	1.08E-05	0.000111
11750119_ZNF33A	0.443775	1.08E-05	0.000111
11727729_ZNF329	0.504664	1.08E-05	0.000111
11756162_PLPP3	0.482794	1.08E-05	0.000111
11732261_RRAGB	0.705079	1.08E-05	0.000111
11721550_POLR3C	0.432003	1.08E-05	0.000112
11745900_GON4L ///	0.378375	1.08E-05	0.000112
11755260_C12orf4	0.482855	1.08E-05	0.000112
11755142_EOGT	0.595685	1.09E-05	0.000112
11718488_ORC3	-0.39005	1.09E-05	0.000112
11721586_CASK	-0.64315	1.09E-05	0.000112
11747754_BTBD2	-0.54778	1.09E-05	0.000112
11751989_ITGB8	0.579662	1.09E-05	0.000112
11738446_NFAT5	0.525954	1.09E-05	0.000112
11755010_GALNT4 //	-0.6183	1.09E-05	0.000112

11731862_CDH19	0.51226	1.09E-05	0.000112
11751297_SUB1	-0.56854	1.09E-05	0.000112
11744999_TXNRD2	0.459724	1.09E-05	0.000112
11731826_ZNF425	0.843043	1.09E-05	0.000113
11741980_DUSP6	0.361023	1.10E-05	0.000113
11723832_TAPT1	0.525098	1.10E-05	0.000113
11716251_RBFOX2	0.392587	1.10E-05	0.000113
11746269_ABHD3	-0.73993	1.10E-05	0.000113
11733147_ZBTB14	-0.4977	1.10E-05	0.000113
11751532_CD46	0.42885	1.10E-05	0.000113
11734049_SEH1L	-0.47799	1.10E-05	0.000114
11718168_MYOF	0.398911	1.10E-05	0.000114
11742501_CLTA	0.463994	1.11E-05	0.000114
11757569_NDRG3	-0.42332	1.11E-05	0.000114
11726102_PPP2R3A	-0.46937	1.11E-05	0.000114
11756161_PMVK	-0.42342	1.11E-05	0.000114
11746476_CALD1	0.373793	1.11E-05	0.000114
11725202_ESCO1	0.40147	1.11E-05	0.000114
11717263_ATPAF1	-0.59635	1.11E-05	0.000114
11754419_C3orf17	-0.46776	1.11E-05	0.000114
11742784_SETX	0.546789	1.11E-05	0.000114
11745812_IDH3B	-0.41449	1.11E-05	0.000114
11748973_CDC25B	-0.46454	1.11E-05	0.000114
11745067_ODF2	-0.49964	1.12E-05	0.000114
11724945_LIG4	0.522375	1.12E-05	0.000114
11721733_GCH1	-0.79644	1.12E-05	0.000115
11747653_CDC25B	-0.42474	1.12E-05	0.000115
11717721_ST6GAL1	-0.44846	1.12E-05	0.000115
11740013_NEIL3	-0.84136	1.12E-05	0.000115
11760886_GPR89B	0.465158	1.12E-05	0.000115
11719461_WDR41	0.41211	1.12E-05	0.000115
11749388_IQCK	0.479432	1.12E-05	0.000115
11734708_SMIM15	-0.60446	1.12E-05	0.000115
11720653_BBX	-0.37511	1.12E-05	0.000115
11744377_USP24	0.503675	1.12E-05	0.000115
11715467_ARHGDIB	0.459465	1.12E-05	0.000115
11755425_FAM179B	0.598712	1.13E-05	0.000115
11731087_TM9SF1	-0.3619	1.13E-05	0.000115
11721325_GCAT	-0.79608	1.13E-05	0.000115
11733644_NECAP2	0.353953	1.13E-05	0.000115
11728997_IQGAP3	-0.63279	1.13E-05	0.000115
11744435_DUSP6	0.351674	1.13E-05	0.000115
11715930_SDHAF2	0.397265	1.13E-05	0.000115
11744317_BRCA1	-0.56557	1.13E-05	0.000115
11729390_MIER1	0.4914	1.13E-05	0.000115
11755089_ZNF615	0.455058	1.13E-05	0.000115
11723842_SNX11	0.454236	1.13E-05	0.000115
11757454_CREBL2	0.455034	1.13E-05	0.000116
11725363_SMAD4	-0.52071	1.13E-05	0.000116
11728482_ZNF181 //	0.364931	1.13E-05	0.000116
11727539_ISCU	0.366665	1.13E-05	0.000116

11740887_PROSER1	-0.49184	1.13E-05	0.000116
11716913_MTUS1	0.379968	1.14E-05	0.000116
11726150_PPIP5K2	-0.45362	1.14E-05	0.000116
11728483_ZNF181	0.504817	1.14E-05	0.000116
11729910_DNAH11	-0.47087	1.14E-05	0.000116
11724334_USP8	0.358155	1.14E-05	0.000116
11725792_MRE11A	-0.8386	1.14E-05	0.000116
11762038_ZNF844	0.800471	1.14E-05	0.000116
11748439_NTM	0.822103	1.14E-05	0.000116
11727486_HAUS6	-0.41732	1.14E-05	0.000116
11748477_RARG	-0.46889	1.14E-05	0.000116
11741124_SAR1B	0.421854	1.14E-05	0.000116
11725191_ADGRG6	0.496914	1.15E-05	0.000116
11722368_TRIM22	1.16088	1.15E-05	0.000116
11737878_GALNT2	-0.37207	1.15E-05	0.000116
11736963_FLCN	0.554631	1.15E-05	0.000116
11721828_ZWILCH	-0.35843	1.15E-05	0.000117
11726437_CMTM8	-0.39618	1.15E-05	0.000117
11721961_RC3H2	0.437824	1.15E-05	0.000117
11715887_PIP4K2B	0.457702	1.15E-05	0.000117
11727468_MAGEA4	-0.39377	1.15E-05	0.000117
11716403_EIF4EBP2	-0.60477	1.15E-05	0.000117
11749614_UBE2I	-0.41422	1.15E-05	0.000117
11731068_FIGNL1	-0.4036	1.15E-05	0.000117
11716786_TRAK2	0.431013	1.15E-05	0.000117
11736093_RBM42	-0.5166	1.15E-05	0.000117
11756133_TMEM138	0.402875	1.15E-05	0.000117
11723394_NEIL2	-1.08929	1.15E-05	0.000117
11715629_LAPTM4A	-0.38946	1.16E-05	0.000117
11737776_ZNF7	0.378837	1.16E-05	0.000117
11744073_PNKP	-0.44822	1.16E-05	0.000117
11729413_LRRC8A	-0.39894	1.16E-05	0.000117
11717585_PSMD10	-0.36967	1.16E-05	0.000117
11755136_ISY1	-0.37058	1.16E-05	0.000117
11752444_NF1	0.454933	1.16E-05	0.000117
11748535_TAF11	0.474306	1.16E-05	0.000118
11724652_LMBR1	0.412074	1.16E-05	0.000118
11757593_TAPT1	0.437218	1.16E-05	0.000118
200071_PMSMNDC1	0.388536	1.16E-05	0.000118
11722436_GOSR2	0.423546	1.17E-05	0.000118
11720141_HMMR	-0.45763	1.17E-05	0.000118
11754798_CD164	0.38491	1.17E-05	0.000118
11722261_EDEM1	0.568676	1.17E-05	0.000118
11728698_HSPA4L	0.506395	1.17E-05	0.000118
11744868_SMC2	-0.53785	1.17E-05	0.000118
11720042_RRP7A ///	-0.51306	1.17E-05	0.000118
11717608_VMA21	-0.4227	1.17E-05	0.000118
11748560_PAFAH1B1	0.399677	1.17E-05	0.000118
11718329_NAPB	0.773814	1.17E-05	0.000118
11732377_BROX	0.522597	1.17E-05	0.000118
11722565_GOLPH3	-0.40531	1.17E-05	0.000118

11754957_HDAC6	-0.6212	1.17E-05	0.000118
11722790_RAB5A	0.368706	1.17E-05	0.000118
11745899_YY1AP1	0.429287	1.17E-05	0.000118
11733451_SLFN11	-0.41915	1.17E-05	0.000118
11716344_ZCCHC3	0.526129	1.17E-05	0.000118
11745667_ANO6	0.405523	1.17E-05	0.000118
11715883_DAP3	0.351468	1.17E-05	0.000118
11718516_AK6 /// T	-0.92341	1.17E-05	0.000118
11734642_BDNF	0.669283	1.17E-05	0.000118
11729793_MAN1A2	0.48505	1.17E-05	0.000118
11727839_ORC1	-0.60506	1.17E-05	0.000118
11719258_TICAM1	0.511217	1.17E-05	0.000118
11718233_PRPF4B	0.379657	1.17E-05	0.000118
11726119_RPGR	0.632422	1.17E-05	0.000118
11730841_ZMYND8	-0.45955	1.18E-05	0.000118
11755017_CHCHD7	-0.37847	1.18E-05	0.000119
11735198_FAM104B	-0.57614	1.18E-05	0.000119
11722807_WDR54	-0.42433	1.18E-05	0.000119
11757773_ZFAS1	0.340043	1.19E-05	0.000119
11721935_DNAJC27	0.475763	1.19E-05	0.000119
11717220_NFIB	-0.44104	1.19E-05	0.000119
11715516_PSMD3	-0.39081	1.19E-05	0.000119
11749067_TRMT10B	0.631512	1.19E-05	0.00012
11727872_NHEJ1	-0.61197	1.19E-05	0.00012
11722339_DIAPH2	0.406793	1.19E-05	0.00012
11743522_KDM5A	0.382426	1.19E-05	0.00012
11737533_SAE1	-0.40438	1.19E-05	0.00012
11758859_RAB2A	-0.38469	1.19E-05	0.00012
11729496_FAM133A	-0.68284	1.20E-05	0.00012
11756564_AUP1	0.38612	1.20E-05	0.00012
11726704_BCL10	0.469144	1.20E-05	0.00012
11733835_TMEM9	-0.37578	1.20E-05	0.00012
11715383_PHB2	-0.37488	1.20E-05	0.00012
11733971_PCDH18	0.485119	1.20E-05	0.000121
11760519_NSD1	0.366772	1.20E-05	0.000121
11722164_RGS5	0.786611	1.20E-05	0.000121
11743353_STAT1	0.383198	1.21E-05	0.000121
11717737_PCYOX1	-0.61293	1.21E-05	0.000121
11736813_PDCD1LG2	1.240966	1.21E-05	0.000121
11725574_NEDD4	0.408625	1.21E-05	0.000121
11727881_BBS12	0.438165	1.21E-05	0.000121
11763534_CNTNAP3	0.582015	1.21E-05	0.000121
11756424_NSMCE4A	-0.36678	1.21E-05	0.000121
11754287_CMTR1	-0.45191	1.21E-05	0.000121
11754609_STMN3	0.518745	1.21E-05	0.000121
11736236_RARA	0.627513	1.21E-05	0.000121
11719267_SERINC1	0.345224	1.21E-05	0.000121
11717040_MTMR4	-0.70782	1.21E-05	0.000121
11743807_GAS7	-0.5567	1.21E-05	0.000121
11746241_FAM133B /	0.496018	1.21E-05	0.000121
11720772_PPIF	0.419437	1.21E-05	0.000121

11747990_ZSCAN26	0.611522	1.22E-05	0.000121
11720622_CDK16	-0.46818	1.22E-05	0.000121
11718119_CFL2	0.369263	1.22E-05	0.000121
11719109_FHL3	0.379154	1.22E-05	0.000122
11725836_NFKBIB	0.520947	1.22E-05	0.000122
11737979_ZNF570	0.463822	1.22E-05	0.000122
11721989_FAM131A	-0.52367	1.23E-05	0.000122
11733148_ANXA4	0.400083	1.23E-05	0.000122
11717281_ASAP1	0.442858	1.23E-05	0.000122
11725096_RABL3	-0.84017	1.23E-05	0.000122
11735261_SDK1	0.686469	1.23E-05	0.000122
11743250_MMP2	0.453194	1.23E-05	0.000122
11720398_NBN	-0.41777	1.23E-05	0.000123
11715387_CDKN1A	0.426015	1.23E-05	0.000123
11747277_NAP1L4	-0.45913	1.24E-05	0.000123
11719508_CSRP2BP	-0.50824	1.24E-05	0.000123
11744619_SNX24	0.483659	1.24E-05	0.000123
11757820_DPY30	-0.38174	1.24E-05	0.000123
11743840_PRDM2	0.463213	1.24E-05	0.000123
11757578_RHOC	0.353585	1.24E-05	0.000123
11756289_CCM2	-0.41238	1.24E-05	0.000123
11741311_ZNF655	0.52595	1.24E-05	0.000123
11724055_SGOL2	-0.53296	1.24E-05	0.000124
11763804_THUMPD2	0.691792	1.24E-05	0.000124
11740896_MIR22HG	0.562608	1.24E-05	0.000124
11736169_NDEL1	0.43952	1.25E-05	0.000124
11743477_SLC25A13	0.422084	1.25E-05	0.000124
11753216_NARS	-0.42504	1.25E-05	0.000124
11739349_AMOT	0.378779	1.25E-05	0.000124
11746118_SYNCRIP	-0.67484	1.25E-05	0.000124
11720359_PHIP	-0.66263	1.25E-05	0.000124
11733990_ANGEL2	-0.35284	1.25E-05	0.000124
11752025_ZMYM2	0.476432	1.25E-05	0.000124
11741376_LRP8	-0.36448	1.26E-05	0.000125
11750085_TSC22D2	0.861084	1.26E-05	0.000125
11734509_KCTD8	0.490354	1.26E-05	0.000125
11725512_SLC38A4	0.542653	1.26E-05	0.000125
11739790_GCFC2	0.3942	1.26E-05	0.000125
11758602_THSD4	0.428537	1.26E-05	0.000125
11728872_RBM24	0.797154	1.26E-05	0.000125
11726639_MIB1	-0.6073	1.26E-05	0.000125
11732467_CXCL11	0.894383	1.26E-05	0.000125
11725424_RAB27A	-0.45637	1.26E-05	0.000125
11734030_FAM73A	0.557091	1.26E-05	0.000125
11755321_POLR1B	0.363937	1.26E-05	0.000125
11718427_HSDL2	-0.5098	1.27E-05	0.000125
11720219_MAP3K3	-0.58192	1.27E-05	0.000125
11754035_IRF1	0.790375	1.27E-05	0.000125
11740006_KIAA0895	0.796145	1.27E-05	0.000125
11758242_YAE1D1	0.450613	1.27E-05	0.000125
11734025_ZNF440	0.445464	1.27E-05	0.000125

11739641_AFAP1	-0.41087	1.27E-05	0.000125
11754429_LAMA2	0.336469	1.27E-05	0.000125
11739239_SNAPC3	0.433297	1.27E-05	0.000125
11748158_PRH1-PRR4	0.418524	1.27E-05	0.000125
11719236_INTS1	-0.36058	1.27E-05	0.000125
11728365_ARPP21	0.637727	1.27E-05	0.000125
11748208_ZNF317	0.54611	1.27E-05	0.000125
11755624_MTFR2	-0.51051	1.27E-05	0.000125
11719293_ITGB1BP1	-1.00154	1.27E-05	0.000125
11743969_ACTN1	0.420869	1.27E-05	0.000125
11728051_FAM126B	0.679123	1.28E-05	0.000126
11751107_VIPAS39	0.418425	1.28E-05	0.000126
11746847_NAPEPLD	0.686865	1.28E-05	0.000126
11728474_CLHC1	0.62023	1.28E-05	0.000126
11720237_MAPK9	-0.34566	1.28E-05	0.000126
11735263_SCN2A	1.965542	1.28E-05	0.000126
11721462_OSBPL2	-0.52565	1.28E-05	0.000126
11719918_PELO	0.346493	1.28E-05	0.000126
11754587_FYN	0.393644	1.28E-05	0.000126
11733750_BDNF	0.672112	1.29E-05	0.000127
11744776_NNMT	0.366535	1.29E-05	0.000127
11759018_MATR3	-0.42112	1.29E-05	0.000127
11742730_CAPS	-0.48314	1.29E-05	0.000127
11727506_RAB21	0.364521	1.29E-05	0.000127
11762632_ZFC3H1	1.057971	1.29E-05	0.000127
11757368_ANXA4	0.392455	1.29E-05	0.000127
11748523_PCGF5	1.092947	1.29E-05	0.000127
11754299_MXD4	0.437845	1.30E-05	0.000127
11745979_LIG1	-0.52962	1.30E-05	0.000127
11726328_GBP1	0.816242	1.30E-05	0.000127
11746842_ABI2	0.359234	1.30E-05	0.000127
11723078_PBDC1	0.388431	1.30E-05	0.000127
11757745_IGF1R	-0.58194	1.30E-05	0.000127
11730864_CTTNBP2NL	0.43657	1.30E-05	0.000128
11747735_LIMS1	0.374871	1.30E-05	0.000128
11720340_STX1A	0.449111	1.30E-05	0.000128
11746347_B3GAT3	0.437669	1.30E-05	0.000128
11721476_AP1S1	-0.44238	1.30E-05	0.000128
11735741_RASA3	0.351907	1.30E-05	0.000128
11759208_GPR176	0.336383	1.30E-05	0.000128
11755200_DCBLD2	0.389577	1.30E-05	0.000128
11718134_PRPS2	-0.36906	1.31E-05	0.000128
11756304_CSNK1A1	0.37223	1.31E-05	0.000128
11718367_ZZZ3	0.439198	1.31E-05	0.000128
11744886_SPAG9	0.501287	1.31E-05	0.000128
11755250_RPL13A	-0.36993	1.31E-05	0.000128
11743894_ZNF780A	0.827803	1.31E-05	0.000128
11716986_GTF2B	0.362797	1.31E-05	0.000128
11747223_EDNRB	0.656485	1.31E-05	0.000129
11756516_PISD	0.419317	1.31E-05	0.000129
11720045_DNAJC10	0.374771	1.31E-05	0.000129

11762067_FCF1	-0.4989	1.32E-05	0.000129
11734674_SAR1A	0.352898	1.32E-05	0.000129
11717753_SEC14L1	0.470804	1.32E-05	0.000129
11756410_KANSL2	0.375867	1.32E-05	0.000129
11745451_UBE2J2	0.350475	1.32E-05	0.000129
11755284_TSPAN13	-0.56071	1.32E-05	0.000129
11754939_FADS2	-0.45301	1.32E-05	0.000129
11743095_TMEM65	0.454166	1.32E-05	0.000129
11730933_AGPAT1	-0.34419	1.32E-05	0.000129
11757987_PATZ1	-0.38041	1.32E-05	0.000129
11719363_PRRX1	-0.47316	1.32E-05	0.000129
11721615_THEMIS2	-0.67396	1.32E-05	0.000129
11749482_LTBP3	0.375407	1.32E-05	0.000129
11758563_METTL21A	-1.32253	1.32E-05	0.000129
11752113_INPP4B	0.469245	1.32E-05	0.000129
11730312_AHSA2	0.364819	1.32E-05	0.000129
11719315_PIBF1	0.455999	1.32E-05	0.000129
11754024_SLC25A11	-0.52536	1.32E-05	0.000129
11720985_YRDC	0.420046	1.33E-05	0.000129
11754436_KIAA0754	0.410565	1.33E-05	0.000129
11754041_FXYD3	-0.45776	1.33E-05	0.000129
11752993_DUSP1	0.361367	1.33E-05	0.00013
11728375_ACTN1	0.343586	1.33E-05	0.00013
11724234_HGSNAT	-0.38504	1.34E-05	0.00013
11715802_PRRC2B	-0.48101	1.34E-05	0.00013
11734687_MBD2	0.358868	1.34E-05	0.00013
11723981_ARHGAP28	0.619319	1.34E-05	0.00013
11746555_SPARC	0.350307	1.34E-05	0.000131
11758450_KDM4D	-1.35229	1.34E-05	0.000131
11747479_MLST8	-0.40391	1.34E-05	0.000131
11739414_ZMYM2	0.399867	1.34E-05	0.000131
11717773_THRA	-0.62215	1.34E-05	0.000131
11733445_MMS22L	-0.75526	1.34E-05	0.000131
11724216_GPATCH2	0.400257	1.35E-05	0.000131
11722166_RGS5	0.820888	1.35E-05	0.000131
11716153_PAFAH1B1	0.436187	1.35E-05	0.000131
11754831_SRPK2	0.445227	1.35E-05	0.000131
11721767_ATAD3A //	-0.38452	1.35E-05	0.000132
11718447_SH3BGRL2	-0.66248	1.36E-05	0.000132
11747011_DCUN1D4	0.508901	1.36E-05	0.000132
11725500_WDR20	0.472044	1.36E-05	0.000132
11719587_MICALL1	-0.38057	1.36E-05	0.000132
11722799_SS18	0.504259	1.36E-05	0.000132
11746185_KIF5B	0.371592	1.36E-05	0.000132
11723997_NKIRAS1	0.38035	1.37E-05	0.000132
11739878_TNIK	0.587456	1.37E-05	0.000133
11718221_GSPT1	-1.30231	1.37E-05	0.000133
11745225_HN1L	-0.38441	1.37E-05	0.000133
11755322_MBD1	0.358084	1.37E-05	0.000133
11739799_LY6K	-0.50474	1.37E-05	0.000133
11753325_FANCA	-0.3657	1.38E-05	0.000133

11733312_PCDHA1 //	0.622682	1.38E-05	0.000133
11716163_CIAPIN1	-0.48533	1.38E-05	0.000134
11715428_ARF4	0.355792	1.38E-05	0.000134
11749178_GALK2	0.572232	1.38E-05	0.000134
11716199_UBE2Q2	0.380599	1.38E-05	0.000134
11739174_SNAP23	0.405886	1.38E-05	0.000134
11723885_NATD1	-0.43031	1.38E-05	0.000134
11748131_LGALS8	0.436029	1.39E-05	0.000134
11746461_TACC3	-0.52325	1.39E-05	0.000134
11717275_COL5A1	0.921229	1.39E-05	0.000134
11720269_IBTK	0.378609	1.39E-05	0.000134
11720383_STX6	0.427396	1.39E-05	0.000134
11744352_RHOC	0.343076	1.39E-05	0.000134
11721264_GRIA2	0.526159	1.39E-05	0.000135
11725947_MPP2	-0.51661	1.40E-05	0.000135
11742197_NF2	0.420626	1.40E-05	0.000135
11732376_TBC1D19	0.805072	1.40E-05	0.000135
11716734_IRF1	0.491141	1.40E-05	0.000135
11722431_C1orf27	0.34552	1.40E-05	0.000135
11723909_ESPL1	-0.38744	1.40E-05	0.000135
11750823_PREB	-0.46624	1.40E-05	0.000135
11756491_SDAD1	0.361878	1.40E-05	0.000135
11743687_HMGB2	-0.43329	1.40E-05	0.000135
11752289_MID1	0.566269	1.40E-05	0.000135
11717274_COL5A1	0.514569	1.40E-05	0.000135
11724215_SPRYD4	-0.58486	1.40E-05	0.000135
11736489_MXD4	0.366521	1.40E-05	0.000135
11748186_SLC1A4	-0.38733	1.40E-05	0.000135
11753017_HSPD1 ///	-0.35316	1.40E-05	0.000135
11746873_MFSD8	0.464217	1.40E-05	0.000135
11726766_TIPRL	-0.42705	1.40E-05	0.000135
11758858_RAB2A	-0.66417	1.41E-05	0.000135
11715872_ZFAND3	0.443452	1.41E-05	0.000135
11751581_SLF1	-0.62758	1.41E-05	0.000135
11756026_DBI	-0.36377	1.41E-05	0.000135
11717246_LSM4	-0.34555	1.41E-05	0.000135
11751348_NDFIP2	0.364406	1.41E-05	0.000135
11747055_GFRA1	0.584327	1.41E-05	0.000135
11723992_PDCL	-0.4425	1.41E-05	0.000135
11723226_SGCB	-0.41718	1.41E-05	0.000135
11736526_CARD9	0.618464	1.41E-05	0.000135
11747506_NAP1L4	-0.41299	1.41E-05	0.000136
11739272_PAK2	-0.42303	1.41E-05	0.000136
11731876_C5orf58	0.756592	1.41E-05	0.000136
11722938_PKN3	-0.61669	1.41E-05	0.000136
11722155_SGMS2	0.765495	1.41E-05	0.000136
11752468_PHC1	-0.41344	1.42E-05	0.000136
11719796_PPP2R5D	-0.45876	1.42E-05	0.000136
11752067_MAP4K4	0.458197	1.42E-05	0.000136
11755922_ARHGEF28	0.621536	1.42E-05	0.000136
11755702_RRP7A	-0.51403	1.42E-05	0.000136

11747147_PPP2R1B	0.34728	1.42E-05	0.000136
11746841_ABI2	0.388352	1.42E-05	0.000136
11718581_GPN2	0.424152	1.43E-05	0.000137
11727577_TMEM237	-0.55972	1.43E-05	0.000137
11752842_FSTL1	0.446575	1.43E-05	0.000137
11744934_CCSER2	0.399264	1.43E-05	0.000137
11722229_PPA2	-0.37447	1.44E-05	0.000137
11717827_DBI	-0.39907	1.44E-05	0.000138
11720791_RHOC	0.345705	1.44E-05	0.000138
11730383_SNX18	0.398196	1.44E-05	0.000138
11730146_KLHL31	0.398156	1.44E-05	0.000138
11726705_USP8	0.425232	1.44E-05	0.000138
11716232_CTNNBIP1	-0.35187	1.45E-05	0.000138
11733842_SSSCA1	-0.47171	1.45E-05	0.000138
11719101_SENP6	0.422111	1.45E-05	0.000138
11755621_DUSP9	-0.55882	1.45E-05	0.000138
11726540_NGLY1	0.373559	1.45E-05	0.000138
11721367_WDR83	-0.48357	1.45E-05	0.000138
11743930_UHRF1BP1L	0.634114	1.45E-05	0.000138
11756919_CAP2	0.582737	1.45E-05	0.000139
11722169_CSE1L	-0.34739	1.45E-05	0.000139
11740175_ARSK	0.525329	1.45E-05	0.000139
11742718_SLTM	0.415606	1.45E-05	0.000139
11750656_LOC441155	0.486458	1.45E-05	0.000139
11730970_THAP2	1.130866	1.45E-05	0.000139
11737757_CARS	0.342223	1.46E-05	0.000139
11716022_ADRBK1	-0.55992	1.46E-05	0.000139
11725945_TINAGL1	0.464106	1.46E-05	0.000139
11748995_MYO1C	0.434578	1.46E-05	0.000139
11736304_MIR22HG	0.454614	1.46E-05	0.000139
11743348_CD47	0.347081	1.46E-05	0.000139
11724613_DYRK2	0.431851	1.46E-05	0.000139
11717360_GCN1	-0.35215	1.46E-05	0.000139
11723141_ZBTB11	0.349822	1.46E-05	0.000139
11736613_RIC1	0.757414	1.46E-05	0.000139
11736517_WHSC1	-0.38363	1.46E-05	0.000139
11727094_TMEM19	-0.51794	1.46E-05	0.000139
11749640_TNKS	0.37816	1.47E-05	0.000139
11747101_CNOT4	0.697849	1.47E-05	0.000139
11747454_ACTN4	-0.39251	1.47E-05	0.00014
11759203_ZNF223	0.45402	1.47E-05	0.00014
11745357_AP1S1	-0.41379	1.47E-05	0.00014
11716430_SLC39A13	-0.36046	1.47E-05	0.00014
11728369_CCP110	0.580649	1.47E-05	0.00014
11747796_LIG1	-0.46519	1.47E-05	0.00014
11748156_TIA1	0.412368	1.47E-05	0.00014
11727034_PAPSS2	-0.3636	1.47E-05	0.00014
11737986_ARHGAP11A	-0.34345	1.47E-05	0.00014
11763351_LOC286052	-0.46978	1.47E-05	0.00014
11747063_LMBRD2	0.795879	1.47E-05	0.00014
11756988_TWF2	-0.38797	1.48E-05	0.00014

11735636_ZMYM5	0.652628	1.48E-05	0.00014
11745191_TRIP6	0.369733	1.48E-05	0.00014
11748452_ARHGEF39	-0.4946	1.48E-05	0.00014
11731516_XPO4	0.444453	1.48E-05	0.00014
11724988_CCDC113	0.426569	1.48E-05	0.00014
11751716_RBM18	0.35987	1.48E-05	0.000141
11756447_GORAB	0.360316	1.48E-05	0.000141
11761846_ANXA4	0.402224	1.48E-05	0.000141
11741050_IMPDH1	-0.54486	1.48E-05	0.000141
11729618_UBE2J2	0.384523	1.49E-05	0.000141
11720952_DCUN1D4	0.424499	1.49E-05	0.000141
11722532_CALD1	0.448292	1.49E-05	0.000141
11751301_POGLUT1	0.653795	1.49E-05	0.000141
11745330_ATP6V1D	0.341574	1.49E-05	0.000141
11763449_ZBTB20	0.656174	1.49E-05	0.000141
11724017_OSCP1	0.750628	1.49E-05	0.000141
11758487_MPZ	0.670576	1.50E-05	0.000141
11725094_TTC33	-0.54257	1.50E-05	0.000141
11740789_CDH12	0.431636	1.50E-05	0.000141
11756832_EGR2	0.565422	1.50E-05	0.000142
11746827_DNAAF5	-0.37	1.50E-05	0.000142
11759192_MAPK8	0.446083	1.50E-05	0.000142
11717999_MPLKIP	-0.45212	1.50E-05	0.000142
11735512_PHTF2	0.466113	1.50E-05	0.000142
11746573_RHOB	0.444135	1.50E-05	0.000142
11729258_MNS1	-0.48719	1.50E-05	0.000142
11758379_SLC25A46	0.381331	1.51E-05	0.000142
11728401_ATP6V0A2	0.371115	1.51E-05	0.000142
11757106_SVIL	0.407559	1.51E-05	0.000142
11746238_RBM14-RBM	0.354343	1.51E-05	0.000142
11748638_BTRC	0.586063	1.51E-05	0.000142
11722119_LZTS1	-0.59036	1.51E-05	0.000142
11718648_ACVR1	-0.39556	1.51E-05	0.000142
11742191_LOC100506	-0.38126	1.51E-05	0.000143
11729391_MIER1	0.385739	1.51E-05	0.000143
11723124_MORC3	0.569612	1.51E-05	0.000143
11744622_STK25	-0.43561	1.52E-05	0.000143
11723232_RNF138	0.346281	1.52E-05	0.000143
11719516_SLC9A6	0.376052	1.52E-05	0.000143
11727871_PPM1K	0.667072	1.52E-05	0.000143
11750762_DHX40	-0.39257	1.52E-05	0.000143
11743728_CCDC47	0.405766	1.52E-05	0.000143
11754984_MYH10	-0.34336	1.53E-05	0.000144
11734135_NANOS1	0.598828	1.53E-05	0.000144
11735019_PPM1B	0.529235	1.53E-05	0.000144
11757723_CD163L1	0.886182	1.53E-05	0.000144
11754308_SEPHS1	-0.44232	1.53E-05	0.000144
11759625_PWAR6	-0.48397	1.53E-05	0.000144
11716224_CKAP5	-0.33282	1.53E-05	0.000144
11754316_PIK3R3	0.426829	1.53E-05	0.000144
11750299_IL13RA1	0.512889	1.53E-05	0.000144

11746438_SHMT2	-0.41564	1.53E-05	0.000144
11734757_METTL6	0.339346	1.54E-05	0.000144
11741373_HIPK3	0.885936	1.54E-05	0.000144
11739731_CSNK1G1	0.437913	1.54E-05	0.000144
11718782_TMED1	-0.3355	1.54E-05	0.000145
11723089_CXorf40A	0.409994	1.54E-05	0.000145
11736931_FAM72A //	-0.39205	1.54E-05	0.000145
11718330_NAPB	0.480716	1.54E-05	0.000145
11721155_USP38	0.416478	1.54E-05	0.000145
11743521_KHSRP	-0.40106	1.54E-05	0.000145
11736740_EPC1	0.540457	1.55E-05	0.000145
11757386_NPM1	-0.36625	1.55E-05	0.000145
11736611_RIC1	0.630708	1.55E-05	0.000145
11721977_PLCE1	-0.59853	1.55E-05	0.000145
11744287_CBS /// C	-0.87096	1.55E-05	0.000145
11749773_GBP1	0.726856	1.55E-05	0.000145
11718725_ZNF655	0.497437	1.55E-05	0.000145
11736783_RAB11FIP1	0.656928	1.55E-05	0.000145
11750019_ATG4D	-0.40639	1.55E-05	0.000145
11756090_ZHX1	0.456732	1.55E-05	0.000146
11743714_ARID4A	0.504705	1.55E-05	0.000146
11754672_CHFR	0.514114	1.56E-05	0.000146
11726934_SOX12	-0.38075	1.56E-05	0.000146
11747861_SNRK	0.380936	1.56E-05	0.000146
11743053_CHORDC1	-0.37865	1.56E-05	0.000146
11729027_RNF175	-0.64764	1.56E-05	0.000146
11716234_CTNNBIP1	0.47147	1.56E-05	0.000146
11728497_SVIL	0.420949	1.56E-05	0.000146
11756573_RIMKLB	0.551172	1.56E-05	0.000146
11751337_DHCR24	-0.68403	1.56E-05	0.000146
11739787_IGF2 ///	0.604954	1.56E-05	0.000146
11719993_NLK	0.496813	1.57E-05	0.000146
11724680_TMEM131	0.435482	1.57E-05	0.000147
11717466_NFAT5	0.560563	1.57E-05	0.000147
11718686_TXNRD2	0.410172	1.57E-05	0.000147
11743020_ZSCAN29	0.489289	1.57E-05	0.000147
11746625_GTF2H1	0.426058	1.57E-05	0.000147
11722026_TBL3	-0.45841	1.57E-05	0.000147
11719017_CBFB	0.390576	1.58E-05	0.000147
11718656_SLC16A1	-0.37571	1.58E-05	0.000147
11724505_MIS18A	-0.42697	1.58E-05	0.000147
11722830_PEX12	0.382232	1.58E-05	0.000147
11723432_ZFP91	0.364087	1.58E-05	0.000148
11743681_S100A16	0.366242	1.58E-05	0.000148
11719665_GFM1	0.416602	1.59E-05	0.000148
11719618_PEX5	-0.41848	1.59E-05	0.000148
11720774_DNAJC19	-0.38795	1.59E-05	0.000148
11760659_LIMCH1	0.47377	1.59E-05	0.000148
11731603_TMEM154	-0.62033	1.59E-05	0.000148
11745020_TNFRSF12A	0.65551	1.59E-05	0.000148
11755270_GANAB	-0.36772	1.59E-05	0.000148

11723091_FNIP2	0.453391	1.59E-05	0.000148
11732570_ZNF582	0.804764	1.59E-05	0.000148
11730790_NPM1	-0.33397	1.59E-05	0.000148
11722797_TMEM263	0.34059	1.59E-05	0.000148
11744673_SP100	0.598642	1.59E-05	0.000148
11721066_ADCY9	-0.66306	1.59E-05	0.000148
11717367_ZC3HAV1	0.380462	1.60E-05	0.000148
11719690_TWSG1	-0.61153	1.60E-05	0.000148
11737168_STRN3	0.424865	1.60E-05	0.000149
11758401_ZNF148	-0.69629	1.60E-05	0.000149
11716178_C5orf15	0.356832	1.60E-05	0.000149
11727090_ZBTB18	0.543864	1.60E-05	0.000149
11744887_ZWILCH	-0.414	1.60E-05	0.000149
11726267_KDM4C	0.511135	1.61E-05	0.000149
11747579_SENP2	-0.35838	1.61E-05	0.000149
11716198_UBE2Q2	0.418199	1.61E-05	0.00015
11752964_SLC25A20	0.412224	1.62E-05	0.00015
11726386_PDZD8	0.584781	1.62E-05	0.00015
11716465_NIF3L1	-0.41547	1.62E-05	0.00015
11718161_KLF13	-0.8436	1.62E-05	0.00015
11736452_SRFBP1	0.456354	1.62E-05	0.00015
11744629_MGC27345	-0.53019	1.62E-05	0.00015
11753489_EED	-0.38452	1.62E-05	0.00015
11744425_KIAA0101	-0.58834	1.62E-05	0.00015
11733359_CFAP45	0.787353	1.62E-05	0.00015
11739571_ASH1L	0.415779	1.62E-05	0.00015
11750036_DDIAS	-0.49963	1.63E-05	0.000151
11716653_EML4	0.621759	1.63E-05	0.000151
11730752_AGPAT5	0.361641	1.63E-05	0.000151
11760451_ZNF484	0.82177	1.63E-05	0.000151
11732250_RCOR3	0.437668	1.63E-05	0.000151
11743479_PIN4	-0.4466	1.63E-05	0.000151
11764014_GART	0.480211	1.63E-05	0.000151
11717868_ZNF263	0.379967	1.64E-05	0.000151
11718184_FCHSD2	-0.35294	1.64E-05	0.000151
11717606_RNPS1	-0.3497	1.64E-05	0.000151
11752412_PPP2R5D	-0.44517	1.64E-05	0.000151
11752121_ZNF324	0.416138	1.64E-05	0.000151
11715261_HNRNPCL2	-0.41756	1.64E-05	0.000151
11755640_RALGPS1	-0.35932	1.64E-05	0.000151
11719747_NF2	-0.5128	1.64E-05	0.000152
11757353_FBXO25	0.537462	1.64E-05	0.000152
11752779_BGN	-0.43587	1.64E-05	0.000152
11742195_ZNF451	0.419307	1.64E-05	0.000152
11751927_COPA	0.332205	1.64E-05	0.000152
11721901_NMRAL1	-0.51379	1.65E-05	0.000152
11757649_CORO7-PAM	-0.43766	1.65E-05	0.000152
11754389_LRRC57	0.475312	1.65E-05	0.000152
11724918_TMTC3	0.465074	1.65E-05	0.000152
11750684_DDX31	0.492222	1.65E-05	0.000152
11743629_IRS1	0.492902	1.66E-05	0.000153

11745015_SP100	0.42992	1.66E-05	0.000153
11717460_CHPF2	0.398482	1.66E-05	0.000153
11739530_PLGLB2	0.509544	1.66E-05	0.000153
11728687_SFXN1	0.391679	1.66E-05	0.000153
11730363_HRH1	1.036797	1.66E-05	0.000153
11732524_CHKB-CPT1	0.433799	1.66E-05	0.000153
11724337_NRCAM	0.405161	1.66E-05	0.000153
11718840_PDHX	0.343197	1.66E-05	0.000153
11747152_PHLDB2	0.508861	1.66E-05	0.000153
11723699_OAS2 ///	-0.45337	1.66E-05	0.000153
11755774_FAM208B	0.356635	1.66E-05	0.000153
11756897_TJP2	0.405499	1.66E-05	0.000153
11744169_MYBL2	-0.61242	1.66E-05	0.000153
11743695_KMT2D	-0.36444	1.66E-05	0.000153
11719012_FZD4	-0.62124	1.67E-05	0.000153
11754671_NFYC	-0.45205	1.67E-05	0.000153
11736771_SCRN1	0.492292	1.67E-05	0.000153
11720674_PPM1A	-0.4395	1.67E-05	0.000153
11749957_REEP5	0.416681	1.67E-05	0.000153
11757302_GMPS	-0.37287	1.67E-05	0.000154
11721996_PARP14	0.51474	1.67E-05	0.000154
11747711_LDB1	0.369667	1.68E-05	0.000154
11757762_IPPK	0.487987	1.68E-05	0.000154
11743649_DCAF6	0.331688	1.68E-05	0.000154
11740586_PTGFR	0.369849	1.68E-05	0.000154
11739193_SLMAP	0.346978	1.68E-05	0.000154
11739864_TTF2	-0.51287	1.68E-05	0.000154
11727813_SRSF1	-0.53599	1.68E-05	0.000154
11731007_MIS18BP1	-0.57595	1.68E-05	0.000154
11740903_PTCH1	-0.4272	1.68E-05	0.000154
11716583_DYNLT1	0.36116	1.68E-05	0.000154
11725714_RPS14	-0.41265	1.68E-05	0.000154
11716648_DHX40	-0.41337	1.69E-05	0.000154
11743156_POFUT1	-0.48515	1.69E-05	0.000155
11726858_TAF5	-0.35638	1.69E-05	0.000155
11716317_CYTH2	0.458863	1.69E-05	0.000155
11742809_BCL2L13	0.3782	1.69E-05	0.000155
11724173_SDSL	0.481225	1.69E-05	0.000155
11739282_TRIM23	0.497431	1.69E-05	0.000155
11725134_GCOM1 ///	0.385231	1.69E-05	0.000155
11723019_SUSD2	0.51216	1.69E-05	0.000155
11718220_HNRNPAB	-0.35466	1.70E-05	0.000156
11716472_RAF1	0.385448	1.70E-05	0.000156
11738081_ZNF235	0.571042	1.71E-05	0.000156
11757894_NFKBIA	0.392167	1.71E-05	0.000156
11728910_ZNF615	0.507056	1.71E-05	0.000156
11718875_PNMA1	-0.3579	1.71E-05	0.000156
11723170_FOXN2	0.818576	1.71E-05	0.000156
11758273_ARF6	-0.38118	1.71E-05	0.000156
11736335_DEDD	0.342357	1.71E-05	0.000156
11754853_PDZK1 ///	0.760996	1.71E-05	0.000156

11717645_GPR107	0.605122	1.71E-05	0.000156
11756063_SRSF5	-0.32701	1.71E-05	0.000156
11739073_RPL7L1	-0.6889	1.72E-05	0.000157
11715609_ATP6V1H	0.375149	1.72E-05	0.000157
11720486_SENP2	-0.36146	1.72E-05	0.000157
11752843_SQSTM1	0.371719	1.72E-05	0.000157
11748147_MRPL2	-0.58475	1.72E-05	0.000157
11757292_LOC101060	0.511347	1.72E-05	0.000157
11744264_SEC11C	0.362794	1.72E-05	0.000157
11753027_PLEKHB2	0.426955	1.73E-05	0.000157
11730074_INTU	1.012784	1.73E-05	0.000158
11732302_CREB1	-0.5426	1.73E-05	0.000158
11758119_TMEM41B	-0.41979	1.73E-05	0.000158
11732274_ELMOD2	0.426418	1.73E-05	0.000158
11747220_COPG2	-0.69995	1.73E-05	0.000158
11754814_NUP93	-0.38937	1.73E-05	0.000158
11736438_DEDD	0.34791	1.73E-05	0.000158
11742120_NFYC	-0.39998	1.74E-05	0.000158
11726010_CBR4	-0.37886	1.74E-05	0.000158
11731137_MYPN	1.258441	1.74E-05	0.000158
11715801_PRRC2B	-0.37965	1.74E-05	0.000158
11738155_CELF1	0.523718	1.74E-05	0.000158
11750709_ZNF33B	0.649745	1.74E-05	0.000159
11732095_TTC8	0.475805	1.74E-05	0.000159
11729216_PIGX	-0.65922	1.74E-05	0.000159
11759578_RASSF8	-0.34799	1.75E-05	0.000159
11717013_SNRPF	-0.48799	1.75E-05	0.000159
11717162_SLC29A1	-0.63831	1.75E-05	0.000159
11755951_PRMT5	-0.34872	1.75E-05	0.000159
11728678_HNRNPD	-0.40339	1.75E-05	0.000159
11721904_CCDC28B	0.433309	1.75E-05	0.000159
11749813_NEK2	-0.6801	1.75E-05	0.000159
11760228_KLHL23 //	-0.64919	1.75E-05	0.000159
11748629_IFRD1	0.354104	1.76E-05	0.000159
11716628_KIF3B	0.4114	1.76E-05	0.000159
11764160_LINC01605	0.639991	1.76E-05	0.000159
11730164_TRIM37	0.346809	1.76E-05	0.000159
11750279_STC1	0.55513	1.76E-05	0.000159
11755483_PPP1R21	0.481277	1.76E-05	0.000159
11719691_TWSG1	-0.58268	1.76E-05	0.000159
11728907_B3GLCT	-0.58176	1.76E-05	0.000159
11718118_CFL2	0.353062	1.76E-05	0.000159
11741076_CASP1	0.44818	1.76E-05	0.000159
11754985_ZNF180	0.500446	1.76E-05	0.000159
11728649_NUCKS1	-0.33805	1.76E-05	0.000159
11755444_MCOLN3	0.508448	1.76E-05	0.000159
11724815_MED13L	0.430709	1.76E-05	0.00016
11756732_TSPAN12	0.862903	1.77E-05	0.00016
11732396_IFT80	0.390937	1.77E-05	0.00016
11715776_HSPB8	0.635608	1.77E-05	0.00016
11749632_BRCA1	-0.5987	1.77E-05	0.00016

11749061_STOM	0.36691	1.77E-05	0.00016
11724054_SGOL2	-0.55195	1.77E-05	0.00016
11745551_KLHDC3	0.41934	1.78E-05	0.00016
11732216_PEF1	-0.42049	1.78E-05	0.000161
11758916_YY1	-0.58923	1.78E-05	0.000161
11734907_ZNF432	0.62119	1.78E-05	0.000161
11721495_WDR33	-0.48773	1.78E-05	0.000161
11735320_RBMS3	0.546661	1.78E-05	0.000161
11732196_KPNA4	0.412645	1.78E-05	0.000161
11746293_KLHL20	0.474793	1.78E-05	0.000161
11748594_RABEP1	0.36706	1.79E-05	0.000161
11733701_FKBP11	-0.39036	1.79E-05	0.000162
11721315_FBXL5	0.325021	1.79E-05	0.000162
11739594_RBM12	-0.94878	1.80E-05	0.000162
11728749_HCG22 ///	-0.55431	1.80E-05	0.000162
11745212_ZNF33A //	0.557769	1.80E-05	0.000162
11746995_NPL	-0.72756	1.80E-05	0.000162
11718789_ZNF302	0.349042	1.80E-05	0.000162
11729156_SLC25A32	0.345712	1.81E-05	0.000163
11718366_FDPS	-0.41053	1.81E-05	0.000163
11716518_VPS37B	-0.35909	1.81E-05	0.000163
11748975_CWF19L1	-0.47107	1.81E-05	0.000163
11743404_ZMAT2	-0.38489	1.81E-05	0.000163
11757482_POLD2	-0.36116	1.81E-05	0.000163
11727580_CSTF3	-0.39925	1.82E-05	0.000163
11755384_OSBP	-0.38963	1.82E-05	0.000164
11722818_GGH	-0.5022	1.82E-05	0.000164
11730786_KLHL20	0.451219	1.82E-05	0.000164
11748481_TUBB	-0.32308	1.82E-05	0.000164
11719367_PLEKHO2	-0.34757	1.82E-05	0.000164
11759087_NHLRC2	0.400482	1.82E-05	0.000164
11715631_LAPTM4A	-0.35041	1.82E-05	0.000164
11748838_C20orf27	-0.36576	1.82E-05	0.000164
11745904_UQCC2	-0.39161	1.82E-05	0.000164
11730301_LIAS	-0.56036	1.82E-05	0.000164
11762499_POLQ	-0.64641	1.83E-05	0.000164
11729222_GATA3	0.405152	1.83E-05	0.000164
11756209_SHMT1	-0.47042	1.83E-05	0.000164
11716662_SUSD6	0.460466	1.83E-05	0.000164
11757291_ISCU	0.350099	1.83E-05	0.000164
AFFX-HUMISTAT1	0.387039	1.83E-05	0.000164
11757301_SDCBP	0.319847	1.83E-05	0.000164
11759174_USP14	-0.4381	1.83E-05	0.000164
11754908_ILF3	-0.35079	1.83E-05	0.000164
11717482_PDHA1	-0.36118	1.83E-05	0.000165
11737428_MSR1	0.511535	1.84E-05	0.000165
11718511_UBE2L3	-0.69922	1.84E-05	0.000165
11728223_UBE2J1	-0.44668	1.84E-05	0.000165
11725333_MZT1	-0.32972	1.84E-05	0.000165
11716294_CARS	0.362474	1.84E-05	0.000165
11726834_DNAJC21	0.401784	1.85E-05	0.000165

11724381_FAM65B	0.547382	1.85E-05	0.000166
11715270_KLF7	0.570477	1.85E-05	0.000166
11729751_LPAR1	0.615502	1.85E-05	0.000166
11756937_DDI2 ///	0.667675	1.85E-05	0.000166
11744743_TSHZ1	-0.42904	1.86E-05	0.000166
11724213_LMAN1	-0.44617	1.86E-05	0.000166
11721815_FCMR	0.675089	1.86E-05	0.000166
11715384_PHB2	-0.38459	1.86E-05	0.000166
11727687_KCTD18	-0.81957	1.86E-05	0.000166
11718496_REEP4	-0.34401	1.86E-05	0.000166
11746229_RPL31	-0.44224	1.86E-05	0.000166
11745242_TNFRSF21	-0.4719	1.86E-05	0.000166
11755072_ICAM3	-0.43252	1.86E-05	0.000166
11745507_ 7-Mar	0.342558	1.86E-05	0.000166
11752735_NARS	-0.35876	1.86E-05	0.000166
11749573_ARRB2	-0.44418	1.86E-05	0.000167
11723121_TRPM7	0.431772	1.87E-05	0.000167
11757710_KHSRP	-0.3636	1.87E-05	0.000167
11717231_EP400	-0.46568	1.87E-05	0.000167
11723161_ZNF776	0.388432	1.87E-05	0.000167
11739549_LOC102724	0.533138	1.88E-05	0.000168
11750198_CASP7	-0.45972	1.88E-05	0.000168
11720866_AGO1 ///	0.378371	1.88E-05	0.000168
11725219_SLK	0.323528	1.88E-05	0.000168
11728371_DNAJC16	0.483352	1.88E-05	0.000168
11716773_SORBS3	-0.52714	1.88E-05	0.000168
11741127_CANX	-0.38293	1.88E-05	0.000168
11743383_PRPF40A	-0.46417	1.89E-05	0.000168
11716258_RHEB	-0.3669	1.89E-05	0.000168
11731589_ZNF576	-0.37559	1.89E-05	0.000168
11759202_CARNMT1	-0.52385	1.89E-05	0.000168
11734196_SAMD4B	-0.3841	1.89E-05	0.000168
11728315_ZNF148	-0.48866	1.89E-05	0.000169
11722887_EMB	0.388014	1.90E-05	0.000169
11747721_CXorf40A	0.330805	1.90E-05	0.000169
11723883_SOWAHC	0.651072	1.90E-05	0.000169
11715705_MGEA5	0.390868	1.90E-05	0.000169
11715998_CAB39	0.368586	1.90E-05	0.000169
11720938_TIMM17B	-0.38061	1.90E-05	0.000169
11731043_ERLIN2	0.465596	1.90E-05	0.000169
11716783_TBL1XR1	-0.40263	1.90E-05	0.000169
11752755_MTRF1	-0.3834	1.91E-05	0.00017
11728612_ADAMTS6	0.577739	1.91E-05	0.00017
11744258_ATP1B3	-0.37376	1.91E-05	0.00017
11715879_PITPNA	-0.46655	1.91E-05	0.00017
11740004_SPATA5	-0.36035	1.91E-05	0.00017
11763444_SNAPC4	0.493395	1.91E-05	0.00017
11751060_GPR89A //	0.494235	1.91E-05	0.00017
11741168_MSRB3	0.690703	1.91E-05	0.00017
11728393_BLOC1S5 /	-0.36514	1.91E-05	0.00017
11737961_RCOR3	0.455871	1.91E-05	0.00017

11744069_LSG1	0.358206	1.91E-05	0.00017
11758398_LYSMD3	0.350355	1.92E-05	0.00017
11745313_SYNRG	0.464386	1.92E-05	0.00017
11724873_NFYC	-0.42268	1.92E-05	0.00017
11736329_ACOT9	0.389661	1.92E-05	0.00017
11755466_CDCA2	-0.34283	1.92E-05	0.00017
11741189_NKIRAS2	0.431729	1.92E-05	0.00017
11721727_TAF6	-0.35048	1.92E-05	0.00017
11761560_TRMT13	0.351698	1.92E-05	0.00017
11739972_NUAK1	0.709047	1.92E-05	0.00017
11739116_EFR3A	0.584302	1.92E-05	0.00017
11731978_PCSK7	0.349246	1.92E-05	0.00017
11757579_RYBP	0.459474	1.92E-05	0.00017
11757737_RBPJ	-0.35487	1.92E-05	0.00017
11746324_SH3GLB1	0.42475	1.92E-05	0.00017
11739653_BIRC5	-0.43569	1.93E-05	0.00017
11720605_POLD2	-0.35165	1.93E-05	0.000171
11734300_INTS6	0.746772	1.93E-05	0.000171
11720109_CREB3L4	-0.57938	1.93E-05	0.000171
11753568_SNAP23	0.36273	1.93E-05	0.000171
11741400_STMN1	-0.32899	1.93E-05	0.000171
11721460_TAPBPL	0.658634	1.93E-05	0.000171
11741213_SSU72	-0.38172	1.94E-05	0.000171
11748111_MAPKAP1	-0.71941	1.94E-05	0.000171
11716054_LAMC1	0.392774	1.94E-05	0.000171
11756431_TXNIP	-0.44474	1.94E-05	0.000171
11740680_RHCE ///	-0.66682	1.94E-05	0.000171
11724299_DDX31	0.38628	1.94E-05	0.000171
11747245_UTP15	0.423993	1.94E-05	0.000171
11743377_NIN	-0.47123	1.94E-05	0.000171
11753919_IDE	0.372739	1.94E-05	0.000172
11758045_KIAA1804	-0.52493	1.95E-05	0.000172
11720628_ATP6V1D	0.383877	1.95E-05	0.000172
11723355_CHD7	0.426685	1.95E-05	0.000172
11751244_CNN2	0.361647	1.95E-05	0.000172
11741313_CLCC1	0.331888	1.95E-05	0.000172
11719085_SMARCC2	-0.38243	1.95E-05	0.000172
11760503_RASSF8	-0.33795	1.95E-05	0.000172
11728383_SNAPC4	0.516081	1.95E-05	0.000172
11720241_KIF4A	-0.57864	1.95E-05	0.000172
11730732_DNASE2	-0.35811	1.95E-05	0.000172
11746058_ZNF410	0.326646	1.95E-05	0.000172
11728321_NSL1	-0.54845	1.95E-05	0.000172
11717106_PSMB5	-0.3637	1.95E-05	0.000172
11737506_NOCT	0.699933	1.95E-05	0.000172
11721245_BGN	-0.40577	1.95E-05	0.000172
11715553_IMMT	-0.3377	1.96E-05	0.000172
11757481_COPS6	-0.37654	1.96E-05	0.000172
11727530_PHF20L1	0.333659	1.96E-05	0.000172
11726661_GPN3	-0.39642	1.96E-05	0.000172
11720530_JAZF1	0.775199	1.96E-05	0.000172

11722440_SEMA4F	-0.53987	1.96E-05	0.000172
11747763_ADD1	-0.46682	1.96E-05	0.000172
11749856_ANTXR2	0.366917	1.96E-05	0.000173
11749495_SLC23A2	0.421903	1.97E-05	0.000173
11756799_TOR1AIP1	-0.33886	1.97E-05	0.000173
11718058_TYMS	-0.32497	1.97E-05	0.000173
11748416_DCAF6	0.398539	1.97E-05	0.000173
11740481_PAQR5	-0.62538	1.97E-05	0.000173
11720054_CCNH	0.4315	1.97E-05	0.000173
11717720_ST6GAL1	-0.32773	1.98E-05	0.000174
200054_PMZPR1	0.46556	1.98E-05	0.000174
11743259_PHF13	-0.35875	1.99E-05	0.000174
11748790_SS18	0.424736	1.99E-05	0.000175
11742772_LSM14A	-0.32241	1.99E-05	0.000175
11729310_SKA3	-0.38327	1.99E-05	0.000175
11754780_ZNF767P	0.45584	1.99E-05	0.000175
11748355_SLC16A1	-0.39862	1.99E-05	0.000175
11730729_TAP1	0.505418	1.99E-05	0.000175
11744694_BIRC2	0.336702	2.00E-05	0.000175
11739041_GTF2H1	0.356894	2.00E-05	0.000175
11757587_SLC39A6	-0.38669	2.00E-05	0.000175
11746839_SKIV2L	-0.38831	2.00E-05	0.000175
11733613_TCOF1	-0.67051	2.00E-05	0.000175
11722162_PLK2	0.357875	2.00E-05	0.000175
11739567_FKBP5	-0.41567	2.00E-05	0.000175
11729188_MAX	0.400148	2.01E-05	0.000175
11745334_LATS1	0.666219	2.01E-05	0.000176
11747073_ACTN4	-0.38454	2.01E-05	0.000176
11743165_ETV6	0.501439	2.01E-05	0.000176
11728805_CADM1	0.571324	2.01E-05	0.000176
11725975_POM121	-0.42558	2.01E-05	0.000176
11722093_PPIC	0.52789	2.01E-05	0.000176
11720966_DCK	-0.34423	2.01E-05	0.000176
11733923_CROT	0.664661	2.01E-05	0.000176
11726690_STAT1	0.382314	2.01E-05	0.000176
11721758_TMEM47	0.393071	2.01E-05	0.000176
11716947_TM9SF3	0.346402	2.02E-05	0.000176
11720742_ZNF605	0.603405	2.02E-05	0.000176
11727488_HAUS6	-0.59655	2.02E-05	0.000176
11718219_HNRNPAB	-0.34561	2.02E-05	0.000176
11726702_AFTPH	0.518268	2.02E-05	0.000176
11725192_TTPAL	-0.36324	2.02E-05	0.000176
11748606_POLR1B	0.403482	2.02E-05	0.000176
11725771_MIER3	0.389638	2.02E-05	0.000176
11743269_SRSF1	-0.49135	2.02E-05	0.000176
11723273_FEM1C	0.428951	2.02E-05	0.000176
11732899_LOC105369	0.355592	2.02E-05	0.000176
11736307_CSNK1A1	0.378621	2.02E-05	0.000176
11748972_CDC25B	-0.51518	2.02E-05	0.000176
11746099_PPP4R1	0.446209	2.03E-05	0.000176
11719964_UBE2E2	0.314669	2.03E-05	0.000177

11724798_MAGOH	-0.42482	2.03E-05	0.000177
11721504_CKLF ///	-0.35311	2.03E-05	0.000177
11722570_VEZT	0.541013	2.03E-05	0.000177
11719940_DSCR3	0.405489	2.03E-05	0.000177
11754887_MSRB3	0.462261	2.03E-05	0.000177
11754569_MZT2A	-0.34499	2.03E-05	0.000177
11729227_GRK5	0.42656	2.03E-05	0.000177
11732542_TRIM7	-0.73157	2.03E-05	0.000177
11717870_ZNF263	0.450047	2.03E-05	0.000177
11760834_MED6	0.512746	2.03E-05	0.000177
11733258_TAOK3	0.528266	2.03E-05	0.000177
11744376_MPI	-0.36073	2.04E-05	0.000177
11746115_ZNF35	0.663719	2.04E-05	0.000177
11746098_PPP4R1	0.481472	2.04E-05	0.000177
11727361_MYLK	0.486131	2.04E-05	0.000177
11730465_KCNJ2	0.673947	2.04E-05	0.000177
11726326_DCUN1D1	0.392081	2.04E-05	0.000177
11723789_FAM167A	0.348248	2.05E-05	0.000178
11722142_PITPNM1	-0.40505	2.05E-05	0.000178
11733827_C19orf48	-0.32898	2.05E-05	0.000178
11727988_SCML1	0.502937	2.05E-05	0.000178
11757464_PLXNB2	0.380848	2.05E-05	0.000178
11730863_CTTNBP2NL	0.597094	2.05E-05	0.000178
11736504_NR3C1	-0.42203	2.05E-05	0.000178
11719494_JOSD1	0.373799	2.05E-05	0.000178
11715405_COL6A1	0.384615	2.06E-05	0.000178
11715983_ADNP	0.381613	2.06E-05	0.000178
11739824_PCDHB16	-0.39764	2.06E-05	0.000178
11721212_THBS4	-0.42792	2.06E-05	0.000178
11750773_KIF2A	0.432775	2.06E-05	0.000178
11747932_LEF1	-0.48187	2.06E-05	0.000178
11717549_BTBD10	0.486325	2.06E-05	0.000179
11749354_WDR41	0.373951	2.06E-05	0.000179
11728610_BBS7	0.585414	2.06E-05	0.000179
11756670_TUBA1C	-0.35864	2.07E-05	0.000179
11724970_FLT1	0.844076	2.07E-05	0.000179
11717068_DIP2B	0.506957	2.07E-05	0.000179
11750722_GPHN	0.525691	2.07E-05	0.000179
11751130_NRBF2	0.353032	2.07E-05	0.000179
11727196_ZNF202	0.391557	2.07E-05	0.000179
11760879_RPL7L1	-0.49577	2.08E-05	0.00018
11725461_MAU2	0.337608	2.08E-05	0.00018
11718089_TNKS	0.503841	2.08E-05	0.00018
11717021_TMEM222	-0.49993	2.08E-05	0.00018
11719827_QPCT	0.41438	2.08E-05	0.00018
11729805_PPHLN1	0.375278	2.08E-05	0.00018
11751094_CEACAM1	0.696637	2.08E-05	0.00018
11756021_SNRPB2	-0.35549	2.08E-05	0.00018
11753483_PARN	-0.53681	2.08E-05	0.00018
11726691_ANGPT1	0.506406	2.08E-05	0.00018
11725706_AMOTL1	-0.50343	2.08E-05	0.00018

11727000_TSNAX	-0.36907	2.09E-05	0.00018
11748895_GRPEL2	0.519891	2.09E-05	0.00018
11716094_KLF6	0.408886	2.09E-05	0.00018
11730804_PRPF38B	0.404618	2.09E-05	0.00018
11752572_SLC2A1	-0.36768	2.09E-05	0.00018
11754958_ATP6AP1	0.375458	2.09E-05	0.000181
11726149_PPIP5K2	-0.37534	2.09E-05	0.000181
11722551_ITSN1	-0.40709	2.10E-05	0.000181
11754934_PTGES2	-0.51579	2.10E-05	0.000181
11727769_BNC2	-0.52778	2.10E-05	0.000181
11746820_SMAD2	0.426224	2.10E-05	0.000181
11759746_ARHGEF28	0.50866	2.10E-05	0.000181
11716182_ALCAM	0.550425	2.10E-05	0.000181
11715204_HIST2H2AC	-0.629	2.10E-05	0.000181
11716152_PAFAH1B1	-0.49219	2.10E-05	0.000181
11719460_WDR41	0.526256	2.11E-05	0.000182
11715905_DDX3X	0.356985	2.11E-05	0.000182
11737612_ZDHHC21	0.528221	2.11E-05	0.000182
11730685_CSMD3	0.636114	2.11E-05	0.000182
11746212_ODF2	-0.40111	2.11E-05	0.000182
11751696_C3orf52	0.534667	2.11E-05	0.000182
11737221_FERMT2	0.58442	2.12E-05	0.000182
11729710_MARS2	0.574489	2.12E-05	0.000182
11728278_CASP6 ///	-0.56264	2.12E-05	0.000182
11758250_FBXL3	0.343152	2.12E-05	0.000182
11718073_IL4R	0.387337	2.12E-05	0.000182
11754486_NME4	-0.34157	2.12E-05	0.000182
11755643_TNS3	-0.34999	2.12E-05	0.000182
11716742_TJP2	0.349542	2.12E-05	0.000182
11729672_FAM200B	0.366277	2.12E-05	0.000182
11725737_UBR3	0.452539	2.12E-05	0.000182
11733117_SLC35B4	-0.40117	2.13E-05	0.000183
11738214_LBX2	-0.33209	2.13E-05	0.000183
11726999_TSNAX	-0.56525	2.13E-05	0.000183
11739222_NF1	0.79729	2.13E-05	0.000183
11719274_PANK4	-0.47715	2.13E-05	0.000183
11756612_BARD1	-0.74797	2.13E-05	0.000183
11717099_HIST1H2BK	0.334137	2.13E-05	0.000183
11720236_KAT8	-0.34631	2.13E-05	0.000183
11755600_GEMIN6	-0.39009	2.14E-05	0.000183
11717738_PCYOX1	-0.57441	2.14E-05	0.000183
11721176_PPP1R26	-0.32055	2.14E-05	0.000183
11758346_PAPSS2	-0.39232	2.14E-05	0.000184
11749077_HDGF	-0.43856	2.14E-05	0.000184
11748693_RAB5A	0.372345	2.15E-05	0.000184
11737416_ZNF69	0.57865	2.15E-05	0.000184
11728232_CLDN1	1.103556	2.15E-05	0.000184
11739672_ZNF253	0.724094	2.15E-05	0.000184
11720299_CHTOP	-0.34526	2.15E-05	0.000184
11747166_WDR41	0.360452	2.15E-05	0.000184
11720833_YEATS4	-0.51038	2.16E-05	0.000185

11719509_CSRP2BP /	-0.48423	2.16E-05	0.000185
11757445_PTEN	0.361642	2.16E-05	0.000185
11720509_FBN1	0.480355	2.16E-05	0.000185
11725314_GBAS	-0.46079	2.16E-05	0.000185
11739710_PHACTR2	0.409029	2.16E-05	0.000185
11759062_SIK2	-0.44417	2.17E-05	0.000185
11757556_BLMH	-0.33147	2.17E-05	0.000185
11735140_TNFSF9	0.455915	2.17E-05	0.000185
11719594_GMEB2	-0.39589	2.17E-05	0.000186
11717105_PSMB5	-0.35165	2.17E-05	0.000186
11743701_GNL3L	0.370944	2.18E-05	0.000186
11717685_JAK1	0.39695	2.18E-05	0.000186
11745010_DCBLD2	0.357265	2.18E-05	0.000186
11726626_CCNG2	-0.50433	2.18E-05	0.000186
11721777_SSH2	0.323242	2.18E-05	0.000186
11733742_DCXR	-0.44926	2.18E-05	0.000186
11724883_HAPLN3	-0.51063	2.18E-05	0.000186
11724190_ATP5B	-0.34693	2.18E-05	0.000186
11742932_SAMD1	-0.56215	2.19E-05	0.000187
11725002_IRX3	-0.5734	2.19E-05	0.000187
11716248_TMED9	-0.44752	2.19E-05	0.000187
11729219_NCK1	0.39428	2.19E-05	0.000187
11736224_SPATS2	0.351353	2.19E-05	0.000187
11723620_OBSL1	-0.59443	2.19E-05	0.000187
11723703_ABHD5	0.68119	2.19E-05	0.000187
11719707_DHRS13	-0.4342	2.20E-05	0.000187
11718885_ARL5A	0.363831	2.20E-05	0.000187
11744259_ATP1B3	-0.33263	2.20E-05	0.000188
11749233_NAP1L1	-0.35216	2.20E-05	0.000188
11718425_HSDL2	-0.41967	2.20E-05	0.000188
11737211_HDGF	-0.40732	2.21E-05	0.000188
11723072_NDUFV3	0.459765	2.21E-05	0.000188
11751308_RBM24	0.787632	2.21E-05	0.000188
11740610_ZNF121	0.338752	2.21E-05	0.000188
11733482_DYRK3	0.358564	2.22E-05	0.000189
AFFX-HUMISTAT1	0.376487	2.22E-05	0.000189
11754122_DBI	-0.38134	2.22E-05	0.000189
11743051_CDKN2C	-0.43132	2.22E-05	0.000189
11721567_POLD1	-0.57915	2.22E-05	0.000189
11733094_FOXRED2	-0.49999	2.22E-05	0.000189
11736405_DNMT1	-0.43725	2.23E-05	0.000189
11717693_CEBPG	-0.48473	2.23E-05	0.00019
11732729_AJAP1	0.583726	2.23E-05	0.00019
11729059_TOR4A	-0.49653	2.23E-05	0.00019
11748777_ZNF671	0.419507	2.24E-05	0.00019
11727173_EPC2	0.524848	2.24E-05	0.00019
11742791_ATP6AP1L	-0.40614	2.24E-05	0.00019
11744160_ATAD3A	-0.4238	2.25E-05	0.000191
11761428_LRRC7	1.359375	2.25E-05	0.000191
11747209_SEC62	0.327078	2.25E-05	0.000191
11748087_C10orf2	-0.59975	2.25E-05	0.000191

11717245_LSM4	-0.32355	2.25E-05	0.000191
11726457_GSPT2	0.569734	2.25E-05	0.000191
11719438_SRGAP2	-0.43809	2.25E-05	0.000191
11722461_TNKS2	0.456463	2.25E-05	0.000191
11736178_TRNT1	0.413902	2.25E-05	0.000191
11716228_MPDU1	-0.50015	2.25E-05	0.000191
11760520_NSD1	0.408111	2.26E-05	0.000191
11721332_BRE	0.326374	2.26E-05	0.000191
11756328_TARDBP	-0.46309	2.26E-05	0.000192
11736009_RSL1D1	0.339902	2.26E-05	0.000192
11719486_EPS15	0.337843	2.26E-05	0.000192
11731650_NTM	0.660853	2.27E-05	0.000192
11730294_SIN3A	-0.40944	2.27E-05	0.000192
11725848_BCORL1	-0.44888	2.27E-05	0.000192
11727810_ELP2	0.380268	2.27E-05	0.000192
11745804_LIMS1	0.349725	2.27E-05	0.000192
11716227_MPDU1	-0.41038	2.27E-05	0.000192
11735156_ZNF585A	0.496764	2.27E-05	0.000192
11736523_TNNT1	-0.3851	2.27E-05	0.000192
11715674_CCNG1	0.477583	2.27E-05	0.000192
11729935_PITX2	-0.31829	2.28E-05	0.000193
11736493_DAZAP2	-0.45138	2.28E-05	0.000193
11726251_SLC1A3	0.763103	2.28E-05	0.000193
11729644_GPX8	-0.44276	2.29E-05	0.000193
11751336_MKRN1	-0.50759	2.29E-05	0.000193
11748830_MKNK1	0.385007	2.29E-05	0.000193
11743541_CEP57	-0.35137	2.29E-05	0.000193
11753493_RAB18	0.511616	2.29E-05	0.000193
11726461_PDCD2	-0.31597	2.29E-05	0.000193
11729885_C18orf54	-0.60445	2.29E-05	0.000193
11729937_NGF	0.410164	2.29E-05	0.000193
11719417_ANKRA2	0.516507	2.30E-05	0.000194
11723321_FBXO30	0.412622	2.30E-05	0.000194
11757642_FHOD1	0.332152	2.30E-05	0.000194
11758483_ARRDC4	-0.64974	2.30E-05	0.000194
11761686_IFIH1	0.694553	2.30E-05	0.000194
11749003_ERAP1	0.510816	2.30E-05	0.000194
11731006_MIS18BP1	-1.01262	2.30E-05	0.000194
11724209_KIFC1	-0.59366	2.30E-05	0.000194
11742903_CETN3	-0.40307	2.30E-05	0.000194
11726469_C1orf106	-0.6628	2.30E-05	0.000194
11752693_SUV39H1	-0.45473	2.31E-05	0.000194
11729145_HNRNPD	-0.42531	2.31E-05	0.000194
11725668_HS2ST1	-0.48239	2.31E-05	0.000194
11748080_DLST	-0.60775	2.31E-05	0.000195
11746940_CD9	-0.33858	2.31E-05	0.000195
11754854_ATF7IP2	0.407292	2.31E-05	0.000195
11754273_SH3RF1	0.479569	2.32E-05	0.000195
11758489_SHROOM3	0.389563	2.32E-05	0.000195
11719323_PTEN	0.419602	2.32E-05	0.000195
11757018_COL12A1	0.337425	2.32E-05	0.000195

11725492_RAB20	0.439761	2.32E-05	0.000195
11721141_TCEA3	0.400977	2.32E-05	0.000195
11751671_LGALS8	0.362614	2.32E-05	0.000195
11726428_DCAF6	0.362592	2.32E-05	0.000195
11730906_PDS5B	-0.39877	2.32E-05	0.000195
11727627_ALKBH8	0.384568	2.32E-05	0.000195
11743139_ 5-Mar	0.354381	2.33E-05	0.000196
11728793_EEF2K ///	-0.31806	2.33E-05	0.000196
11745907_UMAD1	-0.64649	2.34E-05	0.000196
11736484_AGFG1	-0.44296	2.34E-05	0.000197
11748327_ZNF138	0.697153	2.34E-05	0.000197
11735282_SLC10A4	-0.44101	2.34E-05	0.000197
11728754_TEX19	-0.35584	2.34E-05	0.000197
11735789_MTFR1L	-0.62629	2.35E-05	0.000197
11743543_CEP57	-0.32499	2.35E-05	0.000197
11725625_ACE2	0.567195	2.35E-05	0.000197
11746616_WSB1	0.507412	2.36E-05	0.000197
11763322_CSNK2A2	-0.35718	2.36E-05	0.000198
11755709_LARP6	0.36961	2.36E-05	0.000198
11716351_HSPE1 ///	-0.37679	2.36E-05	0.000198
11752897_HBP1	0.37241	2.36E-05	0.000198
11729192_MYNN	0.487599	2.37E-05	0.000198
11747414_ZNF548	0.571903	2.37E-05	0.000198
11739371_CPOX	-0.34846	2.37E-05	0.000198
11753575_COQ7	-0.45957	2.37E-05	0.000198
11751378_TMEM161B	0.337592	2.37E-05	0.000198
11724098_LRIG3	0.369241	2.37E-05	0.000198
11719305_SYNPO	-0.40217	2.37E-05	0.000198
11739544_C19orf12	0.382101	2.38E-05	0.000199
11718272_TAX1BP1	-0.58023	2.38E-05	0.000199
11717415_MLST8	-0.38463	2.38E-05	0.000199
11748011_TMEM9	-0.37457	2.38E-05	0.000199
11752580_AKAP10	0.588518	2.38E-05	0.000199
11753893_PRELP	-0.36506	2.38E-05	0.000199
11727151_PRICKLE1	0.53676	2.38E-05	0.000199
11741154_LIMS1	0.394896	2.38E-05	0.000199
11746604_MTG2	-0.3821	2.39E-05	0.000199
11723297_AKAP2 ///	0.367888	2.39E-05	0.000199
11744897_ARMCX3	0.424239	2.39E-05	0.000199
11747820_UPP1	0.507451	2.39E-05	0.000199
11744085_UBALD2	-0.45313	2.39E-05	0.000199
11726188_SHISA3	-0.75288	2.39E-05	0.000199
11719752_VAC14	-0.53886	2.39E-05	0.000199
11718522_SMG7	0.453206	2.39E-05	0.000199
11730269_NIPBL	0.382934	2.39E-05	0.000199
11730404_MEX3B	-0.36519	2.39E-05	0.0002
11738256_GRK6	-0.4458	2.39E-05	0.0002
11761284_DIMT1	0.521197	2.40E-05	0.0002
11717414_MLST8	-0.38547	2.40E-05	0.0002
11717864_PSME4	0.445808	2.40E-05	0.0002
11729659_TMEM69	-0.37362	2.41E-05	0.000201

11727301_FGFR1	-0.4037	2.41E-05	0.000201
11757044_HILPDA	-0.42397	2.41E-05	0.000201
11719448_RAB14	0.429826	2.42E-05	0.000201
11753169_FKTN	0.87944	2.42E-05	0.000201
11723298_AKAP2 ///	0.381862	2.42E-05	0.000201
11718344_CNOT7	-0.34034	2.42E-05	0.000201
11750545_CNOT7	-0.34034	2.42E-05	0.000201
11722516_PARS2	-0.32164	2.42E-05	0.000202
11737966_C15orf54	0.967207	2.43E-05	0.000202
11754009_PSMB5	-0.38086	2.43E-05	0.000202
11754759_CHTOP	-0.37685	2.43E-05	0.000202
11747931_LEF1	-0.60164	2.43E-05	0.000203
11725158_STEAP1	-0.36129	2.44E-05	0.000203
11755299_SAPCD2	-1.20987	2.44E-05	0.000203
11741038_GPR89A //	0.423755	2.44E-05	0.000203
11724958_NT5M	-0.46352	2.44E-05	0.000203
11743511_CDCA2	-0.54028	2.44E-05	0.000203
11755584_FAM161A	-0.46875	2.44E-05	0.000203
11720832_SOX18	-0.43394	2.44E-05	0.000203
11755106_PDLIM3	-0.47808	2.44E-05	0.000203
11740534_PTGES3L	-0.55693	2.44E-05	0.000203
11758218_MOSPD1	0.444072	2.44E-05	0.000203
11728865_ZMYND19	-0.35918	2.44E-05	0.000203
11757317_PEF1	-0.40322	2.44E-05	0.000203
11754473_CADM1	0.52898	2.44E-05	0.000203
11742029_NR1D2	0.408364	2.44E-05	0.000203
11754252_ 5-Mar	0.355157	2.44E-05	0.000203
11726431_OSBPL3	0.35701	2.45E-05	0.000203
11716108_XPNPEP1	0.353605	2.45E-05	0.000203
11756489_NUDT8	-0.38531	2.45E-05	0.000203
11750284_LINS1	0.44549	2.45E-05	0.000203
11743473_ZNF226	0.507148	2.45E-05	0.000203
11717709_LPCAT4	-0.36223	2.45E-05	0.000203
11737758_CARS	0.364008	2.45E-05	0.000203
11729820_USMG5	-0.38604	2.45E-05	0.000203
11739548_CSPG4	-0.37234	2.46E-05	0.000204
11758107_PCBP1	-0.30796	2.46E-05	0.000204
11754215_GPN3	-0.36769	2.46E-05	0.000204
11726524_ZFHX3	-0.5924	2.46E-05	0.000204
11720904_QSER1	-0.34391	2.47E-05	0.000204
11722048_LDAH	0.435379	2.47E-05	0.000204
11718781_SSBP2	0.470635	2.47E-05	0.000204
11751616_LACTB2	-0.36735	2.47E-05	0.000204
11754830_CD22	0.654169	2.47E-05	0.000204
11742215_APP	0.309685	2.47E-05	0.000204
11725038_HADHB	-0.31367	2.47E-05	0.000204
11746779_NCOA7	0.433338	2.47E-05	0.000204
11724814_MED13L	0.33382	2.47E-05	0.000204
11743760_CDC42SE2	0.654439	2.47E-05	0.000204
11725001_IRX3	-0.59622	2.47E-05	0.000204
11727485_TPR	-0.55453	2.48E-05	0.000205

11723688_CREBL2	0.439749	2.48E-05	0.000205
11749075_PISD	0.359716	2.48E-05	0.000205
11744013_WIZ	-0.3093	2.48E-05	0.000205
11756703_EPS15	0.409234	2.48E-05	0.000205
11751491_PARVA	0.362204	2.48E-05	0.000205
11728964_ZNF585A /	0.479293	2.48E-05	0.000205
11744413_HSPA1A //	-0.36089	2.49E-05	0.000205
11729626_DCAF7	0.381202	2.49E-05	0.000205
11720648_IP6K2	0.327662	2.49E-05	0.000205
11724293_TRIT1	0.379883	2.49E-05	0.000206
11728809_COL8A1	0.562241	2.49E-05	0.000206
11728806_CADM1	0.484255	2.50E-05	0.000206
11715346_EBI3	0.573175	2.50E-05	0.000206
11722168_CSE1L	-0.35815	2.50E-05	0.000206
11742725_ELF2	0.329615	2.50E-05	0.000206
11731121_VASH2	-0.91844	2.50E-05	0.000206
11732160_NUDCD1	0.353502	2.51E-05	0.000206
11729671_FAM200B	0.327793	2.51E-05	0.000207
11727414_CLN8	0.591868	2.51E-05	0.000207
11733800_SLC31A2	0.481553	2.51E-05	0.000207
11754779_MSANTD2	0.407321	2.51E-05	0.000207
11757503_PNN	-0.487	2.51E-05	0.000207
11726641_MIB1	-0.37412	2.51E-05	0.000207
11724097_LRIG3	0.433316	2.51E-05	0.000207
11717265_CAMK2G	-0.84985	2.51E-05	0.000207
11716437_RAD21	-0.42997	2.51E-05	0.000207
11749099_HK2	-0.60542	2.52E-05	0.000207
11723415_CNN2	0.46099	2.52E-05	0.000207
11725095_TTC33	-0.56547	2.52E-05	0.000207
11742425_NR3C1	-0.4936	2.52E-05	0.000207
11750373_BRCA1	-0.38982	2.52E-05	0.000207
11717372_DNAJC3	0.47943	2.52E-05	0.000207
11720549_PEX6	0.321967	2.52E-05	0.000207
11748175_C19orf54	-0.52686	2.52E-05	0.000207
11752357_DBT	0.407321	2.52E-05	0.000207
11721512_TIMM21	-0.40788	2.52E-05	0.000207
11736418_ZNF800	0.440094	2.52E-05	0.000207
11724088_ZSCAN26	0.509175	2.52E-05	0.000207
11719044_ALKBH5	-0.33235	2.53E-05	0.000207
11731641_LOC102724	-0.51456	2.53E-05	0.000207
11744118_MRPL10	-0.34732	2.53E-05	0.000208
11724506_MIS18A	-0.4747	2.53E-05	0.000208
11757510_HP1BP3	-0.35332	2.54E-05	0.000208
11743491_KTN1	0.317006	2.54E-05	0.000208
11718534_NKTR	0.342883	2.54E-05	0.000208
11715520_MLEC	-0.38921	2.54E-05	0.000208
11744243_TRAF6	0.524526	2.54E-05	0.000208
11746097_NPEPPS	0.328429	2.54E-05	0.000208
11763289_EME2	0.56244	2.54E-05	0.000208
11752487_SNX25	0.494389	2.54E-05	0.000208
11743577_CPE	0.377283	2.55E-05	0.000209

11716438_RAD21	-0.39366	2.55E-05	0.000209
11758049_TIA1	-0.41412	2.55E-05	0.000209
11723527_TCTEX1D2	0.347159	2.55E-05	0.000209
11759641_CSTF2	-0.40949	2.55E-05	0.000209
11728834_C4orf32	0.448802	2.56E-05	0.000209
11752039_PHC3	0.726921	2.56E-05	0.000209
11749842_PSEN1	0.356062	2.56E-05	0.000209
11720041_VTA1	0.349504	2.56E-05	0.00021
11721938_RNF169	0.323771	2.57E-05	0.00021
11739531_PLGLB2	0.471461	2.57E-05	0.00021
11763270_IKZF5	-0.70306	2.57E-05	0.00021
11728838_TMCC3	0.491003	2.57E-05	0.00021
11754723_HACL1	0.336591	2.57E-05	0.00021
11758334_BORA	-0.4289	2.57E-05	0.00021
11757779_PPP4R1	0.371357	2.57E-05	0.00021
11722858_ZNF395	-0.32554	2.58E-05	0.00021
11755724_MLF1	-0.48867	2.58E-05	0.00021
11723631_NKIRAS2	0.422989	2.58E-05	0.00021
11721740_VANGL2	-0.43634	2.58E-05	0.00021
11756139_LOC728554	-0.37288	2.58E-05	0.000211
11744212_SNRPB2	-0.30153	2.58E-05	0.000211
11721498_TPST2	0.398086	2.58E-05	0.000211
11728519_ZNF79	1.095109	2.58E-05	0.000211
11750128_IMMT	-0.33768	2.59E-05	0.000211
11736655_PTGR1	-0.34466	2.59E-05	0.000211
11743481_RB1CC1	0.339574	2.59E-05	0.000211
11717759_NDUFV1	-0.33858	2.59E-05	0.000211
11727529_PHF20L1	0.516666	2.59E-05	0.000211
11731853_PPA2	-0.35954	2.59E-05	0.000211
11733663_ARHGAP11A	-0.42496	2.59E-05	0.000211
11747781_CEP95	0.379214	2.60E-05	0.000212
11759968_LOXL3	0.478543	2.60E-05	0.000212
11721342_NCOA1	0.430084	2.60E-05	0.000212
11742683_HIST1H1C	0.366951	2.60E-05	0.000212
11727120_MYCBP2	0.382938	2.60E-05	0.000212
11733841_EVI2A	0.827009	2.60E-05	0.000212
11757717_FAM204A	0.360011	2.60E-05	0.000212
11718474_IDH1	-0.39332	2.60E-05	0.000212
11739522_ST8SIA4	-0.39105	2.60E-05	0.000212
11746091_MAP4K3	0.689424	2.61E-05	0.000212
11747202_MPDU1	-0.38543	2.61E-05	0.000212
11731568_MDM2	0.34258	2.61E-05	0.000212
11715651_FSTL1	0.359199	2.61E-05	0.000212
11730576_ALPK1	-0.54487	2.62E-05	0.000213
11722356_DAB2	0.686916	2.62E-05	0.000213
11753313_PHB2	-0.33732	2.62E-05	0.000213
11739547_CSPG4	-0.32627	2.62E-05	0.000213
11717810_CYTH3	0.775308	2.62E-05	0.000213
11745511_YTHDF1	0.395101	2.62E-05	0.000213
11744155_PIN1	-0.30573	2.62E-05	0.000213
11743879_MAML3	0.405997	2.63E-05	0.000213

11743111_NAMPT	0.410641	2.63E-05	0.000213
11723978_RAB3A	-0.391	2.63E-05	0.000214
11754233_CEP152	-0.41641	2.64E-05	0.000214
11716659_HNRNPH3	-0.32237	2.64E-05	0.000214
11735911_WDFY3	0.73337	2.64E-05	0.000214
11718308_HEG1	0.383244	2.64E-05	0.000214
11754328_PIGG	0.360028	2.64E-05	0.000214
11725342_VPS37A	0.422916	2.65E-05	0.000215
11747286_SLC25A46	0.385853	2.65E-05	0.000215
11719661_FAM188A	0.50236	2.65E-05	0.000215
11733854_CD164	0.588062	2.65E-05	0.000215
11723697_FLI1	0.392809	2.65E-05	0.000215
11734530_HLA-F	0.392495	2.66E-05	0.000215
11720736_LRRC14	-0.52249	2.66E-05	0.000216
11718199_KPNA3	-0.33473	2.66E-05	0.000216
11725472_PAQR8	-0.48502	2.66E-05	0.000216
11750046_TMEM54	-0.33018	2.66E-05	0.000216
11720135_PROS1	0.449414	2.67E-05	0.000216
11717809_SEPN1	-0.37231	2.67E-05	0.000216
11717290_SQSTM1	0.316506	2.67E-05	0.000216
11747250_GRK5	0.34728	2.67E-05	0.000216
11750315_ZNF429	0.602309	2.67E-05	0.000216
11737098_HLTF	-0.47537	2.67E-05	0.000216
11726876_PML	-0.35767	2.67E-05	0.000216
11717548_BTBD10	0.399049	2.67E-05	0.000216
11716500_PIR	-0.32497	2.67E-05	0.000216
11719644_GATAD2A	-0.36935	2.68E-05	0.000216
11722700_KIAA1109	0.498267	2.68E-05	0.000217
11729706_ZNF561	0.707004	2.68E-05	0.000217
11755249_CCNL2	0.366606	2.69E-05	0.000217
11755416_BRCA1	-0.46411	2.69E-05	0.000217
11719430_SOS2	0.365168	2.69E-05	0.000217
11756450_SYNE2	-0.40976	2.69E-05	0.000217
11740429_XRN1	-0.30943	2.69E-05	0.000218
11716584_DYNLT1	0.354828	2.69E-05	0.000218
11735249_SP140L	0.37316	2.69E-05	0.000218
11731624_CCDC186	0.390941	2.70E-05	0.000218
11726402_ALG9	0.682788	2.70E-05	0.000218
11729531_MAP3K4	-0.4401	2.70E-05	0.000218
11758889_FAM46A	0.521656	2.70E-05	0.000218
11753204_PTDSS1	-0.47519	2.70E-05	0.000218
11715554_CYB5R3	-0.37874	2.71E-05	0.000218
11736082_GTSE1	-0.54142	2.71E-05	0.000218
11744464_SNX25	0.510011	2.71E-05	0.000219
11748955_IFT88	0.47711	2.71E-05	0.000219
11725929_NEFL	0.405463	2.71E-05	0.000219
11719146_TMEM54	-0.35141	2.72E-05	0.000219
11731832_DDO	0.599818	2.72E-05	0.000219
11717355_FEZ1	0.325087	2.72E-05	0.000219
11756297_PGK1	-0.32261	2.72E-05	0.000219
11756229_FNTA	0.358828	2.72E-05	0.000219

11718961_NUFIP2	0.540745	2.72E-05	0.000219
11719903_ANKRD40	0.418144	2.73E-05	0.000219
11723173_IL17RD	0.711641	2.73E-05	0.00022
11734698_GOLGA8A /	0.504265	2.73E-05	0.00022
11732203_KPNA5	0.430726	2.73E-05	0.00022
11757412_MDP1 ///	-0.73523	2.73E-05	0.00022
11715562_SCD	-0.38508	2.73E-05	0.00022
11720699_CPA2	0.757858	2.74E-05	0.00022
11761141_LGALS8	0.36172	2.74E-05	0.00022
11728429_LCOR	0.311584	2.74E-05	0.00022
11752333_ITGAV	-0.37075	2.75E-05	0.000221
11735449_NEUROG2	-0.51362	2.75E-05	0.000221
11734989_DEDD	0.432118	2.75E-05	0.000221
11747505_CEP70	-0.33473	2.75E-05	0.000221
11753451_WDR41	0.398756	2.75E-05	0.000221
11756166_TTC32	-0.4734	2.75E-05	0.000221
11750050_CAST	0.327164	2.76E-05	0.000221
11757441_SRRD	0.330962	2.76E-05	0.000221
11727020_ABI2	-0.34958	2.76E-05	0.000221
11718331_NAPB	0.572794	2.76E-05	0.000221
11716754_TPGS2	-0.37826	2.76E-05	0.000222
11755462_TUBGCP4	-0.34137	2.76E-05	0.000222
11726689_STAT1	0.409964	2.76E-05	0.000222
11721076_MTCH2	-0.3188	2.76E-05	0.000222
11750222_ARID5B	0.387441	2.77E-05	0.000222
11721688_ID4	0.396127	2.77E-05	0.000222
11719795_PPP2R5D	-0.43623	2.77E-05	0.000222
11718267_ANGPTL2	0.308898	2.77E-05	0.000222
11746392_INTS6	0.422488	2.77E-05	0.000222
11756468_SCYL3	0.466509	2.77E-05	0.000222
11753812_HMGN1	-0.36687	2.77E-05	0.000222
11752788_VGLL4	0.554127	2.77E-05	0.000222
11733810_UBE2J2	0.358764	2.78E-05	0.000223
11725190_ADGRG6	0.496731	2.78E-05	0.000223
11719410_HIPK2	0.314869	2.78E-05	0.000223
11741405_RPL28	0.404626	2.78E-05	0.000223
11743759_CDC42SE2	0.542927	2.79E-05	0.000223
11735060_CD46	0.338068	2.79E-05	0.000223
11724444_ASB9	-0.37138	2.79E-05	0.000223
11729026_BMP1	0.636595	2.79E-05	0.000223
11754240_MRPS5	-0.32687	2.79E-05	0.000223
11749353_WDR41	0.357599	2.79E-05	0.000223
11754268_MED19	0.371348	2.80E-05	0.000224
11750914_GSPT2	0.48769	2.80E-05	0.000224
11758635_CREBBP	-0.34593	2.81E-05	0.000225
11725262_WDR60	0.403556	2.81E-05	0.000225
11716448_NUDT9	0.316954	2.81E-05	0.000225
11747074_ACTN4	-0.34482	2.81E-05	0.000225
11744299_FDCSP	1.114679	2.82E-05	0.000225
11726224_HNRNPL	-0.3791	2.82E-05	0.000225
11734470_C8orf31	0.404479	2.82E-05	0.000225

11739791_MOCS3	0.356972	2.82E-05	0.000225
11736513_NMT2	0.57743	2.82E-05	0.000225
11717555_SETD7	-0.35556	2.82E-05	0.000225
11728368_CCP110	0.356085	2.82E-05	0.000225
11721204_ANK3	0.486987	2.82E-05	0.000225
11732306_CREB1	-0.4413	2.82E-05	0.000225
11737429_MSR1	0.524254	2.82E-05	0.000225
11724208_DPH2	0.341581	2.83E-05	0.000225
11719616_ZNF12	0.42607	2.83E-05	0.000225
11719419_TMEM251	-0.42624	2.83E-05	0.000226
11732580_ME3	0.443398	2.83E-05	0.000226
11718597_DDRGK1	0.436018	2.84E-05	0.000226
11732400_GSG2	-0.42142	2.84E-05	0.000226
11721279_SUV39H1	-0.48919	2.84E-05	0.000226
11721798_SUMO3	-0.41563	2.84E-05	0.000226
11759630_GPATCH2	0.595012	2.84E-05	0.000226
11726178_SOX6	-0.55402	2.84E-05	0.000226
11731564_MDM2	0.581273	2.84E-05	0.000227
11758112_GZF1	0.446405	2.85E-05	0.000227
11739392_RBPJ	-0.44614	2.85E-05	0.000227
11749258_ATP6AP1	0.33682	2.85E-05	0.000227
11746690_URB2	0.390856	2.85E-05	0.000227
11715287_C15orf40	0.618807	2.86E-05	0.000227
11748572_ANKFY1	0.369295	2.86E-05	0.000227
11720890_AGL	0.441794	2.86E-05	0.000227
11747619_CERS5	0.406313	2.86E-05	0.000227
11731759_RAD52	0.342315	2.86E-05	0.000227
11750881_LMBR1	0.359616	2.86E-05	0.000228
11743542_CEP57	-0.44968	2.86E-05	0.000228
11720727_UBR1	0.366845	2.87E-05	0.000228
11721246_BGN	-0.36181	2.87E-05	0.000228
11740442_APOBEC3A	-0.31803	2.87E-05	0.000228
11736154_XPR1	0.350964	2.87E-05	0.000228
11742086_PRMT3	-0.46682	2.87E-05	0.000228
11723647_DNAJA2	0.367981	2.87E-05	0.000228
11740704_ARID4A	0.430976	2.87E-05	0.000228
11739205_TTL	0.333682	2.88E-05	0.000228
11747970_ARMC4	0.529987	2.88E-05	0.000229
11715654_RTFDC1	-0.39164	2.88E-05	0.000229
11727643_TRERF1	-0.56448	2.88E-05	0.000229
11744436_CIT	-0.52754	2.88E-05	0.000229
11721913_MDM4	-0.37608	2.88E-05	0.000229
11742804_TEX261	-0.47556	2.88E-05	0.000229
11732422_ZNF234	0.519135	2.89E-05	0.000229
11720604_STOM	0.319181	2.89E-05	0.000229
11743067_SIAH2	-0.37248	2.89E-05	0.000229
11732482_PEX13	0.583882	2.89E-05	0.000229
11729399_ZNF597	0.754758	2.89E-05	0.000229
11732526_CSGALNACT	0.72556	2.89E-05	0.000229
11727688_KCTD18	-0.59155	2.90E-05	0.000229
11739854_LYRM1	0.436533	2.90E-05	0.000229

11734199_ZNF844	0.685241	2.90E-05	0.000229
11732037_PTPN12	0.318728	2.90E-05	0.000229
11718793_C4orf27	-0.39088	2.90E-05	0.000229
11736282_PDLIM5	0.395998	2.90E-05	0.000229
11720965_DCK	-0.32444	2.90E-05	0.00023
11733859_CLASP2	-0.40638	2.90E-05	0.00023
11715404_COL6A1	0.37377	2.90E-05	0.00023
11725081_RNFT1	0.492037	2.90E-05	0.00023
11717116_CERK	-0.45744	2.91E-05	0.00023
11716578_TMEM43	0.389579	2.91E-05	0.00023
11716629_IER5	0.340562	2.92E-05	0.000231
11718164_PPP1R15B	0.344549	2.92E-05	0.000231
11748195_SLC39A9	0.593128	2.92E-05	0.000231
11719944_RARG	-0.55424	2.93E-05	0.000232
11759494_ITGA9	-0.47091	2.93E-05	0.000232
11727783_TPM4	-0.33672	2.93E-05	0.000232
11731336_TRPV1	0.779383	2.93E-05	0.000232
11748486_AGPS	0.42765	2.94E-05	0.000232
11752852_PPHLN1	0.391018	2.94E-05	0.000232
11743929_UHRF1BP1L	0.486081	2.94E-05	0.000232
11751138_USP46	0.524053	2.94E-05	0.000232
11754804_CDC16	0.362837	2.94E-05	0.000232
11754418_G3BP1	-0.38109	2.95E-05	0.000232
11746367_CSE1L	-0.34056	2.95E-05	0.000233
11734898_IPP	-0.61133	2.95E-05	0.000233
11758600_SRSF1	-0.4931	2.95E-05	0.000233
11744510_RANGRF	-0.36484	2.95E-05	0.000233
11755852_PRRC1	-0.3054	2.95E-05	0.000233
11720494_CTNNAL1	0.425634	2.96E-05	0.000233
11729072_AASDH	-0.3357	2.96E-05	0.000233
11763619_SNAPC4	0.415581	2.96E-05	0.000233
11723691_CD302 ///	-0.54952	2.96E-05	0.000233
11741731_FBXO34	0.344999	2.96E-05	0.000233
11719459_EXOC5	0.315901	2.96E-05	0.000234
11721754_ALG13	0.448107	2.97E-05	0.000234
11752010_ANO3	0.541082	2.97E-05	0.000234
11720528_LTBP2	1.083372	2.97E-05	0.000234
11716238_ARHGAP1	-0.34572	2.97E-05	0.000234
11733840_ABHD2	0.555116	2.97E-05	0.000234
11728724_TAF6	-0.35715	2.98E-05	0.000234
11745214_PIK3CA	0.308855	2.98E-05	0.000234
11722998_ALDH6A1	-0.5636	2.98E-05	0.000234
11737821_HS6ST2	-0.70282	2.98E-05	0.000235
11740962_UBA5	0.327169	2.98E-05	0.000235
11723042_UBE2D1	-0.63022	2.99E-05	0.000235
11730739_PDLIM4	0.324395	3.00E-05	0.000236
11731511_DBT	-0.51257	3.00E-05	0.000236
11718976_ARPP19	0.410101	3.00E-05	0.000236
11757976_HTATSF1	-0.31046	3.00E-05	0.000236
11753191_SLC17A5	0.567304	3.01E-05	0.000236
11755160_JOSD1	0.380687	3.01E-05	0.000236

11736267_TRAPPC8	0.322669	3.01E-05	0.000236
11740528_AFF3	0.638573	3.01E-05	0.000236
11728767_KLHL5	0.451787	3.01E-05	0.000236
11758092_EFNA5	-0.40287	3.01E-05	0.000236
11734972_MCM8	-0.381	3.01E-05	0.000236
11746612_PTCD3	0.330205	3.02E-05	0.000237
11723906_ZNF75A	0.61853	3.02E-05	0.000237
11715976_VGLL4	0.399593	3.02E-05	0.000237
11763693_GLS	0.373746	3.02E-05	0.000237
11752481_APP	0.342198	3.02E-05	0.000237
11724307_ORAOV1	0.324552	3.03E-05	0.000237
11736938_USP36	0.413063	3.03E-05	0.000237
11721526_PROSER1	-0.40144	3.03E-05	0.000238
11720627_ATP6V1D	0.322683	3.03E-05	0.000238
11725098_RABL3	-0.85835	3.04E-05	0.000238
11726329_GBP1	0.71826	3.04E-05	0.000238
11733254_FXYD3	-0.35889	3.04E-05	0.000238
11756990_ZNF550	0.4903	3.04E-05	0.000238
11733776_DUSP3	-0.53948	3.04E-05	0.000238
11756991_WDR18	-0.30038	3.04E-05	0.000238
11715702_ITGA5	0.406024	3.04E-05	0.000238
11733044_SLC7A2	-0.42683	3.05E-05	0.000238
11747562_AP1G1	0.57404	3.05E-05	0.000239
11754020_GNPTG	0.320932	3.05E-05	0.000239
11716780_CLDN4	-0.48381	3.05E-05	0.000239
11720492_PARN	-0.39587	3.05E-05	0.000239
11733422_LRRFIP2	0.444127	3.06E-05	0.000239
11715210_PNMAL1	-0.31273	3.06E-05	0.000239
11751484_BGN	-0.38338	3.06E-05	0.00024
11758661_CDKN2C	-0.46882	3.07E-05	0.00024
11749782_BCAT1	0.44507	3.07E-05	0.00024
11721146_MKI67	-0.45282	3.07E-05	0.00024
11728237_SMG1	0.333758	3.07E-05	0.00024
11733656_RSRC2	0.377005	3.07E-05	0.00024
11744070_LSG1	0.308826	3.07E-05	0.00024
11748600_ZNF410	0.371506	3.07E-05	0.00024
11746403_CEP70	-0.33419	3.08E-05	0.00024
11740266_JRKL	-0.5815	3.08E-05	0.00024
11739922_ZNF37A	0.348924	3.08E-05	0.00024
11753039_CLMP	-0.39804	3.08E-05	0.000241
11728357_C10orf88	0.376495	3.08E-05	0.000241
11717393_FN3KRP	-0.48493	3.09E-05	0.000241
11753224_TSEN34	-0.34843	3.09E-05	0.000241
11715570_CD9	-0.37136	3.09E-05	0.000241
11743323_CCDC50	-0.36646	3.09E-05	0.000241
11758004_CEP350	0.423585	3.10E-05	0.000241
11754950_SSR4	0.315868	3.10E-05	0.000241
11725448_PLD6	0.420598	3.10E-05	0.000242
11739680_AHR	0.410021	3.10E-05	0.000242
11715827_M6PR	0.314385	3.10E-05	0.000242
11748605_POLR1B	0.428125	3.10E-05	0.000242

11744267_VPS51	-0.36443	3.10E-05	0.000242
11735390_TMEM237	-0.36325	3.11E-05	0.000242
11727275_KDELC2	-0.51109	3.11E-05	0.000242
11739380_FAM198B	0.476845	3.11E-05	0.000243
11718020_LRRC20	-0.48807	3.12E-05	0.000243
11743073_IPO9	-0.33741	3.12E-05	0.000243
11719891_CRYZ	-0.36884	3.13E-05	0.000243
11733642_NECAP2	0.376149	3.13E-05	0.000243
11744777_DPY30	-0.32838	3.13E-05	0.000243
11734691_TAF11	0.416429	3.13E-05	0.000243
11759669_CEP57	-0.49694	3.13E-05	0.000243
11752102_DNAJC10	0.311562	3.13E-05	0.000244
11743389_PHTF2	0.346041	3.13E-05	0.000244
11755312_FBXO25	0.520305	3.13E-05	0.000244
11743314_FASN	-0.34223	3.13E-05	0.000244
11717563_DTX3L	0.605833	3.14E-05	0.000244
11723521_ZNF385A	-0.52962	3.15E-05	0.000245
11747616_ASPH	-0.39144	3.15E-05	0.000245
11731450_SEC62	0.386199	3.15E-05	0.000245
11760108_PHF10	0.389583	3.15E-05	0.000245
11745587_TUBD1	-0.57229	3.15E-05	0.000245
11758735_COASY	-0.51263	3.15E-05	0.000245
11716677_ZNF106	0.502616	3.16E-05	0.000245
11716652_EML4	0.475444	3.16E-05	0.000245
11749303_HNRNPD	-0.36072	3.16E-05	0.000245
11717335_TRIM27	-0.35537	3.17E-05	0.000246
11724301_EEA1	0.370402	3.17E-05	0.000246
11756820_IFIT1	0.326791	3.17E-05	0.000246
11715779_HNRNPH2 /	-0.49307	3.17E-05	0.000246
11749774_MAP3K7CL	0.78666	3.18E-05	0.000246
11743091_SLC36A4	-0.38558	3.18E-05	0.000246
11725393_MAK16	0.339866	3.18E-05	0.000246
11723390_CORO2A	-0.55399	3.18E-05	0.000246
11739507_PBX3	0.313955	3.18E-05	0.000246
11758690_CRIPAK	-0.37273	3.18E-05	0.000247
11717171_BCL2L2	0.39366	3.18E-05	0.000247
11727156_TRIOBP	0.341661	3.19E-05	0.000247
11733400_C3orf62	0.839281	3.19E-05	0.000247
11754739_ARHGAP5	-0.39367	3.19E-05	0.000247
11719169_BRI3	0.348005	3.19E-05	0.000247
11721820_SRPK2	0.364171	3.19E-05	0.000247
11728748_ARAF	-0.30472	3.20E-05	0.000247
11755834_CAST	0.417474	3.20E-05	0.000247
11719872_LTV1	0.354271	3.20E-05	0.000248
11755962_SLC25A28	-0.30742	3.20E-05	0.000248
11752610_THEMIS2	-0.43303	3.20E-05	0.000248
11742723_STAT6	-0.34519	3.21E-05	0.000248
11739481_H3F3A ///	-0.3095	3.21E-05	0.000248
11758224_CCDC80	0.512935	3.21E-05	0.000248
11750750_DDX3X	0.295735	3.21E-05	0.000248
11731223_SHOX2	-0.43117	3.21E-05	0.000248

11729550_ADGRG6	0.494317	3.21E-05	0.000248
11720558_GEM	0.592415	3.21E-05	0.000248
11725804_C21orf91	0.589153	3.22E-05	0.000248
11737518_SNX18	0.435759	3.22E-05	0.000249
11726496_RYR2	0.720497	3.22E-05	0.000249
11758731_PPP2CA	-0.40967	3.22E-05	0.000249
11722525_ANKHD1 //	0.341514	3.23E-05	0.000249
11739879_TNIK	0.826463	3.23E-05	0.00025
11749899_ATAD1	0.332906	3.23E-05	0.00025
11719472_IRF2BP2	-0.39476	3.23E-05	0.00025
11752499_CALD1	0.316909	3.24E-05	0.00025
11747206_C7orf43	0.418756	3.24E-05	0.00025
11727148_ZNF564	0.713307	3.24E-05	0.00025
11748227_GID8	-0.33662	3.24E-05	0.00025
11728014_RIMKLB	0.443298	3.24E-05	0.00025
11726228_GNG4	-0.31691	3.24E-05	0.00025
11741691_GPR89A //	0.485597	3.25E-05	0.00025
11737839_MBOAT7	-0.4326	3.25E-05	0.00025
11752129_BIVM-ERCC	0.550831	3.25E-05	0.000251
11731640_LOC102724	-0.32822	3.26E-05	0.000251
11731515_XPO4	0.455472	3.26E-05	0.000251
11733987_C7orf55-L	-0.37534	3.26E-05	0.000251
11742817_RNF214	0.459822	3.26E-05	0.000251
11749247_ZNF625 //	0.391881	3.27E-05	0.000251
11719324_PTEN	0.356876	3.27E-05	0.000252
11743070_PRPF38A	-0.3174	3.27E-05	0.000252
11743256_VAPA	-0.45108	3.27E-05	0.000252
11754636_ZNF708	0.527385	3.27E-05	0.000252
11757690_LAMTOR2	-0.33749	3.28E-05	0.000252
11746884_RAD52	0.480528	3.28E-05	0.000252
11738592_BLID	-0.38241	3.28E-05	0.000252
11762746_ZNF826P	-0.64878	3.28E-05	0.000252
11736469_SLC35D1	0.623645	3.29E-05	0.000253
11743895_RCOR3	0.445405	3.29E-05	0.000253
11715585_PMP22	0.301955	3.29E-05	0.000253
11744704_GOLGA3	-0.33775	3.29E-05	0.000253
11749395_ZCCHC10	0.37875	3.29E-05	0.000253
11722562_CDK4	-0.29994	3.29E-05	0.000253
11748527_ARHGAP24	0.399434	3.29E-05	0.000253
11762109_CXorf40A	-0.45438	3.30E-05	0.000254
11746057_ZNF410	0.396376	3.30E-05	0.000254
11753145_SLC23A2	-0.46457	3.30E-05	0.000254
11745550_TBC1D26 /	0.51807	3.31E-05	0.000254
11739424_DPYSL5	-0.45513	3.31E-05	0.000254
11721755_ALG13	0.413291	3.31E-05	0.000254
11715622_RNF114	0.355735	3.31E-05	0.000254
11753129_TM4SF1	0.325684	3.31E-05	0.000254
11753088_MCTP1	0.443706	3.31E-05	0.000254
11753952_GALT	0.364595	3.33E-05	0.000255
11755631_RMND1	0.486499	3.33E-05	0.000255
11735618_UHRF1BP1L	0.431306	3.33E-05	0.000255

11724442_PTGIS	-0.36399	3.33E-05	0.000255
11715684_PPA1	-0.32008	3.34E-05	0.000256
11736645_WWP2	0.487075	3.34E-05	0.000256
11725222_SNTB1	0.358551	3.34E-05	0.000256
11720594_DLG3	-0.37639	3.34E-05	0.000256
11756171_PFKFB2	-0.57896	3.35E-05	0.000256
11748258_TMEM67	0.867772	3.35E-05	0.000256
11753663_RDM1	-1.19603	3.35E-05	0.000257
11717389_SEPT11	0.329398	3.35E-05	0.000257
11751527_WDR1	0.307929	3.36E-05	0.000257
11759213_BMP8B	-0.55166	3.37E-05	0.000258
11756268_FBXO4	-0.48847	3.37E-05	0.000258
11730395_ZNF552	0.678153	3.37E-05	0.000258
11746302_POLR1B	0.381582	3.37E-05	0.000258
11750134_DNMBP	0.360514	3.37E-05	0.000258
11717203_SEL1L	0.42923	3.37E-05	0.000258
11715750_SSRP1	-0.33721	3.38E-05	0.000258
11727211_HEATR5B	-0.37439	3.38E-05	0.000258
11720145_CCSAP	-0.38577	3.39E-05	0.000259
11747477_MAPK8	0.508518	3.39E-05	0.000259
11748528_DEDD	0.457946	3.39E-05	0.000259
11751065_EXOG	0.443655	3.39E-05	0.000259
11745840_PAQR8	-0.47614	3.40E-05	0.00026
11718393_PREPL	-0.38422	3.40E-05	0.00026
11750123_CKAP2L	-0.48374	3.40E-05	0.00026
11745713_CDCA2	-0.55734	3.40E-05	0.00026
11742273_BRE	0.316881	3.40E-05	0.00026
11753497_BCL10	0.475783	3.41E-05	0.00026
11751062_TCOF1	-0.39536	3.41E-05	0.00026
11733872_PGK1	-0.32907	3.41E-05	0.00026
11754425_CENPJ	-0.43293	3.41E-05	0.00026
11722381_TXNL4B	0.362505	3.42E-05	0.000261
11715602_SPARC	-0.33311	3.42E-05	0.000261
11725667_HS2ST1	-0.45477	3.42E-05	0.000261
11739807_MAZ	-0.55524	3.42E-05	0.000261
11758159_RELL2	0.476549	3.42E-05	0.000261
11745213_SMARCB1	-0.37245	3.42E-05	0.000261
11757911_RNF111	0.372257	3.42E-05	0.000261
11737209_IPP	-0.43122	3.42E-05	0.000261
11763862_SH3BGRL	0.328591	3.42E-05	0.000261
11756238_SLC2A1	-0.32867	3.43E-05	0.000261
11754792_RGMA	-0.45539	3.43E-05	0.000261
11725844_SREK1IP1	-0.43906	3.43E-05	0.000261
11719243_NAT9	0.365817	3.43E-05	0.000261
11719603_PTPN9	-0.4733	3.43E-05	0.000261
11754508_COG1	0.413419	3.44E-05	0.000262
11756303_SH3TC1	0.389984	3.44E-05	0.000262
11728960_KRT80	0.299317	3.44E-05	0.000262
11763275_ITSN2	0.363952	3.44E-05	0.000262
11753600_H1F0	-0.49485	3.44E-05	0.000262
11754385_WASL	0.36236	3.45E-05	0.000262

11758343_USMG5	-0.29719	3.45E-05	0.000262
11747590_FCMR	0.43183	3.45E-05	0.000262
11751869_SDHC	-0.47272	3.45E-05	0.000263
11741423_MIER1	0.437697	3.46E-05	0.000263
11730990_KIAA1919	0.390401	3.46E-05	0.000263
200073_PM HNRNPD	-0.37137	3.46E-05	0.000263
11719610_ADO	0.309016	3.47E-05	0.000263
11747989_TEAD2	-0.32876	3.47E-05	0.000263
11756492_ZBTB48	0.628598	3.47E-05	0.000264
11755303_HLA-A	0.315302	3.48E-05	0.000264
11716192_SETD2	0.487355	3.48E-05	0.000264
11719547_GOLGA1	0.472568	3.48E-05	0.000264
11763244_CD59	0.415998	3.49E-05	0.000265
11731509_DBT	0.335984	3.50E-05	0.000265
11730928_IKBKB	-0.42992	3.50E-05	0.000265
11750073_WBSCR27	-0.56219	3.50E-05	0.000265
11733828_C19orf48	-0.36262	3.50E-05	0.000266
11722870_RAD1	-0.39661	3.51E-05	0.000266
11727135_PUS7L	0.322588	3.51E-05	0.000266
11743115_KPNB1	-0.33499	3.52E-05	0.000267
11743896_RCOR3	0.379493	3.52E-05	0.000267
11732995_RGS4	0.482687	3.52E-05	0.000267
11726552_ULBP2	-0.37295	3.52E-05	0.000267
11752556_OSMR	0.739795	3.52E-05	0.000267
11754802_C12orf76	0.436905	3.52E-05	0.000267
11756394_KTN1	0.304606	3.53E-05	0.000267
11732331_ARL4C	0.542609	3.53E-05	0.000267
11729610_ELMO1	-0.53545	3.53E-05	0.000267
11756930_DDX11	-0.44026	3.53E-05	0.000267
11725039_FGF2	0.397211	3.53E-05	0.000267
11752429_LRIG3	0.374025	3.54E-05	0.000268
11715960_NCBP2	-0.36021	3.54E-05	0.000268
11752131_RTN4	-0.33284	3.54E-05	0.000268
11748030_STK25	-0.37205	3.54E-05	0.000268
11750871_PAPSS2	-0.37052	3.54E-05	0.000268
11723690_CREBL2	0.326846	3.54E-05	0.000268
11760514_CKAP2	-0.41237	3.55E-05	0.000268
11760999_ARHGEF28	0.723161	3.55E-05	0.000268
11747523_TRAF4	0.494491	3.55E-05	0.000268
11736470_SLC35D1	-0.34663	3.55E-05	0.000268
11723433_TBC1D22A	-0.40135	3.55E-05	0.000268
11737972_RAB39A	0.63072	3.55E-05	0.000268
11754376_JMJD8	-0.33281	3.55E-05	0.000268
11723411_PLP1	-0.35067	3.55E-05	0.000269
11750576_KDM3A	0.365133	3.56E-05	0.000269
11756260_ANKIB1	0.373294	3.56E-05	0.000269
11723151_SUGCT	0.56906	3.56E-05	0.000269
11757830_HNRNPA3	-0.32522	3.56E-05	0.000269
11756377_DOK4	-0.41343	3.56E-05	0.000269
11755553_WDR1	0.329937	3.56E-05	0.000269
11722262_EDEM1	0.333076	3.56E-05	0.000269

11754521_MBNL2	0.384688	3.57E-05	0.000269
11722410_GALNT7	0.372785	3.58E-05	0.00027
11724600_DCAF10	0.381573	3.59E-05	0.00027
11750197_FAM98A	0.34631	3.59E-05	0.000271
11748962_PATL1	0.379906	3.59E-05	0.000271
11732367_FGF14	0.490063	3.59E-05	0.000271
11759316_NFIC	-0.37017	3.59E-05	0.000271
11754085_PMVK	-0.68461	3.59E-05	0.000271
11749485_SEC23A	0.327122	3.60E-05	0.000272
11720526_PEBP1	-0.3964	3.61E-05	0.000272
11754131_BANF1	-0.80194	3.61E-05	0.000272
11729715_CBR1	-0.37174	3.61E-05	0.000272
11736792_NXT2	-0.31369	3.61E-05	0.000272
11737095_CFL2	0.33854	3.61E-05	0.000272
11719406_HIPK2	0.381172	3.62E-05	0.000273
11716516_ADD1	-0.44795	3.62E-05	0.000273
11723217_SFXN3	-0.33738	3.63E-05	0.000273
11749357_TRMT10B	0.557987	3.64E-05	0.000274
11719641_CDS1	0.544144	3.64E-05	0.000274
11735995_USP14	-0.43069	3.65E-05	0.000274
11751401_PRPSAP1	-0.3446	3.65E-05	0.000274
11744942_SLC22A18A	0.340728	3.65E-05	0.000274
11744783_SP100	0.405935	3.65E-05	0.000274
11721002_HEXIM1	-0.32659	3.65E-05	0.000274
11755344_HLA-A	0.301483	3.65E-05	0.000274
11757372_NUDT1	-0.45198	3.65E-05	0.000274
11719025_ZNF652	0.501614	3.66E-05	0.000275
11718992_FKBP2	-0.49429	3.66E-05	0.000275
11726660_GPN3	-0.5086	3.66E-05	0.000275
11718531_CAD	-0.34585	3.66E-05	0.000275
11730207_ARAP2	0.640034	3.66E-05	0.000275
11754283_RANBP2	0.293016	3.66E-05	0.000275
11737076_PPP2R5C	-0.36009	3.66E-05	0.000275
11736385_METTL10	-0.89238	3.66E-05	0.000275
11758593_H3F3B	-0.4037	3.67E-05	0.000275
11727057_RRP8	0.404662	3.67E-05	0.000275
11740404_GALNT10	0.410411	3.67E-05	0.000275
11716453_MYO1C	0.329137	3.67E-05	0.000275
11718862_SEC63	-0.85537	3.67E-05	0.000275
11727985_TENM3	0.316194	3.67E-05	0.000275
11718544_ZBTB44	-0.45001	3.68E-05	0.000276
11743283_TIMM10	-0.33431	3.68E-05	0.000276
11720262_MITF	0.449379	3.68E-05	0.000276
11731654_ARL15	0.422344	3.68E-05	0.000276
11727646_DNAJC25	0.425638	3.68E-05	0.000276
11763276_PWP1	-0.37201	3.68E-05	0.000276
11758048_THRB	0.34513	3.68E-05	0.000276
11733367_C19orf60	-0.3892	3.69E-05	0.000276
11715564_H1FX	-0.34545	3.69E-05	0.000276
11745182_NAA15	-0.39039	3.69E-05	0.000276
11751711_SLC48A1	0.322355	3.69E-05	0.000276

11731495_GDAP2	0.348803	3.69E-05	0.000276
11749872_MKRN2	0.331009	3.69E-05	0.000276
11736364_KIAA0586	-0.41596	3.69E-05	0.000277
11753131_TM4SF1	0.32398	3.70E-05	0.000277
11743671_ZNF292	0.46034	3.70E-05	0.000277
11753588_RTN4	-0.31909	3.70E-05	0.000277
11742454_PHF20L1	0.372158	3.70E-05	0.000277
11749711_XPNPEP3	0.568409	3.71E-05	0.000277
11721879_ARID4B	0.507742	3.71E-05	0.000277
11728608_NSUN5P1 /	0.450285	3.72E-05	0.000278
11725867_EBF3	-0.32777	3.72E-05	0.000278
11730852_ELMO1	-0.4102	3.72E-05	0.000278
11717539_RYBP	0.341683	3.72E-05	0.000278
11754766_MRPL32	0.299951	3.73E-05	0.000279
11721128_DNAJC2	0.372467	3.73E-05	0.000279
11726656_IFIT5	0.381554	3.73E-05	0.000279
11734281_ZNF17	0.451589	3.74E-05	0.000279
11763964_MTG2	0.417363	3.74E-05	0.000279
11759922_PARD3	0.508015	3.74E-05	0.000279
11729465_ZNF816	0.667144	3.74E-05	0.00028
11747719_KIAA0391	-0.35855	3.75E-05	0.00028
11760321_ZMYM6	0.473397	3.75E-05	0.00028
11725931_HSPA5	-0.37343	3.75E-05	0.00028
11716649_DHX40	-0.35452	3.75E-05	0.00028
11759194_TXLNG	0.422297	3.76E-05	0.00028
11715630_LAPTM4A	-0.43874	3.76E-05	0.000281
11749997_VSNL1	-0.33316	3.76E-05	0.000281
11720094_CUL4B	0.356493	3.77E-05	0.000281
11716495_MAP4	-0.43444	3.77E-05	0.000281
11727395_COX15	0.406098	3.77E-05	0.000281
11734712_PDE4B	0.527061	3.78E-05	0.000281
11747935_TNC	0.36435	3.78E-05	0.000282
11741458_NR4A3	0.959415	3.78E-05	0.000282
11749708_FLI1	0.393732	3.79E-05	0.000282
11718553_OTUD4	0.585801	3.79E-05	0.000282
11746178_RHBDF1	0.391396	3.79E-05	0.000282
11742691_UBE2I	-0.29486	3.79E-05	0.000282
11754922_ZNF426	0.300355	3.79E-05	0.000282
11754466_SH3BP4	0.385719	3.79E-05	0.000282
11736599_DLG3	-0.31459	3.79E-05	0.000282
11720456_CAPRIN1	-0.35619	3.80E-05	0.000283
11727171_TRPS1	0.363498	3.80E-05	0.000283
11725715_RPS14	-0.29262	3.80E-05	0.000283
11727469_MAGEA4	-0.32204	3.80E-05	0.000283
11716397_AKR7A2	-0.34301	3.81E-05	0.000283
11743403_CDC27	0.32439	3.82E-05	0.000284
11760963_LOC102724	0.337938	3.82E-05	0.000284
11757022_TFPI2	0.414997	3.82E-05	0.000284
11718593_RTN4	-0.2979	3.82E-05	0.000284
11720830_C1orf21	-0.46838	3.82E-05	0.000284
11741227_NR3C1	-0.40772	3.82E-05	0.000284

11720840_SMNDC1	0.417455	3.83E-05	0.000284
11730029_E2F7	0.330772	3.84E-05	0.000285
11719282_UBE2I	-0.33838	3.84E-05	0.000285
11743762_SNIP1	0.461603	3.84E-05	0.000285
11745855_RNF145	0.466101	3.84E-05	0.000285
11760648_AKTIP	-0.30073	3.84E-05	0.000285
11746767_ITGA4	0.401439	3.85E-05	0.000286
11763320_GLS	0.343567	3.85E-05	0.000286
11733802_CPPED1	0.43138	3.85E-05	0.000286
11725629_CNP	-0.34729	3.86E-05	0.000286
11733782_RAB18	0.520744	3.86E-05	0.000286
11722107_PCNX	-0.63661	3.87E-05	0.000287
11744344_YY1AP1	0.334173	3.87E-05	0.000287
11744697_CCDC90B	0.379938	3.87E-05	0.000287
11755941_TRAIP	-0.35975	3.87E-05	0.000287
11744384_USMG5	-0.33162	3.87E-05	0.000287
11739654_TNFRSF10A	0.556016	3.87E-05	0.000287
11754306_MCCC1	0.462848	3.87E-05	0.000287
11744368_ZMYND11	0.424027	3.89E-05	0.000288
11744997_MPZL1	0.422064	3.90E-05	0.000289
11720665_SYNCRIP	-0.34119	3.90E-05	0.000289
11744580_ZNF271P	0.597579	3.90E-05	0.000289
11732386_TCEANC2	-0.34604	3.91E-05	0.00029
11737079_SLX4	-0.34978	3.91E-05	0.00029
11723191_ZIC2	-0.39686	3.91E-05	0.00029
11715659_ERRFI1	0.294158	3.91E-05	0.00029
11728374_ACTN1	0.288571	3.91E-05	0.00029
11750383_ZMYND8	-0.41418	3.92E-05	0.00029
11728378_EED	-0.34212	3.92E-05	0.00029
11743079_TRMT6	0.305589	3.92E-05	0.00029
11728326_TTC30A	0.353608	3.93E-05	0.00029
11758592_GAR1	-0.28956	3.93E-05	0.00029
11728634_AP4E1	0.439272	3.93E-05	0.00029
11715881_DAP3	0.365741	3.93E-05	0.000291
11730932_ACSS3	0.401724	3.94E-05	0.000291
11745827_SUPT20H	0.303528	3.94E-05	0.000291
11743648_DCAF6	0.361533	3.95E-05	0.000291
11729507_NPAS3	0.668003	3.95E-05	0.000292
11749828_DPH2	0.31134	3.95E-05	0.000292
11725341_SAR1B	0.333297	3.95E-05	0.000292
11763313_SEPHS1	-0.49045	3.95E-05	0.000292
11736308_CSNK1A1	0.346338	3.96E-05	0.000292
11719548_GOLGA1	0.443038	3.96E-05	0.000292
11718208_TMEM9B	0.332769	3.96E-05	0.000292
11743022_CLCC1	0.338689	3.96E-05	0.000292
11749930_AP3M2	-0.34971	3.97E-05	0.000293
11717155_NME1 ///	-0.31893	3.97E-05	0.000293
11727005_ADGRL4	0.404217	3.97E-05	0.000293
11718543_ZBTB44	0.410725	3.98E-05	0.000293
11723659_UBIAD1	0.395394	3.98E-05	0.000293
11742059_CHORDC1	0.379654	3.98E-05	0.000293

11753374_XPO4	0.393048	3.98E-05	0.000294
11718387_RBSN	0.327327	3.99E-05	0.000294
200090_PMFNTA	0.328452	4.00E-05	0.000295
11743324_CCDC50	0.40398	4.00E-05	0.000295
11755404_PHF20	-0.31397	4.00E-05	0.000295
11718081_ATP2B4	-0.32527	4.01E-05	0.000295
11744273_SLBP	-0.34674	4.01E-05	0.000295
11730549_IKZF2	1.020939	4.01E-05	0.000295
11716625_ANAPC13	0.299604	4.01E-05	0.000295
11723763_POLG2	0.369029	4.01E-05	0.000295
11753001_PPARG	-0.3229	4.01E-05	0.000295
11757627_COX17	-0.34266	4.01E-05	0.000295
11716131_FSTL3	0.322482	4.02E-05	0.000296
11722028_UBE2G1	0.317366	4.02E-05	0.000296
11746122_LOC441155	0.291887	4.03E-05	0.000296
11750384_APP	0.312513	4.03E-05	0.000296
11744670_LINC00852	-0.32283	4.03E-05	0.000296
11733954_SLC22A15	0.619462	4.03E-05	0.000296
11744667_DENND5B	0.43707	4.04E-05	0.000297
11757549_C16orf58	-0.29857	4.04E-05	0.000297
11726040_STK17B	0.634762	4.04E-05	0.000297
11718768_LAMTOR3	0.312896	4.04E-05	0.000297
11721895_GAS1	-0.42101	4.05E-05	0.000297
11743366_PLCB4	0.752614	4.05E-05	0.000297
11715653_RTFDC1	-0.43264	4.05E-05	0.000297
11752144_KMT5B	0.470501	4.05E-05	0.000297
11754071_TAGLN2	-0.29744	4.05E-05	0.000297
11756927_SMARCAL1	-0.44076	4.05E-05	0.000297
11744618_DUSP6	0.373795	4.05E-05	0.000297
11739632_MED13	0.704277	4.05E-05	0.000297
11749873_MKRN2	0.332245	4.05E-05	0.000297
11759323_CEP85L	0.627102	4.05E-05	0.000297
11724107_MRPS31P5	0.452601	4.06E-05	0.000298
11739932_FAM111B	-0.77685	4.06E-05	0.000298
11726749_GTF3C4	0.356544	4.06E-05	0.000298
11721587_TRAF1	0.503065	4.07E-05	0.000298
11738872_MAPK1	0.372697	4.07E-05	0.000298
11723239_GNG7 ///	-0.65155	4.07E-05	0.000298
11753947_TTC21B	0.380722	4.07E-05	0.000298
11746760_ERGIC1	-0.84992	4.07E-05	0.000298
11763618_SNAPC4	0.326017	4.07E-05	0.000298
11739237_PDE12	0.319786	4.08E-05	0.000299
11751117_ACTN1	0.377545	4.08E-05	0.000299
11748549_ZDHHC17	0.44294	4.08E-05	0.000299
11725015_MAP9	0.437912	4.08E-05	0.000299
11719048_CPNE1	0.28873	4.08E-05	0.000299
11752733_CSNK1G3	0.403108	4.08E-05	0.000299
11763455_TACC3	-0.39289	4.08E-05	0.000299
11745770_RNPS1	-0.38053	4.09E-05	0.000299
11755190_POLR1D	0.411765	4.09E-05	0.000299
11757873_ATP5B	-0.30137	4.09E-05	0.000299

11750051_CAST	0.335644	4.09E-05	0.000299
11745019_CBS /// C	-0.51928	4.09E-05	0.000299
11716274_ENSA	-0.38542	4.09E-05	0.000299
11743501_TRAF3	0.335195	4.09E-05	0.000299
11732252_RCOR3	0.42278	4.10E-05	0.000299
11741651_POT1	-0.38755	4.10E-05	0.0003
11724210_LMAN1	0.362535	4.10E-05	0.0003
11737922_SERPINB8	0.358769	4.10E-05	0.0003
11754799_NMD3	0.404083	4.10E-05	0.0003
11731561_APC	0.407687	4.10E-05	0.0003
11725301_DDX19A	0.354442	4.11E-05	0.0003
11752331_LOC105369	0.31359	4.11E-05	0.0003
11734295_ZNF772	-0.3805	4.12E-05	0.000301
11741555_LIPA	-0.35464	4.12E-05	0.000301
11739284_TRIM23	0.491576	4.13E-05	0.000301
11728499_SVIL	0.381898	4.13E-05	0.000301
11737769_MYO1C	0.370319	4.13E-05	0.000302
11720349_ROPN1L	1.141913	4.13E-05	0.000302
11721549_POLR3C	0.376935	4.13E-05	0.000302
11727591_OSGEPL1	-0.33143	4.14E-05	0.000302
11737272_ZNF805	0.558053	4.14E-05	0.000302
11739241_NEMF	0.342773	4.14E-05	0.000302
11755240_NR3C1	-0.34837	4.15E-05	0.000302
11745939_DNAJC6	0.666844	4.15E-05	0.000302
11755582_ZFP30	0.725827	4.15E-05	0.000302
11743284_CHD2	0.327598	4.15E-05	0.000302
11729094_GAD1	0.427426	4.15E-05	0.000302
11757931_KIAA0930	-0.34227	4.15E-05	0.000302
11723606_AGFG1	0.38802	4.15E-05	0.000302
11758796_ZZZ3	-0.44237	4.16E-05	0.000303
11724221_NAP1L2	0.516223	4.16E-05	0.000303
11747752_MORF4L1	0.286179	4.16E-05	0.000303
11754643_TMEM234	0.505629	4.17E-05	0.000303
11734825_SPRED1	0.36131	4.17E-05	0.000303
11748501_LOC105376	0.556428	4.17E-05	0.000303
11736659_MAP3K13	0.423099	4.17E-05	0.000303
11726052_FAM35A //	0.296958	4.18E-05	0.000304
11750029_EIF4H	-0.32956	4.18E-05	0.000304
11743342_RMND5A	-0.64871	4.18E-05	0.000304
11744699_TDP1	-0.33811	4.18E-05	0.000304
11744083_ANKIB1	0.357721	4.19E-05	0.000304
11726377_FAM50B	-0.33295	4.19E-05	0.000305
11733491_SLC44A5	0.844843	4.19E-05	0.000305
11754800_GFM1	0.310563	4.19E-05	0.000305
11737629_TLR6	0.455591	4.19E-05	0.000305
11722791_MATN2	0.540388	4.20E-05	0.000305
11719975_WASF3	0.492789	4.20E-05	0.000305
11757775_C6orf120	0.549063	4.20E-05	0.000305
11730744_AKIRIN1	0.380272	4.21E-05	0.000306
11741476_MAP3K7	0.305445	4.21E-05	0.000306
11738058_HIST1H2AD	0.472629	4.21E-05	0.000306

11720414_CAMSAP1	-0.53053	4.21E-05	0.000306
11744560_IFT20	0.350097	4.21E-05	0.000306
11735359_LOC101060	0.420839	4.22E-05	0.000307
11736610_RIC1	0.472517	4.23E-05	0.000307
11740121_LRIG2	0.419556	4.23E-05	0.000307
11752437_KCTD8	0.650463	4.23E-05	0.000307
11758891_TMEM183A	-0.52216	4.24E-05	0.000307
11726190_DOCK4	0.357639	4.24E-05	0.000308
11760989_GLS	0.537292	4.24E-05	0.000308
11749156_ORC3	-0.30758	4.24E-05	0.000308
11763307_NFATC2	-0.3734	4.25E-05	0.000308
11760133_SUPT20H	0.360447	4.25E-05	0.000308
11735788_KCNG1	-0.3163	4.25E-05	0.000308
11722413_SLC7A1	-0.31183	4.25E-05	0.000308
11743928_NSUN3	0.389254	4.25E-05	0.000308
11745908_EFCAB14	0.554898	4.25E-05	0.000308
11764029_CEBPD	0.632656	4.25E-05	0.000308
11730509_ABCC5	0.44445	4.25E-05	0.000308
11743792_TTC39C	0.339007	4.25E-05	0.000308
11741775_IL24	0.647463	4.26E-05	0.000308
11736368_MCM10	-0.50602	4.26E-05	0.000309
11754457_GOLGB1	-0.36911	4.27E-05	0.000309
11721472_HCCS	0.338814	4.27E-05	0.000309
11721912_MDM4	-0.38706	4.27E-05	0.000309
11723472_CHM	0.394427	4.27E-05	0.000309
11726003_KRCC1	0.478291	4.27E-05	0.000309
11743941_PPP1R21	0.490421	4.28E-05	0.000309
11729830_DCLRE1A	-0.36206	4.28E-05	0.000309
11717869_ZNF263	0.467222	4.28E-05	0.000309
11722992_DMD	0.471649	4.28E-05	0.000309
11720090_NRSN2	-0.54718	4.28E-05	0.000309
11719045_PER3	-0.55013	4.29E-05	0.00031
11756175_NSMCE4A	-0.38355	4.29E-05	0.00031
11734263_ZNF780A	0.394201	4.29E-05	0.00031
11735315_DYNC2LI1	0.507219	4.30E-05	0.00031
11731947_PAQR9	-0.44534	4.30E-05	0.000311
11740939_SUMO1	-0.40928	4.30E-05	0.000311
11754902_POLH	0.446714	4.30E-05	0.000311
11720407_PPP6R2	0.314485	4.31E-05	0.000311
11719637_DUSP22	-0.53144	4.31E-05	0.000311
11718537_FAHD2A //	-0.36889	4.31E-05	0.000311
11747946_ACTR1A	-0.35977	4.31E-05	0.000311
11758023_GABARAPL1	0.488118	4.31E-05	0.000311
11747968_PARG	-0.56053	4.31E-05	0.000311
11743345_FTSJ1	0.313709	4.32E-05	0.000311
11736468_SLC35D1	-0.43383	4.32E-05	0.000311
11741075_GALNT11	-0.44263	4.32E-05	0.000312
11723453_RIOK2	0.317342	4.32E-05	0.000312
11751061_TCOF1	-0.47233	4.33E-05	0.000312
11725805_ZNF606	0.502271	4.33E-05	0.000312
11716914_MTUS1	0.318289	4.33E-05	0.000312

11752783_FLRT2	0.732601	4.33E-05	0.000312
11728615_GPR3	-0.35439	4.34E-05	0.000312
11749425_BRD8	-0.29616	4.34E-05	0.000313
11719256_LRP6	0.609063	4.35E-05	0.000313
11742557_ARPP19	0.434193	4.35E-05	0.000314
11742210_APP	0.347391	4.36E-05	0.000314
11734014_SMAD2	0.298914	4.36E-05	0.000314
11757111_ACER2	0.823084	4.36E-05	0.000314
11722800_SS18	0.45174	4.36E-05	0.000314
11756627_TMEM222	-0.43494	4.36E-05	0.000314
11741059_SATB1	0.51442	4.36E-05	0.000314
11728249_TSFM	-0.58078	4.36E-05	0.000314
11731537_FHL5	0.780028	4.37E-05	0.000314
11734122_TONSL	-0.37159	4.37E-05	0.000314
11717643_MRPL24	-0.3139	4.38E-05	0.000315
11721052_KLHL42	-0.28227	4.38E-05	0.000315
11718489_ORC3	-0.34725	4.38E-05	0.000315
11744294_FAM195B	-0.39467	4.38E-05	0.000315
11748368_POR	-0.46853	4.38E-05	0.000315
11722795_GDE1	0.309901	4.39E-05	0.000316
11728794_LDLRAD3	-0.3697	4.39E-05	0.000316
11759560_SLC25A37	-0.43938	4.39E-05	0.000316
11727198_PCDHA1 //	0.498659	4.40E-05	0.000316
11722079_RHBDF1	0.311791	4.40E-05	0.000316
11718011_MRPL4	-0.3293	4.40E-05	0.000316
11727456_OFD1	0.293586	4.40E-05	0.000316
11758719_RPS25	-0.45975	4.40E-05	0.000316
11716957_CTDSP2	0.296453	4.40E-05	0.000316
11717072_KTN1	0.283347	4.40E-05	0.000316
11756210_RCL1	0.321222	4.41E-05	0.000316
11719053_CEP350	0.436174	4.41E-05	0.000316
11719434_ETV4	-0.31686	4.41E-05	0.000316
11742923_LAPTM5	0.884593	4.42E-05	0.000317
11735487_HOXC8	0.353753	4.42E-05	0.000317
11746935_WARS2	0.416376	4.42E-05	0.000317
11737882_XRCC2	-0.62962	4.42E-05	0.000317
11725566_MANEA	-0.48393	4.43E-05	0.000317
11752780_TANK	0.341391	4.43E-05	0.000318
11752044_ANKHD1 //	0.335002	4.43E-05	0.000318
11737219_ITGB1	-0.45121	4.43E-05	0.000318
11719731_TIGAR	0.395667	4.43E-05	0.000318
11762978_SMN1 ///	0.306125	4.44E-05	0.000318
11728255_GLTSCR2	0.313668	4.44E-05	0.000318
11734572_HIST1H1E	-0.69476	4.44E-05	0.000318
11729501_ASCC3	0.562972	4.45E-05	0.000318
11715811_MLLT6	-0.35348	4.45E-05	0.000318
11743405_SMIM14	-0.45714	4.45E-05	0.000318
11721677_RBM17	-0.37919	4.45E-05	0.000318
11716675_ZNF106	0.371617	4.45E-05	0.000318
11740899_PPA2	-0.3142	4.46E-05	0.000319
11744630_NXPE3	0.340783	4.46E-05	0.000319

11738597_CTCFL	-0.37361	4.46E-05	0.000319
11733108_GNAQ	0.39093	4.46E-05	0.000319
11718810_PRKCH	0.651189	4.46E-05	0.000319
11742198_NF2	0.35737	4.47E-05	0.000319
11721081_PARVA	0.335729	4.47E-05	0.000319
11727146_GPR160	-0.5254	4.47E-05	0.00032
11744663_TOM1L1	0.372357	4.47E-05	0.00032
11718408_TMBIM6	0.285378	4.47E-05	0.00032
11750530_ASB9	-0.30103	4.47E-05	0.00032
11723576_OSR2	0.415768	4.48E-05	0.00032
11756557_LRPAP1	0.319368	4.49E-05	0.000321
11758482_HS3ST3A1	-0.48138	4.49E-05	0.000321
11754771_LMBR1	0.326037	4.49E-05	0.000321
11728768_KLHL5	0.428232	4.49E-05	0.000321
11744755_FHL3	0.365523	4.50E-05	0.000321
11739712_PHACTR2	0.392162	4.50E-05	0.000321
11730325_DGCR6 ///	-0.36141	4.50E-05	0.000321
11749943_METTL6	0.328589	4.51E-05	0.000321
11758253_CDK5RAP2	-0.36373	4.51E-05	0.000322
11746031_PTPMT1	-0.45638	4.51E-05	0.000322
11724236_RIPK2	0.464873	4.52E-05	0.000322
11739176_ABHD17B	0.35114	4.52E-05	0.000322
11753152_CFLAR	0.395358	4.53E-05	0.000323
11753518_YWHAE	-0.33929	4.53E-05	0.000323
11729984_FUT10	0.471704	4.53E-05	0.000323
11734399_VPS13C	0.611275	4.54E-05	0.000323
11743038_CRTAP	-0.63561	4.54E-05	0.000323
11729516_ZNF644	0.326828	4.54E-05	0.000323
11724378_PAG1	0.522365	4.54E-05	0.000323
11744495_IFT46	-0.30016	4.54E-05	0.000323
11758517_ZNF274	0.445465	4.54E-05	0.000323
11730733_DNASE2	-0.32472	4.54E-05	0.000323
11728966_ZNF28 ///	0.660552	4.54E-05	0.000323
11755953_DMRTA1	0.784063	4.55E-05	0.000323
11748885_RNPS1	-0.34094	4.55E-05	0.000323
11756434_DNMT1	-0.3175	4.55E-05	0.000323
11760741_MPI	0.432956	4.55E-05	0.000324
11755623_ARSK	0.6218	4.55E-05	0.000324
11754653_BACE1	0.437998	4.55E-05	0.000324
11762283_TMX3	0.649723	4.56E-05	0.000324
11720941_DDX54	-0.3297	4.56E-05	0.000324
11758167_PHTF2	0.333336	4.56E-05	0.000324
11752922_TUBB	-0.29921	4.56E-05	0.000324
11721286_PREP	0.375037	4.57E-05	0.000325
11739423_DPYSL5	-0.40593	4.57E-05	0.000325
11729857_H6PD	-0.53598	4.57E-05	0.000325
11752061_INTS6	0.388906	4.57E-05	0.000325
11727998_MYEF2	-0.49355	4.57E-05	0.000325
11735778_SYNE2	-0.4997	4.57E-05	0.000325
11719828_QPCT	0.4291	4.58E-05	0.000325
11734913_HIST2H2BF	0.635334	4.58E-05	0.000325

11720880_EHD4	0.294569	4.58E-05	0.000325
11720749_INMT	0.324497	4.59E-05	0.000326
11758797_SRSF10	-0.85957	4.59E-05	0.000326
11736195_CREB1	-0.46169	4.60E-05	0.000326
11763872_SPARC	0.295586	4.60E-05	0.000326
11715486_MCL1	0.572307	4.60E-05	0.000327
11716517_ADD1	-0.34911	4.61E-05	0.000327
11718353_ERP44	-0.31331	4.61E-05	0.000327
11745882_CDK8	-0.41176	4.61E-05	0.000327
11739937_DYRK3	0.297137	4.61E-05	0.000327
11751835_LTV1	0.401278	4.61E-05	0.000327
11731804_DCUN1D3	0.553831	4.62E-05	0.000327
11718148_APH1A	-0.34398	4.62E-05	0.000327
11735385_DACT1	0.663361	4.62E-05	0.000327
11749990_HLA-A	0.324313	4.62E-05	0.000327
11758781_EMP1	-0.63973	4.63E-05	0.000328
11741068_GTF2IRD2	0.44867	4.63E-05	0.000328
11747292_HMGN1	-0.35824	4.63E-05	0.000328
11745278_SYNJ1	0.765677	4.64E-05	0.000329
11727948_RAB28	-0.30463	4.64E-05	0.000329
11736976_ZBED9	0.651949	4.65E-05	0.000329
11752200_APP	0.286088	4.65E-05	0.000329
11756741_SH3BGRL	0.433042	4.65E-05	0.000329
11739602_BIRC5	-0.3297	4.65E-05	0.000329
11728227_WDR45	0.3611	4.65E-05	0.000329
11717512_SEPT8	0.358139	4.65E-05	0.000329
11753187_SMARCB1	-0.33382	4.65E-05	0.000329
11743970_ACTN1	0.36069	4.65E-05	0.000329
11747813_ABHD14B	-0.45045	4.65E-05	0.000329
11736060_KIF5B	0.364967	4.65E-05	0.000329
11757313_VAT1	-0.30084	4.66E-05	0.000329
11751929_MYPN	0.884691	4.66E-05	0.000329
11720434_CLCN6	0.287641	4.66E-05	0.000329
11715471_SRPR	-0.56743	4.67E-05	0.00033
11723958_LSM5	-0.31384	4.67E-05	0.00033
11729534_BRE	0.322499	4.67E-05	0.00033
11728739_TBC1D20	0.351757	4.67E-05	0.00033
11729593_LLGL1	-0.30197	4.67E-05	0.00033
11723672_PSEN2	0.453717	4.68E-05	0.00033
11717871_KANK2	-0.29234	4.68E-05	0.00033
11715233_SPATC1L	-0.43097	4.68E-05	0.00033
11722399_DAPK3	0.484365	4.68E-05	0.000331
11723808_STX17	0.340221	4.69E-05	0.000331
11747499_ICAM1	0.428703	4.69E-05	0.000331
11720935_SNRPB	-0.31527	4.69E-05	0.000331
11721140_TCEA3	0.405011	4.70E-05	0.000331
11723607_AGFG1	-0.43995	4.70E-05	0.000332
11729187_PNO1	0.447415	4.71E-05	0.000332
11757674_ZMYND8	-0.37991	4.71E-05	0.000332
11721855_PIGK	-0.4073	4.71E-05	0.000332
11739242_NEMF	0.386758	4.72E-05	0.000332

11755290_FBXL18	-0.30819	4.72E-05	0.000332
11753378_CDK4	-0.32352	4.72E-05	0.000332
11727648_DDX11 ///	-0.31121	4.72E-05	0.000333
11749582_WDR37	0.594725	4.72E-05	0.000333
11754434_CCND3	-0.31856	4.72E-05	0.000333
11729892_ZNF569	0.396374	4.73E-05	0.000333
11739746_SVIL	0.413501	4.73E-05	0.000333
11723588_RC3H1	0.299148	4.73E-05	0.000333
11744590_TRIO	0.585048	4.73E-05	0.000333
11726541_NGLY1	0.343709	4.73E-05	0.000333
11758001_YWHAQ	-0.3867	4.73E-05	0.000333
11721778_ACBD5	0.422469	4.74E-05	0.000333
11758105_CREBBP	0.308666	4.74E-05	0.000334
11752821_ACTR1A	-0.34896	4.74E-05	0.000334
11741324_TRIM51 //	0.523024	4.75E-05	0.000334
11754066_NPM1	-0.29922	4.75E-05	0.000334
11754343_FAM73B	0.392931	4.77E-05	0.000335
11749615_UBE2I	-0.28511	4.77E-05	0.000335
11752668_CNOT4	0.375495	4.77E-05	0.000335
11718490_FABP5 ///	-0.30221	4.77E-05	0.000335
11718286_UBL3	0.314897	4.77E-05	0.000335
11735139_TNFSF9	0.436279	4.78E-05	0.000336
11718062_NME3	-0.33403	4.78E-05	0.000336
11763342_CEP57	-0.48573	4.79E-05	0.000336
11745634_EDRF1	0.423597	4.79E-05	0.000337
11727540_INTS7	0.330987	4.80E-05	0.000337
11719469_PRPF31	-0.31911	4.80E-05	0.000337
11721072_SF3A1	-0.31064	4.80E-05	0.000337
11750992_SHMT2	-0.3765	4.81E-05	0.000337
11724150_SWI5	-0.49063	4.81E-05	0.000337
11757554_PPT2	-0.34191	4.81E-05	0.000338
11746722_ZMYND8	-0.37448	4.81E-05	0.000338
11726287_WARS	-0.29261	4.82E-05	0.000338
11721077_ESD	-0.3309	4.82E-05	0.000338
11757436_IFITM2	0.300781	4.83E-05	0.000339
11732338_KAT8	-0.41775	4.83E-05	0.000339
11749881_PEX1	0.366358	4.83E-05	0.000339
11759893_PLEKHA8 /	0.426291	4.84E-05	0.000339
11719512_PHC1	-0.36174	4.86E-05	0.00034
11725583_FBXO10	0.314141	4.86E-05	0.00034
11727269_S100A3	0.312017	4.86E-05	0.00034
11736262_INHBE	-0.83364	4.86E-05	0.00034
11758579_FLVCR1-AS	-0.3327	4.86E-05	0.000341
11756331_BFAR	-0.35726	4.87E-05	0.000341
11745951_CCNL2	0.305982	4.87E-05	0.000341
11756934_STK4	0.61678	4.87E-05	0.000341
11726724_CKAP2L	-0.59539	4.88E-05	0.000341
11722538_TMCO6	-0.35132	4.88E-05	0.000342
11747543_MPDU1	-0.34999	4.89E-05	0.000342
11716450_RFTN1	0.417562	4.89E-05	0.000342
11716164_CIAPIN1	-0.36382	4.89E-05	0.000342

11722991_DMD	0.442274	4.89E-05	0.000342
11746161_FBXL5	0.323784	4.90E-05	0.000342
11754348_KLHDC4 //	0.34914	4.90E-05	0.000342
11747502_MTRF1L	0.384335	4.91E-05	0.000343
11739107_SEC61A1	0.299963	4.91E-05	0.000343
11733007_AMOTL2	0.373717	4.91E-05	0.000343
11739011_PAFAH1B1	-0.28603	4.92E-05	0.000344
11739605_CCDC88A	-0.45774	4.92E-05	0.000344
11743406_SMIM14	-0.4177	4.92E-05	0.000344
11726087_HSPB3	0.495407	4.92E-05	0.000344
11737237_EOMES	-0.61778	4.93E-05	0.000344
11724644_SPOPL	0.71254	4.93E-05	0.000344
11720400_NCS1	-0.94988	4.94E-05	0.000345
11760226_ANKRD42	0.466549	4.94E-05	0.000345
11746136_LTV1	0.394633	4.94E-05	0.000345
11737113_MED19	0.321212	4.94E-05	0.000345
11726065_RPE	-0.29909	4.94E-05	0.000345
11719666_STMN1	-0.33316	4.95E-05	0.000345
11757542_SSR1	-0.37647	4.95E-05	0.000345
11729190_MAX	0.329656	4.95E-05	0.000345
11732665_VSTM1	0.546581	4.95E-05	0.000345
11752851_PPHLN1	0.417536	4.95E-05	0.000345
11747507_NAP1L4	-0.3865	4.96E-05	0.000346
11725870_ZBTB41	0.38963	4.96E-05	0.000346
11752570_RIN2	0.539635	4.97E-05	0.000346
11729846_BST1	0.34451	4.97E-05	0.000346
11753130_TM4SF1	0.299628	4.97E-05	0.000347
11737396_TROVE2	0.310206	4.98E-05	0.000347
11719346_ARHGAP19	-0.49025	4.98E-05	0.000347
11740702_HMGB2	-0.42555	4.99E-05	0.000347
11716156_B4GALT1	-0.36726	4.99E-05	0.000347
11721119_ANKHD1 //	0.367601	4.99E-05	0.000348
11754189_AKR1C1 //	-0.47594	5.00E-05	0.000348
11756905_DCBLD1	0.584113	5.00E-05	0.000348
11717001_YTHDC1	0.366675	5.01E-05	0.000349
11727999_MYEF2	-0.52073	5.01E-05	0.000349
11758053_NCOA4	0.302121	5.01E-05	0.000349
11762627_NBPF8	0.31883	5.02E-05	0.000349
11752797_PCMTD2	-0.47281	5.02E-05	0.000349
11749912_IGFBP3	0.411509	5.03E-05	0.00035
11723635_MMP3	1.434931	5.03E-05	0.00035
11720739_ITPKB	0.320112	5.04E-05	0.00035
11721983_PID1	0.551385	5.04E-05	0.000351
11752142_FLNA	-0.30805	5.04E-05	0.000351
11734203_HBS1L	0.471194	5.05E-05	0.000351
11741833_KLHL23 //	-0.45768	5.05E-05	0.000351
11716746_FAM60A	0.294445	5.05E-05	0.000351
11727890_PFDN6	-0.39567	5.05E-05	0.000351
11747162_FLT1	0.51069	5.05E-05	0.000351
11715956_ARL6IP5	0.310445	5.06E-05	0.000351
11741655_C1orf198	0.384153	5.06E-05	0.000351

11719933_RTN4	-0.30962	5.06E-05	0.000351
11723598_MAP2K7	-0.34037	5.06E-05	0.000351
11741436_SPRYD7	0.29528	5.06E-05	0.000352
11730199_AHCY	-0.29425	5.07E-05	0.000352
11715751_DPYSL3	-0.35229	5.07E-05	0.000352
11720638_ACBD6 ///	0.315201	5.07E-05	0.000352
11747464_SERINC3	0.309075	5.08E-05	0.000352
11735774_ANAPC10	-0.41185	5.08E-05	0.000352
11754530_CCDC14	-0.3545	5.08E-05	0.000352
11718649_SYNE2	-0.36863	5.09E-05	0.000353
11746459_KMT2C	0.488583	5.09E-05	0.000353
11751488_WWC2	0.411397	5.09E-05	0.000353
11733372_JAK2	0.436039	5.10E-05	0.000354
11723162_ZNF776	0.529093	5.10E-05	0.000354
11716858_MRPL51	-0.28536	5.10E-05	0.000354
11720039_VTA1	0.313815	5.11E-05	0.000354
11743001_ADD2	-0.46924	5.11E-05	0.000354
11722895_PIK3C2A	0.352831	5.11E-05	0.000354
11747420_ARF3	-0.5363	5.11E-05	0.000354
11723077_PBDC1	0.378453	5.12E-05	0.000354
11726201_OAS2	-0.5994	5.12E-05	0.000354
11747759_MOSPD1	0.537917	5.12E-05	0.000354
11729794_UGGT1	0.290359	5.12E-05	0.000354
11743789_FRS2	-0.60714	5.12E-05	0.000354
11732663_OSR2	0.437191	5.13E-05	0.000355
11756810_MFAP2	0.359491	5.13E-05	0.000355
11749394_ZCCHC10	0.400104	5.13E-05	0.000355
11759041_VKORC1L1	-0.35956	5.13E-05	0.000355
11747925_SEC23A	0.311744	5.14E-05	0.000355
11759592_PCGF5	0.310843	5.14E-05	0.000355
11756270_PKP4	0.391465	5.14E-05	0.000355
11734029_FAM73A	0.53647	5.14E-05	0.000355
11721149_LYSMD2	0.381475	5.14E-05	0.000355
11732215_PEF1	-0.34384	5.14E-05	0.000356
11764001_MRPS11	-0.28834	5.15E-05	0.000356
11756062_CASP8	0.457914	5.15E-05	0.000356
11737138_UBQLN1	0.291641	5.16E-05	0.000356
11746396_PARP4	0.3867	5.16E-05	0.000357
11724296_BMP7	-0.59709	5.16E-05	0.000357
11739926_ANK3	0.464595	5.16E-05	0.000357
11744741_LINC00312	-0.3661	5.16E-05	0.000357
11725276_CDK5RAP2	-0.3043	5.17E-05	0.000357
11715724_ITM2B	0.281553	5.18E-05	0.000357
11731506_RAD23B	-0.30323	5.18E-05	0.000357
11736258_PRKCE	0.693826	5.18E-05	0.000358
11749074_PISD	0.455957	5.18E-05	0.000358
11725729_C1orf56	0.296875	5.19E-05	0.000358
11743614_USP47	-0.64482	5.19E-05	0.000358
11746549_CNN2	0.564462	5.20E-05	0.000358
11748851_UCK1	-0.28847	5.20E-05	0.000358
11718121_ATP6V1A	0.437357	5.20E-05	0.000358

11755155_SMARCB1	-0.3661	5.20E-05	0.000359
11756073_HLA-DPB1	-0.33806	5.20E-05	0.000359
11757951_ALG2	0.318825	5.20E-05	0.000359
11724926_LTN1	0.3003	5.21E-05	0.000359
11732193_MLF1	-0.78943	5.21E-05	0.000359
11763593_ARHGEF28	0.382864	5.21E-05	0.000359
11728873_RBM24	0.671356	5.21E-05	0.000359
11739264_VPS4B	0.394293	5.22E-05	0.000359
11739556_C5orf22	0.410575	5.22E-05	0.00036
11733789_HSDL1	0.322738	5.22E-05	0.00036
11717997_NUP205	-0.32148	5.22E-05	0.00036
11730743_AKIRIN1	0.375689	5.22E-05	0.00036
11727145_KLF11	-0.43332	5.23E-05	0.00036
11755304_LONRF3	0.403826	5.23E-05	0.00036
11749665_DOCK7	0.318024	5.23E-05	0.00036
11741638_PLAG1	0.319835	5.24E-05	0.000361
11733136_SLC39A10	0.450888	5.25E-05	0.000361
11751832_ZNF317	0.608598	5.26E-05	0.000362
11737050_HRH1	0.672706	5.26E-05	0.000362
11739263_VPS4B	0.564078	5.26E-05	0.000362
11759344_FMN1	-0.50419	5.26E-05	0.000362
11741040_DAP3	0.301528	5.26E-05	0.000362
11737077_PPP2R5C	-0.33494	5.28E-05	0.000363
11721090_THBS1	0.373929	5.28E-05	0.000363
11717528_SLC25A20	0.431843	5.28E-05	0.000363
11745440_RELA	0.313906	5.28E-05	0.000363
11717944_FNBP4	0.373818	5.28E-05	0.000363
11723289_CRISPLD1	-0.40392	5.29E-05	0.000363
11760502_WSB1	-0.41533	5.29E-05	0.000364
11754556_EIF2AK4	0.331647	5.30E-05	0.000364
11733105_COMMD2	0.404573	5.30E-05	0.000364
11718047_ITPK1	-0.38359	5.30E-05	0.000364
11729374_KRT34 ///	0.412292	5.31E-05	0.000364
11762108_CXorf40A	-0.45305	5.31E-05	0.000364
11759239_ZNF141	0.567317	5.31E-05	0.000365
11733096_UBL7	-0.37407	5.31E-05	0.000365
11743322_OSER1	0.309818	5.32E-05	0.000365
11725679_SMC3	-0.28603	5.32E-05	0.000365
11757148_RPL23 ///	0.456569	5.32E-05	0.000365
11735184_EXOSC6	0.289288	5.32E-05	0.000365
11741300_GCOM1 ///	0.313529	5.33E-05	0.000365
11719337_SLC23A2	0.382493	5.33E-05	0.000365
11758391_ZADH2	-0.35107	5.34E-05	0.000366
11745173_ATF6	0.306945	5.34E-05	0.000366
11739666_RUBCN	-0.33646	5.34E-05	0.000366
11749386_RBM48	0.408725	5.34E-05	0.000366
11717409_SH2B3	0.285219	5.35E-05	0.000366
11721851_CHCHD6	0.330724	5.35E-05	0.000366
11756011_PTPRJ	-0.37166	5.35E-05	0.000366
11741564_CNNM3	-0.5114	5.35E-05	0.000366
11742952_VIMP	0.324037	5.35E-05	0.000366

11743319_DST	0.427637	5.35E-05	0.000366
11727056_SERPIND1	0.753761	5.35E-05	0.000366
11750151_HAUS6	-0.31347	5.36E-05	0.000367
11729699_STXBP1	-0.30614	5.36E-05	0.000367
11744332_NUMA1	-0.28816	5.36E-05	0.000367
11755467_CHEK2	-0.37312	5.37E-05	0.000367
11759643_NIT1	0.423452	5.37E-05	0.000367
11715598_TFG	0.295532	5.38E-05	0.000368
11716603_KBTBD2	0.346265	5.38E-05	0.000368
11729350_MMP8	0.544906	5.38E-05	0.000368
11736148_LYRM2	0.412686	5.39E-05	0.000368
11724633_BRAP	0.438906	5.39E-05	0.000369
11728111_SARM1	0.31947	5.40E-05	0.000369
11740445_ELF1	0.444488	5.40E-05	0.000369
11723340_TTC28	-0.34131	5.40E-05	0.000369
11727015_PAPPA	1.179784	5.41E-05	0.000369
11735733_RPP14	0.313471	5.41E-05	0.000369
11751977_INPP5B	0.311844	5.41E-05	0.000369
11723265_SAYSD1	-0.38756	5.41E-05	0.00037
11733293_PHF20L1	0.574951	5.42E-05	0.00037
11739262_VPS4B	0.362477	5.42E-05	0.00037
11739171_G2E3	-0.48047	5.43E-05	0.000371
11721112_ACP6	-0.39847	5.43E-05	0.000371
11723391_CORO2A	-0.35414	5.43E-05	0.000371
11722988_BTAF1	0.389363	5.43E-05	0.000371
11718685_TXNRD2	0.452021	5.44E-05	0.000371
11715298_SH2D2A	0.351426	5.44E-05	0.000371
11719164_CLCN5	-0.50085	5.44E-05	0.000371
11733154_UBTD2	-0.29837	5.45E-05	0.000371
11725104_EGFR	0.31862	5.46E-05	0.000372
11749440_WDR41	0.366465	5.46E-05	0.000372
11751940_NR1D2	0.576135	5.46E-05	0.000372
11727246_CHRNB1	0.406648	5.47E-05	0.000373
11749316_SURF4	-0.3001	5.47E-05	0.000373
11756185_POLR3K	-0.35352	5.47E-05	0.000373
11728842_KCNK1	-0.46492	5.47E-05	0.000373
11742211_APP	0.313724	5.48E-05	0.000373
11750827_SMAD4	0.313981	5.48E-05	0.000373
11742705_UBR5	0.330916	5.48E-05	0.000373
11716841_UBE4A	0.307143	5.49E-05	0.000373
11734669_KPNA1	0.375641	5.49E-05	0.000374
11721658_LMO4	-0.54027	5.50E-05	0.000374
11735399_VPS13A	0.520613	5.50E-05	0.000374
11719713_PPM1B	0.338546	5.50E-05	0.000374
11759401_UBE2I	-0.57647	5.50E-05	0.000374
11745389_C14orf93	-0.33467	5.50E-05	0.000374
11754622_MEIS3	0.318163	5.51E-05	0.000375
11749281_ANKLE2	0.417679	5.51E-05	0.000375
11717735_ZCCHC24	-0.27974	5.51E-05	0.000375
11754544_ZDHHC11 /	0.677186	5.52E-05	0.000375
11724651_LMBR1	0.406953	5.52E-05	0.000375

11755524_ARCN1	0.318823	5.52E-05	0.000375
11753720_GMFB	0.324137	5.52E-05	0.000375
11718138_LOC105372	-0.37459	5.53E-05	0.000375
11718179_FERMT2	0.427152	5.53E-05	0.000375
11724143_RAB11FIP2	0.405729	5.53E-05	0.000375
11724191_COPS2	0.298061	5.53E-05	0.000376
11759884_MLLT10	-0.80722	5.53E-05	0.000376
11759967_LOC100128	0.634076	5.54E-05	0.000376
11720786_DYNC1LI1	-0.35477	5.54E-05	0.000376
11731643_LOC102724	-0.60498	5.54E-05	0.000376
11723279_CREBZF	0.477913	5.55E-05	0.000376
11723660_UBIAD1	0.405668	5.55E-05	0.000376
11743997_ACTR1A	-0.35818	5.55E-05	0.000377
11744986_SAMD4A	0.65659	5.56E-05	0.000377
11715604_SPARC	0.288445	5.56E-05	0.000377
11717937_SMIM3	0.335929	5.56E-05	0.000377
11739194_SLMAP	0.34932	5.56E-05	0.000377
11728733_ZBTB38	0.292068	5.57E-05	0.000377
11736387_ABAT	-0.93793	5.57E-05	0.000378
11722494_HIP1R	-0.38661	5.57E-05	0.000378
11718257_CCDC130	0.350533	5.57E-05	0.000378
11730684_PTPRM	0.360289	5.58E-05	0.000378
11740895_MIR22HG	0.423663	5.58E-05	0.000378
11762367_RBPJ	-0.41941	5.58E-05	0.000378
11755874_BORA	-0.5088	5.59E-05	0.000379
11750512_CDK4	-0.28247	5.59E-05	0.000379
11750890_MARS2	0.648004	5.59E-05	0.000379
11730877_EBAG9	-0.30102	5.60E-05	0.000379
11740475_NOX4	0.431784	5.60E-05	0.000379
11723483_GAR1	-0.3038	5.60E-05	0.000379
11752001_CUL4A	-0.53715	5.60E-05	0.000379
11726049_FMO5	0.509943	5.60E-05	0.000379
11723687_FAM46A	0.360206	5.60E-05	0.000379
11755333_NFX1	0.39252	5.60E-05	0.000379
11739814_FZD1	-0.30209	5.61E-05	0.000379
11739468_TMEM87B	0.382473	5.61E-05	0.00038
11756843_BMI1 ///	0.342092	5.62E-05	0.00038
11743497_BMP2	1.108692	5.62E-05	0.00038
11750256_RSF1	0.459002	5.62E-05	0.00038
11757967_CCDC50	-0.48054	5.63E-05	0.00038
11744594_SEPT6	-0.37877	5.63E-05	0.000381
11720747_ADCY7	0.319457	5.63E-05	0.000381
11732423_ZNF234	0.583831	5.64E-05	0.000381
11746330_GSS	-0.29043	5.64E-05	0.000381
11733156_CCDC50	0.388558	5.64E-05	0.000381
11735084_FREM2	-0.52595	5.65E-05	0.000382
11717054_SSR1	-0.30727	5.66E-05	0.000382
11758277_NBPF11 //	0.286137	5.66E-05	0.000382
11739606_CCDC88A	-0.41522	5.67E-05	0.000383
11733161_TNFAIP8	-0.54803	5.67E-05	0.000383
11718901_TGFBR3	0.649536	5.68E-05	0.000383

11728967_ZNF468	0.661803	5.68E-05	0.000384
11755766_SNX30	0.330242	5.68E-05	0.000384
11715390_FKBP1A	0.281794	5.69E-05	0.000384
11720620_ACLY	-0.44157	5.69E-05	0.000384
11716213_PLXNB2	0.35462	5.69E-05	0.000384
11723559_SMCO4	-0.96487	5.69E-05	0.000384
11730868_MEA1	-0.3376	5.69E-05	0.000384
11723841_RMI1	-0.70982	5.69E-05	0.000384
11757620_TIA1	-0.29307	5.69E-05	0.000384
11743562_U2SURP	-0.37156	5.70E-05	0.000384
11733830_GMCL1	0.346428	5.70E-05	0.000384
11747578_SENP2	-0.38928	5.71E-05	0.000385
11724860_ACER3	-0.66367	5.72E-05	0.000385
11751182_DBT	0.340112	5.72E-05	0.000385
11718450_NAGA	-0.74781	5.72E-05	0.000385
11721173_VPS8	0.378341	5.72E-05	0.000385
11723125_MORC3	0.440645	5.73E-05	0.000386
11758071_SULF1	0.445029	5.74E-05	0.000386
11756791_RFK	0.369083	5.74E-05	0.000386
11749280_CAP2	0.746556	5.74E-05	0.000386
11727003_CSNK1E //	-0.40675	5.75E-05	0.000387
11715452_COL4A2	0.279132	5.75E-05	0.000387
11758542_ZRSR2	0.422181	5.76E-05	0.000387
11755831_WDR63	0.706964	5.76E-05	0.000387
11739418_IRGQ	0.731705	5.76E-05	0.000387
11753545_RAD52	0.359098	5.76E-05	0.000387
11722486_ALG11 ///	-0.39863	5.76E-05	0.000387
11736292_MAPK8	0.428747	5.77E-05	0.000388
11749390_LAMTOR1	0.292478	5.78E-05	0.000388
11735403_KIAA0754	0.672669	5.78E-05	0.000388
11726662_APOO	-0.27649	5.78E-05	0.000389
11721352_SLC1A1	0.343991	5.79E-05	0.000389
11724859_ACER3	-0.42808	5.79E-05	0.000389
11718309_HEG1	0.305169	5.80E-05	0.000389
11752784_FLRT2	0.619994	5.80E-05	0.00039
11740337_ZNF28	0.53516	5.80E-05	0.00039
11732983_TMEM8B	-0.40403	5.81E-05	0.00039
11729186_PNO1	0.444712	5.82E-05	0.000391
11756056_SLC2A1	-0.33816	5.82E-05	0.000391
11756556_C20orf24	-0.32222	5.83E-05	0.000391
11753485_ATP5S	-0.49397	5.84E-05	0.000392
11717484_PDHA1	-0.30192	5.84E-05	0.000392
11724606_MAP2K6	-0.39539	5.84E-05	0.000392
11731195_SEMA3D	0.449759	5.84E-05	0.000392
11750995_SYAP1	0.31052	5.85E-05	0.000392
11727113_EGF	0.811556	5.85E-05	0.000392
11715780_HNRNPH2 /	-0.47518	5.85E-05	0.000392
11745497_GON4L ///	0.324831	5.86E-05	0.000393
11717244_LSM4	-0.31099	5.86E-05	0.000393
11732793_EYA4	-0.33566	5.86E-05	0.000393
11718931_SLC35D2	0.443221	5.86E-05	0.000393

11736946_PSMD10	-0.27112	5.87E-05	0.000393
11747593_SH3BGRL	0.292915	5.87E-05	0.000394
11726659_ATG4C	-0.36826	5.87E-05	0.000394
11741039_DAP3	0.367469	5.87E-05	0.000394
11722752_ELMSAN1	0.312768	5.88E-05	0.000394
11715955_ARL6IP5	0.318751	5.89E-05	0.000394
11732156_UFM1	0.334633	5.90E-05	0.000395
11751314_SLC39A1	-0.33432	5.90E-05	0.000395
11754625_AP4B1	0.329277	5.90E-05	0.000395
11727050_PPP6C	0.354007	5.90E-05	0.000395
11755309_GLTSCR2	0.310876	5.90E-05	0.000395
11720884_HMBS	-0.29688	5.90E-05	0.000395
11750337_LBX2	-0.39055	5.90E-05	0.000395
11758506_RNF13	0.411497	5.91E-05	0.000395
11737014_HOXD8	0.299902	5.92E-05	0.000396
11746064_OGT	0.338595	5.92E-05	0.000396
11756283_CCAR1	0.310893	5.92E-05	0.000396
11723951_TFPI	0.442508	5.92E-05	0.000396
11717730_SQLE	-0.36285	5.92E-05	0.000396
11715601_PPP1R11	-0.60242	5.92E-05	0.000396
11736509_IL6R	0.551528	5.92E-05	0.000396
11756769_IL12A	-0.74121	5.93E-05	0.000396
11727002_CSNK1E //	-0.39594	5.93E-05	0.000396
11740261_TXK	0.848467	5.93E-05	0.000396
11757695_ZNF512	-0.33222	5.93E-05	0.000396
11723506_ZNF609	0.327474	5.93E-05	0.000396
11733065_NFYB	0.28099	5.93E-05	0.000396
11717326_KLF9	-0.30049	5.93E-05	0.000396
11734975_CLTB	0.317754	5.94E-05	0.000396
11716750_CD99L2	-0.44694	5.95E-05	0.000397
11739448_ROCK1 ///	0.352432	5.96E-05	0.000398
11731627_CCDC186	0.536849	5.96E-05	0.000398
11733520_POU3F2	-0.41839	5.97E-05	0.000398
11726538_KANK2	-0.36411	5.97E-05	0.000398
11722550_ITSN1	-0.29948	5.97E-05	0.000398
11757598_SWAP70	0.300283	5.97E-05	0.000398
11744679_SMAD4	0.30753	5.97E-05	0.000398
11746689_FAM46A	0.487355	5.97E-05	0.000398
11721732_TMX3	0.354869	5.98E-05	0.000399
11717857_DHX29	0.330519	5.99E-05	0.000399
11732492_KIAA1549	-0.49768	5.99E-05	0.000399
11750778_MSR1	0.440116	5.99E-05	0.000399
11731100_MFI2	-0.36765	5.99E-05	0.000399
11724349_PPP1R14C	-0.52781	5.99E-05	0.000399
11722586_G3BP1	-0.27581	6.00E-05	0.000399
11752747_MRS2	0.553927	6.00E-05	0.000399
11755515_DDI2 ///	0.500825	6.00E-05	0.0004
11741134_PTPN3	0.304716	6.01E-05	0.0004
11721190_TTC9C	0.288994	6.02E-05	0.000401
11731761_RAD52	0.458173	6.02E-05	0.000401
11715603_SPARC	0.305461	6.03E-05	0.000401

11716324_ICMT	-0.35031	6.03E-05	0.000401
11728207_XPC	0.305818	6.03E-05	0.000401
11756116_TAF1	0.390001	6.04E-05	0.000402
11757945_TP53BP2	-0.31991	6.04E-05	0.000402
11718440_NSUN4	0.326852	6.04E-05	0.000402
11761271_CARS2	0.282444	6.04E-05	0.000402
11720184_EEF1B2	0.276449	6.05E-05	0.000402
11749921_SDC4	-0.29377	6.05E-05	0.000403
11746345_RANBP3	-0.31168	6.06E-05	0.000403
11721543_CSRNP1	0.410426	6.06E-05	0.000403
11715923_GSS	-0.32839	6.06E-05	0.000403
11727837_ORC6	-0.32398	6.06E-05	0.000403
11717201_DNTTIP1	0.372301	6.07E-05	0.000403
11721591_FANCC	-0.55416	6.07E-05	0.000403
11720642_IVD	0.292571	6.08E-05	0.000404
11740688_PHACTR2	0.597271	6.08E-05	0.000404
11736228_PIGC	-0.33827	6.08E-05	0.000404
11746622_PHC1	-0.65706	6.08E-05	0.000404
11749929_PGPEP1	0.455344	6.08E-05	0.000404
11743964_COL6A3	0.443207	6.08E-05	0.000404
11743672_ZNF292	0.40775	6.09E-05	0.000404
11742062_ANP32E	-0.5419	6.09E-05	0.000404
11750841_PPP1R21	0.356418	6.09E-05	0.000404
11757960_ARAP2	0.560799	6.10E-05	0.000405
11718830_EHD1	0.337952	6.11E-05	0.000405
11732495_AUNIP	-0.47946	6.11E-05	0.000405
11745039_ADCK2	-0.33559	6.11E-05	0.000405
11729665_STK38L	0.59224	6.11E-05	0.000405
11745248_ILVBL	-0.53198	6.11E-05	0.000405
11727470_ZNF544	0.421243	6.12E-05	0.000405
11756954_ZEB2	0.442355	6.12E-05	0.000405
11728379_EFCAB2	-0.76241	6.12E-05	0.000406
11721510_NUS1	-0.39792	6.13E-05	0.000406
11715555_CYB5R3	-0.32788	6.13E-05	0.000406
11727899_CLMN	-0.31931	6.14E-05	0.000407
11725127_EP300	0.32886	6.15E-05	0.000407
11728725_TAF6	-0.30399	6.16E-05	0.000408
11721934_DNAJC27	0.395253	6.16E-05	0.000408
11747174_ERO1B	0.542562	6.17E-05	0.000408
11751625_DCUN1D1	0.32975	6.17E-05	0.000408
11763438_ZKSCAN5	0.440347	6.17E-05	0.000408
11754499_RBM7	-0.26772	6.17E-05	0.000408
11732841_SCN3A	0.652069	6.18E-05	0.000409
11741745_ABL2	0.417319	6.18E-05	0.000409
11754070_SSNA1	-0.39982	6.18E-05	0.000409
11733714_NAA16	0.441265	6.19E-05	0.00041
11740367_PTPN3	0.293772	6.19E-05	0.00041
11729237_MCCC2	0.344062	6.19E-05	0.00041
11743045_FLOT1	-0.37902	6.19E-05	0.00041
11728915_TRIOBP	-0.48993	6.20E-05	0.00041
11721953_RNF41	-0.3538	6.20E-05	0.00041

11727040_MAVS	-0.5488	6.20E-05	0.00041
11723314_PXMP2	-0.31432	6.21E-05	0.00041
11729366_PRKAA2	0.495309	6.21E-05	0.00041
11747504_ZNF510	0.337641	6.21E-05	0.00041
11740930_BVES	0.550017	6.21E-05	0.00041
11725139_FUBP3	-0.4574	6.21E-05	0.00041
11737371_HLCS	-0.49922	6.23E-05	0.000411
11729098_IL1RAP	0.41255	6.23E-05	0.000411
11731454_SEC62	0.306684	6.24E-05	0.000412
11759088_ 1-Mar	0.653915	6.24E-05	0.000412
11758615_FRMD4B	0.475199	6.24E-05	0.000412
11743915_SEL1L3	-0.60045	6.24E-05	0.000412
11723256_PKMYT1	-0.50053	6.25E-05	0.000412
11728217_BCLAF1	-0.38444	6.25E-05	0.000413
11745001_AASDH	-0.29807	6.25E-05	0.000413
11757574_TMEM123	-0.70482	6.26E-05	0.000413
11719424_FAM220A	-0.32489	6.26E-05	0.000413
11722499_LYSMD3	0.430428	6.26E-05	0.000413
11725058_NEDD9	0.55152	6.27E-05	0.000413
11749977_RNF6	0.580834	6.27E-05	0.000413
11715250_KIAA0825	0.837663	6.27E-05	0.000413
11756623_HSPA8	-0.40139	6.27E-05	0.000413
11728282_RBMS2	0.609142	6.28E-05	0.000414
11724553_PCGF5	0.735755	6.28E-05	0.000414
11743187_CFAP97	-0.37828	6.29E-05	0.000414
11740814_ATG7	0.32159	6.29E-05	0.000414
11757682_CYB5R2	-0.32483	6.29E-05	0.000414
11753705_STMN1	-0.30216	6.29E-05	0.000414
11715715_VOPP1	-0.40415	6.29E-05	0.000414
11726120_BMP2K	0.6034	6.30E-05	0.000415
11748139_RNF14	0.310951	6.30E-05	0.000415
11745854_RNF145	0.341716	6.30E-05	0.000415
11753011_EXOSC9	-0.3209	6.30E-05	0.000415
11743914_PKP4	0.439538	6.30E-05	0.000415
11733385_TMEM182	-0.82898	6.31E-05	0.000415
11717348_ADD3	-0.36495	6.31E-05	0.000415
11753509_FOXC1	-0.38019	6.32E-05	0.000415
11755323_MBD1	0.394582	6.33E-05	0.000416
11733970_PCDH18	0.421654	6.33E-05	0.000416
11753625_TMEM9	-0.2977	6.33E-05	0.000416
11732545_NOB1	0.306747	6.33E-05	0.000416
11748105_ASB3 ///	0.293099	6.34E-05	0.000416
11722731_APPL1	-0.30887	6.35E-05	0.000417
11753275_LAPTM4A	-0.33334	6.35E-05	0.000417
11758312_CCL3P1 //	0.968959	6.35E-05	0.000417
11757972_MYLIP	-0.32046	6.35E-05	0.000417
11726307_FAM204A	0.299748	6.35E-05	0.000417
11745274_PARD3	0.536265	6.36E-05	0.000418
11721530_ZDHHC2	0.433824	6.37E-05	0.000418
11739125_CPNE1	0.332989	6.37E-05	0.000418
11751672_LGALS8	0.332428	6.37E-05	0.000418

11719035_ACSF2	-0.28908	6.38E-05	0.000419
11720143_CCSAP	-0.40123	6.39E-05	0.000419
11749009_NFYA	-0.4625	6.40E-05	0.00042
11754548_RMDN1	-0.29983	6.41E-05	0.00042
11757846_ARMCX3	0.412108	6.41E-05	0.00042
11715492_MORF4L1	0.269827	6.41E-05	0.00042
11721157_NTHL1	-0.3295	6.41E-05	0.000421
11741301_GCOM1 ///	0.330319	6.42E-05	0.000421
11743451_ELOVL6	0.342963	6.42E-05	0.000421
11718562_DNMT3A	0.329316	6.42E-05	0.000421
11756662_ERAL1	-0.34102	6.42E-05	0.000421
11748985_VTA1	0.282589	6.42E-05	0.000421
11742822_DNPEP	-0.32139	6.43E-05	0.000421
11736315_WBP5	-0.52878	6.44E-05	0.000422
11720683_B3GALT6	-0.3765	6.44E-05	0.000422
11763916_LOC105379	0.475336	6.45E-05	0.000422
11743414_UBE2K	0.29421	6.45E-05	0.000422
11753462_SLC39A9	0.558762	6.45E-05	0.000423
11744695_CCDC90B	0.345032	6.46E-05	0.000423
11717847_FARSA	-0.27819	6.46E-05	0.000423
11715195_IMPACT	-0.45575	6.46E-05	0.000423
11729581_PPDPF	-0.36732	6.46E-05	0.000423
11740416_BRCA2	-0.58638	6.47E-05	0.000423
11756417_KIZ	-0.30365	6.47E-05	0.000423
11726963_SGTB	0.297651	6.47E-05	0.000423
11715413_TSC22D1	0.327816	6.47E-05	0.000423
11756615_SHMT2	-0.37194	6.47E-05	0.000423
11740045_PHACTR2	0.510159	6.48E-05	0.000423
11763169_FOSL2	0.560301	6.48E-05	0.000424
11725658_MTFR1	-0.31817	6.48E-05	0.000424
11722029_UBE3C	0.305319	6.50E-05	0.000425
11721722_MSH5 ///	-0.33597	6.51E-05	0.000426
11741910_PKNOX2	-0.4114	6.51E-05	0.000426
11717124_SH3PXD2A	-0.32426	6.52E-05	0.000426
11728651_ZRANB2	0.322014	6.52E-05	0.000426
11729326_ZFHX4	0.354711	6.52E-05	0.000426
11743078_UPF3A	0.277267	6.53E-05	0.000427
11723991_ALG6	-0.38217	6.54E-05	0.000427
11744708_LYRM1	0.43932	6.54E-05	0.000427
11719052_CEP350	0.397719	6.54E-05	0.000427
11737246_RALGAPA1	0.48886	6.57E-05	0.000429
11733871_PGK1	-0.32688	6.57E-05	0.000429
11759479_PTP4A2	-0.28082	6.57E-05	0.000429
11717886_PLAU	0.560484	6.57E-05	0.000429
11727033_PAPSS2	-0.38317	6.57E-05	0.000429
11748556_SARNP	0.344917	6.58E-05	0.000429
11742828_PHF3	-0.27517	6.58E-05	0.000429
11726416_ABI1	-0.30717	6.58E-05	0.000429
11758574_THRB	0.524582	6.59E-05	0.00043
11743962_CNST	0.420183	6.59E-05	0.00043
11748824_TTLL12	-0.40315	6.59E-05	0.00043

11744582_USP15	0.581168	6.59E-05	0.00043
11755169_RP9 /// R	0.265678	6.59E-05	0.00043
11751038_ZNF263	0.339727	6.59E-05	0.00043
11736318_SNRNP48	0.518229	6.61E-05	0.000431
11720276_TREX1	-0.33971	6.61E-05	0.000431
11736227_C16orf87	0.44158	6.61E-05	0.000431
11749510_SAMHD1	0.310528	6.62E-05	0.000431
11716456_RHOBTB3	0.30123	6.62E-05	0.000431
11718653_PKD2	0.440566	6.63E-05	0.000431
11720956_IKBKE	0.544911	6.63E-05	0.000431
11721479_GOLPH3L	-0.33426	6.65E-05	0.000433
11734860_WDR37	0.385456	6.65E-05	0.000433
11724321_C11orf54	0.310893	6.65E-05	0.000433
11742689_SRA1	0.3695	6.66E-05	0.000433
11739050_ACPP	0.629135	6.66E-05	0.000433
11743788_GORAB	0.315576	6.66E-05	0.000433
11728469_BTBD11	0.377382	6.66E-05	0.000433
11758149_RACGAP1	-0.56908	6.66E-05	0.000433
11755528_LOC399815	-0.31384	6.66E-05	0.000433
11725847_FAM76B	-0.56009	6.67E-05	0.000434
11748405_MDC1	-0.81456	6.67E-05	0.000434
11726464_IGFLR1	-0.33024	6.68E-05	0.000434
11724464_ANLN	-0.31667	6.68E-05	0.000434
11717812_KIF3C	-0.29536	6.68E-05	0.000434
11716948_TM9SF3	0.355883	6.68E-05	0.000434
11755966_HLA-B	0.343849	6.68E-05	0.000434
11715532_IFNGR2	0.294378	6.68E-05	0.000434
11753878_IL6ST	0.270716	6.69E-05	0.000434
11739217_RSAD2	0.542824	6.69E-05	0.000434
11741135_CHN1	0.373159	6.69E-05	0.000434
11739981_SOCS4	0.463102	6.69E-05	0.000435
11748012_TMEM9	-0.37031	6.69E-05	0.000435
11740613_TMEM30A	0.381428	6.70E-05	0.000435
11743772_LZTFL1	-0.36515	6.70E-05	0.000435
11722881_KIAA1468	0.50421	6.71E-05	0.000436
11728445_TGFBRAP1	-0.32398	6.72E-05	0.000436
11759573_MGEA5	0.369678	6.73E-05	0.000436
11724573_APPBP2	-0.64623	6.73E-05	0.000437
11727021_ABI2	0.307832	6.73E-05	0.000437
11728507_LY96	0.654931	6.73E-05	0.000437
11744136_CADM4	-0.35566	6.74E-05	0.000437
11729191_MAX	0.384117	6.74E-05	0.000437
11755033_GABPA	-0.45875	6.74E-05	0.000437
11752386_NAB1	0.394052	6.74E-05	0.000437
11746346_B3GAT3	0.436876	6.74E-05	0.000437
11717115_CERK	-0.29415	6.75E-05	0.000437
11747537_SHMT2	-0.37252	6.75E-05	0.000437
11750609_ITSN1	-0.64157	6.75E-05	0.000437
11741055_SPRED2	0.417496	6.75E-05	0.000437
11753618_ADM	0.359966	6.76E-05	0.000438
11760964_NBPF1	0.322364	6.76E-05	0.000438

11727986_TENM3	-0.46025	6.76E-05	0.000438
11727496_LRRC75A-A	0.272089	6.76E-05	0.000438
11745541_SSU72	-0.30777	6.78E-05	0.000439
11748157_MRPL42	-0.30025	6.78E-05	0.000439
11754374_HKR1	0.367193	6.78E-05	0.000439
11757053_TMEM43	0.293744	6.79E-05	0.000439
11715861_ACTN4	-0.3025	6.79E-05	0.000439
11737943_SPP1	0.420237	6.79E-05	0.000439
11752011_CEMIP	-0.34809	6.79E-05	0.000439
11749718_PLXNB2	0.302349	6.80E-05	0.00044
11739674_PDP2	0.43339	6.81E-05	0.00044
11725864_DISP2	0.367798	6.81E-05	0.00044
11748039_LOC440434	0.299498	6.81E-05	0.00044
11740973_SLC44A2	-0.2953	6.83E-05	0.000441
11734243_SPTBN5	0.535266	6.84E-05	0.000442
11716303_SRSF6	-0.29818	6.84E-05	0.000442
11725977_NCBP1	-0.35933	6.84E-05	0.000442
11731438_MED6	0.311693	6.85E-05	0.000442
11740186_ZKSCAN5	0.323837	6.85E-05	0.000442
11731008_WDR89	0.396113	6.85E-05	0.000442
11755592_DNAJC24	-0.43504	6.86E-05	0.000443
11740109_HEATR3	0.317879	6.86E-05	0.000443
11763889_SNHG8 ///	0.30853	6.86E-05	0.000443
11748564_REEP5	0.46293	6.87E-05	0.000443
11751900_MON2	0.337209	6.87E-05	0.000443
11748744_ADD2	-0.36505	6.88E-05	0.000444
11721706_GPAM	-0.45397	6.88E-05	0.000444
11716201_PISD	0.316317	6.88E-05	0.000444
11720783_EDF1	-0.30516	6.88E-05	0.000444
11741353_ESM1	0.382691	6.89E-05	0.000444
11723138_POP1	0.313239	6.89E-05	0.000444
11740196_ZNF626	0.346731	6.90E-05	0.000445
11736120_ZFAND5	0.335397	6.91E-05	0.000445
11755359_PHACTR4	0.39937	6.91E-05	0.000446
11724549_RSBN1	0.268882	6.92E-05	0.000446
11755933_HLA-B	0.306184	6.92E-05	0.000446
11729656_SMAD2	0.322025	6.92E-05	0.000446
11756621_MNX1	-0.37163	6.92E-05	0.000446
11742190_NR3C1	-0.47859	6.93E-05	0.000446
11717927_FOXK2	-0.35621	6.93E-05	0.000446
11748657_DHX32	0.29154	6.93E-05	0.000446
11747337_EIF3I	0.286194	6.93E-05	0.000447
11718322_ACAP2	-0.34601	6.94E-05	0.000447
11731400_TMCO1	0.287832	6.94E-05	0.000447
11724225_GGCT	-0.3009	6.95E-05	0.000447
11716888_HEXA	0.313772	6.95E-05	0.000448
11722254_UQCC3	-0.31412	6.96E-05	0.000448
11725973_HNRNPF	-0.40114	6.97E-05	0.000448
11731492_PRPF18	0.289075	6.97E-05	0.000448
11757465_GBAS	-0.34641	6.97E-05	0.000448
11717767_ATMIN	0.337213	6.97E-05	0.000448

11727305_WDSUB1	0.309567	6.98E-05	0.000449
11715766_DUSP1	0.367326	6.98E-05	0.000449
11720847_PHF6	-0.36051	6.98E-05	0.000449
11752354_KIAA1217	0.558158	6.98E-05	0.000449
11764077_EDIL3	0.639799	6.98E-05	0.000449
11728635_AP4E1	0.368456	6.98E-05	0.000449
11741582_SLC3A2	0.300388	6.99E-05	0.000449
11755721_ATP6V1G2	-0.49188	6.99E-05	0.000449
11739113_ADPGK	-0.34434	6.99E-05	0.000449
11730111_DEPDC1	-0.43502	6.99E-05	0.000449
11751558_MSANTD2	0.420322	7.00E-05	0.000449
11759673_DBF4B	-0.47681	7.00E-05	0.00045
11724258_MUC15	-0.57143	7.01E-05	0.00045
11754648_UBFD1	-0.31705	7.01E-05	0.00045
11726058_R3HCC1L	0.479551	7.02E-05	0.00045
11726394_FGD4	0.644519	7.03E-05	0.000451
11728884_TM6SF1	-0.4249	7.03E-05	0.000451
11739931_MCM8	-0.3515	7.03E-05	0.000451
11727587_HAGHL	-0.36793	7.03E-05	0.000451
11744994_CHPF2	0.316348	7.04E-05	0.000451
11757397_CAST	-0.34988	7.05E-05	0.000452
11736552_KLHL36	0.514513	7.07E-05	0.000453
11719794_PPP2R5D	-0.36224	7.07E-05	0.000453
11722679_PARP8	0.312084	7.07E-05	0.000453
11754431_RBMX ///	-0.30903	7.07E-05	0.000453
11742423_STEAP2	-0.55082	7.08E-05	0.000454
11726788_DEPDC7	0.312205	7.08E-05	0.000454
11719108_FHL3	0.297214	7.09E-05	0.000454
11752198_PTPRM	0.315949	7.09E-05	0.000454
11735415_SLC39A6	-0.39743	7.10E-05	0.000455
11750039_ASH1L	0.329484	7.10E-05	0.000455
11759634_TMEM39A	-0.40344	7.10E-05	0.000455
11745327_ARL13B	0.334724	7.10E-05	0.000455
11733052_SCAMP1	-0.42324	7.10E-05	0.000455
11722237_BLOC1S4	0.360743	7.10E-05	0.000455
11725707_AMOTL1	0.301607	7.11E-05	0.000455
11755730_SPP1	0.515	7.11E-05	0.000455
11744739_UBAP2L	0.301092	7.11E-05	0.000455
11754941_KRT18	0.30098	7.11E-05	0.000455
11740558_HOXA13	-0.75517	7.12E-05	0.000455
11754187_EIF2AK1	-0.31807	7.12E-05	0.000455
11748210_MMP8	0.665073	7.12E-05	0.000455
11741789_NDUFB6	-0.33806	7.12E-05	0.000455
11729861_H6PD	-0.36997	7.13E-05	0.000456
11721894_GAS1	-0.31576	7.13E-05	0.000456
11727893_PRELP	-0.55739	7.13E-05	0.000456
11744691_ARMC10	0.294159	7.14E-05	0.000456
11759887_NBPF8	0.303271	7.15E-05	0.000457
11758909_MAP2K6	-0.67383	7.15E-05	0.000457
11762445_LINC01588	0.527987	7.15E-05	0.000457
11733847_CC2D1B	0.288367	7.16E-05	0.000458

11763281_RPL23AP82	0.618587	7.16E-05	0.000458
11723280_CREBZF	-0.50195	7.17E-05	0.000458
11715775_HSPB8	0.511164	7.18E-05	0.000459
11721893_CHEK2 ///	-0.3522	7.18E-05	0.000459
11720505_RAB6B	0.377905	7.19E-05	0.000459
11733911_UTP15	0.42009	7.19E-05	0.000459
11752225_ARNT	0.35172	7.19E-05	0.000459
11718550_NAA60	0.303409	7.20E-05	0.00046
11758496_SMARCAD1	-0.43382	7.20E-05	0.00046
11720204_NT5DC1	-0.40127	7.22E-05	0.000461
11739274_LOC102723	0.394567	7.22E-05	0.000461
11741214_SSU72	-0.30495	7.22E-05	0.000461
11726336_CRLS1	-0.60981	7.23E-05	0.000461
11719714_PPM1B	0.351293	7.23E-05	0.000461
11756876_UQCC2	-0.41	7.23E-05	0.000461
11728817_USP28	-0.29519	7.24E-05	0.000462
11745771_RNPS1	-0.44674	7.24E-05	0.000462
11763855_CD9	-0.27792	7.24E-05	0.000462
11716194_SETD2	0.318947	7.25E-05	0.000462
11754591_FAM57A	-0.30012	7.26E-05	0.000462
11753159_CD274	0.576927	7.26E-05	0.000463
11754258_LIAS	-0.27564	7.26E-05	0.000463
11723871_DOCK2	0.527713	7.27E-05	0.000463
11742200_PREPL	0.354213	7.28E-05	0.000463
11730545_EFCAB11	-0.39961	7.28E-05	0.000464
11730045_KDELR3	-0.26949	7.28E-05	0.000464
11717010_MCFD2	0.320085	7.29E-05	0.000464
11745178_MCFD2	0.320085	7.29E-05	0.000464
11755100_COL16A1	0.291561	7.29E-05	0.000464
11745966_PRRC2B	-0.36481	7.29E-05	0.000464
11717156_ACP1	-0.2826	7.29E-05	0.000464
11753399_ANXA2	0.272953	7.30E-05	0.000464
11747460_TAGLN2	-0.38269	7.31E-05	0.000465
11753802_SRSF5	-0.31629	7.31E-05	0.000465
11731565_MDM2	0.447437	7.31E-05	0.000465
11744891_LOC339803	-0.3922	7.32E-05	0.000465
11744390_TIMM8B	0.272568	7.32E-05	0.000465
11722160_GRB10	-0.32275	7.32E-05	0.000466
11722759_CAMSAP2	0.313388	7.32E-05	0.000466
11722000_HNRNPU	-0.4275	7.34E-05	0.000466
11750359_CD68	-0.3353	7.34E-05	0.000467
11734367_FOXD3	-0.4093	7.38E-05	0.000469
11738083_POC1B	0.457974	7.38E-05	0.000469
11745073_TRMT44	0.481835	7.38E-05	0.000469
11721094_SLC39A7	-0.46352	7.38E-05	0.000469
11715459_COX8A	-0.28332	7.39E-05	0.000469
11761453_SERAC1	0.351577	7.39E-05	0.000469
11720810_MZT2A ///	-0.37755	7.39E-05	0.000469
11728686_SFXN1	0.327006	7.41E-05	0.00047
11716135_MVP	0.350937	7.41E-05	0.00047
11723257_PKMYT1	-0.62153	7.42E-05	0.000471

11719762_GPSM1	-0.35198	7.42E-05	0.000471
11759512_CWC25	0.460869	7.42E-05	0.000471
11749590_FN3KRP	-0.46134	7.43E-05	0.000471
11735066_ITGB3	0.586136	7.43E-05	0.000471
11753902_ACTN4	-0.26531	7.44E-05	0.000472
11728088_DKK2	0.477233	7.44E-05	0.000472
11723569_CAPN7	0.371956	7.44E-05	0.000472
11743090_SLC36A4	-0.40169	7.44E-05	0.000472
11729854_MOK	0.403442	7.44E-05	0.000472
11751744_MMD	-0.2951	7.46E-05	0.000473
11725579_BEND3	-0.53413	7.46E-05	0.000473
11739987_ZNF225	0.639417	7.47E-05	0.000474
11720518_TACC2	0.278668	7.47E-05	0.000474
11763739_SSR1	0.260021	7.48E-05	0.000474
11760100_PSMB2	-0.40802	7.48E-05	0.000474
11747498_SORD	-0.437	7.48E-05	0.000474
11753785_DYNLT1	0.323952	7.48E-05	0.000474
11727494_TCEB3	-0.35595	7.50E-05	0.000475
11759176_GTSE1 ///	-0.44617	7.50E-05	0.000475
11759312_AGO4	0.424102	7.50E-05	0.000475
11751116_ACTN1	0.320193	7.51E-05	0.000475
11721465_OSBPL2	-0.28833	7.52E-05	0.000476
11762098_SOBP	-0.44045	7.52E-05	0.000476
11759429_KLF7	0.757529	7.53E-05	0.000477
11748892_RBM42	-0.37287	7.54E-05	0.000477
11720801_BIN3	-0.33972	7.54E-05	0.000477
11727279_SLC4A7	0.36517	7.54E-05	0.000477
11753918_NUP85	-0.31925	7.55E-05	0.000478
11730580_HECW2	0.609185	7.55E-05	0.000478
11716538_SZRD1	0.337698	7.56E-05	0.000478
11733831_GMCL1 ///	0.349375	7.56E-05	0.000478
11743659_SNRNP27	0.271096	7.57E-05	0.000478
11743075_IPO9	-0.31891	7.58E-05	0.000479
11754424_ST5	-0.40838	7.58E-05	0.000479
11719077_RPUSD4	0.438945	7.59E-05	0.000479
11730293_SIN3A	-0.34968	7.59E-05	0.00048
11756964_DPH1 ///	0.325298	7.60E-05	0.00048
11717532_MAN2A2	-0.48562	7.60E-05	0.00048
11727278_PIGL	-0.2973	7.60E-05	0.00048
11715725_ITM2B	0.260089	7.61E-05	0.00048
11721376_BCL2	-0.47066	7.61E-05	0.00048
11746704_HAUS7	-0.47863	7.62E-05	0.000481
11717493_SSNA1	-0.39071	7.62E-05	0.000481
11744879_RARS	0.265771	7.62E-05	0.000481
11715258_PTMS	-0.30853	7.64E-05	0.000482
11717789_AAR2	0.307015	7.64E-05	0.000482
11730767_NGLY1	0.305555	7.65E-05	0.000483
11722754_SHPRH	0.356643	7.65E-05	0.000483
11715552_IMMT	-0.28347	7.66E-05	0.000483
11728340_RTN4IP1	-0.36119	7.66E-05	0.000483
11718149_APH1A	-0.30063	7.68E-05	0.000484

11738232_CENPI	-0.53446	7.68E-05	0.000484
11751282_SLC35B2	-0.34749	7.68E-05	0.000484
11751004_SH3BP4	0.523241	7.68E-05	0.000484
11739446_NELFCD	-0.2892	7.68E-05	0.000484
11719539_NEMP1	-0.28399	7.69E-05	0.000484
11764171_DCUN1D1	0.426035	7.69E-05	0.000485
11751036_RGL1	0.774886	7.70E-05	0.000485
11730757_IL6ST	0.339954	7.70E-05	0.000485
11747361_HMGA1	-0.37684	7.70E-05	0.000485
11735194_NR3C1	-0.36146	7.70E-05	0.000485
11752166_PKM	-0.29001	7.70E-05	0.000485
11719156_FAM104A	0.299472	7.71E-05	0.000485
11758942_MOB1A	-0.30797	7.73E-05	0.000487
11722585_CAST	-0.28327	7.73E-05	0.000487
11757680_SLC1A4	-0.32824	7.74E-05	0.000487
11723962_KIAA1143	-0.30233	7.74E-05	0.000487
11733088_MAF	0.258095	7.74E-05	0.000487
11732581_SLC24A5	0.378715	7.74E-05	0.000487
11762349_ZNF254	0.337482	7.76E-05	0.000488
11745431_SVIL	0.361196	7.77E-05	0.000488
11732554_FAIM	-0.48372	7.77E-05	0.000488
11741829_FAIM	-0.48372	7.77E-05	0.000488
11759657_TOM1L1	0.324028	7.77E-05	0.000488
11728034_TBC1D7	-0.36877	7.77E-05	0.000488
11742855_ASNSD1	0.362241	7.77E-05	0.000489
11744782_MORC4	0.329364	7.77E-05	0.000489
11763522_RYR2	0.728089	7.78E-05	0.000489
11717217_STK10	-0.35076	7.78E-05	0.000489
11728944_LST1	0.430045	7.79E-05	0.000489
11736583_TRIM36	0.460188	7.80E-05	0.00049
11756928_TBCD	-0.31708	7.80E-05	0.00049
11725014_MAP9	0.419057	7.81E-05	0.00049
11754670_SLC35B2	-0.45936	7.81E-05	0.00049
11728130_DNAAF5	-0.30246	7.81E-05	0.00049
11716147_CHMP7	-0.38227	7.81E-05	0.00049
11757709_MRPL2	-0.47688	7.82E-05	0.000491
11728554_ATP6V1E2	-0.65204	7.82E-05	0.000491
11734871_COMT	0.292926	7.82E-05	0.000491
11719929_KDM5B	0.378364	7.83E-05	0.000491
11753095_SLC22A15	0.88371	7.83E-05	0.000491
11750503_TM9SF4	0.326161	7.84E-05	0.000492
11721821_SRPK2	0.267624	7.84E-05	0.000492
11737990_CEP170 //	0.301029	7.85E-05	0.000492
11728386_SIX5	-0.58884	7.85E-05	0.000492
11740382_RBL1	-0.4563	7.86E-05	0.000492
11717256_PIM1	-0.81901	7.86E-05	0.000493
11723621_C7orf60	0.381246	7.86E-05	0.000493
11755330_TMEM129	-0.59489	7.87E-05	0.000493
11748357_CEP70	-0.33729	7.87E-05	0.000493
11735678_LRTOMT	0.349282	7.88E-05	0.000494
11723311_C6orf136	-0.31583	7.88E-05	0.000494

11757742_PLEKHB2	0.289943	7.88E-05	0.000494
11720616_DNM1	-0.39523	7.88E-05	0.000494
11734874_PPFIBP1	-0.31375	7.89E-05	0.000494
11740214_TBL1X	-0.31959	7.89E-05	0.000494
11726268_KDM4C	0.320015	7.89E-05	0.000494
11755058_BAZ1A	0.371703	7.90E-05	0.000495
11723683_METTL16	-0.30822	7.90E-05	0.000495
11740747_DNMT3A	0.435952	7.91E-05	0.000495
11734077_TNRC6C	0.433741	7.91E-05	0.000495
11742677_PTBP1	-0.25616	7.91E-05	0.000495
11736130_SRSF5	-0.3706	7.91E-05	0.000495
11718884_ARL5A	0.294832	7.92E-05	0.000495
11719748_CDV3	0.275024	7.92E-05	0.000495
11728029_PWWP2A	0.351725	7.95E-05	0.000497
11755542_ZNF671	0.704697	7.96E-05	0.000497
11753817_HMGA1	-0.30547	7.97E-05	0.000498
11751292_PIAS2	0.291262	7.98E-05	0.000499
11759838_ZNF506	0.353765	7.98E-05	0.000499
11744033_S100A11	0.270072	7.98E-05	0.000499
11735761_NAA11	0.376563	7.98E-05	0.000499
11728093_MAP3K1	0.343982	8.00E-05	0.0005
11732275_CCL5	0.615307	8.00E-05	0.0005
11754430_HPS6	-0.28732	8.00E-05	0.0005
11722638_ANKRD28	0.277115	8.00E-05	0.0005
11749424_SMPD4	-0.39442	8.01E-05	0.0005
11742424_POTEM	0.316996	8.01E-05	0.0005
11750676_ZNF567	0.612776	8.02E-05	0.0005
11731984_CERKL	0.525695	8.03E-05	0.000501
11716604_KBTBD2	0.270626	8.04E-05	0.000501
11751145_UFL1	-0.46985	8.04E-05	0.000502
11715791_TUBG1	-0.28709	8.05E-05	0.000502
11760461_SGOL1	-0.5674	8.05E-05	0.000502
11757612_ASRGL1	-0.33766	8.05E-05	0.000502
11736226_SPATS2	0.409677	8.07E-05	0.000503
11763965_MDN1	0.291842	8.09E-05	0.000504
11719902_PHF11	0.324302	8.09E-05	0.000505
11722683_MECP2	0.283635	8.10E-05	0.000505
11730794_MRPL4	-0.29839	8.10E-05	0.000505
11740393_TNFRSF9	0.415914	8.11E-05	0.000505
11749004_PEX6	0.282298	8.11E-05	0.000505
11755331_ANK2	-0.39856	8.11E-05	0.000505
11752789_IMMT	-0.33942	8.12E-05	0.000506
11727348_BAIAP2L2	-0.31744	8.14E-05	0.000507
11742928_AP3D1	-0.28075	8.14E-05	0.000507
11725884_CNEP1R1	0.365006	8.16E-05	0.000508
11760232_SFSWAP	0.364648	8.17E-05	0.000509
11746292_CPOX	-0.44733	8.17E-05	0.000509
11747052_GALNT4 //	-0.28116	8.17E-05	0.000509
11753825_FKBP1A //	0.270247	8.17E-05	0.000509
11757616_C14orf1	-0.35112	8.18E-05	0.000509
11723213_MEF2C	0.453518	8.18E-05	0.000509

11732583_NUDT22	-0.33596	8.19E-05	0.000509
11728609_BBS7	0.41013	8.19E-05	0.000509
11729290_HAND2	-0.55026	8.19E-05	0.000509
11744122_FAM156A /	-0.4055	8.19E-05	0.000509
11724298_DDX31	0.326893	8.19E-05	0.000509
11724012_SIKE1	-0.41453	8.19E-05	0.000509
11717339_SORD	-0.31208	8.19E-05	0.000509
11728493_ZNF197	0.466859	8.19E-05	0.000509
11743571_ANKRD17	0.345253	8.20E-05	0.00051
11719404_HIPK2	0.315591	8.21E-05	0.00051
11735243_ZC3H12C	0.62465	8.21E-05	0.00051
11743176_RSF1	0.441637	8.21E-05	0.00051
11750042_ANXA5	0.267221	8.22E-05	0.000511
11720708_PPARG	-0.4086	8.22E-05	0.000511
11728672_UBE2H	0.306756	8.22E-05	0.000511
11738200_CCDC102B	-0.41014	8.23E-05	0.000511
11752664_S1PR5	-0.48607	8.24E-05	0.000511
11752707_TMPRSS9	-0.30827	8.24E-05	0.000512
11749119_FGD4	0.507229	8.26E-05	0.000513
11733153_UBTD2	-0.32754	8.26E-05	0.000513
11740637_GPR19	0.445605	8.26E-05	0.000513
11715515_PSMD3	-0.33994	8.27E-05	0.000513
11718340_DAZAP2	-0.35507	8.27E-05	0.000513
11754048_ERH	-0.28091	8.27E-05	0.000513
11757927_DCUN1D4	-0.28692	8.28E-05	0.000513
11725533_ZNF618	-0.26705	8.28E-05	0.000514
11717571_CPSF3	-0.26227	8.29E-05	0.000514
11752463_ANKLE1	-0.32623	8.29E-05	0.000514
11743757_MKRN2	0.289755	8.30E-05	0.000514
11733145_ZBTB14	-0.74904	8.30E-05	0.000515
11753590_COMMD9	-0.29007	8.30E-05	0.000515
11744263_RAC2	0.351144	8.31E-05	0.000515
11732707_FGFRL1	-0.29	8.31E-05	0.000515
11755306_ZNF410	0.331381	8.31E-05	0.000515
11762859_TIMM23 //	0.613842	8.32E-05	0.000515
11754060_DAD1	-0.28478	8.32E-05	0.000516
11739156_TMX4	0.282134	8.32E-05	0.000516
11750142_YY1AP1	0.367826	8.33E-05	0.000516
11726735_TLK2	0.28821	8.34E-05	0.000516
11750408_ADGRE5	0.362589	8.34E-05	0.000516
11750970_LOX	0.306833	8.34E-05	0.000516
11745243_NBPF11 //	0.328793	8.34E-05	0.000516
11721272_LRRC59	-0.31637	8.35E-05	0.000517
11719787_PSMB5	-0.3068	8.36E-05	0.000517
11726781_ADD2	-0.27398	8.37E-05	0.000518
11734971_MCM8	-0.32996	8.37E-05	0.000518
11724034_CD2AP	0.524221	8.37E-05	0.000518
11715472_SRPR	-0.34556	8.38E-05	0.000518
11730126_SPICE1	-0.42155	8.38E-05	0.000518
11757908_RALGDS	0.263349	8.38E-05	0.000518
11746312_ZNF326	-0.26001	8.38E-05	0.000518

11720083_NUDT22	-0.35781	8.38E-05	0.000518
11749423_LIG1	-0.36269	8.39E-05	0.000518
11719401_C1orf122	0.3162	8.39E-05	0.000519
11722905_TBC1D9	0.444215	8.40E-05	0.000519
11739474_NAB1	0.366678	8.40E-05	0.000519
11731082_RNF144B	0.771508	8.40E-05	0.000519
11756037_NGLY1	0.370828	8.41E-05	0.000519
11747358_CIAPIN1	-0.36325	8.41E-05	0.000519
11733809_ARMCX5-GF	-0.49974	8.41E-05	0.000519
11739396_UGT8	-0.39947	8.41E-05	0.000519
11745844_MRPL18	-0.27559	8.42E-05	0.00052
11724776_PGM2L1	0.501685	8.42E-05	0.00052
11720797_TIMM9	0.280983	8.43E-05	0.00052
11747977_ZNF180	0.398809	8.43E-05	0.00052
11759766_MR1	0.465159	8.44E-05	0.000521
11736548_FANCD2 //	-0.61017	8.44E-05	0.000521
11716168_MRFAP1L1	-0.26753	8.44E-05	0.000521
11735340_TANC1	0.286582	8.45E-05	0.000521
11716250_RBFOX2	0.3143	8.46E-05	0.000522
11719873_LTV1	0.362147	8.46E-05	0.000522
11744522_BOD1	-0.28781	8.47E-05	0.000522
11746106_CDC16	0.302715	8.47E-05	0.000522
11725160_FBXW2	-0.53698	8.47E-05	0.000522
11721053_KLHL42	-0.29921	8.48E-05	0.000523
11755008_SETDB2	0.476131	8.49E-05	0.000523
11729994_SEMA6B	-0.3833	8.49E-05	0.000523
11753356_TUBB	-0.28779	8.49E-05	0.000523
11752420_NPIPB6	0.460891	8.50E-05	0.000524
11751764_LDAH	0.446031	8.50E-05	0.000524
11752239_ZC3H15	0.269928	8.52E-05	0.000525
11755622_ROBO4	-0.57904	8.53E-05	0.000525
11754120_ARL5A	0.306815	8.53E-05	0.000525
11740008_CHRFAM7A	-0.48542	8.54E-05	0.000526
11763516_CPT1C	-0.48563	8.54E-05	0.000526
11736016_ARCN1	0.300817	8.55E-05	0.000526
11750639_FBXO11	0.26935	8.55E-05	0.000526
11763816_EIF3M	0.311598	8.55E-05	0.000526
11739917_CSNK1D	0.291244	8.56E-05	0.000527
11738453_ACTBL2	0.722181	8.56E-05	0.000527
11730081_MTO1	0.281371	8.57E-05	0.000527
11762311_SPC24	-0.73861	8.58E-05	0.000528
11756092_SERGEF	-0.32566	8.58E-05	0.000528
11733838_UTP14A //	0.279768	8.58E-05	0.000528
11748825_TTLL12	-0.43105	8.59E-05	0.000528
11721244_BGN	-0.38447	8.59E-05	0.000528
11722825_NCAPG	-0.41176	8.62E-05	0.00053
11753125_SF3B3	-0.34305	8.62E-05	0.00053
11726426_DENND4C	0.352029	8.64E-05	0.000531
11725237_RBPMS2	-0.37648	8.64E-05	0.000531
11715458_COX8A	-0.27746	8.65E-05	0.000531
11719468_PRPF31	-0.29254	8.65E-05	0.000531

11739183_MTRF1L	0.462287	8.65E-05	0.000531
11754221_FAM104B	-0.32422	8.65E-05	0.000531
11753258_PPT2 ///	-0.26752	8.65E-05	0.000531
11751751_HNRNPH3	-0.30009	8.65E-05	0.000531
11745450_AUH	0.3539	8.66E-05	0.000532
11742960_MEPCE	-0.32834	8.66E-05	0.000532
11716698_HBP1	0.307041	8.69E-05	0.000533
11747780_GOLGA8DP	0.318532	8.70E-05	0.000534
11754130_BANF1	-0.89099	8.70E-05	0.000534
11727136_PUS7L	-0.3362	8.70E-05	0.000534
11723466_MRPS11	-0.26796	8.70E-05	0.000534
11726151_PPIP5K2	-0.35623	8.73E-05	0.000535
11739627_DDX52	0.305634	8.73E-05	0.000536
11739138_RNF26	-0.36945	8.74E-05	0.000536
11721884_LINC00431	-0.34829	8.74E-05	0.000536
11728259_DIEXF	0.284416	8.74E-05	0.000536
11720254_CNNM4	-0.6178	8.74E-05	0.000536
11724595_RP2	0.376379	8.74E-05	0.000536
11758965_ATG4C	-0.29737	8.74E-05	0.000536
11747240_DPH1 ///	0.356583	8.74E-05	0.000536
11727797_NDFIP2	-0.45881	8.75E-05	0.000536
11748059_EAF1	0.61147	8.75E-05	0.000536
11719649_ALS2	0.443841	8.75E-05	0.000536
11748863_ARL6IP5	0.259804	8.76E-05	0.000536
11733560_PPP1R21	0.379975	8.76E-05	0.000536
11725509_SLC7A6	0.35422	8.76E-05	0.000536
11730551_STAC	0.565044	8.76E-05	0.000536
11716531_ELAVL1	-0.46296	8.76E-05	0.000537
11719118_TPBG	0.413725	8.77E-05	0.000537
11733479_SUPT20H	0.269583	8.79E-05	0.000538
11750775_DENND1B	0.38882	8.80E-05	0.000538
11749135_BRD3	-0.3949	8.80E-05	0.000538
11716261_AP2B1	-0.29828	8.80E-05	0.000539
11739744_MXI1	-0.30875	8.81E-05	0.000539
11748907_RARRES3	0.561592	8.81E-05	0.000539
11762891_BAGE	0.505035	8.82E-05	0.000539
11716838_CDV3	0.273955	8.82E-05	0.000539
11739618_PTPN3	-0.62171	8.83E-05	0.00054
11745848_TJP2	0.349848	8.84E-05	0.00054
11735373_NETO1	0.536294	8.86E-05	0.000541
11719941_DSCR3	0.351577	8.86E-05	0.000541
11717209_RXRB	-0.32753	8.86E-05	0.000542
11753911_TMEM242	-0.39881	8.86E-05	0.000542
11717750_CD320	-0.31204	8.87E-05	0.000542
11755593_ATG13	-0.32329	8.87E-05	0.000542
11718189_FOXJ2	-0.49702	8.88E-05	0.000542
11739271_PAK2	-0.40623	8.88E-05	0.000542
11747339_ABCB9	0.527752	8.88E-05	0.000542
11729193_MYNN	0.440616	8.88E-05	0.000542
11741150_NRCAM	0.350998	8.89E-05	0.000543
11757564_ADI1	-0.33266	8.89E-05	0.000543

11719032_FADS3	0.361199	8.90E-05	0.000543
11730444_CCNJ	0.413021	8.90E-05	0.000543
11742713_TMEM223	-0.29677	8.90E-05	0.000543
11734103_FGGY	0.353362	8.90E-05	0.000543
11721488_DLC1	0.450337	8.91E-05	0.000544
11728263_LUC7L	0.475885	8.91E-05	0.000544
11724139_SACS	-0.33319	8.91E-05	0.000544
11750334_REL	0.395178	8.92E-05	0.000544
11722314_RAPGEF6	0.359932	8.93E-05	0.000544
11758854_ATL3	-0.29948	8.93E-05	0.000544
11755175_HSPA12A	-0.51415	8.93E-05	0.000544
11718769_LAMTOR3	-0.47105	8.93E-05	0.000545
11755406_NPLOC4	0.274327	8.94E-05	0.000545
11716150_PMEPA1	0.605831	8.94E-05	0.000545
11718738_ST3GAL1	0.36063	8.94E-05	0.000545
11751269_SUPT3H	0.505783	8.95E-05	0.000545
11731457_MFN1	0.272342	8.96E-05	0.000546
11758820_DNAJC10	-0.59944	8.98E-05	0.000547
11744926_KTN1	0.259064	8.98E-05	0.000547
11751217_CEACAM1	0.432593	8.98E-05	0.000547
11757507_DSP	-0.34252	8.99E-05	0.000547
11736141_ITSN1	-0.31634	9.00E-05	0.000548
11751502_SLC25A19	-0.30996	9.00E-05	0.000548
11759345_RHOJ	0.487656	9.00E-05	0.000548
11718727_ZNF655	0.302867	9.00E-05	0.000548
11757905_KRT18	0.300919	9.02E-05	0.000549
11727713_KCNJ12	0.603857	9.04E-05	0.00055
11721361_FAM172A	-0.2743	9.04E-05	0.00055
11747389_SRSF5	-0.31063	9.05E-05	0.000551
11727411_MRPL19	-0.29561	9.06E-05	0.000551
11723272_FEM1C	-0.33114	9.06E-05	0.000551
11732490_NPHP3	0.489715	9.07E-05	0.000551
11756829_HEXA	0.295346	9.07E-05	0.000552
11721926_BCL9	-0.30762	9.08E-05	0.000552
11722712_PPOX	-0.28868	9.08E-05	0.000552
11741192_RBKS	0.547947	9.09E-05	0.000552
11724435_TPK1	0.400764	9.09E-05	0.000552
11757639_WDR61	0.27081	9.09E-05	0.000552
11716802_SETD5	0.363329	9.09E-05	0.000552
11721429_TBX3	0.484655	9.11E-05	0.000553
11718981_TAGLN2	-0.30406	9.12E-05	0.000554
11720895_SOS1	0.277379	9.12E-05	0.000554
11722331_INO80	0.320805	9.12E-05	0.000554
11749793_ANXA4	0.336183	9.13E-05	0.000554
11719334_RCBTB1	-0.36661	9.13E-05	0.000554
11726988_EIF2AK1	-0.28138	9.13E-05	0.000554
11733184_OSMR	0.794077	9.15E-05	0.000555
11744375_C20orf27	-0.27007	9.17E-05	0.000556
11725619_GAD1	1.072038	9.17E-05	0.000556
11722184_SPTSSA	-0.36807	9.17E-05	0.000556
11732466_CXCL11	0.804198	9.18E-05	0.000557

11719583_CNNM3	-0.58499	9.18E-05	0.000557
11732535_FANCD2	-0.34113	9.18E-05	0.000557
11721870_HIF1AN	-0.27455	9.19E-05	0.000557
11740338_AGFG1	-0.52042	9.21E-05	0.000559
11722600_P3H4	-0.43575	9.22E-05	0.000559
11723581_SMIM13	0.335136	9.22E-05	0.000559
11748706_RALY	-0.35873	9.22E-05	0.000559
11744650_PHLDB1	0.307434	9.22E-05	0.000559
11723277_CREBZF	0.292114	9.22E-05	0.000559
11733379_DCAF4L1	0.627845	9.23E-05	0.000559
11749155_NET1	0.307135	9.24E-05	0.00056
11733930_LDHAL6B	-0.39914	9.25E-05	0.000561
11733329_CEP57L1	-0.79464	9.27E-05	0.000561
11754524_TFPI2	0.364698	9.27E-05	0.000561
11739421_RCC1	-0.49121	9.28E-05	0.000562
11757054_TMEM43	0.283914	9.28E-05	0.000562
11729722_PIAS2	0.298391	9.30E-05	0.000563
11746333_DENND4C	0.414062	9.31E-05	0.000563
11743802_RC3H2	0.313454	9.31E-05	0.000564
11760097_GTF2I ///	-0.29349	9.32E-05	0.000564
11722923_FAM122B	-0.3159	9.32E-05	0.000564
11720511_CARHSP1	-0.44761	9.33E-05	0.000564
11723858_DNAAF2	-0.57895	9.33E-05	0.000564
11723254_HAT1	-0.40747	9.34E-05	0.000565
11752349_TRAPPC11	0.294741	9.34E-05	0.000565
11750638_SF3A1	-0.30907	9.34E-05	0.000565
11763688_FMNL1	-0.35591	9.34E-05	0.000565
11716598_CHMP1A	-0.29783	9.34E-05	0.000565
11763512_RMDN2	0.434753	9.34E-05	0.000565
11721534_TAF2	0.260756	9.34E-05	0.000565
11727009_ALDH7A1	-0.30024	9.35E-05	0.000565
11734468_LOC101929	-0.44151	9.35E-05	0.000565
11721323_TBC1D10B	-0.26714	9.35E-05	0.000565
11745377_PTRF	-0.34027	9.36E-05	0.000565
11724492_LIG1	-0.42502	9.36E-05	0.000565
11763514_RCAN1	-0.30121	9.36E-05	0.000565
11723258_PKMYT1	-0.3552	9.36E-05	0.000565
11732391_EHMT2	-0.44185	9.37E-05	0.000566
11751346_RBM22	0.282543	9.37E-05	0.000566
11747724_NUDT12	-0.41678	9.39E-05	0.000567
11743419_ZKSCAN5	0.425105	9.39E-05	0.000567
11717077_ZFAND6	0.284208	9.39E-05	0.000567
11759185_GPR153	-0.27091	9.40E-05	0.000567
11728784_ING3	0.384357	9.41E-05	0.000568
11739157_TMX4	0.293645	9.41E-05	0.000568
11757903_COL4A5	0.569598	9.42E-05	0.000568
11757166_HSPA8	-0.34071	9.42E-05	0.000568
11724260_TRIO	0.517104	9.43E-05	0.000569
11717686_JAK1	0.256996	9.43E-05	0.000569
11749927_GNAQ	0.320494	9.45E-05	0.00057
11726537_KANK2	-0.30377	9.45E-05	0.00057

11715815_PSMC3	-0.30838	9.46E-05	0.00057
11717214_NSMAF	0.328599	9.46E-05	0.00057
11753571_HMGA1	-0.30743	9.47E-05	0.00057
11759559_SLC25A37	-0.40218	9.47E-05	0.00057
11719851_RNF139	0.337866	9.48E-05	0.000571
11726708_FAM195B	-0.39415	9.48E-05	0.000571
11743712_ARID4A	0.615102	9.50E-05	0.000572
11728132_EXOC8	0.321122	9.51E-05	0.000572
11715747_MINOS1-NB	-0.33257	9.51E-05	0.000572
11753715_MGST2	0.288393	9.51E-05	0.000573
11744440_CDK5RAP3	-0.26722	9.52E-05	0.000573
11745998_ZNF252P	0.286879	9.53E-05	0.000573
11746818_PGK1	-0.29454	9.53E-05	0.000573
11732210_MON2	0.308917	9.53E-05	0.000573
11727425_CANX	-0.26817	9.54E-05	0.000574
11718065_PVRL2	0.310755	9.55E-05	0.000575
11736726_GEN1	-0.31575	9.55E-05	0.000575
11721113_RPL41	-0.31693	9.55E-05	0.000575
11728974_ACYP1	-0.3326	9.56E-05	0.000575
11756597_TRMT44	0.343554	9.56E-05	0.000575
11751920_STARD13	0.369598	9.56E-05	0.000575
11747232_FGD4	0.50697	9.56E-05	0.000575
11723669_SEMA3C	0.400031	9.57E-05	0.000575
11748020_XPNPEP1	0.350141	9.57E-05	0.000575
11728508_FKTN	-0.46083	9.58E-05	0.000576
11719206_ANKLE2	0.309827	9.58E-05	0.000576
11729189_MAX	0.286327	9.59E-05	0.000576
11747134_TRPS1	0.310048	9.59E-05	0.000576
11730938_PYCR1	-0.33284	9.59E-05	0.000576
11745040_SUSD1	0.330028	9.60E-05	0.000576
11753597_SRP9	-0.26754	9.60E-05	0.000576
11715884_TMEM248	0.321651	9.60E-05	0.000576
11751315_SLC39A1	-0.37358	9.61E-05	0.000577
11748459_ARL13B	0.347365	9.61E-05	0.000577
11742835_UBXN4	0.399886	9.61E-05	0.000577
11725349_TFDP1	-0.28506	9.62E-05	0.000577
11727186_WNT5A	0.252995	9.64E-05	0.000578
11748742_ZNF461	0.964901	9.64E-05	0.000578
11729648_CENPU	-0.38956	9.64E-05	0.000578
11758577_ELOVL7	-0.37516	9.65E-05	0.000578
11755606_IFI30	0.297873	9.65E-05	0.000578
11742733_MORF4L1	0.250037	9.65E-05	0.000579
11740689_PHACTR2	0.325402	9.66E-05	0.000579
11742797_C18orf21	0.288634	9.66E-05	0.000579
11748230_CORO1A	-0.38047	9.66E-05	0.000579
11742925_LAMTOR1	0.293178	9.66E-05	0.000579
11755520_CERS6	-0.42905	9.67E-05	0.000579
11729989_CACNA1A	-0.32847	9.67E-05	0.000579
11733643_NECAP2	0.353264	9.67E-05	0.000579
11756743_LAMTOR1	0.295122	9.67E-05	0.000579
11721282_POLR2H	-0.30863	9.68E-05	0.00058

11723322_DDX11 ///	-0.30193	9.68E-05	0.00058
11744345_TUBA1A //	-0.25634	9.69E-05	0.00058
11719157_ASRGL1	-0.39912	9.69E-05	0.00058
11743122_PRKAR1B	-0.34467	9.69E-05	0.00058
11720317_DAD1	-0.31015	9.69E-05	0.00058
11740178_ANKLE1	-0.36022	9.70E-05	0.000581
11754520_MBNL2	0.262241	9.70E-05	0.000581
11736566_LEF1	-0.40513	9.71E-05	0.000581
11752296_DDX3X	0.344144	9.71E-05	0.000581
11717882_ZNF512	-0.32743	9.72E-05	0.000581
11733186_OSMR	0.472374	9.72E-05	0.000581
11740377_PXMP4	-0.45352	9.73E-05	0.000582
11715718_ZNHIT1	0.281761	9.73E-05	0.000582
11756478_UTP11L	-0.31347	9.74E-05	0.000583
11756213_PGK1	-0.27215	9.74E-05	0.000583
11726697_ZBTB7A	0.259411	9.74E-05	0.000583
11729414_LRRC8A	-0.33951	9.76E-05	0.000583
11729674_SPRTN	0.311817	9.76E-05	0.000583
11757113_SNHG1	-0.28816	9.76E-05	0.000583
11744047_MAP1B	0.262191	9.77E-05	0.000584
11726071_MAP4K4	-0.3355	9.77E-05	0.000584
11757369_MAN2B1	0.387477	9.79E-05	0.000585
11739800_VCPIP1	0.311427	9.79E-05	0.000585
11754301_ATP2A2	-0.46006	9.79E-05	0.000585
11760031_NSUN5P1 /	0.317612	9.80E-05	0.000585
11716390_BACE1	0.417263	9.81E-05	0.000586
11750188_AGPAT1	-0.38986	9.81E-05	0.000586
11739942_SEPSECS	-0.3465	9.81E-05	0.000586
11715706_MGEA5	0.427035	9.82E-05	0.000586
11726023_EDN1	0.649949	9.83E-05	0.000587
11763521_PPIC	0.485832	9.83E-05	0.000587
11746240_RWDD2A	0.305006	9.83E-05	0.000587
11756881_PCDH20	0.511604	9.83E-05	0.000587
11734776_CTTN	0.349953	9.84E-05	0.000587
11727497_ZRANB1	0.323976	9.84E-05	0.000587
11721326_C3orf14	-0.28289	9.85E-05	0.000587
11727891_PRELP	-0.30474	9.85E-05	0.000588
11721159_DRAM2	0.295841	9.86E-05	0.000588
11720089_NRSN2	-0.43368	9.86E-05	0.000588
11741648_MBNL1	0.265211	9.86E-05	0.000588
11728316_CD274	1.036855	9.86E-05	0.000588
11742261_TTC26	0.375085	9.86E-05	0.000588
11748371_CYP20A1	0.341432	9.87E-05	0.000588
11721560_AHCYL2	0.302944	9.90E-05	0.00059
11733375_MSI2	0.437858	9.92E-05	0.000591
11759450_DDI2	0.495586	9.94E-05	0.000592
11722197_NMT1	-0.39815	9.94E-05	0.000592
11739395_MFHAS1	0.319368	9.98E-05	0.000594
11718266_SMAD3	-0.3482	9.98E-05	0.000594
11718966_NUFIP2	0.311415	9.99E-05	0.000594
11748027_MBD3	-0.27984	9.99E-05	0.000594

11725680_SMC3	-0.32048	9.99E-05	0.000594
11717189_DNAJA1	0.257673	9.99E-05	0.000594
11757198_SCAND2P	0.360579	1.00E-04	0.000595
11723421_BTF3L4	0.2932	0.0001	0.000595
11749910_EIF2S1	-0.26786	0.0001	0.000596
11725041_FGF2	0.358185	0.0001	0.000596
11717699_DCAF8	0.391707	0.0001	0.000596
11727516_RAVER2	-0.59122	0.0001	0.000596
11735269_SLC41A2	0.841424	0.0001	0.000597
11756647_XPNPEP3	0.321823	0.0001	0.000597
11731151_LIX1L	0.321524	0.0001	0.000597
11757654_UCP2	-0.39186	0.000101	0.000598
11757050_TAGLN2	-0.32429	0.000101	0.000598
11719723_CCDC93	0.297316	0.000101	0.000598
11723600_PHLPP2	0.798158	0.000101	0.000598
11716716_CTSB	-0.29958	0.000101	0.000598
11726299_LGALS8	0.295736	0.000101	0.000598
11743756_MKRN2	0.344832	0.000101	0.000598
11748375_TEK	0.50472	0.000101	0.000599
11752587_SEMA6B	-0.30114	0.000101	0.000599
11743776_GLT8D2	0.277908	0.000101	0.0006
11750415_TUBB4B	-0.26586	0.000101	0.0006
11753161_HFE	-0.31861	0.000101	0.0006
11739927_OPA1	-0.56406	0.000101	0.000601
11718429_EMD	-0.35706	0.000101	0.000602
11743482_FAM21A //	0.319284	0.000101	0.000602
11747690_FAM21A //	0.319284	0.000101	0.000602
11719073_HSPE1-MOB	0.258154	0.000101	0.000602
11736656_GPR176	0.570372	0.000102	0.000603
11756775_KLHL42	0.366742	0.000102	0.000603
11720805_PAGR1	-0.45302	0.000102	0.000603
11742719_SLTM	0.347178	0.000102	0.000603
11718542_PARP6	0.384555	0.000102	0.000603
11740181_PTPN12	0.303994	0.000102	0.000603
11746796_DAG1	-0.29675	0.000102	0.000604
11728226_CASP7	-0.31523	0.000102	0.000604
11735581_MBNL3	0.474037	0.000102	0.000604
11726609_MTF2	0.503356	0.000102	0.000604
11732209_MON2	0.377728	0.000102	0.000605
11747137_ERAP2	0.753154	0.000102	0.000605
11747758_RTCA	0.273392	0.000102	0.000605
11754475_IQCK	0.40321	0.000102	0.000605
11718288_UBL3	0.285656	0.000102	0.000605
11735200_N4BP2	0.483431	0.000102	0.000606
11758158_FOXP1	0.334822	0.000102	0.000606
11729259_ZNF644	0.324903	0.000102	0.000606
11715711_AKR1C3	0.356244	0.000102	0.000606
11747542_MPDU1	-0.42774	0.000103	0.000607
11717016_LAMTOR2	-0.29676	0.000103	0.000607
11728334_ELL	0.478574	0.000103	0.000607
11757011_SPRYD7	0.305959	0.000103	0.000608

11759670_CEP57	-0.44895	0.000103	0.000608
11724113_SIX1	-0.28986	0.000103	0.000608
11760913_ASAH2B	0.329837	0.000103	0.000609
11749674_SHTN1	0.404317	0.000103	0.000609
11727017_PAPPA	0.361626	0.000103	0.000609
11756334_ANXA3	0.527968	0.000103	0.000609
11745988_ADPGK	-0.35536	0.000103	0.000609
11737952_RALGDS	0.26263	0.000103	0.000609
11752669_SREK1IP1	0.317098	0.000103	0.00061
11749033_NSMCE4A	-0.33199	0.000103	0.00061
11747761_MAGOH	-0.31735	0.000103	0.00061
11719859_SLC2A4RG	-0.29114	0.000103	0.00061
11721455_SERPINA1	0.585837	0.000103	0.00061
11763975_MRPS11	-0.29068	0.000103	0.000611
11719789_TUBB2A	0.282174	0.000103	0.000611
11722215_POLE4	-0.27009	0.000103	0.000611
11747591_GLIPR1	0.381971	0.000104	0.000612
11720977_WARS2	0.433773	0.000104	0.000612
11743177_RSF1	0.264333	0.000104	0.000613
11729697_C2orf69	0.488844	0.000104	0.000613
11736171_RABL6	-0.28663	0.000104	0.000614
11760537_HEXA	0.28578	0.000104	0.000614
11745700_RAET1G //	-0.33854	0.000104	0.000614
11715908_MYO6	0.359388	0.000104	0.000614
11717533_MAN2A2	0.266245	0.000104	0.000615
11734904_ZNF16	0.351895	0.000104	0.000615
11750202_LAMP2	0.367881	0.000104	0.000615
11718736_PCMTD1	0.599012	0.000104	0.000615
11762056_LOC100133	0.366691	0.000104	0.000615
11757591_PAN3	0.298551	0.000104	0.000615
11724993_FAM101B	-0.5633	0.000104	0.000616
11743566_ACBD3	0.268948	0.000105	0.000617
11723102_GNB4	-0.24705	0.000105	0.000617
11755883_PGPEP1	0.577777	0.000105	0.000617
11740112_LACC1	0.48955	0.000105	0.000617
11756464_HDGFRP2	-0.31988	0.000105	0.000617
11717962_KRAS	-0.32204	0.000105	0.000618
11719002_KDELR3	-0.34731	0.000105	0.000618
11723927_PNMA2	-0.3001	0.000105	0.000618
11725213_METTL7B	-0.37292	0.000105	0.000618
11726798_NEXN	0.695677	0.000105	0.000618
11724486_BFAR	-0.29812	0.000105	0.000619
11743434_CHST11	0.440677	0.000105	0.000619
11718283_EIF4EBP1	-0.30646	0.000105	0.000619
11722047_LDAH	0.502812	0.000105	0.000619
11750968_NFIC	-0.2853	0.000105	0.000619
11729768_DGAT1	-0.25583	0.000105	0.00062
11735241_ZC3H12C	0.643589	0.000105	0.00062
11754954_ENG	0.270783	0.000105	0.00062
11726558_PPP2CB	-0.25652	0.000105	0.00062
11719173_PAICS	-0.30134	0.000105	0.00062

11715763_PODXL	0.279954	0.000106	0.000621
11761738_RBMX	-0.36295	0.000106	0.000621
11733976_HAUS5	-0.45933	0.000106	0.000621
11752419_HIVEP1	0.416426	0.000106	0.000621
11737000_ZNF41	0.428595	0.000106	0.000622
11764244_THOC2	0.321447	0.000106	0.000622
11741386_NRF1	-0.40884	0.000106	0.000622
11755952_ARRB2	-0.4865	0.000106	0.000622
11734758_METTL6	0.305719	0.000106	0.000622
11740171_PBRM1	-0.28983	0.000106	0.000622
11720663_GNA11	-0.29823	0.000106	0.000622
11732251_RCOR3	0.36631	0.000106	0.000622
11744989_ARMC10	0.300758	0.000106	0.000622
11749794_ANXA4	0.307772	0.000106	0.000623
11748686_ZC3H15	0.29192	0.000106	0.000623
11722298_RAB29	0.326444	0.000106	0.000623
11718614_RABEP1	0.284666	0.000106	0.000624
11759049_ACSS3	0.51781	0.000107	0.000625
11726995_PINK1	-0.25636	0.000107	0.000625
11746483_MYO6	0.33415	0.000107	0.000625
11737823_POMK	-0.44825	0.000107	0.000626
11756429_OSTC	0.293934	0.000107	0.000626
11724262_TRIO	0.380979	0.000107	0.000627
11727583_EPHA3	0.333402	0.000107	0.000627
11724487_BFAR	-0.29767	0.000107	0.000627
11749514_ZNF641	0.401799	0.000107	0.000627
11752886_EHD1	0.407961	0.000107	0.000627
11719290_TMEM63A	-0.27719	0.000107	0.000627
11724144_RAB11FIP2	0.449362	0.000107	0.000628
11749173_PPP2R5D	-0.516	0.000107	0.000628
11747470_ZNF512	-0.28823	0.000107	0.000628
11731843_RUFY3	0.54416	0.000107	0.000628
11747301_SP100	0.39712	0.000107	0.000628
11754846_MLLT10	0.309476	0.000107	0.000628
11731024_ATXN7	0.262563	0.000107	0.000628
11724465_ANLN	-0.25692	0.000107	0.000628
11758669_RHOQ	0.296261	0.000108	0.000629
11728254_NMNAT2	0.368855	0.000108	0.000629
11750884_AKAP8L	0.547609	0.000108	0.00063
11721705_COL7A1	0.451494	0.000108	0.00063
11756871_SNX10	-0.41053	0.000108	0.00063
11743667_COL8A2	-0.54549	0.000108	0.00063
11742934_POC1A	-0.32859	0.000108	0.000631
11726592_PIGH	0.436154	0.000108	0.000631
11737005_ZNF782	0.483571	0.000108	0.000632
11719429_SOS2	0.350949	0.000108	0.000632
11716680_SLC39A9	0.386821	0.000108	0.000633
11725090_TASP1	0.39879	0.000108	0.000633
11743990_BRD4	0.365956	0.000108	0.000633
11763168_ALG13	0.295732	0.000108	0.000634
11723907_LARP1B	-0.30572	0.000109	0.000634

11742386_CASP1	0.393837	0.000109	0.000635
11719742_C8orf76	0.306763	0.000109	0.000637
11749046_WWOX	0.48677	0.000109	0.000637
11726165_ZNF518B	0.445976	0.000109	0.000637
11722573_ERAP2	0.433988	0.000109	0.000638
11756365_EWSR1 ///	-0.31475	0.000109	0.000638
11718520_KCMF1	-0.35766	0.000109	0.000639
11716867_FEM1A	-0.29233	0.000109	0.000639
11756230_FLNB	0.311151	0.000109	0.000639
11739434_ATP11B	0.456563	0.00011	0.000639
11743538_DNAJC18	-0.24736	0.00011	0.00064
11725750_GAL	-0.3776	0.00011	0.00064
11723781_AGPS	0.399317	0.00011	0.00064
11739223_NF1	0.655424	0.00011	0.000641
11763854_CD9	-0.31379	0.00011	0.000641
11720656_BBX	-0.32874	0.00011	0.000642
11727642_TRERF1	-0.63184	0.00011	0.000642
11738809_MARK3	0.288902	0.00011	0.000642
11722561_CDK4	-0.27138	0.00011	0.000642
11740730_FUOM	-0.26258	0.00011	0.000643
11720978_WARS2	0.345187	0.00011	0.000643
11723122_TRPM7	0.397923	0.00011	0.000643
11761752_CAMKMT	0.4261	0.00011	0.000643
11758548_PREP	0.306603	0.00011	0.000643
11759207_CNKSR2	0.38013	0.00011	0.000643
11724362_PIKFYVE	0.689748	0.00011	0.000644
11750297_MED23	0.372919	0.00011	0.000644
11722389_MTF1	-0.30356	0.000111	0.000644
11751997_MYO18A	-0.4332	0.000111	0.000644
11721907_SGPP1	0.357001	0.000111	0.000645
11718209_TMEM9B	0.266962	0.000111	0.000645
11731983_PTHLH	0.651317	0.000111	0.000645
11715825_PRMT1	-0.28435	0.000111	0.000645
11749672_MORC4	0.384664	0.000111	0.000645
11747709_PITPNB	0.305245	0.000111	0.000645
11722626_KIAA1033	0.386214	0.000111	0.000645
11727079_NUFIP1	0.33813	0.000111	0.000646
11743182_C16orf59	-0.46071	0.000111	0.000646
11717114_SUPT16H	-0.27788	0.000111	0.000646
11746201_NAB1	0.324012	0.000111	0.000646
11718251_TBC1D13	-0.37536	0.000111	0.000646
11744503_RSRP1	0.310111	0.000111	0.000646
11724842_ARMC4	0.911533	0.000111	0.000646
11745929_AHCY	-0.3795	0.000111	0.000646
11748411_FLII	0.292524	0.000111	0.000646
11735482_ZNF699	0.834921	0.000111	0.000646
11716956_CTDSP2	0.292707	0.000111	0.000647
11750264_TMEM19	-0.33168	0.000111	0.000647
11741137_CHN1	0.44905	0.000111	0.000647
11757476_TNNT1	-0.35081	0.000111	0.000647
11756882_RTTN	-0.40842	0.000112	0.000648

11753434_SLC3A2	0.290988	0.000112	0.000648
11722711_PPOX	-0.27863	0.000112	0.000648
11732999_ICAM1	0.497768	0.000112	0.000648
11723776_BRD3	-0.34926	0.000112	0.000648
11746107_CDC16	0.247681	0.000112	0.000648
11722911_USP13	-0.40462	0.000112	0.000649
11717494_SSNA1	-0.30506	0.000112	0.000649
11720131_SPIRE1	-0.5512	0.000112	0.000649
11731469_AKT2	-0.53504	0.000112	0.000649
11725122_GATAD1	0.295017	0.000112	0.00065
11723412_PA2G4	-0.26055	0.000112	0.00065
11743660_SNRNP27	0.258676	0.000112	0.00065
11761314_LOC100133	0.619856	0.000112	0.00065
11717668_POLR2K	-0.28337	0.000112	0.00065
11742278_CTH	0.355211	0.000112	0.00065
11745315_CTNNAL1	0.410185	0.000112	0.00065
11717642_CSTF3	0.318843	0.000112	0.000651
11739054_PTPN6	-0.3111	0.000112	0.000651
11732147_CAT	-0.30446	0.000113	0.000652
11754243_H2AFX	-0.2448	0.000113	0.000652
11724766_FASTKD2	-0.39855	0.000113	0.000652
11757791_DUSP3	-0.33877	0.000113	0.000653
11727946_SHPK	-0.63414	0.000113	0.000653
11758130_HEBP1	-0.42904	0.000113	0.000653
11718710_SLC9A1	0.26375	0.000113	0.000653
11717960_KCTD2	-0.49871	0.000113	0.000654
11742823_LACTB2	-0.42076	0.000113	0.000655
11728262_LUC7L	0.315228	0.000113	0.000656
11749883_FEZ1	0.309679	0.000113	0.000656
11719470_DCP1A	-0.28779	0.000113	0.000657
11763692_VTI1B	-0.35855	0.000114	0.000657
11758776_HMCES	-0.40129	0.000114	0.000657
11727800_POP4	-0.28145	0.000114	0.000657
11730439_AKAP9	0.334842	0.000114	0.000657
11734789_SRR	-0.24924	0.000114	0.000658
11718786_PAN2	-0.32038	0.000114	0.000658
11739256_FAM84B	0.310812	0.000114	0.000658
11736928_BMP2K	0.371045	0.000114	0.000659
11747472_PARPBP	-0.38109	0.000114	0.000659
11746497_MSI2	0.416189	0.000114	0.000659
11728568_CCDC61	-0.32932	0.000114	0.000659
11722287_SLC30A7	0.260261	0.000114	0.000659
11716667_MTHFD1	-0.25316	0.000114	0.000659
11751935_EGF	0.668728	0.000114	0.00066
11716452_MYO1C	0.312877	0.000114	0.00066
11724779_PGM2L1	0.270771	0.000114	0.000661
11757344_SNRPB	-0.30365	0.000114	0.000661
11735039_SHTN1	0.395355	0.000114	0.000661
11744367_ZMYND11	0.446835	0.000114	0.000661
11727038_MAVS	-0.24506	0.000115	0.000662
11753786_PARD6B	0.559549	0.000115	0.000662

11732304_CREB1	-0.25608	0.000115	0.000663
11718360_COIL	0.356845	0.000115	0.000663
11717726_HN1	0.253877	0.000115	0.000663
11731560_FAM89A	0.398296	0.000115	0.000664
11725221_SNTB1	0.302982	0.000115	0.000664
11728614_GPR3	-0.49871	0.000115	0.000664
11747733_LRRC14	-0.48596	0.000115	0.000664
11735083_FREM2	-0.37101	0.000115	0.000664
11715433_RBBP7	-0.32055	0.000115	0.000664
11756654_SUB1	-0.28671	0.000115	0.000665
11728402_ATP6V0A2	0.326155	0.000115	0.000665
11752470_EIF4B	0.271971	0.000115	0.000666
11725653_EMCN	0.582387	0.000115	0.000666
11723090_CXorf40A	0.336819	0.000116	0.000668
11756076_ATAD3A	-0.25091	0.000116	0.000668
11745769_NOTCH2	0.364566	0.000116	0.000668
11721506_DVL3	0.383246	0.000116	0.000668
11748468_DVL3	0.383246	0.000116	0.000668
11718298_CCDC25	0.289643	0.000116	0.000668
11718276_IFRD2	-0.2989	0.000116	0.000669
11757340_RHOQ	0.309853	0.000116	0.000669
11763702_WDR48	0.290523	0.000116	0.000669
11743252_CALD1	0.296716	0.000116	0.000669
11737779_KITLG	0.772366	0.000116	0.000669
11739944_NBPF15 //	0.293001	0.000116	0.000669
11749882_APH1A	-0.24488	0.000116	0.000669
11731062_ZNF516	-0.33845	0.000116	0.000669
11754697_ANAPC13	0.26863	0.000116	0.00067
11746615_UBE3C	0.36033	0.000116	0.00067
11717208_RXRB	-0.37174	0.000117	0.000671
11761943_MRE11A	-0.62294	0.000117	0.000671
11734911_WDR76	-0.36004	0.000117	0.000671
11756740_LRRC75A-A	0.259585	0.000117	0.000671
11729197_STX16	0.327399	0.000117	0.000672
11734692_TAF11	0.36755	0.000117	0.000672
11734865_PSMC3IP	-0.39117	0.000117	0.000672
11728396_SNX14	0.252555	0.000117	0.000672
11753046_MBNL2	0.306158	0.000117	0.000672
11753704_STMN1	-0.30842	0.000117	0.000673
11750204_TRUB1	-0.41678	0.000117	0.000673
11757653_CDK5RAP3	-0.29985	0.000117	0.000673
11735813_C8orf59	0.541039	0.000117	0.000673
11757621_MYLK	0.395223	0.000117	0.000673
11739477_SBF2	0.24679	0.000117	0.000674
11752316_CEP250	-0.30631	0.000117	0.000674
11740241_CAPS	-0.32118	0.000117	0.000674
11719683_MPC1	-0.32488	0.000117	0.000674
11720635_ATP5D	-0.32403	0.000117	0.000674
11754356_MRPS18B	-0.31803	0.000117	0.000674
11748361_MBNL1	0.246646	0.000118	0.000675
11723140_ZBTB11	0.289497	0.000118	0.000676

11754467_MED26	0.359785	0.000118	0.000676
11722810_SP1	-0.2797	0.000118	0.000676
11755104_KIAA1671	0.318309	0.000118	0.000677
11718915_RGS19	-0.30086	0.000118	0.000677
11719291_RRP36	0.299734	0.000118	0.000678
11739808_MAZ	-0.61877	0.000118	0.000678
11752609_OSBPL2	-0.29058	0.000118	0.000678
11731149_APOOL	0.314185	0.000118	0.000678
11730823_COX19	-0.35012	0.000118	0.000679
11755921_GTF3C5	-0.30261	0.000118	0.000679
11725677_STX4	0.399321	0.000119	0.00068
11761421_CD70	0.32674	0.000119	0.00068
11749229_SIRT5	-0.45919	0.000119	0.00068
11715150_PCDHA4	0.297795	0.000119	0.00068
11760491_CASP4	0.735405	0.000119	0.00068
11740676_CLPX	0.280241	0.000119	0.00068
11758209_ZNF644	0.280296	0.000119	0.00068
11719572_TRUB1	-0.3175	0.000119	0.00068
11735820_RIPPLY1	-0.33173	0.000119	0.00068
11732353_NT5C3B	0.295493	0.000119	0.00068
11730118_RPP38	0.271458	0.000119	0.000681
11716461_NELFB	-0.3842	0.000119	0.000681
11749925_ZNF346	0.581317	0.000119	0.000682
11758895_EIF2S2	-0.36472	0.000119	0.000682
11741121_ZNF700	0.297415	0.000119	0.000682
11733780_SC5D	0.280452	0.000119	0.000683
11739895_USP1	-0.28146	0.000119	0.000683
11756548_PKP4	0.423692	0.000119	0.000683
11726059_R3HCC1L	0.446857	0.000119	0.000683
11716599_CHMP1A	-0.25743	0.00012	0.000685
11757968_TERF2IP	0.350588	0.00012	0.000685
11739240_SNAPC3	0.312601	0.00012	0.000685
11716457_RHOBTB3	0.284976	0.00012	0.000685
11728693_LOC100996	0.582272	0.00012	0.000686
11753670_AP1S3	0.298904	0.00012	0.000686
11721135_IGFBP3	0.389977	0.00012	0.000686
11725627_NIPAL3	-0.27688	0.00012	0.000686
11741873_CSNK1E //	-0.32949	0.00012	0.000686
11727932_VTI1A	0.522233	0.00012	0.000687
11746467_HELLS	-0.39383	0.00012	0.000688
11724601_ERAP1	0.292555	0.00012	0.000688
11716310_CYBRD1	0.316616	0.00012	0.000688
11729564_DND1	0.300941	0.000121	0.000689
11724638_PAFAH2	0.336969	0.000121	0.000689
11739599_ZNF398	0.507179	0.000121	0.000689
11721919_ATP13A2	0.343702	0.000121	0.000689
11745552_CAB39	0.3102	0.000121	0.000689
11751817_MBNL2	0.25206	0.000121	0.000689
11727331_SPAG4	-0.60985	0.000121	0.00069
11720224_TBC1D1	-0.34986	0.000121	0.00069
11748318_LDAH	0.443056	0.000121	0.000691

11747512_ARHGDIA	-0.29946	0.000121	0.000691
11727699_TICRR	-0.28352	0.000121	0.000691
11718211_PYCR1	-0.4143	0.000121	0.000691
11730579_HECW2	0.488678	0.000121	0.000691
11757856_OSBP	0.268255	0.000121	0.000691
11733721_MAX	0.416456	0.000121	0.000691
11757535_PRR4	0.298265	0.000121	0.000691
11756340_UPF3A	0.248995	0.000121	0.000691
11717404_TDP2	-0.33052	0.000121	0.000692
11745956_PRRC2C	0.268074	0.000122	0.000693
11747013_PPA2	-0.28413	0.000122	0.000693
11755009_VCAN	0.295416	0.000122	0.000693
11734872_COMT	0.265608	0.000122	0.000693
11724212_LMAN1	-0.34491	0.000122	0.000694
11751710_SLC48A1	0.301715	0.000122	0.000694
11734970_NCOA4	0.286392	0.000122	0.000694
11746937_CTDSP2	0.312466	0.000122	0.000694
11724064_SLC25A19	-0.31648	0.000122	0.000695
11717315_DTD1	-0.26796	0.000122	0.000695
11752920_CDC27	0.318175	0.000122	0.000695
11755248_PPFIBP1	-0.31714	0.000122	0.000695
11750552_CARD16 //	0.796163	0.000122	0.000695
11744115_RPIA	-0.27256	0.000122	0.000695
11723905_ZNF75A	0.473169	0.000122	0.000695
11729859_H6PD	-0.3018	0.000122	0.000695
11725180_RUNX2	0.499851	0.000122	0.000696
11749834_SH2B1	-0.36491	0.000122	0.000696
11724446_PDE4D	0.313692	0.000122	0.000697
11719160_RAPH1	0.288233	0.000123	0.000697
11756503_LARP4B	0.315377	0.000123	0.000697
11734000_THRB	0.257731	0.000123	0.000697
11722281_PHC3	0.624775	0.000123	0.000697
11746310_WIZ	-0.45853	0.000123	0.000697
11759015_SPECC1	0.449205	0.000123	0.000698
11732174_FANCF	-0.27024	0.000123	0.000698
11715735_TMEM123	-0.30049	0.000123	0.000698
11717410_ANO6	0.287863	0.000123	0.000701
11727647_DNAJC25	0.320332	0.000123	0.000701
11763499_ZNF202	0.347152	0.000124	0.000702
11733207_ZNF480	0.313725	0.000124	0.000702
11726522_ZFHX3	-0.30087	0.000124	0.000702
11739831_WAC	0.282139	0.000124	0.000702
11757443_TIMM21	-0.32993	0.000124	0.000702
11719983_INPP5A	-0.42253	0.000124	0.000704
11742690_UBE2I	-0.31955	0.000124	0.000704
11754660_GOLT1A	0.731927	0.000124	0.000704
11718191_VEZF1	-0.30104	0.000124	0.000704
11724895_GATAD2B	-0.36942	0.000124	0.000705
11749900_PPP6R3	0.290928	0.000124	0.000705
11754959_AK4 /// A	0.28246	0.000124	0.000706
11721818_SH3BP5L	-0.36362	0.000124	0.000706

11752037_MAVS	-0.36713	0.000125	0.000707
11723592_LRRC8C	-0.49305	0.000125	0.000707
11717228_CDR2 ///	0.303239	0.000125	0.000707
11742119_NFYC	-0.33727	0.000125	0.000708
11728982_C8orf48	0.450051	0.000125	0.000709
11732140_COX16 ///	-0.59669	0.000125	0.000709
11751803_SPC24	-0.71975	0.000125	0.00071
11721633_TMEM97	-0.31405	0.000125	0.00071
11727101_IGF2BP1	0.245343	0.000125	0.000711
11718316_SLC25A10	-0.51302	0.000126	0.000711
11757962_SRSF3	-0.29464	0.000126	0.000711
11754717_TPP2	0.261548	0.000126	0.000711
11718760_WLS	-0.33093	0.000126	0.000711
11731381_NHLRC3	0.282413	0.000126	0.000711
11728481_ZNF181 //	0.310728	0.000126	0.000711
11757660_SNRPB	-0.25782	0.000126	0.000712
11758016_CCDC34	-0.24595	0.000126	0.000713
11747635_PYGB	-0.29064	0.000126	0.000713
11758143_DUSP8 ///	0.345289	0.000126	0.000713
11759767_MR1	0.482264	0.000126	0.000713
11729104_UBA52	-0.42317	0.000126	0.000713
11720760_IVNS1ABP	0.323694	0.000126	0.000714
11737828_ZKSCAN8	0.450707	0.000126	0.000715
11716631_SERPINB2	0.505469	0.000127	0.000715
11739659_YIF1B	-0.28465	0.000127	0.000715
11758333_RNF138 //	0.333605	0.000127	0.000716
11735044_TRIM5	0.326072	0.000127	0.000716
11758596_ZRANB1	0.344392	0.000127	0.000716
11730350_TLDC1	0.393529	0.000127	0.000717
11735494_SIN3A	-0.31872	0.000127	0.000717
11751955_ABCC5	0.392493	0.000127	0.000718
11728256_GLTSCR2	0.295413	0.000127	0.000718
11756255_YWHAH	-0.25897	0.000127	0.000718
11759853_RPS27A	-0.4111	0.000127	0.000719
11744052_SLC11A2	0.32897	0.000127	0.000719
11738846_GFM2	0.357327	0.000127	0.000719
11742757_GTF2H1	0.343923	0.000127	0.00072
11756416_KIZ	-0.29889	0.000127	0.00072
11753646_CFL1	-0.25738	0.000128	0.00072
11743178_RSF1	0.260099	0.000128	0.000721
11719269_RBM22	0.303077	0.000128	0.000721
11732491_BRAF ///	0.469621	0.000128	0.000721
11757520_ACBD6 ///	0.283927	0.000128	0.000721
11747634_PYGB	-0.27066	0.000128	0.000722
11726031_CSNK1G3	0.310354	0.000128	0.000723
11757358_SCOC	-0.30801	0.000128	0.000724
11718232_KTI12 ///	0.270972	0.000128	0.000724
11733892_FKBP14	0.255219	0.000129	0.000725
11739569_ASH1L	0.269774	0.000129	0.000725
11749763_UBE2I	-0.27716	0.000129	0.000725
11732520_RAD51C	0.256387	0.000129	0.000725

11748312_TMEM161B	0.345685	0.000129	0.000725
11724185_SART3	-0.31405	0.000129	0.000725
11759211_LHX1	-0.43234	0.000129	0.000725
11722398_RWDD4	-0.2887	0.000129	0.000725
11757385_RPL13A //	-0.24053	0.000129	0.000727
11723633_IRF2BPL	-0.24812	0.000129	0.000727
11754447_RPS6KA5	0.263157	0.000129	0.000727
11726962_SGTB	0.3241	0.000129	0.000727
11736337_RUSC1	-0.27252	0.000129	0.000727
11758873_HPSE	-0.47743	0.000129	0.000729
11744723_APH1A	-0.28501	0.000129	0.000729
11737051_HRH1	0.77592	0.000129	0.000729
11724490_AKR1B10	0.523508	0.000129	0.000729
11759553_TIMM8B	0.431816	0.00013	0.000729
11736036_TADA3	-0.29815	0.00013	0.000729
11739376_SREK1	0.27127	0.00013	0.000731
11757865_GADD45B	0.493466	0.00013	0.000731
11754432_RBMX	-0.25537	0.00013	0.000731
11723937_SNX1	0.321227	0.00013	0.000732
11740744_TPM2	0.283951	0.00013	0.000732
11742091_TRAF3	0.325242	0.00013	0.000732
11726373_HAUS8	-0.37342	0.00013	0.000732
11737658_TMEM244	0.378329	0.00013	0.000733
11718518_KCMF1	0.265017	0.00013	0.000733
11715909_MYO6	0.329615	0.00013	0.000733
11744467_SYNCRIP	-0.32696	0.00013	0.000733
11744596_LYPD6B	0.490021	0.00013	0.000733
11735731_PRKRIR	0.2701	0.00013	0.000733
11743768_DLL3	-0.33224	0.000131	0.000733
11740278_PAM	0.244651	0.000131	0.000734
11744574_INO80	0.421085	0.000131	0.000734
11759091_MIPOL1	-0.47393	0.000131	0.000735
11715784_PRR4	0.2676	0.000131	0.000735
11747008_PAFAH1B1	0.314272	0.000131	0.000735
11715951_KCTD12	-0.39229	0.000131	0.000736
11715999_CAB39	0.389919	0.000131	0.000736
11743485_NAA25	0.360323	0.000131	0.000738
11745011_PKMYT1	-0.54961	0.000132	0.000739
11722906_MIA3	0.339255	0.000132	0.000739
11754400_SOX12	-0.29267	0.000132	0.000739
11739352_ARMC8	0.545335	0.000132	0.00074
11730048_SLITRK5	-0.33108	0.000132	0.00074
11725484_DIRC2	-0.24119	0.000132	0.00074
11719409_HIPK2	0.289888	0.000132	0.00074
11743102_PTCD3	0.323187	0.000132	0.00074
11725644_TET2	0.468824	0.000132	0.00074
11750578_PYGB	-0.26048	0.000132	0.00074
11717033_KIAA0141	-0.23791	0.000132	0.00074
11752238_UNKL	-0.38101	0.000132	0.00074
11758709_RDH11	-0.26765	0.000132	0.000741
11730378_PHF14	0.749221	0.000132	0.000741

11731612_PCID2	0.300409	0.000132	0.000741
11751760_LSM12	0.249821	0.000132	0.000742
11722776_PRKD1	-0.31069	0.000133	0.000744
11743011_GPR137B	0.284216	0.000133	0.000744
11754525_RNF146	0.278196	0.000133	0.000744
11751059_PIN1	-0.32538	0.000133	0.000744
11736540_TMEM170A	0.303577	0.000133	0.000744
11725473_PAQR8	-0.38708	0.000133	0.000746
11730110_SLC23A2	0.549318	0.000133	0.000746
11736035_EXOSC2	-0.28037	0.000133	0.000747
11748857_AK3	0.288576	0.000134	0.000748
11731501_UTP23	0.274175	0.000134	0.000748
11746198_METTL9	-0.96658	0.000134	0.000748
11755493_EXOC6	0.329243	0.000134	0.000748
11720140_RILP	-0.46989	0.000134	0.000748
11721554_COLGALT1	-0.29015	0.000134	0.000749
11745849_PPP6R3	0.330444	0.000134	0.000749
11722425_NEDD4L	-0.37346	0.000134	0.00075
11721441_RSRP1	0.434921	0.000134	0.00075
11751802_BFAR	-0.33111	0.000134	0.00075
11723504_PDPR	-0.30157	0.000134	0.00075
11756114_RBM10	-0.25387	0.000134	0.00075
11746103_DTD1	-0.32232	0.000134	0.000751
11736129_SRSF5	-0.35574	0.000134	0.000751
11756603_FAM206A	0.378726	0.000134	0.000751
11751265_PTOV1	-0.27005	0.000135	0.000752
11755809_NBN	-0.47008	0.000135	0.000752
11727263_ENTPD7	0.6381	0.000135	0.000752
11723406_SCMH1	-0.26235	0.000135	0.000752
11758227_ROBO1	0.299875	0.000135	0.000752
11746123_YY1AP1	0.267101	0.000135	0.000753
11724466_ANLN	-0.24641	0.000135	0.000753
11730707_VGLL2	0.625586	0.000135	0.000753
11732463_TERF1	-0.32063	0.000135	0.000753
11756864_ZFAND3	0.339522	0.000135	0.000753
11723530_GOSR1	0.3094	0.000135	0.000753
11717012_VAMP8	0.328311	0.000135	0.000754
11731698_ALG10 ///	-0.35248	0.000135	0.000755
11728885_TM6SF1	-0.43008	0.000135	0.000755
11722185_SPTSSA	-0.49894	0.000135	0.000755
11764007_CDKL3	0.320936	0.000135	0.000755
11743161_HILPDA	-0.30294	0.000135	0.000755
11721997_PARP14	0.268374	0.000136	0.000756
11732394_WWC2	0.294055	0.000136	0.000756
11725877_SCARA3	-0.45992	0.000136	0.000756
11716576_CCDC6	0.311867	0.000136	0.000756
11729631_AP3M1	-0.27679	0.000136	0.000756
11751685_C5orf22	0.332185	0.000136	0.000757
11750788_CSNK1G1	0.438437	0.000136	0.000757
11758367_NACC1	-0.32578	0.000136	0.000757
11754834_INTS6	0.308625	0.000136	0.000757

11717921_YAP1	-0.29369	0.000136	0.000758
11754335_TXNRD3 //	0.372073	0.000136	0.000758
11717663_KLHL12	-0.34504	0.000136	0.000759
11735873_FGFR4	-0.32336	0.000136	0.000759
11721341_NCOA1	0.485197	0.000136	0.000759
11757879_DBNL	-0.24378	0.000136	0.00076
11728787_PTPRR	0.77294	0.000137	0.000761
11750557_ZNF271P	0.420111	0.000137	0.000761
11723477_SLC17A5	0.322819	0.000137	0.000761
11746554_SPARC	0.254863	0.000137	0.000761
11758981_NUTF2	-0.24351	0.000137	0.000762
11717178_STAT5A	-0.29549	0.000137	0.000763
11733774_RPL37	-0.30619	0.000137	0.000764
11719425_FAM220A	-0.28384	0.000137	0.000764
11754567_FBXL12	0.251779	0.000137	0.000764
11745337_DNAJB12	-0.23695	0.000138	0.000765
11725869_HOXC4	-0.31597	0.000138	0.000765
11724187_SART3	-0.32233	0.000138	0.000765
11757040_CNP	-0.30598	0.000138	0.000765
11744359_MBD3	-0.34632	0.000138	0.000765
11745954_KIAA0907	0.342818	0.000138	0.000765
11725835_NFKBIB	0.404279	0.000138	0.000765
11739558_RFWD3	-0.3576	0.000138	0.000765
11747110_PASK	-0.34661	0.000138	0.000765
11756995_GNB2	-0.24692	0.000138	0.000766
11757383_CTSH	-0.36833	0.000138	0.000766
11757661_SNRPB	-0.2419	0.000138	0.000766
11743320_DST	0.454492	0.000138	0.000766
11736995_PPT2	-0.33932	0.000138	0.000767
11733203_SH3TC2	0.251381	0.000138	0.000767
11738876_PPP2R5C	-0.26151	0.000138	0.000768
11728021_CHCHD4	-0.288	0.000138	0.000768
11729088_C8orf34	0.772239	0.000138	0.000768
11757984_RAD17	-0.25397	0.000138	0.000769
11727766_BNC2	-0.37942	0.000138	0.000769
200068_PMCANX	-0.25639	0.000139	0.000769
11740611_ZNF121	0.319573	0.000139	0.000769
11736117_ZFAND5	0.255484	0.000139	0.000769
11759848_KIF16B	0.370954	0.000139	0.000769
11753117_SMAD4	0.296011	0.000139	0.000769
11745878_NFKBIA	0.365585	0.000139	0.000769
11716792_DKFZp686K	0.308712	0.000139	0.00077
11736048_ANTXR2	0.278433	0.000139	0.00077
11729729_METRN	-0.25451	0.000139	0.00077
11738053_PLAUR	0.266116	0.000139	0.000771
11719463_LSR	-0.32368	0.000139	0.000771
11755763_NUP188	-0.34547	0.000139	0.000772
11726881_IDS	0.33369	0.000139	0.000772
11715959_SREBF1	-0.32915	0.000139	0.000772
11734045_KIAA0408	0.465198	0.000139	0.000772
11742225_MAP3K7	0.279011	0.000139	0.000773

11719771_EXOC7	-0.37811	0.00014	0.000774
11733977_HAUS5	-0.28853	0.00014	0.000774
11760712_ITGA10	-0.39584	0.00014	0.000775
11718654_PKD2	0.286357	0.00014	0.000775
11723507_ZNF609	0.381493	0.00014	0.000775
11744265_LAMB2	0.410886	0.00014	0.000776
11763208_CENPI	-0.64524	0.00014	0.000776
11715517_SLC25A39	-0.26654	0.00014	0.000777
11742681_ERP29	-0.33608	0.000141	0.000778
11744715_SLC38A6	-0.26414	0.000141	0.000778
11748211_OSBPL9	0.26609	0.000141	0.000778
11728795_LSM14A	-0.31348	0.000141	0.000779
11746697_SLC3A2	0.26967	0.000141	0.000779
11734625_TRIM49 //	-0.85482	0.000141	0.000779
11742843_RXRA	-0.28465	0.000141	0.000779
11716926_EXOC3	0.27703	0.000141	0.000779
11727903_IL13RA1	0.337334	0.000141	0.000779
11755073_PRPF39	0.382899	0.000141	0.00078
11735737_SH2B1	-0.32743	0.000141	0.00078
11740745_OPA1	-0.58027	0.000141	0.00078
11715839_SDHC	-0.51162	0.000141	0.00078
11754294_STK40	0.295896	0.000141	0.000781
11750734_ZNF782	0.324427	0.000142	0.000782
11725244_SPAST	0.472882	0.000142	0.000782
11731157_ERCC4	0.344091	0.000142	0.000783
11755859_RNF41	-0.34908	0.000142	0.000784
11717670_NDUFB6	-0.24728	0.000142	0.000784
11758531_GALE	0.257352	0.000142	0.000785
11722678_STEAP3	-0.33146	0.000142	0.000786
11744839_KPNB1	-0.3862	0.000142	0.000786
11723721_SLAIN2	0.313317	0.000142	0.000787
11726041_STK17B	0.493222	0.000143	0.000787
11752016_AP4E1	0.391073	0.000143	0.000787
11751763_LDAH	0.480077	0.000143	0.000787
11727134_PUS7L	0.382954	0.000143	0.000787
11759959_SCLT1	0.420986	0.000143	0.000787
11747222_CAPN1	-0.42521	0.000143	0.000789
11719517_SLC9A6	0.299983	0.000143	0.00079
11717998_MPLKIP	-0.30175	0.000143	0.000791
11745196_ADI1	-0.29142	0.000143	0.000791
11718971_CD46	0.263916	0.000143	0.000791
11742930_BIRC6	0.336495	0.000143	0.000791
11751384_ANKRD12	0.314894	0.000143	0.000791
11724552_HPCAL1	-0.25531	0.000143	0.000791
11729309_CLMP	-0.32816	0.000143	0.000791
11748286_FAM219B	0.330902	0.000144	0.000793
11760444_HLA-C	0.305947	0.000144	0.000793
11746881_ZNF155	0.721861	0.000144	0.000793
11715846_EFEMP1	0.465705	0.000144	0.000793
11719761_ARRDC4	-0.41776	0.000144	0.000794
11737876_VMAC	-0.34684	0.000144	0.000795

11741437_SPRYD7	0.247226	0.000144	0.000795
11718362_HS6ST1	-0.29197	0.000144	0.000795
11731089_DDT /// D	-0.34717	0.000145	0.000796
11720355_INTS3	-0.35755	0.000145	0.000796
11759113_LOC100506	0.292754	0.000145	0.000796
11728310_CMTR2	0.328652	0.000145	0.000796
11736068_NDRG4	-0.2851	0.000145	0.000797
11749157_CFHR3 ///	0.580427	0.000145	0.000797
11721838_GLIPR1	0.284916	0.000145	0.000798
11755938_DTX2	-0.31424	0.000145	0.000798
11760509_HAGH	-0.25285	0.000145	0.000799
11732277_FOXP1	0.311353	0.000145	0.0008
11717602_SECISBP2	0.308337	0.000146	0.000801
11731164_ZNF789	0.374124	0.000146	0.000801
11718268_ANGPTL2	0.442346	0.000146	0.000801
11749252_STOM	0.30269	0.000146	0.000801
11741149_SLC3A2	0.267781	0.000146	0.000801
11721260_WDR47	0.28895	0.000146	0.000801
11757378_RMDN3	0.270089	0.000146	0.000802
11756957_DCTN2	-0.39128	0.000146	0.000802
11725016_MAP9	-0.31172	0.000146	0.000802
11721302_CLEC2B	0.283378	0.000146	0.000802
11722993_PLIN3	0.249065	0.000146	0.000803
11745290_PHTF1	0.29752	0.000146	0.000803
11722417_LARP6	0.480589	0.000146	0.000804
11744499_CHMP1A	-0.24928	0.000147	0.000805
11743881_MREG	0.281611	0.000147	0.000805
11733197_KIAA1586	0.379632	0.000147	0.000805
11756721_CASP8AP2	-0.30854	0.000147	0.000805
11743037_CRTAP	-0.33691	0.000147	0.000806
11736999_ZNF41	0.481337	0.000147	0.000806
11757818_CFL1	-0.26869	0.000147	0.000806
11754068_CSRP1	-0.2647	0.000147	0.000807
11762010_GRAMD1B	0.494853	0.000147	0.000807
11724434_TPK1	0.395967	0.000147	0.000807
11758736_PSMC2	-0.46267	0.000147	0.000809
11737022_GRIN2B	-0.265	0.000147	0.000809
11716996_TSPAN4	0.261923	0.000148	0.000809
11760430_GLA	-0.29673	0.000148	0.000809
11716418_SPTBN1	-0.27338	0.000148	0.000809
11741756_CPEB1	0.692268	0.000148	0.00081
11757300_ELOVL5	-0.26773	0.000148	0.00081
11751227_CCDC91	0.276359	0.000148	0.00081
11728776_VDR	-0.25069	0.000148	0.00081
11724053_FGFRL1	-0.26756	0.000148	0.000812
11718941_LSM14B	-0.58893	0.000148	0.000812
11757611_H2AFV	-0.27682	0.000148	0.000812
11751540_CYB561A3	-0.3091	0.000148	0.000813
11722056_ZC3H7B	0.371909	0.000148	0.000813
11748862_ARL6IP5	0.270316	0.000148	0.000813
11746727_FBXO38	0.262479	0.000148	0.000813

11715888_PIP4K2B	-0.2458	0.000149	0.000814
11733646_KYNU	0.389103	0.000149	0.000814
11728433_FAM19A1	0.540436	0.000149	0.000814
11750012_CSNK1D	0.254636	0.000149	0.000815
11744280_TIMM44	-0.32686	0.000149	0.000816
11743033_ANXA5	0.242597	0.000149	0.000816
11725797_USP46	0.402789	0.000149	0.000817
11742324_CASP8	0.476978	0.00015	0.000818
11731839_B4GALT6	-0.37361	0.00015	0.000818
11758264_HNRNPA0	-0.28715	0.00015	0.000819
11726158_SPIN4	-0.34724	0.00015	0.000819
11755144_PHF21B	0.478868	0.00015	0.00082
11743635_ZNRF2	0.486532	0.00015	0.00082
11751080_C7orf55-L	-0.2984	0.00015	0.00082
11742836_UBXN4	0.247732	0.00015	0.00082
11760527_LIG3	-0.30758	0.00015	0.00082
11716014_SERPINF1	-0.31866	0.00015	0.00082
11754681_HLA-DMB	-0.24854	0.00015	0.00082
11739500_APOPT1	-0.27028	0.00015	0.000821
11728734_ZBTB38	0.297473	0.00015	0.000822
11744116_RPIA	-0.3045	0.00015	0.000822
11752846_ARHGEF7	0.273412	0.00015	0.000822
11738936_KLRC3	1.279299	0.00015	0.000822
11753917_NEMF	0.382166	0.00015	0.000822
11723114_CENPC	0.264376	0.000151	0.000823
11716268_AK1	-0.25516	0.000151	0.000823
11723227_SGCB	0.289072	0.000151	0.000823
11725638_EIF3J	0.27759	0.000151	0.000824
11718412_GPKOW	-0.38693	0.000151	0.000824
11743379_ABI1	-0.2606	0.000151	0.000824
11739337_POLR1B	0.295449	0.000151	0.000824
11750020_SURF4	-0.29459	0.000151	0.000824
11753016_CEP250	-0.26065	0.000151	0.000825
11715559_YWHAQ	-0.31357	0.000151	0.000825
11718561_RCOR1	0.290617	0.000151	0.000826
11750258_ATXN2L	-0.39699	0.000151	0.000826
11755305_ZNF410	0.354441	0.000151	0.000826
11757390_KLHL24	-0.42372	0.000152	0.000826
11743675_ABL2	0.299335	0.000152	0.000826
11743106_DHX9	0.246595	0.000152	0.000827
11731541_SESTD1	-0.6461	0.000152	0.000827
11757896_RSRP1	0.393624	0.000152	0.000827
11741995_WBSCR22	0.257765	0.000152	0.000828
11743368_RPP14	0.33607	0.000152	0.000828
11738021_PLCB4	0.461192	0.000152	0.000828
11726600_ZMPSTE24	0.292048	0.000152	0.000828
11717188_DNAJA1	0.260991	0.000152	0.000828
11717814_ST3GAL4	-0.24162	0.000152	0.000828
11719214_LOC100996	0.450006	0.000152	0.000828
11726985_CCNC	0.300668	0.000152	0.000829
11718780_SSBP2	0.415275	0.000152	0.00083

11751699_USP3	0.335745	0.000152	0.00083
11743814_KLC1	0.339253	0.000152	0.00083
11716043_FAM168B	0.55424	0.000152	0.00083
11748449_ITGA5	0.439834	0.000153	0.00083
11745598_AGPAT2	-0.27464	0.000153	0.00083
11739462_TGFBR1	0.579798	0.000153	0.000831
11736547_FANCD2	-0.36334	0.000153	0.000831
11721664_TP53I13	-0.26367	0.000153	0.000831
11751937_KIF27	0.55335	0.000153	0.000831
11722973_FOXK1	-0.31375	0.000153	0.000831
11728065_SALL4	-0.2882	0.000153	0.000831
11723342_C11orf84	-0.30779	0.000153	0.000832
11727437_RALGAPB	0.275236	0.000153	0.000832
11719281_UBE2I	-0.27404	0.000153	0.000833
11718878_SMC1A	-0.71197	0.000153	0.000833
11730345_C6orf58	0.5908	0.000153	0.000833
11717705_VPS13D	-0.45644	0.000153	0.000833
11726125_HOXA5	0.266703	0.000153	0.000833
11722032_SLC12A4	0.301726	0.000154	0.000835
11727674_RFXAP	-0.30377	0.000154	0.000835
11730738_SCCPDH	-0.76509	0.000154	0.000836
11750401_UVRAG	0.372369	0.000154	0.000836
11725830_PLCL2	0.851545	0.000154	0.000836
11715774_IMP3	0.252723	0.000154	0.000837
11739172_G2E3	-0.28086	0.000154	0.000838
11749526_STK38L	0.326545	0.000154	0.000838
11725030_PNN	0.260212	0.000154	0.000838
11743754_ZFAND5	0.256207	0.000154	0.000838
11748347_ANKRD28	0.279047	0.000154	0.000838
11716352_TOMM34	-0.23893	0.000155	0.000839
11731521_ZNF592	-0.38792	0.000155	0.000842
11727721_BCAP29	-0.39423	0.000155	0.000842
11733995_NADK2	-0.29058	0.000155	0.000842
11725740_OGFOD3	-0.41462	0.000155	0.000843
11728208_ISY1	-0.33331	0.000156	0.000844
11730936_AKNA	-0.39863	0.000156	0.000844
11741680_SERPINE2	0.242202	0.000156	0.000844
11746374_CCDC25	0.269509	0.000156	0.000844
11729385_FOXF1	-0.48855	0.000156	0.000845
11722384_UBTF	-0.33447	0.000156	0.000845
11748818_TADA3	-0.2571	0.000156	0.000846
11730960_DGKG	0.284035	0.000156	0.000847
11750396_ZNF302	0.306996	0.000157	0.000848
11719299_CTDSP1	-0.31158	0.000157	0.000849
11758134_PPM1H	0.391167	0.000157	0.000849
11737321_RREB1	0.372326	0.000157	0.000849
11719768_RNF111	-0.46808	0.000157	0.00085
11742299_ING4	-0.29134	0.000157	0.00085
11746845_TMEM143	-0.24507	0.000157	0.00085
11721960_SNX30	0.239941	0.000157	0.000851
11722239_HIPK1	0.251188	0.000157	0.000851

11718054_LSM1	0.249693	0.000157	0.000852
11725083_HDAC6	-0.51015	0.000158	0.000853
11749137_FAM169A	0.339664	0.000158	0.000853
11727407_PLEKHA3	0.379163	0.000158	0.000853
11751951_TJP2	0.249388	0.000158	0.000853
11745853_RNF145	0.302439	0.000158	0.000854
11753891_TUBB	-0.25715	0.000158	0.000854
11720064_GLIPR2	-0.3145	0.000158	0.000854
11750789_EPS15	0.363476	0.000158	0.000855
11743416_C5orf30	-0.39088	0.000158	0.000855
11740674_TCERG1	0.264852	0.000158	0.000855
11750889_GLTSCR2	0.264084	0.000158	0.000855
11734778_PCTP	-0.28697	0.000158	0.000855
11744853_ZNF880	0.281392	0.000158	0.000856
11729864_MYBPC2	-0.26855	0.000158	0.000857
11734922_ZNF276	0.267538	0.000159	0.000857
11720951_DCUN1D4	-0.27028	0.000159	0.000858
11747385_STK25	-0.3061	0.000159	0.000858
11757904_MAST3	-0.37187	0.000159	0.000859
11736107_ENTPD4	0.511646	0.000159	0.000859
11725731_LUC7L	0.324931	0.000159	0.000859
11717684_RBM15B	-0.27766	0.000159	0.00086
11745279_SYNJ1	0.547972	0.000159	0.00086
11730787_KLHL20	0.324034	0.000159	0.000861
11721612_IPO11 ///	-0.32483	0.00016	0.000862
11752417_IMMT	-0.26014	0.00016	0.000862
11754716_GATAD2A	-0.29616	0.00016	0.000863
11723889_HELQ	0.443707	0.00016	0.000863
11746863_ARSJ	0.308924	0.00016	0.000863
11757557_CADM1	0.394684	0.00016	0.000864
11748273_RAF1	0.33856	0.00016	0.000865
11716436_RAD21	-0.34239	0.00016	0.000865
11721862_STX12	0.278764	0.00016	0.000866
11715587_MAPRE1	0.267662	0.000161	0.000867
11720370_RTCA	0.299961	0.000161	0.000867
11753207_MOB1B	0.581278	0.000161	0.000867
11720958_SAT2	0.421067	0.000161	0.000867
11737796_KIAA1524	-0.53344	0.000161	0.000868
11722027_UBE2G1	-0.34003	0.000161	0.000868
11736925_UBP1	0.274636	0.000161	0.000868
11748150_MDC1	-0.48222	0.000161	0.000868
11760241_SKP2	0.326613	0.000161	0.000869
11753156_GALNT2	0.441498	0.000161	0.000869
11716743_TJP2	0.25795	0.000161	0.000869
11743537_DNAJC18	0.292095	0.000162	0.000871
11724747_CHMP2B	0.316746	0.000162	0.000872
11758088_SOCS6	-0.31587	0.000162	0.000872
11727525_C12orf29	0.255223	0.000162	0.000872
11725354_EPM2AIP1	0.41693	0.000162	0.000872
11759374_HIST1H3G	-0.31675	0.000162	0.000873
11740154_ZNF384	0.243663	0.000162	0.000873

11741678_SERPINE2	0.273388	0.000162	0.000874
11763439_HNRNPU	-0.28366	0.000162	0.000875
11750865_CRISPLD1	-0.32092	0.000163	0.000875
11722927_CCDC137	-0.24095	0.000163	0.000875
11748112_MAPKAP1	-0.46785	0.000163	0.000875
11748473_ZNF28	0.484737	0.000163	0.000876
11747392_IMMT	-0.29738	0.000163	0.000876
11736424_STARD4	-0.31774	0.000163	0.000876
11745918_USPL1	0.292845	0.000163	0.000876
11740308_SLC5A12	0.244852	0.000163	0.000877
11744901_NDUFS1	-0.23418	0.000163	0.000877
11743775_MTRR	0.283576	0.000163	0.000877
11750630_MAP3K5	0.303963	0.000163	0.000878
11726327_DCUN1D1	0.299082	0.000163	0.000878
11759583_ERCC6L2	0.376545	0.000163	0.000878
11724418_UNKL	-0.28301	0.000163	0.000878
11749664_DOCK7	0.447437	0.000163	0.000878
11719193_CBY1	0.241808	0.000163	0.000879
11729473_FANCA	-0.39507	0.000164	0.000879
11744469_TBCD	-0.30232	0.000164	0.000879
11758327_BAZ1A	0.299645	0.000164	0.000881
11748946_UBTF	-0.35394	0.000164	0.000881
11754188_EIF2AK1	-0.34484	0.000164	0.000881
11740166_FMN1	-0.3916	0.000164	0.000881
11760085_HNRNPA1	0.277488	0.000164	0.000882
11735132_DNAJC21	0.289865	0.000164	0.000882
11756690_HN1L	-0.29413	0.000164	0.000883
11721964_TCAIM	0.319456	0.000164	0.000883
11717648_MCMBP	-0.29364	0.000164	0.000883
11727114_MARK2	0.280876	0.000165	0.000884
11746525_RBM48	0.308076	0.000165	0.000884
11754191_SRSF11	0.253047	0.000165	0.000884
11716988_PNRC2	0.233718	0.000165	0.000885
11723154_CAMTA1	0.308174	0.000165	0.000885
11717338_CUX1	0.30304	0.000165	0.000886
11727189_WNT5A	-0.37634	0.000165	0.000886
11739508_NAA30	0.2446	0.000165	0.000886
11729963_ANKEF1	0.698834	0.000165	0.000886
11742743_CNN3	0.244933	0.000165	0.000886
11722760_CAMSAP2	0.274471	0.000165	0.000886
11726744_TAOK2	0.280312	0.000165	0.000886
11741096_CORO2A	-0.47055	0.000165	0.000887
11744295_FAM195B	-0.24772	0.000165	0.000887
11748573_TTC8	0.401621	0.000165	0.000887
11718605_SCRG1	0.456934	0.000166	0.000887
11748152_IFNGR1	0.284053	0.000166	0.000887
11725367_IER5L	-0.29971	0.000166	0.000888
11745829_BTAF1	0.440008	0.000166	0.000888
11740309_ZNF460	0.525519	0.000166	0.000888
11745159_TGFA	0.537711	0.000166	0.00089
11746626_HAGH	-0.27854	0.000166	0.00089

11717940_U2AF2	-0.25478	0.000166	0.00089
11721273_NR2C1	-0.51134	0.000166	0.00089
11731684_RIF1	0.262908	0.000166	0.00089
11746618_RASSF8	-0.42675	0.000166	0.00089
11741129_CAPN2	0.237361	0.000166	0.000891
11743875_CEP72	-0.25934	0.000167	0.000891
11716658_HNRNPH3	-0.25888	0.000167	0.000891
11749889_SYAP1	0.247181	0.000167	0.000891
11748232_DIS3	0.306385	0.000167	0.000892
11739595_RBM12	-0.35341	0.000167	0.000893
11749118_SORBS1	0.56615	0.000167	0.000893
11720084_NUDT22	-0.37217	0.000167	0.000893
11743351_COBLL1	0.459383	0.000167	0.000894
11728248_TSFM	-0.26799	0.000167	0.000894
11754027_CYC1	-0.24179	0.000168	0.000896
11757490_TSPYL1	0.236682	0.000168	0.000897
11758589_ERCC4	0.302471	0.000168	0.000897
11759428_KLF7	0.301471	0.000168	0.000897
11720493_MOB3A	0.325968	0.000168	0.000898
11742825_GTF3C1	-0.27818	0.000168	0.000899
11715625_SH3BGRL	0.303625	0.000168	0.000899
11727016_PAPPA	0.57228	0.000168	0.000899
11721969_BDP1	0.324903	0.000168	0.000899
11727576_TMEM237	-0.32413	0.000168	0.000899
11745083_MANEA	-0.38128	0.000168	0.000899
11743268_PTP4A2	0.256528	0.000168	0.000899
11726333_LEF1	-0.32105	0.000168	0.0009
11727406_TEK	0.896317	0.000169	0.0009
11723129_ARHGEF7	0.257638	0.000169	0.000901
11720404_EFNB2	-0.24372	0.000169	0.000901
11759303_MFHAS1	0.301363	0.000169	0.000902
11745980_DIMT1	-0.25321	0.000169	0.000902
11762282_HLA-A	0.265806	0.000169	0.000903
11723032_PLP2	-0.29675	0.000169	0.000903
11740449_CFAP58	1.189997	0.000169	0.000904
11758267_ZBTB1	0.296547	0.00017	0.000905
11754689_TUSC3	0.267508	0.00017	0.000905
11750582_HDAC6	-0.70827	0.00017	0.000905
11725268_FAT4	0.702718	0.00017	0.000905
11743484_NAA25	0.291146	0.00017	0.000905
11720519_LOC100996	0.491178	0.00017	0.000906
11755851_UBR4	0.296809	0.00017	0.000906
11716809_CDC16	0.250574	0.00017	0.000907
11716474_PUM1	0.275763	0.00017	0.000907
11717700_DCAF8	-0.26981	0.00017	0.000908
11748884_SRSF11	0.242872	0.00017	0.000908
11735173_SNRPN ///	-0.27023	0.00017	0.000908
11718284_EIF4EBP1	-0.25948	0.000171	0.000908
11751728_TNRC6A	0.626146	0.000171	0.000908
11756266_CYFIP2	0.351368	0.000171	0.000908
11756148_PHLDB1	0.248559	0.000171	0.00091

11741627_ST7L	0.731025	0.000171	0.00091
11762368_KLHL8	0.323894	0.000171	0.00091
11746227_LOC652276	0.272876	0.000171	0.00091
11752692_NRBF2	0.26096	0.000171	0.000911
11746109_MPP2	-0.36088	0.000171	0.000911
11722487_UTP14C	-0.26592	0.000171	0.000911
11728713_LOC100996	0.274414	0.000171	0.000911
11723571_OTUD3	0.39643	0.000172	0.000913
11744060_UQCRC2	-0.25191	0.000172	0.000913
11749955_SEC61A1	0.267412	0.000172	0.000913
11715773_IMP3	0.314834	0.000172	0.000913
11754737_ZNF41	0.399262	0.000172	0.000913
11748188_NAGA	-0.35072	0.000172	0.000913
11728443_TAOK1	0.361118	0.000172	0.000913
11753184_KLF16	-0.2603	0.000172	0.000914
11722332_INO80	0.234108	0.000172	0.000915
11726523_ZFHX3	-0.26933	0.000172	0.000915
11719188_NRAS	0.326246	0.000172	0.000916
11756344_ARMC10	0.292241	0.000172	0.000916
11756428_SLC25A16	0.393815	0.000172	0.000916
11724781_HERC4	0.366074	0.000172	0.000916
11755222_GNA13	-0.33411	0.000173	0.000917
11746791_BECN1	0.254525	0.000173	0.000917
11729235_E2F6 ///	0.271904	0.000173	0.000918
11749988_AUH	0.311751	0.000173	0.000918
11727984_TENM3	0.313819	0.000173	0.000919
11760244_CARD8	-0.35048	0.000173	0.00092
11717713_PALM	-0.31799	0.000173	0.000921
11754314_TTC7B	-0.29904	0.000174	0.000923
11734108_LSM11	0.286894	0.000174	0.000924
11760414_SLC44A5	0.395604	0.000174	0.000924
11748301_TFDP2	-0.31821	0.000174	0.000925
11721419_POLI	-0.65127	0.000174	0.000925
11724141_RAB11FIP2	0.363682	0.000174	0.000925
11755810_TAPT1	0.284244	0.000174	0.000925
11722485_UTP14C	-0.32562	0.000174	0.000925
11721693_CYB561	0.244539	0.000174	0.000925
11716373_PTK2	0.287898	0.000175	0.000926
11718615_RABEP1	0.287718	0.000175	0.000926
11728659_HN1L	-0.30181	0.000175	0.000926
11729150_PIGM	-0.38885	0.000175	0.000926
11733071_SLC3A2	0.286907	0.000175	0.000927
11761979_ALG13	0.384109	0.000175	0.000927
11741414_RECQL4	-0.28106	0.000175	0.000928
11717327_KLF9	-0.27569	0.000175	0.000929
11725899_LRRC8D	-0.30018	0.000176	0.00093
11763181_RQCD1	-0.30566	0.000176	0.00093
11744304_MFSD3	-0.34644	0.000176	0.00093
11720290_IQCE	0.301033	0.000176	0.000931
11748285_SENP7	0.51929	0.000176	0.000931
11748974_CWF19L1	-0.40026	0.000176	0.000931

11723353_CHD7	0.418792	0.000176	0.000931
11734394_KLRC3	0.740915	0.000176	0.000931
11742684_LAMP2	-0.40837	0.000176	0.000932
11753481_SNAPC3	0.324937	0.000176	0.000933
11731995_SPANXA1 /	0.256822	0.000176	0.000933
11722751_AKAP8	0.353365	0.000176	0.000933
11727897_LSM8 ///	-0.28499	0.000176	0.000934
11723946_ACSL5	0.35888	0.000177	0.000935
11751219_GOLGA4	0.476532	0.000177	0.000935
11719339_SLC23A2	-0.55346	0.000177	0.000935
11758163_ZFP62	-0.32016	0.000177	0.000936
11744862_DHRS7	0.344945	0.000177	0.000936
11731362_ZNF624	0.452264	0.000177	0.000936
11725690_ZNF410	0.340211	0.000177	0.000936
11717470_EPT1	-0.26194	0.000177	0.000937
11744392_PAPOLA	-0.24618	0.000177	0.000937
11730524_PTPN13	0.336535	0.000177	0.000937
11716690_GOLGA3	0.248115	0.000177	0.000937
11728908_B3GLCT	-0.25751	0.000178	0.000938
11746416_APEX2	-0.29306	0.000178	0.000941
11763987_VEPH1	0.921763	0.000178	0.000941
11748984_VTA1	0.317758	0.000178	0.000941
11739472_MAGI1	0.425218	0.000178	0.000941
11757014_ARL15	0.44715	0.000178	0.000941
11727276_ZNF77	0.411406	0.000178	0.000942
11752552_MCCC1	0.354854	0.000178	0.000942
11730051_CA13	0.465841	0.000178	0.000942
11743922_NUDT11	-0.70808	0.000179	0.000943
11720333_SLC5A6	-0.23838	0.000179	0.000943
11736492_DAZAP2	-0.51829	0.000179	0.000943
11752069_VCPIP1	0.302978	0.000179	0.000943
11725330_USP9X	-0.52351	0.000179	0.000944
11725736_UBR3	0.500433	0.000179	0.000944
11746598_KHSRP	-0.2961	0.000179	0.000945
11726528_FAM81A	-0.22632	0.000179	0.000946
11752367_RABEP2	-0.3177	0.000179	0.000947
11757154_SNORA3B	-0.25586	0.00018	0.000947
11745527_HSF2	0.298229	0.00018	0.000948
11756208_SNX9	0.261951	0.00018	0.000948
11747777_RAB6A ///	0.35437	0.00018	0.000949
11747823_NUP85	-0.26365	0.00018	0.000949
11757448_ADD3	-0.25436	0.00018	0.000949
11734221_CREG2	-0.36251	0.00018	0.00095
11747514_GABARAPL2	0.250879	0.00018	0.000951
11718970_CD46	0.232585	0.000181	0.000951
11761869_PSIP1	-0.44641	0.000181	0.000952
11742060_CHORDC1	0.360085	0.000181	0.000952
11721115_SPCS3	0.236272	0.000181	0.000953
11746953_PPIL2	-0.42828	0.000181	0.000953
11715810_TRIP12	0.284075	0.000181	0.000953
11749419_KIF4A	-0.47833	0.000181	0.000954

11738624_ZNF268	0.281423	0.000181	0.000955
11744996_PDE1C	0.381162	0.000181	0.000955
11741572_RGS4	0.714784	0.000181	0.000955
11750037_CHMP7	-0.24811	0.000181	0.000955
11748166_EPHA2	0.386451	0.000181	0.000955
11752620_YPEL5	0.240936	0.000182	0.000956
11731154_MTRR	0.302568	0.000182	0.000957
11726214_WWOX	-0.40652	0.000182	0.000957
11754806_CADPS2	0.311369	0.000182	0.000957
11734373_CSPP1	0.260296	0.000182	0.000957
11749890_SYAP1	0.259523	0.000182	0.000957
11719528_PVRL2	0.31259	0.000182	0.000958
11718304_MYO18A //	-0.25102	0.000182	0.000958
11741064_RARG	-0.40741	0.000182	0.000958
11763888_SNORA33	-0.63471	0.000182	0.000958
11756581_PRSS23	0.275458	0.000182	0.000959
11734086_GALE	0.270529	0.000182	0.000959
11749103_ITGB8	0.429362	0.000182	0.000959
11721103_POLDIP3	-0.31141	0.000183	0.00096
11760128_LIMS1	0.283942	0.000183	0.00096
11760127_LIMS1	0.280054	0.000183	0.00096
11730778_HSD17B11	-0.2782	0.000183	0.000961
11756891_ZNF12	0.270643	0.000183	0.000961
11733746_GCNT2	0.254645	0.000183	0.000962
11721383_ATG2B	-0.44023	0.000183	0.000962
11724585_SYT1	0.545831	0.000183	0.000962
11726022_FAM177A1	0.292814	0.000183	0.000963
11734371_SCML1	0.395092	0.000183	0.000963
11725525_DSG2	0.31854	0.000183	0.000963
11744861_DHRS7	0.354999	0.000184	0.000964
11762573_BAGE	0.269489	0.000184	0.000964
11718129_ARL2	-0.25036	0.000184	0.000964
11723001_RANBP1	-0.26189	0.000184	0.000964
11724161_GXYLT1	0.557527	0.000184	0.000964
11717647_MCMBP	-0.27811	0.000184	0.000965
11759515_GTF3C3	0.289611	0.000184	0.000965
11722359_EPB41L2	0.28242	0.000184	0.000966
11759540_RPL35A	-0.30486	0.000184	0.000966
11755562_GPR39	0.3056	0.000184	0.000966
11740879_ANXA2	0.231558	0.000184	0.000966
11723344_TRIM35	0.316717	0.000184	0.000966
11726407_SRRD	0.303557	0.000184	0.000966
11743851_ZNF146	0.267653	0.000184	0.000967
11715911_MYO6	-0.79123	0.000185	0.000967
11724893_GATAD2B	-0.25539	0.000185	0.000967
11751096_SNX10	-0.48406	0.000185	0.000968
11752584_CEP104	0.284821	0.000185	0.000968
11755757_SYNM	-0.25971	0.000185	0.000969
11720751_INPP1	0.321868	0.000185	0.000969
11759765_PLSCR1	-0.82345	0.000185	0.000969
11745428_DFFB	-0.48519	0.000185	0.000969

11760515_LIAS	-0.40701	0.000185	0.00097
11740162_ARAP1	-0.2768	0.000185	0.00097
11751150_SNIP1	0.268305	0.000185	0.000971
11736225_SPATS2	0.252159	0.000186	0.000972
11755481_HELB	0.528917	0.000186	0.000972
11738474_SYAP1	0.283551	0.000186	0.000973
11728769_ST6GALNAC	0.370877	0.000186	0.000974
11752562_CDH13	0.236132	0.000186	0.000974
11722015_OLR1	0.46211	0.000186	0.000975
11749130_TAF6	-0.37667	0.000186	0.000975
11716915_MTUS1	0.357879	0.000186	0.000975
11753165_RAB27A	0.290987	0.000186	0.000975
11725120_GATAD1	0.300862	0.000187	0.000976
11726244_RORA	0.862936	0.000187	0.000976
11753645_CFL1	-0.25625	0.000187	0.000977
11755770_HEATR5B	-0.40075	0.000187	0.000977
11718699_C6orf89	-0.39497	0.000187	0.000977
11731122_VASH2	-0.55232	0.000187	0.000977
11722781_SLC6A19	-0.27881	0.000187	0.000977
11752650_RALGAPB	0.269111	0.000187	0.000977
11757331_RPL13A	-0.23938	0.000187	0.000977
11744854_ZNF28	0.348909	0.000187	0.000977
11721710_SHROOM3	0.354969	0.000187	0.000977
11728528_CGREF1	-0.31102	0.000187	0.000977
11719648_CASP7	-0.39824	0.000187	0.000977
11752916_LOC440434	0.257981	0.000187	0.000977
11725948_KRI1	0.245923	0.000187	0.000977
11759001_LDLRAD3	-0.29949	0.000187	0.000977
11731003_RAB28	-0.2854	0.000187	0.000978
11737897_ABCB7	0.265383	0.000187	0.000978
11721757_TMEM47	0.409404	0.000187	0.000978
11732630_ZNF765	0.451383	0.000187	0.000978
11729647_ESM1	0.312541	0.000188	0.000979
11715882_DAP3	0.270949	0.000188	0.00098
11756286_SFXN2	-0.2914	0.000188	0.00098
11743110_NAMPT	0.227416	0.000188	0.00098
11720592_HDAC8	-0.30513	0.000188	0.00098
11740364_PTPN3	0.261459	0.000188	0.00098
11750129_KLC1	0.394069	0.000188	0.00098
11724567_USP48	-0.2506	0.000188	0.000981
11720681_TIFA	-0.837	0.000188	0.000982
11720133_PROS1	0.327765	0.000188	0.000982
11728410_INO80C	-0.27958	0.000188	0.000983
11737753_SNX5	-0.25714	0.000188	0.000983
11718866_FAM192A	-0.43146	0.000189	0.000983
11725329_USP9X	0.229532	0.000189	0.000983
11717584_VAPB	-0.24795	0.000189	0.000983
11745335_MTMR1	0.381553	0.000189	0.000983
11744444_NDUFB10	-0.28516	0.000189	0.000983
11744488_ACOT9	0.245791	0.000189	0.000983
11722490_HS1BP3	-0.28361	0.000189	0.000983

11750861_CMAHP	0.620862	0.000189	0.000983
11719593_RPRD2	-0.41867	0.000189	0.000984
11731611_PCID2	0.265491	0.000189	0.000984
11758420_TRIM43 //	-0.35914	0.000189	0.000984
200023_PMEIF3F	0.231237	0.000189	0.000985
11754915_CCDC115	-0.2308	0.000189	0.000985
11743415_NDRG4	-0.27427	0.000189	0.000985
11751641_PHF11	0.345898	0.000189	0.000985
11757499_WBP1L	0.301505	0.000189	0.000985
11725332_CTSL	0.247639	0.000189	0.000986
11758217_ABHD17C	-0.29086	0.00019	0.000986
11731204_TOR1AIP2	0.443523	0.00019	0.000986
11725187_KLF12	0.363087	0.00019	0.000986
11751826_FMR1	0.444856	0.00019	0.000986
11737991_CEP170	0.271603	0.00019	0.000989
11753289_LOC100506	0.725355	0.00019	0.000989
11759575_UQCRH	0.253957	0.00019	0.00099
11716172_SYVN1	0.233696	0.00019	0.00099
11735202_N4BP2	0.287101	0.000191	0.00099
11755048_MED22	-0.29427	0.000191	0.00099
11747594_SH3BGRL	0.272117	0.000191	0.00099
11733221_DLL3	-0.26585	0.000191	0.00099
11739387_CDC73	0.280735	0.000191	0.000991
11757977_SLC12A7	-0.33675	0.000191	0.000991
11718053_TBC1D23	0.298827	0.000191	0.000991
11732148_CAT	-0.25746	0.000191	0.000991
11739431_FCF1	-0.29219	0.000191	0.000993
11753942_UCA1	0.400261	0.000191	0.000993
11721606_ADD3	-0.30353	0.000191	0.000993
11744840_KPNB1	-0.2617	0.000191	0.000994
11754720_C16orf86	-0.33347	0.000192	0.000995
11754295_FAM107B	-0.30421	0.000192	0.000995
11746646_DDX59	-0.24561	0.000192	0.000995
11720281_EVA1C	-0.39514	0.000192	0.000995
11756942_ING5	0.508931	0.000192	0.000995
11743004_EXOSC9	-0.27481	0.000192	0.000996
11725005_C8orf58	0.446775	0.000192	0.000996
11744054_ZNFX1	-0.27254	0.000192	0.000996
11746464_YAF2	0.363539	0.000192	0.000996
11733129_ZSCAN26	0.453893	0.000192	0.000998
11726308_FAM204A	0.265573	0.000192	0.000998
11754322_KCTD10	-0.45536	0.000193	0.000998
11724802_NSRP1	0.269021	0.000193	0.000999
11747585_C8orf34	0.538995	0.000193	0.001
11755417_KIAA0922	0.381472	0.000193	0.001
11750467_UBE3B	-0.24469	0.000193	0.001
11721284_CDS2	0.363425	0.000193	0.001001
11749815_SPIN1	0.28155	0.000193	0.001001
11737454_ZNF763	0.492079	0.000193	0.001001
11750860_CMAHP	0.696285	0.000193	0.001001
11744223_ATP5D	-0.31785	0.000193	0.001001

11754132_COMT	0.271756	0.000193	0.001002
11749153_KLHL18	0.432447	0.000193	0.001002
11716962_PPM1F	0.386168	0.000194	0.001003
11719939_CAND1	-0.25477	0.000194	0.001004
11718936_MMD	-0.30657	0.000194	0.001004
11750588_CTNND1 //	0.397255	0.000194	0.001005
11749146_TAF6	-0.27822	0.000194	0.001005
11745137_ZNF331	-0.37584	0.000194	0.001006
11748180_SERPINB8	0.362634	0.000194	0.001006
11754778_MRPL55	-0.25286	0.000195	0.001008
11728312_ZNF148	-0.41791	0.000195	0.001008
11761138_PRKAG1	-0.30359	0.000195	0.001009
11717736_ZCCHC24	-0.32921	0.000195	0.00101
11727215_NRG1	0.385449	0.000195	0.00101
11758560_HERC4	0.37496	0.000196	0.001011
11739925_ZNF37A	0.376858	0.000196	0.001011
11762163_RNF5	-0.31338	0.000196	0.001012
11745718_GTF2I ///	-0.24144	0.000196	0.001013
11746737_TMBIM1	-0.31192	0.000196	0.001014
11750216_SLC39A1	-0.35682	0.000196	0.001014
11724797_MAGOH	-0.43483	0.000196	0.001014
11718606_DESI1	0.283026	0.000196	0.001014
11755375_DGKH	0.690337	0.000196	0.001014
11753247_MTERF4	0.468276	0.000196	0.001015
11758624_ERBB2IP	0.275778	0.000196	0.001015
11727333_RSPO4	-0.23413	0.000197	0.001015
11757853_TWIST2	-0.30878	0.000197	0.001017
11756693_MAGED1	-0.31243	0.000197	0.001017
11719788_TUBB2A	0.267393	0.000197	0.001017
11747979_MAP4	-0.23385	0.000197	0.001017
11719008_PAPOLA	-0.24408	0.000197	0.001018
11731774_GABPB2	0.39898	0.000197	0.001018
11717835_GLG1	-0.22647	0.000197	0.001019
11728650_NUCKS1	-0.33576	0.000197	0.001019
11763133_RAB6A	0.24529	0.000197	0.001019
11719568_KIAA2013	-0.26149	0.000197	0.001019
11755357_ACO1	0.294245	0.000198	0.00102
11731760_RAD52	0.41447	0.000198	0.001021
11718117_NFS1	-0.28773	0.000198	0.001023
11754619_TLK2	-0.3171	0.000198	0.001023
11720263_MITF	0.38263	0.000198	0.001024
11721869_HIF1AN	-0.35414	0.000199	0.001024
11724379_NHSL1	-0.30227	0.000199	0.001024
11716974_PDK4	0.385139	0.000199	0.001024
11716881_ZZEF1	0.244784	0.000199	0.001024
11715957_PEPD	-0.28509	0.000199	0.001025
11731896_TNFSF4	1.150518	0.000199	0.001025
11727704_ZNF439	0.464069	0.000199	0.001025
11747996_ETV4	-0.33488	0.000199	0.001027
11745449_ZNF121	0.350913	0.000199	0.001027
11732858_ADGRA2	-0.38856	0.000199	0.001027

11717498_SLC25A36	0.244739	0.000199	0.001027
11748709_GLI2	0.246715	0.000199	0.001027
11730679_ASB7	0.288274	0.000199	0.001027
11722823_SKP1	-0.28587	0.0002	0.001028
11720740_MCF2L	0.355299	0.0002	0.001028
11750993_MAP3K7	0.274013	0.0002	0.001028
11723854_SAMD9	0.254739	0.0002	0.001029
11755876_IFRD2	-0.31568	0.0002	0.001029
11756575_CABIN1	-0.28987	0.0002	0.00103
11736339_PCSK1	0.431691	0.0002	0.00103
11724769_FCGR2A //	0.290404	0.0002	0.001031
11736045_MYH10	-0.2668	0.0002	0.001031
11739413_MTR	-0.25257	0.0002	0.001031
11728670_UBE2H	0.267432	0.0002	0.001031
11757797_EPM2A	-0.24315	0.0002	0.001031
11716145_PDCD6IP	0.22963	0.0002	0.001031
11731795_EPHX4	0.30028	0.000201	0.001032
11758622_MXRA7	0.244701	0.000201	0.001032
11732450_AGRN	0.240569	0.000201	0.001032
11759304_UGT8	-0.26702	0.000201	0.001033
11741824_PPA2	-0.30898	0.000201	0.001033
11717939_U2AF2	-0.34678	0.000201	0.001034
11745919_RANBP6	0.317438	0.000201	0.001035
11746636_LEF1	-0.54705	0.000201	0.001035
11762798_PALM	-0.33855	0.000201	0.001035
11721188_ARHGEF9	-1.00319	0.000202	0.001035
11723450_FTO	-0.29499	0.000202	0.001035
11757946_CPNE3	-0.2701	0.000202	0.001035
11749886_BCL2L13	0.492212	0.000202	0.001037
11760107_PHF10	0.479615	0.000202	0.001038
11721795_SMO	-0.24286	0.000202	0.001038
11755464_TBC1D31	-0.34449	0.000202	0.001039
11748252_CEP57	-0.23923	0.000202	0.001039
11747459_GMPPA	-0.23382	0.000202	0.001039
11725785_BORA	-0.51834	0.000202	0.001039
11759668_CEP57	-0.39588	0.000202	0.001039
11727991_STX11	0.277829	0.000203	0.001039
11718554_MCOLN1	-0.30199	0.000203	0.001041
11759574_NCOR1	0.319454	0.000203	0.001041
11726089_VPS53	0.245729	0.000203	0.001042
11717527_RDX	0.286185	0.000203	0.001042
11721437_STC2	0.442924	0.000203	0.001043
11733941_TBCEL	0.641458	0.000203	0.001043
11715993_SPG7	0.272635	0.000203	0.001043
11727244_CCDC66	0.474513	0.000204	0.001044
11739771_OXR1	0.314492	0.000204	0.001044
11751003_TUFT1	0.325878	0.000204	0.001044
11740135_USP40	0.34038	0.000204	0.001044
11731510_DBT	0.281828	0.000204	0.001044
11755028_DENND1A	0.269153	0.000204	0.001045
11725932_HSPA5	-0.22844	0.000204	0.001045

11720562_PLXNA2	0.42741	0.000204	0.001045
11734245_ICAM5	-0.4702	0.000204	0.001046
11741982_CRACR2A	-0.39127	0.000204	0.001046
11724071_PHF10	-0.2981	0.000204	0.001046
11729233_E2F6	0.276707	0.000204	0.001046
11743903_CD46	0.28297	0.000204	0.001047
11717431_SNRPD3	-0.30004	0.000204	0.001047
11720864_SLC25A24	0.359507	0.000205	0.001048
11754892_P3H4	-0.35496	0.000205	0.001048
11735630_CLP1	0.263923	0.000205	0.001049
11722953_BIK	0.308879	0.000205	0.001049
11754777_SH2D2A	0.508509	0.000205	0.001049
11730849_ABHD13	0.350356	0.000205	0.001049
11750683_NEK4	0.242847	0.000205	0.001049
11751211_NCAM2	-0.52081	0.000205	0.001049
11730254_ELK3	0.298763	0.000205	0.00105
11738988_GANAB	-0.28701	0.000205	0.00105
11717731_SQLE	-0.36947	0.000205	0.00105
11722382_TXNL4B	0.278817	0.000206	0.001051
11750792_SLC6A9	-0.37604	0.000206	0.001052
11732831_RNF216P1	0.299435	0.000206	0.001052
11750541_SSFA2	0.323526	0.000206	0.001052
11731146_RGPD1 ///	0.520831	0.000206	0.001052
11758057_GATA3	0.417042	0.000206	0.001053
11730218_CERS5	0.29744	0.000206	0.001053
11759821_RPL37A	-0.3818	0.000206	0.001054
11738141_ZNF621	0.389211	0.000206	0.001054
11721772_INO80D	0.31572	0.000206	0.001055
11751289_NR3C1	-0.28705	0.000207	0.001056
11715809_PYGB	-0.28023	0.000207	0.001056
11754190_SRSF11	0.274283	0.000207	0.001058
11720886_ZNF629	-0.54166	0.000207	0.001058
11753886_IL6ST	0.296602	0.000207	0.00106
11732689_TTC14	0.290643	0.000208	0.00106
11742982_DOPEY1	0.440652	0.000208	0.00106
11741710_COL6A3	0.465053	0.000208	0.00106
11730989_KIAA1919	0.588568	0.000208	0.001061
11742950_CNOT8	-0.38971	0.000208	0.001061
11736989_PRPF40B	-0.25082	0.000208	0.001061
11719607_RRN3	0.280969	0.000208	0.001062
11747380_FBXL4	0.312853	0.000208	0.001063
11741875_AKTIP	-0.30351	0.000208	0.001063
11744296_DEDD2	0.278308	0.000208	0.001063
11720717_FGF13	0.238382	0.000208	0.001063
11730355_ATRIP	-0.28252	0.000208	0.001063
11759328_CASC5	-0.32729	0.000209	0.001063
11755877_APLP1	-0.32914	0.000209	0.001065
11736602_BRIP1	-0.55851	0.000209	0.001065
11721404_NCOA3	0.489368	0.000209	0.001065
11729441_KDM4D	-0.77965	0.000209	0.001065
11761350_PPIH	-0.27398	0.000209	0.001066

11754946_C7orf49	-0.33884	0.00021	0.001069
11717285_WASL	0.384132	0.00021	0.001069
11722772_ZNF473	0.250163	0.00021	0.001069
11733496_COMMD6	0.224064	0.00021	0.001069
11715239_IFITM3	0.233336	0.00021	0.00107
11748577_IFT20	0.307586	0.00021	0.00107
11733247_SPG21	-0.24306	0.00021	0.00107
11754192_SRSF11	0.264458	0.00021	0.00107
11748280_SERPINB1	0.293037	0.00021	0.00107
11758070_PBX1	-0.30407	0.00021	0.00107
11728498_SVIL	0.326466	0.00021	0.00107
11719997_TLE4	-0.26645	0.00021	0.00107
11753352_SLC47A1	-0.29403	0.00021	0.001071
11731514_XPO4	-0.51879	0.000211	0.001073
11718560_RCOR1	-0.23842	0.000211	0.001074
11733146_ZBTB14	-0.35334	0.000211	0.001074
11721084_TRIM37	0.321066	0.000211	0.001074
11759666_HLA-DPB1	-0.44419	0.000211	0.001075
11755544_EFHB	0.346667	0.000211	0.001075
11750233_ATF2	0.35344	0.000211	0.001075
11760750_PRKD3	-0.49075	0.000211	0.001075
11729180_DHRSX	0.28552	0.000211	0.001075
11732361_CTPS2	-0.76787	0.000212	0.001076
11721377_BCL2	-0.56337	0.000212	0.001079
11733189_INCENP	-0.61946	0.000212	0.001079
11729103_RARS	0.254859	0.000212	0.00108
11754134_PDLIM3	-0.4203	0.000213	0.001081
11726085_FAM69B	-0.25508	0.000213	0.001082
11743747_PHKA1	-0.48645	0.000213	0.001082
11756571_PSEN1	0.373601	0.000213	0.001082
11741462_PRKAG1	-0.28012	0.000213	0.001083
11758115_PTBP2	0.536148	0.000213	0.001083
11724586_SYT1	0.513412	0.000213	0.001083
11733442_SAA1	0.588526	0.000213	0.001083
11747961_PSMB8	-0.30302	0.000213	0.001084
11718529_PCIF1	-0.24987	0.000214	0.001085
11753506_CEACAM1	0.455057	0.000214	0.001085
11746342_CFLAR	0.406441	0.000214	0.001085
11744787_ANLN	-0.27043	0.000214	0.001085
11722517_SMC5	0.272549	0.000214	0.001086
11756077_NDE1	-0.2795	0.000214	0.001087
11727154_LRRC1	0.593442	0.000214	0.001088
11724324_SLC12A6	0.399537	0.000214	0.001088
11754318_SSH1	-0.24342	0.000214	0.001088
11752883_UPF3A	0.251908	0.000215	0.001088
11721991_SLC6A6	0.299912	0.000215	0.001088
11753068_CFI	0.760301	0.000215	0.001088
11751215_SLAIN1	-0.29184	0.000215	0.001089
11718332_TSPAN3	0.243757	0.000215	0.001089
11725634_MRPS25	0.249318	0.000215	0.001089
11723255_HAT1	-0.36971	0.000215	0.00109

11741967_CDYL	0.363782	0.000215	0.00109
11728342_DCLK1	0.730857	0.000215	0.00109
11718670_MED9	-0.39736	0.000215	0.00109
11754158_CFL1	-0.26232	0.000215	0.001091
11726869_MFSD12	0.222394	0.000215	0.001091
11756800_CELSR1	-0.31864	0.000215	0.001091
11752436_MAP9	0.234861	0.000215	0.001091
11725117_NLN	0.24923	0.000215	0.001091
11724247_TMEM127	-0.44087	0.000216	0.001092
11752456_ABHD17C	-0.27715	0.000216	0.001092
11729427_GLI1	-0.25179	0.000216	0.001092
11722489_UTP14C	-0.29527	0.000216	0.001092
11757498_TMEM106C	-0.24984	0.000216	0.001092
11730899_WAPL	-0.25465	0.000216	0.001093
11722204_FBXO22	0.245871	0.000216	0.001094
11733035_C7orf73	-0.45951	0.000216	0.001095
11757494_HSD17B11	-0.26104	0.000216	0.001095
11736247_AMIGO2	0.236251	0.000217	0.001097
11763463_ATF3	0.382176	0.000217	0.001097
11723418_VSNL1	-0.29459	0.000217	0.001098
11717887_PTPN12	0.22412	0.000217	0.001098
11729993_STOX2	-0.33376	0.000217	0.0011
11722829_SDAD1	0.252038	0.000217	0.0011
11754940_TSN	-0.26457	0.000218	0.0011
11718461_SLC39A11	0.326418	0.000218	0.0011
11744175_EIF2B5	0.297556	0.000218	0.001101
11717922_YAP1	-0.34	0.000218	0.001102
11754341_ELAC2	-0.25322	0.000218	0.001102
11746869_EVI5	0.326125	0.000218	0.001103
11736518_WHSC1	-0.40877	0.000218	0.001103
11719467_PRPF31	-0.30527	0.000218	0.001103
11759119_TBCEL	0.262198	0.000218	0.001104
11727819_DDX46	-0.32809	0.000219	0.001105
11733042_SLC7A2	-0.50062	0.000219	0.001105
11736639_CNKSR2	0.332997	0.000219	0.001105
11723235_IFI44L	0.521986	0.000219	0.001106
11758402_FAM179B	0.418914	0.000219	0.001106
11745375_ZNF43	0.367892	0.000219	0.001106
11729875_FAM209A	0.580452	0.000219	0.001106
11729421_TTC26	0.395267	0.000219	0.001106
11727115_MARK2	0.296268	0.000219	0.001106
11727213_DTWD1	-0.23414	0.000219	0.001106
200063_PMNPM1	-0.22767	0.000219	0.001107
11730198_TECR	-0.245	0.000219	0.001107
11720229_STAT5B	0.451888	0.000219	0.001107
11719056_PDE8A	0.254999	0.000219	0.001107
11723769_SNX24	-0.29276	0.000219	0.001108
11730440_CERS6	0.597747	0.00022	0.001108
11763339_SIVA1	-0.3065	0.00022	0.001108
11715757_RGS2	0.262665	0.00022	0.001108
11727059_MRPS36	-0.46156	0.00022	0.001108

11739325_PTPN2	0.290384	0.00022	0.001108
11743499_PSMG4	-0.30856	0.00022	0.001109
11752005_RGPD4	0.315669	0.00022	0.001109
11757932_LOC100996	0.226423	0.00022	0.001109
11726159_IQCG	0.469956	0.00022	0.00111
11721663_HDAC11	-0.58928	0.00022	0.00111
11717147_PTPN1	0.312828	0.00022	0.00111
11751074_ARHGDIA	-0.22448	0.00022	0.00111
11721805_KLF2	0.363629	0.000221	0.001111
11745358_SAMD15	0.358488	0.000221	0.001111
11760162_WSB1	-0.40737	0.000221	0.001112
11728074_PLEKHM3	0.251994	0.000221	0.001112
11719379_RAP1GDS1	-0.38088	0.000221	0.001113
11758075_SLC18B1	0.454407	0.000221	0.001113
11719183_DEF8	-0.24128	0.000221	0.001113
11763704_SAT1	0.231682	0.000221	0.001113
11751000_UBXN4	0.379052	0.000221	0.001113
11740743_TPM2	0.268247	0.000221	0.001113
11759193_TXLNG	0.264237	0.000221	0.001114
11738204_SPAM1	-0.2968	0.000222	0.001115
11728566_FAM210A	-0.42269	0.000222	0.001116
11751027_TMC7	0.592473	0.000222	0.001116
11750353_MR1	0.385934	0.000222	0.001116
11721847_REEP2	-0.34197	0.000222	0.001116
11763357_TMEM170A	0.331936	0.000222	0.001117
AFFX-HUMISTAT1	0.266726	0.000222	0.001117
11716240_RAB6A	0.271412	0.000222	0.001118
11747394_ORC3	-0.24535	0.000222	0.001118
11746913_SLC9B2	0.288523	0.000223	0.00112
11730719_AP1S2	0.278545	0.000223	0.00112
11723574_MORC4	0.342367	0.000223	0.001121
11753435_SLC3A2	0.287165	0.000223	0.001121
11722113_ZNF232	-0.24568	0.000223	0.001122
11757727_ATP5O	-0.27944	0.000223	0.001123
11721955_RFX7	0.26077	0.000224	0.001125
11752336_MFSD11	0.428047	0.000224	0.001126
11726568_EIF4G1	-0.23727	0.000224	0.001127
11728712_ZFP64	-0.26918	0.000224	0.001128
11723807_STX17	0.310367	0.000225	0.001129
11739882_GLIS3	0.286563	0.000225	0.001129
11755360_SLC38A10	-0.22791	0.000225	0.001129
11739293_IGSF3	-0.32814	0.000225	0.001129
11755915_HPS3	-0.33929	0.000225	0.001129
11753537_CCDC102B	-0.35178	0.000225	0.00113
11742996_MCM3	-0.32394	0.000225	0.00113
11720085_KIAA0368	-0.75879	0.000225	0.00113
11758658_EPM2AIP1	0.284146	0.000225	0.001131
11744490_C20orf24	-0.23023	0.000225	0.001131
11723002_RANBP1	-0.35229	0.000225	0.001131
11763209_DOCK7	0.404842	0.000226	0.001132
11733645_KYNU	0.678303	0.000226	0.001132

11737820_MAP3K3	-0.49256	0.000226	0.001133
11747643_BUB3	-0.25331	0.000226	0.001133
11743509_UFL1	-0.46804	0.000226	0.001133
11731404_ATP6V1G1	-0.28271	0.000226	0.001133
11737103_GDAP2	0.299369	0.000226	0.001134
11719720_LSM7	-0.25448	0.000226	0.001135
11746191_KIAA2013	-0.27167	0.000227	0.001136
11715666_TPM3	-0.34395	0.000227	0.001136
11738887_MICA	0.277702	0.000227	0.001136
11742856_ASNSD1	0.282055	0.000227	0.001136
11745903_SLAMF7	0.62504	0.000227	0.001136
11751101_OTUD6B	0.349363	0.000227	0.001137
11757571_UBLCP1	0.290737	0.000227	0.001138
11752737_CLEC7A	0.733175	0.000227	0.001138
11743810_TFAM	0.365751	0.000227	0.001138
11732381_USP6NL	0.289746	0.000227	0.001139
11727994_STX11	0.667983	0.000227	0.001139
11755670_SYT14	0.72015	0.000228	0.00114
11729895_CHRAC1	-0.27167	0.000228	0.001142
11741949_NF1	-0.30267	0.000228	0.001143
11742884_LTBP3	0.360285	0.000228	0.001144
11730893_UBA6	0.276691	0.000228	0.001144
11749053_ZNF552	0.578772	0.000229	0.001144
11754785_ZFYVE19	-0.26141	0.000229	0.001145
11731382_BEST3	0.614571	0.000229	0.001145
11755261_MPV17	0.247778	0.000229	0.001145
11757795_RCOR3	0.350662	0.000229	0.001146
11715819_LRPAP1	0.279301	0.000229	0.001147
11718621_DUSP12	0.2422	0.000229	0.001147
11737847_MR1	0.50054	0.00023	0.001149
11748075_NFE2L1	-0.28211	0.00023	0.001149
11721850_CHCHD6	0.24305	0.00023	0.001149
11736539_RAB29	0.302648	0.00023	0.00115
11730937_PYCR1	-0.34669	0.00023	0.00115
11758806_CGRRF1	0.421856	0.00023	0.00115
11749709_ABLIM1	0.302849	0.00023	0.00115
11726258_RNF141	-0.34973	0.00023	0.00115
11755479_ZNF528	0.417837	0.00023	0.00115
11759737_BIRC5	-0.35106	0.00023	0.00115
11734206_ZNF155	0.453106	0.00023	0.00115
11752012_SREBF2	-0.39591	0.00023	0.001151
11726949_RBM15	0.281464	0.000231	0.001152
11739557_VAMP5	0.288274	0.000231	0.001152
11745210_PCDH9	-0.2618	0.000231	0.001152
11754319_SSH1	-0.25047	0.000231	0.001152
11725759_C6orf203	0.322279	0.000231	0.001152
11754049_CCDC91	0.256058	0.000231	0.001152
11724681_ATG16L1	0.342665	0.000231	0.001152
11732957_RNPEPL1	-0.25435	0.000231	0.001152
11719414_PBX1	-0.28899	0.000231	0.001152
11753910_TMEM242	-0.41795	0.000231	0.001152

11728750_OSBP	0.373151	0.000231	0.001153
11756312_SERPINF1	-0.28926	0.000231	0.001153
11747075_TMEM165	0.241735	0.000231	0.001153
11723710_COMMD3	0.228257	0.000231	0.001153
11751526_WDR1	0.25798	0.000231	0.001153
11726147_C17orf96	-0.46597	0.000231	0.001153
11718231_HLA-F	0.283094	0.000231	0.001153
11747748_ETV1	0.296008	0.000231	0.001153
11729982_PARP11	0.367587	0.000231	0.001154
11746203_ASB13	-0.49644	0.000231	0.001154
11728700_BMPR2	0.287462	0.000231	0.001154
11746746_AKAP9	0.244499	0.000232	0.001155
11761775_PLGLB1 //	0.575773	0.000232	0.001155
11722894_PIK3C2A	-0.30007	0.000232	0.001156
11752146_SOX5	-0.22955	0.000232	0.001156
11719450_RAB14	0.281888	0.000232	0.001156
11750969_SQLE	-0.3535	0.000232	0.001157
11715652_PCOLCE	-0.237	0.000232	0.001157
11718032_ATP6V0A1	0.227532	0.000233	0.00116
11721480_GOLPH3L	-0.26617	0.000233	0.001162
11746053_LOC100996	0.343495	0.000233	0.001163
11716281_COL18A1	0.298433	0.000234	0.001164
11736907_KLF17	0.391839	0.000234	0.001164
11718547_ILF3	-0.22819	0.000234	0.001166
11743996_ACTR1A	-0.32423	0.000234	0.001166
11754241_ATAD1	0.229109	0.000234	0.001167
11744987_WBSCR22	0.291559	0.000234	0.001167
11758452_CENPQ	-0.35463	0.000235	0.001167
11722885_WDR24	-0.37863	0.000235	0.001167
11751733_RNMT	0.344217	0.000235	0.001168
11748832_RAD52	0.32995	0.000235	0.001168
11719317_PIBF1	0.377708	0.000235	0.001168
11751656_TMEM154	-0.32849	0.000235	0.001168
11719969_NVL	0.284114	0.000235	0.00117
11748813_TRIP12	0.296662	0.000235	0.00117
11719982_INPP5A	-0.31374	0.000235	0.001171
11740260_CDC42BPB	-0.26384	0.000236	0.001172
11750041_ANXA5	0.232022	0.000236	0.001172
11758475_HMGXB4	-0.23122	0.000236	0.001172
11721213_CLIP2	-0.44737	0.000236	0.001172
11717617_PXN	0.243685	0.000236	0.001172
11722097_ZNF131	0.303788	0.000236	0.001173
11751374_PDYN	-0.2982	0.000236	0.001173
11758073_ZC3H15	0.252018	0.000236	0.001173
11738621_XKR5	-0.2402	0.000236	0.001174
11734826_SPRED1	0.308461	0.000237	0.001176
11725659_MTFR1	-0.25716	0.000237	0.001176
11728617_CARD6	0.342214	0.000237	0.001176
11718355_ERP44	0.261737	0.000237	0.001176
11719569_QARS	-0.25063	0.000237	0.001178
11739106_TMEM33	0.255323	0.000237	0.001178

11739709_PHACTR2	0.417693	0.000237	0.001178
11727608_OGG1	-0.22385	0.000237	0.001179
11756246_TTC28	-0.33427	0.000237	0.001179
11720099_CEP70	-0.27815	0.000238	0.001179
11750608_PELP1	-0.28331	0.000238	0.001182
11754439_NELFCD	-0.25308	0.000238	0.001182
11757026_SERPINF1	-0.29391	0.000238	0.001183
11739471_MAGI1	0.26815	0.000238	0.001183
11744349_DDR2	-0.25485	0.000239	0.001184
11720544_AGPAT2	-0.29245	0.000239	0.001184
11748541_PRADC1	-0.35603	0.000239	0.001184
11745875_VCP	-0.30967	0.000239	0.001184
11715949_KCTD12	-0.70249	0.000239	0.001185
11747179_ARL1	-0.4865	0.000239	0.001185
11740002_CTBP2	-0.42281	0.000239	0.001185
11725762_PELI3	-0.34191	0.000239	0.001186
11729990_CACNA1A	-0.30873	0.000239	0.001186
11730982_RNF25	0.329809	0.000239	0.001186
11718192_VEZF1	-0.26369	0.000239	0.001187
11751095_CEACAM1	0.328539	0.000239	0.001187
11746531_TYW5	0.34895	0.00024	0.001187
11756536_C5orf22	0.239444	0.00024	0.001187
11740880_ANXA2	0.221736	0.00024	0.001188
11756858_MKNK1	0.264616	0.00024	0.001188
11722418_UTP3	0.267192	0.00024	0.001188
11753028_NDUFA10	-0.4582	0.00024	0.001188
11763988_CLPX	0.255509	0.00024	0.001189
11754087_CTNND1 //	0.472613	0.00024	0.001189
11723045_FRG1 ///	-0.27283	0.00024	0.001189
11724314_LUZP1	0.282174	0.00024	0.001189
11728810_ECHDC3	-0.32188	0.00024	0.001189
11748247_ZNF235	0.469452	0.00024	0.00119
11721950_EFR3B	-0.2337	0.000241	0.001191
11754868_ERGIC3	0.263489	0.000241	0.001192
11749866_GALK1	-0.24644	0.000241	0.001192
11715878_PITPNA	-0.33975	0.000241	0.001193
11735155_ZNF585A	0.435332	0.000241	0.001193
11731585_OMA1	-0.25854	0.000241	0.001193
11739269_LOC102723	0.374039	0.000241	0.001193
11717996_NUP205	-0.25777	0.000241	0.001193
11735696_ENTPD6	0.247217	0.000241	0.001194
11730724_EIF3C ///	-0.29669	0.000242	0.001194
11723438_AK3	0.239455	0.000242	0.001194
11727191_BFSP1	0.624783	0.000242	0.001196
11745533_PPP5C	-0.34374	0.000242	0.001198
11722419_UTP3	0.236339	0.000242	0.001199
11733311_PCYT1B	-0.38786	0.000243	0.0012
11737812_NNT	-0.29668	0.000243	0.0012
11729983_PARP11	0.47935	0.000243	0.001201
11715904_DDX3X	0.300019	0.000243	0.001203
11718504_SRSF10	-0.31458	0.000244	0.001203

11746556_SLBP	-0.25604	0.000244	0.001204
11731629_MIOS	0.264144	0.000244	0.001204
11730846_PIK3R3	0.282858	0.000244	0.001205
11739856_TENM2	0.428439	0.000244	0.001205
11759733_FAM91A1	0.935627	0.000244	0.001206
11747371_ANKRD17	0.241435	0.000244	0.001206
11719917_PELO	0.27001	0.000244	0.001206
11745818_H1F0	-0.39465	0.000244	0.001207
11722990_BTAF1	0.284476	0.000244	0.001207
11728646_NUCKS1	-0.23605	0.000245	0.001207
11745977_SOX12	-0.48315	0.000245	0.001207
11744804_HIPK1	0.297211	0.000245	0.001207
11736915_HIST1H1D	-0.64495	0.000245	0.001208
11727846_CDKN2AIP	0.342889	0.000245	0.001208
11753596_SMIM15	0.246212	0.000245	0.001208
11742732_MORF4L1	0.217558	0.000245	0.001208
11724768_FCGR2A //	0.228743	0.000245	0.001209
11751615_TM9SF3	0.319855	0.000245	0.00121
11746235_LIN37	0.238564	0.000245	0.00121
11744850_SSH2	0.36168	0.000246	0.001211
11718572_SERBP1	-0.22207	0.000246	0.001212
11739610_CALML4	-0.29502	0.000246	0.001212
11758829_CHTOP	-0.24451	0.000246	0.001212
11723229_PHF23	0.258056	0.000246	0.001213
11757895_TULP4	-0.47144	0.000246	0.001214
11730327_DCAF13	-0.2549	0.000247	0.001215
11741242_ZNF674	0.48517	0.000247	0.001216
11756180_PHKB	0.256523	0.000247	0.001216
11724261_TRIO	0.364999	0.000247	0.001217
11723689_CREBL2	0.352439	0.000247	0.001217
11728049_HIST1H4J	0.240794	0.000247	0.001217
11720018_SMAP1	-0.27438	0.000247	0.001217
11744656_AZIN2	-0.2258	0.000247	0.001217
11719370_SLC25A44	0.253256	0.000247	0.001218
11728558_HSF2BP //	0.309645	0.000247	0.001218
11735115_CNOT4	0.32189	0.000247	0.001218
11749246_ENG	0.283699	0.000248	0.001218
11749145_RUFY3	0.463951	0.000248	0.001219
11719011_TMEM99	-0.25337	0.000248	0.001219
11730498_MXI1	-0.23487	0.000248	0.001219
11728911_CDK5R2	-0.23276	0.000248	0.001219
11755828_SZRD1	-0.29331	0.000248	0.001219
11716666_ID3	-0.28479	0.000248	0.001219
11717298_LRPPRC	0.281457	0.000248	0.001221
11752422_ANKLE1	-0.32294	0.000248	0.001222
11745508_YY1AP1	0.291663	0.000248	0.001222
11721522_APEX2	-0.23566	0.000248	0.001222
11756630_TMED10	0.274186	0.000249	0.001222
11715165_DNAJC12	0.223675	0.000249	0.001224
11730270_NIPBL	0.243943	0.000249	0.001224
11734988_FEM1B	0.27734	0.000249	0.001224

11747429_ZNF260	0.506796	0.000249	0.001224
11733403_CLEC7A	0.722866	0.000249	0.001224
11743167_ETV6	0.329414	0.000249	0.001224
11755298_UBE2Q2	0.286052	0.000249	0.001225
11724742_ZBTB17	0.301348	0.000249	0.001225
11751612_SOCS2	0.313779	0.00025	0.001226
11759947_KU-MEL-3	-0.23444	0.00025	0.001228
11759951_ZNF599	-0.42619	0.00025	0.001228
11725361_SMAD4	0.274103	0.00025	0.001228
11732227_PEX10	-0.32669	0.00025	0.001228
11754469_ZXDC	-0.34052	0.00025	0.001229
11719958_SGPL1	-0.26875	0.000251	0.00123
11746393_INTS6	0.243306	0.000251	0.00123
11722787_PKM	-0.25294	0.000251	0.001231
11718048_GLRX	0.262443	0.000251	0.001231
11758757_ADH5 ///	-0.37078	0.000251	0.001233
11757990_CFH /// C	0.259279	0.000251	0.001234
11746111_G3BP1	-0.28639	0.000252	0.001234
11756520_AKR7A2	-0.39218	0.000252	0.001235
11729675_SPRTN	0.321541	0.000252	0.001235
11757566_NOL8	0.222418	0.000252	0.001236
11761289_FPGT	-0.50224	0.000252	0.001236
11725121_GATAD1	0.330222	0.000252	0.001236
11732728_SLITRK4	-0.40811	0.000252	0.001236
11725828_ARMC9	0.313315	0.000252	0.001237
11743526_RALBP1	-0.37965	0.000252	0.001237
11716627_KIF3B	0.335078	0.000252	0.001237
11746816_PIAS2	0.28273	0.000252	0.001237
11757722_SEPT8	0.256354	0.000253	0.001237
11726305_FAM204A	0.274571	0.000253	0.001238
11743878_PPP1R1C	0.427473	0.000253	0.001239
11755894_MFN1	0.251819	0.000253	0.001239
11720963_DCK	-0.30317	0.000253	0.00124
11740310_ZNF460	0.599913	0.000253	0.00124
11757876_LYRM9	-0.34255	0.000253	0.001241
11723014_EXT2	0.278234	0.000253	0.001241
11743533_RUFY3	0.393336	0.000253	0.001241
11738115_ROR1	0.463801	0.000253	0.001241
11721108_ZNF627	0.27169	0.000254	0.001242
11725920_HEXIM2	-0.26318	0.000254	0.001243
11754160_MAX	0.276488	0.000254	0.001244
11743287_ZNF384	0.232011	0.000254	0.001244
11749656_ARHGAP11A	-0.2695	0.000254	0.001244
11758868_RAPGEF6	0.286026	0.000254	0.001245
11747332_HSPBP1	-0.2564	0.000254	0.001245
11728370_DNAJC16	0.237082	0.000255	0.001245
11735273_TBX18	0.275992	0.000255	0.001246
11743138_ 5-Mar	-0.31687	0.000255	0.001246
11716200_UBE2Q2	0.311666	0.000255	0.001246
11721860_STX12	0.314506	0.000255	0.001247
11729287_MSL3	0.247583	0.000255	0.001248

11749437_NFIC	-0.29478	0.000256	0.00125
11757619_SOD1	-0.25487	0.000256	0.00125
11747190_FCHSD1	0.305779	0.000256	0.001251
11718552_OTUD4	-0.30605	0.000256	0.001251
11734763_HIP1	-0.24223	0.000256	0.001252
11729686_LYRM7	-0.309	0.000256	0.001252
11735248_DUSP19	-0.26885	0.000256	0.001252
11746768_SUV39H1	-0.34954	0.000256	0.001252
11759039_SVIP	0.244261	0.000256	0.001253
11743980_ZFPL1	-0.26867	0.000257	0.001253
11744485_WDR81	-0.30598	0.000257	0.001253
11747975_RNF145	0.304696	0.000257	0.001253
11759719_PASK	-0.34108	0.000257	0.001256
11733010_VCP	-0.28207	0.000257	0.001256
11754694_ANKRD36 /	-0.44328	0.000258	0.001258
11760407_TMEM136	0.328253	0.000258	0.001258
11749731_SH2B3	0.22048	0.000258	0.001258
11718720_BRD8	-0.29244	0.000258	0.001259
11747155_FXYD6 ///	-0.38479	0.000258	0.001259
11716078_ELOVL5	-0.25979	0.000258	0.001259
11732585_IL1RAP	0.55088	0.000258	0.00126
11729140_SLAMF7	0.609737	0.000258	0.00126
11753400_SGOL1	-0.39421	0.000258	0.00126
11725758_ISL1	0.303055	0.000259	0.001262
11763580_BIRC5	-0.44134	0.000259	0.001262
11744698_TPRG1L	-0.3092	0.000259	0.001262
11754844_COPS5	0.239078	0.000259	0.001263
11750478_THEM6	-0.32237	0.000259	0.001263
11750704_SCMH1	-0.30293	0.000259	0.001264
11742480_GCDH	-0.25645	0.00026	0.001265
11726028_MC1R	-0.33644	0.00026	0.001265
11761331_ZNF720	0.933281	0.00026	0.001266
11730250_LNX1	0.32501	0.00026	0.001266
11744361_NCOA6	-0.25922	0.00026	0.001267
11731055_FNIP1	0.422272	0.00026	0.001268
11751213_SCOC	-0.27382	0.00026	0.001268
11726655_IFIT5	0.245375	0.000261	0.001269
11749532_RASGRF1	0.356313	0.000261	0.001269
11760918_MRRF	0.365838	0.000261	0.00127
11754242_TJP1	0.328783	0.000261	0.00127
11734069_SSR3	0.270795	0.000261	0.00127
11758984_ZRANB1	-0.37543	0.000261	0.00127
11760289_VAMP4	0.377275	0.000261	0.00127
11719061_ATRN	-0.26041	0.000261	0.00127
11755974_TUBB4B	-0.2457	0.000261	0.001271
11742435_DNM1L	-0.23278	0.000261	0.001272
11727394_COX15	-0.45973	0.000261	0.001273
11719552_RHOBTB1	0.426979	0.000262	0.001274
11759607_ATF7	-0.39014	0.000262	0.001274
11741302_GCOM1 ///	0.298867	0.000262	0.001274
11751174_SLC39A9	0.330456	0.000262	0.001275

11718765_NDUFA6	-0.23466	0.000262	0.001276
11721239_ATP5L	-0.24901	0.000262	0.001276
11747319_FXYD6 ///	-0.2766	0.000262	0.001276
11739918_RASGRP3	0.666301	0.000263	0.001278
11755790_FER1L4	0.602413	0.000263	0.001278
11721227_LEMD3	0.377512	0.000263	0.00128
11757687_DAD1	-0.2364	0.000263	0.00128
11720105_SLC26A2	0.256393	0.000263	0.00128
11746969_MBTD1	0.318858	0.000263	0.00128
11716528_ELAVL1	-0.40537	0.000263	0.00128
11759604_BCL2L13	-0.36101	0.000263	0.00128
11724315_LUZP1	0.337669	0.000264	0.001281
11757602_ERP29	-0.24739	0.000264	0.001283
11758454_DENND6A	-0.26407	0.000264	0.001284
11731095_TRAF3IP1	-0.26635	0.000265	0.001285
11720942_DDX54	-0.36701	0.000265	0.001287
11719276_MRPL17	-0.37883	0.000265	0.001287
11730476_KCNV1	0.418654	0.000265	0.001288
11737879_GALNT2	-0.26954	0.000265	0.001289
11719348_ARHGAP19	-0.24759	0.000266	0.001291
11716025_GOLGA7	0.247149	0.000266	0.001291
11744761_ARHGEF1 /	-0.23401	0.000266	0.001291
11757072_HS6ST1	-0.2817	0.000266	0.001292
11721095_SLC39A7	-0.39529	0.000266	0.001293
11721351_SLC1A1	0.280519	0.000266	0.001293
11758513_SPEN	0.232312	0.000266	0.001293
11731296_SPATA6L	0.522467	0.000266	0.001293
11721117_SPCS3	0.237112	0.000267	0.001293
11721541_GMEB1	0.280516	0.000267	0.001293
11755618_ALDH4A1	-0.40943	0.000267	0.001293
11719149_SAP130	-0.24127	0.000267	0.001293
11754489_NDUFAF5	0.262813	0.000267	0.001295
11721450_ETF1	0.220422	0.000267	0.001297
11729687_LYRM7	-0.3196	0.000268	0.001297
11722191_TTC37	0.251822	0.000268	0.001298
11735587_KCNH1	0.358385	0.000268	0.001301
11719145_TMEM54	-0.24857	0.000268	0.001301
11719104_CPNE3	-0.27982	0.000268	0.001301
11733198_DDX39A	-0.27801	0.000268	0.001301
11751505_YIPF1	0.313534	0.000269	0.001301
11727294_ALG10 ///	-0.37408	0.000269	0.001302
11753667_ITM2C	-0.2548	0.000269	0.001304
11749254_PRSS23	0.258922	0.000269	0.001304
11730827_MOB2	0.22569	0.000269	0.001305
11721360_FAM172A	-0.28519	0.000269	0.001305
11750694_RECQL	-0.26099	0.00027	0.001305
11754195_XIST	0.344826	0.00027	0.001305
11751916_KIAA0586	-0.23774	0.00027	0.001306
11729893_ZNF569	0.306983	0.00027	0.001306
11724546_HCFC2	0.593003	0.00027	0.001308
11721158_DRAM2	0.263394	0.00027	0.001308

11753967_SLC6A2	-0.34586	0.000271	0.001309
11719625_CDC16	0.275872	0.000271	0.00131
11720096_LIMD2	0.26233	0.000271	0.001311
11739930_MCM8	-0.25572	0.000271	0.001311
11758355_CLDN4	-0.47128	0.000271	0.001311
11745208_SAMD4A	0.413534	0.000271	0.001312
11756397_PTOV1	-0.24385	0.000271	0.001313
11744414_HTATSF1	-0.28135	0.000272	0.001314
11722108_PCNX	-0.27199	0.000272	0.001314
11749586_RMDN2	0.337044	0.000272	0.001314
11743786_THAP9	0.387116	0.000272	0.001316
11722552_ITSN1	-0.54609	0.000272	0.001316
11754000_CD58	0.21644	0.000272	0.001316
11753385_C19orf54	-0.36024	0.000272	0.001316
11748518_CSRP1	-0.24093	0.000273	0.001318
11731052_CEP126	0.267456	0.000273	0.001319
11731583_LARP1B	0.361297	0.000274	0.001322
11725789_DPYD	0.244405	0.000274	0.001323
11751829_PINLYP	0.296369	0.000274	0.001324
11739301_TERF1	-0.28822	0.000274	0.001326
11728697_HSPA4L	0.435268	0.000275	0.001326
11747409_ARHGDIA	-0.2258	0.000275	0.001326
11758040_BAZ2B	0.616986	0.000275	0.001326
11738297_CNGA2	-0.26263	0.000275	0.001327
11740012_NEIL3	-0.9682	0.000275	0.001327
11756387_ARL4A	0.257245	0.000275	0.001328
11737883_MAGEA10	-0.22855	0.000275	0.001328
11750906_RAD1	-0.37045	0.000275	0.001329
11716038_SLC25A29	-0.32221	0.000275	0.001329
11737772_ZNF93	0.333857	0.000276	0.001331
11754404_PARP14	0.366966	0.000276	0.001332
11722376_MEAF6	-0.40035	0.000276	0.001332
11719067_EIF2S3	0.244543	0.000276	0.001332
11754690_HUWE1	-0.27507	0.000276	0.001333
11726807_ZAK	-0.22507	0.000276	0.001333
11752872_SEC23A	0.300731	0.000277	0.001334
11757839_NECAP2	0.268498	0.000277	0.001334
11745421_BID	0.354163	0.000277	0.001335
11757162_SNORA40 /	-0.33013	0.000277	0.001335
11740560_NSMAF	0.280055	0.000277	0.001336
11754476_DNM1	-0.30894	0.000277	0.001336
11721293_GIT2	0.278213	0.000277	0.001336
11731581_WASF1	0.267815	0.000277	0.001336
11737338_ZNF527	0.508216	0.000278	0.001338
11746899_UROS	0.217876	0.000278	0.001339
11741277_TMEM144	-0.2301	0.000278	0.001341
11749840_CPEB1	0.358814	0.000278	0.001341
11744492_SRSF5	-0.30193	0.000278	0.001341
11745996_APH1A	-0.2174	0.000278	0.001341
11745890_WSB1	0.305345	0.000278	0.001341
11757880_DAG1	-0.26851	0.000278	0.001341

11729719_SMAD5	-0.36463	0.000279	0.001343
11719297_MAPKAP1	-0.23052	0.000279	0.001343
11744501_ASXL1	0.349948	0.000279	0.001343
11744222_ATP5D	-0.32892	0.000279	0.001344
11751791_CCDC80	0.531645	0.000279	0.001344
11744794_MBNL1	0.310357	0.000279	0.001344
11724917_TMTC3	0.255959	0.000279	0.001345
11741147_ZNF207	0.234018	0.00028	0.001345
11731805_DCUN1D3	0.453773	0.00028	0.001346
11743611_ZMYND11	0.248885	0.00028	0.001347
11744171_DNAJC4	-0.27544	0.00028	0.001347
11720468_DPAGT1	-0.24712	0.00028	0.001347
11743725_MFSD1	0.254058	0.00028	0.001347
11754155_ANP32A //	-0.2597	0.00028	0.001347
11715749_SSRP1	-0.2297	0.00028	0.001348
11741228_PHACTR4	0.226805	0.00028	0.001348
11763997_IL31RA	0.576738	0.000281	0.001349
11723809_STX17	0.340314	0.000281	0.001351
11718829_EHD1	0.27481	0.000281	0.001353
11748810_EVA1C	-0.49986	0.000282	0.001354
11724888_CARD10	0.322349	0.000282	0.001355
11744829_HLA-E	0.261173	0.000282	0.001356
11729099_IL1RAP	0.427254	0.000282	0.001356
11721726_IFT88	0.40787	0.000282	0.001356
11731613_RHEBL1	-0.49955	0.000282	0.001356
11725800_USP46	0.264887	0.000282	0.001356
11719283_UBR7	-0.37274	0.000282	0.001357
11719830_ERCC1	0.24887	0.000283	0.001357
11718371_NRBF2	0.281996	0.000283	0.001358
11722402_CSTF2	-0.23907	0.000283	0.001359
11757462_OSGEP	0.292888	0.000283	0.001359
11721357_NMB	-0.22982	0.000283	0.001359
11763981_XPNPEP3	-0.24604	0.000283	0.001359
11723482_TIA1	0.248939	0.000283	0.001359
11754232_CMTR2	0.313285	0.000283	0.00136
11758392_NCAM2	-0.45208	0.000284	0.001361
11759302_ROCK2	0.2895	0.000284	0.001362
11725140_FUBP3	-0.3354	0.000284	0.001362
11720868_ZNF275	0.285958	0.000284	0.001362
11759079_PLRG1	-0.22456	0.000284	0.001363
11750126_MANBA	0.268108	0.000284	0.001363
11740375_RGS10	0.233075	0.000284	0.001363
11716678_SLC39A9	0.326639	0.000284	0.001364
11732481_ITGAM	0.306172	0.000284	0.001364
11719667_STMN1	-0.22069	0.000284	0.001364
11756789_ATRNL1	-0.36271	0.000285	0.001364
11719286_PEX11B	-0.25301	0.000285	0.001365
11718657_MIR21 ///	0.221654	0.000285	0.001366
11750864_CRISPLD1	-0.28905	0.000285	0.001367
11727207_DDHD1	0.543409	0.000285	0.001367
11718317_SLC25A10	-0.41422	0.000285	0.001368

11745509_IQGAP2	0.226827	0.000285	0.001368
11739244_ANKFY1	0.294119	0.000285	0.001368
11720374_PRUNE	0.306497	0.000286	0.001369
11750949_NMT2	0.325096	0.000286	0.00137
11716300_ITGAV	-0.63546	0.000286	0.001371
11737761_HSD17B4	-0.24372	0.000286	0.001371
11741138_GSDMD	-0.22681	0.000286	0.001372
11761481_MPI	0.458488	0.000287	0.001373
11724514_GPD1L	-0.59023	0.000287	0.001375
11744584_HYI	0.328812	0.000287	0.001376
11750132_PAN3	0.287578	0.000287	0.001376
11737993_DST	0.223006	0.000287	0.001376
11758616_H2AFV	-0.38802	0.000288	0.001377
11733064_SIL1	0.291984	0.000288	0.001377
11723430_ZFP91 ///	0.263575	0.000288	0.001377
11722954_BIK	0.239358	0.000288	0.001377
11717229_CDR2 ///	0.281376	0.000288	0.001378
11756955_GZF1	0.379392	0.000288	0.001378
11745727_TCF12	0.235823	0.000288	0.00138
11752588_ABCE1	-0.26047	0.000289	0.00138
11763377_RBM5	0.285004	0.000289	0.001381
11743806_MRPL42	-0.23527	0.000289	0.001381
11750011_MLST8	-0.23227	0.000289	0.001382
11754251_USP36	0.406654	0.000289	0.001382
11722341_PRR12	-0.30156	0.000289	0.001383
11720170_MRPS16	-0.247	0.00029	0.001384
11722530_CALD1	0.252461	0.00029	0.001384
11729666_STK38L	-0.26629	0.00029	0.001385
11732582_NUDT22	-0.31356	0.00029	0.001385
11747520_PMS2CL	0.501447	0.00029	0.001386
11759758_LOC339803	-0.3455	0.00029	0.001387
11731994_SPANXA1 /	0.274274	0.00029	0.001387
11763542_SLC12A4	0.282253	0.000291	0.001389
11734358_RNASEL	0.373744	0.000291	0.001389
11730931_ACSS3	0.349822	0.000291	0.00139
11742995_RAB10	0.254338	0.000291	0.00139
11751753_FAM122B	-0.36916	0.000291	0.00139
11730749_CRNKL1	0.282232	0.000291	0.00139
11715698_NOLC1	-0.23255	0.000291	0.001391
11757805_ATRAID	-0.23622	0.000292	0.001392
11761938_TRIO	0.327308	0.000292	0.001392
11746829_DDX55	0.229215	0.000292	0.001392
11715727_OAZ2	-0.37689	0.000292	0.001392
11741750_FBXO9	0.23811	0.000292	0.001393
11754040_FXYD3	-0.30207	0.000292	0.001393
11734520_SCLT1	-0.26479	0.000292	0.001393
11725197_CAMKMT	0.368813	0.000292	0.001393
11754438_GJC1	-0.27314	0.000292	0.001394
11742431_DGKI	0.347916	0.000292	0.001394
11716634_TMEM50A	-0.24321	0.000293	0.001396
11735090_SLC16A6	0.6664	0.000293	0.001397

11718920_IQSEC1	-0.31014	0.000293	0.001398
11717159_NDUFB3	-0.29219	0.000293	0.001398
11752258_ACSL4	0.321752	0.000293	0.001399
11751483_BGN	-0.30356	0.000293	0.001399
11716934_CCDC80	0.602695	0.000294	0.0014
11742052_ARNT	0.311664	0.000294	0.0014
11759993_ORAOV1	0.413213	0.000294	0.001401
11752957_DZANK1	0.287769	0.000294	0.001401
11750921_LCLAT1	0.440997	0.000294	0.001401
11717856_PSMG1	-0.30093	0.000294	0.001403
11743563_MAP7D3	-0.31481	0.000295	0.001404
11727709_LIPT1	0.405494	0.000295	0.001404
11759293_KMT2A	0.252728	0.000295	0.001404
11719892_VASH1	-0.31331	0.000295	0.001405
11722701_KIAA1109	0.338175	0.000295	0.001405
11755075_NADSYN1	0.465501	0.000295	0.001406
11725049_ARHGDIA	-0.24088	0.000295	0.001406
11747567_CNP	-0.34819	0.000295	0.001406
11746150_AGAP9 ///	0.257207	0.000296	0.001408
11719751_VAC14	-0.23632	0.000296	0.001409
11754298_CSNK1D	0.260886	0.000296	0.001409
11731224_SHOX2	-0.54257	0.000296	0.00141
11757051_TWF2	-0.28345	0.000296	0.00141
11720457_CAPRIN1	-0.5739	0.000297	0.001411
11759723_TUBGCP3	-0.41517	0.000297	0.001411
11743074_IPO9	-0.36063	0.000297	0.001412
11752489_SLC47A1	-0.76035	0.000297	0.001413
11755719_MLLT11	-0.22275	0.000297	0.001414
11756849_NOP2	0.24935	0.000298	0.001415
11742401_C9orf9	0.305083	0.000298	0.001415
11720641_MRPS27	0.240407	0.000298	0.001415
11747415_CKAP5	-0.23073	0.000298	0.001416
11716042_PPP2CB	-0.23078	0.000298	0.001416
11721276_TADA1	-0.24751	0.000298	0.001417
11733920_SERTAD2	0.306099	0.000298	0.001417
11717150_USB1	-0.25664	0.000298	0.001417
11725303_RAC1	-0.27401	0.000298	0.001418
11763259_CFL1	-0.24601	0.000298	0.001418
11755381_PLGLA ///	0.357923	0.000298	0.001418
11756264_IPW	-0.42236	0.000299	0.001418
11744575_ELOVL2	0.396806	0.000299	0.001419
11755343_SETMAR	-0.23547	0.000299	0.00142
11745444_TMEM161B	0.313379	0.000299	0.00142
11716953_TMEM126A	0.226885	0.000299	0.00142
11724596_TANK	0.330351	0.000299	0.00142
11759322_BCL6	0.329005	0.000299	0.001421
11734087_GALE	0.249442	0.0003	0.001422
11727466_FLJ10038	0.298762	0.0003	0.001423
11718347_S100P	-0.30168	0.0003	0.001426
11724400_ENKD1	-0.23158	0.000301	0.001426
11718850_SRPK1	0.262594	0.000301	0.001426

11742238_DEPDC1B	-0.29012	0.000301	0.001426
11746137_SRSF5	-0.25881	0.000301	0.001428
11732318_KHDRBS3	0.379238	0.000301	0.001428
11749387_IQCK	0.306643	0.000301	0.001428
11716177_METAP2	-0.27691	0.000301	0.001429
11759734_BLZF1	0.853133	0.000301	0.001429
11720112_SNTB2	-0.23294	0.000302	0.001429
11758285_GTF3C1	-0.25791	0.000302	0.001429
11736157_TBC1D2B	-0.4623	0.000302	0.001429
11744289_JMJD6	0.259204	0.000302	0.00143
11757167_HSPA8	-0.26379	0.000302	0.00143
11741016_AP2A2	0.307168	0.000302	0.001431
11739061_SLC7A5	-0.26405	0.000302	0.001431
11717577_VPS26A	-0.24641	0.000302	0.001431
11753994_IQCB1	0.279706	0.000302	0.001431
11716241_RAB6A	0.275423	0.000302	0.001431
11739268_PAK2	0.291788	0.000302	0.001432
11717543_MAPK3	-0.34831	0.000302	0.001432
11739346_TNFRSF1A	0.303372	0.000303	0.001433
11746384_RICTOR	0.300285	0.000303	0.001433
11753860_GNG4	-0.30875	0.000303	0.001433
11758177_LRWD1	0.296524	0.000303	0.001433
11734806_RDH13	-0.23071	0.000303	0.001434
11727047_SGCE	-0.25038	0.000303	0.001434
11727065_ANKRD11	0.270926	0.000303	0.001435
11743008_ARHGEF1 /	-0.24336	0.000303	0.001435
11717535_TMEM259	-0.35879	0.000303	0.001436
11730222_PNPLA2	-0.28912	0.000304	0.001437
11715804_PPP2CA	-0.28813	0.000304	0.001439
11739990_SRI	-0.52725	0.000304	0.001439
11748108_TEX9	0.559911	0.000304	0.001439
11755665_VARS2	-0.27093	0.000304	0.001439
11729505_HDDC3	-0.23258	0.000304	0.00144
11751253_GOLPH3	-0.25153	0.000305	0.00144
11733741_SYMPK	-0.25857	0.000305	0.00144
11731499_ZNF134	-0.25554	0.000305	0.00144
11753142_PSMD11	-0.22032	0.000305	0.00144
11736821_TP73	-0.369	0.000305	0.001441
11751151_BRAP	0.465249	0.000305	0.001442
11757686_PCID2	0.281044	0.000306	0.001444
11752928_PPIA	-0.36757	0.000306	0.001445
11736731_RIMS2	0.967138	0.000306	0.001445
11732413_LRRC8B	-0.32702	0.000306	0.001445
11716433_SCD5	-0.41596	0.000306	0.001447
11723475_TRMT61B	-0.29121	0.000306	0.001447
11736357_CLSPN	-0.35143	0.000306	0.001447
11717366_ZC3HAV1	0.30376	0.000307	0.001449
11759542_PACSIN2	-0.32273	0.000307	0.001451
11728045_EXOC3-AS1	-0.27937	0.000307	0.001451
11763467_WHSC1	-0.40578	0.000308	0.001452
11755502_TECR	-0.24415	0.000308	0.001454

11739377_SREK1	0.279064	0.000308	0.001455
11720435_CLCN6	0.256421	0.000308	0.001455
11751347_FMR1	0.374708	0.000308	0.001455
11743336_UIMC1	0.256054	0.000308	0.001455
11730992_EIF2AK2	0.229663	0.000308	0.001455
11730657_CHD2	0.236905	0.000309	0.001457
11715500_TARDBP	-0.22589	0.000309	0.001458
11730272_PPP2R5E	0.243157	0.000309	0.001458
11730197_RNF187	-0.24156	0.000309	0.00146
11716331_DNAJB6	0.23544	0.00031	0.001461
11747937_DNAJB6	0.23544	0.00031	0.001461
11728703_KIAA0232	-0.29129	0.00031	0.001463
11716308_CYBRD1	0.258188	0.00031	0.001464
11742946_UBE2E3	0.222971	0.000311	0.001467
11733040_DPH3	0.239185	0.000311	0.001467
11741086_APLP2	-0.27326	0.000311	0.001467
11716129_IGF2R	0.280016	0.000311	0.001468
11751739_PTPRE	0.428324	0.000312	0.001468
11726539_OSBPL9	0.247141	0.000312	0.00147
11717600_SECISBP2	0.258494	0.000312	0.00147
11717511_UTP4	0.228883	0.000312	0.00147
11718599_TM2D2	-0.2851	0.000312	0.00147
11733219_TFAP4	-0.32416	0.000312	0.001471
11718023_SSFA2	0.256736	0.000312	0.001471
11747793_PTK2	0.298884	0.000312	0.001471
11724950_CPEB2	0.358461	0.000313	0.001472
11758883_SP3	-0.31991	0.000313	0.001473
11743755_ZFAND5	0.270039	0.000313	0.001473
11753122_TMEM109	-0.23759	0.000313	0.001473
11741316_RTN3	-0.24221	0.000313	0.001474
11754338_RABL6	-0.22996	0.000313	0.001474
11727223_CSGALNACT	0.476277	0.000313	0.001475
11728018_RHPN1	0.271701	0.000314	0.001475
11750622_ALS2	0.274971	0.000314	0.001475
11756472_TRMT61B	-0.28216	0.000314	0.001476
11722085_PDCD2L	0.269396	0.000314	0.001476
11750765_ORC3	-0.3059	0.000314	0.001477
11726747_TMEM57	-0.31928	0.000314	0.001478
11721696_MRPL18	-0.2144	0.000315	0.001479
11732604_TRIM45	-0.32607	0.000315	0.00148
11730367_ZNF557	0.319644	0.000315	0.00148
11754117_ST3GAL4	-0.26606	0.000315	0.00148
11736543_CARNMT1	-0.36491	0.000315	0.00148
11725819_PAQR3	0.27056	0.000315	0.00148
11743101_EIF3F	0.356375	0.000315	0.001481
11726293_WDR44	0.302037	0.000315	0.001481
11721739_LATS2	0.309595	0.000315	0.001481
11724252_SLC39A8	-0.37617	0.000315	0.001481
11724313_ALDH16A1	-0.379	0.000315	0.001481
11746591_CDH13	0.231584	0.000315	0.001482
11758785_SLC25A1	-0.22823	0.000315	0.001482

11723888_ZNF251	0.285165	0.000316	0.001482
11739029_CLTC	0.219115	0.000316	0.001483
11757703_SNRPG	-0.24094	0.000316	0.001483
11748203_PPP6R2	0.24485	0.000316	0.001483
11757816_MARCKS	-0.23533	0.000316	0.001484
11731680_NOX4	0.264734	0.000316	0.001484
11757025_SERPINF1	-0.34703	0.000317	0.001487
11724133_SLC12A2	-0.39422	0.000317	0.001488
11717829_TSC22D3	0.279473	0.000317	0.001489
11731737_PGAP1	0.309677	0.000317	0.00149
11728177_DYX1C1	0.425385	0.000318	0.001491
11748894_ACD	-0.22553	0.000318	0.001491
11754979_ARIH2	0.291001	0.000318	0.001492
11729298_SDE2	-0.2334	0.000318	0.001493
11763721_APH1A	-0.30061	0.000318	0.001494
11725974_POM121 //	-0.22853	0.000319	0.001495
11722025_ABCF2	0.284301	0.000319	0.001495
11719298_MAPKAP1	-0.24977	0.000319	0.001495
11718896_SPAG9	-0.32395	0.000319	0.001495
11751633_PRSS23	0.209406	0.000319	0.001495
11761781_SRSF11	0.312957	0.000319	0.001495
11754783_COMMD3	0.216875	0.000319	0.001495
11745285_AFF4	0.352688	0.00032	0.001498
11738847_GFM2	0.306467	0.00032	0.001498
11742891_C17orf53	-0.21537	0.00032	0.001499
11729645_GPX8	-0.32569	0.00032	0.0015
11759804_PDCD6	-0.30056	0.00032	0.0015
11736346_HFE	-0.31412	0.00032	0.001501
11743972_DDIT4	-0.2451	0.00032	0.001501
11722273_MSANTD4	0.254968	0.000321	0.001502
11734364_RFX3	0.316396	0.000321	0.001502
11742415_BAHCC1	-0.33531	0.000321	0.001503
11757683_IRF2BP2	-0.42298	0.000321	0.001504
11721861_STX12	-0.34431	0.000321	0.001504
11726454_MYO5B	0.494663	0.000321	0.001504
11736209_METTL2B	0.255209	0.000322	0.001505
11756206_YIPF1	0.292266	0.000322	0.001506
11744792_SLF2	-0.31992	0.000322	0.001506
11744807_POLM	0.243696	0.000322	0.001507
11734361_SLC16A7	0.367024	0.000322	0.001508
11762406_GBP2	-0.35649	0.000323	0.00151
11724010_GPR162	-0.22416	0.000323	0.00151
11749517_EHD1	0.281543	0.000323	0.001511
11755771_ZNF275	0.259649	0.000323	0.001511
11746213_PAAF1	-0.22563	0.000323	0.001512
11760043_XRN1	0.587306	0.000323	0.001512
11740788_CDH12	0.408285	0.000324	0.001516
11722222_TMEM160	-0.35234	0.000324	0.001516
11727557_TK2	-0.27344	0.000324	0.001517
11716899_ARL3	-0.35514	0.000324	0.001517
11721607_RAPGEF2	0.527234	0.000324	0.001517

11728417_ELMOD1 //	0.292678	0.000325	0.001517
11745604_NFKBIB	0.519526	0.000325	0.00152
11754757_ARID2	-0.49225	0.000325	0.00152
11724198_STC1	0.47162	0.000325	0.001521
11751867_C8orf59	-0.24264	0.000325	0.001521
11721000_HEXIM1	-0.26685	0.000325	0.001521
11755415_EIF2B4	0.259336	0.000326	0.001521
11721388_P2RX5	0.404634	0.000326	0.001521
11747156_FXYD6 ///	-0.27914	0.000326	0.001521
11760463_MED6	0.490338	0.000326	0.001521
11727922_PURA	-0.29219	0.000326	0.001522
11743000_CD83	-0.46759	0.000326	0.001523
11748356_CEP70	-0.28163	0.000326	0.001524
11717230_EP400	-0.24415	0.000327	0.001525
11722275_ZBED4	-0.3744	0.000327	0.001526
11760234_GPATCH11	-0.21544	0.000327	0.001526
11749838_DCTN4	0.270903	0.000327	0.001526
11731432_PCDH10	0.317866	0.000327	0.001526
11723908_KCNMA1	0.429989	0.000327	0.001527
11719432_KIAA0513	-0.24135	0.000327	0.001527
11733480_SUPT20H	0.245651	0.000327	0.001528
11747778_RAB6A	0.24134	0.000328	0.001528
11755586_MAN2C1	0.229471	0.000328	0.00153
11715403_COL6A1	0.28723	0.000329	0.001534
11733140_ARL4A	0.299666	0.000329	0.001535
11757296_EIF3F	0.210614	0.000329	0.001535
11725730_LUC7L	0.272926	0.000329	0.001536
11750876_SCFD1	0.262278	0.00033	0.001537
11744612_NUDCD1	0.257098	0.00033	0.001537
11753896_PDHA1	-0.24935	0.00033	0.001538
11722591_BCAP29	0.224275	0.00033	0.001539
11750529_ASB9	-0.30316	0.00033	0.001539
11751943_LRCH2	-0.32851	0.00033	0.001539
11743751_NUDT13	0.564934	0.000331	0.001542
11723913_CACTIN	-0.25747	0.000331	0.001542
11739234_SAFB2	0.242387	0.000331	0.001544
11716987_HMGCS1	0.223347	0.000332	0.001545
11755213_KIAA1522	-0.37122	0.000332	0.001546
11743395_BMI1 ///	0.248771	0.000332	0.001546
11729275_NPAS2	0.371423	0.000332	0.001546
11753327_ZNF274	0.215185	0.000332	0.001546
11753023_IL31RA	0.675776	0.000333	0.00155
11725528_ZNF248	0.572264	0.000333	0.00155
11739236_PDE12	0.266002	0.000333	0.00155
11755904_RILPL1	0.269393	0.000333	0.001552
11729252_SLC44A3	0.400118	0.000333	0.001552
11727578_TCP11	-0.32925	0.000333	0.001552
11724300_RABGGTB	0.220919	0.000334	0.001553
11744493_ADGRA2	-0.29151	0.000334	0.001553
11728028_PWWP2A	0.479934	0.000334	0.001554
11718491_FABP5	-0.32944	0.000334	0.001554

11729448_TINF2	-0.29434	0.000334	0.001554
11720130_COMTD1	-0.21608	0.000334	0.001554
11719687_SETDB1	0.315409	0.000335	0.001558
11745913_GOLGA8A /	0.382364	0.000335	0.001559
11756326_BPNT1	-0.23306	0.000336	0.001561
11720282_NACC1	-0.26997	0.000336	0.001561
11724876_AEBP2	0.228885	0.000336	0.001561
11722641_DGAT2	-0.27775	0.000336	0.001561
11744975_DOC2A	-0.22996	0.000337	0.001564
11719606_RRN3	0.220645	0.000337	0.001564
11737058_PANK2	0.306146	0.000337	0.001566
11734106_FBXO27	-0.25649	0.000337	0.001567
11724417_C7orf43	0.272908	0.000337	0.001567
11755484_NUP107	-0.24183	0.000338	0.001571
11740024_RUFY1	0.267103	0.000338	0.001571
11716955_CTDSP2	-0.26304	0.000338	0.001572
11716026_SELT	0.289592	0.000338	0.001572
11721863_SLC37A3	0.382767	0.000339	0.001573
11728349_STIM2	0.246975	0.000339	0.001575
11721039_CAPN15	0.205851	0.000339	0.001576
11722963_COPZ2	0.254117	0.000339	0.001576
11717652_SPRY1	-0.29938	0.000339	0.001576
11738073_SPRY1	-0.29938	0.000339	0.001576
11748725_PHEX	0.388994	0.00034	0.001577
11737700_DDX31	0.352566	0.00034	0.001577
11725260_REG3G	-0.25727	0.00034	0.001577
11731420_UBE2G2	0.224924	0.00034	0.001579
11723420_BTF3L4	0.257177	0.00034	0.00158
11739868_ATF2	0.420581	0.000341	0.001581
11753235_E2F6	0.262387	0.000341	0.001581
11729806_UBXN11	0.356683	0.000341	0.001581
11739400_FBXO7	-0.24358	0.000341	0.001583
11724824_LSM12	0.221706	0.000342	0.001585
11763511_RMDN2	0.344815	0.000342	0.001585
11718007_MYLIP	-0.26752	0.000342	0.001585
11761907_NR1I3	0.279361	0.000342	0.001585
11717566_EMP1	0.26273	0.000342	0.001586
11759943_FAM13A	0.655728	0.000342	0.001586
11757157_RPL27A //	-0.28748	0.000342	0.001586
11763757_CLPX	0.271653	0.000342	0.001586
11727904_IL13RA1	0.534139	0.000342	0.001586
11725228_P2RY6	-0.28287	0.000342	0.001586
11756058_CEACAM1	0.26648	0.000342	0.001586
11737902_METTL5	-0.23521	0.000343	0.001587
11739880_GLIS3	0.261872	0.000343	0.001587
11758212_EFCAB14	0.243505	0.000343	0.001587
11754883_RFC3	-0.36416	0.000343	0.001587
11755602_ENO3	0.479708	0.000343	0.001587
11718368_LAMA5	0.254743	0.000343	0.001589
11748670_PIK3R1	0.262626	0.000343	0.00159
11742338_KRTAP4-9	0.304802	0.000344	0.001591

11751800_MDC1	-0.35051	0.000344	0.001591
11741113_PDCD4	-0.24103	0.000344	0.001592
11730455_SLC35A3	0.372341	0.000344	0.001592
11744628_ZCCHC7	0.234304	0.000344	0.001592
11750774_FBXO27	-0.28966	0.000344	0.001593
11733891_FKBP14	0.318909	0.000344	0.001594
11720933_DAGLB	0.353088	0.000345	0.001595
11753221_RANBP3	-0.23124	0.000345	0.001595
11717250_NAE1	-0.21386	0.000345	0.001596
11718900_TGFBR3	0.422038	0.000345	0.001596
11721759_ASAP3	-0.36006	0.000345	0.001596
11752004_GCC2 ///	0.326758	0.000345	0.001596
11722355_DAB2	0.275127	0.000345	0.001597
11719507_CSRP2BP	-0.48523	0.000345	0.001597
11734883_ARID2	0.244638	0.000345	0.001597
11731480_SNRPB2	-0.3941	0.000346	0.001597
11740134_CALD1	0.243768	0.000346	0.001597
11736551_REL	0.477691	0.000346	0.001598
11722363_LMBR1L	0.262514	0.000346	0.001598
11750404_RNF145	0.241687	0.000346	0.001598
11715678_NAP1L4	-0.24384	0.000346	0.001599
11745409_LARP1B	0.294638	0.000346	0.001599
11755478_PROSC	0.218362	0.000346	0.001599
11757623_RNF5 ///	-0.29065	0.000346	0.0016
11750911_TAF15	0.269676	0.000346	0.0016
11726924_ASB6	0.261304	0.000347	0.0016
11757440_PTP4A2	0.214322	0.000347	0.001601
11726290_CD22	0.271174	0.000347	0.001602
11744767_BCKDK	-0.21501	0.000347	0.001604
11744919_TPGS2	-0.29079	0.000348	0.001605
11744335_PFKL	-0.26959	0.000348	0.001605
11726014_GPC6	0.244269	0.000348	0.001605
11760138_NCAPG2	-0.33371	0.000348	0.001606
11750016_MXD1	0.875006	0.000348	0.001607
11749029_SART3	-0.24712	0.000348	0.001607
11728648_NUCKS1	-0.25696	0.000348	0.001607
11751496_LATS1	0.590972	0.000348	0.001607
11756272_NAA16	0.337964	0.000349	0.001609
11727345_FBLN1	-0.36633	0.000349	0.001609
11722871_DYNC2H1	0.28286	0.000349	0.001611
11753774_TMEM203	-0.26453	0.000349	0.001611
11756313_SERPINF1	-0.26582	0.00035	0.001614
11724033_CD2AP	0.354046	0.00035	0.001614
11748637_REEP4	-0.44013	0.00035	0.001615
11747856_SH3PXD2A	-0.3687	0.00035	0.001616
11745991_UVRAG	0.281193	0.000351	0.001616
11718880_ZCCHC14	-0.2158	0.000351	0.001617
11744343_ZNF664	-0.21605	0.000351	0.001617
11757910_EIF2S3	0.216173	0.000351	0.001617
11753664_RDM1	-1.2009	0.000352	0.00162
11726971_TRIM41	0.241324	0.000352	0.001621

11721507_DVL3	0.237148	0.000352	0.001622
11735203_MEGF10	0.673935	0.000352	0.001623
11735803_NAT6	-0.43965	0.000353	0.001624
11740096_TMCC1	0.331573	0.000353	0.001624
11726343_DNAJB14	0.307086	0.000353	0.001625
11744209_SERPINH1	-0.26377	0.000353	0.001625
11715563_SCD	-0.36104	0.000353	0.001625
11740444_ELF1	0.309344	0.000353	0.001625
11746299_CNTLN	-0.31277	0.000353	0.001625
11758369_HLA-DPB1	-0.27593	0.000353	0.001626
11739999_SCN2A	0.798144	0.000354	0.001628
11744400_MYL6B	-0.23154	0.000354	0.001628
11728796_LSM14A	-0.24875	0.000354	0.001629
11724392_ASPHD2	0.257658	0.000354	0.00163
11730219_SPG20	-0.3359	0.000354	0.00163
11726623_DIS3	0.265598	0.000354	0.00163
11717130_SAR1A	0.258776	0.000354	0.00163
11723945_PTTG1IP	0.249632	0.000355	0.001633
11728461_CARD9 ///	-0.25356	0.000355	0.001633
11753266_TCEA3	0.449481	0.000356	0.001635
11734416_ZNF718	-0.28647	0.000356	0.001636
11723446_TMEM132A	0.266234	0.000356	0.001636
11728311_ERCC1	0.240106	0.000356	0.001636
11737511_SP100	0.369814	0.000356	0.001637
11719631_BIN1	0.348599	0.000356	0.001638
11717053_SSR1	0.255687	0.000357	0.001639
11724626_IFT74	0.225579	0.000357	0.001639
11736276_AMDHD2	-0.27749	0.000357	0.001639
11726380_WHSC1	-0.48378	0.000357	0.00164
11722110_PCNX	-0.41196	0.000357	0.00164
11729236_MCCC2	-0.24177	0.000357	0.00164
11753174_ZNF410	0.359523	0.000357	0.001641
11719782_RPS23	-0.20648	0.000357	0.001641
11746866_APP	0.289134	0.000357	0.001641
11720437_ACVR1B	0.296251	0.000358	0.001642
11726614_CDH2	0.226665	0.000358	0.001643
11721277_SUV39H1	-0.3043	0.000358	0.001643
11743152_TUSC3	0.260526	0.000358	0.001643
11730261_PRKAR2B	-0.24068	0.000358	0.001644
11763279_TMEM136	0.355122	0.000358	0.001644
11745609_WDR1	0.241226	0.000358	0.001644
11764141_SLC25A1	-0.2171	0.000358	0.001644
11721631_TMEM97	-0.30631	0.000359	0.001645
11754105_SNRPB	-0.24606	0.000359	0.001646
11716657_TRAF4	0.327656	0.000359	0.001647
11717884_ZHX3	-0.3627	0.000359	0.001648
11760371_LOC100507	0.283507	0.000359	0.001648
11718269_ANGPTL2	0.416522	0.00036	0.001651
11759454_TUBB8	-0.74325	0.00036	0.001651
11749243_FAM98A	0.249415	0.00036	0.001651
11736038_TADA3	-0.41127	0.00036	0.001651

11718622_PPP5C	-0.32844	0.00036	0.001651
11718150_PRADC1	-0.25886	0.00036	0.001651
11746412_VCP	-0.29039	0.00036	0.001651
11753995_SNCG	-0.23566	0.000361	0.001653
11726298_LGALS8	0.266651	0.000361	0.001654
11740081_EPOR	-0.31453	0.000362	0.001659
11732571_ZNF582	0.622352	0.000362	0.001659
11716176_METAP2	-0.22858	0.000362	0.00166
11747355_FSCN1	-0.22912	0.000363	0.001661
11721238_ATP5L	-0.29062	0.000363	0.001663
11731772_KIAA1161	-0.24756	0.000363	0.001664
11748536_DIMT1	-0.27063	0.000363	0.001664
11725705_AMOTL1	-0.25476	0.000363	0.001664
11746197_HUS1	0.24114	0.000364	0.001665
11718026_NCOA7	0.244612	0.000364	0.001665
11721501_SWAP70	0.22088	0.000364	0.001666
11736693_TRIM38	0.459376	0.000364	0.001666
11726205_CCNJL	0.480973	0.000364	0.001666
11755687_TGOLN2	0.223309	0.000364	0.001667
11716785_TRAK2	0.452408	0.000364	0.001667
11722238_DPM3	-0.23845	0.000365	0.001671
11716435_RAPGEF1	0.25202	0.000365	0.001671
11751064_EXOG	0.359514	0.000365	0.001671
11746221_ATG4D	-0.36317	0.000365	0.001672
11727019_ABI2	-0.31967	0.000366	0.001672
11737555_NUDT17	0.23262	0.000366	0.001672
11728392_LRP12	0.287253	0.000366	0.001673
11728462_DNLZ	-0.30898	0.000366	0.001673
11748708_SRCAP	-0.31093	0.000366	0.001673
11745397_KLHL8	0.341749	0.000366	0.001673
11721839_GLIPR1	0.296914	0.000367	0.001678
11758268_MEST	-0.27767	0.000367	0.001679
11720062_IER3	-0.27518	0.000367	0.001679
11729860_H6PD	-0.29806	0.000367	0.001679
11740005_KIAA0895	0.687609	0.000368	0.001682
11744801_NR2C2AP	-0.3426	0.000368	0.001684
11721180_FAM175B	0.239243	0.000369	0.001686
11719021_WDFY1	-0.28142	0.000369	0.001686
11726815_GPRIN1	-0.50127	0.000369	0.001686
11744237_ARHGEF19	-0.27262	0.000369	0.001687
11732382_USP6NL	0.375464	0.000369	0.001688
11754053_TPI1	-0.22049	0.00037	0.001689
11756878_FBL	-0.21831	0.00037	0.001691
11733354_RFESD	0.386327	0.00037	0.001692
11744730_DLEU2 ///	-0.26054	0.00037	0.001692
11741548_MBNL1	0.239237	0.000371	0.001692
11739673_PDP2	0.29057	0.000371	0.001692
11736868_MMP16	0.250852	0.000371	0.001692
11727680_ADSL	0.231045	0.000371	0.001692
11724186_SART3	-0.3373	0.000371	0.001692
11750165_FARSB	-0.22269	0.000371	0.001692

11716834_PRRC2A	-0.31651	0.000371	0.001693
11727458_KIF18A	-0.28858	0.000371	0.001693
11720957_IKBKE	0.420735	0.000371	0.001693
11751901_MON2	0.264339	0.000372	0.001695
11723028_MEST	-0.41692	0.000372	0.001695
11733328_CEP57L1	-0.44086	0.000372	0.001697
11744408_GOLGA8A	0.316496	0.000372	0.001698
11748317_MCFD2	0.249336	0.000372	0.001699
11739478_SBF2	0.255779	0.000373	0.001699
11727575_TMEM237	-0.36068	0.000373	0.0017
11716309_CYBRD1	0.283075	0.000373	0.0017
11763808_ATP6V0D1	0.224858	0.000373	0.001701
11751983_EGFR	0.335184	0.000373	0.001701
11743050_DNAJC15	0.205951	0.000373	0.001701
11757442_SERPINE2	0.205751	0.000374	0.001703
11740216_LMOD3	0.492037	0.000374	0.001703
11758445_EDNRB	0.310016	0.000374	0.001703
11764234_INTS7	-0.5949	0.000374	0.001703
11718844_BCL2L2-PA	-0.25878	0.000374	0.001705
11749610_CD58	0.236517	0.000374	0.001705
11738863_PTPN2	-0.22513	0.000375	0.001706
11737623_DSTYK	0.274453	0.000375	0.001706
11728180_CHIC1	0.349991	0.000375	0.001708
11740117_RNF24	0.232406	0.000375	0.001708
11717557_POM121 //	-0.26275	0.000377	0.001716
11739230_ARL4A	0.271121	0.000377	0.001717
11744737_RTKN2	-0.29432	0.000377	0.001718
11738008_APP	0.235287	0.000378	0.001719
11744098_NOP58	0.214977	0.000378	0.001719
11723080_C2orf47	0.214861	0.000378	0.00172
11716391_BACE1	0.249594	0.000378	0.001721
11747868_SDHA	-0.26254	0.000378	0.001721
11739801_VCPIP1	0.28828	0.000378	0.001721
11758007_YAF2	-0.32642	0.000378	0.001721
11740368_PTPN3	0.318088	0.000379	0.001722
11724746_CHMP2B	0.286188	0.000379	0.001722
11747999_TUBD1	-0.37614	0.000379	0.001724
11732153_ZDHHC6	0.226293	0.000379	0.001724
11759587_HTRA3	-0.45769	0.000379	0.001725
11725766_COL11A1	0.555432	0.000379	0.001725
11732334_SEC31A	0.255513	0.00038	0.001726
11756984_GNB2	-0.24262	0.00038	0.001727
11722276_ZBED4	-0.37879	0.00038	0.001729
11749858_AP4B1	0.327431	0.00038	0.001729
11764017_APH1A	-0.23057	0.000381	0.001729
11748402_HDAC6	-0.34723	0.000381	0.00173
11740630_ZNF780B	0.601015	0.000381	0.001732
11722574_AP1G1	-0.22785	0.000381	0.001733
11740156_SH2D1B	0.433274	0.000382	0.001734
11725483_UNC13A	0.407877	0.000382	0.001735
11746613_ 7-Mar	0.2387	0.000382	0.001735

11758135_C14orf2	0.216915	0.000382	0.001736
11723795_SLC29A2	-0.41145	0.000383	0.001737
11730836_ZDHHC4	-0.25961	0.000383	0.001738
11750587_CTNND1 //	0.287793	0.000383	0.001739
11750203_TRUB1	-0.41544	0.000383	0.001739
11739512_TIMM50	-0.25863	0.000383	0.001739
11722474_DGKQ	-0.26362	0.000384	0.001741
11721443_RNASEH2C	-0.24403	0.000384	0.001742
11718721_BRD8	-0.23385	0.000384	0.001742
11751207_MLX	0.23145	0.000384	0.001742
11759567_TMEM265	0.621303	0.000384	0.001742
11734696_GOLGA8A /	0.281956	0.000384	0.001744
11716539_SZRD1	-0.29934	0.000384	0.001744
11743745_CSPP1	0.257533	0.000385	0.001745
11726991_EIF2AK1	-0.29233	0.000385	0.001746
11761569_TMC6	-0.24782	0.000385	0.001747
11743854_FBXO21	-0.22663	0.000386	0.001748
11753682_LSM12	0.226721	0.000386	0.001748
11739288_KMT2A	0.414731	0.000386	0.001749
11751759_LSM12	0.239019	0.000386	0.001749
11746973_POR	-0.27787	0.000386	0.001749
11755693_LDLRAD3	-0.34156	0.000386	0.001749
11753624_ARL2 ///	-0.24101	0.000386	0.001749
11721015_WTAP	0.242694	0.000386	0.001751
11731605_HSD17B12	-0.23518	0.000386	0.001751
11735004_JADE1	-0.33396	0.000387	0.001752
11717361_GPC1	-0.30522	0.000387	0.001752
11731123_TMEM200A	0.605029	0.000387	0.001753
11717278_THRAP3	-0.21601	0.000387	0.001753
11728404_SHCBP1	-0.30194	0.000387	0.001753
11731593_STAM2	0.352496	0.000387	0.001754
11723234_IFI44L	0.42921	0.000387	0.001754
11730442_FAM155A	-0.27695	0.000387	0.001754
11717319_SEC24D	0.241346	0.000387	0.001754
11740559_NSMAF	0.30253	0.000387	0.001754
11733111_TLK1	0.380473	0.000388	0.001755
11739372_MAPRE2	0.288835	0.000388	0.001758
11744430_KIF9	-0.21979	0.000389	0.001758
11747334_DDX39A	-0.22931	0.000389	0.001759
11744366_ZFAS1	0.270657	0.000389	0.00176
11737331_PDE1C	0.262528	0.000389	0.00176
11716306_ATXN10	0.234975	0.000389	0.001761
11760820_IDS	0.48618	0.00039	0.001763
11718027_PUS1	0.310275	0.00039	0.001763
11759531_NUDT22	0.289229	0.00039	0.001764
11743724_MFSD1	0.230672	0.00039	0.001766
11720391_SLC39A10	0.270305	0.000391	0.001766
11755935_MYL5	0.223029	0.000391	0.001766
11754529_SLC3A2	0.235223	0.000391	0.001766
11754061_IFITM2	0.28716	0.000391	0.001767
11730338_SMAD6	0.303413	0.000391	0.001767

11729775_PCDHB7	0.229953	0.000391	0.001767
11733347_CNOT7	-0.23605	0.000391	0.001768
11747833_KLF11	-0.35708	0.000391	0.001768
11743830_PAM	0.213943	0.000391	0.001769
11715566_COPZ1	0.266653	0.000392	0.001771
11760081_CEACAM1	0.312834	0.000392	0.001772
11718796_PAQR4	-0.30916	0.000393	0.001777
11729894_CHRAC1	-0.27308	0.000393	0.001777
11729442_ENTHD2	0.314496	0.000393	0.001777
11757922_DDX52	0.304099	0.000394	0.001777
11737039_PHLDB2	0.533158	0.000394	0.001777
11721259_WDR47	0.227235	0.000394	0.001778
11723119_DSTYK	0.25084	0.000394	0.001779
11745435_ZBTB1	0.255874	0.000394	0.001779
11725523_DSG2	0.260313	0.000394	0.001779
11743347_CD47	-0.2346	0.000394	0.00178
11752182_DLG1	0.326963	0.000394	0.00178
11760237_CRELD2	-0.35831	0.000395	0.001781
11717107_PDLIM5	0.220705	0.000395	0.001781
11727044_TMEM173	0.33723	0.000395	0.001783
11731034_SPTY2D1	0.226357	0.000395	0.001783
11718200_KPNA3	-0.24547	0.000395	0.001783
11756861_RAET1G //	-0.29477	0.000395	0.001784
11744685_B4GALNT1	-0.26478	0.000396	0.001786
11718893_RFC1	-0.26509	0.000397	0.001788
11731602_TMEM154	-0.36393	0.000397	0.001788
11730948_MRPL52	0.362794	0.000397	0.001789
11732224_ZNF664	-0.22609	0.000397	0.001792
11753162_YIPF5	0.228127	0.000398	0.001792
11725879_UBR2	0.473694	0.000398	0.001792
11751761_INTS6	0.352148	0.000398	0.001793
11756872_SAMD4A	0.620491	0.000398	0.001794
11757700_NDFIP2	0.213307	0.000399	0.001798
11744525_MTERF2	-0.27068	0.000399	0.0018
11718345_CNOT7	-0.22741	0.0004	0.001801
11760564_SP140L	0.452742	0.0004	0.001801
11733217_USP53	0.23042	0.0004	0.001802
11758841_PARVA	0.219266	0.0004	0.001803
11731136_NCKAP5	0.335517	0.0004	0.001804
11717558_POM121 //	-0.26552	0.0004	0.001804
11749940_BACE1	0.319291	0.000401	0.001805
11758360_MRAS	0.245699	0.000401	0.001805
11733340_HDGFRP2	-0.37689	0.000401	0.001807
11746173_DDHD1	0.485672	0.000401	0.001807
11715892_PYCR2	-0.22983	0.000402	0.00181
11729998_B3GNT7	0.25664	0.000402	0.001811
11744570_MAN2C1	0.220775	0.000402	0.001811
11735610_RPS6KA5	0.228501	0.000402	0.001811
11715373_TRIM28	-0.29059	0.000403	0.001811
11725429_LAMP3	-0.51487	0.000403	0.001812
11744456_GSK3B	0.241556	0.000403	0.001812

11748647_PTPRR	0.907143	0.000403	0.001812
11743900_CIITA	0.295446	0.000403	0.001813
11731685_RIF1	0.318127	0.000403	0.001813
11739813_FZD1	-0.28869	0.000403	0.001813
11722725_CSF2RB	-0.31665	0.000403	0.001814
11719976_WASF3	0.275511	0.000403	0.001814
11763184_IDE	0.231989	0.000404	0.001816
11751475_DCTN4	0.243848	0.000404	0.001818
11719490_TMEM189	0.243742	0.000405	0.001819
11758117_LRRFIP2	0.244364	0.000405	0.001819
11737864_PIGF	-0.30132	0.000405	0.00182
11733287_CYP20A1	0.300424	0.000406	0.001823
11715521_MLEC	-0.22704	0.000406	0.001825
11761765_HOXA2	-0.43962	0.000407	0.001828
11724819_SH3KBP1	0.209511	0.000407	0.001829
11749818_ARFIP2	0.267329	0.000407	0.00183
11727834_WDR43	0.275973	0.000407	0.00183
11763297_TRMT13	0.27667	0.000408	0.001831
11753318_ST6GALNAC	0.27209	0.000408	0.001833
11737334_AMIGO2	0.236826	0.000409	0.001836
11717982_FBXL3	0.210638	0.000409	0.001838
11716536_SZRD1	0.337197	0.000409	0.001838
11740840_ABL2	0.254876	0.000409	0.001838
11721151_RAB40C	-0.34881	0.000409	0.001838
11757788_DDR2	-0.27591	0.000409	0.001838
11721399_SLIT2	0.327137	0.00041	0.001838
11716449_DDX56	0.210521	0.00041	0.001839
11724022_TRIM13	-0.26781	0.00041	0.001839
11746695_PAFAH1B1	0.209155	0.00041	0.00184
11739752_SLC25A16	0.381738	0.00041	0.00184
11744540_RTN3	-0.2179	0.00041	0.001841
11757794_PAPD5	0.228986	0.00041	0.001841
11733509_LIMK2	0.273723	0.00041	0.001841
11750764_ROBO4	-0.30669	0.00041	0.001841
11715696_NOLC1	-0.31096	0.00041	0.001841
11750021_SURF4	-0.22992	0.000411	0.001843
11730655_CNOT1	0.257717	0.000411	0.001844
11721196_SLC18B1	0.468406	0.000411	0.001844
11730940_ZBTB8A	0.214053	0.000411	0.001845
11728904_SATB2	0.304093	0.000412	0.001845
11723488_GNAZ	-0.34996	0.000412	0.001847
11726066_BUD13	-0.26694	0.000412	0.001847
11718052_TBC1D23	0.246195	0.000412	0.001847
11741802_KIAA1324L	0.293587	0.000413	0.001851
11718917_PAK4	-0.31137	0.000413	0.001852
11725518_FRAT2	0.288066	0.000413	0.001852
11739143_VPS39	0.241801	0.000413	0.001852
11720563_PLXNA2	0.502211	0.000414	0.001853
11739604_CCDC88A	-0.23387	0.000414	0.001854
11718306_GSE1	0.270166	0.000414	0.001854
11724821_NEK6	0.250169	0.000414	0.001855

11741207_ZNF207	0.214363	0.000414	0.001855
11744818_ZNF24	0.235382	0.000414	0.001856
11717347_ADD3	-0.22143	0.000414	0.001856
11746674_ZNF232	-0.27647	0.000414	0.001856
11740324_SLC6A2	-0.2584	0.000415	0.001856
11717788_AAR2	0.248594	0.000415	0.001857
11719612_LARP4	0.24297	0.000415	0.001857
11721040_TRAPPC11	0.334864	0.000415	0.001857
11723494_RNF166	-0.34893	0.000416	0.001861
11749601_LRTOMT	0.651521	0.000416	0.001862
11752435_EXT2	0.219075	0.000416	0.001862
11763592_TARID	-0.21482	0.000416	0.001862
11724059_TECPR2	0.320294	0.000416	0.001863
11743797_ADM5	-0.27216	0.000417	0.001865
11723005_WASF1	0.279127	0.000417	0.001866
11723646_TAF15	0.214065	0.000417	0.001867
11727388_RSU1	-0.35125	0.000418	0.001868
11743282_SULF1	0.442824	0.000418	0.001868
11716540_SZRD1	0.27728	0.000418	0.001868
11717167_KDM2A	-0.51586	0.000418	0.001869
11719724_CCDC93	0.476369	0.000418	0.001869
11758867_WDR33	0.512125	0.000419	0.001871
11747666_WARS	-0.27127	0.000419	0.001871
11723238_GNG7	-0.27217	0.000419	0.001873
11758168_MAGEF1	-0.26858	0.000419	0.001873
11746127_CLPTM1	0.201696	0.000419	0.001873
11748533_IST1	0.231959	0.00042	0.001877
11715393_C3	0.474747	0.00042	0.001877
11748905_CTHRC1	0.228601	0.00042	0.001878
11756446_PITHD1	-0.23756	0.00042	0.001878
11742882_PPID	0.239573	0.000421	0.001879
11737181_UHMK1	0.252893	0.000421	0.001881
11728732_STRA13	-0.24383	0.000421	0.001882
11733730_DPH1 ///	0.254879	0.000422	0.001882
11752887_EHD1	0.321424	0.000422	0.001883
11728819_HAUS2	-0.25445	0.000422	0.001884
11715790_MRPL37	-0.21847	0.000422	0.001885
11736699_BMP8B	-0.49643	0.000424	0.001892
11722734_APPL1	-0.32212	0.000425	0.001895
11725587_RAB36	-0.37188	0.000425	0.001895
11742209_PDE8A	0.290067	0.000425	0.001895
11730991_EIF2AK2	0.227278	0.000425	0.001895
11733495_COMMD6	0.20651	0.000425	0.001895
11725137_SYNGR1	-0.20762	0.000426	0.001899
11744007_PBRM1	-0.22778	0.000426	0.0019
11751746_GAMT	-0.22245	0.000426	0.0019
11722581_LOC100630	0.311558	0.000426	0.0019
11757799_VAMP7	0.236135	0.000426	0.001901
11745154_NCL	-0.20546	0.000427	0.001902
11719510_HELZ	-0.34285	0.000427	0.001902
11718607_DESI1	0.274257	0.000427	0.001903

11743647_HMGN4	-0.22158	0.000427	0.001904
11729387_RPL31	-0.33149	0.000427	0.001905
11733844_AMACR ///	0.2572	0.000427	0.001905
11742724_STAT6	-0.31075	0.000428	0.001907
11729308_CLMP	-0.4085	0.000428	0.001908
11720664_GNA11	-0.25387	0.000428	0.001908
11761552_KATNBL1	-0.35971	0.000428	0.001908
11734955_SCML1	0.289559	0.000429	0.001909
11763529_SLC12A4	0.344471	0.000429	0.001909
11739769_TMOD2	0.663852	0.000429	0.00191
11718195_ATP6V0D1	0.215939	0.000429	0.001911
11718083_ATP2B4	-0.25069	0.000429	0.001912
11729843_VNN1	0.351565	0.00043	0.001913
11758508_ZNF548	0.382573	0.00043	0.001913
11755521_CTSV	-0.74452	0.00043	0.001913
11757518_COX14	-0.21636	0.00043	0.001914
11730712_ADCK1	-0.22307	0.00043	0.001915
11751034_NLE1	-0.23353	0.000431	0.001916
11746468_DARS	-0.25759	0.000431	0.001916
11717722_ASAH1	0.216618	0.000431	0.001917
11720583_TCF12	0.228479	0.000431	0.001919
11755477_METAP2	-0.28345	0.000431	0.001919
11736793_NXT2	-0.44792	0.000432	0.001921
11754836_ZNF232	-0.21571	0.000432	0.001922
11720603_STOM	0.290404	0.000433	0.001923
11726371_EXOSC9	-0.28673	0.000433	0.001923
11740473_CLIP1	0.236182	0.000433	0.001924
11716141_SIGMAR1	-0.23008	0.000433	0.001924
11716944_YIPF3	0.224002	0.000433	0.001925
11757261_SCARNA14	0.305554	0.000433	0.001926
11743722_MARCKSL1	-0.23291	0.000433	0.001926
11740624_CEP131	-0.30874	0.000434	0.001928
11716041_PPP2CB	-0.24891	0.000434	0.001929
11749978_MVP	0.296028	0.000434	0.00193
11716432_MNAT1	0.244167	0.000434	0.00193
11725802_USP46	0.377021	0.000434	0.00193
11740259_STXBP4	-0.21289	0.000434	0.00193
11722176_SF3B3	-0.24732	0.000435	0.00193
11719422_ABCC1	0.212604	0.000435	0.001931
11735168_QPCTL	-0.23414	0.000435	0.001932
11737143_CAST	-0.2528	0.000436	0.001935
11756408_LMBR1	0.376683	0.000436	0.001936
11758959_EMSY	-0.35612	0.000436	0.001938
11722798_TMEM263	0.234545	0.000437	0.001938
11743960_SLC16A3	0.236658	0.000437	0.001938
11748155_ZNF670-ZN	-0.42654	0.000437	0.001939
11725709_WDHD1	-0.63518	0.000437	0.00194
11753268_GALE	0.221613	0.000437	0.001941
11758811_HNRNPA1	-0.3573	0.000437	0.001941
11751982_CNTNAP3 /	0.329256	0.000437	0.001941
11759545_PRMT2	-0.37779	0.000437	0.001941

11729278_CRCP	0.243045	0.000437	0.001941
11743420_ZKSCAN5	0.416528	0.000438	0.001941
11732314_ANKRD13C	0.384158	0.000438	0.001941
11721355_YPEL3	0.241156	0.000438	0.001944
11730612_MBOAT7	-0.27006	0.000439	0.001946
11722435_GOSR2	0.274456	0.000439	0.001948
11750387_RTN3	-0.22126	0.000439	0.001948
11717439_FMO4 ///	0.250494	0.000439	0.001949
11739755_ZNF74	0.271145	0.00044	0.00195
11744091_ATXN7L3B	0.284193	0.00044	0.00195
11717984_PITPNB	0.247528	0.00044	0.001951
11722109_PCNX	-0.52147	0.000441	0.001953
11719134_COMMD9	-0.28241	0.000441	0.001954
11762530_FERMT1	-0.33713	0.000441	0.001954
11720098_CEP70	-0.28405	0.000441	0.001955
11721978_PLCE1	-0.33735	0.000441	0.001955
11722733_APPL1	-0.27138	0.000442	0.001957
11734941_FAM212B /	0.286281	0.000442	0.001958
11745719_HMG20A	0.353012	0.000443	0.001961
11758707_SIMC1	-0.21626	0.000443	0.001961
11716817_ENOPH1	-0.27533	0.000443	0.001962
11725606_TRIM9	0.541693	0.000443	0.001963
11742726_TMEM87A	0.197489	0.000443	0.001964
11722755_SHPRH	0.369974	0.000444	0.001964
11762344_RPS18	0.338982	0.000444	0.001966
11727445_TMEM255A	0.616729	0.000444	0.001967
11743782_ANKRD55	0.681105	0.000445	0.001967
11755121_OSCP1	0.452936	0.000445	0.001967
11735001_ST7L	0.55144	0.000445	0.001967
200046_PM DAD1	-0.24323	0.000445	0.001967
11745782_ZNF540	0.600219	0.000445	0.001969
11715889_ATP5I	-0.22714	0.000445	0.001969
11722989_BTAF1	0.318048	0.000445	0.001969
11755346_ATP5O	-0.23701	0.000445	0.001969
11747980_MAP4	-0.23381	0.000445	0.00197
11744866_CEL	0.316633	0.000446	0.001973
11741107_MRPS14	-0.23003	0.000446	0.001974
11756395_AP1S2	0.280372	0.000446	0.001974
11760500_ZNF451	0.411727	0.000447	0.001976
11763863_IP6K2	0.347996	0.000447	0.001976
11734958_PCNXL4	0.402854	0.000448	0.00198
11724432_TRAPPC2	-0.31376	0.000448	0.001981
11756712_ACP2	-0.26287	0.000448	0.001982
11751037_WDR48	0.34523	0.000449	0.001985
11717381_FOPNL	-0.25284	0.000449	0.001986
11731437_MED6	0.220275	0.00045	0.001986
11719152_LDOC1L	0.303799	0.00045	0.001987
11720467_DPAGT1	-0.25409	0.00045	0.001987
11740472_CLIP1	0.366229	0.00045	0.001987
11725475_ZNF354A	0.267148	0.00045	0.001988
11734717_CBLL1	0.214151	0.00045	0.001989

11722024_ABCF2	0.273056	0.000451	0.001991
11743042_STIP1	-0.26484	0.000451	0.001991
11721285_TBPL1	0.250368	0.000451	0.001992
11727835_EVL	-0.33101	0.000451	0.001992
11763248_EIF4EBP1	-0.20861	0.000452	0.001994
11740422_HTR7	0.304418	0.000452	0.001996
11740208_GPR155	-0.63902	0.000452	0.001997
11746326_CHFR	0.421317	0.000453	0.001997
11753405_NEK6	0.211225	0.000453	0.001997
11730570_DIP2A	-0.32831	0.000453	0.001998
11733401_TMEM201	-0.34299	0.000453	0.002
11750215_SLC39A1	-0.31352	0.000453	0.002
11725928_NEFL	0.270019	0.000454	0.002
11744383_CLTA	0.256708	0.000454	0.002
11716592_BTBD1	0.278612	0.000454	0.002002
11737854_P2RY1	-0.23716	0.000455	0.002005
11756432_TMEM41A	0.230111	0.000455	0.002005
11723015_EXT2	0.235945	0.000455	0.002005
11754995_PCBP2	-0.29829	0.000455	0.002005
11733025_CLCA2	0.623138	0.000455	0.002005
11733408_PXDNL	0.230575	0.000455	0.002007
11752344_STAC2	-0.36667	0.000455	0.002008
11733308_PHF12	-0.2214	0.000456	0.002008
11743727_ZNF207	0.220407	0.000456	0.002008
11737872_ZCCHC9	0.238352	0.000456	0.002012
11716370_FAM89B	-0.22679	0.000457	0.002012
11760424_TACC3	-0.44026	0.000457	0.002012
11743310_WIPI2	0.212458	0.000457	0.002013
11716730_ERH	-0.20149	0.000458	0.002016
11723828_ANO1	-0.27657	0.000458	0.002017
11755133_SNHG7	0.231735	0.000458	0.002017
11726945_PPM1A	-0.31666	0.000458	0.002017
11741762_C14orf93	-0.30805	0.000458	0.002018
11748634_ZCCHC24	-0.25595	0.000458	0.002018
11757981_WASF3	0.298879	0.000458	0.002018
11749436_NFIC	-0.21622	0.000458	0.002018
11739867_GPR107	0.368663	0.000459	0.002019
11726286_WARS	-0.27495	0.000459	0.002019
11743613_TMOD3	0.335229	0.000459	0.002019
11731671_FUNDC1	-0.23046	0.00046	0.002022
11727980_SNX21	-0.27205	0.00046	0.002025
11736365_KIAA0586	-0.28445	0.00046	0.002025
11735538_EN2	0.295088	0.00046	0.002026
11716233_CTNNBIP1	0.271263	0.000461	0.002028
11715225_VPS54	0.258401	0.000461	0.002029
11720516_ZBTB4	-0.20077	0.000461	0.00203
11750223_APOA1BP	-0.20603	0.000462	0.002031
11754018_PRDX3	-0.20291	0.000462	0.002032
11730594_EBLN2	-0.23674	0.000463	0.002038
11736564_DOCK5	0.561582	0.000464	0.002038
11743097_ITPK1	-0.32005	0.000464	0.002038

11717641_UBE3A	0.211959	0.000464	0.002038
11763183_RQCD1	0.249716	0.000464	0.002038
11747697_CLPTM1	0.220785	0.000464	0.002039
11730914_ERLIN1	0.265218	0.000464	0.002041
11715580_TBCB	-0.23071	0.000464	0.002041
11717023_SDF2	0.237079	0.000465	0.002042
11726694_C1orf35	-0.21492	0.000465	0.002042
11753879_IL6ST	0.263913	0.000465	0.002043
11733530_C1RL	-0.24925	0.000465	0.002044
11741433_ARCN1	0.226661	0.000465	0.002044
11718459_SLC39A11	0.24598	0.000466	0.002046
11753510_PIBF1	0.365337	0.000466	0.002046
11739028_CLTC	0.212684	0.000466	0.002048
11742113_ABL2	0.50636	0.000467	0.002048
11752464_SLC1A3	0.37516	0.000467	0.00205
11739996_SMARCA2	0.238691	0.000467	0.00205
11715821_SYPL1	0.213187	0.000467	0.002051
11753740_CYB5A	-0.24191	0.000467	0.002051
11717601_SECISBP2	0.265166	0.000467	0.002051
11748728_CDC14B	0.253682	0.000468	0.002052
11725317_VAPA	0.210931	0.000468	0.002054
11717845_CDK7	0.217313	0.000469	0.002056
11722219_WBP4	0.268345	0.000469	0.002056
11736606_TAF5L	0.269742	0.000469	0.002058
11754076_ANP32A	-0.24721	0.000469	0.002058
11743285_GOLT1B	-0.2312	0.000469	0.002058
11736512_NMT2	0.425214	0.000469	0.002058
11730650_ANO3	0.501965	0.000469	0.002058
11715304_PLCB3	-0.23851	0.00047	0.00206
11730237_ABHD10	-0.23544	0.00047	0.002062
11734960_TRHDE	0.271544	0.00047	0.002062
11742144_RHCE	-0.43782	0.00047	0.002062
11756679_ALDH3A2	-0.2331	0.00047	0.002062
11720758_IVNS1ABP	0.209901	0.00047	0.002062
11716455_RHOBTB3	0.231698	0.000471	0.002062
11757869_AKAP13	0.199695	0.000471	0.002062
11725723_SDCCAG8	0.243477	0.000471	0.002062
11715211_ZNF680	0.376256	0.000471	0.002062
11723117_PCNT	-0.24504	0.000471	0.002062
11730613_MBOAT7	-0.26433	0.000471	0.002065
11759907_ITGBL1	0.283207	0.000472	0.002066
11720548_HNRNPUL2	-0.27891	0.000473	0.002072
11730818_CTDSPL2	0.27577	0.000473	0.002073
11749114_DLG1	0.248424	0.000474	0.002074
11735434_GPR158	0.36154	0.000474	0.002075
11720614_CLNS1A	-0.24177	0.000474	0.002077
11719895_FAM91A1	0.194768	0.000474	0.002077
11723171_IL17RD	0.299893	0.000475	0.002078
11754973_MANSC1	0.349254	0.000475	0.002079
11745238_TMEM218	-0.2813	0.000476	0.002081
11719711_PDPK1	0.37048	0.000476	0.002081

11756591_MBNL1	0.295686	0.000476	0.002081
11725499_GOLGA7B	0.319393	0.000476	0.002081
11739821_ZNF714	-0.46706	0.000476	0.002081
11729349_MMP8	0.311745	0.000476	0.002081
11755409_EP400	-0.32391	0.000476	0.002082
11743153_C16orf72	-0.30938	0.000476	0.002082
11731864_NKX2-2	-0.32184	0.000477	0.002084
11736727_GEN1	-0.31095	0.000477	0.002085
11717761_G6PC3	-0.21575	0.000477	0.002087
11726723_WDFY2	0.346879	0.000478	0.002088
11730106_PLCB1	0.285476	0.000478	0.002088
11758155_ARHGAP10	0.428763	0.000478	0.002089
11720686_CDR2L	-0.23924	0.000478	0.002091
11715407_PSMB4	-0.21126	0.000479	0.002092
11744882_PPP1R21	0.37418	0.000479	0.002092
11728350_STIM2	0.208499	0.000479	0.002094
11720005_NPTX2	0.325664	0.000479	0.002094
11761590_GTF3C3	-0.45786	0.00048	0.002095
11735518_HES7	-0.21707	0.00048	0.002096
11733255_CHKB-CPT1	0.379427	0.00048	0.002096
11724000_ZNF581	0.210482	0.000481	0.0021
11717321_OAF	-0.21142	0.000481	0.0021
11757752_STK36	0.224318	0.000481	0.0021
11745133_FYCO1	-0.39809	0.000481	0.002101
11737217_ZNF124	0.213748	0.000482	0.002103
11718978_CAPN2	0.20958	0.000482	0.002104
11746006_VPS52	-0.21679	0.000482	0.002105
11755308_TMEM189	0.213523	0.000482	0.002106
11745775_LIPA	-0.31185	0.000483	0.002106
11743146_FBXO11	0.222572	0.000483	0.002108
11716708_DDR1	-0.26872	0.000483	0.002108
11741394_EXOG	0.474448	0.000483	0.002109
11722524_TMCO4	-0.26221	0.000484	0.00211
11748502_IFT122	0.3052	0.000484	0.002113
11749312_HS6ST2	-0.30847	0.000484	0.002113
11722081_RUFY3	0.261347	0.000485	0.002115
11716594_BTBD1	0.23676	0.000486	0.002119
11718548_ILF3	-0.22762	0.000486	0.00212
11722535_CCNYL1	0.242475	0.000486	0.00212
11763296_ZNF85	-0.43159	0.000486	0.002121
11721756_DENND2A	-0.33227	0.000487	0.002122
11747675_SUN1	0.261321	0.000487	0.002122
11753957_DHPS	-0.26547	0.000487	0.002125
11749267_SRD5A1	-0.29361	0.000487	0.002125
11741043_MBLAC2	-0.30025	0.000488	0.002125
11756778_NEFL	0.220578	0.000488	0.002127
11725755_BTN2A2	0.54611	0.000488	0.002128
11754761_HMGN1	-0.20933	0.000489	0.00213
11730391_SEMA3A	0.238724	0.000489	0.002131
11719803_UBL4A	-0.26097	0.000489	0.002131
11748202_PTK7	-0.29526	0.000489	0.002131

11734454_WFDC3	0.213398	0.000489	0.002132
11718334_GRB2	0.22674	0.00049	0.002133
11758111_FOXC1	-0.62157	0.00049	0.002133
11749781_BCAT1	0.297502	0.00049	0.002133
11756650_CHP1	0.22381	0.00049	0.002134
11732706_FGFRL1	-0.24117	0.000491	0.002138
11737156_TMEM241	-0.31936	0.000491	0.002139
11740410_RNF24	0.292436	0.000491	0.002139
11752165_PKM	-0.21864	0.000491	0.002139
11723738_WDR36	0.27282	0.000492	0.00214
11741219_EDARADD	-0.27736	0.000492	0.00214
11737896_TAF13	0.38018	0.000492	0.002143
11732859_DNHD1	0.532777	0.000493	0.002143
11726713_COG1	0.361733	0.000493	0.002144
11720555_TSEN15	-0.29128	0.000493	0.002145
11759759_CCNL1	-0.31286	0.000493	0.002147
11733792_SLC25A12	0.266601	0.000493	0.002147
11729289_BMX	0.499776	0.000494	0.002147
11752814_TNFRSF1A	0.297304	0.000494	0.002148
11728264_NEK4	0.216989	0.000495	0.00215
11744350_RPS27L	0.210109	0.000495	0.002151
11729410_ZNF415	0.489348	0.000495	0.002151
11748729_CDC14B //	0.252758	0.000495	0.002151
11718802_PSRC1	-0.20549	0.000495	0.002151
11740999_GAP43	0.354871	0.000495	0.002152
11736830_MAP3K7	0.224459	0.000496	0.002156
11718480_MID1IP1	-0.29127	0.000496	0.002156
11724389_ZDHHC16	0.214833	0.000496	0.002157
11758694_RAB28	-0.25379	0.000497	0.002158
11727097_NT5C1B-RD	0.214537	0.000497	0.002161
11752240_ANKRD28	0.279616	0.000497	0.002161
11756675_SLC25A1	-0.26171	0.000498	0.002162
11717620_LSS	-0.24275	0.000499	0.002168
11728800_FBXL4	0.317092	0.000499	0.002169
11720698_RUFY1	0.230562	0.0005	0.00217
11731710_ZNF160	0.275808	0.0005	0.00217
11757293_MRPL18	-0.27564	0.0005	0.00217
11758594_CSTF2T	-0.24015	0.0005	0.00217
11754513_SNHG11 //	0.294985	0.0005	0.002171
11723329_GSN	0.22755	0.0005	0.002172
11719492_IFI35	-0.24428	0.000501	0.002174
11728000_CERS1 ///	-0.26478	0.000501	0.002174
11742875_C18orf32	0.295752	0.000501	0.002175
11723074_BCL9L	0.242027	0.000502	0.002176
11737247_ZNF141	0.235006	0.000502	0.002176
11758620_MMAB	-0.53669	0.000502	0.002177
11749525_AKT2	-0.34586	0.000503	0.00218
11745387_KIAA1217	0.315343	0.000503	0.002181
11740984_NRDC	0.205137	0.000503	0.002181
11727058_MRPS36	-0.46209	0.000503	0.002181
11732906_EDNRB	0.536692	0.000503	0.002182

11743489_C14orf93	-0.33409	0.000503	0.002182
11750815_DDX5	0.20364	0.000503	0.002183
11739729_RECQL	-0.2848	0.000504	0.002184
11745131_ZNF763	0.323252	0.000504	0.002184
11743080_BBS7	0.231391	0.000504	0.002184
11757500_CTDSPL	-0.43209	0.000504	0.002184
11758888_MUT	-0.50474	0.000504	0.002186
11723160_ZNF776	0.667583	0.000504	0.002186
11717653_GIT1	-0.2513	0.000505	0.002187
11721111_ACOT13	-0.25458	0.000505	0.002187
11746770_RWDD4	-0.28824	0.000505	0.002187
11741570_UBE2Q2	0.272099	0.000505	0.002187
11719271_GUSB	-0.23691	0.000505	0.002187
11741143_NOX4	0.411817	0.000505	0.002187
11732947_MED23	0.311171	0.000505	0.002187
11725971_HNRNPF	-0.45446	0.000505	0.002187
11726432_OSBPL3	-0.29457	0.000505	0.002188
11759289_AKT3	-0.34848	0.000505	0.002189
11755723_CFHR3 ///	0.356134	0.000506	0.002189
11722295_PCF11	-0.24912	0.000506	0.002191
11717070_KTN1	0.210078	0.000506	0.002191
11716074_HSBP1	-0.28392	0.000507	0.002193
11760011_LINC00656	-0.23755	0.000508	0.002198
11745739_SCOC	-0.24261	0.000508	0.002198
11718300_RPP25	-0.22891	0.000509	0.002202
11722577_P3H1	0.220406	0.000509	0.002202
11757410_C6orf106	-0.25318	0.000509	0.002202
11743041_STIP1	-0.24442	0.000509	0.002204
11717697_ARIH1	-0.28642	0.00051	0.002204
11727803_GJA9-MYCB	0.252203	0.00051	0.002205
11750185_CCDC71L	0.263124	0.00051	0.002206
11734797_HIST1H2BG	0.3712	0.00051	0.002206
11744819_PAAF1	-0.2301	0.00051	0.002206
11754339_NADSYN1	0.311928	0.000511	0.002207
11741375_CADM1	0.212069	0.000511	0.002209
11736884_STXBP5	0.386667	0.000511	0.002209
11716575_CCDC6	0.395957	0.000511	0.002209
11730935_AKNA	-0.49202	0.000511	0.002209
11741658_SYDE1	0.254117	0.000511	0.00221
11756526_MRPL48	-0.21364	0.000512	0.002213
11735583_NIN	-0.48733	0.000512	0.002213
11750307_SHTN1	0.367464	0.000512	0.002214
11716356_TMEM167A	0.253827	0.000513	0.002214
11733707_COL11A1	0.475405	0.000513	0.002214
11726989_EIF2AK1	-0.22551	0.000513	0.002214
11742128_DEGS1	0.201271	0.000513	0.002215
11756396_PTOV1	-0.24326	0.000513	0.002216
11726140_SIRT1	-0.23959	0.000513	0.002216
11749518_SRD5A1	-0.28385	0.000514	0.002218
11720129_COMTD1	-0.28289	0.000514	0.002219
11725350_KIAA0930	-0.27428	0.000514	0.00222

11743670_ZNF292	0.255732	0.000515	0.002221
11715572_CLSTN1	-0.1997	0.000515	0.002222
11719001_TADA3	-0.1954	0.000515	0.002224
11759004_SLC33A1	-0.42238	0.000516	0.002226
11748651_PMS1	-0.25655	0.000517	0.00223
11754373_MARK3	0.303764	0.000517	0.00223
11733470_CD300LB	-0.24069	0.000518	0.002234
11717475_CHGB	0.931297	0.000518	0.002234
11717175_ABL1	-0.35539	0.000518	0.002234
11753308_RAET1G //	-0.30213	0.000518	0.002236
11747710_SMARCD2	-0.22393	0.000519	0.00224
11759694_LOC145783	-0.48936	0.00052	0.00224
11750276_EHD1	0.3167	0.00052	0.00224
11722592_BCAP29	-0.38084	0.00052	0.002241
11756508_TAF6	-0.21322	0.00052	0.002242
11725401_C11orf57	-0.2594	0.00052	0.002244
11728661_GABARAPL2	0.247824	0.000521	0.002244
11721440_TOR1AIP1	-0.2121	0.000521	0.002245
11737775_NOP2	0.229853	0.000521	0.002245
11740627_ANKRD30A	-0.23281	0.000521	0.002246
11727370_TSN	-0.28875	0.000522	0.002248
11730069_USP33	0.226494	0.000522	0.002248
11743212_PTTG2	-0.47169	0.000522	0.002248
11752197_ENG	0.220141	0.000522	0.002249
11739527_SECTM1	0.212798	0.000523	0.002251
11742700_C1R	-0.35887	0.000523	0.002252
11736019_NOP2	0.226934	0.000523	0.002252
11730134_ICK	0.232879	0.000523	0.002252
11751621_SPOCK3	-0.25223	0.000523	0.002252
11728752_OSBP	-0.30251	0.000523	0.002253
11745943_GUCD1	-0.2608	0.000524	0.002254
11746705_RGS16	-0.26214	0.000524	0.002255
11748700_TOMM34	-0.27236	0.000524	0.002256
11733734_AAMP	-0.43159	0.000524	0.002256
11764039_MAPRE2	-0.21795	0.000524	0.002256
11741846_RAPH1	0.25398	0.000524	0.002257
11719945_RARG	-0.47613	0.000525	0.002257
11718238_CAPRIN1	0.300362	0.000526	0.002262
11755682_POU6F1	-0.32025	0.000527	0.002265
11744559_SLC25A37	0.2293	0.000527	0.002269
11754840_LINC00470	-0.3548	0.000527	0.002269
11733784_PPP2R1B	0.398671	0.000528	0.002269
11758491_AGPS	-0.37714	0.000528	0.002269
11717168_PER1	0.288331	0.000528	0.002269
11749289_CHMP1A	-0.23419	0.000528	0.002269
11731331_ZIC1	-0.36134	0.000528	0.00227
11721536_SURF1	0.251139	0.000528	0.002271
11727861_TIMM8A	0.282517	0.000528	0.002272
11718533_SIK3	0.307065	0.000529	0.002272
11758377_TLR1	0.397394	0.000529	0.002272
11754288_LMAN2L	0.248849	0.000529	0.002273

11739910_ELOVL6	0.228322	0.00053	0.002275
11751183_EWSAT1	0.310485	0.00053	0.002275
11747865_EIF3C ///	-0.20583	0.00053	0.002276
11762444_TRPV3	-0.30442	0.00053	0.002278
11747586_MCM3AP	0.253907	0.000531	0.002282
11751523_TMED5	0.42803	0.000531	0.002282
11721292_GIT2	0.227573	0.000531	0.002283
11746236_LIN37	0.252518	0.000532	0.002286
11734852_KIAA0226L	0.581254	0.000533	0.002288
11741133_HNRNPF	-0.22739	0.000534	0.002293
11726376_MPV17L2	-0.20349	0.000534	0.002294
11757940_PGF	0.325046	0.000535	0.002295
11754093_HSPBP1	-0.25685	0.000535	0.002296
11724268_TMEM185A	-0.37753	0.000535	0.002296
11757669_PPIL1	-0.20166	0.000535	0.002297
11745834_ECT2	-0.31365	0.000535	0.002298
11743866_MPHOSPH10	0.216965	0.000536	0.002299
11748649_VPS13C	0.418804	0.000536	0.0023
11740748_MAX	0.238243	0.000536	0.0023
11722537_MSANTD3-T	0.243731	0.000537	0.002303
11754823_KIAA0226L	0.74492	0.000537	0.002303
11755451_PQLC2	0.343101	0.000537	0.002303
11727939_SLC45A3	-0.33624	0.000537	0.002304
11754030_PABPC4	0.206801	0.000537	0.002304
11722896_PIK3C2A	0.398899	0.000537	0.002304
11723405_SCMH1	-0.35287	0.000537	0.002304
11715120_HIST1H2BE	-0.29197	0.000538	0.002306
11746914_ACAD8	0.372371	0.000538	0.002306
11720183_EEF1B2	0.227858	0.000538	0.002307
11757701_VAMP4	0.197643	0.000538	0.002307
11759532_FIP1L1	-0.6548	0.000539	0.002309
11751220_BLMH	-0.25611	0.000539	0.002309
11751367_EDEM1	0.584034	0.000539	0.002312
11718348_TUSC2	0.244511	0.00054	0.002312
11732057_CDC7	-0.25272	0.00054	0.002312
11730942_OSCAR	-0.24283	0.00054	0.002313
11757684_TPD52L2	-0.22844	0.00054	0.002315
AFFX-HUMISTAT1	0.288242	0.00054	0.002315
11754420_PREPL	0.233431	0.00054	0.002315
11740441_APOBEC3A	-0.22011	0.000541	0.002315
11757986_MIS12	-0.26733	0.000541	0.002316
11722696_SFI1	-0.29638	0.000541	0.002316
11758275_EIF5	-0.20561	0.000541	0.002316
11721581_CAMKK1	-0.22389	0.000542	0.002319
11744843_PAPPA	0.570816	0.000542	0.00232
11756886_TSC2	0.296403	0.000542	0.002321
11720134_PROS1	0.34713	0.000542	0.002321
11722709_CCSER2	0.222584	0.000542	0.002322
11740513_PDE4D	0.386733	0.000543	0.002324
11741065_EPHX1	-0.31369	0.000543	0.002325
11715544_TMED10	0.274433	0.000543	0.002325

11715430_AKR1B1	-0.22773	0.000544	0.002326
11745964_ALDOA	-0.19681	0.000544	0.002326
11742061_CHORDC1	-0.28955	0.000544	0.002327
11717090_HIST2H2BE	0.715182	0.000544	0.002327
11756025_S100A13	0.209279	0.000544	0.002327
11746321_SERPINH1	-0.24994	0.000544	0.002327
11746720_PPP1CB	0.309518	0.000544	0.002328
11760038_GLS	0.320012	0.000545	0.002328
11719030_TSPYL2	-0.23842	0.000545	0.00233
11717446_DHPS	-0.27537	0.000546	0.002332
11741044_PLS1	0.280382	0.000546	0.002333
11746260_PIGA	0.263253	0.000546	0.002335
11740256_GJC1	-0.27581	0.000546	0.002335
11742846_CUL4B	0.252602	0.000546	0.002335
11757532_RBM47	-0.40367	0.000547	0.002335
11760110_GSDMD	-0.30544	0.000547	0.002336
11719654_SV2A	-0.32137	0.000547	0.002336
11728030_IGSF10	0.513849	0.000547	0.002337
11728701_CD55	0.213211	0.000547	0.002337
11757085_ASAH2 ///	0.297642	0.000547	0.002337
11744563_FMR1	0.258526	0.000548	0.002338
11727214_DTWD1	0.253353	0.000548	0.002339
11720171_MRPS16	-0.23487	0.000548	0.00234
11753044_USP25	0.411707	0.000548	0.002341
11743991_MRGBP	-0.34238	0.000549	0.002342
11729074_HYKK	-0.28018	0.000549	0.002345
11756298_SNRPA	-0.24067	0.000549	0.002345
11725825_GJB3	-0.34448	0.000549	0.002345
11748259_CUEDC1	0.213657	0.00055	0.002345
11755385_BTBD2	-0.24398	0.00055	0.002346
11723145_RANBP17	0.440244	0.00055	0.002346
11720086_KIAA0368	-0.22656	0.00055	0.002346
11717137_CDC42EP4	-0.40665	0.00055	0.002346
11730130_HOXD10	0.257501	0.00055	0.002348
11736222_FOXRED2	-0.2458	0.00055	0.002348
11756426_TRIM41	0.225036	0.000551	0.002348
11755433_ARHGEF1 /	-0.20104	0.000551	0.002348
11741885_CASP1	0.557072	0.000551	0.002348
11739164_PYCRL	-0.21762	0.000551	0.002349
11739820_ZNF714	-0.6071	0.000551	0.002349
11724757_GPBP1L1	0.227926	0.000551	0.002351
11746991_ATP1B1	0.299946	0.000552	0.002352
11733601_NPAS4	-0.31268	0.000552	0.002352
11734725_PNPT1	0.220715	0.000552	0.002354
11723458_RBM48	0.441766	0.000552	0.002354
11756245_ANXA5	0.201947	0.000553	0.002355
11741591_ABCD3	0.266902	0.000553	0.002356
11725685_PTPRO	0.612646	0.000553	0.002356
11741381_ARNT	0.286812	0.000553	0.002358
11758990_SLC9A3R1	-0.24504	0.000554	0.002358
11730304_FANCA	-0.29756	0.000554	0.002359

11743432_C17orf100	-0.29589	0.000554	0.00236
11759254_TNFSF18	0.617255	0.000554	0.00236
11719026_PSD3	0.348143	0.000554	0.00236
11759597_HSPA14	0.224778	0.000555	0.002362
11743121_PRKAR1B	-0.30522	0.000555	0.002362
11746441_TGFBR1	0.58201	0.000555	0.002363
11731139_KLHL8	0.245007	0.000555	0.002363
11719580_HIGD2A	-0.20521	0.000555	0.002363
11719692_RARRES3	0.423514	0.000555	0.002363
11722589_BCAP29	0.232568	0.000555	0.002364
11734056_PTGR2	-0.30721	0.000556	0.002364
11716975_PDK4	0.381247	0.000556	0.002365
11717990_SIDT2	0.302067	0.000556	0.002367
11715101_HIST1H3G	-0.22773	0.000557	0.002368
11757524_CYC1	-0.20089	0.000557	0.002369
11748667_SDCCAG8	0.256856	0.000557	0.00237
11753979_RAB13	-0.28597	0.000557	0.002371
11722888_EMB /// E	-0.23421	0.000557	0.002371
11732704_FGFRL1	-0.277	0.000558	0.002371
11734900_UCK1	-0.23389	0.000558	0.002372
11731686_RIF1	0.268797	0.000558	0.002373
11717395_ARIH2	0.235434	0.000558	0.002373
11716789_TARS	0.195687	0.000558	0.002374
11747356_FSCN1	-0.23779	0.000559	0.002377
11742813_SEMA6A	-0.32587	0.000559	0.002377
11718441_RAVER1	-0.32058	0.00056	0.002379
11743961_CNST	0.396319	0.00056	0.002381
11749237_MRPS27	0.237863	0.00056	0.002381
11748068_CCPG1 ///	0.216984	0.000561	0.002383
11716428_PMF1	-0.27287	0.000562	0.002387
11720321_AK2	-0.23072	0.000562	0.002387
11754451_SON	0.204006	0.000562	0.002388
11725992_SNX11	0.295724	0.000563	0.002389
11727749_NR5A2	0.301847	0.000564	0.002394
11724986_CCDC113	0.376873	0.000565	0.002398
11722578_P3H1	0.263022	0.000565	0.002399
11723730_KREMEN1	-0.21046	0.000565	0.0024
11757401_KRT10	0.201192	0.000565	0.0024
11716454_MYO1C	0.207536	0.000566	0.002402
11759598_HSPA14	0.301035	0.000566	0.002402
11750141_CTPS1	-0.20542	0.000566	0.002402
11721905_PSMA4	-0.2423	0.000566	0.002402
11747672_KTN1	0.293449	0.000566	0.002402
11749645_ZNF268	0.256455	0.000566	0.002402
11750982_SHKBP1	-0.26571	0.000566	0.002402
11721519_ZDHHC17	0.436156	0.000566	0.002402
11750493_RNF216	0.255057	0.000567	0.002403
11743734_GNPTG	0.293006	0.000567	0.002405
11738174_PTCHD4	0.35236	0.000568	0.002407
11735295_LRRK2	0.655301	0.000568	0.002408
11752399_FUBP1	0.199649	0.000568	0.002409

11729474_FANCA	-0.51805	0.000568	0.002409
11756637_RNF219	-0.24734	0.000568	0.00241
11736002_DCN	0.448803	0.000569	0.002411
11753196_CDC40	0.211858	0.000569	0.002412
11718201_HEY1	-0.20086	0.000569	0.002413
11744631_FLT1	0.313443	0.000569	0.002413
11751479_CCDC152	0.334635	0.00057	0.002416
11757517_POR	-0.26794	0.00057	0.002417
11750180_ASPH	0.212315	0.00057	0.002417
11718598_DDRGK1	0.265196	0.000571	0.002418
11718346_CNOT7	-0.2143	0.000571	0.002418
11752247_TCF12	0.206289	0.000571	0.002418
11725708_WDHD1	-0.49796	0.000571	0.002419
11715769_SET	-0.19024	0.000571	0.00242
11757652_P3H1	0.225243	0.000572	0.002421
11722689_SPTBN1	-0.26739	0.000572	0.002422
11747610_AP1S2 ///	0.349943	0.000572	0.002423
11758304_PNRC2	0.201832	0.000572	0.002424
11743586_PAPD4	0.200857	0.000573	0.002425
11733428_BICD1	0.288892	0.000573	0.002427
11750766_TACC2	0.250049	0.000573	0.002427
11744666_FAN1	0.302934	0.000573	0.002427
11746192_NAP1L2	0.663461	0.000574	0.002427
11730740_PDLIM4	0.29649	0.000574	0.002431
11753073_TNFSF4	0.593529	0.000575	0.002433
11756958_NLE1	-0.20213	0.000575	0.002433
11748622_CTSA	0.235311	0.000575	0.002433
11731288_MBD2	0.279699	0.000575	0.002433
11719957_LOC441155	0.234835	0.000576	0.002434
11754519_PRR4	0.199954	0.000576	0.002435
11727480_RDH13	-0.24742	0.000576	0.002435
11757455_GPX1	0.204352	0.000576	0.002436
11719820_CLN5	0.2471	0.000576	0.002436
11724199_LSS	-0.20713	0.000577	0.002438
11724816_SH3KBP1	0.201587	0.000577	0.002438
11752224_SLFN5	0.280557	0.000577	0.002438
11756301_TSTA3	0.235118	0.000577	0.002439
11745112_C8orf31	0.376921	0.000577	0.002439
11750078_GYG1	0.255959	0.000577	0.002439
11749317_ATP6V0D1	0.236581	0.000577	0.002439
11728571_PLCL1	0.482727	0.000577	0.00244
11750058_UBE2W	-0.2931	0.000578	0.002441
11725495_ROBO1	0.356697	0.000578	0.002441
11731442_ARHGEF12	-0.3961	0.000578	0.002441
11755345_C9orf142	-0.20333	0.000578	0.002442
11724387_HOXB2	0.219802	0.000578	0.002442
11754829_PI4KA ///	0.206028	0.000579	0.002445
11755181_TCF7L2	0.227208	0.000579	0.002447
11763337_LHX6	-0.41023	0.000579	0.002447
11763269_MYSM1	0.210472	0.00058	0.00245
11723572_OTUD3	0.462168	0.00058	0.00245

11717374_DNAJC3	0.241213	0.00058	0.00245
11724257_MUC15	-0.25205	0.000581	0.002451
11722665_GABRB3	-0.30339	0.000581	0.002451
11750236_AGBL1	-0.31017	0.000581	0.002452
11755505_ZSWIM7	0.240498	0.000581	0.002453
11756589_NSUN5P1	0.267702	0.000582	0.002455
11747039_EHD1	0.324191	0.000582	0.002457
11736568_LEF1	-0.31285	0.000583	0.002458
11739570_ASH1L	0.260322	0.000583	0.002458
11741377_MMP2	0.5112	0.000583	0.002458
11736446_PDCD7	-0.27915	0.000583	0.002459
11725347_TFDP1	-0.22427	0.000583	0.002459
11741152_PLAGL1	-0.40029	0.000583	0.002459
11753950_ENTPD4	0.305198	0.000583	0.002459
11742734_WDR3	0.201678	0.000583	0.00246
11745471_ZNF276	0.304997	0.000583	0.00246
11731544_SIK2	0.231318	0.000584	0.002461
11737703_ADCK1	-0.26654	0.000584	0.002461
11744393_PAPOLA	-0.2451	0.000584	0.002461
11743260_MLX	0.221311	0.000584	0.002462
11758967_TFPI2	0.428482	0.000585	0.002464
11745194_OXCT1	-0.27818	0.000585	0.002465
11751350_APP	0.289046	0.000585	0.002465
11723806_STX17	0.297831	0.000585	0.002465
11726977_EIF4A2	-0.22665	0.000586	0.002468
11751114_ARFIP1	0.217444	0.000586	0.002469
11757070_LLPH	0.220055	0.000586	0.002469
11735041_C1orf52	-0.33522	0.000586	0.002469
11748991_CDK5RAP2	-0.22026	0.000587	0.00247
11724925_LTN1	0.272684	0.000587	0.002471
11759619_TRAPPC10	0.196964	0.000587	0.002471
11718279_RPRD1B	0.215442	0.000588	0.002476
11724822_CWF19L2	-0.22003	0.000588	0.002476
11755846_EIF3C ///	-0.19535	0.000588	0.002476
11723305_PYGO2	-0.2251	0.000588	0.002477
11733013_PEX2	-0.34562	0.000589	0.002479
11723977_RAB3A	-0.60824	0.000589	0.002479
11761067_TALDO1	-0.22621	0.000589	0.00248
11761009_TES	0.567645	0.00059	0.002482
11752342_PKM	-0.20766	0.00059	0.002483
11758368_SGTA	-0.22216	0.000591	0.002486
11742805_TEX261	-0.38794	0.000591	0.002486
11717165_RANGAP1	-0.22024	0.000591	0.002487
11731502_UTP23	0.208507	0.000591	0.002487
11740363_PTPN3	0.204203	0.000592	0.002489
11717420_SLC35A2	-0.22442	0.000592	0.002491
11755972_MGEA5	0.24385	0.000592	0.002491
11732525_CSGALNACT	0.432831	0.000592	0.002491
11750751_DDX3X	0.265124	0.000593	0.002493
11730196_RNF187	-0.20234	0.000594	0.002495
11723043_UBE2D1	0.240537	0.000594	0.002496

11759416_HFE	-0.47383	0.000594	0.002498
11747081_INPP5A	-0.31574	0.000595	0.002499
11747747_MVK	-0.23418	0.000595	0.002502
11732097_CDC14A	0.552573	0.000596	0.002503
11721461_COPS5	0.225741	0.000596	0.002504
11750108_INTS3	-0.21342	0.000596	0.002504
11747058_DTWD1	0.264658	0.000596	0.002505
11723786_ZNF766	0.233631	0.000597	0.002506
11739359_CHSY1	0.245506	0.000597	0.002506
11758223_GNPDA1	-0.2994	0.000597	0.002506
11763336_DKFZP586I	-0.34269	0.000597	0.002508
11758417_HLA-DPA1	-0.24591	0.000597	0.002508
11748431_VPS33B	0.242582	0.000598	0.002511
11739297_METTL2A	0.257885	0.000598	0.002511
11737028_OTX1	-0.45311	0.000598	0.002511
11747405_EPDR1	0.192088	0.000599	0.002513
11757304_ARL2BP	0.205316	0.000599	0.002513
11756382_SUPT7L	0.24649	0.000599	0.002514
11727268_ZBED3	-0.34875	0.000599	0.002514
11719657_MMP9	0.247282	0.000599	0.002514
11757845_ENDOD1	-0.22449	0.000599	0.002514
11729420_TTC26	0.375831	0.000599	0.002514
11727452_FAM21C	0.200638	0.000599	0.002514
11729886_C18orf54	-0.39508	0.0006	0.002515
11737189_RECQL5	-0.29369	0.0006	0.002517
11721436_STC2	0.26768	0.0006	0.002517
11750903_ATXN10	0.228203	0.000601	0.00252
11717565_EMP1	0.228559	0.000601	0.002521
11754725_SATB1	0.361355	0.000602	0.002522
11717524_RDX	0.202353	0.000602	0.002524
11738642_NOX3	0.313025	0.000602	0.002524
11758282_TUBA1C	-0.27647	0.000603	0.002526
11721178_PITX1	-0.22172	0.000603	0.002526
11748780_TOM1L1	0.229119	0.000603	0.002527
11757513_NFKBIZ	0.244659	0.000603	0.002528
11728876_CSF2	0.390284	0.000604	0.002529
11755076_ORAI2	0.245514	0.000604	0.00253
11730854_TMEM39B	-0.20696	0.000604	0.002531
11743069_PRPF38A	0.205839	0.000604	0.002532
11754340_ELAC2	-0.23401	0.000605	0.002534
11739980_SOCS4	0.443136	0.000605	0.002536
11724347_ASTN2	0.303234	0.000606	0.002538
11730231_TIAL1	0.193637	0.000606	0.002538
11749090_FOXP1	0.251438	0.000606	0.002539
11716071_PIM3	-0.22925	0.000606	0.002539
11727700_HOXC13	0.215337	0.000607	0.002541
11717164_RANGAP1	-0.19189	0.000607	0.002543
11758540_ZNF449	0.331002	0.000608	0.002545
11738216_RELL1	0.33047	0.000609	0.002548
11717681_GNA13	0.204683	0.000609	0.002548
11743131_APEH	-0.23981	0.00061	0.002551

11719307_RASSF4	-0.41461	0.00061	0.002552
11719932_KIAA0319L	0.206741	0.00061	0.002552
11720766_METTL9	-0.19401	0.00061	0.002552
11751620_RABL6	-0.31848	0.00061	0.002553
11724907_MAP4K5	0.222949	0.000611	0.002554
11722172_TOLLIP	0.212421	0.000611	0.002555
11718354_ERP44	0.224482	0.000611	0.002555
11740176_ARSK	0.241397	0.000611	0.002556
11760398_PHBP19	-0.32219	0.000612	0.00256
11731481_STK25	-0.22171	0.000612	0.00256
11745633_TRAPPC8	0.396007	0.000613	0.002563
11746792_BECN1	0.22723	0.000613	0.002563
11716635_TMEM50A	-0.20289	0.000613	0.002565
11733020_LDHA	-0.19109	0.000614	0.002567
11722597_ACKR3	-0.47424	0.000614	0.002568
11725346_GPX1	0.208238	0.000614	0.002568
11737301_NUP155	-0.21021	0.000614	0.002568
11741326_ENSA	-0.24909	0.000615	0.00257
11738100_C20orf195	-0.22361	0.000616	0.002574
11717146_PTPN1	0.21895	0.000616	0.002574
11734658_FOS	0.402305	0.000616	0.002574
11749241_RPP14	0.23097	0.000616	0.002574
11731170_DEFB126	-0.27141	0.000617	0.002577
11726878_ZNF436-AS	-0.2711	0.000617	0.002578
11735022_CEP63	0.424282	0.000618	0.002582
11732579_ZNF880	0.436592	0.000619	0.002586
11739436_KDM3B	-0.26243	0.000619	0.002586
11716463_GBA2	0.245135	0.000619	0.002586
11715761_TBC1D14	0.214626	0.000619	0.002586
11720211_TP53BP2	-0.31523	0.00062	0.002589
11756064_CBWD1 ///	0.231975	0.00062	0.00259
11758189_ITGB1	-0.19751	0.000621	0.002591
11740976_SLAIN1	-0.25812	0.000621	0.002591
11722611_TBC1D25	-0.39472	0.000621	0.002591
11728086_FAM179B	0.412575	0.000621	0.002592
11747627_TYW1 ///	-0.22659	0.000621	0.002593
11758557_ZFP36L1	0.211035	0.000622	0.002596
11755232_ZNF468	0.449508	0.000622	0.002597
11732670_CCDC107	0.245916	0.000623	0.002597
11728279_CASP6 ///	-0.322	0.000623	0.002597
11731596_ZNF503	-0.28985	0.000623	0.002597
11715408_PSMB4	-0.19988	0.000623	0.002598
11753580_SERF1A //	0.309229	0.000624	0.0026
11740980_CCNG1	0.373199	0.000624	0.0026
11736023_MED28	0.299046	0.000624	0.0026
11734578_CFH	0.568998	0.000624	0.0026
11736867_MMP16	0.300635	0.000624	0.002602
11751627_KDSR	0.295863	0.000624	0.002602
11731258_TMEM170B	0.308571	0.000625	0.002604
11753529_CDK11A //	0.199063	0.000626	0.002607
11728512_TRAF5	0.281093	0.000626	0.002607

11717380_FOPNL	-0.2009	0.000626	0.002608
11752913_SESN3	0.445918	0.000626	0.002609
11730203_FAM104A	0.249212	0.000626	0.002609
11759842_PTPRG	0.254537	0.000626	0.00261
11739757_FAM57A	-0.25458	0.000627	0.00261
11748804_MTG2	-0.23023	0.000627	0.002611
11747669_ITM2C	-0.23665	0.000627	0.002612
11720381_NFU1	0.202264	0.000628	0.002613
11719749_EBPL	-0.19173	0.000628	0.002613
11725649_DCAF5	0.380637	0.000628	0.002613
11720246_CDPF1	-0.27459	0.000628	0.002616
11747424_RNPS1	-0.2263	0.000628	0.002616
11729731_TPD52L3	-0.32451	0.000629	0.002617
11723629_TAF1	-0.28052	0.000629	0.002618
11761102_FCAR	0.404335	0.000629	0.002619
11717457_NLGN2	-0.25149	0.00063	0.00262
11747365_QARS	-0.25488	0.00063	0.002621
11756252_KLHDC3	0.244023	0.00063	0.002621
11716791_DKFZp686K	0.241009	0.00063	0.002621
11732443_BPTF	-0.46809	0.00063	0.002621
11716738_MSRB1	-0.22068	0.00063	0.002622
11732194_KPNA4	0.195138	0.000631	0.002622
11745649_SS18	0.257233	0.000631	0.002623
11726695_C1orf35	-0.30482	0.000631	0.002623
11725283_ZFP90	0.193567	0.000631	0.002623
11715623_RNF114	0.209519	0.000631	0.002623
11720520_LOC100996	0.358495	0.000631	0.002624
11741650_LRP12	0.204947	0.000632	0.002626
11719078_AAGAB	0.223948	0.000632	0.002627
11725670_IPO7	-0.2189	0.000632	0.002628
11719785_B4GALT4	-0.25067	0.000632	0.002628
11743258_LTBP3	0.280214	0.000633	0.002629
11737008_KIAA1429	0.244689	0.000633	0.00263
11739974_ZNF117	0.488599	0.000634	0.002633
11741017_AP2A2	0.283696	0.000634	0.002633
11725697_TRIM56	0.232015	0.000635	0.002638
11721348_SSH3	-0.2661	0.000635	0.002638
11740062_SIGMAR1	-0.23253	0.000635	0.002638
11732480_ITGAM	0.745656	0.000636	0.00264
11744217_TSPAN17	-0.34694	0.000636	0.00264
11746489_GPAA1	-0.20168	0.000636	0.00264
11759886_ANKRD36 /	-0.48437	0.000636	0.002643
11738526_GFM2	0.264454	0.000637	0.002643
11720905_QSER1	0.2108	0.000637	0.002643
11726567_EIF4G1	-0.21747	0.000637	0.002643
11724897_FZR1	-0.29147	0.000637	0.002644
11743107_DHX9	0.292628	0.000637	0.002645
11750362_CNTD1	-0.24211	0.000638	0.002646
11724889_POLRMT	-0.24921	0.000638	0.002648
11739772_OXR1	0.226918	0.000638	0.002648
11751868_C8orf59	-0.26126	0.000639	0.00265

11733868_HLA-DOA	-0.33935	0.000639	0.00265
11758179_ZNF175	0.299347	0.000639	0.00265
11747025_ERBB3	-0.2202	0.000639	0.00265
11721403_NCOA3	0.196196	0.000639	0.00265
11747837_ZFAND1	0.227962	0.000639	0.00265
11758507_SHC4	0.347759	0.000639	0.00265
11729264_ADAT1	0.246525	0.000639	0.002651
11716844_DNAJC13	0.254416	0.00064	0.002653
11722764_WDR62	-0.23999	0.00064	0.002653
11755498_ATP6V0E2	-0.25122	0.00064	0.002653
11736521_ZNF701	0.429137	0.00064	0.002653
11721306_PHLDA1	0.201041	0.00064	0.002653
11724116_UCKL1 ///	-0.22064	0.000641	0.002657
11733295_ZC3H14	-0.26706	0.000642	0.002659
11757158_RPL27A //	-0.2776	0.000642	0.002659
11729285_NFU1	0.193773	0.000642	0.002659
11741079_CREM	0.199638	0.000642	0.002659
11721622_KATNAL1	0.330349	0.000642	0.00266
11727763_PRODH2	-0.29678	0.000642	0.002661
11719072_WDR18	-0.2027	0.000643	0.002664
11730929_IKBKB	-0.24299	0.000644	0.002666
11722463_TNKS2	0.302811	0.000644	0.002667
11729678_POLK	0.317364	0.000645	0.00267
11758451_VCP	-0.22633	0.000645	0.00267
11721515_IPO4 ///	-0.19692	0.000645	0.002671
11718801_C1orf216	-0.27972	0.000646	0.002673
11725166_MED23	0.190858	0.000646	0.002674
11741966_TMTC4	-0.23795	0.000647	0.002676
11723664_SREBF2	-0.28837	0.000647	0.002676
11756790_ITGA3	0.197201	0.000647	0.002676
11727378_TBC1D15	-0.19803	0.000647	0.002677
11722100_RICTOR	0.218278	0.000647	0.002678
11754977_CTSB	-0.20252	0.000648	0.002681
11718929_TGIF2	0.209091	0.000649	0.002685
11751707_TASP1	0.311421	0.000649	0.002686
11725089_TASP1	0.30415	0.00065	0.002687
11720556_TSEN15	-0.19592	0.00065	0.002688
11747076_DBNL	-0.2019	0.00065	0.002688
11756725_SHISA5	-0.2466	0.00065	0.002689
11727339_ZBTB2	-0.33666	0.00065	0.002689
11763212_CBLB	0.436077	0.000651	0.00269
11756606_RWDD2B	0.24977	0.000651	0.00269
11726580_PSME1	0.199915	0.000651	0.002691
11748612_ETV5	-0.20723	0.000651	0.002692
11747310_WBP11	-0.26051	0.000651	0.002692
11723426_ETFB	-0.20117	0.000652	0.002694
11720310_SERP1	-0.20751	0.000652	0.002696
11749737_CPEB1	0.53821	0.000652	0.002696
11717806_ZSWIM8	0.23071	0.000653	0.002696
11717646_MCMBP	-0.23115	0.000653	0.002696
11735701_CD151	-0.22292	0.000653	0.002698

11727682_ACVR2B	-0.27765	0.000653	0.002699
11756818_PATL2	-0.23787	0.000654	0.0027
11747954_PYROXD1	0.273898	0.000654	0.002701
11724118_SAMD9L	0.517066	0.000654	0.002701
11743787_GORAB	0.253088	0.000654	0.002702
11719860_SLC2A4RG	-0.20565	0.000654	0.002702
11750859_ZFP37	0.364942	0.000655	0.002702
11736546_FANCD2	-0.29855	0.000655	0.002702
11731044_ERLIN2	0.315155	0.000655	0.002702
11734768_THOC2	-0.21987	0.000655	0.002703
11732330_MAP7D1	-0.21909	0.000655	0.002704
11717657_EZH2	-0.22741	0.000656	0.002708
11719024_ZNF652	0.432569	0.000656	0.002708
11727936_SLC9A3R1	-0.22677	0.000657	0.002709
11717775_GYPC	0.266437	0.000657	0.002709
11749722_NDUFS1	-0.18702	0.000657	0.002709
11717083_TSPAN9	0.334821	0.000657	0.002709
11756458_MSH5-SAPC	-0.20686	0.000658	0.002712
11715402_ILF2	-0.18537	0.000658	0.002713
11717059_CECR5	-0.21765	0.000658	0.002713
11756765_BCL2L13	0.254449	0.000658	0.002713
11726301_ATG4A	0.246447	0.000658	0.002713
11727806_UBE2W	-0.29999	0.000658	0.002713
11717732_PFKFB3	0.240707	0.000658	0.002714
11757693_PIAS3	-0.23739	0.000658	0.002714
11720413_ARRDC1-AS	-0.20814	0.000659	0.002716
11718818_SLC26A6	0.235924	0.000659	0.002717
11717503_NUB1	-0.19344	0.00066	0.002719
11760103_PRKAR2A	-0.21215	0.00066	0.00272
11752890_SNTA1	-0.38763	0.00066	0.002722
11718812_MKNK1	0.360473	0.000661	0.002722
11742035_PLCL1	0.451682	0.000661	0.002724
11734893_SOBP	-0.24306	0.000661	0.002724
11762396_KIAA1324	0.303905	0.000661	0.002725
11716225_LIMA1	0.486071	0.000662	0.002725
11735316_CLPSL1	-0.20819	0.000662	0.002725
11756722_STOML2	-0.22552	0.000662	0.002727
11724375_PAG1	0.454716	0.000662	0.002727
11739743_MXI1	-0.40696	0.000663	0.002728
11756915_CYP4F11	0.238156	0.000663	0.002731
11747487_PPP2R2B	0.214764	0.000663	0.002731
11716850_MFSD14B	-0.23255	0.000664	0.002733
11751319_EIF4A1 //	-0.18801	0.000664	0.002733
11760177_ZSCAN32	-0.30563	0.000665	0.002735
11753151_HDAC8	-0.2147	0.000665	0.002735
11747484_JADE2	-0.27987	0.000665	0.002737
200080_PM H3F3A ///	-0.2654	0.000666	0.002739
11744364_INPP5D	0.257239	0.000666	0.002739
11730828_CPSF2	0.194582	0.000666	0.002739
11749179_MTMR9	0.355295	0.000666	0.00274
11739726_NAP1L1	0.189129	0.000666	0.00274

11719602_MOXD1	0.199488	0.000666	0.002741
11742444_PIP4K2C	0.251581	0.000668	0.002746
11739121_XPO7	-0.2143	0.000668	0.002748
11716840_UBE4A	0.210075	0.000668	0.002748
11719270_RBM22	0.198254	0.000669	0.002749
11749455_KDM5B	0.330056	0.000669	0.002749
11760507_ELF2	-0.25184	0.000669	0.002751
11722588_BCAP29	0.230135	0.000669	0.002752
11747746_HADHB	-0.22033	0.00067	0.002752
11745483_BECN1	0.202756	0.00067	0.002753
11722595_AKAP13	0.216298	0.00067	0.002753
11727081_TIPIN	-0.34548	0.00067	0.002754
11733870_HLA-DOA	-0.41373	0.000671	0.002755
11752022_NCOA3	0.237295	0.000671	0.002755
11748980_USP46	0.434245	0.000671	0.002758
11715808_PYGB	-0.22528	0.000671	0.002758
11757768_AK3	0.225931	0.000672	0.00276
11749066_BGN	-0.24162	0.000672	0.00276
11723118_DSTYK	0.237118	0.000672	0.00276
11751450_ALKBH5	-0.19762	0.000672	0.002761
11758278_BCDIN3D	0.205001	0.000672	0.002761
11724723_HSPA2	-0.42996	0.000673	0.002762
11732965_DMD	0.257857	0.000673	0.002764
11719440_SRGAP2	-0.30371	0.000673	0.002764
11754064_ANXA2	0.189069	0.000673	0.002764
11720114_UBE2D4	-0.28204	0.000674	0.002766
11719986_CCDC117	-0.20497	0.000674	0.002766
11732705_FGFRL1	-0.24134	0.000674	0.002766
11727505_RAB21	-0.23718	0.000674	0.002767
11723481_TIA1	0.252772	0.000675	0.002769
11723187_FGD6	0.476041	0.000675	0.002771
11730891_NSUN6	-0.23664	0.000676	0.002771
11717513_SEPT8	0.22736	0.000676	0.002772
11755457_SLAIN1	-0.3106	0.000676	0.002773
11761058_CCNT2-AS1	-0.24625	0.000676	0.002773
11753480_RTN4IP1	-0.33086	0.000677	0.002775
11751239_SHPK ///	-0.48891	0.000677	0.002775
11720809_MZT2B ///	-0.22493	0.000677	0.002777
11753976_ZMYM3	-0.2477	0.000677	0.002777
11746619_RTEL1 ///	-0.24003	0.000678	0.00278
11751954_MAGI3	-0.36909	0.000678	0.002781
11730229_TIAL1	0.240867	0.000678	0.002781
11759076_HIST1H3H	0.269764	0.000679	0.002781
11758472_RICTOR	0.249874	0.000679	0.002782
11747146_TMBIM6	-0.29216	0.000679	0.002783
11716069_DESI2	-0.20499	0.000679	0.002783
11737905_CASC4	0.210857	0.00068	0.002784
11743371_DNM1L	-0.28704	0.00068	0.002784
11716032_ARF3	-0.24649	0.00068	0.002785
11726758_CDC25A	-0.24097	0.00068	0.002786
11750331_INTS7	0.252288	0.00068	0.002787

11719981_RBPMS	0.287008	0.000681	0.00279
11736176_TRNT1	0.2408	0.000681	0.002791
11755295_SIGMAR1	-0.20138	0.000682	0.002791
11724302_EEA1	0.401927	0.000682	0.002792
11726084_FAM69B	-0.30194	0.000682	0.002792
11717311_FBXO7	-0.20559	0.000682	0.002792
11726001_TRA2A	0.291879	0.000682	0.002792
11724049_COG6	-0.24502	0.000682	0.002792
11715558_TMEM59	0.198268	0.000682	0.002792
11727216_FRS3	-0.25863	0.000682	0.002792
11747831_FGFR1OP	0.313111	0.000683	0.002794
11728880_PPP3R1	0.249443	0.000683	0.002794
11762908_TNFRSF14	0.243284	0.000683	0.002794
11743743_ATP11A	-0.2602	0.000684	0.002796
11730915_ERLIN1	0.26082	0.000684	0.002797
11726113_FAM46B	0.288203	0.000684	0.002797
11731880_NLRP1	0.448276	0.000685	0.0028
11758934_RAB27A	-0.49927	0.000685	0.002801
11735732_RPP14	0.364832	0.000686	0.002806
11717254_HSPA4	-0.27419	0.000686	0.002806
11730285_SMU1	0.270865	0.000687	0.002808
11755268_DDX18	0.239887	0.000687	0.002808
11739826_DNAJB12	-0.20557	0.000687	0.002809
11747471_PARPBP	-0.31709	0.000687	0.00281
11750480_ATF2	0.313741	0.000688	0.00281
11719811_TRIB3	-0.28509	0.000688	0.002811
11738146_ZRANB3	-0.23909	0.000689	0.002814
11734489_PUSL1	-0.25815	0.000689	0.002817
11759026_SLC25A40	-0.20261	0.000689	0.002817
11741791_RPS27A	-0.23752	0.00069	0.002817
11721413_MBTD1	0.292488	0.00069	0.002818
11757449_IFI27L1	-0.2524	0.00069	0.002818
11738551_ANXA2	0.289283	0.00069	0.002819
11718894_RFC1	-0.24732	0.000691	0.002821
11740548_YAP1	-0.28764	0.000691	0.002822
11748854_RPUSD4	0.311227	0.000692	0.002825
11725861_MYBL1	0.250426	0.000692	0.002827
11755895_FAM129A	-0.24278	0.000693	0.002829
11722445_TRAPPC5	0.217261	0.000693	0.002831
11746439_GLO1	-0.20375	0.000694	0.002831
11719885_KAZN	0.429784	0.000694	0.002831
11720389_PTPN23	-0.28451	0.000694	0.002832
11760344_ATP10D	0.376966	0.000694	0.002833
11731436_CETN2	0.229593	0.000695	0.002836
11722817_ZNF3	0.351495	0.000695	0.002836
11745887_USP42	0.249054	0.000696	0.00284
11739231_TMEM106B	-0.21038	0.000697	0.002844
11733905_RRP15	0.323263	0.000697	0.002845
11723343_TRIM35	0.410992	0.000697	0.002845
11721564_PLA2G4C	0.308482	0.000698	0.002845
200028_PMSTARD7	-0.19106	0.000698	0.002846

11715927_SH3GL1	-0.18992	0.000698	0.002848
11734057_PTGR2	-0.26587	0.000699	0.00285
11746711_NDUFAF7	0.222709	0.000699	0.00285
11758724_YWHAQ ///	-0.2119	0.000701	0.002859
11719491_IFI35	-0.23873	0.000702	0.00286
11733845_GRK6	-0.19766	0.000702	0.00286
11726148_PPIP5K2	-0.25387	0.000702	0.002861
11754631_PIK3R1	0.252188	0.000702	0.002861
11754249_WHAMMP2 /	0.238225	0.000703	0.002864
11745009_LZTS2	-0.18749	0.000703	0.002864
11755215_JRK	-0.35543	0.000703	0.002864
11756939_BRD7	0.221098	0.000703	0.002866
11757666_SNRPD2	-0.18778	0.000704	0.002869
11758641_BLCAP	0.288912	0.000705	0.00287
11721275_TADA1	-0.20626	0.000705	0.00287
11723594_TNK2	-0.23194	0.000705	0.00287
11759514_BCL2L1	0.225013	0.000706	0.002873
11739768_PTPRE	0.246226	0.000706	0.002874
11759647_ZC3H7A	0.448995	0.000706	0.002874
11761371_BMS1P21	-0.29155	0.000707	0.002878
11727182_ZNF175	0.293369	0.000707	0.00288
11748135_TYW1 ///	-0.31679	0.000708	0.00288
11728003_C4orf48	0.194594	0.000708	0.00288
11732150_DCAF11	-0.20172	0.000708	0.00288
11752180_VHL	-0.21731	0.000708	0.002882
11732056_SNRNP35	-0.30162	0.000708	0.002882
11723750_TRMO	0.312126	0.000709	0.002883
11730497_ZNF239	0.342321	0.000709	0.002884
11723251_RPS15A	0.206991	0.000709	0.002884
11730958_CHCHD4	-0.23494	0.000709	0.002884
11756131_UPF3A	0.189424	0.000711	0.002892
11729138_SLAMF7	0.378164	0.000711	0.002892
11721297_ABRACL	-0.20472	0.000711	0.002893
11756288_NRDE2	-0.23946	0.000711	0.002893
11754527_RQCD1	0.246465	0.000712	0.002894
11754097_GTF2A2	0.194869	0.000712	0.002896
11746920_C5orf34	-0.51491	0.000712	0.002896
11718571_NT5DC3	-0.20521	0.000713	0.002897
11740281_TSEN2	-0.44232	0.000713	0.002899
11723306_PYGO2	-0.2383	0.000713	0.002899
11717403_SCAMP3	0.197867	0.000713	0.0029
11751417_GNA12	-0.24786	0.000714	0.002903
11755059_TCF3	-0.25457	0.000714	0.002903
11756100_TMEM60	-0.25546	0.000715	0.002904
11728253_NMNAT2	0.207459	0.000715	0.002904
11717813_ST3GAL4	-0.26246	0.000715	0.002904
11721393_NINJ2	0.279817	0.000715	0.002906
11757609_DLG5	0.224097	0.000716	0.002906
11760529_RDM1	-0.35325	0.000716	0.002908
11727221_XYLT1	-0.46073	0.000716	0.002908
11737418_ERCC6L2	0.264753	0.000716	0.002908

11719922_GM2A	-0.25024	0.000716	0.002909
11719609_ADO	0.256441	0.000717	0.00291
11734780_ZNF639	0.217855	0.000717	0.002912
11716206_GLUD1	-0.20248	0.000717	0.002912
11715260_RRNAD1	-0.2531	0.000717	0.002912
11754947_NAV1	-0.23176	0.000718	0.002914
11749398_B9D1	0.189514	0.000719	0.002918
11748889_RBM18	0.395654	0.000719	0.002918
11760354_CNTLN	-0.93077	0.000719	0.002918
11730217_CERS5	0.318657	0.000719	0.002918
11747034_IL7	-0.25836	0.000719	0.002918
11738152_RBM24	0.391902	0.00072	0.00292
11716689_GOLGA3	0.374708	0.00072	0.002923
11731600_FOSL2	0.473863	0.000721	0.002925
11738015_SLC10A3	-0.25116	0.000721	0.002925
11761960_TRAC ///	0.344038	0.000721	0.002926
11733204_SH3TC2	0.258568	0.000721	0.002926
11747871_ZMYND11	0.331423	0.000722	0.002926
11722181_ATP6V1C1	0.249574	0.000722	0.002926
11756117_ZFP28	0.490182	0.000722	0.002926
11742315_RHCE ///	-0.68088	0.000722	0.002926
11757310_TMEM98	-0.27943	0.000722	0.002926
11717294_SRSF7	-0.20451	0.000722	0.002926
11740981_DTX2	-0.35634	0.000722	0.002927
11727092_IL18	0.572755	0.000723	0.002928
11717680_UNC13B	0.199318	0.000723	0.002929
11716771_LOC102724	0.325436	0.000723	0.002929
11755053_ELAC2	-0.24255	0.000723	0.002929
11757585_MAGED1	-0.2029	0.000723	0.00293
11747729_SDHA ///	-0.24959	0.000723	0.002931
11745026_ERLIN2	-0.22739	0.000724	0.002932
11717073_AEBP1	0.27052	0.000724	0.002933
11756985_MAT2A	0.195467	0.000724	0.002933
11754326_GPATCH8	-0.25245	0.000725	0.002935
11747681_THRA	-0.36537	0.000726	0.002938
11725572_TAOK2	-0.22526	0.000726	0.002939
11726780_YPEL3	0.23632	0.000726	0.002939
11751836_GPX6	-0.38318	0.000726	0.00294
11730359_CCDC126	0.46989	0.000726	0.00294
11743739_NR3C1	-0.2435	0.000726	0.00294
11753470_TIA1	0.308909	0.000727	0.002942
11724376_PAG1	0.27477	0.000727	0.002943
11721199_IFT122	0.255312	0.000727	0.002943
11722286_SLC30A7	0.213192	0.000727	0.002943
11724979_DPEP2	-0.28627	0.000728	0.002943
11717340_PTGFRN	0.265098	0.000728	0.002943
11734209_CNTF ///	0.380394	0.000728	0.002944
11743380_NFE2L2	0.191935	0.000729	0.002948
11718500_UBE2Q1	0.242566	0.000729	0.002948
11745611_PDDC1	0.198623	0.000729	0.002948
11760020_PIGP	-0.27174	0.000729	0.002948

11749308_FCHO2	0.304709	0.00073	0.00295
11760025_EXD3	0.545906	0.00073	0.00295
11743931_ERP29	-0.21084	0.00073	0.002951
11758026_SLC35A5	-0.21819	0.00073	0.002952
11725123_POLB	0.198579	0.00073	0.002952
11752611_SSR1	0.260515	0.000731	0.002953
11762047_USP32	0.260467	0.000731	0.002953
11753719_GMFB	0.27158	0.000731	0.002953
11747905_NID2	-0.38288	0.000731	0.002953
11755913_LPO	-0.25802	0.000731	0.002955
11727782_TPM4	-0.29437	0.000731	0.002955
11732397_IFT80	-0.25402	0.000732	0.002955
11751236_TBC1D14	0.267958	0.000732	0.002957
11730686_WT1	-0.21564	0.000732	0.002958
11716037_GPAA1	-0.19895	0.000732	0.002958
11731550_PLSCR4	0.339576	0.000733	0.002958
11716876_TSTA3	0.212375	0.000733	0.002958
11728432_FAM19A1	0.414261	0.000733	0.002959
11739341_TGDS	-0.20886	0.000734	0.002962
11730030_E2F7	0.239676	0.000734	0.002962
11728513_NDUFAF6	0.237804	0.000734	0.002963
11742953_ACTR8	0.337989	0.000734	0.002963
11759924_TRIM4	0.307899	0.000734	0.002964
11736149_LYRM2	0.241611	0.000735	0.002967
11755887_MTMR12	0.297677	0.000735	0.002967
11718307_GSE1	-0.33907	0.000736	0.002968
11757241_KRT17P5	-0.33906	0.000736	0.002968
11736246_AMIGO2	0.266071	0.000736	0.002969
11733846_GRK6	-0.32251	0.000736	0.002969
11739707_SLC35A2	-0.22891	0.000736	0.00297
11737200_PRRX1	-0.24497	0.000736	0.00297
11736860_CPEB1	0.415398	0.000737	0.002971
11720428_CDC40	0.245319	0.000737	0.002972
11757861_TSHZ1	-0.34189	0.000738	0.002975
11753757_NUTF2	-0.18134	0.000738	0.002975
11733089_ZNF277	0.227605	0.000738	0.002976
11749414_EIF2B4	0.218378	0.000738	0.002976
11719204_ANKLE2	0.216293	0.000738	0.002976
11715849_APOLD1 //	0.183437	0.000738	0.002976
11763423_ATP6V0D1	0.197453	0.000739	0.002979
11723656_DDX19A //	0.24312	0.000739	0.00298
11725639_NSL1	-0.28506	0.00074	0.00298
11744167_DPP3	-0.23257	0.00074	0.002982
11731662_KIAA1217	0.363226	0.00074	0.002983
11758642_NF1	-0.21692	0.000742	0.002989
200069_PMSART3	-0.25245	0.000743	0.002993
11745359_MLIP	0.230699	0.000744	0.002995
11727007_SORT1	0.245966	0.000744	0.002996
11755659_CTAGE5	-0.22397	0.000744	0.002996
11756105_BMPR2	0.226785	0.000744	0.002997
11730262_TBCD	-0.28685	0.000744	0.002997

11751173_PGPEP1	0.209619	0.000745	0.002998
11720324_POLR2F	-0.21235	0.000745	0.003
11720235_RHOBTB2	-0.25703	0.000745	0.003
11748413_DDB1	-0.19098	0.000745	0.003001
11740562_MASP2	-0.28253	0.000746	0.003001
11736428_STYX	-0.28593	0.000746	0.003001
11725348_TFDP1	-0.21676	0.000746	0.003001
11733806_AMN	-0.22946	0.000746	0.003001
11718388_NUDT2	-0.25228	0.000746	0.003002
11748580_STX1A	0.669265	0.000746	0.003002
11727195_ZNF202	0.390081	0.000747	0.003003
11731247_GTPBP8	0.242305	0.000747	0.003004
11726185_PARPBP	-0.26145	0.000747	0.003004
11720608_S100B	0.242612	0.000747	0.003004
11735722_PNKD	0.238771	0.000747	0.003006
11748843_USP42	0.247998	0.000748	0.00301
11736230_CRIM1	0.408378	0.000749	0.003011
11753619_EIF5A ///	-0.19226	0.000749	0.003013
11753681_TRAF4	0.221556	0.00075	0.003014
11741488_EFCAB2	-0.24976	0.00075	0.003014
11759357_PAXBP1	0.379027	0.000751	0.003017
11717673_EIF2B2	0.204972	0.000751	0.003018
11757509_RAB6A	0.187294	0.000751	0.003018
11759225_EDF1	-0.22972	0.000751	0.003018
11723856_TXNDC16	-0.32672	0.000751	0.003019
11751528_CWC25	0.221803	0.000751	0.003019
11725846_SREK1IP1	0.194649	0.000751	0.003019
11721634_TMEM97	-0.25433	0.000751	0.003019
11720499_CDC42EP3	-0.22402	0.000751	0.003019
11758066_VAPB	-0.50453	0.000753	0.003024
11751001_PAK4	-0.2546	0.000753	0.003024
11730176_C4orf3	-0.21425	0.000753	0.003026
11746044_ELF2	0.235349	0.000754	0.003028
11757432_EBAG9	-0.25233	0.000754	0.003029
11723135_SRC	-0.20058	0.000755	0.003033
11733388_ZKSCAN3	0.349837	0.000755	0.003033
11736733_DPY19L3	0.303595	0.000757	0.003038
11715531_PRPF8	-0.21161	0.000758	0.003042
11719415_YES1	0.21645	0.000758	0.003044
11718695_YIF1B	-0.22988	0.000758	0.003044
11723671_BNIP2	0.194787	0.000759	0.003044
11763213_NSUN5P1 /	0.20258	0.000759	0.003045
11750994_SYAP1	0.235932	0.000759	0.003046
11751509_DKFZp686K	0.230882	0.000759	0.003046
11734502_NUP98	0.199255	0.00076	0.003048
11720049_GBE1	-0.25608	0.00076	0.003049
11726938_PHPT1	0.208348	0.00076	0.00305
11730366_ZNF557	0.339791	0.000761	0.00305
11719542_PAXBP1	0.216845	0.000761	0.003051
11721395_MUM1	-0.28187	0.000761	0.003051
11723566_CAPN7	0.262147	0.000761	0.003053

11756086_HNRNPA1L2	-0.18872	0.000762	0.003053
11757151_SNORA72	-0.23776	0.000762	0.003056
11741104_CPEB1	0.244097	0.000762	0.003057
11748526_HVCN1	-0.32134	0.000763	0.003057
11764275_SLC11A2	-0.19844	0.000763	0.003057
11744109_ZC3H15	0.187342	0.000763	0.003059
11717334_HMGXB3	0.203328	0.000763	0.003059
11715748_SSRP1	-0.20859	0.000764	0.003061
11745751_MSL1	0.224195	0.000764	0.003061
11717051_SSR1	0.190601	0.000764	0.003061
11734969_RUSC1	-0.2213	0.000764	0.003061
11753598_FHL1	0.432267	0.000765	0.003063
11716208_GLUD1	-0.47798	0.000765	0.003064
11724039_EHHADH	-0.71776	0.000765	0.003064
11758598_SERPINE2	0.185526	0.000765	0.003065
11729987_ROBO2	0.291456	0.000765	0.003066
11740203_BORCS7 //	0.302159	0.000766	0.003066
11758887_RHBDD1	-0.2345	0.000766	0.003066
11719381_PSENEN	0.203359	0.000767	0.00307
11741295_DDI2 ///	0.23157	0.000767	0.00307
11732730_AJAP1	0.349039	0.000767	0.00307
11719776_NDST1	0.319262	0.000767	0.003072
11719589_TOM1L1	0.218632	0.000768	0.003074
11749587_FCGR2A //	0.307819	0.000768	0.003074
11739000_SLC38A2	-0.18991	0.000768	0.003074
11745035_LINC01296	0.217464	0.000769	0.003076
11720586_SMCHD1	-0.23021	0.000769	0.003076
11758924_LOC100129	-0.34887	0.000769	0.003078
11759522_ALKBH7	-0.24209	0.000769	0.003078
11742326_GHRHR	-0.24886	0.00077	0.003079
11717370_ARSD	0.301418	0.000771	0.003082
11745740_SCOC	-0.22968	0.000771	0.003082
11731131_JRK	-0.29048	0.000771	0.003084
11749652_ZBTB21	0.377687	0.000772	0.003086
11717207_GOLGA2	0.211394	0.000772	0.003088
11732785_C16orf45	-0.20917	0.000772	0.003088
11759854_RPS27A	-0.35303	0.000772	0.003088
11744224_DCAF15	-0.27947	0.000773	0.003088
11753401_TPI1	-0.1856	0.000773	0.00309
11724222_YIPF4	0.197194	0.000773	0.00309
11744027_TMEM181	-0.21057	0.000773	0.00309
11743593_DIMT1	-0.20125	0.000773	0.00309
11756067_GNAQ	0.386747	0.000774	0.003091
11737602_NLGN1	0.348968	0.000774	0.003094
11744399_MYEOV2	0.185802	0.000776	0.003099
11747386_STK25	-0.32895	0.000776	0.003099
11724689_CD58	0.205195	0.000776	0.003099
11719617_UCK1	-0.22527	0.000776	0.003099
11716745_FAM60A	0.188108	0.000776	0.003099
11739484_CCDC88A	0.248307	0.000776	0.0031
11752076_SEPT10	-0.20423	0.000776	0.0031

11730469_LMO2	0.226146	0.000777	0.003101
11719476_FAM210B	0.208888	0.000777	0.003103
11728017_RHPN1	-0.25059	0.000778	0.003106
11743376_XRCC6BP1	-0.33238	0.000778	0.003107
11723383_PRR3	-0.23697	0.000779	0.003107
11716869_UTP18	0.232434	0.000779	0.003108
11720618_TRIM9	0.329045	0.000779	0.003109
11744539_RTN3	-0.21103	0.000779	0.003109
11716252_RBFOX2	0.223503	0.000779	0.003109
11754743_CWC22	0.244156	0.00078	0.003112
11720201_MGMT	-0.27168	0.00078	0.003114
11742909_ANKRD50	0.211956	0.000781	0.003115
11740039_EHBP1	-0.21411	0.000781	0.003116
11748576_CYB5R1	-0.20142	0.000781	0.003116
11725185_KLF12	-0.29553	0.000781	0.003116
11717122_C6orf106	0.271603	0.000781	0.003116
11748034_CMAHP	0.423169	0.000782	0.003118
11739728_RECQL	-0.20967	0.000782	0.003119
11758726_ATP5C1	-0.44075	0.000782	0.003119
11716083_AHSA1	-0.19584	0.000782	0.003119
11719439_SRGAP2	-0.28296	0.000783	0.00312
11761424_RAG1	-0.32034	0.000783	0.00312
11752223_POLH	0.393871	0.000783	0.003121
11756723_LDHA	-0.21159	0.000783	0.003121
11727393_COX15	0.182165	0.000784	0.003125
11723422_BTF3L4	0.228292	0.000785	0.003127
11752372_ABCA10	0.244987	0.000785	0.003127
11730788_APLP2	-0.21944	0.000785	0.003128
11727929_FOXO1	0.601903	0.000786	0.00313
11717501_HAGH	-0.24558	0.000786	0.003131
11752593_CEP170	0.196232	0.000786	0.003131
11723319_FAM234B	0.422798	0.000787	0.003135
11745030_LINC00597	0.435614	0.000787	0.003135
11716336_PRCC	0.209448	0.000787	0.003136
11722768_RAPGEF5	-0.28857	0.000788	0.003139
11745022_TRMT2B	-0.21393	0.000788	0.003139
11729799_MTTP	0.265746	0.000789	0.003142
11716679_SLC39A9	0.21218	0.00079	0.003144
11719120_KYNU	0.281616	0.00079	0.003146
11754495_GABARAPL2	0.192144	0.000791	0.003149
11728233_CLDN1	0.347785	0.000791	0.00315
11757957_SOX6	-0.40259	0.000792	0.003154
11727793_DLAT	-0.19293	0.000794	0.003158
11737762_HSD17B4	-0.20549	0.000794	0.003158
11739611_CALML4	-0.28783	0.000794	0.003158
11732461_SPINK2	-0.41881	0.000794	0.003159
11717949_SC5D	-0.55729	0.000794	0.003159
11730211_PFKL	-0.21334	0.000795	0.003163
11731962_ZNF709	0.302231	0.000795	0.003163
11724142_RAB11FIP2	0.315206	0.000796	0.003165
11759307_RREB1	0.253029	0.000797	0.003168

11741200_AKAP2 ///	0.273076	0.000797	0.003169
11736537_RHCE ///	-0.29553	0.000797	0.00317
11757632_LIMK2	0.380105	0.000797	0.00317
11726757_CDC25A	-0.23052	0.000798	0.003171
11721499_CTSA	0.203027	0.000798	0.003172
11757081_OXCT2 ///	-0.227	0.000798	0.003172
11744337_GLUL	-0.24203	0.000798	0.003172
11724288_PRMT9	0.265088	0.000798	0.003172
11723987_NR2F2	0.215353	0.000798	0.003173
11750562_ZMYM4	0.192257	0.0008	0.003177
11744015_KDELR1	-0.22438	0.0008	0.003177
11719590_SUGP2	0.206651	0.000801	0.00318
11725967_THUMPD1	0.261085	0.000801	0.003181
11719920_TIMM10B	0.26997	0.000801	0.003182
11746575_LTBP3	0.298242	0.000801	0.003182
11744947_ZDHHC5	0.219935	0.000802	0.003186
11757759_PEG10	-0.93354	0.000803	0.003188
11726356_ENPP1	-0.54875	0.000803	0.00319
11752003_IQGAP1	-0.24214	0.000804	0.003191
11717895_PTP4A1	0.203669	0.000804	0.003191
11754610_FICD	-0.2271	0.000804	0.003192
11719844_SMAP2	-0.21458	0.000804	0.003193
11729985_FUT10	0.317179	0.000805	0.003194
11739613_CALML4	-0.22369	0.000805	0.003195
11753061_SLFN5	0.297537	0.000805	0.003196
11747090_NOP58	0.24029	0.000806	0.003196
11742245_HTRA2	-0.22978	0.000806	0.003197
11735216_CHRNA5	-0.18827	0.000806	0.003197
11761703_DNAJA1	0.213225	0.000806	0.003198
11730192_SEC14L2	-0.19164	0.000807	0.003202
11758648_KCTD10	-0.2103	0.000807	0.003202
11738191_MTFR1	-0.26389	0.000808	0.003203
11756135_NUBP2	-0.19133	0.000808	0.003206
11752185_MSH5 ///	-0.26218	0.00081	0.00321
11756792_DBNL	-0.20666	0.000811	0.003215
11755090_MTG1	-0.20258	0.000811	0.003217
11742747_SPPL3	0.202313	0.000811	0.003217
11715274_TPM2	0.302218	0.000813	0.003222
11724728_CD8A	-0.21999	0.000813	0.003222
11719574_RINT1	0.202466	0.000813	0.003223
11738906_BLVRA	-0.2529	0.000813	0.003223
11759150_CNOT4	-0.22287	0.000814	0.003224
11723986_NR2F2	0.195251	0.000814	0.003227
11746961_HLA-DMB	-0.26384	0.000815	0.003228
11733500_ZBTB25	0.229144	0.000815	0.003228
11718174_C1D	-0.22227	0.000815	0.003228
11731159_TAF1C	0.261134	0.000816	0.003231
11757013_CAV1	0.198728	0.000816	0.003231
11748596_KLHL2	0.315144	0.000816	0.003233
11722150_EIF2AK3	0.267224	0.000817	0.003234
11759588_SLC25A35	-0.24982	0.000817	0.003235

11752199_APP	0.26042	0.000817	0.003235
11744585_ATRN	-0.38812	0.000817	0.003236
11736831_SEC23B	-0.25306	0.000817	0.003236
11746469_DARS	-0.19302	0.000817	0.003236
11720188_NDUFA7	-0.19007	0.000817	0.003236
11742143_RHCE ///	-0.64254	0.000818	0.003238
11728573_MBNL1	0.219245	0.000818	0.003238
11744641_CLDND1	0.197616	0.000819	0.003239
11722277_ZBED4	-0.24987	0.000819	0.003239
11723652_MUT	-0.23039	0.000819	0.003239
11759408_AQR	-0.28332	0.000819	0.003241
11722527_PTPN21	0.417949	0.000819	0.003242
11735730_PRKRIR	0.206134	0.000821	0.003246
11730670_OPHN1	0.343388	0.000821	0.003247
11715948_KCTD12	-0.35323	0.000821	0.003247
11748055_TFDP1	-0.21567	0.000821	0.003247
11741821_NAF1	0.290604	0.000821	0.003248
11722857_FBXO16 //	-0.23998	0.000821	0.003248
11760395_IQCH	0.382376	0.000821	0.003248
11718895_RFC1	-0.23634	0.000822	0.003248
11733066_NFYB	-0.22342	0.000822	0.003249
11746275_FKBP5	-0.33049	0.000822	0.003249
11742789_SYCN	-0.19067	0.000822	0.003249
11716707_ARL2BP	0.246938	0.000822	0.003249
11739030_CLTC	0.206736	0.000822	0.003249
11757185_CDK16	-0.46247	0.000823	0.003251
11754311_DPF2	0.194611	0.000823	0.003251
11716719_GLA	-0.18825	0.000823	0.003253
11716061_FAM162A	-0.20919	0.000824	0.003254
11758414_ZBTB21	0.208155	0.000824	0.003254
11749430_NDUFAF6	0.251554	0.000824	0.003254
11754517_CHP1	0.198698	0.000824	0.003256
11740917_WDFY3	0.550663	0.000825	0.003257
11724099_MAP3K6	-0.24814	0.000825	0.003258
11724722_HAGHL	-0.24217	0.000826	0.003262
11727237_UST	0.55055	0.000827	0.003264
11754403_PARP14	0.293218	0.000827	0.003264
11740462_SMIM19	-0.26865	0.000828	0.003267
11750293_RAD51C	0.230233	0.000828	0.003267
11728626_HOXA4	-0.19909	0.000828	0.003268
11749667_AFAP1L2	0.30377	0.000829	0.003271
11729576_TM9SF4	0.313214	0.00083	0.003275
11730251_LNX1	0.278135	0.00083	0.003276
11724109_C9orf116	-0.27839	0.000831	0.003277
11758220_TOR1A	0.182636	0.000831	0.003278
11733214_SLFN5	0.258999	0.000832	0.003281
11723016_HM13	0.273445	0.000832	0.003282
11719018_CBFB	0.208342	0.000832	0.003282
11751089_GTF2H4 //	-0.22683	0.000832	0.003283
11747307_GDI1	0.228245	0.000832	0.003283
11739012_ASH2L	-0.20923	0.000833	0.003284

11717695_TYSND1	-0.26104	0.000833	0.003284
11746409_LPAR1	0.242892	0.000833	0.003284
11757506_PYGO2	-0.2185	0.000833	0.003284
11742844_RXRA	-0.21234	0.000833	0.003285
11758907_ZNF827	-0.24241	0.000835	0.003294
11726189_HCFC1R1	-0.20277	0.000836	0.003294
11751030_ZNF155	0.4943	0.000836	0.003294
11736061_SERPINB5	-0.57206	0.000836	0.003295
11718401_NUDT21	-0.22845	0.000836	0.003296
11744486_WDR81	-0.19033	0.000837	0.003298
11741136_CHN1	0.197465	0.000838	0.003301
11748313_MTFR1	-0.25895	0.000838	0.003301
11729051_PPFIBP1	-0.21961	0.000839	0.003305
11740022_LRRCC1	-0.38351	0.000839	0.003306
11732925_PEAR1	0.442329	0.000839	0.003306
11725236_ZBTB39	0.246385	0.000841	0.003312
11753982_PSMD11	-0.20776	0.000841	0.003312
200043_PMERH	-0.20688	0.000842	0.003315
11738257_GRK6	-0.36581	0.000843	0.003319
11720542_B3GALNT2	0.294035	0.000843	0.003319
11722576_AP1G1	0.195252	0.000843	0.00332
11751128_SHISA5	-0.21787	0.000844	0.003321
11752433_SMPD4	-0.23968	0.000844	0.003324
11716830_ATG9A	0.211185	0.000844	0.003324
11745774_AES	-0.20365	0.000845	0.003326
11720922_HCP5	-0.55372	0.000845	0.003327
11743449_BICD2	-0.22509	0.000846	0.00333
11744606_SLC35F3	-0.66954	0.000846	0.00333
11757332_DDX5	0.191848	0.000846	0.00333
11756104_SMC1A	-0.81348	0.000846	0.00333
11733110_TLK1	0.266639	0.000847	0.003331
11722809_SP1	0.353383	0.000847	0.003331
11754156_ARF5	-0.18896	0.000847	0.003332
11719821_CLN5	0.230183	0.000848	0.003334
11756407_RFX3	0.379778	0.000848	0.003334
11764018_CLCN3	-0.24634	0.000848	0.003336
11753181_BAZ2A	-0.26648	0.000849	0.003338
11733918_PSMD14	-0.27629	0.00085	0.00334
11719066_EIF2S3	0.186901	0.00085	0.003341
11735225_GAMT	-0.20642	0.00085	0.003341
11746513_RPRD2	-0.21015	0.000852	0.003347
11720307_SERP1	-0.26284	0.000852	0.003347
11738897_DAZAP2	-0.32162	0.000852	0.003348
11754304_CDK10	-0.25213	0.000852	0.003348
11725812_KLC1	0.208879	0.000853	0.00335
11756535_SLC25A45	0.245424	0.000854	0.003356
11733834_ANKMY2	0.193159	0.000855	0.003361
11731023_ATXN7	0.270008	0.000856	0.003361
11763367_NABP1	0.648167	0.000856	0.003361
11719503_DHX36	0.209414	0.000856	0.003362
11716527_GSTK1	0.195119	0.000856	0.003362

11733856_EGLN1	0.207431	0.000856	0.003362
11754023_COX7A2L	0.211359	0.000856	0.003362
11757866_FBXO38	0.213385	0.000857	0.003365
11730651_SYTL4	-0.29217	0.000857	0.003365
11756512_ZHX3	0.303121	0.000858	0.003368
11752845_ARHGEF7	0.300333	0.000858	0.003368
11728708_EMP2	0.205068	0.000858	0.003369
11740648_SPIRE1	-0.36993	0.000858	0.003369
11750167_CAPN2	0.209919	0.000859	0.003373
11716243_PPP1R16A	0.27947	0.00086	0.003374
11726300_KLHL26	-0.23981	0.000861	0.003379
11735721_KRT79	-0.2317	0.000861	0.00338
11718094_NPRL2	-0.19482	0.000862	0.003381
11718028_PUS1	0.259973	0.000862	0.003383
11731777_POMGNT2	-0.25533	0.000863	0.003384
11717729_SQLE	-0.30371	0.000864	0.003388
11719300_CTDSP1	-0.40794	0.000864	0.003388
11734840_GTF2F1	0.207251	0.000864	0.003388
11730572_CEP97	-0.278	0.000865	0.003391
11745923_MAOA	0.192341	0.000865	0.003393
11756214_PCMT1	-0.21453	0.000866	0.003394
11747757_RNF167	-0.18428	0.000866	0.003395
11715197_PYGO1	0.255718	0.000866	0.003396
11732415_TIAM1	0.225717	0.000867	0.0034
11716419_SPTBN1	-0.30813	0.000867	0.0034
11721827_ZWILCH	-0.20924	0.000868	0.003404
11754366_TMEM161B	0.2417	0.000869	0.003404
11726186_PARPBP	-0.20547	0.000869	0.003405
11746218_TEAD2	-0.2357	0.000869	0.003406
11760301_HEXA	0.246308	0.000869	0.003406
11750411_HSPA1A //	-0.19168	0.000869	0.003406
11759141_HIP1	-0.22344	0.000869	0.003406
11732328_RBM26	0.195356	0.000869	0.003406
11718066_TCTN1	0.225787	0.00087	0.003407
11750443_TNIP1	0.190201	0.00087	0.003409
11737850_PPP1R21	0.186331	0.000871	0.003409
11747427_SEC14L2	-0.19927	0.000871	0.003409
11723518_ELAC1	0.303889	0.000871	0.003413
11759810_PPP2R3C	0.461923	0.000872	0.003413
11754091_SMAD3	-0.22141	0.000872	0.003413
11730635_GRHL3	0.469257	0.000872	0.003414
11741142_TTI2	0.182489	0.000872	0.003414
11736383_METTL10	-0.3155	0.000873	0.003415
11737318_ZNF546	0.496534	0.000873	0.003415
11742927_GSTA4	-0.20723	0.000873	0.003416
11722007_PRKAR1A	0.191872	0.000873	0.003417
11754369_UPRT	-0.29098	0.000873	0.003417
11736977_ZNF431	0.293088	0.000875	0.003422
11729492_PPFIA4	-0.35023	0.000875	0.003424
11715530_CCT6A	0.192736	0.000876	0.003426
11723207_MBTPS2	-0.1905	0.000876	0.003428

11748869_CLN3	-0.19358	0.000876	0.003428
11752652_METTL16	-0.32323	0.000877	0.003429
11738937_SSX3	-0.2435	0.000877	0.003429
11746880_FLNA	-0.21729	0.000877	0.003429
11746163_LARP4B	0.252906	0.000877	0.003429
11728395_TAC1	0.214654	0.000877	0.003429
11730696_TBC1D26	-0.29962	0.000878	0.003433
11717920_YAP1	-0.27327	0.000878	0.003434
11757006_RNF4	0.254683	0.000879	0.003435
11727433_NUTF2	-0.24417	0.000879	0.003437
11736678_PODXL	0.361469	0.00088	0.003438
11715506_MAGED1	-0.20091	0.00088	0.003438
11739348_PIP4K2A	0.245031	0.00088	0.00344
11725775_TBC1D24	-0.25377	0.000881	0.003441
11755468_WASH2P	0.196034	0.000881	0.003441
11756730_ATP13A2	0.26866	0.000881	0.003443
11744541_ACKR3	-0.36416	0.000882	0.003446
11754021_GNPTG	0.279829	0.000882	0.003446
11750997_CERK	-0.21721	0.000883	0.003449
11721189_CNPY4	-0.25648	0.000883	0.003449
11721920_SENP5	0.4488	0.000884	0.003452
11752150_ADAM22	0.46459	0.000884	0.003453
11745381_COBLL1	0.24845	0.000884	0.003453
11751729_SAE1	-0.19267	0.000885	0.003456
11746484_FAR2P1 //	0.432326	0.000885	0.003457
11719349_PIK3R4	-0.23678	0.000886	0.003457
11747858_ABCE1	-0.19937	0.000886	0.003457
11738623_ZNF268	0.269756	0.000887	0.003462
11748470_MTA2	-0.20929	0.000888	0.003466
11746537_EIF4G3	0.200776	0.000888	0.003467
11752388_ARAF	-0.23091	0.000888	0.003467
11743976_MYL12A	0.240908	0.000888	0.003467
11746096_TLDC1	0.306713	0.000889	0.003469
11756291_CCDC53	-0.21922	0.00089	0.003472
11716775_RAI14	-0.24286	0.00089	0.003472
11749637_SEC24B	0.246247	0.00089	0.003473
11732494_AUNIP	-0.40658	0.000891	0.003474
11735349_NDRG3	-0.40605	0.000891	0.003474
11720757_IVNS1ABP	0.207598	0.000891	0.003475
11717723_RAB1A	-0.29596	0.000892	0.003477
11739619_PTPN3	0.32124	0.000892	0.003477
11721715_ATRX	-0.19643	0.000892	0.003479
11755710_GTPBP8	0.191234	0.000893	0.00348
11716815_SLC2A1	-0.23378	0.000893	0.00348
11718944_PGPEP1	0.236431	0.000893	0.00348
11740487_FBF1	-0.22278	0.000893	0.003481
11744995_PANK1	0.304804	0.000894	0.003482
11727430_LINC01590	-0.27105	0.000894	0.003482
11717644_GPR107	-0.21978	0.000895	0.003486
11725004_C8orf58	0.213969	0.000895	0.003486
11749348_SLC25A12	0.229941	0.000895	0.003488

11720431_COL15A1	0.445428	0.000896	0.00349
11747986_SS18	0.232918	0.000896	0.00349
11755424_HMOX2	0.184606	0.000896	0.00349
11753002_DMBT1	-0.2698	0.000897	0.003494
11758309_YWHAE	-0.20916	0.000897	0.003494
11732327_RBM26	0.18459	0.000898	0.003498
11757831_SNAP23	0.25744	0.000899	0.0035
11745863_LOC730098	-0.26275	0.000899	0.003501
11719807_BLOC1S6	0.221259	0.000899	0.003501
11720666_SYNCRIP	-0.19122	0.0009	0.003502
11725343_VPS37A	-0.53446	0.0009	0.003504
11755869_JAM3	-0.18675	0.000901	0.003506
11756072_SAA1 ///	0.392267	0.000901	0.003508
11750173_CLTCL1	0.336731	0.000902	0.00351
11732818_TMEM171	0.186372	0.000902	0.00351
11764062_RAPGEF2	0.217921	0.000903	0.003513
11722788_ENG	0.188654	0.000904	0.003516
11725371_VEGFA	-0.20497	0.000904	0.003517
11723159_TMEM229B	-0.2515	0.000905	0.00352
11720621_ACLY	-0.22046	0.000906	0.003522
11715722_GOLM1	0.253458	0.000906	0.003523
11736022_FKBP4	-0.59617	0.000906	0.003524
11728235_CD5	-0.29436	0.000906	0.003524
11751824_SNRPD2P2	-0.20648	0.000907	0.003525
11743072_FBXO34	0.235978	0.000908	0.00353
11746193_METTL3	-0.2467	0.000908	0.003531
11728747_ARAF	-0.26818	0.000909	0.003533
11758459_API5	0.210564	0.000909	0.003533
11733893_NPLOC4	0.247902	0.000909	0.003534
11731260_KCND1	-0.21655	0.00091	0.003536
11738440_FGF22	-0.2088	0.00091	0.003536
11756221_ASTN1	-0.23765	0.00091	0.003536
11726976_PERP	-0.42095	0.00091	0.003536
11720429_CDC40	0.18419	0.00091	0.003537
11727927_FOXO1	0.679874	0.000911	0.003539
11720484_MRPL9	-0.19906	0.000911	0.00354
11757338_GRHPR	0.202834	0.000912	0.003543
11763365_CARD8	-0.3262	0.000912	0.003544
11758554_RFWD2	0.1901	0.000913	0.003546
11744790_LRCH1	0.445058	0.000913	0.003546
11716816_ENOPH1	-0.19493	0.000914	0.003548
11763513_NF1	0.203582	0.000914	0.003549
11761351_SYNGR1	-0.23285	0.000914	0.003549
11740290_HOXC6	0.189733	0.000914	0.003549
11732933_LOC100506	0.258013	0.000914	0.003549
11740553_XPC	0.255299	0.000914	0.003549
11756592_CCDC115	-0.24329	0.000914	0.003549
11752133_CEP97	0.27769	0.000915	0.003551
11719822_CLN5	0.266906	0.000915	0.003551
11716924_PURB	0.272718	0.000916	0.003555
11746102_WDR76	-0.34184	0.000918	0.00356

11717140_COG4	0.198656	0.000918	0.003561
11733563_KLHDC10	0.207634	0.000918	0.003561
11755644_CDK11A //	0.211595	0.000918	0.003561
11717623_SUCO	-0.23899	0.000918	0.003562
11731882_ZNF540	0.27945	0.000918	0.003562
11751856_TIE1	-0.22296	0.000919	0.003562
11743036_SAT1	0.216041	0.000919	0.003562
11724569_USP48	-0.209	0.000919	0.003564
11756661_DARS	-0.19967	0.00092	0.003566
11740161_KCNK17	-0.29415	0.00092	0.003566
11757857_XIST	-0.20481	0.00092	0.003567
11724058_CPTP	-0.19281	0.00092	0.003567
11761170_ACAN	-0.21498	0.00092	0.003567
11716997_TSPAN4	0.218855	0.00092	0.003567
11731746_IPMK	0.371038	0.000921	0.003568
11757341_OPTN	0.29672	0.000921	0.00357
11723024_CREB3	0.188007	0.000922	0.003573
11763283_PIK3CA	0.23653	0.000923	0.003575
11740692_VPS13A	0.221703	0.000923	0.003576
11758405_SMO	-0.29226	0.000925	0.003582
11762286_WISP3	0.325278	0.000925	0.003582
11747215_C20orf24	-0.19496	0.000925	0.003582
11717449_ASCC2	0.189487	0.000925	0.003582
11723046_MALSU1	-0.18866	0.000925	0.003582
11719147_FAM117A	-0.3442	0.000925	0.003583
11728643_ACTR3	0.183363	0.000926	0.003584
11748218_ARFGAP2	-0.40033	0.000926	0.003584
11719596_EHD3	0.227806	0.000927	0.003588
11746565_CHSY1	0.29553	0.000928	0.003591
11735772_LOC440040	-0.46569	0.000928	0.003592
11720741_ZNF605	0.403285	0.000928	0.003592
11752172_FGFR4	-0.29863	0.000928	0.003593
11737690_ZP3	-0.2246	0.000929	0.003596
11754015_TIMM50	-0.21059	0.000929	0.003596
11753754_SNCG	-0.22287	0.000929	0.003596
11753178_LOC283788	-0.26396	0.00093	0.003596
11746368_FAM222B	0.278023	0.00093	0.003596
11742775_GPR161	-0.3738	0.00093	0.003596
11744514_YIPF2	-0.2224	0.00093	0.003599
11743932_NCAM2	-0.33452	0.000931	0.0036
11758156_SLC17A5	0.213537	0.000931	0.0036
11717896_PTP4A1	0.279481	0.000931	0.003602
11741053_LAMP2	-0.45909	0.000932	0.003602
11740991_TSPAN4	0.224961	0.000932	0.003603
11722053_NQO2	-0.23722	0.000932	0.003604
11733100_USP12	0.235301	0.000933	0.003606
11729166_DCTD	0.218903	0.000933	0.003606
11728460_HSCB	0.253734	0.000933	0.003606
11759332_ZNF117	0.50806	0.000933	0.003607
11719451_RAB14	0.230876	0.000933	0.003607
11722839_LYAR	-0.185	0.000934	0.003609

11727385_PCCA	0.213034	0.000935	0.003612
11747311_WBP11 ///	-0.20196	0.000936	0.003618
11730553_TICAM2 //	0.189485	0.000936	0.003618
11752096_CEP120	0.254153	0.000938	0.003623
11721808_IST1	0.228862	0.000938	0.003624
11741325_TRIM51	0.288118	0.000939	0.003628
11720214_C17orf75	-0.21925	0.000939	0.003628
11739096_CPSF7	-0.19458	0.00094	0.003628
11729850_MLLT10	0.232352	0.000941	0.003632
11742858_WBP11 ///	-0.17684	0.000941	0.003632
11718867_FAM192A	-0.20654	0.000941	0.003633
11741331_ANAPC11	-0.21923	0.000942	0.003638
11751155_PPP1R15B	0.207856	0.000943	0.003638
11752140_GIT1	-0.18472	0.000943	0.003642
11717615_OXSR1	0.268946	0.000944	0.003643
11747345_ACADM	0.199043	0.000944	0.003644
11720792_AACS	0.195582	0.000944	0.003645
11746034_FAM193A	0.239071	0.000944	0.003645
11717961_MED11	-0.23708	0.000945	0.003646
11758283_MGA	0.219372	0.000945	0.003648
11746265_FAR2P1	0.217818	0.000946	0.003649
11754765_KPNA2 ///	-0.19732	0.000946	0.00365
11717421_SLC35A2	-0.25424	0.000946	0.003651
11731153_MTRR	0.285804	0.000947	0.003652
11720529_JAZF1	0.639724	0.000947	0.003652
11722914_BMPR1A	0.22424	0.000947	0.003653
11749393_ANP32E	-0.18753	0.000947	0.003653
11747270_NELFCD	-0.20972	0.000948	0.003656
11716070_DESI2	-0.22423	0.000949	0.003659
11729077_AKAP5	0.25882	0.000949	0.003659
11750782_ATG7	0.260658	0.00095	0.003661
11719647_CASP7	-0.28318	0.00095	0.003661
11755143_BNC2	-0.23973	0.00095	0.003662
11748861_ACPP	0.400237	0.000951	0.003664
11747664_UBXN8	0.194349	0.000951	0.003664
11724410_ 2-Mar	-0.19385	0.000951	0.003664
11716334_RBX1	-0.22502	0.000951	0.003664
11759654_SLC6A13	0.373873	0.000951	0.003664
11754411_CLUAP1	0.263529	0.000951	0.003664
11741463_PRR4	0.274585	0.000951	0.003664
11743925_SBNO1	0.181474	0.000951	0.003664
11715425_FLNA	-0.18415	0.000952	0.003666
11753579_IL6ST	0.363124	0.000952	0.003666
11725840_SLC24A1	-0.28021	0.000952	0.003666
11724280_ING3	0.208629	0.000954	0.003674
11735511_PHTF2	0.223785	0.000954	0.003674
11723401_STK4	0.175984	0.000955	0.003676
11754655_UBE4B	0.217827	0.000955	0.003676
11719124_TIMELESS	-0.29619	0.000957	0.003684
11739732_CSNK1G1	0.371991	0.000957	0.003685
11745259_C11orf21	-0.24812	0.000957	0.003685

11722209_PTGS1	0.263728	0.000957	0.003685
11763395_ZC3HAV1L	0.296851	0.000958	0.003686
11731099_DNPH1	-0.20156	0.000958	0.003687
11747575_SORBS3	-0.39349	0.000958	0.003687
11716385_TGFB1	-0.21188	0.000958	0.003687
11718024_IFT52	0.179035	0.000958	0.003687
11744173_DNAJC4	-0.18513	0.000959	0.00369
11722213_NPM3	-0.18646	0.000959	0.00369
11734881_TAF15	0.23194	0.00096	0.003691
11761299_SCNM1 ///	0.20175	0.00096	0.003691
11719288_PEX11B	-0.26566	0.00096	0.003691
11733484_PCLO	0.324057	0.000961	0.003695
11741696_NSUN5P1	0.304813	0.000962	0.003697
11719938_CAND1	0.213525	0.000962	0.003699
11759724_ZHX1-C8or	0.362574	0.000962	0.003699
11750973_ALAS1	-0.183	0.000962	0.003699
11744990_TOR3A	-0.70801	0.000962	0.003699
11715449_ERGIC3	0.210998	0.000963	0.003702
11740125_HIST1H2AM	-0.32602	0.000963	0.003702
11740179_CDYL2	0.268509	0.000964	0.003705
11739687_FAM107B	-0.23625	0.000965	0.003706
11754008_ID3	-0.19509	0.000965	0.003707
11757121_EIF4A2 //	-0.31577	0.000965	0.003708
11754039_CNBP	0.193087	0.000965	0.003709
11758276_ITGB1	-0.17527	0.000967	0.003715
11739013_ASH2L	-0.22521	0.000967	0.003715
11726166_ZNF518B	0.258349	0.000967	0.003716
11716597_SEC24B	0.200353	0.000968	0.003716
11748521_SURF4	-0.2366	0.000968	0.003717
11750054_PRIM2	-0.34362	0.000968	0.003718
11741663_EIF3C ///	-0.20942	0.000968	0.003718
11728459_CDH10	0.270416	0.000969	0.003718
11736664_JDP2	-0.28206	0.000969	0.003718
11717306_ADIRF	-0.21614	0.000969	0.003718
11743315_TXNL1	0.1923	0.000969	0.003721
11735592_MYO3B	-0.21945	0.00097	0.003722
11755036_CNP	-0.21841	0.000972	0.003729
11745302_STARD3	-0.31776	0.000972	0.00373
11718679_PSEN1	-0.27302	0.000973	0.003733
11736076_URM1	-0.17694	0.000973	0.003735
11724789_KIF3A	0.264104	0.000974	0.003737
11722375_GGA2	0.190058	0.000974	0.003737
11721125_SPPL2A	0.21515	0.000975	0.003739
11759562_PPP4R2	0.205428	0.000975	0.003741
11738036_APLP2	-0.23116	0.000976	0.003743
11728702_KIAA0232	0.218261	0.000976	0.003744
11726512_TCHP	-0.21495	0.000977	0.003747
11732719_EREG	0.323635	0.000978	0.00375
11719481_FABP7	-0.26262	0.000978	0.003751
11749320_TGDS	-0.22792	0.000978	0.003751
11722625_FASTKD1	-0.22457	0.000979	0.003751

11720731_SUMF1	0.215041	0.000979	0.003754
11721096_TMX1	-0.32514	0.000979	0.003754
11742854_UGDH	-0.18877	0.00098	0.003755
11729314_PRKAA1	-0.47929	0.00098	0.003757
11719619_TMEM187	-0.46882	0.000981	0.003759
11743142_COX11	-0.22549	0.000981	0.003759
11763422_ATP6V0D1	0.233564	0.000981	0.00376
11727792_DLAT	-0.20946	0.000982	0.00376
11750988_ILF3	-0.23514	0.000982	0.003761
11735679_ACOT8	0.260421	0.000982	0.003761
11746933_ATP6V0A2	0.240981	0.000982	0.003761
11718886_TPRKB	-0.3524	0.000982	0.003761
11760618_ZXDC	-0.29753	0.000983	0.003765
11759832_IL15	0.563892	0.000983	0.003765
11728286_RBMS2	-0.25608	0.000983	0.003765
11739415_IRGQ	0.300487	0.000985	0.003769
11754196_XIST	0.200506	0.000985	0.00377
11737954_SLC16A5	-0.17543	0.000985	0.003772
11749287_ARL2BP	0.182838	0.000986	0.003773
11760216_TMEM170A	0.439703	0.000986	0.003773
11760938_MAT2A	0.278176	0.000986	0.003774
11721304_RAMP1	0.224509	0.000987	0.003775
11725153_NFKB2	0.283469	0.000987	0.003775
11747545_BMP1	0.335856	0.000987	0.003775
11752355_KIAA1217	0.319637	0.000988	0.00378
11745460_NDUFV3	0.194696	0.000988	0.00378
11737913_CCT7	-0.19259	0.000988	0.003781
11737861_ZSCAN5A	-0.31017	0.000988	0.003781
11744428_SYAP1	0.185978	0.000989	0.003781
11744351_LOC728392	-0.21916	0.000989	0.003781
11727996_MYEF2	0.349431	0.000989	0.003782
11723748_ALPP	-0.50516	0.00099	0.003784
11760229_KLHL23 //	-0.46459	0.00099	0.003785
11715344_GSTP1	-0.1959	0.00099	0.003785
11719966_U2AF1 ///	-0.19067	0.000992	0.003793
11723084_SCN1B	0.24614	0.000993	0.003796
11719638_NEK3	0.336446	0.000995	0.003803
11724089_MXD3 ///	0.203726	0.000995	0.003803
11730099_FLT3LG	-0.24829	0.000995	0.003804
11742031_PPIP5K1	-0.26374	0.000996	0.003806
11757315_TAB2	0.219025	0.000996	0.003807
11718365_LYPLA1	0.187261	0.000996	0.003807
11728633_AP4E1	0.518238	0.000996	0.003807
11724845_NR2C2AP	-0.33849	0.000996	0.003807
11749406_TMEM54	-0.25442	0.000997	0.003809
11744089_SH2B2	-0.21439	0.000999	0.003814
11727552_FZD2	-0.26544	0.000999	0.003817
11728444_TAOK1	-0.30104	0.001	0.003818
11764006_TXNL1	0.178842	0.001	0.00382
11752302_LAMB3	0.272205	0.001	0.00382
11736919_ATAD5	-0.27083	0.001001	0.00382

11752870_LRRCC1	-0.35966	0.001002	0.003826
11754157_JMJD8	-0.37212	0.001003	0.003829
11717545_MRPS5	-0.20395	0.001003	0.00383
11760814_KANK3	0.209382	0.001003	0.00383
11759037_IL6ST	0.227208	0.001004	0.003833
11715717_MSN	-0.17744	0.001004	0.003833
11723860_MPP6	0.186004	0.001005	0.003835
11752366_RABEP2	-0.43269	0.001005	0.003836
11748552_STX5	-0.23249	0.001006	0.003837
11757181_FGFR4	-0.30967	0.001006	0.003837
11763326_FER	-0.22897	0.001006	0.003837
11752385_NGEF	-0.25448	0.001006	0.003838
11732994_RGS4	0.342738	0.001006	0.003839
11723814_ARHGAP29	-0.19028	0.001007	0.003841
11747452_ABLIM1	0.335352	0.001008	0.003843
11726384_FRY	-0.20404	0.001008	0.003845
11718372_NRBF2	0.201306	0.00101	0.003851
11728141_SHISA2	-0.17949	0.00101	0.003851
11740841_NFIA	0.252697	0.001012	0.003857
11735137_WIPI2	0.220441	0.001012	0.003857
11719592_GLTP	0.205683	0.001012	0.003857
11755779_ADAMTS20	0.36872	0.001013	0.00386
11746363_SYNGR2	0.207988	0.001014	0.003863
11742904_BAG2	0.276327	0.001014	0.003863
11763853_FAAP20	-0.56207	0.001014	0.003866
11741784_BCAS4	-0.2303	0.001015	0.003866
11725845_SREK1IP1	0.211977	0.001015	0.003866
11763451_SSU72	-0.20253	0.001015	0.003867
11726624_CCNG2	-0.1985	0.001015	0.003868
11746220_ATG4D	-0.36762	0.001016	0.003872
11749876_LAMP2	-0.32344	0.001017	0.003873
11742798_CLCN3	0.222082	0.001017	0.003874
11746440_GLO1	-0.21628	0.001018	0.003879
11730080_IFNLR1	-0.32572	0.001019	0.00388
11715610_ARPC1A	0.219367	0.001019	0.003881
11733512_MTMR1	0.1987	0.00102	0.003882
11733282_KRBOX4 //	0.257141	0.00102	0.003882
11758802_ENY2	0.262258	0.00102	0.003883
11753116_SOX10	-0.2401	0.00102	0.003884
11716511_SRRM2	-0.22587	0.00102	0.003884
11743817_TEAD1	0.239322	0.001021	0.003886
11742330_OR9K2	-0.29069	0.001021	0.003887
11722729_METTL23	-0.17848	0.001023	0.003893
11743553_KIN	0.251673	0.001023	0.003893
11745933_EIF4E2	0.200926	0.001023	0.003893
11718767_PRSS23	0.234434	0.001023	0.003893
11743370_PPP1R2	0.233724	0.001024	0.003895
11747680_THRA	-0.33779	0.001024	0.003896
11715482_MCL1	-0.22056	0.001024	0.003897
11736953_SETDB2	0.586534	0.001025	0.003897
11732195_KPNA4	0.174733	0.001025	0.003898

11718832_LOXL2	0.195215	0.001026	0.0039
11755446_ANKH ///	0.17604	0.001026	0.003902
11725183_KLF12	-0.19232	0.001026	0.003902
11727787_CACYBP	-0.31442	0.001027	0.003903
11723073_BCL9L	0.227898	0.001027	0.003903
11718700_C6orf89	-0.35757	0.001028	0.003908
11716405_HTRA1	-0.2165	0.001029	0.00391
11716027_SELT	0.214424	0.001029	0.003912
11721308_PHLDA1	0.19337	0.001029	0.003912
11748770_PMPCA	0.188076	0.00103	0.003915
11742039_PTK7	-0.24385	0.001031	0.003919
11730055_NPC1L1	-0.18741	0.001032	0.00392
11726174_SAMD5	0.224676	0.001032	0.00392
11729735_SPECC1	0.264743	0.001032	0.003922
11718040_SNRPE	-0.1807	0.001033	0.003924
11717104_GRHPR	0.200763	0.001033	0.003925
11728391_LRP12	0.230187	0.001035	0.003929
11753127_ARNTL	0.560151	0.001035	0.003931
11756651_TAF12	-0.22261	0.001035	0.003931
11716579_CYSTM1	0.194582	0.001036	0.003933
11750044_NFS1	-0.22406	0.001038	0.00394
11758617_TRMT10B	0.266598	0.001039	0.003943
11730287_ARHGAP5	-0.23362	0.001039	0.003945
11743441_PDIA3	0.179191	0.001039	0.003945
11718323_ACAP2	0.426857	0.00104	0.003946
11736507_FRMD6	0.207481	0.001041	0.003949
11733139_ARL4A	0.281695	0.001041	0.003949
11757404_ID3	-0.20452	0.001041	0.00395
11740347_NRG1	0.282231	0.001042	0.003954
11746915_ACAD8	0.20958	0.001043	0.003955
11754710_ZNF185	-0.27679	0.001043	0.003955
11736996_PPT2	-0.21577	0.001043	0.003955
11719086_SMARCC2	-0.22484	0.001043	0.003956
11749409_DMKN	0.392948	0.001044	0.003961
11741793_FUOM	-0.31541	0.001044	0.003961
11728509_FKTN	0.353752	0.001044	0.003961
11735107_CENPO	-0.22283	0.001045	0.003961
11744690_ARMC10	0.221739	0.001046	0.003965
11754632_THAP8	-0.25147	0.001046	0.003968
11715736_CASC3	-0.19453	0.001047	0.003971
11730451_SDHAF4	-0.19899	0.001049	0.003975
11748206_ZSWIM4	0.262285	0.001049	0.003977
11728804_NAA16	0.372187	0.001049	0.003977
11755678_ZNF257	0.213162	0.00105	0.003978
11752328_IGF1R	0.390888	0.00105	0.003978
11743758_ACO1	0.185919	0.001052	0.003985
11722967_ZDHHC8	-0.25161	0.001052	0.003986
11731658_TRAM1L1	-0.35376	0.001054	0.003992
11730873_RASSF5	-0.28908	0.001054	0.003993
11726593_PIGH	0.359797	0.001055	0.003997
11715694_COX7B	-0.17869	0.001056	0.003999

11759384_DEFB128	-0.22808	0.001056	0.004
11762418_SCAND2P	0.182943	0.001056	0.004001
11741216_APP	0.201075	0.001056	0.004001
11717483_PDHA1	-0.18607	0.001057	0.004001
11733697_CTAG1A //	-0.22398	0.001057	0.004002
11753390_RAB7A	0.178962	0.001057	0.004002
11728909_B3GLCT	-0.22938	0.001057	0.004003
11715593_YWHAH	-0.21512	0.001057	0.004003
11749659_HSPH1	0.172253	0.001058	0.004003
11717781_DHX35	-0.24826	0.001058	0.004003
11754638_USP30	-0.31172	0.001058	0.004006
11732255_ZBTB33	0.178537	0.001059	0.004009
11723033_NOV	-0.24769	0.00106	0.004009
11756531_MEF2C	0.444925	0.00106	0.004009
11757355_RPL41	-0.17325	0.00106	0.004009
11746504_ICA1	0.35071	0.00106	0.004011
11743781_XRN1	-0.18756	0.00106	0.004011
11760313_CLK4	0.299245	0.001061	0.004014
11757808_RERE	-0.18593	0.001062	0.004015
11750091_TPRKB	-0.35009	0.001062	0.004015
11735217_ELF2	0.218013	0.001063	0.004019
11730193_SEC14L2	0.327434	0.001063	0.004019
11729790_CUZD1 ///	0.356379	0.001063	0.004019
11729184_RPAP3	-0.2112	0.001063	0.00402
11717879_PPFIA1	0.179499	0.001064	0.004022
11754151_STXBP5	0.275584	0.001065	0.004026
11747127_RPRD1B	0.495656	0.001066	0.004029
11733017_PIK3R1	0.266238	0.001066	0.00403
11746759_HSD52	0.194118	0.001066	0.00403
11757854_PLEKHA5	0.240617	0.001068	0.004034
11756875_COMMD6	0.199075	0.001069	0.004038
11719801_C6orf62	-0.23657	0.001069	0.004038
11725891_ABHD14A-A	-0.19006	0.001069	0.004041
11724428_ZNF337	0.248768	0.001071	0.004045
11735936_IFNK	0.300155	0.001071	0.004046
11726359_KMT2E	0.173114	0.001072	0.004048
11746934_SPIRE1	-0.35883	0.001072	0.004048
11730714_XRCC6	-0.26708	0.001072	0.004048
11736554_ZNF223	0.220961	0.001072	0.004048
11734039_FEZ1	0.265742	0.001073	0.004051
11716106_TOMM22	-0.23084	0.001074	0.004056
11758737_PGRMC2	-0.19508	0.001075	0.004059
11739866_GPR107	0.184933	0.001076	0.004061
11723895_TEP1	0.250987	0.001077	0.004064
11744142_SARS	0.209983	0.001077	0.004064
11760390_CEP78	-0.35036	0.001077	0.004065
11748391_ZDHHC16	0.226492	0.001077	0.004066
11760327_BPTF	0.18274	0.001078	0.004067
11735260_ERLIN2	-0.27616	0.001078	0.004068
11754229_ANAPC11	-0.21431	0.001078	0.004069
11748926_GANAB	-0.27414	0.001079	0.00407

11745879_SARAF	0.206055	0.001079	0.004071
11757458_LRRC8A	-0.2708	0.001079	0.004071
11751019_FBXL20	-0.20229	0.001079	0.004072
11721013_WTAP	0.21049	0.00108	0.004072
11754553_LRRC41	-0.23669	0.00108	0.004075
11720280_KCNMA1	-0.31265	0.001081	0.004076
11720132_SPIRE1	-0.46334	0.001081	0.004078
11756299_SNRPA	-0.20942	0.001081	0.004078
11753935_ATP6V1G3	0.405255	0.001082	0.004078
11749772_C6orf118	0.197502	0.001082	0.004078
11753530_CLTA	0.173928	0.001083	0.004081
11755065_ITGB3	0.241671	0.001083	0.004081
11715926_PLEC	0.196617	0.001083	0.004081
200072_PMHNRNPM	-0.19328	0.001083	0.004081
11740157_SH2D1B	0.236937	0.001084	0.004086
11750729_PARN	-0.4169	0.001085	0.004087
11741959_IL6R	0.380692	0.001086	0.004092
11717531_UBA2	-0.24985	0.001086	0.004092
11762048_EZH2	-0.20674	0.001086	0.004093
11748107_UBP1	0.307643	0.001088	0.004098
11755167_LINC00847	-0.20584	0.001089	0.004101
11745563_DNAJC4	-0.3012	0.001089	0.004101
11733936_PAIP1	-0.21846	0.001089	0.004101
11716729_EMC3	0.174678	0.001089	0.004103
11750695_RECQL	-0.21137	0.00109	0.004105
11716709_DDR1	-0.21487	0.00109	0.004106
11726257_RNF141	-0.44288	0.00109	0.004106
11718228_R3HDM4	0.237185	0.00109	0.004106
11724108_FAM58A	-0.26841	0.001091	0.004106
11725246_SDK1	0.449873	0.001091	0.004108
11747592_ERI3	-0.22951	0.001093	0.004113
11758673_SORD	-0.18321	0.001093	0.004113
11731907_FBXO11	-0.76399	0.001093	0.004114
11736600_DLG3	-0.28494	0.001094	0.004118
11716165_MGST3	-0.20576	0.001094	0.004118
11750913_ME1	0.217706	0.001095	0.00412
11743631_ALMS1	-0.22691	0.001096	0.004122
11749894_DCK	-0.24967	0.001096	0.004125
11762480_SP140	0.276045	0.001097	0.004126
11729228_GRK5	0.394999	0.001098	0.00413
11754878_UBAP1	0.185205	0.001098	0.004131
11756994_GNB2	-0.22667	0.001098	0.004131
11727179_MTHFR	0.272721	0.001099	0.004132
11754303_RAD21	-0.32021	0.001099	0.004133
11726401_ALG9	0.378718	0.0011	0.004135
11724535_UBA5	0.200461	0.0011	0.004135
11718969_DDX6	-0.24959	0.0011	0.004135
11760798_CIB2	-0.21852	0.0011	0.004136
11726711_PFAS	-0.17997	0.001101	0.004138
11718406_TMBIM6	0.18778	0.001101	0.004139
11756987_TWF2	-0.2516	0.001103	0.004146

11742703_C17orf89	0.185558	0.001103	0.004146
11724332_C6orf226	-0.24818	0.001104	0.004148
11727622_VIT	0.300327	0.001104	0.004148
11754477_GNL1	-0.25604	0.001105	0.00415
11735773_TRIM48	-0.32712	0.001105	0.00415
11753092_YWHAE	-0.20286	0.001105	0.00415
11754098_AGO2	0.182038	0.001106	0.004154
11724223_YIPF4	0.22488	0.001106	0.004155
11761311_IFT20	0.411791	0.001107	0.004159
11763230_FBXO11	0.264521	0.001108	0.004162
11746340_NOL8	0.256925	0.00111	0.004167
11717320_OAF	-0.21177	0.00111	0.004168
11745512_ANO3	0.447351	0.00111	0.004168
11718963_NUFIP2	-0.1989	0.001111	0.004173
11725983_BHLHE40	0.368819	0.001112	0.004173
11750259_ATXN2L	-0.2289	0.001112	0.004174
11736849_FSD1L	0.40857	0.001112	0.004174
11740780_FOXD2	-0.24875	0.001112	0.004175
11729527_POMT2	-0.28356	0.001113	0.004175
11761473_SPATA41	-0.3273	0.001113	0.004175
11735476_PALM2	0.419939	0.001114	0.004179
11717030_PACS1	-0.2596	0.001115	0.004182
11746199_METTL9	-0.17409	0.001115	0.004182
11735681_CD46	0.239703	0.001115	0.004185
11746587_TMEM9B	0.25846	0.001116	0.004185
11756655_MCAT	-0.25146	0.001116	0.004186
11744993_SIRT7	0.207548	0.001117	0.004189
11751668_AGPAT1	-0.21734	0.001117	0.004189
11717259_STX5	-0.18007	0.001118	0.004192
11721494_WDR33	-0.27021	0.00112	0.004199
11752497_MEF2D	0.40578	0.001121	0.004201
11717923_YAP1	-0.1729	0.001122	0.004205
11750095_FAM65C	-0.2559	0.001122	0.004205
11730808_FPGT	-0.20671	0.001122	0.004205
11743476_SLC25A13	0.360405	0.001122	0.004206
11720123_GIGYF1	-0.2439	0.001122	0.004207
11715448_ERGIC3	0.202404	0.001123	0.004208
11728403_SHCBP1	-0.1949	0.001124	0.004213
11745258_RBM14	-0.19875	0.001126	0.00422
11754734_ATP6V1C2	-0.18383	0.001127	0.004222
11735394_XCL1 ///	0.260058	0.001127	0.004223
11755613_FERMT1	-0.18486	0.001129	0.004228
11734670_KPNA1	-0.35777	0.00113	0.004232
11726890_MAPKAP1	-0.18352	0.00113	0.004233
11746447_FAM76B	0.22878	0.00113	0.004233
11719879_UBXN2B	0.538881	0.001131	0.004234
11725252_FAM120C	-0.29186	0.001131	0.004234
11752636_TRPC4AP	0.213775	0.001131	0.004234
11763280_TMEM136	0.370298	0.001131	0.004234
11747565_PPP2R4	-0.27601	0.001131	0.004234
11728198_ALG1	0.200289	0.001131	0.004236

11736732_DPY19L3	0.378328	0.001132	0.004236
11730730_PTRF	-0.19804	0.001132	0.004237
11751013_GLRB	0.255108	0.001132	0.004238
11745505_ITSN1	-0.20833	0.001133	0.004239
11737498_SLC25A53	-0.21174	0.001133	0.00424
11733120_SLC35B4	0.261272	0.001134	0.004244
11750837_SCYL3	0.249551	0.001136	0.004252
11757766_MAP1B	0.23672	0.001138	0.004256
11747305_CD1E	-0.19632	0.001138	0.004257
11763805_LSM14B	-0.32625	0.001138	0.004258
11727556_GRHL1	0.32334	0.001138	0.004258
11723698_OAS2 ///	-0.33148	0.001139	0.004258
11716409_PKDCC	-0.20008	0.001139	0.004261
11728532_FCER1A	-0.24783	0.00114	0.004263
11725489_ATG14	0.2295	0.00114	0.004264
11762384_GOLGA4	0.372157	0.001141	0.004267
11719436_ZNF746	0.269918	0.001143	0.004273
11734483_TADA2A	-0.1881	0.001143	0.004273
11737224_MAS1	0.198703	0.001144	0.004275
11755428_POLK	0.27429	0.001145	0.004278
11761685_IST1	0.268504	0.001145	0.004281
11743063_PLAUR	0.234281	0.001147	0.004286
11747364_CYB561A3	-0.19675	0.001147	0.004287
11720195_CLPB	-0.28202	0.001147	0.004288
11759061_SIK2	-0.32324	0.001149	0.004292
11726276_SNN	-0.40894	0.001149	0.004295
11750201_FLJ10038	-0.41754	0.00115	0.004295
11764037_DNAJA1	0.195256	0.00115	0.004298
11726911_MCC	0.343607	0.001152	0.004301
11722444_ELP5	-0.19405	0.001153	0.004305
11742892_PSMA4	-0.17717	0.001153	0.004305
11724025_TRIM13	0.244109	0.001154	0.004308
11727302_TMEM71	0.635923	0.001154	0.004308
11741914_ISM2	0.227626	0.001154	0.004308
11752356_ABCC4	0.321805	0.001154	0.004308
11754718_CCDC47	0.215726	0.001154	0.004309
11720759_IVNS1ABP	0.312653	0.001154	0.004309
11754888_ADSSL1	0.422181	0.001156	0.004314
11738077_ATP6V0A4	0.39833	0.001156	0.004314
11761992_KDM4B	-0.23879	0.001156	0.004315
11737337_ZNF527	0.296756	0.001156	0.004315
11758949_ASXL2	0.255197	0.001157	0.004316
11739905_ZNF583	0.298574	0.001157	0.004316
11755296_PSAP	0.18744	0.001157	0.004316
11756653_FGF1	0.227431	0.001157	0.004317
11757681_RNASEH2C	-0.20605	0.001158	0.004318
11759513_LZIC	-0.30133	0.001158	0.004319
200047_PM_YY1	0.187273	0.001159	0.004321
11732505_TP73-AS1	-0.25225	0.001159	0.004321
11720617_TRIM9	0.222149	0.001161	0.004328
11741358_PTPN4	0.272768	0.001161	0.004328

11749713_KBTBD6	-0.36322	0.001161	0.004328
11718086_SHKBP1	-0.38851	0.001162	0.004332
11733465_XDH	0.237203	0.001162	0.004333
11741304_NRG1	0.339651	0.001162	0.004333
11744270_SNRPA	-0.25248	0.001163	0.004335
11721043_ERI1	0.193882	0.001164	0.004337
11741783_BNIP1	-0.19595	0.001164	0.004339
11756311_PPT2	-0.19719	0.001164	0.004339
11752117_ACAD11 //	-0.26537	0.001165	0.004341
11735695_ARF5	-0.19108	0.001165	0.004343
11720136_ATP13A3	0.191586	0.001166	0.004346
11722124_RNF219	-0.23566	0.001167	0.004348
11732735_CHSY3	-0.54812	0.001167	0.004348
11741679_SERPINE2	0.207189	0.001168	0.004352
11728521_ART3	-0.18293	0.001169	0.004353
11717802_ATF5	-0.29109	0.00117	0.004357
11723590_TTC13	0.243317	0.00117	0.004357
11752162_IQSEC3	-0.39537	0.001171	0.00436
11744135_MIB2	0.244313	0.001171	0.004362
11722697_SFI1	-0.28941	0.001172	0.004363
11758036_TDG	0.193035	0.001172	0.004365
11727778_GNAI3	0.193567	0.001172	0.004365
11722437_GOSR2	0.32046	0.001172	0.004365
11743035_DDX1	0.180917	0.001173	0.004365
11721219_CPS1	0.32642	0.001174	0.004368
11725892_PLEKHH1	0.409176	0.001174	0.004369
11723793_OSBPL11	0.177499	0.001174	0.004369
11733232_HPS1	-0.18628	0.001174	0.004369
11733986_TUBA3E	-0.24042	0.001175	0.004372
11749427_MTM1	0.248547	0.001175	0.004372
11763689_FMNL1	-0.23699	0.001175	0.004372
11756108_CHAF1B	-0.21912	0.001176	0.004374
11741636_OR2W3 ///	0.447836	0.001176	0.004374
11717556_POM121 //	-0.22966	0.001176	0.004375
11727717_ZBTB42	-0.46661	0.001176	0.004375
11721340_NCOA1	0.324904	0.001178	0.004381
11738272_PSMG4	-0.17707	0.001179	0.004385
11739631_RPGR	0.316105	0.00118	0.004386
11726646_CDHR5	-0.18875	0.00118	0.004387
11727671_SYT17	-0.22022	0.00118	0.004388
11725344_VPS37A	-0.2182	0.001181	0.004389
11723508_ZNF609	0.487164	0.001181	0.004389
11716116_NDUFS6	-0.19046	0.001181	0.004391
11724140_CRIPAK	-0.22189	0.001182	0.004392
11743856_CTHRC1	0.194316	0.001182	0.004394
11757152_SNORA18 /	-0.17009	0.001183	0.004395
11719372_NGFRAP1	-0.21111	0.001183	0.004396
11757294_RABGGTB	0.205021	0.001183	0.004397
11733549_TEC /// T	0.380148	0.001185	0.004403
11715969_EPRS	-0.18845	0.001186	0.004404
11757511_HLA-DPA1	-0.18843	0.001186	0.004405

11763131_IGKC	-0.28261	0.001186	0.004406
11748133_SLC2A11	0.236053	0.001186	0.004406
11718510_UBE2L3	-0.45654	0.001186	0.004406
11756768_TEKT3	0.377401	0.001187	0.004406
11750282_DBNL	-0.22109	0.001187	0.004406
11745408_TMEM161B	0.32722	0.001187	0.004406
11727968_ESCO2	-0.28158	0.001187	0.004406
11747745_HADHB	-0.20282	0.001187	0.004406
11756614_RNF145	0.17258	0.001187	0.004406
11762466_GOLGA2P10	-0.25271	0.001187	0.004407
11730303_FANCA	-0.21725	0.001188	0.004409
11730737_SCCPDH	-0.19726	0.001188	0.004409
11729457_BCL6B	-0.28451	0.001188	0.004409
11751448_DDB1	-0.23885	0.001189	0.004412
11760501_WSB1	-0.22596	0.00119	0.004416
11754975_USP30	0.183348	0.001191	0.004419
11733382_AFAP1	0.276761	0.001192	0.004422
11722317_PTPN13	0.29305	0.001194	0.004429
11752915_RYR2	0.525174	0.001194	0.00443
11748228_BPNT1	-0.19581	0.001195	0.004434
11715971_PFKP	-0.2	0.001196	0.004435
11725952_RNF10	-0.25481	0.001197	0.004437
11715400_SLC25A5	-0.17257	0.001199	0.004448
11724237_LAT2	0.268236	0.0012	0.004448
11758058_DPY19L4	0.178234	0.0012	0.004449
11726262_DFFA	-0.20778	0.001201	0.004452
11758383_USP6NL	0.383954	0.001202	0.004454
11731996_TRIM58	0.187814	0.001202	0.004455
11758788_PHF5A	-0.41258	0.001202	0.004455
11720127_ITPKC	0.459662	0.001203	0.004458
11726017_FAM222B	0.184308	0.001203	0.00446
11721467_CD276	-0.32206	0.001204	0.00446
11717093_TCTA	0.193033	0.001204	0.004461
11757618_IFT43	0.318036	0.001205	0.004464
11749295_GPR161	-0.37578	0.001205	0.004464
11739559_RFWD3	-0.22611	0.001206	0.004469
11727403_GPC4	-0.27924	0.001207	0.00447
11743583_PRELID3B	-0.19297	0.001208	0.004473
11739591_MINA	-0.23395	0.00121	0.00448
11742679_HNRNPA2B1	-0.23757	0.001211	0.004485
11759223_EDF1	-0.33819	0.001211	0.004486
11716266_UBE2A	-0.1879	0.001214	0.004497
11752984_LOC100996	0.219041	0.001216	0.004504
11739899_SLC19A3	0.521407	0.001217	0.004504
11744826_WBSCR22	0.222461	0.001217	0.004505
11743006_RECQL	-0.33527	0.001218	0.004509
11750374_RAPGEF6	0.209556	0.001219	0.004512
11733212_EDARADD	-0.28418	0.001219	0.004513
11733085_ENO3	0.243083	0.001219	0.004513
11715584_CAV1	0.178051	0.00122	0.004515
11745715_CAV1	0.178051	0.00122	0.004515

11732387_TCEANC2	0.211482	0.001221	0.004517
11739884_DOCK7	0.222566	0.001221	0.004518
11724919_HSD17B8	-0.30969	0.001221	0.004519
11740380_DDX55	0.185658	0.001223	0.004523
11750414_RARA	0.268955	0.001223	0.004524
11726234_GIT2	0.27953	0.001224	0.004527
11753045_MBNL2	0.224437	0.001225	0.004531
11732130_EIF4E	-0.39588	0.001226	0.004534
11753998_CD58	0.197656	0.001229	0.004546
11755170_DPY30 ///	0.176212	0.00123	0.004547
11737326_SLC6A9	-0.36265	0.00123	0.004548
11717042_GID8	-0.28429	0.00123	0.004548
11748707_NDUFA10	-0.22841	0.001232	0.004554
11744696_CCDC90B	0.274522	0.001234	0.004561
11733119_SLC35B4	0.251729	0.001234	0.004563
11721521_ZDHHC17	0.528234	0.001235	0.004563
11730523_ISM1	-0.21213	0.001235	0.004564
11742138_UBE2E3	0.186554	0.001235	0.004565
11724967_USP31	0.36736	0.001235	0.004565
11756771_SPTY2D1	0.222689	0.001235	0.004565
11718001_C10orf54	0.263354	0.001236	0.004566
11735167_RFC2	-0.25044	0.001237	0.00457
11720503_ALDH3A2	-0.22591	0.001237	0.00457
11759770_UCHL5	0.231144	0.001238	0.004571
11727158_STRBP	0.249235	0.001238	0.004571
11754264_KHSRP	-0.19647	0.00124	0.00458
11728394_ANO4	0.343622	0.00124	0.00458
11718432_SASH1	0.196207	0.001241	0.004583
11725568_ATP8A1	-0.28996	0.001242	0.004584
11720220_FBXL2	0.300156	0.001242	0.004584
11757624_PARP12	0.275701	0.001242	0.004586
11756380_TRMT112	-0.18468	0.001243	0.004587
11722196_NMT1	0.200066	0.001243	0.004588
11747921_PUS1	0.276555	0.001244	0.004592
11734699_GOLGA8A /	0.180418	0.001244	0.004592
11733275_PCNXL2	0.260578	0.001246	0.004596
11729299_SDE2	0.229072	0.001246	0.004597
11753791_PRKAR1A	-0.20524	0.001246	0.004597
11723083_HEMK1	-0.20706	0.001246	0.004597
11716107_TOMM22	-0.18067	0.001247	0.004598
11716643_ERLEC1	-0.18418	0.001248	0.004605
11750292_IST1	0.199594	0.001249	0.004606
11747272_STK26	-0.26753	0.00125	0.00461
11728652_ZRANB2	0.179741	0.00125	0.004611
11717444_BYSL	0.231412	0.001251	0.004612
11720339_FIBIN	-0.24073	0.001251	0.004612
11755944_KRT39	-0.30131	0.001252	0.004615
11736436_ZNF430	0.253879	0.001252	0.004616
11754484_COPS7B	-0.21784	0.001252	0.004616
11742456_PTBP1	-0.17259	0.001253	0.004618
11719095_CYB5B	-0.20308	0.001253	0.004619

11749360_LYPLA1	0.184355	0.001253	0.004619
11723988_NR2F2	-0.51681	0.001254	0.004623
11744489_C20orf24	-0.19826	0.001256	0.004627
11757837_HNRNPA1	-0.17023	0.001256	0.004627
11757832_TRA2B	-0.1813	0.001256	0.004629
11725944_STT3A	-0.19387	0.001257	0.00463
11752820_PID1	0.421682	0.001258	0.004634
11731709_TMEM5	0.177332	0.001258	0.004635
11752459_TMEM5	0.177332	0.001258	0.004635
11749498_NFIB	-0.20424	0.001258	0.004635
11730903_JDP2	-0.2549	0.001259	0.004635
11759580_SYNCRIP	-0.68193	0.00126	0.004639
11722534_RPRM	-0.2079	0.00126	0.00464
11722031_RNF11	0.26965	0.00126	0.004641
11756996_CFLAR	0.31674	0.00126	0.004641
11720180_DDX28	-0.20389	0.001261	0.004641
11743805_MRPL42	-0.31252	0.001261	0.004642
11722546_DHRS11	-0.25563	0.001261	0.004642
11744921_SLC43A1	-0.28981	0.001262	0.004644
11717219_NFIB	-0.24075	0.001262	0.004644
11746383_ZNF131	0.256791	0.001262	0.004645
11758700_SMC3	-0.22226	0.001263	0.004647
11757102_BOP1	-0.19224	0.001263	0.004649
11719846_TFPT	0.258966	0.001263	0.004649
11722547_HIVEP1	0.587437	0.001264	0.004649
11739437_PPTC7	0.216855	0.001264	0.004651
11748930_PGAM5	-0.19949	0.001265	0.004653
11749481_TRIM35	0.344927	0.001266	0.004655
11754116_S100B	0.220581	0.001266	0.004656
11720961_NBAS	0.21277	0.001266	0.004656
11732345_ZNF33A	-0.25507	0.001267	0.004658
11744395_CALCOCO2	-0.19928	0.001267	0.004659
11762801_ZNF496	-0.29922	0.001267	0.004659
11736146_LYRM2	0.220036	0.001268	0.004664
11753857_NF2	0.412142	0.00127	0.004667
11745197_BOLA2 ///	0.201528	0.00127	0.004667
11717804_NTN4	0.351516	0.00127	0.004668
11744323_PWWP2A	0.202622	0.00127	0.004668
11743633_STX2	-0.19963	0.00127	0.004668
11722149_YTHDC2	0.433914	0.001271	0.004671
11756509_BAMBI	0.206059	0.001272	0.004674
11743039_CRTAP	-0.2913	0.001273	0.004677
11728471_PLPPR5	-0.24486	0.001273	0.004677
11718303_PAFAH1B3	-0.17515	0.001273	0.004677
11758664_RHOBTB3	0.241987	0.001273	0.004677
11720587_SMCHD1	0.23714	0.001273	0.004677
11743946_CCDC15	-0.36646	0.001274	0.004679
11744067_SYNGR2	0.19745	0.001274	0.004681
11734915_ARHGAP11A	-0.51162	0.001275	0.004683
11719411_RAB32	0.216749	0.001276	0.004685
11721716_ATRX	0.268724	0.001276	0.004685

11745968_BCLAF1	-0.38882	0.001276	0.004686
11725854_VWA8	0.213798	0.001278	0.004691
11722249_C10orf11	0.308816	0.001278	0.004691
11725886_LCAT	0.213984	0.001278	0.004693
11739489_ATP10D	0.232391	0.001279	0.004695
11755994_ITLN2	0.300337	0.00128	0.004697
11746186_LTBP4	-0.17316	0.00128	0.004699
11739065_GNG12	0.190125	0.001281	0.004702
11755683_TNFRSF8	-0.24614	0.001282	0.004703
11742118_NEIL2	-0.43847	0.001282	0.004705
11761390_ANKRD10	-0.18397	0.001282	0.004705
11762436_PCDHA2 //	-0.22735	0.001283	0.004707
11718635_KIF1B	-0.19225	0.001284	0.00471
11740041_ATP10A	-0.37637	0.001284	0.004711
11731678_FAM20C	-0.31273	0.001286	0.004716
11744159_VPS33A	0.213618	0.001286	0.004716
11754440_GAS6	0.250279	0.001286	0.004716
11748721_LONP1	-0.19446	0.001286	0.004717
11744775_BZW1	0.166585	0.001287	0.00472
11734802_TMEM159	0.38642	0.001288	0.004721
11749020_SASH1	0.254594	0.001289	0.004726
11719841_PHLDA3	0.238592	0.001289	0.004727
11717388_SEPT11	-0.1815	0.00129	0.004728
11763531_METTL15	0.448582	0.00129	0.004728
11721214_TMEM106B	-0.18649	0.00129	0.004728
11758387_ZNF613	0.310128	0.00129	0.004729
11739702_ISG20L2	0.196812	0.001291	0.004731
11727387_NPY1R	-0.38644	0.001291	0.004732
11754664_RAP2C	0.176616	0.001291	0.004732
11756098_SYTL5	0.188697	0.001292	0.004734
11753058_UGGT2	0.215955	0.001292	0.004734
11749282_MAGEA2 //	-0.18086	0.001293	0.004736
11744936_LINGO2	0.172306	0.001294	0.004738
11719897_FAM91A1	-0.35296	0.001295	0.004741
11721852_NCAPH	-0.25544	0.001295	0.004743
11739912_TSEN15	-0.1758	0.001295	0.004743
11735247_DUSP19	-0.32893	0.001296	0.004744
11716229_TMEM167B	0.230754	0.001296	0.004744
11753977_YDJC	-0.16631	0.001296	0.004747
11730187_IDH3B	-0.2076	0.001297	0.004748
11722835_KPTN	-0.17613	0.001297	0.004749
11756089_MSR1	0.364384	0.001298	0.00475
11715451_COL4A2	0.193062	0.001298	0.00475
11719877_TRPT1	-0.2389	0.001298	0.00475
11731417_NDUFS1	-0.18809	0.001298	0.004751
11760729_DTWD1	0.310804	0.001299	0.004753
11751560_ROBO1	0.218298	0.001299	0.004753
11727091_ING2	-0.18467	0.0013	0.004756
11749671_NPAS2	0.264487	0.0013	0.004757
11734707_SMIM15	0.173487	0.001301	0.004758
11733837_VTI1B	0.18443	0.001301	0.004758

11736356_MDN1	0.342326	0.001301	0.004759
11715607_PPIB	0.170891	0.001301	0.004759
11738299_HUS1B	0.343698	0.001301	0.004759
11732278_FOXP1	0.309302	0.001302	0.00476
11728125_GZMH	-0.28722	0.001302	0.004762
11724112_SIX1	-0.27241	0.001303	0.004765
11729801_EIF1AD	0.349379	0.001304	0.004769
11761577_MOP-1	-0.36617	0.001306	0.004774
11763427_ANKRD36BP	-0.27015	0.001307	0.004776
11732184_PAPOLG	0.202333	0.001307	0.004777
11756228_NECAP2	0.289059	0.001308	0.00478
11718253_PPP1R10	0.212886	0.001308	0.004781
11732281_FOXP1	0.364854	0.001309	0.004783
11746855_APOA1BP	-0.19068	0.001309	0.004783
11754852_USP37	0.246484	0.001311	0.00479
11718382_PARP4	0.189026	0.001311	0.00479
11739100_CSTF1	0.202953	0.001311	0.004791
11757819_HELZ	0.206912	0.001312	0.004793
11725286_ANKS6	-0.23933	0.001312	0.004793
11733405_CLDN19	-0.27466	0.001313	0.004795
11722837_FUBP1	0.197033	0.001314	0.0048
11721830_CHURC1	0.202839	0.001314	0.0048
11724475_MTUS1	0.549471	0.001315	0.0048
11758237_AGO4	0.480463	0.001315	0.0048
11751589_NMT1	0.172892	0.001315	0.004801
11753459_HNRNPA1	-0.21371	0.001315	0.004802
11756533_YTHDF3	-0.20658	0.001317	0.004806
11719102_SENP6	-0.28487	0.001317	0.004806
11756153_SERF2	0.165234	0.001317	0.004806
11739199_TMEM106C	-0.19783	0.001317	0.004807
11726269_ITFG1	0.204935	0.001318	0.004811
11731228_DLK2	-0.21202	0.001319	0.004812
11731093_BRD1	0.184993	0.001319	0.004814
11754857_MRPL22	-0.17385	0.00132	0.004814
11728811_OTUD7B	0.21794	0.00132	0.004817
200000_PMPRPF8	-0.21236	0.001321	0.004817
11732232_ORAI2	0.170571	0.001321	0.004819
11718527_MSC	-0.35231	0.001321	0.00482
11720636_ISCA2	-0.21045	0.001322	0.004821
11725411_NAV1	0.245802	0.001323	0.004823
11722084_INTS5	-0.25657	0.001323	0.004826
11719093_CYB5B	-0.24616	0.001324	0.004829
11743554_KIN	0.205514	0.001325	0.004831
11743363_EIF5B	0.191269	0.001325	0.004831
11746511_SEPHS1	-0.1732	0.001325	0.004832
11759034_STT3B	-0.1817	0.001325	0.004832
200052_PMILF2	-0.17794	0.001326	0.004834
11731478_CHMP1B	-0.22789	0.001327	0.004836
11737829_ZKSCAN8	0.58447	0.001327	0.004837
11736662_CADPS2	0.437967	0.001328	0.004839
11753941_SFR1	-0.27573	0.001329	0.004841

11721350_ANAPC11	-0.18933	0.001329	0.004843
11754732_CNDP1	0.238752	0.001329	0.004843
11731108_STAG2	-0.16802	0.001331	0.004851
11722171_TOLLIP	-0.23711	0.001332	0.004851
11759707_ASPH	0.557033	0.001332	0.004852
11760039_LRP1	-0.23064	0.001332	0.004853
11718665_SRSF9	-0.17708	0.001332	0.004853
11758127_CHCHD2	-0.17421	0.001333	0.004855
11716752_GNS	0.237053	0.001333	0.004856
11742770_AGAP1	0.185344	0.001334	0.004856
11729599_GABRG2	-0.34338	0.001335	0.00486
11755037_KIAA0319L	0.236717	0.001335	0.004861
11754038_CNBP	0.19542	0.001336	0.004864
11717234_MKLN1	-0.27834	0.001337	0.004867
11729328_ZFHX4	-0.16414	0.001338	0.004869
11752371_ABCA10	0.183414	0.001338	0.004869
11728841_PLEKHA5	0.189791	0.001338	0.00487
11756262_NUBP1	-0.20987	0.001338	0.00487
11730411_PXMP4	-0.36612	0.001339	0.004873
11720723_ZNF408	0.226002	0.001339	0.004873
11739198_DDX52	0.263769	0.001341	0.004878
11731004_MIS18BP1	-0.33575	0.001342	0.004882
11741374_ATM	0.323127	0.001342	0.004883
11724924_PPP1R9A	0.314702	0.001342	0.004883
11718827_ITM2C	-0.24102	0.001343	0.004886
11722693_ZMYM4	-0.22474	0.001343	0.004886
11717086_RBM39	0.225143	0.001343	0.004886
11721485_TP53RK	-0.18717	0.001344	0.004886
11728580_ADRA2C	-0.28388	0.001344	0.004886
11722358_EPB41L2	0.266609	0.001344	0.004886
11717373_DNAJC3	0.170742	0.001344	0.004887
11725831_OTUB2	-0.23696	0.001345	0.00489
11737848_RSPH3	0.312699	0.001346	0.004892
11738365_PBLD	0.213773	0.001347	0.004896
11744902_FADS2	-0.21745	0.001347	0.004897
11718469_USP33	0.190536	0.001348	0.004899
11721916_MDM4	-0.29872	0.00135	0.004904
11726332_LEF1	-0.30654	0.00135	0.004905
11745963_PPP4R1L	0.20832	0.001351	0.004909
11734570_DCTN4	0.211956	0.001351	0.00491
11759222_KIAA1958	-0.34519	0.001352	0.00491
11748441_SLC9B2	0.328043	0.001352	0.004911
11751050_NUCB1	-0.1767	0.001353	0.004916
11723283_CEACAM1	0.198638	0.001354	0.004919
11737947_CSNK2B //	0.332513	0.001355	0.00492
11749810_ISYNA1	0.176189	0.001355	0.004921
11724023_TRIM13	-0.31591	0.001356	0.004923
11719114_PJA2	0.181621	0.001356	0.004924
11744872_ARF5	-0.19181	0.001358	0.00493
11756127_PGM1	-0.20177	0.001358	0.00493
11748453_SNRPA	-0.25568	0.00136	0.004936

11757220_RPS2P32	-0.17944	0.00136	0.004937
11720767_METTL9	-0.22958	0.00136	0.004937
11754208_NTAN1	-0.27666	0.00136	0.004937
11740731_FUOM	-0.27655	0.001361	0.004938
11717799_ZER1	-0.22957	0.001361	0.004938
11724436_FLJ10038	-0.28468	0.001361	0.004938
11748060_ADAMTSL4	-0.28267	0.001361	0.004938
11754629_SLC16A6	0.681248	0.001364	0.004947
11725637_EIF3J	0.16735	0.001364	0.004948
11759334_AMACR ///	-0.21568	0.001364	0.004949
11733735_AAMP	-0.35668	0.001364	0.004949
11724756_FAM53C	0.173898	0.001365	0.004949
11731434_MRPL34	0.16696	0.001365	0.004951
11732757_ESPNL	-0.32233	0.001365	0.004951
11730328_DCAF13	-0.29472	0.001366	0.004953
11728756_NHLRC3	0.446823	0.001366	0.004953
200033_PMDDX5	0.171366	0.001367	0.004955
11745765_TCF4	0.305496	0.001368	0.00496
11738270_SERP2	-0.18177	0.00137	0.004965
11719756_COA6	-0.17359	0.001371	0.004969
11744532_PNN	0.193909	0.001372	0.004974
11726888_PORCN	-0.26265	0.001373	0.004974
11759229_NBPF4	0.518696	0.001373	0.004975
11730445_CCNJ	0.224067	0.001373	0.004976
11717674_EIF2B2	0.198365	0.001373	0.004976
11720439_ACVR1B	0.24535	0.001373	0.004976
11717734_ZCCHC24	-0.21266	0.001375	0.00498
11718910_OTUD5	-0.20845	0.001375	0.004981
11717239_RBBP4	-0.18888	0.001375	0.004982
11741649_SLC12A4	0.292972	0.001376	0.004986
11761874_SLMAP	0.335787	0.001377	0.004988
11758922_STXBP6	0.69371	0.001377	0.004988
11743009_ARHGEF1 /	-0.20813	0.001378	0.004989
11715349_COX7C	-0.17228	0.001378	0.004989
11746187_MRPS18B	-0.26021	0.001379	0.004991
11760764_ADAMTS16	-0.45447	0.001379	0.004991
11727508_CLDN12 //	0.210689	0.00138	0.004996
11734781_SORBS3	-0.38822	0.00138	0.004997
11751573_LBR	0.178588	0.001381	0.004998
11745224_RIF1	0.223158	0.001382	0.005001
11745666_ASXL1	-0.26005	0.001382	0.005001
11758769_MRPL20	0.494147	0.001383	0.005005
11717443_JAGN1	0.170567	0.001385	0.005011
11721823_ARPC5L	0.193234	0.001385	0.005011
11740332_ATP2A2	0.179673	0.001386	0.005015
11757429_CYC1	-0.21342	0.001388	0.005023
11756225_DNAJB6	0.188602	0.001388	0.005023
11755205_HSPH1	0.209929	0.001389	0.005024
11735738_SH2B1	-0.18065	0.00139	0.005027
11753679_SNCG	-0.21726	0.001391	0.00503
11755440_SPDYE1 //	0.271649	0.001391	0.005032

11740245_PTHLH	0.770209	0.001392	0.005034
11736397_RFT1	-0.29034	0.001392	0.005035
11737276_HTR7	0.266088	0.001394	0.005038
11762823_DHRS4 ///	-0.27145	0.001394	0.005038
11738890_BPIFB6	-0.18268	0.001395	0.005044
11716072_ATP6V1B2	0.201917	0.001395	0.005044
11746151_BMS1P4	0.229925	0.001396	0.005048
11737815_METTL21B	-0.20355	0.001397	0.005051
11756544_C22orf29	-0.23011	0.001399	0.005057
11721456_SERPINA1	0.425593	0.001401	0.005063
11747881_BCL2L13	0.191802	0.001402	0.005065
11754342_ELAC2	-0.19798	0.001402	0.005065
11724227_SUPT20H	0.245274	0.001403	0.005068
11751902_CDON	-0.22067	0.001403	0.005069
11754455_CABLES1	0.192143	0.001406	0.00508
11764012_TMPO	-0.4315	0.001408	0.005084
11720964_DCK	-0.24557	0.001409	0.00509
11756322_CNDP2	-0.19085	0.001409	0.00509
11719923_GM2A	-0.33846	0.00141	0.005091
11721051_C5orf51	0.180206	0.00141	0.005092
11748381_GALC	0.314519	0.001411	0.005095
11753842_UFM1	0.198294	0.001411	0.005096
11740707_PPP6C	0.167742	0.001413	0.005102
11748251_USP34	0.194945	0.001413	0.005102
11716295_FLOT2	-0.19416	0.001413	0.005102
11751586_SLC36A2	0.189422	0.001414	0.005102
11732590_TRIM5	0.347897	0.001414	0.005102
11717530_UBA2	-0.28487	0.001414	0.005103
11746128_CLPTM1	0.17002	0.001415	0.005106
11746672_ZNF8	0.243695	0.001415	0.005107
11745077_CRNKL1	0.252218	0.001417	0.005114
11758366_ABT1	0.294283	0.001418	0.005115
11730206_PSMD9	-0.21826	0.001418	0.005115
11717079_VPS52	-0.1677	0.001418	0.005115
11756066_LRRC23	-0.25581	0.001419	0.005117
11724355_LOC102724	0.174696	0.00142	0.005122
11720720_C22orf29	-0.30907	0.00142	0.005122
11723926_PNMA2	-0.43033	0.00142	0.005122
11725665_HS2ST1	-0.17286	0.001423	0.005131
11760362_PGAM5	-0.23521	0.001424	0.005133
11736302_FN3K	-0.2899	0.001424	0.005133
11738443_OR6B1	-0.23048	0.001424	0.005134
11740346_NRG1	0.219333	0.001424	0.005135
11748243_DVL2	-0.17017	0.001426	0.005139
11750977_SCMH1	-0.27241	0.001426	0.005139
11746509_TDG	0.291606	0.001426	0.00514
11748410_FLII	0.190714	0.001426	0.005141
11749683_COL4A3BP	-0.19565	0.001428	0.005146
11732142_TSR1	0.182004	0.001428	0.005146
11745198_CDCA4	-0.17068	0.001429	0.005149
11717849_CCDC167	-0.25414	0.001429	0.005149

11732132_IDS	0.241785	0.00143	0.005152
11743303_ICE1	0.195052	0.00143	0.005152
11754388_QSOX2	-0.2111	0.00143	0.005152
11718853_ZBED5	0.201569	0.001431	0.005153
11758665_TEAD2	-0.24824	0.001431	0.005153
11755206_HSPH1	0.187598	0.001431	0.005154
11740182_RHOJ	0.303279	0.001431	0.005154
AFFX-HSACACTB	0.16992	0.001434	0.005165
11742018_CACNA1D	-0.22009	0.001434	0.005165
11745487_ZNF512	-0.21992	0.001435	0.005166
11732135_ADCK3	-0.23109	0.001436	0.005168
11758323_VOPP1	-0.33616	0.001437	0.005173
11723039_SRSF3	-0.21085	0.001437	0.005173
11760017_SNRPA1	-0.25768	0.001437	0.005173
11720272_RIMS4	-0.29899	0.001437	0.005173
11755573_FAXDC2	-0.20477	0.001438	0.005174
11727761_PALM3	-0.48613	0.001439	0.005177
11718385_C2orf68	0.232403	0.001439	0.005178
11719812_SLC48A1	0.254043	0.001439	0.005178
11758656_UBQLN2	-0.19184	0.001439	0.005178
11722563_CYP51A1 /	-0.33783	0.00144	0.005179
11752317_NVL	0.211515	0.00144	0.005179
11734445_AARD	-0.27323	0.00144	0.005179
11744100_GMPPB	-0.2795	0.001441	0.005181
11721724_GLB1L2	-0.19256	0.001443	0.005189
11720753_SDC2	0.269775	0.001443	0.005189
11742755_VRK2	0.218544	0.001444	0.005191
11723880_KCNC4	-0.33045	0.001444	0.005194
11755495_LRRC27	0.447862	0.001446	0.005197
11750723_MICB	-0.23347	0.001446	0.005199
11731202_AIM1L	0.245879	0.001447	0.005201
11719804_ASPN	0.394802	0.001447	0.005201
11740127_LATS1	0.537684	0.001448	0.005203
11721060_PLPP5	0.238517	0.001449	0.005206
11730725_GABBR1	0.270003	0.001449	0.005207
11759569_CDK5RAP3	-0.32594	0.00145	0.005209
11756787_HAPLN3	-0.32264	0.00145	0.005209
11747315_ENTPD6	0.161008	0.00145	0.005211
11733165_YIPF5	-0.23918	0.001451	0.005212
11764051_CHST1	-0.26511	0.001451	0.005213
11729668_ZCCHC8	0.197888	0.001452	0.005216
11739398_UBE2F	0.192727	0.001452	0.005216
11746524_FAM81A	-0.17961	0.001453	0.005219
11718877_SMC1A	-0.20132	0.001453	0.005219
11744949_NELFE	-0.19727	0.001453	0.005219
11748389_GON4L ///	0.190822	0.001457	0.005233
11759387_OR10H5	-0.30641	0.001459	0.005239
11763292_ENDOV	0.201258	0.001459	0.005239
11759760_HIST1H2BC	0.549824	0.00146	0.005241
11753468_AMT	0.230123	0.00146	0.005242
11724786_GSKIP	0.187503	0.00146	0.005242

11739624_ZNF740	-0.20692	0.001461	0.005243
11728668_TRIM16 //	0.186013	0.001461	0.005244
11750689_ADAM11	-0.21314	0.001461	0.005244
11757540_YIPF3	0.198381	0.001463	0.005248
11757789_EPB41L2	-0.17588	0.001463	0.00525
11734919_TCEA1	-0.18597	0.001464	0.005252
11728246_FAM134A	-0.37707	0.001464	0.005253
11746610_CAND2	-0.39934	0.001464	0.005253
11743753_ZFAND5	-0.25776	0.001465	0.005254
11755844_DNAJC16	0.258606	0.001467	0.00526
11718352_VPS33B	0.167347	0.001468	0.005265
11757265_EIF3K	-0.17255	0.001469	0.005268
11733899_TROVE2	0.189068	0.00147	0.005271
11718634_KIF1B	-0.1697	0.001471	0.005274
11729431_PALD1	-0.38779	0.001473	0.005282
11717914_BIVM-ERCC	0.246151	0.001474	0.005284
11728755_NHLRC3	0.253104	0.001474	0.005284
11729377_TMEM205	-0.1815	0.001475	0.005288
11715664_STARD7	-0.18982	0.001476	0.005292
11758099_CHCHD2	-0.17702	0.001477	0.005293
11735692_RAP1GDS1	0.197545	0.001477	0.005294
11749757_SDHA	-0.20379	0.001477	0.005294
11744624_TRIQK	-0.29676	0.001479	0.005299
11746408_SORT1	0.239943	0.001479	0.005299
11730139_RIOK1	0.185322	0.00148	0.005302
11755292_CYB561A3	-0.17823	0.001481	0.005305
11717255_HSPA4	-0.20471	0.001481	0.005305
11751134_MAT2A	0.196415	0.001483	0.005312
11736675_SNRPB	-0.17639	0.001485	0.005317
11749034_RASA2	0.345803	0.001485	0.005319
11729601_PPP6C	0.161333	0.001485	0.00532
11744053_SLC11A2	0.239347	0.001486	0.00532
11760254_IFI44	0.355877	0.001486	0.00532
11718009_STX18	0.17098	0.001486	0.00532
11745744_AMPD2	-0.20221	0.001487	0.005323
11717193_OST4	0.166211	0.001487	0.005323
11735502_TAPBP	0.323459	0.001488	0.005325
11718446_SH3BGRL2	-0.42873	0.001488	0.005325
11720328_ATP5O	-0.21977	0.001488	0.005326
11734222_ZNF543	0.374185	0.001488	0.005327
11720303_RTN3	-0.16624	0.001489	0.00533
11755069_KDM5A	0.227757	0.001491	0.005334
11747264_BCAS1	-0.29941	0.001491	0.005335
11717332_BMS1	-0.29782	0.001492	0.005337
11737824_STX16	-0.17693	0.001492	0.005337
11727563_PCDHB5	-0.21181	0.001492	0.005338
11757871_PBX1	-0.2401	0.001492	0.005339
11717988_C17orf49	-0.22467	0.001493	0.005341
11739088_MESDC2	0.201662	0.001495	0.005346
11715918_CD24	0.349948	0.001495	0.005347
11726417_ABI1	-0.19822	0.001496	0.005348

11744482_TMEM246	-0.21847	0.001496	0.00535
11716605_ADAM12	-0.20303	0.001497	0.005351
11743281_WNK1	-0.22209	0.001497	0.005352
11734393_ERBB3	-0.2391	0.001498	0.005354
11724390_ZDHHC16	0.196102	0.001498	0.005355
11720710_KIF1B	0.266099	0.001498	0.005356
11736440_INF2	-0.21135	0.0015	0.00536
11717667_POLA2	-0.2471	0.0015	0.005362
11733430_C10orf90	0.176902	0.001501	0.005363
11756961_HYOU1	0.172087	0.001502	0.005367
11738631_PCSK6	-0.19094	0.001503	0.005369
11742922_LAPTM5	0.2273	0.001503	0.005369
11746840_PPP1R9A	0.252122	0.001503	0.005371
11716859_ETFA	0.187822	0.001504	0.005372
11761868_TPTE2	0.244095	0.001504	0.005374
11735633_RPS6KA5	0.417363	0.001505	0.005374
11721783_SLC9A8	-0.19426	0.001505	0.005377
11722354_SEC23A	0.173603	0.001506	0.005377
11718690_ACOT8	0.173046	0.001506	0.005378
11731132_JRK	-0.22857	0.001506	0.005379
11719716_CD79A	-0.36998	0.001507	0.005381
11728084_DGKE	0.331815	0.001509	0.005387
11735720_ARMCX5	0.184424	0.001509	0.005387
11720298_CXCL10	0.454888	0.00151	0.00539
11721017_FBRSL1	-0.29789	0.00151	0.005391
11726279_RAN	-0.17768	0.001511	0.005394
11747463_PHKG2	0.182112	0.001512	0.005395
11716646_HM13	0.205303	0.001512	0.005395
11756698_AKT2	-0.24278	0.001512	0.005395
11744678_C16orf62	-0.17612	0.001512	0.005395
11760746_STK32B	-0.20852	0.001512	0.005395
11726488_DHX57	0.244127	0.001512	0.005395
11747133_ZFHX3	-0.23701	0.001512	0.005395
11750124_NCAM2	-0.3309	0.001513	0.005396
11735121_TUBGCP4	-0.17802	0.001515	0.005403
11719038_ARAP1	-0.21287	0.001515	0.005403
11746331_ADK	0.185203	0.001515	0.005404
11729592_LLGL1	-0.25939	0.001516	0.005407
11751288_LOXL2	0.182793	0.001516	0.005407
11723739_WDR36	-0.1731	0.001517	0.005408
11753652_FPGS	-0.23536	0.001518	0.005411
11746962_HLA-DMB	-0.26265	0.001518	0.005413
11760561_RBM26	0.179944	0.001519	0.005415
11753333_RTN3	-0.20388	0.001519	0.005416
11739213_ARL14EP	0.184648	0.001519	0.005416
11745761_AHSA1	-0.18312	0.00152	0.005416
11744565_PPWD1	-0.21335	0.00152	0.005417
11727588_ZNF48	-0.16426	0.001521	0.005421
11719195_POLR1C	0.21876	0.001522	0.005423
11717307_IARS2	-0.18204	0.001522	0.005423
11725031_PNN	0.185971	0.001525	0.005432

11754595_SLC26A2	0.168941	0.001526	0.005435
11732112_IL17A	-0.25176	0.001527	0.00544
11737365_PCDHA1 //	0.327945	0.001527	0.00544
11730252_AHCTF1	0.171052	0.001527	0.00544
11757833_RAB31	0.186389	0.001528	0.005443
11748125_PPP6C	0.183137	0.001529	0.005446
11715550_DAG1	-0.1675	0.001529	0.005446
11745245_ITGBL1	0.366424	0.00153	0.005447
11761047_MKKS	-0.24117	0.00153	0.005448
11758794_ORM1	-0.21496	0.001531	0.005449
11750092_TPRKB	-0.20361	0.001531	0.005449
11755174_CGN	0.256567	0.001531	0.005451
11718296_SEMA4B	-0.19681	0.001532	0.005452
11722212_PQLC1	-0.22239	0.001533	0.005457
11754010_GOLGA2	0.201089	0.001533	0.005457
11727350_RARA	0.419266	0.001534	0.005457
11752518_ATG2B	0.545796	0.001535	0.005461
11737983_CYHR1	-0.19596	0.001535	0.005461
11749211_LOC100126	-0.23888	0.001536	0.005464
11722560_ILF3	-0.24188	0.001536	0.005465
11733001_PMM2	0.204143	0.001537	0.005466
11719919_TIMM10B	0.221957	0.001537	0.005469
11744362_PSMD14	-0.17187	0.001539	0.005474
11755435_POLI	0.259017	0.00154	0.005477
11719329_ALAS1	-0.19078	0.00154	0.005477
11737422_FAM163B	-0.1945	0.001541	0.00548
11715831_SURF4	-0.19324	0.001541	0.005481
11754317_ANXA4	0.18067	0.001543	0.005486
11755523_RBM47	-0.31236	0.001544	0.00549
11749647_ZKSCAN2	0.348997	0.001544	0.00549
11745263_SNX17	0.16403	0.001545	0.005493
11753987_DOCK7	0.225578	0.001546	0.005494
11720401_NCS1	-0.2824	0.001547	0.005499
11725186_KLF12	-0.23724	0.001547	0.005499
11722691_AVEN	0.261529	0.001549	0.005504
11763480_DLEU2	-0.17555	0.001551	0.00551
11753118_POLR3D	-0.16611	0.001551	0.005511
11725240_RTP4	0.345375	0.001552	0.005513
11736869_MMP16	-0.17857	0.001552	0.005514
11730871_RASSF5	-0.25771	0.001552	0.005514
11752434_EXT2	0.182326	0.001552	0.005514
11722920_DOLPP1	-0.19646	0.001553	0.005515
11760760_UCHL5	0.294989	0.001553	0.005516
11758086_WHAMMP3	0.212664	0.001553	0.005517
11761065_C11orf73	-0.33224	0.001554	0.005519
11760141_RBM3	0.507281	0.001555	0.005522
11739200_TMEM106C	-0.16713	0.001558	0.005532
11726805_PTPMT1	-0.26139	0.001558	0.005533
11730422_FLVCR1	0.30078	0.001559	0.005533
11748140_AMD1	0.174733	0.001559	0.005534
11747727_LMAN2L	0.199641	0.00156	0.005535

11758896_CCNI	-0.17508	0.00156	0.005536
11732503_ICA1L	-0.27215	0.001561	0.00554
11722569_VEZT	0.275153	0.001561	0.00554
11753934_AGAP2-AS1	-0.38138	0.001562	0.005541
11728485_LIMD1	-0.26056	0.001563	0.005545
11730734_PPP1R8	-0.35492	0.001563	0.005545
11750172_CLTCL1	0.384225	0.001563	0.005545
11743919_MAP3K7	0.222223	0.001564	0.00555
11753718_LRTOMT	0.29687	0.001565	0.00555
11719287_PEX11B	-0.30351	0.001565	0.00555
11727785_CACYBP	-0.33825	0.001565	0.005552
11746190_PLIN4	-0.19228	0.001566	0.005552
11720216_PIGU	-0.17005	0.001567	0.005557
11753548_SIGMAR1	-0.21437	0.001568	0.00556
11725540_GATA4	-0.31477	0.001569	0.005563
11751820_SLC9B2	0.166747	0.00157	0.005566
11749188_ENTPD1	0.174195	0.001571	0.005568
11743059_COMMD10	0.216487	0.001571	0.005569
11717694_CEBPG	0.236309	0.001572	0.00557
11736601_BRIP1	-0.47041	0.001573	0.005573
11715222_MAGI3	-0.32058	0.001573	0.005573
11746148_SLC25A29	-0.2513	0.001574	0.005578
11760170_SNRPB	-0.20355	0.001577	0.005588
11761460_CBWD1 ///	0.269134	0.001577	0.005588
11717368_ZC3HAV1	0.323801	0.001577	0.005589
11754144_MET	0.332357	0.001578	0.00559
11750599_OSBPL6	-0.19903	0.001578	0.00559
11753841_COMMD6	0.203323	0.001578	0.00559
11755657_DMXL1	0.311016	0.001579	0.005592
11720585_SMCHD1	0.199427	0.001579	0.005592
11733049_NSMCE1	-0.19279	0.001581	0.005598
11736245_PRKAG2	0.194792	0.001581	0.005599
11736594_ZBTB24	0.330677	0.001582	0.005601
11739375_SYT7	-0.21386	0.001583	0.005603
11733883_TTF1	-0.19212	0.001583	0.005604
11739104_TMEM33	0.173494	0.001585	0.005611
11723616_PLTP	-0.16817	0.001586	0.005613
11746281_LPCAT3	-0.23003	0.001586	0.005614
11748098_ZNF331	-0.29149	0.001586	0.005614
11748919_CCSAP	-0.26681	0.001587	0.005616
11750621_TMOD2	0.507399	0.00159	0.005627
11734157_LENG1	-0.18337	0.001591	0.005627
11742448_LRRIQ3	0.334432	0.001591	0.005627
11736037_TADA3	-0.25052	0.001591	0.005627
11733054_SCAMP1	-0.20642	0.001591	0.005627
11723960_SCFD2	0.225543	0.001591	0.005627
11746268_ZNF630	0.315249	0.001591	0.005628
11758440_RFX7	0.210737	0.001593	0.005634
11721463_OSBPL2	-0.20939	0.001594	0.005638
11748162_EML5	0.297679	0.001595	0.00564
11736575_GPR155	-0.28239	0.001595	0.005641

11763747_CLPX	0.198985	0.001597	0.005646
11731486_NFIC	-0.20951	0.001597	0.005646
11719313_FAM111A	-0.21903	0.001598	0.005649
11733915_DSC3	0.475545	0.001598	0.005649
11733182_ETV1	0.235617	0.001598	0.00565
11724840_PKN2	0.257884	0.001598	0.00565
11751179_TMPRSS5	-0.25049	0.001599	0.00565
11721584_TMCC1	0.277312	0.001599	0.005652
11720715_NIPA1	-0.20013	0.001602	0.005661
11726280_BCR	-0.21982	0.001603	0.005663
11740581_ASAH1	0.201035	0.001603	0.005665
11754407_ZMIZ2	0.356304	0.001603	0.005665
11745338_RAB3IL1	-0.20919	0.001604	0.005668
11725694_SDPR	0.281417	0.001606	0.005672
11718696_YIF1B	-0.25552	0.001606	0.005672
11738715_OR52D1	-0.34373	0.001606	0.005673
11763325_FER	-0.17285	0.001608	0.005679
11758809_RRAGC	0.208569	0.001608	0.005681
11744078_SDHA ///	-0.25727	0.001609	0.005683
11735450_TMEM255B	0.208309	0.00161	0.005684
11718038_DCAF12	0.17196	0.00161	0.005686
11758738_SREBF1	-0.17717	0.001611	0.005688
11721263_GRIA2	0.415974	0.001611	0.005688
11723787_ZNF766	0.298417	0.001611	0.005688
11758467_CCZ1 ///	-0.17438	0.001611	0.005688
11748160_LAMP2	0.221527	0.001611	0.005688
11716497_CHMP4B	-0.17659	0.001612	0.005691
11749366_ATP9B	0.175043	0.001613	0.005692
11745689_CMPK2	-0.20452	0.001613	0.005692
11722103_PAN3	0.289014	0.001613	0.005693
11723792_OSBPL11	0.216654	0.001614	0.005695
11747038_METTL20	-0.43479	0.001614	0.005695
11748299_SHC4	0.356165	0.001616	0.0057
11748967_RDX	0.164126	0.001617	0.005705
11730614_SLC1A4	-0.27794	0.001617	0.005706
11734736_FAR1	0.225203	0.001618	0.005706
11731249_SLC35A2	-0.38966	0.001618	0.005707
11719894_FAM91A1 /	0.184338	0.001618	0.005707
11736607_ZNF528	0.329704	0.001619	0.005709
11729306_ATAD1	0.216726	0.00162	0.005712
11759671_KCNMA1	0.19352	0.00162	0.005714
11737618_RFPL4B	-0.21154	0.001621	0.005714
11739233_SAFB2	0.208037	0.001621	0.005714
11733073_DDX21	0.189468	0.001622	0.005717
11720960_NBAS	0.256398	0.001622	0.005718
11743181_C16orf59	-0.33637	0.001622	0.005718
11729940_TMEM56	0.365532	0.001626	0.005731
11738630_RBL1	-0.19449	0.001627	0.005732
11757041_CNP	-0.20768	0.001629	0.005742
11719611_ADO	0.238218	0.001631	0.005748
11753471_HSPG2	-0.27842	0.001632	0.005749

11754224_LOC102724	-0.54717	0.001633	0.005752
11746217_HPS3	-0.35912	0.001633	0.005753
11749113_DLG1	0.231257	0.001634	0.005754
11734164_ARHGEF26	0.323537	0.001635	0.005759
11746140_ARHGEF26	0.323537	0.001635	0.005759
11744682_EIF5A	-0.16358	0.001635	0.005759
11719400_DOCK6	0.190672	0.001636	0.00576
11759182_FAM168A	-0.16906	0.001637	0.005764
11716319_IGBP1	0.222878	0.001638	0.005766
11761436_CIAPIN1	-0.24011	0.001638	0.005766
11745842_CNDP2	-0.17952	0.001638	0.005766
11722192_UBE2M	-0.20244	0.001641	0.005775
11739087_MESDC2	0.193925	0.001645	0.005789
11716992_CIC	-0.18803	0.001645	0.005789
11725480_XRCC1	-0.20216	0.001646	0.005792
11745865_SSFA2	0.162582	0.001646	0.005792
11750566_LPIN1	0.171929	0.001646	0.005793
11721985_SLC37A3	0.401758	0.001648	0.005798
11716927_TBC1D5	0.166349	0.001648	0.005798
11719335_DCP1B	0.162674	0.001648	0.005799
11758296_CDC34	0.173392	0.001648	0.005799
11727461_ZXDC	-0.22994	0.001649	0.005801
11751665_PARPBP	-0.24609	0.00165	0.005805
11733002_PMM2	0.251545	0.001651	0.005808
11756248_PDPK1	0.196068	0.001652	0.005811
11749871_GBGT1	-0.23436	0.001653	0.005814
11756261_C16orf59	-0.22255	0.001654	0.005815
11716374_PTK2	0.195322	0.001654	0.005816
11720673_PPM1A	0.186303	0.001654	0.005816
11748370_MAGED2	0.161538	0.001655	0.005816
11720838_RAD17	-0.19705	0.001655	0.005816
11723449_NDUFV3	0.215767	0.001655	0.005816
11753958_GARS	0.178145	0.001655	0.005816
11722338_PEX7	0.289134	0.001655	0.005817
11719042_ACSL4	-0.21011	0.001656	0.00582
11748928_PAFAH2	0.381467	0.001657	0.005822
11753448_PHAX	0.178581	0.001659	0.00583
11741434_SEPT10	-0.19637	0.001659	0.00583
11719203_ERI3	-0.16847	0.001659	0.005831
11727064_ANKRD11	0.181517	0.00166	0.005833
11742880_UBE2B	0.175908	0.001661	0.005833
11719721_LSM7	-0.20289	0.001661	0.005836
11718808_TP53BP1	0.171442	0.001663	0.005841
11717702_RB1	-0.33408	0.001663	0.005842
11758595_NBEA	0.280516	0.001664	0.005844
11745685_SCLT1	-0.40939	0.001664	0.005845
11754584_RHNO1	-0.25353	0.001665	0.005845
11719921_GM2A	-0.29858	0.001665	0.005847
11746081_FAM60A	0.242951	0.001667	0.005852
11761634_SLC37A3	0.536496	0.001667	0.005853
11744252_RFX3	0.457359	0.00167	0.005863

11732465_USP44	0.378878	0.001671	0.005864
11740193_UBE2A	-0.19857	0.001671	0.005865
11734894_CTC1	-0.17631	0.001672	0.005868
11743145_GTF2H4 //	-0.20079	0.001672	0.005869
11740514_YTHDC1	0.297873	0.001672	0.005869
11726195_METTL21B	-0.17426	0.001673	0.00587
11727144_KLF11	-0.17657	0.001675	0.005876
11754115_S100B	0.20077	0.001675	0.005876
11715507_MAGED1	-0.19758	0.001675	0.005876
11725809_ARC	0.256935	0.001675	0.005876
11733634_HOXD13	-0.19096	0.001675	0.005877
11759980_NF1	0.185454	0.001677	0.005881
11723382_PRR3	-0.25875	0.001677	0.005881
11733175_ZNF507	0.267986	0.001678	0.005885
11742888_GCSH ///	-0.16668	0.00168	0.00589
11762228_DEPDC7	0.478462	0.00168	0.005892
11722387_MIDN	0.245537	0.00168	0.005892
11720743_CROT	0.254419	0.001682	0.005896
11745164_HADHB	-0.16933	0.001683	0.0059
11722034_SLC12A4	0.354659	0.001683	0.0059
11749998_SLC47A1	-0.30794	0.001683	0.005901
11726511_TCHP	-0.21189	0.001684	0.005902
11745603_CLTA	0.180787	0.001685	0.005904
11754795_PIGV	-0.24772	0.001685	0.005905
11731069_NIT1	0.173156	0.001686	0.005907
11747772_PDHA1	-0.23418	0.001687	0.005909
11718630_BOK	-0.43384	0.001687	0.005911
11746451_COG5	0.232743	0.001687	0.005911
11743773_LZTFL1	-0.35117	0.001689	0.005916
11743002_DCTN6	0.196956	0.001689	0.005917
11727533_FEZ2	0.186461	0.00169	0.005918
11746772_REG4	-0.24932	0.00169	0.005918
11716462_RFXANK	-0.23581	0.00169	0.005918
11728835_GLMN	0.23054	0.00169	0.005919
11730292_ARHGAP5	-0.28577	0.001691	0.005923
11724093_SLC35E2 /	-0.20868	0.001692	0.005926
11733021_LDHA	-0.16766	0.001693	0.005928
11763165_PABPN1	0.225586	0.001694	0.005929
11760137_IGHA1	-0.1898	0.001695	0.005934
11759511_MOSPD2	-0.25304	0.001697	0.00594
11754016_TIMM50	-0.20024	0.001697	0.00594
11734723_EDIL3	0.428647	0.001697	0.00594
11725738_ 3-Mar	-0.18028	0.001697	0.00594
11763406_BICC1	-0.22247	0.001697	0.00594
11759373_CSRP1	-0.19233	0.001698	0.005942
11758558_ATP2A2	0.159044	0.001699	0.005944
11733008_AMOTL2	0.213703	0.0017	0.005948
11733336_HDX	0.601391	0.001701	0.005951
11726281_BCR	-0.23159	0.001701	0.005951
11742240_ZMAT3	0.173896	0.001702	0.005952
11726115_MTAP	-0.22246	0.001702	0.005953

11728400_MDFIC	-0.2612	0.001702	0.005953
11733097_ALDOA	-0.17993	0.001706	0.005967
11728858_CEP19	0.354837	0.001706	0.005967
11742756_GTF2H1	0.201901	0.001707	0.005969
11723891_CAND2	-0.44714	0.001707	0.005969
11763355_EP400NL	0.244753	0.001709	0.005977
11733036_CEBPZ	0.209406	0.00171	0.005978
11757550_C16orf58	-0.22335	0.00171	0.005979
11716601_KBTBD2	0.218482	0.001711	0.005981
11746160_FLT1	0.561231	0.001711	0.005981
11737565_DHPS	-0.23747	0.001711	0.005981
11729682_TMEM231	-0.21442	0.001711	0.005981
11736964_C8orf37	-0.18629	0.001712	0.005983
11754965_TRIM52	0.316372	0.001714	0.00599
11724476_MTUS1	0.34338	0.001715	0.005992
11736110_ARL8A	0.185391	0.001717	0.006
11757219_LINC00473	-0.32956	0.001718	0.006002
11721751_FZD7	-0.20927	0.001719	0.006005
11745723_MALAT1	-0.18329	0.001719	0.006005
11717778_USP15	0.32432	0.001719	0.006005
11721423_AMZ2	0.212693	0.00172	0.006008
11720940_DDX54	-0.19954	0.001721	0.006011
11718647_ACVR1	-0.27347	0.001725	0.006022
11753373_EIF3L	0.170235	0.001725	0.006022
11754898_ZNF573	0.238599	0.001725	0.006023
11720360_PHIP	0.176014	0.001727	0.006031
11743442_RHOB	0.172905	0.00173	0.006038
11746945_UBL4A	-0.25354	0.00173	0.006039
11740138_MCTP2	0.217975	0.001732	0.006045
11743429_RBM27	0.352326	0.001732	0.006045
11734621_VPS13B	0.315786	0.001733	0.006047
11749931_AP3M2	-0.23306	0.001733	0.006047
11741272_GOPC	0.314006	0.001734	0.006049
11763901_EIF4A2	-0.25487	0.001734	0.006049
11753648_TRAF4	0.174456	0.001735	0.006055
11720158_WDR19	0.211106	0.001737	0.006059
11744871_ARF5	-0.24449	0.001737	0.006061
11763327_FER	-0.21047	0.001739	0.006066
11725013_MMACHC	-0.19412	0.001739	0.006066
11725739_ 3-Mar	-0.23293	0.001739	0.006067
11723357_SCAF11	0.204913	0.00174	0.006069
11737593_KLLN	0.288115	0.001741	0.00607
11740781_ILF3	-0.28197	0.001743	0.006079
11753362_IER5	0.242228	0.001744	0.00608
11724524_CDK5RAP2	-0.22501	0.001745	0.006083
11727767_BNC2	-0.40859	0.001746	0.006086
11720447_SOX9	0.272068	0.001746	0.006086
11748515_SLC35B2	-0.29845	0.001746	0.006087
11721626_SEPT6	-0.27041	0.001746	0.006087
11745029_NKRF	0.263799	0.001747	0.006088
11736057_BORCS7	0.206338	0.001747	0.006089

11745216_KBTBD4	-0.16535	0.001747	0.006089
11722302_LRRFIP1	-0.42128	0.001748	0.00609
11715935_NBEAL2	-0.25876	0.001748	0.00609
11717076_ATP8B5P /	0.184531	0.00175	0.006096
11759594_ERVK-19 /	0.323259	0.00175	0.006097
11748715_PEX1	0.274161	0.00175	0.006097
11742696_WAC	0.164786	0.001751	0.006099
11719624_PLEKHM1	0.204728	0.001753	0.006105
11745795_DDX5	0.173568	0.001755	0.006113
11737395_SOWAHD	-0.19107	0.001756	0.006114
11755773_FAM63B	0.255328	0.001757	0.006117
11748705_PCMT1	-0.20162	0.001757	0.006117
11717346_ADD3	-0.21543	0.001757	0.006118
11719249_MXD1	0.475932	0.001757	0.006118
11715899_SUCLG1	-0.27617	0.001757	0.006118
11753903_TP53TG1	0.224176	0.001758	0.00612
11757651_OST4	0.157607	0.001759	0.006123
11762434_CSNK1A1	-0.24008	0.001762	0.006131
11746481_TMEM219	0.177877	0.001762	0.006131
11716880_MAD2L1BP	0.196774	0.001765	0.00614
11732280_FOXP1	0.262193	0.001765	0.006141
11719232_PTPRS	-0.17935	0.001765	0.006141
11750457_GTPBP10 /	0.300663	0.001765	0.006142
11736522_TNNT1	-0.27355	0.001767	0.006146
11717445_DHPS	-0.19299	0.001768	0.006152
11749459_RAP1GAP2	-0.1929	0.00177	0.006158
11722348_REPS2	-0.21986	0.001772	0.006163
11726717_C1orf74	0.192919	0.001773	0.006168
11751783_MFSD1	0.220647	0.001774	0.00617
11723128_HERC6	0.223694	0.001775	0.006171
200098_PMANAPC5	-0.17449	0.001775	0.006173
11724571_APPBP2	0.214957	0.001776	0.006176
11722087_NR1D2	0.178597	0.001777	0.00618
11718847_SH3GLB1	-0.26416	0.001779	0.006186
11724084_KLHDC9	-0.24276	0.00178	0.006188
11715427_SNU13	-0.1837	0.00178	0.006189
11717751_HUWE1	-0.1685	0.001781	0.006189
11718907_ADCK2	-0.2389	0.001781	0.00619
11727963_CYP4F2	0.377895	0.001781	0.00619
11754935_NPRL2	-0.19987	0.001783	0.006194
11757893_TNKS2	0.16802	0.001783	0.006196
11730968_RIC8B	-0.23293	0.001783	0.006197
11754633_PPP1CA	-0.17326	0.001784	0.006199
11748511_RNF220	-0.20652	0.001784	0.006199
11726459_FAM117B	-0.34098	0.001787	0.006209
11747294_TKFC	-0.20649	0.00179	0.006219
11750770_GCLC	0.173405	0.001791	0.006222
11724582_TPRG1L	-0.27732	0.001792	0.006223
11732496_AP1S3	0.337527	0.001792	0.006225
11730175_C4orf3	-0.21653	0.001793	0.006228
11724761_CALM1 ///	-0.16182	0.001794	0.00623

11752728_SLC35A2	-0.24176	0.001797	0.006241
11726275_ATG101	0.1676	0.001797	0.006241
11730895_UBA6	-0.3664	0.001799	0.006245
11731309_C15orf56	-0.33322	0.001799	0.006245
11742975_USP42	0.295703	0.0018	0.006249
11754121_BIK	0.311743	0.001802	0.006254
11736762_ZNF33B	0.249804	0.001802	0.006256
11740421_HTR7	0.306633	0.001805	0.006262
11763927_SNORD12B	-0.42079	0.001805	0.006263
11716023_ADRBK1	-0.16473	0.001805	0.006263
11746360_REEP2	-0.22455	0.001806	0.006265
11737256_SP100	0.431786	0.001807	0.006271
11718697_C6orf89	0.205109	0.001808	0.006272
11743335_LEMD2	0.214928	0.001809	0.006275
11740817_DLC1	0.217745	0.001809	0.006275
11722104_TPRA1	0.23173	0.00181	0.006277
11725046_ANP32E	-0.20957	0.00181	0.006278
11716098_RAP1A	0.166963	0.00181	0.006278
11727049_PPP6C	0.16501	0.001811	0.006281
11758238_ABCC4	0.160721	0.001811	0.006281
11721908_SGPP1	0.304898	0.001812	0.006281
11721841_GLIPR1	0.172404	0.001812	0.006282
11730750_CRNKL1	0.182736	0.001813	0.006284
11754445_C12orf29	-0.15916	0.001813	0.006284
11716015_CMTM3	0.172688	0.001813	0.006284
11723457_MON1A	0.201157	0.001813	0.006285
11730525_SSC4D	-0.16758	0.001814	0.006288
11741341_ADAMTS12	0.247808	0.001815	0.006288
11743785_CLIP1	0.209563	0.001815	0.006288
11736445_RBAK	0.270115	0.001815	0.006288
11747521_PMS2 ///	-0.19532	0.001815	0.006289
11753062_SRRM2	-0.18484	0.001816	0.006291
11722649_RGS12	-0.18835	0.001816	0.006291
11722018_TCP1	-0.16808	0.001818	0.006299
11750796_DNER	0.307044	0.00182	0.006302
11740124_HIST1H2AM	-0.3819	0.00182	0.006304
11754345_TRAPPC12	-0.19681	0.001821	0.006306
11718790_HES6	-0.34323	0.001821	0.006306
11757320_CYB5A	-0.29726	0.001822	0.00631
11756576_GNB2	-0.22366	0.001823	0.006311
11718995_DDX20	0.211713	0.001823	0.006311
11763984_PLEKHA8	0.240651	0.001823	0.006312
11720334_ZMIZ1	-0.20422	0.001823	0.006312
11731118_PLEKHA8P1	-0.35606	0.001823	0.006312
11728790_DGCR6L	-0.33152	0.001824	0.006312
11754405_CLIC4	-0.21922	0.001824	0.006313
11749639_ZNF449	0.304086	0.001825	0.006317
11757813_AKT2	-0.2906	0.001826	0.006317
11733309_PHF12	-0.2424	0.001828	0.006325
11725794_PTGER4	0.190149	0.001829	0.006327
11717249_MRPL20	0.159459	0.001829	0.006328

11724848_DIXDC1	0.240924	0.001829	0.006328
11758411_CD99L2	-0.39736	0.001829	0.006328
11736829_MAP3K7	0.200803	0.00183	0.006329
11723970_MAPKAPK5	0.244801	0.00183	0.006331
11718668_MED9	-0.44737	0.001831	0.006332
11750548_CYP4A11	-0.22371	0.001832	0.006334
11721343_NCOA1	0.314089	0.001833	0.006338
11757885_VSIG10	-0.47113	0.001834	0.006342
11721871_HIF1AN	-0.22853	0.001835	0.006345
11759986_TMEM161B	0.231016	0.001837	0.006351
11717257_PIM1	-0.31976	0.001838	0.006356
11725909_TCL1B	0.25189	0.001839	0.006356
11719261_LMNA	-0.18033	0.001839	0.006356
11755376_TTC12	0.306725	0.001842	0.006366
11747282_ANXA2P2	0.169322	0.001842	0.006366
11723425_IFNAR1	0.259282	0.001843	0.00637
11726828_TYW1B	-0.22806	0.001845	0.006374
11752320_EIF2AK1	-0.27678	0.001846	0.006379
11720860_ARHGEF3	-0.21518	0.001846	0.006379
11752176_AREL1	0.232641	0.001848	0.006385
11723567_CAPN7	0.262951	0.00185	0.00639
11738990_GANAB	-0.17645	0.00185	0.006392
11724536_UBA5	0.246309	0.001852	0.006396
11754612_SEPT11	-0.17912	0.001852	0.006396
11748968_RDX	0.190349	0.001854	0.006402
11745034_LINC00313	-0.25535	0.001854	0.006404
11736354_CAMK2G	-0.26632	0.001855	0.006404
11745654_ST6GALNAC	0.332021	0.001857	0.006411
11740658_SLC22A5	0.400864	0.001859	0.006419
11725289_RHBDL3	-0.23146	0.00186	0.006421
11752559_PLXNB2	0.224455	0.001861	0.006426
11743716_BRWD1	0.458676	0.001862	0.006426
11725313_TMED7	-0.27483	0.001862	0.006428
11724720_GALNT12	-0.39432	0.001862	0.006428
11730736_PPP1R8	-0.22195	0.001863	0.006429
11747792_SLC31A1	0.254254	0.001863	0.006429
11724650_MEIS2	-0.25175	0.001864	0.006433
11719345_UBTD1	0.214889	0.001865	0.006435
11726566_EIF4G1	-0.17283	0.001865	0.006436
11737881_XRCC2	-0.1818	0.001866	0.006436
11730452_KIF24	-0.53723	0.001866	0.006438
11759555_CAMTA1	-0.17947	0.001868	0.006443
11720326_FRZB	0.280248	0.001868	0.006443
11748778_C1orf27	0.177379	0.001869	0.006447
11744163_AADAC	0.471379	0.00187	0.006448
11757539_STIM2	0.227183	0.00187	0.006448
11758096_ABR	-0.18385	0.00187	0.006449
11730533_COL13A1	0.225613	0.00187	0.006449
11727519_INTS6	-0.31392	0.001871	0.00645
11721849_REEP2	-0.2009	0.001872	0.006452
11756685_SEC23IP	0.173748	0.001872	0.006453

11735096_MLX	0.175372	0.001872	0.006453
11750800_LNX1	0.333585	0.001873	0.006455
11743551_PAK1IP1	0.160608	0.001874	0.006459
11720937_YARS2	0.165804	0.001875	0.00646
11751949_FAM21C	0.203005	0.001876	0.006465
11757463_IGSF8	-0.18113	0.001877	0.006467
11739825_DNAJB12	-0.17109	0.001878	0.006469
11726403_OPTC	-0.19646	0.00188	0.006478
11744442_CCDC82	0.223915	0.00188	0.006479
11718390_TRIM26	0.18672	0.001881	0.006481
11726679_AFAP1L2	0.161719	0.001882	0.006481
11743741_CWC25	0.257582	0.001882	0.006484
11715832_SURF4	-0.171	0.001883	0.006484
11759864_ZNF609	0.197956	0.001884	0.006487
11727818_DDX46	-0.17423	0.001884	0.006489
11763960_NDUFB3	-0.24111	0.001885	0.006492
11727930_ATP2B1	0.229362	0.001887	0.006496
11755182_FILIP1L	-0.18651	0.001887	0.006497
11716807_CSNK2A2	0.215987	0.001887	0.006497
11718137_LOC105372	-0.18247	0.001887	0.006497
11737955_C15orf40	0.163206	0.001887	0.006497
11723657_ZNF668	0.20586	0.001888	0.006498
11733553_MAGI1	0.344872	0.001888	0.006499
11732239_TMEM259	-0.2303	0.001889	0.0065
11741003_ZNF3	0.216703	0.00189	0.006504
11757112_SNHG8	0.241555	0.00189	0.006504
11748298_BNIP2	0.209301	0.00189	0.006504
11728105_TMPRSS3	0.250873	0.00189	0.006504
11757964_SNTB1	-0.237	0.001892	0.006507
11757115_HSPA8	-0.33494	0.001893	0.006511
11722836_FUBP1	0.195177	0.001895	0.006517
11757523_WDR35	-0.25396	0.001895	0.006519
11722046_LDAH	0.208857	0.001895	0.006519
11717536_TMEM259	-0.20412	0.001896	0.006519
11745762_AHSA1	-0.17652	0.001896	0.006519
11718063_C11orf98	-0.20134	0.001896	0.006519
11749584_UBA2	-0.18187	0.001896	0.00652
11747104_CYP1B1	0.370317	0.001897	0.006523
11726622_DIS3	-0.20419	0.001898	0.006524
11753082_ZDHHC15	0.203134	0.001898	0.006525
11720040_VTA1	0.188351	0.001901	0.006536
11755132_SNHG7 ///	0.221998	0.001902	0.006536
11736177_TRNT1	0.178504	0.001903	0.006538
11716128_IGF2R	0.214142	0.001903	0.006538
11755128_SRSF3	-0.17715	0.001903	0.00654
11749995_RNF216P1	0.206664	0.001905	0.006546
11745235_DPYD	0.27448	0.001905	0.006546
11743123_TLN1	-0.22067	0.001907	0.006551
11734748_LOC100507	-0.20147	0.001908	0.006554
11746589_PRSS54	-0.26753	0.001909	0.006556
11719952_FKBP15	0.280061	0.001909	0.006557

11717928_FOLR1	0.482087	0.001909	0.006557
11732726_PIGW	-0.17948	0.00191	0.006559
11740606_HYKK	-0.29327	0.001912	0.006565
11745516_STX16	0.268843	0.001912	0.006565
11725195_ZSWIM7	0.3067	0.001912	0.006565
11746723_NPC1	0.309038	0.001915	0.006575
11719057_PDE8A	0.201322	0.001915	0.006575
11723623_TRIM21	0.244544	0.001916	0.006577
11748307_AIMP1	-0.15873	0.001917	0.006579
11719396_EXTL2	-0.18443	0.001918	0.006583
11719506_DHX36	0.157826	0.001918	0.006584
11719965_U2AF1 ///	-0.21769	0.00192	0.006591
11732514_LYN	0.333193	0.001921	0.006592
11754052_NUP85	-0.25766	0.001921	0.006593
11751640_PHF11	0.190658	0.001922	0.006593
11721435_STC2	0.583304	0.001922	0.006594
11743369_PPP1R2	-0.20783	0.001922	0.006594
11757321_TAPBP	0.211655	0.001922	0.006595
11724817_SH3KBP1	0.19657	0.001926	0.006608
11742096_SCLY ///	0.169143	0.001927	0.00661
11750575_ZFPM2	-0.27296	0.001928	0.006614
11732876_TRPM8	0.435759	0.00193	0.006619
11745122_FNIP1	0.448706	0.00193	0.006619
11736223_IKZF5	0.23587	0.001932	0.006625
11757512_ADI1	-0.19766	0.001932	0.006626
11763859_UTY	0.376722	0.001933	0.006627
11721459_TAPBPL	0.18442	0.001933	0.006627
11722448_KCNMB4	-0.22269	0.001934	0.006629
11720375_PRUNE	0.218887	0.001934	0.00663
11749779_TTC26	0.277199	0.001934	0.006631
11741568_FUT8	-0.16854	0.001935	0.006632
11758290_CCDC6	-0.18393	0.001939	0.006644
11745636_MON2	0.294766	0.001939	0.006645
11743585_DENR	0.16183	0.001939	0.006645
11744010_TMEM175	-0.18321	0.001939	0.006646
11727001_CSNK1E //	-0.20555	0.001943	0.006657
11756598_HKR1	0.251402	0.001945	0.006663
11733925_RIOK1	0.170709	0.001945	0.006663
11746025_MILR1	0.351299	0.001947	0.006669
11758800_SERBP1	-0.19581	0.001947	0.006669
11749318_TSTA3	0.191597	0.001948	0.006671
11739872_NME6	0.255468	0.001948	0.006673
11747193_ZNF185	-0.2112	0.001949	0.006674
11750567_MTOR	-0.24651	0.001951	0.00668
11724914_SPATA18	-0.29301	0.001952	0.006685
11733310_PCYT1B	-0.4002	0.001955	0.006693
11759883_SRP19 ///	0.256705	0.001956	0.006695
11734981_RALY	-0.23442	0.001956	0.006697
11750263_ZNF786	0.157766	0.001958	0.006702
11746086_LOC728752	-0.19989	0.001958	0.006702
11759125_FBXL17	-0.36484	0.001959	0.006704

11757399_PRKDC	-0.24522	0.001959	0.006704
11727546_PANK3	0.217037	0.001962	0.006714
11750610_ZNF268	0.250523	0.001962	0.006714
11726050_ABHD17C	-0.21192	0.001963	0.006718
11721718_ATRX	0.247589	0.001964	0.006719
11726627_ZC3H10	-0.24039	0.001964	0.00672
11748332_HLA-E	0.218403	0.001964	0.00672
11757272_JTB	0.160848	0.001965	0.00672
11717992_DNAJC7	0.18154	0.001965	0.006721
11744931_ELF2	0.217618	0.001965	0.006722
11741398_GPR63	0.196757	0.001967	0.006726
11748532_IST1	0.208586	0.001967	0.006727
11735642_FAM132A	-0.19771	0.001968	0.00673
11722488_ALG11 ///	-0.31721	0.001969	0.006732
11751980_RASGRF1	0.40714	0.001969	0.006732
11759310_FAM171B	-0.24449	0.001971	0.006738
11722076_DONSON	-0.27313	0.001972	0.006741
11727877_MIA-RAB4E	0.237494	0.001972	0.006741
11737228_SETD4	0.26884	0.001972	0.006741
11717177_SNX27	-0.16559	0.001973	0.006743
11763545_ATP10B	-0.25311	0.001973	0.006743
11731254_C16orf13	-0.23812	0.001973	0.006743
11748148_COQ6	0.164285	0.001975	0.00675
11718132_RBP4	-0.28289	0.001976	0.006753
11722186_AKT1	-0.18266	0.001977	0.006755
11727337_ADD2	-0.18662	0.001978	0.006758
11746170_LOC440173	-0.24435	0.001979	0.006762
11724470_MGST1	-0.24077	0.001979	0.006762
11733101_USP12	0.256783	0.00198	0.006763
11743500_PSMG4	-0.20368	0.001981	0.006767
11732840_ERC2	-0.32446	0.001981	0.006767
11761019_LINC00525	-0.35858	0.001982	0.006768
11752833_PGAP2	-0.21695	0.001982	0.00677
11739914_CRELD1	-0.23405	0.001983	0.00677
11727920_OSTF1	0.237112	0.001984	0.006774
11723262_AMN1	-0.24206	0.001985	0.006776
11753714_HBE1	0.19915	0.001986	0.00678
11723290_CRISPLD1	-0.26266	0.001986	0.00678
11747933_NAPRT	-0.15769	0.001988	0.006784
11725245_SDK1	0.424798	0.001988	0.006784
11725271_PIGA	0.165922	0.001988	0.006785
11740434_MARK3	0.274769	0.001988	0.006785
11760517_AP2M1	-0.25508	0.001988	0.006786
11739877_BSG	-0.17083	0.001989	0.006787
11720372_TESC	0.30616	0.00199	0.006789
11729728_METRN	-0.22981	0.00199	0.006789
11744709_HRAS	0.178101	0.001992	0.006796
11743907_CTPS1	-0.2059	0.001992	0.006796
11760664_TINAG	0.339588	0.001993	0.006798
11720100_SPIN1	0.205258	0.001993	0.006798
11739520_CHID1	-0.20974	0.001995	0.006804

11759346_MAML2	0.214548	0.001995	0.006804
11733879_HMGXB4	-0.18603	0.001995	0.006804
11719325_KDM1A	-0.17353	0.001997	0.006809
11716960_FAM3A	-0.18609	0.001998	0.006811
11728631_LYSMD1	0.364048	0.001999	0.006817
11759148_FBXO3	-0.18949	0.002001	0.006822
11741162_SLC12A4	0.202507	0.002002	0.006824
11748421_TMEM18	-0.26523	0.002002	0.006825
11743984_AKAP1	-0.22614	0.002002	0.006825
11757750_DHX8	0.196334	0.002003	0.006825
11763033_RPS16	-0.1738	0.002003	0.006825
11764270_PLGLB1 //	0.404918	0.002003	0.006826
11761131_NOL12	-0.18462	0.002004	0.006828
11729213_PCBP2	-0.25677	0.002004	0.006829
11718841_CXCL8	0.166159	0.002005	0.00683
11724972_PIGN	-0.23697	0.002005	0.006831
11757746_ST13	-0.17928	0.002005	0.006831
11753932_PHAX	0.188469	0.002006	0.006834
11716736_PRPF19	-0.2482	0.002007	0.006836
11731542_SESTD1	-0.28057	0.002011	0.006849
11720946_CA12	-0.20203	0.002012	0.006852
11715990_COX7A2L	0.155947	0.002013	0.006855
11745888_USP42	0.241833	0.002014	0.006859
11754464_GIT1	-0.23034	0.002017	0.006867
11751365_SLC47A1	-0.38206	0.002019	0.006872
11756554_PSMC5	-0.16333	0.002021	0.00688
11734751_MAT2A	0.168046	0.002021	0.00688
11743666_SPEN	0.177342	0.002022	0.006884
11721458_MAN1B1	-0.18877	0.002022	0.006884
11752055_TERT	-0.26611	0.002023	0.006885
11731308_KCNIP4	0.35302	0.002024	0.006887
11717989_SUN1	0.155589	0.002024	0.006888
11715695_COX7B	-0.15568	0.002024	0.006888
11748931_MUC5B	-0.16161	0.002025	0.006888
11758505_HOXD13	-0.23118	0.002025	0.006888
11731484_NFIC	-0.20728	0.002026	0.006891
11731573_FRMD4B	0.253154	0.002026	0.006892
11743466_MPHOSPH6	-0.15357	0.002027	0.006894
11721528_ZDHHC2	0.277363	0.002029	0.006902
11734676_ITIH5	0.422129	0.002031	0.006908
11719378_RAP1GDS1	0.187704	0.002031	0.006908
11716747_AFF1	0.255837	0.002032	0.006908
11718742_DCTN4	0.175182	0.002034	0.006916
11763273_ZBTB8OS	0.252745	0.002034	0.006917
11756523_SHISA5	-0.21432	0.002035	0.006917
11720726_UBR1	0.230901	0.002035	0.006917
11757741_SOX4	-0.2511	0.002039	0.00693
11763691_VTI1B	-0.47376	0.002039	0.00693
11747834_SPATA5	-0.3219	0.002039	0.006931
11757822_C11orf31	0.159593	0.00204	0.006932
11752509_PSAP	0.173908	0.00204	0.006932

11718756_APLP1	-0.46074	0.00204	0.006932
11746824_PXMP4	-0.3851	0.00204	0.006932
11757239_NFYC-AS1	0.228332	0.002041	0.006934
11743084_BNIP3	-0.15841	0.002041	0.006935
11726625_CCNG2	-0.25385	0.002042	0.006936
11748153_PTCD2	0.200442	0.002042	0.006938
11728060_LRTOMT	0.382079	0.002044	0.006944
11726621_TMEM106C	-0.17297	0.002044	0.006944
11721719_NAAA	-0.26735	0.002045	0.006944
11730843_MXI1	-0.19732	0.002045	0.006944
200021_PMCFL1	-0.15704	0.002047	0.00695
11745190_FUS	-0.16977	0.002047	0.006952
11750883_EIF2A	0.174126	0.002047	0.006952
11746868_GIT2	0.219711	0.002048	0.006953
11720415_CAMSAP1	0.194605	0.002048	0.006954
11744927_SLC9B2	0.360455	0.002049	0.006956
11739463_GIGYF2	0.267337	0.00205	0.006957
11716900_ARL3	-0.19547	0.002051	0.006963
11719753_ADAMTS9	0.215157	0.002053	0.006967
11743456_AP5M1	-0.20281	0.002053	0.006968
11730360_CCDC126	0.167437	0.002054	0.006969
11722218_WBP4	0.194912	0.002055	0.006973
11723253_SCNM1 ///	0.190817	0.002056	0.006975
11743587_BECN1	0.181843	0.002056	0.006976
11761741_DEPDC7	0.249078	0.002057	0.006979
11755799_TIE1	-0.48231	0.002058	0.00698
11744228_ORAI3	-0.17914	0.002059	0.006983
11762614_TRIM63	-0.25926	0.00206	0.006987
11756421_HTRA2	-0.19796	0.002061	0.006989
11751049_NUCB1	-0.18018	0.002061	0.006989
11732366_SCAPER	0.331854	0.002061	0.006989
11761007_FIBP	0.435092	0.002062	0.006991
11758187_ST13	0.155298	0.002062	0.006991
11726116_MTAP	-0.19222	0.002063	0.006995
11740197_ISPD	-0.622	0.002064	0.006996
11728465_ZNF562	0.233086	0.002065	0.007
11739967_DNAJC24	-0.43573	0.002065	0.007001
11724989_CLTCL1	0.410307	0.002066	0.007004
11761480_MAST4	-0.23702	0.002067	0.007005
11762129_CFHR3	0.235842	0.002067	0.007005
11739876_BSG	-0.20404	0.002068	0.007008
11751210_CYB561	-0.16621	0.002072	0.007021
11739316_KDR	0.178679	0.002072	0.007021
11743951_ZNF214	0.453387	0.002072	0.007021
11752017_SPECC1L /	-0.17478	0.002073	0.007022
11718642_OSBPL8	-0.19439	0.002073	0.007023
11734965_TRIM65	-0.2565	0.002073	0.007023
11759541_RPL35A	0.196134	0.002073	0.007023
11743834_RSBN1L	0.194193	0.002074	0.007025
11736207_PLXNC1	0.292114	0.002075	0.007028
11727247_CHRNB1	0.326692	0.002076	0.007028

11742168_FAM27L	-0.17207	0.002076	0.007028
11722808_SP1	-0.18661	0.002076	0.00703
11715732_PSMB3	-0.16743	0.002077	0.007033
11716735_RNF185	0.18792	0.002077	0.007033
11750697_SLC44A5	0.482687	0.002078	0.007033
11715886_PIP4K2B	0.393876	0.002079	0.007037
11751090_CBLB	0.456761	0.00208	0.00704
11716519_SDR39U1	-0.22672	0.002081	0.007042
11725029_PNN	0.1927	0.002081	0.007042
11744898_PRRC2C	0.156057	0.002082	0.007046
11733812_SLC25A17	0.179386	0.002083	0.007048
11731635_GAB1	0.230971	0.002084	0.007051
11723867_SYNJ1	0.505419	0.002084	0.007051
11722623_TMEM8A	0.216481	0.002086	0.007056
11735284_KMT2E	0.173301	0.002086	0.007057
11733727_SNAP25	0.393199	0.002087	0.00706
11717197_SCFD1	0.203818	0.002088	0.007062
11716158_RNF40	0.164092	0.002089	0.007065
11746259_PIGA	0.241754	0.002089	0.007066
11734569_DCTN4	0.177536	0.00209	0.007067
11738389_TCF23	-0.17783	0.00209	0.007067
11759484_STAG1	-0.22932	0.00209	0.007067
11750081_TIE1	-0.24665	0.00209	0.007067
11719758_SMCR8	0.200669	0.002091	0.007069
11749124_SMCR8	0.200669	0.002091	0.007069
11746138_AKT2	-0.21676	0.002092	0.007072
11748239_TBCD	-0.26621	0.002092	0.007073
11720588_HSD17B14	-0.18032	0.002094	0.007078
11723603_C14orf79	0.184687	0.002095	0.00708
11732799_ACER2	0.640273	0.002095	0.00708
11722090_EFNA4	-0.23905	0.002097	0.007084
11739459_COX20	0.197425	0.002097	0.007084
11726715_DDX59	-0.25832	0.002097	0.007085
11735653_FAM213B	-0.40298	0.002098	0.007087
11736332_PHF20	-0.20316	0.002099	0.00709
11753090_OGFR	-0.19063	0.002099	0.007091
11758655_RRAGA	0.162738	0.0021	0.007094
11764011_TMPO	-0.33341	0.0021	0.007094
11721088_YLPM1	-0.18232	0.002101	0.007094
11733895_TADA2B	0.219618	0.002102	0.007097
11758940_MOB1A	-0.18446	0.002103	0.007102
11740797_POLR1C	0.210375	0.002105	0.007107
11716932_CCDC80	0.22757	0.002106	0.00711
11752465_RPS6KB1	0.281572	0.002107	0.007112
11759638_MESDC2	0.26011	0.002107	0.007112
11755472_DBR1	0.169854	0.002107	0.007113
11757643_SERPINE2	0.172474	0.002109	0.00712
11745272_ATP6V1C1	0.245037	0.00211	0.007121
11739308_DLG1	0.158168	0.002111	0.007125
11739700_STON1	0.275885	0.002111	0.007126
11750594_PLCL1	0.277578	0.002113	0.00713

11757348_SLU7	0.206372	0.002113	0.00713
11732392_TPI1	-0.17707	0.002114	0.007133
11739525_LPP	0.248519	0.002114	0.007133
11746276_RGS12	-0.37676	0.002115	0.007134
11718566_KIAA1147	0.192891	0.002116	0.007137
11728013_RIMKLB	0.245829	0.002116	0.007137
11736962_MBNL2	0.198687	0.00212	0.007149
11748716_ERCC6L2	0.230274	0.00212	0.007151
11763518_PMS1	-0.22998	0.002122	0.007156
11728302_TGFA	0.182209	0.002122	0.007156
11755146_SGIP1	0.223297	0.002123	0.007158
11721486_TP53RK	-0.18921	0.002123	0.007158
11752015_AP4E1	0.311121	0.002125	0.007164
11739903_STAG2	0.179776	0.002125	0.007165
11751525_CCDC69	-0.23016	0.002126	0.007167
11738255_C7orf57	0.588149	0.002126	0.007167
11757662_FBXO16	-0.20506	0.002126	0.007167
11722568_NT5C2	0.233979	0.002128	0.007171
11753930_ZNF821	0.301069	0.002128	0.007171
11716296_FLOT2	-0.23703	0.002129	0.007174
11725949_KRI1	0.250235	0.002129	0.007174
11740957_SLC2A6	0.163883	0.002129	0.007174
11717296_LRPPRC	0.192684	0.002129	0.007174
11723936_SNX1	-0.17937	0.00213	0.007176
11719059_ATRN	-0.23587	0.002131	0.007179
11736326_SWSAP1	-0.23992	0.002133	0.007185
11740992_DBNL	-0.18175	0.002135	0.00719
11746247_GK3P	-0.24757	0.002136	0.007192
11736430_NOL8	0.179899	0.002141	0.007212
11736667_PTER	-0.25815	0.002143	0.007216
11731771_PCDHB15	0.339838	0.002143	0.007216
11752673_LMNA	-0.16195	0.002143	0.007216
11715830_PLS3	-0.16209	0.002145	0.007223
11754605_TCF4	0.18262	0.002146	0.007225
11723266_SAYSD1	-0.27825	0.002147	0.007228
11758727_ATP5C1	-0.21371	0.002148	0.007229
11715399_SLC25A5	-0.16165	0.002149	0.007234
11724801_NSRP1	0.265322	0.002149	0.007234
11718039_DCAF12	0.205698	0.00215	0.007236
11727306_ZNF320	0.219576	0.00215	0.007236
11715401_ILF2	-0.15758	0.002151	0.00724
11755024_MYEOV	0.312201	0.002152	0.007241
11735345_C1QTNF5 /	-0.21414	0.002154	0.007249
11750870_NUP160	0.290858	0.002155	0.007252
11741411_ERC1	0.241383	0.002156	0.007252
11754261_SLC39A1	-0.24115	0.002156	0.007253
11746452_ZHX1	0.389532	0.002159	0.007262
11759577_MRS2	0.19049	0.002163	0.007277
11739517_DFFA	-0.18886	0.002164	0.007278
11721853_NCAPH	-0.27475	0.002165	0.007281
11737874_SULF1	0.425262	0.002166	0.007283

11723711_SLC24A3	0.317735	0.002166	0.007283
11722156_PRKRA	0.162415	0.002168	0.007288
11731098_FAM161A	-0.24505	0.002168	0.007288
11732459_ZNF599	-0.21573	0.002168	0.007289
11726370_BACH1 ///	0.352461	0.002171	0.007298
11729575_XBP1	0.159077	0.002171	0.007298
11731187_PPM1E	0.320479	0.002172	0.0073
11731881_PPP2R1B	0.198334	0.002172	0.007301
11760565_AKAP9	0.217396	0.002173	0.007304
11739553_GAS7	0.233616	0.002176	0.007311
11745517_SDHB	-0.15619	0.002176	0.007312
11757596_TRIM13	0.190673	0.002177	0.007315
11718012_IRS2	0.185128	0.002178	0.007317
11739985_AMACR ///	0.239225	0.002179	0.00732
11740683_PRKG1	-0.28376	0.00218	0.007322
11733211_BRCC3	0.187055	0.00218	0.007323
11747200_GTF2F1	0.177178	0.002181	0.007325
11747374_ZNF738	0.310221	0.002182	0.007328
200020_PM TARDBP	-0.18796	0.002182	0.007329
11724913_ILK	0.175816	0.002183	0.007329
11726551_RAET1L //	-0.24134	0.002183	0.007329
11733144_IMPA1	0.153218	0.002183	0.00733
11746814_NAB1	0.209338	0.002184	0.007331
11742702_C17orf89	0.206392	0.002184	0.007333
11715857_NCKAP1	0.185625	0.002185	0.007334
11715416_DDB1	-0.19173	0.002185	0.007334
11719712_PPM1B	0.205489	0.002185	0.007334
11723762_PLCG1	-0.34957	0.002186	0.007335
11728814_OTUD7B	0.189728	0.002186	0.007336
11733067_NFYB	-0.18115	0.002188	0.007342
11748609_ZNF841	0.267824	0.00219	0.00735
11755087_C4orf47	0.279834	0.002191	0.00735
11735030_CLASP1	0.328017	0.002194	0.007359
11743529_OPA1	0.235517	0.002194	0.007362
11726118_CPN1	-0.19343	0.002195	0.007364
11754471_HLA-DMB	-0.25634	0.002196	0.007367
11723335_GMIP	-0.19956	0.002196	0.007367
11748954_MBD1	0.243474	0.002197	0.007368
11725536_ANKRD34A	0.168207	0.002198	0.00737
11715898_TNIP1	0.162627	0.002198	0.007371
11715873_OLFML3	0.217473	0.002198	0.007372
11731580_KCTD20	0.293133	0.002199	0.007374
11748297_PPP2R5E	0.182669	0.002201	0.00738
11733009_LUC7L3	0.154014	0.002203	0.007384
11718437_ATPAF2	0.204778	0.002203	0.007384
11744236_DDX60	0.28669	0.002203	0.007384
11744449_ARHGAP4	-0.18273	0.002205	0.00739
11723035_GTF2A2	0.191178	0.002205	0.00739
11749585_RMDN2	0.410843	0.002205	0.007391
11727804_PRDX3	-0.18621	0.002207	0.007396
11720117_SIPA1	-0.22899	0.002208	0.007398

11728570_ZFPM2	-0.22274	0.002208	0.007398
11720590_PHF21A	-0.25111	0.002212	0.00741
11718194_VEZF1	0.165651	0.002212	0.00741
11756646_IRF2BP2	0.15943	0.002213	0.007412
11753466_ACSS2	0.254707	0.002213	0.007413
11717412_ANO6	0.212197	0.002213	0.007413
11735948_ANAPC7	0.237342	0.002215	0.007419
11756427_ARL16	0.192097	0.002216	0.007422
11725664_EDNRB	0.241877	0.002217	0.007425
11722397_RWDD4	-0.26542	0.002218	0.007425
11727831_DDX42	-0.39324	0.002218	0.007425
11754225_SEC23B	-0.17646	0.002218	0.007425
11757760_DNAJB12	-0.21969	0.002218	0.007425
11742478_NRG1	0.304777	0.002219	0.007427
11715277_AURKAIP1	-0.2039	0.002219	0.007429
11752616_DLG1	0.258263	0.002223	0.007442
11753739_CYB5A	-0.24401	0.002224	0.007445
11728690_RABIF	0.264418	0.002226	0.00745
11740232_ASL	-0.20907	0.002226	0.00745
11763572_POLE3	-0.17844	0.002226	0.00745
11716593_BTBD1	0.194412	0.002228	0.007455
11751575_ACTN2	0.349377	0.00223	0.00746
11754594_SLC26A2	0.155692	0.00223	0.007461
11724853_LRP3	-0.22443	0.00223	0.007461
11740100_ZNF37A	0.222544	0.00223	0.007461
11764080_USP6NL	-0.3529	0.002231	0.007463
11724320_C11orf54	0.20061	0.002232	0.007465
11742899_CGGBP1	0.201379	0.002233	0.007467
11748624_HYOU1	0.194298	0.002233	0.007467
11746194_METTL3	-0.17854	0.002237	0.007481
11715608_PPIB	0.155495	0.002237	0.007481
11725111_NDE1	-0.18794	0.002239	0.007485
11719814_BET1	-0.44144	0.00224	0.007488
11721321_LYRM4	0.225652	0.002241	0.007494
11717336_RBM6	0.176022	0.002242	0.007494
11763478_PIF1	-0.2954	0.002242	0.007495
11743547_ASAH1	0.184788	0.002243	0.007497
11759895_PCDH9	-0.19891	0.002245	0.007503
11761399_WRB	-0.25387	0.002249	0.007515
11728666_PCBP2	-0.162	0.002249	0.007517
11733924_RIOK1	0.207639	0.002252	0.007524
11739601_AASS	-0.27061	0.002253	0.007529
11740371_PDHX	0.206917	0.002253	0.007529
11752409_NDC1	0.201022	0.002254	0.00753
11755156_REPS1	0.211212	0.002255	0.007533
11758850_EMC1	-0.28435	0.002255	0.007533
11725159_FBXW2	0.242146	0.002257	0.00754
11758386_MANBA	0.320564	0.002258	0.007542
11738552_ANXA2	0.185526	0.002258	0.007542
11758202_ATPIF1	-0.18926	0.002259	0.007544
11717891_ECM1	0.178697	0.002262	0.007553

11756700_TRIM26	0.16441	0.002262	0.007553
11743837_NCBP3	0.271012	0.002265	0.007562
11745084_FAM168B	0.239847	0.002266	0.007566
11753034_ZNFX1	-0.20031	0.002267	0.007569
11735220_LPAR3	-0.30839	0.002268	0.007572
11718423_SMARCA5	0.167676	0.002269	0.007573
11738057_TMEM136	0.180921	0.00227	0.007578
11733012_PEX2	-0.27195	0.00227	0.007578
11760622_C8orf34	-0.2431	0.002271	0.007579
11737339_ABCG8	-0.19629	0.002272	0.007581
11742103_TLE2	-0.38271	0.002273	0.007584
11745934_LOC102724	0.170518	0.002274	0.007586
11755567_DMPK	0.253445	0.002276	0.007592
11754274_ST6GALNAC	0.319082	0.002276	0.007594
200014_PMHNRNPC	-0.18607	0.002277	0.007596
11755669_DHX33	0.343921	0.002278	0.007599
11744750_LGI4	-0.15774	0.002279	0.0076
11750323_HMGN2P46	-0.18897	0.00228	0.007603
11719275_PSMA2	-0.16137	0.002281	0.007606
11726136_TMSB15B	0.195176	0.002282	0.007608
11750600_CLCA3P	0.264154	0.002282	0.007611
11717280_URM1	-0.1629	0.002284	0.007616
11748839_NFS1	-0.22483	0.002285	0.007617
11741688_LRP5L	0.377441	0.002287	0.007624
11734267_HOXC11	0.219925	0.002288	0.007626
11723040_FABP1	-0.33668	0.002288	0.007627
11759517_GRIP2	-0.29115	0.002288	0.007627
11724438_CCDC24	0.201646	0.002289	0.007627
11729260_ZNF644	0.203682	0.00229	0.007632
11719772_C5orf24	-0.33188	0.00229	0.007632
11754307_CAPN1	-0.20529	0.00229	0.007632
11759729_TTC14	0.351893	0.002291	0.007632
11737755_ABCB6 ///	0.158818	0.002291	0.007632
11761553_KATNBL1	-0.25375	0.002291	0.007633
11748865_TMEM139	0.286377	0.002292	0.007634
11742306_SULT1C3	-0.24505	0.002292	0.007634
11756729_CTDSP2	0.200072	0.002292	0.007635
11744717_C18orf8	0.161105	0.002293	0.007637
11742457_PTBP1	-0.17009	0.002294	0.007641
11750255_ARID1A	-0.1575	0.002296	0.007646
11739145_ITGA3	0.220636	0.002298	0.007652
11716813_GATM	0.252576	0.002298	0.007652
11742701_C1R	-0.31682	0.0023	0.007656
11757713_ATXN2L	-0.18141	0.0023	0.007656
11721891_ADGRL1	-0.22125	0.0023	0.007657
11759073_DNAJC9	-0.24897	0.0023	0.007657
11715411_EPAS1	0.191674	0.002302	0.007664
11738807_CMIP	-0.18159	0.002303	0.007664
11761114_AKR1C2	-0.26852	0.002303	0.007665
11737289_FLJ44635	-0.15439	0.002303	0.007665
11756979_TUBA1A //	-0.16504	0.002304	0.007666

11722147_PAIP2B	-0.20067	0.002305	0.007668
11730952_PDK3	-0.27835	0.002305	0.00767
11739149_SAMHD1	0.270704	0.002305	0.00767
11717893_PTP4A1	0.19863	0.002306	0.007671
11727978_SRGAP1	0.176654	0.002307	0.007673
11729580_CDH13	0.172573	0.002307	0.007675
11723716_CYB5D1	0.212508	0.002309	0.007679
11740484_USP51	-0.27525	0.002309	0.007679
11743241_LINC01198	0.197904	0.00231	0.007683
11743567_ACBD3	0.168076	0.002311	0.007686
11748987_REEP5	-0.22717	0.002313	0.00769
11758581_IPO9	-0.21248	0.002315	0.007697
11719889_GIGYF2	-0.28961	0.002315	0.007697
11740221_ZNF273	-0.33353	0.002315	0.007697
11727938_SEMA3B	0.210082	0.002317	0.007701
11720352_ETV5	-0.16203	0.002317	0.007701
11742116_LDHA	-0.17263	0.002318	0.007704
11737901_METTL5	-0.19753	0.002318	0.007704
11722875_LRBA	0.597875	0.00232	0.007709
11724976_ZNF579	0.163357	0.002322	0.007717
11743410_RBM25	0.202262	0.002324	0.007723
11721381_GNPTAB	0.249175	0.002325	0.007725
11746448_ARHGAP35	-0.24299	0.002327	0.007731
11723704_ABHD5	0.330198	0.00233	0.007741
11740736_TMC7	0.445287	0.002331	0.007744
11762560_RGS11	-0.29775	0.002331	0.007744
11763956_SCARNA14	0.229877	0.002333	0.007749
11742552_OR13C8	0.20751	0.002335	0.007755
11722695_ZMYM4	0.166778	0.002337	0.00776
11735606_RAB12	0.203162	0.002338	0.007766
11733409_MPND	-0.21416	0.002339	0.007768
11735038_SORCS1	0.218541	0.00234	0.007768
11759331_B3GAT2	0.248679	0.00234	0.007768
11716019_RAB31	0.23597	0.00234	0.00777
11737852_PPP1R18	0.18499	0.002341	0.007773
11725372_VEGFA	-0.18651	0.002343	0.007779
11756753_IQGAP3	-0.31454	0.002344	0.00778
11751563_GLYCTK	-0.22281	0.002344	0.00778
11747069_DDX11 ///	-0.40168	0.002344	0.00778
11720351_ETV5	-0.2279	0.002344	0.00778
11718887_TPRKB	-0.21107	0.002345	0.007781
11723528_LENG8	0.184164	0.002346	0.007783
11738041_TNIP3	-0.25905	0.002346	0.007784
11745942_PITRM1	0.164338	0.002346	0.007784
11722484_C8orf82	-0.28278	0.002348	0.007788
11756454_EXOC7	-0.16451	0.002349	0.007793
11761671_ETV7	0.360991	0.00235	0.007794
11729539_IMMP1L	-0.24249	0.00235	0.007794
11731924_KCNRG	-0.19458	0.002351	0.007797
11752326_ANKRD19P	0.238412	0.002354	0.007807
11762347_RNF7	0.312382	0.002355	0.007809

11725578_BEND3	-0.26043	0.002357	0.007816
11721464_OSBPL2	-0.18676	0.002358	0.007818
11718836_NDUFS2	-0.17639	0.00236	0.007823
11732342_ZNF33A //	0.380342	0.002361	0.007827
11729872_SLC8A3	-0.23699	0.002361	0.007827
11754753_SCAF11	0.188692	0.002362	0.007829
11743062_PLAUR	0.155045	0.002365	0.00784
11749641_ZNF254	0.344629	0.002366	0.00784
11759566_NPL	-0.2325	0.002367	0.007844
11754297_TNFRSF1A	0.187123	0.002369	0.00785
11727277_TMEM169	0.212828	0.002369	0.007852
11730119_CLDND2	-0.24413	0.00237	0.007853
11738493_OR4F17 //	0.182454	0.00237	0.007853
11739175_HNRNPM	-0.18025	0.00237	0.007853
11719342_ATF3	0.298605	0.002372	0.007858
11739430_FCF1 ///	-0.22691	0.002373	0.00786
11740582_ASAH1	0.158636	0.002375	0.007867
11734892_SOBP	-0.24203	0.002378	0.007876
11751713_C6orf62	-0.206	0.002379	0.007877
11719773_C5orf24	-0.18497	0.00238	0.00788
11726382_SLAMF1	-0.18058	0.00238	0.00788
11745311_SH3GLB2	-0.19974	0.002383	0.007889
11743195_PRRG2	-0.27208	0.002384	0.007893
11718669_MED9	-0.45715	0.002384	0.007893
11731626_CCDC186	0.294853	0.002384	0.007893
11743794_CFAP69	0.415319	0.002385	0.007893
11752304_ITGB4	0.236448	0.002386	0.007897
11751113_SNX11	0.308295	0.002387	0.0079
11756481_FDXR	0.194976	0.002395	0.007926
11730186_IDH3B	-0.19334	0.002396	0.007928
11742752_ETS2	0.18125	0.002396	0.007929
11749715_TUBB4A	-0.45149	0.002397	0.007932
11721319_LYRM4	0.198748	0.002399	0.007937
11747312_WBP11	-0.17469	0.0024	0.007941
11748888_FTO	-0.27422	0.002402	0.007946
11734995_SKI	0.173052	0.002403	0.007949
11716466_ISCA1	-0.19391	0.002403	0.007949
11753261_DENR	0.16818	0.002404	0.00795
11720882_BOLA1	-0.36648	0.002404	0.007951
11723898_CCBL1	-0.17436	0.002404	0.007952
11739201_ATP5G3	-0.16328	0.002406	0.007957
11731645_CAMKK2	-0.2172	0.002407	0.00796
11721373_C10orf35	-0.25539	0.002409	0.007965
11734066_IRAK4	0.178259	0.002413	0.007977
11749767_PCYOX1	-0.25248	0.002414	0.007982
11717284_WASL	0.22131	0.002415	0.007984
11724748_CHMP2B	0.15942	0.002416	0.007985
11728242_SCO1	0.165815	0.002419	0.007995
11716868_UTP18	0.14901	0.002419	0.007995
11743293_BCKDK	-0.15637	0.002419	0.007996
11753175_ADIPOR1	0.190285	0.00242	0.007999

11753732_PAOX	-0.27384	0.002421	0.008001
11737894_SLC12A6	0.30146	0.002422	0.008002
11757914_KNOP1	-0.17422	0.002422	0.008002
11755533_ADGRA3	-0.22978	0.002422	0.008003
11747650_LRRC41	-0.22676	0.002423	0.008005
11760960_EML2	-0.20928	0.002424	0.008007
11723295_PIGB	0.185633	0.002424	0.008007
11758682_TMEM261	-0.28796	0.002426	0.008013
11719688_TCF25	-0.1994	0.002427	0.008014
11731532_MTPAP	-0.37815	0.002427	0.008014
11719301_GRPEL2	0.203826	0.002427	0.008014
11726672_ACCS	0.173187	0.002428	0.008018
11716282_FURIN	0.197146	0.00243	0.008024
11754383_GFOD2	0.203222	0.002434	0.008037
11724975_HRASLS5	-0.23853	0.002434	0.008037
11717184_GTPBP2	0.198752	0.002435	0.00804
11746825_FPGT	-0.24211	0.002438	0.008048
11746294_HERC2 ///	0.225977	0.002439	0.00805
11724649_VANGL1	-0.25232	0.00244	0.008053
11720273_SFT2D3	-0.17948	0.00244	0.008053
11755105_DGKZ	0.190334	0.002441	0.008057
11720077_EXOSC4	0.189965	0.002444	0.008063
11759803_PDCD6	-0.21004	0.002444	0.008063
11717100_SLC38A1	-0.17182	0.002445	0.008067
11741271_GOPC	-0.16447	0.002446	0.008069
11754271_PSMB4	-0.16512	0.002448	0.008075
11730180_KDSR	-0.2369	0.002448	0.008075
11722420_PPP3CC	-0.19602	0.002454	0.008093
11759259_C10orf111	-0.23418	0.002454	0.008095
11723203_MTMR6	0.165376	0.002455	0.008098
11752130_STAG1	0.187808	0.002456	0.008098
11739476_SBF2	0.241699	0.002456	0.008099
11718601_ATXN2	0.188661	0.002457	0.008101
11758904_RCAN3	-0.26872	0.002458	0.008105
11739036_PLP1	-0.16788	0.002458	0.008105
11744812_SCAP	-0.20842	0.00246	0.008108
11719704_CD2BP2	-0.16221	0.00246	0.008108
11717378_SRPRB	0.178051	0.00246	0.008108
11740230_PHEX	0.27613	0.002461	0.008111
11745336_DMXL1	0.348596	0.002461	0.008112
11717497_SLC25A36	0.176526	0.002463	0.008115
11759184_PLXNC1	0.354841	0.002463	0.008115
11726745_TAOK2	-0.19644	0.002464	0.00812
11734788_OLFM3	0.221692	0.002465	0.008121
11751591_RMND1	0.309722	0.002465	0.008121
11746110_LOC102724	-0.16071	0.002465	0.008122
11729629_AP3M1	0.149423	0.002466	0.008122
11717971_FAM20B	-0.17241	0.002466	0.008122
11718465_NCK2	0.187727	0.002467	0.008126
11720483_POLM	0.183488	0.002469	0.008132
11749309_PRUNE	0.198788	0.002469	0.008132

11755189_TMEM175	-0.1838	0.00247	0.008134
11757801_HLA-DPB1	-0.20163	0.00247	0.008135
11752074_GK5	0.209973	0.002472	0.00814
11751919_DIXDC1	0.261706	0.002473	0.008141
11762426_MYT1L	-0.32998	0.002475	0.008148
11726438_MALT1	0.231824	0.002476	0.008149
11764246_STT3B	-0.18298	0.002476	0.00815
11725656_MAGOHB	-0.46112	0.002476	0.00815
11748460_RHOBTB1	0.269564	0.002476	0.00815
11740749_OGG1	-0.19682	0.002477	0.008153
11760182_ZNF549	0.312199	0.002478	0.008155
11715535_GPS1	-0.17145	0.002478	0.008155
11715353_COL1A2	-0.35555	0.002478	0.008155
11746164_MEAF6	0.160341	0.00248	0.008161
11724765_FASTKD2	0.162578	0.002481	0.008161
11715578_RAC1	-0.16542	0.002481	0.008162
11754774_NPAS2	0.169219	0.002481	0.008163
11718703_INO80E	-0.18017	0.002485	0.008173
11757994_ZNF674	0.514267	0.002485	0.008173
11755141_CACNA2D4	0.343572	0.002486	0.008175
11727532_FEZ2	0.177296	0.002487	0.008178
11748290_PRPSAP2	-0.16077	0.002487	0.008178
11744621_LOC100130	0.165082	0.002488	0.008182
11724790_PKP3	-0.16966	0.002488	0.008182
11740415_DNAJC5B	-0.19691	0.002488	0.008182
11745524_CTNNBIP1	-0.24401	0.00249	0.008185
11721139_UCKL1	-0.20831	0.002491	0.008187
11758021_DDX3X	0.16675	0.002492	0.008191
11716954_CTDSP2	-0.23636	0.002493	0.008193
11760508_VRK2	0.399577	0.002496	0.008204
11721806_EMC1	0.189934	0.002497	0.008205
11721825_ARPC5L	0.178628	0.002498	0.00821
11724311_SH3BP2	-0.19955	0.002503	0.008226
11754714_EP400	-0.28342	0.002504	0.008229
11719395_EXTL2	-0.18137	0.002506	0.008234
11716937_NUPR1	-0.17911	0.002507	0.008238
11724241_TCF4	0.193647	0.002508	0.00824
11746329_MAEA	0.164323	0.002509	0.008241
11717629_KIF1BP	-0.19977	0.00251	0.008243
11720806_ING4	-0.18742	0.00251	0.008245
11748002_HPCAL1	-0.18107	0.002511	0.008246
11718906_ADCK2	-0.1567	0.002511	0.008247
11718693_TMEM60	-0.2276	0.002512	0.00825
11751590_NCOR1	0.224622	0.002513	0.008253
11739754_SLAMF8	-0.17667	0.002513	0.008253
11751884_PSME4	0.222266	0.002514	0.008253
11758573_MORC4	0.214936	0.002514	0.008254
11757077_LOC102723	-0.17743	0.002514	0.008254
11734820_APBB2	0.328354	0.002515	0.008255
11732223_ZP3	-0.16446	0.002515	0.008255
11757855_GARS	0.154265	0.002518	0.008264

11719398_RRAGC	0.174264	0.00252	0.008269
11758422_SLC35G1	0.234477	0.002523	0.008281
11723753_ZFAND2A	0.154141	0.002527	0.008292
11737782_PROSC	0.240424	0.002527	0.008292
11719847_C7orf25	0.152352	0.002531	0.008303
11739450_ROCK1	0.163367	0.002534	0.008313
11720571_JADE2	-0.16828	0.002534	0.008314
11746921_RANBP17	-0.279	0.002536	0.008318
11752736_CHID1	-0.20008	0.002538	0.008325
11759078_PLRG1	-0.20446	0.002538	0.008325
11734607_SHQ1	0.167427	0.002539	0.008327
11747410_MRRF	0.222465	0.002539	0.008327
11729939_CRYBB3	-0.18817	0.00254	0.00833
11730677_RNF150	-0.22932	0.00254	0.00833
11760837_RAET1E	0.505099	0.002541	0.00833
11749971_CYB561	0.17356	0.002541	0.00833
11730340_MSX2	0.279252	0.002545	0.008342
11751748_CHN2 ///	-0.29355	0.002545	0.008344
11719402_RAP2A	-0.21189	0.002546	0.008344
11720527_LTBP2	0.458632	0.002548	0.008352
11740038_ANKRD36 /	0.167838	0.002549	0.008356
11762127_CD33	-0.25503	0.002553	0.008366
11717032_KIAA0141	-0.20711	0.002553	0.008366
11737164_TRPC3	-0.27745	0.002553	0.008366
11760678_PPIL2	-0.49279	0.002554	0.008367
11764090_DNAJC5	0.21003	0.002554	0.008367
11724641_ARHGAP44	-0.20713	0.002555	0.00837
11716883_RAP1B	0.169652	0.002555	0.00837
11730582_USP43	-0.42326	0.002557	0.008375
11735369_UNC5C	0.276215	0.002558	0.008377
11745626_PCDHGA2	-0.3157	0.002558	0.008377
11737130_PGAM5	-0.28712	0.00256	0.008382
11725047_ANP32E	-0.15348	0.00256	0.008382
11724359_BTRC	0.253486	0.00256	0.008383
11741625_SLC22A23	-0.23252	0.002561	0.008385
11759808_PLEKHS1	0.352517	0.002562	0.00839
11746885_RAD52	0.237397	0.002564	0.008394
11759463_ADAMTS20	-0.26133	0.002565	0.008397
11746895_BIN1	0.156937	0.002566	0.008399
11723995_GLCCI1	-0.30585	0.002568	0.008405
11720055_METAP1	-0.14795	0.002571	0.008416
11758544_RDH10	-0.27314	0.002574	0.008426
11749391_QPRT	-0.19082	0.002575	0.008427
11754961_RNF220	-0.16284	0.002576	0.008428
11723812_STX17	0.472219	0.002577	0.008433
11752305_PARPBP	-0.28033	0.002579	0.008437
11740799_PALM2	0.427155	0.002582	0.008446
11756309_MXD3 ///	0.205595	0.002582	0.008447
11721555_COLGALT1	-0.17071	0.002583	0.00845
11748547_TMEM39B	-0.15061	0.002591	0.008475
11742710_SRGN	0.19732	0.002592	0.008478

11715863_DDAH2	-0.32796	0.002594	0.008485
11762199_LOC100287	-0.31912	0.002596	0.008489
11727959_ING1	-0.19348	0.002597	0.008492
11726075_PDCD2	0.146772	0.002597	0.008493
11724132_GFRA1	0.572606	0.002598	0.008496
11721742_COL5A3	0.175852	0.002599	0.008499
11744427_SYAP1	0.17935	0.0026	0.008499
11745307_NFKBID	-0.26631	0.002601	0.008503
11746769_RWDD4	-0.22028	0.002604	0.008511
11721809_IST1	0.164899	0.002604	0.008511
11749879_PGAP2	-0.24895	0.002604	0.008511
11715642_TTC3	0.160578	0.002605	0.008512
11719080_CDC5L	-0.17343	0.002605	0.008513
11731045_ERLIN2	0.193786	0.002605	0.008513
11745916_ZNF549	0.334068	0.002606	0.008516
11754349_SRSF5	-0.1635	0.002607	0.008519
11762890_SUN1	-0.1538	0.002608	0.00852
11752702_MAP2K7	-0.20611	0.002611	0.00853
11732159_YIF1A	0.190772	0.002614	0.00854
11716143_SIGMAR1	-0.17667	0.002615	0.008541
11750065_IFT52	0.178505	0.002618	0.00855
11716903_TGOLN2	0.186739	0.002618	0.008552
11725965_CAV2	0.169396	0.002621	0.008561
11729334_CAV2	0.169396	0.002621	0.008561
11735135_TLL2	-0.17744	0.002625	0.008573
11757420_ZFP36L2	0.186958	0.002626	0.008573
11724590_TCP10L	0.212287	0.002626	0.008574
11722842_ENAH	-0.15406	0.002627	0.008576
11721579_DIO2	0.305816	0.002627	0.008577
11720223_GFPT1	0.153546	0.002629	0.008582
11757586_SLC27A5	-0.17219	0.002629	0.008583
11729406_IFNGR1	0.190335	0.00263	0.008585
11749065_MCU	0.247082	0.002634	0.008595
11755830_MYO1G	-0.21093	0.002635	0.008598
11722543_TRPC1	-0.23975	0.002635	0.008598
11724758_GPBP1L1	0.154868	0.002636	0.008601
11728536_MCC	0.367336	0.002639	0.008609
11718519_KCMF1	-0.34599	0.002639	0.008611
11723575_MAPKAP1	-0.28257	0.00264	0.008614
11739221_CACUL1	-0.26212	0.002641	0.008614
11735597_PTCH1	-0.19741	0.002642	0.008618
11743341_SMARCA2	0.178227	0.002642	0.008619
11754284_CYB561	0.171947	0.002643	0.008619
11720554_ARHGEF18	0.17354	0.002643	0.008621
11734061_PSTK	0.178501	0.002644	0.008622
11721692_CYB561	0.177862	0.002645	0.008625
11748037_CS	-0.15655	0.002645	0.008625
11754289_LMAN2L	0.20184	0.002646	0.008626
11733386_TMEM182	-0.3029	0.002648	0.008631
11757098_GAS6-AS1	-0.16285	0.002648	0.008631
11733887_FBXO42	0.177492	0.00265	0.008638

11717703_KIFC3	0.164452	0.002652	0.008643
11736121_ZFAND5	0.187133	0.002653	0.008646
11735094_LEP	-0.28653	0.002654	0.008648
11755217_ZNF142	0.398168	0.002656	0.008656
11735119_NBR1	-0.16973	0.002656	0.008656
11742316_RHCE	-0.34973	0.002657	0.008658
11748454_PUDP	-0.22772	0.002658	0.008659
11756677_CYB5D2	0.230311	0.002658	0.00866
11725072_JADE1	-0.27144	0.002658	0.00866
11724159_ECSCR	0.151382	0.002659	0.008662
11727741_SYNDIG1L	-0.25716	0.00266	0.008665
11739350_AMOT	0.538894	0.002662	0.008671
11719393_NR1H3	-0.21382	0.002662	0.008671
11755795_PRDM2	0.338878	0.002663	0.008673
11726675_ASCL1	-0.16937	0.002667	0.008684
11751794_GLI2	0.183597	0.002667	0.008684
11717187_MEF2D	0.222653	0.002667	0.008684
11748588_ORC3	-0.19841	0.002668	0.008686
11748054_ESPL1	-0.25384	0.002668	0.008686
11757858_EIF2S3	0.20481	0.002669	0.008687
11751928_EIF4G3	0.154333	0.00267	0.008689
11742893_LAMTOR5	-0.17521	0.00267	0.008689
11740114_CGN	0.452078	0.002673	0.008699
11743863_ATP2B1	0.280798	0.002673	0.0087
11740937_LIAS	-0.54804	0.002676	0.008709
11753706_CALM2 ///	0.186303	0.002677	0.008712
11732979_IFNA21	-0.41194	0.002682	0.008728
11742678_PTBP1	-0.15936	0.002683	0.008731
11726354_ENPP1	-0.22611	0.002685	0.008735
11723063_GCHFR	-0.23786	0.002686	0.008739
11735896_DEFB106A	-0.21045	0.002687	0.00874
11755083_APBA3	0.169033	0.00269	0.00875
11753560_RAN	-0.15231	0.002691	0.008751
11751891_ASXL2	0.21771	0.002692	0.008756
11759859_BACH1 ///	0.650177	0.002692	0.008756
11728331_KCTD15	-0.23879	0.002694	0.008759
11752847_NFIC	-0.19479	0.002694	0.008759
11723819_PM20D2	0.434423	0.002694	0.008759
11735097_MLX	0.175899	0.002695	0.008764
11763617_COL12A1	0.370881	0.0027	0.008777
11747902_EML1	0.179607	0.002704	0.008789
11752403_STEAP3	-0.19533	0.002704	0.008789
11721585_TMCC1	0.380233	0.002708	0.008801
11736797_RUNX1T1	-0.27417	0.002708	0.008802
11728632_AP4E1	0.365084	0.00271	0.008807
11740533_KRTAP5-9	-0.29414	0.00271	0.008807
11719328_ALAS1	-0.1616	0.002712	0.008814
11757878_UBE2E1	0.156122	0.002712	0.008814
11744538_CALHM2	-0.18322	0.002713	0.008814
11763143_LAMTOR5P1	-0.19276	0.002713	0.008814
11743186_CFAP97	-0.19283	0.002713	0.008815

11758133_COL4A3BP	-0.21852	0.002716	0.008821
11731022_ATXN7	0.178371	0.002716	0.008822
11750013_CSNK1D	0.271575	0.002717	0.008826
11722952_SLC27A1	-0.19619	0.002717	0.008826
11728181_CHIC1	0.471394	0.002718	0.008827
11715890_ATP5I	-0.15335	0.002721	0.008835
11716075_HSBP1	0.18714	0.002723	0.008843
11764020_PPTC7	0.191205	0.002724	0.008844
11745432_CDK12	0.228812	0.002726	0.00885
11745794_OXR1	0.210438	0.002726	0.00885
11724749_CEND1	-0.21	0.002727	0.008851
11750935_BMP6	-0.26799	0.002727	0.008851
11719094_CYB5B	-0.21738	0.002727	0.008852
11723914_CACTIN	-0.16516	0.002728	0.008855
11723955_CLINT1	0.156263	0.002729	0.008857
11754980_SMARCD3	0.19142	0.002729	0.008857
11747620_FBXO22	0.182711	0.002731	0.008862
11736309_CSNK1A1	0.298402	0.002731	0.008862
11737662_TET2	-0.2152	0.002734	0.00887
11731070_NIT1	0.162962	0.002736	0.008876
11747433_C11orf54	0.188034	0.002737	0.00888
11756405_GCSH ///	-0.14771	0.002738	0.008881
11753674_PFN2	-0.18985	0.002739	0.008883
11750082_ERCC2	0.210852	0.00274	0.008888
11738153_RBM24	0.587585	0.002741	0.008888
11715805_PPP2CA	0.155214	0.002741	0.008891
11739151_TTYH3	-0.14879	0.002742	0.008892
11715714_VOPP1	-0.23515	0.002745	0.0089
11754204_PEX11A	0.181609	0.002746	0.008903
11748550_COQ6	0.2526	0.002747	0.008905
11750897_THRA	-0.34708	0.00275	0.008914
11732282_SLC30A5	-0.18224	0.00275	0.008914
11722813_PABPC4	0.156695	0.00275	0.008916
11728164_EPPIN	-0.34414	0.002754	0.008928
11716535_PPT1	0.158894	0.00276	0.008945
11754760_GRAMD1A	0.151533	0.002762	0.008952
11727889_HYAL3	0.185379	0.002763	0.008955
11717288_PI4KA	0.145211	0.002764	0.008958
11747953_PYROXD1	0.206761	0.002766	0.008961
11732256_SHB	0.205743	0.002769	0.008971
11756534_TMEM108	-0.2029	0.002769	0.008972
11716270_SLIRP	-0.16451	0.00277	0.008973
11755197_PGM3	0.234693	0.00277	0.008973
11748219_UGT1A1 //	-0.16171	0.002772	0.008978
11752108_PPP6R3	0.184602	0.002773	0.008982
11750189_CD44	0.173091	0.002778	0.008997
11735400_BNC1	0.180407	0.00278	0.009002
11745678_KIF6	0.251317	0.00278	0.009003
11758801_TM4SF1	-0.17052	0.002781	0.009005
11748450_METTL17	-0.23083	0.002781	0.009005
11740385_METTL20	-0.24744	0.002781	0.009005

11736173_KIAA0355	-0.31542	0.002783	0.009008
11754624_CYB5A	-0.31808	0.002783	0.009008
11748948_BIVM-ERCC	0.342681	0.002783	0.009008
11757213_FLJ34503	0.265094	0.002784	0.009012
11755948_SH3RF1	0.326983	0.002785	0.009015
11721906_PSMA4	-0.15825	0.002786	0.009016
11757999_RAB38	-0.15747	0.002786	0.009016
11728969_AASDHPPT	0.169056	0.002787	0.009019
11733455_SP4	-0.30113	0.002788	0.00902
11717075_ZFAND6	0.200984	0.002789	0.009025
11719240_PANX1	0.169667	0.002791	0.009029
11716001_FAF2	0.177701	0.002794	0.009038
11747285_TOM1L1	0.173027	0.002794	0.009038
11723692_CD302 ///	-0.23302	0.002795	0.009042
11754290_LMAN2L	0.197423	0.002798	0.00905
11719205_ANKLE2	0.205252	0.002799	0.009052
11749958_DSCR3	0.183021	0.002799	0.009053
11747598_CLPTM1	0.218034	0.002802	0.00906
11758804_MON1B	-0.24028	0.002806	0.009073
11731833_AK4	0.179663	0.002806	0.009073
11720547_PDSS1	0.163781	0.002806	0.009074
11743129_SORL1	0.532435	0.002807	0.009075
11720138_ALKBH2	-0.1802	0.002808	0.009077
11749223_PTRF	-0.26507	0.00281	0.009083
11749102_SECISBP2	0.242205	0.00281	0.009084
11720591_PHF21A	0.238107	0.002812	0.00909
11758982_ST6GALNAC	0.536233	0.002813	0.009093
11736156_FAM168A	0.305316	0.002816	0.009099
11742988_EZR	0.168719	0.002816	0.0091
11736673_SIRT7	0.173036	0.002817	0.009104
11746285_FDXACB1	-0.18255	0.002819	0.00911
11761361_PPARGC1B	-0.21205	0.002822	0.009116
11763330_VPS36	0.407637	0.002824	0.009122
11743606_ACSL3	-0.15982	0.002824	0.009122
11722030_UBE3C	0.18721	0.002826	0.009129
11740855_KRIT1	0.14534	0.002828	0.009136
11734815_GSG1	0.368597	0.002829	0.009137
11719681_TNFRSF10B	0.163152	0.002831	0.009142
11744700_TDP1	-0.21401	0.002831	0.009144
11728073_ITPRIP	0.175265	0.002832	0.009146
11718692_SBNO2	0.228808	0.002834	0.009149
11736766_C14orf28	0.449288	0.002835	0.009152
11748859_TFEC	0.26718	0.002835	0.009152
11736590_RAMP3	-0.19823	0.002837	0.009157
11759546_CXXC5	-0.19264	0.002837	0.009158
11757001_SEC14L2	0.209861	0.002838	0.00916
11758539_DOCK10	0.170144	0.002842	0.009173
11759236_ZNF284	0.444885	0.002842	0.009173
11715415_COL3A1	0.299364	0.002844	0.009179
11727450_MORN4	0.190606	0.002845	0.00918
11748846_NIT1	0.17162	0.002845	0.009181

11752514_SLC6A9	-0.17442	0.002845	0.009181
11717586_SCAP	-0.19857	0.002852	0.009202
11741294_DDI2	0.361593	0.002856	0.009215
11737751_TMEM254	-0.26849	0.002857	0.009215
11732616_FEV	-0.27116	0.002857	0.009217
11764239_TBRG1	0.248341	0.002858	0.009217
11719750_POLE	-0.17925	0.002858	0.009217
11728711_NFATC2IP	0.201997	0.002858	0.009217
11717973_FAM219B	0.233015	0.002858	0.009217
11723313_PXMP2	-0.18938	0.00286	0.009223
11723979_PHKA2	-0.1761	0.002861	0.009226
11722773_ZNF473	0.193313	0.002861	0.009226
11752718_GDI1	0.166338	0.002862	0.009226
11729352_MYF6	-0.2372	0.002863	0.009231
11723651_CIAO1	0.229815	0.002864	0.009234
11731302_ITPRIPL1	-0.2024	0.002866	0.009238
11733320_HRAS	0.193088	0.002866	0.009238
11752705_FAM225A /	-0.22727	0.002866	0.009238
11721333_PLOD2	0.165854	0.002867	0.009238
11752267_RPS6KA4	-0.17237	0.002867	0.009239
11739936_HMGXB4	-0.17081	0.002867	0.009239
11720894_SOS1	0.184085	0.002869	0.009245
11757860_QKI	-0.19523	0.002871	0.009249
11720063_GLIPR2	-0.3141	0.002871	0.009251
11761037_ARL16	0.205139	0.002873	0.009255
11747170_METTL2B	0.18489	0.002874	0.009259
11756227_PEX2	-0.21505	0.002879	0.009272
11729317_UACA	0.227913	0.002879	0.009274
11762413_CDCA3	-0.32019	0.002881	0.009277
11739122_EPB41	0.287919	0.002883	0.009285
11723759_ZFR	0.229313	0.002884	0.009286
11741883_MRPL39	-0.18744	0.002884	0.009287
11739249_FGFR2	-0.17181	0.002885	0.009288
11724548_RSBN1	0.149193	0.002886	0.009292
11747149_SHOX2	-0.53623	0.002886	0.009292
11718186_UHRF2	0.161347	0.002886	0.009292
11756574_RNF167	-0.15647	0.002888	0.009297
11752624_PDE4A	-0.18239	0.002891	0.009304
11732166_LMF1	-0.26864	0.002893	0.00931
11740439_FUT6	-0.21611	0.002895	0.009316
11720787_DYNC1LI1	-0.16338	0.002895	0.009316
11746402_ZFAND5	0.220855	0.002895	0.009316
11745473_NEK11	0.280529	0.002896	0.00932
11720332_SLC5A6	-0.26245	0.002897	0.009321
11761821_NEK2	-0.33126	0.002897	0.009321
11755782_C20orf194	0.239012	0.002898	0.009324
11733041_ALAS1	-0.17553	0.0029	0.009328
11716623_XPO1	-0.15008	0.002901	0.00933
11715820_BUD31	0.167556	0.002901	0.009331
11743120_EIF4A1 //	-0.14329	0.002902	0.009334
11763667_FER	-0.22338	0.002902	0.009334

11725378_GSTO1	0.143568	0.002904	0.009337
11726555_SALL1	-0.23798	0.002904	0.009337
11736369_MBOAT1	-0.18408	0.002905	0.009339
11756430_GBGT1	-0.19443	0.002905	0.009341
11744434_PARP9	0.193081	0.002906	0.009343
11737562_RAD51B	0.228107	0.002906	0.009343
11741773_TCEA2	-0.21336	0.002906	0.009343
11734424_GPR26	-0.26107	0.002907	0.009343
11732907_C8orf74	-0.17883	0.002908	0.009346
11723681_USP34	0.142865	0.002911	0.009355
11755140_CUL9	-0.22621	0.002911	0.009355
11744494_JTB	0.151084	0.002914	0.009365
11748517_ACP2	-0.1508	0.002916	0.009369
11746343_RANBP3	-0.26153	0.002916	0.00937
11736570_SLC39A14	-0.16048	0.002916	0.00937
11725953_RNF10	0.202077	0.002917	0.00937
11740974_CYFIP1	0.16799	0.002922	0.009386
11720056_METAP1	-0.19113	0.002922	0.009387
11757402_MED13	-0.15521	0.002923	0.009388
11746583_ZNF558	0.329884	0.002925	0.009395
11722283_CA11	-0.16314	0.002926	0.009396
11715970_EPRS	-0.1479	0.00293	0.009408
11749703_SIRT1	-0.15702	0.002932	0.009415
11722715_STK35	-0.18971	0.002933	0.009417
11744239_CCDC159	0.26977	0.002933	0.009417
11723586_GRB14	-0.33441	0.002934	0.009418
11729905_LRCH2	-0.32482	0.002934	0.009419
11755853_IL22RA1	-0.31482	0.002936	0.009425
11738325_OR14I1	-0.19244	0.002938	0.00943
11746668_ALDH9A1	0.160755	0.00294	0.009436
11736765_C14orf28	0.316879	0.00294	0.009437
11754086_VAPA	0.213677	0.002941	0.009437
11751598_DPP7	-0.35103	0.002941	0.009438
11733370_PSME1	0.170622	0.002942	0.009439
11715744_ATP5J	-0.16862	0.002943	0.009442
11746450_MEIS2	-0.17054	0.002943	0.009443
11728602_CIB2	-0.21066	0.002944	0.009445
11746206_RHOF	0.175343	0.00295	0.009465
11729133_STK24	-0.18613	0.002953	0.009471
11716674_ACIN1	-0.18155	0.002953	0.009471
11729247_SIRT5	-0.28913	0.002954	0.009474
11724868_IPO8	-0.16948	0.002956	0.00948
11758080_PPP1R15B	0.147513	0.002958	0.009487
11757537_RAD23A	-0.1602	0.002959	0.009489
11756168_RAB13	-0.20563	0.00296	0.00949
11746231_ZMYM6	0.182377	0.00296	0.009491
11739015_ERMN	-0.28023	0.00296	0.009491
11736842_KLRAP1	-0.27071	0.002961	0.009491
11723548_PEX1	0.165001	0.002962	0.009496
11742913_GLOD5	0.282289	0.002964	0.0095
11747382_SERGEF	-0.17005	0.002966	0.009508

11718540_TMEM243	0.159567	0.002971	0.009521
11738305_VAX1	-0.19105	0.002972	0.009525
11750526_CYP11A1	-0.27904	0.002974	0.00953
11750213_ADPRHL2	-0.22904	0.002974	0.009531
11727981_SNX21	-0.26736	0.002977	0.00954
11719853_SEPT10	-0.17433	0.00298	0.009546
11760179_WASH3P	-0.16514	0.002984	0.009559
11751398_NDRG3	0.176992	0.002984	0.009559
11757883_RNF4	0.171468	0.002985	0.009561
11717166_KDM2A	-0.22773	0.002987	0.009566
11721876_SLC7A11	-0.16435	0.002987	0.009569
11720557_TRMT11	0.159047	0.00299	0.009576
11754355_NSMCE1	-0.16948	0.00299	0.009576
11762897_TMEM80	-0.15763	0.002991	0.009576
11715429_ARF4	0.158651	0.002991	0.009576
11724277_SLC35C1	-0.21088	0.002991	0.009577
11736859_FAM89B	-0.16285	0.002993	0.009582
11749330_SIGMAR1	-0.22373	0.002998	0.009599
11759797_ANXA1	0.222749	0.002999	0.009599
11758734_EIF5A	-0.16266	0.003	0.009601
11724024_TRIM13	-0.17955	0.003	0.009602
11724067_TNS3	-0.22504	0.003	0.009602
11758668_ARL17A //	0.230864	0.003	0.009602
11744284_MRPS12	-0.18247	0.003002	0.009606
11721016_USP5	-0.16793	0.003004	0.009611
11739592_MINA	-0.28	0.003005	0.009614
11759085_KIAA1715	-0.23418	0.003005	0.009614
11753384_C19orf54	-0.3187	0.003005	0.009614
11725611_TMEM200B	-0.30017	0.003005	0.009614
11723103_GNB4	-0.18517	0.003005	0.009614
11720575_CRAT	-0.1582	0.00301	0.009627
11716149_PMEPA1	0.148991	0.003013	0.009636
11724196_STC1	0.244635	0.003017	0.009648
11763321_CSNK2A2	0.260814	0.003017	0.009648
11721936_CHN2 ///	-0.29896	0.003017	0.00965
11745601_ERAP2	0.258808	0.003018	0.009651
11763274_SIPA1L1	-0.29303	0.003019	0.009654
11730555_TRIM34 //	0.262141	0.003023	0.009666
11740961_CD33	0.208281	0.003025	0.009672
11719861_SLC2A4RG	-0.16577	0.003026	0.009673
11744500_ASXL1	-0.22065	0.003027	0.009675
11757471_TBC1D15	-0.31102	0.003028	0.009679
11748038_CS	-0.14965	0.00303	0.009683
11759025_PPP2R5E	-0.17469	0.003034	0.009698
11754827_FBXO17	-0.26039	0.003037	0.009706
11733882_TTF1	-0.15069	0.003037	0.009706
11720260_TRIM33	0.151529	0.003042	0.00972
11718664_SLC35B2	-0.33344	0.003043	0.009724
11725624_ACE2	0.369992	0.003044	0.009724
11717796_ZDHHC3	0.175202	0.003044	0.009724
11747255_MEIS1	0.21443	0.003046	0.009729

11736098_CDK20	0.257966	0.003047	0.009735
11742776_GPR161	-0.18683	0.00305	0.009742
11716567_PRRC2C	0.147675	0.003051	0.009745
11760195_ANKS1B	-0.34042	0.003051	0.009745
11752312_ISLR2	-0.20832	0.003053	0.009749
11730173_C4orf3	0.185246	0.003055	0.009755
11723543_ESRP1	0.251347	0.003055	0.009756
11724489_AKR1B10 /	0.51681	0.003056	0.009757
11737055_ZNF678	0.799373	0.003056	0.009757
11716581_G3BP2	0.154593	0.003056	0.009757
11760163_WSB1	-0.38773	0.003057	0.009757
11743664_COG5	0.172767	0.003057	0.009759
11727621_LIPH	0.414433	0.003059	0.009765
11750069_ALKBH2	-0.19876	0.003061	0.009769
11750187_AGPAT1	-0.18921	0.003062	0.009772
11743508_ARHGAP26	0.354885	0.003064	0.009779
11746162_FAM49B	0.149647	0.003067	0.009785
11758035_MAPKBP1	0.218348	0.003068	0.009788
11720336_PHC2	-0.19769	0.003068	0.009789
11735478_CHST6	-0.29661	0.003069	0.009792
11753511_GTF2F2	0.146261	0.003069	0.009792
11753975_TRA2B	-0.14966	0.003071	0.009797
11764111_ELMOD2	-0.17548	0.003072	0.009797
11734917_METTL4	-0.33343	0.003072	0.009797
11719170_IPO4	0.159121	0.003073	0.009802
11733573_DMRT3	-0.25073	0.003073	0.009802
11730845_PIK3R3	0.388006	0.003075	0.009806
11746606_ANKRD36 /	-0.20098	0.003077	0.009812
11747687_SUPT6H	0.231585	0.00308	0.009821
11717044_TCERG1	0.193836	0.003081	0.009824
11724994_FAM101B	-0.35805	0.003084	0.00983
11737900_TOX2	-0.15263	0.003084	0.009833
11751678_GULP1	0.154154	0.003085	0.009833
11760239_MCM9	-0.17598	0.003085	0.009835
11749916_AGBL2	0.236005	0.003086	0.009837
11717354_PCBD1	-0.1737	0.003087	0.009837
11742935_RTN4R	-0.39201	0.003087	0.009837
11726698_ZBTB7A	0.281562	0.003089	0.009842
11756236_FBLN1	-0.25546	0.00309	0.009845
11746775_CGREF1	-0.42574	0.003091	0.009849
11749936_HDAC8	-0.2766	0.003093	0.009853
11734347_PRPS1L1	-0.38549	0.003093	0.009853
11723285_ITGBL1	0.327288	0.003093	0.009853
11734571_DCTN4	0.152526	0.003093	0.009853
11753779_ENSA	-0.20085	0.003093	0.009853
11757613_EIF3K	-0.15116	0.003094	0.009854
11739665_RUBCN	-0.18854	0.003094	0.009855
11744929_JARID2	0.193508	0.003095	0.009855
11718187_BNIP1	-0.14973	0.003098	0.009866
11721811_SF3A2	-0.18228	0.003099	0.009866
11723801_PYROXD1	-0.17212	0.003099	0.009867

11755775_TMEM181	-0.19563	0.0031	0.009869
11728955_KITLG	0.579313	0.003102	0.009874
11745928_TMEM68	-0.23531	0.003102	0.009875
11749635_FLII	0.20313	0.003102	0.009875
11755443_MAOA	0.205102	0.003102	0.009875
11738206_PKD2L2	0.277312	0.003104	0.009879
11718460_SLC39A11	0.225641	0.003108	0.009889
11720733_INPPL1	-0.18971	0.003109	0.009892
11726497_LIF	0.364783	0.003109	0.009892
11754029_VAPA	0.214533	0.00311	0.009895
11718468_NECAB3	-0.15416	0.003111	0.009897
11748661_KCNJ11	-0.32418	0.003111	0.009897
11755253_USF3	-0.19841	0.003112	0.009898
11738970_PYDC2	-0.17098	0.003112	0.009898
11741352_ZNF334	0.159505	0.003113	0.009902
11721833_CHURC1	0.310006	0.003113	0.009902
11720454_YME1L1	0.157328	0.003114	0.009904
11723870_ASB7	-0.18866	0.003114	0.009904
200074_PMRPL14 ///	0.148589	0.003115	0.009905
11757980_EP400	-0.17234	0.003115	0.009905
11722705_B3GNT5	0.180878	0.003118	0.009915
11723190_ADAM19	-0.18029	0.003122	0.009925
11722313_RAPGEF6	0.167233	0.003124	0.009933
11721069_DGKD	0.160922	0.003126	0.009938
11729272_ASB1	-0.17012	0.003128	0.009942
11718273_EIF3L	0.150707	0.003129	0.009945
11722441_SEMA4F	-0.19549	0.00313	0.009947
11736562_ZNF518A	0.191773	0.003133	0.009956
11746632_MAP3K11	-0.19492	0.003135	0.009962
11733576_IDH3B	-0.22515	0.003136	0.009966
11744579_CDADC1	-0.24534	0.003137	0.009967
11746001_ZNF252P	0.219009	0.003137	0.009967
11726696_ZBTB7A	0.200297	0.003138	0.009969
11742049_DMTF1	0.207482	0.003139	0.009971
11737433_WTH3DI	-0.28795	0.003139	0.009972
11726400_ALG9	0.181742	0.00314	0.009973
11747116_JMY	0.271008	0.00314	0.009973
11727402_ERGIC2	0.144472	0.00314	0.009973
11721565_OSTC	0.215944	0.003141	0.009974
11722761_GATA2	-0.20327	0.003141	0.009974
11741119_ANKEF1	0.320502	0.003141	0.009974
11740526_TSPAN4	0.197796	0.003141	0.009974
11758176_ATP10D	0.190193	0.003142	0.009975
11736691_CCDC89	0.188949	0.003145	0.009986
11720609_PHB	-0.15751	0.003147	0.00999
11728764_PVRL4	0.676183	0.003149	0.009996
11753660_UBE2V1	0.183278	0.003149	0.009996
11756856_HELQ	0.295909	0.003151	0.01
11760942_STAG3L1 /	0.225871	0.003151	0.01
11757289_CLTA	0.152537	0.003154	0.010008
11732079_POM121L12	-0.18702	0.003159	0.010025

11733027_CLCA2	0.242948	0.003159	0.010025
11744770_HOXA11	-0.2233	0.00316	0.010026
11719014_FZD4	-0.2379	0.003161	0.010029
11716923_PURB	0.190557	0.003161	0.010029
11716188_SEC13	-0.17432	0.003163	0.010035
11733109_TLK1	0.245131	0.003164	0.010037
11729136_STK24	-0.51261	0.003165	0.010039
11751999_UBAP2	0.150275	0.003165	0.010039
11751234_SLC26A4	0.48931	0.003165	0.010039
11749210_HNRNPK	-0.2618	0.003166	0.01004
11758214_SRRM2	-0.2386	0.003167	0.010044
11753729_XRRA1	0.227297	0.003168	0.010044
11720569_NGEF	-0.17376	0.003168	0.010045
11719632_ABCC4	0.199376	0.00317	0.010049
11759721_LOC105372	-0.16335	0.00317	0.010049
11722020_CDK16	-0.23482	0.003173	0.010058
11724971_FLT1	0.2166	0.003175	0.010063
11742053_COG5	0.218767	0.003176	0.010065
11760644_C21orf58	-0.25599	0.003177	0.010069
11718930_TGIF2	0.14912	0.003178	0.010073
11722915_BMPR1A	0.195598	0.00318	0.010077
11738740_OR10G7 //	-0.28068	0.00318	0.010077
11722268_NFRKB	0.19375	0.003182	0.010082
11740706_NFRKB	0.19375	0.003182	0.010082
11733048_ABI1	0.182464	0.003184	0.010088
11739206_TTL	0.147142	0.003185	0.010089
11750240_GHITM	0.142527	0.003185	0.010091
11715726_OAZ2	0.161731	0.003186	0.010092
11728425_BOLA3	-0.18006	0.003186	0.010092
11739968_ZNF101	-0.20251	0.003187	0.010094
11723261_ZNF24	0.166892	0.003188	0.010096
11750043_MTG1	-0.14715	0.003188	0.010096
11763565_ATP10B	-0.1496	0.00319	0.010101
11742300_GEMIN2	0.172171	0.003192	0.010108
11751125_ABCC6P1	-0.37401	0.003193	0.010108
11748720_USP28	-0.23976	0.003193	0.010108
11719037_UVRAG	0.206773	0.003195	0.010114
11732144_BBS2	-0.23724	0.003197	0.010119
11720517_ZBTB4	-0.16815	0.003197	0.010119
11733369_HIST1H4I	-0.40648	0.003199	0.010127
11723919_LMAN2	-0.19045	0.003204	0.010141
11728290_SH2D1A	-0.17425	0.003206	0.010145
11761376_ZNF90	-0.46515	0.003209	0.010154
11761676_TAMM41	0.150895	0.003211	0.01016
11718835_PELP1	-0.21072	0.003212	0.010163
11756118_KMT2E	0.254851	0.003213	0.010167
11746876_TRA2A	0.205572	0.003214	0.010167
11750170_TRIM22	0.263198	0.003214	0.010169
11720685_CDR2L	-0.35191	0.003218	0.010179
11748736_PARPBP	-0.20229	0.003219	0.010182
11720632_SDHB	-0.17083	0.003221	0.010186

11741435_GRB2	-0.15151	0.003223	0.010194
11731903_CD207	-0.22684	0.003223	0.010194
11722266_ADCY1	-0.25894	0.003224	0.010197
11739716_KIAA0556	0.242771	0.003225	0.010199
11720106_SLC26A2	0.146818	0.003226	0.010201
11731636_GAB1	-0.27221	0.003227	0.010203
11763223_GAP43	-0.19618	0.003227	0.010203
11736559_MAP2	0.36247	0.003227	0.010203
11757724_BSCL2 ///	-0.17403	0.003228	0.010204
11729685_LYRM7	-0.31644	0.003229	0.010206
11731152_MTRR	0.249441	0.00323	0.010207
11720735_LRRC14	0.195715	0.003231	0.01021
11745820_PLAGL1	-0.18427	0.003232	0.010214
11756263_UBE3B	0.189922	0.003232	0.010214
11748525_ECM1	0.164291	0.003234	0.010217
11745136_AAK1	0.330005	0.003234	0.010217
11743334_MRPL35	-0.17802	0.003234	0.010217
11718431_PPP6R1	-0.28626	0.003237	0.010225
11749697_NDUFS1	-0.15531	0.00324	0.010233
11745876_SEC11A	-0.1585	0.00324	0.010233
11750642_ABCB5	0.203899	0.00324	0.010234
11748200_FANCL	-0.15344	0.003241	0.010237
11743275_COPB1	0.159855	0.003242	0.01024
11716931_DGCR2	-0.23925	0.003243	0.01024
11727429_SMIM8	-0.36599	0.003244	0.010244
11758836_MED4	-0.25156	0.003249	0.010259
11761237_TRIT1	-0.22394	0.003249	0.010259
11723230_RNF138	-0.28142	0.00325	0.01026
11723110_PLEKHB2	0.157354	0.00325	0.01026
11745905_PML	-0.30323	0.003251	0.010262
11734993_SKI	0.187522	0.003253	0.010267
11739147_MKNK2	-0.24557	0.003253	0.010267
11748514_SLC35B2	-0.16135	0.003253	0.010267
11730547_PMVK	-0.20868	0.003255	0.010274
11728517_NSUN5P1	-0.22776	0.003256	0.010276
11721589_TRAF1	0.373191	0.003259	0.010284
11747321_TRIM26	0.190833	0.003259	0.010284
11748276_CANT1	-0.16945	0.00326	0.010284
11732357_ZNRF1	0.160544	0.00326	0.010285
11715145_KRTAP20-2	-0.29195	0.003261	0.010288
11741605_CHD3	0.191259	0.003262	0.01029
11725133_GCOM1 ///	-0.15702	0.003264	0.010295
11737191_ZGRF1	-0.3037	0.003267	0.010303
11736275_CEL	0.327594	0.003267	0.010305
11717733_ZCCHC24	-0.188	0.003269	0.01031
11745839_C9orf3	0.160052	0.00327	0.010311
11744170_NAT14	-0.15188	0.003272	0.010317
11717182_PDS5A	-0.17369	0.003274	0.010321
11716714_ARHGAP17	0.169959	0.003274	0.010321
11718356_ERP44	-0.229	0.003274	0.010321
11725652_EMCN	0.305703	0.003278	0.010334

11747370_CDC42SE1	0.303175	0.003279	0.010334
11755803_FAAP20	-0.16845	0.003279	0.010334
11744786_OGFR	-0.158	0.00328	0.010337
11747291_UTP6	0.176585	0.003283	0.010347
11743889_ZNF10	0.252508	0.003285	0.010351
11725243_SPAST	-0.17649	0.003285	0.010353
11720169_MRPS16	-0.23787	0.003286	0.010353
11732151_DCAF11	-0.25267	0.003287	0.010358
11735640_DNAH14	0.188025	0.003291	0.010368
11731927_CST8	-0.16608	0.003291	0.010369
11744207_IDH3B	-0.21443	0.003291	0.010369
11730534_COL13A1	0.145538	0.003292	0.010369
11725071_JADE1	-0.17105	0.003292	0.010369
11763552_FKBP9	-0.31459	0.003294	0.010376
11755011_ZNF106	0.195187	0.003295	0.010379
11743861_NIPSNAP3A	-0.14408	0.003297	0.010381
11761438_NDUFS3	0.156436	0.003297	0.010381
11761409_LOC105376	-0.23652	0.003298	0.010384
11730885_FAM63B	0.157242	0.003298	0.010386
11742544_OR5AS1	-0.32095	0.003299	0.010386
11757133_SNORA8 //	-0.2644	0.0033	0.010388
11736947_ZNF334	0.161003	0.003302	0.010395
11717438_TOP1	0.151094	0.003302	0.010396
11753288_ZNF148	-0.27043	0.003303	0.010397
11750477_THEM6	-0.19611	0.003304	0.010398
11738951_SPRED3	0.31433	0.003305	0.010401
11759843_ARL6	-0.21114	0.003306	0.010403
11732157_UFM1	0.37653	0.003306	0.010403
11745174_ATF6	0.212187	0.003306	0.010403
11748639_RBAK	0.198119	0.003306	0.010403
11742262_TTC26	0.265606	0.003308	0.010407
11745549_ARHGEF2	-0.16193	0.003309	0.01041
11730216_CSRP1	-0.14725	0.003309	0.01041
11733115_CA2	0.213579	0.003309	0.01041
11721524_ZNF706	-0.24949	0.00331	0.01041
11754728_MAL	-0.21051	0.003311	0.010413
11752854_PIGA	0.18459	0.003312	0.010416
11737403_TMEM89	-0.14088	0.003313	0.010418
11717746_ 6-Mar	0.141445	0.003314	0.01042
11736801_PLAG1	0.176147	0.003314	0.01042
11735524_GDPGP1	-0.19288	0.003316	0.010427
11724136_ZBTB4	-0.16059	0.003318	0.010431
11730590_KCTD21	0.224321	0.003321	0.010442
11739103_TMEM33	0.180602	0.003323	0.010447
11737287_GALR1	-0.18435	0.003325	0.01045
11727637_PRR14L	0.179828	0.003327	0.010458
11760264_POLR2J2 /	-0.1663	0.003328	0.010459
11740274_TMEM242	-0.19099	0.00333	0.010466
11728760_SSBP1	-0.16493	0.003331	0.010467
11731461_CPM	0.222683	0.003331	0.010468
11729670_BDH1	-0.28793	0.003335	0.010479

11719755_COA6	-0.18116	0.003337	0.010485
11758015_HPRT1	-0.14508	0.00334	0.010494
11759981_TSC1	0.156755	0.003341	0.010496
11742159_OR2M4	-0.18722	0.003341	0.010496
11741489_EFCAB2	-0.26506	0.003345	0.010506
11733185_OSMR	0.249841	0.003346	0.010509
11733662_KCNH2	-0.2822	0.003347	0.010513
11719968_HTT /// M	-0.29424	0.003348	0.010514
11723060_RILPL2	-0.20366	0.003349	0.010516
11740972_MAPRE2	-0.24439	0.003349	0.010517
11749472_CALM1 ///	-0.18887	0.003351	0.010523
11737748_GMFB	0.155416	0.003352	0.010525
11724673_BCKDHB	0.298713	0.003357	0.010539
11743870_TENM4	0.265409	0.003358	0.010541
11720092_RPUSD1	-0.16933	0.003358	0.010541
11723008_CCPG1	0.208459	0.003358	0.010541
11716801_SETD5	0.161781	0.003361	0.010549
11733283_KRBOX4	0.300834	0.003361	0.010549
11740344_GNAT1	-0.22536	0.003362	0.010551
11722793_ZNHIT3	-0.16741	0.003365	0.010559
11741446_MED7	-0.2282	0.003366	0.010563
11727997_MYEF2	0.248234	0.003369	0.010571
11744257_TUBB4A	-0.47321	0.00337	0.010575
11750133_TNIP1	0.156783	0.003372	0.01058
11725735_NFYA	-0.26472	0.003373	0.01058
11760367_DLGAP1	-0.2241	0.003373	0.01058
11742612_OR10G7 //	-0.27841	0.003373	0.010581
11717474_CHGB	0.256161	0.003378	0.010595
11741330_ANAPC11	-0.18988	0.00338	0.0106
11759829_C1orf43	-0.34945	0.003382	0.010605
11761070_MRPL20	-0.20336	0.003384	0.010611
11755163_FANCL	-0.1503	0.003384	0.010612
11746166_R3HDM2	-0.15399	0.003386	0.010616
11717126_SH3PXD2A	-0.1508	0.003386	0.010617
11747376_CCPG1	0.235156	0.003386	0.010617
11761121_PIGL	-0.21736	0.003389	0.010624
11763484_LRP10	0.300688	0.003389	0.010624
11760155_FUBP1	0.208618	0.00339	0.010626
11728182_CHIC1	0.333876	0.00339	0.010626
11744358_TACC1	0.154181	0.003393	0.010635
11760142_CDK1	-0.525	0.003395	0.01064
11718407_TMBIM6	0.177004	0.003396	0.010641
11749234_NAP1L1	-0.15112	0.003398	0.010646
11717544_POLR2A	-0.15336	0.003398	0.010646
11738911_TAS2R43 /	0.192706	0.003399	0.010648
11727432_NUTF2	-0.15075	0.003401	0.010655
11715713_VOPP1	-0.25485	0.003402	0.010659
11747698_TCF4	0.268751	0.003404	0.010663
11762339_AKR7A2	-0.28188	0.003406	0.010668
11726633_TRIM8	0.198874	0.003407	0.010673
11747252_GTF2F1	0.163321	0.00341	0.01068

11720943_RGS16	-0.22527	0.00341	0.01068
11758580_TNRC6B	0.164018	0.003412	0.010683
11719128_LMF2	-0.20618	0.003412	0.010684
11753251_JTB	0.160422	0.003415	0.010692
11719650_ARHGEF10	0.177757	0.003415	0.010692
11749480_LONP1	-0.18099	0.003415	0.010692
11745632_SEMA3D	0.357379	0.003416	0.010693
11745828_BNIP3L	0.157403	0.003419	0.010701
11726824_ZNF565	-0.2385	0.003419	0.010701
11735786_BARX2	-0.21719	0.00342	0.010704
11715950_KCTD12	-0.20346	0.00342	0.010705
11736417_CCDC82	0.179079	0.003421	0.010706
11764061_NDUFB3	-0.1607	0.003423	0.01071
11744746_TNIP1	0.184164	0.003424	0.010713
11754250_WHAMMP3	0.299198	0.003425	0.010717
11718064_S100A4	0.140573	0.003427	0.010723
11740360_FOXN3	0.218378	0.00343	0.010729
11716299_ITGAV	-0.14969	0.00343	0.010729
11763182_RQCD1	-0.18865	0.003431	0.010732
11716822_BDH2 ///	-0.14413	0.003434	0.010742
11726321_ERO1A	0.210018	0.003436	0.010747
11736056_BORCS7	0.23905	0.003439	0.010755
11725018_RCOR2	-0.24782	0.00344	0.010758
11716641_PSMB10	0.207303	0.00344	0.010758
11729991_CACNA1A	-0.15697	0.003441	0.010758
11757548_ADAMTSL1	0.273271	0.003441	0.010758
11728047_RAB3IP	0.267241	0.003442	0.010762
11746271_ZNF639	0.162762	0.003443	0.010763
11736384_METTL10	-0.3277	0.003443	0.010763
11745608_WDR1	0.18421	0.003447	0.010774
11718361_COIL	0.298571	0.003448	0.010777
11715478_TFRC	0.173539	0.003448	0.010777
11756931_ZNF257	0.246751	0.003449	0.010778
11718731_MAPT	-0.1808	0.003449	0.010778
11759195_MDN1	0.210925	0.003452	0.010787
11723221_EXOC6	0.149658	0.003452	0.010787
11720335_ZMIZ1	-0.2433	0.003453	0.010789
11739093_AZIN1	-0.17056	0.003454	0.01079
11746546_LOC102723	-0.19621	0.003454	0.010792
11747835_FUBP1	0.145786	0.003457	0.010798
11763435_CAPN10	-0.18354	0.003459	0.010803
11749817_CSF1R	0.179198	0.003459	0.010804
11717082_TSPAN9	-0.20035	0.003459	0.010805
11739254_SLC35E1	0.140329	0.003462	0.010811
11734557_GALNT5	0.238226	0.003463	0.010813
11756757_SDR39U1	-0.19709	0.003465	0.010819
11753317_TBCEL	0.350788	0.003466	0.010821
11715251_CHST7	0.238831	0.003468	0.010827
11752087_TEAD1	0.400735	0.00347	0.010833
11718043_MED17	0.171756	0.003471	0.010836
11730751_CRNKL1	0.220078	0.003472	0.010837

11734044_SOGA3	0.748601	0.003472	0.010839
11763022_CLEC7A	-0.33054	0.003474	0.010842
11732364_ZNF2	0.159607	0.003476	0.01085
11728870_ELAVL3	0.194498	0.003479	0.010857
11727409_NCLN	0.167169	0.003481	0.010863
11724066_HCFC1R1	-0.15273	0.003482	0.010865
11720227_YDJC	-0.15022	0.003483	0.010868
11762512_ERICH1	0.23059	0.003485	0.010872
11759865_ZNF609	0.176318	0.003486	0.010877
11746492_SGCE	-0.17194	0.003489	0.010885
11760332_ZFAND2B	-0.22445	0.003489	0.010885
11727303_ZDHHC16	0.144398	0.003491	0.010889
11723006_S100A4	0.141889	0.003492	0.010892
11735429_RNF135	-0.15267	0.003498	0.010908
11724148_SFTPD	-0.16759	0.003498	0.010908
11756036_MLH1	-0.14219	0.003501	0.010919
11721518_SUDS3	0.377434	0.003505	0.010931
11763369_CHORDC1	-0.17933	0.003506	0.010932
11755499_METAP1D	-0.17574	0.003509	0.010941
11746574_RHOB	0.255509	0.003509	0.010941
11753267_PRPSAP2	-0.15223	0.003509	0.010941
11739063_GNG12	-0.17028	0.00351	0.010942
11758684_DUSP8 ///	0.266493	0.00351	0.010942
11754845_FAM109A	-0.22804	0.003512	0.010946
11752103_HK1	-0.16529	0.003513	0.010949
11722739_CEP250	-0.17399	0.003514	0.010952
11715335_OR6P1	-0.23156	0.003515	0.010954
11753409_AKR1E2	-0.22285	0.003519	0.010965
11719179_PVR	-0.17557	0.003519	0.010966
11717237_AMD1	-0.1409	0.00352	0.010968
11749082_PIN1P1	-0.17252	0.003526	0.010984
11729669_EIF3K	-0.16668	0.003527	0.010989
11726647_BTN3A1 //	-0.17994	0.003531	0.010999
11752159_HDLBP	-0.15236	0.003531	0.010999
11747924_SCML2	0.349445	0.003532	0.011003
11751399_NDRG3	0.177742	0.003533	0.011004
11724080_COL21A1	0.146723	0.003535	0.011008
11748555_CDKL3	0.303302	0.003535	0.011009
11755611_PFKFB3	-0.17515	0.003536	0.01101
11751852_TRAF4	0.202664	0.003537	0.011013
11735231_SPATA12	-0.24472	0.003537	0.011013
11728564_RTKN2	-0.31571	0.003538	0.011015
11719004_ANKH	0.156587	0.003539	0.011019
11721660_LMO4	-0.16912	0.003541	0.011023
11720065_C12orf4	0.192267	0.003544	0.011033
11718420_CLPTM1L	-0.17907	0.003545	0.011034
11746929_MOB3C	0.23737	0.003545	0.011035
11742281_NAF1	-0.21947	0.003549	0.011045
11720828_S100PBP	-0.19801	0.003549	0.011045
11732628_ZNF765	0.275374	0.003549	0.011045
11731814_GATA1	-0.17608	0.003553	0.011055

11729131_PSME3	-0.21619	0.003554	0.011056
11727719_BCAP29	0.151583	0.003558	0.011071
11756803_MS4A14	0.22171	0.003559	0.011071
11716399_TP53INP2	-0.17004	0.003559	0.011072
11717331_BMS1	0.164361	0.00356	0.011074
11762345_LOC100128	-0.29931	0.003561	0.011077
11755968_NRG1	0.299708	0.003562	0.011078
11733632_P2RY14	-0.19594	0.003563	0.01108
11756565_IDH3A	-0.15321	0.003563	0.01108
11732285_DZIP3	-0.52021	0.003563	0.01108
11733511_MTMR1	0.150317	0.003564	0.011083
11721497_WDR33	-0.38355	0.003565	0.011084
11745657_PAX5	-0.166	0.003565	0.011084
11720111_SNTB2	-0.19518	0.003567	0.011087
11748378_BTBD7	0.242808	0.003567	0.011088
11727457_OFD1	0.162524	0.003568	0.011092
11717031_KIAA0141	-0.20585	0.003569	0.011094
11737195_RMDN2	0.241668	0.003572	0.0111
11744758_PSORS1C1	0.178277	0.003572	0.011102
11716860_PIAS3	-0.158	0.003573	0.011103
11731639_GAB1	0.482042	0.003573	0.011104
11759892_PLEKHA8	0.290421	0.003574	0.011105
11719233_PTPRS	-0.24032	0.003575	0.011108
11731730_VPS13A	0.314413	0.003577	0.011112
11744371_CLDN4	-0.23144	0.00358	0.011123
11755394_ZNF575	-0.33471	0.003582	0.011128
11756295_ARFIP2	0.165683	0.003584	0.011133
11754141_FXYD6 ///	-0.20124	0.003586	0.011137
11763762_RPUSD1	-0.1685	0.003586	0.011137
11733118_SLC35B4	0.221622	0.003591	0.011151
11716671_PIGY ///	-0.15114	0.003593	0.011157
11720697_HDAC7	-0.15708	0.003593	0.011157
11742095_SCLY ///	0.16254	0.003594	0.011159
11727460_ZXDC	-0.21953	0.003598	0.011171
11747859_ABCE1	-0.16905	0.0036	0.011177
11742127_PAOX	-0.24615	0.003603	0.011186
11734316_WDR17	0.226141	0.003604	0.011188
11740219_ZFP37	0.343278	0.003607	0.011195
11737053_HSPD1	-0.19668	0.003608	0.011198
11720305_VAT1	-0.23706	0.003609	0.011201
11727386_NPY1R	-0.1455	0.003613	0.011212
11752379_MCTP1	0.371516	0.003617	0.011226
11730608_HNF4G	0.259424	0.003618	0.011227
11744609_ATXN7	0.246984	0.003622	0.011237
11719265_MLPH	-0.21871	0.003622	0.011237
11732923_ENAM	-0.20643	0.003622	0.011237
11724201_TMEM143	-0.24011	0.003624	0.011244
11761858_CCM2	-0.2241	0.003625	0.011244
11733225_RLTPR	-0.19525	0.003625	0.011246
11754917_SP100	0.186884	0.003626	0.011246
11717408_SH2B3	0.1779	0.003626	0.011246

11761888_PSG9	-0.19665	0.003626	0.011246
11720292_LIN28B	0.814371	0.003628	0.011251
11739929_MAN1C1	-0.259	0.00363	0.011257
11727707_BBC3	0.268851	0.003632	0.011263
11745207_CDK5RAP1	-0.16711	0.003636	0.011275
11728877_ARID1B	0.237182	0.003637	0.011276
11735190_FBXO48	-0.27805	0.003638	0.011277
11739727_NAP1L1	0.153647	0.003639	0.01128
11753962_SSR3	-0.22509	0.003639	0.011281
11750288_DYX1C1	0.208795	0.003642	0.011288
11748672_POLR1E	0.207097	0.003643	0.01129
11749515_SHKBP1	-0.21459	0.003643	0.01129
11718854_MRPS30	0.16861	0.003645	0.011295
11734333_LINC01559	-0.18399	0.003645	0.011295
11718256_URGCP	0.20127	0.003646	0.011297
11717635_MLH1	-0.21759	0.003647	0.011299
11753064_MX2	-0.19673	0.003648	0.011302
11748390_ZDHHC16	0.17585	0.003648	0.011302
11729440_TMEM31	-0.3111	0.003648	0.011302
11719677_MED21	-0.19683	0.003649	0.011304
11744380_COL3A1	0.291247	0.00365	0.011304
11719705_BCKDK	-0.18122	0.003651	0.011307
11756192_CLASP2	-0.3526	0.003651	0.011307
11760786_C2CD2	0.204753	0.003651	0.011307
11760233_SFSWAP	0.236137	0.003653	0.011312
11760288_VAMP4	0.303307	0.003658	0.011326
11756449_RBCK1	0.15291	0.003663	0.011342
11745567_USP48	-0.15934	0.003663	0.011342
11719502_DHX36	0.156963	0.003665	0.011344
11749232_ZCCHC6	0.291621	0.003665	0.011344
11732185_PAPOLG	0.247344	0.003666	0.011347
11740796_CALHM3	-0.22856	0.003666	0.011348
11718902_TGFBR3	0.2432	0.003667	0.01135
11729118_CDKN1C	0.198104	0.003668	0.011353
11722720_BAIAP2	0.186094	0.003671	0.011359
11758246_ARL4D	-0.15253	0.003672	0.011362
11726072_EMC2	-0.25659	0.003673	0.011364
11720863_PNPLA4	-0.19259	0.003673	0.011365
11716460_TUBA4A	-0.18035	0.003675	0.01137
11722492_SEZ6L2	-0.17943	0.003675	0.01137
11717840_ETNK1	0.18806	0.003677	0.011375
11727958_ING1	-0.27709	0.003678	0.011376
11718312_ACADSB	-0.29658	0.003679	0.01138
11740467_MAGIX	-0.27681	0.00368	0.011382
11718576_NET1	0.219638	0.003682	0.011387
11737483_RAET1G	-0.23123	0.003682	0.011387
11745382_COG7	0.247641	0.003683	0.01139
11731763_RPS6KA2	-0.17442	0.003684	0.01139
11745624_INADL	0.408509	0.003684	0.011391
11728572_MBNL1	0.152511	0.003685	0.011392
11759678_RAB12	-0.29723	0.003685	0.011392

11739310_DLG1	0.278202	0.003685	0.011393
11725669_HS2ST1	0.267848	0.003686	0.011393
11715613_BTG2	0.346629	0.003688	0.0114
11764038_STYXL1	-0.16851	0.003691	0.011408
11757061_GCSH ///	-0.14934	0.003692	0.011409
11716585_PDZD11	-0.18741	0.003693	0.011411
11744202_C19orf70	-0.15684	0.003693	0.011412
11728875_LIN9	-0.23582	0.003694	0.011415
11720983_PSMC6	-0.16307	0.003697	0.011422
11728025_PPM1J	-0.34099	0.003699	0.011428
11734011_TRAPPC13	0.171852	0.003703	0.011438
11753889_STAT6	-0.23617	0.003705	0.011444
11758748_RAB6A	0.303111	0.003705	0.011445
11753292_CD44	0.142303	0.003705	0.011445
11715389_FKBP1A	0.207627	0.003706	0.011447
11743676_TTLL7	0.353578	0.003708	0.01145
11759097_ZNF703	-0.17456	0.003708	0.011451
11731252_PSMG4	-0.17384	0.003709	0.011451
11736577_IRAK1BP1	-0.26573	0.003709	0.011451
11741307_KLK3	-0.22105	0.003709	0.011451
11734873_SCAPER	0.166692	0.003709	0.011451
11745830_ALAD	-0.16566	0.003709	0.011451
11758024_TTC31	-0.1466	0.00371	0.011451
11728476_CXCL3	0.526517	0.003711	0.011454
11726322_ACOX1	-0.20176	0.003712	0.011456
11738997_DPYSL2	0.155604	0.003712	0.011456
11716223_SEC61G	-0.17675	0.003712	0.011456
11750006_COA1	-0.16244	0.003713	0.011458
11757538_GOT1	-0.14143	0.003713	0.011459
11740280_SAMD15	0.298509	0.003714	0.01146
11725007_DACT3	-0.19232	0.003716	0.011465
11735073_ZNF596	0.336381	0.003717	0.011468
11731282_TMEM72	-0.18529	0.003718	0.011469
11752318_BTN3A3	-0.1654	0.003718	0.011469
11756680_APH1B	0.206174	0.003719	0.011471
11737792_IDH3A	-0.17915	0.003719	0.011471
11733958_GTPBP3	-0.1693	0.00372	0.011473
11723022_BCKDHB	-0.27254	0.003721	0.011477
11719829_SLC26A11	-0.17886	0.003722	0.011478
11752516_ANKHD1 //	0.180295	0.003723	0.011481
11727184_ZNF175	0.267109	0.003725	0.011487
11718433_SASH1	0.146364	0.003727	0.01149
11759635_THOC2	0.266057	0.003728	0.011493
11731598_KLHL1	0.210167	0.003728	0.011493
11725402_C11orf57	-0.17445	0.003729	0.011495
11728763_GAL3ST4	-0.36382	0.003729	0.011495
11761643_DHPS	-0.17775	0.003729	0.011495
11747093_PPP3R1	0.210323	0.003731	0.011498
11747359_JADE1	-0.18135	0.003732	0.011503
11757502_SCRG1	0.441035	0.003737	0.011515
11741057_ENOX2	-0.25534	0.003737	0.011517

11753398_CLTA	0.143069	0.003739	0.011521
11755796_ADAM9	0.227638	0.003739	0.011521
11721133_GFPT2	-0.17437	0.003742	0.011529
11717952_ZDHHC18	0.163657	0.003742	0.01153
11744504_POLR2J	-0.18676	0.003744	0.011533
11747599_CLPTM1	0.175249	0.003744	0.011533
11758654_BLOC1S5 /	-0.19275	0.003746	0.011539
11762501_VGLL3	-0.21651	0.003748	0.011544
11715589_DYNC1H1	-0.18556	0.003749	0.011545
11749928_PGPEP1	0.228535	0.003749	0.011546
11723136_SRC	-0.16844	0.003753	0.011556
11727085_GJB2	-0.16434	0.003753	0.011556
11752390_MED15	-0.15035	0.003757	0.011566
11726838_SCAI	-0.23892	0.003757	0.011566
11758091_LCMT2	-0.19769	0.003758	0.011569
11718644_OSBPL8	0.150862	0.00376	0.011575
11729172_NIFK	0.141038	0.003762	0.011581
11751141_ACTR3	0.154148	0.003764	0.011586
11761012_LOC102724	-0.17114	0.003767	0.011595
11726958_TMEM81	-0.24064	0.003768	0.011596
11718278_NDUFB4	-0.15969	0.003768	0.011596
11745384_NDUFB4	-0.15969	0.003768	0.011596
11744283_COQ4	0.202809	0.00377	0.011602
11739365_FAM92A1	-0.14023	0.003771	0.011604
11732344_ZNF33A	-0.22255	0.003773	0.011608
11720102_SPIN1	0.176635	0.003774	0.011611
11719161_GPT2	-0.1685	0.003775	0.011614
11720390_PTPN23	-0.20412	0.003777	0.011618
11757067_ING5	-0.18002	0.003778	0.011621
11719653_SV2A	-0.17175	0.00378	0.011625
11733230_PTPRB	0.300217	0.00378	0.011626
11759035_STT3B	-0.15648	0.00378	0.011626
11759844_ZNF778	0.174953	0.003781	0.011626
11752859_ABHD6	0.18634	0.003782	0.01163
11752291_SLC30A6	0.235521	0.003785	0.011638
11748674_AFF4	0.264698	0.003785	0.011638
11740074_SCN8A	0.226291	0.003786	0.01164
11741456_WIPF3	-0.15108	0.003791	0.011654
11723529_GOSR1	-0.20091	0.003792	0.011656
11722528_PTPN21	0.393764	0.003793	0.011658
11747078_SPOCK3	-0.32018	0.003796	0.011667
11721237_LHFPL2	0.221916	0.003797	0.011669
11748043_KMT5A	0.181646	0.003799	0.011675
11716048_TRIB1	0.237331	0.0038	0.011678
11730405_MEX3B	-0.21694	0.003802	0.011684
11735122_TIMM8A	-0.21244	0.003806	0.011694
11762400_PPIL2	-0.19731	0.003808	0.011701
11724370_KIAA1644	-0.15712	0.00381	0.011704
11752823_KLHDC3	0.165408	0.00381	0.011706
11751776_PHB	-0.16618	0.003811	0.011706
11721430_SYBU	0.176798	0.003814	0.011715

11740461_ATF6	0.276014	0.003815	0.011718
11746710_DOCK10	0.207005	0.003821	0.011734
11734506_MECOM	-0.20665	0.003821	0.011735
11761017_SDHAP1 //	-0.23282	0.003822	0.011735
11729870_EMILIN1	-0.21648	0.003822	0.011735
11737486_SRF	-0.15685	0.003822	0.011736
11715125_MORC2	-0.15537	0.003823	0.011737
11756877_GNGT2	-0.24654	0.003823	0.011738
11737111_ERC1	0.150759	0.003825	0.011742
11734430_BPIFC	-0.17921	0.003825	0.011743
11742863_CD200	-0.38635	0.003826	0.011743
11743619_DUS4L	-0.14303	0.003826	0.011744
11719241_CAMK2N1	-0.21297	0.003828	0.01175
11725799_USP46	-0.15589	0.00383	0.011754
11759735_OBSCN	-0.22926	0.003831	0.011756
11739101_SYF2	0.174394	0.003831	0.011756
11723535_PDGFRL	-0.29434	0.003834	0.011764
11735895_BCL2L11	-0.26167	0.003834	0.011764
11720459_CAPRIN1	-0.15761	0.003838	0.011774
11737773_MCU	0.172628	0.00384	0.011779
11758582_SIX4	0.169706	0.003843	0.01179
11735578_VPS13B	0.151334	0.003848	0.011804
11717406_FAM120AOS	0.336871	0.003848	0.011804
11719845_SMAP2	0.178684	0.003849	0.011805
11755875_STRN	0.228515	0.00385	0.011806
11731578_ACSS1	-0.31053	0.003855	0.01182
11763785_KCNN4	0.145287	0.003855	0.011821
11756167_FOXF2	0.145098	0.003856	0.011823
11717260_CCDC142 /	0.173631	0.003857	0.011825
11757343_FIBP	-0.1527	0.003858	0.011829
11759984_ZNF273	-0.27029	0.003859	0.011831
11738138_HSPBP1	-0.16007	0.00386	0.011832
11754267_HBA1 ///	-0.18826	0.003862	0.011837
11730144_KLHL31	-0.18649	0.003865	0.011845
11722816_RANBP2	0.16905	0.003867	0.011851
11736220_TANGO2	-0.17059	0.003867	0.011852
11762709_CYP2U1	-0.16964	0.00387	0.011858
11731762_RPS6KA2	-0.22401	0.003872	0.011866
11726733_AGGF1	-0.14929	0.003874	0.011869
11756386_WDR1	-0.16366	0.003875	0.011872
11717213_NSMAF	0.208426	0.003875	0.011872
11752360_RBM22	0.136713	0.003875	0.011872
11715897_SCARB2	-0.14721	0.003878	0.011878
11722929_CNKSR3	0.199325	0.003878	0.011881
11727117_NPPB	0.289398	0.003879	0.011881
11722599_P3H4	-0.28892	0.00388	0.011884
11722506_PLPPR2	-0.24932	0.003883	0.011893
11733045_SLC7A2	-0.32933	0.003884	0.011893
11744144_EIF3H	0.145447	0.003885	0.011898
11739097_ALDH3A2	-0.16492	0.003895	0.011927
11748073_LDLRAD4	-0.25012	0.003899	0.011937

11744087_GPANK1	0.151151	0.003904	0.011952
11756699_AKT2	-0.24885	0.003906	0.011958
11762262_NUB1	0.225137	0.003908	0.011964
11732693_PTH2R	-0.14973	0.003909	0.011966
11715903_DDX3X	0.147948	0.003914	0.011981
11718196_ATP6V0D1	0.176818	0.003916	0.011985
11743061_DCTPP1	-0.15116	0.003917	0.011987
11729969_WNT6	-0.20975	0.003917	0.011987
11747428_CDK20	0.234819	0.003919	0.011992
11752712_CEP128	-0.381	0.003924	0.012007
11726996_PINK1	-0.25549	0.003925	0.012011
11755680_SLC30A6	0.255097	0.003928	0.012018
11742667_RPS28	0.135887	0.00393	0.012023
11723707_AIFM2	0.178347	0.00393	0.012023
11718041_SNRPE	-0.26653	0.003931	0.012024
11727960_ING1	-0.20388	0.003931	0.012024
11720914_ACP2	-0.17953	0.003932	0.012026
11727923_PURA	-0.1508	0.003933	0.012029
11719225_NXN	-0.27948	0.003934	0.012032
11725282_YJEFN3	0.205597	0.003935	0.012033
11729766_ZNF331	-0.25065	0.003938	0.01204
11757259_SCARNA9L	-0.21126	0.003938	0.01204
11751167_P2RX5	0.185649	0.003938	0.012041
11726716_DDX59	-0.25246	0.003939	0.012042
11750027_SLC35F2	0.141884	0.003943	0.012055
11728896_LRP8	-0.19686	0.003947	0.012065
11741339_GAB1	0.302452	0.003947	0.012065
11749333_GNL3	0.138761	0.003953	0.012081
11741165_FAM193A	0.188886	0.003954	0.012083
11737722_GLIS3	0.196905	0.003958	0.012095
11736623_TMEM110	0.195316	0.003958	0.012095
11726750_GTF3C4	0.170501	0.003961	0.012102
11739019_RAB5C	-0.14992	0.003961	0.012103
11749739_ASNA1	-0.19122	0.003961	0.012103
11730271_PPP2R5E	0.147776	0.003962	0.012104
11753019_EWSR1	0.22318	0.003963	0.012105
11719022_ZNF652	0.237426	0.003963	0.012105
11749891_HLF	-0.1834	0.003963	0.012106
11742465_PTP4A2	-0.16764	0.003965	0.012111
11724120_TRIM59	0.180598	0.003966	0.012114
11718146_HPCAL1	-0.16613	0.003968	0.012117
11716981_MIEN1	-0.15804	0.003968	0.012118
11716682_SLC39A9	-0.15181	0.00397	0.012123
11754932_XRCC6	-0.163	0.003972	0.012128
11731827_ZNF425	0.224849	0.003975	0.012135
11748586_XPO1	-0.1367	0.003976	0.012139
11720824_JAG1	0.200819	0.003977	0.012141
11731574_FRMD4B	0.164914	0.003978	0.012142
11726077_ANAPC15	-0.1692	0.003982	0.012154
11760998_COQ6	0.207918	0.003982	0.012155
11716353_STARD3	-0.20405	0.003986	0.012165

11716376_SERPINA5	-0.15667	0.003995	0.012193
11757362_NPM1	-0.15653	0.003998	0.0122
11747326_IPO4 ///	-0.17521	0.003999	0.012204
11751500_SLC22A10	-0.17682	0.004	0.012205
11751559_LRRK1	-0.25356	0.004004	0.012217
11745655_MED16	-0.15657	0.004005	0.01222
11726983_CCNC	0.187778	0.004007	0.012223
11745779_KMT2E	0.272133	0.004007	0.012223
11750291_IST1	0.158745	0.004007	0.012223
11757919_MEGF9	0.183111	0.004008	0.012225
11744450_ARHGAP4	-0.15979	0.00401	0.01223
11759931_PHTF1	0.205997	0.00401	0.01223
11747309_VTA1	0.16406	0.004013	0.012237
11761452_GOLGA3	-0.36015	0.004014	0.012241
11719709_PDPK1	0.199106	0.004017	0.012249
11721453_MRPL44	0.160394	0.004017	0.012249
11723705_ABHD5	0.242007	0.004018	0.012249
11745796_WBP11	-0.15924	0.004025	0.012271
11759568_SLC45A4	-0.35092	0.004026	0.012273
11755607_KIAA1549	0.166787	0.004028	0.012279
11738368_CLPSL2	-0.22068	0.00403	0.012285
11749968_RAD52	0.20373	0.004033	0.012292
11725323_ZNHIT2	0.199866	0.004037	0.012302
11757079_CCDC166	-0.17553	0.00404	0.01231
11757104_CCDC166	-0.17553	0.00404	0.01231
11722827_SDAD1	0.359895	0.004041	0.012312
11736118_ZFAND5	-0.20261	0.004041	0.012313
11737134_ZNF385A	-0.21922	0.004043	0.012317
11718087_SHKBP1	-0.16922	0.004043	0.012318
11734527_RECQL5	-0.35021	0.004045	0.012323
11739142_HNRNPF	-0.15688	0.00405	0.012338
11745309_FRMD3	0.228094	0.004051	0.012338
11751318_EIF4A1 //	-0.13628	0.004055	0.01235
11735866_OR12D2	-0.19048	0.004056	0.012353
11731752_RNF213	0.285502	0.004056	0.012353
11716647_HM13	0.194577	0.004057	0.012355
11722280_PHC3	-0.15345	0.004058	0.012356
11761180_TMED9	-0.20958	0.004058	0.012356
11744113_COQ5	-0.15424	0.004059	0.012359
11740267_CCDC18	-0.17623	0.004062	0.012365
11757658_LZIC	-0.16564	0.004063	0.012368
11731491_HK1	-0.15013	0.004065	0.012375
11736080_CAMLG	-0.17879	0.004066	0.012376
11744515_SLC25A38	0.147303	0.00407	0.012389
11742881_UBE2B	0.188253	0.004073	0.012396
11725288_RHBDL3	-0.26161	0.004073	0.012396
11742265_HFE	-0.22797	0.004074	0.012397
11753319_KCNMB3	0.232613	0.004074	0.012397
11752622_PXK	-0.24445	0.004074	0.012397
11719540_NEMP1	0.144745	0.004075	0.012397
11726665_TLE3	0.275864	0.004075	0.012397

11753150_CELF2	-0.27721	0.004075	0.012397
11748399_VCL	0.19681	0.004076	0.012399
11747088_GRID2	0.330944	0.004078	0.012404
11726984_CCNC	0.164691	0.004081	0.012414
11722299_RAB29	0.15758	0.004087	0.012431
11725464_SLC4A2	-0.14156	0.004088	0.012432
11755332_TJAP1	0.193132	0.004088	0.012433
11753748_MED16	-0.16924	0.004089	0.012434
11720898_MTM1	0.206842	0.00409	0.012436
11745786_CSNK2B	0.167489	0.004091	0.012438
11743031_S100A6	0.147118	0.004093	0.012443
11754203_PNPLA8	0.149573	0.004094	0.012446
11729569_CLEC12B	0.176955	0.004095	0.012448
11725152_L3HYPDH	0.15991	0.004095	0.012448
11749812_FOXO3 ///	0.154134	0.0041	0.012461
11746129_GSTM3	0.190719	0.0041	0.012463
11752633_OLFM1	-0.21937	0.004102	0.012465
11745874_EVL	-0.16951	0.004104	0.012473
11754547_ENOX2	-0.1727	0.004105	0.012473
11724194_SERF2	0.135852	0.004105	0.012473
11761966_TMC2	-0.30351	0.004105	0.012473
11757303_BNIP3L	0.168009	0.004105	0.012473
11715807_STARD10	0.195677	0.004106	0.012474
11757478_MAP2K1	-0.15012	0.004106	0.012474
11723612_TOX2	-0.32634	0.004107	0.012476
11751480_TMEM231	-0.26795	0.004109	0.012482
11721116_SPCS3	-0.14776	0.004116	0.012503
11731608_EGFL8 ///	-0.15357	0.004119	0.01251
11748143_SLC39A1	-0.16242	0.004121	0.012514
11730772_ZFYVE1	0.205313	0.004123	0.01252
11756463_TUBG2	0.181628	0.004125	0.012525
11741629_POLR3H	0.220235	0.004126	0.012528
11754733_TCF4	0.275797	0.004128	0.012532
11738252_HRH2	-0.16877	0.004128	0.012532
11719386_MAPRE3	0.22514	0.004132	0.012543
11729472_FANCA	-0.36455	0.004132	0.012544
11748445_HP1BP3	0.165701	0.004133	0.012545
11718171_RFWD2	0.180869	0.004138	0.012559
11724115_ZNF512B	-0.20006	0.004139	0.012563
11757279_TIMMDC1	0.145614	0.004146	0.012582
11755969_NUCB1	-0.14352	0.004147	0.012585
11729037_PRKX ///	0.274992	0.00415	0.012594
11760323_RBM4	0.242415	0.004157	0.012613
11724564_CHCHD1	-0.15346	0.004158	0.012617
11759084_SLC30A5	-0.28804	0.004159	0.012619
11718666_SRSF9	-0.14115	0.004162	0.012625
11741451_RDM1	-0.29105	0.004162	0.012625
11720934_DAGLB	0.25617	0.004162	0.012626
11724220_NAP1L2	0.394297	0.004163	0.012627
11720316_SLC44A2	-0.17601	0.004167	0.012638
11762882_C1S /// E	0.410442	0.004171	0.01265

11759906_EXD3	-0.15286	0.004172	0.012652
11723896_JAG2	-0.18704	0.004174	0.012658
11721533_TAF2	0.158522	0.004175	0.01266
11738177_NKX3-2	-0.31395	0.004176	0.012662
11755060_TCF3	-0.15352	0.004178	0.012668
11760399_PHBP19	-0.27396	0.004179	0.01267
11756828_RNF144A	0.269677	0.004181	0.012673
11761530_SRSF2	0.276991	0.004184	0.012682
11762457_ZFYVE26	-0.24037	0.004186	0.012689
11752536_DNAH6	0.274211	0.004187	0.01269
11724072_ZSWIM3	0.211144	0.004188	0.012691
11721186_RABGGTA	-0.15545	0.004191	0.0127
11753291_CD44	0.154906	0.004192	0.012703
11729286_MSL3	0.232741	0.004193	0.012705
11763758_VILL	-0.17001	0.004195	0.012709
11737163_ATP13A2	0.198638	0.004198	0.012718
11739105_TMEM33	-0.16017	0.0042	0.012723
11717974_FAM219B	0.236347	0.0042	0.012724
11732447_EXOG	0.190991	0.004201	0.012724
11719575_RINT1	0.145185	0.004201	0.012724
11729364_PRKAA2	0.266071	0.004207	0.012744
11740492_AGAP1	0.163622	0.004208	0.012745
11743684_GPR21 ///	0.14779	0.004208	0.012745
11748636_FPGT	-0.20738	0.00421	0.012749
11719213_LOC100996	0.179622	0.004212	0.012753
11723649_CIAO1	0.18834	0.004212	0.012755
11739229_FBXW11	-0.17565	0.004214	0.01276
11731620_ARRB2	-0.4058	0.004215	0.01276
11759656_KLHL7	-0.17908	0.004215	0.01276
11750394_MED15	-0.15706	0.004215	0.01276
11724124_PIDD1	0.154924	0.004216	0.012762
11719314_NMRK1	0.170027	0.004217	0.012763
11719207_NPTN	-0.18909	0.004218	0.012765
11748717_ERCC6L2	0.298933	0.004218	0.012765
11723944_PTTG1IP	0.16356	0.004218	0.012765
11760231_TDGF1P3	-0.21338	0.004219	0.012766
11741766_AGAP3	-0.13807	0.004222	0.012774
11735067_CERS1 ///	-0.15884	0.004223	0.012776
11753550_LINC00189	0.360746	0.004224	0.012779
11724672_ELP5	-0.20283	0.004225	0.012781
11747368_CDK10	-0.17817	0.004227	0.012786
11734933_SRSF2	-0.21374	0.00423	0.012796
11737329_SLC26A4	0.380417	0.004232	0.012802
11728946_GATA2	-0.19366	0.004234	0.012807
11730775_CFDP1	0.160311	0.004237	0.012816
11761165_VWA9	-0.25045	0.00424	0.012822
11742189_WDR17	0.324136	0.00424	0.012823
11727705_CCDC42	-0.1948	0.004242	0.012827
11720238_MAPK9	-0.20372	0.004242	0.012827
11728874_LIN9	-0.15466	0.004243	0.012827
11747027_SLC1A4	-0.1526	0.004245	0.012834

11730202_FAM104A	0.208096	0.004249	0.012846
11747044_WBSCR16	-0.15302	0.00425	0.012846
11719309_MKS1	-0.17319	0.004251	0.012849
11715771_MRPL13	-0.16691	0.004252	0.012851
11728897_FBXO17	-0.20017	0.004254	0.012856
11763500_ZNF93	0.161973	0.004256	0.012862
11747333_HSD17B4	-0.20504	0.00426	0.012875
11747860_ABCE1	-0.16186	0.004264	0.012884
11762850_ESPNP	-0.16962	0.004265	0.012887
11721826_ARPC5L	0.199266	0.004265	0.012887
11742931_BIRC6	0.205704	0.004266	0.012889
11751251_PML	-0.18022	0.004267	0.01289
11731669_GPR65	0.163024	0.004269	0.012894
11750026_DNM1L	-0.14503	0.004272	0.012904
11743350_C15orf48	0.457711	0.004273	0.012906
11725428_ABLIM3	0.498422	0.004274	0.012907
11719364_SPATS2L	-0.1916	0.004278	0.01292
11715980_AIP	-0.17275	0.004279	0.012921
11727610_ENSA	-0.18117	0.004281	0.012927
11718389_TRIM26	0.1896	0.004282	0.012929
11724336_HSPA12A	-0.26145	0.004284	0.012935
11742056_ZNF365	-0.27218	0.004289	0.012948
11744114_GMPPA	-0.27411	0.004294	0.012962
11752608_ST7 /// S	0.148267	0.004294	0.012963
11752970_RAD52	0.270279	0.004296	0.012966
11731346_DGKZ	0.16371	0.004296	0.012966
11759067_CCDC186	-0.37245	0.004297	0.012969
11724439_TSACC	-0.20904	0.0043	0.012979
11715470_TXN	-0.13976	0.004301	0.012979
11748238_TBCD	-0.22416	0.004301	0.01298
11746267_CHRND	-0.22379	0.004302	0.01298
11734127_CYP11B1	-0.20423	0.004303	0.012983
11755398_USP43	-0.25172	0.004304	0.012984
11750853_PRRC2A	-0.18762	0.004304	0.012985
11760856_FAM185A	-0.18956	0.004307	0.012994
11732564_ADAT2	-0.26159	0.004313	0.013009
11721153_STK3	0.149334	0.004313	0.013009
11716493_MAP4	-0.14246	0.004314	0.01301
11733698_SGK1	-0.14886	0.004315	0.013015
11762940_HLA-C	0.192532	0.004316	0.013016
11755113_UPF2	0.158272	0.004316	0.013016
11721628_SEPT6	-0.19134	0.00432	0.013026
11724061_OSBPL1A	-0.15044	0.00432	0.013027
11724126_MID2	0.165817	0.004321	0.013028
11761949_NALCN	0.355186	0.004321	0.013028
11735697_ENTPD6	0.158444	0.004324	0.013036
11716692_NPEPPS	0.140473	0.004325	0.013036
11739211_HNRNPK	-0.22342	0.004326	0.013041
11762130_CFHR3	0.217492	0.00433	0.013052
11737786_KCNAB2	-0.20426	0.004331	0.013053
11719998_SYNJ2BP /	0.138165	0.004331	0.013053

11726306_FAM204A	-0.26572	0.004331	0.013053
11721866_GNPDA2	0.183796	0.004334	0.013059
11734713_DBF4	-0.17504	0.004335	0.013061
11715220_C2orf44	0.270003	0.004337	0.013067
11746897_CRTAP	0.189109	0.004338	0.013068
11740240_FGFR4	-0.17211	0.00434	0.013074
11725546_OPCML	-0.17835	0.004341	0.013077
11761965_TMC2	-0.22473	0.004343	0.013083
11732077_IGSF22	-0.21257	0.004351	0.013106
11739108_CXorf56	-0.2181	0.004351	0.013106
11749568_DDX55	0.14373	0.004358	0.013126
11739220_CACUL1	-0.28945	0.004364	0.013141
11757484_MAMDC4	0.177333	0.004364	0.013142
11741823_NBPF7	-0.31419	0.004366	0.013146
11737312_CCL1	-0.24457	0.00437	0.013157
11722636_ELL3	-0.3238	0.004371	0.013161
11729203_PLXND1	-0.17434	0.004372	0.013161
11725466_LRRC28	-0.1928	0.004373	0.013164
11736480_TREML2	-0.19853	0.004373	0.013164
11761872_RAB3GAP1	-0.29258	0.004374	0.013164
11720991_SOX2	-0.18419	0.004375	0.013169
11737841_FAM214B	-0.20369	0.004376	0.01317
11756146_S100A4	0.144744	0.004377	0.013171
11727965_ELANE	-0.21575	0.004378	0.013174
11755629_OGFOD2	-0.20594	0.004381	0.013181
11720358_SMAP1	0.158973	0.004381	0.013182
11749338_GALNT11	-0.22247	0.004388	0.013202
11723989_NR1D1 ///	0.27303	0.004391	0.01321
11722523_TMCO4	-0.16287	0.004393	0.013216
11748970_TRA2A	0.177814	0.004394	0.013219
11763719_RPL18	-0.22443	0.004398	0.013228
11764251_MOSPD1	0.160477	0.004401	0.013237
11763366_ANKRD36 /	0.21592	0.004401	0.013237
11722464_C17orf80	0.177226	0.004404	0.013245
11746517_ZNF484	0.193453	0.004406	0.013248
11720116_STK26	-0.22757	0.004408	0.013254
11749324_KCTD15	-0.34555	0.004412	0.013265
11744766_NPRL2	-0.15284	0.004416	0.013278
11735844_CCDC144A	0.298587	0.004417	0.01328
11748041_MAVS	-0.19143	0.004418	0.013281
11761101_FCAR	0.376357	0.004423	0.013296
11742259_ENTPD2	-0.20109	0.004423	0.013296
11757281_ATP5G3	-0.13982	0.004424	0.013297
11752368_DMRT2	-0.14325	0.004426	0.013301
11716591_BTBD1	0.165207	0.004427	0.013304
11728905_SATB2	0.246038	0.004429	0.013311
11732641_JKAMP	-0.18429	0.004432	0.013317
11761377_ZNF90	-0.30294	0.004433	0.013321
11750604_GTF2IRD1	-0.17216	0.004434	0.013321
11749694_CD276	-0.21384	0.004435	0.013326
11716017_MAL2	0.228135	0.00444	0.01334

11737047_KCNH6	-0.31791	0.004442	0.013343
11720813_INTS10	-0.15436	0.004442	0.013344
11731533_MTPAP	0.202135	0.004444	0.013349
11721654_AGPAT4	0.251297	0.004445	0.013352
11757179_RPL4 ///	0.260359	0.004447	0.013354
11744445_DUSP14	0.155594	0.00445	0.013365
11731297_MAGEA2 //	-0.14503	0.004451	0.013367
11723402_STK4	0.169924	0.004452	0.013368
11736461_RAB3B	0.257796	0.004453	0.013371
11744401_CAMTA1	-0.15031	0.004454	0.013371
11748360_RAD23A	-0.14898	0.004455	0.013374
11722957_SMURF1	0.177379	0.004455	0.013375
11739828_CYS1	-0.30017	0.004456	0.013375
11747640_TDP2	-0.21651	0.004459	0.013384
11725503_TJP1	0.184962	0.00446	0.013387
11741182_PROSER3	-0.17669	0.004461	0.013387
11717270_SNAPC2	-0.23109	0.004462	0.013392
11730064_INSM2	0.270873	0.004463	0.013393
11759433_POLR3A	0.164961	0.004467	0.013405
11732004_ADAL	0.239543	0.004468	0.013405
11719191_FBXO9	0.147382	0.004469	0.013407
11748223_LIPH	0.463406	0.004471	0.013415
11729566_GTDC1	0.177011	0.004472	0.013417
11734749_LIN52	-0.17921	0.004474	0.013422
11758348_PTTG1IP	0.191573	0.004475	0.013423
11758000_CXADR	0.252387	0.004475	0.013423
11715104_OTOP2	-0.17093	0.004475	0.013423
11760099_SCARB1	-0.22822	0.004476	0.013424
11763742_AMT	-0.2043	0.004477	0.013426
11755310_ACOT1 ///	0.148012	0.004478	0.013429
11752561_CDH13	0.155094	0.004479	0.013432
11760262_STARD3	-0.20586	0.00448	0.013433
11742559_OR2T27	-0.25337	0.004481	0.013435
11761475_ULBP3	0.367677	0.004484	0.013443
11758255_CSNK1G3	-0.15534	0.004484	0.013443
11727157_STRBP	0.17261	0.004486	0.013447
11727858_NAA40	-0.25677	0.004487	0.01345
11717049_DES /// S	-0.1782	0.004488	0.013451
11727479_HECTD1	0.142229	0.004491	0.01346
11730858_ZC2HC1A	0.145916	0.004491	0.01346
11743910_FAM69A	-0.19743	0.004492	0.013461
11758623_KMT2A	0.177953	0.004492	0.013461
11718098_ENDOD1	-0.21945	0.004493	0.013463
11742968_MED8	0.199864	0.004494	0.013463
11751106_NUP35	0.140681	0.004495	0.013466
11715513_NDFIP1	-0.14947	0.004498	0.013474
11758376_CFAP69	0.29073	0.0045	0.01348
11731214_GNB1L	0.159909	0.0045	0.01348
11754571_GMDS	-0.1916	0.004505	0.013492
11730401_RASGRF1	0.5697	0.004505	0.013493
11739586_CLCN3	0.19154	0.004505	0.013493

11724421_EPHB3	-0.1716	0.004507	0.013497
11716582_G3BP2	-0.17861	0.004508	0.013498
11755504_RBM34	0.346597	0.004514	0.013515
11729654_SMAD2	-0.293	0.004516	0.013523
11756542_GPR87	0.325446	0.004518	0.013526
11727681_ACVR2B	-0.16074	0.004519	0.013527
11742749_SPPL3	0.169309	0.004519	0.013527
11742071_RAB27A	-0.2186	0.004519	0.013527
11727408_NCLN	-0.15702	0.004521	0.013532
11736347_HFE	-0.28427	0.004522	0.013535
11762511_IL31RA	0.326221	0.004522	0.013535
11748461_RHOBTB1	0.162082	0.004523	0.013537
11751510_RARA	0.211966	0.004524	0.013537
11729101_AKR1C2	-0.22596	0.004524	0.013538
11720306_SERP1	-0.14408	0.004526	0.013542
11722476_CMC1	-0.20523	0.004528	0.013546
11739758_KBTBD3	-0.16041	0.004533	0.013562
11722634_ICE2	-0.23164	0.004535	0.013566
11754998_MCTP1	0.257099	0.004539	0.013577
11720441_OLFML2B	-0.21233	0.004539	0.013577
11759260_EIF5A	-0.14843	0.004539	0.013577
11752189_ARHGEF6	0.322996	0.00454	0.013578
11741635_GPR6	-0.21776	0.004543	0.013586
11716971_MRAS	0.164192	0.004543	0.013586
11754797_CHUK	0.179807	0.004543	0.013586
11726897_ADGRL3	-0.3186	0.004549	0.013601
11726630_HTRA2	-0.16732	0.004551	0.013606
11752791_EPHB4	-0.18228	0.004552	0.013608
11739690_DCAF11	-0.22586	0.004553	0.013611
11750693_FTSJ3	0.140781	0.004553	0.013612
11721760_TSEN54	-0.20593	0.004555	0.013614
11715599_ANAPC5	-0.16119	0.004555	0.013614
11743193_PARD6G	-0.18265	0.004558	0.013624
11733363_ZFP64	0.219725	0.00456	0.013628
11743883_NRF1	-0.20253	0.00456	0.013629
11750002_RAB2A	-0.1534	0.004566	0.013644
11726802_EBF1	0.233971	0.00457	0.013656
11726083_FAM69B	-0.18362	0.004572	0.013661
11719091_TAB1	-0.16616	0.004573	0.013663
11737030_SLC9A7	0.275346	0.004574	0.013664
11717277_THRAP3	-0.26689	0.004578	0.013676
11722766_MBNL2	0.225674	0.004579	0.013678
11733546_HCN1	0.250792	0.004581	0.013685
11755832_XRRA1	0.195327	0.004582	0.013686
11730856_HMGN3	-0.15461	0.004583	0.013686
11720762_FBXO3	0.155332	0.004584	0.013689
11730298_SPARCL1	-0.19964	0.004584	0.013689
11724384_RAP2C	0.163079	0.004584	0.013689
11740585_PTGFR	0.347907	0.004586	0.013693
11720308_SERP1	-0.13796	0.004588	0.013699
11739364_FAM92A1	-0.15098	0.00459	0.013703

11717816_ZBTB5	0.157678	0.004591	0.013705
11743278_WNK1	-0.15757	0.004596	0.013719
11746518_BVES	0.249214	0.004598	0.013724
11753955_C16orf13	-0.13715	0.004599	0.013726
11719831_KMT5A	-0.20745	0.004599	0.013726
11739976_ITGA11	0.146935	0.004603	0.013738
11715154_ZNF675	0.485995	0.004604	0.013741
11720597_CSRNP2	0.17341	0.004607	0.013748
11759778_ASXL1	0.270354	0.004607	0.013748
11751985_EPS15	0.17462	0.004611	0.013757
11719070_HSPA13	-0.20678	0.004611	0.013757
11745880_GUCA1A	-0.28526	0.004611	0.013757
11736498_TNRC6B	0.171451	0.004613	0.013762
11724117_SAMD9L	0.231432	0.004615	0.013766
11746595_PCM1	0.153784	0.004617	0.013771
11722272_MSANTD4	0.144999	0.004617	0.013771
11734667_KPNA1	0.209112	0.004618	0.013773
11738464_TPH2	-0.15119	0.004619	0.013776
11749220_C6orf89	0.155621	0.00462	0.013776
11721859_MIPEP	-0.17226	0.004622	0.013782
11742202_DSPP	-0.23312	0.004624	0.013786
11722811_RAB7A	0.144801	0.004625	0.01379
11722193_C12orf75	-0.13349	0.00463	0.013804
11757387_RPS28	0.137529	0.004631	0.013804
11725488_ATG14	0.166351	0.004631	0.013804
11730374_FYB	0.317258	0.004631	0.013804
11728347_ABI3	-0.20518	0.004631	0.013804
11722859_ZNF395	-0.17637	0.004634	0.01381
11728438_BRWD1	0.272476	0.004634	0.01381
11746040_ZFHX2	0.265044	0.004637	0.013818
11759424_ANXA5	-0.24855	0.004638	0.01382
11760817_CDKL3	-0.41251	0.004638	0.01382
11754209_SLC44A2	-0.17124	0.004638	0.01382
11731490_NUP43	-0.15713	0.004639	0.013821
11741500_DAGLB	0.224625	0.004642	0.01383
11717310_GNL3	0.150745	0.004645	0.013836
11737477_C2orf54	-0.2339	0.004645	0.013836
11722365_SLC4A1AP	0.161272	0.004646	0.013837
11717651_SPRY1	-0.14847	0.004646	0.013837
11728448_ADCK5	-0.18155	0.004649	0.013845
11737771_PKIG	0.186732	0.00465	0.013849
11748129_RNF220	-0.16209	0.004651	0.01385
11724297_BMP7	-0.3132	0.004652	0.013851
11763791_IL22RA1	-0.22873	0.004653	0.013854
11730431_ZDHHC7	-0.13928	0.004654	0.013856
11760689_ZGRF1	-0.24404	0.004656	0.013861
11720967_GPSM2	-0.17932	0.004657	0.013865
11716077_HSBP1	0.133881	0.004658	0.013867
11740419_XRRA1	0.224464	0.00466	0.013871
11721410_STK16	-0.20306	0.004661	0.013873
11734847_ERBB2IP	0.278349	0.004661	0.013873

11721624_WSB1	0.165779	0.004665	0.013882
11759431_TAF3	0.283081	0.004665	0.013882
11731734_LYPD6	-0.18399	0.004668	0.01389
11729165_XPO5	-0.1746	0.00467	0.013895
11742790_ATP6AP1L	-0.17153	0.00467	0.013895
11742072_PBX3	0.175378	0.004672	0.013901
11716451_RFTN1	0.18149	0.004673	0.013901
11758087_DPYS	0.19349	0.004673	0.013901
11752578_SOX5	-0.17286	0.004675	0.013905
11759833_NDUFS7	-0.20346	0.004676	0.013908
11745351_CEP104	0.173883	0.004676	0.013908
11739669_SS18L1	-0.16139	0.004677	0.01391
11757268_HNRNPA1 /	-0.22514	0.004679	0.013916
11717520_LOC102724	0.169107	0.004681	0.01392
11740870_ZNF682	0.159455	0.004681	0.01392
11728695_ARG2	-0.14671	0.004686	0.013932
11739745_MLH3	0.170747	0.004687	0.013934
11761468_TFAP2A	0.234482	0.004692	0.013948
11755960_LRRC39	0.292516	0.004698	0.013967
11745962_QRICH1	-0.2329	0.004699	0.013967
11737862_PIGF	-0.28217	0.004703	0.013979
11752251_SPARCL1	-0.17777	0.004708	0.013994
11758300_B2M	0.132242	0.004717	0.014019
11740497_SPATA31E1	-0.25076	0.004721	0.01403
11754647_MAN1B1	-0.18477	0.004724	0.014037
11716644_ERLEC1	-0.15277	0.004724	0.014037
11755497_WBSCR16	-0.17318	0.004724	0.014037
11715267_EPPK1	0.284359	0.004725	0.01404
11744718_CTHRC1	0.141937	0.004727	0.014044
11749808_ZNF107	0.243856	0.004732	0.014057
11734095_ZNF845	0.139498	0.004737	0.014072
11762529_FERMT1	-0.27562	0.004737	0.014072
11745185_LOC644762	-0.14727	0.004738	0.014073
11755516_KIF16B	0.203031	0.004744	0.014092
11718778_PCMT1	-0.15902	0.004745	0.014093
11729542_CAMKK2	-0.21617	0.004746	0.014093
11739187_CCDC142 /	0.208683	0.004748	0.014098
11742422_CEP192	-0.17638	0.004756	0.014121
11761173_HIF3A	0.156352	0.004757	0.014122
11761174_HIF3A	0.156352	0.004757	0.014122
11751041_PCMTD2	0.253655	0.004757	0.014123
11746159_PBX3	0.162094	0.004759	0.014127
11754361_GSK3B	0.155221	0.00476	0.014129
11727374_HMGCR	-0.15638	0.004762	0.014136
11725623_JMJD4	-0.21077	0.004763	0.014137
11716819_CUL1	0.167663	0.004763	0.014137
11748958_ACSS1	-0.24941	0.004765	0.014141
11733635_FAM92B	-0.25131	0.004769	0.014152
11742563_OR10A7	-0.15383	0.00477	0.014154
11716485_STK38	-0.33583	0.004773	0.014161
11721523_ZNF706	0.144248	0.004774	0.014166

11739841_BCL6	-0.26401	0.004775	0.014167
11719310_CRY2	0.204558	0.004777	0.014171
11718384_C2orf68	-0.17254	0.004782	0.014187
11758621_FAM134A	0.179672	0.004784	0.014191
11745354_SNX17	0.145073	0.004785	0.014192
11761290_CTU2 ///	-0.17475	0.00479	0.014207
11718974_LPL	-0.14564	0.00479	0.014207
11724991_GTPBP3	-0.16947	0.004792	0.014212
11744048_MAP1B	0.18817	0.004794	0.014217
11717478_LAMP2	0.146363	0.004796	0.014223
11746288_LOC100289	0.191528	0.0048	0.014233
11734962_ADAM33	-0.18844	0.004801	0.014233
11756415_RBM6	0.161274	0.004803	0.01424
11759186_CCDC103	-0.22912	0.004803	0.01424
11716099_RAP1A	0.14623	0.004804	0.01424
11723288_CRISPLD1	-0.29678	0.004805	0.014242
11736316_WBP5	-0.20766	0.004805	0.014242
11737590_PHTF2	0.378295	0.004805	0.014242
11742962_IP6K2	0.211569	0.004806	0.014242
11740517_FAM76A	-0.47899	0.004808	0.014249
11745889_KIDINS220	0.183741	0.004811	0.014255
11734026_ZNF440	-0.40889	0.004811	0.014255
11753248_TPP1	0.175422	0.004815	0.014266
11723096_CYLD	-0.18194	0.004816	0.014268
11748142_SLC39A1	-0.25289	0.004816	0.014269
11719129_POLR3D	-0.14705	0.004817	0.01427
11740944_MPL	-0.1841	0.004818	0.01427
11759010_SLC25A6	-0.17288	0.004818	0.01427
11730835_MTX2	0.16171	0.004818	0.01427
11739188_BAG5	-0.15576	0.00482	0.014275
11731166_PARL	0.158038	0.004822	0.014281
11725688_RHPN2	-0.2008	0.004828	0.014296
11723517_CAB39L	0.238951	0.004829	0.014298
11754817_REG1P	-0.14583	0.004832	0.014306
11725994_MRPL11	-0.33228	0.004833	0.014309
11756797_TTC39B	0.149869	0.004836	0.014318
11721010_GDPD5	0.235208	0.00484	0.014328
11750439_SLC17A1	-0.34104	0.00484	0.014328
11762040_GUCY1B2	0.283431	0.004842	0.014333
11732563_ADAT2	0.218023	0.004843	0.014334
11748489_MAGED2	0.188784	0.004844	0.014336
11718916_IRF7	-0.17867	0.004849	0.014351
11746472_XRN2	0.182427	0.004852	0.014359
11763740_ACTR6	-0.1559	0.004854	0.014364
11718379_PRKAG2	0.161727	0.004855	0.014366
11727895_G3BP2	0.158979	0.004859	0.014376
11717669_IWS1	-0.16575	0.004861	0.014382
11747958_CDIP1	-0.23113	0.004866	0.014396
11750717_TNFRSF8	-0.30283	0.004868	0.014402
11746261_KIF5C	0.209738	0.004871	0.014408
11728741_COQ3	-0.18605	0.004872	0.014411

11717839_PDCD6	0.132986	0.004873	0.014411
11760243_CNTN1	-0.19105	0.004875	0.014418
11744944_FAM153A /	-0.20107	0.004876	0.014421
11760806_SLC4A2	-0.15579	0.004881	0.014434
11757242_KRT17P5	-0.25697	0.004882	0.014435
11763831_SLC12A4	0.172448	0.004882	0.014436
11755555_FANCM	-0.25833	0.004883	0.014437
11720910_NELL2	0.331805	0.004885	0.014442
11728862_DCAF17	0.198806	0.004887	0.014447
11751109_KCTD20	0.373511	0.004888	0.014448
11730527_DAPK2	0.14413	0.004888	0.014448
11740764_SLC39A8	-0.21218	0.004888	0.014449
11730201_GPM6A	0.228672	0.004892	0.01446
11753009_UCKL1	-0.2422	0.004894	0.014464
11734495_GRB10	0.207742	0.004895	0.014466
11739057_NFE2L2	0.145489	0.004896	0.014468
11723670_BNIP2	0.145013	0.004897	0.014471
11745146_ZNF624	0.380974	0.004898	0.014473
11724881_ZNF217	0.154721	0.004899	0.014473
11730781_SCRN1	-0.15107	0.004901	0.014479
11745925_ZEB1-AS1	0.21658	0.004903	0.014483
11718067_MYO1E	-0.21772	0.004907	0.014495
11724854_RND2	-0.26392	0.004907	0.014495
11726422_WDR53	-0.16271	0.004908	0.014496
11747642_TMEM129	-0.18998	0.004913	0.01451
11733599_METRNL	0.208403	0.004914	0.014512
11722044_METTL17	-0.16873	0.004916	0.014517
11743518_NNT	-0.20001	0.004919	0.014525
11749786_HNRNPF	-0.20206	0.00492	0.014527
11726837_SCAI	-0.36754	0.004921	0.014529
11716718_GLA	-0.17024	0.004922	0.014531
11736985_NUBPL	-0.17792	0.004923	0.014533
11744805_DHRS4 ///	-0.18546	0.004924	0.014534
11728037_CMTM5	-0.22523	0.004924	0.014534
11748056_SOX15	0.352698	0.004925	0.014537
11735098_MLX	0.285564	0.004926	0.014538
11727048_PPP6C	0.190725	0.004927	0.014542
11752153_NBEAL2	-0.18897	0.004931	0.014551
11723486_WDR20	-0.23222	0.004931	0.014552
11737958_EIF5A	-0.14588	0.004933	0.014556
11726444_SAMD13	0.178213	0.004933	0.014556
11747341_ARL6	0.188275	0.004937	0.014567
11756337_HNRNPC	-0.13337	0.004938	0.014567
11759024_PPP2R5E	-0.21303	0.004944	0.014584
11723903_PLEKHG1	0.224364	0.004949	0.014599
11752346_OS9	0.161361	0.004952	0.014606
11721154_STK3	-0.29412	0.004953	0.014609
11736658_MAP3K13	0.410002	0.004954	0.01461
11716367_PRR13	-0.13331	0.004956	0.014615
11757756_ASXL2	0.146159	0.004956	0.014615
11734121_LRP1B	0.321859	0.004958	0.01462

11719248_AIM1	0.184026	0.004959	0.014622
11757982_KIF21A	-0.21396	0.004959	0.014622
11716961_FAM3A	-0.17518	0.004964	0.014634
11731027_CREM	0.141449	0.004964	0.014635
11718327_WWC3	0.169121	0.004967	0.014641
11725266_COL4A4	-0.20139	0.004967	0.014642
11742208_VCAN	0.258575	0.004969	0.014647
11759187_TNFAIP8L1	-0.19917	0.00497	0.014649
11715514_HERPUD1	-0.14211	0.00497	0.014649
11725398_MLLT1	-0.2943	0.004974	0.014659
11726978_SUGP2	0.187347	0.004976	0.014665
11726813_KDM5D	-0.15334	0.00498	0.014675
11723811_STX17	0.181954	0.004981	0.014676
11725772_MIER3	0.223172	0.004985	0.014687
11716113_STAMBP	0.157722	0.004994	0.014712
11755797_ADAM9	0.199535	0.004995	0.014714
11757488_POP7	-0.17043	0.004996	0.014716
11730593_SLC16A12	0.25413	0.004996	0.014718
11717269_FPGS	-0.16147	0.004998	0.014721
11717659_CD109	0.171007	0.004999	0.014723
11736286_CHKA	-0.26882	0.005	0.014725
11716142_SIGMAR1	-0.18611	0.005003	0.014733
11762490_CAPS2	-0.21786	0.005003	0.014733
11732230_NEUROD6	-0.24546	0.005004	0.014733
11749325_OLFML2B	-0.14219	0.005006	0.01474
11746204_NFASC	-0.22479	0.005007	0.014742
11757361_PSMA2	-0.19572	0.005011	0.014753
11719148_ARRDC2	-0.24053	0.005015	0.014763
11745246_SEPT7	0.137955	0.005018	0.01477
11744062_ACTR1B	-0.1357	0.00502	0.014775
11716861_TMEM184B	-0.15921	0.005021	0.014777
11737719_GAGE10	-0.16111	0.005021	0.014778
11749257_HERPUD1	-0.13769	0.005022	0.014779
11746446_DUOXA1	-0.16665	0.005023	0.014781
11719570_RCHY1	0.170574	0.005027	0.014794
11726206_SH2D5	0.209267	0.005029	0.014798
11751838_DMRT1	-0.14459	0.005032	0.014806
11754164_BCL2L15	0.151659	0.005035	0.014813
11755246_GREB1	0.367152	0.005036	0.014816
11746325_HLF	-0.19565	0.005038	0.014819
11734493_KCP	-0.16429	0.005038	0.01482
11739823_CPNE8	0.134475	0.005039	0.014823
11755086_XIAP	0.151134	0.00504	0.014823
11751693_KCNK4	-0.22547	0.005043	0.014832
11761209_MESDC2	0.155495	0.005043	0.014832
11748992_PFAS	-0.2362	0.005045	0.014836
11728857_CEP19	0.294052	0.005049	0.014846
11758570_CDADC1	0.167204	0.005052	0.014853
11725505_GDF11	-0.2071	0.005052	0.014854
11758998_STIM2	-0.15417	0.005055	0.01486
11758148_B2M	0.133632	0.005055	0.01486

11758192_B2M	0.133632	0.005055	0.01486
11762669_MTIF3	0.164926	0.005061	0.014876
11755603_ARL8B	0.141047	0.005062	0.014879
11717852_NAGLU	0.142647	0.005064	0.014884
11753716_AP4S1	0.2246	0.005068	0.014894
11717221_PPP3CB	-0.19176	0.005068	0.014894
11754507_ZCCHC11	-0.23966	0.00507	0.014897
11736709_LIMCH1	-0.26326	0.005073	0.014906
11756381_TRMT112	-0.13359	0.005074	0.014907
11726582_PLPP7	-0.22974	0.005075	0.014909
11743117_XRN2	0.133565	0.005075	0.014909
11742937_ASL	-0.15098	0.005076	0.014911
11756645_CXCL16	0.178857	0.005078	0.014916
11729207_PTPN11	0.247586	0.005078	0.014916
11754789_SMYD2	-0.20817	0.00508	0.014921
11755269_MRPS24 //	-0.15787	0.005081	0.014924
11743954_EHBP1L1	-0.16368	0.005082	0.014926
11723436_CEP41	-0.15704	0.005084	0.014928
11737147_CLEC7A	0.306598	0.005087	0.014937
11753599_FHL1	0.181116	0.005087	0.014937
11725985_NONO	-0.13847	0.005088	0.014937
11754159_CD28	0.187607	0.00509	0.014942
11723460_NLE1	-0.14827	0.005093	0.01495
11747918_UBAP2	0.225245	0.005104	0.014984
11741777_PAOX	-0.20612	0.005106	0.014986
11726680_HNRNPK	-0.2014	0.005106	0.014986
11737294_TMIGD1	-0.28797	0.005106	0.014987
11720406_PPP6R2	0.158475	0.005107	0.014987
11722512_SP110	-0.17821	0.005109	0.014994
11763576_LSM14B	-0.33825	0.00511	0.014995
11730803_PRPF38B	0.159182	0.005111	0.014997
11747624_SNX17	0.145725	0.005112	0.014998
11757769_VCL	0.149929	0.005114	0.015002
11724283_ANXA3	0.359217	0.005114	0.015002
11750822_SHQ1	0.165147	0.005119	0.015016
11721132_HCFC1	-0.14757	0.005119	0.015016
11716191_VWA9	-0.17495	0.005119	0.015016
11725832_OTUB2	-0.21891	0.005122	0.015022
11720215_PIGU	-0.16516	0.005123	0.015026
11741474_FOXN1	-0.29078	0.005127	0.015035
11764013_LOC105375	-0.14953	0.005128	0.015037
11720950_PFDN4	-0.13893	0.005129	0.015041
11726208_C19orf44	0.281622	0.005134	0.015053
11761177_DLST	-0.19882	0.005135	0.015055
11742742_SNX2	0.175989	0.005136	0.015057
11719304_EMC8	0.175534	0.005143	0.015078
11733600_NPAS4	-0.22749	0.005144	0.01508
11715469_TXN	-0.1629	0.005146	0.015083
11749864_RHOJ	0.249843	0.005148	0.015089
11726245_HIST1H4C	-0.13759	0.005149	0.015091
11741234_CASC1	-0.15928	0.005151	0.015097

11738686_OR1L3	-0.21382	0.005155	0.015108
11719530_KLF16	-0.15853	0.005161	0.015123
11747689_NFATC2IP	0.146905	0.005162	0.015125
11726861_DCAKD	-0.14978	0.005162	0.015125
11749122_MAP7D3	-0.21492	0.005163	0.015125
11763635_UBE2I	-0.25453	0.005166	0.015135
11761589_HNRNPD	-0.16285	0.005166	0.015135
11726714_DDX59	-0.17142	0.005167	0.015136
11718097_MEF2A	-0.13641	0.005169	0.015142
11723231_RNF138 //	0.170241	0.00517	0.015142
11724703_ELF5	-0.1828	0.005175	0.015155
11717287_POGZ	-0.16273	0.005175	0.015156
11727814_UBASH3B	0.165275	0.005178	0.015164
11725086_LANCL2	-0.13261	0.005179	0.015166
11719513_ADAM15	-0.20768	0.005182	0.015172
11742352_OR2Y1	-0.14466	0.005183	0.015174
11756172_TBC1D22A	-0.14564	0.005183	0.015174
11753406_EPHB4	-0.17661	0.005185	0.015179
11729866_UGT3A1	-0.17854	0.005186	0.015181
11752363_SBNO1	0.182965	0.005188	0.015186
11743344_RMND5A	-0.20043	0.005192	0.015198
11723615_DYRK1A	0.417054	0.005192	0.015198
11759414_GCSH	-0.26888	0.005193	0.015198
11763264_PCYT1A	0.225486	0.005194	0.015201
11743852_FBXO21	-0.2246	0.005195	0.015201
11751243_OTUD7B	-0.18201	0.005198	0.015211
11756883_PRRC2C	0.132798	0.005198	0.015211
11746986_HSPD1	-0.14845	0.0052	0.015216
11753544_RAD52	0.420998	0.005201	0.015216
11759768_FSBP ///	0.202359	0.005201	0.015216
11763502_LOC100287	-0.25288	0.005202	0.015216
11754246_PABPC1	0.140103	0.005202	0.015216
11721627_SEPT6	0.232886	0.005203	0.015218
11735046_ATP6V0D2	-0.1764	0.005206	0.015225
11727417_ATP5SL	-0.142	0.005206	0.015225
11731531_DOK1	-0.16462	0.005208	0.015231
11722177_MAN2C1	0.177751	0.005209	0.015231
11747023_RPS6KC1	-0.13653	0.005211	0.015236
11732950_AHI1	0.252928	0.005213	0.015243
11759313_AGO4	0.180335	0.005214	0.015243
11747441_GLRX	0.166066	0.005214	0.015243
11756917_ZNF618	-0.20767	0.005215	0.015246
11735387_ZNF560	0.428105	0.005216	0.015248
11755474_ADAM15	-0.16798	0.005216	0.015248
11738150_AVIL	0.420687	0.005217	0.015249
11750836_EFHC1	0.24985	0.005219	0.015254
11727623_EID3	0.30719	0.005221	0.015258
11731402_TMCO1	-0.57989	0.005225	0.015268
11758633_PDCD5	-0.17072	0.005225	0.015268
11735705_BSG	-0.16645	0.005225	0.015269
11741579_SLC30A2	-0.25784	0.005226	0.015269

11763012_CCL4L1 //	-0.22537	0.005228	0.015274
11758404_BCLAF1	-0.49475	0.005229	0.015277
11763434_CAPN10	-0.21168	0.005235	0.015294
11742874_CINP	-0.17295	0.005238	0.015299
11757120_EIF4A2 //	-0.35565	0.005239	0.015302
11754200_CREBBP	0.232925	0.005239	0.015303
11747451_ZNF76	-0.20405	0.00524	0.015304
11726997_FIBP	-0.15976	0.005241	0.015307
11715431_TMBIM1	-0.24609	0.005242	0.015308
11737601_DIO1	0.130548	0.005244	0.015312
11723050_HSPA6	-0.1499	0.005245	0.015315
11724018_LDHC	-0.23582	0.005247	0.01532
11725963_LONP1	-0.15086	0.005249	0.015326
11719227_KCNN4	0.180118	0.005253	0.015337
11745345_PYCR2	-0.20371	0.005254	0.015338
11731155_GLYAT	-0.19388	0.005258	0.015348
11716504_CRMP1	-0.19499	0.00526	0.015354
11729139_SLAMF7	0.336195	0.005265	0.015367
11733584_TM4SF19 /	-0.25431	0.005267	0.01537
11718230_HLA-F	0.295737	0.00527	0.015379
11725853_XRCC4	-0.16987	0.005271	0.015382
11757461_BTBD3	-0.14239	0.005272	0.015385
11748260_ELP3	0.153431	0.005274	0.015387
11753176_GTPBP3	-0.21067	0.005274	0.015387
11719034_FTSJ3	0.140382	0.005274	0.015388
11755574_GALK1	-0.17678	0.005275	0.015388
11719541_PAXBP1	0.178545	0.005275	0.015388
11726645_CDHR5	-0.21158	0.005278	0.015395
11746892_PPWD1	-0.14584	0.005279	0.015396
11763609_DRG1	0.143054	0.005289	0.015425
11723104_GNB4	-0.13912	0.005291	0.015429
11737973_RAB39A	0.236887	0.005291	0.015429
11760006_GNB5	-0.1893	0.005294	0.015436
11747254_POLK	0.136005	0.005294	0.015436
11743588_BECN1	0.130938	0.005294	0.015437
11734782_WDR92	-0.2008	0.005299	0.01545
11718987_LARS2	-0.16343	0.005307	0.015472
11754329_TANC2	-0.21252	0.005308	0.015472
11733180_ETV1	-0.14253	0.005309	0.015476
11716467_ISCA1	-0.15584	0.005312	0.015484
11759480_PTP4A2	-0.13676	0.005312	0.015484
11724663_TMEM120B	-0.14933	0.005314	0.015489
11758778_LOC102724	-0.13935	0.005315	0.01549
11747236_COG4	0.139846	0.005316	0.015492
11720419_NCKIPSD	-0.17142	0.005317	0.015494
11718909_CHST2	-0.13918	0.005318	0.015495
11725297_MAPRE2	0.132473	0.005319	0.015497
11735152_SLC8A1	-0.32567	0.005322	0.015506
11739875_BSG	-0.13489	0.005324	0.015509
11720210_CHID1	-0.17925	0.005324	0.01551
11719212_LOC100996	0.159489	0.005325	0.015511

11723403_PPARGC1A	0.691622	0.005325	0.015511
11738811_INPP4A	0.128808	0.005327	0.015516
11733357_CCDC185	-0.18835	0.005328	0.015518
11748807_RDH11	-0.17983	0.005329	0.015518
11727589_IL20RB	0.348137	0.005329	0.015518
11745999_ZNF252P	0.252885	0.00533	0.01552
11738402_WDPCP	0.301078	0.005331	0.015523
11716137_COX7A2 //	0.144219	0.005333	0.015526
11755019_SCUBE3	-0.25377	0.005333	0.015527
11750538_TXN	-0.12715	0.005334	0.015529
11756912_AOX1	0.223369	0.005336	0.015532
11717950_SC5D	0.170469	0.005337	0.015536
11763724_ZNF668	0.189731	0.005338	0.015537
11715444_CPB1	0.173356	0.005345	0.015558
11726800_EBF1	0.192481	0.005349	0.015566
11728843_PROSER2	0.151517	0.005349	0.015568
11760192_TMEM68	0.205069	0.005352	0.015573
11723872_ADGRG1	0.203388	0.005353	0.015576
11761914_MR1	0.329341	0.005355	0.01558
11751229_PPP2R2D	0.135577	0.005359	0.015591
11721708_GPAM	-0.20747	0.00536	0.015593
11736971_MKRN3	0.30985	0.005363	0.015601
11751262_MBOAT1	-0.15799	0.005364	0.015602
11728356_BBS10	0.172518	0.005365	0.015606
11754446_ZNF761	0.25243	0.005366	0.015606
11725643_MRPS26	-0.14258	0.005366	0.015607
11750109_MYO18A	-0.24411	0.005372	0.015622
11738247_TNNT1	-0.25816	0.005372	0.015623
11718141_DKK1	0.156551	0.005375	0.015629
11755964_NRXN1	0.216426	0.005378	0.015636
11747637_ST5	-0.16134	0.00538	0.015641
11745859_ATP5SL	-0.14908	0.00538	0.015642
11745799_ETV1	-0.14111	0.00538	0.015642
11743459_HDLBP	-0.14874	0.005381	0.015642
11763323_GRAMD1B	0.308791	0.005387	0.015659
11757032_DNAJB12	-0.16943	0.005391	0.015669
11722840_LYAR	-0.1421	0.005394	0.015679
11758381_C22orf46	-0.25232	0.005396	0.015683
11721717_ATRX	0.157818	0.005397	0.015683
11735927_PRDM7	0.187323	0.005397	0.015683
11736124_CPLX2	-0.2243	0.005399	0.015688
11758030_FOXG1	-0.40615	0.005399	0.015689
11755430_C19orf25	-0.27629	0.0054	0.015689
11731489_NUP43	-0.14328	0.005401	0.015691
11720314_ATP6V0B	0.138993	0.005402	0.015693
11746901_PPEF2	0.269985	0.005403	0.015694
11727715_ITGA10	-0.1871	0.005403	0.015694
11762205_ZNF718	-0.2546	0.005404	0.015696
11739165_PYCRL	-0.18953	0.005405	0.015699
11716941_TRIB2	0.155609	0.005408	0.015706
11745778_GABRE	-0.1465	0.005408	0.015707

11754942_C17orf62	-0.15211	0.00541	0.015709
11756016_CLN3	-0.14235	0.00541	0.015709
11725555_TMEM133	0.218839	0.00541	0.015709
11756200_CHI3L2	-0.18078	0.005413	0.015714
11744143_EIF3H	0.138548	0.005413	0.015714
11730867_MEP1A	-0.1822	0.005414	0.015718
11747530_CSNK2B	0.159261	0.005416	0.015723
11718262_ERF	-0.22783	0.005417	0.015723
11755509_ARHGEF9	-0.13908	0.005418	0.015726
11742601_OR6C75	-0.22864	0.005418	0.015726
11761316_DCP1B	0.222179	0.005421	0.015733
11746428_PAQR3	0.175097	0.005424	0.015739
11762874_PCSK9	-0.1762	0.005424	0.015739
11730941_FAM71A	-0.3823	0.005427	0.015747
11762322_APOL4	-0.1995	0.005429	0.015753
11729596_TKFC	-0.18651	0.005431	0.015757
11732040_SSX4 ///	-0.27381	0.005435	0.015767
11740963_UBA5	0.155531	0.005436	0.015769
11746687_ZSCAN1	-0.16662	0.005437	0.015772
11754460_RGMA	-0.29166	0.005437	0.015772
11755015_CPS1	0.198278	0.005439	0.015775
11750880_METTL16	-0.24278	0.00544	0.015779
11763642_FMNL1	-0.23084	0.005442	0.015783
11721062_ARFGEF2	-0.15049	0.005443	0.015785
11761896_POU5F1P3	-0.16412	0.005448	0.015797
11749060_LSP1	-0.17926	0.005448	0.015797
11715989_JTB	0.136165	0.005449	0.015798
11724754_ALG2	0.267052	0.00545	0.015802
11761014_PKMYT1	-0.34017	0.005464	0.01584
11736215_CASP6	0.163385	0.005464	0.01584
11735201_N4BP2	0.298029	0.005464	0.01584
11749660_MOV10	0.169265	0.005467	0.015846
11745071_TCEAL4	-0.16618	0.00547	0.015852
11763314_ARL4C	0.221981	0.00547	0.015852
11746022_TDGF1 ///	-0.23504	0.005471	0.015855
11754359_ZFYVE27	0.172189	0.005472	0.015858
11718478_STAR	-0.18313	0.005476	0.015869
11730510_EID2B	-0.45278	0.005478	0.015872
11739964_FDXACB1	-0.17737	0.005479	0.015873
11715758_TTC1	0.168891	0.005479	0.015873
11721194_RRP1B	-0.23903	0.005479	0.015874
11756068_BACE2	-0.16241	0.00548	0.015874
11725618_GAD1	0.42624	0.00548	0.015875
11723188_ADAM19	-0.17856	0.005481	0.015876
11760753_PDGFRA	0.365475	0.005482	0.015877
11758851_EMC1	-0.25744	0.005482	0.015877
11718314_LRP11	-0.14841	0.005483	0.015879
11763588_NSD1	0.190204	0.005484	0.015881
11742786_SETX	0.215618	0.005485	0.015884
11757470_TCF25	-0.1638	0.005488	0.01589
11746681_VNN3	-0.21852	0.00549	0.015896

11715646_ADIPOR1	0.147081	0.005491	0.015897
11748438_FANCA	-0.22616	0.005495	0.015909
11754880_DOCK9	0.165628	0.005497	0.015914
11735893_MBD3L2 //	-0.20457	0.005498	0.015915
11717195_RAB5B	0.136939	0.005498	0.015915
11740975_TP53I11	-0.20325	0.005505	0.015931
11754722_PI4K2B	-0.14748	0.005505	0.015931
11763525_COL12A1	0.373082	0.005509	0.015943
11762350_ZNF28	-0.14409	0.005514	0.015956
11752672_NARFL	-0.13934	0.005514	0.015956
11744097_KAT2A	0.146934	0.005515	0.015956
11743688_GLI2	0.164312	0.005515	0.015956
11748695_SYNGR1	-0.16115	0.005521	0.015974
11751296_EPHA1	-0.1698	0.005522	0.015976
11728988_FAHD2B	-0.28654	0.005526	0.015986
11733396_FLG2	0.228712	0.005532	0.016001
11742607_OR4S2	-0.22618	0.005533	0.016004
11731930_OPALIN	-0.1792	0.005534	0.016004
11716443_RNF181	0.133151	0.005534	0.016005
11750339_GGNBP1	-0.2055	0.005536	0.016009
11732404_NKRF	0.193857	0.005539	0.016018
11715979_BRK1	0.154884	0.005546	0.016036
11736578_AP1G2	0.16824	0.005548	0.01604
11748028_MBD3	-0.24181	0.005548	0.01604
11756179_SEC13	-0.14208	0.005549	0.016042
11731632_MAP1LC3B	0.160738	0.005549	0.016042
11715658_SLC39A1	-0.20494	0.005552	0.016049
11720729_CSDC2	-0.1644	0.005554	0.016055
11716000_FAF2	0.178871	0.005559	0.016066
11741276_TMEM144	-0.30909	0.00556	0.016068
11754365_ROR2	-0.22894	0.005561	0.016072
11732942_LOC101929	-0.19916	0.005562	0.016072
11717768_PDE6G	-0.16529	0.005562	0.016072
11732783_RGSL1	-0.15539	0.005565	0.016079
11716768_UBQLN2	-0.17089	0.00557	0.016092
11751632_TFAP4	-0.16863	0.005574	0.016104
11757541_TXNDC17	0.137042	0.005576	0.016108
11721181_VLDLR	-0.1454	0.005576	0.016109
11753897_SSB	-0.23723	0.005579	0.016116
11745075_GSTA4	-0.14702	0.00558	0.016118
11750891_UBE3C	0.323393	0.005582	0.016124
11759149_FBXO3	-0.42982	0.005583	0.016125
11748057_SOX15	0.222082	0.005586	0.016131
11725811_PLA2G5	-0.24557	0.005587	0.016133
11742247_EGFR	0.175588	0.005587	0.016133
11760995_WRB	-0.38168	0.005589	0.016137
11754956_SNHG7	-0.16207	0.00559	0.016141
11733418_RET	-0.12827	0.005595	0.016153
11763240_TRAV12-2	-0.33126	0.005596	0.016155
11724325_RABGAP1L	0.300493	0.0056	0.016164
11745836_DYSF	0.314238	0.0056	0.016164

11729398_FBXL20	-0.13514	0.005602	0.016171
11746371_AASDHPPT	0.148748	0.005603	0.016172
11733762_ADAMTSL1	0.216208	0.005606	0.016178
11719545_ZFAND2B	-0.19573	0.005607	0.016181
11749803_ARL8B	0.14673	0.005608	0.016183
11722865_NETO2	0.150107	0.005609	0.016185
11715818_ZFP36L2	0.183994	0.00561	0.016187
11758618_SOX11	-0.16475	0.005611	0.01619
11761845_ANXA4	0.183139	0.005613	0.016195
11756327_RPS19	0.144754	0.005619	0.016211
11753775_TXN	-0.15599	0.005623	0.016221
11719268_TNNC1	0.366395	0.005626	0.016227
11731644_LOC102724	0.216954	0.005628	0.016232
11746906_CARD8	0.249669	0.005629	0.016234
11723525_TBC1D8	0.136779	0.00563	0.016236
11716146_PRKACA	-0.16148	0.00563	0.016236
11718295_SEMA4B	-0.25393	0.005638	0.016257
11722478_CUL5	0.23483	0.005638	0.016257
11723268_KPNA6	-0.13491	0.005642	0.016267
11733410_MPND	-0.17935	0.005646	0.016277
11717080_VPS52	-0.14476	0.005647	0.01628
11741587_ERLIN2	-0.15043	0.005648	0.016282
11718813_PVRL1	-0.24334	0.005651	0.016291
11737531_CCDC144NL	-0.23143	0.005654	0.016298
11759308_MAGI1	0.270618	0.005654	0.016298
11731250_EPHA8	-0.13525	0.005655	0.016299
11746070_UGT8	-0.19227	0.005658	0.016307
11759750_ZNF880	0.255669	0.005659	0.016309
11759002_CACNA1E	-0.19144	0.005659	0.016309
11719832_KMT5A	0.130415	0.005661	0.016312
11762093_CFHR3	0.369782	0.005662	0.016315
11717485_MYDGF	-0.1884	0.005663	0.016316
11736125_CPLX2	-0.14469	0.005663	0.016316
11720969_GART	-0.13352	0.005665	0.016319
11715648_ADIPOR1	0.156113	0.005668	0.016328
11751246_MET	0.167958	0.005669	0.016329
11759728_SEC24A	0.28052	0.005669	0.016329
11720939_DDX54	-0.15374	0.005673	0.016339
11757134_SNORA65	-0.23891	0.005674	0.016341
11723023_BCKDHB	-0.17145	0.005676	0.016346
11749823_SLC6A9	-0.13761	0.005678	0.016349
11746653_SETD6	-0.13537	0.005685	0.016369
11733343_UTP6	0.159497	0.005685	0.016369
11717640_UBE3A	0.149956	0.005685	0.016369
11725101_ESF1	0.130996	0.005687	0.016371
11755950_CCDC71	-0.16041	0.005687	0.016372
11718049_GLRX	0.146965	0.005691	0.016383
11762617_ANKRD36	-0.26464	0.005693	0.016386
11730829_AMPD2	-0.14119	0.005693	0.016387
11737282_ZC3HAV1L	0.48978	0.005695	0.01639
11724176_TSPAN11	-0.21625	0.005695	0.01639

11763345_C1orf174	-0.14792	0.005696	0.01639
11742796_HNRNPK	-0.32839	0.005696	0.01639
11744517_SLC2A10	-0.29643	0.005704	0.016414
11748755_MEIS2	-0.17605	0.005705	0.016415
11734614_GABRE	-0.16419	0.005708	0.016423
11725661_DPP8	0.180005	0.005709	0.016423
11731859_ZNF720	-0.15869	0.005709	0.016423
11725666_HS2ST1	0.331464	0.005709	0.016423
11732406_PRPH2	-0.25895	0.005711	0.016426
11729802_EIF1AD	0.144026	0.005711	0.016426
11751918_BNC1	0.28041	0.005711	0.016426
11761702_ADAM33	-0.16771	0.005713	0.016429
11724497_NPRL3	-0.14776	0.005714	0.016431
11727748_TMEM63C	-0.17197	0.005715	0.016435
11736395_BPGM	-0.16229	0.005717	0.016439
11741030_PTCD2	0.180388	0.005719	0.016444
11753157_LMAN2L	0.172036	0.005727	0.016466
11742951_MED10	0.166067	0.00573	0.016474
11751587_UCHL5	-0.19449	0.005732	0.016479
11729332_ZNF879	0.35974	0.005735	0.016485
11723591_LRRC8C	0.151616	0.00574	0.016499
11747041_CCT4	-0.14023	0.00574	0.016499
11757771_ABCC8	-0.19935	0.005745	0.01651
11735120_SREBF1	-0.25087	0.005745	0.016511
11747870_MLLT3	0.17267	0.005747	0.016516
11745228_TMEM144	-0.3155	0.005753	0.01653
11763196_DYM	-0.26269	0.005756	0.016538
11763197_DYM	-0.26269	0.005756	0.016538
11752204_AP3D1	-0.14419	0.005758	0.016542
11751542_ALDH3B2	-0.25839	0.005759	0.016545
11718826_ITM2C	-0.19233	0.005764	0.016558
11757605_EPB41L4B	-0.29671	0.005764	0.016558
11727029_MAP1A	-0.13168	0.005765	0.016559
11745119_DST	0.159784	0.005768	0.016566
11743162_CTSE	0.471189	0.00577	0.016571
11725531_DSC2	0.18441	0.00577	0.016572
11719126_CGRRF1	0.226881	0.005773	0.016577
11759662_MORN4	-0.19587	0.005774	0.016579
11758455_THAP6	0.308851	0.005774	0.01658
11719645_GATAD2A	-0.22058	0.005778	0.016591
11728866_NMRK2	-0.22401	0.005779	0.016591
11718568_WIPF1	0.285899	0.00578	0.016593
11747723_DAXX	0.174813	0.00578	0.016593
11728977_SRRM3	-0.21321	0.005783	0.016598
11744852_KIAA0125	-0.14163	0.005783	0.016598
11743573_TMEM184C	0.195882	0.005785	0.016603
11738813_LEPR	0.210151	0.005786	0.016605
11749863_TBP	0.206779	0.005787	0.016607
11732732_DNAH8	-0.15599	0.005787	0.016608
11742819_PLAUR	-0.13411	0.005788	0.016608
11720923_HCP5	-0.12951	0.005789	0.016609

11738574_TECTB	-0.19607	0.00579	0.016613
11749443_PYCRL	-0.17095	0.005793	0.016619
11728421_PLA2G7	-0.17583	0.005793	0.016619
11744150_LPIN2	-0.14761	0.005794	0.01662
11718116_METTL13	-0.13097	0.005796	0.016625
11743081_TAF3	0.236538	0.005796	0.016625
11760242_SRSF5	-0.19208	0.005796	0.016625
11724793_PGS1	0.148527	0.005798	0.016628
11719421_ABCC1	0.2002	0.005801	0.016635
11729023_KCNA5	-0.19198	0.005804	0.016643
11751261_ITPK1	-0.15792	0.005807	0.016651
11720442_INAFM1	-0.26251	0.00581	0.016658
11720901_GRAMD1C	-0.25821	0.005813	0.016666
11744724_CERS2	-0.13323	0.005813	0.016667
11730242_PRKAG1	-0.18067	0.005814	0.016667
11723544_ESRP1	0.297543	0.005814	0.016667
11723148_SLC19A2	0.244662	0.005815	0.016669
11718698_C6orf89	0.198586	0.005816	0.01667
11755316_TMEM139	0.146433	0.005817	0.016671
11719189_NRAS	-0.1364	0.005822	0.016685
11719285_PEX11B	-0.24192	0.005823	0.016686
11742577_OR6C6	-0.19779	0.005823	0.016686
11718541_MTSS1	-0.27976	0.005823	0.016686
11760773_HLA-DRA	-0.28541	0.005826	0.016692
11735085_FREM2	-0.53591	0.00583	0.016705
11739035_ACTR2	-0.15264	0.005831	0.016707
11727603_BMP5	0.195257	0.005835	0.016717
11720565_NAV2	0.256628	0.005836	0.016717
11756815_PCDH17	0.242761	0.005837	0.016719
11715582_SF3B2	-0.1656	0.005838	0.016721
11745344_PYCR2	-0.18049	0.005839	0.016723
11744061_ACTR1B	-0.14628	0.005841	0.016728
11716691_NPEPPS	0.147009	0.005845	0.016739
11761522_CHURC1 //	-0.16413	0.005846	0.016741
11745160_TGFA	0.178617	0.005848	0.016746
11727231_ZNF19	0.281277	0.005851	0.016752
11761875_ROCK1P1	0.21003	0.005852	0.016756
11726179_SOX6	-0.25658	0.005854	0.01676
11730428_AP1B1	-0.16786	0.005855	0.016761
11726860_DCAKD	-0.22311	0.005855	0.016761
11731116_PLEKHA8P1	-0.4052	0.005857	0.016765
11727841_PRKCD	0.143363	0.005861	0.016775
11736553_TGFB2	-0.20997	0.005862	0.016778
11717459_MRPL39	-0.12792	0.005865	0.016784
11715617_RNF130	-0.14639	0.005866	0.016786
11756859_UBE2D2	-0.13013	0.00587	0.016797
11720879_CABIN1	-0.20664	0.005872	0.016801
11759177_MAN1A1	-0.25058	0.005872	0.016802
11759564_RPS2 ///	-0.38303	0.005874	0.016805
11757184_SC5D	0.153591	0.005874	0.016806
11753914_SSX2IP	-0.16708	0.005875	0.016806

11751332_CRLF3	0.176188	0.005875	0.016806
11742771_KIAA0100	-0.14854	0.00588	0.016819
11755827_SMARCD1	-0.12574	0.005881	0.016822
11721451_ETF1	0.160686	0.005883	0.016824
11755349_GDPD3	-0.27683	0.005891	0.016847
11723833_TAPT1	0.334767	0.005892	0.01685
11741450_P2RX5 ///	0.145845	0.005893	0.016851
11749848_CTNS	-0.138	0.005894	0.016853
11754910_MICALL2	0.156382	0.005895	0.016855
11750143_PIGZ	-0.20515	0.005897	0.016859
11753956_C16orf13	-0.13941	0.005897	0.016859
11723764_MCM3AP	0.200842	0.005897	0.016859
11723463_C14orf80	-0.28484	0.005898	0.016859
11759382_GRK7	-0.28162	0.005898	0.016859
11721817_SH3BP5L	-0.1433	0.0059	0.016864
11736441_UROC1	-0.17462	0.005901	0.016864
11756247_ATXN2	0.150298	0.005901	0.016866
11744355_SUN2	-0.19457	0.005904	0.016871
11735131_DNAJC21	0.208071	0.005905	0.016873
11748262_PMS1	-0.22244	0.005905	0.016873
11756022_RHOJ	0.29832	0.005908	0.016879
11720234_RHOBTB2	-0.15671	0.005908	0.01688
11737295_KIAA1328	-0.19871	0.005912	0.016889
11757096_ZNF492 //	-0.24498	0.005912	0.01689
11744256_TUBB4A	-0.2518	0.005915	0.016896
11721222_MIER2	0.151342	0.005915	0.016896
11757370_POLR2J	-0.17425	0.005916	0.016897
11729760_PLA2G2D	-0.25317	0.005918	0.016901
11725960_CALM2 ///	-0.13033	0.005922	0.016912
11758018_UBR3	0.230374	0.005923	0.016913
11752583_CD6	-0.25677	0.005926	0.016922
11715228_SLC51B	-0.21462	0.00593	0.016931
11761250_ARL5A	0.130966	0.00593	0.016931
11732044_FBLN1	-0.28414	0.005938	0.016952
11738934_GSX1	-0.31084	0.005938	0.016952
11759914_GANC	-0.24541	0.005942	0.016963
11725373_TRADD	-0.1776	0.005943	0.016963
11717714_CHST10	-0.21936	0.005944	0.016966
11719679_EPN2	-0.19638	0.005949	0.016979
11744523_NBAS	0.185426	0.005951	0.016985
11735743_ARL14	0.683864	0.005953	0.01699
11738264_ZNF679	0.201726	0.005954	0.016993
11757435_BIN1	0.21012	0.005959	0.017004
11757467_TSPAN6	0.146115	0.005961	0.017009
11751439_NECAP1	0.168882	0.005961	0.017009
11748420_ATXN7	0.28928	0.005963	0.017013
11750014_SSBP1	-0.14537	0.005968	0.017026
11717223_EXTL3	-0.19128	0.00597	0.01703
11723816_ARHGAP29	-0.15741	0.005972	0.017036
11748392_GAL3ST4	-0.15335	0.005975	0.017042
11734033_C15orf61	-0.14381	0.005975	0.017042

11747888_SH3YL1	0.327131	0.005976	0.017044
11760801_SPHK2	-0.19614	0.005979	0.017051
11755885_UBXN7	-0.21663	0.005983	0.017061
11716973_NSF	0.174364	0.005985	0.017066
11754924_ADTRP	0.311422	0.005985	0.017066
11759317_EXOC6B	0.18184	0.005989	0.017076
11722749_HOOK3	-0.17162	0.00599	0.017078
11749996_VSNL1	-0.30018	0.005995	0.017092
11735077_CYP2C18	-0.22499	0.005997	0.017095
11736811_PDCD1LG2	0.197837	0.005999	0.017101
11716895_ISG15	0.209806	0.006	0.017101
11760165_AP1S2	0.167296	0.006002	0.017106
11731467_CYP2E1	0.251287	0.006002	0.017106
11723658_ZNF668	0.257506	0.006005	0.017114
11717762_CPVL	-0.16678	0.006006	0.017114
11716507_CUL3	-0.20844	0.006008	0.017119
11736336_RBM19	0.203103	0.00601	0.017125
11731879_NLRP1	0.245333	0.006012	0.017129
11748559_DBNL	-0.14042	0.006013	0.017132
11735757_GATSL2	-0.25211	0.006015	0.017135
11716889_TMEM261	-0.27518	0.006017	0.01714
11735660_AGBL4	-0.13582	0.006017	0.017141
11725726_C1orf56	-0.16849	0.006018	0.017142
11741879_PML	-0.12886	0.006021	0.017149
11724767_PMM1	-0.15364	0.006023	0.017153
11725050_FAIM2	-0.19199	0.006023	0.017153
11727629_APBA2	-0.20504	0.006032	0.017178
11761027_FCRL5	-0.18388	0.006035	0.017184
11743089_LRRC6	0.306055	0.006038	0.017193
11758578_AEBP2	0.160467	0.006038	0.017193
11716189_SEC13	-0.1266	0.00604	0.017197
11725615_PPP1CB	0.153502	0.006043	0.017204
11728273_GINM1	0.152719	0.006046	0.017212
11732268_KLHL40	-0.27003	0.006047	0.017213
11737056_ACTR3B	0.153618	0.006048	0.017216
11719394_FBXO32	0.201501	0.006054	0.017231
11754412_CLUAP1	0.150358	0.006056	0.017235
11744225_HERC1	0.218704	0.006056	0.017235
11762235_KIAA0907	0.230953	0.006058	0.017238
11726666_MFSD8	0.209304	0.006062	0.017251
11743524_KDM5A	0.285683	0.006065	0.017256
11755609_HYDIN	-0.14635	0.006065	0.017257
11756459_SLC22A18	-0.21	0.006071	0.017271
11718948_MPP5	0.190085	0.006072	0.017274
11721752_FZD7	-0.14091	0.006074	0.017277
11741478_SPATA1	0.271847	0.006074	0.017277
11754606_HSP90B1	-0.16895	0.006085	0.01731
11730007_RETN	-0.13447	0.006087	0.017312
11721287_WWOX	0.270034	0.006088	0.017314
11734798_RLIM	0.132794	0.00609	0.01732
11761288_RNLS	-0.20131	0.006093	0.017326

11762589_RALGPS1	-0.19149	0.006099	0.017341
11735795_GPSM1	-0.16703	0.006099	0.017341
11749787_SEPT2	0.138873	0.0061	0.017344
11743766_CUL4A	-0.14459	0.006102	0.017348
11720069_COMMD8	0.178167	0.006107	0.017363
11763700_FAAP20	-0.15414	0.006109	0.017367
11717437_TOP1	0.142442	0.00611	0.017369
11718860_SUPV3L1	0.146823	0.006111	0.017369
11721064_ELP4	-0.19319	0.006111	0.01737
11750017_STX3	0.156712	0.006117	0.017385
11758906_DNAJC5	-0.18857	0.006121	0.017397
11733014_RNF213	0.148061	0.006123	0.0174
11734067_IRAK4	-0.24344	0.006124	0.017403
11723424_IFNAR1	0.157991	0.006134	0.01743
11760693_SIGLEC9	-0.16896	0.006142	0.017452
11746004_LTBP1	-0.16914	0.006143	0.017453
11746717_KDM4C	0.193549	0.006143	0.017453
11741197_KIF5C	-0.41451	0.006147	0.017461
11745396_MYO5B	0.362398	0.006147	0.017461
11757419_UXT	-0.14342	0.006148	0.017464
11743750_FAM169A	-0.16569	0.006151	0.017471
11745572_DPY19L1 /	0.15828	0.006152	0.017472
11744447_HLCS	-0.22309	0.006153	0.017474
11720631_SDHB	-0.22163	0.006153	0.017474
11747072_TUBB6	0.133415	0.006156	0.01748
11763718_CDKN1C	0.26253	0.006156	0.01748
11756087_AMFR	0.177834	0.006157	0.017482
11761097_ARFGAP2	-0.22411	0.006159	0.017486
11737733_CAPS2	0.25173	0.006162	0.017493
11716757_DHX30	0.139949	0.006164	0.017499
11758772_HLA-DPB1	-0.16266	0.006166	0.017504
11730595_LYG1	0.168637	0.006167	0.017504
11743428_RUNX3	-0.19483	0.006167	0.017505
11752002_PLEKHG1	0.233437	0.006169	0.017508
11718448_SH3BGRL2	-0.45217	0.006176	0.017526
11736167_CCDC34	-0.1339	0.006176	0.017526
11715867_MRPL41	-0.13859	0.006184	0.017548
11728470_BTBD11	0.220747	0.006188	0.017559
11719515_ADNP2	0.168853	0.006189	0.017559
11716607_TCEB2	-0.13634	0.006189	0.017559
11724333_USP8	0.160453	0.006194	0.017572
11744045_OSGIN1	0.154541	0.006196	0.017576
11727977_SRGAP1	0.219238	0.006197	0.017579
11750137_PLCD4	0.14239	0.006202	0.017592
11740036_CARD8	-0.33835	0.006204	0.017598
11721392_LOXL3	-0.12649	0.006207	0.017605
11727008_SORT1	0.255003	0.006212	0.017618
11720011_JARID2	0.221429	0.006218	0.017633
11746519_NOC2L	-0.22002	0.006219	0.017636
11742834_FAM76A	-0.38566	0.00622	0.017637
11750791_NEK11	0.22193	0.00622	0.017637

11718715_B4GALT2	-0.14785	0.006221	0.017638
11746079_ADCY7	0.266112	0.006223	0.017641
11746972_ 1-Mar	0.428988	0.006232	0.017666
11731673_LCORL	-0.19777	0.006235	0.017674
11726579_PSME1	0.169241	0.006235	0.017674
11726833_HSPB2 ///	-0.17677	0.006239	0.017682
11753717_KDM7A	0.18832	0.006242	0.017692
11728216_BCLAF1	-0.15075	0.006246	0.017702
11752760_TMED7	0.135681	0.006247	0.017703
11759603_BCL2L13	-0.20368	0.006249	0.017708
11717186_MEF2D	-0.19444	0.00625	0.017708
11735569_ZAR1L	-0.24316	0.00625	0.017708
11750793_SLC6A9	-0.21475	0.00625	0.017708
11758321_GATSL2	-0.15618	0.006251	0.017709
11725972_HNRNPF	-0.15676	0.006252	0.017711
11720353_ETV5	-0.14754	0.006256	0.017721
11760436_DST	0.184925	0.006258	0.017726
11758905_TMED4	-0.15605	0.006259	0.017727
11733962_LGR5	-0.17325	0.006261	0.017733
11728100_NXPH3	-0.15884	0.006263	0.017737
11750361_SLAMF1	-0.15788	0.006272	0.017763
11725093_RFX5	0.136641	0.006276	0.017771
11727775_GNAI3	0.143428	0.006278	0.017777
11724537_ABCG2	-0.20516	0.006281	0.017783
11750328_WAPL	-0.16745	0.006282	0.017784
11753367_NUCKS1	-0.14306	0.006282	0.017784
11746015_MSTO1 ///	-0.1448	0.006285	0.017794
11737291_OXGR1	-0.19704	0.006291	0.01781
11727786_CACYBP	-0.1314	0.006294	0.017816
11726933_SOX12	-0.27538	0.0063	0.01783
11748199_TMEM189	0.162845	0.0063	0.017832
11719916_IL1B	0.141712	0.006302	0.017836
11726091_VPS53	0.148728	0.006305	0.017843
11744095_DDX24	0.130949	0.006309	0.017852
11740423_PRKDC	-0.13944	0.006309	0.017852
11729179_POLH	0.332254	0.006309	0.017852
11735706_BSG	-0.13722	0.00631	0.017853
11723012_CAPZA2	0.134555	0.00631	0.017853
11720122_GIGYF1	-0.17568	0.006321	0.01788
11758928_NLN	-0.16066	0.006323	0.017887
11751139_ZNF101	-0.20948	0.006327	0.017897
11756093_IGHG1 ///	-0.17903	0.006327	0.017897
11740395_FGFR2	-0.18488	0.00633	0.017904
11748618_CCDC80	0.355048	0.006336	0.017918
11742687_FCGRT	-0.20997	0.006342	0.017933
11732645_SYT6	-0.20015	0.006342	0.017933
11728577_C2CD4C	-0.14565	0.006343	0.017933
11725924_RASSF5	-0.24229	0.006343	0.017933
11722650_RGS12	-0.18289	0.006344	0.017936
11757208_C22orf34	-0.20159	0.006346	0.017941
11721445_ERBB3	-0.15721	0.006349	0.017947

11741443_RBMX	-0.15063	0.00635	0.017952
11725626_ACE2	0.24985	0.006353	0.017958
11746887_NUDT16P1	-0.2354	0.006359	0.017975
11744497_FAM149B1	-0.20876	0.006361	0.017976
11753854_CD28	-0.15286	0.006361	0.017976
11754822_TDP1	-0.26913	0.006361	0.017976
11735903_RAET1E	0.201156	0.006361	0.017976
11746878_ID2	0.181223	0.006366	0.017988
11721331_RNGTT	0.152764	0.006368	0.017993
11733229_PTPRB	0.334814	0.00637	0.017998
11735117_VAMP4	-0.35077	0.006373	0.018004
11735256_RNF122	-0.16296	0.006376	0.018013
11729119_HSPA1A //	-0.12995	0.006382	0.018027
11746344_RANBP3	-0.23234	0.006383	0.018029
11745731_TNIP1	0.162847	0.006383	0.01803
11737845_FAM102A	-0.15123	0.006384	0.018032
11736987_NUBPL	0.209587	0.006387	0.018038
11743813_EVI2B	0.210579	0.006388	0.01804
11719581_BPGM	-0.15793	0.006389	0.01804
11725087_LANCL2	-0.21107	0.006389	0.01804
11744111_TMEM94	-0.15617	0.00639	0.018042
11717371_CHD1L	0.161249	0.00639	0.018043
11723648_DNAJA2	-0.1595	0.006392	0.018046
11745532_ZNF518A	0.26357	0.006396	0.018058
11752048_MYRIP	0.171572	0.006399	0.018065
11736979_FSIP1	0.227668	0.006401	0.018068
11748756_MEIS2	-0.1592	0.006402	0.018069
11725284_ZFP90	0.209306	0.006403	0.018071
11730183_SH3GL2	0.273431	0.006405	0.018077
11720482_POLM	0.161211	0.006409	0.018086
11738839_DYTN	-0.14655	0.006412	0.018095
11756398_VPRBP	-0.18344	0.006413	0.018097
11723250_EML2	-0.18593	0.006414	0.018099
11724138_PRSS21	-0.20117	0.00642	0.018114
11734568_MUTYH	-0.13898	0.006423	0.018121
11762876_CAPRIN2	-0.15524	0.006423	0.018121
11759701_SLC31A1	-0.14633	0.006426	0.018128
11751998_ZYG11A	0.214157	0.006431	0.01814
11717547_INTS12	0.197939	0.006434	0.018147
11717043_GID8	0.14843	0.006434	0.018147
11748265_LDLRAD3	-0.18224	0.00644	0.018163
11732517_SCRN2	-0.14956	0.006442	0.018168
11725801_USP46	0.164391	0.006445	0.018175
11726032_CSNK1G3	0.191261	0.006446	0.018176
11725062_USP7	-0.38193	0.006446	0.018177
11761153_CYP51A1-A	-0.15006	0.006449	0.018182
11747346_ACADM	0.138561	0.006449	0.018183
11724491_NUDCD2	-0.13909	0.00645	0.018183
11737390_ZSCAN4	-0.2174	0.006452	0.018189
11739812_HDGFRP3	-0.14492	0.006456	0.018197
11720997_ADCY2	-0.15976	0.006458	0.018203

11730501_CACNA2D3	0.329803	0.006459	0.018205
11737628_TLR6	0.216107	0.00646	0.018207
11723994_STYXL1	-0.14388	0.006468	0.018227
11741544_SLFN14	-0.20438	0.006468	0.018228
11752230_BPTF	0.18587	0.006469	0.018228
11737885_ZFAND4	-0.15268	0.00647	0.018229
11734058_ADAM30	-0.19289	0.006471	0.018233
11729598_TKFC	-0.26116	0.006473	0.018236
11750951_TMEM182	-0.30594	0.006473	0.018237
11744443_CCDC82	0.197431	0.00648	0.018254
11750539_KCNMB2	-0.16809	0.00648	0.018254
11721230_BEND7	0.155224	0.006482	0.018257
11730427_AP1B1	-0.20254	0.006486	0.018267
11752418_AFAP1L1	-0.16505	0.006494	0.018291
11754821_SLC38A1	0.129599	0.006497	0.018298
11746543_SNX9	0.154138	0.006499	0.018301
11738251_CHEK1	-0.15972	0.006499	0.018301
11758005_RANBP2	0.182871	0.006501	0.018306
11718546_ILF3	-0.1265	0.006502	0.018308
11720675_CCS	-0.20415	0.006508	0.018322
11756545_GKN2	-0.27424	0.006508	0.018322
11754505_SLC39A4	-0.21571	0.00651	0.018325
11726776_WNT10B	-0.2713	0.006513	0.018335
11739503_ABCA1	0.386419	0.006515	0.018338
11753946_PCBP2	-0.12336	0.006515	0.018338
11763945_RPSAP9	0.170389	0.00652	0.018349
11716832_TM7SF3	0.124483	0.006525	0.018362
11725535_TBC1D31	-0.19706	0.006526	0.018365
11749832_UBXN2A	0.179282	0.006533	0.018383
11754449_ZNF584	0.216487	0.006541	0.018404
11753556_TTTY13	-0.14173	0.006541	0.018404
11729480_GUK1	0.157418	0.006547	0.018418
11758846_NUS1	0.14008	0.006548	0.018423
11755997_SAMD9L	0.170106	0.006549	0.018423
11756951_STT3B	-0.14214	0.006554	0.018436
11723113_CENPC	0.224438	0.006555	0.018439
11732803_VSX2	-0.16125	0.006564	0.018461
11737377_LUZP4	-0.2252	0.006566	0.018466
11757243_FBXL20	-0.14861	0.006568	0.018471
11742802_SRD5A1	-0.14455	0.006569	0.018474
11719719_LAD1	-0.17314	0.006574	0.018486
11755138_ZXDB	-0.28753	0.006576	0.01849
11739792_MOCS3	0.158833	0.006582	0.018508
11757763_IGF2BP2	0.15824	0.006584	0.018509
11756004_DHRS4 ///	-0.23171	0.006584	0.018509
11756015_DHRS4 ///	-0.23171	0.006584	0.018509
11746219_EYA3	0.124938	0.006585	0.01851
11720955_CWC22	0.220642	0.006588	0.018517
11720420_AFF4	0.18743	0.006591	0.018525
11722220_WBP4	0.13219	0.006597	0.018541
11739405_FPGS	-0.13686	0.006599	0.018546

11758799_SERBP1	-0.14412	0.006611	0.01858
11759916_YME1L1	-0.14878	0.006627	0.018622
11719888_GIGYF2	0.189278	0.006629	0.018627
11760738_SPOCK3	-0.2725	0.00663	0.018627
11715803_ANXA6	0.160947	0.006636	0.018643
11750651_ABCC4	0.233757	0.006641	0.018657
11736997_MOSPD3	-0.16498	0.006641	0.018657
11758172_SLCO3A1	-0.15388	0.006643	0.01866
11758644_ZNF639	0.17191	0.006652	0.018685
11719192_FBXO9	-0.2933	0.006652	0.018685
11742500_PRY /// F	-0.18149	0.006654	0.018688
11757299_GUK1	0.14	0.006655	0.01869
11740098_ANK1	0.202392	0.006659	0.018701
11735274_TBX18	0.16391	0.006661	0.018704
11744112_COQ5	-0.13591	0.006666	0.018718
11715278_AURKAIP1	-0.15868	0.006668	0.018722
11755870_YTHDF1	0.125259	0.006672	0.018732
11760592_SLC35E1	0.154565	0.006672	0.018732
11742748_SPPL3	0.134871	0.006676	0.018741
11733945_RAP2B	-0.31916	0.006677	0.018742
11725527_ZNF248	-0.15157	0.006684	0.018762
11725990_MARK3	0.133568	0.006689	0.018774
11718035_PPIL1	-0.13437	0.00669	0.018777
11742162_KRTAP4-2	-0.15241	0.006692	0.018781
11717169_TRPC4AP	0.126566	0.006694	0.018785
11716904_TGOLN2	0.136614	0.006696	0.01879
11751467_PIGU	-0.21424	0.006699	0.018796
11745056_CACTIN	-0.17432	0.006699	0.018796
11744373_GYG1	0.161025	0.0067	0.018798
11743564_MAP7D3	-0.13811	0.006701	0.018799
11722466_PAX6	-0.16756	0.006701	0.018799
11718684_ANP32B	-0.12647	0.006702	0.018802
11742727_RER1	-0.19104	0.006708	0.018816
11718239_CAPRIN1	0.160977	0.006711	0.018823
11754815_LDLRAP1	-0.1404	0.006711	0.018823
11747002_CPA1	-0.23039	0.006713	0.018826
11717411_ANO6	0.208102	0.006714	0.018829
11717111_REPIN1	0.128497	0.006715	0.01883
11756244_FBXO31	0.202023	0.006718	0.018837
11744648_AIFM2	0.156861	0.006718	0.018837
11752746_PFKL	-0.20854	0.006719	0.018839
11733051_SCAMP1	-0.14274	0.006723	0.018849
11741099_TP53BP2	-0.25481	0.006724	0.018851
11740919_RSPO1	-0.26804	0.006725	0.018851
11745412_LOC441178	-0.15361	0.006725	0.018852
11734179_PTGFR	0.312725	0.006731	0.018867
11744977_MGC45922	-0.15307	0.006739	0.018888
11739083_SLC39A14	-0.21568	0.006743	0.018898
11733674_WDR49	-0.18575	0.006749	0.018915
11754838_DSC3	0.202851	0.006752	0.01892
11749016_AMPD3	0.28102	0.006755	0.01893

11746208_BAK1	-0.23402	0.006757	0.018934
11722702_DZIP1	-0.15334	0.006759	0.018937
11743917_FKBP5	-0.29326	0.006761	0.01894
11751605_SP140	0.182958	0.006761	0.01894
11754683_ALS2CL	0.232965	0.006763	0.018946
11752976_SYNRG	0.239581	0.006763	0.018946
11729515_SLC26A9	-0.21128	0.006765	0.018949
11753115_CLEC12B	0.179165	0.006767	0.018953
11759664_B4GALNT1	-0.22803	0.006768	0.018957
11726692_ANGPT1	0.178024	0.00677	0.018961
11715647_ADIPOR1	0.135507	0.006772	0.018964
11737924_LECT2	-0.20545	0.006773	0.018965
11758956_WWP1	0.204142	0.006785	0.018998
11739247_FAM208B	0.227135	0.006786	0.019001
11717568_NQO1	-0.15012	0.006786	0.019001
11725454_MYO1B	0.181434	0.006788	0.019004
11728477_CXCL3	0.328911	0.006795	0.019021
11744472_TLK2	0.158919	0.006796	0.019023
11763132_IGKC	-0.2377	0.006798	0.019027
200079_PM KARS	0.140316	0.006798	0.019029
11756475_C6orf1	-0.19023	0.006801	0.019035
11735698_ENTPD6	0.201955	0.006803	0.019038
11729066_ZNF211	0.207215	0.006808	0.019053
11731372_NODAL	-0.17917	0.006809	0.019055
11739713_NCOA2	0.271062	0.006816	0.019072
11751063_RAB40B	-0.13684	0.00682	0.019083
11755369_KDM5D	-0.22475	0.006826	0.019099
11737205_GIPR	-0.14314	0.006832	0.019112
11745926_ADAM15	-0.15449	0.006833	0.019116
11730487_DOK6	-0.31824	0.006834	0.019116
11743231_TOR1AIP2	-0.17436	0.006834	0.019116
11718557_CREB3L2	0.26593	0.006835	0.019117
11749465_SMC1B	-0.26614	0.006835	0.019117
11725650_DCAF5	0.302503	0.006836	0.019118
11749288_ST5	-0.16131	0.006837	0.019121
11724807_CBL	-0.14407	0.006838	0.019121
11722205_CDC42EP1	-0.16255	0.006839	0.019124
11730773_ZFYVE1	0.226899	0.006842	0.019131
11723061_RILPL2	-0.17658	0.006843	0.019133
11746466_PRMT2	-0.17719	0.00685	0.019152
11728230_RNF2	0.1757	0.006854	0.019162
11738984_MAP1LC3B	0.125499	0.006855	0.019162
11753404_HLA-E	0.189707	0.006857	0.019168
11719071_HSPA13	-0.17884	0.006858	0.01917
11727318_PURG	-0.33766	0.006859	0.019172
11739289_KMT2A	0.132989	0.006861	0.019174
11755192_TM9SF4	0.142084	0.006861	0.019174
11740735_TMC7	0.179808	0.006862	0.019176
11763489_NSD1	-0.16562	0.006869	0.019193
11753929_ST3GAL2	-0.2141	0.006869	0.019193
11726246_HIST1H4C	-0.12476	0.006871	0.019197

11741641_ZFX	0.312542	0.006873	0.019202
11738049_ATP2C2	-0.19171	0.006873	0.019202
11745784_LOC148413	-0.17979	0.006874	0.019203
11741545_FBXO11	0.152714	0.006876	0.019207
11729906_LRCH2	-0.28179	0.006876	0.019207
11744381_HNRNPU	-0.18974	0.00688	0.019216
11723766_GFER	0.133286	0.006883	0.019224
11755183_MLLT4	0.175336	0.006884	0.019226
11717711_MTFP1	-0.17182	0.006885	0.019228
11732788_ZNF436-AS	-0.18287	0.006889	0.019238
11749769_B4GALNT1	-0.14826	0.006892	0.019243
11722114_RPS6KC1	-0.14829	0.006896	0.019255
11732126_UBB	-0.12274	0.006904	0.019276
11725145_MIS12	-0.21463	0.006905	0.019277
11726294_CEBPA	-0.3533	0.006906	0.019278
11732080_FSD1L	0.309021	0.006907	0.019281
11719211_HSF2	0.216205	0.00691	0.019288
11758152_LRRTM4	-0.19007	0.00691	0.019288
11725006_ARHGEF25	-0.19115	0.006912	0.019292
11743570_EML1	0.22684	0.006918	0.019308
11737131_FITM2	0.257026	0.006919	0.01931
11757024_RNASEK //	0.137681	0.006921	0.019314
11745817_CDIP1	-0.20877	0.006927	0.019328
11747158_PPP6R1	-0.2294	0.006934	0.019347
11726527_MFAP3L	-0.33396	0.006941	0.019365
11728921_CTTNBP2	0.321639	0.006952	0.019396
11733781_RAB18	0.177296	0.006954	0.019401
11726368_ERICH1	0.138167	0.006956	0.019405
11761373_PKMYT1	-0.17947	0.006957	0.019405
11740235_RAB27B	0.496689	0.006958	0.019407
11718984_KLHL21	-0.13472	0.00696	0.019411
11763774_ITM2C	-0.23716	0.006962	0.019416
11743446_SUPT6H	0.20492	0.006963	0.019419
11760878_HLA-DPB1	-0.28847	0.006964	0.01942
11751363_CCDC86	0.152753	0.006965	0.019421
11715977_VGLL4	0.218529	0.006966	0.019421
11750858_PCDHB11	0.184874	0.006966	0.019421
11739071_EPDR1	0.215222	0.006967	0.019424
11726967_COL22A1	-0.19835	0.006971	0.019433
11719626_RDH10	0.192667	0.006973	0.019437
11739034_ACTR2	-0.14529	0.006977	0.019447
11757956_PRTFDC1	-0.30223	0.006979	0.019454
11730213_MRPS7	0.145846	0.00698	0.019454
11728950_LSMEM2	-0.20235	0.00698	0.019454
11748602_BAG2	0.202829	0.006982	0.019459
11723719_C12orf45	0.157756	0.006984	0.019462
11759164_GATSL2	-0.18968	0.006986	0.019466
11734441_FOXI1	-0.21566	0.006988	0.019472
11723679_CD69	0.288119	0.006992	0.01948
11758941_MOB1A	-0.13551	0.007004	0.019513
11755596_NLRP4	-0.17219	0.007009	0.019526

11721018_FBRSL1	-0.16061	0.00701	0.019529
11734704_LAMA4	0.132455	0.007011	0.019531
11736268_TRAPPC8	-0.35746	0.007013	0.019534
11730716_PAGE5	0.122823	0.007015	0.019539
11753531_ZNF428	-0.24232	0.007018	0.019547
11720523_RRP9	-0.17705	0.00702	0.019549
11740078_RGS17	0.253281	0.007021	0.019551
11716606_STOML2	-0.15766	0.007022	0.019553
11717004_FKBP9 ///	-0.12424	0.007026	0.019563
11755918_PTK7	-0.1514	0.007026	0.019563
11724890_POLRMT	-0.2295	0.007027	0.019565
11753721_TRAPPC2 /	0.135361	0.007029	0.019568
11732737_NUP62CL	-0.29147	0.007033	0.019579
11734864_SARNP	0.139463	0.007035	0.019582
11739033_ACTR2	-0.15246	0.007036	0.019584
11744096_DDX24	0.150654	0.007039	0.019593
11751268_MYO18A	-0.28029	0.00704	0.019594
11722145_USP37	0.282459	0.007042	0.019599
11724479_FOSL1	-0.12185	0.007045	0.019604
11735676_OSGIN1	0.176266	0.007045	0.019604
11749426_PLXNB1	0.165517	0.007048	0.019611
11746989_NRBP1	0.139063	0.007052	0.01962
11715858_NCKAP1	0.152994	0.007054	0.019625
11718224_GSPT1	0.132133	0.007056	0.01963
11728710_NFATC2IP	0.135756	0.007062	0.019646
11726706_IL34	0.147583	0.007063	0.019647
11760071_LOC440313	-0.18823	0.007065	0.019652
11739538_ITPR2	-0.17573	0.007073	0.019672
11717141_COG4	0.139426	0.007075	0.019676
11752980_ABHD12	-0.17557	0.007075	0.019676
11724609_FUZ	-0.19694	0.007076	0.019677
11745085_SALL1	-0.15946	0.007076	0.019677
11724427_TMPRSS5	-0.17693	0.007078	0.019682
11744036_CMIP	-0.17086	0.007079	0.019684
11756152_PCNP	-0.13191	0.007087	0.019705
11749609_ZNF321P /	-0.24642	0.007091	0.019714
11741581_SNAP23	0.212784	0.007094	0.01972
11722661_NFE2L3	0.245608	0.007096	0.019726
11731943_AGAP4 ///	0.143145	0.007103	0.019745
11751945_ZNF765	0.148285	0.007104	0.019746
11730872_RASSF5	-0.17224	0.007104	0.019746
11729292_SPPL2B	-0.18324	0.007109	0.019758
11726862_FOXO4	-0.22377	0.00711	0.019758
11730075_KIAA1024	0.326969	0.007114	0.019769
11757273_MBP	-0.15936	0.007115	0.019771
11742795_LRP10	0.145875	0.007122	0.019789
11722117_LZTS1	-0.28926	0.007124	0.019793
11745569_LOC100996	0.157394	0.007124	0.019794
11721566_KBTBD11	-0.38141	0.007132	0.019813
11747890_CTPS1	-0.16009	0.007133	0.019814
11740236_FFAR3	-0.2309	0.007135	0.01982

11752660_SIRPB1	-0.16633	0.007135	0.01982
11726295_ZNF500	0.14954	0.007138	0.019827
11749197_NPAT	0.160588	0.007139	0.019828
11755704_AFAP1	0.226543	0.007148	0.019852
11747524_GALC	0.132944	0.00715	0.019856
11738076_ZIC4	-0.23803	0.007152	0.019861
11724522_DNAJC5	0.139541	0.007158	0.019876
11729579_CDH13	0.130768	0.007162	0.019887
11727376_HMGCR	-0.14852	0.007167	0.019897
11729481_GUK1	0.133562	0.007168	0.019901
11740645_NXPE4	-0.15707	0.007169	0.019903
11747492_ACAD8	0.129935	0.00717	0.019903
11729927_ZNF510	0.249766	0.007172	0.019907
11753256_SLC12A7	-0.20119	0.007172	0.019907
11719264_SPRYD3	-0.14785	0.007174	0.019911
11764146_MOSPD2	-0.16697	0.007177	0.019919
11726827_STAP1	-0.26826	0.007179	0.019922
11747994_TBC1D1	0.178792	0.007182	0.01993
11733143_IMPA1	0.16126	0.007183	0.019933
11730993_NLGN4X	-0.15837	0.007186	0.019939
11726572_SEMA7A	0.175539	0.007188	0.019943
11737175_WNK1	-0.17873	0.007189	0.019945
11717273_COL5A1	0.634565	0.007193	0.019954
11734835_TAF9B	0.131948	0.007195	0.019959
11746726_SUPT6H	0.138721	0.007197	0.019962
11733426_HIST1H3I	0.192013	0.007197	0.019962
11758612_MPPED2	-0.24122	0.007197	0.019962
11761321_ANXA8 ///	-0.2068	0.007198	0.019963
11734252_TMEM87A	0.155583	0.007198	0.019963
11729800_MTTP	-0.22358	0.007199	0.019964
11758509_ENPEP	0.365063	0.0072	0.019964
11723400_STK4	0.132286	0.007206	0.019982
11746754_ZDHHC7	-0.21159	0.007209	0.019988
11746631_CEP41	-0.16135	0.007209	0.019988
11723341_OXR1	0.167735	0.007212	0.019994
11760297_NTMT1	0.134857	0.007215	0.020001
11735123_POLR1E	0.133236	0.007215	0.020001
11753928_ST3GAL2	-0.29602	0.007223	0.020022
11756480_CSDE1	-0.12057	0.007225	0.020026
11757131_SNORD15B	-0.18765	0.007226	0.020027
11737827_ZKSCAN8	0.246228	0.007231	0.020041
11742676_IRF2BP2	0.123817	0.007232	0.020041
11720226_TBC1D1	0.13711	0.007239	0.020059
11749973_TIE1	-0.13755	0.00724	0.020062
11717854_ZSCAN18	0.280203	0.007241	0.020062
11731681_KATNAL2	-0.13258	0.007249	0.020085
11753771_XRN2	0.131766	0.00725	0.020087
11743330_PGP	-0.1343	0.007255	0.020097
11755293_GLO1	-0.15331	0.007255	0.020097
11725115_NCOR1	0.142834	0.007258	0.020104
11752091_THADA	0.178768	0.007258	0.020104

11764149_DUSP16	-0.17927	0.007265	0.020121
11749294_TPD52L2	-0.1863	0.007269	0.020132
11738971_PYDC2	-0.15614	0.007271	0.020135
11716237_MGAT1	-0.12512	0.007271	0.020135
11727418_ATP5SL	-0.17273	0.007272	0.020135
11756300_ANP32B	-0.13566	0.007273	0.020138
11730113_ZDHHC13	-0.1962	0.007274	0.020138
11747845_PRKCZ	0.143858	0.007275	0.02014
11750574_ATP8B3	-0.34078	0.00728	0.020154
11756211_C11orf73	0.141242	0.007282	0.020158
11729040_ZNF469	0.170727	0.007283	0.020159
11725550_PDE4A	-0.2518	0.007284	0.020159
11716717_NINJ1	0.151373	0.007284	0.020159
11750107_SEL1L3	-0.18259	0.007284	0.020159
11750910_ARHGEF4	0.141525	0.007287	0.020166
11734521_SCLT1	-0.22132	0.007288	0.020168
11744654_CD44	-0.13227	0.007291	0.020175
11716085_SF3B1	0.127549	0.007292	0.020176
11718908_CHST2	-0.19725	0.007294	0.02018
11716814_GATM	0.275966	0.007296	0.020184
11753113_DDX11 ///	-0.21406	0.007297	0.020186
11715874_ATP5H	-0.12975	0.007297	0.020186
11757066_ING5	0.235952	0.007301	0.020196
11744411_RSL24D1	-0.12718	0.007302	0.020198
11719478_FKBP3	-0.147	0.007307	0.02021
11764081_FAM26E	-0.19375	0.00731	0.020218
11733861_CLASP2	-0.16406	0.007311	0.020219
11734680_ATG4B	-0.32694	0.007312	0.020221
11742327_GHRHR	-0.16789	0.007313	0.020223
11720031_TRIM29	-0.37143	0.007315	0.020227
11744324_SLX4IP	-0.15555	0.007319	0.020237
11717898_MMS19	-0.18898	0.007319	0.020237
11738833_TCL6	0.204697	0.007327	0.020255
11742185_CALN1	-0.19768	0.007328	0.020258
11763968_SFTPC	-0.22412	0.007336	0.02028
11725687_PRIM2	-0.16399	0.007339	0.020285
11749405_NCKIPSD	-0.18384	0.007339	0.020285
11741494_BTBD1	0.164387	0.00734	0.020286
11725141_FUBP3	-0.23976	0.007351	0.020314
11753179_FAM134B	-0.12276	0.007351	0.020314
11739037_PLP1	-0.18918	0.007355	0.020325
11728102_ZNF367	0.121752	0.007356	0.020326
11759543_PRMT2	0.151186	0.007357	0.020328
11739443_CCDC71L	0.217921	0.007357	0.020328
11741425_MRO	0.179766	0.007364	0.020344
11746582_ZNF558	0.273741	0.007366	0.02035
11758535_GPAM	-0.20551	0.007369	0.020357
11732154_OGT	-0.19789	0.00737	0.020358
11737391_OR2L13	-0.25418	0.007372	0.020363
11723260_ZNF24	-0.13561	0.007373	0.020363
11720658_SH3BP4	0.261389	0.007373	0.020363

11758230_NFIA	0.296327	0.007374	0.020363
11754487_NADK2	0.15631	0.007374	0.020364
11759747_MBD1	0.165018	0.007376	0.020367
11720865_IFT27	0.288464	0.007379	0.020374
11733291_TRPM1	-0.15445	0.007379	0.020374
11763844_UBXN6	0.145984	0.007384	0.020386
11754991_RXRB	-0.35536	0.007387	0.020393
11730435_SRSF12	-0.1824	0.007388	0.020393
11720181_DRG1	0.150506	0.007388	0.020393
11752607_ST7 /// S	0.158793	0.007388	0.020393
11741520_CD14	0.194492	0.007392	0.020403
11750845_CSPG5	-0.25273	0.007396	0.020413
11739488_RBM47	-0.14057	0.007399	0.020419
11760325_MSI2	0.30567	0.007401	0.020424
11748250_NDRG3	0.136047	0.007407	0.02044
11757817_BASP1	-0.21727	0.007409	0.020445
11761970_ALS2	0.459821	0.007413	0.020453
11716864_NISCH	0.153279	0.007413	0.020453
11762747_MMP13	0.174343	0.007419	0.020466
11737572_HIST1H2AL	-0.31305	0.007419	0.020466
11752054_DGKG	0.286198	0.00742	0.020467
11739689_DCAF11	-0.23532	0.007426	0.020484
11758458_GPX4	-0.13183	0.007427	0.020486
11721984_ZBED6CL	-0.16077	0.00743	0.020493
11751730_SAE1	-0.13961	0.007436	0.020507
11749447_NBR2	-0.1886	0.007437	0.020508
11759809_PLEKHS1	0.534477	0.00744	0.020518
11716028_SELT	0.154559	0.007446	0.020529
11726628_SEMA4A	0.178345	0.007446	0.020529
11717179_EDC4	0.140302	0.007446	0.020529
11726099_NKAIN1	-0.1354	0.007448	0.020534
11724264_IFT22	0.146813	0.007451	0.020542
11748913_B4GALT4	-0.19519	0.007457	0.020557
11763382_NUMB	0.340662	0.007461	0.020567
11726979_SUGP2	-0.29063	0.007461	0.020567
11747605_RPP30	-0.15201	0.007464	0.020572
11727620_LIPH	0.445544	0.007464	0.020572
11755784_JADE2	-0.13213	0.007469	0.020584
11750739_SLF1	-0.14977	0.007474	0.020597
11756656_RPS6KC1	-0.13128	0.007476	0.0206
11743520_STAG2	0.154199	0.00748	0.02061
11724944_CLSTN2	0.183279	0.007481	0.020612
11719230_PCDH1	0.129986	0.007482	0.020612
11753523_MED16	-0.14086	0.007486	0.020624
11757952_LRP11	-0.24631	0.007488	0.020627
11763696_NIPA2	-0.1706	0.007489	0.02063
11718376_LOC401127	0.123639	0.007492	0.020636
11758266_PSMD2	-0.14093	0.007492	0.020636
11735891_EDA	-0.26599	0.007496	0.020646
11742553_OR52N1	-0.17356	0.007497	0.020647
11722539_CLDN15	0.172025	0.007504	0.020665

11741029_CSTF1	0.145956	0.007505	0.020667
11731287_GRXCR1	-0.25437	0.007506	0.020667
11734335_SIGIRR	-0.1615	0.007507	0.02067
11739883_DOCK7	0.237481	0.007507	0.02067
11729003_ADRB1	-0.30683	0.007512	0.020682
11757708_GZF1	0.290298	0.007513	0.020682
11730959_DGKG	0.204624	0.007518	0.020695
11754514_ACACA	-0.21495	0.007521	0.020704
11754073_TCL1A	-0.21319	0.007523	0.020707
11731614_ETFA	0.122973	0.007525	0.02071
11753813_RBM15	0.128925	0.007525	0.02071
11751112_ADAT1	0.265242	0.007531	0.020725
11719635_NUP153	0.159438	0.007531	0.020725
11764187_LOC105377	-0.25072	0.00754	0.020747
11743098_TARSL2	0.308508	0.007542	0.020753
11735857_IFNE	0.218171	0.007544	0.020757
11751600_DAPP1	-0.17642	0.007545	0.020758
11728008_FUT3	-0.2383	0.007547	0.020762
11751875_MGA	0.214449	0.007548	0.020765
11727200_ZRANB2	0.130693	0.00755	0.020768
11742268_CLEC4M	-0.22377	0.007555	0.02078
11757114_SNORA12	0.260294	0.007555	0.020781
11744702_KCNMA1	0.149192	0.007557	0.020783
11762910_LOC100506	0.268351	0.007561	0.020793
11753185_KLF16	-0.14881	0.007561	0.020794
11752767_SPAG9	0.135025	0.007563	0.020798
11716512_SRRM2	-0.15274	0.007564	0.020799
AFFX-HSACACTB	0.119719	0.007565	0.0208
11740604_ 15-Sep	0.123012	0.007566	0.020802
11747963_ARFGAP2	-0.21084	0.007567	0.020803
11722318_EFNB3	-0.12955	0.007569	0.020808
11723380_CEP170B	-0.14453	0.007569	0.020808
11748063_HDAC7	-0.16578	0.007573	0.020816
11749802_ARL8B	0.131428	0.007575	0.020821
11721271_LRRC59	-0.1908	0.007578	0.020829
11744203_PRPF6	-0.16291	0.00758	0.020833
11762338_GSTP1	-0.1776	0.007581	0.020834
11719936_EDC3	0.182983	0.007582	0.020834
11742978_CHMP4C	0.314269	0.007582	0.020834
11759100_EIF5A	-0.13316	0.007584	0.020839
11724684_ARL6IP4	-0.19413	0.007585	0.020841
11763806_KCNN4	0.133717	0.007586	0.020841
11762211_DLEU2	-0.22856	0.007587	0.020843
11763743_AMT	-0.18583	0.007589	0.020847
11750551_STK4	0.13562	0.00759	0.020849
11722687_LIFR	0.191968	0.007597	0.020866
11758745_SYVN1	0.140321	0.007603	0.020884
11754017_RANBP3	-0.13778	0.007609	0.020896
11758494_RAD51D	-0.21448	0.007609	0.020896
11728031_KERA	-0.20049	0.007613	0.020906
11752829_WAC	0.13351	0.007622	0.020931

11715234_UCN	0.150008	0.007627	0.020943
11732104_TMEM208	-0.1424	0.007634	0.02096
11746050_VPS52	-0.16917	0.007635	0.020963
11748648_PTPRR	0.361147	0.007638	0.020968
11732149_DCAF11	-0.17271	0.007638	0.020968
11725776_ATF7IP2	0.229105	0.007639	0.020968
11721819_MST1	-0.16292	0.00764	0.020971
11754824_LGALSL	0.32131	0.007641	0.020972
11751457_MAT2A	0.156205	0.007646	0.020984
11726413_USP54	0.200758	0.007648	0.020989
11754936_C14orf119	0.126026	0.007649	0.020992
11753478_CCDC24	0.15959	0.007655	0.021005
11716758_DHX30	0.141008	0.007655	0.021006
11726237_TRIM36	-0.19091	0.007659	0.021013
11717129_SAR1A	-0.14998	0.007659	0.021013
11729386_RPL31	-0.16801	0.007661	0.021016
11728314_ZNF148	-0.18701	0.007661	0.021016
11732340_BCL11A	0.182479	0.007666	0.021028
11732753_FAM205A	0.157228	0.007669	0.021036
11725673_HINT3	0.181218	0.007673	0.021045
11726693_ANGPT1	0.15472	0.007675	0.02105
11744396_CALCOCO2	-0.1786	0.00769	0.02109
11732540_HSD17B13	-0.13858	0.007693	0.021098
11751714_TESK2	-0.27054	0.0077	0.021115
11728451_PCOLCE2	-0.16626	0.007702	0.021121
11747213_CFHR3	0.414048	0.007707	0.021132
11731487_NFIC	-0.15163	0.007708	0.021134
11720480_BCCIP	-0.14913	0.007723	0.021174
11735996_USP14	-0.17118	0.007725	0.021178
11721781_ITGAE	0.141134	0.007728	0.021184
11743116_KPNB1	-0.12988	0.007729	0.021186
11719143_INSIG2	-0.17169	0.007736	0.021203
11720460_F11R	-0.1413	0.007738	0.021209
11740047_ANKRD17	-0.13315	0.00774	0.021212
11719260_LMNA	-0.133	0.007741	0.021215
11741780_C10orf91	-0.21074	0.007742	0.021216
11744543_KDELR1	-0.15591	0.007743	0.021219
11715835_PSMA3	-0.12805	0.007744	0.021219
11725156_CCDC91	0.162345	0.007748	0.021229
11725654_GTF2H5	-0.14603	0.007752	0.021239
11730830_AMPD2	-0.1328	0.007759	0.021257
11748921_NSMF	-0.14476	0.007759	0.021257
11763558_FMNL1	-0.20164	0.007767	0.021277
11716530_ELAVL1	-0.13237	0.007768	0.021277
11753777_KCNMB2	0.208669	0.00777	0.021282
11726925_HCFC1R1	-0.13886	0.00777	0.021282
11745277_SSH1	0.260388	0.007771	0.021284
11721054_KLHL42	-0.16899	0.007773	0.021287
11725573_CST9L	-0.15962	0.007775	0.021291
11719308_RASSF4	-0.26819	0.007776	0.021292
11745145_METTL16	-0.14724	0.00778	0.021302

11744092_ATXN7L3B	-0.15576	0.007781	0.021302
11748296_PPP2R5E	0.197299	0.007781	0.021302
11756071_ACTB	0.122257	0.007781	0.021303
11717170_TRPC4AP	0.134635	0.007785	0.02131
11734830_SUV39H2	-0.15139	0.007791	0.021327
11734959_TRHDE	-0.15909	0.007798	0.021344
11753031_AES	-0.20303	0.007801	0.021353
11762420_FBXO11	-0.18021	0.007803	0.021356
11741031_HAX1	-0.14432	0.007805	0.02136
11715392_MTCH1	-0.12111	0.007805	0.02136
11737012_APLF ///	0.250765	0.007806	0.021361
11752208_GATA2	-0.26239	0.007814	0.021381
11741167_PCDHB8	0.210919	0.007818	0.02139
11734980_RALY	-0.15038	0.007818	0.02139
11731810_FCRL4	-0.17195	0.00782	0.021395
11722723_UNC13D	-0.19468	0.007823	0.021402
11759899_ATF7IP	-0.32946	0.007825	0.021405
11745008_KATNB1	-0.15865	0.007831	0.02142
11746179_RHBDF1	0.168568	0.007833	0.021425
11726175_CACNA1D	-0.32605	0.007835	0.02143
11724224_AMPD2	-0.13679	0.007838	0.021436
11756517_LOC105379	-0.18083	0.007845	0.021454
11757930_PAAF1	-0.18277	0.007845	0.021454
11743356_ZBTB40	0.156013	0.007849	0.021462
11747009_MTRF1	-0.26521	0.007854	0.021476
11745652_EPCAM	0.2958	0.007857	0.021482
11751423_OVGP1	0.275108	0.007857	0.021482
11740759_VPS13D	-0.18676	0.007864	0.0215
11715719_CDC37	0.1366	0.007866	0.021502
11749450_DNASE2	-0.19382	0.007868	0.021506
11760928_LRCH3	0.138391	0.007872	0.021515
11748010_CTNNA2	-0.18879	0.007872	0.021515
11727553_PKIB	0.131464	0.007874	0.021519
11742350_OR52H1	-0.32506	0.007876	0.021525
11744757_TARS	0.132979	0.007881	0.021537
200008_PMGDI2	0.130029	0.007881	0.021537
11722208_PTGS1	-0.18014	0.007885	0.021546
11752942_DBP	-0.14641	0.007887	0.021548
11742476_GLRA4	-0.24441	0.007887	0.021548
11724086_FIZ1	-0.13565	0.00789	0.021556
11721944_RABL2A //	0.173215	0.007892	0.021561
11751352_HMG20A	-0.23056	0.0079	0.021579
11746679_SRRM1	0.133911	0.007902	0.021584
11723541_TMEM80	-0.16944	0.007902	0.021584
11719987_CCDC117	-0.18544	0.007907	0.021595
11716600_CHMP1A	-0.17388	0.00791	0.021603
11755591_MSTO1	0.150376	0.007919	0.021625
11734109_LSM11	0.225001	0.007924	0.021639
11718318_CD93	-0.23103	0.007931	0.021658
11735944_TTC21B	0.281082	0.007935	0.021665
11743565_CABLES2	-0.16781	0.007935	0.021665

11745514_PRPSAP2	-0.13523	0.007936	0.021666
11725554_TMEM133	0.274429	0.007938	0.021671
11736029_ITGA6	0.120063	0.007941	0.021677
11749174_PLEKHS1	-0.13138	0.007944	0.021684
11757345_TNNI2	0.221141	0.007945	0.021686
11734903_C7orf26	0.127328	0.007954	0.021709
11730323_RPS29	0.21094	0.007955	0.02171
11719651_SPAG6	0.256312	0.007955	0.02171
11716796_IFI16	-0.12628	0.007957	0.021716
11757095_HGH1	-0.177	0.007964	0.021734
11757697_FAM96A	-0.142	0.007968	0.021743
11727055_SERPIND1	0.191072	0.007969	0.021745
11717819_TOB2	-0.16368	0.007976	0.021762
11756640_LRWD1	0.223842	0.007977	0.021762
11759518_KIAA0754	0.224276	0.007977	0.021762
11751700_KCNK2	-0.14757	0.00798	0.021768
11725716_KIF21A	-0.1962	0.007984	0.021779
11747455_TAF1A	0.166656	0.007985	0.021781
11720245_CDPF1	-0.17141	0.007986	0.021782
11739541_PIK3R1	0.190065	0.007995	0.021804
11730050_CA13	0.379515	0.007998	0.021811
11737899_TOX2	-0.16462	0.008	0.021816
11758742_RNF40	0.155857	0.008004	0.021825
11750040_ANXA5	0.188835	0.008004	0.021825
11761725_PNOC	-0.30932	0.008005	0.021825
11739681_AHR	0.180326	0.008005	0.021825
11739120_XPO7	-0.13548	0.00801	0.021836
11746742_ASL	-0.13188	0.008011	0.021839
11758650_SNRPC	-0.12603	0.008014	0.021845
11754147_LZTS1	-0.19278	0.008015	0.021847
11730178_SPOP	0.162763	0.008017	0.021851
11762414_ABCC13	-0.25387	0.008019	0.021857
11715731_CYP19A1	0.161321	0.008022	0.021863
11738907_OR52L1	-0.14977	0.008023	0.021863
11734337_THSD7B	0.148987	0.008026	0.02187
11724101_MAP3K6	-0.30449	0.008026	0.02187
11741850_TCTE3	-0.20712	0.008028	0.021873
11741498_ZNF484	0.208286	0.008032	0.021884
11719534_STX3	0.13728	0.008033	0.021886
11758364_C19orf52	-0.14269	0.008038	0.021896
11749622_TRIM41	0.137191	0.008038	0.021897
11724983_PCDH7	0.365389	0.008042	0.021906
11745780_OXR1	0.144343	0.008048	0.021921
11753055_RAP1B ///	0.185577	0.008051	0.021929
11734576_ASB16-AS1	-0.13073	0.008054	0.021934
11724608_FUZ	-0.19938	0.008057	0.021941
11716446_PRAF2	0.15219	0.00806	0.02195
11718042_MED17	-0.18397	0.008063	0.021954
11740145_CEP164	0.155144	0.008064	0.021958
11753603_RAC1	-0.14181	0.008067	0.021964
11723512_CDC14B	0.234088	0.008068	0.021966

11734162_TFEC	0.248738	0.008068	0.021966
11742857_TXNDC17	0.208795	0.008074	0.021979
11720637_CXADR	0.278583	0.008074	0.021979
11736734_DPY19L3	0.433676	0.008075	0.02198
11734336_SIGIRR	-0.15078	0.008077	0.021984
11754923_HPS4	-0.20869	0.008078	0.021984
11724634_ABCB10	-0.14878	0.008078	0.021985
11732305_CREB1	-0.18419	0.00808	0.02199
11719911_TNFSF12	-0.17872	0.008084	0.021999
11750986_TNIP1	0.176886	0.008085	0.021999
11763462_ZNF780A	0.229945	0.008085	0.021999
11719420_ABCC1	0.152771	0.008086	0.022001
11726315_METTL22	0.204696	0.008089	0.022008
11755084_CD55	0.155271	0.008091	0.02201
11730148_ADGRG5	-0.26754	0.008092	0.022014
11746149_BCHE	-0.20965	0.008094	0.022017
11724485_CSK	0.12183	0.008103	0.022041
11727066_ANKRD11	0.17042	0.008108	0.022053
11763227_C6orf48 /	-0.37695	0.00811	0.022057
11720551_IFFO1	0.137461	0.008111	0.022058
11742841_SMARCA1	-0.15786	0.00812	0.02208
11736898_NAIP	0.188414	0.008121	0.022084
11755636_RIC8A	0.12268	0.008127	0.022097
11719956_POLR3H	0.135929	0.008127	0.022097
11730499_MXI1	-0.41059	0.008127	0.022097
11736283_NKX3-1	0.169696	0.00813	0.022102
11740079_ZNF219	-0.21248	0.008133	0.022108
11728453_DOCK9	0.140962	0.008135	0.022114
11731610_GPALPP1	-0.18634	0.008137	0.022117
11717972_FAM219B	0.213061	0.008137	0.022117
11741986_NXPE4	-0.1987	0.008144	0.022135
11759688_PANK2	0.1569	0.008153	0.022157
11758855_ZNF689	0.248385	0.008157	0.022167
11729823_DPH7	0.146149	0.00816	0.022173
11749292_NIPAL2	0.359714	0.008163	0.022182
11756103_HS3ST5	0.186064	0.008167	0.022191
11734870_NADK	-0.1176	0.008168	0.022193
11732674_C1orf53	-0.18308	0.008171	0.0222
11718512_TSPAN14	-0.18259	0.00818	0.022222
11756997_LENG8	0.138795	0.008182	0.022227
11732770_FOXD4 ///	0.261518	0.008183	0.022228
11733213_SLFN5	0.206452	0.008184	0.022228
11760175_UQCC2	-0.16808	0.008184	0.022228
11756624_RABL2A	0.198469	0.008184	0.022228
11741805_WNT2B	-0.1592	0.008191	0.022246
11717489_SEPT5 ///	-0.18124	0.008195	0.022254
11760861_WBSCR16	0.218631	0.008207	0.022286
11717206_GOLGA2	-0.12702	0.008209	0.022289
11756051_MTR	-0.2386	0.008214	0.022302
11730379_KRIT1	0.128051	0.008214	0.022302
11725520_ICA1	0.174374	0.008215	0.022302

11724647_VANGL1	0.172001	0.008215	0.022302
11727415_CLN8	0.268463	0.008217	0.022304
11757105_UBTFL1 //	-0.21162	0.008219	0.02231
11734253_ZNF572	-0.24314	0.008221	0.022314
11734594_LIN7B	0.194961	0.008225	0.022322
11728276_PLA2G12A	-0.13875	0.008226	0.022324
11758317_DENND3	-0.17099	0.008234	0.022345
11763315_KCNAB2	-0.22545	0.008235	0.022347
11722657_SMN1 ///	0.133219	0.008236	0.022348
11724060_TECPR2	-0.27608	0.008239	0.022355
11747889_SH3YL1	0.201189	0.008246	0.022374
11730410_PXMP4	-0.19625	0.00825	0.022383
11747482_HNRNPR	-0.13415	0.00825	0.022383
11763996_FBXL17	0.190221	0.008257	0.022399
11724614_LIMK1	-0.19066	0.008258	0.022399
11763394_LOC105377	-0.25561	0.008258	0.022399
11736152_XPR1	0.211147	0.008272	0.022436
11756237_FBLN1	-0.22822	0.008277	0.022448
11736396_RFT1	-0.16448	0.008278	0.022449
11763142_LAMTOR5P1	-0.14372	0.008282	0.022461
11747444_NCF1 ///	-0.21386	0.008284	0.022464
11759490_OSER1	0.193306	0.008288	0.022475
11733631_LRRC10	-0.23633	0.00829	0.022478
11764283_PNISR	0.151255	0.008294	0.022486
11746505_ICA1	0.224397	0.008294	0.022486
11739368_DMXL2	-0.17196	0.008295	0.022487
11749331_PNPO	0.188757	0.008297	0.022491
11763083_LSM12	0.172388	0.0083	0.022498
11743349_TRAF7	-0.13465	0.008305	0.022512
11746243_E2F6	0.163317	0.008307	0.022516
11728952_SLC38A10	0.165659	0.008309	0.02252
11763354_PGAM5	-0.13972	0.00831	0.022521
11748423_PGM1	-0.15142	0.008313	0.022528
11740200_FCMR	0.232139	0.008314	0.022528
11756223_DDX21	0.127343	0.008314	0.022528
11726632_TRIM8	0.118558	0.008316	0.022532
11739501_DSP	-0.15712	0.008317	0.022533
11743686_ZNF436	0.19617	0.008319	0.022538
11734320_SLC17A4	-0.17457	0.00832	0.02254
11718270_HNRNPUL1	-0.13297	0.008321	0.02254
11726415_ANKRD6	0.519463	0.008321	0.02254
11752884_UPF3A	0.140276	0.008322	0.02254
11740866_CDK10	-0.14568	0.008322	0.02254
11743331_KIDINS220	0.201992	0.008324	0.022543
11745736_ACADVL	-0.16025	0.008325	0.022545
11745852_NUBP2	-0.13572	0.008327	0.022548
11719199_CCND2	-0.18118	0.008329	0.022553
11722510_CRTC3	0.14085	0.00833	0.022554
11762795_RUSC1-AS1	-0.14789	0.00833	0.022554
11737803_YIPF2	-0.14356	0.008333	0.022559
11716751_GNS	0.146407	0.008335	0.022564

11743641_TFAP2A	0.170388	0.008338	0.022572
11763344_FAM204A	-0.30061	0.008343	0.022583
11727820_CISD1	-0.15454	0.008353	0.022609
11743643_DHODH	-0.17804	0.008354	0.02261
11718274_EIF3L	0.137681	0.00836	0.022626
11715733_NIPSNAP1	-0.14879	0.008362	0.022631
11724568_USP48	-0.15384	0.008365	0.022637
11732307_CREB1	-0.38202	0.008366	0.022638
11748628_ZNF252P	0.304602	0.008366	0.022638
11725541_GATA4	-0.26346	0.008367	0.022639
11748903_PARVG	-0.13313	0.00837	0.022645
11731953_SLC4A8	-0.20817	0.008371	0.022647
11748665_PICALM	0.139937	0.008375	0.022656
11746328_MAEA	0.150595	0.008376	0.022658
11738783_HIST1H3C	-0.22895	0.008386	0.022684
11728671_UBE2H	0.18142	0.00839	0.022692
11737564_CCDC74B	0.160074	0.008398	0.022714
11742651_C16orf13	-0.14121	0.0084	0.022718
11742043_CD44	0.130963	0.008401	0.022719
11729615_HMOX2	0.130976	0.008408	0.022738
11758110_SIX1	-0.12897	0.00841	0.02274
11726264_STRADB	0.229487	0.008411	0.02274
11724266_C2orf72	-0.12277	0.008411	0.02274
11734590_NPBWR2	-0.23358	0.008413	0.022745
11718569_WIPF1	-0.14884	0.008413	0.022745
11744589_PCGF2	-0.17055	0.008421	0.022765
11740670_TNRC6B	0.156551	0.008429	0.022785
11755003_ADCY4	-0.17381	0.008432	0.022791
11719843_ABI3BP	-0.14396	0.008433	0.022791
11740705_NYAP2	-0.16328	0.008433	0.022791
11734666_POLR2B	-0.13388	0.008447	0.022827
11753804_TXN	-0.12785	0.008447	0.022827
11745041_CIDEC	-0.12915	0.00845	0.022833
11729877_ZNF445	-0.19642	0.008458	0.022852
11737384_PCDH7	0.334951	0.008458	0.022852
11716368_PRR13	-0.12062	0.008459	0.022854
11763963_NKD1	-0.12683	0.00846	0.022857
11737721_COL25A1	-0.1219	0.008466	0.022871
11720399_NCS1	-0.21627	0.008466	0.022871
11745458_EYA3	0.245787	0.008469	0.022877
11741662_EIF3C ///	-0.13641	0.008471	0.022881
11722475_ARID3B	0.268203	0.008472	0.022883
11744657_FGFR4	-0.27666	0.008473	0.022884
11754179_GOLT1B	0.159484	0.008474	0.022886
11748558_DBNL	-0.14178	0.00848	0.0229
11751821_CYBB	-0.26205	0.008481	0.022901
11726325_MAGEC2	0.125548	0.00849	0.022925
11717344_FOSB	-0.16153	0.008496	0.022938
11720671_SNX7	-0.12861	0.008496	0.022938
11726409_GID4	0.178657	0.0085	0.022947
11717078_ATP8B5P /	0.15018	0.008503	0.022953

11725386_HOMER1	0.135539	0.008506	0.022962
11720921_ACAA1	0.135761	0.008507	0.022964
11756425_ANKS1A	-0.1824	0.008509	0.022966
11734070_SSR3	-0.16015	0.008511	0.02297
11755677_ZNF257	0.138397	0.008511	0.02297
11753906_SMARCA1	-0.12951	0.008518	0.022988
11730791_SVIP	-0.1433	0.008519	0.022989
11756109_MGAT2	0.117281	0.00852	0.022989
11718996_MSRB2	0.277132	0.008521	0.02299
11747320_FAM3A	-0.18217	0.008521	0.02299
11750239_VPS13B	0.148719	0.008523	0.022995
11750579_HDAC4	-0.13635	0.008534	0.023023
11741609_PTPRS	-0.1929	0.008537	0.023031
11724239_TCF4	-0.15471	0.008539	0.023033
11719493_AK6 /// T	0.133839	0.008546	0.023053
11726164_EPHA7	0.209588	0.008548	0.023056
11735875_PCDHGA4	-0.23457	0.008556	0.023075
11737135_HOXC6	0.144379	0.00856	0.023086
11731191_KIAA2022	-0.19884	0.008573	0.023118
11758767_SEL1L	-0.24024	0.008576	0.023127
11732326_PBLD	0.249442	0.008579	0.023132
11755673_WHSC1	-0.13268	0.008585	0.023147
11727160_NFIX	-0.15336	0.008587	0.023152
11726920_HAPLN4	-0.25173	0.008599	0.023183
11725915_POU6F1	-0.31755	0.0086	0.023185
11759106_NKD1	-0.20681	0.008603	0.02319
11737813_NNT	-0.18514	0.008604	0.023192
11738110_PIKFYVE	0.290925	0.008605	0.023193
11744154_SMG5	0.161232	0.008607	0.023198
11746266_CHRND	-0.31874	0.008607	0.023198
11718702_ARFIP1	0.166523	0.008609	0.0232
11717953_DCPS	-0.20498	0.00861	0.023201
11745750_PNPLA8	0.126137	0.008613	0.023209
11750230_ABHD14A-A	-0.13224	0.008615	0.023213
11741438_CLGN	-0.24446	0.008615	0.023213
11741748_DLG1	-0.16687	0.008617	0.023215
11748690_EZH2	-0.14429	0.008617	0.023215
11735413_CRCP	0.157543	0.008618	0.023217
11733084_PALLD	0.198634	0.008622	0.023226
11718845_BCL2L2-PA	0.126974	0.008623	0.023227
11736828_ST3GAL1	0.146744	0.008623	0.023227
11745617_TRIM15	-0.15682	0.008624	0.023227
11718530_PCIF1	-0.15547	0.008624	0.023227
11742504_CLTA	0.157217	0.008625	0.023229
11733888_FBXO42	0.152046	0.008635	0.023251
11754964_NDUFA10	-0.27296	0.008635	0.023251
11756460_CEP41	-0.12191	0.008636	0.023251
11739320_ATG12	0.152544	0.008636	0.023251
11720341_NDUFA13	-0.21792	0.008636	0.023251
11726709_PFAS	-0.21524	0.008637	0.023251
11750375_RAPGEF6	0.176378	0.00864	0.02326

11719783_RPS23	-0.11883	0.008654	0.023296
11753848_DGCR6 ///	-0.19815	0.008658	0.023305
11730709_C9orf57	0.228294	0.008658	0.023305
11721318_PARM1	0.174478	0.008665	0.023322
11724622_HPN	-0.14382	0.008671	0.023336
11753765_CHN2 ///	-0.21913	0.008677	0.023352
11734742_LIMK2	0.162256	0.008679	0.023356
11737704_ADCK1	-0.22982	0.008684	0.023368
11741759_PAK3	-0.13692	0.008685	0.02337
11761582_LRCH3	0.147142	0.008687	0.023373
11724471_MGST1	-0.14287	0.008689	0.023377
11720058_ATG16L1	0.13947	0.008693	0.023386
11752310_SEMA6B	-0.25403	0.008699	0.023401
11760675_CCDC74B	-0.19858	0.008699	0.023401
11743362_ADSS	-0.12968	0.008704	0.023412
11740211_BPTF ///	0.1462	0.00871	0.023426
11751793_MLXIP	-0.14695	0.008719	0.02345
11721698_UBE3B	0.159776	0.008724	0.023463
11722654_STK17A	-0.27691	0.008726	0.023465
11743204_NANOS3	-0.13377	0.008726	0.023465
11749078_CEP104	0.193189	0.008727	0.023466
11750703_PAPPA-AS1	0.131902	0.008735	0.023486
11763746_CLPX	0.174956	0.008736	0.023487
11748652_PMS1	-0.22276	0.00874	0.023497
11747089_PTPRS	-0.13857	0.008744	0.023505
11725292_DAO	-0.3716	0.008747	0.023512
11717828_LTBR	0.130088	0.008748	0.023514
11719340_MYRF	-0.14122	0.008751	0.023522
11735605_NAT16	-0.13793	0.008752	0.023522
11734093_CCER1	-0.16795	0.008755	0.023529
11763764_GPATCH3	-0.18733	0.008757	0.023534
11744614_SLC6A13	-0.23932	0.008762	0.023545
11731274_CLEC4G	-0.25462	0.008767	0.023557
11762867_ABCB4	0.118618	0.00877	0.023564
11726256_RNF141	-0.24057	0.008772	0.023569
11761559_TRMT13	0.149851	0.008785	0.023602
11732468_ATP12A	-0.16772	0.008788	0.023608
11718795_PAQR4	-0.1664	0.008788	0.023608
11757084_GLRX3	0.139431	0.00879	0.02361
11743691_MRPL30	-0.15529	0.00879	0.02361
11754253_COLGALT2	0.208383	0.008794	0.023619
11741611_DGKG	0.273664	0.008796	0.023624
11731215_MATN4	-0.18384	0.0088	0.023634
11726760_RCE1	0.211916	0.008803	0.023641
11746365_EPB41	0.276491	0.008806	0.023646
11756296_MFF	-0.13322	0.008807	0.023648
11761682_CD5L	-0.31726	0.008808	0.02365
11730132_CACNA1F	-0.24891	0.008809	0.023652
11717499_SLC25A36	0.135829	0.008812	0.023659
11762298_TMEM234	-0.15826	0.008815	0.023665
11745990_SECISBP2	0.164023	0.00882	0.023676

11748050_CAPN10	-0.16564	0.008821	0.023678
11746357_PARG	-0.18781	0.008824	0.023684
11719630_BRD2	0.116001	0.008824	0.023684
11736682_LHX1	-0.25145	0.008825	0.023686
11745215_KBTBD4	-0.21895	0.008827	0.023689
11754589_XPO6	0.126193	0.008827	0.023689
11731963_ZNF709	0.131327	0.008828	0.023689
11732842_KCNS2	-0.2597	0.008828	0.023689
11754083_BSG	-0.13282	0.00883	0.023692
11739845_MSR1	0.174325	0.008833	0.023697
11716624_XPO1	-0.16383	0.008833	0.023697
11717851_ZNF282	0.181962	0.008836	0.023704
11717783_PHF5A	-0.15521	0.008837	0.023706
11729869_EMILIN1	-0.20967	0.008839	0.023711
11733457_INTS2	0.169886	0.008841	0.023713
11749751_WIPF1	0.180457	0.008845	0.023721
11748061_PHF21A	0.202693	0.008845	0.023721
11763823_TAT	-0.21456	0.008849	0.023732
11752062_SUN2	-0.21166	0.00885	0.023733
11716477_LARP1	-0.11963	0.008852	0.023736
11722916_BMPR1A	0.174687	0.008853	0.023737
11722307_GLCE	0.15635	0.008853	0.023737
11747661_DMTN	-0.13843	0.008856	0.023743
11728172_MOB3C	0.204287	0.008861	0.023756
11735466_FGF17	0.156887	0.008863	0.023759
11759068_RIF1	-0.20956	0.008868	0.023772
11753134_SGK2	-0.26017	0.00887	0.023776
11719201_PDE6D	-0.13718	0.00888	0.023801
11735368_ZNF549	0.255295	0.008886	0.023816
11740959_DMD	0.281581	0.008892	0.023828
11762470_SH3GL3	-0.22984	0.008892	0.023828
11749121_IFNAR2	0.229787	0.008892	0.023828
11747266_TNNC2	0.22902	0.008896	0.023838
11715276_AURKAIP1	-0.17759	0.008901	0.023851
11740835_CYP27C1	-0.24571	0.008902	0.023851
11753074_PON3	-0.36394	0.008903	0.023852
11721789_PTPRD	0.288235	0.008905	0.023858
11732137_AATF	-0.11915	0.008912	0.023875
11737068_CCDC27	-0.20142	0.008921	0.023897
11732474_ALPI	-0.15543	0.008923	0.0239
11727100_NUMBL	-0.15747	0.008923	0.0239
11760743_DYNC1LI2	-0.39211	0.008924	0.023901
11757395_MCM3AP	0.170887	0.008925	0.023902
11740956_PLEKHN1	-0.20435	0.008928	0.023908
11731421_UBE2G2	0.16709	0.008931	0.023914
11717564_EMP1	-0.26887	0.008933	0.023919
11761238_LOC100506	-0.20899	0.008934	0.023921
11739296_ICOSLG //	-0.2024	0.008938	0.02393
11715848_ATP5G2	-0.11966	0.008938	0.02393
11743346_C11orf24	-0.11966	0.008941	0.023936
11718275_EIF3L	0.118088	0.008944	0.023942

11743076_PRKRA	0.122349	0.008945	0.023943
11721236_LHFPL2	0.217255	0.008946	0.023945
11718578_GSTM3	0.136276	0.008946	0.023945
11731854_C8G	-0.18799	0.008956	0.023968
11760809_CACYBP	-0.29214	0.008956	0.023969
11734339_POU3F1	-0.29849	0.008957	0.023969
11745171_DIAPH1	-0.11961	0.008957	0.023969
11728163_HSD17B3	-0.15723	0.008958	0.02397
11718825_MVB12B	-0.14707	0.008959	0.02397
11744655_AZIN2	-0.13242	0.008959	0.02397
11725900_LRRC8D	-0.18106	0.00897	0.023999
11721426_FZD5	0.205103	0.008974	0.024008
11744858_LHPP	-0.17771	0.008976	0.024011
11730053_SNX16	0.357322	0.008984	0.02403
11734555_NRBP1	0.142162	0.008986	0.024035
11723107_RBFA	-0.16324	0.008996	0.024061
11720244_PLCG2	-0.19215	0.008996	0.024061
11729449_TINF2	-0.15664	0.009009	0.024093
11735350_FAM71D	-0.14419	0.00901	0.024094
11743685_GPR21 ///	0.151716	0.009016	0.024109
11737695_KCNQ2	-0.19347	0.009019	0.024117
11757576_CSNK2B	0.139682	0.009025	0.024131
11720240_TMSB15A	-0.18754	0.009027	0.024134
11757237_HTR7P1	-0.23292	0.009029	0.024138
11740824_TPTE2	0.218241	0.009033	0.024148
11758293_RIC8B	-0.12383	0.009035	0.024153
11749206_TUB	0.277959	0.009036	0.024153
11758029_SUPT7L	0.18192	0.009038	0.024157
11757339_NR1H2	0.137073	0.009039	0.024159
11736866_MMP16	0.22039	0.009041	0.024163
11750747_SLC9A8	0.179909	0.009054	0.024197
11754816_GAS8	0.155936	0.009056	0.024201
11722962_LINS1	0.153174	0.00906	0.024209
11733544_LOC100996	-0.21977	0.009062	0.024215
11748585_XPO1	-0.11966	0.009063	0.024215
11745161_MED25	-0.11712	0.009065	0.024217
11753643_MED16	-0.14057	0.009065	0.024217
11748587_AFF1	0.267613	0.009065	0.024217
11749255_ACP2	-0.1639	0.009066	0.024217
11745513_ANO3	0.159258	0.009071	0.024228
11744554_CROT	0.147801	0.009072	0.024232
11715183_ZNF391	0.237391	0.009074	0.024234
11741841_C10orf71	-0.1793	0.009074	0.024234
11728043_PANX2	-0.20435	0.009076	0.024237
11731019_AIF1	-0.19971	0.009078	0.024241
11760308_OSBPL10	-0.14496	0.009079	0.024244
11734186_PPIL6	-0.2781	0.009081	0.024247
11718359_PEG10	-0.19254	0.009087	0.024262
11715445_DNAJB1	0.128135	0.009089	0.024266
11721843_ATL3	0.124609	0.009096	0.024284
200048_PM JTB	0.137589	0.009098	0.024286

11758640_FARSB	-0.16198	0.009099	0.024286
11762410_DNAJB14	0.191781	0.009099	0.024286
11747416_FGFR1	-0.19019	0.009101	0.024291
11739440_KRT4	-0.19452	0.009104	0.024297
11747805_BAG4	0.185667	0.009111	0.024315
11716420_SPTBN1	-0.13808	0.009112	0.024317
11745896_UPF2	0.13081	0.00912	0.024336
11715931_SGK1	-0.14613	0.009122	0.024339
11740099_ZNF37A	0.223015	0.009127	0.024352
11759086_RBM26	-0.19605	0.009133	0.024367
11716387_BAZ1B	-0.12592	0.009134	0.024367
11749605_CYP4F3	0.285587	0.009137	0.024375
11727743_CAMK2N2	-0.21501	0.009137	0.024375
11723791_APOC3	-0.22708	0.009143	0.02439
11752430_ADAM21 //	-0.1462	0.009145	0.024393
11761527_LPCAT3	-0.23939	0.009148	0.024399
11752095_PTPRC	0.165556	0.009155	0.024416
11736491_ICA1	0.225523	0.009155	0.024416
11722667_MAPT	-0.15478	0.009158	0.024423
11729033_ITPRIPL2	0.137127	0.009161	0.024429
11745074_GSTA4	-0.16337	0.009166	0.024439
11749059_RTN4	-0.14684	0.009168	0.024445
11733047_ABI1	-0.19312	0.009178	0.024471
11726736_PDLIM7	-0.15838	0.009179	0.024472
11752396_RAPGEF4	0.386099	0.009189	0.024498
11749754_EEF1A1	-0.45669	0.009191	0.024499
11742974_USP42	0.257132	0.009193	0.024505
11746043_TRA2B	-0.14868	0.009197	0.024513
11735520_FGF18	0.136491	0.009201	0.024523
11737843_SMIM11A /	0.18391	0.009202	0.024523
11745958_CD99	0.117515	0.009203	0.024527
11757356_RPL30	0.119678	0.009205	0.024529
11741199_LRRCC1	-0.35475	0.009208	0.024535
11754713_PLXNB1	0.116897	0.009209	0.024538
11723328_PTRH1	-0.18976	0.00921	0.024538
11757036_SAC3D1	-0.12971	0.009215	0.024552
11730898_WAPL	0.163384	0.009218	0.024557
11748324_VARS2	-0.37607	0.00922	0.024562
11750241_KNG1	-0.312	0.009221	0.024563
11727640_ZNF335	0.197456	0.009222	0.024564
11761989_ADGRF5	-0.23618	0.009222	0.024564
11728815_AARS2	-0.15079	0.009226	0.024572
11755221_VPS41	0.129191	0.009227	0.024572
11716471_GNL2	-0.13254	0.009236	0.024595
11737541_ZACN	-0.17449	0.009238	0.024601
11748792_GFRA3	-0.25706	0.009242	0.02461
11729086_TBX19	-0.17626	0.009247	0.024623
11752967_HSP90B1	-0.165	0.009252	0.024634
11723520_GRTP1	-0.13965	0.009254	0.024638
11745886_USP42	0.149532	0.009255	0.024639
11745941_PSD3	0.285857	0.009256	0.024641

11743332_MRPL35	-0.2817	0.009258	0.024643
11725267_FAT4	0.325688	0.009274	0.024686
11723389_C16orf74	-0.17889	0.009278	0.024695
11742711_THBS2	-0.27069	0.009281	0.024699
11738230_FOXR2	0.154085	0.009281	0.024699
11756356_FAM81B	-0.28859	0.009282	0.024701
11715662_TMEM189-U	0.116558	0.009283	0.024703
11725548_PDE4A	-0.19916	0.009284	0.024705
11746470_MAP4K4	0.154415	0.009298	0.024739
11757160_SNORA30	-0.1645	0.009304	0.024754
11755093_CLSTN1	-0.1151	0.009306	0.02476
11746254_TMTC4	-0.13364	0.009313	0.024775
11736432_PPP4R2	0.141691	0.009315	0.024778
11740206_GPR155	-0.4355	0.009318	0.024786
11722290_ZBTB43	0.149774	0.009319	0.024789
11748304_ENTPD6	0.127613	0.009322	0.024795
11761596_HERPUD1	-0.32228	0.009327	0.024805
11717365_SNTA1	-0.30369	0.009327	0.024805
11720695_C1orf54	-0.13888	0.009327	0.024805
11756865_PACRGL	-0.13229	0.009332	0.024816
11762217_LOC100132	-0.23677	0.009338	0.024829
11763324_VAMP4	0.191237	0.00934	0.024834
11724270_CACNB1	-0.15435	0.009341	0.024836
11755924_RAB11FIP4	-0.14827	0.009348	0.024854
11715619_SDF4	0.167256	0.009349	0.024854
11722939_PKN3	-0.26148	0.009361	0.024883
11762334_NFATC2IP	-0.40216	0.009364	0.024891
11749746_EZR	0.171224	0.009367	0.024897
11723270_KPNA6	-0.13975	0.009373	0.024911
11754674_RAB12	0.222752	0.009373	0.024911
11720426_WDR7	0.166917	0.009376	0.024919
11735870_TRIM64	-0.19913	0.009385	0.02494
11718523_ANTXR1	0.176941	0.009389	0.024949
11746984_INTS3	-0.12061	0.009392	0.024956
11743396_CSDE1	-0.12554	0.009394	0.024961
11762989_TRAV38-1	-0.18365	0.009395	0.024961
11726782_KATNBL1	0.258597	0.009397	0.024964
11718240_CAPRIN1	0.16346	0.009397	0.024964
11751577_TMED2	-0.15466	0.009408	0.024992
11741024_TBCCD1	0.144853	0.009409	0.024992
11716566_PRRC2C	-0.13746	0.009411	0.024997
11733653_FTMT	0.162715	0.009414	0.025005
11722969_TRPV4	-0.14421	0.009415	0.025005
11734839_GTF2F1	0.133424	0.009417	0.025008
11756676_NBPF22P /	0.231109	0.009417	0.025008
11762806_COMMD5 //	-0.1351	0.009419	0.025012
11742340_OR6T1	-0.21791	0.009436	0.025055
11744029_BBS4	0.139956	0.009442	0.02507
11747171_MXD4	-0.2331	0.009444	0.025073
11753585_HRH3	-0.14506	0.009445	0.025075
11753358_ACVR1	-0.12656	0.009447	0.025078

11720543_B3GALNT2	-0.11858	0.009451	0.025087
11725501_AHNAK	0.180096	0.009451	0.025087
11758627_ASS1	-0.23309	0.009453	0.025089
11749314_ABI1	0.125626	0.009453	0.025089
11745221_ATP2C1	0.135139	0.009454	0.02509
11734148_SLC6A4	-0.22008	0.009454	0.02509
11734759_HYAL2	-0.14598	0.009459	0.025101
11725037_SEC23IP	0.123609	0.009463	0.025111
11750510_EFNA3	-0.17321	0.009464	0.025111
11742644_SPATA31B1	-0.2086	0.009464	0.025112
11735818_THADA	0.123174	0.009466	0.025114
11716790_TARS	0.127915	0.009468	0.025116
11732128_CCT4	-0.14531	0.009468	0.025116
11731910_RIMBP2	-0.16961	0.009469	0.025118
11717782_PHF5A	-0.13727	0.009471	0.025121
11747684_SRSF1	-0.12339	0.009472	0.025123
11731178_FAM189A2	0.201537	0.009475	0.025129
11734561_WBSCR17	-0.21962	0.009478	0.025136
11719597_EHD3	0.146494	0.009481	0.025143
11747768_FCRL5	-0.29294	0.009483	0.025148
11763195_ARAF	0.183449	0.00949	0.025164
11758457_GPX4	-0.11692	0.009492	0.025167
11724137_ST3GAL3	-0.14744	0.009493	0.02517
11744651_NOX1	-0.18654	0.009495	0.025175
11759255_PCDHGB5	-0.31839	0.009497	0.025178
11760480_CIDECP	-0.17232	0.009499	0.025179
11732536_ACRV1	0.376788	0.009499	0.025179
11718988_RNASE4	-0.33089	0.009505	0.025195
11724178_DAAM2	-0.2623	0.009506	0.025195
11728627_DACH2	0.222843	0.009507	0.025196
11733638_CHM	0.245353	0.009508	0.025199
11724912_NDNF	-0.29739	0.009514	0.025213
11753269_DSTYK	0.205937	0.009515	0.025214
11753586_CXADR ///	0.351153	0.009521	0.02523
11727901_HPS1	0.131182	0.009526	0.02524
11756694_RNF44	0.13954	0.009527	0.025243
11726553_TMEM155	-0.14042	0.009532	0.025254
11730381_SNX18	0.150306	0.009535	0.025261
11730847_BTK	-0.12468	0.009536	0.025261
11732439_SMPDL3B	-0.17043	0.009537	0.025263
11762120_SUCLG2	0.528685	0.009544	0.025281
11715366_SEPT2	0.130542	0.009547	0.025287
11757083_CHCHD3	-0.12215	0.009549	0.025291
11715924_TRAM1	0.137781	0.009557	0.02531
11758821_HLA-DQB1	-0.12988	0.009558	0.025312
11761145_ZNF273	-0.22156	0.009559	0.025312
11716297_CLPTM1	0.153869	0.009559	0.025312
11754395_BRD4	0.126596	0.009561	0.025316
11741836_IL17C	-0.18368	0.009571	0.025339
11719247_AIM1	0.12735	0.009575	0.025349
11748925_LRRC7	0.324794	0.009575	0.025349

11738000_DMC1	-0.28992	0.009576	0.025349
11750987_TNIP1	0.199859	0.009578	0.025354
11758433_SLC4A1AP	0.130353	0.009585	0.02537
11731849_COLQ	0.13815	0.009586	0.025372
11753658_BACE1	0.228529	0.009591	0.025383
11755373_ECHDC2	0.152106	0.009592	0.025385
11734765_FGFR1	-0.17308	0.009597	0.025398
11760124_KLF6	-0.21983	0.0096	0.025404
11747947_AKT2	-0.23982	0.009603	0.02541
11737865_IGSF11	-0.2686	0.009605	0.025414
11752961_FAM168A	0.238432	0.009606	0.025416
11717313_IKBKAP	0.150855	0.009608	0.025419
11748574_RAB11B	-0.15429	0.009609	0.025421
11719106_ELMO2	0.114273	0.00961	0.025422
11717133_MAFG	0.140648	0.009622	0.02545
11740103_MAFG	0.140648	0.009622	0.02545
11738202_DAXX	0.124641	0.009623	0.025453
11715573_KDELR2	-0.15789	0.009626	0.025459
11722844_ENAH	-0.12687	0.009628	0.025463
11731367_TSPAN32	-0.15219	0.00963	0.025466
11717071_KTN1	0.222398	0.009649	0.025514
11739039_CEACAM5	-0.21963	0.009652	0.025521
11735175_NOS1	-0.17074	0.009654	0.025526
11715595_PSMB7	-0.14829	0.009661	0.025541
11758032_DBF4	-0.17954	0.009661	0.025541
11742924_PRDX1	-0.11458	0.009661	0.025541
11737940_RIPK1	-0.15332	0.00967	0.025562
11756445_R3HDM4	0.151951	0.009679	0.025584
11729723_UGT3A2	-0.24375	0.009683	0.025596
11744298_RCN3	-0.30796	0.009685	0.025598
11725773_TBC1D24	-0.13836	0.009687	0.025601
11751814_NME9	-0.17102	0.009687	0.025601
11757045_C7orf34	-0.22225	0.009694	0.025617
11757850_PA2G4	-0.11774	0.009695	0.025619
11754811_CTNNA1	0.121851	0.009696	0.025619
11753516_NADK2	0.129608	0.009696	0.02562
11725519_ICA1	0.123147	0.009698	0.025621
11759915_C11orf21	-0.27257	0.009698	0.025621
11758693_PSME2	0.128901	0.009701	0.025629
11723595_MAP2K7	-0.14531	0.009705	0.025636
11749538_MICB	-0.17263	0.009705	0.025636
11745422_FCRL5	-0.19783	0.009706	0.025638
11730756_TXNDC12	0.116129	0.009709	0.025642
11739971_NUAK1	0.24253	0.009719	0.025668
11758723_IDH2	-0.67845	0.00973	0.025695
11733460_PSG6	-0.11269	0.00973	0.025695
11719485_EPS15	-0.16027	0.009732	0.025698
11749835_SH2B1	-0.17486	0.009733	0.025701
11723461_NLE1	-0.16104	0.009737	0.025709
11743574_TMEM184C	0.126661	0.009743	0.025724
11753583_SCAND2P	0.266502	0.009761	0.025771

11757330_SNRPC	-0.11942	0.009773	0.0258
11750168_GLUD1	-0.1206	0.009774	0.025802
11717085_PRPS1	0.149225	0.009779	0.025813
11754916_CCDC115	-0.17002	0.00978	0.025814
11720702_PARP16	-0.14362	0.009783	0.025821
11746016_MSTO1 ///	-0.18802	0.009784	0.025822
11740750_HACD4	-0.18156	0.009791	0.025838
11761677_TAMM41	0.129703	0.009791	0.025838
11742643_MBD3L5	-0.2126	0.009792	0.025838
11751847_ORAOV1	0.310397	0.009793	0.025841
11725423_ZNF521	-0.28151	0.009798	0.025853
11716170_LMNB2	-0.42533	0.009801	0.025859
11720582_TCF12	0.137648	0.009806	0.02587
11727270_TRIM55	-0.19016	0.009818	0.0259
11746510_PSMD6	0.114782	0.009821	0.025908
11746949_HNRNPDL	-0.14659	0.009826	0.025919
11746997_PRRT3	-0.15565	0.009827	0.025919
11716524_PFDN2	-0.14238	0.009844	0.025964
11733807_KARS	0.114173	0.009845	0.025964
11733177_ZNF507	-0.38657	0.009845	0.025964
11715737_CASC3	-0.12583	0.009846	0.025965
11757739_RAB3GAP2	0.117847	0.00985	0.025975
11733529_ULK2	-0.14775	0.009853	0.025981
11756594_UROD	-0.14086	0.009856	0.025987
11741755_SEMA7A	0.200933	0.009865	0.026008
11725602_DENND5B	0.165921	0.009866	0.026009
11724841_PKN2	0.146779	0.009866	0.026009
11736897_PRRG4	0.478406	0.009867	0.026009
11750395_GLI2	0.253611	0.00987	0.026018
11732708_MSI2	0.34306	0.009874	0.026025
11718057_UTP11L	-0.16908	0.009874	0.026025
11759623_C11orf71	-0.19252	0.009875	0.026027
11739406_FPGS	-0.12317	0.009877	0.026028
11739668_SS18L1	0.171283	0.009877	0.026028
11750907_RAD1	-0.16533	0.009887	0.026052
11745427_RTEL1 ///	-0.2072	0.009889	0.026056
11745308_EPB41L4A-	-0.14535	0.00989	0.026059
11746112_MPHOSPH8	0.183826	0.009892	0.026062
11752830_ZNF747 //	-0.22781	0.009901	0.026085
11748418_RSPO1	-0.33411	0.009906	0.026095
11742028_ANGPT2	-0.13048	0.00991	0.026105
11721932_KIF23	-0.17807	0.00991	0.026105
11726975_PERP	-0.1343	0.009912	0.026109
11731063_DBF4B	-0.29115	0.009914	0.026112
11731986_XG /// XG	0.240085	0.009917	0.026118
11751904_COL11A1	0.440438	0.009921	0.026124
11728213_TRMT1L	0.173167	0.009921	0.026124
11727547_RBBP8	-0.162	0.009921	0.026124
11732500_FAM3B	-0.26014	0.009927	0.026136
11726443_KCTD6	-0.13975	0.009927	0.026136
11731576_CTBS	0.136215	0.009927	0.026136

11727172_TRPS1	-0.16606	0.009927	0.026136
11753189_MBOAT1	-0.38333	0.009933	0.026149
11743847_CFH	0.199131	0.009934	0.026151
11755429_KIAA1549	0.182542	0.009939	0.026162
11758597_AGPAT3	-0.19187	0.00994	0.026164
11734612_KRTAP10-4	-0.1263	0.009941	0.026165
11764157_TDRP	-0.18203	0.009943	0.026169
11716724_DPY19L1	-0.11726	0.009948	0.026179
11746614_ 7-Mar	0.116663	0.009949	0.026182
11732082_STOML3	-0.15706	0.009953	0.026192
11762863_HNRNPDL	-0.18635	0.009955	0.026194
11720752_OGDH	-0.13829	0.009959	0.026202
11733513_WLS	-0.13141	0.00997	0.026232
11729876_FAM209A /	0.271589	0.009972	0.026235
11720687_HOOK2	0.124678	0.009973	0.026235
11761853_LLPH	0.158136	0.009976	0.026243
11733240_IRAK2	0.242835	0.009979	0.026249
11763149_GYPB	-0.26226	0.009984	0.026261
11762151_NFATC2	-0.13672	0.009985	0.026263
11726320_ERO1A	0.156904	0.009992	0.026278
11757206_DLEU2	-0.15484	0.009993	0.02628
11763464_ZCCHC2	-0.21576	0.01	0.026298
11759080_GSR	-0.14021	0.010003	0.026305
11715660_RPN2	0.115361	0.010004	0.026305
11757508_ATP5SL	-0.26136	0.010009	0.026316
11736847_B4GALNT3	-0.12831	0.010009	0.026316
11728297_RNMT	0.150204	0.010012	0.026322
11744063_MUC5B	-0.12807	0.010015	0.026327
11743005_BRF2	0.188912	0.010016	0.026329
11731449_SEC62	0.163289	0.010019	0.026334
11755787_TCTN1	0.117957	0.010021	0.026339
11763039_OFCC1	0.13161	0.010025	0.026348
11745438_TTC3	0.134643	0.010026	0.02635
11724542_TKTL2	-0.24669	0.010028	0.026354
11725760_BORCS8	-0.14697	0.010032	0.026363
11741653_ABCG4	-0.31862	0.010037	0.026374
11757394_LOC102724	-0.16094	0.010043	0.026389
11737750_SGK1	-0.13613	0.010046	0.026394
11734153_DLX6	-0.43513	0.010046	0.026394
11735302_ADGRA1	-0.14474	0.010048	0.026397
11733304_ZMAT3	-0.13557	0.010056	0.026415
11732105_TMEM208	-0.13973	0.010056	0.026415
11717630_KIF1BP	-0.13133	0.010064	0.026436
11724342_LGALS7 //	-0.18462	0.010065	0.026436
11725502_LGALS7 //	-0.18462	0.010065	0.026436
11735180_DLX2	-0.15039	0.010074	0.026458
11763875_LOC100506	-0.20276	0.010075	0.026459
11755365_ERC1	0.151228	0.010084	0.02648
11745200_ATP1A3	-0.26012	0.010084	0.02648
11734242_KIF19	-0.16739	0.010086	0.026482
11716148_TRAF3IP2	0.125099	0.010087	0.026482

11730964_DAZAP1	-0.16284	0.010087	0.026482
11751395_SLC28A1	-0.17098	0.010087	0.026482
11760957_BCL7C	-0.18099	0.010088	0.026482
11750086_FGFBP2	-0.16087	0.010089	0.026485
11730107_PLCB1	0.165713	0.010091	0.026487
11756704_ZNF284	0.238735	0.010092	0.026489
11763173_PGM3	-0.169	0.010093	0.02649
11728727_CEACAM7	-0.19756	0.010094	0.026492
11724751_MRTO4	0.146905	0.0101	0.026506
11760668_STX17	-0.33156	0.010105	0.026518
11725917_RNF19B	0.189205	0.010107	0.026522
11758994_FAR2	0.137482	0.010113	0.026537
11749857_KAZN	0.27871	0.010118	0.026548
11727264_LRRC8E	-0.18725	0.01012	0.026551
11732431_MFAP3L	-0.21291	0.010121	0.026551
11734678_DLGAP4	0.174335	0.010121	0.026552
11743440_PDIA3	-0.13516	0.010122	0.026553
11727307_ZNF320	0.226612	0.010124	0.026556
11755397_HDAC9	0.309393	0.010124	0.026556
11716967_NRP2	-0.13969	0.010126	0.026557
11722526_PTPN21	0.217958	0.010127	0.02656
11745229_TRMT2B	-0.22871	0.010129	0.026561
11717362_GPC1	-0.16446	0.010129	0.026561
11753867_NDUFA1	-0.12827	0.010129	0.026561
11760225_KIZ	-0.25647	0.010138	0.026583
11717712_SDHAF1	0.138799	0.010148	0.026607
11742764_BCAS2	-0.15055	0.010155	0.026625
11731941_PRSS33	-0.23312	0.010156	0.026625
11746571_SLC25A47	-0.1642	0.010156	0.026625
11756806_ISG20	0.13632	0.01016	0.026632
11726775_POU2F1	0.156051	0.010168	0.026653
11726529_BRCC3	0.165135	0.010173	0.026664
11753776_LAIR2	0.15707	0.010175	0.026669
11756671_PGAP2	-0.15419	0.010176	0.026669
11735776_HIST1H4K	0.159117	0.010178	0.026673
11721012_SMOC1	-0.15943	0.01018	0.026676
11745293_PTPN23	-0.17978	0.010184	0.026685
11734142_METTL8	0.152208	0.010186	0.02669
11716670_RAB1A ///	-0.14697	0.01019	0.0267
11748220_SIRT2	0.144468	0.010191	0.0267
11761252_SUCO	0.132179	0.010193	0.026703
11747939_BPHL	0.207717	0.010193	0.026703
11738227_HRNR	-0.20577	0.010194	0.026703
11749651_RECQL5	-0.17985	0.010195	0.026706
11741348_DLG4	0.169942	0.010197	0.026709
11720199_DVL2	-0.15627	0.010205	0.026729
11718494_SLC1A2	-0.18913	0.010211	0.026742
11715944_TSG101	0.124749	0.010219	0.02676
11728309_ARPP21	-0.14945	0.010219	0.02676
11718018_ADARB1	0.168451	0.010219	0.02676
11762087_EVX1	-0.13384	0.010224	0.026771

11763128_YWHAQP8	-0.18278	0.010226	0.026773
11763129_YWHAQP8	-0.18278	0.010226	0.026773
11733503_TBC1D16	-0.13477	0.010227	0.026774
11723925_KRT13	-0.16966	0.010229	0.026777
11724520_ZNF526	0.119386	0.01023	0.026777
11728543_MRO	-0.15934	0.01023	0.026777
200078_PM_ATP6V0B	0.126294	0.010236	0.026791
11750127_PHF14	0.174442	0.010238	0.026797
11750647_ASCC3	0.279252	0.010241	0.026802
11716284_GLO1	-0.12002	0.010245	0.02681
11753524_RPL14	0.118894	0.010248	0.026818
11739204_TTL	0.147724	0.010249	0.026818
11747115_EHHADH	-0.24173	0.010249	0.026818
11736073_ALDH1B1	-0.17166	0.010252	0.026821
11749417_CEP128	-0.19349	0.010252	0.026821
11724105_C8orf44-S	-0.1359	0.010252	0.026821
11727898_CLMN	-0.28095	0.010254	0.026825
11743134_FKBP8	-0.13209	0.010255	0.026825
11757921_COL14A1	0.163636	0.010257	0.026829
11723636_C11orf58	-0.14345	0.010258	0.026831
11724869_IPO8	-0.13633	0.010261	0.026837
11736324_CD96	0.139528	0.010264	0.026843
11739770_TMOD2	-0.24219	0.010265	0.026844
11722033_SLC12A4	0.266661	0.010265	0.026844
11741844_NPAS3	0.143825	0.010267	0.026849
11738363_ANKUB1	-0.19102	0.010271	0.026856
11757396_ANXA8 ///	-0.1185	0.010272	0.026856
11757749_ARNT	0.221244	0.010272	0.026856
11726331_CEP170	0.127848	0.010274	0.026858
11736109_ARL8A	0.184679	0.010274	0.026858
11739089_OSBPL5	-0.15975	0.01028	0.026873
11725274_RGS20	0.238215	0.010284	0.026881
11722396_RWDD4	-0.15649	0.01029	0.026895
11716911_NR2F6	-0.14224	0.01029	0.026895
11754199_INPPL1	-0.1817	0.010293	0.026902
11762968_MEI1	-0.18784	0.010298	0.026912
11720908_LRRC16A	0.187274	0.010298	0.026912
11731135_LTB4R	0.223297	0.010299	0.026912
11762549_MCTP1	0.149893	0.010305	0.026926
11742112_C1orf141	-0.1665	0.010306	0.026927
11756831_CXXC1	-0.15834	0.010307	0.026927
11750208_DCX	-0.16826	0.010307	0.026927
11741551_GOLGA6A /	-0.20448	0.010309	0.026932
11762099_IGHA1	-0.20686	0.010312	0.026937
11744049_MAP1B	0.131433	0.010318	0.026951
11759938_ITFG2 ///	-0.14599	0.010319	0.026954
11756220_ROBO3	-0.13423	0.010326	0.026968
11715420_CCT2	0.121123	0.010326	0.026968
11723485_GTSF1	-0.13519	0.010326	0.026968
11761860_AUP1	0.186663	0.010332	0.02698
11727520_INTS6	-0.156	0.010338	0.026996

11722941_MUC2	-0.18412	0.01034	0.026999
11757599_NUTF2	-0.11863	0.010342	0.027002
11720846_CD248	-0.18921	0.010346	0.027012
11757221_RPS2P32	-0.23302	0.010349	0.027018
11760122_RBM14-RBM	0.267767	0.01035	0.027019
11734556_DDX27 ///	0.138491	0.010354	0.027027
11729356_PAPD4	-0.19803	0.010354	0.027027
11725790_DPYD	0.1177	0.010355	0.027028
11728824_SLC25A3	0.119784	0.010358	0.027032
11763381_STYXL1	-0.18821	0.010358	0.027032
11744547_MED16	-0.12414	0.010359	0.027032
11745255_FBXO16	-0.20623	0.010359	0.027032
11735435_GPR158	0.172199	0.010365	0.027047
11758109_ZNF428	-0.1999	0.010367	0.027052
11716181_MED15	-0.11943	0.01037	0.027056
11754384_COG4	0.128814	0.010372	0.027062
11730434_SRSF12	-0.21713	0.010374	0.027065
11759771_UCHL5	0.145461	0.010377	0.027071
11750981_SHKBP1	-0.14997	0.010378	0.027074
11746648_IKBKB	-0.14398	0.010386	0.027092
11753149_CELF2	0.146822	0.010395	0.027114
11749069_PAQR4	-0.16666	0.010396	0.027114
11749602_HPN-AS1	-0.19269	0.0104	0.027123
11722744_BORCS8	-0.32957	0.0104	0.027123
11731358_ADAMTS3	-0.33561	0.010407	0.027139
11748828_PIK3R3	0.18264	0.010409	0.027142
11729211_ORC2	-0.16187	0.010416	0.027161
11738779_EPGN	0.152499	0.01042	0.02717
11758675_RABL2A //	0.183772	0.010421	0.02717
11742829_PHF3	-0.12358	0.010427	0.027184
11718619_CHRDL1	-0.34972	0.010427	0.027184
11744822_NDUFB2	0.138571	0.010437	0.027207
11722357_ACADM	0.142972	0.010441	0.027216
11755276_GPX2	-0.18762	0.010443	0.027221
11748910_LOC100129	0.138547	0.010444	0.027222
11729777_TMTC2	0.177225	0.010449	0.027232
11754443_GTF2F1	0.124841	0.010451	0.027237
11719875_MTX1	-0.15576	0.010455	0.027245
11738192_MTFR1	-0.13175	0.010465	0.027269
11746753_CCDC180 /	-0.17508	0.010471	0.027285
11754491_C16orf46	0.237226	0.010478	0.027302
11749983_SERPINB5	-0.23858	0.010485	0.027317
11752510_PSAP	0.127212	0.010491	0.027333
11760916_MLH1	-0.20476	0.010492	0.027333
11726453_TIGD5	-0.1258	0.010495	0.02734
11727474_TMEM260	0.203216	0.010497	0.027344
11752977_SYNRG	0.173864	0.0105	0.027349
11747628_VMP1	0.118719	0.010507	0.027366
11720345_USP4	0.128396	0.010516	0.027388
11739214_SLC25A23	-0.19089	0.010518	0.027393
11757486_NIT1	0.123419	0.010519	0.027395

11716548_FGFR1	0.156395	0.01052	0.027395
11733949_PAX9	0.260334	0.010523	0.0274
11733777_DUSP3	-0.1537	0.01053	0.027419
11744034_VASP	0.12352	0.010531	0.02742
11758753_STARD3	-0.20224	0.010533	0.027422
11718788_ADA	0.11736	0.010535	0.027426
11750110_MYO18A //	-0.13472	0.010536	0.027429
11751965_ESYT3	-0.30774	0.010545	0.027448
11754742_CWF19L1	-0.24751	0.010545	0.027448
11722070_CHPT1	-0.13007	0.010546	0.027448
11727811_SRSF1	-0.12273	0.010547	0.027449
11748503_IFT122 //	0.134594	0.010547	0.027449
11733273_MICB	-0.13838	0.010549	0.027453
11748953_SLC2A2	-0.11359	0.01055	0.027455
11732603_TRIM45	-0.2638	0.010554	0.027463
11725986_NONO	0.119111	0.010556	0.027466
11732666_PSMB5	-0.12845	0.010559	0.027474
11762944_IL19	-0.27537	0.010567	0.027491
11717894_PTP4A1	0.151808	0.010572	0.027504
11747239_LZTS2	-0.12928	0.010573	0.027506
11732343_ZNF33A	-0.17249	0.010576	0.02751
11717330_BMS1	0.126004	0.01058	0.027521
11752274_MYH2	0.14954	0.010583	0.027527
11742543_OR6Y1	-0.20858	0.010584	0.027527
11761538_TSTA3	0.169294	0.010584	0.027527
11763211_LINC00654	-0.23663	0.010586	0.027531
11746422_C9orf3	0.319737	0.010589	0.027537
11740867_BTBD19	0.368369	0.010595	0.027551
11722540_LCLAT1	0.141308	0.010596	0.027551
11759096_GNAQ	0.159052	0.010597	0.027552
11757916_TBX3	0.137095	0.010597	0.027552
11725727_C1orf56	-0.15914	0.010603	0.027565
11752817_TPP1	0.200433	0.010603	0.027565
11738033_PCDHGB4	-0.15768	0.010607	0.027572
11734777_PCTP	-0.2187	0.010617	0.027597
11716526_SPON2	-0.14917	0.010619	0.0276
11724527_FAHD1	0.174681	0.010619	0.0276
11758662_ZNF358	-0.16096	0.01062	0.0276
11722263_MRPS10	0.151423	0.010627	0.027616
11728004_RALGPS2	0.115085	0.010635	0.027638
11740935_BANP	0.112038	0.010637	0.02764
11728742_ARAP3	0.152438	0.010637	0.02764
11727027_ATG7	0.174938	0.010648	0.027666
11758957_WWP1	0.174212	0.010651	0.027672
11718075_IL4R	0.217526	0.010652	0.027674
11742824_TMEM199	-0.15308	0.010666	0.027709
11733863_TMOD1 ///	0.156292	0.010672	0.027722
11736116_FGFR1OP2	0.144462	0.010673	0.027723
11757187_PLD2	-0.14097	0.010674	0.027725
11746965_ANPEP	0.127448	0.010678	0.027733
11719560_SOX4	-0.17939	0.010679	0.027736

11723662_SPHK2	-0.26035	0.010684	0.027746
11740209_ZSCAN12	0.20679	0.010692	0.027766
11734844_MPHOSPH8	0.121114	0.010693	0.027766
11721646_ARL1	-0.12182	0.010695	0.027769
11747818_ARL1	-0.12182	0.010695	0.027769
11758775_SRPRB	0.159405	0.010695	0.027769
11759975_XRCC6BP1	-0.16367	0.010701	0.027782
11727332_CATSPER3	-0.29412	0.010702	0.027782
11738302_KRTAP3-1	0.16539	0.010704	0.027786
11747207_ELAVL1	-0.11604	0.010708	0.027795
11747728_SYP	0.175831	0.01071	0.027799
11744900_FADS2	-0.14524	0.010716	0.027813
11720070_TIMM23	0.112408	0.010722	0.027827
11722699_KIAA1109	0.280489	0.010726	0.027836
11746751_RAPGEF6	0.24823	0.01073	0.027846
11746857_WDR55	0.211321	0.010732	0.027849
11744668_SLC4A2	-0.14452	0.010738	0.027863
11749820_CHRNB1	0.187688	0.01074	0.027866
11715296_KRT33A	-0.20259	0.010745	0.027878
11715398_APEX1	-0.11632	0.010747	0.027882
11732922_GJA3	0.256793	0.010751	0.027891
11722195_NMT1	0.121917	0.010757	0.027905
11758758_SPINT1	-0.15877	0.010758	0.027907
11743034_RDH11	-0.16606	0.010759	0.027907
11719462_HNRNPA1 /	-0.12408	0.01076	0.027907
11726951_FAM131B	-0.22123	0.010766	0.027922
11760558_COL4A1	0.162458	0.010767	0.027924
11717149_USB1	-0.16996	0.01077	0.027929
11752862_KLF8	0.196468	0.010775	0.027942
11728200_LOX	0.168431	0.010777	0.027944
11740021_ACBD7	-0.13846	0.010777	0.027944
11744178_C19orf66	0.172118	0.01078	0.02795
11723923_IPO13	0.181018	0.010781	0.027952
11745675_TRPV4	-0.14169	0.010784	0.027955
11727286_ZSCAN31	0.234566	0.010784	0.027955
11749822_FAM167A	-0.19299	0.010786	0.027959
11728667_PCBP2	-0.13377	0.010787	0.027961
11717063_SMARCD2	-0.26591	0.010788	0.027962
11726374_GPR34	-0.13007	0.01079	0.027965
11753018_HSPD1	-0.11941	0.010792	0.027968
11758931_HEATR5A	0.249702	0.010794	0.027973
11727108_DROSHA	-0.148	0.010795	0.027973
11717148_C11orf31	0.141984	0.010806	0.028
11757877_KIF1A	-0.19758	0.010807	0.028
11756366_EWSR1	-0.13008	0.010807	0.028
11754621_EWSAT1	0.263127	0.010807	0.028
11749329_ARHGAP9	-0.27384	0.010809	0.028004
11746948_HNRNPDL	-0.1405	0.01081	0.028004
11716080_CALCOCO1	-0.15665	0.010814	0.028012
11725108_POLR3GL	-0.14681	0.010818	0.02802
11719200_PDE6D	-0.13785	0.010818	0.02802

11751431_C7orf60	0.203515	0.01082	0.028025
11756310_NXT2	-0.2284	0.010821	0.028025
11724290_ZNF641	0.385802	0.010822	0.028025
11717930_FOLR1	0.261425	0.010825	0.028032
11751195_KCNQ5	0.224107	0.010827	0.028037
11752154_NBEAL2	-0.12954	0.010828	0.028038
11743683_MFSD14A	-0.184	0.010847	0.028085
11749693_ARHGAP17	0.131125	0.01085	0.028091
11735632_KRT82	-0.19742	0.010856	0.028104
11751255_GUSBP11	-0.15786	0.010859	0.028112
11737649_ANKFY1	0.231861	0.010865	0.028126
11716322_PRKCDBP	-0.16664	0.010867	0.028129
11756898_PGM5	0.258506	0.010879	0.028159
11716389_BAZ1B	-0.11351	0.010884	0.028171
11744518_SLC2A10	-0.26391	0.010889	0.028181
11741502_HSD3B7	-0.15461	0.01089	0.028183
11746233_PI4KA ///	-0.12006	0.010895	0.028193
11719055_CEP350	0.202523	0.010899	0.028202
11748988_REEP5	-0.20032	0.010901	0.028206
11749698_STRA6	0.141133	0.010902	0.028207
11736011_MGAT2	0.130985	0.010906	0.028217
11762751_WASH1 ///	-0.20345	0.010914	0.028235
11754174_SNX12	0.175191	0.010916	0.02824
11759356_KCNH5	-0.15884	0.010919	0.028246
11724805_LAIR1	-0.18781	0.01092	0.028246
11760431_RASSF8	-0.19793	0.010921	0.028248
11718955_FBLIM1	0.282666	0.010926	0.02826
11738398_LHX6	-0.44799	0.01093	0.028269
11752319_EIF2AK1	-0.24937	0.010932	0.028271
11733208_ZNF480	0.12669	0.010932	0.028271
11720446_NSD1	-0.19645	0.010934	0.028275
11753109_CDH11	-0.19501	0.010937	0.02828
11742080_SMARCD2	-0.15389	0.010939	0.028283
11746391_C5orf28	0.190368	0.01095	0.028309
11720289_CDC42BPA	0.11683	0.01095	0.028309
11759897_OFD1	0.169023	0.010955	0.02832
11719216_TDRD7	0.160758	0.010956	0.02832
11759709_NSUN5P1 /	0.186258	0.010956	0.02832
11717993_CBX7	-0.20024	0.010958	0.028323
11742438_NLRP8	-0.19962	0.010966	0.028344
11763226_CXCL8	0.15865	0.010967	0.028346
11743286_DTX4	-0.21278	0.010968	0.028346
11751242_FCGR2A //	0.199228	0.010971	0.028352
11718745_ARHGAP21	-0.3012	0.010972	0.028353
11719663_GDI1	0.124931	0.010975	0.028358
11730890_NSUN6	-0.17978	0.010979	0.028367
11761851_RANBP1	-0.15072	0.010983	0.028377
11721439_TOR1AIP1	-0.14952	0.010984	0.028377
11757901_CXXC5	-0.15648	0.010984	0.028377
11742709_P4HTM	-0.2035	0.010991	0.02839
11722928_AP5Z1	0.15642	0.010991	0.02839

11726135_CRB3	-0.14111	0.011003	0.028421
11741469_NPC1L1	-0.13938	0.011004	0.028422
11758955_LGALS8	0.140173	0.011008	0.028432
11748999_PARP11	0.171269	0.011013	0.028441
11759438_EIF3F	0.246559	0.011016	0.028447
11728289_TBC1D2	0.13428	0.011017	0.02845
11718370_NRBF2	0.160916	0.011028	0.028477
11720822_ASL	-0.14647	0.011037	0.028497
11738180_BTBD9	0.203844	0.011038	0.028498
11729397_GOSR2	0.221686	0.011042	0.028507
11739215_SLC25A23	-0.17771	0.011044	0.028511
11725641_EFHD2	0.170567	0.011046	0.028515
11755289_MORF4L2	0.112105	0.011047	0.028515
11738043_MPV17L	-0.23122	0.011047	0.028515
11726034_PTPN6	-0.23857	0.011049	0.028519
11746930_BHLHE41	0.15775	0.01105	0.028519
11751051_DDX24	0.117299	0.011058	0.028538
11754201_ALDH7A1	0.139991	0.011058	0.028538
11736515_HTR5A	-0.14947	0.011064	0.028551
11718185_FCHSD2	-0.23002	0.011065	0.028551
11721382_ATG2B	-0.15549	0.011071	0.028566
11750985_EXTL2	-0.16158	0.011078	0.028583
11748062_CCR5	-0.29141	0.011084	0.028596
11721683_MRPS9	0.122578	0.011085	0.028598
11725106_AZIN1	0.115344	0.011089	0.028607
11741120_ELK1	-0.16908	0.011093	0.028615
11747353_CUL1	0.158698	0.011094	0.028617
11743697_TEX30	-0.14227	0.011095	0.028618
11738169_ANKRD30B	0.260186	0.011104	0.02864
11742328_NR1I3	0.186732	0.011107	0.028645
11746795_NADK2	0.178666	0.011116	0.028666
11716056_STMN3	0.188665	0.01112	0.028677
11738128_SARDH	-0.19528	0.011122	0.028677
11756558_DXO	0.122493	0.011122	0.028677
11742885_BAG5	-0.14747	0.011124	0.028682
11730265_CCDC69	0.159	0.011128	0.028689
11733421_LRRFIP2	0.24512	0.011131	0.028698
11760396_ZNF333	-0.25109	0.011134	0.028704
11728718_LTBP1	-0.16163	0.011135	0.028704
11753894_CNPPD1	-0.16345	0.011139	0.028712
11756863_DDX3Y	0.198498	0.011143	0.028721
11733852_EGLN3	0.20905	0.011144	0.028724
11743824_TOPBP1	-0.12297	0.011146	0.028724
11757628_TSPYL2	0.123439	0.011146	0.028724
11761287_HDAC1	0.172835	0.011146	0.028724
11748768_NR1H2	0.149805	0.011154	0.028742
11750671_MMRN2	-0.15838	0.011157	0.02875
11746894_MPP1	-0.1242	0.011159	0.028752
11756890_ATP5A1	-0.16185	0.011163	0.028761
11716180_VPS25	0.119398	0.01117	0.028776
11744211_NELFE	-0.15708	0.01117	0.028776

11737073_COPE	0.123175	0.011173	0.028783
11726965_L3MBTL3	-0.35832	0.011175	0.028785
11752919_ACADM	0.134843	0.011187	0.028814
11716329_GJA1	-0.24159	0.011188	0.028816
11750904_RBM6	0.185382	0.01119	0.028821
11746911_CALCOCO2	-0.12572	0.011195	0.028831
11753830_MLLT11	-0.12723	0.011199	0.02884
11728268_FAAP100	-0.15799	0.011204	0.028851
11755975_PKIB	0.179777	0.011204	0.028851
11757825_C7orf55 /	-0.12392	0.011211	0.028865
11752958_MPP1	-0.14149	0.011215	0.028876
11744182_FARS2	0.144883	0.011218	0.028881
11738460_NUTM2F //	-0.23256	0.011221	0.028886
11721400_ELOF1	-0.12207	0.011225	0.028896
11737941_HDHD2	-0.23646	0.011225	0.028896
11757941_THBS2	-0.26802	0.011227	0.028899
11719686_MICAL1	0.174904	0.011236	0.02892
11749207_SH2D1B	0.1773	0.011239	0.028925
11762256_AKR1E2	0.11027	0.011239	0.028925
11749283_MAGEA2 //	-0.12453	0.01124	0.028926
11748989_TMEM35	-0.11686	0.011242	0.028929
11751994_JAG1	0.255963	0.011244	0.028934
11746669_FAM118A	0.201594	0.011248	0.028943
11753818_MUC1	-0.22254	0.011252	0.028949
11753630_ARL1	-0.15791	0.011252	0.028949
11761432_LOC100506	-0.16344	0.011254	0.028952
11742166_KRTAP7-1	-0.20698	0.01126	0.028967
11741245_OTUD5	0.139006	0.011263	0.028972
11753746_CHRAC1	-0.12852	0.011266	0.028979
11743015_DIP2C	0.194381	0.011271	0.02899
11740989_HYAL1	-0.20687	0.011272	0.028992
11758856_ZNF689	0.148475	0.011273	0.028992
11747799_ENO1	-0.1159	0.011274	0.028993
11732228_PEX10	-0.12136	0.011278	0.029001
11723985_PDCD11	0.137442	0.011278	0.029001
11757827_TTC9B	-0.13373	0.011281	0.029007
11726349_ANKRD2	-0.14051	0.011288	0.029023
11747914_MAP4	-0.12425	0.01129	0.029025
11721674_GLRX5	-0.12902	0.01129	0.029025
11730971_CYP4F12	-0.16269	0.011306	0.029066
11719526_LZTR1	-0.14244	0.011323	0.029106
11755002_FAAP100	-0.11662	0.011323	0.029106
11759456_TUBB8	-0.19581	0.011326	0.029112
11719848_C7orf25	0.140401	0.011333	0.029128
11740511_PDE4D	0.236593	0.011335	0.029133
11722493_HIP1R	-0.16922	0.011339	0.02914
11736243_MRPL52	0.11583	0.011347	0.029161
11753417_HLA-G	-0.17141	0.011355	0.029178
11742289_GABRR3	0.206983	0.011357	0.029182
11717198_MRPL14	0.13122	0.011358	0.029184
11748123_ZNF691	0.128918	0.01136	0.029186

11719333_DMKN	0.242357	0.011363	0.029193
11750690_VAC14	-0.26243	0.011364	0.029195
11750075_SP4	-0.21182	0.011367	0.029201
11746139_AKT2	-0.24374	0.01137	0.029207
11724774_GHDC	-0.14223	0.01138	0.029231
11738717_OR4C12	-0.27194	0.011392	0.02926
11757398_LINC00657	-0.14247	0.011395	0.029266
11762473_LOC100131	-0.18783	0.011395	0.029266
11755278_CYP2S1	-0.25157	0.0114	0.029274
11726213_CCL15	0.172034	0.0114	0.029274
11737605_ZBTB8B	-0.21678	0.011401	0.029276
11742272_TM6SF1	-0.20032	0.011406	0.029287
11720430_ORMDL1	0.17466	0.011409	0.029293
11731667_PDGFB	0.197157	0.011413	0.029301
11726357_KMT2E	0.120523	0.011425	0.02933
11759602_RSRP1	0.372301	0.011428	0.029338
11749474_AACS	0.125452	0.011433	0.029348
11739897_ZNF280C	0.319225	0.011439	0.029362
11723248_LRRC40	-0.12685	0.01144	0.029362
11723333_FOXA1	-0.25162	0.011444	0.029372
11718608_UBA7	0.155771	0.01145	0.029385
11745764_SEC31A	0.132417	0.011451	0.029386
11749371_CDADC1	0.192773	0.011459	0.029405
11736933_POU2F3	-0.20375	0.011464	0.029418
11718805_PUM2	0.154406	0.011467	0.029423
11718172_C2CD5	-0.13597	0.011469	0.029426
11754399_RNF121	-0.26503	0.011471	0.029428
11717396_CHTF8	-0.13381	0.011471	0.029428
11759969_STAT5B	-0.12015	0.011471	0.029428
11757282_ATP5G3	-0.1178	0.011476	0.029438
11755337_ENPP2	0.218149	0.011492	0.029479
11726448_ZNF614	0.192113	0.011495	0.029483
11760961_EML2	-0.14007	0.011498	0.02949
11720187_SLC27A3	-0.19486	0.011498	0.02949
11723029_MEST	-0.20113	0.0115	0.029493
11749384_COG3	0.192272	0.011501	0.029494
11758231_HLA-DPA1	-0.13297	0.011503	0.029498
11746141_ARHGEF26	0.241042	0.011504	0.029498
11751070_TOMM40	-0.20776	0.011505	0.029498
11720855_NFIA	0.203036	0.011509	0.029509
11716204_ASNA1	-0.15043	0.01151	0.029509
11743443_RHOB	0.24134	0.011512	0.029512
11723538_KNOP1	-0.13465	0.011512	0.029512
11731776_CCL11	-0.20248	0.011525	0.029542
11734491_SPATA24	-0.16287	0.011525	0.029542
11749462_ZNF845	0.221712	0.011527	0.029545
11715432_TMBIM1	-0.19004	0.01153	0.02955
11737976_ZSCAN23	-0.12801	0.011534	0.029559
11739009_MYH9	0.121896	0.011537	0.029565
11757295_KIAA0391	-0.11666	0.011539	0.02957
11749489_IFT140	-0.17367	0.011551	0.029599

11754279_MYEF2	0.170458	0.011552	0.0296
11729787_SOX13	-0.14917	0.011559	0.029616
11728704_KIAA0232	-0.22552	0.011562	0.029621
11730151_PATE1	-0.20787	0.011569	0.029637
11751654_KIAA1456	-0.19641	0.011579	0.029661
11742818_PLAUR	0.155999	0.011579	0.029661
11721505_SARS2	-0.13819	0.01158	0.029661
11727593_KIAA2026	0.202695	0.011583	0.029667
11715641_TTC3	0.159102	0.011583	0.029667
11763261_PKIA	0.228563	0.011587	0.029674
11724447_PDE4D	-0.36241	0.011592	0.029687
11726653_ARHGAP32	-0.22482	0.011594	0.02969
11749070_PAQR4	-0.1824	0.011601	0.029706
11738380_FGF23	-0.19713	0.011603	0.029711
11759905_EXD3	-0.14563	0.011606	0.029714
11752631_SASH3	-0.19671	0.011606	0.029714
11754551_ENO1	-0.13121	0.011607	0.029715
11727615_SCN2B	-0.14145	0.011608	0.029716
11753861_PRICKLE4	-0.12695	0.011609	0.029716
11727849_NIP7	0.15271	0.01161	0.029716
11728428_CCDC136	0.129998	0.01161	0.029716
11718589_CHMP6	-0.15409	0.011611	0.029718
11761710_HSFY1 ///	-0.19623	0.011613	0.029721
11724572_APPBP2	0.181003	0.011615	0.029725
11743426_AHCYL1	-0.1275	0.011625	0.02975
11720709_KIF1B	0.156086	0.011631	0.029762
11736077_NEK9	-0.22166	0.011631	0.029762
11756593_UROD	-0.13887	0.011633	0.029766
11756579_PSMD1	-0.11704	0.011637	0.029773
11719027_PSD3	0.174544	0.011642	0.029785
11740130_CACNA1B	-0.13694	0.011643	0.029785
11726420_HFE2	-0.3703	0.011644	0.029788
11757909_XAB2	-0.27743	0.011648	0.029797
11727203_ZNF230	0.168789	0.011653	0.029807
11719521_SEMA5A	0.240188	0.011656	0.029812
11757379_TYK2	-0.17731	0.011669	0.029845
11751968_FAM193A	0.11788	0.01167	0.029846
11715759_RPL36AL	0.122632	0.011679	0.029867
11720693_IL17D	0.140691	0.01168	0.029869
11732987_IFNA6	-0.17623	0.011682	0.029872
11747306_ELP5	-0.13778	0.011686	0.029879
11762927_HSP90AA5F	-0.16231	0.011694	0.029899
11722391_MTF1	-0.31495	0.01171	0.029937
11715800_PRRC2B	-0.20479	0.011714	0.029945
11736557_TMEM121	-0.15343	0.011714	0.029945
11735582_ZSCAN2	-0.16343	0.011714	0.029945
11719904_ANKRD40	0.141144	0.011725	0.029971
11760013_CCDC88C /	-0.27737	0.011727	0.029975
11758295_CAPNS1	0.112641	0.01173	0.029981
11729276_CRCP	0.115539	0.011735	0.029991
11736341_EGR3	-0.12375	0.01174	0.030002

11747005_EML4	0.21269	0.011747	0.030018
11748685_NEBL	-0.14726	0.011747	0.030018
11741948_NF1	0.2782	0.011748	0.030019
11718779_S100A2	-0.13113	0.01175	0.030021
11737281_ZC3HAV1L	0.333227	0.011755	0.030032
11716207_GLUD1 ///	-0.11663	0.011756	0.030032
11753452_PLCD4	-0.18915	0.011756	0.030032
11730002_NRTN	-0.12445	0.01176	0.030039
11756279_FAM118B	0.130304	0.01176	0.030039
11728059_SERTAD3	0.141615	0.01176	0.030039
11745353_C2orf54	-0.13685	0.011762	0.030041
11721613_KIT	-0.13004	0.011764	0.030044
11762138_PPHLN1	0.293423	0.011765	0.030047
11737016_PPP1R12B	0.187739	0.011766	0.030048
11727491_ZNF350	0.205878	0.011768	0.030049
11759065_ZNF576	-0.2224	0.011768	0.030049
11753382_ZC4H2	-0.1276	0.01177	0.030052
11746850_STAT6	-0.17759	0.011773	0.030057
11729812_ZADH2	-0.29733	0.011777	0.030067
11742345_OR2T11	-0.15089	0.011779	0.030069
11755157_KLHL8	0.188268	0.011779	0.030069
11762503_ADGRG6	0.10814	0.011787	0.030085
11732430_PDE7A	-0.43442	0.011787	0.030085
11716096_SGTA	-0.14517	0.011787	0.030085
11761644_PIGP	-0.13791	0.011789	0.030088
11747168_CIB2	-0.44878	0.01179	0.03009
11743669_ZNF292	0.180872	0.011795	0.0301
11749725_NR4A1	-0.14247	0.011807	0.030131
11752452_NDST4	-0.16382	0.011812	0.03014
11764054_FMR1	0.131716	0.011813	0.030142
11748244_ZNF300	0.140898	0.011815	0.030145
11723513_CDC14B	0.122277	0.011816	0.030147
11745220_ARHGAP12	0.120889	0.011824	0.030166
11735479_CCDC125	-0.2275	0.011835	0.030192
11745914_GOLGA8A	0.189614	0.011836	0.030192
11758626_UBA2	-0.26123	0.01184	0.0302
11719702_FARP1	-0.13012	0.011842	0.030204
11759693_RTKN2	-0.35282	0.011853	0.03023
11734641_IRGM	0.180446	0.011853	0.03023
11735760_POPDC2	0.210791	0.011865	0.030259
11756085_MAGEC1	-0.19306	0.011868	0.030265
11730485_CAPNS2	0.163625	0.011872	0.030274
11753104_PARPBP	-0.18452	0.011873	0.030275
11722994_ADH1A ///	-0.21931	0.011875	0.030277
11729083_GIPC3	-0.204	0.011881	0.030291
11737048_RIBC1	-0.18684	0.011882	0.030292
11756035_PFKFB3	-0.17247	0.011885	0.030299
11717276_MAPK14	0.130535	0.011889	0.030306
11748282_PPP3CB	-0.16688	0.011889	0.030306
11734843_MPHOSPH8	0.227322	0.011891	0.030308
11729395_HECTD2	0.268669	0.011891	0.030308

11736822_TP73	-0.16076	0.011894	0.030314
11755689_NCLN	0.135036	0.011908	0.030347
11735397_CNGB1	-0.24092	0.011908	0.030347
11745047_SLC27A4	0.121097	0.01191	0.030349
11721942_ZFAT	0.19943	0.011916	0.030363
11760920_PHKG2	0.181518	0.011917	0.030363
11751666_TUFT1	0.294974	0.011922	0.030376
11740983_MB	-0.15933	0.011924	0.030379
11757201_MESTIT1	-0.18651	0.011938	0.030411
11717797_ZER1	0.15729	0.011938	0.030411
11716423_SPTBN1	-0.16779	0.011942	0.03042
11758822_KIF2A	0.184881	0.01195	0.030437
11754585_WDR82	0.156163	0.01195	0.030437
11732625_RIMS1	0.262803	0.011953	0.030443
11746790_BECN1	0.12046	0.011958	0.030455
11763990_ALKBH7	-0.15966	0.011959	0.030456
11717607_VMA21	-0.25994	0.011962	0.030462
11727618_EFNA3	-0.17679	0.011965	0.030467
11746485_FAR2P2 //	0.1448	0.011977	0.030497
11759595_ERVK-6	0.259162	0.011984	0.030511
11754782_KIAA1614	-0.26723	0.011984	0.030511
11736698_NEBL	-0.22016	0.011985	0.030511
11739956_SLC25A15	0.127874	0.012005	0.030561
11725756_LRRC47	-0.12547	0.012009	0.03057
11740874_FES	-0.1411	0.012012	0.030577
11758534_SLC25A17	-0.15345	0.012017	0.030588
11728604_RSPH14	-0.32266	0.012026	0.030608
11731291_PIAS2	0.191631	0.012028	0.030613
11719371_GYS1	-0.12856	0.01203	0.030615
11758384_CYP2U1	-0.13192	0.012033	0.030623
11726783_CEP112	0.228651	0.012036	0.030629
11750386_CTNNA1	0.134131	0.012042	0.030642
11736776_CBLN1	-0.16414	0.012047	0.030651
11756332_MFSD10	0.132506	0.01205	0.030658
11725704_AMOTL1	-0.13084	0.012055	0.030667
11762008_ATP10B	-0.19763	0.012055	0.030667
11734385_PROCA1	-0.24204	0.012055	0.030667
11752864_FER	0.190422	0.012059	0.030673
11749851_HSDL1	0.238337	0.012059	0.030673
11745195_UNC45A	-0.15277	0.012065	0.030686
11760236_CBR1	-0.12659	0.012065	0.030686
11725927_CLIC4	-0.11376	0.012067	0.03069
11738652_KRTAP24-1	0.234184	0.012069	0.030694
11742026_CCDC113	0.160903	0.012072	0.030698
11742111_DRC3	-0.14695	0.012074	0.030701
11752507_LMNA	-0.1173	0.012076	0.030706
11742986_PARK7	0.112831	0.012078	0.030709
11757228_ABHD11-AS	-0.1451	0.012088	0.030733
11739684_PHF2	-0.12933	0.012099	0.030759
11739155_FAM129A	-0.11464	0.012116	0.030801
11733055_SCAMP1	-0.14658	0.012117	0.030801

11760068_YME1L1	-0.13707	0.012119	0.030805
11751743_HRH3	-0.19071	0.012119	0.030805
11747555_ENO3	0.212174	0.012121	0.030808
11722021_CDK16	-0.1486	0.012127	0.030821
11764147_ZNF546	0.189831	0.012129	0.030824
11739363_FKRP	0.157763	0.012142	0.030856
11717752_SEC14L1	0.115174	0.012144	0.03086
11732672_CCDC107	0.234276	0.012148	0.030867
11724846_NR2C2AP	-0.20292	0.01215	0.03087
11746802_BNIP3L	0.12975	0.012151	0.030872
11741909_PKNOX2	-0.16166	0.012152	0.030872
11737916_NEK11	0.222145	0.012156	0.030881
11716808_ITPA	0.128768	0.012162	0.030893
11723980_PHKA2	-0.14732	0.012162	0.030893
11727039_MAVS	-0.29768	0.012163	0.030893
11754146_THPO	-0.26163	0.012163	0.030893
11729218_GCLC	0.134537	0.012177	0.030926
11746977_GAPVD1	-0.13074	0.012178	0.030929
11760816_C21orf58	-0.25795	0.012182	0.030935
11732071_SLC16A8	-0.13778	0.012182	0.030935
11736735_ZNF621	0.208517	0.012196	0.030969
11728662_GABARAPL2	0.134839	0.012197	0.030971
11732959_SPRR3	-0.14255	0.012201	0.030978
11722319_EFNB3	-0.15211	0.012205	0.030986
11748455_SLC46A1	0.150629	0.012211	0.031
11743809_WBP5	-0.14299	0.012211	0.031
11763257_LZTS1	-0.15602	0.012213	0.031002
11739588_RERE	0.126679	0.012213	0.031002
11717123_PPP1R12B	0.151574	0.012214	0.031002
11746630_INPP5J	-0.18929	0.012217	0.031008
11758945_FAM163A	-0.13739	0.012218	0.031008
11756779_JTB	0.123109	0.012219	0.031008
11758870_GOSR2	0.185912	0.012227	0.031029
11753747_CHRAC1	-0.16101	0.012234	0.031044
11742845_AQR	0.13357	0.012235	0.031044
11758439_TRIM7	-0.1422	0.012236	0.031046
11732402_UBAC1	-0.12205	0.012237	0.031048
11754987_C21orf58	-0.15453	0.012239	0.031049
11758747_TMEM167B	-0.384	0.012239	0.031049
11764221_MUM1L1	-0.21393	0.012246	0.031066
11745326_DUS1L	0.116466	0.012256	0.031089
11723879_KCNC4	-0.28001	0.012257	0.03109
11744133_ZSWIM6	0.11172	0.012258	0.03109
11738032_FAM153C	-0.19452	0.012265	0.031108
11762037_ESR2	-0.17454	0.012273	0.031127
11726067_PDDC1	0.212444	0.012276	0.03113
11763925_SNORD9	-0.21139	0.012276	0.03113
11718113_SF3A3	0.112669	0.012279	0.031136
11722174_UQCC1	0.116924	0.012281	0.03114
11760067_NPSR1-AS1	-0.15997	0.012282	0.03114
11727088_DACH1	0.367451	0.012283	0.03114

11730306_TERF2	0.16176	0.012285	0.031145
11717850_ZNF282	0.163457	0.012286	0.031145
11753445_HMOX1	0.139366	0.012287	0.031146
11742020_INPP4A	-0.13606	0.012288	0.031147
11723497_YBEY	0.133984	0.012289	0.031147
11760623_CKLF ///	0.221602	0.012294	0.03116
11728139_ADAMTS13	-0.14975	0.012302	0.031178
11763924_SNORD9	-0.21108	0.012305	0.031184
11743242_OMP	-0.20367	0.012319	0.031218
11735523_FAM47E	0.324353	0.012324	0.031228
11729279_FAM175A	0.152951	0.012325	0.03123
11741018_FAM65A	-0.12455	0.012327	0.031234
11746535_LOC100132	-0.14817	0.012331	0.031241
11753505_PGAP2	-0.15381	0.012339	0.031261
11755999_DEPDC4	-0.4291	0.01234	0.031261
11756285_IGF2BP3	0.112774	0.012343	0.031268
11760364_DPAGT1	-0.17842	0.012345	0.031271
11721265_GATB	-0.19499	0.012347	0.031274
11761554_POLR2C	-0.1701	0.012348	0.031276
11733766_CSN2	-0.16246	0.012349	0.031276
11737706_FAM181A	-0.15836	0.012354	0.031286
11758953_RAN	-0.13801	0.012354	0.031286
11725958_CALM2 ///	-0.279	0.012358	0.031294
11723810_STX17	0.150559	0.012362	0.0313
11754037_UBE2F	0.153682	0.012362	0.0313
11737536_SAMD12	0.174377	0.012362	0.0313
11751592_WDR3	0.192357	0.012363	0.0313
11718455_INPP5K	0.167151	0.012364	0.0313
11732347_TCF20	-0.23289	0.012372	0.031319
11721006_TMCO3	-0.14367	0.012374	0.031323
11717573_NECAP1	0.134453	0.012375	0.031323
11726472_TAF5L	-0.13133	0.012379	0.031333
11735849_CSNK1A1L	0.208712	0.012381	0.031336
11754645_STT3B	-0.12095	0.012388	0.031353
11730584_ABCG4	-0.15391	0.012389	0.031353
11720732_SUMF1	0.171742	0.01239	0.031354
11718777_SRRM1	0.108018	0.012391	0.031354
11752063_CTNNA2	0.192068	0.012391	0.031354
11762232_ICAM3	-0.22463	0.012396	0.031365
11726835_DNAJC21	-0.27732	0.0124	0.031374
11761994_ALS2	0.166721	0.012415	0.031407
11716020_RAD23A	-0.12857	0.012415	0.031407
11718291_HK1	-0.13034	0.012417	0.03141
11751221_PADI1	-0.1357	0.012418	0.031411
11729503_CFAP53	0.162214	0.012419	0.031412
11728192_LCN1	-0.16686	0.01242	0.031412
11728816_AARS2	-0.19532	0.01242	0.031412
11735170_MXD3	-0.24977	0.012421	0.031412
11721402_NCOA3	0.156021	0.012423	0.031417
11741511_SCD5	-0.1664	0.01243	0.031431
11762871_CHTF18	-0.159	0.012432	0.031435

11752543_TFCP2	0.113864	0.012432	0.031435
11749010_BRSK2	-0.22073	0.012437	0.031444
11748328_KRT16 ///	-0.27751	0.012443	0.03146
11719133_COMMD9	-0.15155	0.012447	0.031467
11718681_PHF14	0.196535	0.012451	0.031475
11762239_LOC101926	0.124917	0.012455	0.031484
11731572_FRMD4B	0.182223	0.012456	0.031484
11731536_FHL5	0.246619	0.012466	0.031509
11756467_TMEM50A	-0.12563	0.01247	0.031516
11749035_ITGA3	0.161515	0.01247	0.031516
11736495_TNRC6B	0.193417	0.012471	0.031517
11725524_DSG2	0.143847	0.012472	0.031517
11750863_PHF19	-0.16851	0.012479	0.031533
11727250_ZSCAN22	-0.16448	0.012482	0.031539
11739663_RIMS3	-0.15244	0.012484	0.031544
11742799_ATF7IP	0.14347	0.012487	0.031548
11747982_GAB1	0.348944	0.01249	0.031556
11717935_ERMP1	-0.16498	0.012492	0.031558
11761136_OBSCN	-0.29468	0.012493	0.03156
11757143_SNORA15	-0.39433	0.012494	0.03156
11727910_RELT	-0.14386	0.012496	0.031564
11759696_SLITRK3	-0.42783	0.0125	0.031573
11750872_SLC30A10	-0.22866	0.012509	0.031592
11733335_HDX	0.175832	0.012514	0.031604
11749623_TYW1 ///	-0.15123	0.012517	0.031611
11748911_SAFB2	0.151629	0.012521	0.031618
11750061_DAZAP2	-0.15154	0.012527	0.031633
11747486_TMEM254	-0.21601	0.01253	0.031637
11742596_OR51B6	-0.14082	0.012531	0.031638
11745433_PHC2	-0.1434	0.012533	0.031642
11715932_TUBB6	0.144467	0.012534	0.031644
11733788_HSDL1	0.111954	0.012537	0.031649
11725362_SMAD4	0.153186	0.012544	0.031664
11729813_ZADH2	-0.17408	0.012554	0.031689
11737402_PRG3	-0.14727	0.012556	0.031691
11748568_METTL3	-0.1421	0.012557	0.031694
11734467_AMER1	-0.15524	0.012571	0.031726
11737296_TMTC4	-0.21818	0.012576	0.031736
11729602_CHMP2A	-0.13044	0.012576	0.031737
11749512_STAT4	0.116469	0.012584	0.031752
11721372_TCF7L1	-0.13511	0.012584	0.031752
11758708_PRAMEF1 /	-0.11558	0.012594	0.031776
11725910_COL4A6	0.413703	0.012596	0.031781
11763628_POU3F3	-0.30652	0.012613	0.031821
11716082_WDR77	-0.14773	0.012615	0.031821
11741441_FGF13	0.171257	0.012616	0.031821
11731939_UCP3	0.298515	0.012616	0.031821
11742736_MCEE	0.157061	0.012616	0.031821
11761827_C21orf58	-0.22019	0.012625	0.031843
11760202_IGFBP7	-0.28079	0.012628	0.03185
11749259_IST1	0.16009	0.012631	0.031854

11752414_BRSK2	-0.15516	0.012638	0.031872
11755685_LOC100130	-0.13143	0.012639	0.031872
11737788_SHISA5	-0.15049	0.012641	0.031876
11731413_GALM	-0.18577	0.012649	0.031893
11760342_PPP3CB	0.138453	0.012652	0.031899
11746861_CACNA2D4	0.166249	0.012658	0.031913
11729394_HECTD2	0.148614	0.012659	0.031913
11763596_JAKMIP3	-0.13006	0.012661	0.031916
11753381_BTBD19 //	0.160695	0.012663	0.031919
11721420_RAP1GAP2	-0.15251	0.012663	0.031919
11757247_OR6W1P	-0.14499	0.012665	0.031923
11748225_ABHD10	-0.14422	0.012668	0.031928
11746350_PIM1	-0.34322	0.01267	0.031932
11760897_CCDC40	-0.24445	0.012676	0.031946
11747212_ZNF174	0.122058	0.012678	0.031946
11752710_ZNF215	0.281803	0.012678	0.031946
11722594_AKAP13	-0.17707	0.012681	0.031952
11723274_SLC35F2	0.149256	0.012689	0.031971
11742006_KRTAP12-2	0.262426	0.012694	0.031981
11738737_OR2A12	-0.15869	0.012696	0.031985
11751084_PLXND1	-0.13477	0.012697	0.031986
11753158_PDGFD	0.303152	0.012698	0.031988
11721221_ZNF318	0.135119	0.0127	0.031992
11747767_MBD1	0.133786	0.012705	0.032001
11760316_PXN	0.135889	0.012706	0.032002
11744601_RPS17	-0.11382	0.012706	0.032002
11743948_BMP6	-0.21108	0.012709	0.032006
11752351_PPP1R15B	0.212568	0.012709	0.032006
11732120_DBN1	-0.12894	0.012713	0.032013
11759262_ADARB2-AS	-0.14528	0.012713	0.032013
11735854_TAAR5	-0.2983	0.01272	0.032028
11733222_DLL3	-0.15696	0.012725	0.032039
11744011_AMDHD2	-0.151	0.012726	0.032039
11734309_NPVF	-0.21876	0.012727	0.03204
11757601_COPS3	0.109556	0.012727	0.03204
11728082_ELFN1	-0.14195	0.01274	0.032071
11739759_KBTBD3	0.415113	0.012744	0.032077
11746734_SCN1B	0.17154	0.012755	0.032104
11718358_PEG10	-0.22452	0.012757	0.032107
11749300_WDR77	0.117813	0.01276	0.032115
11725216_FCN3	-0.12909	0.012766	0.032128
11743305_SMIM7	-0.11039	0.012768	0.032132
11757199_LOC100190	-0.20333	0.01277	0.032133
11756728_GAS2	-0.17722	0.012772	0.032137
200065_PMARF1	-0.11342	0.012772	0.032137
11723034_GTF2A2	0.236758	0.012773	0.032137
11740242_TOX	0.237617	0.012775	0.03214
11759571_IGLC1 ///	-0.18825	0.01278	0.03215
11719535_AP3M2	-0.19145	0.012785	0.03216
11752676_OGFR	-0.11177	0.012785	0.03216
11735886_NEK11	0.316005	0.012789	0.032168

11749142_DHX35	-0.16978	0.01279	0.032169
11743254_ 15-Sep	0.112288	0.012797	0.032186
11722297_LRFN4	0.112038	0.012799	0.03219
11722919_DOLPP1	-0.18584	0.012801	0.032192
11741644_ATXN7L1	0.140964	0.012811	0.032216
11742912_GAREM1	0.169843	0.012818	0.032232
11743558_NOA1	0.114078	0.012819	0.032233
11731422_FCGR3A	-0.18666	0.012833	0.032265
11758625_GSTA4	-0.12317	0.012833	0.032265
11720462_F11R	-0.1871	0.012835	0.032268
11753730_SNCA	0.139879	0.012843	0.032285
11716046_RGL2	0.128732	0.012852	0.032307
11744001_LRRC45	-0.15878	0.012858	0.032318
11728298_SCARB1	-0.11915	0.012858	0.032318
11739070_PPIL2	-0.17567	0.012863	0.03233
11717795_ZDHHC3	-0.14954	0.012874	0.032355
11760415_SLC44A5	0.464694	0.012876	0.032358
11763830_CD72	-0.18184	0.012879	0.032364
11741560_HOXA6	0.191514	0.01289	0.03239
11725445_BCL3	0.167281	0.012892	0.032393
11719131_POLR3D	-0.11554	0.012893	0.032393
11731468_CYP2E1	0.237721	0.012894	0.032393
11724874_RAD9A	-0.11601	0.012894	0.032393
11760159_USP36	-0.24229	0.012894	0.032393
11753164_SKIV2L	-0.33541	0.012895	0.032393
11764058_PRNP	0.162368	0.012898	0.032399
11733989_CMTR1	-0.17209	0.012904	0.032413
11757009_LPCAT3	-0.14232	0.012907	0.032417
11742165_SPANXN3	-0.14225	0.012907	0.032417
11740522_TPO	-0.20031	0.012919	0.032445
11717807_RNF103	0.144053	0.012927	0.032465
11751460_DDX20	0.120037	0.012928	0.032465
11729754_ULK2	-0.12395	0.01293	0.032468
11755746_CNIH4	-0.12011	0.012933	0.032474
11741761_C14orf93	-0.32024	0.012937	0.032482
11758926_PSPC1	0.190242	0.012938	0.032484
11743835_DSC3	0.16811	0.012944	0.032496
11721014_WTAP	0.161211	0.012952	0.032516
11746171_KLHL17	-0.18972	0.012953	0.032517
11719138_MGME1	0.10844	0.012955	0.032517
11727598_C9orf64	-0.19435	0.012955	0.032517
11722618_RIPK4	-0.22698	0.012957	0.03252
11723630_CRYBG3	0.151454	0.012962	0.032532
200051_PMSART1	0.110053	0.012963	0.032533
11758184_KRT8	0.116548	0.012966	0.032538
11734720_PLPP2	-0.13711	0.012968	0.032542
11747696_WDR24	-0.21462	0.012976	0.03256
11737078_CARD16	0.339386	0.012978	0.032563
11724078_E2F2	-0.22678	0.012985	0.032581
11747112_ZNF766	0.158528	0.012987	0.032584
11751200_ARHGEF16	-0.14705	0.01299	0.03259

11749580_PCDH18	0.147142	0.012993	0.032595
11725116_NCOR1	0.181563	0.012998	0.032607
11733095_SET /// S	-0.11642	0.013004	0.032618
11736431_NOL8	0.126154	0.013005	0.032619
11758556_PGM2	0.151101	0.013008	0.032626
11736147_LYRM2	0.139548	0.013013	0.032636
11744353_TATDN2	-0.15454	0.013017	0.032645
11743452_CDCA4	-0.12883	0.01302	0.032652
11742518_OR7G2	-0.18634	0.013022	0.032653
11724555_FOLH1 ///	0.250705	0.013031	0.032675
11749147_ATP7B	-0.18255	0.013035	0.032682
11754572_THNSL2	0.170836	0.013035	0.032682
11725968_THUMPD1	0.109967	0.01304	0.032693
11757099_FAM207A	0.161393	0.013045	0.032704
11750164_SCRN3	0.138562	0.013047	0.032706
11729052_PPFIBP1	0.228609	0.013053	0.032719
11733858_CLASP2	0.133293	0.013054	0.032722
11754193_PCYT1A	0.133671	0.013059	0.032731
11742079_FAM222A	-0.22648	0.013061	0.032735
11739639_CDK12	-0.15845	0.013066	0.032745
11736805_ZNF43	0.199378	0.013069	0.032753
11758862_PAIP2B	-0.1825	0.013076	0.032768
11723149_SLC19A2	0.147003	0.013084	0.032785
11744616_SLC19A2	0.147003	0.013084	0.032785
11735947_ZNF221	0.275057	0.013087	0.032791
11755022_ARL6IP1	-0.12785	0.013096	0.032811
11743436_TBC1D12	0.149919	0.013098	0.032814
11761267_DLG2	-0.18781	0.013099	0.032815
11748671_CUL4A	-0.1158	0.013101	0.032818
11723055_SYNGR3	-0.19412	0.013102	0.032819
11761929_FUT10	0.429597	0.013104	0.032824
11762993_YME1L1	0.156135	0.013109	0.032833
11759563_EPC1	0.234111	0.013109	0.032833
11729622_CLCN4	0.278434	0.013112	0.032837
11754747_QTRT1	-0.17037	0.013113	0.032838
11761262_PPIC	0.325292	0.01312	0.032854
11762657_C6orf141	-0.20734	0.013122	0.032858
11760256_BDH2	-0.36855	0.013123	0.032858
11750471_PRSS16	-0.23353	0.013124	0.032859
11736379_MSL2	0.177307	0.013126	0.032863
11752013_CHD9	0.244595	0.013127	0.032863
11760098_CD37	-0.1802	0.013128	0.032863
11752849_CWC27	-0.16296	0.013129	0.032865
11746177_RHBDF1	0.126629	0.013139	0.032889
11753457_PDDC1	0.158255	0.013143	0.032896
11748316_JPH4	-0.18209	0.013154	0.032923
11737105_ARL17A	0.137441	0.013159	0.032932
11720233_RHOBTB2	-0.14369	0.013159	0.032932
11741758_TRPV4	-0.13194	0.01316	0.032932
11756738_FANCC	-0.17137	0.013162	0.032936
11721408_PAOX	-0.31267	0.013167	0.032946

11716044_FAM168B	0.138449	0.013167	0.032946
11715301_NOP16	0.144465	0.013168	0.032946
11729683_TMEM231	-0.20577	0.013176	0.032964
11734229_FRRS1	0.352535	0.013177	0.032964
11715692_CIRBP	-0.1664	0.013192	0.033
11750509_TROAP	-0.17831	0.013194	0.033005
11763814_OPRK1	-0.25906	0.013196	0.033008
11750277_POLR3D	-0.12016	0.013211	0.033044
11718921_IQSEC1	0.127995	0.013212	0.033045
11731688_SI	0.300532	0.013215	0.033049
11754435_WRAP73	-0.12704	0.013217	0.033051
11728641_DEFA5	-0.13213	0.013217	0.033051
11735558_TBC1D28	-0.15828	0.013219	0.033051
11753726_IGF2 ///	-0.11427	0.013219	0.033051
11720137_ANAPC4	-0.11527	0.013219	0.033051
11729415_ST20	0.145502	0.013222	0.033057
11730824_COX19	0.162649	0.013226	0.033066
11719082_GADD45GIP	-0.12617	0.013234	0.033083
11737020_AGAP2	-0.2165	0.013247	0.033115
11733551_ZIC5	-0.46259	0.013252	0.033124
11755038_SREK1	0.142759	0.013252	0.033124
11748051_DPH7	0.168448	0.013254	0.033127
11760050_LOC100134	-0.13641	0.01326	0.033141
11747525_C6orf89	0.120626	0.013261	0.033142
11751969_FAM193A	0.124541	0.013265	0.03315
11720909_TMEM44	-0.24035	0.013267	0.033153
11721824_ARPC5L	0.133564	0.01327	0.033158
11755559_ADCK4	-0.13171	0.013275	0.033169
11744108_SAE1	-0.12858	0.013289	0.033202
11757190_LITAF	0.106945	0.013291	0.033205
11749278_FETUB	0.204036	0.013296	0.033217
11731715_ACKR2	-0.23825	0.013305	0.033237
11743516_LAMC2	-0.18058	0.013309	0.033245
11756274_ALDH1L1	0.2368	0.013311	0.033248
11720870_PDE7A	-0.27251	0.013311	0.033248
11747390_FBXO44	-0.20444	0.013313	0.033251
11761234_NFATC4	-0.21467	0.013319	0.033264
11741289_NXPH4	-0.1645	0.013324	0.033275
11754539_LOC90768	0.173599	0.013329	0.033284
11720322_AK2	0.118889	0.013332	0.03329
11746522_FOXO3 ///	0.170245	0.013335	0.033297
11752506_LMNA	-0.11475	0.013343	0.033315
11749607_LOC399815	-0.22814	0.013349	0.033329
11728280_HSPBAP1	-0.14379	0.013352	0.033333
11744074_GLI4 ///	-0.11623	0.013352	0.033333
11735931_FRG2 ///	-0.2053	0.013369	0.033373
11732067_RGAG1	-0.21649	0.013373	0.033381
11757359_TMA7	-0.11147	0.013379	0.033394
11719533_STX3	0.152539	0.013386	0.03341
11715597_PSMB7	-0.11073	0.013393	0.033427
11718037_MDK	-0.12736	0.013394	0.033427

11721131_HCFC1	-0.17655	0.013395	0.033428
11726057_PPP4R3B	-0.17393	0.013401	0.033442
11734927_BPNT1	-0.17809	0.013402	0.033442
11753414_DMAP1	-0.12561	0.013409	0.033458
11756578_RHOT1	0.111678	0.013415	0.033469
11755230_PDZD3	-0.14227	0.013415	0.033469
11716661_GRAMD4	-0.12422	0.013416	0.033471
11733237_RPGRIP1L	-0.25407	0.013417	0.033471
11746609_CAND2	-0.28047	0.013418	0.033472
11730072_CNTN3	0.119114	0.013419	0.033472
11756620_RTEL1 ///	-0.20835	0.01342	0.033472
11744327_UBB	-0.10648	0.01342	0.033472
11749821_CCT4	-0.10941	0.013421	0.033472
11761122_PIGL	-0.13436	0.013429	0.03349
11732875_GAGE1 ///	0.122421	0.013438	0.033511
11726681_HNRNPK	-0.1663	0.013446	0.03353
11747465_NDST1	0.333659	0.013453	0.033546
11760940_HDAC6	-0.17706	0.013459	0.033559
11725291_FUT1	-0.147	0.01346	0.03356
11746834_ACADM	0.160169	0.013466	0.033572
11753042_KREMEN2	-0.16062	0.013469	0.033578
11761365_MIR181A1H	0.170283	0.013473	0.033588
11715621_UFC1	-0.11424	0.013474	0.033588
11725469_LIPG	0.161653	0.01348	0.033601
11725806_KCNJ8	-0.26176	0.013487	0.033618
11735728_ZNF385A	-0.16126	0.01349	0.033622
11721496_WDR33	-0.27474	0.013493	0.033627
11743294_BCKDK	-0.13156	0.013494	0.033629
11717787_CLUH	-0.15484	0.013499	0.033639
11739314_AES	-0.1259	0.013502	0.033646
11729841_GRM2	-0.17355	0.013509	0.03366
11762705_LOC100128	-0.18788	0.013509	0.03366
11763062_NPSR1-AS1	-0.13901	0.013516	0.033674
11762690_PPP1R1C	0.303552	0.01352	0.033683
11718869_PALMD	-0.26535	0.013525	0.033694
11740660_IGFALS	-0.13255	0.013529	0.033703
11753985_DMKN	0.181474	0.013568	0.033796
11724932_CREM	0.136482	0.013568	0.033796
11732809_PROK2	-0.22991	0.013591	0.03385
11749177_FAM63A	0.285187	0.013597	0.033865
11716940_TRIB2	-0.13157	0.013601	0.033873
11728665_PCBP2	-0.11955	0.013606	0.033884
11723049_CCHCR1	-0.23325	0.013617	0.033906
11727550_DOCK3	0.360001	0.013617	0.033906
11739822_CPNE8	0.225976	0.013617	0.033906
11736712_TMIE	-0.1846	0.013619	0.033909
11754688_UBAP2	0.159214	0.013621	0.033912
11722118_LZTS1	-0.17506	0.013623	0.033914
11755259_C12orf74	-0.16217	0.013626	0.033922
11751698_EAF2	-0.17583	0.013632	0.033935
11760477_EP400NL	0.216624	0.013634	0.033937

11718442_MAPK8IP3	0.171143	0.013636	0.033941
11732584_WRN	0.240745	0.013638	0.033944
11750500_RECQL5	-0.1416	0.013642	0.033952
11763650_GET4	-0.18305	0.013646	0.033961
11739027_CLTC	-0.20952	0.013647	0.033962
11720944_RGS16	-0.13188	0.013649	0.033964
11718236_PRPF4B	0.109247	0.013655	0.033978
11745831_NRXN1	-0.16786	0.013669	0.034011
11749500_SLC4A1AP	0.16102	0.013671	0.034013
11718339_DAZAP2	-0.13181	0.013674	0.03402
11717036_HDAC5	-0.13051	0.013677	0.034025
11723760_ZFR	0.152899	0.013691	0.03406
11722886_EMB /// E	0.264385	0.013693	0.034063
11757049_CTDP1	-0.26939	0.013695	0.034065
11737290_OXGR1	-0.17874	0.013696	0.034067
11739986_AMACR ///	0.211355	0.013702	0.03408
11741231_GGPS1	-0.12534	0.013704	0.034082
11749467_PRKDC	-0.15925	0.013707	0.034089
11745669_KANSL1L	-0.12063	0.013716	0.03411
11751276_CALCOCO2	-0.11825	0.013718	0.034113
11721335_PLOD2	0.130971	0.013728	0.034134
11756003_IGF2 ///	0.333694	0.013729	0.034134
11752577_FTH1	-0.14119	0.013729	0.034134
11756639_NFASC	-0.17082	0.013732	0.034139
11753604_IL21	-0.17589	0.013735	0.034147
11745841_CNDP2	-0.25003	0.01374	0.034157
11755212_SVOPL	-0.23034	0.013745	0.034167
11741239_COPE	0.117521	0.013746	0.034168
11749133_NLRX1	-0.13329	0.013754	0.034186
11745971_EIF3B	0.116504	0.013755	0.034188
11729512_CSF3	-0.14179	0.013756	0.034189
11722394_P4HTM	-0.1512	0.01377	0.034221
11725396_TWISTNB	0.114037	0.013771	0.034222
11738019_RTP2	-0.14938	0.01378	0.034242
200044_PM SRSF9	-0.10691	0.013782	0.034244
11719717_PPP1R14B	-0.1118	0.013787	0.034255
11733754_PLEKHA4	0.125832	0.013791	0.034263
11741146_ALDH3B1	0.135632	0.013795	0.034272
11735754_EPHB4	-0.14563	0.013806	0.034297
11715406_COL6A1	0.201223	0.013809	0.034303
11731689_TUBA3D	-0.23579	0.013817	0.034319
11744308_TTC39A	-0.15594	0.013817	0.034319
11726485_CPED1	-0.49881	0.013818	0.034321
11725356_PRTFDC1	-0.19555	0.013822	0.034328
11720152_DENND4B	-0.13014	0.013823	0.03433
11751865_PLA2G1B	-0.26208	0.013826	0.034335
11721080_PARVA	0.187718	0.013837	0.034359
11754871_TSPAN32	-0.14556	0.013838	0.034359
11746406_TLR10	0.251407	0.013838	0.034359
11742019_ARID1A	-0.14928	0.013843	0.03437
11760123_PIAS3	-0.19843	0.013847	0.034379

11723966_SLC22A11	-0.24183	0.013848	0.03438
11719685_NTS	-0.16227	0.013853	0.034389
11747263_TRIML1	-0.16887	0.013855	0.034392
11752362_SBNO1	0.17715	0.013855	0.034392
11750445_SLC22A24	-0.15781	0.013859	0.034401
11761620_SSX6	-0.26751	0.013861	0.034403
11726446_KCNK3	-0.16873	0.013862	0.034404
11716764_NSMF	-0.17296	0.013865	0.034407
11748077_ARPC4-TTL	0.161063	0.013865	0.034407
11728524_SPOCD1	-0.19505	0.013866	0.034407
11720463_F11R	-0.11135	0.013875	0.034428
11752028_DIAPH3	0.181947	0.013876	0.03443
11757371_ISYNA1	0.130997	0.013877	0.034431
11731081_RBP2	-0.18586	0.013879	0.034434
11748522_ARFIP2	0.207468	0.013885	0.034446
11737145_SH3YL1	0.154596	0.013887	0.034449
11755226_KLHDC7A	0.19147	0.013887	0.034449
11753781_THPO	-0.20575	0.013894	0.034461
11725880_ZFP41	-0.10841	0.013894	0.034461
11757497_CMPK1	0.133553	0.013896	0.034465
11761425_GALNT11	0.52796	0.013899	0.034472
11760870_PARPBP	-0.33406	0.013903	0.034479
11716525_SPON2	-0.12621	0.013906	0.034486
11746765_SCEL	0.222074	0.01391	0.034493
11737910_SLC39A14	-0.11991	0.013914	0.0345
11715523_HADHA	0.121813	0.013915	0.034502
11718733_MAPT	-0.19415	0.013921	0.034516
11754861_SUCLG1	-0.12804	0.013926	0.034526
11725102_EGFR	0.306049	0.013927	0.034526
11722202_FBXO22	0.112649	0.013929	0.034529
11750354_TMEM184C	0.223246	0.013931	0.034532
11743292_GALNT1	0.10964	0.013934	0.034538
11720356_SMAP1	0.193731	0.013943	0.03456
11750980_ARFGAP3	0.126653	0.013946	0.034565
11719453_BBS1	0.158491	0.013947	0.034565
11743445_SUPT6H	0.200997	0.013951	0.034573
11749382_STK11IP	-0.1469	0.013954	0.034579
11716235_RAE1	-0.1155	0.013968	0.034613
11738461_NUTM2F	-0.16839	0.01397	0.034615
11721298_MRPS21	-0.12292	0.013975	0.034627
11757561_NOC2L	-0.13031	0.01398	0.034636
11729018_SPRY3	-0.12695	0.01398	0.034636
11738746_OR2T29 //	-0.12281	0.013986	0.034647
11715716_MSN	-0.12287	0.013988	0.034652
11733413_SLC26A6	0.128177	0.013992	0.034659
11753761_KLK6	-0.16484	0.013998	0.034673
11727856_NUP50	-0.13034	0.014	0.034675
11724940_CDHR3	-0.2096	0.01401	0.034699
11763999_GPRIN3	-0.24579	0.014021	0.034723
11753988_SPRY2	0.108543	0.014027	0.034738
11717867_ZNRD1	0.131554	0.014032	0.034749

11727918_MUC20	0.205584	0.014041	0.034768
11737578_LRRC7	0.507242	0.014044	0.034774
11752703_CHD6	-0.23046	0.014048	0.034782
11753163_CDC73	0.137333	0.014051	0.034786
11763139_KRTAP22-2	-0.24236	0.014051	0.034786
11729007_OVOL2	-0.17548	0.014064	0.034817
11720812_C9orf16	0.117495	0.014068	0.034825
11728261_ZNF300	0.120499	0.014069	0.034825
11715665_PSMB8	-0.15232	0.014077	0.034843
11740776_CCDC36	-0.12651	0.014078	0.034843
11755311_ITGB1BP1	-0.11439	0.014082	0.034852
11723901_PLEKHG1	0.32543	0.014085	0.034857
11727375_HMGCR	-0.13868	0.014091	0.034871
11731363_KLC3	-0.21447	0.014093	0.034875
11755760_GDAP2	0.133184	0.014101	0.034893
11746226_GAS6-AS1	0.189073	0.014104	0.034899
11759579_CNPY2	0.112289	0.014113	0.034919
11746581_PCCA	0.123574	0.014117	0.034926
11743744_FAM26E	0.231077	0.014121	0.034934
11761130_BOLA2	0.220359	0.014123	0.034938
11725304_RAC1	-0.12822	0.014126	0.034944
11753672_GTF2H2 //	0.13391	0.014127	0.034944
11727190_THAP6	0.153468	0.014131	0.034952
11721897_DHX58	0.244663	0.014136	0.034964
11743591_ZDHHC7	-0.10661	0.014142	0.034977
11749164_TMEM206	0.146121	0.014149	0.034992
11742380_OR52A5	-0.17386	0.014151	0.034995
11716487_WDR59	0.119858	0.014152	0.034995
11727657_EPHB1	0.19768	0.014154	0.034998
11716486_FCGBP	-0.14737	0.014156	0.035001
11745003_POLR2K	-0.10848	0.014157	0.035003
11756506_CHL1	0.150427	0.014158	0.035003
11747503_TAF6L	-0.18823	0.01416	0.035005
11762185_FOXRED1	-0.1769	0.014164	0.035015
11736373_MLLT4-AS1	-0.27303	0.014167	0.035021
11753569_LZTS1	-0.33168	0.014169	0.035021
11755180_TCF7	0.148635	0.014169	0.035021
11729971_SPTB	0.185012	0.014175	0.035034
11715640_TTC3	0.181901	0.014176	0.035037
11751637_XXYLT1	-0.12777	0.014182	0.035048
11752721_CNOT10	0.143032	0.014192	0.035072
11716057_EFTUD2	-0.21203	0.014193	0.035072
11720649_FBXW9	0.221747	0.014199	0.035084
11756401_FAM49B	0.108331	0.014199	0.035084
11720532_C9orf72	-0.12766	0.014202	0.035088
11727658_KLK10	0.139687	0.014206	0.035096
11725273_C5orf45	0.135205	0.014206	0.035096
11725981_CCR7	-0.23031	0.014208	0.0351
11727866_SLC25A27	0.169033	0.014213	0.035108
11752423_F13A1	-0.24281	0.014215	0.035112
11726644_PEX3	-0.15283	0.014217	0.035116

11761944_UTP14A	0.215649	0.014219	0.035118
11756963_SOS2	0.127669	0.014234	0.035153
11745664_KRTAP21-1	0.125902	0.014236	0.035157
11724167_COLGALT2	0.168689	0.01424	0.035165
11753000_MOBP	-0.19283	0.014242	0.035168
11740437_MYOM1	0.256557	0.014243	0.035169
11722704_DZIP1	-0.19849	0.014248	0.035178
11724062_RAD51C	-0.13743	0.014248	0.035178
11743206_SLC38A8	-0.15328	0.014251	0.035184
11754227_WHSC1L1	0.149995	0.014256	0.035193
11733927_CTNS	-0.14036	0.014259	0.0352
11741110_ZNF57	0.182368	0.014261	0.035202
11736903_KLHDC1	-0.21179	0.014267	0.035215
11744084_ANKIB1	0.151801	0.014268	0.035216
11721364_IREB2	-0.15503	0.01427	0.035219
11753713_AGER	-0.17671	0.014275	0.035231
11754667_CPNE6	-0.1449	0.01428	0.035241
11735913_TNXA ///	-0.17545	0.014281	0.035241
11731366_TSPAN32	-0.15699	0.014285	0.03525
11742803_SRD5A1	-0.14828	0.014297	0.035277
11743823_TOPBP1	-0.12201	0.014299	0.035281
11736471_RAB9B	-0.17584	0.01431	0.035304
11748791_AP5M1	-0.14374	0.014311	0.035304
11738118_CARD14	0.153601	0.014312	0.035304
11738943_LOC105371	-0.30156	0.014312	0.035304
11758144_HNRNPC	-0.10431	0.014313	0.035304
11739566_FKBP5	-0.23443	0.014313	0.035304
11719142_INSIG2	-0.14818	0.014313	0.035304
11729544_MAGEC1	-0.1568	0.014316	0.035307
11737995_LRRC3	-0.11819	0.014324	0.035326
11731169_SPRR2G	-0.16101	0.014326	0.035326
11730448_DHRS12	-0.24409	0.014326	0.035326
11726731_AGGF1	0.146656	0.014326	0.035326
11743804_ABCE1	-0.10985	0.01433	0.035333
11737981_PHF6	-0.15517	0.01433	0.035333
11750939_SLC39A7	-0.15572	0.014345	0.035366
11722198_UBE2D2	-0.16813	0.014348	0.035372
11726122_MYO1F	-0.1257	0.014348	0.035372
11734613_KRTAP10-4	-0.12091	0.014349	0.035373
11725208_LETM1	-0.1345	0.014351	0.035375
11719760_ARRDC4	-0.1988	0.014355	0.035383
11720581_TCF12	0.147802	0.014361	0.035397
11757364_BLOC1S6	0.128938	0.014365	0.035405
11763285_KLHDC10	0.132647	0.014366	0.035405
11762536_BAGE	0.157316	0.014367	0.035405
11747457_UGT1A1 //	-0.14629	0.014373	0.035419
11748465_POGK	0.151017	0.014382	0.03544
11727501_ALDH3B2	-0.20434	0.014383	0.03544
11737764_TSKU	-0.13205	0.014384	0.035441
11752921_SPTY2D1	0.171545	0.014394	0.035462
11723973_CHST12 //	-0.15015	0.014398	0.035472

11734282_SLC25A48	-0.21689	0.014404	0.035483
11730133_ICK	-0.23639	0.014409	0.035493
11747476_SPIDR	-0.12235	0.014409	0.035493
11731807_CATSPER1	0.132165	0.01441	0.035493
11746313_TCF7L2	0.158434	0.014417	0.035506
11759572_STAU2	-0.16271	0.014418	0.035506
11716713_ARHGAP17	0.153597	0.014418	0.035506
11718313_LRP11	-0.13104	0.014418	0.035506
11740618_ADRA1D	0.260272	0.014422	0.035512
11730033_RPS6KL1	0.162733	0.014422	0.035512
11736760_FKBP11	0.134235	0.014431	0.03553
11761690_CD37	-0.16074	0.014431	0.03553
11761556_ACADL	-0.19431	0.014437	0.035542
11747379_ATP6AP2	0.10956	0.014437	0.035542
11760925_MSTO1 ///	-0.18357	0.01444	0.035547
11734248_SYCE1	-0.14592	0.014442	0.03555
11762165_ERBB2	-0.22107	0.014444	0.035555
11740184_CCHCR1	-0.16233	0.014448	0.035562
11745728_TCF12	0.157845	0.01445	0.035565
11761501_GALC	-0.12453	0.014451	0.035567
11752156_ATG9B	-0.14036	0.014452	0.035567
11746377_GRIN1	0.155462	0.014461	0.035588
11763312_MXD4	-0.12279	0.014464	0.035592
11763236_ZNF362	-0.12576	0.014466	0.035595
11760021_CTNNA3	-0.22412	0.01447	0.035603
11749200_PIGG	0.122325	0.014476	0.035617
11756918_KIF23	-0.17877	0.014479	0.035623
11715869_TINF2	-0.15783	0.014484	0.035633
11745702_SHPRH	-0.1457	0.014488	0.035641
11748610_PLOD2	0.128236	0.014498	0.035662
11735076_CYP2C18	-0.12858	0.014501	0.035668
11740139_ABCA2	-0.18343	0.014505	0.035676
11726968_BCAS4	-0.12817	0.014505	0.035676
11730648_GP9	-0.15558	0.014506	0.035676
11747547_SFI1	-0.12215	0.014511	0.035687
11760445_ZNF302	0.116492	0.014513	0.03569
11720012_JARID2	0.195705	0.014515	0.035692
11740090_DPH6	-0.19394	0.014516	0.035692
11738383_IL22RA2	-0.21611	0.014517	0.035692
11752394_SULT1C4	-0.15475	0.014517	0.035692
11726513_ZNF407	0.116455	0.014518	0.035692
11727201_ZNF230	0.172357	0.014519	0.035693
11736660_MAP3K13	0.258704	0.014522	0.035699
11758205_CAPNS1	0.12273	0.014528	0.035713
11745179_DCAF11	-0.17553	0.014529	0.035713
11715777_LUZP6 ///	0.110681	0.014531	0.035715
11737918_PTPRS	-0.15574	0.014534	0.035721
11732212_ZKSCAN1	0.150459	0.014538	0.03573
11746719_LMCD1	-0.1223	0.014556	0.035773
11716369_SUMO2	-0.11809	0.014561	0.035781
11734009_KDM8	-0.13593	0.014578	0.035822

11757155_SNORA3B	-0.13776	0.014584	0.035835
11730138_KLF1	-0.17059	0.014596	0.035863
11755336_KRI1	0.15789	0.0146	0.035871
11717261_AMFR	0.111394	0.014602	0.035873
11736496_TNRC6B	0.156448	0.014603	0.035873
200016_PMHNRNPA1	-0.10482	0.014603	0.035873
11729772_RNASE2	-0.18978	0.014607	0.035882
11722874_MSL3	0.117084	0.014613	0.035895
11716105_MPZL1	-0.1965	0.014629	0.035933
11759101_UBXN10	-0.27203	0.014633	0.03594
11729177_FBXO38	0.144276	0.014634	0.035941
11750057_SPSB3	-0.1545	0.014638	0.035948
11730266_CCDC69	-0.13222	0.01464	0.035951
11740044_MTFMT	-0.18052	0.014642	0.035953
11726274_PCK1	-0.15955	0.014647	0.035965
11729070_HS2ST1	0.165298	0.014658	0.035991
11762261_HSPA8	-0.12057	0.014675	0.03603
11739577_IGFBPL1	-0.23087	0.014678	0.036036
11739343_NNAT	-0.17614	0.014679	0.036036
11744451_DHX38	0.115282	0.014684	0.036047
11718629_BOK	-0.14107	0.014689	0.036057
11750737_SMPD4	-0.26815	0.014694	0.036068
11739487_SUZ12	-0.10673	0.014698	0.036075
11728840_TMCC3	0.189242	0.014708	0.036099
11716936_UNC45A	-0.1173	0.014709	0.0361
11721857_PIGK	-0.18048	0.014716	0.036114
11727077_NUFIP1	0.14438	0.01472	0.036123
11720819_CXCL12	-0.19916	0.014723	0.036128
11722096_EIF4ENIF1	-0.1593	0.014725	0.036128
11733880_HMGXB4	-0.14039	0.014725	0.036128
11727358_SLC6A3	-0.17393	0.014725	0.036128
11758741_TRAF3IP2	-0.22006	0.01473	0.036137
11724759_CALM1 ///	0.116133	0.014731	0.036139
11744639_SH3D21	-0.25404	0.014734	0.036142
11754305_ULK3	0.121268	0.014734	0.036142
11738764_TAS2R16	-0.15138	0.014735	0.036142
11752800_KCNK4	-0.15794	0.014736	0.036142
11733264_EML1	0.119052	0.014736	0.036142
11745706_GSTM2P1	-0.11427	0.014746	0.036164
11749581_CHST10	-0.17645	0.014756	0.036186
11724560_TSR2	-0.15544	0.014764	0.036204
11734697_GOLGA8A /	0.184413	0.014768	0.036213
11731860_ZNF720	-0.11233	0.014774	0.036226
11755341_RPE	-0.13594	0.014778	0.036234
11721967_IGSF11	-0.16507	0.014781	0.036239
11751882_LYST	0.249227	0.014784	0.036244
11728970_RNF133	0.193885	0.014791	0.036259
11735852_OR10H1 //	-0.17122	0.014792	0.036259
11742325_OR10H1 //	-0.17122	0.014792	0.036259
11742778_TBCC	0.120139	0.0148	0.036277
11743453_HDAC4	-0.15081	0.014811	0.036302

11725560_RECK	0.225846	0.014818	0.036317
11720444_ERLIN1	0.12671	0.01482	0.03632
200050_PM_ZNF146	0.147371	0.014822	0.036323
11718008_CPNE2	-0.1451	0.014825	0.036328
11760723_KIF6	0.372291	0.01483	0.03634
11723371_SHANK3	-0.22442	0.014835	0.036349
11749994_CD300LG	-0.12544	0.014839	0.036359
11716778_EIF1AX	-0.11188	0.014846	0.036374
11721226_MTSS1L	0.330937	0.014849	0.036379
11735863_GHRHR	-0.14832	0.014854	0.03639
11724249_SLC39A8	-0.14753	0.014859	0.036399
11762028_ARHGAP29	0.138389	0.01486	0.036401
11761723_MKRN3	-0.13854	0.014865	0.03641
11732433_GK	-0.17153	0.01487	0.036422
11742699_C1R	-0.21756	0.014873	0.036427
11717377_SRPRB	0.143435	0.014876	0.036433
11761030_SLC11A1	-0.22041	0.014881	0.036441
11754417_RPL14	0.104613	0.014881	0.036441
11745735_MRPS14	-0.12903	0.014884	0.036445
11726128_EPYC	-0.23196	0.014886	0.036449
11760380_SEPT6	-0.17736	0.014889	0.036455
11718416_PLA2G2A	-0.17503	0.014897	0.036472
11753616_ARL8B	0.104481	0.014898	0.036472
11734020_SHANK2	-0.1274	0.014898	0.036472
11720037_VTA1	-0.12229	0.014901	0.036477
11760261_PPIG	-0.2807	0.014902	0.036478
11750641_BCAR1	0.12524	0.014904	0.036481
11738754_OR4D10	-0.15477	0.014907	0.036485
11755983_UBE3D	0.143706	0.01491	0.036492
11759201_TMEM38A	-0.21641	0.014915	0.036503
11748730_CDC14B	0.189805	0.014917	0.036503
11715547_TMEM109	-0.11972	0.014917	0.036503
11740669_KLRF1	-0.23613	0.014918	0.036503
11739339_FAM46C	0.375151	0.014918	0.036503
11723533_BRMS1L	0.15776	0.01493	0.03653
11759421_SNRNP70	-0.22004	0.014938	0.036547
11736071_C8orf33	-0.12886	0.014939	0.036547
11730483_MYB	-0.16175	0.01495	0.036574
11716891_TMEM261	-0.22254	0.014956	0.036586
11721958_YPEL2	-0.14774	0.01496	0.036595
11719551_RHOBTB1	0.16998	0.014962	0.036597
11716837_CDV3	0.136282	0.014964	0.036597
11732271_ANGPTL3	0.175691	0.014965	0.036597
11726069_PDDC1	-0.12116	0.014965	0.036597
11717066_GREM1	-0.19663	0.014966	0.036597
11730423_APCDD1L	-0.16353	0.014966	0.036597
11738176_NKX3-2	-0.14252	0.014967	0.036597
11753954_VPS26A	0.116932	0.014969	0.036601
11743720_KSR2	-0.18343	0.014975	0.036613
11752619_FCRL2	-0.1852	0.014978	0.036619
11724616_MOCS1	-0.15485	0.014984	0.036633

11741772_TCEA2	-0.11723	0.014986	0.036636
11752548_ALDH1A1	0.200167	0.014988	0.036639
11733432_AK7	-0.1709	0.014993	0.036649
11744892_GOLT1B	0.103404	0.014995	0.036653
11740978_MGAT1	-0.19154	0.014999	0.036659
11743568_ADGRL2	0.219796	0.015003	0.036667
11737873_ACER1	-0.20851	0.015006	0.036673
11763939_LOC284441	0.173467	0.015012	0.036686
11763282_RPL23AP82	0.518871	0.015013	0.036687
11756231_TAF6L	-0.11458	0.015023	0.036709
11757377_DNAH14	0.167911	0.015026	0.036714
11733083_PALLD	0.151347	0.015037	0.036739
11751601_DPP7	-0.14196	0.015046	0.03676
11718632_PCBP4	-0.11842	0.015054	0.036778
11740779_CFAP74	-0.14692	0.015062	0.036795
11745368_WDR86	-0.14559	0.015067	0.036805
11751787_DBF4B	-0.16275	0.015068	0.036806
11733733_DPH5	-0.11613	0.015072	0.036815
11737588_KCNA7	-0.21354	0.015073	0.036815
11758193_B2M	0.111742	0.015075	0.036818
11724809_CBL	-0.15409	0.015076	0.036819
11720968_GART	-0.10438	0.01508	0.036826
11751614_PYROXD2	0.113106	0.015085	0.036837
11729313_RAC3	-0.1364	0.015087	0.036839
11747244_KCTD20	0.144469	0.015088	0.036839
11734329_TNN	-0.27465	0.015089	0.03684
11720061_WBP2	-0.10664	0.015095	0.036853
11735872_PTF1A	0.13012	0.015096	0.036855
11722748_HOOK3	-0.13824	0.015109	0.036885
11720047_ARMCX1	-0.1413	0.015112	0.036889
11726871_LEPR	0.109913	0.015113	0.03689
11736865_MMP16	0.112969	0.015117	0.036897
11747920_CLASP2	0.120575	0.015122	0.036908
11740915_RFX8	-0.21327	0.015126	0.036916
11727298_CASC10	0.17705	0.015129	0.036922
11718254_PPP1R10	0.128119	0.015135	0.036935
11723526_HOXB9	-0.12565	0.015137	0.036938
11759366_ZNF835	-0.19626	0.01514	0.036944
11736007_MAPK1IP1L	-0.30048	0.015142	0.036945
11738780_EPGN	-0.14104	0.015145	0.036951
11761780_GS1-124K5	0.137405	0.015146	0.036952
11739197_PCGF3	0.14309	0.015152	0.036965
11758372_EI24	0.119121	0.015156	0.036974
11737425_RASGRP4	-0.18329	0.015161	0.036983
11752677_GNL2	-0.1087	0.01518	0.037029
11735782_HHLA3	0.170843	0.015183	0.037031
11743918_ANK2	-0.22073	0.015183	0.037031
11750090_ZBTB8OS	0.125061	0.015184	0.037033
11729163_BTG3	-0.15051	0.015193	0.037052
11741380_ARNT	0.170061	0.015198	0.037062
11724027_PPP6R3	-0.12854	0.0152	0.037065

11719833_MPZL2	-0.17878	0.015215	0.037101
11763492_NRN1	-0.23336	0.015217	0.037104
11726253_AKIRIN2	-0.14995	0.01522	0.03711
11762493_GPR135	0.180475	0.015232	0.037137
11730215_CSRP1	-0.11514	0.015237	0.037146
11740542_CNGB3	-0.17856	0.015243	0.037159
11721848_REEP2	-0.14562	0.015244	0.037159
11738762_OR6Q1	-0.14658	0.015245	0.03716
11755661_KCNMA1	0.16734	0.015246	0.03716
11715770_SET	-0.10485	0.01525	0.03717
11757232_KCNQ1OT1	0.181555	0.015254	0.037176
11763329_DOK1	-0.12496	0.015256	0.037179
11735377_MSTN	-0.18774	0.015262	0.037192
11753549_CMTM3	0.111089	0.015264	0.037196
11746431_FBXO11	0.15043	0.015283	0.037239
11727983_LRRC46	-0.15557	0.015286	0.037245
11719060_ATRN	-0.16112	0.01529	0.037252
11754741_DZANK1	0.118876	0.01529	0.037252
11720502_UBE4B	-0.13002	0.015291	0.037252
11721782_SLC9A8	0.111269	0.015304	0.037281
11755390_GAPVD1	0.103188	0.015304	0.037281
11729348_HMGN5	-0.30364	0.015311	0.037296
11745126_FAM120C	-0.1261	0.015316	0.037306
11749487_SNRNP200	0.114571	0.015321	0.037317
11729803_LYPD5	0.299013	0.015328	0.037331
11755078_TADA2B	0.148516	0.015333	0.037341
11741109_ZNF57	0.169496	0.015339	0.037353
11759406_SEC16B	-0.20405	0.01534	0.037355
11739378_SREK1	0.130215	0.015343	0.03736
11727212_DTWD1	-0.29114	0.015346	0.037366
11743475_ITGAX	-0.14312	0.01535	0.037372
11757647_LINC00909	0.175437	0.015352	0.037375
11746134_NOP56	0.107214	0.015354	0.03738
11740412_PRKD2	-0.13	0.015358	0.037388
11741363_RAB23	0.134508	0.01536	0.037391
11742610_OR6C3	-0.12105	0.015363	0.037395
11760102_UBE2B	0.196633	0.015365	0.037399
11746046_UBQLN2	-0.12927	0.015366	0.037399
11760248_OXNAD1	0.136064	0.015368	0.037403
11761354_LOC100289	-0.15004	0.015374	0.037416
11761163_GS52	-0.24571	0.015379	0.037427
11718752_FUK	0.148158	0.015381	0.037429
11763117_IGHG1	0.309036	0.015383	0.037433
11720531_C9orf72	-0.15122	0.015387	0.037439
11729528_POMT2	0.196498	0.015393	0.037453
11758010_GOLGA6L1	-0.21603	0.015396	0.037457
11751837_HSPA4	-0.11709	0.015405	0.037478
11718661_NENF	0.129056	0.015409	0.037484
11731989_HESX1	0.185842	0.015412	0.037491
11748993_COG3	0.156485	0.015414	0.037495
11741327_GAGE1 ///	0.188323	0.015436	0.037543

11742411_CCDC168	0.174237	0.015436	0.037543
11743935_FGD1	-0.12628	0.015439	0.037549
11721228_LEMD3	0.114375	0.015444	0.037559
11735661_PLA2G2F	-0.13457	0.015462	0.037602
11740239_CFAP161	-0.19244	0.015463	0.037602
11750268_LINC00612	0.283207	0.015464	0.037602
11759796_HELZ2	0.141528	0.015466	0.037605
11748052_EI24	0.145625	0.01547	0.037613
11739756_ZNF74	0.208384	0.015474	0.03762
11716421_SPTBN1	-0.15199	0.015474	0.03762
11730157_CYP8B1	-0.12723	0.015481	0.037634
11733231_PTPRB	-0.20373	0.015484	0.037639
11734741_LIMK2	0.218733	0.015492	0.037657
11732231_ORAI2	0.138113	0.015513	0.037706
11732388_TCEANC2	0.12689	0.015522	0.037727
11759547_DDR2	-0.32074	0.015525	0.037733
11758991_FAM171A2	-0.21062	0.015528	0.037737
11737307_ATP1A4	-0.19355	0.01554	0.037765
11724717_DMAP1	-0.11286	0.015545	0.037775
11732668_APITD1-CC	-0.14814	0.015549	0.037782
11751676_AK2	-0.15293	0.015552	0.037789
11760227_GNAI2	-0.15597	0.015553	0.037789
11742057_CNTN1	-0.17453	0.015557	0.037795
11736804_ZNF43	0.147905	0.015557	0.037795
11725408_DEAF1	0.127561	0.015563	0.037807
11725196_ZSWIM7	0.225806	0.015566	0.037812
11737084_ZNF490	0.115698	0.015567	0.037814
11729000_CD84	-0.19965	0.015568	0.037814
11742972_CCAR1	0.162112	0.015574	0.037827
11738034_PCDHGB4	-0.21727	0.015576	0.03783
11740642_PLAC8	0.195991	0.015582	0.037842
11724955_LOC105369	-0.14458	0.015595	0.037871
11751058_OTX1	-0.15805	0.015595	0.037871
11729164_XPO5	-0.17076	0.0156	0.037881
11736266_TRAPPC8	-0.13314	0.015605	0.037891
11761637_MST1	-0.16186	0.015606	0.037891
11715914_EFNB1	-0.14758	0.01563	0.037945
11744331_CFB	-0.14619	0.01563	0.037945
11739873_ATAD3C	-0.11938	0.015633	0.03795
11761001_RHD	-0.16315	0.015644	0.037977
11753623_RAP1B ///	0.160437	0.015651	0.03799
11750314_WDR87	-0.14735	0.015651	0.03799
11721655_CISD2	-0.1397	0.015659	0.038004
11732868_FARP1	-0.26033	0.015659	0.038004
11719003_ANKH	-0.43769	0.015659	0.038004
11743238_LRRC30	-0.15022	0.01566	0.038004
11753290_CHODL	-0.11457	0.015666	0.038017
11746952_MDK	-0.11906	0.015673	0.038032
11732751_CC2D2A	0.193387	0.01568	0.038045
11741237_C4orf17	-0.2323	0.015682	0.038048
11718045_RAB11B	-0.11676	0.015685	0.038056

11741674_NTRK1	-0.12234	0.015688	0.03806
11736295_FGFBP3	-0.3289	0.015689	0.03806
11730949_MRPL52	0.106317	0.015689	0.03806
11761571_STK11IP	-0.25619	0.015696	0.038074
11741115_CYP3A7 //	0.269183	0.015712	0.03811
11731262_SLC4A5	-0.43522	0.015713	0.038111
11746665_PAXBP1	0.133489	0.015716	0.038115
11739697_AFF3	0.245728	0.015717	0.038115
11734976_FAM218A	0.160002	0.015717	0.038115
11751326_RAB11A	-0.10561	0.015726	0.038136
11740531_CLEC10A	-0.17959	0.015728	0.038139
11718103_RELA	0.181099	0.015731	0.038143
11718473_CLIP3	-0.16612	0.015734	0.038148
11715985_HTN3	-0.1458	0.015745	0.038173
11728709_EMP2	0.12412	0.015747	0.038176
11722377_PNPO	-0.11046	0.015754	0.038191
11729834_EIF5A2	-0.18588	0.015775	0.038241
11756351_SOD1	-0.10435	0.015778	0.038247
11745028_NKRF	0.119361	0.015779	0.038248
11734241_KIF19	-0.1834	0.015783	0.038253
11729443_PML	-0.12729	0.015783	0.038253
11745404_ADAMTSL5	0.118214	0.015799	0.038289
11725070_JADE1	-0.15087	0.0158	0.038289
11750614_GRIN1	-0.17253	0.015801	0.038289
11743397_CSDE1	-0.12846	0.015805	0.038299
11741525_C22orf24	-0.22677	0.015808	0.038302
11724533_CCDC184	-0.1588	0.015809	0.038304
11732835_MBD3L1	-0.27704	0.015812	0.03831
11747830_BAP1	-0.13748	0.015818	0.038321
11747105_HSPH1	0.10649	0.015819	0.038321
11724607_RTKN	-0.1554	0.01582	0.038321
11716589_TMEM165	0.106306	0.015821	0.038321
11751812_TMEM241	-0.18339	0.015821	0.038321
11720232_UCHL3	0.111525	0.015822	0.038321
11731786_ERC1	0.136574	0.015829	0.038338
11733091_TSPAN8	0.229859	0.015835	0.038349
11729597_TKFC	-0.13846	0.015835	0.038349
11750514_TP53	-0.11237	0.015856	0.038398
11751795_CPQ	0.310585	0.015864	0.038415
11725008_SEPT6	-0.17378	0.015873	0.038434
11734574_HIST1H3F	-0.18924	0.015884	0.038459
11717007_POLDIP3	-0.13678	0.015886	0.038461
11754067_TXNDC9	0.125173	0.015892	0.038474
11743132_SLN	-0.18061	0.015901	0.038495
11715336_SP9	-0.20868	0.015903	0.038499
11729368_GANC	0.136677	0.015913	0.03852
11728844_PROSER2	-0.13144	0.015914	0.03852
11752613_JAK3	-0.17139	0.01593	0.038557
11732951_ENTPD8	-0.14803	0.015937	0.038573
11732273_COA7	0.137365	0.015938	0.038573
11732287_DZIP3	-0.10962	0.01594	0.038577

11720843_N4BP1	-0.109	0.015942	0.038577
11750623_FILIP1L	-0.20601	0.015942	0.038577
11723751_TFF2	-0.18545	0.015946	0.038583
11740603_APH1B	0.177059	0.015946	0.038583
11752626_PBX1	-0.18784	0.015948	0.038585
11751959_NEK1	0.31834	0.015963	0.03862
11741878_PML	-0.15217	0.015969	0.038632
11735484_LANCL3	0.152931	0.015983	0.038664
11716968_NRP2	-0.26566	0.015984	0.038664
11720291_IQCE	-0.14585	0.015995	0.038691
11745410_SLC36A1	0.231396	0.015996	0.038691
11749559_CYP4F8	-0.15782	0.016	0.038698
11744665_RAB40C	-0.17616	0.016006	0.038711
11728454_DOCK9	0.16711	0.016007	0.038712
11726808_ZAK	-0.1552	0.016018	0.038736
11760909_LTBP4	-0.13324	0.01602	0.038738
11729462_CTNS	-0.12444	0.01602	0.038738
11758234_MED21	-0.13558	0.016022	0.03874
11722515_PARS2	-0.19285	0.016028	0.038753
11753513_UBXN2A	0.178549	0.016039	0.038776
11727187_WNT5A	0.238987	0.016041	0.038779
11716951_NANS	-0.11241	0.016042	0.038779
11751362_SUGCT	0.240034	0.016046	0.038788
11735881_MYBBP1A	-0.11277	0.016047	0.038789
11725165_C7orf31	-0.19475	0.016053	0.038801
11725969_THUMPD1	0.121908	0.016055	0.038803
11723718_CSNK2B	0.136635	0.016061	0.038817
11754763_GNPDA1	-0.12602	0.016068	0.038831
11760077_CEP290	-0.26206	0.016071	0.038837
11747453_PPP1R18	0.120147	0.016076	0.038847
11730513_LSMEM1	0.24829	0.016081	0.038856
11760101_IP6K2	-0.13415	0.016082	0.038857
11750965_SRL	-0.19758	0.016082	0.038857
11730305_FANCA	-0.12989	0.016085	0.038862
11728961_DISC1 ///	0.128092	0.01609	0.038869
11735303_HOXA11	-0.16605	0.01609	0.038869
11740108_BCL7A	-0.21154	0.016091	0.038869
11748802_GLDN	-0.13935	0.016092	0.038871
11746983_INTS3	-0.14703	0.016097	0.038881
11759460_ZNF836	-0.13683	0.016101	0.038888
11728033_SFT2D2	0.12266	0.016103	0.038892
11752810_DOLK	0.113471	0.016106	0.038896
11726884_LDHD	-0.12213	0.016111	0.038905
11738803_CACNA1G	-0.21382	0.016123	0.038932
11763622_FKBP9	-0.21115	0.016123	0.038932
11754616_UPF1	-0.1604	0.016129	0.038945
11755301_TOLLIP	0.192607	0.016131	0.038946
11735619_LEXM	0.164509	0.01614	0.038968
11750972_CACTIN	-0.14434	0.016145	0.038978
11722372_GGA2	-0.20626	0.016162	0.039015
11732629_ZNF765	0.306072	0.01617	0.039034

11760238_RWDD3	0.379471	0.016172	0.039037
11758322_GATSL2	-0.16796	0.0162	0.039101
11737302_CHST5	-0.16285	0.016203	0.039108
11749566_LCMT2	-0.14191	0.016204	0.039109
11730505_RD3	-0.11695	0.016207	0.039114
11756521_IREB2	-0.18279	0.01621	0.039118
11739608_ZNF805	0.112451	0.016217	0.039133
11736573_PRKCD	0.136303	0.016218	0.039133
11740470_HM13	0.128176	0.016223	0.039144
11757923_VPS41	0.169826	0.016225	0.039146
11739315_KDR	0.167846	0.016246	0.039196
11737090_NOS1AP	-0.19367	0.016247	0.039196
11724808_CBL	0.186442	0.016248	0.039197
11726899_SLC6A17	-0.14495	0.016252	0.039204
11755625_CCDC81	0.254557	0.01626	0.039221
11743738_NR3C1	-0.12439	0.016263	0.039228
11747587_VWA9	-0.11163	0.016271	0.039244
11739130_ABAT	-0.24327	0.016276	0.039255
11732773_CCDC57	0.12464	0.016278	0.039257
11742468_RASSF10	-0.1788	0.016286	0.039276
11743288_PEAK1	-0.12466	0.016292	0.039288
11740144_CEP164	0.13061	0.0163	0.039304
11755798_AMER1	-0.23348	0.0163	0.039304
11760324_MSI2	0.148991	0.016308	0.03932
11736858_ZNF182	-0.19248	0.016341	0.039397
11716870_PHC2	-0.12066	0.016348	0.039413
11721036_SUFU	-0.12536	0.016355	0.039428
11727254_TMEM51	-0.1511	0.016357	0.03943
11730043_C19orf45	-0.10638	0.016357	0.03943
11716669_RAB1A ///	-0.11367	0.016358	0.03943
11725529_ZNF248	-0.15863	0.016367	0.03945
11725582_IL17B	-0.12797	0.016369	0.039453
11762509_CBWD1 ///	0.191502	0.01637	0.039453
11731353_RIMBP3 //	-0.1784	0.016372	0.039455
11734384_AKR1E2	-0.20054	0.016375	0.03946
11760010_LINC00656	-0.23633	0.016381	0.039472
11758780_NOP16	0.114432	0.016383	0.039475
11718689_NES	-0.11996	0.016385	0.039478
11760896_SLC29A1	-0.18143	0.016395	0.0395
11716723_DPY19L1	-0.12145	0.016396	0.039502
11762058_FAM120B	0.163285	0.016398	0.039504
11721888_MID1	-0.12192	0.016399	0.039504
11747184_SHBG	-0.16122	0.016399	0.039504
11753464_MED16	-0.13056	0.016407	0.03952
11743906_UXS1	-0.12617	0.016413	0.039533
11756009_SHANK2	-0.2719	0.016422	0.039552
11715320_TTN	-0.15229	0.016433	0.039577
11761727_AQP7	-0.28893	0.016444	0.039602
11743227_CR1L	-0.21116	0.016447	0.039606
11722214_QTRT1	-0.1541	0.016448	0.039607
11719601_MOXD1	0.262554	0.01645	0.03961

11729034_ITPRIPL2	0.132996	0.016454	0.039618
11746657_VRK3	0.152461	0.016455	0.039618
11724875_CD36	0.160641	0.016471	0.039655
11718588_BEX4	-0.2821	0.016473	0.039658
11734159_HACE1	0.190276	0.016476	0.039662
11718328_SGSM2	-0.10427	0.016477	0.039662
11718377_PRKAG2	0.177485	0.016478	0.039664
11724009_GPR162	-0.1257	0.016479	0.039664
11718977_CAPN2	0.154583	0.016487	0.039682
11719950_BCAN	0.144575	0.016498	0.039705
11736089_TRIOBP	0.108628	0.016499	0.039706
11761230_CD79B	-0.23395	0.016516	0.039744
11745402_CYP2U1	0.262524	0.016516	0.039744
11729143_KDM5C	0.129049	0.016518	0.039746
11717728_HSPB7	-0.13799	0.016526	0.039764
11760917_GBF1	0.126214	0.016527	0.039765
11759621_TCL6	-0.17413	0.016529	0.039766
11753482_NPSR1	-0.1883	0.016545	0.039802
11760188_DDTL	-0.23354	0.016545	0.039802
11763798_STMN4	0.186985	0.016555	0.039824
11762111_PPT2 ///	-0.12931	0.016583	0.039888
11763479_FAAP20	-0.26839	0.016584	0.039889
11757033_IL13RA2	0.110563	0.016586	0.039893
11745936_SLC50A1	0.116179	0.016601	0.039926
11716609_CRABP2	0.120317	0.016622	0.039974
11763235_HSP90AB4F	-0.13942	0.016632	0.039996
11747178_GM2A	-0.35172	0.016633	0.039996
11754534_ZFYVE16	0.142271	0.01664	0.040012
11723309_TNFRSF10D	-0.15948	0.016641	0.040013
11751663_CD300LB	-0.18582	0.016645	0.04002
11746432_FBXO11	0.147059	0.016646	0.04002
11758670_STK3	-0.11297	0.016655	0.040041
11717943_EIF2B1	0.119432	0.016662	0.040055
11726879_TTBK1	-0.16665	0.01667	0.040073
11727267_SRPK3	-0.14904	0.016671	0.040073
11754509_SORBS1	0.153227	0.016674	0.040077
11741713_KCP	-0.12013	0.016678	0.040086
11743237_TMEM8C	-0.2234	0.016679	0.040087
11724974_MYRIP	0.232066	0.016682	0.040091
11727078_NUFIP1	0.137986	0.016693	0.040115
11721067_MGRN1	0.114066	0.016693	0.040115
11744360_RTN4	-0.18057	0.016695	0.040117
11736398_RFT1	-0.198	0.016701	0.04013
11734545_NARFL	-0.11645	0.016704	0.040136
11724581_ZNF827	0.216568	0.016706	0.040138
11740829_SYDE2	0.249128	0.016714	0.040153
11755370_BZRAP1	-0.20141	0.016715	0.040156
11719121_PPP1R14A	0.198894	0.016723	0.040172
11733248_DBNDD2 //	0.101529	0.016725	0.040174
11757562_CLEC18A /	-0.21551	0.01673	0.040184
11750818_TTC39B	0.261059	0.016735	0.040195

11755135_ABHD1	-0.24757	0.016737	0.040197
11734211_TMPRSS11B	-0.16313	0.016737	0.040197
11757588_ALG5	0.113809	0.016738	0.040197
11751196_VPS13D	-0.24176	0.01674	0.040198
11747318_TTC26	0.138505	0.016741	0.0402
11751556_NUDT13	0.259595	0.016743	0.040201
11763494_FRMD8	0.13746	0.016748	0.040212
11753167_SYPL2	-0.14894	0.016752	0.040221
11721502_SWAP70	-0.13857	0.016755	0.040225
11718246_SPIDR	-0.1102	0.016762	0.040241
11764166_EHF	0.27671	0.016771	0.040258
11757274_ARGLU1	0.102617	0.016771	0.040258
11733018_PIK3R1	0.13502	0.016773	0.040262
11727220_FITM1	-0.11818	0.016782	0.040278
11728222_TRAPPC3	-0.12381	0.016782	0.040278
11762121_DNMT3B	-0.24243	0.016785	0.040284
11763779_RGS12	-0.1605	0.016791	0.040297
11728525_C11orf63	0.337523	0.016792	0.040297
11746605_KIAA0586	-0.10209	0.016794	0.040298
11717519_LOC102724	0.129227	0.016799	0.04031
11757267_SIRT2	-0.13329	0.016806	0.040323
11727716_ITGA10	-0.16516	0.016813	0.04034
11716990_AREL1	0.183528	0.016815	0.040341
11728836_LRRTM1	-0.14735	0.016817	0.040344
11753666_PTN	0.201138	0.016818	0.040344
11745564_DNAJC4	-0.13403	0.016839	0.040391
11760952_HNRNPD	-0.24324	0.016839	0.040391
11736531_LRRFIP1	-0.19195	0.016841	0.040393
11738246_OR3A2	-0.14887	0.016842	0.040395
11729116_PRKCG	-0.15555	0.016848	0.040406
11733824_AGPAT3	-0.28712	0.016849	0.040407
11758006_SMARCAD1	-0.12859	0.016851	0.040411
11760319_HBP1	-0.34789	0.016852	0.040411
11758511_AK5	-0.24663	0.016853	0.040412
11723785_LIMCH1	-0.11563	0.016857	0.040419
11718711_SLC9A1	0.153828	0.01686	0.040425
11720285_TRAK1	0.12623	0.016862	0.040425
11752853_HNF1A	0.147473	0.016862	0.040425
11715263_SPEF2	-0.21344	0.016865	0.040429
11753677_WNT5A	0.130358	0.016866	0.040431
11751596_ABI1	0.109699	0.016869	0.040436
11737668_TAS2R4	-0.13344	0.016882	0.040464
11753007_BCR	-0.2305	0.016896	0.040496
11726849_SHC4	0.172328	0.016899	0.0405
11740019_TMEM200C	-0.18116	0.016931	0.040575
11754503_CEPT1	-0.1077	0.016931	0.040575
11739941_SENP1	-0.14877	0.016936	0.040583
11749068_ARMT1	0.131324	0.016939	0.04059
11744599_NUP62	-0.1145	0.016944	0.040599
11726414_EPM2A	-0.15865	0.016963	0.040644
11746304_NOP14	0.112223	0.01697	0.040658

11763717_CDKN1C	0.224595	0.016975	0.040665
11722996_NOL11	-0.12325	0.016976	0.040665
11736064_P2RX7	-0.16919	0.016976	0.040665
11733536_KCNG3	-0.21819	0.016982	0.040678
11747265_BCAS1	-0.26406	0.016984	0.04068
11749271_DNAH9	-0.21579	0.016985	0.04068
11715965_ST14	-0.17915	0.016985	0.04068
11725757_ISL1	0.220328	0.016986	0.040681
11720688_ZNF133	0.107387	0.016989	0.040686
11757004_KCTD10	-0.15438	0.016991	0.040688
11725987_MMP14	-0.15418	0.017003	0.040714
11743598_RNF207	0.16627	0.017005	0.040718
11734258_PRKAG3	-0.19758	0.017006	0.040718
11739452_ME2	0.119577	0.01702	0.040748
11761094_ZNF614	-0.15512	0.017021	0.040751
11752119_SLC17A5	0.141608	0.017023	0.040753
11722257_SCRN2	-0.15277	0.017028	0.040762
11723804_AKAP12	-0.11524	0.01703	0.040766
11724146_RAB38	-0.12508	0.017041	0.040789
11720589_PHF21A	0.119784	0.017042	0.040789
11754531_SUGP1	-0.11976	0.01705	0.040807
11741892_FOXI3	-0.16422	0.017052	0.04081
11760209_RPL37A	-0.15324	0.017054	0.040814
11758874_C17orf51	-0.16633	0.017066	0.04084
11719084_SMARCC2	-0.11015	0.017067	0.04084
11741901_KIR2DL5A	-0.1109	0.017074	0.040854
11759937_LINC01547	-0.13217	0.017076	0.040858
11760201_MAN1B1	-0.16622	0.01708	0.040865
11740028_CREM	0.111226	0.017081	0.040865
11748641_ZADH2	-0.19523	0.017086	0.040875
11736774_ZIC3	-0.27743	0.017104	0.040916
11725834_ALKBH1	-0.15093	0.017105	0.040917
11762666_AP5M1	0.260038	0.017109	0.040925
11762372_GSN-AS1	-0.2831	0.017122	0.040954
11755556_EXOC2	0.121464	0.017129	0.040968
11727475_TMEM260	0.183359	0.017131	0.040972
11735951_SSTR4	-0.17934	0.017134	0.040975
11717880_ATXN7L3	-0.14764	0.017134	0.040975
11732832_RNASEH2B	-0.14908	0.017139	0.040985
11751921_AHR	-0.11034	0.017147	0.041002
11719600_HOMER2	-0.11865	0.017151	0.041009
11730135_ZFR2	-0.13334	0.017153	0.041013
11753211_DHRS4 ///	-0.1371	0.017157	0.041018
11734875_PPFIBP1	0.13799	0.017162	0.041028
11732867_CEACAM21	-0.1264	0.017168	0.041041
11744928_SIAH1	-0.12384	0.01717	0.041043
11733973_PCDH18	0.143876	0.01717	0.041043
11741005_PARG	-0.18787	0.017175	0.041051
11736511_PTPRJ	0.273976	0.017184	0.041071
11729035_ITPRIPL2	0.134745	0.017185	0.041073
11749775_GTDC1	0.190889	0.017194	0.041091

11750028_ATP6AP2	0.111878	0.017207	0.04112
11753834_CXADR	0.239262	0.017211	0.041127
11752137_ADAMTS6	0.23055	0.017218	0.041142
11718814_PVRL1	-0.17269	0.017218	0.041142
11740671_COX15	0.124116	0.017221	0.041146
11747926_IFI16	-0.1038	0.017223	0.041148
11725034_SYP	-0.13432	0.017223	0.041148
11719007_BTBD6	-0.12298	0.017233	0.041168
11744869_B3GNT2	-0.19828	0.017244	0.041193
11729357_PAPD4	-0.14993	0.017254	0.041215
11718617_DNAJA4	-0.10792	0.017256	0.041217
11731253_C16orf13	-0.12171	0.017265	0.041236
11733210_BRCC3	0.190351	0.017269	0.041243
11726019_EEPD1	-0.19425	0.017273	0.041251
11749313_ABI1	0.125067	0.017286	0.041279
11726763_RLBP1	-0.1729	0.017286	0.041279
11738625_SLC17A8	-0.19428	0.017287	0.041279
11739115_TSPYL1	0.120558	0.01729	0.041285
11755317_CBWD1 ///	0.142517	0.017303	0.041314
11717469_EPT1	0.200449	0.017311	0.04133
11726047_ASXL2	0.159223	0.017311	0.04133
11724271_HLF	0.124876	0.017314	0.041333
11746752_NOTCH4	-0.13188	0.017316	0.041334
11718414_NONO	0.105011	0.017316	0.041334
11724968_USP31	0.164352	0.017316	0.041334
11717625_TBL2	0.132136	0.017323	0.041346
11754019_CD99	0.148711	0.017326	0.041353
11738821_NOX5	-0.11902	0.017331	0.041361
11718883_ARL5A	0.121603	0.017339	0.041379
11729476_GGN	-0.18046	0.017343	0.041385
11724589_MMP12	0.208313	0.017343	0.041385
11732631_KCNN3	-0.1639	0.017351	0.041401
11727940_SLC45A3	-0.14477	0.017357	0.041414
11725084_UCHL5	-0.11016	0.017363	0.041428
11760365_DPAGT1	-0.13018	0.017374	0.041451
11731216_MATN4	-0.21798	0.017379	0.04146
11734403_MTCP1	0.17877	0.017382	0.041467
11759488_EYA3	0.126356	0.017383	0.041467
11734620_VPS13B	0.276556	0.017396	0.041494
11739911_PCBD2	0.184223	0.017398	0.041498
11758480_COG8 ///	0.109434	0.017406	0.041515
11717955_STK39	0.129392	0.017421	0.041548
11732286_DZIP3	-0.24443	0.017424	0.041554
11763450_MPHOSPH8	0.131451	0.017427	0.041559
11730145_KLHL31	0.139386	0.017433	0.04157
11763849_STYXL1	-0.27607	0.017438	0.041581
11747878_STX3	0.11657	0.017455	0.041618
11730240_SLC2A12	0.17005	0.017455	0.041618
11760208_AK2	0.174471	0.017458	0.041623
11753293_BEST1	-0.24304	0.017462	0.041631
11715738_SNX3	-0.11334	0.01747	0.041644

11719226_MOB1B	0.132972	0.01747	0.041644
11744551_CHRNA3	0.236336	0.017483	0.041674
11731990_CYP3A43	0.131083	0.017485	0.041678
11742420_GRM5	0.247457	0.017493	0.041694
11731874_CTAGE5	-0.16142	0.017498	0.041704
11734488_HIPK1	0.182208	0.017503	0.041714
11757545_PECAM1	-0.13232	0.017507	0.041722
11746000_ZNF252P	0.160244	0.017522	0.041755
11727052_HOXB13	-0.17136	0.017529	0.041769
11752988_BCR	-0.26981	0.017545	0.041805
11717463_NSUN2	0.116396	0.017546	0.041807
11754944_USP39	-0.10612	0.017548	0.04181
11739095_CPSF7	-0.14093	0.017551	0.041814
11756317_ZNF276	0.156157	0.017553	0.041817
11756355_TP73	-0.15578	0.017563	0.041838
11750486_HDAC8	-0.21879	0.017571	0.041853
11737437_CNTLN	-0.20173	0.017571	0.041853
11728879_PPP3R1	0.174646	0.017572	0.041854
11761051_ROBO3	-0.17018	0.017573	0.041854
11738454_ACTBL2	0.202021	0.017575	0.041857
11730121_ATP8B4	0.341399	0.017576	0.041857
11737172_CD80	-0.19718	0.017583	0.041869
11723276_CREBZF	0.162863	0.017583	0.041869
11744487_CNBP	0.126453	0.017584	0.041869
11738012_INHBC	-0.16333	0.017586	0.041874
11731092_BRD1	0.206125	0.017589	0.041877
11730585_ABCG4	-0.22025	0.017597	0.041895
11721712_SHROOM3	-0.12188	0.017608	0.04192
11719809_BLOC1S6	0.114435	0.017615	0.041934
11748595_GABRR2	-0.17499	0.017617	0.041937
11743584_PRELID3B	-0.11161	0.01762	0.04194
11755865_SLC22A8	-0.14119	0.017622	0.041945
11746252_DENR	0.123068	0.017623	0.041946
11723900_USF3	-0.14758	0.017625	0.041946
11747049_B4GALT1	-0.24358	0.01763	0.041956
11717413_WIPI1	-0.10195	0.01764	0.041979
11742391_OR1L4	-0.22594	0.017646	0.041991
11739482_PLAC8	0.161833	0.017649	0.041996
11736994_DNAJB7	-0.16186	0.01765	0.041997
11755139_RPL14	0.102615	0.017656	0.042007
11731631_SOX30	-0.27541	0.017657	0.042007
11736764_ZNF347	0.227871	0.017657	0.042007
11746246_GK3P	-0.17187	0.017665	0.042024
11753397_DLK1	-0.12466	0.017668	0.042028
11760843_CTAGE3P	-0.14217	0.017682	0.04206
11730630_WDR72	-0.15509	0.01769	0.042078
11743525_KDM5A	0.13935	0.017692	0.042079
11720610_PHB	-0.12654	0.017701	0.042098
11718821_MTIF2	-0.11535	0.017701	0.042098
11728333_ELL	0.114041	0.017703	0.0421
11722206_CDC42EP1	-0.15119	0.017706	0.042105

11746992_MFAP3L	-0.21016	0.017713	0.04212
11753944_TRA2B	-0.1432	0.017714	0.042121
11760134_LRCH3	0.249719	0.017719	0.04213
11755471_NECAB2	-0.14251	0.017736	0.042167
11731902_AXDND1	0.104351	0.017737	0.042167
11763383_STYXL1	-0.14654	0.017737	0.042167
11721396_MUM1	-0.15489	0.017739	0.042168
11761546_HDGFL1	-0.24606	0.01774	0.04217
11726350_ANKRD39	-0.20572	0.017741	0.04217
11764249_LOC105369	0.174813	0.017747	0.042182
11723323_TDP1	-0.13647	0.017753	0.042193
11741073_TCP11L1	0.15315	0.017755	0.042195
11719361_SLF2	0.327572	0.017756	0.042195
11749014_SEMA3F	-0.12764	0.017756	0.042195
11717594_ELF3	-0.23192	0.017769	0.042224
11735567_MMP27	-0.16418	0.017772	0.042229
11731923_POFUT2	0.378418	0.017777	0.042239
11718986_IFI6	0.113955	0.017779	0.042241
11759970_STAT5B	-0.13861	0.017799	0.042288
11723735_RNF216	-0.13102	0.017822	0.04234
11749551_RNGTT	0.107531	0.017826	0.042348
11719087_CCDC124	-0.14459	0.017851	0.042405
11755224_RBM33	-0.19628	0.017861	0.042425
11718645_OSBPL8	0.131688	0.017861	0.042425
11719550_RHOBTB1	0.272104	0.017865	0.042429
11731316_CPNE9	-0.15682	0.017866	0.042429
11728788_SLC32A1	-0.13497	0.017866	0.042429
11723320_CALY	-0.12149	0.017873	0.042444
11753669_CHEK2	-0.14985	0.017879	0.042456
11756217_SNX22	-0.15596	0.017882	0.042462
11740361_FOXN3	-0.13789	0.017886	0.042469
11747268_NBR1	0.146459	0.017898	0.042496
11752308_ADAM18	-0.20514	0.017899	0.042496
11723374_GLI3	0.121042	0.017901	0.042499
11731186_PPM1E	0.253085	0.017905	0.042506
11756318_TRA2B	-0.10369	0.017928	0.042559
11719484_EPS15	-0.13879	0.01793	0.042561
11732978_ALB	-0.29599	0.017936	0.042573
11732541_HSD17B13	-0.13925	0.017946	0.042595
11757010_ACP2	-0.18131	0.01795	0.042603
11753392_MSH5 ///	-0.19042	0.017959	0.042621
11753112_MED25	-0.14425	0.017961	0.042625
11728439_BRWD1	-0.12258	0.017963	0.042627
11753659_RPL30	0.102503	0.017967	0.042633
11739721_GIMAP8	-0.1831	0.017975	0.042652
11721150_RAB40C	-0.17049	0.017979	0.042658
11755265_NT5DC1	-0.10804	0.017982	0.042665
11755836_NARFL	-0.12105	0.017986	0.042672
11736795_GHSR	-0.12174	0.017987	0.042672
11743158_GTF2F2	0.109636	0.017999	0.042697
11729681_PHYH	-0.14542	0.018002	0.042702

11715792_STOM	0.122589	0.018002	0.042702
11738775_CLEC2A	-0.13178	0.018004	0.042703
11739579_PGBD1	0.155661	0.018006	0.042708
11748524_PIGK	-0.16027	0.018029	0.042759
11729887_HACD1	-0.11048	0.018031	0.042761
11763926_SNORA70C	0.249181	0.018032	0.042761
11739862_DDX26B	0.311392	0.018034	0.042764
11732725_GCM1	-0.14511	0.018042	0.042782
11716797_LBR	0.128118	0.018043	0.042782
11719442_WNT5B	-0.10731	0.01805	0.042797
11751756_RBM14	-0.19735	0.018057	0.04281
11731479_TXNDC9	-0.12694	0.018059	0.042813
11753188_DOC2A	-0.12012	0.018068	0.042833
11717216_ATP9A	-0.14756	0.018069	0.042834
11757020_MGST3	-0.11531	0.018078	0.042853
11725517_ABCG1	-0.30089	0.018085	0.042866
11735364_AKTIP	-0.23302	0.018089	0.042874
11737615_ERICH6B	-0.12232	0.0181	0.042899
11746685_PXK	-0.14487	0.018102	0.0429
11740893_RASSF7	-0.1893	0.018105	0.042905
11729196_STX16	-0.11822	0.018109	0.04291
11721807_EMC1	-0.14295	0.018109	0.04291
11716470_UQCRB	-0.20377	0.018109	0.04291
11759252_HIST1H2AI	0.158296	0.01811	0.04291
11733683_FAM221B	-0.20394	0.018115	0.042919
11761330_NME9	-0.14481	0.01812	0.04293
11729217_DDX4	-0.15435	0.018128	0.042945
11746694_PAFAH1B1	0.259607	0.018136	0.042963
11744473_BAG6	0.125257	0.018143	0.042974
11763846_SPATA20	0.11451	0.018143	0.042974
11754256_MCPH1	0.209596	0.018143	0.042974
11723942_PTTG1IP	0.125488	0.018151	0.042991
11726898_ADGRL3	-0.17559	0.018157	0.043002
11720818_CXCL12	0.140546	0.018167	0.043023
11760892_LY9	-0.26039	0.018171	0.04303
11742572_OR5D14	-0.16867	0.018174	0.043035
11757253_CDRT15P1	-0.19524	0.018177	0.043041
11760206_GNL1	-0.14428	0.018179	0.043042
11717848_QRICH1	-0.1158	0.01818	0.043043
11715712_NRDC	-0.12289	0.018196	0.04308
11751172_TRIB3	-0.23643	0.018198	0.043083
11725389_CD6	-0.16912	0.018203	0.043091
11742101_QRFPR	-0.16893	0.018204	0.043092
11732717_SYCE3	-0.12594	0.018205	0.043093
11717293_SRSF7	0.100732	0.01821	0.043103
11761674_PLA2G7	-0.22539	0.018215	0.043111
11719397_RRAGC	0.111779	0.01822	0.043122
11750925_TOX	0.157546	0.018226	0.043134
11755115_NRBP1	0.119344	0.018238	0.043159
11723929_OGDHL	-0.28625	0.018247	0.043179
11752471_NEU3	-0.12765	0.018253	0.043191

11756585_AQP3	0.171763	0.018254	0.043193
11764057_RSRP1	0.327095	0.018256	0.043193
11749011_BRSK2	-0.113	0.018257	0.043195
11728611_FAM159A	-0.14635	0.018259	0.043195
11755736_NOX4	0.36319	0.018259	0.043195
11746077_NARFL	-0.13253	0.018263	0.043203
11753123_CNP	-0.25564	0.018264	0.043203
11749340_SIAH1	0.150614	0.018267	0.043208
11752272_MAN1B1	-0.10122	0.018268	0.043208
11741576_LEMD2	0.127767	0.018273	0.043216
11721471_EIF3A	0.117871	0.018273	0.043216
11755917_NXF5	-0.20292	0.018274	0.043216
11717500_CTSF	-0.21253	0.018276	0.043219
11726479_MX2	-0.13453	0.018279	0.043223
11746181_TOE1	-0.16474	0.01828	0.043223
11720514_LURAP1L	0.113532	0.018282	0.043228
200097_PM_HNRNPK	-0.11778	0.018292	0.043249
11750475_FAM153A /	-0.18515	0.018294	0.043252
11733706_COL11A1	0.168229	0.018296	0.043254
11741566_SYNPO2L	-0.21108	0.018297	0.043254
11730162_SLC6A10P	0.11866	0.0183	0.043258
11727690_IL11	0.193359	0.018301	0.04326
11727604_EPB41L4A	-0.19992	0.018305	0.043266
11740635_C19orf57	-0.20433	0.018311	0.043279
11715661_TMEM189-U	0.101127	0.018319	0.043293
11735277_TMEM189-U	0.101127	0.018319	0.043293
11726604_PAFAH1B2	0.146032	0.018322	0.043298
11747483_TNFAIP8	-0.39235	0.018323	0.043298
11730354_CYP26B1	-0.17423	0.018327	0.043304
11715836_PSMA3	-0.10566	0.018328	0.043304
11720139_VSTM2L	0.109982	0.018328	0.043304
11724507_DUS2	0.134714	0.018346	0.043344
11749695_NDC1	0.129161	0.018349	0.043349
11757327_FTL	0.102687	0.018355	0.043362
11762007_SLC4A8	-0.30957	0.01836	0.043372
11721289_TPM3	-0.24158	0.018361	0.043372
11715479_GABARAP	0.115032	0.018366	0.043381
11716422_SPTBN1	-0.12484	0.01837	0.043388
11715693_CIRBP	-0.15908	0.018392	0.043438
11742045_PAK6	-0.17171	0.018401	0.043458
11728917_SLC7A4	-0.18119	0.018403	0.04346
11759297_FAM114A1	-0.11608	0.018407	0.043467
11729755_ULK2	-0.25405	0.018412	0.043478
11725851_ZNF319	0.207989	0.018414	0.043481
11715480_GABARAP	0.113314	0.018417	0.043485
11723056_MRPL33	0.104865	0.01842	0.043489
11745867_PSEN2	0.172207	0.018421	0.043491
11758380_TRIM45	-0.16631	0.018449	0.043554
11721764_ADRBK2	-0.14697	0.018452	0.043559
11718898_SPAG9	-0.11331	0.018461	0.043576
11754478_ATP6V0B	0.112679	0.018461	0.043576

11746093_POLR2A	-0.14271	0.018465	0.043583
11748592_NFASC	0.117554	0.018471	0.043594
11760954_EIF3G	-0.16171	0.018471	0.043594
11763508_CINP	-0.20232	0.018474	0.043599
11762957_DPY30	0.140356	0.018478	0.043605
11740672_COX15	0.11033	0.018481	0.04361
11762323_HCN3	0.149998	0.018482	0.043611
11752723_CD200R1	0.268049	0.018483	0.043611
11742010_S100G	-0.25798	0.018487	0.043618
11756749_EGF	0.217519	0.018488	0.04362
11750281_TLDC1	-0.21208	0.018504	0.043654
11763360_VSIG2	-0.20727	0.018507	0.04366
11743208_GPRC5D	-0.17294	0.018513	0.043671
11716431_SLC39A13	-0.14189	0.018514	0.043673
11733928_ARMC8	0.247586	0.01852	0.043685
11754332_SYDE1	0.178806	0.018541	0.043731
11719496_HMGB4	-0.19282	0.018544	0.043736
11752701_RHCE ///	-0.13536	0.018558	0.043766
11757707_HIGD1B	-0.13884	0.018559	0.043766
11728932_FXYD7	-0.18314	0.018563	0.043775
11736785_HOXA2	-0.17984	0.018572	0.043794
11730946_TRMT2A	-0.1919	0.018574	0.043797
11721187_ARHGEF9	0.132708	0.018577	0.0438
11746743_RUVBL2	-0.10663	0.018587	0.043824
11749264_DDX25	-0.18631	0.018596	0.043839
11745461_C1QTNF9 /	-0.13832	0.018596	0.043839
11759467_VSTM5	-0.13887	0.018608	0.043866
11734573_HIST1H3F	-0.14066	0.018609	0.043866
11753413_DLK1	-0.19691	0.01861	0.043866
11752338_SYTL5	0.188312	0.018611	0.043868
11759138_FAM45A //	-0.37873	0.018614	0.043872
11719995_TLE4	0.140202	0.018617	0.043874
11757939_SEPT11	0.124671	0.018617	0.043874
11750059_MLX	0.109758	0.018622	0.043883
11723487_LAMB1	0.130168	0.018624	0.043885
11741961_CD209	-0.18831	0.018624	0.043885
11736605_XPO7	-0.12754	0.018638	0.043915
11736723_PDLIM7	-0.14223	0.018639	0.043915
11715311_DEFB103A	-0.2495	0.018639	0.043915
11746860_SGSM1	-0.16528	0.018649	0.043935
11761303_POLR1C	0.247079	0.018658	0.043955
11762939_ERVH-6	-0.12533	0.018661	0.043958
11718030_RAB11A	-0.1144	0.018667	0.043972
11733574_PGBD4	-0.15922	0.01867	0.043976
11722105_PIGF	-0.13148	0.018672	0.043979
11744341_SARNP	0.125118	0.01869	0.044019
11753997_RLTPR	-0.20297	0.018695	0.044028
11744126_PRKCSH	-0.11056	0.018707	0.044054
11729168_DCTD	0.116004	0.018708	0.044054
11717160_CLTA	0.108611	0.01871	0.044058
11734379_C11orf53	-0.18319	0.018713	0.044062

11715618_SDF4	0.144384	0.01872	0.044078
11725351_KIAA0930	-0.24262	0.018733	0.044104
11763971_LINC01082	0.178455	0.018737	0.044113
11729951_ZBTB37	0.15103	0.018745	0.04413
11752100_E2F3	0.147618	0.018762	0.044166
11732032_HMGA2	-0.19429	0.018765	0.044172
11747040_RABEP2	-0.24928	0.018766	0.044172
11734412_ZNF280A	0.246884	0.018775	0.044191
11722344_GNB5	-0.11514	0.018778	0.044196
11749499_MED25	-0.12313	0.018782	0.044204
11723956_APOBEC3C	0.103711	0.018784	0.044207
11740158_ATG10	-0.15102	0.018793	0.044226
11760676_NUPL2	-0.16875	0.018794	0.044226
11743289_PEAK1	-0.12572	0.018799	0.044235
11719655_SV2A	-0.15298	0.018801	0.044237
11759788_NUP210P1	-0.33216	0.01881	0.044257
11718623_PLA2G1B	-0.27175	0.018812	0.04426
11734999_SIPA1L3	-0.10105	0.018819	0.044273
11762070_SLCO2B1	-0.19526	0.018821	0.044277
11747165_LOC100499	-0.13125	0.018823	0.044278
11761482_MPI	0.147531	0.018836	0.044305
11725833_ALKBH1	0.128989	0.018836	0.044305
11721546_ZEB1	-0.12305	0.018839	0.044311
11720377_SAP30L	-0.16003	0.018842	0.044315
11760315_PXN	0.119554	0.018843	0.044316
11738707_OR8J1	-0.18619	0.018845	0.044317
11730617_ULBP1	-0.25093	0.01885	0.044327
11762598_CARF	-0.17804	0.018855	0.044338
11762293_C9orf3	0.160025	0.018856	0.044338
11747705_TBC1D3P2	0.216447	0.018859	0.044342
11747786_CLN3	-0.21136	0.018861	0.044346
11724015_FRA10AC1	-0.12896	0.018865	0.044353
11717476_CHGB	0.240865	0.018873	0.04437
11721657_LMO4	-0.26707	0.01888	0.044383
11719865_ENO1 ///	-0.19997	0.018887	0.044398
11763229_IGLJ3 ///	-0.20471	0.01889	0.044401
11744976_DOC2A	-0.17153	0.01889	0.044401
11754062_YIPF3	0.125055	0.018892	0.044404
11763399_MEI1	-0.13319	0.018897	0.044411
11723057_MRPL33	0.118757	0.0189	0.044418
11763105_TTTY6B	0.276683	0.018902	0.044419
200030_PM_SLC25A3	0.10081	0.018913	0.044443
11742058_ECE2	-0.14842	0.018923	0.044466
11725728_C1orf56	0.272926	0.018924	0.044466
11759432_TDRP	-0.15744	0.018926	0.044467
11737982_SMYD3	0.124157	0.018936	0.044489
11734008_NDOR1	-0.12493	0.018943	0.044503
11731753_ZNF577	0.189391	0.018945	0.044507
11739416_IRGQ	0.131641	0.018949	0.044514
11736703_MAP3K9	-0.10568	0.018952	0.044519
11732958_USF1	-0.11562	0.01896	0.044536

11762971_NARFL	-0.11607	0.018967	0.044549
11744569_RNMT	0.221032	0.01897	0.044553
11751648_HGS	0.114286	0.018979	0.044573
11756764_RPL14	0.111077	0.01898	0.044573
11763204_KRT16P1	-0.1225	0.018982	0.044575
11728850_RAB39B	0.136405	0.018985	0.04458
11741620_SMARCD1	-0.1174	0.018991	0.044591
11728688_SFXN1	0.105889	0.018994	0.044597
11758768_AMD1	0.126733	0.018999	0.044608
11739898_ZNF280C	0.225544	0.019001	0.044609
11720763_SELPLG	0.121375	0.019004	0.044613
11721680_TM2D1	0.119234	0.019004	0.044613
11722115_RPS6KC1	-0.11284	0.019006	0.044615
11739896_ZNF280C	0.148061	0.019021	0.044649
11754292_TSSC1	-0.10064	0.019022	0.044649
11725551_PDE4A	-0.16378	0.019038	0.044684
11718539_NRN1	-0.16595	0.019044	0.044696
11747663_L3HYPDH	0.100187	0.019047	0.044701
11725721_MRM1	-0.17984	0.019056	0.044718
11730780_ADH1C	-0.16341	0.019056	0.044718
11723712_MLXIP	-0.15604	0.01909	0.044795
11734715_DBF4	-0.12455	0.019098	0.04481
11724091_RNF208	-0.15494	0.019098	0.04481
11722982_LYST	0.265566	0.019108	0.04483
11734317_STARD8	-0.13408	0.019109	0.04483
11716140_SMS	0.100595	0.019131	0.044879
11756854_EHBP1	0.1151	0.01914	0.0449
11722877_NDUFAF1	-0.12535	0.019151	0.044923
11754210_CACNA2D2	-0.13878	0.019161	0.044944
11744134_ZSWIM6	0.116557	0.019165	0.044952
11742014_METTL20	-0.22739	0.01917	0.044961
11723844_TMEM183A	0.109201	0.019172	0.044962
11718967_DDX6	0.107259	0.019173	0.044963
11745288_NOX5	0.279703	0.019181	0.044979
11763134_ZBTB8OS	0.278988	0.019181	0.044979
11730888_USF2	-0.20562	0.019183	0.044982
11727696_OGG1	-0.1248	0.019188	0.04499
11740805_RPUSD4	0.147379	0.019189	0.04499
11763884_LINC01000	0.251518	0.019208	0.045033
11741235_JKAMP	0.116112	0.01921	0.045035
11718746_ARHGAP21	-0.10607	0.019216	0.045047
11753789_ST3GAL3	-0.23473	0.019218	0.045049
11745937_TCF3	-0.18528	0.019219	0.045049
11751366_GEMIN4	-0.18189	0.01922	0.045051
11738678_OR8G5	-0.12565	0.019234	0.045081
11756499_KLHDC2	-0.11807	0.019249	0.045114
11722926_MMRN1	-0.18245	0.019251	0.045117
11761169_ACAN	-0.16543	0.019254	0.04512
11741592_ABCD3	0.146957	0.019257	0.045124
11747907_PDZK1 ///	0.129263	0.019257	0.045124
11740201_FCMR	0.121471	0.019264	0.045138

11759552_MPI	-0.1374	0.019266	0.04514
11725946_MPP2	-0.21558	0.019269	0.045144
11731547_TMEM67	0.368775	0.01927	0.045144
11725881_C15orf52	0.10093	0.019282	0.045171
11716561_STRN4	0.158625	0.019297	0.045204
11743202_IGF1	-0.1317	0.019305	0.045221
11743312_TMEM218	-0.11684	0.019307	0.045225
11742566_OR2V2	-0.16538	0.019314	0.045238
11716016_MAL2	0.253541	0.019316	0.04524
11747276_AKAP1	-0.12525	0.019318	0.045242
11751970_ABCC13	0.270244	0.019321	0.045247
11716314_MRPL15	-0.32673	0.019325	0.045254
11720366_FDX1L	0.097619	0.019326	0.045254
11718290_FXYD5	0.118553	0.01933	0.045262
11752201_SP140 ///	0.318759	0.019332	0.045266
11727602_PF4	-0.14766	0.019336	0.045271
11759622_DDX21	0.099025	0.019346	0.045292
11759486_GPBP1	-0.17771	0.019347	0.045292
11717603_SECISBP2	0.163384	0.019363	0.045329
11747636_PLA2G6	-0.14784	0.019364	0.045329
11725327_CAMK2D	-0.12384	0.01937	0.045341
11747289_PER1	0.19525	0.019372	0.045342
11744837_CDH13	0.159005	0.019375	0.045348
11751085_GBA /// G	0.116158	0.019376	0.045348
11749328_CD99L2	-0.19245	0.019385	0.045367
11722303_LRRFIP1	-0.11464	0.019389	0.045375
11727341_ATG9B	-0.20257	0.019396	0.04539
11733679_TMIGD2	-0.16542	0.019398	0.045391
11722849_PPP3CA	0.111852	0.019403	0.045401
11734564_C1QTNF3	0.152689	0.019405	0.045403
11752148_FOXP2	-0.16124	0.019434	0.045467
11726530_BRCC3	0.191231	0.019435	0.045467
11759822_RPL37A	-0.34217	0.019435	0.045467
11760036_PPIL6	-0.21142	0.019438	0.045471
11746388_SPIDR	-0.129	0.019439	0.045471
11729400_PCP4L1	-0.23738	0.019441	0.045475
11726851_ZC3H3	-0.13544	0.019444	0.04548
11722917_IGF2BP3	0.112603	0.019447	0.045484
11722614_CYP2J2	0.206898	0.019456	0.045502
11737152_C4orf51	-0.12225	0.019462	0.045514
11735110_FBXO3	0.134749	0.019464	0.045516
11718536_NKTR	0.106877	0.019465	0.045518
11753798_TSG101	0.124367	0.019474	0.045536
11736451_SRFBP1	0.25577	0.019477	0.045541
11717416_RHOU	-0.17278	0.019479	0.045543
11718006_MYLIP	-0.16143	0.01948	0.045544
11733469_TMEM164	0.166259	0.019488	0.04556
11724716_BCOR	0.130923	0.019489	0.04556
11758103_TMED3	-0.11697	0.019494	0.04557
11724857_CEP63	0.129414	0.019495	0.04557
11758989_USP1	-0.33344	0.019498	0.045575

11757929_RALGAPA2	-0.10035	0.019503	0.045584
11733415_PAGE5	0.130214	0.019507	0.04559
11732478_XAGE2	0.214832	0.019507	0.04559
11760393_GRK4	0.232696	0.019508	0.04559
11725954_RNF10	0.107995	0.01951	0.04559
11746502_ZNF814	0.135009	0.01951	0.04559
11730797_FAM114A2	-0.14065	0.019517	0.045604
11735819_C3orf20	-0.21062	0.01952	0.04561
11734688_MBD2	-0.16635	0.019527	0.045624
11719388_MAFK	0.143259	0.019536	0.045641
11753602_HLA-G	-0.16751	0.019553	0.045679
11756488_KIF2B	-0.217	0.019554	0.045679
11731364_CCDC63	-0.20485	0.019556	0.045682
11733393_KCNMB3	0.194745	0.01957	0.04571
11759923_TRIM4	0.165057	0.019571	0.04571
11744106_ATP9B	0.188495	0.019571	0.04571
11741503_CREM	-0.26149	0.019575	0.045719
11721552_GABBR1	0.106146	0.019578	0.045723
11717425_CHMP3 ///	0.109267	0.01958	0.045725
11741461_PCDHGB2	-0.17748	0.019584	0.045732
11734863_SARNP	0.119094	0.019586	0.045736
11747062_PROM2	-0.14507	0.01959	0.045742
11737543_TMEM202	-0.15454	0.019599	0.04576
11760788_BBS1	-0.14056	0.019601	0.045763
11724699_ARHGEF37	-0.15216	0.019603	0.045765
11716510_GTF2E2	0.099107	0.019604	0.045765
11734173_CALR3	-0.11644	0.019624	0.045811
11736017_PXN	-0.10328	0.019646	0.045858
11744855_SERPINB9F	-0.16869	0.019655	0.045879
11717088_RBM39	0.109461	0.019669	0.04591
11751828_NFS1	-0.17578	0.019676	0.045923
11716943_EXOSC5	-0.1168	0.01968	0.045929
11726487_CPED1	-0.20008	0.01968	0.045929
11753032_GPR85	0.226782	0.019684	0.045934
11740043_MTFMT	-0.17667	0.019687	0.04594
11723815_ARHGAP29	0.128191	0.019688	0.045941
11749492_RPS6KA1	-0.11307	0.01969	0.045941
11725993_SLC22A8	-0.14571	0.019694	0.045947
11742837_RPS6KB2	-0.1304	0.019695	0.045947
11756659_FBXL7	-0.12285	0.019695	0.045947
11762210_PRKAA1	-0.17723	0.0197	0.045958
11728078_TBC1D22B	0.158715	0.019709	0.045976
11739715_NCOA2	0.198402	0.019717	0.045991
11721728_ARHGAP30	-0.12402	0.01972	0.045994
11745188_BET1L	-0.11906	0.01972	0.045994
11737774_RNF182	0.168805	0.01972	0.045994
11744235_TJAP1	0.131754	0.019723	0.045998
11755955_FAP	0.13339	0.019724	0.045998
11757544_ICAM2	-0.14597	0.019733	0.046017
11739118_CNPY3	-0.13554	0.019736	0.046021
11719006_ANKH	-0.23718	0.01974	0.046028

11748650_ADAM33	-0.12532	0.019748	0.046045
11761982_USP32P1	-0.1546	0.019749	0.046045
11726046_FCGR2B	-0.20948	0.01975	0.046046
11734796_HIST1H2BG	0.375699	0.019754	0.046051
11743947_GGACT	0.243194	0.019755	0.046051
11727313_BHLHE22	-0.16496	0.019763	0.046066
11729153_CEACAM6	-0.14877	0.019763	0.046066
11745176_TPD52L2	-0.16451	0.019764	0.046066
11727634_BAG6	0.117068	0.019768	0.046074
11722265_WDR33	0.146175	0.01978	0.0461
11761086_AKT2	-0.16102	0.019786	0.046111
11761507_SLC35D1	0.194028	0.019797	0.046135
11755867_HNRNPUL1	-0.11698	0.019804	0.046148
11734427_TRIM71	-0.1489	0.019805	0.046148
11747948_SMAP2	0.196982	0.019813	0.046164
11721438_DEFA1 ///	-0.21575	0.019813	0.046164
11761127_CEACAM3	-0.19827	0.019826	0.046192
11745556_C11orf74	-0.10024	0.019833	0.046205
11760627_SDHAP3	0.294813	0.01984	0.046219
11722158_SLC7A8	-0.16384	0.019855	0.046253
11748222_TRIM27	0.135002	0.019859	0.046258
11757551_MRPL3	-0.11889	0.01987	0.046283
11716305_ATXN10	-0.19389	0.019879	0.046301
11757638_CD93	-0.17153	0.01988	0.046301
11759443_SLC19A1	-0.16224	0.019895	0.046334
11749058_TSPAN32	-0.14884	0.019897	0.046338
11752114_ABCC11	-0.1837	0.019899	0.046339
11724472_SART1	0.123599	0.019911	0.046364
11737062_MTMR3	-0.15638	0.019913	0.046368
11752903_CYP24A1	-0.20125	0.019923	0.046388
11734172_HSD3B7	-0.17552	0.019925	0.04639
11757038_H3F3B	0.101789	0.019929	0.046397
11722010_AZI2	0.126351	0.019936	0.046411
11718749_C1QL1	-0.23252	0.019937	0.046412
11762626_OR9H1P	-0.30093	0.019943	0.046424
11760169_SNRPB	-0.1863	0.019947	0.046432
11757073_THAP4	-0.13182	0.019951	0.046438
11764008_LOC102724	-0.14069	0.019955	0.046446
11749310_PRUNE	0.12281	0.01997	0.046479
11750731_EOMES	-0.23371	0.019971	0.046479
11749432_LILRB1	0.197806	0.019979	0.046494
11729381_AK5	-0.21836	0.019982	0.046497
11758121_RBM33	-0.13062	0.019982	0.046497
11729904_AGFG2	-0.15453	0.019983	0.046497
11721779_ACBD5	0.103223	0.019995	0.046523
11750851_SLC6A6	0.306616	0.019996	0.046523
11755095_CFAP20	-0.11153	0.02	0.04653
11755821_HDLBP	-0.10825	0.020003	0.046535
11745510_CTBP1-AS2	-0.16731	0.020005	0.046537
11723327_CA9	-0.12334	0.020009	0.046544
11728912_CDK5R2	-0.1399	0.020011	0.046547

11724657_RBP3	-0.10392	0.020012	0.046547
11717675_SLC39A3	-0.13653	0.020019	0.04656
11748302_GUSBP9 //	0.110213	0.02002	0.046561
11759242_FCN2	-0.18825	0.020022	0.046561
11733558_PIP5KL1	-0.22802	0.020023	0.046561
11737655_PIP5KL1	-0.22802	0.020023	0.046561
11723788_FAM167A	0.135825	0.020028	0.046571
11762953_NARFL	-0.14116	0.020033	0.046581
11722743_TLE2	-0.15961	0.020038	0.046589
11750611_ERBB2	-0.1102	0.020041	0.046596
11745710_CD274	0.168405	0.020043	0.046598
11722775_DIDO1	0.124945	0.020044	0.046598
11734928_BPNT1	-0.13742	0.020046	0.046598
11735422_MYOG	-0.19022	0.020047	0.046598
11740353_EFTUD2	-0.22818	0.020047	0.046598
11738835_NDRG2	-0.17889	0.020057	0.046616
11761709_WDR13	-0.15973	0.020059	0.046616
11720613_ELP3	0.145318	0.020059	0.046616
11728931_FXYD7	-0.19254	0.02006	0.046616
11757944_MIEF1	-0.13374	0.02006	0.046616
11753253_GHRHR	-0.20838	0.020061	0.046616
11745745_SLC39A1	-0.18076	0.020072	0.046639
11726309_GLRB	0.251034	0.020076	0.046647
11718265_SMAD3	-0.11825	0.020079	0.046652
11748887_RING1	-0.15093	0.020081	0.046653
11751419_TMEM185A	-0.15537	0.020103	0.046704
11717258_PIM1	-0.27589	0.020118	0.046737
11741504_NREP	0.127079	0.02012	0.046738
11746024_LOC100293	0.204363	0.020129	0.046757
11715314_RABGEF1	-0.17362	0.020134	0.046766
11730639_GCM2	-0.13133	0.020138	0.046774
11724339_SLC35F1	-0.15473	0.02014	0.046776
11715208_CLCN6	-0.19299	0.020143	0.04678
11739685_EXOC6B	0.416816	0.020156	0.046808
11757101_SPEF2	-0.1806	0.020166	0.046829
11725959_CALM2 ///	-0.1928	0.020167	0.04683
11723018_LCMT2	-0.11332	0.020169	0.046833
11733935_GEMIN4	-0.1716	0.020171	0.046833
11758104_RAB2B	-0.13778	0.020172	0.046833
11724523_CDK5RAP2	-0.15891	0.020173	0.046835
11733675_WDR49	-0.28086	0.020181	0.046852
11735894_BCL2L11	-0.19427	0.020184	0.046856
11726352_EFNA5	0.139417	0.020205	0.0469
11723904_UNC80	0.174124	0.020205	0.0469
11748372_C16orf62	0.131784	0.020222	0.046938
11750686_KIF3C	-0.13347	0.020227	0.046946
11724705_ST8SIA3	-0.23082	0.020255	0.047009
11730016_NEUROD2	-0.23123	0.020257	0.047011
11721184_TSPAN33	-0.19914	0.020267	0.047029
11757068_FAAP20	0.150145	0.020268	0.047029
11727829_SNAP29	-0.15219	0.020268	0.047029

11718452_AXIN2	-0.28874	0.020268	0.047029
11741685_TAS1R1	-0.17341	0.020293	0.047085
11756538_JMJD7 ///	-0.19164	0.020296	0.047089
11761061_UQCRC1	-0.10972	0.020311	0.047122
11740754_LAIR2	-0.13927	0.020313	0.047123
11764175_LOC105371	-0.14253	0.020315	0.047127
11717488_SEPT5 ///	-0.19361	0.020316	0.047127
11736290_PTPRG	0.242304	0.02032	0.047133
11760348_CRYAB	-0.17369	0.020322	0.047135
11725171_PPARD	-0.16812	0.020322	0.047135
11753800_SMIM11A /	0.14969	0.020326	0.04714
11722935_CHML	-0.19863	0.02033	0.047147
11758098_TPT1	0.096535	0.020335	0.047157
11760794_STMN4	-0.16067	0.020354	0.047198
11728848_RAB39B	-0.20305	0.02036	0.047209
11741002_RNF123	0.163798	0.020361	0.047209
11762972_WDR34	-0.12067	0.020361	0.047209
11739587_RERE	0.127062	0.020363	0.047211
11743986_AKAP1	-0.14939	0.020365	0.047212
11744050_MAP1B	0.103338	0.020366	0.047214
11736808_VAV2	0.140205	0.020369	0.047217
11761404_RING1	-0.13464	0.02037	0.047217
11761779_MPZL3	-0.1591	0.020377	0.047233
11761450_SRRM2	-0.15335	0.020384	0.047245
11718193_VEZF1	0.142496	0.020385	0.047247
11763874_LOC100128	-0.19655	0.020387	0.047249
11749111_MTTP	-0.20396	0.020394	0.047262
11730166_C11orf16	-0.16865	0.020395	0.047263
11725510_SLC7A6	0.106088	0.020403	0.047278
11720337_C19orf43	0.105304	0.020404	0.047278
11751608_TTC33	-0.1703	0.020411	0.047291
11760706_CDK11A	0.176178	0.020411	0.047291
11740405_LHX4	0.109967	0.02042	0.047309
11743718_ZIK1	0.123146	0.020421	0.047309
11756926_TMEM63B	0.151795	0.020422	0.047309
11734939_SLC16A9	0.1815	0.020427	0.047319
11725223_SNTB1	-0.13391	0.020435	0.047334
11732420_TYW5	-0.15678	0.020438	0.047341
11734924_ATP6V1G2	-0.18749	0.020448	0.047361
11752077_AP2A1	-0.12022	0.020451	0.047366
11717181_OCRL	0.170998	0.020452	0.047366
11715814_TRAP1	-0.13735	0.020466	0.047396
11752666_B3GNT5	0.158576	0.020469	0.047401
11762359_SPRY4	0.288686	0.020471	0.047403
11719443_PTPRZ1	0.314445	0.020479	0.04742
11761850_KMT5C	-0.13502	0.020493	0.04745
11750498_SLC1A4	-0.15597	0.020527	0.047526
11741832_SNRPN ///	-0.12049	0.020538	0.047548
11739329_WDR82	-0.10989	0.020541	0.047554
11729322_RASD2	-0.1427	0.020542	0.047554
11719591_GLTP	0.108697	0.020549	0.047569

11739043_LIMK2	0.188987	0.02055	0.047569
11742419_MYLK3	0.185596	0.020556	0.047578
11727046_HPDL	-0.26913	0.020556	0.047578
11752709_ZNF215	0.250734	0.020569	0.047604
11730212_MRPS7	0.126361	0.02057	0.047606
11751854_GSG1L	-0.13504	0.020572	0.047606
11725024_LOC100129	-0.16527	0.02058	0.047622
11721130_HCFC1	-0.15172	0.020589	0.047643
11719132_FAM49B	0.123873	0.020597	0.047658
11761223_USP15	0.25869	0.020618	0.047706
11720861_ARHGEF3	-0.11388	0.02064	0.047751
11734134_NANOS1	0.301208	0.02064	0.047751
11736927_PCDHA12	0.246763	0.020644	0.047759
11724102_PDZRN3	-0.12561	0.020649	0.047767
11715734_NIPSNAP1	-0.10971	0.020654	0.047777
11717618_PXN	-0.10209	0.02066	0.047788
11729475_GGN	-0.16888	0.020678	0.047827
11723873_TMEM104	-0.13518	0.020682	0.047835
11733992_TMEM150C	-0.10179	0.020688	0.047846
11752803_PCSK9	-0.15619	0.020692	0.047854
11749116_DNM2	-0.11097	0.020697	0.047863
11743575_CCDC180 /	-0.10676	0.020718	0.047909
11759094_GNAQ	0.111929	0.020723	0.047918
11715673_JUNB	0.139594	0.020727	0.047926
11744915_NEGR1	-0.18332	0.020742	0.047957
11755793_WDR27	0.10981	0.020765	0.048008
11721794_CR2	-0.16757	0.020772	0.048022
11752009_TNC	0.17423	0.020775	0.048028
11762380_TRO	-0.19313	0.020779	0.048034
11731091_CACHD1	0.132119	0.020784	0.048043
11735348_ZFP69	0.131411	0.020785	0.048043
11725308_CALU	0.108118	0.020786	0.048043
11754721_TXNDC15	0.154815	0.020793	0.048057
11749343_ABHD4	-0.14493	0.020794	0.048057
11740773_LIN54	-0.23941	0.020797	0.048061
11720845_CD248	0.151291	0.020799	0.048065
11742132_MFSD2B	-0.11487	0.020805	0.048075
11758831_POLR2C	-0.422	0.020807	0.048078
11740693_FAM83C	-0.21304	0.020808	0.048078
11727240_C4orf19	0.243891	0.020819	0.048102
11734180_PTGFR	-0.15911	0.020823	0.048109
11734504_ZNF177 //	0.26422	0.020824	0.048109
11758047_MFSD14A	-0.10152	0.02083	0.048119
11760673_LYRM4	-0.19666	0.020834	0.048126
11732688_UBXN2A	0.115502	0.020835	0.048126
11744194_ABCC3	0.167643	0.020839	0.048134
11742603_OR10K2	-0.19624	0.020842	0.048139
11760769_SNRPB	-0.17652	0.020851	0.048158
11759027_AHSA2	0.201514	0.020854	0.048161
11724413_PPARA	-0.14693	0.020857	0.048166
11752948_PRCP	0.114382	0.020875	0.048204

11732502_ICA1L	-0.18789	0.020875	0.048204
11747256_TTC39C	0.246405	0.02088	0.048213
11734473_EXD1	-0.15608	0.02089	0.048234
11719955_POLR3H	0.182601	0.020891	0.048234
11743892_ZNF780A	0.154415	0.020901	0.048255
11741007_PARG	-0.12851	0.020942	0.048347
11746756_KY	-0.15316	0.020976	0.048423
11762363_NEUROD2	-0.26971	0.020986	0.048443
11746785_SLC35E1	0.18646	0.02099	0.04845
11717089_RBM39	0.112393	0.020991	0.04845
11745673_CEP290	-0.27367	0.021002	0.048473
11742429_ZNF716	0.222657	0.021003	0.048473
11729160_UCP2	-0.1709	0.021005	0.048475
11749736_RBBP5	0.185095	0.02101	0.048485
11731455_TPM3	0.179573	0.021029	0.048527
11752874_B4GALNT1	-0.27438	0.021033	0.048533
11752241_JPH4	-0.14814	0.021043	0.048555
11726314_TBL1X	-0.13787	0.021044	0.048555
11724995_KIAA1804	-0.21663	0.02105	0.048567
11748193_MAEA	0.112218	0.021058	0.048582
11722519_HKDC1	-0.10098	0.021067	0.048601
11763103_FCN2	-0.17969	0.021068	0.048601
11730477_RNF125	-0.23918	0.02107	0.048604
11753270_HABP2	-0.19166	0.021077	0.048618
11721201_TRIM62	-0.15989	0.021111	0.048694
11745986_SALL2	-0.28465	0.021113	0.048695
11760966_SLC25A14	-0.17047	0.021131	0.048736
11735438_NRXN2	-0.10342	0.021133	0.048737
11743704_ABHD11	-0.12434	0.021138	0.048747
11727754_PLA2G4A	0.138809	0.021149	0.048768
11737582_SGCD	0.10098	0.021156	0.048783
11746637_MARS	-0.10066	0.02116	0.048788
11737619_RFPL4B	-0.14703	0.02116	0.048788
11759974_TMEM75	-0.15866	0.021168	0.048804
11755082_ZNF836	-0.24027	0.021169	0.048805
11750758_ABCC6 ///	-0.15257	0.021176	0.048817
11741452_NDUFAF3	-0.10322	0.021187	0.04884
11718757_HIF1A	-0.10806	0.02119	0.048845
11745757_DCLK1	0.170502	0.021194	0.048853
11744564_PPWD1	-0.11681	0.021196	0.048856
11746249_PIFO	-0.20961	0.021199	0.048859
11732298_KIAA1715	0.113959	0.021205	0.04887
11722558_MPP1	-0.21614	0.02121	0.04888
11753194_LINC00893	-0.15926	0.021215	0.048888
11726669_PPIL4	-0.17592	0.021222	0.048903
11748977_DMKN	-0.13482	0.021227	0.04891
11734259_SCP2	0.160083	0.021227	0.04891
11753933_PHAX	0.159689	0.021228	0.04891
11743313_FARP2	-0.17523	0.02124	0.048934
11754563_CCBE1	0.38066	0.021247	0.048946
11762988_YME1L1	-0.22609	0.021247	0.048946

11723547_PEX1	0.108471	0.021248	0.048946
11752250_RSPO1	-0.12779	0.021249	0.048948
11746105_PLEKHM1 /	0.121159	0.021252	0.048948
11741139_MPPED2	-0.17983	0.021253	0.048948
11740082_AGMO	-0.13578	0.021253	0.048948
11761714_TFAP2A	-0.20122	0.021254	0.048948
11725989_MMP14	-0.11728	0.021257	0.048954
11720778_KLHL22	-0.18708	0.021266	0.048971
11722291_ZBTB43	0.142995	0.021268	0.048974
11757238_DISC2	-0.19216	0.02127	0.048975
11729616_UBE2J2	0.129993	0.021276	0.048987
11737766_TIGD1	-0.13729	0.021279	0.048989
11760329_RCBTB1	-0.10985	0.021279	0.048989
11746059_PSMD7	-0.15377	0.02128	0.048989
11730967_ACTL6B	-0.23882	0.021283	0.048994
11723817_ARHGAP29	-0.19382	0.021285	0.048997
11760479_SLC25A47	-0.12881	0.021287	0.048999
11729561_AHRR	0.123139	0.02129	0.049002
11759318_ISG20L2	0.124197	0.02129	0.049002
11718454_INPP5K	0.124787	0.021291	0.049002
11718861_HCK	0.179894	0.021295	0.049008
11744199_RETSAT	-0.1861	0.0213	0.049018
11754943_SLC25A35	-0.1315	0.021302	0.04902
11755285_BCAS3	-0.11569	0.021304	0.049024
11753989_PQBP1	0.121208	0.021315	0.049046
11746449_MEIS2	-0.13837	0.021319	0.049052
11755761_FANCM	-0.20979	0.021322	0.049056
11719355_SH3BP5	0.160149	0.021323	0.049056
11744734_SIN3B	0.122015	0.021328	0.049067
11719098_MVK	-0.1316	0.021329	0.049067
11760557_TTLL5	-0.21301	0.021332	0.04907
11729746_DNAJC17	-0.20207	0.021338	0.049083
11717593_CENPB	-0.12613	0.021341	0.049086
11723172_IL17RD	0.334942	0.021342	0.049086
11741184_AMPD3	0.169541	0.021344	0.04909
11746147_EIF2B1	0.099891	0.021346	0.049091
11747638_FARSB	-0.1378	0.021349	0.049094
11727780_SCARA5	0.157853	0.021349	0.049094
11718764_COX5B	-0.12347	0.021351	0.049095
11747516_EXOSC10	-0.14059	0.021352	0.049095
11735306_C9orf139	0.159163	0.021353	0.049095
11752196_ENG	0.107387	0.021353	0.049095
11732931_ERI2	-0.24524	0.021355	0.049095
11720553_ZXDC	-0.18199	0.021357	0.049099
11742230_SUPT20HL1	-0.12308	0.021363	0.04911
11718691_SBNO2	0.173435	0.021371	0.049125
11735358_VAX2	-0.25079	0.021372	0.049125
11760545_DACH2	-0.21595	0.021376	0.049129
11722940_SRGAP2	0.120763	0.021376	0.049129
11758142_RAB10	-0.30162	0.021376	0.049129
11740785_RGPD4	-0.20341	0.021394	0.049167

11728773_ZDHHC3	-0.10464	0.021401	0.049181
11748496_ZNF785	-0.15116	0.021421	0.049224
11730972_CYP4F12	-0.13724	0.021424	0.049229
11759197_RAB9B	-0.17965	0.021425	0.049229
11720522_NSDHL	-0.10408	0.02145	0.049285
11753071_TASP1	0.116419	0.021471	0.049331
11751146_KLHL14	-0.11624	0.021474	0.049334
11756176_EIF4A3	-0.11335	0.021483	0.049353
11746707_LINC00314	-0.21796	0.02149	0.049368
11745610_PDDC1	0.179488	0.021498	0.049383
11762700_OFD1	-0.11733	0.021503	0.049392
11725261_C20orf144	-0.11354	0.021508	0.049402
11731078_SLCO5A1	0.096966	0.021515	0.049416
11723291_NDUFA1	-0.09442	0.021525	0.049433
11748683_NXN	-0.16823	0.021526	0.049433
11746601_GGA3	0.106811	0.021526	0.049433
11737681_SLC26A10	0.147172	0.021528	0.049435
11732050_PRR20A //	-0.13547	0.02153	0.049439
11754863_VPS37C	0.103259	0.021534	0.049445
11750882_KCNV1	0.305164	0.021536	0.049447
11743637_DIS3L2	0.136477	0.021538	0.04945
11721366_TRIM47	0.100463	0.021543	0.049459
11740411_PRKD2	-0.11038	0.021553	0.04948
11734567_RASGRP2	-0.15877	0.021558	0.049487
11747841_ACVR1	-0.15384	0.02156	0.049489
11742717_TBRG1	0.116439	0.021562	0.049492
11727126_RAPGEFL1	-0.20398	0.021563	0.049492
11733246_DTNBP1	-0.17426	0.021564	0.049492
11738963_KRTAP12-3	-0.11155	0.021569	0.049501
11755229_ENGASE	-0.16195	0.021584	0.049535
11760921_ZNF451	0.221848	0.021586	0.049536
11756502_COA5	-0.11742	0.021594	0.049552
11727354_CALN1	-0.13709	0.021603	0.04957
11725474_MAP3K5	0.166667	0.021606	0.049574
11763555_SNX12	0.130067	0.021621	0.049606
11722137_GUCY1A3	-0.17953	0.021622	0.049606
11728923_ONECUT2	-0.16579	0.021622	0.049606
11743706_MGA	0.162811	0.021628	0.049616
11749025_PPP1R12C	-0.13988	0.021641	0.049645
11737787_SHISA5	-0.13155	0.021644	0.049648
11756550_YJEFN3	0.115459	0.02166	0.049684
11752738_RIF1	-0.12535	0.021674	0.049712
11736823_VN1R1	-0.16705	0.021678	0.049717
11722121_PC	-0.39233	0.021678	0.049717
11757055_SH3BP5L	-0.13576	0.021685	0.049731
11764261_TMPRSS11A	0.097594	0.021693	0.049748
11734992_ARFIP1	0.1666	0.021719	0.049803
11736233_PTER	-0.1689	0.021723	0.049812
11752832_CD55	0.10399	0.021725	0.049813
11758951_NEURL1	-0.28223	0.021738	0.04984
11742704_CHD3	0.10336	0.02174	0.049842

11745423_PRKAR1B	-0.29994	0.021741	0.049842
11756191_MAN1C1	-0.27453	0.021748	0.049856
11735729_ALG12	0.212649	0.021749	0.049856
11727738_AKAP7	-0.17279	0.021766	0.049893
11752483_STK32B	0.146545	0.021771	0.049901
11763970_PRRX2-AS1	-0.24934	0.021772	0.049901
11755791_SASS6	-0.18467	0.021798	0.049958
11763782_TTC5	-0.13377	0.021798	0.049958
11720488_HSPB6	-0.25368	0.021808	0.049975
11760988_LRRC41	-0.15122	0.021808	0.049975
11730492_LRRC4B	-0.21183	0.021814	0.049986
11730690_FAM111A	-0.10537	0.021817	0.049992
11759502_TRAF3	-0.11413	0.021831	0.050022
11728435_ATP5S	-0.1187	0.021835	0.050027
11738410_KRTAP8-1	-0.12922	0.021836	0.050028
11743607_COG2	-0.11135	0.021839	0.050032
11737963_PCDHGA1 /	-0.12793	0.02184	0.050032
11761166_ZNF283	0.188682	0.021851	0.050055
11750624_MAP3K13	0.341618	0.02186	0.050072
11755525_ATOH8	-0.13581	0.021883	0.050125
11755768_SPATA31D4	-0.1784	0.021889	0.050135
11746055_CARD8	0.253111	0.021897	0.05015
11761599_PMS2	-0.18729	0.021897	0.05015
11750633_OGDH	-0.11319	0.021905	0.050162
11757439_GDI2	0.110375	0.021905	0.050162
11729047_ZNF34	0.281212	0.021913	0.050179
11731543_SESTD1	-0.18778	0.021915	0.050179
11743279_WNK1	-0.10851	0.021915	0.050179
11752192_PTPRB	-0.25182	0.021919	0.050184
11731466_CTSC	-0.10391	0.021928	0.050204
11732736_CHSY3	-0.21341	0.021931	0.050208
11733043_SLC7A2	-0.15419	0.021951	0.05025
11720667_KAT2B	0.254095	0.021971	0.050295
11750585_SEPT9	0.103268	0.021995	0.050347
11750093_PRICKLE4	-0.14939	0.022	0.050356
11747544_TPCN1	-0.17577	0.022001	0.050356
11750946_RIN1	-0.13855	0.022003	0.050359
11750518_LINC01135	-0.267	0.022007	0.050365
11719556_PDK2	-0.16524	0.02201	0.050371
11722148_YTHDC2	0.134434	0.022019	0.050389
11738720_OR2T3 ///	-0.17146	0.022028	0.050406
11747998_SNX17	0.108972	0.022043	0.050435
11754729_LOC102724	-0.165	0.022044	0.050435
11737884_TMEM82	-0.14631	0.022044	0.050435
11723310_TNFRSF10D	-0.13074	0.022058	0.050464
11723573_MORC4	0.246722	0.022058	0.050464
11753310_NARF	-0.1355	0.022061	0.050468
11725425_RAB27A	-0.16238	0.022063	0.050468
11749087_FKTN	-0.16859	0.022063	0.050468
11758012_APOA1	-0.23433	0.022071	0.050484
11760218_DNAJC10	0.245237	0.022077	0.050495

11757172_SNORA47	-0.14526	0.022085	0.05051
11746736_CACNA2D3	0.179757	0.022086	0.05051
11754615_UFM1	0.115605	0.022087	0.050511
11738238_PURG	0.370205	0.022096	0.050529
11746488_B9D1	0.110463	0.022098	0.050532
11727421_CANX	-0.11789	0.022103	0.05054
11759237_CLEC7A	0.154628	0.022105	0.050542
11741042_PSMA1	0.119802	0.022111	0.050553
11749516_PAF1	0.106984	0.022112	0.050554
11737586_ACOT11	-0.20896	0.022117	0.050562
11759017_SLC2A5	-0.18882	0.022118	0.050562
11748281_CNNM4	-0.28375	0.022123	0.050571
11732638_NOVA2	-0.11349	0.022125	0.050573
11732697_COL24A1	0.210465	0.022141	0.050608
11729404_DOC2A	-0.14266	0.022149	0.050624
11729733_ZNF281	0.102537	0.022161	0.050648
11748582_ART3	-0.11908	0.022162	0.050649
11738741_MYL10	-0.12709	0.02217	0.050664
11751103_TTTY10	-0.13999	0.022174	0.050672
11760473_TOR1AIP2	-0.14646	0.022181	0.050682
11719369_LACRT	-0.2307	0.022181	0.050682
11724092_SLC35E2 /	-0.10699	0.022182	0.050682
11735330_ZNF837	-0.1048	0.022191	0.0507
11717210_NEO1	0.16103	0.022193	0.050703
11752696_PGM2	0.120176	0.022196	0.050708
11740031_DUSP14	0.115066	0.022197	0.050708
200075_PMGUK1	0.125177	0.022202	0.050715
11761761_CD300LG	-0.15662	0.022228	0.050773
11748909_BNIP3L	0.104251	0.022232	0.050778
11753422_BNIP3L	0.104251	0.022232	0.050778
11745425_USP25	0.208438	0.022233	0.050778
11755614_FERMT1	-0.14401	0.022239	0.050788
11732031_OTOS	-0.14713	0.022244	0.050798
11717006_POLDIP3	-0.11659	0.022247	0.0508
11724538_ABCG2	-0.20307	0.022248	0.0508
11748711_EIF4G3	0.119363	0.022248	0.0508
11744404_TXNDC11	-0.16734	0.022261	0.050828
11745493_TELO2	-0.19772	0.022275	0.050856
11751773_SCRN1	0.118108	0.022277	0.050859
11756482_STT3B	-0.11142	0.022292	0.050888
11723736_RNF216	0.128947	0.022293	0.050888
11750436_IRX5	-0.17532	0.022293	0.050888
11744410_STMN4	-0.15363	0.022306	0.050914
11722812_RAB7A	-0.10628	0.022306	0.050914
11758783_COX6C	-0.18228	0.022311	0.050922
11739419_AMPD3	0.220154	0.022317	0.050935
11752149_UBASH3A	-0.24344	0.022319	0.050935
11740933_FOXA2	-0.14679	0.02233	0.050959
11736790_FBXO4	-0.13155	0.022335	0.050967
11718890_FOXO3	0.114823	0.022338	0.050972
11735059_WISP2	-0.26175	0.022339	0.050972

11761003_CBWD1 ///	0.247791	0.022355	0.051006
11753512_DNAJB6	0.099085	0.022364	0.051025
11744234_TJAP1	0.110561	0.022367	0.051028
11735703_MT1A ///	-0.13656	0.02237	0.051033
11729981_PARP11	-0.17969	0.022382	0.051059
AFFX-HUMGGAPDH	-0.09433	0.022384	0.05106
11750193_NFATC4	-0.12729	0.022386	0.051063
11748871_ACSM5	0.1546	0.022392	0.051074
11751353_RRAGC	0.114221	0.022396	0.05108
11739323_WDR4	0.217938	0.022397	0.05108
11736135_OAS2	-0.18218	0.022402	0.05109
11743280_WNK1	0.113386	0.022404	0.051092
11725280_PRDM8	-0.13928	0.022406	0.051093
11725254_FREM1	0.174445	0.022417	0.051116
11715962_ACTR10	-0.15976	0.022435	0.051157
11729701_STXBP1	-0.11241	0.022437	0.051157
11754693_AHI1	0.246412	0.022438	0.051157
11736806_GABRA4	-0.15841	0.02244	0.051159
11732014_ZNF821	0.226225	0.022447	0.05117
11727584_SPIN3	-0.21433	0.022447	0.05117
11760499_ZNF451	0.184823	0.022455	0.051186
11721922_PRKCB	-0.16021	0.022467	0.051211
11742041_ATXN2L	-0.10879	0.022478	0.051235
11724791_PKP3	-0.20771	0.022497	0.051276
11749874_OXCT1	0.13722	0.022515	0.051314
11751455_REEP1	-0.17933	0.022516	0.051315
11726830_ANTXR1	0.148613	0.022538	0.051358
11751218_CA10	-0.20611	0.022538	0.051358
11718090_TPCN1	-0.10805	0.022553	0.051389
11745295_ANKRD66	-0.16995	0.022553	0.051389
11742201_APAF1	-0.19419	0.022564	0.05141
11720737_ZNF559	0.118655	0.022564	0.05141
11746858_LPA	-0.1339	0.022582	0.051448
11752945_SLC36A1	-0.1126	0.022586	0.051454
11759941_UHRF1	-0.14994	0.02259	0.051462
11725820_PAQR3	0.100835	0.022592	0.051464
11717794_ZDHHC3	-0.17138	0.022599	0.051476
11760697_CCM2	-0.19173	0.0226	0.051477
11723347_MTERF3	-0.09494	0.022612	0.051501
11743916_NPC1	0.167709	0.022626	0.05153
11720294_PLPP2	-0.13661	0.022638	0.051555
11740546_ADAM22	0.344061	0.022649	0.051572
11722944_PALB2	-0.1484	0.022649	0.051572
11756892_C2CD2	0.223802	0.022649	0.051572
11720696_HDAC7	-0.10895	0.022649	0.051572
11743291_GALNT1	-0.13356	0.022655	0.051581
11741718_PDE10A	-0.28337	0.022656	0.051582
11715577_RAC1	-0.13292	0.022657	0.051582
11715910_MYO6	0.152279	0.022669	0.051607
11724177_TSPAN11	-0.19087	0.022674	0.051615
11715283_CCKAR	-0.13696	0.022681	0.051629

11725648_DCAF5	-0.14141	0.022682	0.051629
11720318_MROH7-TTC	0.115035	0.022695	0.051656
11731609_GPALPP1	-0.14478	0.022704	0.051674
11719715_FXR1	0.101966	0.022705	0.051674
11737216_SIGLECL1	-0.17395	0.022709	0.051681
11719444_PTPRZ1	0.286374	0.022725	0.051715
11730076_SHF	-0.11416	0.022735	0.051737
11731836_SLC35C2	0.102757	0.022744	0.051753
11738384_CLRN2	-0.14022	0.022748	0.05176
11732883_F7	-0.15079	0.022755	0.051773
11719725_PUM3	-0.10409	0.022761	0.051785
11764021_CAMTA1	-0.11138	0.022767	0.051797
11754482_PSMB1	-0.11181	0.022775	0.051813
11742365_OR51I1	-0.11455	0.022781	0.051823
11748916_PDE4DIP	0.273685	0.02279	0.051841
11750094_TOMM6	-0.11879	0.0228	0.051862
11745090_PLXDC2	-0.17642	0.022802	0.051863
11753734_WFDC2	-0.1214	0.022806	0.05187
11761072_NEURL3	-0.18974	0.02281	0.051877
11717979_C7	-0.12703	0.022815	0.051886
11757770_TOMM70A	0.093994	0.022823	0.051902
11759823_RPL37A	-0.26742	0.022824	0.051902
11741528_ACP1	-0.10011	0.022834	0.051922
11762521_CHRNB1	-0.22028	0.022835	0.051922
11760476_RPTOR	-0.20198	0.022837	0.051924
11756379_H3F3A ///	-0.10384	0.022843	0.051936
11720639_MRPS27	0.108251	0.02285	0.051949
11721100_SGSH	-0.17655	0.022867	0.051985
11758017_PPIA	-0.09707	0.02287	0.05199
11743689_PTCHD2	-0.141	0.022872	0.051992
11742182_POTEE ///	0.10809	0.022896	0.052044
11733604_SLC52A1	-0.16467	0.022907	0.052067
11751659_MAPRE3	0.327771	0.022917	0.052088
11745163_HADHB	-0.12085	0.022922	0.052096
11720146_DAPK1	0.187056	0.022923	0.052096
11749170_KRT6A	-0.16163	0.022927	0.052103
11763602_ZNF839	0.175642	0.022937	0.05212
11752825_ARHGEF6	0.157892	0.022937	0.05212
11754576_LEAP2	0.147719	0.022941	0.052128
11753210_HYAL1	-0.15636	0.022958	0.052162
11719553_C2CD2L	0.118403	0.022959	0.052162
11733205_AFAP1L1	-0.1027	0.02296	0.052162
11748855_CYB5R1	-0.1209	0.022962	0.052163
11725838_FAM163A	-0.15555	0.022962	0.052163
11734342_ZMYM1	-0.1048	0.022972	0.052182
11759948_HDAC8	0.133509	0.022973	0.052182
11748994_AMBRA1	-0.19938	0.022981	0.052198
11763416_KBTBD3	-0.15214	0.022986	0.052204
11736874_ZNF514	0.183601	0.022987	0.052204
11719112_PJA2	-0.11435	0.022987	0.052204
11727036_SLC9B2	0.108977	0.022988	0.052204

11745637_ADARB2	-0.11335	0.022989	0.052204
11730708_C9orf57	-0.2609	0.022997	0.05222
11737853_TNFSF18	0.233565	0.022998	0.05222
11744833_TSHZ2	-0.18069	0.023001	0.052225
11734324_PAQR7	-0.22076	0.023027	0.052279
11762450_KLF14	0.147727	0.023027	0.052279
11746052_CHD1L	0.129594	0.023044	0.052315
11753036_ANKS1B	0.17001	0.023045	0.052315
11716335_RBX1	-0.10105	0.023046	0.052315
11744471_CRP	-0.28294	0.023063	0.05235
11721109_NAP1L3	-0.39258	0.023067	0.052358
11731552_HAP1	-0.11691	0.023077	0.052377
11739486_SUZ12	-0.18146	0.023083	0.052389
11722726_ZNF286A /	-0.10597	0.023107	0.05244
11754197_FAM120B	0.106021	0.02311	0.052444
11751476_INPP5B	0.200805	0.023117	0.052458
11745415_DIAPH1	-0.10593	0.023126	0.052477
11754138_PSPH	-0.16748	0.02313	0.052484
11730491_LRRC4B	-0.11177	0.023136	0.052495
11751669_ADH6	-0.20819	0.023139	0.052498
11738419_ANGPT4	0.118971	0.023146	0.052512
11724452_PATZ1	0.10449	0.023155	0.05253
11760190_ZSCAN30	-0.12817	0.023162	0.052543
11759298_SCYL2	-0.13526	0.023173	0.052565
11736885_PAPOLB	-0.155	0.023176	0.052571
11715634_TMEM14C	-0.13669	0.023182	0.052581
11754216_ZBTB8OS	0.096492	0.023185	0.052585
11736212_MRPL57	-0.10717	0.023188	0.05259
11739300_CISD3	0.121141	0.023199	0.052613
11746202_NAB1	0.155102	0.0232	0.052614
11738663_TUBB7P	-0.19013	0.023213	0.052637
11719733_SENP3	-0.13881	0.023213	0.052637
11725099_ESF1	0.097463	0.023228	0.052668
11746696_DOCK8	0.146057	0.02323	0.052671
11747466_STK19	-0.15563	0.023236	0.052681
11731415_PSMD6	0.095419	0.023239	0.052685
11738400_RGMA	-0.18125	0.023256	0.052724
11719010_TMEM99	0.108402	0.023258	0.052726
11738226_GPR179	-0.16132	0.023261	0.05273
11735480_FAM160A1	0.140877	0.023272	0.052753
11736408_XYLB	0.228948	0.023276	0.052758
11747655_TM9SF4	0.143699	0.023278	0.05276
11736864_KIAA1958	-0.13338	0.023288	0.052779
11737640_ZNF556	0.168738	0.023289	0.052779
11731182_ACTL8	-0.16684	0.023303	0.05281
11749109_ADGRF5	-0.13728	0.023325	0.052856
11715161_KRTAP21-2	-0.15519	0.023332	0.05287
11759950_LCNL1	0.110947	0.023348	0.052905
11738345_VSTM4	-0.12938	0.023352	0.052911
11740483_TNR	-0.32923	0.023356	0.052918
11727089_DACH1	0.292958	0.023359	0.052921

11761218_CORO1B	0.098481	0.023374	0.052954
11725051_FAIM2	-0.12141	0.023376	0.052954
11758462_ZNF215	0.292157	0.023377	0.052954
11721281_CELA3A	-0.16415	0.023378	0.052955
11726055_B3GALT4	-0.22931	0.023387	0.052972
11716087_AKT1	-0.12614	0.023392	0.052982
11729435_ZBTB22	-0.21734	0.0234	0.052997
11761235_NFATC4	-0.17984	0.023402	0.052999
11752228_MAP3K12	-0.15748	0.023407	0.053008
11754593_TUBB3	-0.10977	0.023411	0.053011
11745862_TCF12	0.149228	0.023411	0.053011
11747607_P2RX7	-0.19397	0.023412	0.053011
11752426_CAMTA1	0.122137	0.023414	0.053011
11756346_FAM102B	0.273145	0.023414	0.053011
11755225_TTLL4	-0.19695	0.023417	0.053016
11715483_MCL1	0.110048	0.023454	0.053097
11743387_CCDC127	-0.14582	0.023455	0.053098
11762435_FGFR2	-0.33927	0.023459	0.053103
11718592_RTN4	-0.1678	0.023473	0.053131
11732322_FXN	0.118269	0.023473	0.053131
11759793_FAM71D	-0.13893	0.023476	0.053134
11750072_RTP1	-0.16682	0.023477	0.053134
11743723_GALC	0.151973	0.023482	0.053143
11745102_NOX4	0.118501	0.023491	0.053161
11742153_OR1B1	-0.18531	0.023498	0.053175
11717679_UNC13B	0.120058	0.023506	0.053189
11715865_ATP6V1F	0.11186	0.023507	0.053189
11758914_YY1	-0.26801	0.023507	0.053189
11752179_NBR1	0.120315	0.023528	0.053233
11723519_ELAC1	0.159055	0.023531	0.053237
11715596_PSMB7	-0.09321	0.023546	0.05327
11756643_SMAP1	0.171314	0.023548	0.053271
11716139_SMS	0.101716	0.02355	0.053272
11762596_CEP57L1	-0.2407	0.023551	0.053272
11751567_EGR3	0.17275	0.023554	0.053277
11759773_LOC102723	0.23174	0.023562	0.053292
11741131_NLGN4X	-0.11184	0.023567	0.053301
11760902_NAPSB	-0.14713	0.023583	0.053334
11728258_DIEXF	0.098796	0.023599	0.05337
11756559_SNUPN	-0.1172	0.023619	0.053411
11715921_SEPT9	0.099806	0.023621	0.053412
11739597_AGO4	-0.15177	0.023621	0.053412
11755363_WDR91	-0.20739	0.023636	0.053443
11756045_AQP1	-0.13219	0.023641	0.053451
11750332_ASZ1	0.219213	0.023643	0.053453
11756400_QDPR	-0.11115	0.02365	0.053466
11746230_ZMYM6	0.164061	0.023665	0.053497
11722446_SIRT3	0.112475	0.023683	0.053536
11737743_ARSF	-0.20274	0.023686	0.053541
11751872_EPGN	0.152406	0.023691	0.053549
11727887_ZNF25	0.112724	0.023694	0.053554

11724123_ZNF853	-0.19909	0.023707	0.053581
11754876_PCDH20	0.199971	0.023709	0.053581
11738784_PLA2G2E	-0.17181	0.02371	0.053581
11763955_SCARNA10	-0.18963	0.023711	0.053581
11717936_ERMP1	-0.13182	0.023711	0.053581
11756541_ABHD12	-0.12763	0.023715	0.053587
11726721_MPG	-0.1216	0.02372	0.053596
11763027_HFE	-0.1615	0.023722	0.053597
11740391_CTBP1-AS2	-0.13532	0.023729	0.05361
11741340_GAB1	-0.21253	0.023733	0.053617
11751731_PADI1	-0.20549	0.023745	0.053642
11728168_KAZN	-0.17968	0.023765	0.053684
11763098_TSL	0.168516	0.023766	0.053684
11747657_CDK10	-0.09905	0.023772	0.053695
11751752_HSPBAP1	-0.19879	0.023776	0.0537
11748341_TPP1	0.200754	0.023776	0.0537
11733900_EPN1	-0.09944	0.023791	0.05373
200001_PMCAPNS1	0.104649	0.023792	0.053732
11739513_TIMM50	-0.20746	0.023798	0.05374
11744397_ABCC5	0.10044	0.023798	0.05374
11751861_HYAL1	-0.13076	0.023813	0.053771
11761259_ACOT7	-0.26911	0.023817	0.053777
11751291_SFXN4	-0.12651	0.023825	0.053793
11761936_MLIP	-0.25861	0.023826	0.053793
11753797_TSG101	0.135988	0.023834	0.053806
11736142_ENPP4	0.203217	0.023834	0.053806
11748840_MTAP	-0.09731	0.023841	0.053819
11738857_ANTXR1	-0.19628	0.023844	0.053822
11717253_PRM1	-0.18015	0.023847	0.053826
11746184_DHDDS	-0.1649	0.023848	0.053826
11747844_PPIL2	-0.18088	0.023852	0.053833
11751504_QTRTD1	0.129368	0.023854	0.053836
11715576_RAC1	-0.1699	0.023856	0.053838
11749962_BCAS1	-0.19657	0.023862	0.053849
11740510_SSPN	-0.24096	0.023865	0.053853
11748788_CCNJL	0.113469	0.023868	0.053856
11761551_DHRS3	-0.15867	0.02387	0.053859
11718167_PRDM4	0.101663	0.023887	0.053896
11732626_NEMP2	-0.24987	0.023891	0.053903
11749633_CLOCK	0.230191	0.023901	0.053923
11745938_AIM1	0.147599	0.023924	0.053972
11730530_ZNF684	0.118991	0.023929	0.05398
11730168_LITAF	0.098525	0.023939	0.054001
11742364_OR5AR1	-0.17158	0.023941	0.054003
11744808_TRPM8	0.256609	0.023948	0.054016
11730759_CRIPT	0.104034	0.023954	0.054028
11758069_C1orf186	-0.18322	0.023958	0.054032
11720501_UBE4B	0.143499	0.023962	0.05404
11756079_RHBDD1	-0.11484	0.023971	0.054058
11738555_TAS2R40	-0.11151	0.023973	0.05406
11763488_CLASP2	0.159066	0.023974	0.05406

11754832_PPP1R12C	-0.15088	0.023977	0.054062
11724477_FOSL1	-0.1181	0.024004	0.054121
11743183_SLC19A1	-0.13129	0.024012	0.054138
11750450_UBAC2	-0.11681	0.024016	0.054145
11722066_C12orf49	0.124942	0.024018	0.054147
11722660_NFE2L3	0.117278	0.024022	0.054152
11739260_YIPF6	0.116137	0.024034	0.054177
11726737_PDLIM7	-0.17115	0.024039	0.054186
11738561_OR2D3	-0.18709	0.02405	0.054209
11728556_KCNF1	-0.21312	0.024057	0.054219
11723582_MTHFSD	-0.1085	0.024058	0.054219
11743949_FRAT1	-0.2149	0.024058	0.054219
11759402_VCAN	0.194231	0.024066	0.054235
11740383_RBL1	-0.24705	0.024071	0.054243
11743731_TNFSF10	-0.23722	0.024074	0.054248
11735687_C20orf173	-0.19429	0.024077	0.05425
11747819_THEM6	-0.16808	0.024079	0.054254
11735861_FAM9A	-0.16863	0.024088	0.054271
11728355_BBS10	0.103241	0.024094	0.054282
11750226_ADCK1	-0.13983	0.024099	0.05429
11758667_YWHAZ	-0.10471	0.024103	0.054298
11729331_MAB21L2	-0.20875	0.024111	0.054314
11753222_MAGT1	0.14988	0.024113	0.054314
11738933_CDX4	-0.15728	0.024121	0.054332
11749801_BAIAP2	-0.12744	0.024131	0.054351
11720479_BCCIP	-0.16329	0.024134	0.054353
11751334_BPNT1	-0.12701	0.024134	0.054353
11735468_MC4R	-0.15291	0.024136	0.054355
11757787_FTL	0.096239	0.024138	0.054356
11744254_COPB2	0.110023	0.024146	0.054371
11759383_KRTAP20-2	-0.18317	0.024147	0.054371
11720875_CORO2B	0.13711	0.024151	0.054377
11720300_SLA	-0.09694	0.024181	0.054443
11715594_RBM38	-0.161	0.024188	0.054453
11744562_EVA1B	0.144493	0.024188	0.054453
11729137_UGT2B7	0.149221	0.024189	0.054453
11751175_SORCS3	-0.27561	0.024193	0.054461
11749055_MRVI1	-0.20343	0.024197	0.054467
11719178_PVR	-0.11911	0.024212	0.054498
11721417_SCNN1B	-0.22141	0.024214	0.054499
11743450_ELOVL6	0.169128	0.024217	0.054504
11738793_DEFB124	-0.12724	0.02422	0.054509
11743517_NNT	-0.09752	0.024229	0.054526
11728637_ATP5A1	-0.09234	0.024233	0.054531
11756278_SAGE1	0.155472	0.024234	0.054531
11736666_PTER	-0.14838	0.024235	0.054532
11715565_PSMD2	-0.11369	0.024246	0.054554
11737466_RUSC1-AS1	-0.13124	0.024267	0.0546
11723953_CLINT1	0.099903	0.02427	0.054604
11746602_GGA3	-0.10381	0.024279	0.054621
11717151_APMAP	-0.16384	0.024285	0.054631

11756525_IDI2-AS1	0.152021	0.024286	0.054631
11750402_ZNF606	0.281791	0.024287	0.054631
11715760_RPL36AL	0.097824	0.024293	0.054641
11747622_APLP1	-0.13478	0.0243	0.054655
11724916_TMTC3	-0.15842	0.024309	0.054674
11752217_PRDM4	0.107817	0.024318	0.054692
11754170_TBRG1	0.134887	0.024325	0.054702
11717956_STK39	0.231419	0.024325	0.054702
11749558_CYP4F8	-0.11355	0.024331	0.054712
11750376_KCNMA1	-0.18082	0.024355	0.054764
11725247_ZSCAN25	-0.2113	0.024384	0.054828
11749129_C10orf90	0.13956	0.024397	0.054853
11738884_CASP1	0.393523	0.0244	0.054858
11764231_LOC105378	-0.24462	0.024414	0.054886
11729953_STARD5	-0.15633	0.02442	0.054898
11734113_ADAMTS19	0.284116	0.024422	0.054899
11720283_JMY	0.094091	0.02444	0.054938
11760223_MBD4	-0.13583	0.024444	0.054944
11754745_CEACAM21	-0.12956	0.024452	0.054957
11734192_CTAG2	-0.23599	0.024452	0.054957
11752644_TIAL1	0.099731	0.024458	0.054966
11753033_GPR85	0.347964	0.024458	0.054966
11738501_LY6G6F	-0.19754	0.024461	0.054969
11728193_PSMB2	-0.09708	0.024463	0.05497
11729257_IPPK	-0.15769	0.024463	0.05497
11730825_COX19	0.216615	0.024465	0.054972
11736358_LGI2	-0.13437	0.02448	0.055001
11726767_SLC39A3	-0.17448	0.024483	0.055008
11717588_CLPP	-0.09438	0.024489	0.055018
11730005_SEMA4D	-0.13158	0.024492	0.055021
11745931_TMEM150A	-0.16641	0.024493	0.055022
11761844_TTLL9	-0.16342	0.024496	0.055026
11761904_MCF2L2	-0.18157	0.024501	0.055034
11733539_MROH1	0.196925	0.024503	0.055037
11743224_IQSEC3	-0.20702	0.02451	0.05505
11757087_LOC101060	-0.13837	0.024512	0.055051
11732727_SLITRK4	-0.10952	0.024518	0.055063
11738263_AUTS2	-0.2036	0.024527	0.05508
11747901_LOC102724	0.135412	0.02453	0.055083
11747566_UBXN11	0.170717	0.024532	0.055085
11721702_CMTM7	-0.10556	0.024535	0.05509
11763191_PRICKLE3	-0.09882	0.024536	0.05509
11732886_VENTX	-0.14418	0.024544	0.055106
11741170_MC2R	-0.1829	0.024549	0.055114
11734566_FAM188B /	0.106843	0.024553	0.055121
11734064_AMPD3	0.237903	0.024554	0.055121
11716901_TGOLN2	0.159227	0.024565	0.055143
11735745_SERPINA10	0.216855	0.024567	0.055144
11762439_ADRA1A	-0.12551	0.024568	0.055144
11763826_NDUFAF1	-0.11994	0.024581	0.05517
11724676_KLHL2	0.178143	0.024584	0.055175

11735725_TP53BP1	0.124598	0.024594	0.055195
11722128_FAM102B	-0.15842	0.024595	0.055195
11716863_NISCH	0.112747	0.024599	0.055202
11760345_HES1	-0.16267	0.024606	0.055214
11758298_KRT8	0.118505	0.024624	0.055251
11716722_PHF1	-0.11383	0.024627	0.055256
11738902_KCNK18	-0.15048	0.024636	0.055274
11752532_PIKFYVE	0.284804	0.024638	0.055275
11744880_ZNF165	0.407498	0.02464	0.055278
11733384_TMEM182	-0.14092	0.02465	0.055298
11721611_IPO11	-0.12537	0.02467	0.05534
11760924_RBM8A	-0.11136	0.024674	0.055348
11749969_TSPAN5	0.120852	0.024677	0.055352
11723887_TRMU	0.145765	0.024687	0.055371
11748005_FRY	-0.23278	0.024692	0.055379
11743066_PTDSS1	0.11714	0.024694	0.055382
11728994_AZI2	-0.10817	0.024705	0.055403
11728430_HEY2	-0.15618	0.024716	0.055426
11744991_ACACB	-0.26679	0.024724	0.055441
11742243_ATF3	0.110295	0.024725	0.055441
11731932_KANK1	0.116677	0.024744	0.05548
11749902_PCNP	-0.10683	0.02475	0.055491
11724642_TMEM88	0.270689	0.024751	0.055491
11727630_APBA2	-0.14064	0.024757	0.055501
11759379_OR5L1	-0.17196	0.024772	0.055533
11742656_MSGN1	-0.17958	0.024773	0.055533
11727807_FECH	-0.11833	0.024776	0.055537
11750145_TOP3B	0.095477	0.024786	0.055557
11720540_ANAPC2	-0.10281	0.024792	0.055568
11762892_TMCO1	0.149278	0.0248	0.055584
11758055_RGPD5 ///	-0.10894	0.024806	0.055595
11740552_ASPG	-0.17546	0.024808	0.055597
11751747_ERG	-0.27522	0.024852	0.055693
11761224_KRT16P1	-0.17013	0.024857	0.0557
11723911_GAB3	0.194173	0.024864	0.055715
11744129_MYO9B	-0.11122	0.024866	0.055715
11753075_TNFRSF10B	0.119372	0.024872	0.055726
11727183_ZNF175	0.273718	0.024873	0.055726
11720877_ABTB2	-0.11429	0.024874	0.055726
11758725_H1FX	-0.20909	0.024875	0.055727
11752815_SARAF	0.112838	0.024883	0.055742
11725010_SCTR	-0.21021	0.024884	0.055742
11731014_BBS5	0.168723	0.024906	0.055787
11752773_PAX4	-0.18823	0.024924	0.055825
11748561_DAAM2	-0.17393	0.024925	0.055825
11757780_RPS4X	-0.10102	0.024927	0.055825
11738992_ATP6V0E2	-0.21231	0.024927	0.055825
11754826_EEF1D	0.104415	0.024938	0.055847
11746318_PCDHB18P	0.209738	0.024945	0.055861
11744268_GIPC1	0.097988	0.024954	0.055877
11757572_UBAC2	-0.10922	0.02496	0.055888

11722271_MSANTD4	0.212451	0.024979	0.055927
11741929_HTR3C	-0.22216	0.024982	0.055933
11725570_RAB7B	-0.10967	0.024988	0.055942
11746475_MFSD4	-0.21291	0.025022	0.056018
11758657_PKIG	0.092706	0.025039	0.056052
11729013_ACPP	0.190431	0.025041	0.056053
11761206_FAM153B /	0.175841	0.025044	0.056059
11730307_RIC3	-0.28024	0.025046	0.056059
11737387_S100PBP	-0.20071	0.025047	0.056059
11753048_VPS53	-0.10741	0.02506	0.056087
11734961_SYNJ2	0.111854	0.025064	0.056091
11760247_ZNF226	0.171854	0.025064	0.056091
11757920_ZYG11B	-0.11507	0.025068	0.056095
11731736_PGAP1	-0.17983	0.025069	0.056095
11750795_KLHL1	-0.12957	0.025076	0.056109
11739767_PTPRE	0.19441	0.025093	0.056142
11728295_PKIG	0.095475	0.025094	0.056142
11730662_RASL10A	0.124496	0.025094	0.056142
11729365_PRKAA2	0.18215	0.025109	0.056173
11746300_CFLAR	0.156944	0.025111	0.056174
11745189_APOL1	-0.1249	0.025139	0.056234
11743913_SPOP	0.115518	0.025141	0.056237
11723855_CASKIN2	-0.11529	0.025154	0.056263
11716162_LBH	-0.15246	0.025159	0.056272
11760700_LSAMP	-0.13768	0.025161	0.056273
11761668_DMPK	-0.13932	0.02517	0.056292
11736870_C10orf12	0.181725	0.025173	0.056296
11720842_RHOD	-0.17357	0.025176	0.056299
11750290_ALDOB	0.120685	0.025178	0.056302
11731406_YIPF5	0.113709	0.025185	0.056313
11722873_RTKN	-0.11896	0.025192	0.056328
11736403_GABRA3	0.221451	0.025212	0.05637
11722459_ZNF768	-0.26474	0.025214	0.056372
11740657_SLC22A5	0.252073	0.02522	0.056382
11729183_RPAP3	0.101004	0.025221	0.056382
11733885_TRIM25	-0.146	0.025225	0.056388
11738695_FANCD2OS	-0.16433	0.025236	0.05641
11734982_GPRASP1	0.146518	0.025251	0.05644
11760320_RPL31	-0.12391	0.025251	0.05644
11763476_LOC100506	-0.17433	0.025255	0.056444
11760851_ARID5A	-0.195	0.025258	0.05645
11736880_FCRL1	-0.14834	0.025269	0.056471
11727217_CETP	0.115688	0.025273	0.056477
11755112_HNRNPUL1	-0.11289	0.025294	0.056522
11754986_WDR25	0.105864	0.025296	0.056524
11724627_MAT1A	-0.15331	0.025303	0.056535
11735752_CXXC5	-0.11826	0.025303	0.056535
11736101_MAN1A1	0.151536	0.025309	0.056545
11761412_ZNF232	-0.26135	0.025318	0.056564
11750561_LYST	0.162561	0.025321	0.056568
11757384_UROD	-0.09807	0.025329	0.056583

11738945_MRAP	-0.16807	0.025335	0.056592
11730275_CALCRL	0.15582	0.025336	0.056593
11741201_NAT10	0.117159	0.025338	0.056593
11760459_EPS8L2	-0.11492	0.025349	0.056617
11760607_AGR2	0.275878	0.025367	0.056653
11719930_ARGLU1	0.108911	0.025382	0.056686
11719477_FASTKD5	-0.09885	0.025386	0.056691
11730565_PNMT	-0.11519	0.025399	0.056718
11762291_AGPAT4	-0.12922	0.025406	0.056731
11741888_LINC00303	-0.12412	0.02541	0.056737
11717660_PPP1R16B	-0.1511	0.025438	0.056797
11755754_ATRX	0.098784	0.02545	0.056821
11758365_TTLL8	0.220355	0.025452	0.056823
11722767_MBNL2	0.110495	0.025457	0.056832
11719326_DMTF1	0.118379	0.02546	0.056836
11729409_ZNF764	0.154471	0.025461	0.056836
11756618_TAPBP	0.1113	0.025464	0.056839
11740638_NOG	0.109806	0.025475	0.056862
11748608_ARHGEF11	0.093357	0.025508	0.056932
11747019_HMHA1	-0.20631	0.025509	0.056933
11741952_PAQR5	-0.18581	0.025515	0.056942
11761921_PYY	-0.13104	0.025518	0.056946
11745036_NRG1	0.124227	0.025525	0.05696
11723984_PDCD11	0.096934	0.025527	0.056962
11722288_ZBTB43	0.139961	0.025534	0.056974
11763727_RPL10	0.111002	0.025539	0.056985
11757422_RPL23	0.099064	0.025543	0.056989
11722672_AKAP11	0.105884	0.02555	0.057002
11715551_GAS6	0.14331	0.025554	0.05701
11764024_SFTPC	0.126939	0.025556	0.057012
11750720_GK	-0.1859	0.02557	0.057039
11738103_HIST1H4F	-0.17198	0.02559	0.057082
11758652_KANK1	0.112263	0.025598	0.057094
11740396_NLRP12	-0.14737	0.025599	0.057094
11763773_ITM2C	-0.18317	0.025599	0.057094
11759388_OR9Q2	-0.15181	0.025604	0.057103
11747548_PICALM	0.121015	0.02562	0.057135
11761866_NCOA7	-0.1801	0.025631	0.057157
11724275_TMEM158	-0.15656	0.025636	0.057166
11731661_DBP	-0.15426	0.025647	0.057187
11752284_ALOX15B	-0.14557	0.025654	0.0572
11750131_FGD1	-0.22133	0.025656	0.057204
11755566_MROH1	-0.11175	0.02567	0.057231
11719979_RAI1	-0.09937	0.025674	0.057238
11752300_PADI2	-0.10072	0.025679	0.057246
11733366_NR2E1	-0.22086	0.025687	0.057261
11725027_PSPC1	0.10946	0.025688	0.057261
11743507_ARHGAP26	0.239729	0.025689	0.057261
11737049_RIBC1	-0.20697	0.025693	0.057268
11744080_PRSS1 ///	-0.10107	0.025696	0.057272
11762173_NFYC	-0.14537	0.025699	0.057275

11724619_RSPO3	-0.24247	0.025702	0.05728
11719664_GDI1	0.150734	0.025723	0.057324
11742787_DHX34	-0.14769	0.025726	0.057327
11725865_EBF3	-0.1099	0.025727	0.057328
11715624_SH3BGRL	0.113029	0.025743	0.057361
11743778_HERC2	0.106662	0.025748	0.05737
11748066_LRRC49	0.121641	0.025755	0.057381
11755335_C1orf21	-0.14613	0.025759	0.057388
11745168_DIAPH1	-0.10897	0.025762	0.057392
11721749_INF2	-0.1836	0.025768	0.057402
11747584_C8orf34	0.10786	0.025769	0.057402
11750211_CYYR1	-0.19719	0.025772	0.057405
11727979_MAN2B2	0.149205	0.025778	0.057416
11756760_GCDH	-0.11512	0.025779	0.057416
11758247_ARL4D	-0.10826	0.025782	0.05742
11755392_SATB2	0.120487	0.025783	0.057421
11749466_GATS ///	-0.15022	0.025785	0.057421
11753231_GATS ///	-0.15022	0.025785	0.057421
11732487_TRIM50	-0.12089	0.025795	0.057438
11724014_SOAT1	-0.12733	0.025796	0.057439
11752635_KCNH2	-0.15333	0.025828	0.057507
11716402_EIF4EBP2	-0.14106	0.025829	0.057507
11763736_MBNL3	0.20614	0.025838	0.057525
11755288_KLHL26	-0.19918	0.025845	0.057538
11762346_MTAP	0.117344	0.025851	0.057549
11757426_GLB1L2	-0.11812	0.025854	0.057553
11760330_GPALPP1	-0.1258	0.025856	0.057555
11720167_SELL	-0.23518	0.025869	0.05758
11717559_ABLIM1	0.136693	0.02587	0.057581
11752454_TBKBP1	-0.18076	0.02588	0.0576
11738014_SMTNL2	-0.12782	0.025886	0.05761
11745418_COG3	0.230201	0.025891	0.057618
11733327_NKD1	-0.25985	0.025893	0.05762
11729304_UBE3B	0.097735	0.025894	0.05762
11748209_ABCA4	-0.19434	0.025895	0.057621
11756744_DUSP11	0.116575	0.0259	0.057627
11756932_ZNF257	0.146385	0.025907	0.057641
11757007_NUTM1	-0.17275	0.025908	0.057641
11754673_ABCA10	0.167191	0.025917	0.057659
11721432_MAP1S	0.11816	0.025919	0.05766
11749550_SCPEP1	-0.1777	0.025934	0.057692
11755595_TGDS	-0.10065	0.025937	0.057696
11729605_ATL1	0.16034	0.025947	0.057715
11731446_SNCA	-0.17274	0.025956	0.057731
11755662_LRSAM1	0.10821	0.025958	0.057734
11740737_ECE2	-0.17678	0.025974	0.057764
11742140_CFHR4	0.174889	0.025974	0.057764
11762515_ODF2L	0.196346	0.025975	0.057764
11763487_GLG1	-0.20052	0.025977	0.057767
11719566_MRPS15	-0.10685	0.026012	0.05784
11727842_SLC11A1	-0.13331	0.026014	0.057842

11762351_ZNF28	-0.16954	0.026021	0.057855
11759825_MR1	0.343667	0.026027	0.057864
11718109_DLD	0.10755	0.026027	0.057864
11755111_DLGAP4	0.111337	0.026033	0.057875
11740968_LOC391322	-0.18507	0.026036	0.057878
11747945_PPFIA2	0.16515	0.026041	0.057882
11751212_ABHD18	0.153896	0.026041	0.057882
11725042_YWHAB	-0.10675	0.026041	0.057882
11738866_PVRL1	-0.21262	0.026042	0.057882
11739060_SLC7A5	-0.13946	0.026058	0.057911
11727096_TMEM19	-0.10137	0.026058	0.057911
11724526_FAHD1	0.163661	0.026077	0.057952
11750406_DPY19L3	0.145969	0.026083	0.057961
11749972_TIE1	-0.13944	0.026087	0.057969
11749761_PCED1A	0.094793	0.026104	0.058002
11750355_CCPG1 ///	0.126872	0.026117	0.05803
11730234_CTNND2	0.170851	0.026147	0.058094
11726814_KDM5D	-0.233	0.02615	0.058098
11760540_PTCHD4	0.227599	0.026163	0.058125
11759606_YIPF2	-0.16174	0.026165	0.058125
11726243_ANKDD1A	0.138044	0.026168	0.058129
11725420_ARHGAP35	-0.1119	0.026182	0.058159
11754036_C20orf194	0.113524	0.026191	0.058176
11735064_ITGB3	-0.15286	0.026197	0.058187
11737911_GPR84	-0.18839	0.026199	0.058187
11750632_ZFYVE16	0.203418	0.026204	0.058193
11750701_MAN2C1	0.103143	0.026204	0.058193
11744204_PAF1	0.127868	0.02621	0.058205
11733785_PPP2R1B	-0.1233	0.026216	0.058215
11718213_SLC27A2	-0.11527	0.026231	0.058245
11722465_C17orf80	0.097929	0.026236	0.058253
11760615_SKIV2L	-0.16435	0.026237	0.058253
11737723_GLIS3	0.11181	0.026243	0.058265
11716021_RAD23A	-0.10555	0.026253	0.058283
11716549_ISLR	-0.17864	0.026254	0.058283
11725868_SSC5D	-0.11959	0.026256	0.058286
11747136_POLR1A	-0.19781	0.026263	0.058296
11743480_RB1CC1	0.115949	0.026263	0.058296
11745625_PCDHGA2	-0.12508	0.02627	0.058309
11751511_ETV7	0.180779	0.026292	0.058354
11749521_CPT1C	-0.12513	0.026307	0.058386
11747197_CR1L	0.142075	0.026322	0.058416
11753304_APBB3	-0.09057	0.026339	0.058448
11740512_PDE4D	0.227016	0.026339	0.058448
11727233_EPG5	0.190595	0.026376	0.058527
11741303_PRICKLE4	0.18505	0.026383	0.05854
11744307_TTC39A	0.225482	0.026384	0.05854
11736968_TMEM213	0.194316	0.026385	0.05854
11760680_FSD1	-0.22208	0.026389	0.058546
11724839_XRCC3	-0.16645	0.026401	0.05857
11739144_SKP2	0.12173	0.026406	0.058577

11735965_PLXNA4	-0.12747	0.026413	0.058591
11736024_MED28	0.24893	0.026421	0.058605
11717384_RNF34	0.101155	0.026427	0.058617
11761849_CRADD	-0.13566	0.026433	0.058628
11747491_KIAA0513	-0.17383	0.026451	0.058664
11748933_CCDC150	-0.2268	0.026459	0.058679
11734392_TAS2R10	0.186372	0.026475	0.058713
11734729_SLC39A12	0.200535	0.026481	0.058723
11744549_COX20 ///	0.113687	0.026483	0.058724
11721537_COL4A3BP	-0.17765	0.026494	0.058748
11733179_SH3GL3	-0.27657	0.0265	0.058758
11729752_EPS15L1	-0.1034	0.026507	0.05877
11727359_SLC6A3	-0.18908	0.026508	0.05877
11725201_BSN	0.347931	0.026511	0.058774
11762092_LETM1	-0.13266	0.026513	0.058777
11719544_MSLN	-0.12548	0.026533	0.058817
11744922_RPL13P5	-0.14026	0.026546	0.058842
11760368_TXNRD2	-0.38132	0.026547	0.058842
11725700_XIAP	0.102036	0.026549	0.058842
11729274_GBP5	0.224834	0.026549	0.058842
11731094_TRAF3IP1	0.219328	0.026557	0.058857
11762550_SCML4	-0.26366	0.026559	0.05886
11725686_PTPRO	0.2312	0.026564	0.058867
11750917_C15orf39	0.116079	0.026566	0.058868
11728576_PYROXD2	0.121475	0.026574	0.058885
11735270_GCNT1	-0.18054	0.026601	0.058942
11762249_RPF2	0.305825	0.026603	0.058942
11742037_CACNB2	-0.17322	0.026611	0.058956
11728589_MFSD13A	0.097418	0.026612	0.058956
11758525_SOX4	-0.107	0.026612	0.058956
11736877_DTWD2	0.180227	0.026627	0.058983
11736747_ADAM28	-0.23032	0.026627	0.058983
11725745_SORBS1	0.185832	0.026638	0.059003
11735527_IFT74	0.128952	0.026638	0.059003
11717101_SLC38A1	-0.18437	0.026643	0.059011
11733612_DOCK8	0.198763	0.026671	0.05907
11754533_NUDCD3	-0.13437	0.026677	0.059081
11728473_PLPPR5	-0.22629	0.026684	0.059093
11735027_LOC105369	-0.12775	0.026703	0.059133
11736704_C6	-0.15765	0.026709	0.059144
11744640_SH3D21	-0.23154	0.02672	0.059165
11758885_MRPS11	-0.11294	0.026722	0.059168
11762355_LOC554207	0.247376	0.026741	0.059206
11717304_PDAP1	-0.1002	0.026784	0.059296
11740985_NRDC	0.094901	0.026784	0.059296
11761254_NRP1	-0.16779	0.026798	0.059324
11719628_PUDP	-0.12196	0.0268	0.059325
11759530_NUDT22	0.248051	0.026801	0.059325
11722294_NAPSA	-0.12607	0.026819	0.059362
11716088_SNW1	-0.10821	0.026827	0.059378
11747934_DTX2P1-UF	0.134049	0.026829	0.059379

11758817_RRP7A	0.144447	0.026835	0.05939
11762504_ZRANB3	-0.17038	0.026848	0.059416
11720662_TSR3	-0.11009	0.026856	0.05943
11753862_KRTAP20-4	-0.16345	0.026858	0.059432
11744876_LARP4	0.093216	0.02687	0.059457
11736846_STARD13	0.118528	0.026873	0.05946
11751458_TRIM68	-0.12354	0.02689	0.059496
11759800_AKAP6	0.189717	0.02691	0.059537
11729396_NEK1	0.099727	0.026912	0.059538
11741613_KCNB2	-0.10841	0.026922	0.059558
11718799_PPCDC	-0.13425	0.026929	0.05957
11719518_SEMA5A	0.134003	0.02693	0.05957
11730100_FLT3LG	-0.15641	0.026936	0.05958
11747417_FGFR1	-0.21505	0.026937	0.05958
11746603_ACAP3	-0.15752	0.026943	0.059592
11723233_LEO1	0.103038	0.02695	0.059605
11726637_MIB1	-0.09595	0.026957	0.059615
11741246_FOXB1	-0.13351	0.026957	0.059615
11722685_LIFR	-0.16896	0.026966	0.059632
11761416_CTHRC1	0.114473	0.026969	0.059636
11741936_METTL24	-0.16311	0.02698	0.059656
11731048_RIMKLA	-0.14309	0.026984	0.059663
11761876_SKCG-1	0.096846	0.026988	0.059669
11762310_LOC202181	-0.16317	0.026991	0.059672
11747059_ZBTB7C	-0.15197	0.027002	0.059694
11717917_WFDC2	-0.14738	0.027007	0.059703
11752397_CPAMD8	-0.18674	0.027024	0.059738
11748035_PDLIM7	-0.14466	0.02703	0.059748
11756054_HSPB1	-0.10418	0.027042	0.059773
11748419_GATS	-0.20879	0.027048	0.059782
11728036_CMTM5	-0.12814	0.02705	0.059784
11751408_PYCRL	-0.1114	0.027058	0.0598
11734543_SPINK8	-0.14895	0.027063	0.059808
11723445_TMEM132A	0.120596	0.027073	0.059828
11739590_DRAXIN	-0.20822	0.02708	0.05984
11723532_BRMS1L	0.119917	0.027106	0.059894
11730339_SMAD6	0.123277	0.027113	0.059908
11723863_DOCK10	0.266378	0.02713	0.059942
11748923_SCLY ///	0.092521	0.027135	0.059951
11750190_CD44	0.10472	0.027136	0.059951
11719857_ARID1A	-0.11832	0.027141	0.059959
11733528_CRB2	-0.1595	0.027153	0.059982
11716476_LARP1	-0.10268	0.027154	0.059983
11754426_CHPF	-0.09448	0.027156	0.059983
11741812_LCNL1	-0.11671	0.027161	0.059992
11737814_GYG1	0.102405	0.027164	0.059997
11733461_SUSD5	-0.13853	0.02717	0.060006
11725035_LMBRD1	0.146436	0.027173	0.060009
11742078_FAM222A	-0.10447	0.027188	0.060039
11730147_ADGRG5	-0.15911	0.027189	0.060039
11720378_SAP30L	0.092695	0.027193	0.060046

11757947_PSMD5	0.114592	0.0272	0.060059
11760979_NR4A1	-0.19329	0.027221	0.060102
11735777_SPDYC	-0.29403	0.02723	0.06012
11716696_TSPAN6	0.091911	0.027241	0.060141
11749120_SCUBE2	0.183999	0.027249	0.060155
11738364_CEACAM16	-0.14433	0.02725	0.060155
11734608_SHQ1	0.115192	0.027251	0.060155
11744161_RFX1	-0.10682	0.027256	0.060163
11724904_TIMP4	-0.09877	0.027263	0.060176
11743119_XRN2	0.110128	0.027265	0.060178
11730643_CT55	-0.24575	0.027273	0.060191
11762894_GOSR1	0.201842	0.027273	0.060191
11753637_RPS12 ///	0.093546	0.027275	0.060191
11762454_COL8A1	0.260564	0.027288	0.060217
11724973_MYRIP	0.235425	0.027296	0.060233
11741975_OR11A1	-0.12845	0.027301	0.060237
11742032_NUTM2G	-0.17826	0.027301	0.060237
11759708_NSUN5 ///	-0.13833	0.027302	0.060237
11717002_YTHDC1	-0.15619	0.02732	0.060275
11762025_DOCK5	0.108125	0.027323	0.060278
11754611_EPS8L2	-0.18744	0.027324	0.060278
11717132_AHNAK2	0.170555	0.027334	0.060297
11761247_CDKAL1	0.200895	0.027337	0.060301
11735086_CUBN	0.23023	0.02734	0.060305
11727809_FECH	-0.15697	0.027342	0.060307
11723632_IRF2BPL	-0.13239	0.027349	0.060321
11739834_HSPE1-MOB	-0.10454	0.027353	0.060324
11751572_TAPBP	0.148396	0.027353	0.060324
11732399_RAD51D	-0.13323	0.027355	0.060325
11742965_MTERF4	0.185272	0.027363	0.06034
11717899_MAGT1	-0.1253	0.027369	0.060351
11733162_PSTPIP1	-0.09902	0.027379	0.06037
11732934_DPPA5	-0.12014	0.027394	0.0604
11731349_UEVLD	0.188855	0.027395	0.0604
11744784_TXNDC9	0.108091	0.0274	0.060408
11747995_TBC1D1	0.172756	0.027411	0.06043
11740864_FBXO22	0.221555	0.027429	0.060466
11725325_TSPAN31	0.119824	0.02743	0.060466
11755980_SLC25A3	0.090413	0.027437	0.060478
11733623_ZNF831	-0.17938	0.02744	0.060478
11724862_THNSL1	-0.12974	0.02744	0.060478
11749788_L2HGDH	-0.13721	0.027441	0.060478
11729748_IL10RA	-0.15315	0.027444	0.060482
11763461_PLCB3	-0.11113	0.027455	0.060504
11737891_PAX5	-0.09837	0.027463	0.060519
11743836_NCBP3	0.142585	0.027486	0.060566
11748593_ADAT2	-0.27156	0.027487	0.060566
11724531_MBIP	-0.10507	0.027503	0.060598
11749422_FAM65A	-0.11975	0.027503	0.060598
11734842_MPHOSPH8	0.181961	0.027508	0.060604
11724180_DNAJB5	0.135814	0.027511	0.060609

11721829_C9orf78	0.105674	0.027516	0.060618
11739736_GNAT1	-0.14523	0.027524	0.060631
11754808_CPAMD8	-0.12865	0.027529	0.06064
11729113_PSMD7	-0.10824	0.027546	0.060674
11744688_HNRNPDL	-0.10265	0.027554	0.06069
11760745_STK32B	0.194788	0.027555	0.06069
11742126_TMEM74B	-0.17152	0.027558	0.060693
11751914_ELF3	-0.13447	0.027563	0.060702
11731107_STAG2	-0.0956	0.027565	0.060703
11757514_RPS27	0.090193	0.02757	0.060711
11734923_ATHL1	-0.17437	0.027576	0.060722
11732409_KTN1	0.174475	0.027603	0.060778
11734099_ZNF578	0.198462	0.027604	0.060778
11733862_TSTD2	0.102952	0.027624	0.06082
11751879_IYD	-0.15841	0.027626	0.060822
11740509_ARHGAP26	0.306832	0.027632	0.060833
11739714_NCOA2	0.135317	0.027637	0.060838
11716415_NDUFB8	0.100279	0.027638	0.060838
11722943_ATXN1	0.146118	0.027662	0.060889
11749408_ZFR	0.105031	0.027665	0.060893
11730917_TAF6L	-0.14966	0.027668	0.060896
11731161_ITGA2B	-0.17333	0.027669	0.060897
11736085_CTNNBL1	0.118373	0.027681	0.06092
11742985_LY9	-0.16603	0.027686	0.060926
11755102_SELO	-0.09579	0.027686	0.060926
11719028_PSD3	0.151573	0.027689	0.060929
11743113_S100A10	0.097622	0.027693	0.060935
11726875_PML	-0.17477	0.027697	0.060941
11759364_GPR157	0.140086	0.0277	0.060946
11753758_NUTF2	-0.09599	0.027707	0.060958
11762695_SORBS2	0.185317	0.027725	0.060995
11757726_NHP2	-0.09915	0.027727	0.060997
11721235_CHAD	-0.09811	0.027732	0.061005
11734943_SLAMF6	-0.14421	0.027733	0.061005
11733979_CD28	-0.12695	0.027737	0.06101
11734125_NOD1	-0.11617	0.02774	0.061014
11733183_ETV1	0.148538	0.027744	0.061021
11742109_FBLN7	-0.20065	0.027746	0.061023
11755565_KLK8	-0.16742	0.027756	0.061041
11727473_CLC	-0.16821	0.027794	0.061122
11726029_UPK1B	-0.18609	0.027798	0.061129
11750397_RASGRP1	-0.20097	0.027803	0.061137
11725431_PBX2	0.136699	0.027807	0.061142
11733982_PDK1	0.160962	0.02781	0.061145
11729141_KDM5C	-0.2289	0.027813	0.061151
11717074_LZTS2	-0.09755	0.027825	0.061174
11760713_TMEM126B	-0.14375	0.027835	0.061191
11726891_SFTA3	-0.09854	0.027836	0.061191
11757670_UQCR10	-0.09692	0.027856	0.061233
11746419_SNX17	0.09368	0.027871	0.061261
11737740_FGF4	-0.1265	0.027872	0.061261

11723761_PLCG1	-0.13047	0.027872	0.061261
11736421_ZNF800	0.102125	0.027874	0.061263
11724733_ABCB8	-0.12657	0.027883	0.061279
11748306_M1AP	-0.1067	0.027886	0.061284
11724712_LRRC23	-0.20429	0.027905	0.061322
11761176_POLL	-0.16931	0.027908	0.061325
11752235_IKBKB	-0.13122	0.027916	0.06134
11746620_FCRL2	-0.13284	0.027917	0.06134
11722756_TMEM37	-0.19901	0.02792	0.061341
11762308_DHRS4 ///	-0.12515	0.02792	0.061341
11726389_NLGN3	0.219275	0.027921	0.061341
11735798_OPN1MW //	-0.13488	0.027924	0.061341
11727478_AGTRAP	-0.11844	0.027924	0.061341
11739153_PSMD5	0.113473	0.027931	0.061354
11760932_TEFM	0.235218	0.027935	0.06136
11727926_ADAP1	-0.24729	0.027937	0.061363
11760857_CACNB3	-0.16757	0.02794	0.061365
11743535_PPP2R5C	-0.09583	0.027943	0.061369
11746069_GATM	0.205198	0.027949	0.06138
11746049_VPS52	-0.14144	0.027954	0.061388
11762718_WDR92	-0.1475	0.027961	0.0614
11731339_ECE2	-0.1622	0.027965	0.061407
11743421_ITGA9	-0.24342	0.027971	0.061417
11760950_UROD	-0.15464	0.027973	0.061417
11728845_NCOA1	0.255138	0.027974	0.061417
11740030_CREM	0.122081	0.027974	0.061417
11722099_RICTOR	0.123681	0.027976	0.061417
11722828_SDAD1 ///	0.13259	0.027983	0.061431
11734444_AARD	-0.13662	0.027993	0.06145
11727273_SHROOM2	-0.11581	0.027997	0.061454
11752523_ST8SIA5	-0.18074	0.027999	0.061454
11726712_TFIP11	0.137349	0.027999	0.061454
11729073_TSPAN19	0.142788	0.028006	0.061466
11724576_MYCBPAP	-0.0928	0.028007	0.061466
11731143_ADGRD1	0.149948	0.028014	0.061477
11731327_GFAP	-0.14732	0.028024	0.061498
11742682_COPS8	-0.10504	0.028026	0.061499
11740579_NTRK3	-0.19961	0.028038	0.061522
11726173_SCARA3	-0.12536	0.02804	0.061525
11741840_PIWIL3	0.165923	0.028056	0.061556
11723693_TMEM183A	0.095275	0.028063	0.061569
11752629_RBM39	0.112089	0.028064	0.061569
11732170_GRSF1	0.116114	0.028066	0.06157
11752675_OGFR	-0.11023	0.028076	0.06159
11738253_PCDHA10	0.176146	0.028083	0.061603
11744557_STOML1	0.121451	0.028093	0.061622
11748841_SAMHD1	0.155934	0.028097	0.061626
11749141_TRAF5	0.184103	0.028097	0.061626
11743658_H2AFY2	-0.1772	0.0281	0.061629
11729041_DLEC1	-0.11626	0.028107	0.061641
11757149_EEF1B2 //	-0.12308	0.028109	0.061642

11762992_ATP6 ///	0.090245	0.028116	0.061655
11759640_CLEC16A	0.2678	0.028126	0.061675
11723308_SCAF8	-0.0915	0.028152	0.061728
11749420_MAMLD1	-0.14694	0.028153	0.061728
11753407_FUT6	0.130961	0.028155	0.061729
11733450_SLFN11	-0.19084	0.028158	0.061733
11716024_GOLGA7	0.107997	0.028169	0.061755
11753498_SLED1	-0.14269	0.028172	0.061757
11748204_CTNND2	-0.14977	0.028173	0.061757
11721390_HTRA3	-0.12498	0.028174	0.061758
11752405_SPATA13	-0.1183	0.028181	0.061767
11757573_FZD5	0.093114	0.028181	0.061767
11716952_DPH1 ///	0.145439	0.028205	0.061817
11742148_KRTAP10-1	-0.12347	0.028221	0.06185
11720640_MRPS27	0.107409	0.028223	0.061851
11763835_HACD1	-0.24411	0.028228	0.06186
11745716_CAV1	0.09403	0.028232	0.061866
11754007_GNAS	-0.08924	0.028242	0.061884
11739152_TTYH3	-0.12094	0.028256	0.061912
11735037_SORCS1	0.148337	0.028258	0.061913
11725561_OGFOD3	-0.12004	0.028263	0.061922
11748446_IL17RC	-0.11529	0.028274	0.061943
11738856_CERKL	-0.13944	0.028306	0.06201
11734409_FOXD4L1	0.15397	0.028313	0.062022
11729923_TMEM134	0.10333	0.02832	0.062035
11737446_SYT16	0.290547	0.028322	0.062038
11721022_DIDO1	-0.098	0.028324	0.06204
11739952_GFOD1	-0.13896	0.028327	0.062043
11735455_IFT81	0.256127	0.028331	0.062048
11736323_BAIAP3	-0.15282	0.028336	0.062056
11738985_DKK3	-0.11776	0.028337	0.062057
11727226_NAALAD2	-0.17775	0.028341	0.062063
11727295_ZSCAN32	0.1781	0.028347	0.062072
11756895_SBF1	-0.10053	0.028349	0.062075
11753554_SPINK13	0.202421	0.028359	0.062094
11733732_GFOD2	0.146226	0.028364	0.062101
11715167_ARHGAP22	-0.14716	0.028367	0.062107
11762313_ECHDC2	-0.10182	0.028377	0.062124
11745566_INTS4	-0.09423	0.028383	0.062135
11761245_CHRD	-0.28885	0.02839	0.062146
11747391_FBXO44	-0.21315	0.02839	0.062146
11721073_SF3A1	-0.18036	0.028392	0.062146
11758332_LINC01001	0.10106	0.028402	0.062165
11748677_ULK3	0.156327	0.028411	0.062183
11731017_KLK4	-0.12075	0.028432	0.062224
11747880_BCL2L13	0.114137	0.028448	0.062258
11732310_CTU2	-0.09856	0.028468	0.062298
11747560_RPS6KB2	-0.13421	0.028469	0.062298
11754145_IGKV1-27	0.128611	0.02847	0.062298
11756059_CXCL14	-0.17804	0.028472	0.062299
11745946_C5orf28	0.135279	0.028496	0.062349

11744172_DNAJC4	-0.12343	0.028501	0.062357
11751765_RAVER2	-0.15293	0.028513	0.062381
11749021_CTNNB1	0.092824	0.028516	0.062384
11739578_PGBD1	-0.17108	0.028532	0.062417
11715720_C1orf43	0.089107	0.028541	0.062433
11729934_HSF4	-0.11801	0.028542	0.062433
11736221_CCDC103 /	-0.14599	0.02855	0.062448
11757844_GNG5	0.094242	0.028554	0.062453
11751411_CCDC64	-0.13124	0.028564	0.062473
11761745_IGSF6	-0.24941	0.02857	0.062482
11740316_MIP	0.110849	0.028575	0.062491
11733851_EIF2A	-0.15803	0.028577	0.062491
11757074_MLLT4	-0.16172	0.028584	0.062504
11721031_SH3BGR	0.195212	0.028585	0.062504
11751057_SH2D4A	0.156413	0.028596	0.062527
11747248_MAP3K13	0.340007	0.028606	0.062542
11760035_GRIN2C	-0.09041	0.028607	0.062542
11724896_FZR1	-0.09835	0.028607	0.062542
11747153_ACOT9	0.111001	0.028608	0.062542
11728214_NRP1	0.15628	0.02861	0.062542
11742255_POTEG ///	0.142171	0.028614	0.062549
11760416_TMEM39B	0.154459	0.028628	0.062575
11719150_SAP130	-0.17914	0.028647	0.062614
11733694_C1orf61	-0.1192	0.028668	0.062658
11758653_ADGRL4	0.165308	0.028674	0.062665
11758808_CNIH4	0.127252	0.028674	0.062665
11722159_SLC7A8	0.10755	0.028676	0.062665
11750677_FAM65C	-0.1178	0.028676	0.062665
11728584_ZMAT1	-0.19015	0.028706	0.062727
11745816_CDIP1	-0.18081	0.028718	0.062751
11742163_MYEOV2	0.119295	0.028721	0.062755
11761214_APOD	0.125385	0.028724	0.062757
11730481_GJB6	0.137089	0.028725	0.062757
11740808_PRAMEF1 /	-0.11789	0.028732	0.062767
11762592_DBF4B	-0.14971	0.028732	0.062767
11719254_CDK19	0.343891	0.028759	0.062824
11716469_UQCRB	-0.11104	0.028766	0.062835
11720373_PRUNE	0.143529	0.028769	0.062838
11759818_DDHD2	-0.17376	0.02879	0.062881
11756013_BCL2L10	-0.09546	0.028796	0.062892
11739839_GNAL	-0.27478	0.028811	0.062922
11737155_OXT	0.10814	0.028817	0.062931
11755956_POLE3	-0.09992	0.028818	0.062931
11759115_GMCL1	0.156273	0.028824	0.062943
11739955_IL2RA	-0.11159	0.028833	0.062959
11751202_C19orf66	0.149916	0.028835	0.06296
11761517_MACF1	0.212396	0.028853	0.062998
11756852_KIAA0100	-0.12461	0.028866	0.063023
11745076_CRNKL1	0.113441	0.028903	0.0631
11762696_ADAMTSL1	-0.16215	0.02891	0.063114
11738562_GFRA4	-0.16878	0.028914	0.063118

11758425_ZNF782	0.173032	0.028915	0.063118
11727392_COX15	0.123084	0.028916	0.063118
11717592_CENPB	-0.10172	0.028926	0.063137
11748085_ST14	-0.12492	0.02894	0.063165
11753750_SUMO2	-0.13164	0.028948	0.06318
11725914_POU6F1	-0.17267	0.028956	0.063194
11737756_ATG9A	0.097251	0.028973	0.063227
11746370_ENTPD6	0.095795	0.028976	0.063231
11752615_DLG1	0.149157	0.028983	0.063242
11740345_SNX29	-0.12965	0.028983	0.063242
11721711_SHROOM3	-0.17032	0.028985	0.063242
11755755_DDX26B	0.16884	0.028996	0.063263
11743166_ETV6	0.156724	0.028997	0.063264
11761257_ARMC6	-0.16028	0.029003	0.063275
11763720_SNAP29	-0.1547	0.029033	0.063336
11741160_SWT1	0.181179	0.029036	0.063341
11718044_RAB11B	-0.13921	0.029039	0.063343
11724639_PAFAH2	0.241829	0.029045	0.063354
11732488_HIST1H2BA	-0.14688	0.029047	0.063354
11725534_ZNF618	-0.11825	0.029047	0.063354
11716720_AKT1S1	-0.15105	0.029069	0.063399
11732462_ADAMTSL1	0.289976	0.029082	0.063423
11747387_CTNNB1	0.132568	0.029088	0.063435
11748621_CDK11A //	0.10485	0.029091	0.063438
11753572_EMC4	-0.09291	0.029096	0.063447
11730494_KCNK2	0.138339	0.029122	0.063499
11753380_C3orf67	-0.17303	0.029123	0.063499
11746500_GSTO2	-0.09864	0.029128	0.063507
11750869_ZNF852	0.133582	0.029143	0.063537
11735381_MAP3K19	-0.17646	0.029151	0.06355
11737935_PCDHGA5	-0.13427	0.029152	0.06355
11721329_RNGTT	0.170879	0.029153	0.06355
11762731_SUMO1	-0.1808	0.029154	0.063551
11741236_TRIM17	-0.16999	0.029163	0.063564
11724653_ZBTB47	-0.29242	0.029163	0.063564
11723983_KRT6B	-0.13719	0.02918	0.063597
11727310_PLIN4	-0.11845	0.029186	0.063608
11752816_LIG1	-0.14062	0.029189	0.063611
11744542_POLR3F	-0.11584	0.029198	0.063629
11733365_NR2E1	-0.19105	0.029212	0.063656
11744420_MIR600HG	-0.16903	0.029223	0.063674
11762537_LINC00328	0.13396	0.029224	0.063674
11758025_INPP5F	-0.09507	0.029224	0.063674
11762507_GTDC1	-0.19566	0.029229	0.063683
11733626_FAM196A	-0.1731	0.029236	0.063694
11737978_NIM1K	0.155084	0.029246	0.063714
11718204_REC8	0.187274	0.029255	0.06373
11748506_HTR2C	-0.13574	0.029258	0.063734
11726430_C11orf52	0.104454	0.029268	0.063753
11724040_EHHADH	-0.16101	0.029276	0.063767
11716886_VPS45	-0.09246	0.029278	0.063767

11752211_VWA2	0.154796	0.029279	0.063767
11744649_HP	-0.24198	0.029282	0.063771
11716668_RAB1A ///	-0.10138	0.029289	0.063784
11734629_GPHB5	-0.10772	0.029299	0.063802
11743163_ATF1	-0.15213	0.029318	0.063841
11715643_ATP6AP2	0.100216	0.02932	0.063843
11715342_BRI3	0.216068	0.029322	0.063843
11737380_PRR19	-0.1799	0.029322	0.063843
11730541_TAF1A	0.098071	0.029339	0.063876
11737074_COPE	0.099564	0.029345	0.063887
11731460_CPM	0.090642	0.029368	0.063932
11759828_SP100	0.148607	0.029392	0.063982
11756975_TBX2	0.10754	0.029406	0.06401
11762432_WDR31	0.24007	0.029408	0.064012
11740594_HIST1H3E	0.223896	0.029413	0.06402
11741769_SNX21	-0.17503	0.029414	0.06402
11732566_SLC6A14	0.226498	0.029437	0.064066
11732937_CPT1A	0.164867	0.029441	0.064072
11737928_C6orf141	-0.11463	0.029449	0.064088
11749892_RGS11	-0.15712	0.029452	0.064091
11716788_B4GAT1	0.114369	0.029456	0.064093
11739695_ATL1	0.268573	0.029456	0.064093
11719951_FKBP15	0.145417	0.029458	0.064095
11719130_POLR3D	-0.09728	0.02946	0.064097
11753559_SAA3P	-0.18273	0.029472	0.064119
11731708_EGFLAM	-0.23133	0.029473	0.064119
11745860_ATP5SL	-0.11073	0.029476	0.064124
11719727_SIRPA	-0.09974	0.029484	0.064138
11726146_TMEM61	-0.11322	0.029497	0.064162
11720925_TCAP	-0.11433	0.029516	0.064199
11723362_SOCS6	0.095939	0.029516	0.064199
11733227_TTC5	-0.10815	0.029528	0.064222
11756860_RNH1	0.108284	0.029541	0.064245
11734931_LRRTM3	-0.14493	0.029542	0.064245
11757417_CD63	0.107284	0.029552	0.064265
11729500_HHATL	-0.10979	0.029564	0.064288
11764003_ARMC9	0.230121	0.029585	0.064331
11748249_NDRG3	0.132223	0.029588	0.064336
11734838_ACSF3	-0.10437	0.029591	0.064338
11761340_RRN3P1	0.197353	0.029602	0.064361
11747024_CHRM5	-0.19595	0.029608	0.064371
11737448_DNAJC22	0.118465	0.029631	0.064418
11740865_CLEC9A	-0.16152	0.029634	0.06442
11762208_ATG16L2	-0.13832	0.029656	0.064466
11747629_SYNPO2	-0.15092	0.029661	0.064473
11740379_RHOT1	0.098654	0.02967	0.06449
11735669_MROH8	-0.21373	0.029687	0.064524
11724127_MID2	0.150689	0.029711	0.064572
11725162_CYP39A1	-0.10028	0.029711	0.064572
11724426_CNIH2	-0.10551	0.029721	0.06459
11717665_TSPYL4	0.108585	0.029725	0.064596

11734450_ACTRT1	-0.15655	0.029727	0.064597
11727365_MED29	-0.10776	0.029731	0.064603
11737667_KCNH6	-0.11326	0.029743	0.064627
11733026_CLCA2	0.234079	0.029751	0.06464
11724244_TCF4	-0.13706	0.029752	0.06464
11753676_AGER	-0.17092	0.029762	0.064658
11732746_HGF	-0.13358	0.029768	0.064669
11743494_SPATA5L1	0.11703	0.029779	0.06469
11748270_KMT5A	0.123048	0.029781	0.06469
11743179_RSF1	0.113764	0.029782	0.064691
11733921_GJB6	-0.14996	0.029789	0.064703
11745110_GLYCTK	-0.17367	0.029808	0.064742
11738450_GP5	-0.12119	0.029812	0.064748
11755886_C5orf42	0.109542	0.029814	0.064748
11750707_GLP1R	-0.12637	0.029825	0.064766
11750005_CNTROB	-0.10451	0.029826	0.064766
11745373_SNX22	-0.13466	0.029826	0.064766
11727653_SRRM4	-0.20097	0.02983	0.064771
11753702_TMEM220	-0.17615	0.029833	0.064776
11730084_MINK1	0.20767	0.029835	0.064776
11733160_FAM84A	-0.13342	0.029839	0.064783
11723178_SLC15A4	-0.10753	0.029862	0.06483
11748912_LMF1	-0.14476	0.029877	0.06486
11727617_EFNA3	-0.15782	0.029885	0.064873
11737332_TRPM3	0.271104	0.029894	0.064885
11720533_HMGCS2	-0.28385	0.029895	0.064885
11758613_ZNF507	0.121718	0.029896	0.064885
11761950_NALCN	0.185962	0.029898	0.064885
11723802_F10	-0.14426	0.029899	0.064885
11738591_IQCJ	-0.161	0.0299	0.064885
11734856_ERO1B	0.218899	0.029901	0.064885
11749634_CDON	-0.18236	0.029901	0.064885
11732838_GUCA2B	-0.16572	0.029909	0.0649
11744246_KCNE2	0.097421	0.029915	0.06491
11758280_HSD17B6 /	-0.15819	0.02992	0.064918
11730200_ALAD	0.164668	0.029923	0.064922
11722102_TYRO3	-0.12687	0.029935	0.064944
11721386_CUL7	0.155092	0.02995	0.064974
11716739_GRAMD1A	0.134834	0.029952	0.064975
11719043_ALKBH5	-0.13587	0.029955	0.06498
11721684_ZW10	-0.10043	0.030003	0.06508
11746108_MPP2	-0.16646	0.030007	0.065087
11721442_RNASEH2C	-0.09796	0.030018	0.065108
11759646_ZC3H7A	0.251892	0.030028	0.065127
11720773_DNAJC19	-0.22388	0.030061	0.065195
11723169_FOXN2	-0.0928	0.030067	0.065204
11751543_HFE	-0.19706	0.030078	0.065226
11729315_ULK4	0.097678	0.030079	0.065226
11751531_CD99L2	-0.15663	0.030082	0.065229
11727483_TPR	-0.09756	0.03009	0.065243
11715639_TTC3	0.12002	0.030097	0.065255

11744123_BRINP2	-0.17679	0.030102	0.065263
11728959_LCLAT1	0.135562	0.030113	0.065284
11763059_LOC100128	-0.14559	0.030117	0.06529
11752679_TAB3	0.195329	0.030128	0.065312
11730017_EPHA10	-0.13459	0.030134	0.065322
11737417_ERCC6L2	0.234229	0.030136	0.065322
11723634_GDF3	-0.1076	0.030147	0.065341
11719710_PDPK1	0.127339	0.030147	0.065341
11745108_MYT1	-0.14731	0.030166	0.065379
11743791_FRS2	0.113099	0.030171	0.065388
11737678_C1QTNF8	-0.14675	0.030177	0.065397
11746805_ERCC8	0.110645	0.030185	0.065411
11722073_HEYL	-0.17595	0.030186	0.065411
11718124_AIMP1	-0.10204	0.0302	0.065437
11729703_DBN1	-0.1649	0.030216	0.065469
11762477_CPXM2	-0.15557	0.030217	0.065469
11739780_ARL17A //	0.121621	0.030226	0.065486
11727366_MED29	-0.12275	0.030227	0.065486
11737996_LRRC3	0.091858	0.030241	0.065512
11738009_DGKB	-0.13181	0.030243	0.065513
11722462_TNKS2	0.10004	0.030246	0.065517
11760945_BMP1	-0.12681	0.030248	0.065518
11716640_PSMB10	0.106675	0.030261	0.065545
11763498_CPT1C	-0.127	0.030275	0.065572
11717127_ANPEP	0.102479	0.030281	0.065581
11723292_ORAI1	0.10475	0.030297	0.065612
11715155_TUBB7P //	-0.08837	0.030297	0.065612
11753368_OAZ1	-0.10981	0.030306	0.065627
11754658_WSCD2	-0.14966	0.030307	0.065627
11717447_DSTN	-0.08904	0.030326	0.065663
11723579_SMIM13	0.181199	0.030327	0.065663
11752628_SYNCRIP	-0.10705	0.030329	0.065665
11726616_MARCO	-0.11609	0.030332	0.065669
11735977_HCCAT5	-0.22607	0.030342	0.065688
11720987_SEC22A	0.125658	0.030362	0.065728
11722507_CLIC5	-0.12712	0.030371	0.065745
11731394_VIM	-0.08733	0.030385	0.065773
11746660_TMEM260	0.143258	0.030391	0.065782
11723865_CHODL	-0.24292	0.030401	0.065799
11737151_ACOT6	-0.169	0.030401	0.065799
11727041_TTI2	0.099649	0.030407	0.065808
11753807_TIMM10B	0.100516	0.030411	0.065811
11757043_MAGED2	0.120443	0.030411	0.065811
11740838_FAM98B	-0.168	0.030418	0.065822
11750668_DENND4A	0.122884	0.030421	0.065827
11763176_NPRL3	-0.10405	0.030438	0.06586
11735933_C2CD4D	-0.1095	0.030446	0.065875
11727632_SLC16A10	-0.12792	0.030465	0.065912
11747903_SAMD4B	-0.1476	0.030466	0.065912
11755688_ADAMTSL4	-0.18787	0.030473	0.065922
11747529_CALCOCO1	-0.19687	0.030475	0.065922

11745383_XRCC6BP1	-0.09144	0.030475	0.065922
11734936_CNOT2	-0.11177	0.030479	0.065928
11756827_RTN1	-0.17503	0.030481	0.06593
11719777_NDST1	-0.11515	0.030483	0.065932
11763482_GRK1	-0.13201	0.030485	0.065932
11753195_SCUBE1	-0.11042	0.030496	0.065953
11730618_ULBP1	-0.14269	0.030497	0.065953
11759381_ADAM19	-0.18639	0.030511	0.065981
11749723_FUT8	-0.10623	0.030519	0.065995
11750483_DIP2C	-0.12715	0.030528	0.066011
11739815_DLG4	0.172477	0.03054	0.066035
11756308_FAM50A	0.118966	0.030543	0.066038
11749944_GABRP	0.165021	0.030548	0.066046
11735406_RNF215	-0.15144	0.030553	0.066055
11746037_RBM19	0.136381	0.030559	0.066064
11734575_HDAC7	-0.10177	0.030562	0.066067
11756570_TMEM50B	0.121696	0.030564	0.06607
11728185_ZSWIM5	0.218294	0.03057	0.066078
11755745_VEGFA	-0.14278	0.030572	0.06608
11739246_ANKFY1	0.169802	0.030583	0.066099
11745120_MYO5C	-0.20197	0.030583	0.066099
11748130_HPCAL4	-0.14644	0.03059	0.06611
11760901_NAPSB	-0.20372	0.030599	0.066125
11753171_PGC	-0.19243	0.0306	0.066125
11750435_IRX5	-0.16848	0.030608	0.06614
11738573_MS4A2	0.203858	0.03062	0.066163
11717397_DDB2	0.114195	0.030621	0.066163
11749764_KMT5B	-0.12777	0.030626	0.06617
11758188_KRT8	0.098049	0.030628	0.066173
11760740_NSMCE4A	-0.1383	0.030634	0.066182
11723139_POP1	-0.13863	0.030656	0.066227
11737500_SLC25A53	-0.19148	0.030659	0.06623
11763752_CCNB1IP1	0.257784	0.030686	0.066285
11757139_SNORA5A	-0.18774	0.030689	0.06629
11744712_ZNF823	0.098094	0.030691	0.066291
11737405_ST8SIA4	0.131282	0.030692	0.066291
11750074_RHBDL2	0.116765	0.030699	0.066302
11744637_OPRL1	-0.11898	0.030701	0.066303
11747561_RPS6KB2	-0.14892	0.030702	0.066303
11760084_LOC254896	-0.18104	0.030708	0.066313
11715269_ALG1L	-0.11228	0.03071	0.066313
11759523_ALKBH7	-0.18825	0.030711	0.066314
11745891_PTPRN	-0.20073	0.030718	0.066323
11744626_RPAIN	0.107897	0.030718	0.066323
11731259_SCUBE1	-0.13019	0.030747	0.066381
11720992_SOX2	-0.16942	0.030748	0.066381
11752758_EIF4B	0.088398	0.030756	0.066395
11740183_ZNF568	0.16088	0.030763	0.066407
11732198_PLAGL2	-0.12569	0.030768	0.066415
11729924_TET3	-0.1193	0.03077	0.066417
11754532_C1orf127	-0.10329	0.030776	0.066426

11719520_SEMA5A	0.198124	0.030784	0.066443
11759596_PCAT4	-0.11855	0.030794	0.066459
11752282_PPFIBP2	0.173038	0.030812	0.066495
11760602_LINC00337	-0.26961	0.030813	0.066495
11728117_ADAMTSL3	0.143351	0.030831	0.06653
11720267_KLC4	-0.13929	0.030832	0.06653
11715868_TINF2	-0.11988	0.03084	0.066542
11731472_TMEM70	0.09462	0.03084	0.066542
11763193_GDPD1	0.124808	0.030841	0.066542
11750757_RPAP2	0.113614	0.030847	0.066552
11726682_MEIS1	0.194476	0.030878	0.066615
11760191_UQCRQ	-0.10946	0.030886	0.066625
11715943_PSMB1	-0.10065	0.030886	0.066625
11727248_MYH3	-0.35095	0.030887	0.066625
11735384_DIP2A	-0.14641	0.030888	0.066626
11744725_GPR17	0.183124	0.0309	0.066648
11763781_EZH1	-0.18102	0.030903	0.06665
11741617_GPRIN3	0.134246	0.030911	0.066665
11740330_ZNF575	-0.11881	0.030914	0.066669
11750000_CLEC7A	0.197007	0.030923	0.066686
11718163_UGT1A1 //	-0.16017	0.030926	0.06669
11728251_GK2	-0.23709	0.030932	0.066699
11761459_SLC6A11	-0.22718	0.03094	0.066715
11763219_GABARAP	-0.1106	0.030951	0.066732
11737509_LDLRAD2	-0.09484	0.030951	0.066732
11757725_NHP2	-0.08772	0.030965	0.066758
11752662_MKS1	-0.14965	0.030973	0.066772
11760787_ARHGAP11B	0.137634	0.030977	0.066776
11726436_ANKRD16	-0.18872	0.030977	0.066776
11737998_LRRC3	-0.0998	0.030994	0.066808
11727812_SRSF1	-0.08905	0.030995	0.066808
11716013_TMEM258	-0.08762	0.030999	0.066813
11719295_AP3B1	0.148015	0.031004	0.066822
11734389_GLYATL2	-0.41215	0.031015	0.066842
11726449_ZNF614	0.110202	0.031021	0.066853
11729897_C11orf87	0.118217	0.031045	0.066902
11716034_BST2	-0.10343	0.031063	0.066937
11752301_ITIH5	-0.15257	0.031069	0.066943
11715866_ATP6V1F	0.11228	0.031069	0.066943
11752098_DHX8	0.14191	0.03107	0.066943
11741835_PDX1	-0.13584	0.031072	0.066944
11730437_PPIL3	-0.10296	0.031079	0.066958
11737539_PVR	-0.11252	0.031086	0.066966
11732414_LRRC8B	-0.31279	0.031088	0.066966
11744810_ZBTB24	0.139805	0.031089	0.066966
11744860_LOC100287	0.158081	0.03109	0.066966
11737136_HOXC6	0.195003	0.031091	0.066966
11760682_DNAJB2	-0.1833	0.031092	0.066966
11736956_ZNF687	-0.15146	0.031093	0.066966
11732549_FBXL14	-0.15756	0.031101	0.066982
11745768_NOTCH2	0.124352	0.031103	0.066983

11736252_MUC21	-0.17519	0.031106	0.066986
11761535_HERC2P9	0.12299	0.03111	0.066992
11720068_APH1B	0.17698	0.031124	0.067018
11749161_SPHK2	-0.14595	0.031135	0.067038
11733471_PLEKHG4B	-0.17555	0.031137	0.067039
11737553_C15orf32	-0.15569	0.031138	0.067039
11757125_SNORA74A	-0.22919	0.03114	0.067041
11759107_TTC39B	0.19757	0.031144	0.067046
11755982_TNPO3	-0.10527	0.031145	0.067046
11741632_TAP2	-0.16802	0.03116	0.067075
11737459_ADARB2	-0.15	0.031163	0.067078
11718618_DNAJA4	-0.13702	0.03117	0.067091
11751599_DPP7	-0.15956	0.031178	0.067105
11735883_DNAH9	-0.20503	0.031182	0.067109
11733245_DTNBP1	-0.1107	0.031182	0.067109
11731993_CLECL1	-0.17708	0.031188	0.067118
11731767_ARSB	0.167414	0.031189	0.067118
11723214_MEF2C	-0.17107	0.031197	0.067131
11763686_FMNL1	-0.22533	0.031215	0.067168
11729922_RENBP	-0.27326	0.03122	0.067175
11745915_GOLGA8A /	0.091604	0.031222	0.067177
11720059_TTYH1	-0.13389	0.031224	0.067179
11741365_IFT22	0.096943	0.031228	0.067184
11725138_S100A12	-0.13734	0.031245	0.067218
11734138_ABHD18	-0.09061	0.031257	0.067238
11751607_CAMKV	-0.16308	0.031258	0.067238
11734591_GTF2IRD2	0.247553	0.031267	0.067255
11732989_HIST3H2BE	-0.15282	0.031271	0.067262
11762587_MAML3	-0.20639	0.031274	0.067263
11748267_ZNF282	0.148439	0.031275	0.067263
11749190_CLGN	-0.13243	0.031297	0.067304
11746959_ABCF3	-0.11023	0.031297	0.067304
11746434_OXCT1	-0.13893	0.031321	0.06735
11750079_ADRM1	-0.11411	0.031321	0.06735
11748295_EIF3G	0.100299	0.031324	0.067354
11726543_TULP2	-0.24421	0.031327	0.067358
11717834_GLG1	-0.11599	0.031333	0.067368
11737932_LILRA1	-0.09887	0.031334	0.067368
11730687_WT1	-0.10688	0.031344	0.067384
11728889_SERF1A //	0.150698	0.031354	0.067403
11722151_EIF2AK3	0.156393	0.031357	0.067405
11750325_LOC151760	-0.18677	0.031358	0.067405
11762508_SLC35A3	-0.16367	0.031359	0.067405
11741798_ZNF304	0.14478	0.03136	0.067405
11746315_TCF7L2	0.089605	0.031361	0.067405
11736787_CATSPER2	-0.12922	0.031366	0.067412
11732253_MED18	0.158709	0.031371	0.067417
11728563_FOXD1	0.120079	0.031371	0.067417
11741865_CSTL1	-0.10303	0.031373	0.067418
11752730_PAX4	-0.19779	0.03138	0.06743
11730599_KRT74	-0.10241	0.031383	0.067433

11721320_LYRM4	0.162213	0.031404	0.067475
11761697_C21orf59	-0.15065	0.031405	0.067475
11738366_MUC1	-0.13928	0.031406	0.067475
11736055_BORCS7	0.111083	0.031418	0.067497
11732093_PEX16	0.1046	0.031449	0.067561
11731933_GABRA2	-0.14136	0.031476	0.067615
11757958_POGZ	-0.11397	0.031494	0.067651
11749502_RELN	0.176096	0.031501	0.067664
11718565_KIAA1147	0.192017	0.031503	0.067665
11755350_XIRP1	-0.16055	0.031515	0.067687
11745602_CLTA	0.089449	0.031518	0.06769
11763318_CSNK1A1	0.116886	0.031523	0.067697
11730352_ZNF423	-0.21876	0.031524	0.067697
11737019_AGAP2	-0.17024	0.031525	0.067697
11750555_NUP214	-0.1121	0.031528	0.0677
11755062_KDELC1	0.136725	0.031538	0.067719
11725073_JADE1	-0.12832	0.031543	0.067727
11729817_IGF1	-0.12668	0.031564	0.067768
11740056_HARBI1	0.121381	0.031568	0.067774
11725557_FBXL18	-0.12401	0.031577	0.067791
11716545_PSMC1	-0.09684	0.031581	0.067796
11729344_BPI	-0.22945	0.031597	0.067829
11718302_HSD11B2	-0.11068	0.031599	0.06783
11731683_RIF1	-0.10774	0.031601	0.067831
11720826_JAG1	0.234322	0.031602	0.067831
11739859_PACSIN1	-0.17425	0.031604	0.067831
11737054_PHRF1	-0.15963	0.031606	0.067833
11745523_MYBPC1	-0.11666	0.03161	0.067838
11721617_THEMIS2	-0.15323	0.031615	0.067845
11719913_POMT1	0.149607	0.031616	0.067845
11718381_PSD4	-0.12165	0.03163	0.067873
11739425_DPYSL5	-0.12546	0.031646	0.067904
11718458_STK11IP	-0.09803	0.031649	0.067907
11730441_FAM155A	-0.19678	0.031651	0.067909
11723546_PLD1	0.138519	0.031654	0.067912
11720650_DUSP23	-0.09001	0.031674	0.067951
11737415_BPIFB4	-0.13766	0.031679	0.06796
11732578_NCCRP1	-0.11938	0.031694	0.067988
11753731_SNCA	0.117776	0.031695	0.067988
11732990_HIST3H2BB	-0.13406	0.031707	0.06801
11741709_PRCD	-0.10883	0.031714	0.068023
11755356_PSMF1	0.097226	0.031726	0.068045
11762389_USP38	0.28162	0.031727	0.068045
11724001_LNP1	-0.17527	0.031736	0.06806
11715933_PPP1R7	-0.14838	0.031758	0.068105
11752359_DDX24	0.091983	0.031764	0.068111
11727159_LRP5	0.130138	0.031765	0.068111
11737652_FAM9B	0.323602	0.031765	0.068111
11724677_TRMT5	-0.10481	0.031769	0.068116
11740834_DST	-0.15274	0.031809	0.068199
11736817_KIRREL	-0.10369	0.031818	0.068215

11715447_TALDO1	-0.09459	0.031819	0.068215
11757466_FAM177A1	-0.12183	0.031821	0.068217
11722343_ZNF558	0.153712	0.031825	0.068223
11759042_VKORC1L1	-0.12513	0.031829	0.068228
11760672_AGGF1	0.125795	0.031844	0.068256
11739465_ALKBH4	-0.12998	0.031854	0.068273
11745446_LMO3	0.123847	0.031855	0.068273
11721695_DUSP2	0.13039	0.031863	0.068287
11719380_THOC7	0.092915	0.031875	0.06831
11734857_ERO1B	-0.1599	0.031895	0.068351
11727463_ZXDC	-0.09163	0.031918	0.068397
11760818_CDKL3	0.131172	0.031923	0.068405
11736264_FBXO44	-0.22482	0.031933	0.068422
11751692_GSTO2	-0.15332	0.031936	0.068428
11717987_MRPL40	-0.09546	0.03194	0.068431
11729479_CLEC12A	-0.18193	0.031976	0.068506
11755546_LFNG	-0.1379	0.032003	0.068562
11754600_SHC3	-0.17673	0.032045	0.068647
11735624_MADD	0.112926	0.032066	0.068689
11719537_CPXM2	-0.13169	0.032072	0.068697
11741561_ADGRF5	0.219816	0.032072	0.068697
11745038_FRAT1	-0.12419	0.032073	0.068697
11726926_CT47A1 //	-0.22019	0.032098	0.068747
11735771_WFDC8	0.157376	0.032106	0.068761
11751415_TSC22D3	0.131266	0.032133	0.068814
11731324_LSS	-0.1688	0.032135	0.068816
11748458_ATXN2L	-0.124	0.032137	0.068818
11719819_EMC10	0.111599	0.032145	0.06883
11733603_SLC52A1	-0.17209	0.032145	0.06883
11731037_FIBCD1	-0.17987	0.032166	0.068871
11732612_CELF3	-0.17218	0.032171	0.068876
11756359_ADRA2C	-0.17026	0.032171	0.068876
11761833_PI16	-0.11126	0.032174	0.068878
11729680_KHK	-0.24964	0.032179	0.068887
11721703_TNRC18	-0.09311	0.032202	0.068933
11752478_SLC6A13	-0.16167	0.03221	0.068947
11763469_AZIN2	-0.14107	0.032217	0.068956
11727514_TTC9	0.138306	0.032217	0.068956
11735305_FLCN	0.108731	0.032219	0.068957
11761606_CDH22	-0.15928	0.032263	0.069048
11741419_KCNQ1	-0.11284	0.032269	0.069057
11753004_CKB	0.099628	0.032279	0.069073
11740861_SNX17	0.105038	0.032279	0.069073
11744620_ZNF821	0.245763	0.032283	0.069076
11751779_VWF	-0.12668	0.032283	0.069076
11729491_PPFIA4	-0.11843	0.032287	0.069082
11757758_RPS10 ///	0.089053	0.032291	0.069086
11750456_RHCE ///	-0.16331	0.032295	0.069093
11756048_LCN10	-0.17926	0.032298	0.069096
11743912_MYOC	-0.14469	0.032308	0.069114
11758790_STMN2	-0.23675	0.032309	0.069115

11723921_KLHL41	0.182254	0.032325	0.069145
11762928_DNAJC18 /	-0.2502	0.032342	0.069178
11752174_MST1R	-0.16993	0.032344	0.06918
11757161_SNORA30	-0.17326	0.032351	0.069191
11746585_NFATC1	-0.20294	0.032358	0.069203
11738708_OR10K1	-0.25146	0.032367	0.06922
200009_PMGDI2	0.094111	0.032377	0.069238
11738556_KIR2DL4	-0.17829	0.032386	0.069254
11723075_BCL9L	0.108966	0.032389	0.069255
11745149_C2CD5	-0.0989	0.032389	0.069255
11757656_RPAIN	0.098721	0.032409	0.069292
11736881_FCRL1	-0.12417	0.03241	0.069292
11722112_ADRA2A	-0.16375	0.032411	0.069292
11745155_NCL	-0.0941	0.032414	0.069293
11756271_FANK1	0.138902	0.032414	0.069293
11752246_STAB1	-0.20418	0.032419	0.069299
11750802_LIMK2	0.166512	0.032448	0.069359
11742907_TTLL5	0.111791	0.032459	0.069379
11763174_PGM3	-0.11017	0.032476	0.069414
11750269_LINC00612	-0.14152	0.032492	0.069444
11724778_PGM2L1	0.120485	0.032496	0.069449
11755571_MAP7D1	-0.09405	0.032507	0.06947
11741690_SUMF2	0.100805	0.03251	0.069473
11759784_SVIL	-0.20317	0.032513	0.069477
11747568_FUZ	-0.20905	0.03252	0.069488
11760513_PRMT1	-0.24426	0.032524	0.069495
11762387_MYO3B	-0.26506	0.032532	0.069509
11736293_SPATS2L	-0.09904	0.032544	0.069531
11727396_CTNNA1	0.107385	0.03255	0.069541
11763419_AP5S1	-0.09069	0.032559	0.069556
11742879_CRYZL1	0.120683	0.03256	0.069556
11727509_GTPBP10 /	0.169536	0.032572	0.069578
11757082_TMEM167A	0.089676	0.032573	0.069578
11737599_CXorf67	-0.11721	0.032576	0.069581
11715982_DUS1L	0.107515	0.032581	0.069588
11754727_VPS39	-0.12348	0.032606	0.069638
11721796_ALDH9A1	0.096822	0.03261	0.069644
11750756_SBNO2	0.10872	0.032616	0.069655
11755439_UBAC2	-0.09307	0.032635	0.069693
11716307_UQCR11	0.135993	0.032637	0.069693
11722671_AKAP11	0.105362	0.032645	0.069706
11741676_MANEAL	0.215119	0.032661	0.069739
11726445_KCNK3	0.180497	0.032666	0.069746
11758314_NDUFAF2	0.098435	0.032668	0.069746
11732682_ZNF471	0.149273	0.032672	0.069753
11757307_CTSD	-0.10307	0.032676	0.069756
11747192_NFIC	-0.22202	0.032676	0.069756
11760550_FBXW4P1	0.162055	0.032687	0.069773
11760551_FBXW4P1	0.162055	0.032687	0.069773
11736653_CFAP57	-0.17816	0.032699	0.069794
11723153_GON4L	-0.14642	0.0327	0.069794

11761483_CLK3	-0.1094	0.032702	0.069796
11738732_OR2G6	-0.21263	0.032708	0.069805
11737159_SLC9A2	-0.13397	0.032711	0.069808
11753144_KLRAP1	-0.19173	0.032718	0.069821
11756595_ZNF142	0.128909	0.032721	0.069823
11734630_DSG4	-0.12959	0.032744	0.069869
11743431_LYRM9	-0.15967	0.032754	0.069888
11733281_KPRP	-0.15643	0.032758	0.069893
11746132_MYO10	-0.10107	0.032762	0.069898
11718485_EI24	0.105165	0.032785	0.069946
11758932_CHAF1B	-0.16236	0.032792	0.069957
11751283_ADPRH	-0.20497	0.032808	0.069988
11736668_CLEC4E	-0.16151	0.032815	0.069999
11742766_DENND5A	-0.14165	0.032833	0.070035
11732483_PEX13	0.112028	0.032856	0.070081
11751298_TBL2	0.088378	0.032869	0.070106
11745031_PPP1R2P9	-0.2112	0.032874	0.070114
11751056_ZBP1	-0.3208	0.032882	0.070128
11744262_RNF167	-0.10838	0.032894	0.07015
11722847_PPP3CA	0.099242	0.032901	0.070162
11724966_BLOC1S1-R	0.130194	0.032903	0.070164
11763713_AAR2	0.146502	0.03292	0.070194
11723284_REEP6	-0.16111	0.03292	0.070194
11734814_PDE1A	-0.19092	0.032928	0.070207
11739417_IRGQ	0.202735	0.03293	0.070207
11749275_SKAP2	0.133341	0.032931	0.070207
11761696_MRPL48	-0.14584	0.032932	0.070207
11727107_DROSHA	-0.09997	0.032949	0.070241
11734348_NEUROG1	-0.14423	0.032952	0.070243
11732460_PLEKHS1	0.246005	0.032962	0.070261
11725366_IER5L	-0.09243	0.032968	0.070268
11736567_LEF1	-0.14566	0.032968	0.070268
11731073_ZNF587	0.142807	0.032972	0.070273
11763060_NPSR1-AS1	-0.1211	0.03298	0.070287
11747108_RPGRIP1L	0.12669	0.032983	0.070291
11739630_RAG2	-0.09375	0.032995	0.070313
11738280_KLK12	-0.13704	0.032997	0.070314
11732684_ABCA12	0.41515	0.033008	0.070336
11746943_SH2D6	-0.22542	0.033018	0.070353
11746038_RBM19	0.118006	0.033037	0.07039
11758911_DYRK2	-0.18065	0.033042	0.070399
11721398_DOT1L	0.148403	0.033051	0.070413
11723000_MYL7	-0.15969	0.033057	0.070423
11746628_RIC3	-0.18846	0.03306	0.070426
11736143_ENPP4	-0.14129	0.033068	0.070442
11737808_CDK18	0.098016	0.033085	0.070472
11754282_GYG2	0.148898	0.033085	0.070472
11755096_ANKRD16	0.151526	0.033128	0.070561
11736824_C10orf95	-0.09494	0.03313	0.070561
11755054_RUNX3	-0.1027	0.033138	0.070574
11756500_DPH5	-0.11624	0.033159	0.070617

11739290_KMT2A	0.087959	0.033162	0.070619
11763352_ZNF576	-0.13502	0.033172	0.070637
11725732_LOC102724	0.105313	0.033173	0.070637
11748277_ASCC1	0.110438	0.033179	0.070647
11730922_NHLRC4	-0.11509	0.033188	0.070664
11720302_SLA	-0.14193	0.033195	0.070673
11743408_RBM25	0.110256	0.033196	0.070673
11745535_CRB1	0.103054	0.033199	0.070677
11757699_PSMD4	-0.10175	0.033204	0.070685
11756697_OSBPL5	-0.11287	0.033209	0.070692
11725324_TSPAN31	0.098062	0.033212	0.070695
11727063_UCK2	-0.10816	0.03323	0.070731
11718229_R3HDM4	0.112094	0.033234	0.070735
11736033_VAMP1	-0.12048	0.033237	0.070739
11723758_ZFR	0.089005	0.033256	0.070777
11743717_SMTNL1	-0.22115	0.03326	0.070781
11753172_GRB7	0.103239	0.033262	0.070783
11734987_ZNF418	0.183975	0.033266	0.070789
11734182_CCDC9	-0.15167	0.033271	0.070798
11747352_ZSCAN32	0.11973	0.033278	0.070805
11726460_FAM117B	-0.14721	0.033278	0.070805
11739479_NINL	-0.21419	0.033292	0.070826
11730564_ZC2HC1B	-0.13325	0.033292	0.070826
11746222_TTBK2	0.097883	0.033292	0.070826
11734018_URB1	-0.14585	0.033295	0.070826
11743622_TM9SF2	0.093729	0.033295	0.070826
11716930_DGCR2	0.154728	0.033311	0.070857
11759022_ELK3	0.085494	0.033319	0.070868
11743392_MATR3	-0.12287	0.033319	0.070868
11742973_ACAT1	0.107092	0.033347	0.070924
11721765_ADRBK2	-0.22744	0.03336	0.070949
11748224_AMDHD2	-0.15909	0.033368	0.070963
11753847_KRTAP21-3	-0.09778	0.033394	0.071011
11753640_MUC1	-0.10491	0.033394	0.071011
11732887_VENTX	-0.17122	0.033398	0.071017
11744441_BASP1	-0.18652	0.033405	0.071029
11757525_BICD2	0.106795	0.033407	0.071031
11725935_CAMK2A	-0.16034	0.033423	0.071062
11749726_NR4A1	-0.09371	0.03343	0.071073
11761807_FER	-0.13631	0.033432	0.071073
11728337_LINGO1	-0.18329	0.033441	0.071091
11760356_PMPCB	0.274699	0.033446	0.071097
11759475_CYP2C19	-0.26164	0.033466	0.071136
11744326_RPL37 ///	-0.09262	0.033467	0.071136
11753351_SHISA6	-0.1744	0.033469	0.071137
11729303_UBE3B	0.157115	0.033473	0.071143
11751286_SLC35A5	-0.10635	0.033475	0.071144
11763626_MLLT1	-0.36	0.033496	0.071186
11744248_LRRC4C	0.190714	0.033516	0.071225
11753447_SGCZ	-0.14418	0.033519	0.071228
11729702_DBN1	-0.0923	0.033547	0.071285

11737166_ANKRD34B	-0.14103	0.033552	0.07129
11729382_AK5	0.161926	0.033553	0.07129
11760699_LSAMP	0.116788	0.033584	0.071354
11749675_BCL11A	-0.11948	0.033585	0.071354
11738532_KLHL4	-0.10844	0.033588	0.071355
11734866_CENPJ	-0.30102	0.033594	0.071366
11716115_ZFP36L1	0.093749	0.033599	0.071372
11730665_FOXP1	0.103518	0.033608	0.071388
11748883_FAM217B	-0.183	0.033613	0.071397
11742692_APRT	-0.10762	0.033661	0.071496
11729430_CCDC121	0.248396	0.033663	0.071497
11732511_C10orf25	-0.18229	0.033672	0.071513
11753527_RAB23	0.154819	0.033676	0.071517
11747879_CYP2C8	-0.18973	0.033679	0.071522
11751397_COMMD9	-0.11572	0.033682	0.071525
11751843_CRIP3 ///	-0.28228	0.0337	0.07156
11718207_COQ6	0.143494	0.033713	0.071583
11763218_DEFB121	-0.11892	0.033731	0.071619
11755992_ICAM2	-0.13662	0.033744	0.071643
11723287_TYROBP	-0.14222	0.033754	0.071662
11741447_RNASE4	-0.2793	0.03376	0.071672
11730602_APOBEC3F	0.255662	0.03377	0.07169
11745321_ZFP28	0.22854	0.033791	0.071732
11747997_SNX17	0.134535	0.033815	0.071779
11755377_NARF	-0.09131	0.033822	0.071792
11729778_CDH17	-0.11591	0.03383	0.071806
11734663_TRA2B	-0.14127	0.033835	0.071811
11754182_IL2RG	0.222025	0.033845	0.071831
11751588_UCHL5	-0.102	0.033847	0.071831
11735181_DLX2	-0.08491	0.033851	0.071836
11723794_SLC29A2	-0.12249	0.033862	0.071857
11734007_NDOR1	-0.14576	0.033872	0.071875
11719231_PTPRS	-0.13098	0.033886	0.071901
11729253_GEMIN8	0.190373	0.033888	0.071904
11761046_RNF34	-0.17398	0.033901	0.071928
11763071_NPSR1-AS1	0.276554	0.033907	0.071938
11758014_TGFBR1	-0.09164	0.033911	0.071942
11752875_SATB2-AS1	-0.1044	0.033929	0.071976
11744227_MACROD1	-0.10457	0.033934	0.071986
11747160_RUBCN	-0.15636	0.033941	0.071995
11724611_DYRK2	0.122735	0.033942	0.071995
11739998_SMARCA2	0.097127	0.033944	0.071997
11750957_NOX4	0.229595	0.033947	0.072001
11754077_RAB11B	-0.16283	0.033954	0.072012
11733378_DCAF4L1	0.319153	0.033958	0.072016
11726358_KMT2E	0.132184	0.03396	0.072017
11758406_KIAA1644	-0.21046	0.033961	0.072017
11740883_RWDD3 ///	0.122934	0.033965	0.072021
11729148_SMDT1	0.087995	0.033966	0.072021
11716375_PRKCZ	0.101994	0.033994	0.072078
11718226_SMIM12	0.123462	0.034025	0.07214

11758901_RIPK2	0.228455	0.034029	0.072144
11729346_ZNF513	0.096073	0.03403	0.072144
11716683_SLC39A9	-0.1319	0.034032	0.072145
11727714_KCNJ12	0.192012	0.034033	0.072145
11718031_ATP6V0A1	0.104619	0.034045	0.072166
11727951_DHX33	0.130243	0.034051	0.072177
11748982_CYP2S1	-0.17347	0.03406	0.072193
11747541_ARMC4	0.28958	0.034066	0.072202
11725818_PAQR3	0.107761	0.034088	0.072245
11739076_NFE2L1	-0.23899	0.034092	0.07225
11750115_PLXNB3	-0.14929	0.034099	0.072263
11756524_RBBP9	-0.15149	0.034103	0.072267
11763786_NARFL	-0.15468	0.034104	0.072267
11763200_FBXW8	-0.152	0.034118	0.072293
11723026_FAM26F	-0.11897	0.034133	0.072322
11735440_RBM11	-0.18394	0.034144	0.072343
11744313_ST20	0.106599	0.034153	0.072358
11759900_TMEM144	-0.22736	0.034157	0.072364
11732820_TMEM217	-0.14834	0.034159	0.072366
11746735_IFT140	-0.10899	0.034161	0.072366
11725996_DDX25	-0.12002	0.034169	0.072379
11742286_PAX3	-0.13727	0.034178	0.072396
11760908_MYL6	-0.21331	0.034193	0.072425
11730574_ZNF470	0.112638	0.034195	0.072425
11729318_UACA	-0.26265	0.0342	0.072433
11750392_IL17RE	-0.17482	0.034202	0.072435
11729873_USP19	-0.12733	0.034207	0.072441
11718816_PLCD1	-0.15899	0.03421	0.072445
11732122_PMPCB	-0.12062	0.034213	0.072449
11758787_FUCA2	-0.18255	0.03423	0.072481
11731700_C12orf66	-0.13457	0.034239	0.072497
11741021_KLK3	-0.12499	0.034265	0.072549
11750249_FAM221B	0.170178	0.034277	0.072571
11716503_CRMP1	-0.12436	0.034279	0.072571
11716425_PCDHGA1 /	-0.1204	0.034284	0.07258
11740699_ENTPD8	-0.1298	0.034291	0.072591
11740547_KCNJ14	-0.12675	0.03431	0.072628
11717200_DPM2	-0.09104	0.034314	0.072633
11717748_MCTS1	-0.09675	0.034316	0.072635
11733478_OLIG3	-0.12536	0.034319	0.072637
11755412_IKBKB	-0.14993	0.034326	0.072649
11742883_ST6GALNAC	-0.13742	0.034337	0.072669
11728820_HAUS2	-0.11047	0.034341	0.072675
11728993_CCDC70	-0.10977	0.034386	0.072767
11750716_RBM47	-0.15328	0.034394	0.07278
11728616_CARD6	0.13949	0.034399	0.072787
11730226_QRSL1	0.15992	0.034401	0.072788
11751358_MEI1	-0.18015	0.034402	0.072788
11754072_TCL1A	-0.10264	0.034445	0.072877
11726990_EIF2AK1	0.150998	0.034447	0.072877
11727510_UBN2	-0.17079	0.034449	0.072878

11748134_SLC2A11	-0.14471	0.034481	0.072943
11716715_CTSB	-0.08578	0.034495	0.072968
11738789_HIST1H4G	-0.18423	0.034501	0.072977
11736924_DLGAP1	0.188203	0.034523	0.073023
11729627_DCAF7	0.094316	0.03454	0.073055
11720369_RANBP6	0.112538	0.034541	0.073055
11751449_CEP41	-0.16141	0.034548	0.073065
11760895_SLC29A1	-0.22237	0.034553	0.073073
11757367_HSPA6 ///	-0.16327	0.034562	0.073087
11745014_WT1 /// W	-0.11479	0.034565	0.073092
11755907_ATP13A1	0.105354	0.034576	0.073109
11750121_HDAC4	-0.10909	0.034576	0.073109
11754111_PNOC	-0.20139	0.034578	0.07311
11758704_COMMD5	-0.09401	0.034581	0.073113
11718036_PPIL1	-0.11691	0.034583	0.073114
11718333_SCAMP4	-0.12072	0.034604	0.073151
11731981_ADTRP	0.104562	0.034604	0.073151
11761076_ACOT8	0.147364	0.034605	0.073151
11742966_MTERF4	0.112464	0.034616	0.073171
11718214_DDA1	-0.11417	0.034637	0.073212
11715633_MAF1	-0.0967	0.034651	0.073238
11731623_AGXT	-0.11464	0.034653	0.073239
11759611_SMIM11A /	0.186395	0.03466	0.073252
11761446_STAU2	-0.17602	0.034662	0.073252
11730357_KCNE4	0.176541	0.03467	0.073265
11717199_POLR1D	0.116992	0.034677	0.073278
11728669_UBE2H	0.108956	0.034679	0.073278
11718170_KMT2B	0.088571	0.034686	0.07329
11736096_GGA1	-0.12851	0.034695	0.073307
11724517_GUF1	0.089967	0.034698	0.07331
11736772_SCRN1	-0.09135	0.0347	0.07331
11735800_P2RX3	-0.16729	0.034729	0.073365
11747203_CLN3	-0.10492	0.034729	0.073365
11745683_HTR3E	-0.13146	0.034732	0.073368
11747732_ADCK3	-0.13118	0.034737	0.073376
11764170_DNAJC10	-0.14966	0.034745	0.07339
11756522_SCARB1	-0.10791	0.034759	0.073417
11730073_CNTN3	0.149859	0.034761	0.073418
11718068_MYO1E	-0.1131	0.034763	0.073418
11719079_AAGAB	0.127834	0.034783	0.073456
11719622_GTPBP1	-0.18773	0.03479	0.073468
11735816_PCDHGA1 /	-0.10579	0.034792	0.073469
11726204_CCNJL	0.098916	0.034797	0.073478
11755912_YEATS2	-0.1024	0.034811	0.073504
11755025_FAM193B	0.107886	0.034812	0.073504
11758320_GATSL2	-0.1672	0.034823	0.073522
11735504_NLRC4	0.119749	0.034829	0.073532
11724711_DMXL1	0.145765	0.034839	0.073549
11740435_GRPR	0.206591	0.034847	0.073565
11717096_HSPH1	0.09222	0.03485	0.073567
11739273_PAK2	0.127378	0.034851	0.073567

11746749_ZBBX	-0.17127	0.03486	0.073582
11726154_FAM19A5	-0.11954	0.034862	0.073582
11750842_KY	-0.0981	0.034872	0.0736
11755063_NKAP	-0.10734	0.034899	0.073655
11725270_SLC12A3	0.194721	0.034904	0.073661
11730065_TIGD7	-0.13275	0.034924	0.073702
11740386_SHROOM4	-0.21602	0.034942	0.073735
11753736_MT1JP ///	-0.1127	0.034945	0.073738
11720365_COL6A2	-0.08833	0.03495	0.073746
11729570_MAP4K1	-0.17777	0.034952	0.073748
11760649_MKNK1	-0.10475	0.034957	0.073754
11737061_MTMR3	0.143167	0.034958	0.073754
11753822_KCNMB2	-0.16187	0.034967	0.07377
11726074_ARHGEF7	-0.09441	0.034997	0.073829
11755247_DAND5	-0.23378	0.035013	0.07386
11728195_TRO	-0.2107	0.035023	0.073879
11733545_NWD1	-0.18413	0.035025	0.07388
11732547_KALRN	0.131646	0.035038	0.073904
11754810_ITGB5	0.114724	0.035045	0.073916
11759044_VKORC1L1	0.088433	0.035063	0.073947
11761728_SMARCE1	-0.21781	0.035063	0.073947
11727853_NUP50	-0.09817	0.035072	0.073962
11733173_ARHGEF39	-0.1687	0.035079	0.073973
11760304_RBBP6	-0.10497	0.035087	0.073988
11759411_MIF	0.093068	0.03512	0.074053
11721075_MTCH2	-0.10092	0.035123	0.074057
11722721_CENPV	0.149555	0.035131	0.07407
11741232_GGPS1	-0.10575	0.035151	0.074106
11723897_TESK2	-0.15497	0.035151	0.074106
11726546_PITPNM3	-0.12803	0.035158	0.074119
11742627_OR4N5	-0.1952	0.035181	0.074163
11753843_NDUFA3	0.091843	0.035184	0.074166
11760544_FAM160A2	-0.12102	0.035189	0.074173
11752141_EEPD1	-0.16986	0.035192	0.074177
11723066_NAIF1	0.114697	0.035202	0.074195
11742827_PHF3	-0.09507	0.035217	0.074224
11739394_MFHAS1	0.102566	0.035224	0.074235
11731803_COLCA1	-0.16259	0.035227	0.074237
11754496_FMO1	-0.08902	0.035236	0.074254
11752908_TCEA1	-0.12433	0.035241	0.074261
11752299_HERC1	0.117707	0.035277	0.074333
11729992_DQX1	-0.1482	0.03528	0.074334
11729209_PDYN	-0.19654	0.03528	0.074334
11763915_MAGED2	-0.12836	0.035285	0.074341
11743266_RPL34	-0.13693	0.035293	0.074355
11729541_CAMKK2	-0.13254	0.035312	0.07439
11755847_SEC14L2	0.088152	0.035313	0.07439
11726732_AGGF1	-0.12451	0.035315	0.07439
11743800_DNAH2	0.257503	0.035325	0.074409
11744242_ANKRD29	0.27006	0.035335	0.074428
11731447_SNCA	0.11379	0.035352	0.074459

11745895_UPF2	0.121211	0.035358	0.074468
11761345_ZNF812P	0.098349	0.035371	0.074493
11762260_MST1P2	-0.27497	0.035388	0.074521
11758528_PCDHB6	0.088677	0.035388	0.074521
11727735_TMEM91	-0.11782	0.035389	0.074521
11730407_PSMA8	-0.14428	0.035391	0.074522
11764053_RBPJ	-0.13513	0.035392	0.074522
11740061_CSK	0.108369	0.03541	0.074555
11759144_ZNF710	-0.13058	0.035413	0.074558
11745984_SRRM1	0.099803	0.035422	0.074574
11726720_MPG	-0.08506	0.035435	0.074596
11761301_B4GALNT1	-0.14996	0.035435	0.074596
11716776_EPS8L2	-0.1348	0.035437	0.074597
11737423_NLRP1	-0.15459	0.035455	0.07463
11762375_FCRL5	-0.16936	0.035459	0.074636
11720286_TRAK1	-0.09728	0.035468	0.074652
11739636_CDK12	-0.18028	0.035471	0.074656
11762624_VRK3	-0.1099	0.035473	0.074656
11728973_HES5	-0.2338	0.035476	0.074659
11757646_UBL5	-0.13381	0.035477	0.074659
11761959_TRAC ///	0.182948	0.035503	0.074709
11730748_PAIP2	0.089162	0.035504	0.07471
11763526_CHRNA3	-0.17407	0.035508	0.074714
11751801_CCDC107	0.12562	0.03551	0.074714
11751938_EVC2	-0.15043	0.035515	0.074722
11740591_DTNB	-0.08721	0.035517	0.074724
11749218_TMPRSS4	-0.12698	0.035533	0.074754
11718506_TFF3	-0.09286	0.035543	0.074773
11763410_GLG1	0.240876	0.035549	0.074782
11716501_CRMP1	-0.15576	0.035555	0.074791
11758972_ZNF354C	-0.12921	0.03556	0.074797
11746116_PINX1	-0.08944	0.035571	0.074818
11757210_ASMTL-AS1	-0.11423	0.035582	0.074837
11726906_PIGO	-0.10438	0.035599	0.074869
11750852_PRRC2A	-0.13982	0.0356	0.074869
11722414_SLC7A1	0.203236	0.035603	0.074873
11726008_DPY19L2	0.135328	0.035613	0.074889
11715745_KIAA1191	0.087981	0.035616	0.074894
11731387_TTC22	-0.14299	0.035626	0.074911
11732810_HHIPL1	-0.10589	0.035629	0.074914
11749706_CCDC138	-0.10555	0.035631	0.074916
11744939_C11orf74	-0.10319	0.03565	0.074952
11730268_NIPBL	0.186738	0.035665	0.074979
11741725_SCAF4	0.08692	0.035676	0.075001
11733666_GPATCH2L	0.119195	0.035678	0.075002
11764083_SLC6A11	-0.18085	0.035694	0.075032
11731890_ADAMTS16	-0.14333	0.035714	0.075069
11755517_ATRX	-0.12617	0.035717	0.075072
11722234_FBXO2	-0.14846	0.035729	0.075095
11719445_UGGT2	0.116963	0.035736	0.075106
11729933_ZNF135	-0.12806	0.035739	0.075109

11741996_VN1R10P	-0.1837	0.035763	0.075158
11724617_MOCS1	-0.14608	0.035769	0.075166
11754586_WDR82	0.111368	0.035773	0.075172
11727507_GTPBP10 /	0.178166	0.035785	0.075193
11731471_AKT2	-0.16308	0.035799	0.07522
11756664_CD19	-0.18679	0.035812	0.075242
11758171_KTN1	0.125758	0.035813	0.075242
11744389_ARFRP1	-0.10257	0.035819	0.075249
11738018_ARV1	0.100094	0.035819	0.075249
11749463_SEMA5B	-0.14867	0.035834	0.075276
11715326_VSTM5	-0.18847	0.035848	0.075303
11755527_PRPSAP2	-0.13849	0.035855	0.075315
11748996_PUM2	0.0989	0.035857	0.075316
11736065_P2RX7	-0.15077	0.035859	0.075316
11756555_SCAMP5	-0.09173	0.035864	0.075323
11722333_FNDC5	-0.11146	0.03587	0.075334
11761523_ATP2C2	-0.1215	0.035872	0.075334
11736628_ARHGEF34F	-0.12594	0.03588	0.075348
11764047_CCIN	-0.18164	0.035896	0.075379
11743399_STARD3NL	0.106745	0.035907	0.075393
11747407_AAGAB	0.110766	0.035907	0.075393
11718775_SRRM1	0.104177	0.035908	0.075393
11763520_PIF1	-0.25178	0.035914	0.075402
11750070_ATG2A	-0.13352	0.035927	0.075426
11756923_LOC102724	-0.14003	0.035929	0.075427
11754346_DBNDD1	0.119975	0.035939	0.075446
11717516_EFHD1	-0.15996	0.035945	0.075454
11749862_APOL3	-0.10947	0.035946	0.075454
11723593_TNK2	-0.09757	0.035956	0.075472
11734535_SPATA31A1	0.099378	0.035959	0.075475
11724295_TSPAN18	-0.13965	0.035961	0.075475
11761253_ZNF638	0.192306	0.035984	0.075518
11745686_SCLT1	-0.17816	0.035985	0.075518
11721003_HEXIM1	0.087296	0.036013	0.075575
11734496_C6orf222	-0.11936	0.036018	0.075583
11745391_MRPL3	-0.09105	0.036021	0.075586
11733709_TMEM189-U	0.095329	0.036032	0.075605
11748119_RNF44	-0.09427	0.036043	0.075625
11745135_TMEM239	-0.13669	0.036046	0.075629
11735235_SNX7	-0.09033	0.036064	0.075663
11749735_SPATA5L1	0.117641	0.036082	0.075697
11760588_ITSN1	0.152535	0.036086	0.075702
11739628_CSF1	-0.10491	0.036095	0.075719
11739154_FAM129A	-0.15299	0.036098	0.075721
11721025_TRAPPC6A	-0.16634	0.036115	0.075754
11761694_LINC01166	-0.13779	0.036118	0.075755
11754286_BCL7B	0.125058	0.036119	0.075755
11733443_LCE2B	-0.12314	0.036145	0.075807
11737364_BZRAP1	-0.183	0.036148	0.075809
11741923_THRB	0.187123	0.036159	0.075829
11734071_MORN1	-0.14264	0.036166	0.075837

11734909_WDR26	0.090156	0.036166	0.075837
11740662_TEX12	-0.18632	0.036174	0.075849
11748194_POLL	-0.22671	0.036175	0.075849
11720651_DUSP23	-0.11347	0.03618	0.075857
11721598_EFS	0.119005	0.036182	0.075858
11722681_RBP7	-0.16376	0.036185	0.075861
11757342_DCTD	0.093933	0.036187	0.075861
11743876_CETN1	-0.15361	0.036189	0.075863
11745206_RAD23B	-0.1157	0.036203	0.07589
11729439_CAPSL	0.105215	0.036257	0.076
11762287_TRBC2 ///	-0.14574	0.036269	0.07602
11726578_PEX14	-0.0877	0.036284	0.076049
11758240_ARMC6	-0.09579	0.036299	0.076075
11740506_FRRS1L	-0.25214	0.0363	0.076075
11759014_ZNF281	0.103502	0.036301	0.076075
11743612_ZNF830	0.122977	0.036305	0.07608
11736819_RPE65	-0.17573	0.036307	0.076081
11744873_KLKP1	-0.17435	0.03633	0.076125
11758939_MOB1A	-0.09897	0.036336	0.076136
11763682_ABCB9	-0.13984	0.03636	0.076181
11729444_SULT2B1	-0.21398	0.036373	0.076205
11717205_RSAD1	-0.10659	0.036378	0.076214
11751259_CACNA2D1	0.236372	0.036381	0.076214
11743849_SLC2A5	-0.15236	0.036382	0.076214
11726886_SLC45A1	-0.11852	0.036383	0.076214
11749966_CCDC78	-0.14002	0.036389	0.076223
11724445_LYZL1 ///	-0.09843	0.036393	0.076227
11750023_H1FOO	-0.1407	0.036396	0.07623
11741730_ANO6	-0.12588	0.036402	0.07624
11735867_C5orf46	0.135672	0.036405	0.076244
11749880_PGAP2	-0.17164	0.036407	0.076244
11760567_RAPGEF6	0.174507	0.036408	0.076244
11763903_SNORA50A	0.174934	0.036439	0.076304
11735342_TBXA2R	-0.12568	0.03644	0.076304
11741046_SETD3	0.117879	0.036444	0.07631
11761576_EIF3G	-0.12237	0.036446	0.076311
11741799_BCOR	0.101987	0.036464	0.076344
11763707_CCDC93	0.188679	0.036477	0.076369
11741422_ADPRHL1	-0.10677	0.036491	0.076395
11717337_ITGB2	0.144992	0.036497	0.076404
11750349_GRID1	-0.14199	0.036515	0.076439
11751874_ATM	0.171478	0.036533	0.076471
11721078_PNISR	0.092023	0.036541	0.076485
11726683_CDIP1	-0.10241	0.036549	0.076499
11721744_NID2	-0.20602	0.036562	0.076524
11758566_BCS1L	-0.10591	0.036576	0.076549
11748294_CHRD	-0.19019	0.036583	0.076562
11719050_BTN2A1	0.105321	0.036609	0.07661
11731954_CEACAM19	-0.11047	0.03661	0.07661
11751264_MAN2A2	-0.09865	0.036642	0.076673
11747976_EPB41L1	-0.12611	0.036643	0.076673

11760489_TMED3	-0.13273	0.036652	0.076688
11739796_RHBDF2	-0.09637	0.036657	0.076691
11720707_BCCIP	0.088655	0.036657	0.076691
11735522_C9orf106	-0.13338	0.036658	0.076691
11732611_LVRN	0.302485	0.03666	0.076693
11726112_METTL25	0.158725	0.036666	0.076702
11748173_RIMS1	0.253231	0.036678	0.076724
11755410_KLC2	0.104652	0.036698	0.076761
11750488_GRAP2	-0.10779	0.036711	0.076785
11761826_LOC101929	0.145313	0.036712	0.076785
11762161_AKAP8	0.14054	0.036739	0.076838
11741464_UPK2	-0.12433	0.036749	0.076856
11726020_EEPD1	-0.13179	0.036752	0.076856
11739312_HUS1	0.114225	0.036752	0.076856
11748969_MCOLN1	-0.17147	0.036758	0.076864
11715103_TNFAIP8L1	-0.17412	0.036764	0.076873
11752421_ANKLE1	-0.17994	0.036779	0.076902
11743971_KATNB1	-0.12813	0.036785	0.076912
11758039_SDS	-0.19683	0.036813	0.076967
11753133_MCM8	-0.12293	0.03682	0.076977
11727165_CEP295	0.110325	0.036826	0.076988
11762936_AQP7	-0.11883	0.036834	0.077
11727336_SAMD12	0.142594	0.03685	0.077031
11748116_PKNOX2	-0.12486	0.036863	0.077054
11721999_HNRNPU	-0.0909	0.036871	0.077067
11741811_NME9	-0.27501	0.036881	0.077086
11748632_LRRC27	0.101903	0.036889	0.077099
11745066_MYT1L	-0.1069	0.036898	0.077112
11716084_SF3B1	0.093744	0.036899	0.077112
200085_PMTCEB2	-0.11221	0.0369	0.077112
11742649_CLDN25	-0.19697	0.036902	0.077112
11721005_TMCO3	-0.09083	0.036904	0.077114
11729195_STX16	-0.09842	0.036909	0.077122
11746327_SNX6	0.099776	0.036919	0.077139
11727251_RDH12	-0.13104	0.036923	0.077143
11723331_VAT1L	-0.13824	0.036949	0.077193
11744550_COX20	0.113094	0.03695	0.077193
11752614_NUP214	-0.09139	0.036973	0.077237
11717062_AP1M2	-0.09143	0.036976	0.077242
11728238_SMG1	0.114173	0.036983	0.077253
11736958_TNFSF15	0.166714	0.036991	0.077262
11763215_DMKN	0.302914	0.036992	0.077262
11725761_CNTNAP1	-0.10072	0.036992	0.077262
11757634_LCN2	-0.20499	0.037001	0.077277
11759168_HIST1H2BE	-0.17034	0.03701	0.077292
11749974_PGAP3	-0.12981	0.037013	0.077295
11733499_LPAR4	-0.16854	0.03703	0.077328
11732749_SEC14L5	-0.13019	0.037052	0.07737
11733790_HSDL1	0.156788	0.037055	0.077372
11764059_GJC2	-0.14537	0.037057	0.077375
11737375_RUNDC3A	-0.09851	0.037061	0.077376

11726476_HS6ST3	-0.15615	0.037061	0.077376
11729167_DCTD	0.097393	0.037071	0.07739
11743164_ATF1	-0.13769	0.037071	0.07739
11735794_CDHR5	-0.19386	0.03708	0.077405
11743899_LIN54	-0.17672	0.03709	0.077423
11749710_ABLIM1	0.117853	0.037093	0.077426
11745885_UBE2Z	-0.15731	0.037104	0.077445
11727043_TTI2	0.113851	0.037106	0.077446
11721019_MTMR3	0.205205	0.037108	0.077447
11746777_ELN	-0.16683	0.037111	0.077449
11743645_MXRA7	0.109404	0.03712	0.077466
11755608_KIAA1549	0.127019	0.037124	0.07747
11718513_TSPAN14	-0.11257	0.037126	0.077472
11742969_MAPK12	-0.14373	0.037153	0.077525
11715685_ARPC5	-0.08659	0.037178	0.077573
11760286_EIF2AK4	-0.19629	0.037186	0.077588
11764139_LOC101929	-0.14326	0.03719	0.077591
11723185_CNOT6L	-0.11883	0.037191	0.077591
11746056_CCDC163P	-0.14917	0.037209	0.077625
11762958_LOC100128	0.212343	0.037213	0.077629
11729763_LSM10	0.082488	0.037222	0.077645
11735941_STH	-0.09986	0.037247	0.077694
11748617_SLC30A9	0.099693	0.037256	0.077709
11730204_EEF1D	0.103851	0.037266	0.077726
11753964_HSPH1	0.093295	0.037291	0.077776
11748103_ZNF513	0.095162	0.037315	0.077822
11715581_SF3B2	-0.09864	0.037318	0.077825
11754327_TBC1D10A	0.16234	0.037322	0.077831
11755233_CCDC33	-0.0956	0.037328	0.077838
11726316_SELE	0.253442	0.037329	0.077838
11740282_TSEN2	0.102705	0.037338	0.077854
11718203_PRICKLE4	-0.08758	0.037346	0.077867
11738664_OR6A2	-0.20114	0.037362	0.077898
11750084_FAM69A	-0.23413	0.037366	0.077898
11740440_NLRP6	-0.18072	0.037367	0.077898
11756190_CLK3	0.094614	0.037368	0.077898
11739042_LIMK2	0.16713	0.037369	0.077898
11742332_OR14J1	-0.12016	0.03737	0.077898
11718287_UBL3	0.128871	0.037376	0.077907
11736816_KIRREL	0.122685	0.037379	0.07791
11739069_PSAP	0.089236	0.037387	0.077924
11756102_GUCY2F	-0.26827	0.037394	0.077934
11754386_LOC728743	-0.11175	0.037409	0.077961
11719943_CXCL9	-0.14065	0.037412	0.077964
11751642_LOC100132	-0.2117	0.037417	0.077971
11743639_PPM1N	-0.22742	0.037418	0.077971
11717382_FOPNL	-0.09142	0.03742	0.077972
11756495_SPAG6	-0.15069	0.037445	0.078021
11722669_BAIAP2L1	-0.13742	0.03745	0.078026
11754493_CSRP1	-0.11044	0.037452	0.078026
11728971_PTPRJ	-0.20016	0.037453	0.078026

11737427_IL12RB2	-0.16069	0.037455	0.078026
11739894_CNTD1	0.212268	0.037456	0.078026
11745498_IRF3	0.092543	0.037457	0.078026
11757477_LASP1	0.085046	0.037512	0.078136
11727956_PIANP	-0.19335	0.037517	0.078144
11720257_TRAPPC6B	0.091468	0.037537	0.078182
11716684_MYO1D	0.137264	0.037545	0.078195
11746092_ROGDI	-0.11339	0.037556	0.078214
11718415_NONO	-0.08937	0.037564	0.078228
11731114_SGCG	-0.13253	0.037565	0.078228
11743950_POU4F1	-0.14416	0.037585	0.078266
11747532_PHF21B	-0.12883	0.037592	0.078276
11716197_ADIPOR2	0.10607	0.037596	0.078282
11761451_FRAS1	-0.17563	0.037624	0.078336
11740576_SORCS1	0.157246	0.037627	0.07834
11725913_ERBB4	-0.20499	0.037631	0.078344
11739596_KIAA1429	0.092032	0.037644	0.078368
11747913_UBXN2A	0.099889	0.037656	0.07839
11727685_NTRK3	-0.13005	0.037667	0.07841
11742648_MORF4L1	0.089855	0.037674	0.078421
11723004_F2	-0.14848	0.037682	0.078433
11733652_TMEM225	-0.25433	0.037698	0.078464
11757223_LOC100268	-0.18425	0.037705	0.078475
11752314_ATF7IP	-0.09053	0.03771	0.078482
11759653_NRF1	-0.31027	0.037722	0.078504
11728415_POMZP3 //	-0.10771	0.03773	0.078516
11721223_ACOX2	0.096379	0.037732	0.078516
11738278_TRIM34	0.197138	0.037735	0.078516
11742199_NBR1	-0.12011	0.037735	0.078516
11744583_AMDHD2	-0.1127	0.037739	0.078516
11760438_DSTNP2	-0.19489	0.037739	0.078516
11760439_DSTNP2	-0.19489	0.037739	0.078516
11752466_RPS6KB1	0.106701	0.037752	0.07854
11741887_WDR64	0.187838	0.037756	0.078545
11741427_OSBPL1A	-0.10049	0.037765	0.078559
11717572_NECAP1	0.103179	0.037778	0.078584
11752585_PDZD7	-0.13699	0.037781	0.078587
11747497_CES4A	-0.31393	0.037808	0.07864
11752202_HAL	-0.25345	0.037812	0.078642
11724042_PDZD4	-0.12312	0.037812	0.078642
11737802_MAD1L1	-0.12478	0.037846	0.078706
11720120_GIGYF1	-0.15155	0.037847	0.078706
11737953_SLC28A2	-0.13726	0.037849	0.078707
11764150_ZNF281	0.103816	0.037856	0.078719
11761343_ADAM8	0.170701	0.037882	0.07877
11738841_SPAM1	-0.15581	0.037889	0.07878
11729241_SLC35E3	0.103747	0.037897	0.078794
11717552_FNBP1L	0.113738	0.037901	0.078798
11716560_STRN4	0.10877	0.037911	0.078816
11739995_C5orf28	-0.08777	0.037934	0.078858
11750727_C5orf28	-0.08777	0.037934	0.078858

11729809_CEP104	0.110371	0.037953	0.078892
11757202_LOC202181	-0.16943	0.037954	0.078892
11745520_ABI1	0.084043	0.037959	0.078897
11735551_FCRL6	-0.17672	0.037959	0.078897
11735690_KBTBD4	-0.08225	0.03797	0.078915
11729707_FKBP1A	0.100011	0.037989	0.078949
11731416_NDUFS1	-0.16116	0.03799	0.078949
11723407_HOXD1	-0.12496	0.037991	0.078949
11761302_B4GALNT1	-0.19912	0.037996	0.078956
11728442_CHD5	-0.16999	0.038	0.078961
11745534_CRB1	-0.12602	0.038019	0.078998
11725382_RAPGEF3	0.094661	0.038022	0.079001
11732200_PLAGL2	-0.10007	0.038024	0.079001
11725452_MCUR1	-0.10992	0.038027	0.079002
11732002_WDR86	-0.13116	0.038028	0.079002
11737589_PHTF2	0.176463	0.038038	0.079021
11729808_CEP104	0.106835	0.03804	0.079022
11743958_SNX8	-0.10648	0.038046	0.07903
11753500_HOMER1	0.090718	0.038055	0.079045
200067_PM SNX3	-0.10497	0.038079	0.079091
11759814_CCDC122	-0.14942	0.038081	0.079093
11749379_UXS1	-0.13815	0.038108	0.079145
11723130_ARHGEF7	0.09089	0.038123	0.079173
11756290_PRRC1	0.111401	0.038129	0.079181
11736145_SPATA20	0.096443	0.038143	0.079208
11763615_HNF4A	-0.25018	0.038147	0.079214
11760276_SRSF5	-0.12596	0.038149	0.079215
11762984_SH3TC1	-0.14311	0.038162	0.079235
11754990_EPB41	-0.11531	0.038163	0.079235
11727225_GALNT16	-0.12608	0.038164	0.079235
11761622_TIPIN	-0.13819	0.038179	0.079262
11732695_DRD5	-0.16853	0.038181	0.079263
11738926_KRTAP5-1	-0.17254	0.038184	0.079266
11740087_CSNK2A1	-0.10894	0.038192	0.07928
11745193_SERPINA12	-0.15121	0.038231	0.079356
11748076_ZNF487	-0.17833	0.038242	0.079376
11758174_FAM222B	0.152274	0.038245	0.079379
11763401_VAV3	-0.1955	0.038254	0.079395
11741743_CACNA1C	-0.26348	0.038258	0.079399
11749006_ZFYVE28	-0.12147	0.038263	0.079407
11746239_SIN3B	-0.12517	0.038265	0.079408
11760144_HLA-F	0.149678	0.038268	0.079411
11747480_CENPT	-0.11348	0.038273	0.079414
11727641_NOM1	0.143295	0.038273	0.079414
11730346_PUS7	0.102933	0.038275	0.079414
11752145_SLITRK2	-0.14	0.038282	0.079426
11748509_VDAC2	0.085067	0.038292	0.079443
11722216_GPR108	0.086345	0.038294	0.079444
11726822_CXorf36	-0.09939	0.038304	0.079462
11741176_CHRDL1	-0.13209	0.038322	0.079492
11752431_ANAPC2	-0.1563	0.038322	0.079492

11751248_MTMR7	-0.14723	0.03834	0.079525
11718212_PYCR1	-0.14909	0.038344	0.079531
11738794_DEFB124	-0.15775	0.038381	0.079604
11735570_SORBS2	-0.10206	0.038382	0.079604
11745696_C9orf57	-0.18383	0.03839	0.079616
11742534_FAM58BP	-0.18662	0.038395	0.079622
11716067_VAMP2	-0.12217	0.038401	0.079632
11756411_STAB1	-0.1929	0.038408	0.079644
11735519_ART5	-0.21776	0.038418	0.079662
11752160_FPGT-TNNI	-0.11801	0.03842	0.079662
11739111_MASP1	-0.20296	0.038423	0.079665
11743820_NTPCR	0.091612	0.038456	0.079729
11735074_ST20-AS1	0.104107	0.038459	0.079733
11752542_MUC21	-0.13962	0.038463	0.079736
11738593_FXYD4	-0.11683	0.038466	0.079741
11738112_RGPD5 ///	0.084802	0.038468	0.079741
11729839_ZCCHC2	0.250609	0.038483	0.079767
11763629_LIG3	-0.20854	0.038498	0.079795
11715202_TCEAL1	-0.09785	0.038513	0.079823
11762766_NGRN	0.144502	0.038537	0.079871
11742231_RSPO4	-0.17662	0.03854	0.079874
11749567_DDX55	0.093029	0.038544	0.079879
11715499_CBX3	-0.11951	0.038551	0.079885
11724232_ALDH1A2	0.104571	0.038551	0.079885
11755740_PRM2	-0.11074	0.038553	0.079885
11750308_APMAP	-0.09388	0.038554	0.079885
11741249_NCDN	-0.11823	0.038556	0.079885
11726045_AGER	-0.10025	0.038557	0.079885
11752744_LGMN	-0.09325	0.038583	0.079934
11735973_POU4F3	-0.19682	0.038595	0.079956
11760450_ADAM15	-0.09851	0.0386	0.079963
11749350_BPHL	0.152523	0.038614	0.07999
11761497_PPP3CB	0.17975	0.038629	0.080016
11758269_MAT2B	-0.11143	0.038634	0.080024
11744552_SMUG1	-0.10274	0.038646	0.080046
11731485_NFIC	-0.09748	0.038671	0.080094
11731079_HCAR1	-0.12307	0.038674	0.080097
11748499_SYT5	-0.10871	0.038697	0.080139
AFFX-HUMGGAPDH	-0.08215	0.038698	0.080139
11760370_HLA-H	0.212542	0.0387	0.080139
11754402_ZNF488	-0.10108	0.038711	0.080159
11752273_CSF1R	-0.16312	0.038729	0.080193
11763620_MLLT1	-0.16274	0.038735	0.080201
11725355_EPM2AIP1	0.114119	0.038738	0.080203
11742312_OR4K15	-0.24333	0.038739	0.080203
11742715_TBRG1	-0.10672	0.038747	0.080216
11721867_GNPDA2	0.140269	0.038763	0.080247
11759645_COX11	0.093285	0.038772	0.080262
11751104_ZNF2	0.141268	0.038782	0.08028
11733514_SPAG11A /	-0.12072	0.038794	0.0803
11726556_TAF7L	0.169666	0.038801	0.080311

11750329_WAPL	-0.10237	0.038802	0.080311
11746564_FAM65A	-0.10855	0.038804	0.080312
11760041_MAGT1	-0.15047	0.038809	0.080318
11760070_LOC645010	-0.1243	0.038823	0.080343
11738996_DPYSL2	0.089157	0.038826	0.080343
11723685_FAM46A	0.135561	0.038826	0.080343
11759786_BRF1	-0.1765	0.038846	0.08038
11725339_NOL12	-0.12984	0.038852	0.080391
11751177_RAB5B	0.092137	0.038861	0.080406
11716547_TLR9	-0.13779	0.03887	0.080421
11731185_PPM1E	0.236126	0.038873	0.080423
11718252_HINFP	0.132341	0.038882	0.080439
11726998_CLDN6	-0.12648	0.038884	0.08044
11719735_BAG6	0.089037	0.038905	0.080478
11730558_BCO2	0.129071	0.038911	0.080488
11757212_FLJ36777	-0.16451	0.038914	0.080492
11757425_VCAM1	0.2029	0.038922	0.080499
11747445_KIFC3	-0.10481	0.038922	0.080499
11741098_A2ML1	0.179569	0.038923	0.080499
11735237_EFCAB3	-0.22592	0.038925	0.080501
11750371_INGX	-0.11599	0.038927	0.080501
11737120_DACH2	-0.15853	0.03893	0.080504
11715305_HOXA10	-0.11776	0.038936	0.080513
11738275_SPATA21	-0.1725	0.038958	0.080554
11750462_CLEC4M	0.114747	0.038976	0.080589
11739865_GPR107	0.092797	0.039009	0.080653
11756343_DPEP1	-0.15081	0.039017	0.080667
11749375_VAMP1	0.16015	0.039032	0.080694
11753974_SNRPG	-0.0893	0.039055	0.080738
11727915_NCAM1	-0.30101	0.039064	0.080752
11723166_EPHB2	0.202641	0.039065	0.080752
11740264_ETV3	-0.14296	0.039082	0.080784
11727740_CD300LF	-0.21568	0.039098	0.080813
200077_PM OAZ1	-0.08329	0.039119	0.080851
11742741_GEMIN5	-0.10626	0.039121	0.080851
11733349_ZNF287	0.272847	0.039121	0.080851
11722960_CYB561D1	0.151894	0.03915	0.080908
11720899_GRAMD1C	-0.20598	0.03916	0.080924
11725905_S1PR3	-0.22162	0.039168	0.080938
11762125_KIAA0895	0.1879	0.03919	0.080979
11726936_HOXB4	-0.11742	0.039196	0.080988
11758226_UBE2J1	-0.12027	0.039197	0.080988
11723417_HSP90AB1	-0.08899	0.03921	0.081012
11735285_ERCC2	0.088136	0.039213	0.081013
11759071_PROX1	0.236895	0.039216	0.081016
11715838_NEU1	0.132706	0.039218	0.081017
11751850_ZNF169	-0.09624	0.039225	0.081029
11745748_XRCC1	-0.12886	0.039236	0.081047
11741825_GBX1	-0.14778	0.03924	0.081053
11751778_TIMM23	0.094443	0.039243	0.081055
11759362_PHKG1	0.164711	0.039246	0.081058

11752377_CASC3	-0.08755	0.039262	0.081087
11735463_NTNG1	-0.21413	0.039271	0.081103
11744950_FAM71D //	-0.08593	0.03928	0.081115
11724019_C21orf2	-0.10844	0.03928	0.081115
11750644_PPIP5K1	-0.15359	0.039283	0.081117
11752094_EIF4G2	0.088573	0.039305	0.081159
11749144_ETFDH	-0.13195	0.039309	0.081163
11730851_ELMO1	-0.12281	0.039313	0.081169
11718487_EI24	0.083704	0.039317	0.081173
11725645_WDR13	-0.09366	0.039323	0.081181
11715453_COL4A2	0.112807	0.039336	0.081207
11730853_P4HA1	-0.10895	0.039351	0.081233
11738903_LILRA5	-0.2016	0.039373	0.081275
11759710_TXNDC9	0.123774	0.039377	0.08128
11753966_ZNF204P	0.243971	0.039381	0.081285
11740639_NMUR1	-0.17479	0.039401	0.081322
11725601_SOWAHA	-0.30205	0.039405	0.081326
11727639_MPHOSPH9	-0.10307	0.039406	0.081326
11754278_GAK	-0.08717	0.039429	0.08137
11743407_RBM25	0.108723	0.039431	0.081371
11722061_PPP1R1A	-0.14232	0.039458	0.08142
11719737_FAM134B	0.111335	0.039458	0.08142
11716928_TBC1D5	0.10002	0.039467	0.081434
11725249_LY75	0.14086	0.039472	0.081442
11747827_KLHL17	-0.10933	0.03948	0.081454
11730921_NHLRC4	-0.17648	0.039486	0.081462
11726491_SLC4A1	-0.17957	0.039487	0.081462
11738503_OR7D4	-0.15687	0.039496	0.081478
11741589_EEF1D	0.088986	0.039498	0.081478
11722072_MESDC1	0.091581	0.039505	0.08149
11720118_HEATR6	-0.13783	0.03951	0.081497
11729144_KDM5C	0.112308	0.039521	0.081516
11733249_ARHGAP31	0.100115	0.039525	0.081521
11749448_NBR2	-0.11991	0.039531	0.081529
11724267_C2orf72	-0.13555	0.039558	0.081583
11724214_SPRYD4	-0.15352	0.039561	0.081583
11755184_PLEKHM3	0.104366	0.039573	0.081606
11715852_PDGFRB	-0.13087	0.039579	0.081614
11762942_SOSTDC1	-0.10584	0.039584	0.081621
11760839_ANKS1A	-0.18363	0.039595	0.081639
11758352_RRNAD1	-0.0971	0.039596	0.081639
11730627_SPHKAP	-0.14922	0.039612	0.081669
11721994_UBE2L6	0.10731	0.039624	0.081689
11754712_TBC1D10A	0.171995	0.039626	0.08169
11759966_ITPKB	-0.25136	0.039636	0.081707
11728009_VPREB3	-0.1387	0.039655	0.081736
11760129_ZFPL1	-0.19798	0.039656	0.081736
11756433_MSL1	0.193018	0.039656	0.081736
11731723_ALDH3B1	0.204497	0.039657	0.081736
11749819_CNOT8	-0.08234	0.039692	0.081806
11717183_PDS5A	0.092775	0.039702	0.081822

11755114_NRBP1	0.12054	0.039703	0.081822
11752684_C12orf40	-0.08418	0.039715	0.081843
11753166_WDR6	-0.14408	0.039717	0.081843
11723181_MAPK4	-0.17596	0.039726	0.081859
11740888_MRO	0.118777	0.039745	0.081895
11739847_MSR1	-0.10519	0.039764	0.081931
11716620_TNPO1	0.084315	0.039783	0.081966
11737082_ASXL3	-0.21623	0.03979	0.081976
11727538_NME5	-0.16837	0.039793	0.081979
11717661_PPP1R16B	-0.15979	0.039797	0.081983
11756944_SNTG1	0.112557	0.0398	0.081983
11742285_PAX3	-0.14265	0.039801	0.081983
11722792_APLNR	-0.19642	0.039803	0.081983
11715756_UBL5	-0.09543	0.039803	0.081983
11731184_MESP2	-0.11409	0.039811	0.081993
11753650_SRSF3	-0.09402	0.039813	0.081993
11735354_GSTM4	0.095884	0.039813	0.081993
11739940_SENP1	-0.12411	0.039846	0.082057
11721973_JADE3	0.188465	0.039848	0.082059
11753312_DNAJC5G	-0.1261	0.039855	0.082066
11728939_RASEF	-0.1151	0.039855	0.082066
11760956_GGNBP2	-0.12807	0.039865	0.082083
11750451_UBAC2	-0.09794	0.039885	0.082119
11756136_PLGRKT	-0.09058	0.039886	0.082119
11756420_LRCH4	-0.1371	0.03989	0.082124
11718888_TMEM59L	-0.14079	0.039901	0.082143
11759677_HERC4	0.136151	0.039907	0.082152
11724440_LNX2	0.111326	0.03991	0.082155
11737439_BMF	-0.1143	0.039918	0.082169
11740878_ZNF669	0.12086	0.039946	0.082223
11760149_PRSS1	-0.12149	0.039957	0.082238
11723007_CCPG1 ///	0.088559	0.039958	0.082238
11745070_LOC440040	-0.22457	0.039959	0.082238
11728807_SIRT5	-0.16245	0.039966	0.08225
11742635_GSTA5	-0.18384	0.039992	0.082299
11732984_FTHL17	-0.10415	0.040004	0.082319
11722783_FOXQ1	0.170795	0.040005	0.082319
11716124_NAT10	0.104619	0.040007	0.082321
11731000_AP5B1	0.148524	0.040012	0.082327
11717888_CHAMP1	-0.12311	0.040021	0.082342
11717462_NSUN2	0.087741	0.040048	0.082395
11730649_TEX43	0.217237	0.040052	0.082398
11763350_FAM212B /	0.115334	0.040058	0.082407
11730760_CRIPT	0.095746	0.040087	0.082464
11750176_SVILP1	-0.1006	0.040094	0.082474
11759355_GPR32	-0.11738	0.040096	0.082475
11760180_CSNK2B	-0.14376	0.040097	0.082475
11750708_EFCAB5	-0.13894	0.040101	0.082479
11738515_LIPK	0.174092	0.040117	0.082508
11737663_EML5	0.165826	0.040134	0.082539
11729888_BRINP3	0.223766	0.040138	0.082544

11739951_GFOD1	-0.12523	0.040157	0.082579
11748278_ASCC1	0.090678	0.040162	0.082587
11753276_CYP4A11 /	-0.09477	0.040171	0.0826
11718624_PLA2G1B	-0.33681	0.040172	0.0826
11728215_NRP1	0.14436	0.040178	0.08261
11724276_SLC35C1	-0.12432	0.040188	0.082627
11732829_CCL16	-0.20777	0.040196	0.082639
11758473_ZEB1	0.096033	0.040223	0.082692
11748731_ZNF616	0.167725	0.040229	0.082699
11762224_SLC1A3	-0.1395	0.040237	0.082714
11733703_SKIDA1	0.36204	0.040246	0.082727
11762296_PDE7B	-0.14368	0.04025	0.082733
11722243_FBXO25	-0.14593	0.040268	0.082766
11761470_APBB1	0.102919	0.040281	0.08279
11737754_ABCB6 ///	0.099443	0.040287	0.082799
11760628_TTLL5	0.191847	0.040302	0.082825
11725365_IER5L	0.15612	0.040307	0.082833
11737461_LINGO4	-0.19213	0.040317	0.08285
11744711_C21orf2	-0.11592	0.04032	0.082852
11745553_SMAP2	0.194903	0.040345	0.082901
11749039_PNRC1	-0.09658	0.040361	0.082929
11756838_FCN2	0.115821	0.040364	0.082932
11733731_BCKDHA	-0.09581	0.04038	0.082962
11736254_NUP160	0.094817	0.040404	0.083008
11745481_GLYCTK	-0.16018	0.040415	0.083026
11739287_KMT2A	0.107387	0.040425	0.083043
11722088_NR1D2	-0.27186	0.040435	0.083061
11720936_CACFD1	0.087701	0.040441	0.08307
11744457_MDK	-0.11126	0.040483	0.083152
11718351_NLRP2	-0.14223	0.0405	0.083184
11747857_MPDZ	0.129265	0.040508	0.083198
11736058_BORCS7	0.194555	0.04052	0.083218
11728212_TRMT1L	-0.18898	0.040526	0.083227
11726969_BCAS4	-0.2641	0.040538	0.083248
11742083_KIRREL2	-0.15195	0.04055	0.083268
11752956_C5orf51	0.141719	0.040561	0.083288
11740471_CLIP1	0.090518	0.040578	0.083319
11754272_SNRPN ///	-0.08977	0.040601	0.083359
11733169_FBXO45	-0.14759	0.040601	0.083359
11731582_WASF1	0.162206	0.040611	0.083377
11750492_RNF216	0.112967	0.040616	0.083384
11715636_SERPINE1	0.17905	0.040636	0.083418
11727942_FPR3	0.087935	0.040638	0.083418
11729307_GPR35	-0.12759	0.040638	0.083418
11730604_EPB41L4B	0.131898	0.040641	0.08342
11737783_PROSC	0.123884	0.040648	0.083431
11723215_MEF2C	-0.12011	0.040652	0.083435
11750975_GBA	0.082657	0.040653	0.083435
11749191_ZAK	-0.13762	0.040671	0.083468
11756540_TMPRSS5	-0.11467	0.040676	0.083475
11763268_FAM120B	0.110854	0.040682	0.083484

11748097_PABPC1	0.083785	0.040686	0.083488
11724451_ZDHHC22	-0.12648	0.040688	0.083489
11722853_HABP4	0.158943	0.04069	0.08349
11726790_CNTN2	-0.15646	0.040711	0.083529
11745815_CDIP1	-0.11579	0.040714	0.083531
11721941_CYP3A5	0.115039	0.040755	0.083612
11744068_APOE	-0.09086	0.040758	0.083615
11745125_TUBGCP2	0.096545	0.040771	0.083637
11741442_RBMX ///	-0.09577	0.040778	0.08365
11753628_ABHD11	-0.09166	0.040781	0.083652
11727596_PADI2	-0.11203	0.040787	0.083661
11721580_DIO2	0.155947	0.040793	0.08367
11715772_MRPL13	-0.09372	0.040827	0.083736
11741705_NR4A3	0.105459	0.040833	0.08374
11734744_SON	0.106832	0.040835	0.08374
11752540_PPIL2	-0.09573	0.040835	0.08374
11737393_PATE2	0.17054	0.040836	0.08374
11732290_TRMT61A	-0.10838	0.040849	0.08376
11762998_YME1L1	0.096219	0.040849	0.08376
11755159_COG3	0.123629	0.040856	0.083771
11741372_SPDYE6	0.195465	0.040862	0.08378
11753905_SMARCA1	-0.11812	0.040884	0.083821
11733907_CYP2D6 //	0.147001	0.040886	0.083821
11756989_KRT8	0.106345	0.04089	0.083826
11726710_PFAS	-0.11409	0.040904	0.083851
11760049_LINC00308	-0.13472	0.040913	0.083867
11729815_ZADH2	-0.12527	0.040915	0.083868
11740279_IRX2	-0.12988	0.040917	0.083868
11760629_LIPC	-0.18147	0.040922	0.083874
11733577_DOCK9	-0.10224	0.040951	0.08393
11738122_NR5A2	0.246617	0.040954	0.083933
11720471_MED20	0.108773	0.040975	0.083972
11756553_GUK1	0.105079	0.040988	0.083995
11753287_TECPR2	0.254598	0.040991	0.083998
11760363_FAM227B	-0.12751	0.041028	0.08407
11721951_MRAP2	-0.19883	0.041031	0.084073
11732739_ARSB	0.152445	0.041033	0.084073
11722593_BCAP29	-0.08996	0.041037	0.084078
11739309_DLG1	-0.10559	0.041063	0.084127
11715813_HIGD1A	-0.18292	0.041066	0.084131
11752257_SLC22A2	-0.14098	0.041076	0.084147
11756305_SMARCA4	0.104033	0.041081	0.084154
11734577_SCN5A	-0.15534	0.041101	0.084192
11715795_MANF	-0.10014	0.041106	0.084199
11736175_ARPIN	-0.12003	0.041136	0.084256
11741108_MRPS14	-0.11203	0.041142	0.084264
11747900_DMTF1	0.153539	0.041143	0.084264
11760106_ATP5SL	-0.1483	0.041154	0.084279
11715138_CGB /// C	-0.0867	0.041155	0.084279
11744973_SLC30A5	-0.1214	0.041155	0.084279
11760650_ANKRD37	0.210678	0.041167	0.084298

11715249_MAGEA12	-0.11316	0.041188	0.084338
11731257_TRIM67	0.245786	0.041207	0.084374
11734834_TAF9B	0.092541	0.041212	0.08438
11723772_SMPD2	0.203756	0.041222	0.084397
11753654_PRAP1 ///	-0.15803	0.041224	0.084398
11726114_FAM46B	0.110235	0.041233	0.084412
11763278_FGFR1OP2	0.137152	0.041248	0.08444
11757229_TPH1	-0.13569	0.041256	0.084448
11743155_POFUT1	-0.11966	0.041256	0.084448
11727218_TSSK2	-0.12379	0.041275	0.084484
11732371_KLK7	-0.10245	0.041281	0.08449
11720288_CDC42BPA	0.108326	0.041281	0.08449
11756438_SAFB	0.092962	0.041307	0.08454
11722003_CYCS	-0.15294	0.041323	0.084568
11720848_PHF6	-0.14802	0.041332	0.084584
11753683_KLK4	-0.15212	0.041335	0.084586
11737376_CFAP46	-0.13636	0.041346	0.084602
11722293_NAPSA	-0.09017	0.041346	0.084602
11743112_S100A10	0.089611	0.041392	0.084692
11728091_SHISA7	-0.12217	0.041415	0.084736
11736581_GCNT1	-0.1527	0.041423	0.084749
11726170_FLT4	-0.12137	0.041441	0.084781
11761926_RHBDF1	0.149951	0.041446	0.084788
11731900_PPAT	0.115894	0.041457	0.084807
11718466_NCK2	0.082889	0.041461	0.08481
11722921_MVB12A	-0.10894	0.041462	0.08481
11760187_DDT /// D	-0.22417	0.041481	0.084846
11738170_ADCYAP1	0.205249	0.04149	0.08486
11717212_NEO1	-0.16501	0.041501	0.084873
11742728_SNX5	0.089771	0.041501	0.084873
11726629_CDK11B	0.117959	0.041502	0.084873
11739900_SLC19A3	0.115733	0.041503	0.084873
11745059_RIN1	-0.0954	0.041546	0.084957
11742075_LMX1A	-0.1207	0.041551	0.084958
11740035_CARD8	0.1593	0.041553	0.084958
11735823_ZP4	0.111186	0.041553	0.084958
11717790_APPL2	-0.10422	0.041554	0.084958
11720172_SF1	0.092417	0.041571	0.08499
11742943_CKM	-0.13521	0.04159	0.085026
11722655_STK17A	0.087186	0.041592	0.085026
11746812_BCOR	0.124604	0.041631	0.085102
11760186_ACTA2	-0.25855	0.04165	0.085137
11763203_PROCA1	-0.31314	0.041661	0.085157
11725586_RAB36	-0.12523	0.041673	0.085174
11753411_FAF2	0.105256	0.041674	0.085174
11748845_SLAMF7	0.215325	0.041675	0.085174
11718714_B4GALT2	-0.11177	0.041677	0.085176
11747956_FAM117A	-0.13597	0.041698	0.085215
11731175_KDF1	-0.16051	0.041706	0.085227
11756157_PSG2	0.146562	0.041724	0.085261
11732441_BPTF	0.105283	0.041744	0.085294

11719627_RDH10	0.190872	0.041744	0.085294
11736325_SWSAP1	-0.15624	0.041755	0.085313
11737122_ANKFN1	0.271595	0.041769	0.085338
11748714_COL21A1	0.118367	0.041788	0.085372
11762059_PDGFA	0.11674	0.041789	0.085372
11729031_C1orf194	-0.16907	0.041804	0.0854
11734683_UMPS	-0.17856	0.041807	0.085401
11723219_IAPP	-0.19527	0.041816	0.085416
11756097_ZMYND10	-0.19902	0.041827	0.085435
11739044_SLC15A2	0.140031	0.041838	0.085453
11733298_VIPR1	0.121499	0.041851	0.085477
11754651_KRBA1	0.09141	0.041853	0.085477
11751031_CSK	0.106722	0.041856	0.085479
11744636_OPRL1	-0.20065	0.041866	0.085497
11742114_OR7A5	-0.12122	0.041873	0.085508
11758431_U2AF2	-0.16589	0.041887	0.085533
11762518_KIAA2012	-0.20068	0.041893	0.085541
11749942_ADORA2A /	-0.1024	0.0419	0.08555
11742484_SLC22A20	-0.14987	0.0419	0.08555
11717995_NR4A1	-0.08446	0.041944	0.085635
11725610_DTNA	0.184455	0.041957	0.085658
11732880_TBX1	-0.1431	0.041976	0.085693
11748565_EBNA1BP2	0.108194	0.041978	0.085693
11724810_CBL	-0.12214	0.04198	0.085694
11744815_SPATA2L	0.139304	0.041983	0.085696
11759516_GTF3C3	-0.20446	0.041996	0.085719
11742409_KLK8	-0.17123	0.042011	0.085743
11752878_ZNF750	-0.13406	0.042011	0.085743
11729928_AQP2	-0.10956	0.042025	0.085768
11725451_TMEM45B	0.151411	0.042039	0.085788
11746854_SPINT1	-0.15702	0.042039	0.085788
11749311_GRPEL1	0.098015	0.04204	0.085788
11744200_SNRNP200	0.087283	0.042043	0.085788
11717358_RTF1	-0.09659	0.042044	0.085788
11722959_ABLIM2	-0.12567	0.042045	0.085788
11734822_APBB2	-0.20488	0.04206	0.085814
11761496_ESCO2	-0.28591	0.042062	0.085814
11750828_HIP1R	-0.21304	0.042075	0.085835
11729509_FLRT1	-0.13456	0.042075	0.085835
11756060_RPS13	0.081194	0.04209	0.08586
11735065_ITGB3	0.173372	0.042091	0.08586
11739708_CACYBP	-0.15437	0.042094	0.085861
11745013_MORN1	-0.15514	0.042104	0.085878
11719387_GOLGA5	0.088928	0.042124	0.085917
11735294_LRRK2	0.199524	0.042127	0.08592
11739983_PRH1	0.157245	0.042137	0.085937
11748698_LYVE1	-0.14192	0.04214	0.085937
11720034_CASP2	0.119067	0.04216	0.085976
11745858_ATP5SL	-0.10794	0.042179	0.086009
11734352_ANK1	0.254297	0.04218	0.086009
11737240_TEX15	-0.26506	0.042204	0.086054

11721487_TP53RK	-0.10978	0.042214	0.086071
11740536_KRTAP19-3	-0.12482	0.042216	0.086072
11726507_CDKN2B	-0.1757	0.042224	0.086078
11735347_MIEF2	0.121439	0.042225	0.086078
11715612_TMX2	0.081564	0.042225	0.086078
11715193_UNCX	-0.11691	0.042226	0.086078
11733912_HS3ST1	-0.13275	0.042244	0.08611
11747908_CUL7	0.149575	0.042262	0.086143
11759132_SH3D21	-0.12524	0.042266	0.086149
11728765_PVRL4	0.188559	0.04228	0.086173
11726525_ZFHX3	-0.14682	0.042284	0.086176
11732506_TP73-AS1	-0.08752	0.042285	0.086176
11726904_COBL	0.113143	0.042317	0.086235
11748673_LOC644794	0.111057	0.042319	0.086235
11723625_SPSB4	-0.22569	0.042319	0.086235
11733206_AFAP1L1	0.140911	0.042328	0.086249
11755812_BNC2	-0.14626	0.042346	0.086283
11722337_TBC1D9B	-0.11121	0.042351	0.08629
11756177_CMTM6	-0.10114	0.042359	0.086301
11729282_PTP4A3	-0.18393	0.042361	0.086303
11736041_KIF1A	-0.14642	0.042364	0.086305
11728087_ZXDB	0.149815	0.042367	0.086308
11751235_NR1H2	0.154124	0.042371	0.086313
11761713_TCTE1	-0.13393	0.042374	0.086315
11731026_CREM	0.202935	0.042377	0.086318
11718932_FKBP1A-SD	0.099349	0.042396	0.086352
11731474_SFRP4	0.109814	0.042402	0.086361
11760860_SUMF1	-0.10803	0.042409	0.086371
11754269_SOCS1	0.222547	0.04243	0.086409
11730705_OR51E2	-0.21517	0.042431	0.086409
11755029_HID1	-0.11579	0.042447	0.086438
11744844_GON4L	0.099014	0.042466	0.086474
11756008_AGBL4	-0.10046	0.042483	0.086504
11750696_RASAL1	-0.12212	0.042491	0.086516
11746282_TMEM53	-0.09115	0.042513	0.086559
11720451_PLEKHH2	-0.1204	0.042519	0.086561
11740575_SORCS1	0.104073	0.042519	0.086561
11744909_HDHD2	-0.14649	0.04252	0.086561
11735477_CHST6	-0.17791	0.042526	0.08657
11738795_KRTAP4-3	-0.14842	0.042541	0.086593
11758897_APOBEC3C	0.091337	0.042542	0.086593
11750152_SLC23A2	0.121956	0.042543	0.086593
11731977_DNAJB3 //	-0.18386	0.042549	0.086603
11738035_RTN4	-0.10037	0.042559	0.086619
11740943_SHOX	-0.21065	0.042567	0.086631
11749552_LAMP3	-0.1587	0.042589	0.086672
11762085_MAP3K7CL	-0.23076	0.04259	0.086672
11729524_ASIC2	-0.14233	0.042598	0.086683
11746563_FAM65A	-0.09545	0.042626	0.086738
11726501_MYPOP	-0.08856	0.042641	0.086765
11741751_NLRP14	-0.17175	0.042668	0.086815

11757124_SNORA74B	-0.13436	0.042669	0.086815
11719153_LDOC1L	-0.17058	0.042674	0.08682
11755419_LRTM2	-0.14719	0.042679	0.086826
11750200_ZNF215	0.091363	0.042689	0.086843
11757382_MRPL52	0.098375	0.042693	0.086848
11753675_PFN2	0.14803	0.042698	0.086854
11747915_MAP4	-0.11933	0.042701	0.086857
11744446_SUN2	-0.19935	0.042723	0.086899
11724725_EIF5	0.119968	0.042758	0.086966
11724371_SLC22A7	-0.11913	0.042776	0.086999
11730826_TMEM106A	0.125345	0.042787	0.087019
11738969_DEFB116	-0.21208	0.042795	0.087031
11726181_SMYD4	-0.11585	0.042812	0.087061
11731972_PTPN22	0.122224	0.042832	0.087098
11725383_C15orf39	0.100305	0.042842	0.087113
11739092_LUC7L3	0.112722	0.042843	0.087113
11749108_ADGRF5	-0.12085	0.042844	0.087113
200045_PMABCF1	0.107682	0.042852	0.087124
11731398_FGA	-0.16237	0.042853	0.087124
11745356_IKBKB	-0.12844	0.042862	0.087138
11751686_WDR77	0.081257	0.042893	0.087197
11739699_STON1	0.146567	0.042895	0.087197
11761923_RXFP1	-0.15545	0.042901	0.087205
11733755_PKD2L1	-0.11397	0.042902	0.087205
11751717_SLC6A13	-0.13105	0.042907	0.087212
11726652_ARHGAP32	-0.11098	0.042919	0.087232
11747004_SUOX	-0.0837	0.042939	0.087269
11725970_HNRNPF	-0.09147	0.042952	0.087293
11755291_DENND2D	-0.13028	0.042961	0.087307
11760992_L3HYPDH	0.219028	0.042998	0.087378
11735759_DNM2	-0.09321	0.043001	0.087378
11741089_CD79A	-0.18813	0.043001	0.087378
11746925_UMODL1	-0.15484	0.043006	0.087383
11732609_NBPF3	-0.10919	0.043014	0.087397
11727616_RASL10B	-0.19245	0.043051	0.087468
11728354_CHCHD5	-0.13794	0.043095	0.087554
11749504_RELN	0.154842	0.043099	0.087558
11739338_FAM46C	0.098029	0.043113	0.087582
11755177_XYLT2	-0.10496	0.043142	0.087639
11762265_C4BPB	-0.14477	0.043148	0.087647
11758059_ANKRD49	0.106932	0.043159	0.087665
11738568_KRTAP5-3	-0.10166	0.043167	0.087677
11747094_C19orf43	0.101328	0.043171	0.087682
11755759_MEGF11	0.305744	0.043182	0.0877
11720775_RNF8	0.101698	0.043193	0.08772
11729174_SKA2	-0.09141	0.043236	0.087801
11739775_LYPD5	0.327958	0.043237	0.087801
11733195_FMNL3	-0.10138	0.043239	0.087802
11726941_ZKSCAN2	0.115306	0.043257	0.087834
11756474_CPT1A	0.096982	0.043269	0.087856
11757030_ECH1 ///	0.100666	0.043273	0.087861

11762888_DIO3OS	-0.10219	0.043278	0.087866
11728898_CDH15	-0.11815	0.043306	0.08792
11732675_RNF180	-0.24675	0.043339	0.087982
11733832_MAP2	0.251473	0.04334	0.087982
11727511_UBN2	-0.21053	0.043353	0.088005
11716613_RPLP2	0.081624	0.043369	0.088032
11760907_PPA2	0.254849	0.043411	0.088115
11730874_UBQLN3	-0.10748	0.043417	0.088124
11761202_NIPAL3	-0.15609	0.04342	0.088124
11734755_CCBL2	0.107175	0.043421	0.088124
11745367_FANCM	-0.19761	0.043435	0.088146
11720793_AACS	0.137808	0.043435	0.088146
11722077_SEPT3	-0.11627	0.043438	0.088147
11726399_SPTB	-0.09919	0.04344	0.088148
11759973_PDSS1	-0.19265	0.043467	0.088199
11732561_FZD3	0.22551	0.043472	0.088206
11718737_ST3GAL1	0.113118	0.043479	0.088216
11736626_BAAT	0.191991	0.043488	0.088231
11717822_SLC4A4	-0.23479	0.043496	0.088242
11729093_IGF2-AS	-0.13125	0.043497	0.088242
11749367_FXYD5	0.086735	0.0435	0.088242
11749935_TPM3	-0.08875	0.043501	0.088242
11762610_YIF1B	-0.20847	0.043505	0.088247
11750489_ALPK1	0.213225	0.043512	0.088258
11717364_BCL2L12	-0.09692	0.043516	0.088262
11723564_NR2C2	-0.0916	0.043526	0.088279
11738381_FGF23	-0.1552	0.043537	0.088298
11737445_SYT16	0.225239	0.043552	0.088324
11752490_NMUR1	-0.15092	0.043568	0.088353
11738061_S100A7 //	0.147741	0.043574	0.088361
11718570_NT5DC3	-0.19231	0.043579	0.088368
11761184_GPR137	0.100852	0.043603	0.088412
11728221_NRIP3	-0.15043	0.043607	0.088416
11754599_NISCH	0.114472	0.043611	0.088421
11756074_MMP7	0.150582	0.043619	0.088433
11731958_FOLH1 ///	-0.20344	0.043634	0.088462
11721295_STAM	-0.1017	0.043637	0.088464
11725069_JADE1	-0.10649	0.043644	0.088474
11740583_POGZ	0.114773	0.04368	0.088541
11757552_ARPC3	0.092558	0.043681	0.088541
11716157_BAHD1	0.094404	0.043699	0.088574
11720125_SRD5A3	-0.18444	0.043701	0.088575
11738335_TP53	-0.12843	0.043706	0.088581
11732769_RNF152	-0.10907	0.043723	0.088611
11748656_FHL1	-0.13508	0.043725	0.088613
11723737_RNF216	-0.11034	0.043733	0.088624
11738705_OR4K2	-0.187	0.043739	0.088632
11760691_CHRDL2	-0.22034	0.043751	0.088655
11744379_COL3A1	0.24313	0.043755	0.088658
11728539_MDGA1	-0.10768	0.043762	0.088669
11720038_VTA1	-0.11186	0.043766	0.088674

11763579_GLI2	-0.1662	0.043779	0.088697
11716159_RNF40	0.153994	0.043783	0.0887
11719973_AKAP17A	0.139079	0.043796	0.088723
11724934_THAP7	-0.11351	0.043803	0.088733
11750452_CYP2C8	-0.12026	0.043805	0.088733
11730228_QRSL1	-0.10075	0.043808	0.088737
11729884_C18orf54	-0.20841	0.043818	0.088753
11755800_CCDC30	-0.13104	0.043827	0.088767
11746851_FAM192A	-0.10195	0.04385	0.088809
11730540_TAF1A	0.127584	0.043852	0.08881
11759282_SERINC5	-0.15734	0.043857	0.088816
11750114_PLXNB3	-0.10558	0.043858	0.088816
11743226_CCDC160	0.174078	0.043863	0.088822
11757076_C7orf72	-0.19321	0.043868	0.088828
11749578_COX10	0.104096	0.043877	0.088842
11757406_NEK1	0.164683	0.04389	0.088865
11738056_TMEM136	0.09059	0.043896	0.088875
11743413_UBE2K	0.094739	0.043902	0.088879
11736661_MAP3K13	0.371239	0.043902	0.088879
11719703_CD2BP2	-0.09375	0.043937	0.088947
11726531_BRCC3	0.185526	0.043943	0.088955
11741308_XPR1	-0.17351	0.043949	0.088963
11756583_ESRRG	0.114284	0.043956	0.088974
11752661_MKS1	-0.09996	0.043968	0.088994
11743040_CRTAP	0.086183	0.043987	0.089028
11739537_ITPR2	0.155765	0.043989	0.08903
11759495_BAG4	0.096954	0.043994	0.089032
11719234_RASL12	-0.19758	0.043994	0.089032
11744720_GSG1	0.231335	0.044008	0.089056
11745965_IDH3A	-0.10334	0.044009	0.089056
11719543_C3orf18	-0.29618	0.044018	0.08907
11724460_MCAM	-0.08608	0.044029	0.089088
11719116_GOT2	-0.09799	0.044043	0.089113
11746404_TCF7L2	0.156631	0.044065	0.089154
AFFX-HUMGGAPDH	-0.08042	0.044107	0.089234
11762996_YME1L1	0.237891	0.04411	0.089236
11763343_AZIN2	-0.12813	0.044112	0.089237
11735081_GALNT13	-0.15105	0.044119	0.089248
11746361_CPED1	-0.13647	0.044124	0.089255
11744460_PI4KB	0.094204	0.044126	0.089255
11757528_NOL12	0.106892	0.044141	0.089281
11728434_GH2	-0.27212	0.044152	0.089299
11719728_SIRPA	-0.24827	0.044157	0.089305
11722404_MTHFS ///	-0.08142	0.044167	0.089324
11748848_DAW1	-0.11203	0.044183	0.089351
11752255_ZNF417	0.176643	0.044196	0.089374
11758162_FDX1	-0.11531	0.044202	0.089382
11759718_SETMAR	-0.18841	0.044217	0.089408
11738219_GJB4	-0.10944	0.044238	0.089447
11743077_CEBPB	-0.10955	0.044264	0.089498
11741946_LIPM	-0.18267	0.044266	0.089498

11755926_CNGA4	-0.15254	0.044268	0.089498
11727987_DNMT3B	-0.09817	0.044273	0.089501
200070_PMCNPPD1	-0.09277	0.044273	0.089501
11764046_IGHM	-0.17582	0.044277	0.089505
11756383_FARSB	-0.13071	0.044281	0.089509
11758678_ZNF423	-0.1838	0.044307	0.089558
11734779_ZNF639	-0.12854	0.044322	0.089584
11752777_BMP8A ///	0.140382	0.044329	0.089596
11725200_PTH1R	-0.1665	0.044336	0.089605
11732139_TIMM13	-0.11827	0.04434	0.08961
11717624_TBL2	-0.08759	0.044371	0.089669
11761031_RPS28	0.082681	0.044379	0.089681
11730249_MRPS28	0.081769	0.044401	0.089722
11737347_ASB16	-0.11426	0.044423	0.089762
11740180_KCNQ2	-0.09157	0.044436	0.089785
11752786_CARD6	0.249103	0.044438	0.089785
11746027_NMI	0.10368	0.044457	0.089821
11747051_SLC35C2	0.114451	0.044475	0.089852
11738918_CRLF2	-0.09384	0.044478	0.089856
11753170_CD300C	-0.14055	0.044482	0.089859
11721956_RFX7	0.122409	0.044489	0.089869
11737701_DDX31	-0.15839	0.044493	0.089872
11729979_PAGE2 ///	0.114471	0.044494	0.089872
11725146_MIS12	-0.12437	0.044498	0.089878
11727614_SCN2B	-0.10135	0.044542	0.089962
11742618_OR4K14	-0.30112	0.044557	0.089988
11741941_GML	-0.12376	0.044564	0.09
11746210_SCYL1	-0.08849	0.04457	0.090008
11741973_UNC80	-0.14831	0.044574	0.090011
11735968_NDRG2	-0.25201	0.044584	0.090029
11757357_SPSB2	-0.09143	0.044589	0.090034
11727565_TNFSF13B	0.274632	0.044612	0.090077
11736708_ZNF547	0.241456	0.044621	0.090092
11739077_NFE2L1	-0.08378	0.044636	0.090119
11750368_KRTAP5-2	-0.1483	0.044649	0.090141
11755717_DOK2	-0.12764	0.044658	0.090153
11762425_WRNIP1	-0.12018	0.044659	0.090153
11760708_EIF4A2	-0.10142	0.044663	0.090157
11746455_JAM2	0.209218	0.044664	0.090157
11732779_PSMD12	-0.15561	0.044672	0.090168
11719555_PDK2	-0.12334	0.044683	0.090188
11734094_PROM2	-0.16519	0.044696	0.090208
11763559_FMNL1	-0.15267	0.044697	0.090208
11761319_LOC101060	0.106899	0.04475	0.090312
11723275_MAPK8IP1	0.175226	0.044756	0.09032
11754769_BEND5	-0.13064	0.044762	0.090329
11733337_FANCE	0.137567	0.044776	0.090353
11736211_MRPL57	-0.09142	0.044817	0.09043
11731192_UNC13C	-0.10133	0.044818	0.09043
11743329_BRICD5 //	-0.13408	0.044829	0.090449
11747122_ALG9	0.148191	0.044838	0.090463

11741349_SLFN12L	0.161869	0.044845	0.090472
11760679_TNFRSF10C	-0.22583	0.044848	0.090475
11737547_CDH9	-0.13689	0.044856	0.090488
11735932_FRG2C ///	-0.17029	0.044866	0.090504
11719793_MAP3K11	-0.13088	0.04487	0.090509
11736680_SLC46A1	-0.2381	0.044895	0.090555
11725515_ABCG1	-0.29667	0.0449	0.090559
11722001_HNRNPU	-0.0808	0.0449	0.090559
11729489_ATP2B3	-0.1201	0.044905	0.090565
11758385_HEY2	-0.2968	0.044911	0.090572
11752689_OS9	0.115139	0.044918	0.090584
11753738_DIO1	-0.20802	0.044937	0.090617
11737907_PPP1CA	-0.08831	0.04494	0.090621
11753622_FGF4	-0.12466	0.044963	0.090661
11720156_ACAD10	-0.13475	0.044964	0.090661
11758816_RRP7A	0.141214	0.04497	0.090668
11737860_SEPT9	-0.09654	0.044985	0.090695
11747241_PPP2R5C	-0.0998	0.044988	0.090698
11737580_HLA-L	0.231621	0.045014	0.090747
11720239_MAPK9	-0.08049	0.045026	0.090768
11725763_PELI3	-0.13155	0.04503	0.090771
11739334_PTPRC	0.099382	0.045032	0.090771
11738283_WFDC9	-0.12846	0.045043	0.090787
11734519_PAGE2 ///	0.107637	0.045043	0.090787
11748959_ZNF559	0.113246	0.045046	0.090788
11752586_C19orf47	-0.1357	0.045052	0.090797
11736930_G6PC	-0.12758	0.045059	0.090808
11743127_CHCHD10	-0.1109	0.045074	0.090833
11743812_TFAM	0.088031	0.045154	0.090992
11728548_ZNF536	-0.09886	0.045166	0.09101
11719360_SLF2	0.15475	0.045167	0.09101
11735843_CCDC144A	-0.15654	0.045177	0.091021
11761768_PACRGL	-0.10039	0.045179	0.091021
11740050_ZNF461	0.207827	0.04518	0.091021
11726571_SEMA7A	0.279522	0.045182	0.091021
11724833_MUC13	0.114857	0.045182	0.091021
11741792_PARD6G-AS	-0.09982	0.045204	0.091063
11746387_SPIDR	-0.17851	0.04524	0.091131
11744874_CLN3	-0.09402	0.045248	0.091144
11746762_ZNF211	0.11457	0.04525	0.091144
11744128_CXCL2	0.124212	0.045279	0.091198
11723459_NLE1	0.112101	0.045297	0.09123
11760694_C3orf52	-0.11848	0.045298	0.09123
11745759_TERF1	-0.08967	0.045307	0.091243
11736126_CPLX2	-0.14466	0.045317	0.091259
11722664_GABRB3	-0.15839	0.045333	0.091287
11717719_ST6GAL1	-0.13947	0.045345	0.091309
11720652_BBX	0.090583	0.045348	0.09131
11736571_WDR31	-0.07986	0.045362	0.091335
11753024_NFIB	-0.12014	0.045367	0.091338
11739225_NF1	-0.09026	0.045367	0.091338

11738502_OR2L3 ///	0.234624	0.045373	0.091346
11724852_LRP3	-0.10414	0.045376	0.091347
11715895_SCARB2	-0.10529	0.045386	0.091364
11756619_C12orf43	0.080785	0.045406	0.091397
11722311_COQ2	0.098724	0.045408	0.091397
11721234_ZNF75D	0.097521	0.045409	0.091397
11760252_IFI44	0.210755	0.045409	0.091397
11721492_ANKRD7	0.219711	0.045421	0.091416
11755486_MAGT1	-0.10552	0.045429	0.091428
11728520_PLPP4	0.118695	0.045443	0.091453
11738655_FAHD1	-0.14751	0.045455	0.091473
11735946_ZPLD1	0.13506	0.045465	0.09149
11744255_ACADVL	-0.0883	0.045482	0.09152
11762428_METTL2B	-0.09463	0.045515	0.091583
11717262_C7orf50	-0.10193	0.045523	0.091595
11734234_HIST1H2AG	0.134065	0.045533	0.091612
11717954_MOGS	0.100583	0.045551	0.091645
11743628_IRS1	0.125169	0.045565	0.091668
11745615_TRIM15	-0.16193	0.045574	0.091683
11750654_ITPR1	-0.21743	0.045585	0.091701
11764113_LINC00581	0.18231	0.045618	0.091765
11759990_VSX1	-0.24527	0.045625	0.091773
11747026_SLC1A4	-0.13084	0.045628	0.091777
11761334_TROAP	-0.14468	0.045643	0.091799
11734323_PAQR7	-0.09516	0.045645	0.091799
11750214_RNF114	-0.1644	0.045645	0.091799
11763992_ATPAF1	0.260223	0.045647	0.091799
11736502_IL17RC	-0.08687	0.045658	0.091817
11729658_SP110	0.131507	0.04568	0.091858
11729208_PTPN11	0.109765	0.045691	0.091874
11757629_RPS29	0.07892	0.045692	0.091874
11721469_EFHC1	-0.12523	0.045699	0.091881
11729312_NMNAT1	0.153875	0.045699	0.091881
11724669_MRPS22	-0.10201	0.045704	0.091887
11757240_NFYC-AS1	0.123381	0.045711	0.091897
11721790_PTPRD	-0.16441	0.045718	0.091908
11759392_KRTAP21-2	-0.13643	0.045732	0.091933
11758353_APOA1	-0.15572	0.045759	0.091983
11727512_UBN2	0.137955	0.04577	0.092002
11760782_MGAT4EP	-0.1127	0.045778	0.09201
11744979_RNF123	0.142728	0.045778	0.09201
11752690_RBCK1	0.121121	0.0458	0.092051
11761229_CD79B	-0.23508	0.045803	0.092052
11737456_CXorf66	-0.15491	0.045813	0.092069
11739091_LUC7L3	0.088962	0.045823	0.092084
11764115_LOC101929	-0.18446	0.045833	0.092101
11715876_P2RX5-TAX	-0.09488	0.045838	0.092108
11758692_TRIM24	0.105043	0.045846	0.09212
11757521_TCEA2	-0.1183	0.045854	0.092132
11755548_OSBPL8	0.135157	0.045864	0.092143
11745447_SAC3D1	-0.10163	0.045867	0.092143

11724386_C20orf196	-0.11856	0.045867	0.092143
11759790_SPPL2B	0.134895	0.045867	0.092143
11761431_PGAM5 ///	-0.09852	0.045881	0.092167
11741558_HOXB6	-0.13922	0.045889	0.09218
11731409_SLC8A2	-0.15805	0.045898	0.092194
11762650_LOC100289	-0.10031	0.0459	0.092194
11742042_IRAK4	0.145494	0.045902	0.092194
11735442_ZNF781	0.272788	0.045903	0.092194
11730920_SLC10A7	0.094136	0.045906	0.092194
11745730_POLR1E	0.1273	0.045926	0.092231
11735439_NRXN2	-0.11138	0.045929	0.092233
11759327_ATP8A2	0.160923	0.045957	0.092286
11755400_STK39	0.171442	0.045975	0.092319
11754661_CAMK1G	-0.14465	0.04598	0.092324
11742158_OR52A1	-0.18166	0.045987	0.092336
11727823_TPMT	0.128859	0.046022	0.092401
11743677_P2RY8	-0.14669	0.046024	0.092402
11757953_TTC39C	-0.13724	0.046028	0.092406
11735112_PTGER3	0.171996	0.046036	0.092417
11747388_CTNNB1	0.088623	0.046038	0.092417
11753233_NDRG1	-0.08779	0.046046	0.092431
11738346_ZNF713	-0.12495	0.046073	0.092479
11760488_SNTB1	-0.17575	0.046074	0.092479
11729833_EIF5A2	-0.16546	0.04609	0.092507
11750367_CPA2	0.208439	0.046108	0.092539
11715418_SKP1	-0.11732	0.04611	0.09254
11729867_UGT3A1	-0.09589	0.046131	0.092578
11764253_IFITM10	0.158092	0.046151	0.092614
11758362_EXTL2	-0.09308	0.046154	0.092618
11724002_DPP6	-0.11535	0.046157	0.09262
11746875_GNAI2	-0.09995	0.046163	0.092628
11750931_RGS6	-0.22424	0.046172	0.092641
11723974_APOL3	0.159407	0.046178	0.092649
11731883_CCDC150	-0.25055	0.046182	0.092654
11731050_CEP126	-0.11592	0.046209	0.092705
11736448_GIN1	-0.09646	0.046212	0.092707
11741321_CREM	-0.22103	0.046243	0.092765
11742105_CASP10	-0.16022	0.046247	0.092769
11742321_EDA	-0.16235	0.046271	0.092813
11718776_SRRM1	0.087418	0.046278	0.092823
11729126_SLC25A3	0.081057	0.046288	0.092839
11716824_BDH2	-0.24387	0.046302	0.092865
11756012_SULT6B1	-0.17251	0.046335	0.092927
11758345_CCL3P1 //	0.216054	0.046344	0.092942
11754265_YIPF6	-0.07965	0.046349	0.092947
11754371_FAM102A	-0.08498	0.046367	0.09298
11732854_SCARF1	0.226795	0.046371	0.092983
11751819_PPP2R3A	0.097928	0.046386	0.093009
11734694_NECAB1	0.156952	0.046398	0.09303
11734610_KRTAP5-7	-0.10566	0.046402	0.093034
11743144_COX11	-0.10118	0.046407	0.09304

11757892_EXOC1	0.083981	0.046431	0.093084
11751519_TMED4	0.107551	0.046435	0.093089
11747694_ANO9	-0.20835	0.046455	0.093126
11729127_SLC25A3	0.07988	0.046458	0.093127
11746774_SLC27A1	-0.18773	0.046478	0.093162
11760387_IL16	-0.31259	0.046479	0.093162
11762301_WRAP73	-0.15808	0.046492	0.093185
11747784_LOC100287	-0.11489	0.046496	0.093189
11758937_EIF3B	-0.25802	0.046506	0.093205
11743936_FIP1L1	-0.09661	0.046523	0.093235
11735089_MAP3K12	0.122397	0.046536	0.093258
11748126_TIMP2	0.087422	0.046552	0.093285
11759936_SEC13	-0.08813	0.046556	0.09329
11734616_PRR23A	-0.16873	0.046566	0.093305
11734638_OR51B4	-0.08942	0.046567	0.093305
11715503_LASP1	0.086844	0.04657	0.093307
11757333_NUDT4 ///	-0.09415	0.046574	0.093311
11738612_OR2B2	0.175716	0.046589	0.093337
11732896_HSD11B1L	-0.09391	0.046603	0.09336
11762880_NARF	-0.11345	0.04662	0.093392
11717465_LXN	-0.12695	0.046624	0.093396
11759114_LOC100506	0.111014	0.046626	0.093396
11758893_MLXIP	-0.10021	0.046641	0.093422
11739351_AMOT	0.082492	0.046643	0.093422
11734126_NOD1	-0.13983	0.046649	0.09343
11747812_EGR3	0.08855	0.046656	0.09344
11760389_PI4KAP2	-0.09659	0.04666	0.093444
11732009_QRICH2	-0.1007	0.046673	0.093467
11740629_ZNF611	-0.136	0.046679	0.093472
11739449_ROCK1	0.150917	0.04668	0.093472
11724724_EIF5	0.096601	0.046681	0.093472
11736376_MPP3	-0.16564	0.046691	0.093487
11762739_PSTPIP1	-0.14044	0.046699	0.093499
11757123_SNORA74B	-0.09298	0.046705	0.093507
11733326_NKD1	-0.13501	0.04671	0.093514
11738991_ATP6V0E2	-0.12872	0.046721	0.093532
11751481_PRMT6	-0.09984	0.04673	0.093546
11737912_TM2D3	-0.13092	0.046734	0.093551
11724016_FRA10AC1	-0.0823	0.046736	0.093551
11755149_MEF2D	0.143104	0.04674	0.093555
11718336_LMOD1	-0.17559	0.046754	0.093578
11732329_MAP7D1	-0.09312	0.04676	0.093584
11717654_CMC4 ///	-0.09454	0.04676	0.093584
11731179_ESPN	-0.11106	0.04678	0.093619
11755731_KLK3	-0.21908	0.046782	0.093621
11740388_TFAP2A	-0.14169	0.046796	0.093644
11750357_ETNK2	-0.12315	0.046815	0.093678
11738254_GREB1	-0.32759	0.046836	0.093716
11763993_ATPAF1	0.097276	0.046849	0.093739
11722050_CC2D1A	-0.10616	0.04687	0.093774
11731088_TM9SF1	-0.12247	0.046871	0.093774

11741523_SLC35G4 /	-0.09752	0.046882	0.093794
11726831_ANTXR1	0.165818	0.046889	0.093804
11741839_RBBP8NL	-0.1318	0.046898	0.093818
11726599_PLPPR1	-0.14199	0.046908	0.093832
11749123_HIF1A	-0.08863	0.046939	0.093891
11745286_EIF4E3	0.146993	0.046953	0.093915
11758898_APOBEC3C	0.096469	0.046955	0.093915
11754755_POLR3B	0.121561	0.046979	0.093957
11723796_SFMBT1	0.169658	0.04698	0.093957
11717309_WDR75	-0.10285	0.04699	0.093971
11732562_FZD3	0.17193	0.04699	0.093971
11735700_SLC13A2	-0.16361	0.04702	0.094026
11743877_PPP1R1C	0.147133	0.047025	0.094032
11741350_ANXA11	0.084204	0.047051	0.094081
11744448_MSRA	0.122406	0.047062	0.0941
11732640_JKAMP	0.096377	0.047069	0.094108
11724656_MAP4K1	-0.15574	0.047116	0.094196
11758860_HNRNPU	-0.54911	0.047116	0.094196
11730918_SLC10A7	-0.13768	0.047121	0.094201
11763198_PHACTR1	-0.14147	0.047125	0.094205
11754677_FAM169A	0.105384	0.047126	0.094205
11742716_TBRG1	0.096719	0.047153	0.094254
11754254_MAP7D2	-0.14848	0.047164	0.094272
11726366_ERICH1	0.091274	0.047167	0.094273
11758160_EEF2KMT /	-0.0931	0.047179	0.094292
11760613_METTL3	-0.18639	0.04718	0.094292
11760303_HEXA	0.127492	0.047203	0.094335
11756235_DGCR2	-0.15428	0.04722	0.094359
11735283_KMT2E	0.116744	0.047221	0.094359
11747422_WBSCR22	0.105433	0.047221	0.094359
11751168_EGFL8 ///	-0.28758	0.047231	0.094374
11756922_VASN	-0.11394	0.047257	0.094422
11743540_ACAD9	-0.07975	0.047265	0.094432
11747330_ACAD9	-0.07975	0.047265	0.094432
11738186_PDE8B	-0.19229	0.047281	0.09446
11730647_SIRPD	-0.13421	0.047297	0.094488
11748336_GIF	0.096456	0.047299	0.094488
11746640_RASSF2	-0.1027	0.047301	0.094489
11753353_SLC47A1	-0.14474	0.047316	0.094513
11746999_CDHR3	-0.15667	0.047323	0.094525
11731938_FTCD	-0.10072	0.04733	0.094535
11738849_SERINC5	-0.2087	0.047339	0.094549
11742887_BAG5	-0.11547	0.047358	0.094583
11725632_NR4A2	-0.18786	0.047362	0.094586
11722374_GGA2	0.103722	0.047365	0.094588
11746080_MYO3B	-0.17502	0.047387	0.094628
11715341_MROH9	-0.26016	0.047395	0.094636
11735923_AMELY	-0.11685	0.047395	0.094636
11757433_TRIM27	0.113217	0.047397	0.094637
11742242_PCSK6	-0.10804	0.0474	0.09464
11733490_ZNF704	0.122141	0.047403	0.094642

11746401_ZSWIM3	0.113947	0.047408	0.094647
11732119_USP10	0.094363	0.047414	0.094656
11732017_IL18RAP	-0.15194	0.047421	0.094666
11734120_THRA	-0.08772	0.047444	0.094708
11730909_CCR5	-0.15968	0.047447	0.09471
11754065_ORM1	-0.12875	0.047449	0.09471
11734895_CTC1	-0.15688	0.047452	0.09471
11737229_TGIF2LX /	-0.14295	0.047453	0.09471
11718730_CCDC97	0.088589	0.047457	0.094713
11742671_GH1	-0.15675	0.047458	0.094713
11752551_AMOT	-0.16964	0.047493	0.094779
11721529_ZDHHC2	0.10847	0.047503	0.094795
11743799_SPAG17	0.096569	0.047507	0.094798
11723163_NR3C2	-0.12762	0.047513	0.094807
11718082_ATP2B4	0.137485	0.047518	0.094813
11734745_ALAS2	-0.11934	0.04752	0.094813
11735472_CCDC169 /	0.205717	0.047525	0.09482
11747955_PDZD3	-0.08332	0.047559	0.094884
11720722_REXO1	0.080698	0.047589	0.094939
11740990_HYAL1	-0.19425	0.047597	0.09495
11722986_UBOX5	0.157387	0.047598	0.09495
11753799_SMIM11A /	0.133494	0.047609	0.094968
11744744_SLITRK3	-0.27191	0.047612	0.094969
11727299_CASC10	0.157337	0.047615	0.094972
11718259_NOTCH1	-0.09482	0.047619	0.094976
11756259_NFATC1	-0.12111	0.047669	0.095069
11716488_PIGT	-0.08713	0.04767	0.095069
11718535_NKTR	0.078564	0.047673	0.095073
11760779_MMP19	0.123414	0.04772	0.095161
11750540_KCNMB2	-0.12199	0.04777	0.095257
11763025_MUC1	-0.11588	0.047783	0.095273
11736270_NANP	0.10399	0.047784	0.095273
11746169_LOC440173	-0.21624	0.047784	0.095273
11750199_HCLS1	-0.11988	0.047796	0.09529
11754504_AGAP4 ///	0.100963	0.047796	0.09529
11760955_NDUFA8	-0.08831	0.047825	0.095341
11733585_TEX29	-0.13708	0.047825	0.095341
11729277_CRCP	0.105027	0.047834	0.095354
11732647_PCDH8	0.15009	0.047843	0.095368
11729856_CNFN	-0.13477	0.047873	0.095424
11742545_OR5R1	-0.20677	0.047878	0.09543
11737124_TRAPPC3L	-0.21796	0.047882	0.095434
11738530_PCDHGA6	0.132188	0.047889	0.095444
11721971_JADE3	0.088091	0.047904	0.09547
11753900_MT2A	-0.08122	0.047908	0.095475
11744407_GOLGA8A	0.094504	0.047922	0.095498
11763980_SRRT	0.091771	0.047937	0.095523
11736718_NCALD	0.209229	0.047953	0.095553
11755107_RIN3	-0.094	0.047955	0.095553
11726117_MTAP	-0.08513	0.047959	0.095556
11751976_LRP2	-0.14299	0.047966	0.095566

11739589_DRAXIN	-0.09574	0.047981	0.095592
11760028_MCF2L	-0.16326	0.047983	0.095592
11732518_GPR52	0.120504	0.04799	0.0956
11738487_PSKH2	-0.19866	0.047991	0.0956
11762424_BRD4	-0.08362	0.047993	0.0956
11733113_MANBAL	0.094315	0.047999	0.095608
11728892_TPPP	-0.20484	0.048004	0.095615
11761068_AKR1C1 //	0.154228	0.048053	0.095709
11744422_CRADD	-0.07797	0.048059	0.095716
11736321_ANKRD26	0.093032	0.048066	0.095727
11754218_SLC45A4	0.168212	0.04807	0.095731
11726451_B3GAT1	-0.15829	0.048076	0.095738
11731773_KIAA1161	-0.11349	0.048086	0.095755
11734626_PRY /// F	-0.13921	0.048094	0.095766
11725205_RPH3AL	-0.11001	0.048119	0.095813
11760655_LRP3	-0.20688	0.048132	0.095835
11731800_C3orf70	0.128247	0.048148	0.095863
11759487_ARHGEF40	-0.2193	0.048156	0.095875
11752021_ADGRF3	-0.15619	0.048208	0.095969
11761568_TMC6	-0.13924	0.048209	0.095969
11717379_PUF60	-0.08636	0.048209	0.095969
11737844_FAM102A	-0.14479	0.048226	0.09599
11749888_ORAI3	-0.13536	0.048227	0.09599
11753753_TTTY6 ///	-0.14735	0.048228	0.09599
11736435_SEMA3E	0.234516	0.048228	0.09599
11720036_CASP2	0.145551	0.04823	0.09599
11763102_FCN2	-0.14919	0.048256	0.096038
11731979_FNDC7	-0.26419	0.048261	0.096045
11718133_CDK5RAP1	-0.10628	0.048293	0.096104
11763106_TTTY6B	0.228089	0.048314	0.096142
11738455_C20orf197	-0.18589	0.048317	0.096144
11758688_OLA1	0.083216	0.048321	0.096148
11748042_KMT5A	-0.09847	0.048327	0.096157
11733559_EDAR	-0.14324	0.048329	0.096157
11760598_ATP5L2	-0.09282	0.048343	0.096181
11745280_ANKRD19P	0.159794	0.048353	0.096196
11731405_ATP6V1G1	-0.24135	0.048355	0.096197
11741585_DNAJC28	-0.16347	0.048391	0.096264
11724771_FCGR2A //	0.116704	0.048403	0.096278
11753089_OGFR	-0.10928	0.048403	0.096278
11753547_C9orf66	-0.12564	0.048405	0.096278
11717837_AIF1	-0.18972	0.048406	0.096278
11722602_PLEKHF1	0.130449	0.048414	0.09629
11730433_ZNF341	-0.20659	0.048421	0.0963
11741087_RTKN	-0.09238	0.048434	0.096321
11741371_GRID2IP	-0.14638	0.048442	0.096334
11743493_KTN1	0.126918	0.048444	0.096334
11730763_CRIPT	0.126923	0.048448	0.096339
11734101_ZNF578	-0.13172	0.048454	0.096345
11725876_GPIHBP1	-0.15772	0.048456	0.096346
11742539_OR10X1	-0.19884	0.04848	0.09639

11748917_GPD1	-0.09619	0.048511	0.096449
11728699_BMPR2	0.142991	0.048525	0.096472
11725603_HOXA10-HC	-0.22865	0.04853	0.096477
11757997_OAT	-0.12104	0.048545	0.096503
11717878_PPFIA1	0.107053	0.048563	0.096536
11734762_HIP1	-0.12545	0.04857	0.096543
11732600_CHRNB2	-0.14195	0.048571	0.096543
11724164_RUBCN	-0.24243	0.048573	0.096544
11720197_TOMM40L	0.126729	0.048594	0.096579
11740093_NTRK2	-0.16354	0.048597	0.096579
11743539_ACAD9	-0.11851	0.048597	0.096579
11735846_ASB14	-0.14267	0.048599	0.096579
11718498_F8A1 ///	-0.08716	0.048618	0.096613
11717664_KLHL12	-0.10118	0.04864	0.096651
11728605_LIMS1 ///	-0.10277	0.048641	0.096651
11741070_ZNF146	0.095772	0.048678	0.096722
11754163_IL7	-0.10794	0.048686	0.096732
11746761_PXK	-0.11757	0.048708	0.096771
11762016_CDK15	-0.13635	0.048709	0.096771
11724052_PIGQ	-0.11788	0.048712	0.096773
11730082_GFI1B	-0.11357	0.048718	0.096778
11730815_LILRB4	-0.20675	0.048719	0.096778
11751991_MOV10L1	-0.10252	0.048721	0.096778
11720465_SKIV2L2	-0.08376	0.048741	0.096814
11743471_WDFY3	0.142087	0.048824	0.096975
11735032_UPRT	-0.24952	0.048865	0.097052
11716458_MRPS35	0.080799	0.048874	0.097066
11717008_BNIP3L	0.091345	0.04888	0.097075
11756199_ATMIN	0.110499	0.048893	0.097098
11719117_GOT2	-0.0805	0.048917	0.09714
11726809_SLC12A8	0.116702	0.048926	0.097155
11725214_HAAO	-0.10559	0.048933	0.097165
11751593_STX1A	0.110188	0.048955	0.097203
11731493_ITCH	-0.09442	0.048972	0.097234
11715290_CLDN3	-0.09694	0.048974	0.097234
11755722_RPP21 ///	-0.13088	0.048977	0.097235
11717567_NQO1	0.078529	0.049002	0.097282
11745223_ATP2C1	-0.13578	0.049013	0.097299
11752896_MIF4GD	-0.08249	0.049016	0.097299
11754652_ECI2	-0.09397	0.049017	0.097299
11715370_LGALS3BP	0.087043	0.049026	0.097313
11723068_CRHBP	0.229353	0.04903	0.097317
11754994_SART1	0.101948	0.049042	0.097337
11737606_ZNF438	0.17309	0.04906	0.09737
11729553_PRR7	-0.09797	0.049068	0.097381
11726493_ZNF775	-0.14018	0.049084	0.097409
11730283_SMU1	0.160651	0.049118	0.097469
11751054_SPOCK3	-0.21467	0.049118	0.097469
11731966_PODNL1	-0.10716	0.049123	0.097473
11749183_ALDH4A1	-0.12327	0.049124	0.097473
11715273_TPM2	0.147253	0.04913	0.097473

11748018_KCNJ13	-0.10767	0.04913	0.097473
11730695_TBC1D26	-0.14873	0.04913	0.097473
11740459_TRIM39 //	-0.11667	0.049145	0.097498
11758400_IGF2 ///	-0.15267	0.049153	0.097511
11719252_DNAJB5	-0.12538	0.049157	0.097514
11730280_RAD50	0.194041	0.049186	0.097564
11741026_PIP5K1A	0.14711	0.049186	0.097564
11759118_FKBP14	0.123285	0.049221	0.097629
11762685_LILRB3	-0.11832	0.049241	0.097664
11750849_RIPK1	-0.12045	0.049248	0.097674
11734325_CADM2	-0.08373	0.04925	0.097674
11721160_TARBP2	0.087086	0.049256	0.097682
11726018_FAM222B	0.088312	0.049264	0.097695
11748498_ANKRD19P	0.194563	0.049273	0.097709
11729149_MRPS23	-0.07931	0.049294	0.097747
11760762_DENND2A	-0.13157	0.049299	0.097752
11762719_IREB2	-0.17258	0.049303	0.097757
11733178_ZBTB1	-0.11699	0.049307	0.097761
11742686_FCGRT	-0.18271	0.049368	0.097876
11734435_TMEM132E	-0.12336	0.049387	0.09791
11748046_SLC44A4	-0.10737	0.049444	0.098019
11741407_SFMBT2	0.111744	0.049476	0.098079
11725217_WDSUB1	0.139743	0.049479	0.098082
11761724_SCGB2A2	0.180826	0.049487	0.098093
11763983_SNRPC	-0.13854	0.049492	0.098099
11745570_LSP1P3	0.100109	0.049498	0.098099
11752794_STAT2	-0.09547	0.049498	0.098099
11750449_GRM2	0.131551	0.049502	0.098099
11741890_NKX2-4	-0.10442	0.049502	0.098099
11726187_SHISA3	-0.09851	0.049502	0.098099
11715498_OXA1L	0.096658	0.049523	0.098136
11731785_IL9	-0.14837	0.049534	0.09815
11734512_ASCL4	-0.144	0.049536	0.09815
11760783_FN3K	-0.14136	0.049536	0.09815
11725322_ZNHIT2	0.121984	0.049546	0.098167
11715913_EFNB1	-0.13274	0.049549	0.098168
11728202_DNTTIP2	0.092176	0.049565	0.098195
11721793_RGMB	-0.19963	0.049581	0.09822
11732213_ZKSCAN1	0.102695	0.049581	0.09822
11723743_CADM3	-0.12241	0.049628	0.098309
11741238_COPE	0.080265	0.049639	0.098326
11747488_TMX2	0.087286	0.049646	0.098336
11756169_OAZ1	-0.07886	0.049658	0.098356
11760377_EFCAB11	-0.14196	0.049664	0.098364
11742450_COL13A1	0.130534	0.049675	0.098378
11758337_ADA	0.096883	0.049675	0.098378
11759430_KLF7	0.169316	0.049682	0.098387
11757603_NTNG1	0.174896	0.049685	0.09839
11742068_CALHM1	-0.1204	0.049693	0.098402
11727982_SNX21	-0.19984	0.049704	0.098418
11723910_CMKLR1	-0.14939	0.049711	0.098428

11722997_NOL11	-0.09198	0.049737	0.098475
11747677_BZW2	0.09621	0.049738	0.098475
11723952_KRT1	-0.14589	0.04976	0.098515
11715785_CRYBB2	-0.13835	0.049766	0.098522
11732815_CPN2	-0.12864	0.049779	0.098544
11750549_NPHS2	-0.13186	0.049794	0.098569
11722980_F8	-0.20699	0.049799	0.098575
11732289_TRMT61A	-0.10502	0.049803	0.098575
11721449_ETF1	0.100594	0.049803	0.098575
11734422_C17orf98	-0.1546	0.049818	0.098602
11741776_TMEM179	-0.15263	0.049831	0.098623
11762160_AKAP8	-0.20501	0.049842	0.09864
11735627_ZNF841	0.104344	0.049847	0.098647
11719636_NUP153	0.104936	0.049865	0.098678
11732435_SCIMP	-0.1105	0.049868	0.098679
11717241_MAEA	0.082855	0.049887	0.098713
11717983_GGPS1	-0.11746	0.049892	0.098719
11739948_PFKFB2	-0.14385	0.049894	0.098719
11738975_DEFB130 /	-0.16409	0.049896	0.09872
11734083_PDHA2	-0.13972	0.049902	0.098727
11752242_SBNO2	0.136444	0.049915	0.098748
11749015_VASH2	-0.12006	0.049951	0.098816
11734637_TAS2R60	-0.15262	0.049969	0.098848
11735262_CATSPER2	-0.21433	0.049971	0.098848
11752601_MRC1	-0.21152	0.049985	0.098872
11763537_RAD51D	-0.0959	0.049997	0.098891
11757985_PDE7B	0.175491	0.050021	0.098935
11750745_HSPA9	0.082631	0.050023	0.098935
11743983_SH3GLB2	-0.10325	0.05003	0.098943
11749562_MTMR11	0.160248	0.050031	0.098943
11742746_PLIN2	-0.08489	0.050036	0.098949
11735008_ZNF555	0.105295	0.050061	0.098995
11739885_DOCK7	0.157625	0.050072	0.099011
11741206_SCAMP5	-0.10865	0.050108	0.09908
11761695_MRPL48	0.107005	0.050134	0.099127
11731096_WNT10A	-0.15289	0.050139	0.099132
11751444_TRABD	-0.10282	0.050156	0.099161
11757491_ROMO1	0.083209	0.050198	0.099241
11762054_HNRNPC	-0.15874	0.050205	0.099251
11734539_MS4A15	-0.18707	0.050216	0.099268
11746947_PTPRA	-0.12275	0.050222	0.099276
11717534_TMEM259	-0.12392	0.050249	0.099326
11716989_PNRC2	0.087126	0.050251	0.099326
11727962_ING1	-0.09566	0.05026	0.09934
11731607_SCIN	-0.14945	0.050275	0.099365
11763725_HNRNPU	-0.10061	0.050288	0.099387
11721968_IGSF11	-0.17757	0.05031	0.099426
11716489_STATH	-0.16707	0.050314	0.099429
11758550_C9orf91	-0.11271	0.050321	0.099439
11732315_SGCD	-0.12945	0.050331	0.099455
11732627_NEMP2	-0.11867	0.050356	0.099501

11728006_PRAC1	-0.12785	0.050385	0.099554
11756057_MED16	-0.10019	0.050408	0.099595
11718839_MON1B	-0.09776	0.050413	0.099601
11761402_ISCU	0.124703	0.05043	0.099631
11724372_ANXA10	0.149018	0.050452	0.09967
11733459_PSG6	-0.09958	0.050455	0.099673
11747150_SLC28A1	-0.18987	0.050459	0.099676
11723531_GOSR1	0.106692	0.050497	0.099746
11743458_FAM49A	-0.13846	0.050518	0.099784
11717226_CBFA2T2	-0.10027	0.050548	0.099839
11723467_NDUFA3	0.082373	0.050562	0.099864
11736018_PXN	0.115245	0.050576	0.099887
11743388_NAGPA	-0.11434	0.050579	0.099888
11729254_NKAIN2	-0.09522	0.050587	0.099899
11722750_AKAP8	0.097622	0.050588	0.099899
11741495_KCNQ3	-0.09104	0.050616	0.099949
11740251_SPTLC1	0.101866	0.050636	0.099985
11763899_SNORA70B	0.194125	0.050639	0.099986
11755841_KIF26B	0.106343	0.050646	0.099993
11729591_KLK2	-0.07784	0.050646	0.099993
11753066_PAH	-0.16777	0.050656	0.100009
11756276_NUP210	-0.19738	0.050666	0.100025
11716340_NDUFS4	-0.08428	0.050675	0.100038
200062_PMRPL30	0.075856	0.050727	0.100137
11731462_CPM	0.217491	0.050735	0.100144
11756888_TFCP2L1	-0.18949	0.050735	0.100144
11729980_PLCXD3	-0.1603	0.050742	0.100147
11718925_WWP2	0.089615	0.050742	0.100147
11761605_SPTY2D1	0.09198	0.050743	0.100147
11729068_MYH8	0.119222	0.050748	0.100155
11721110_NAP1L3	-0.17595	0.050755	0.100163
11721240_ZNF787	-0.13285	0.050777	0.100202
11752801_SP110	0.09519	0.050783	0.100211
11729069_KLK11	0.17474	0.050786	0.100213
11761261_NAAA	0.170504	0.050791	0.100218
11727990_FAXC	0.116259	0.050798	0.100227
11724361_PIKFYVE	0.092303	0.050803	0.100227
11751390_CYSLTR2	0.29996	0.050803	0.100227
11759128_CD300E	-0.12082	0.050804	0.100227
11741493_PEX5	-0.14674	0.050807	0.100228
11727018_PAPPA	0.208504	0.050808	0.100228
11729046_TCERG1L	0.09226	0.050828	0.100262
11738040_ZNF630	0.18451	0.050839	0.100274
11721023_DIDO1	-0.11003	0.050841	0.100274
11752276_DIDO1	-0.11003	0.050841	0.100274
11745024_A4GALT	-0.08876	0.050842	0.100274
11753446_HMOX1	0.089094	0.050852	0.10029
11716439_TAF7	-0.09523	0.050869	0.100319
11717786_CLUH	-0.15122	0.050881	0.10034
11716929_DGCR2	0.213422	0.050915	0.100402
11739633_MED13	0.093041	0.05095	0.100468

11730037_DEGS2	-0.11606	0.050953	0.100469
11744772_MLANA	-0.17139	0.050974	0.100503
11751894_GPR155	-0.21722	0.050975	0.100503
11735764_NOSTRIN	-0.15189	0.050978	0.100506
11719909_MLLT10 //	0.096701	0.050983	0.100511
11730311_TFCP2L1	-0.17463	0.050991	0.100524
11749212_ITGBL1	0.210578	0.051013	0.100564
11763821_CHST1	-0.17031	0.051031	0.100595
11743550_BRWD1	0.148346	0.051038	0.100604
11735603_OSBPL7	-0.10854	0.05104	0.100604
11722479_CUL5	-0.12903	0.051045	0.10061
11737379_MTNR1B	-0.19842	0.051053	0.100621
11756781_OPALIN	-0.17494	0.051056	0.100624
11733597_AWAT2	-0.12462	0.051068	0.100644
11715310_MLXIPL	-0.14223	0.051075	0.100653
11744884_NCOR1	0.106456	0.05108	0.100658
11719350_GCC2	-0.17481	0.051087	0.100667
11724008_ENTPD1	-0.15136	0.05109	0.100667
11745927_ADAM15	-0.12034	0.051091	0.100667
11756130_GSK3A	-0.09088	0.051095	0.100671
11763982_SNRPC	-0.139	0.051115	0.100707
11757640_ART3	-0.09626	0.051117	0.100708
11733158_NOP9	-0.09561	0.051125	0.100719
11751312_BAIAP3	-0.09715	0.051128	0.100721
11753234_TAMM41	0.092758	0.051138	0.100736
11738069_HAS3	-0.17894	0.051146	0.100748
11761762_FSD1	-0.1105	0.051153	0.100758
11760505_LOC100506	-0.11299	0.051158	0.100764
11745475_MAML3	-0.09574	0.051185	0.100813
11718979_HTATIP2	0.092811	0.051201	0.10084
11733267_PIRT	-0.12165	0.051222	0.100872
11745138_FAM223A /	-0.10094	0.051222	0.100872
11743023_RTN4RL2	-0.12276	0.051223	0.100872
11740544_NRG1	-0.12946	0.051256	0.100932
11741240_APAF1	-0.09508	0.051261	0.100937
11729540_NCAPH2	-0.11824	0.051264	0.100939
11738657_CDKL4	-0.14023	0.051277	0.10096
11721311_SRSF10	0.0929	0.051283	0.100969
11740486_RUNX3	-0.08712	0.05131	0.101019
11724206_MAD1L1	-0.08989	0.051314	0.10102
11741533_EVC2	0.120859	0.051315	0.10102
11720777_POLL	-0.09058	0.051324	0.101033
11722039_FES	-0.11264	0.051371	0.101123
11742595_OR51F1	-0.23238	0.051373	0.101123
11747731_DBF4	-0.11957	0.051386	0.101144
11758679_NOP56	0.078099	0.051408	0.101182
11762767_NGRN	0.141673	0.051428	0.101218
11736720_CCR4	-0.14057	0.051437	0.101232
11736779_ZNF486	0.099598	0.051439	0.101232
11751009_IK	-0.08538	0.051444	0.101237
11742050_API5	0.118373	0.051487	0.101316

11735831_FGF21	-0.165	0.051488	0.101316
11754095_TAGLN	-0.15288	0.051495	0.101325
11739532_DAW1	0.117245	0.051499	0.10133
11729972_CYP4Z1	0.183038	0.051502	0.10133
11742027_ANGPT2	-0.22651	0.05151	0.101343
11723269_KPNA6	-0.12155	0.051519	0.101356
11726910_MRI1	0.084181	0.051542	0.101398
11733191_FAAP24	-0.1639	0.051546	0.101401
11756040_ANGPTL8	-0.20824	0.051557	0.101417
11755244_ADAMTS13	-0.13728	0.051558	0.101417
11718421_TMEM115	0.077686	0.051567	0.10143
11760903_RSAD1	-0.1941	0.051583	0.101458
11730569_MS4A5	-0.22251	0.0516	0.10148
11734660_CLSTN3	-0.13995	0.0516	0.10148
11755572_MAP7D1	-0.08365	0.051601	0.10148
11748159_PHLDB1	0.083467	0.051624	0.101522
11721414_TMEM62	0.142089	0.051626	0.101522
11749606_LOC399815	-0.13151	0.05164	0.101546
11727202_ZNF230	0.121358	0.051673	0.101605
11741945_NHSL2	0.202771	0.051675	0.101605
11746101_PQLC1	-0.11992	0.051679	0.101607
11740336_GPATCH11	-0.12759	0.05168	0.101607
11727566_OTULIN	0.107157	0.051683	0.101608
11731971_XKR4	-0.23914	0.051684	0.101608
11757058_TSPO	0.082557	0.051688	0.101611
11755988_AIPL1	-0.13786	0.05173	0.101689
11737198_TMPRSS15	-0.15599	0.051732	0.10169
11750504_ACY3	-0.14614	0.051736	0.101693
11740207_GPR155	-0.18005	0.051743	0.101702
11732982_OR2J2	-0.10839	0.051753	0.101716
11724721_GALNT12	-0.19476	0.051754	0.101716
11752132_DLG2	-0.10921	0.051761	0.101726
11721931_TMCC2	-0.14054	0.051774	0.101748
11740034_PCDH1	-0.17509	0.051787	0.101768
11730103_SCN9A	0.218011	0.051825	0.101839
11718526_ESRRA	-0.10421	0.051843	0.10187
11747061_COG2	-0.10398	0.051845	0.10187
11729903_AGFG2	-0.11248	0.051848	0.101873
11733748_GPR63	0.134101	0.051859	0.101889
11728001_TMEM114	-0.14579	0.051869	0.101904
11737473_ZNF213	0.094449	0.051883	0.101928
11746832_ARID1A	-0.15761	0.051914	0.101984
11726510_SMPD3	-0.15768	0.051917	0.101984
11735608_C12orf56	0.106659	0.051917	0.101984
11724330_DGCR8	-0.1154	0.051941	0.102026
11748936_POMT1	0.081558	0.051983	0.102104
11743952_CFAP206	0.167489	0.051986	0.102107
11723626_ENHO	-0.09056	0.052016	0.10216
11761312_EDC4	-0.11532	0.052034	0.102189
11731352_IL19	-0.12091	0.052034	0.102189
11724395_ZNF83	-0.09704	0.052055	0.102226

11763017_HSPD1	0.164806	0.052058	0.102227
11751655_OAZ1	-0.0944	0.05206	0.102228
11741418_KCNQ1	-0.17594	0.052064	0.10223
11743998_SSBP4	-0.10483	0.052075	0.102248
11737554_GJD4	-0.13915	0.052084	0.102262
11763668_FER	-0.29164	0.052086	0.102263
11751092_LOC101928	0.118115	0.052092	0.10227
11728492_KANK4	-0.1283	0.052112	0.102305
11761182_NACAP1	-0.28895	0.052145	0.102365
11757531_HSPD1	-0.07819	0.052148	0.102367
200006_PMPARK7	0.07607	0.052153	0.102368
11750873_RAB11FIP1	-0.13076	0.052154	0.102368
11720266_TBCK	-0.12051	0.052155	0.102368
11724156_SULT1E1	0.124647	0.052163	0.102379
11716434_SCD5	-0.15409	0.052166	0.102379
11718935_STK19	0.115222	0.052167	0.102379
11738724_OR4D6	-0.11083	0.052202	0.102445
11759872_PDXDC1	-0.2538	0.052223	0.102481
11734819_APBB2	0.115839	0.052242	0.102515
11762060_CD55	0.130156	0.052268	0.102561
11738829_C1orf109	0.080889	0.052278	0.102576
11722116_RARB	0.083964	0.05229	0.102596
11749244_FAM98A	0.11697	0.052294	0.102601
11759998_SLC3A1	0.126319	0.052297	0.102601
11728527_PEX5L	-0.0903	0.052301	0.102605
11751806_STARD4	0.157843	0.052306	0.102609
11760994_WARS	0.242387	0.052308	0.102609
11729060_DNAH5	0.122459	0.052309	0.102609
11751792_MLXIP	-0.11435	0.052319	0.102622
11734731_MRPS12	-0.10351	0.05232	0.102622
11750807_KIRREL	-0.09896	0.05234	0.102654
11720445_NSD1	0.131357	0.05234	0.102654
11738600_KRTAP5-6	-0.15897	0.052345	0.102654
11745023_SLC25A27	0.115069	0.052345	0.102654
11718716_PKD1	-0.10268	0.052347	0.102654
11762519_ERVMER61-	-0.14613	0.052351	0.102658
11744771_TTTY12	-0.16515	0.052368	0.102687
11758839_CA12	-0.21489	0.052381	0.102708
11754247_ATRX	0.080631	0.052386	0.102715
11750746_FRMD3	0.084098	0.052393	0.102724
11735961_BHLHA15	-0.09241	0.052405	0.102744
11718613_ABCD4	-0.13576	0.052415	0.102759
11728092_RASIP1	-0.11342	0.052417	0.102759
11757366_UNC50	0.080541	0.052441	0.102801
11724193_COPS2	-0.11731	0.052443	0.102801
11762243_CDKN3	-0.17043	0.052449	0.102809
11725154_TDRD9	0.15933	0.052452	0.102812
11762485_RFX4	-0.27286	0.052456	0.102813
11733619_ACVR1C	-0.18311	0.052457	0.102813
11751597_ABI1	0.098244	0.052462	0.102818
11742074_ENSA	0.097796	0.05247	0.102829

11759867_CNBD2	-0.10677	0.05248	0.102845
11721147_SYNGR1	0.097003	0.05253	0.102939
11745646_EGR4	0.114455	0.052541	0.102956
11753160_PCDHGB6	-0.15393	0.052547	0.102965
11731483_CNRIP1	0.087638	0.052554	0.102974
11762044_SIGLEC16	-0.16126	0.052558	0.102977
11747181_LMAN1	-0.08713	0.052562	0.102981
11752649_ACO2	-0.11212	0.052569	0.10299
11751416_ERBB2	-0.13227	0.052571	0.10299
11726360_ALDH3A1	0.170455	0.052581	0.103003
11763833_PLIN1	-0.12794	0.052582	0.103003
11721068_FHOD3	0.110809	0.052601	0.103033
11737119_NEK10	0.173238	0.052601	0.103033
11753219_KLRF1	-0.12675	0.052608	0.103043
11736319_SNRNP48	0.096588	0.05261	0.103043
11753081_CCT5	-0.08411	0.052639	0.103094
11730225_POLDIP2	-0.08121	0.052647	0.103106
11721170_TRIM32	0.153738	0.052652	0.103112
11733998_THRB	0.165194	0.052656	0.103115
11763178_UBE2F	0.156108	0.05267	0.103139
11728598_ALLC	-0.12205	0.052672	0.103139
11752715_CCNI	0.078513	0.052679	0.103149
11721409_STK16	-0.10358	0.052701	0.103188
11760125_KLF6	-0.16504	0.052724	0.103228
11725103_EGFR	0.170915	0.052735	0.103242
11745457_CANX	-0.08966	0.052735	0.103242
11734753_TATDN3	0.145224	0.052751	0.103268
11745005_POLD4	0.102713	0.052763	0.103288
11756232_VDAC2	0.080104	0.052767	0.103288
11763744_TMEM67	0.221185	0.052767	0.103288
11728603_CLDN23	0.080107	0.052781	0.103306
11746516_ADAM15	-0.10436	0.052781	0.103306
11748182_SYNPO	-0.17282	0.0528	0.103339
11760160_USP36	-0.14697	0.052803	0.103341
11726429_YPEL1	-0.07867	0.052821	0.103372
11718604_MTURN	0.197427	0.052827	0.103381
11741924_GRB10	0.163475	0.052866	0.10345
11731824_GPR83	-0.12637	0.052867	0.10345
11715186_PRB1	-0.18228	0.052874	0.103459
11755780_FCRL5	-0.1863	0.052897	0.103501
11715787_ATP6V0C	-0.08555	0.052899	0.103501
11733481_CNGB1	-0.12476	0.052939	0.103573
11718319_CD93	-0.20792	0.05294	0.103573
11753021_TOM1	-0.10119	0.052949	0.103585
11735684_PPP1R3G	-0.09442	0.052951	0.103585
11738607_KCTD16	-0.1699	0.052971	0.10362
11718785_R3HCC1	-0.08411	0.052999	0.103668
11734250_SEPT14	-0.17095	0.052999	0.103668
11721317_PARM1	0.08096	0.053004	0.10367
11762036_LOC339059	-0.1525	0.053005	0.10367
11722228_DNAJC30	-0.13122	0.053007	0.103671

11720379_GALNS	0.085927	0.053014	0.103679
11763661_FMNL1	-0.09833	0.053018	0.103683
11741416_EIF4E	0.084581	0.053034	0.10371
11731272_KCNE1 ///	-0.12538	0.053041	0.103721
11742929_AP3D1	-0.09461	0.053078	0.103788
11724662_PAX8	-0.13672	0.053082	0.103791
11730078_SHF	-0.10823	0.053099	0.103821
11732352_SAC3D1	-0.09711	0.053102	0.103823
11730321_CLEC2D	-0.15871	0.053108	0.103828
11748696_SYT4	-0.07942	0.053109	0.103828
11745252_LINC00346	0.155471	0.053128	0.103861
11730314_ERMAP	-0.09748	0.05314	0.10388
11717243_YTHDF2	0.07724	0.053153	0.103902
11736255_INPP4A	-0.13055	0.053157	0.103904
11721249_GNG2	-0.10022	0.053163	0.103913
11743821_NTPCR	0.085752	0.053168	0.103918
11757676_C7orf76 /	-0.097	0.053203	0.10398
11728823_SLC25A3	0.077668	0.053204	0.10398
11734942_FAM212B /	0.148097	0.053209	0.103987
11730950_TMEM151B	-0.14157	0.053213	0.103987
11754428_ZFYVE26	-0.12579	0.053215	0.103987
11715966_ABHD17A	0.083044	0.053216	0.103987
11729938_C1orf111	-0.09725	0.053218	0.103988
11747683_ARHGAP27	-0.09253	0.053221	0.103989
11730548_ZNF789	-0.13652	0.053226	0.103993
11761108_PDLIM7	-0.09885	0.053229	0.103996
11753424_RAB11B	-0.12422	0.053241	0.104016
11747703_CARD8	0.135966	0.053252	0.104034
11740969_NPAP1	-0.09739	0.053283	0.10409
11760253_IFI44	0.372118	0.053292	0.104103
11725747_ATP2C1	-0.09365	0.053302	0.104118
11760437_DNPEP	-0.08766	0.053306	0.104122
11744065_ZNF589	0.084124	0.053335	0.104173
11734478_TMEM252	-0.14492	0.053342	0.104184
11715282_CGB /// C	-0.10151	0.053346	0.104187
11753198_SMARCA2	0.087197	0.053364	0.104217
11762455_COL8A1	0.23008	0.053388	0.104261
11732877_CYP24A1	0.221344	0.053394	0.104269
11715421_CCT2	0.079016	0.053401	0.104277
11752324_TBC1D30	-0.1149	0.053403	0.104277
11738408_SPACA3	-0.21092	0.053416	0.104298
11732436_RHOV	-0.12658	0.053429	0.104321
11738976_DEFB131 /	-0.14016	0.053433	0.104323
11721741_VANGL2	-0.19486	0.053452	0.104356
11729408_CNTNAP4	-0.15396	0.053459	0.104366
11725676_RORA	0.369777	0.053462	0.104366
11747362_ARHGAP1	-0.13666	0.053463	0.104366
11749805_MASP1	-0.14725	0.053475	0.104386
11726483_ZNF260	0.117711	0.053484	0.104398
11720032_TRIM29	-0.22267	0.053514	0.104454
11748269_PHF23	0.085613	0.053527	0.104474

11744470_MIF	0.081965	0.053537	0.10449
11752860_CD80	-0.1387	0.053551	0.104512
11740258_MAML2	0.134176	0.053562	0.104529
11731778_MARC1	-0.09956	0.053593	0.104587
11746823_RASSF4	-0.11884	0.053617	0.104629
11760722_HCRP1	-0.21226	0.053623	0.104637
11728012_PRTG	0.144446	0.05364	0.104662
11762408_RASGEF1B	-0.2574	0.05364	0.104662
11734756_METTL6	0.098404	0.053655	0.104685
11728323_PPP3R2	0.085753	0.053658	0.104688
11759281_SERINC5	-0.10619	0.053663	0.104693
11747630_XAF1	0.145928	0.053665	0.104693
11749673_CTNNA1	0.080887	0.053685	0.104727
11723352_TMEM178A	0.106366	0.053695	0.104743
11732445_RBPMS	0.127488	0.05371	0.104767
11718587_SAP30BP	-0.07815	0.053713	0.104767
11741275_TMEM144	-0.12276	0.053715	0.104767
11760265_PIH1D2	-0.10402	0.053716	0.104767
11753396_PDCD2	0.100279	0.053718	0.104767
11739075_NFE2L1	-0.1074	0.053736	0.104794
11722579_CHMP4A	0.098399	0.053737	0.104794
11741584_ASB10	0.156304	0.053738	0.104794
11718101_CD59	-0.08829	0.053746	0.104804
11745930_PAK6	-0.09283	0.053753	0.104814
11723542_ABHD14A	-0.12093	0.053758	0.10482
11758232_RNGTT	-0.15089	0.05376	0.10482
11748943_CHD4	-0.10498	0.053764	0.104823
11735958_WFDC12	-0.13886	0.053779	0.104848
11759644_OSMR	-0.17143	0.053784	0.104854
11720716_FGF13	0.11596	0.053793	0.104868
11720010_JARID2	0.109152	0.053815	0.104906
11748047_MFSD14B	-0.1193	0.053825	0.104922
11734859_WDR37	-0.11767	0.053849	0.104964
11740769_FSCN2	0.154407	0.053872	0.105005
11726902_PRDM15	0.132169	0.053882	0.10502
11732577_NANOG	-0.19747	0.053904	0.105057
11759013_CHEK1	-0.12909	0.053909	0.105057
11718928_ANKS1A	-0.08515	0.05391	0.105057
11760156_EVL	-0.15944	0.05391	0.105057
11726734_TLK2	-0.10164	0.053919	0.105063
11735236_SNX7	-0.0897	0.053919	0.105063
11730536_CHST13	-0.13921	0.053919	0.105063
11741704_HIST1H2BC	0.143355	0.053936	0.105087
11760863_NBR1	-0.22068	0.053938	0.105087
11760114_HERC2P2 /	-0.08819	0.053939	0.105087
11757633_FTH1	0.095786	0.05394	0.105087
11747959_KLHDC3	0.080099	0.053954	0.105109
11735443_ZNF781	0.212261	0.053959	0.105116
11728638_ADH1A	-0.11545	0.05397	0.105133
11740320_CFAP47	-0.09884	0.053981	0.10515
11719659_ATP1A2	-0.12351	0.053993	0.105165

11738756_OR10AG1	-0.16985	0.053994	0.105165
11741123_ADK	0.170377	0.053995	0.105165
11732202_TMEM39A	-0.084	0.054009	0.105188
11750646_PDE2A	-0.08602	0.054017	0.105199
11753898_HLA-DQA1	-0.10028	0.054023	0.105207
11730181_KDSR	0.095866	0.054031	0.105217
11749967_GABRB2	-0.09987	0.054032	0.105217
11748021_SUMF2	0.080545	0.054041	0.105227
11746990_PEX14	-0.11301	0.054042	0.105227
11759354_GPR32	-0.10237	0.054061	0.105261
11724561_TSR2	-0.16241	0.054065	0.105262
11733016_CCIN	-0.10089	0.054067	0.105262
11750496_MRPL43	-0.09018	0.054115	0.105353
11750089_PFDN5	0.081815	0.054124	0.105365
11762517_COBL	0.156201	0.054128	0.105369
11737190_ZGRF1	-0.08771	0.054132	0.105374
11752410_PBOV1	-0.1109	0.054159	0.105422
11739001_PRKCZ	0.09755	0.054178	0.105454
11717173_RNF31	-0.09095	0.054192	0.105477
11744686_SHARPIN	-0.10948	0.054198	0.105484
11733389_DEF6	-0.2177	0.054203	0.10549
11715592_DYNC1H1	0.084564	0.054215	0.105505
11729811_WFIKKN2	-0.1241	0.054215	0.105505
11752581_GGT6	-0.19046	0.054229	0.105527
11758245_SUB1	0.091891	0.054234	0.105533
11761711_FEZ1	-0.16322	0.05424	0.10554
11749625_PQLC3	0.142545	0.054242	0.10554
11728299_AP2A1	-0.10422	0.054287	0.105625
11734096_TMEM108	-0.13258	0.054296	0.105638
11756241_PSMF1	-0.08565	0.054299	0.105639
11736807_GABRA4	-0.14633	0.054317	0.105669
11730258_EPN3	-0.10394	0.054327	0.105685
11734356_DRP2	-0.11389	0.054339	0.105701
11760274_EIF3H	0.219858	0.05434	0.105701
11735304_HOXA11	-0.20599	0.054359	0.105734
11746072_TRPM2	-0.08057	0.054369	0.105749
11727597_PADI2	-0.11792	0.054377	0.105761
11759137_POMT1	-0.132	0.054393	0.105784
11745653_CASP10	0.143361	0.054393	0.105784
11735193_ADGRE2	0.12523	0.054402	0.105798
11758081_ENSA	0.096822	0.054406	0.105801
11755485_OPLAH	-0.13683	0.05442	0.105822
11747647_MSTO1	0.106957	0.054421	0.105822
11733004_FCGR3A //	0.201132	0.054437	0.105848
11735749_GEMIN8	0.113664	0.05444	0.105849
11749506_PLOD3	-0.09638	0.054442	0.105849
11720376_SAP30L	-0.12922	0.054452	0.105863
11755925_CTSD	-0.09779	0.054453	0.105863
11750485_ESR1	-0.10755	0.054476	0.105903
11763376_PCBP2	0.102635	0.054491	0.105928
11758532_IFT57	-0.18541	0.054495	0.105931

11742235_SUPT20HL2	-0.15799	0.054505	0.105947
11747396_DHX38	-0.09004	0.05451	0.105952
11737797_STRIP1	-0.11748	0.054534	0.105995
11739991_SRI	-0.08633	0.054543	0.106009
11736520_KCNA4	-0.16653	0.054556	0.106029
11751950_MUC4	-0.08914	0.054568	0.106047
11725718_CDC42	-0.11252	0.054569	0.106047
11718584_SAP30BP	0.118688	0.054573	0.10605
11736487_RASGEF1B	-0.14205	0.05459	0.106079
11761570_WDR74	-0.15321	0.054599	0.106091
11719433_HECA	-0.11882	0.054607	0.106104
11749847_MSR1	0.188401	0.054612	0.106104
11737499_SLC25A53	-0.11211	0.054612	0.106104
11719806_ASPN	0.164747	0.054634	0.106144
11727073_PLAC4	-0.10168	0.05464	0.106151
11749901_KIF6	0.277932	0.054653	0.106168
11735031_UPRT	-0.1371	0.054654	0.106168
11742870_ARPC2	0.087398	0.054657	0.106171
11742362_OR6V1	-0.1981	0.054679	0.106209
11757864_INSM1	-0.15242	0.054688	0.106221
11754423_CASKIN2	-0.10919	0.05469	0.106221
11734677_ITIH5	0.282936	0.054709	0.106255
11735985_TAS2R41	-0.12573	0.054712	0.106257
11730955_P2RX1	-0.18297	0.054724	0.106275
11735177_HAL	-0.08884	0.054735	0.106292
11727482_TPR	-0.07891	0.054747	0.106309
11742507_PRR21	-0.11713	0.054748	0.106309
11716799_FADD	-0.0784	0.054801	0.106409
11737201_CSF1	-0.1081	0.054813	0.106427
11759333_AMACR ///	-0.17166	0.054834	0.106463
11715255_TAS2R30	-0.15178	0.054842	0.106475
11737303_ACOX3	0.144876	0.054856	0.106498
11756879_STARD9	-0.17266	0.054866	0.106512
11764042_BLOC1S3	-0.0922	0.054879	0.106535
11723601_ATG2A	-0.10662	0.054902	0.10657
11752868_ATG2A	-0.10662	0.054902	0.10657
11726718_GEMIN7	-0.13106	0.054911	0.106583
11738905_MOGAT1	-0.10453	0.054918	0.106592
11718342_TUBGCP2	0.111692	0.05492	0.106592
11726311_GLRB	-0.26911	0.054925	0.106597
11761011_PRC1	-0.17688	0.054945	0.106632
11725850_USP48	0.089612	0.054959	0.106656
11724558_GNA15	-0.1749	0.054963	0.106659
11743974_MRPL38	-0.11065	0.054998	0.106722
11727141_EPB41L3	0.151552	0.055	0.106722
11757560_NPY	-0.13637	0.055011	0.106739
11742869_PBXIP1	-0.10033	0.05502	0.106752
11761625_RFC4	0.17627	0.05503	0.106767
11758294_ARMCX1	0.129062	0.05504	0.106784
11721385_CUL7	0.085539	0.055052	0.106801
11745665_SCN5A	-0.13211	0.055062	0.106817

11757388_C7orf76 /	-0.08893	0.055079	0.106846
11747325_LDB3	-0.21468	0.055092	0.106867
11741174_PKIA	0.11328	0.055099	0.106876
11757823_TNRC6B	0.08454	0.055101	0.106877
11741268_LDB2	0.193413	0.055132	0.106932
11737626_SETD4	0.087996	0.05515	0.106959
11764241_LOC105373	-0.20455	0.055151	0.106959
11747437_CARD8	0.143629	0.055155	0.10696
11762115_DDI2	-0.12401	0.055155	0.10696
11748945_ABI3BP	-0.09291	0.055168	0.106981
11734580_CSN3	-0.22405	0.055179	0.106997
11747097_RIBC1	-0.15041	0.055203	0.107039
11757594_PDAP1	-0.07516	0.055221	0.107071
11755178_MYT1	-0.1508	0.055231	0.107085
11732956_TARP ///	-0.10823	0.055245	0.107108
11721700_RERG	0.101372	0.055255	0.107123
11756125_NDUFA1	-0.08634	0.055257	0.107123
11750378_ATP2B2	-0.12374	0.055262	0.107128
11757836_SEC24A	0.094074	0.055265	0.10713
11747328_FHL1	0.11328	0.055273	0.107142
11739789_GCFC2	0.102733	0.055294	0.107178
11760813_ADAMTS7	-0.22482	0.055309	0.107203
11758779_TMEM50B	-0.12606	0.055311	0.107203
11753375_SLC25A35	-0.14578	0.055325	0.107226
11747811_POLR2M	0.103515	0.055348	0.107261
11737184_SAA2	0.241307	0.055348	0.107261
11758131_NDNL2	-0.09582	0.055359	0.107279
11740787_FBXO28	0.092687	0.055363	0.107282
11759167_HIST1H2BE	-0.19989	0.055375	0.1073
11754370_SYTL1	-0.12895	0.055382	0.10731
11737746_ADGRG1	0.087634	0.055386	0.107314
11727585_SPIN3	0.196615	0.055392	0.107322
11745776_TTC31	-0.07849	0.055402	0.107336
11734059_PSTK	0.141403	0.055433	0.107392
11733988_POLR2J2 /	0.116599	0.055449	0.107419
11761886_C21orf58	-0.21305	0.055457	0.10743
11737742_TTBK2	0.106906	0.055487	0.107484
11739210_COG6	-0.21095	0.05549	0.107484
11725315_PRELID1	0.083023	0.055501	0.107503
11716877_B4GALT5	-0.12089	0.055526	0.107546
11746445_ZNF626	0.153057	0.05554	0.107568
11733688_DPH6	-0.21689	0.055548	0.107578
11733050_HP1BP3	0.086621	0.055551	0.107578
11724985_EXPH5	-0.1209	0.055551	0.107578
11761854_LLPH	0.126767	0.055557	0.107584
11741256_APOBEC3F	0.141199	0.05557	0.107607
11715657_GLB1	-0.10152	0.055584	0.107629
11754949_HSPB7	-0.14982	0.05559	0.107637
11726240_PAX3	0.105941	0.055603	0.107658
11733720_MAX	-0.12336	0.055617	0.10768
11763527_MAP2K5	-0.25605	0.055624	0.10769

11744431_LIPF	-0.129	0.05563	0.107698
11715369_NDUFA4	0.080714	0.055638	0.107708
11729899_C4BPB	-0.16482	0.055704	0.107832
11737832_KIR2DL2 /	-0.19442	0.055722	0.107858
11720225_TBC1D1	0.089023	0.055722	0.107858
11752627_CLGN	-0.11135	0.055731	0.107872
11760536_HEXA	0.129923	0.055745	0.107894
11750682_TPCN1	-0.1121	0.055763	0.107925
11727843_SLC11A1	-0.10739	0.05577	0.107933
11715798_WDR45B	0.08063	0.055779	0.107947
11729771_KREMEN2	-0.08165	0.055795	0.107974
11724783_MRPS18C	-0.08017	0.055806	0.107991
11757126_SNORA74A	-0.18876	0.055815	0.108003
11752901_AZIN2	-0.16286	0.05582	0.108008
11717058_RPL5	0.079587	0.055831	0.108026
11750868_ZNF852	0.14191	0.055847	0.108052
11761005_NDUFS2	-0.09081	0.055853	0.10806
11760475_STK4	0.116136	0.055892	0.108132
11716570_NOTCH3	-0.08767	0.055899	0.10814
11761066_CLK4	0.19105	0.055941	0.108217
11722400_DAPK3	-0.10135	0.055959	0.108249
11730120_TMPRSS3	0.116423	0.055963	0.108251
11730994_S1PR4	0.143393	0.055968	0.108256
11722051_PRPH	-0.17831	0.055985	0.108285
11749219_CACNA2D4	0.099371	0.055993	0.108296
11716003_FAF2	0.117942	0.056024	0.108353
11722292_NYNRIN	-0.12123	0.056032	0.108359
11737491_WFDC10B	-0.13444	0.056032	0.108359
11750351_MADCAM1	-0.09606	0.056037	0.108364
11729764_LSM10	0.133808	0.056047	0.108379
11737684_ZNF517	-0.20574	0.05606	0.108401
11762332_NDUFA13	0.197885	0.056067	0.108409
11752203_ELMO2	0.121655	0.056071	0.108412
11734098_C14orf37	0.291862	0.056077	0.108419
11746651_SLC29A2	-0.10008	0.056081	0.108419
11737675_KCNK7	0.141916	0.056082	0.108419
11755995_OAZ1	-0.07537	0.056083	0.108419
11718338_NHP2	-0.07623	0.056086	0.10842
11744090_ZNF397	-0.08648	0.056099	0.108442
11735562_PRKACG	0.120289	0.05611	0.108458
11740911_LDHAL6A	0.182616	0.056122	0.108477
11738925_KRTAP5-1	-0.15303	0.056127	0.108482
11732451_AGRN	-0.11012	0.056131	0.108482
11738096_CATIP	-0.09934	0.056133	0.108482
11753212_TMEM131	0.197628	0.056134	0.108482
11716769_CHERP	-0.10473	0.056163	0.108535
11761859_AUP1	0.145948	0.056167	0.108539
11749175_TAF1B	-0.10905	0.056191	0.108579
11723585_SMPDL3A	0.162243	0.056192	0.108579
11733903_SPATA4	0.175332	0.056202	0.108593
11741224_EIF5	-0.09738	0.056224	0.108631

11722014_CWC15	-0.07832	0.056227	0.108633
11732961_ZP2	-0.0976	0.05623	0.108635
11737075_RBFOX2	0.105856	0.05626	0.108688
11739094_CXCR4	-0.11001	0.056267	0.108698
11743071_LPAR6	0.106761	0.056271	0.108701
11731628_OSGIN2	-0.11326	0.056283	0.108719
11760714_LOC101060	-0.10484	0.056289	0.108727
11739818_C9	-0.12647	0.056294	0.108733
11724484_CSK	0.099521	0.056303	0.108746
11750163_NLRP2	-0.11198	0.056315	0.108765
11760029_ABCA8	-0.1073	0.056324	0.108778
11735363_AKTIP	-0.21272	0.05634	0.1088
11732180_PTAFR	-0.2339	0.05634	0.1088
11723474_LYPLAL1	-0.15517	0.056343	0.108801
11716231_TMEM167B	0.091758	0.056351	0.108811
11753053_CLN3	-0.11273	0.056365	0.108836
11744026_EGLN2 ///	0.089059	0.056368	0.108838
11728837_MLH3	-0.10947	0.056397	0.108889
11729936_CLCNKA	-0.08822	0.056436	0.10896
11756804_FSTL4	-0.13253	0.056443	0.108969
11746523_FOXO3	0.092677	0.05645	0.108975
11753453_GTF2H2 //	0.073744	0.056451	0.108975
11719376_DAXX	0.082862	0.056464	0.108996
11754481_CORO7	-0.07388	0.056492	0.109047
11752000_UNC13D	-0.16485	0.05655	0.10915
11751769_GLYATL1 /	-0.14435	0.056551	0.10915
11741388_TSPAN14	0.123613	0.0566	0.109242
11723469_LPGAT1	0.084913	0.056619	0.109273
11717631_KLHL9	-0.09862	0.056622	0.109276
11749663_HFM1	0.154682	0.056631	0.109285
11748741_SCNN1A	-0.13298	0.056631	0.109285
11730393_GPR4	-0.15214	0.056634	0.109286
11731315_CPNE9	-0.15221	0.056691	0.109387
11751815_CDK5	-0.15642	0.056691	0.109387
11762728_CTCFL	-0.11428	0.056708	0.109415
11760768_DLL1	-0.14433	0.056713	0.109421
11744845_SLC22A2	-0.15805	0.056723	0.109435
11724478_FOSL1	-0.08444	0.056735	0.109454
11759256_HIST2H4A	-0.18654	0.056773	0.109523
11718891_FOXO3 ///	0.108231	0.056775	0.109523
200056_PMC1D	-0.09816	0.056796	0.109559
11738059_HIST1H2AD	0.288937	0.056806	0.109574
11742622_OR4S1	-0.20121	0.056811	0.10958
11734901_CYP4V2	0.101464	0.056832	0.109612
11733351_KLHL11	0.141972	0.056833	0.109612
11737784_CST4	-0.10317	0.05685	0.109641
11735036_SORCS1	0.201744	0.056852	0.109641
11731837_SLC35C2	0.079204	0.056874	0.109679
11716831_XPOT	-0.11417	0.056895	0.109715
11756156_TFRC	-0.0885	0.056899	0.109718
11741194_ACP2	-0.12722	0.056912	0.109739

11760341_ANKUB1	-0.28455	0.056926	0.109757
11757231_CHODL-AS1	-0.16498	0.056927	0.109757
11753577_AZIN2	-0.16102	0.05693	0.109757
11725234_ZBTB39	-0.15286	0.056932	0.109757
11728307_GULP1	0.086167	0.056932	0.109757
11716699_CMAS	0.08368	0.056936	0.10976
11745506_PIGP	-0.08878	0.056942	0.109767
11738342_MAGEA10-M	-0.10809	0.056946	0.10977
11759147_ATP13A5	-0.11646	0.056952	0.109777
11762761_LOC100288	-0.19481	0.056958	0.109779
11742497_SSX6	-0.08047	0.056959	0.109779
11731140_ZNF333	0.145246	0.056959	0.109779
11718004_NCOA5	-0.11135	0.056972	0.109799
11745811_IDH3B	-0.14238	0.057017	0.109881
11730834_KATNA1	0.131793	0.057019	0.109882
11738028_LAMA1	0.12377	0.057025	0.109888
11753087_XPNPEP2	-0.17936	0.057029	0.109892
11754099_SEC16A	-0.18813	0.057042	0.109913
11742589_OR4L1	-0.13607	0.057051	0.109926
11758929_TFDP2	0.11294	0.057064	0.109946
11726829_TYW1B	-0.14587	0.057078	0.109968
11724665_APLN	-0.11029	0.05709	0.109988
11736005_PRH2	0.143598	0.057134	0.110068
11718985_TMED5	0.328832	0.057138	0.110068
11760278_HCG8 ///	-0.09679	0.057139	0.110068
11753359_PIGA	0.119805	0.057148	0.110082
11750816_CD96	0.101506	0.057165	0.110111
11716845_STIM1	-0.08564	0.057185	0.110145
11734079_LONP2	0.096227	0.057204	0.110177
11724340_PRIMA1	-0.121	0.057217	0.110197
11761562_HEATR1	0.099071	0.057229	0.110216
11763266_FAM120B	0.092611	0.057239	0.11023
11744214_TRAFD1	0.112893	0.057243	0.110233
11761905_MCF2L2	-0.1613	0.057252	0.110247
11734973_L2HGDH	0.13196	0.057291	0.110313
11738268_FAM122C	-0.32012	0.057293	0.110313
11742038_CACNB2	-0.13584	0.057294	0.110313
11738088_C5orf56	0.1149	0.057295	0.110313
11746085_LOC728752	-0.12692	0.057315	0.110346
11761216_BEST1	-0.09429	0.057328	0.110367
11760810_BST2	-0.1496	0.057341	0.110388
11736456_KCNK6	-0.13365	0.057348	0.110398
11757918_MAP7	0.26013	0.057355	0.110407
11739381_GPR85	-0.18705	0.057358	0.110408
11716045_FAM168B	0.083327	0.057361	0.110409
11735620_SPO11	0.158337	0.057364	0.11041
11739292_EHF	-0.1419	0.05737	0.110418
11730205_PSMD9	0.091374	0.057394	0.110459
11764252_ATP10A	-0.20045	0.057404	0.110474
11738899_SERPINB12	-0.10729	0.05741	0.110481
11720954_RPL30	0.079124	0.057422	0.1105

11760824_MORN5	-0.15061	0.057426	0.110505
11737890_LAT2	0.130384	0.057439	0.110525
11729562_TBX4	-0.13156	0.057442	0.110527
11761219_CRACR2B	-0.1033	0.057446	0.110529
11721407_FLYWCH1	-0.09838	0.057448	0.11053
11741921_DGAT2L6	-0.1538	0.057452	0.110533
11730645_SP7	-0.09412	0.057472	0.110566
11744535_TCEB1	-0.09221	0.057507	0.11063
11751396_SRCIN1	-0.16309	0.057522	0.110652
11759122_TMEM150C	-0.09553	0.057523	0.110652
11743953_MSANTD1	-0.11681	0.057528	0.110657
11760072_PCDHA4	0.136537	0.057535	0.110666
11731615_CNR1	0.186989	0.057544	0.110679
11738589_OR5F1	-0.21814	0.05755	0.110687
11715824_NOP10	0.09269	0.057578	0.110735
11751925_VWDE	-0.12157	0.057581	0.110736
11727863_LIX1	0.147416	0.057598	0.110765
11726210_KLF15	0.108417	0.057611	0.110785
11745537_NFATC3	0.089743	0.057628	0.110815
11751618_HLA-E	0.079181	0.057632	0.110818
11743149_ZNF22	-0.08724	0.057651	0.110846
11733745_GCNT2	0.13928	0.057651	0.110846
11750045_TPM3	-0.08909	0.057692	0.110921
11739072_EPDR1	0.114562	0.057701	0.110933
11730019_MLIP	-0.07674	0.057714	0.110953
11761828_OSBPL1A	-0.15735	0.057718	0.110956
11757954_AGAP1	0.093384	0.057757	0.111027
11762578_MKL2	0.144662	0.057759	0.111028
11751454_MEST	-0.21524	0.057772	0.111048
11726475_TMEM52	-0.09994	0.057806	0.111108
11737767_BRDT	-0.16438	0.057834	0.111157
11749732_LONP2	0.079187	0.057851	0.111187
11741509_RPE	-0.09169	0.057861	0.111201
11760611_SETD6	-0.09478	0.057863	0.111201
11719770_EXOC7	-0.0913	0.057911	0.111288
11748630_TRMT2A	-0.09511	0.057918	0.111298
11718059_TTYH2	-0.15329	0.057951	0.111356
11723975_APOL3	0.105034	0.057974	0.111395
11734461_RFPL2	0.092091	0.057985	0.111414
11716982_CMSS1	-0.08148	0.058038	0.111505
11723976_ABCC8	-0.10759	0.058041	0.111505
11761222_GORASP1	-0.16914	0.058041	0.111505
11716862_TMEM184B	-0.15707	0.058042	0.111505
11723496_YBEY	0.147513	0.05807	0.111552
11737945_LCE1B	-0.12272	0.058071	0.111552
11736671_SPERT	-0.08231	0.058087	0.111578
11743825_PODXL2	0.139942	0.058093	0.111586
11743140_PPP1R12C	-0.1196	0.058104	0.111602
11715778_LUZP6 ///	-0.08255	0.058114	0.111616
11749918_ELMOD3	0.145073	0.058116	0.111616
11763265_PCYT1A	0.085233	0.058151	0.111679

11758054_DR1	-0.07793	0.058178	0.111726
11725622_JMJD4	0.091238	0.058182	0.11173
11752979_ABHD12	-0.09211	0.058184	0.11173
11745737_ACADVL	0.078669	0.058199	0.111754
11743994_KCTD5	0.095002	0.05821	0.111771
11739355_CHDH	-0.19505	0.058234	0.111811
11742562_OR10H4	-0.15812	0.058236	0.111811
11726076_PDCD2	0.08867	0.05824	0.111813
11727836_CPZ /// G	-0.20632	0.058241	0.111813
11752741_SLC29A3	-0.13325	0.058259	0.111842
11715358_CST3	0.106102	0.058264	0.111848
11744516_SLC2A10	-0.08324	0.058293	0.1119
11730488_DOK6	-0.17396	0.0583	0.111905
11734481_RTP1	0.211262	0.058301	0.111905
11735458_FBXO39	-0.12293	0.058317	0.111933
11763010_LOC100506	-0.19836	0.058327	0.111948
11746888_BPHL	0.175134	0.058352	0.111991
11750313_VEGFA	-0.07886	0.058355	0.111991
11744153_RTN1	-0.19202	0.058383	0.112037
11758716_NDUFA4	-0.15496	0.058388	0.112037
11747981_APCDD1	-0.13146	0.058389	0.112037
11735209_MDGA2	-0.12083	0.05839	0.112037
11754999_RBM25	0.083081	0.058392	0.112037
11744491_SEMA3B	0.083691	0.058392	0.112037
11717308_WDR75	-0.15295	0.058441	0.112125
11724282_UGT1A1 //	-0.08319	0.058458	0.112154
11750007_S100Z	0.188887	0.058502	0.112234
11739461_ECE1	0.117856	0.058535	0.112292
11750928_CDK20	0.093234	0.058537	0.112293
11721661_TBXAS1	-0.16384	0.058541	0.112296
11747693_MME	0.108058	0.058571	0.112346
11757211_H1FX-AS1	-0.12721	0.058572	0.112346
11737270_ASB4	-0.12766	0.058577	0.112352
11716407_MET	0.094195	0.058608	0.112407
11760543_RGS16	-0.09885	0.058629	0.112442
11715922_SEPT9	0.082551	0.058633	0.112446
11729300_GLYAT	0.148581	0.058639	0.112454
11719988_SNX19	0.081021	0.058642	0.112455
11753096_VPS37C	-0.12441	0.058663	0.112491
11755064_ZNF669	0.110028	0.058667	0.112494
11725542_GATA4	-0.16125	0.058714	0.112579
11729488_DCLRE1C	-0.10915	0.058719	0.112585
11734679_TXNL4A	-0.16797	0.058732	0.112606
11717853_CCL21	-0.14058	0.058747	0.112629
11719456_EXOC5	-0.11946	0.058749	0.112629
11736144_ENPP4	-0.20061	0.05877	0.112665
11731086_NXPH1	0.382014	0.058784	0.112686
11721300_ROMO1	0.078543	0.058797	0.112707
11761633_SLC37A3	0.28847	0.058835	0.112776
11743623_DPP9	0.080644	0.058856	0.112812
11731281_NFATC1	-0.13682	0.058876	0.112846

11758178_CORO1B	0.087848	0.058908	0.112903
11751445_TRABD	-0.12317	0.05895	0.112978
11761010_CKMT1A //	-0.16393	0.05898	0.113032
11748512_RNF220	-0.16168	0.059001	0.113067
11720464_F11R	-0.16071	0.059003	0.113067
11743325_CCDC50	-0.08864	0.059013	0.113082
11761353_CCM2	-0.14854	0.059025	0.1131
11745027_C11orf80	-0.10242	0.059028	0.113102
11724151_TNS4	-0.11912	0.059041	0.113121
11727026_NDUFB5	-0.07619	0.059056	0.113145
11757381_GTF2A2	0.10356	0.059065	0.113158
11750763_FKBP9 ///	-0.10216	0.059082	0.113188
11719399_DOCK6	-0.10314	0.059095	0.113207
11743569_EML1	0.188317	0.059098	0.113209
11728832_DSG3	-0.19534	0.059101	0.113209
11715663_STARD7	-0.10485	0.059107	0.113213
11754263_CHD9	-0.10006	0.059108	0.113213
11762330_NPSR1-AS1	-0.13738	0.059109	0.113213
11722210_PTGS1	0.161788	0.059116	0.113219
11757938_NMI	0.096889	0.059117	0.113219
11719605_RRN3	0.105238	0.059125	0.11323
11731801_C3orf70	0.269617	0.059137	0.113247
11761260_SART1	-0.10975	0.05915	0.113269
11720785_CLASRP	0.10675	0.059159	0.11328
11730102_SCN9A	0.234644	0.059172	0.113302
11735594_SLC24A4	-0.16617	0.059179	0.113311
11749242_SMOC2	-0.1066	0.059214	0.113374
11743942_CCDC152	0.299319	0.059258	0.113453
11722582_LOC100630	0.099159	0.059262	0.113456
11735544_C8orf44	-0.16191	0.059273	0.113472
11737799_R3HDM1	-0.08667	0.059279	0.113479
11756628_NPFFR2	0.211612	0.059287	0.113492
11723993_STYXL1	-0.10693	0.059295	0.113499
11725633_MRPS25	0.130624	0.059296	0.113499
11737411_SPATA45	-0.18286	0.059325	0.113546
11745969_SFXN5	-0.10239	0.059325	0.113546
11760474_TOR1AIP2	-0.09741	0.059333	0.113554
11732613_CELF3	-0.20553	0.059334	0.113554
11753649_SRSF3	-0.07393	0.059344	0.113568
11715382_PLD3	-0.09845	0.05935	0.113575
11752442_MIR17HG /	0.106006	0.059352	0.113575
11760560_RBM26	0.145529	0.059371	0.113603
11728545_SMYD1	0.157423	0.059371	0.113603
11741060_HVCN1	0.099458	0.059384	0.113623
11747601_SLC25A14	-0.11157	0.059395	0.11364
11727360_GJD3	-0.13156	0.059428	0.113698
11715226_PCDHA6	-0.122	0.059461	0.113756
11715964_SPTAN1	-0.11994	0.059468	0.113766
11755663_FOXO3 ///	0.097246	0.059475	0.113776
11726559_LCP1	0.1689	0.059483	0.113785
11759659_LOC100130	0.102676	0.059493	0.1138

11747885_TAF1C	-0.11284	0.059521	0.11385
11746084_PCMT1	-0.08614	0.059524	0.11385
11761292_RGL3	-0.1199	0.059556	0.113907
11753922_C7orf13	-0.10388	0.059584	0.113952
11743468_DKC1	-0.08386	0.059584	0.113952
11759105_SNRNP35	-0.16748	0.059591	0.113961
11758064_FZD8	0.104269	0.059593	0.113961
11733475_KCNJ6	-0.1192	0.059599	0.113964
11751636_GGT5	-0.08188	0.0596	0.113964
11735525_CLDN14	0.08937	0.059652	0.114061
11734326_RERE	-0.12913	0.059696	0.114138
11759655_SLC6A13	0.224139	0.059697	0.114138
11756692_SH3TC2	0.101221	0.059725	0.114186
11717506_TRAM2	-0.07828	0.059734	0.114195
11733152_COX18	-0.14212	0.059734	0.114195
11716410_PKDCC	-0.10131	0.05976	0.114239
11737114_ZNF483	0.168957	0.059769	0.114252
11750769_CCR2	-0.13151	0.059789	0.114286
11761593_ZNF384	-0.2531	0.059798	0.114294
11762804_APP	0.091433	0.059798	0.114294
11752446_KLHL32	-0.11117	0.059807	0.114303
11722334_FNDC5	-0.1003	0.059807	0.114303
11725976_WDR91	-0.09248	0.05981	0.114304
11742673_SPINK14	-0.09028	0.059827	0.114333
11724493_FAM71D //	-0.0783	0.059879	0.114426
11717329_KLF9	-0.15838	0.059882	0.114428
11736893_C3orf67	-0.17045	0.059908	0.114472
11759824_LILRA1	-0.14108	0.05991	0.114472
11736719_ZNF474	-0.14918	0.059912	0.114472
11735336_CPLX4	0.157672	0.05992	0.114484
11738873_KIF25	0.113784	0.059941	0.114518
11740786_SYNM	-0.14727	0.059992	0.114611
11734190_OOSP2	-0.14182	0.060006	0.114635
11728822_GDPD2	-0.14579	0.060012	0.114641
11720657_HLA-DRB5	-0.08969	0.060015	0.114642
11735334_HRH3	-0.0897	0.060037	0.114679
11737851_PPP1R18	0.084207	0.060051	0.114703
11759685_IGLV1-40	-0.17011	0.060058	0.11471
11743465_TNPO3	-0.10652	0.06006	0.11471
11719949_SPDL1	-0.07627	0.060067	0.11472
11762680_CADM1 ///	0.243651	0.060084	0.114747
11736714_LMOD2	0.131012	0.0601	0.114773
11729460_AK9	0.246089	0.060108	0.114783
11746870_SLC17A1	-0.1746	0.060125	0.114812
11733825_C1orf198	-0.07786	0.060138	0.114832
11757194_LMLN	-0.09829	0.060145	0.11484
11732653_ANKRD65	-0.08839	0.060159	0.114862
11755856_C2orf16	-0.10748	0.060173	0.114885
11755848_C17orf51	0.145369	0.060179	0.114893
11729868_UGT3A1	-0.10198	0.060204	0.114936
11754856_CA12	-0.19533	0.060228	0.114977

11730800_CRTC1	-0.09227	0.060234	0.114984
11725770_MIER3	0.123179	0.060239	0.114988
11754905_PLCZ1	0.266108	0.060254	0.115013
11762776_ACTR2	0.228544	0.060259	0.115018
11738632_GPAT2	-0.11986	0.060279	0.115052
11740722_IL1RN	-0.08008	0.060282	0.115053
11733228_DNMT3L	-0.09007	0.060287	0.115059
11732863_OXER1	-0.20216	0.0603	0.115079
11746817_AGPAT3	-0.12918	0.060305	0.115084
11727451_MORN4	0.157507	0.060309	0.115086
11720765_TOR1A	0.088151	0.060321	0.115106
11749362_MMGT1	0.093863	0.060367	0.115188
11736800_PDE10A	-0.20502	0.060375	0.115199
11741740_LRRC14B	-0.16201	0.060407	0.115256
11718411_NDUFAF3	-0.08852	0.06042	0.115276
11762758_BAIAP2	-0.07675	0.060424	0.11528
11721356_YPEL3	0.080188	0.060448	0.11532
11748900_RAB37	-0.10292	0.06045	0.11532
11745850_SCP2	0.098806	0.060453	0.11532
11726093_KANK3	-0.1032	0.060473	0.115355
11762031_UNC80	0.18198	0.060493	0.115388
11724584_ZKSCAN4	0.150662	0.060528	0.115451
11751885_PSME4	0.19558	0.060559	0.115505
11725296_PHF7	0.120511	0.060569	0.115519
11744881_GLYCTK	-0.13786	0.06059	0.115556
11721508_DVL3	-0.14722	0.060594	0.115558
11737805_TMPRSS11D	-0.1095	0.060596	0.115558
11748545_RNLS	-0.12378	0.060598	0.115558
11740029_CREM	0.137587	0.06061	0.115575
11759954_PCDHB17P	-0.18223	0.060615	0.11558
11742668_FABP9	0.075381	0.060625	0.115594
11752248_MET	0.09889	0.060638	0.115615
11723108_GPBP1	0.075639	0.060666	0.115664
11739529_PLGLB1 //	0.151595	0.060674	0.115672
11715667_TPM3	-0.07256	0.06068	0.115672
11740632_REP15	-0.11887	0.060681	0.115672
11718865_COX10	-0.08581	0.060682	0.115672
11730776_CEP68	-0.12442	0.060682	0.115672
11761494_CFAP65	-0.15118	0.060694	0.115691
11734662_TRA2B	-0.07849	0.0607	0.115697
11741440_PLPP5	0.166889	0.060715	0.115721
11748007_CMAHP	-0.11611	0.060728	0.115742
11742528_OR6K3	-0.11555	0.060745	0.11577
11735118_VAMP4	0.131491	0.060755	0.115784
11763051_RNF165	-0.09305	0.060786	0.115834
11721162_CTDSPL	0.136606	0.060787	0.115834
11743299_SRP72	-0.08042	0.060789	0.115834
11740142_BEND6	-0.21652	0.060792	0.115837
11757276_RPL24	-0.09941	0.060816	0.115878
11734247_KRT3	-0.21664	0.060864	0.115965
11720179_DDX28	-0.09371	0.060866	0.115965

11750596_BPESC1	0.157612	0.060871	0.115969
11718292_HK1	-0.08555	0.060879	0.115979
11724563_RHOF	0.094777	0.060946	0.116103
11748472_CTNNA2	0.127099	0.06096	0.116125
11734343_P2RY4	-0.18756	0.060963	0.116125
11735842_LCE4A	-0.17428	0.060966	0.116125
11751118_TUBB4A	-0.16489	0.060967	0.116125
11717113_ETHE1	0.078647	0.060995	0.116173
11746013_NCF1 ///	-0.14505	0.060999	0.116176
11754607_HSP90B1	-0.08964	0.06103	0.116231
11762193_ANGPT1	0.170246	0.061034	0.116235
11742152_OR1Q1	-0.09063	0.06104	0.116241
11762320_C9orf114	-0.09729	0.061044	0.116244
11755176_PAXIP1	0.087769	0.061066	0.116282
11742638_SEBOX	-0.16292	0.061072	0.116289
11762858_TIMM23 //	0.118051	0.061079	0.116298
11722975_HOXB5	-0.14862	0.061083	0.116301
11731597_ZNF503	-0.21696	0.061107	0.116342
11723837_SPNS2	-0.16367	0.061117	0.116357
11732930_SLC6A18	-0.11533	0.061142	0.116398
200059_PM RHOA	0.076517	0.061144	0.116399
11752060_ERN2	-0.16757	0.061161	0.116426
11740150_DDTL	-0.14187	0.061169	0.116436
11729649_PRF1	-0.14434	0.061179	0.116447
11729416_CCDC102A	-0.09592	0.061181	0.116447
11720422_AFF4	0.097089	0.061181	0.116447
11723742_CADM3	-0.14749	0.061223	0.11652
11737825_STX16	-0.08844	0.061244	0.116557
11739652_HNRNPLL	0.099138	0.061256	0.116575
11758056_TARSL2	0.153532	0.061271	0.116599
11753450_SPATA3	-0.13834	0.061297	0.116644
11750501_CLNK	-0.15155	0.061313	0.116669
11736186_DDX51	0.103361	0.061321	0.116681
11762430_DAB1	0.107552	0.061346	0.11672
11717969_FGFR3	-0.13454	0.061347	0.11672
11762264_C4BPB	-0.134	0.061378	0.116776
11735246_TRPV3	-0.17479	0.061395	0.116803
11718162_MCRS1	-0.09591	0.061402	0.116811
11726138_MTMR9	0.142646	0.061416	0.116833
11759932_CLK4	-0.11104	0.061435	0.116866
11761818_TMEM26	-0.18042	0.061443	0.116874
11743746_PHKA1	0.08573	0.061444	0.116874
11742512_KRTAP4-8	0.117766	0.061455	0.11689
11736700_RBM43	0.166393	0.061465	0.116904
11737888_HRH4	-0.12731	0.061487	0.116942
11723902_PLEKHG1	0.199783	0.061531	0.11702
11738541_FAM129C	-0.11249	0.061559	0.117069
11719726_POLD4	0.083577	0.061565	0.117077
11759612_FNDC3B	0.085764	0.061576	0.117093
11732024_MAMDC4	0.095403	0.061591	0.117114
11748395_MYADM	0.076676	0.061592	0.117114

11761889_RPL14	-0.12701	0.061596	0.117118
11728721_PDE1B	-0.10868	0.061609	0.117138
11738471_OR56B1	-0.1201	0.061633	0.117177
11718739_ST3GAL1	-0.13425	0.061635	0.117177
11734490_VSX1	-0.15622	0.061646	0.117193
11738444_OR51G2	-0.20567	0.061657	0.117211
11758760_GSTK1	0.137423	0.061683	0.117255
11732098_BRMS1	-0.0824	0.061714	0.117309
11717490_TDRD3	0.10534	0.06174	0.117355
11716828_NPC2	0.077993	0.061758	0.117385
11755630_OGFOD2	-0.13134	0.061763	0.11739
11735163_OSBPL5	-0.12038	0.061791	0.117438
11718945_PGPEP1	0.113908	0.061826	0.117498
11715655_VDAC1	-0.09292	0.061829	0.117498
11730637_CTLA4	-0.1402	0.06183	0.117498
11748325_MRPS22	-0.0904	0.061865	0.11756
11731212_GGT6	-0.17154	0.061873	0.117572
11761932_TUBB4B	-0.10907	0.061891	0.117601
11754421_VSIG2	-0.13451	0.061915	0.117641
11728097_CROCC	-0.17571	0.061934	0.117668
11763587_ZC3H4	-0.09613	0.061934	0.117668
11745659_POM121 //	-0.07502	0.061952	0.117697
11729551_MKLN1	-0.08471	0.06196	0.117709
11738329_LKAAEAR1	-0.09118	0.061982	0.117747
11744043_BCAM	-0.09652	0.062004	0.117783
11717454_SPOCK2	-0.09275	0.062027	0.117815
11758713_RP1L1	-0.09614	0.062029	0.117815
11752502_ALDH7A1	0.103077	0.062029	0.117815
11759295_EHF	0.20082	0.06203	0.117815
11763922_SNORA71C	-0.18878	0.062035	0.117819
11760178_RTFDC1	-0.14895	0.062051	0.117844
11748554_THAP8	-0.11061	0.062055	0.117849
11740051_DUSP27	0.258827	0.062083	0.117896
11753311_CARNMT1	-0.18431	0.062113	0.117949
11754891_CCM2L	-0.13973	0.062116	0.117949
11744597_KIAA0100	-0.08766	0.062118	0.117949
11761194_TGOLN2	0.182351	0.062173	0.118049
11715768_SIRT2	0.088274	0.062176	0.118049
11739788_C11orf57	-0.09898	0.062178	0.11805
11717666_TSPYL4	0.105409	0.062184	0.118057
11722663_GABRB3	0.293801	0.062204	0.11809
11736137_CTNNA2	-0.12739	0.062218	0.118112
11743232_MLLT4	0.150299	0.062224	0.118117
11761825_CDK13	-0.2074	0.062226	0.118117
11729389_MIER1	-0.09038	0.062255	0.118167
11730140_LGALS13	-0.20601	0.062257	0.118167
11729691_KLHL24	0.187451	0.062259	0.118167
11742606_OR1N1	-0.10193	0.062263	0.11817
11728338_CENPBD1	-0.09264	0.062271	0.118179
11736003_GDI2	0.082799	0.062279	0.11819
11728818_USP28	-0.16427	0.062284	0.118196

11762611_YIF1B	-0.14925	0.062292	0.118202
11751490_MAPKAP1	-0.07885	0.062292	0.118202
11729419_ATP2A3	-0.27115	0.0623	0.118211
11715880_PITPNA	0.092105	0.062302	0.118211
11736652_CFAP57	0.140129	0.06231	0.118222
11725702_AQP9	-0.14809	0.062313	0.118223
11742920_HSPA9	0.079288	0.062323	0.118234
11734249_SEPT14	-0.19579	0.062326	0.118234
11762855_CYP4F24P	-0.07849	0.062329	0.118234
11762856_CYP4F24P	-0.07849	0.062329	0.118234
11743838_ATR	0.098541	0.062336	0.118243
11730512_CHST8	-0.1235	0.062396	0.118352
11760597_ERCC8	0.096151	0.062398	0.118352
11739429_ZDHHC24	-0.09023	0.062401	0.118352
11745504_MPP1	-0.18124	0.062406	0.118357
11760891_GPAT2	-0.14413	0.062437	0.118411
11732218_BID	0.081822	0.062439	0.118411
11727851_TEX264	0.086052	0.062444	0.118414
11731317_MMP28	-0.125	0.062445	0.118414
11725426_RAB27A	-0.07538	0.06245	0.118418
11750635_PTPRH	0.110383	0.062454	0.118422
11741090_ESRRG	0.149586	0.062461	0.118431
11762652_ADRA1A	-0.0947	0.062466	0.118435
11730083_TCTE1	-0.21721	0.062479	0.118454
11742469_MAGEB16	0.136214	0.062482	0.118456
11744544_KDELR1	-0.08821	0.062513	0.11851
11726348_EMSY	-0.10019	0.062526	0.11853
11753100_PER2	0.090048	0.062549	0.118569
11724404_WTAP	0.075889	0.062605	0.118672
11729532_REM2	-0.12539	0.062623	0.118701
11747668_NECAP1	0.106047	0.062631	0.118712
11721032_FAM107A	-0.12241	0.062634	0.118712
11746951_REXO2	-0.08892	0.062638	0.118715
11724654_ZBTB47	-0.18087	0.062695	0.118819
11734174_MEIOB	-0.16935	0.062707	0.118836
11744014_WIZ	-0.12344	0.062724	0.118864
11719734_MFSD12	0.078733	0.062761	0.11893
11733658_GDPD4	-0.17217	0.062776	0.118953
11742222_ZNF99	-0.16097	0.062783	0.118962
11751434_ACER3	0.089352	0.062794	0.118979
11751192_SPOCK3	-0.10194	0.062807	0.118998
11743198_SLC6A16	0.126634	0.062811	0.119001
11733650_FAM228B	-0.15366	0.062826	0.119026
11715268_GP1BB ///	-0.08969	0.062834	0.119035
11734727_IL16	-0.19557	0.062836	0.119035
11745162_BCL2L11	0.169422	0.062844	0.119046
11740063_CAMK2D	0.092665	0.062879	0.119108
11717811_KIF3C	-0.08519	0.062895	0.119132
11726347_LAT2	0.153614	0.062907	0.119151
11761654_KDM5D	-0.10272	0.062936	0.119201
11721832_CHURC1	0.07294	0.062939	0.119202

11742125_TPTE2	0.138104	0.062967	0.119251
11761085_ADAM33	-0.19522	0.062971	0.119254
11758715_DEFB126	-0.1213	0.062985	0.119276
11725040_FGF2	-0.1419	0.062988	0.119277
11737534_CIDEB	-0.13181	0.063003	0.1193
11744830_LOC105369	0.083069	0.063018	0.119322
11738391_DEFB121	-0.14868	0.063019	0.119322
11723410_PLP1	0.090522	0.063037	0.119347
11758350_PARG ///	0.131321	0.063037	0.119347
11720947_CA12	-0.19182	0.063044	0.119355
11750888_PMCHL1 //	-0.17537	0.063068	0.119397
11731378_MED25	-0.10099	0.063099	0.11945
11736439_INF2	-0.13816	0.063102	0.119451
11741866_OR1J2	-0.18582	0.063148	0.119534
11763234_CCDC91 //	0.123029	0.063169	0.119565
11761092_PSMB8	-0.15759	0.06317	0.119565
11741749_LRRC2	0.089372	0.063179	0.119577
11762443_HIPK2	-0.08856	0.063185	0.119585
11718580_COQ9	0.080752	0.063189	0.119588
11720121_GIGYF1	-0.12915	0.063197	0.119599
11759615_PRPF38B	0.096576	0.063211	0.11962
11760418_RCAN1	-0.07503	0.063216	0.119623
11736311_FCGR1A	-0.14597	0.063217	0.119623
11732592_SMC1B	-0.16348	0.063224	0.119624
11751317_EI24	0.089653	0.063224	0.119624
11730805_BCAS3	0.092865	0.063225	0.119624
11740493_AGAP1	0.146911	0.063234	0.119636
11738107_SHANK1	0.119491	0.063236	0.119636
11729742_IFI27L2	-0.0936	0.063263	0.119681
11756966_ERICH6	-0.09764	0.063271	0.119689
11744051_TSPAN1	-0.10294	0.063273	0.119689
11748964_ETV1	0.196877	0.063274	0.119689
11755297_ARHGEF40	-0.07668	0.063295	0.119724
11720510_APOBEC3G	-0.10895	0.063337	0.119799
11716818_VSIG4	-0.21404	0.06335	0.119816
11719005_ANKH	0.090159	0.063353	0.119816
11734189_TSNARE1	0.096865	0.063354	0.119816
11726948_TLR8	-0.10032	0.063367	0.119837
11749953_POU2F1	0.101518	0.063386	0.119868
11731408_CLN3	-0.08497	0.063404	0.119897
11763676_ACSL5	-0.20506	0.063408	0.119898
11726068_PDDC1	0.283864	0.063409	0.119898
11753926_ARMC6	-0.07698	0.063428	0.119929
11755314_VARS2	-0.10401	0.063432	0.119932
11719306_WRAP53	-0.08768	0.063451	0.119961
11734514_TEPP	-0.21015	0.063454	0.119961
11732980_IFNA21	-0.20427	0.063455	0.119961
11728720_RHOH	-0.10841	0.063475	0.119995
11743826_FSTL5	0.150141	0.063481	0.12
11723643_ANXA7	0.082123	0.063483	0.12
11719823_ZAK	-0.12202	0.063495	0.120018

11755706_BBX	0.085438	0.063505	0.120032
11736476_TCHP	-0.07935	0.063523	0.120061
11743839_ST3GAL6	0.202383	0.06355	0.120108
11752630_RBM39	0.081842	0.063557	0.120117
11761687_ENTPD6	-0.10578	0.063567	0.12013
11716959_POMGNT1	-0.09162	0.06357	0.120133
11736164_CNDP1	-0.14605	0.06358	0.120147
11724731_YY1	-0.0853	0.063591	0.120163
11755742_ADH7	-0.14016	0.063626	0.120225
11757998_GOLGA7	0.075631	0.063641	0.120247
11730001_NRTN	-0.15312	0.063658	0.120275
11761511_TOR1A	-0.2198	0.063701	0.120351
11741928_SH2D7	-0.18041	0.063704	0.120351
11743985_AKAP1	-0.08645	0.063705	0.120351
11750064_PLRG1	0.073555	0.063721	0.120376
11741694_SERHL ///	-0.10475	0.063734	0.120395
11742334_OR52W1	-0.19016	0.063751	0.120424
11750821_MIPOL1	0.104633	0.06376	0.120433
11742002_OR2H2	-0.11963	0.063761	0.120433
11731313_PAXBP1	-0.09076	0.063769	0.120444
11718975_ARPP19	-0.07927	0.063807	0.120508
11730115_ 10-Mar	-0.14605	0.063808	0.120508
11753940_NRL	-0.14885	0.063814	0.120514
11722310_TMC4	-0.14299	0.063824	0.120525
11751683_RAB11A	-0.07863	0.063825	0.120525
11745898_PIAS3	-0.13776	0.063837	0.120543
11752051_MYH9	0.106066	0.063843	0.12055
11763897_SNORA77	-0.10643	0.063874	0.120604
11730170_GPX3	-0.11742	0.063907	0.120661
11730028_MT1B	-0.13181	0.063912	0.120666
11721452_ACTR6	-0.08513	0.063914	0.120666
11724111_ARHGAP4	-0.08221	0.063922	0.120675
11737571_SCX	-0.15988	0.063926	0.120679
11741863_OR5V1	-0.13344	0.063938	0.120696
11726944_ZKSCAN2	0.300955	0.063948	0.120712
11737759_RABGGTA	-0.07639	0.063951	0.120712
11722082_RUFY3	0.129416	0.06399	0.120777
11763510_LINC01013	-0.20948	0.063991	0.120777
11750285_NFIX	-0.09388	0.064019	0.120827
11723857_TXNDC16	-0.27042	0.064026	0.120835
11762411_OK/SW-CL.	-0.13019	0.064046	0.120869
11758009_AMPD3	0.162559	0.064052	0.120875
11757988_PRUNE2	-0.17052	0.064106	0.120971
11761754_MST1P2	-0.10518	0.064108	0.120971
11758930_HEATR1	-0.07986	0.064124	0.120996
11718055_BTBD3	0.093328	0.064127	0.120997
11763723_SRRT	0.080428	0.064132	0.121001
11725713_DIRAS3	-0.15423	0.064144	0.12102
11720015_CGNL1	-0.12731	0.064151	0.121029
11752404_SIGLEC10	-0.22558	0.064166	0.121053
11755436_ARID1A	-0.09943	0.064186	0.121086

11761077_SPOCK2	-0.12081	0.064197	0.121098
11729089_C1orf87	-0.14615	0.064202	0.121098
11761255_KCNG1	-0.15145	0.064202	0.121098
11729959_ASIC1	-0.17119	0.064203	0.121098
11721559_ZNF638	-0.14774	0.064212	0.121105
11722717_GABPA	0.166923	0.064213	0.121105
11754161_PIGP	-0.08667	0.064213	0.121105
11730371_EIF4E3	-0.19905	0.064217	0.121106
11739509_NAA30	0.177051	0.064234	0.121134
11729057_ERICH4	-0.13111	0.064249	0.121157
11727296_TGFB3	0.138002	0.064252	0.121159
11720301_SLA	-0.12177	0.064294	0.121233
11748789_CCNJL	0.081135	0.064303	0.121245
11760997_ARHGAP27	-0.24686	0.064313	0.121259
11741148_PRDX2	0.076559	0.064316	0.12126
11716898_ARL3	-0.26061	0.064361	0.121341
11725655_GTF2H5	-0.0831	0.064378	0.121367
11735940_DEFB103A	-0.10198	0.064381	0.12137
11761296_TSPAN16	0.143345	0.064397	0.121395
11723617_C3orf79	-0.18493	0.064405	0.121405
11721697_UBE3B	0.086413	0.064415	0.121416
11750309_ERI2	-0.10991	0.064416	0.121416
11739499_GRIA1	-0.08554	0.064422	0.121423
11742063_BTN3A1	0.149779	0.064428	0.12143
11728640_EDARADD /	-0.09168	0.064433	0.121435
11751355_CEACAM7	-0.16537	0.06444	0.121442
11743600_OTUD5	0.082654	0.064446	0.121447
11759215_CCR4	0.113872	0.064447	0.121447
11744825_CNBP	0.089766	0.064453	0.121452
11737728_XKR6	0.14226	0.064533	0.121597
11728145_SHC2	-0.10203	0.064534	0.121597
11735515_PATE4	-0.24724	0.064538	0.1216
11760381_STK19	-0.13095	0.064555	0.121625
11729021_CNTN4	0.129275	0.064557	0.121625
11744955_ANXA1	0.082659	0.064568	0.121641
11738524_MICAL3	0.1433	0.06457	0.121641
11721254_ARMCX3	-0.13684	0.064574	0.121643
11746724_BTN2A3P	0.114689	0.064597	0.121683
11734529_PILRA	-0.12926	0.064611	0.121704
11761790_TRDC	-0.17911	0.064629	0.121733
11760400_SUCLG1	-0.12352	0.064635	0.12174
11755599_POU5F1	0.107975	0.064644	0.121753
11749470_TRPM8	0.162632	0.064659	0.121775
11721397_DOT1L	0.106642	0.064664	0.12178
11763291_CFAP65	-0.09499	0.064667	0.121782
11720745_BCL6	-0.13383	0.064681	0.121803
11745810_KIAA0040	0.178117	0.064694	0.121823
11717542_NFKB1	-0.07817	0.064721	0.121868
11721161_CTDSPL	0.090024	0.064738	0.121894
11741715_POTEG ///	0.085119	0.064739	0.121894
11759585_RAB18	-0.11502	0.06475	0.12191

11748615_IFNAR1	-0.10168	0.064756	0.121916
11749054_PXN	0.116437	0.064764	0.121926
11729244_DDX18	0.076354	0.064776	0.121944
11731377_RABL2A //	0.126175	0.064785	0.121957
11735452_NHLRC1	-0.09351	0.064792	0.121965
11761471_ZNF169	-0.12155	0.064794	0.121965
11762255_LIAS	-0.20602	0.064829	0.122026
11751201_GYS1	-0.08845	0.064834	0.122031
11716909_NR2F6	0.078526	0.064902	0.122148
11730782_SLC2A6	0.096044	0.064903	0.122148
11740222_FAM196B	-0.07415	0.064906	0.122148
11752866_FAM47E-ST	-0.21502	0.064906	0.122148
11756691_GBX2	-0.15481	0.064936	0.122198
11730241_SLC2A12	-0.13679	0.064939	0.1222
11734273_GPR18	0.192403	0.064946	0.122207
11745558_BNIPL	-0.12269	0.064951	0.122213
11761197_SLC43A2	0.082477	0.064966	0.122236
11732190_NOTCH2	-0.14248	0.064987	0.12227
11763836_HACD1	-0.16447	0.065004	0.122299
11757592_SRP14	0.072787	0.065027	0.122337
11745722_FHL1	0.105842	0.065043	0.122362
11740967_LOC391322	-0.12361	0.065077	0.122419
11752591_CIT	-0.18022	0.065078	0.122419
11743605_VBP1	-0.08895	0.065096	0.122447
11753070_EPHB4	-0.09853	0.065098	0.122447
11763551_IGLJ3 ///	-0.1103	0.065147	0.122535
11723101_DDX10	0.128374	0.065155	0.122544
11735825_SLC9A3	-0.11722	0.065157	0.122544
11755079_ZC3H12A	0.094925	0.065178	0.122579
11752079_ADAMTS4	-0.13574	0.065203	0.12262
11759134_CHRNA3	0.118515	0.065241	0.122687
11716050_GBF1	0.144685	0.065245	0.12269
11731226_TDRD6	-0.17737	0.065264	0.12272
11755858_MS4A1	-0.0961	0.065266	0.12272
11715133_PCDHGA1	-0.16245	0.065304	0.122787
11759162_KRTAP19-1	-0.10802	0.065314	0.122801
11743644_MXRA7	0.148093	0.065344	0.122844
11737004_PDE3A	-0.12988	0.065347	0.122844
11764027_MRPS11	-0.24627	0.065347	0.122844
11729774_PCDHB7	0.093482	0.065348	0.122844
11734849_CELSR2	-0.12367	0.065349	0.122844
11746572_TTC39B	0.131386	0.065393	0.122922
11729405_DOC2A	-0.12594	0.065405	0.122939
11733361_PKHD1L1	0.139426	0.065412	0.122947
11728914_TRIOBP	0.10952	0.06544	0.122995
11744231_MAPK7	-0.07508	0.065449	0.123008
200039_PMPSMB2	-0.08424	0.065455	0.123014
11723044_SMAD1	0.12224	0.065462	0.123023
11758863_ATP6V1C1	-0.21808	0.065477	0.123045
11758100_C1S	0.164002	0.065485	0.123057
11729611_ARPC4	-0.09716	0.065504	0.123084

11722023_ABCF2	0.079315	0.065505	0.123084
11763137_HSP90AA6P	-0.25693	0.065513	0.123095
11761952_PARPBP	-0.24848	0.065519	0.123102
11738331_B3GALT5	-0.11371	0.065526	0.123109
11741774_PPP1R17	-0.0991	0.065551	0.123152
11748196_ACADSB	0.156383	0.065574	0.123191
11715159_SFT2D1	0.089994	0.065584	0.123205
11723017_HM13	0.114965	0.0656	0.123225
11747221_FBXO10	-0.09875	0.0656	0.123225
11727420_CNBP	-0.08793	0.065628	0.123274
11726196_SYN2	-0.13892	0.065659	0.123326
11750209_HEY1	-0.13808	0.065661	0.123326
11735892_RESP18	-0.19696	0.065676	0.123345
11755016_LOC101928	0.099894	0.065676	0.123345
11753828_GNAS	-0.07385	0.065687	0.12336
11757423_SEC11A	-0.07222	0.065693	0.123367
11732741_PAK3	-0.13744	0.065697	0.12337
11732116_KRTAP19-5	-0.08206	0.0657	0.123371
11730003_CACNB1	-0.18728	0.065713	0.12339
11763356_LOC157503	-0.18646	0.065723	0.123404
11737804_TMPRSS11D	-0.13546	0.065738	0.123428
11738967_DEFB112	-0.14987	0.065755	0.123455
11734328_FAM227B	0.188039	0.065765	0.123469
11761985_ITSN1	-0.09267	0.065778	0.123489
11763019_PNRC1	0.110311	0.06579	0.123506
11745106_MEIS3	0.092973	0.065802	0.123523
11715464_IGFBP4	-0.11932	0.065808	0.12353
11725866_EBF3	-0.15932	0.065817	0.123542
11738595_PF4V1	-0.12383	0.065828	0.123559
11723064_TMEM18	-0.09406	0.065857	0.123608
11763969_SFTPC	-0.12613	0.06586	0.123608
11746066_CLINT1	0.074771	0.065866	0.123615
11761680_MLH3	-0.12351	0.065872	0.123622
11748234_URGCP	0.130104	0.065879	0.12363
11745861_TCF12	0.10578	0.065883	0.123634
11715837_HEXB	0.091091	0.065961	0.12377
11749909_MLC1	-0.15286	0.065961	0.12377
11747788_AURKC	-0.10763	0.065999	0.123837
11722668_SCAF1	-0.09771	0.066018	0.123867
11721885_CDC42	-0.14655	0.066055	0.123932
11761865_ZNF396	-0.13668	0.066071	0.123957
11749181_MBD1	0.124944	0.066085	0.123978
11736814_GIPC2	-0.10821	0.066181	0.124154
11763232_SUMO1	-0.08482	0.06619	0.124165
11722924_MMRN1	-0.19725	0.066196	0.124172
11738776_SH2D6	-0.14136	0.066218	0.124209
11755727_AXDND1	-0.22467	0.066236	0.124237
11756642_DECR2	0.086154	0.066238	0.124237
11726236_ACY3	-0.08364	0.066271	0.12429
11754554_ECHDC2	0.121696	0.066271	0.12429
11730358_CCDC54	-0.21014	0.066274	0.12429

11731245_GDF9	-0.25318	0.066279	0.124294
11738994_CYP21A1P	-0.10411	0.066308	0.124345
11733438_C16orf92	-0.15129	0.066318	0.124358
11747130_PRSS8	-0.11695	0.066331	0.124379
11731505_C22orf39	-0.087	0.066334	0.124379
11739634_MED13	-0.08881	0.066337	0.12438
11716049_TRIB1	0.118586	0.066352	0.124403
11746883_ABCC12	0.24348	0.066356	0.124405
11761834_EED	0.201293	0.066359	0.124406
11752023_MGAT4A	-0.16133	0.066361	0.124406
11726250_AGR2	-0.13536	0.066368	0.124414
11746205_DMWD	0.099114	0.066378	0.124429
11729330_EPB41L4B	-0.12961	0.066388	0.124443
11754033_PLA2G2A	-0.08317	0.066391	0.124443
11732337_BCAS1	-0.13768	0.06643	0.124509
11749705_NCOA5	-0.08115	0.066431	0.124509
11739390_OXTR	0.158012	0.066444	0.124528
11759180_GOLIM4	-0.12255	0.066453	0.124539
11716160_LBH	-0.19026	0.066455	0.124539
11751404_ABI2	0.126985	0.066463	0.12455
11746896_ALDH2	-0.0867	0.06647	0.124557
11733141_PFKFB3	-0.16808	0.06649	0.124591
11736585_KCTD4	-0.14963	0.066518	0.124638
11751807_TTTY11	-0.12065	0.066532	0.12466
11732764_CCDC120	-0.07655	0.066545	0.124678
11761195_TGOLN2	0.136431	0.06655	0.124684
11724739_TMEM204	-0.09245	0.066598	0.124767
11749248_WIPF2	-0.15734	0.066599	0.124767
11738017_LOC102030	-0.08839	0.066609	0.12478
11749213_TSGA10	-0.20236	0.066614	0.124785
11750516_ATPAF1	0.083386	0.06663	0.124809
11752960_COL9A1	0.110713	0.066633	0.124811
11746465_EPS15L1	-0.08766	0.066651	0.124839
11747910_PARP10	0.097764	0.066664	0.124858
11750933_ZNF480	0.106475	0.066704	0.124929
11716902_TGOLN2	0.088309	0.066707	0.124929
11747614_ALOXE3	-0.1339	0.066715	0.124941
11736685_FGF5	0.110361	0.066743	0.124988
11750003_BRCC3	0.126833	0.06675	0.124996
11744244_MASP1	-0.08855	0.066785	0.125058
11735926_C10orf120	-0.16149	0.066798	0.125076
11746153_VWA3B	-0.10041	0.066802	0.125079
11754494_DNHD1	0.100256	0.066839	0.125144
11755590_PRMT8	-0.18945	0.066846	0.125152
11727477_SAP30	-0.10418	0.066851	0.125157
11752243_CMIP	-0.1174	0.066868	0.125183
11754211_PPIG	-0.09427	0.066877	0.125196
11719390_SIRT6	0.104974	0.066883	0.125203
11722651_RUNDC1	0.129485	0.06692	0.125267
11759524_UCN	0.124978	0.066928	0.125276
11755532_RABGGTA	-0.09447	0.066936	0.125286

11756137_BTF3	0.072755	0.066938	0.125286
11729565_DND1	-0.106	0.066951	0.125306
11716805_PLEKHA2	-0.17056	0.066962	0.125321
11743467_DKC1	-0.08655	0.066967	0.125325
11720416_CAMSAP1	0.080163	0.066973	0.125332
11732015_TTC23L	0.1032	0.066976	0.125332
11722762_GATA2	-0.25576	0.066989	0.125352
11724871_CLDN2	-0.09884	0.067025	0.125412
11724128_FAM65C	0.136292	0.067026	0.125412
11731102_PFN4	-0.19243	0.067054	0.12546
11738815_EGFR	-0.1029	0.067065	0.125476
11728645_NUCKS1	-0.11519	0.06707	0.125481
11727549_NOL4	-0.14422	0.067077	0.125488
11750547_GALNT14	0.116515	0.067092	0.125512
11753608_GPER1	-0.11306	0.067096	0.125514
11715112_ERICH3	0.096506	0.0671	0.125517
11747519_TG	-0.15772	0.067125	0.125559
11756494_SETD6	0.077868	0.06713	0.125563
11741513_LDHAL6A	-0.10329	0.067176	0.125644
11750162_WDR45B	-0.10229	0.067205	0.125691
11735199_GJA9 ///	-0.15559	0.067206	0.125691
11731411_SLC8A2	-0.13093	0.067221	0.125714
11726086_C12orf65	-0.10322	0.067237	0.12574
11735709_KRT2	-0.13538	0.067296	0.125845
11720536_MMAB	-0.13403	0.067301	0.125849
11721892_ANAPC1	0.100143	0.067304	0.125849
11746786_STXBP1	-0.07405	0.067312	0.12586
11753916_LINC00526	-0.129	0.06733	0.125889
11741600_C1QL3	-0.12974	0.067336	0.125892
11761609_ZNF586	0.25262	0.067337	0.125892
11735228_FAM71E2	-0.11881	0.067369	0.125946
11732375_HTATIP2	0.073698	0.067371	0.125946
11749766_KCNH5	0.208425	0.067376	0.12595
11731937_HFM1	0.17549	0.067397	0.125986
11759753_ATXN7L1	0.124828	0.067409	0.125999
11750843_CLCNKA	-0.10066	0.067409	0.125999
11749159_SCG3	0.120624	0.067414	0.126003
11729630_AP3M1	0.089535	0.067424	0.126016
11727911_ANKRD23 /	-0.11533	0.067453	0.126066
11742087_DCHS2	-0.13393	0.067483	0.126115
11764159_LOC101060	-0.19064	0.067484	0.126115
11738823_SLC26A5	-0.20538	0.067506	0.12615
11742777_TBCC	0.07773	0.067509	0.126152
11742451_TAS1R2	-0.21883	0.06752	0.126167
11740700_CACNG6	-0.08581	0.067537	0.126194
11737646_CTXN2	-0.20809	0.067546	0.126207
11747201_IDS	0.095526	0.067557	0.126221
11730316_RGS7BP	-0.15681	0.067584	0.126267
11736780_ZNF486	0.200207	0.067586	0.126267
11724718_DEXI	-0.07838	0.067589	0.126268
11736184_FSCB	-0.09229	0.067624	0.126328

11753072_FAM170A	-0.14333	0.06767	0.126409
11745756_TMEM41B	0.122809	0.067744	0.126542
11734380_RAD51B	-0.13273	0.067753	0.126554
11715919_SH3BGRL3	-0.07114	0.067767	0.126576
11754790_FAM193A	0.111814	0.067772	0.12658
11715488_PGRMC1	-0.07664	0.067782	0.126595
11740922_C17orf80	0.097848	0.067792	0.126608
11734537_GAS2L3	-0.07754	0.067813	0.126642
11743469_WDFY3	0.140809	0.067828	0.126662
11760556_MGC24103	0.151465	0.067829	0.126662
11735688_C21orf59	-0.08101	0.067831	0.126662
11754228_RPL23AP82	0.090709	0.067837	0.126666
11753114_KCTD7	-0.10696	0.067839	0.126666
11754929_UBE2L3	-0.07247	0.067846	0.126674
11747686_ARFIP2	0.082972	0.067854	0.126686
11743229_PCDHGC4	-0.14118	0.06786	0.126689
11762828_CCT5	0.151348	0.067861	0.126689
11740710_ACAD10	-0.08382	0.06787	0.126698
11716205_NDUFA8	-0.07553	0.067873	0.126698
11750421_PPWD1	-0.09216	0.067874	0.126698
11750358_LOC401296	-0.15993	0.067915	0.12677
11761632_OCIAD1	0.261792	0.067919	0.12677
11746068_NBPF3	0.127164	0.06792	0.12677
11755110_LRIG1	0.082785	0.067941	0.126804
11740399_MCTS1	-0.10942	0.06796	0.126831
11736291_NIPAL2	0.165088	0.067961	0.126831
11720761_FBXO3	0.098237	0.067964	0.126831
11730638_FRMPD1	-0.15763	0.067975	0.126844
11762999_IGH	-0.13821	0.067975	0.126844
11740010_ZFP14	0.115412	0.068004	0.126893
11748466_PIK3CB	0.10728	0.068016	0.12691
11736322_BAIAP3	-0.08999	0.068046	0.126958
11728426_UHRF1BP1	-0.10294	0.068047	0.126958
11742889_FBXO6	-0.11721	0.068061	0.12698
11760213_TMC5	-0.10956	0.068076	0.127003
11724530_APOB	-0.14274	0.06811	0.12706
11747876_EDNRA	-0.23093	0.068139	0.12711
11763907_PABPC4 //	-0.14633	0.068142	0.127111
11755804_SEMA4D	-0.14388	0.068172	0.127162
11722946_MUM1L1	0.150786	0.068184	0.127179
11741221_COLCA2	-0.09207	0.068196	0.127197
11751571_SGCA	-0.11826	0.068211	0.127221
11716090_PRG2	0.092456	0.068219	0.127231
11743265_ENSA	0.134856	0.068223	0.127233
11744592_GON4L	-0.11729	0.068238	0.127255
11760391_CYP2C9	-0.13576	0.068246	0.127262
11752232_ECT2L	-0.11864	0.068246	0.127262
11751402_REM2	-0.09747	0.068259	0.127281
11752524_CELSR3	-0.13604	0.068269	0.127294
11749115_BCR	-0.08688	0.068276	0.127302
11756986_KLHDC1	-0.11739	0.068291	0.127325

11723437_CEP41	-0.09049	0.0683	0.127338
11746560_TREML1	-0.07641	0.06831	0.127346
11730753_AGPAT5	-0.21343	0.06831	0.127346
11735192_ADGRE2	0.278191	0.068325	0.12737
11762244_SLC36A2	-0.21309	0.06833	0.127375
11745614_LINC00161	-0.08619	0.068334	0.127376
11762883_EMG1	0.151199	0.068343	0.127388
11729831_CLDN8	-0.11595	0.068354	0.127404
11764116_LOC100996	0.115292	0.068385	0.127455
11728707_EMP2	-0.07952	0.068386	0.127455
11760448_TXLNGY	0.088398	0.068437	0.127545
11730646_ECEL1	-0.08986	0.068441	0.127546
11741526_SUMO2	-0.07217	0.068469	0.127594
11716821_ATPIF1	-0.07776	0.068511	0.127667
11743386_PRPF40A	-0.07316	0.068523	0.127685
11763594_HS3ST3B1	0.119044	0.068537	0.127706
11735411_FZD9	-0.1471	0.068575	0.127771
11731072_ZNF587	0.081201	0.068579	0.127776
11752059_SARDH	-0.12895	0.068593	0.127796
11742137_OTOL1	-0.1504	0.068603	0.127809
11760890_LOC220729	-0.20206	0.068609	0.127816
11737146_SOCS1	0.190452	0.068644	0.127876
11731333_GPR55	-0.09968	0.068647	0.127876
11718694_YIF1B	-0.12917	0.068683	0.12794
11739275_PAK2	-0.07623	0.068718	0.128
11731144_ADGRD1	-0.11434	0.068732	0.12802
11763819_FAM149A	-0.13365	0.068752	0.128053
11741346_TBC1D32	-0.09399	0.068763	0.128068
11747533_GRSF1	0.080374	0.068784	0.128103
11724047_TAF1C	-0.08266	0.068789	0.128107
11727031_SQSTM1	0.073687	0.068806	0.128134
11717134_MAFG	-0.08902	0.068826	0.128167
11748178_XYLT2	-0.13202	0.068831	0.128171
11762292_TNRC6A	-0.10652	0.068849	0.1282
11749043_SLC22A7	0.111482	0.068858	0.128212
11732127_CCT4	-0.0749	0.068861	0.128213
11742395_CACNG5	-0.1569	0.068864	0.128213
11763396_SEC24D	0.211639	0.068874	0.128226
11741366_TEAD2	-0.12824	0.068889	0.12825
11744266_PLEKHM2	-0.10895	0.068897	0.12826
11732295_ZNF566	-0.13547	0.06894	0.128335
11716573_KDM3A	0.134411	0.068942	0.128335
11751720_ATP1A2	-0.13057	0.068948	0.128341
11759772_USP45	0.083228	0.068965	0.128366
11740224_CHST4	-0.15921	0.068989	0.128407
11734515_TEPP	-0.15652	0.068997	0.128416
11738210_GPR17	0.162675	0.06902	0.128454
11760111_ACTN1	-0.11252	0.069028	0.128464
11737592_EYS	-0.27913	0.069036	0.128474
11750996_ZNF577	0.084935	0.069051	0.128498
11755965_PAFAH1B1	-0.09681	0.069058	0.128506

11723726_USP32 ///	-0.0888	0.069096	0.128572
11743580_ACTR2	-0.08355	0.069103	0.128579
11748578_UROD	-0.08963	0.069122	0.12861
11757352_TRMT1	0.139213	0.069193	0.128732
11727702_C9orf152	-0.07158	0.069193	0.128732
11736155_XPR1	0.111212	0.069204	0.128747
11718378_PRKAG2	0.098594	0.06921	0.128753
11716426_PCDHGA1 /	-0.07628	0.069228	0.128782
11754875_C20orf96	-0.12453	0.069233	0.128787
11727944_ARHGAP8 /	-0.10506	0.069236	0.128788
11747474_NR4A2	-0.09453	0.069304	0.128907
11760433_SLC22A11	-0.08803	0.069305	0.128907
11756070_MT1IP	-0.2032	0.069318	0.128926
11734813_TMEM86A	-0.07073	0.069354	0.128988
11761484_CLK3	0.099368	0.069359	0.128992
11737927_C6orf141	0.150125	0.069363	0.128994
11737579_HLA-L	0.165915	0.069374	0.129011
11715126_HIST1H2BN	0.115063	0.0694	0.129053
11717292_JAM3	-0.09819	0.069422	0.12909
11752064_RGSL1	0.104771	0.069427	0.129095
11729920_BBOF1	0.172215	0.06944	0.129114
11726576_CYP2A6	-0.13847	0.069451	0.129129
11733434_MCF2	0.10588	0.069471	0.129161
11745949_CHEK1	-0.07456	0.069475	0.129163
11738349_POMZP3	-0.08787	0.069478	0.129163
11736188_ORMDL3	0.089964	0.069483	0.129163
11721164_KHNYN	-0.08382	0.069484	0.129163
11758765_HIST2H2BE	-0.1427	0.069488	0.129163
11758766_HIST2H2BE	-0.1427	0.069488	0.129163
11758378_RBMXL1	-0.08793	0.069494	0.129166
11762714_FILIP1L	-0.17307	0.069495	0.129166
11751501_FAXDC2	-0.09182	0.069502	0.129175
11732168_STAG3	-0.10222	0.069537	0.129235
11741263_LTBP1	-0.19093	0.06954	0.129236
11759466_ARL17A //	-0.18876	0.069551	0.129247
11740003_SPATA5	-0.16721	0.069551	0.129247
11740927_C14orf177	-0.18994	0.069554	0.129247
11737126_CTAGE15 /	-0.1101	0.069592	0.129313
11729739_SIGLEC5	0.217164	0.069614	0.129345
11762303_HNMT	0.124628	0.069615	0.129345
11749476_UNC13D	-0.11503	0.069624	0.129357
11751349_ZNF345	0.165367	0.069633	0.129369
11718402_NUDT21	0.078757	0.069643	0.129377
11754918_HMGCS1	0.095903	0.069643	0.129377
11752602_TBC1D17	-0.08855	0.069655	0.129392
11716366_CELA3A //	-0.1034	0.069658	0.129392
11761119_SH3GLB2	-0.10707	0.069663	0.129392
11749221_SLC13A3	0.073647	0.069665	0.129392
11764031_GADD45B	0.121275	0.069667	0.129392
11715308_MLXIPL	-0.08565	0.069667	0.129392
11727976_KLK1	-0.11425	0.069672	0.129397

11727884_ERAP1	-0.15381	0.069685	0.129416
11761048_ANKRD46	-0.13733	0.0697	0.129439
11733261_CELF2	-0.16942	0.069711	0.129455
11719336_TEAD3	-0.13025	0.069718	0.129463
11723036_TRMT12	0.087595	0.069755	0.129526
11745104_PJA1	-0.07886	0.069762	0.129533
11728743_ARAP3	-0.14	0.069764	0.129533
11762195_TCEA3	-0.08843	0.069796	0.129587
11761900_LOC554174	-0.14736	0.069826	0.129639
11759442_DDR2	0.123366	0.069829	0.129639
11747381_SEPT1	-0.14779	0.06989	0.129747
11721671_RPS6KA4	-0.13396	0.069899	0.12976
11728882_PRSS35	0.193625	0.069907	0.129769
11753295_COL1A2	-0.17966	0.069933	0.129812
11735414_CRCP	0.090276	0.069936	0.129814
11732685_POGZ	0.116121	0.069964	0.129859
11754976_CNPY3	-0.09298	0.069977	0.12988
11738199_PDE6B	-0.2065	0.069987	0.129894
11758881_RPS15A	-0.12279	0.069992	0.129894
11719538_BBOX1	-0.10698	0.069993	0.129894
11747347_ARHGEF10L	0.084781	0.07	0.129903
11746998_AGBL2	0.145208	0.070016	0.129926
11761498_FAM129C	-0.12329	0.070035	0.129957
11716803_SBF1	-0.10026	0.070046	0.129974
11736420_ZNF800	0.089682	0.070049	0.129974
11722329_PGBD5	-0.12972	0.070056	0.129982
11750340_ADGRD2	-0.12908	0.07006	0.129984
11744745_TNIP1	-0.0886	0.07008	0.130016
11750736_STEAP4	-0.1458	0.070088	0.130024
11747829_TCF7L2	0.134346	0.070089	0.130024
11745436_ZNF148	-0.08492	0.070094	0.130027
11750225_RNF215	-0.11086	0.070101	0.130035
11745115_SLC1A2	-0.11413	0.070153	0.130124
11759860_GRIK1-AS2	0.09187	0.070154	0.130124
11733937_PAIP1	-0.07913	0.070179	0.130166
11734945_CYSLTR2	-0.19216	0.07021	0.130217
11720871_PDE7A	0.076591	0.070223	0.130238
11756969_ALB	-0.15186	0.070232	0.130249
11720814_INTS10	-0.07422	0.070235	0.13025
11750713_GRM4	-0.10051	0.070254	0.130275
11762583_GRIK2	-0.13552	0.070255	0.130275
11728504_BDKRB1	0.121752	0.070257	0.130275
11759074_BRSK2	-0.15124	0.07026	0.130276
11732039_TPD52L3	0.278455	0.070292	0.130329
11756727_PEX14	-0.09963	0.070294	0.130329
11753297_BIRC7	-0.1048	0.070298	0.130332
11725933_HSPA5	-0.07652	0.070402	0.130517
11724310_SH3BP2	0.104951	0.070406	0.130517
11760061_FCRL2	-0.21454	0.070406	0.130517
11759489_TXNL1	-0.09114	0.070431	0.130559
11762230_HAAO	-0.13549	0.070439	0.130568

11725852_ZNF319	-0.10438	0.070442	0.130568
11719435_LRP4	-0.0858	0.070466	0.130608
11726821_CXorf36	-0.21529	0.070469	0.13061
11717707_VPS13D	-0.14679	0.070484	0.130632
11749442_PYCRL	-0.0885	0.070505	0.130666
11751979_MYOM3	0.193351	0.07052	0.130689
11746600_MCOLN3	0.164366	0.070542	0.130725
11748819_FRMD1	-0.10992	0.070548	0.130731
11729102_CDC42	-0.08701	0.070559	0.130747
11763530_TMC5	-0.14051	0.070577	0.130775
11725206_OTOR	-0.20719	0.070604	0.130819
11752595_HSPA9	0.073393	0.070608	0.130822
11732955_FDXR	0.100178	0.070632	0.130862
11737252_SLC16A11	-0.10328	0.070646	0.130879
11741257_APOBEC3F	0.120149	0.07065	0.130879
11750726_ZNF568	0.187483	0.070652	0.130879
11749604_CYP4F2 //	0.085165	0.070652	0.130879
11718950_MPP5	0.094617	0.070661	0.13089
11763611_GLI2	-0.15655	0.070672	0.130907
11756713_WIPF2	-0.13138	0.07068	0.130915
11733704_TMEM229A	0.138963	0.070684	0.130918
11729956_KNG1	-0.09135	0.070687	0.130919
11733394_TRPM6	-0.08689	0.070691	0.130921
11737469_TMEM240	-0.20846	0.070698	0.13093
11727444_TMEM255A	0.134901	0.07071	0.130943
11717158_ZNF593	0.08362	0.070711	0.130943
11734585_KLRC4	0.249831	0.070725	0.130965
11736769_USP45	-0.12435	0.070759	0.131023
11750330_MYOCD	-0.09477	0.070772	0.131039
11757414_PUF60	-0.07105	0.070773	0.131039
11727906_MMP10	0.333315	0.070782	0.13105
11721887_CRYM	-0.17489	0.070788	0.131055
11725675_RORA	0.107808	0.0708	0.131072
11723577_OSR2	0.31	0.070802	0.131072
11750877_ANKS3	0.092734	0.070805	0.131073
11733263_BSDC1	-0.08558	0.070841	0.131135
11740728_KRTAP5-3	-0.12394	0.070844	0.131136
11734145_ZNF778	0.18345	0.070882	0.131201
11742348_OR51I2	-0.21577	0.070897	0.131223
11732167_STAG3	-0.16467	0.070924	0.131269
11756324_WDR26	0.102889	0.070929	0.131273
11751722_FBXO3	0.090571	0.070969	0.13134
11738184_PARD3B	-0.11675	0.070971	0.13134
11757411_MRPL33	0.108262	0.07099	0.131371
11735012_DYNLL2	0.093845	0.071026	0.131432
11748124_CIRBP	-0.10308	0.071032	0.131435
11737261_BICC1	-0.14217	0.071033	0.131435
11747164_U2SURP	-0.08428	0.071045	0.131451
11738868_SLC2A7	-0.13393	0.071047	0.131452
11734705_LAMA4	-0.12017	0.071086	0.131518
11717029_BAP1	-0.11519	0.071097	0.131534

11751266_SLC44A3	0.173359	0.071107	0.131547
11733948_PAX9	0.127787	0.071115	0.131556
11743561_PTPRC	0.129088	0.071117	0.131556
11720927_SRM	0.073241	0.071133	0.131581
11736119_ZFAND5	0.093422	0.071163	0.131631
11715816_ZFP36L2	0.119572	0.071179	0.131656
11730460_C17orf74	-0.12893	0.071206	0.131701
11749162_ZSCAN30	-0.10217	0.071216	0.131713
11736459_NOX1	-0.08875	0.071218	0.131713
11759358_PYGO1	0.196153	0.071223	0.131716
11738095_FASTK	-0.10685	0.071239	0.131741
11756218_MIIP	-0.07426	0.071243	0.131745
11735890_TRIM14	-0.09184	0.071298	0.131841
11732550_PTGER2	-0.13536	0.071314	0.131866
11729248_RNF24	0.166106	0.071331	0.131893
11720412_SLC41A1	0.084136	0.071361	0.131942
11736916_C17orf58	-0.0739	0.071377	0.131964
11741382_ZNF114	-0.1822	0.071378	0.131964
11725628_CNP	-0.18034	0.071381	0.131964
11760249_ZNF511	-0.23748	0.071386	0.131969
11760670_FMO3	-0.11335	0.071434	0.132048
11746739_ORC2	-0.08902	0.071435	0.132048
11743133_FKBP8	-0.0762	0.071453	0.132078
11760884_ZNF397	0.261121	0.071504	0.132167
11719708_PDPK1	0.128353	0.071517	0.132185
11724473_AMPH	-0.13069	0.071544	0.132231
11743601_OTUD5	0.093261	0.07155	0.132234
11726856_ADAMTS5	0.228589	0.071552	0.132234
11715972_SMOC2	-0.08869	0.071569	0.132262
11755148_DMXL2	0.093765	0.071574	0.132266
11744183_ACADS	-0.08068	0.071589	0.132288
11729871_ASGR2	-0.20704	0.0716	0.132304
11728593_FOXL2	-0.13548	0.071607	0.132311
11748344_IFT22	0.071995	0.071611	0.132314
11739062_GTPBP4	-0.08579	0.07162	0.132326
11738914_CD200R1 /	-0.11828	0.07163	0.132339
11751186_UBE2K	0.122523	0.071632	0.132339
11725275_IRX1	-0.12406	0.071655	0.132375
11729302_GLYAT	-0.19645	0.071675	0.132408
11742971_COLEC12	-0.09498	0.071679	0.13241
11734979_BTNL3	0.133535	0.071683	0.132413
11761133_KDM5C	-0.21671	0.071726	0.132487
11739915_FAM13C	0.151185	0.071729	0.132487
11720343_NUDT4	-0.09138	0.071771	0.13256
11744309_DBH	-0.13757	0.071774	0.13256
11753209_HYAL1	-0.13401	0.07179	0.132586
11715102_HIST1H3G	-0.13326	0.071799	0.132592
11722130_ECHDC2	0.083338	0.071799	0.132592
11759806_WBSCR16	-0.08181	0.071866	0.13271
11755280_FTL	0.075403	0.071878	0.132727
11740168_HUNK	-0.10796	0.071884	0.132734

11737537_SAMD12	0.10698	0.071888	0.132736
11743200_ARMC2	-0.108	0.071911	0.132774
11728567_KCNC1	-0.11742	0.071914	0.132774
11727347_ITPKA	-0.07004	0.071963	0.132859
11752603_RTCB	-0.09876	0.071982	0.132885
11732300_KIAA1715	-0.1174	0.071985	0.132885
11745416_DIAPH1	-0.07352	0.071985	0.132885
11724457_HCN4	-0.15017	0.072036	0.132975
11740732_SYT14	0.110471	0.072048	0.132988
11760151_LOC730098	-0.09688	0.072049	0.132988
11760447_FAM124A	0.170094	0.072114	0.133102
11729761_PLA2G2D	-0.13885	0.07213	0.133127
11731822_WISP1	-0.09254	0.072133	0.133127
11751876_ADGRG4	-0.19503	0.072141	0.133137
11746927_FAM155A	-0.16339	0.072158	0.133164
11730762_CRIPT	0.101483	0.072168	0.133173
11756251_RPS5	0.075246	0.072169	0.133173
11763417_HCG8 ///	0.110438	0.072177	0.133183
11751638_NT5C	-0.08269	0.072185	0.133193
11754873_ZNF641	0.104884	0.072194	0.133205
11735976_NR1I3	0.172013	0.072204	0.133218
11760533_ALDH9A1	0.102686	0.072232	0.133266
11725843_SCP2D1	-0.18151	0.072248	0.133289
11746530_SLC25A15	0.095896	0.072256	0.133299
11738662_TUBB7P	-0.16148	0.072265	0.133311
11751942_DNMT3B	-0.14507	0.072274	0.133323
11751071_PABPC5	-0.098	0.072281	0.13333
11722146_PAIP2B	-0.13152	0.072285	0.133333
11743668_CEP120	-0.10513	0.072291	0.133338
11733368_HIST1H4I	-0.2643	0.072308	0.133365
11754536_ERCC2	0.127877	0.072314	0.133372
11756345_TMEM91	-0.14072	0.072325	0.133387
11738164_C11orf42	-0.09759	0.072337	0.133403
11722716_STK35	-0.1079	0.072343	0.133406
11742866_PHACTR1	0.3083	0.072344	0.133406
11732083_STOML3	-0.12292	0.072368	0.133446
11730658_MAPK15	-0.2076	0.072375	0.133453
11757457_NENF	0.098472	0.072381	0.13346
11722383_MDM1	-0.11853	0.07239	0.133471
11728204_FAM120A	-0.07929	0.072395	0.133475
11740249_CFHR5	-0.16835	0.072423	0.133516
11763190_SIGIRR	-0.14824	0.072427	0.133516
11725287_ANKS6	0.138164	0.072428	0.133516
11747030_NETO1	0.098742	0.072428	0.133516
11728533_SNX3	-0.08047	0.072462	0.133574
11731850_COLQ	0.243149	0.072478	0.133598
11748654_PTPN22	0.089781	0.072481	0.133598
11748899_CD244	0.188138	0.072498	0.133625
11763822_EREG	-0.16121	0.072509	0.133639
11763958_FCGRT	-0.16774	0.072515	0.133647
11718069_NCOR2	-0.12392	0.072535	0.133677

11761766_CNTROB	-0.12349	0.072551	0.133701
11759636_THOC2	0.07134	0.072553	0.133701
11736538_RHCE ///	-0.3252	0.072556	0.133702
11726423_SCN3B	-0.17641	0.07256	0.133704
11755353_MYO7B	-0.14138	0.072567	0.133708
11729064_OPA3	0.119834	0.072568	0.133708
11746322_ADH1B	-0.1189	0.072581	0.133727
11730659_OXSM	-0.08202	0.07259	0.133739
11764258_LOC102723	-0.23504	0.072619	0.133787
11727827_NDUFAF7	-0.15122	0.072623	0.133789
11726465_ADORA3 //	-0.18239	0.072625	0.133789
11745857_SGSM3	-0.0958	0.072649	0.133828
11753475_BRF1	-0.11443	0.072667	0.133855
11742646_LINC00207	-0.14363	0.072696	0.133904
11750434_SPIDR	-0.09621	0.072704	0.133913
11761933_RPS4X	-0.23206	0.072738	0.133969
11728353_MSMP	-0.11896	0.07274	0.133969
11732884_F7	-0.19438	0.072775	0.134025
11761437_VRK3	-0.21702	0.072775	0.134025
11756370_CRIP2	-0.0758	0.072795	0.134057
11722236_FBXO2	0.210007	0.07283	0.134115
11747806_RNGTT	0.077364	0.072838	0.134122
11726137_LDB3	-0.08224	0.072839	0.134122
11741970_SIM2	-0.17476	0.07286	0.13415
11762986_ARL2BP	-0.08985	0.07286	0.13415
11763961_SFTPC	-0.09216	0.072874	0.134165
11735682_XKR7	-0.1459	0.072874	0.134165
11736371_MBOAT1	-0.16389	0.072876	0.134165
11733411_TCF15	-0.1017	0.072894	0.134185
11725306_CALU	0.079834	0.072894	0.134185
11751799_PRDX6	0.078354	0.072896	0.134185
11741417_EIF4E	0.088786	0.072977	0.134331
11744747_FGFR1OP2	-0.06862	0.073002	0.134371
11750418_PLEKHD1	-0.09167	0.073013	0.134385
11740507_ARHGAP26	0.278626	0.073015	0.134385
11760379_GTPBP6	-0.14283	0.073075	0.134488
11739748_KLHL7	-0.09954	0.073077	0.134488
11747126_ART3	-0.13292	0.07308	0.13449
11735836_PPHLN1	0.072776	0.073099	0.134516
11749038_ARHGEF2	-0.08131	0.0731	0.134516
11763790_SYCP1	-0.12116	0.073105	0.13452
11744940_ABHD17A	0.086771	0.073109	0.134522
11742516_OR2T4	-0.11248	0.073132	0.134559
11716882_ZZEF1	0.151955	0.073147	0.134582
11726877_GJB5	-0.14222	0.073218	0.134708
11742499_ARID3C	-0.14818	0.073224	0.134713
11725170_PPARD	-0.1438	0.073275	0.134803
11761406_TPRKB	-0.09987	0.073286	0.134813
11758639_HSPH1	0.081316	0.073286	0.134813
11753833_DYNLL1	0.076711	0.073295	0.134824
11724870_IPO8	0.093422	0.073317	0.134861

11762463_C8orf17	0.120345	0.073347	0.134909
11726914_ZNF182	0.080898	0.073378	0.134961
11738638_TGM6	-0.12149	0.073388	0.134975
11744623_POLDIP2	0.070412	0.0734	0.134992
11726304_MFSD4	0.140744	0.073423	0.135028
11732335_SLC31A1	0.093409	0.073428	0.135033
11741867_P2RX2	-0.08708	0.073435	0.135041
11730641_UNC93A	-0.08905	0.073443	0.135051
11738124_SIGLEC10	0.164346	0.073475	0.135105
11751275_CALCOCO2	-0.0887	0.073487	0.135122
11754762_TCIRG1	0.069005	0.073499	0.135138
11754988_SYNPO	-0.11867	0.073506	0.135141
11729912_IBA57	-0.11457	0.073511	0.135141
11754378_SERPINB6	-0.07683	0.073511	0.135141
11753514_PNRC1	0.099094	0.073511	0.135141
11737639_CST9	-0.12051	0.073525	0.135161
11743235_PKD1L3	-0.16299	0.073531	0.135164
11758195_TPT1	0.072031	0.073532	0.135164
11719016_PQLC3	0.069319	0.073544	0.13518
11740802_INVS	0.125054	0.07355	0.135187
11758097_MATN3	-0.19726	0.073564	0.135203
11732416_TIAM1	0.109275	0.073565	0.135203
11750978_CRHR2	-0.0705	0.073599	0.135258
11721616_THEMIS2	-0.0706	0.0736	0.135258
11725978_NCBP1	0.079732	0.073638	0.135323
11743184_CYB5R1	-0.11768	0.073646	0.135332
11756953_IL1R1	-0.19077	0.07365	0.135335
11728535_BGLAP	0.110381	0.073655	0.135339
11749429_RPN1	0.083377	0.073666	0.135354
11729477_LY6G6C	-0.07707	0.073673	0.135362
11726502_LSAMP	0.128309	0.073695	0.135393
11732619_CD164L2	-0.13099	0.073696	0.135393
11729273_SH3RF2	0.082462	0.073711	0.135415
11729962_ADCYAP1	-0.12213	0.073724	0.135434
11741664_PCDHGA11	-0.14971	0.073731	0.135443
11749186_CDADC1	-0.14263	0.073739	0.135452
11719640_PLXNA1	0.094276	0.07375	0.135467
11757266_GPX3	-0.15449	0.073759	0.135479
11725823_JPH1	-0.12969	0.073775	0.135503
11724155_SULT1E1	-0.14831	0.073807	0.135557
11738952_LCN9	0.10757	0.073812	0.135562
11720385_MRC2	-0.11848	0.07382	0.13557
11747336_MED24	-0.08126	0.073835	0.135593
11759930_PHTF1	0.099223	0.073845	0.135607
11752451_PLPPR4	-0.11188	0.073865	0.135638
11751241_FAM47E-ST	-0.12979	0.07389	0.135679
11747022_ATP13A4	-0.11098	0.0739	0.135691
11748121_CD96	0.069224	0.073931	0.135743
11758660_DLX4	-0.11619	0.073947	0.135768
200064_PMHSP90AB1	-0.06948	0.073953	0.135775
11753227_DDX11L2	-0.08267	0.073967	0.135794

11762083_CSNK2A1	-0.17802	0.073989	0.135829
11762807_COMMD5 //	-0.09172	0.074013	0.135868
11732026_CRIP3	-0.08234	0.074025	0.135886
11750553_CASP1	0.101333	0.074067	0.135958
11761716_COA3	-0.11332	0.074087	0.135987
11729822_DPH7	0.140396	0.074088	0.135987
11737239_EOMES	-0.20033	0.074095	0.135993
11753615_ARL8B	0.080213	0.074122	0.136035
11748088_AKAP1	-0.09407	0.074123	0.136035
11730983_ZFYVE9	0.106532	0.074157	0.136093
11718806_PUM2	-0.0991	0.074201	0.136168
11745455_ABCB8	-0.11641	0.074222	0.136201
11739607_BAIAP2	-0.08	0.074246	0.136241
11730189_OLA1	0.076165	0.074252	0.136247
11748433_NDUFC2	-0.08167	0.074281	0.136294
11719784_RPS23	0.080849	0.074284	0.136294
11732052_ZSCAN2	-0.14156	0.07429	0.1363
11754219_SIPA1L1	-0.09832	0.074335	0.136377
11721299_MRPS21	-0.08716	0.074379	0.136453
11756030_PQBP1	0.079125	0.074384	0.136458
11719495_CLK2	-0.07518	0.074398	0.136475
11724157_SULT1E1	0.110458	0.074399	0.136475
11755801_IGSF22	-0.07779	0.074412	0.136493
11741426_ISLR2	-0.15611	0.074445	0.136549
11762563_WHSC1	-0.18736	0.07445	0.136553
11750710_CPEB3	0.147173	0.074476	0.136595
11734128_CYP11B1	-0.12996	0.07448	0.136597
11740691_AIF1	-0.10621	0.074489	0.13661
11719047_WEE1	0.081238	0.074508	0.13664
11761801_MAP4K2	-0.11203	0.074521	0.136658
11721568_C9orf3	0.078573	0.074527	0.136663
11763348_CYP3A7	0.09461	0.074531	0.136666
11715152_PCDHGB1	0.117953	0.074544	0.136681
11749270_GPRC5B	-0.20671	0.074545	0.136681
11730628_SPHKAP	-0.10832	0.074557	0.136699
11750117_PHF2	0.101695	0.074574	0.136723
11761440_MICAL2	-0.09606	0.074603	0.136768
11754731_MTA3	0.087578	0.074604	0.136768
11736414_MFI2	-0.18498	0.074611	0.136776
11723478_CDC123	-0.08121	0.074613	0.136776
11721690_CYB561	-0.11391	0.074622	0.136787
11747057_C10orf142	-0.16175	0.074646	0.136825
11739330_WDR82	0.0817	0.074669	0.136863
11751021_LRRC15	-0.15794	0.07468	0.136877
11763726_RPL10	0.075041	0.074682	0.136877
11747300_CDK14	-0.08756	0.074699	0.1369
11758666_FAM230A /	-0.1407	0.074701	0.1369
11720837_HIRA	0.081671	0.074705	0.136902
11744865_MAGI2	0.15226	0.074711	0.136909
11737708_ORC5	0.110306	0.074714	0.136909
11738161_NANOS2	-0.10493	0.074732	0.136932

11717477_CHGB	0.20253	0.074732	0.136932
11741195_SIGLEC14	0.121949	0.074738	0.136937
11735104_HDLBP	-0.09439	0.074759	0.136971
11730230_TIAL1	0.075556	0.074861	0.137152
11726229_HTR3A	-0.13537	0.074887	0.137195
11760270_CDC16	0.145229	0.074894	0.137203
11763210_ZMYND8	-0.12464	0.074904	0.137216
11730504_ADAMTS2	-0.09847	0.074918	0.137236
11724597_KIFC2	-0.11836	0.074923	0.13724
11761188_VIL1	0.075702	0.07494	0.137266
11759815_IGHM	-0.08681	0.074943	0.137267
11736453_ZNF197	0.075784	0.074986	0.137337
11748908_ORAI2	0.12382	0.074987	0.137337
11727290_CDK3 ///	0.161529	0.074992	0.137341
11738896_CD200R1L	-0.14435	0.075046	0.137435
11739565_FKBP5	-0.13582	0.075065	0.137464
11756173_INTS9	-0.12073	0.075088	0.137501
11720678_CREB3L1	-0.15518	0.075099	0.137516
11747785_LCP1	0.08836	0.075116	0.137537
11731208_PTPRH	0.107002	0.075116	0.137537
11761692_CSMD2	0.165009	0.075156	0.137606
11726746_TAOK2	-0.12437	0.07517	0.137625
11755326_ABHD16A	0.118083	0.075173	0.137625
11740438_FUT6	-0.33771	0.075214	0.137695
11760733_CCNY	-0.12204	0.07522	0.137696
11727140_MUSTN1	-0.12999	0.075224	0.137696
11719967_HTT	-0.08839	0.075225	0.137696
11725163_CYP39A1	0.21282	0.075225	0.137696
11751547_HDAC6	-0.15544	0.075231	0.137702
11738377_IFNA2	0.126838	0.075238	0.137709
11728089_DKK2	0.113992	0.075256	0.137737
11743173_SLCO4A1	-0.07006	0.07527	0.137757
11729747_IL10RA	-0.09397	0.075287	0.137784
11733959_GTPBP3	-0.09872	0.075293	0.137789
11762687_MUC1	0.079004	0.075299	0.137791
11762113_NXF3	-0.09372	0.0753	0.137791
11729590_KLK2	-0.11895	0.075339	0.137859
11729784_CMTM2	-0.09035	0.075347	0.137868
11739994_TMEM25	0.141424	0.075388	0.137937
11763491_TTC39A	0.135169	0.075395	0.137944
11757720_EVPLL	-0.10107	0.075401	0.13795
11757128_RPSA ///	-0.13065	0.075405	0.137953
11744754_DIS3L2	0.15094	0.075419	0.137973
11715426_SNU13	-0.0754	0.075423	0.137976
11760894_SRSF5	-0.13897	0.075446	0.138012
11715841_MAPK1	-0.09438	0.075448	0.138012
11739946_PKP1	-0.11943	0.075461	0.138029
11721198_TMEM119	-0.08691	0.075474	0.138048
11762076_SERHL2	0.220768	0.075493	0.138077
11740527_DEFB118	-0.10079	0.075526	0.138129
11727410_MRPL19	0.096636	0.075527	0.138129

11743043_FBLN5	-0.1455	0.07555	0.138166
11748352_WDR81	-0.13895	0.075561	0.138181
11729978_CARD18	-0.09648	0.075582	0.138215
11725856_DNAH1	0.116422	0.075589	0.138222
11726959_GRIN2A	-0.15272	0.075603	0.138242
11740647_CREBBP	-0.12897	0.075629	0.138284
11733466_NEMP1	0.224006	0.075639	0.138298
11737167_ANKRD34B	-0.15723	0.075656	0.138324
11735469_OPN1SW	-0.0974	0.075663	0.138331
11757374_RPL18A	0.068777	0.075677	0.138352
11762321_TRAV12-2	0.12616	0.075697	0.138383
11738075_PDE4A	-0.11752	0.075735	0.138447
11744082_MRPS31	0.077875	0.075745	0.138461
11715136_OR8U1 ///	-0.134	0.075751	0.138466
11730844_PIK3R3	0.092195	0.075801	0.138551
11758900_YIPF4	-0.19882	0.075803	0.138551
11732683_ABCA12	0.132583	0.075814	0.138566
11719019_CBFB	-0.08736	0.075847	0.138621
11749486_DIP2B	0.071292	0.075863	0.138641
11730490_UNC5A	-0.08905	0.075864	0.138641
11755792_PPFIA3	-0.09071	0.075908	0.138717
11715502_RPL18A	0.068658	0.075922	0.138737
11741726_PIP5K1A	0.114546	0.075933	0.138752
11755650_MROH2B	-0.13652	0.075977	0.138828
11761902_FAM186A	-0.16875	0.075997	0.138859
11733736_CD2	0.137968	0.076021	0.138898
11755575_KCNU1	-0.14052	0.076043	0.138932
11763722_SIRT6	0.107225	0.076048	0.138937
11726748_TMEM57	-0.09772	0.076056	0.138946
11734645_PCDHA3	0.128006	0.076121	0.139059
11760164_TRDN	0.103886	0.07619	0.13918
11757645_FIBIN	-0.12181	0.076208	0.139208
11739871_DNAJB2	0.108178	0.076213	0.139213
11746975_MLC1	-0.08918	0.076221	0.139222
11741169_MC2R	-0.16113	0.07624	0.139251
11742428_HR	-0.07954	0.076261	0.139284
11748424_DDX3Y	-0.1326	0.076264	0.139285
11734672_NFATC4	-0.08491	0.076272	0.139294
11736123_FCGRT	-0.14503	0.076284	0.139307
11721345_PLEK2	0.198381	0.076285	0.139307
11752270_TRPC7	-0.08005	0.076307	0.139343
11740794_C1orf220	-0.17726	0.076339	0.139395
11723152_GON4L	0.090007	0.076359	0.139427
11724710_SOX11	-0.15281	0.076393	0.139483
11754331_SYDE1	0.111302	0.076404	0.139497
11762104_MOCS1	-0.22537	0.076437	0.139548
11722403_PLEKHO1	-0.08716	0.076437	0.139548
11732872_MTRF1	-0.15556	0.076465	0.139595
11720402_EFNB2	-0.07998	0.076475	0.139606
11734225_LIMCH1	-0.10587	0.07652	0.139684
11759799_ZDHHC11	0.227373	0.076535	0.139705

11744458_RAB2B	-0.12989	0.076551	0.139731
11729014_CACNA1E	-0.10249	0.076574	0.139766
11736550_RAB29	0.084057	0.076593	0.139797
11720874_CORO2B	-0.10749	0.076609	0.139821
11762140_BTNL9	-0.08379	0.076618	0.139831
11740906_CCDC129	0.212394	0.076626	0.13984
11746794_NADK2	0.070593	0.076683	0.13994
11739528_SECTM1	0.104327	0.07669	0.139948
11760687_CCDC178	-0.19024	0.076726	0.140009
11761721_GATB	-0.21441	0.076747	0.140039
200053_PMSPAG7	0.07321	0.076749	0.140039
11715262_MTNR1A	-0.1143	0.076752	0.14004
11718946_PGPEP1	0.132757	0.07679	0.140104
11744137_CAMK1D	-0.08107	0.076798	0.140114
11741027_BTBD3	0.088572	0.076807	0.140123
11728779_ZAP70	-0.11072	0.076812	0.140127
11736049_FAM229B	-0.10556	0.076843	0.140178
11740387_SHROOM4	-0.09355	0.076845	0.140178
11753539_ZNF573	0.120008	0.076854	0.140188
11718050_BASP1	-0.1071	0.07686	0.140196
11747120_HS6ST1	-0.0874	0.076876	0.140219
11763703_ZNF839	-0.11991	0.076915	0.140284
11721594_ARHGAP39	0.160181	0.07693	0.140306
11727568_OTULIN	0.111226	0.076979	0.14039
11763054_YIPF7	-0.11904	0.076996	0.140417
11759329_B3GAT2	-0.17866	0.077009	0.140435
11730227_QRSL1	-0.07696	0.07705	0.140504
11750846_SLC22A6	-0.10819	0.077062	0.140521
11722411_SLC7A1	0.078439	0.077074	0.140537
11755027_TGFB1I1	-0.1227	0.077078	0.140539
11755949_PUS10	0.086009	0.077082	0.14054
11727443_MPO	-0.12513	0.077086	0.14054
11728934_LELP1	-0.14828	0.077087	0.14054
11736696_HEMGN	-0.13163	0.07709	0.140541
11733223_ZNF71	0.116313	0.077106	0.140565
11730916_RBBP6	0.071896	0.077129	0.140601
11734482_ETV3L	-0.07269	0.077132	0.140602
11716825_FMOD	-0.14014	0.077152	0.140632
11741542_CNTN5	-0.08916	0.077157	0.140637
11736832_PEX26	-0.10921	0.077201	0.140712
11762045_GLP2R	-0.11262	0.077222	0.140745
11743811_TFAM	0.085597	0.077238	0.140768
11739347_PIP4K2A	0.196156	0.077248	0.140782
11734055_CFAP61	-0.10987	0.077254	0.140787
11758646_MICU1	-0.07745	0.077259	0.140789
11732088_ISX	-0.15991	0.077261	0.140789
11729205_DTNA	-0.21171	0.077301	0.140852
11726913_ZNF182	-0.18661	0.077303	0.140852
11730911_SPRR1A	-0.18148	0.077304	0.140852
11761612_DDX11	-0.10397	0.077335	0.140902
11718852_SRPK1	-0.07395	0.077337	0.140902

11740271_MAP10	-0.07436	0.077348	0.140917
11745318_ALDH1A3	-0.15681	0.077367	0.140945
11751670_G6PC	-0.1399	0.077379	0.140962
11730141_AKIP1	0.101442	0.077385	0.140967
11731591_NOC4L	-0.06979	0.07739	0.140973
11749716_SLC4A8	-0.18028	0.077407	0.140998
11740762_GCC2 ///	0.100824	0.077413	0.141003
11756981_HDAC7	-0.08509	0.077424	0.141018
11741859_ZAR1	-0.14805	0.077441	0.141044
11756306_PARP9	0.158596	0.07746	0.141073
11742388_OR5J2	-0.13822	0.077487	0.141113
11758524_GRIK2	0.174811	0.077487	0.141113
11749452_ZFYVE27	0.113594	0.077492	0.141116
11747167_ADH1B	-0.12315	0.077516	0.141151
11733818_FHL1	0.116127	0.077517	0.141151
11738739_OR6C4	-0.11742	0.077523	0.141157
11724294_HPCA	-0.08071	0.077527	0.141159
11730150_ENPP7	-0.12557	0.077537	0.141172
11756002_NBEAL1	0.108558	0.077569	0.14122
11756904_STAB2	-0.13776	0.077569	0.14122
11761021_LOC100507	0.078872	0.077576	0.141227
11717318_GPAT4	-0.16287	0.077603	0.14127
11741291_REG3G	-0.1349	0.077615	0.141287
11744374_HLA-DRB1	0.079214	0.077626	0.141302
11716553_RHBDD2	-0.08531	0.077633	0.141308
11716906_CDH1	-0.10493	0.077646	0.141327
11740760_TRPA1	-0.17981	0.077658	0.141343
11736111_ARHGAP18	0.086325	0.077668	0.141357
11735880_MYBBP1A	-0.0956	0.077676	0.141366
11749896_FMOD	-0.16785	0.077682	0.141373
11741009_FGF12	-0.08911	0.077701	0.141402
11726804_HELZ2	-0.14561	0.07771	0.141413
11717578_VPS26A	0.084123	0.077725	0.141432
11755868_CLCC1	0.097224	0.077727	0.141432
11746237_AKTIP	-0.2257	0.077739	0.14145
11736234_PTER	-0.1052	0.077759	0.14148
11760815_C21orf58	-0.10882	0.077765	0.141486
11761095_ZNF614	-0.08368	0.077774	0.141496
11718409_STXBP2	-0.07578	0.077776	0.141496
11722703_DZIP1	-0.08836	0.077803	0.141539
11745390_PC	-0.09352	0.077805	0.141539
11737863_PIGF	-0.11106	0.077819	0.141558
11760872_TNFRSF1A	0.117383	0.077825	0.141563
11727744_CAMK2N2	-0.12978	0.077827	0.141563
11746256_ORM2	-0.09784	0.077858	0.141614
11754699_NPHP4	-0.10406	0.077865	0.141621
11744981_ZNF541	-0.10926	0.077884	0.141645
11757809_KIF5B	0.0705	0.077884	0.141645
11734916_METTL4	-0.11489	0.077918	0.141697
11751579_TTC31	-0.09001	0.077918	0.141697
11760435_CBR4	0.204928	0.077954	0.141757

11717975_METTL3	-0.07973	0.077965	0.141772
11731361_TREH	-0.11651	0.077978	0.14179
11719658_TF	-0.11356	0.077986	0.141799
11748897_AQP7	-0.07573	0.077997	0.141814
11746514_KIF21B	-0.10695	0.07803	0.141868
11721988_CCDC191	0.115313	0.078046	0.141891
11754127_BRMS1	-0.0727	0.078054	0.141902
11752667_LZTS1	-0.15384	0.078061	0.141908
11725293_USP9Y	-0.18719	0.078076	0.14193
11731666_PDGFB	0.149595	0.078091	0.141952
11744943_RHBDL1	0.109277	0.078096	0.141956
11744463_CNPY2	-0.07202	0.078136	0.142024
11738078_AVPR1B	-0.09107	0.078152	0.142048
11762100_NRP2	-0.11685	0.078196	0.142122
11746089_LTBP4	-0.08989	0.078202	0.142127
11747509_FAM107B	-0.0788	0.078271	0.142248
11722677_STEAP3	-0.11588	0.078299	0.142294
11751102_TCF7L2	0.11372	0.078322	0.142331
11756921_TRPC3	-0.14667	0.078416	0.142495
11724525_FAHD1	0.080143	0.078441	0.142537
11746133_TRA2A	0.105549	0.078461	0.142566
11716556_TNS1	0.096121	0.078518	0.142666
11735223_KLRG1	-0.11969	0.078525	0.142673
11759934_C22orf15	-0.12357	0.078535	0.142685
11737060_MTMR3	0.128652	0.078553	0.142712
11725397_TWISTNB	0.085244	0.078555	0.142712
11744832_RPAIN	0.077768	0.07856	0.142715
11745701_METRNL	0.103529	0.078563	0.142716
11738022_MEIOC	0.135899	0.078581	0.142743
11735658_LIG3	-0.17126	0.078595	0.142762
11731878_REC114	-0.12808	0.078619	0.142801
11732257_SHB	-0.1296	0.078631	0.142818
11728920_RASGRP1	-0.11031	0.078645	0.142837
11750743_EPN1	-0.09784	0.078669	0.142876
11731744_TRDMT1	-0.17579	0.078688	0.142899
11729819_IGF1	-0.08818	0.07869	0.142899
11747141_KIF26B	-0.09885	0.078693	0.142899
11730988_MCPH1	-0.16765	0.078693	0.142899
11740684_HEPHL1	-0.11827	0.078714	0.14293
11730032_MKX	0.185437	0.078722	0.142939
11762781_WRB	-0.1258	0.07873	0.142949
11737245_AGAP5	0.072045	0.078738	0.142958
11747912_PAPD7	-0.11184	0.078741	0.142958
11737693_LOC391343	-0.11378	0.078762	0.142992
11724982_PCDH7	0.19688	0.078774	0.143008
11756552_SMARCAD1	0.085682	0.07878	0.143013
11742344_OR4K1	-0.11908	0.078783	0.143013
11751204_GPM6A	0.091158	0.078815	0.143066
11716216_E2F4	0.084007	0.07884	0.143107
11721963_SLC4A8	0.157203	0.078851	0.143121
11725693_BOC	-0.10995	0.0789	0.1432

11740965_GTF2A1	-0.09131	0.0789	0.1432
11757814_SLC25A30	-0.08141	0.078926	0.143242
11747070_PRUNE	0.097974	0.078935	0.143253
11754090_SNCA	0.096369	0.078954	0.143282
11757826_DHX8	0.081961	0.07896	0.143286
11749987_CEP72	-0.07829	0.078973	0.143306
11722144_USP37	0.106152	0.079004	0.143356
11754788_POLD4	0.092911	0.079019	0.143378
11761044_BCRP3	-0.07867	0.079068	0.143463
11756164_ADIPOR2	-0.08085	0.079072	0.143463
11738909_H1FOO	-0.18351	0.079077	0.143468
11758943_AK4	0.15391	0.079087	0.14348
11720019_RTEL1 ///	-0.10661	0.079099	0.143497
11717830_TSC22D3	0.166145	0.079104	0.1435
11728928_CPNE7	-0.0949	0.079108	0.143502
11753299_FCF1	-0.09711	0.079145	0.143563
11754352_RNF213	0.109494	0.079147	0.143563
11748190_MYRF	-0.09484	0.079152	0.143566
11744851_DDX31	0.082109	0.079167	0.143587
11722346_ACSS2	0.083666	0.079171	0.14359
11757400_DDX42	-0.08446	0.079188	0.143615
11733462_SUSD5	-0.1658	0.079203	0.143638
11736510_IL6R	-0.1004	0.079228	0.143677
11727465_ZXDC	-0.1059	0.079254	0.143718
11716938_SEC61B	0.067953	0.079256	0.143718
11738634_LMX1A	-0.0849	0.079292	0.143777
11763914_SNHG17	-0.15096	0.079304	0.143793
11752531_MDH1B	-0.1321	0.079317	0.143812
11735497_ANKAR	-0.11074	0.079325	0.143821
11739067_MAZ	-0.1112	0.079359	0.143877
11759851_IQCE	-0.14792	0.079376	0.143903
11716119_PDIA4	-0.07909	0.079393	0.143928
11741360_RALGDS	0.181835	0.079439	0.144006
11740792_POU6F2	-0.08128	0.079472	0.144061
11736906_ROR2	-0.12696	0.079481	0.144073
11717981_ACP5	-0.08776	0.079544	0.14418
11730013_MYO3A	0.079612	0.079556	0.144195
11742370_OR52A4P	-0.15171	0.07956	0.144195
11721791_PTPRD	0.184799	0.079561	0.144195
11724683_CD300A	-0.11144	0.07958	0.144225
11758552_CORIN	0.093303	0.079586	0.14423
11758485_ANKRD13C	0.157031	0.079602	0.144255
11754884_WDFY1	-0.06654	0.079607	0.144258
11749134_POMT1	0.142685	0.079623	0.144282
11753939_PSMB10	0.086839	0.079631	0.14429
11717994_NR4A1	-0.09138	0.079641	0.144303
11748712_PPRC1	0.091709	0.07965	0.144314
11715606_TSC22D1	0.095307	0.079657	0.144315
11741103_ANXA11	0.069381	0.079658	0.144315
11731829_GPC2	-0.32832	0.079659	0.144315
11731355_IGFL3	-0.09554	0.079672	0.144333

11739581_PRKD3	0.084993	0.07968	0.144343
11756287_AOAH	-0.13083	0.079701	0.144375
11720079_NTRK2	-0.10992	0.079716	0.144392
11737389_MTMR11	-0.08681	0.079719	0.144392
11730656_LILRA3	0.088479	0.079721	0.144392
11760887_PIGL	-0.1457	0.079723	0.144392
11750834_B3GALNT1	-0.1073	0.079798	0.144524
11735259_ERLIN2	-0.23109	0.079842	0.144598
11728329_PRICKLE3	-0.08722	0.079856	0.144618
11751181_ZNF763	0.146383	0.079861	0.144621
11728679_CD163	-0.08241	0.079871	0.144634
11744593_GRASP	-0.08273	0.079887	0.144657
11715322_FABP12	0.122713	0.079913	0.144699
11750112_RNF40	0.093304	0.079921	0.144708
11738529_PCDHA8	-0.11222	0.079938	0.144734
11737293_TACR3	-0.12525	0.07995	0.144751
11719354_SH3BP5	0.100848	0.079962	0.144766
11735407_LMTK3	-0.07532	0.079966	0.144768
11724628_MAT1A	-0.11576	0.079995	0.144811
11721004_HEXIM1	0.08075	0.079995	0.144811
11754700_SH3BP1	-0.12479	0.080002	0.144814
11722909_GATS	-0.15219	0.080003	0.144814
11734996_SKI	0.080588	0.08003	0.144858
11755088_MYO9A	-0.07219	0.080051	0.14489
11761990_ADGRF5	-0.15048	0.080079	0.144936
11740431_ZFP62	-0.14907	0.080121	0.145001
11760983_L3MBTL4	0.101878	0.080121	0.145001
11716541_PRSS8	-0.11277	0.080159	0.145065
11723071_SGCE	-0.07179	0.080179	0.145096
11745352_KIF19	-0.13103	0.080185	0.1451
11732602_CEP135	-0.07827	0.080194	0.145106
11744627_RPAIN	0.080181	0.080194	0.145106
11743893_ZNF780A	0.135382	0.080207	0.145125
11740882_PRSS38	-0.07753	0.080229	0.145158
11718261_NOTCH1	-0.1121	0.080245	0.145182
11715590_DYNC1H1	0.086802	0.080276	0.145234
11727644_TRERF1	0.11674	0.080289	0.145251
11745793_PIAS3	-0.1227	0.0803	0.145265
11761006_CDK10	-0.1396	0.080328	0.145311
11746597_LOX	-0.10149	0.080352	0.145349
11725754_GRIA3	-0.17543	0.08036	0.145358
11750862_ADGRE3	0.156723	0.080372	0.145365
11738480_CRLF2	-0.1162	0.080373	0.145365
11749565_HERC2 ///	0.079066	0.080376	0.145365
11716508_CUL3	-0.22425	0.080376	0.145365
11754637_SYTL1	-0.1087	0.080392	0.145389
11730221_PNPLA2	-0.13426	0.080415	0.145425
11738778_CABYR	0.103219	0.080418	0.145425
11722771_PSD2	-0.09813	0.080439	0.145458
11727852_TEX264	0.08527	0.080477	0.145518
11747526_TSPAN6	0.09667	0.080478	0.145518

11741072_PRDM8	-0.08052	0.080494	0.145541
11738583_OR8G2 ///	0.101679	0.080518	0.145579
11756689_HN1L	-0.13923	0.080528	0.145592
11752264_TRIM3	0.093373	0.080537	0.1456
11730247_PVRIG ///	-0.19041	0.080538	0.1456
11720275_CFLAR	0.076593	0.080554	0.145623
11738276_SPDYE3	-0.11577	0.080571	0.145647
11715477_TFRC	-0.07822	0.080591	0.145679
11752453_GPD2	-0.07901	0.080599	0.145688
11741596_SMR3A	-0.08104	0.080606	0.145696
11762030_AVL9	0.09537	0.080624	0.145713
11762762_LOC100288	-0.18758	0.080624	0.145713
11727832_DDX42	-0.07795	0.080624	0.145713
11736359_LGI2	-0.10758	0.080634	0.145724
11734139_TBC1D10C	-0.16844	0.08064	0.145729
11723922_IPO13	0.116757	0.080656	0.145754
11763340_LOC642852	0.16602	0.080661	0.145758
11748215_C8orf44	-0.13967	0.080675	0.145777
11735205_MMAA	0.097254	0.080686	0.145792
11755936_ENOSF1	-0.08305	0.08072	0.145845
11716826_FMOD	0.150673	0.080722	0.145845
11725022_VILL	-0.10557	0.080732	0.145859
11758225_IQGAP2	0.153755	0.080738	0.145864
11732121_PMPCB	0.070584	0.08078	0.145929
11716269_KRTCAP2	-0.07002	0.08078	0.145929
11761151_GGA3	-0.1359	0.080802	0.145963
11719855_FAAH	-0.12872	0.080807	0.145966
11748870_CLN3	-0.08298	0.080815	0.145977
11740335_GPR173	-0.15446	0.08084	0.146015
11721500_SWAP70	0.147574	0.080847	0.146023
11724623_CLEC1A	-0.14759	0.080861	0.146037
11729560_FOXL1	-0.11451	0.080861	0.146037
11763566_WDR48	-0.20527	0.080863	0.146037
11761618_SGCA	-0.18455	0.080873	0.146048
11742660_URAD	-0.07709	0.080888	0.146071
11757046_SLCO4A1-A	-0.07895	0.080907	0.146099
11736926_TMSB4Y	-0.1097	0.080913	0.146103
11745362_RPS11	0.073348	0.080915	0.146103
11731458_PRH2	-0.14084	0.080948	0.146154
11762881_AGK	0.110357	0.08095	0.146154
11724701_AS3MT ///	-0.24553	0.081015	0.146266
11759898_ECM2	-0.22879	0.08103	0.146289
11733170_FBXO45	0.070852	0.081038	0.146298
11722458_TMEM206	0.089722	0.08106	0.14633
11744831_RPAIN	0.084382	0.081062	0.14633
11734185_ZNF677	-0.08373	0.081075	0.146345
11744644_HSFX1 ///	-0.06605	0.081076	0.146345
11751452_MYH2	0.104429	0.081083	0.146352
11732816_CPN2	-0.10748	0.081099	0.146375
11733980_CD28	-0.10601	0.081135	0.146435
11715391_MTCH1	-0.0689	0.08114	0.146438

11742263_SPAG8	-0.15244	0.081151	0.146453
11733436_SLC7A10	0.085505	0.08117	0.146482
11731424_CXCR2	0.163596	0.081204	0.146536
11725490_ATG14	-0.12036	0.081206	0.146536
11729661_GLB1	-0.07643	0.08122	0.146556
11719790_MYL3	-0.12006	0.081229	0.146566
11762105_FGFR2	-0.09872	0.081302	0.146688
11721106_RAB3GAP1	0.090987	0.081303	0.146688
11741742_LEUTX	-0.11826	0.08131	0.146688
11752997_OPRM1	-0.15241	0.08131	0.146688
11750786_DGCR2	-0.07687	0.081311	0.146688
11739018_USP22	0.103694	0.081351	0.146754
11739055_PTPN6	-0.11817	0.081361	0.146768
11747787_HTRA1	-0.08389	0.081368	0.146773
11758229_GREM1	0.130833	0.081376	0.146784
11736197_DCX	-0.11941	0.081402	0.146825
11731042_FBXO24	-0.16777	0.081413	0.146839
11716242_PES1	0.082665	0.081426	0.146857
11730710_TERT	-0.11607	0.081435	0.146865
11757890_MTERF2	-0.09223	0.081437	0.146865
11718717_TOM1L2	-0.15881	0.081456	0.146894
11738326_TAS2R19	-0.17904	0.081471	0.146917
11738072_LRCH3	0.074858	0.081479	0.146925
11751498_SLC10A7	0.083112	0.081517	0.146988
11728317_FAM122A	0.109405	0.081548	0.147039
11730408_C19orf33	0.082826	0.081578	0.147088
11725032_TPP1	0.092083	0.081607	0.147134
11715709_MB	-0.1485	0.081611	0.147137
11725207_LETM1	0.095719	0.081617	0.147142
11736909_C1orf210	-0.12518	0.081667	0.147227
11751740_ACER3	0.125748	0.081674	0.147234
11722758_NPR3	-0.11899	0.081708	0.14729
11750425_CCDC17	-0.18417	0.081715	0.147297
11762960_C3orf67	-0.20377	0.08178	0.147408
11748497_GCA	-0.15065	0.081795	0.14743
11721987_PRICKLE2	0.129838	0.081826	0.147481
11738675_OR5AU1	-0.12537	0.081834	0.147489
11742008_SPAG11A /	0.215182	0.081842	0.147498
11726073_ARHGEF7	-0.11229	0.081849	0.147505
11722131_PLCB2	-0.09345	0.081868	0.147534
11731431_PCDH10	0.164519	0.081883	0.147554
11715230_PARD6A	-0.08412	0.081885	0.147554
11718650_SYNE2	-0.14564	0.081892	0.147561
11740900_C4orf45	0.078895	0.081901	0.147568
11743409_RBM25	0.09083	0.081902	0.147568
11761168_SLC35A2	-0.11948	0.081905	0.147568
11759624_PWAR6	-0.09348	0.081943	0.147631
11757887_YWHAB	-0.06918	0.081946	0.147632
11742430_N6AMT1	0.173008	0.081963	0.147655
11740084_LGALS9B /	-0.11382	0.081965	0.147655
11727727_DMRT2	-0.08498	0.081982	0.14768

11736213_MRPL57	0.111791	0.081998	0.147698
11756448_EBNA1BP2	0.080854	0.081998	0.147698
11748997_MYBPC1	-0.1466	0.082016	0.147725
11737186_DUOX1	-0.1231	0.082055	0.14779
11728930_RNASE11	-0.15558	0.082063	0.147799
11722615_HCAR2 ///	-0.10047	0.08207	0.147806
11715928_SH3GL1	-0.09005	0.082088	0.147832
11719850_RNF139	-0.12996	0.08209	0.147832
11761588_SORD	0.134418	0.08211	0.14786
11743474_SMIM4	-0.06919	0.082115	0.14786
11720684_B3GALT6	0.080257	0.082115	0.14786
11727745_METTL21C	0.161346	0.082136	0.147893
11764082_TNPO1	-0.17252	0.082157	0.147924
11733420_ZNF563	-0.14898	0.082161	0.147927
11740955_PLEKHN1	-0.17384	0.082186	0.147958
11762854_EIF4A1	0.078089	0.082188	0.147958
11717597_GNAS	-0.0663	0.082188	0.147958
11739010_MYH9	-0.13695	0.082191	0.147958
11726176_C17orf58	-0.07569	0.082202	0.147972
11745380_SLC25A27	0.132832	0.082206	0.147975
11722498_RPTOR	-0.08396	0.082227	0.148007
11718108_DLD	0.066572	0.082232	0.148009
11745482_PRNP	0.08028	0.082245	0.148028
11754325_GPATCH8	-0.08136	0.082249	0.148029
11733773_OSTN	-0.08596	0.082266	0.148049
11749717_RP1	-0.14206	0.082266	0.148049
11716118_PDIA4	-0.0699	0.082269	0.148049
11731230_RPUSD3	-0.08927	0.082324	0.148143
11734624_TAC4	-0.19449	0.082328	0.148145
11761848_SNRPD3	-0.12272	0.082343	0.148167
11742983_GALNT9	-0.071	0.082355	0.148182
11726612_SLC22A6	-0.09428	0.082376	0.14821
11723155_CAMTA1	0.135244	0.082379	0.14821
11720078_NTRK2	-0.11404	0.082381	0.14821
11730812_LRRTM2	-0.11801	0.082383	0.14821
11728095_DSCAM	-0.16741	0.082393	0.148225
11736563_ZNF518A	-0.22093	0.082427	0.148279
11735821_AQP12A //	-0.12081	0.082436	0.14829
11741695_GEMIN2	0.087296	0.082451	0.148312
11730557_DMRTC2	0.128726	0.082486	0.148366
11753722_PFN1	-0.07125	0.082488	0.148366
11734154_KBTBD3	0.10712	0.082493	0.148366
11735162_OSBPL5	-0.11267	0.082493	0.148366
11758067_PPIA	-0.06957	0.082515	0.148397
11729338_PSTPIP2	-0.10303	0.082517	0.148397
11755906_FAM135A	0.158006	0.082522	0.1484
11753885_MAPK14	-0.09828	0.082525	0.1484
11736896_PRRG4	0.147601	0.082544	0.14843
11742252_SLC22A24	-0.14859	0.082551	0.148437
11760429_CHCHD3	-0.16313	0.082563	0.148452
11748743_ADD2	-0.07414	0.082583	0.148484

11749509_CREB3L1	-0.19972	0.082587	0.148484
11726893_ST7 /// S	0.068026	0.082589	0.148484
11755526_NDUFC2	-0.12474	0.082596	0.14849
11724480_NREP	-0.102	0.082598	0.14849
11719452_SLC30A9	0.071923	0.082603	0.148492
11742850_HLA-DRA	-0.07604	0.082615	0.148509
11751024_XYLB	0.098265	0.082637	0.148542
11719780_TNFAIP8L2	-0.10405	0.082642	0.148545
11729829_FAM110B	-0.11589	0.082653	0.14856
11752294_USP6	0.143437	0.082668	0.148581
11760064_HIC1	-0.11383	0.082674	0.148586
11722892_SLC17A7	-0.13231	0.082698	0.148624
11763517_FILIP1L	-0.13059	0.082701	0.148624
11737583_SGCD	0.102824	0.08271	0.148636
11747685_ARFIP2	0.074272	0.082751	0.148704
11755861_LPIN3	-0.13784	0.082758	0.148711
11748373_SFTPA1 //	0.131775	0.082788	0.148759
11747966_SKIDA1	-0.07329	0.082864	0.148889
11756720_PCSK1	-0.11729	0.082882	0.148918
11734132_SLC2A4	-0.1789	0.082897	0.148935
11748874_MRPS25	-0.12271	0.082898	0.148935
11728035_RS1	-0.1817	0.082933	0.148993
11748820_CMAS	0.086186	0.082944	0.148997
11748319_TP53	-0.09647	0.082944	0.148997
11729848_BIRC7	-0.08376	0.082945	0.148997
11736943_WFIKKN1	-0.125	0.082961	0.149016
11741014_DNM2	-0.08675	0.082962	0.149016
11738064_MGAT5	0.162637	0.082973	0.149031
11730669_OPHN1	0.156394	0.082991	0.149058
11742633_OR4K13	-0.14129	0.083011	0.149089
11758265_KIAA1468	-0.0813	0.083016	0.149092
11762299_COMMD4	-0.17342	0.083021	0.149095
11716035_BST2	-0.1045	0.08303	0.149106
11742926_CPT2	0.08731	0.083034	0.149108
11759423_EPPK1	0.250299	0.083045	0.149123
11737785_KCNAB2	-0.13434	0.083062	0.149147
11738773_STK32A	-0.14115	0.083076	0.149167
11723058_TAP2	0.068157	0.083083	0.149174
11741270_IL12RB1	-0.11438	0.083091	0.14918
11760976_APBB1	-0.09409	0.083093	0.14918
11760541_SRI	0.130423	0.0831	0.149188
11748583_ART3	-0.0847	0.083107	0.149194
11746130_EIF3H	0.076556	0.08315	0.149261
11726549_SMAD3	-0.08916	0.083151	0.149261
11742276_EPM2A	-0.17908	0.083153	0.149261
11719165_DAB2IP	0.094984	0.08319	0.149322
11754885_PLEKHH3	-0.07587	0.083197	0.149328
11715212_SCAND1	0.085449	0.083199	0.149328
11753703_TMEM44	-0.15709	0.083205	0.149332
11718156_THTPA	-0.08831	0.083217	0.149348
11725567_ATP8A1	-0.13946	0.083239	0.149382

11742878_SLC52A2	-0.07361	0.083245	0.149389
11744960_ZNF311	0.138391	0.083334	0.149543
11755432_AGO1	0.095767	0.083354	0.149572
11737021_GRIN2B	-0.15443	0.083357	0.149573
11750295_CLEC11A	-0.09451	0.083389	0.149624
11730195_SEC14L2	0.19935	0.083414	0.149663
11754770_ZNF646	0.08977	0.083419	0.149668
11720250_RWDD1	0.072071	0.083462	0.149739
11742519_OR11G2	-0.14499	0.083484	0.149772
11737840_CYP2W1	-0.17318	0.083502	0.149799
11737800_ECE1	-0.09592	0.083514	0.149816
11727317_KCNE5	-0.20156	0.083529	0.149835
11727733_CCDC40	0.201604	0.083531	0.149835
11729120_HSPA1B	-0.09243	0.083541	0.149848
11743304_EYA3	0.113323	0.083555	0.149867
11719449_RAB14	-0.14094	0.083558	0.149867
11720584_TCF12	0.084129	0.083569	0.149881
11753307_UPK1A	-0.10491	0.08358	0.149896
11754879_SDR16C5	-0.12125	0.083588	0.149904
11755313_ZNF559	0.121032	0.083592	0.149906
11731748_HEPACAM	-0.07888	0.083596	0.149907
11746987_ADAM2	0.125816	0.083613	0.149933
11716614_TSPO	0.079909	0.083622	0.149943
11728418_ACSBG2	-0.09035	0.083641	0.149972
11715264_UTS2R	-0.13063	0.083647	0.149977
11724166_COLGALT2	-0.08743	0.083661	0.149995
11744117_THSD7A	0.153071	0.083663	0.149995
11718743_CXCL12	-0.12528	0.083687	0.150032
11719935_EDC3	0.071908	0.083697	0.150037
11754463_SNRNP70	-0.07436	0.083701	0.150037
11755283_SNRNP70	-0.07436	0.083701	0.150037
11729123_RCN2	-0.09508	0.083702	0.150037
11761895_LOC100130	0.098167	0.083717	0.150058
11743721_LGALS1	0.066923	0.083729	0.150076
11744319_PIGZ	-0.0855	0.083735	0.15008
11744305_LRRK1	-0.11125	0.08375	0.150096
11721792_RGMB	-0.09289	0.08375	0.150096
11734442_FOXI1	-0.10853	0.083755	0.1501
11725044_YWHAB	0.070463	0.083762	0.150106
11737510_ZNF695	0.230573	0.083795	0.15016
11741985_KCNC4	-0.08992	0.083803	0.150169
11743548_SLC22A23	0.13438	0.083836	0.150223
11729865_SPATA2L	0.092701	0.08385	0.150242
11754860_ZKSCAN1	-0.07857	0.083874	0.150279
11732152_AGBL5	0.128239	0.083879	0.150284
11756376_TTC19	0.076681	0.083887	0.150289
11753296_KIR2DL4	-0.14807	0.08389	0.150289
11730795_MRPL4	-0.09224	0.083892	0.150289
11750238_SARM1	-0.10094	0.08392	0.150332
11742831_BRD4	-0.08551	0.083922	0.150332
11725063_CNOT10	0.08723	0.083945	0.150369

11725817_CABP1	-0.17525	0.083954	0.150378
11752671_NARFL	-0.09568	0.083963	0.15039
11756776_GCGR	-0.08253	0.083981	0.150416
11754408_LDB2	0.145236	0.08399	0.150426
11716255_ECE1	-0.18203	0.083998	0.150435
11760654_LRP3	-0.1105	0.084001	0.150436
11734808_DTNA	0.201484	0.084029	0.15048
11743222_AWAT1	-0.07541	0.084048	0.150509
11742196_VPS41	0.13865	0.084059	0.150523
11759504_MAP7D3	-0.13806	0.084072	0.15054
11755447_OTOP3	-0.31774	0.084091	0.150568
11736782_RAB11FIP1	0.116245	0.084109	0.150596
11744055_METTL1	0.086427	0.084117	0.150602
11740071_PSMF1	0.074811	0.084119	0.150602
11736815_GIPC2	0.10689	0.084125	0.150607
11734169_CD300E	-0.16096	0.084141	0.150631
11741944_AADACL3	-0.13451	0.084149	0.150639
11746287_LGMN	-0.08912	0.084154	0.150644
11717514_ANXA1	0.075658	0.084163	0.150654
11726106_LRFN3	-0.08313	0.084167	0.150655
11736095_GATC	0.093259	0.084175	0.150664
11725930_HSPA5	-0.0809	0.084192	0.15069
11747246_MAPT	-0.09664	0.084217	0.150724
11751953_PAPLN	0.134846	0.084218	0.150724
11723278_CREBZF	-0.09461	0.084224	0.150729
11751514_ZNF772	0.099669	0.084237	0.150747
11762792_POLR3K	-0.07922	0.084273	0.150806
11738704_OR52B2	-0.21764	0.084298	0.150845
11762033_TTLL5	0.172542	0.084312	0.150865
11729714_IRX2	-0.18348	0.084326	0.150885
11751673_ZMIZ2	0.106096	0.084337	0.1509
11741697_ITPA	0.071395	0.084368	0.150948
11751035_EPHB2	-0.11252	0.084395	0.150987
11720645_SPTLC2	-0.08282	0.084395	0.150987
11718438_NSUN4	-0.08278	0.084405	0.150993
11762496_BAIAP2	-0.09876	0.084405	0.150993
11746932_ASPHD1	0.096594	0.084422	0.151013
11716558_TNS1	-0.12829	0.084428	0.151013
11741971_KRT38	-0.16108	0.084428	0.151013
11756811_TOM1	0.117726	0.084429	0.151013
11729009_MYLK4	-0.1726	0.084433	0.151016
11753430_WBP2	-0.08659	0.084479	0.151092
11723638_C11orf58	0.114525	0.084513	0.151145
11727770_NIN	-0.1107	0.084515	0.151145
11720807_EIF1AY	0.089198	0.084543	0.151189
11724377_PAG1	-0.2534	0.084546	0.151189
11726215_DENND1A	0.109446	0.084555	0.1512
11759083_RAB11FIP3	-0.10559	0.084568	0.151217
11759732_LARP1B	-0.09104	0.084573	0.151222
11750022_LCORL	0.081449	0.084584	0.151236
11748806_ACTR2	-0.06654	0.084588	0.151238

11758182_FTL	0.071688	0.084629	0.151305
11727517_TMEM79	0.106493	0.084654	0.151345
11755927_CACNA1C	-0.12389	0.084702	0.151424
11762571_GNPDA2	0.089878	0.084721	0.151453
11725476_ACAP1	0.100228	0.084734	0.151471
11743853_FBXO21	-0.09895	0.084742	0.151478
11752287_PRPF4B	0.089363	0.084755	0.151497
11736622_C1orf106	-0.09117	0.084799	0.151571
11751594_SRSF10	-0.12358	0.084828	0.151616
11724879_ZNF217	-0.08874	0.08486	0.151668
11729478_LY6G6C	-0.12428	0.084888	0.151712
11741144_CD151	0.074245	0.084899	0.151727
11725553_UGT2A3	-0.08971	0.08491	0.151741
11728925_NFAM1	-0.20247	0.084985	0.151862
11719107_REXO4	-0.09162	0.084987	0.151862
11720026_VGF	0.065984	0.084987	0.151862
11761743_UCK2	-0.1334	0.084995	0.151871
11738728_OR5B2	-0.08793	0.085002	0.151877
11741920_GRXCR2	-0.11111	0.085006	0.151879
11721434_CRBN	0.097381	0.085019	0.151891
11721037_SUFU	-0.12656	0.085019	0.151891
11723813_ARHGAP29	-0.09446	0.085052	0.151944
11758983_SAP30	-0.06697	0.085056	0.151947
11735533_HIVEP3	-0.0882	0.085073	0.151972
11760460_RBM48	-0.08574	0.085085	0.151987
11760800_SPHK2	-0.11162	0.085092	0.151994
11744710_TTLL4	-0.0732	0.085111	0.152021
11741957_LRRIQ1	0.13463	0.08513	0.152046
11720623_MYD88	0.108456	0.085131	0.152046
11747919_YTHDF2	0.084144	0.085142	0.152061
11744347_MYO10	0.067588	0.085153	0.15207
11742406_CSH1 ///	-0.20687	0.085156	0.15207
11736686_FGF5	0.098202	0.085156	0.15207
11755223_KDM7A	-0.12359	0.085172	0.152093
11734650_VHLL	-0.09822	0.085176	0.152094
11760199_ITIH4	-0.08623	0.085183	0.152101
11731940_UCP3	0.134379	0.085198	0.152118
11723106_NCF4	0.164884	0.0852	0.152118
11750300_ZNF277	0.114659	0.085205	0.152118
11735346_C1QTNF5 /	-0.13985	0.085205	0.152118
11719041_CYGB	-0.10147	0.085218	0.152136
11754442_ELN	-0.10347	0.085249	0.152185
11759879_NELL1	-0.14793	0.085252	0.152185
11718800_PTPRU	-0.09732	0.085263	0.152198
11731397_YWHAB	0.092351	0.085274	0.15221
11752687_ASB15	-0.1987	0.085275	0.15221
11759090_ZNF571	-0.09774	0.085279	0.15221
11728344_SYNPO	-0.16031	0.085319	0.152277
11728706_EMP2	0.086676	0.085327	0.152285
11734455_WFDC3	0.083553	0.08533	0.152285
11739099_CSTF1	-0.12708	0.08534	0.152293

11722876_ARHGEF15	-0.06534	0.085341	0.152293
11748329_PMM2	0.138268	0.085363	0.152328
11746963_DNAJB2	-0.13513	0.085386	0.152358
11761090_HEXDC	-0.137	0.085386	0.152358
11746182_TOE1	-0.07234	0.085391	0.152361
11752695_SASH3	-0.11411	0.085416	0.152396
11742814_SEMA6A	-0.1824	0.085417	0.152396
11758805_NUDCD3	-0.16471	0.08542	0.152396
11725369_SPRR1B	-0.11179	0.085444	0.152433
11718104_RELA	0.136556	0.085462	0.15246
11734407_MATN4	-0.10294	0.085474	0.152476
11739651_HNRNPLL	0.104774	0.085506	0.152522
11760153_LOC730098	-0.12649	0.085507	0.152522
11744181_FARS2	0.118794	0.085513	0.152528
11749539_MICB	-0.09135	0.085567	0.152619
11721087_YLPM1	-0.1898	0.0856	0.152673
11730902_JDP2	-0.14851	0.085639	0.152736
11728846_HAND1	-0.12007	0.085661	0.15277
11747354_IQGAP2	0.163086	0.085701	0.152837
11734116_LLGL2	-0.12354	0.08571	0.152847
11718646_OSBPL8	-0.06492	0.085716	0.152851
11727571_MARK1	-0.09835	0.085743	0.152891
11763026_MUC1	-0.10792	0.085745	0.152891
11746659_NR2F6	-0.08255	0.08576	0.152913
11759437_EIF3F	0.081397	0.085853	0.153073
11744526_INPP5F	0.092891	0.085874	0.153101
11760423_TACC3	-0.18059	0.085882	0.153101
11754781_HDAC2	-0.07366	0.085882	0.153101
11732702_CITED1	-0.13324	0.085883	0.153101
11722948_CDRT4 ///	-0.13939	0.085884	0.153101
11736871_TRPC6	-0.1381	0.085922	0.153162
11724394_KIAA1211L	-0.08548	0.085927	0.153167
11763857_COPE ///	0.091709	0.085944	0.153191
11745902_NPC2	-0.0835	0.085977	0.153244
11732049_PRR20A //	-0.19334	0.08598	0.153244
11761968_KIAA2012	-0.11255	0.086009	0.153291
11749676_BCL11A	-0.08147	0.086024	0.153311
11751079_ADGRG5	-0.08837	0.086029	0.153314
11750534_CRYZL1	0.158572	0.086036	0.153319
11762754_CCT8	-0.16363	0.086038	0.153319
11735212_STYK1	0.149187	0.08606	0.153353
11759582_AKAP17A	-0.08538	0.086065	0.153356
11719159_RAPH1	0.296352	0.086075	0.153363
11732830_ETV2	0.142989	0.086075	0.153363
11734908_CADPS2	-0.14588	0.086079	0.153364
11760841_ZNF99	0.158836	0.08609	0.153378
11739811_SIRT3	-0.1301	0.086094	0.15338
11716569_NOTCH3	0.114145	0.086106	0.153396
11723348_GAPVD1	0.068	0.086128	0.153429
11717941_U2AF2	-0.1905	0.086135	0.153436
11742936_TSEN2	0.083986	0.086154	0.153464

11732365_NT5C3A	0.090014	0.086162	0.153473
11742592_OR2M5	-0.11638	0.086182	0.153503
11749151_FAM129C	0.157976	0.08619	0.153512
11748029_TFE3	-0.06961	0.086205	0.153532
11717507_TRAM2	-0.06968	0.086213	0.153542
11753553_DNAJC27	0.122484	0.086247	0.153597
11760742_SYT8	-0.13415	0.086251	0.153598
11732914_MEIG1	0.153237	0.086258	0.1536
11720701_C1orf101	-0.14281	0.086258	0.1536
11750962_JPH2	-0.07505	0.086262	0.153601
11740971_RINL	0.076779	0.086271	0.153611
11751456_REEP1	-0.08318	0.08629	0.153638
11725277_TRIM6	-0.09872	0.086292	0.153638
11762212_TRAIP	0.15302	0.086308	0.153661
11757704_WDR13	-0.06934	0.086323	0.153681
11738065_PCDH10	-0.13535	0.086335	0.153698
11715368_SERPING1	-0.17213	0.086377	0.153762
11734711_GPR37L1	-0.07067	0.086378	0.153762
11754180_SPRR1A	-0.12847	0.086396	0.15379
11723746_SLC13A5	-0.09242	0.086415	0.153817
11739778_RAD17	-0.08585	0.086424	0.153827
11716552_LRRC49	0.101591	0.08643	0.153833
11732254_MED18	0.065031	0.086439	0.153838
11716755_RNF7	-0.07346	0.086439	0.153838
11762775_ACTR2	0.264044	0.086443	0.153839
11756805_EHMT1	0.065804	0.086447	0.15384
11736724_PDLIM7	-0.10871	0.08646	0.153854
11743495_SV2B	-0.108	0.086461	0.153854
11719529_KLF16	-0.0869	0.086482	0.153883
11752949_COX6B2	-0.15229	0.086483	0.153883
11737032_FBXL13	-0.10677	0.086507	0.15392
11725979_CALB1	0.246241	0.086518	0.153933
11741022_SEPT7	0.109181	0.086534	0.153956
11755637_ARHGEF10L	0.086652	0.086539	0.15396
11731357_ADAMTS3	-0.11285	0.08655	0.153973
11715481_SST	-0.09169	0.086557	0.15398
11759121_SLC5A8	0.140342	0.086579	0.154009
11721930_TMCC2	-0.14241	0.086579	0.154009
11753762_KLK6	-0.12205	0.086634	0.1541
11746716_KIRREL3	0.130603	0.086644	0.154112
11731248_TMEM116	-0.12761	0.086666	0.154142
11728058_SERTAD3	0.130988	0.086667	0.154142
11755838_PCDHB13	0.072763	0.086709	0.15421
11737150_IL26	-0.1241	0.086711	0.15421
11726980_PAQR6	0.098159	0.086742	0.154258
11728949_LSMEM2	-0.09699	0.086769	0.154301
11758165_MRTO4	0.072295	0.086775	0.154305
11726761_RCE1	0.101865	0.086809	0.15436
11753525_MED25	-0.07917	0.086815	0.154366
11757318_TRIP10	-0.06607	0.086821	0.154366
11716424_PCDHGA1 /	-0.07609	0.086821	0.154366

11762835_DNASE1	0.081203	0.086859	0.154428
11737141_RSPH1	-0.12239	0.08689	0.154477
11727896_LSM8 ///	-0.12777	0.086922	0.154528
11738581_OR8D1	-0.07514	0.086954	0.154579
11754730_EHMT1	-0.10342	0.086976	0.154606
11731735_VCX2	0.07538	0.086978	0.154606
11753638_VCX2	0.07538	0.086978	0.154606
11764256_ND4	0.064721	0.086989	0.154618
11734565_C1QTNF3	-0.12555	0.086991	0.154618
11742851_SPINT2	0.08972	0.087009	0.154643
11753607_REG3G	-0.18342	0.087012	0.154644
11716253_RBFOX2	0.096535	0.087017	0.154646
11762799_FGFR4	-0.09307	0.087044	0.154689
11762146_CGREF1	0.080934	0.087052	0.154694
11734428_MAGEA11	-0.07934	0.087054	0.154694
11720296_PLEKHF2	-0.09715	0.087056	0.154694
11725175_ADAP2	-0.1628	0.087171	0.154892
11721810_PIM2	-0.07803	0.087201	0.154939
11748625_HYOU1	0.077184	0.087209	0.154948
11716772_C11orf68	0.078288	0.087232	0.154983
11722891_SLC17A7	-0.16567	0.087258	0.155024
11753350_KRR1	0.083284	0.087276	0.155049
11751017_EARS2	0.069216	0.087278	0.155049
11750780_LOC51145	-0.13992	0.087294	0.155071
11731423_CXCR2	0.173144	0.087303	0.155079
11731384_DFFB	-0.07607	0.087305	0.155079
11732197_PLAGL2	0.083149	0.087323	0.155106
11758197_APOA1	-0.17999	0.087331	0.155114
11734396_HRASLS2	-0.08988	0.087341	0.155127
11748246_CUL2	-0.08032	0.08735	0.155136
11749484_AXDND1	-0.23721	0.087353	0.155136
11750752_RANBP3L	-0.08733	0.087367	0.155156
11759816_IGHM	-0.10951	0.087419	0.15524
11725118_BORCS5	-0.12742	0.087421	0.15524
11739723_ATP2B2	-0.17674	0.087443	0.155275
11748852_HMP19	-0.23684	0.087463	0.155303
11738116_GABRQ	-0.13633	0.087491	0.155348
11743159_FGG	-0.12381	0.087501	0.15536
11731205_SPATA33	0.102741	0.087505	0.155362
11741310_ZNF772	0.164101	0.087526	0.155392
11749727_RNGTT	0.1212	0.087539	0.155411
11762599_PPFIBP2	-0.21606	0.087597	0.155507
11733602_DNAJC5G	-0.09857	0.087666	0.155625
11728414_PDIA2	-0.10935	0.087686	0.155655
11747549_PICALM	0.077208	0.087709	0.15569
11740092_MSMO1	0.090829	0.087715	0.155694
11757648_MLLT4	-0.12431	0.087767	0.155776
11724045_USP2	-0.07281	0.087767	0.155776
11749759_SCN3A	0.190874	0.087797	0.155824
11715934_TAGLN	0.084778	0.087802	0.155826
11756795_ADCY2	-0.11322	0.08781	0.155835

11761104_C12orf10	-0.15326	0.087814	0.155836
11724592_TCP10L	-0.07501	0.087837	0.155872
11755711_ANKRD30BF	-0.17946	0.087849	0.155887
11745370_EBF2	-0.23617	0.087854	0.15589
11734896_MATN1	-0.10016	0.087858	0.155893
11761894_LOC100130	0.095165	0.087867	0.155903
11740847_ZNF229	-0.19122	0.08788	0.155919
11751440_MTUS2	-0.16888	0.087884	0.15592
11750744_TMC8	-0.12581	0.087886	0.15592
11719989_SNX19	-0.08651	0.087898	0.155934
11729455_CYP2B6	-0.08309	0.087905	0.155942
11761385_ARGLU1	0.094937	0.087955	0.156021
11746848_ZNF532	0.071656	0.087957	0.156021
11727663_STOX1	-0.10534	0.087992	0.156078
11758291_MRPS10	0.084659	0.087995	0.156078
11724110_ARHGAP4	-0.07743	0.087999	0.15608
11730489_DOK6	-0.16415	0.088014	0.1561
11721525_BOLA2 ///	0.104728	0.088026	0.156112
11740815_CNGA1	0.090811	0.088027	0.156112
11720600_CAPZB	0.079503	0.088036	0.156123
11737410_C4orf36	-0.11367	0.088041	0.156126
11739671_GRAP ///	-0.1397	0.088068	0.156168
11748440_LOC100287	0.118089	0.088082	0.156186
11749844_CSF2RA	-0.09177	0.088092	0.1562
11734861_KCNH8	0.203597	0.088097	0.156203
11762969_MEI1	-0.08919	0.088109	0.156218
11746359_NUCB2	-0.07792	0.088118	0.156228
11724957_ENOX1	0.169822	0.088183	0.156333
11729896_C11orf87	-0.15339	0.088183	0.156333
11735762_RGS12	-0.08686	0.088186	0.156333
11722559_PSG1	0.101847	0.088209	0.156363
11748847_GOLGA2P5	-0.18939	0.088212	0.156363
11728398_MT1M	-0.07573	0.088213	0.156363
11761887_C21orf58	-0.18468	0.088218	0.156367
11734682_PSMA7	-0.06575	0.088244	0.156407
11723866_CHODL	0.152308	0.088254	0.156419
11737557_AMTN	-0.12026	0.088262	0.156426
11734891_SOBP	-0.1602	0.088277	0.156448
11745430_PODNL1	-0.09365	0.088288	0.156462
11735465_ZNF326	-0.10749	0.088295	0.156468
11735474_CD8B	0.079356	0.088305	0.156481
11757209_ASMTL-AS1	-0.13357	0.088346	0.156547
11736549_RAB29	0.130655	0.088349	0.156548
11742794_SBDS ///	0.090422	0.088372	0.156582
11759464_OTOP2	-0.08233	0.088384	0.156598
11736179_RBBP5	0.070926	0.088464	0.156733
11722738_ICK	0.202492	0.088472	0.156742
11760023_VPS29	0.093139	0.088497	0.156775
11760024_VPS29	0.093139	0.088497	0.156775
11751379_USP17L1 /	-0.13593	0.088513	0.156797
11752796_C3orf33	0.152479	0.088574	0.156897

11733617_RXRG	0.155232	0.088578	0.156897
11725980_CFD	-0.13035	0.08858	0.156897
11758495_C1GALT1C1	-0.10116	0.088582	0.156897
11734110_TIGIT	-0.10185	0.088608	0.156938
11730295_SLC25A40	0.090731	0.088633	0.156976
11737383_IFITM5	-0.09454	0.088666	0.157029
11744913_NFATC4	-0.14721	0.088684	0.15705
11762196_TCEA3	0.07312	0.088684	0.15705
11751231_HAL	-0.20134	0.088695	0.157064
11741516_SELV	-0.20608	0.088706	0.157078
11716857_SUPT7L	-0.0874	0.088727	0.157109
11715143_OR9Q2	-0.13673	0.088734	0.157115
11738584_KRTAP9-3	-0.14511	0.088737	0.157115
11740811_TPSG1	-0.11742	0.088761	0.157152
11740218_SIRPB1	-0.13678	0.088766	0.157155
11735437_NRXN2	0.13681	0.088772	0.157159
11752188_GIGYF2	-0.08253	0.088786	0.157179
11741860_CAV3	-0.13848	0.088823	0.157234
11727808_FECH	-0.06547	0.088823	0.157234
11752947_CHRNG	-0.09438	0.088827	0.157234
11718897_SPAG9	-0.09115	0.088857	0.157282
11759679_TRPM1	-0.14436	0.088874	0.157306
11757189_TMEM254-A	-0.11406	0.088888	0.157326
11730522_SLITRK2	0.160743	0.088901	0.157343
11732385_RELT	0.111571	0.088906	0.157345
11736541_NRXN1	0.150676	0.088915	0.157356
11727349_TBC1D21	-0.08612	0.088929	0.157375
11735421_NKD2	-0.11321	0.088958	0.15742
11756561_MED20	-0.07029	0.088964	0.157425
11759473_TAS2R30 /	0.17105	0.088972	0.157434
11731348_FAM71F2	0.112005	0.088984	0.157449
11755963_TTLL13P	-0.22237	0.088997	0.157464
11717964_KRAS	0.079081	0.089002	0.157464
11753551_KDM8	0.086535	0.089005	0.157464
11733395_PPFIA1	0.092954	0.089005	0.157464
11733373_JAK2	0.104182	0.089022	0.157488
11737581_CAPN9	-0.08296	0.089026	0.15749
11740225_CHST4	-0.12055	0.089037	0.157504
11764066_EPB41L5	0.322797	0.089059	0.157537
11728830_RAB3IP	0.124177	0.089084	0.157576
11721503_A4GALT	-0.18574	0.089096	0.157591
11724803_TMEM55B	0.077776	0.089123	0.157631
11724254_B3GNT3	-0.17363	0.089125	0.157631
11741763_C1orf229	0.209715	0.089129	0.157633
11727169_PDLIM7	-0.09868	0.089135	0.157638
11742959_SMARCA4	0.065908	0.089153	0.157663
11754344_SPRY4	0.083146	0.089192	0.157725
11763881_FAM24B	-0.10356	0.089208	0.157744
11742551_OR2W1	0.09746	0.089208	0.157744
11724858_CEP63	-0.09788	0.089239	0.157793
11756841_SLC25A33	-0.0705	0.089248	0.157803

11755130_CLIP3	0.081617	0.08927	0.157836
11738888_MICA	0.094583	0.08928	0.157848
11760059_ZNF445	-0.12319	0.089284	0.157849
11761705_HMGA2	-0.07241	0.089323	0.157911
11717701_DCAF8	0.070978	0.089328	0.157915
11763505_NF1	0.141401	0.08935	0.157949
11740538_SCGB1C1 /	-0.09123	0.089354	0.15795
11721928_TMCC2	-0.09828	0.089362	0.157958
11731295_SPATA6L	0.170938	0.089373	0.157973
11726974_PERP	-0.08487	0.089382	0.157983
11715229_GRIP2	-0.08352	0.089393	0.157991
11742135_ACBD4	-0.22219	0.089394	0.157991
11735446_TVP23A	-0.22435	0.089404	0.157993
11729369_GANC	0.156076	0.089404	0.157993
11754634_SPATA31D4	-0.10573	0.089404	0.157993
11746274_SLC25A44	0.161088	0.089408	0.157994
11719475_BEX1	0.090697	0.08943	0.158028
11763159_OR6C4 ///	-0.20132	0.089474	0.158099
11723540_TMEM80	-0.13402	0.089478	0.158101
11760606_EGLN3	-0.10799	0.089485	0.158107
11733866_RARS2	0.074887	0.089492	0.158109
11717793_ZDHHC3	-0.06694	0.089492	0.158109
11763153_THPO	-0.10982	0.089509	0.158132
11717328_KLF9	-0.08806	0.089538	0.158177
11759238_CLEC7A	0.103324	0.089548	0.158189
11761116_FAM65B	-0.21034	0.089551	0.15819
11724449_ZDHHC22	-0.15624	0.089566	0.158211
11737521_CHRNA9	-0.21576	0.089601	0.158266
11728243_SLC12A1	-0.10205	0.089642	0.158333
11746216_FKBP8	-0.10098	0.089655	0.158351
11763820_GABRA2	-0.11171	0.08967	0.158371
11745983_GRIA1	-0.13149	0.089676	0.158375
11738113_PCDHB1	-0.15424	0.089712	0.158433
11763116_IGHG1	0.259823	0.089732	0.158463
11757607_SUSD4	0.140862	0.089763	0.158513
11753564_PPP1R14A	0.086398	0.089784	0.158543
11727993_STX11	0.196744	0.089791	0.158546
11760576_PXK	-0.2041	0.089792	0.158546
11747773_SPTSSB	-0.12043	0.089813	0.158576
11737315_LOC102724	-0.14344	0.089815	0.158576
11721917_ANKRD37	-0.10901	0.089834	0.158603
11730616_PLCD4	0.136912	0.089881	0.158681
11727862_LIX1	-0.08138	0.089908	0.158722
11756107_MEX3A	-0.07838	0.089922	0.158741
11755510_PCCB	-0.07483	0.08993	0.158749
11748388_GLYR1	-0.09458	0.089943	0.158767
11755166_ESAM	0.133271	0.08996	0.158792
11735467_C10orf82	-0.12873	0.089981	0.158823
11729606_PGM2	0.075179	0.089994	0.158841
11722407_CHAC1	-0.08328	0.090022	0.158879
11760948_ANAPC2	-0.147	0.090023	0.158879

11748990_KIF1B	0.097227	0.090028	0.158882
11729954_FHIT	0.09466	0.090082	0.158972
11715419_LY6E	-0.08022	0.090104	0.159006
11747704_CLDN11	-0.1088	0.090112	0.159013
11734078_TNRC6C	0.101541	0.090116	0.159015
11743557_PPP1R3F	-0.17878	0.090161	0.159088
11757230_CHODL-AS1	-0.11205	0.090211	0.159171
11741448_DUSP13	-0.15373	0.090224	0.159188
11752527_ZNF554	-0.14252	0.090227	0.159188
11742203_PCDHGA11	-0.12002	0.090269	0.159256
11761488_SDF4	0.103222	0.090275	0.159261
11758342_COL3A1	0.164093	0.090295	0.15929
11731742_TRDMT1	0.182704	0.090299	0.159293
11762353_NPSR1-AS1	0.24047	0.090328	0.159337
11721896_GAS1	-0.09714	0.090337	0.159343
11733521_NTSR1	-0.10552	0.090338	0.159343
11727668_MTA1	-0.10373	0.090349	0.159355
11721946_MRVI1	0.156083	0.090351	0.159355
11725916_BVES	0.1378	0.090376	0.159393
11743828_LDLRAD4	-0.17014	0.090414	0.159454
11763507_CINP	0.129082	0.090429	0.159474
11743534_PPP2R5C	-0.08093	0.090443	0.159494
11736592_LDB3	-0.11985	0.090471	0.159538
11722675_HIF3A	-0.08644	0.090475	0.159539
11755000_SPAG9	0.119808	0.090481	0.159541
11763301_RPL39	0.069559	0.090483	0.159541
11724911_TMPRSS2	-0.14163	0.090545	0.159645
11751578_TTC31	-0.08273	0.090565	0.159675
11738269_RUFY4	-0.12969	0.090579	0.159693
11719366_CXCL1	-0.06648	0.090594	0.159714
11746703_FAM219A	-0.08659	0.090623	0.159761
11742108_PPT1	0.070495	0.09065	0.159802
11756039_MRPL52	0.096227	0.090678	0.159845
11764052_MARC2	-0.18598	0.090709	0.159894
11736683_CYFIP1	0.074359	0.090724	0.159915
11762921_COX3	-0.14829	0.090728	0.159916
11740304_KIAA0319	-0.11907	0.090735	0.159922
11731562_APC	-0.14787	0.090748	0.159941
11732504_HPN	-0.09832	0.090758	0.159951
11722111_HLX	0.162628	0.090763	0.159951
11722289_ZBTB43	0.098878	0.090764	0.159951
11731401_TMCO1	-0.08085	0.09077	0.159955
11732065_RIPPLY2	-0.13383	0.090795	0.159994
11716978_NSFL1C	-0.07217	0.09083	0.160049
11751734_ODF2	-0.10975	0.090851	0.160072
11728079_TBC1D22B	0.072942	0.090852	0.160072
11751846_ORAOV1	0.137927	0.090853	0.160072
11758307_TPT1	0.073943	0.090871	0.160099
11760970_RBM23	-0.17402	0.090906	0.160154
11746776_RPRD1A	-0.08183	0.090916	0.160166
11726895_IRAK3	-0.13667	0.090933	0.160191

11746980_CH17-351M	-0.18826	0.090941	0.160199
11725883_ERVFRD-1	-0.10836	0.090958	0.16022
11721886_CDC42	-0.10913	0.09096	0.16022
11762134_ZNF76	-0.16443	0.090965	0.160224
11760240_MCM9	0.089068	0.090985	0.160252
11745783_TGM3	-0.14861	0.090988	0.160252
11761472_SLC35B3	-0.16413	0.090995	0.160259
11738273_PSMG4	0.130582	0.091013	0.160284
11734501_NUP98	-0.10175	0.091022	0.160295
11736114_TTC14	0.086435	0.091074	0.160381
11754526_PTGES	0.079118	0.091085	0.160395
11735639_KRTAP5-2	-0.09571	0.091091	0.160395
11740564_FAM76A	-0.12617	0.091092	0.160395
11751144_ERCC8	0.078812	0.091099	0.160403
11737497_ADRA2B	-0.09849	0.091123	0.160439
11742065_KCNQ2	-0.1124	0.091153	0.160486
11716011_TNFAIP1	0.095025	0.09119	0.160545
11724984_EXPH5	0.178692	0.091221	0.160594
11740210_ZSCAN12	0.231393	0.091231	0.160604
11745476_TRMU	0.079859	0.091233	0.160604
11732434_ZNF250	0.138848	0.091238	0.160606
11720202_GMPR	-0.10725	0.091282	0.160678
11759876_STARD13	0.102708	0.091304	0.160711
11753148_MADCAM1	-0.12817	0.091325	0.160741
11728332_KCTD15	-0.09842	0.091353	0.160785
11741659_CDH26	-0.13635	0.091411	0.160883
11759483_WWTR1	-0.07405	0.091421	0.160893
11722642_DGAT2	-0.06674	0.09147	0.160974
11749949_LBX1-AS1	-0.1342	0.091476	0.16098
11742826_PHF3	-0.07607	0.091501	0.161017
11751966_IARS	0.068014	0.091523	0.161051
11721422_SPAG16	0.158591	0.091569	0.161125
11753402_RAB35	-0.09066	0.091582	0.161143
11737139_MURC	0.168698	0.091593	0.161157
11728689_RABIF	-0.08724	0.091603	0.161168
11749345_MVB12B	0.126472	0.091612	0.161178
11725114_ANKHD1	0.086537	0.091624	0.161194
11761581_MPHOSPH6	-0.15137	0.091645	0.161224
11736051_FAM229B	-0.09607	0.091661	0.161246
11760634_ARHGEF10	0.137716	0.091669	0.161255
11732454_KCNJ15	-0.14475	0.091744	0.161377
11730717_PAGE5	0.072382	0.091745	0.161377
11729970_SPTB	0.109264	0.091879	0.161601
11720902_CACNB3	-0.16028	0.091879	0.161601
11734938_CSF2RA	-0.13828	0.09192	0.161662
11726345_GPA33	-0.11286	0.091921	0.161662
11757008_LPCAT3	-0.12811	0.091942	0.16169
11735267_SIGLEC8	-0.11711	0.091943	0.16169
11751368_CD34	-0.1181	0.091956	0.161705
11746838_GIGYF2	-0.08002	0.091961	0.161705
11738557_BARHL2	-0.08825	0.091961	0.161705

11761022_LOC100507	-0.07576	0.091968	0.161711
11742455_DPF3	0.134189	0.091994	0.161747
11716081_WDR77	0.07308	0.091995	0.161747
11746477_COX6B2	-0.10407	0.092	0.161747
11747862_BCAR1	0.096982	0.092002	0.161747
11760758_UCHL5	0.231053	0.092028	0.161788
11742493_EME2	-0.11124	0.092048	0.161817
11752745_LGMN	-0.11885	0.09206	0.161832
11730165_GSC	-0.15174	0.092063	0.161832
11745044_PCYT1B	-0.0744	0.092069	0.161835
11731664_UTP20	0.093559	0.092081	0.161851
11729412_C9orf142	-0.10515	0.092111	0.161898
11746389_CDH3	-0.07325	0.092116	0.161902
11756687_MRPL46	0.08162	0.092124	0.161906
11749036_TRAF2	-0.08039	0.092126	0.161906
11749131_DDX24	0.077512	0.092199	0.16203
11734165_ARHGEF26	0.094859	0.092213	0.162048
11726260_RNF141	-0.07043	0.092243	0.162096
11755067_CLEC16A	0.082393	0.092268	0.162133
11735286_MYADML2	-0.08639	0.092274	0.162138
11751008_SMARCD1	-0.11947	0.092298	0.162175
11736330_SLC2A11	0.069309	0.092304	0.162179
11741290_TNFSF14	-0.17337	0.09231	0.162183
11727436_AIPL1	-0.12247	0.092336	0.162224
11728922_ONECUT2	-0.14287	0.092369	0.162271
11726351_EFNA5	-0.09342	0.09237	0.162271
11718712_SFSWAP	0.081545	0.092401	0.16232
11716357_AHSG	-0.13565	0.092415	0.162338
11754433_CLRN3	-0.17375	0.092449	0.162392
11717874_IRF8	-0.11269	0.092452	0.162393
11728979_ADGRG3	-0.11847	0.092462	0.162403
11735691_RBM4B	0.109443	0.092477	0.162424
11731906_PRRG3	-0.14743	0.092532	0.162516
11742896_H2AFY	-0.07808	0.092542	0.162528
11739564_ITPR1	-0.1664	0.092563	0.162558
11724463_GPNMB	-0.09666	0.092567	0.162559
11723388_EMX2	-0.14534	0.092592	0.162597
11724777_PGM2L1	0.147239	0.092613	0.162629
11761647_ZNF333	0.113352	0.092621	0.162636
11740852_TRAPPC2	-0.15879	0.092633	0.162653
11728211_TRMT1L	-0.13893	0.092652	0.16268
11724632_SYT13	-0.11003	0.092696	0.16275
11723770_SNX24	-0.07112	0.092709	0.162768
11760982_CDK10	-0.09548	0.092722	0.162781
11748386_SOX13	-0.09802	0.092723	0.162781
11738372_CA5A	-0.14137	0.092739	0.162802
11716132_FSTL3	-0.07405	0.092763	0.162834
11717108_PCM1	-0.08727	0.092763	0.162834
11731138_MYPN	0.066417	0.092785	0.162865
11762069_SEPT6	-0.15783	0.092801	0.162889
11727676_BLOC1S3	-0.08811	0.092825	0.162925

11717518_OCIAD1	0.067442	0.092852	0.162966
11728929_CPNE7	-0.09149	0.092887	0.163022
11719353_GCC2	-0.09242	0.092892	0.163024
11727854_NUP50	-0.09075	0.092906	0.163043
11735835_PPHLN1	0.07745	0.092916	0.163055
11759006_ZNF771	0.126927	0.092927	0.163068
11716645_TTC19	0.091853	0.092958	0.163118
11715173_KRTAP10-1	-0.11156	0.092974	0.163131
11727054_HOXB13	-0.07577	0.092977	0.163131
11720546_ANGEL1	-0.09805	0.092979	0.163131
11733758_ZNF446	-0.09089	0.092979	0.163131
11731115_ACTL9	-0.17783	0.092993	0.163149
11759944_ZFP28	-0.09753	0.093009	0.163168
11726792_ALS2CL	-0.09166	0.093011	0.163168
11715174_KRTAP10-1	-0.11566	0.09302	0.163174
11748113_RPN1	0.07346	0.09302	0.163174
11724709_SOX11	-0.07872	0.093038	0.163199
11748177_XYLT2	-0.07328	0.093068	0.163245
11716475_STRAP	-0.07515	0.093072	0.163247
11760874_USP36	0.148501	0.093083	0.16326
11757360_BAG3	-0.06334	0.093115	0.163311
11727850_NIP7	0.093516	0.093123	0.163318
11757706_DCUN1D5	0.064332	0.093154	0.163367
11755620_HYAL1	-0.10112	0.093163	0.163377
11742416_USP24	0.068624	0.093183	0.163401
11757250_INGX	-0.15586	0.093184	0.163401
11763241_TRAV12-2	-0.0771	0.093196	0.163418
11751754_RTCB	-0.08499	0.093232	0.163474
11741974_SLC26A5	-0.11071	0.093247	0.163496
11735158_ZNF501	-0.09804	0.093306	0.163593
11762961_TPT1	-0.24157	0.093323	0.163617
11757064_WBSCR16	-0.07128	0.09333	0.163624
11731083_RNF144B	0.080297	0.093343	0.16364
11722947_MUM1L1	0.111923	0.093349	0.163644
11730945_TRMT2A	-0.0958	0.09336	0.163657
11738767_OR8B4	-0.12843	0.093366	0.163662
11746553_SSX2IP	0.131197	0.093374	0.163671
11728419_KIF7	-0.14847	0.093379	0.163674
11736100_GBA /// G	0.08815	0.093401	0.163701
11727595_KIAA2026	0.066145	0.093403	0.163701
11738242_TLX1NB	-0.12468	0.093407	0.163701
11733743_AAMDC	-0.08781	0.093408	0.163701
11735787_BARX2	-0.09231	0.09345	0.163763
11726923_ASB6	0.092546	0.09345	0.163763
11732599_CHRNB2	-0.07195	0.093456	0.163768
11737508_WDR87	0.14304	0.09348	0.163804
11731976_DNAJB3	-0.12594	0.093503	0.163836
11732882_FBXO43	-0.17621	0.093505	0.163836
11721929_TMCC2	-0.10594	0.093516	0.163849
11735942_HIST1H4D	-0.13992	0.093558	0.163917
11726231_NR0B2	-0.1641	0.093569	0.16393

11746442_SLC35C2	0.073859	0.093586	0.163955
11730188_OLA1	0.071693	0.09367	0.164095
11745600_TSKS	-0.1691	0.093688	0.16412
11748303_PSMC1	-0.07867	0.093704	0.164143
11730380_KRIT1	0.07233	0.093812	0.164326
11737435_HMX2	-0.14809	0.093818	0.164332
11761953_FXYD2	-0.09807	0.093829	0.164345
11760015_CELP	-0.11126	0.09384	0.164358
11726079_GALNT15	-0.10679	0.093848	0.164366
11737763_TIAM2	0.071261	0.09389	0.164434
11737451_FGF5	0.112893	0.093902	0.164445
11750571_AGTPBP1	-0.10694	0.093903	0.164445
11752052_MYH9	0.096592	0.093908	0.164448
11756369_MECR	-0.07658	0.093943	0.164502
11762073_ADGRG5	0.128385	0.094025	0.164641
11725575_EML6	0.107309	0.094036	0.164653
11742355_OR2A2	-0.1536	0.094044	0.164661
11735026_LOC105369	-0.07088	0.094074	0.16471
11724250_SLC39A8	0.084523	0.094086	0.164724
11738660_GAL3ST2	-0.12016	0.09411	0.164759
11734184_PRDM5	-0.09225	0.094139	0.164805
11730973_CA4	-0.10792	0.094164	0.164843
11728890_SLIT1	-0.14602	0.094196	0.164893
11733550_ZIC5	-0.09843	0.094211	0.164913
11748939_TOM1L2	0.099654	0.094224	0.16493
11759216_PCDHB13	-0.11923	0.094262	0.16499
11737015_KCNQ4	-0.28712	0.094274	0.165006
11753237_LOC728989	-0.10432	0.094291	0.16503
11753056_ZNF667	-0.07594	0.094299	0.165038
11731747_HEPACAM	-0.08977	0.094304	0.16504
11761541_CASP1	-0.12159	0.094313	0.16505
11732752_RBM44	-0.10489	0.094329	0.165073
11728142_ZBTB32	0.091683	0.094341	0.165087
11744813_AANAT	-0.08701	0.094351	0.165099
11722521_HKDC1	-0.06486	0.094355	0.1651
11732926_VNN3	0.0758	0.094365	0.165112
11748488_DLD	0.070915	0.094399	0.165166
11728486_LIMD1	0.145778	0.094442	0.165235
11717252_NDUFB11	0.070542	0.094451	0.165242
11743579_MRPS6	-0.07775	0.094453	0.165242
11742695_WAC	0.083623	0.094458	0.165245
11760120_LOC100507	-0.11742	0.094498	0.165309
11752698_PCED1A	0.071546	0.09451	0.165324
11717333_HACD2	-0.10562	0.094524	0.165339
11750808_ALOX15	-0.12674	0.094525	0.165339
11735615_PRR35	-0.11945	0.094559	0.165391
11724930_DTX1	-0.08289	0.094564	0.165395
11755816_COL21A1	-0.12217	0.094575	0.165409
11746706_EMC9	-0.06357	0.094591	0.16543
11727076_PLAC4	-0.114	0.094601	0.165441
11726803_HELZ2	-0.09768	0.094604	0.165441

11758720_RPS25	-0.08511	0.094673	0.165556
11757753_IPO7	0.066437	0.094682	0.165566
11732401_DKK4	-0.07788	0.094703	0.165596
11748627_UGDH	0.083765	0.094726	0.16563
11733338_FANCE	-0.13461	0.094743	0.165655
11715526_ATP6V1G2-	0.069969	0.09475	0.165661
11731828_GPC2	-0.34454	0.094783	0.165713
11758082_FAM46D	-0.15019	0.094795	0.165728
11743245_OR52E6	-0.11555	0.094842	0.165804
11747667_C16orf58	-0.11685	0.094877	0.165858
11749792_PBX1	0.168748	0.094882	0.165863
11740288_CHRNA1	-0.0884	0.094938	0.165953
11744799_XRCC4	-0.10788	0.094949	0.165967
11753416_ABCA2	-0.11274	0.094957	0.165975
11756649_SEH1L	0.072445	0.09498	0.166004
11732163_ZCRB1	-0.10684	0.09498	0.166004
11761020_RTCA	-0.16282	0.095003	0.166034
11761112_GPATCH4	0.13373	0.095004	0.166034
11717791_APPL2	-0.09346	0.095085	0.166169
11742070_CYP11B2	-0.12592	0.09512	0.166224
11740612_SLC8A1	-0.07903	0.09513	0.166236
11719621_GTPBP1	0.099357	0.095165	0.166292
11744689_ARMC10	0.096842	0.095178	0.166309
11753999_CD58	-0.10879	0.095196	0.166334
11719454_BBS1	0.096151	0.09526	0.166439
11745555_C11orf74	-0.07275	0.095289	0.166484
11725595_IYD	0.081702	0.095303	0.166503
11733552_MAGI1	0.129662	0.095321	0.166525
11761640_TERF2	0.104623	0.095322	0.166525
11761338_TCAF2	0.104326	0.095346	0.16656
11718029_RAB11A	-0.07049	0.095362	0.166582
11748914_AP3B2	0.15887	0.095391	0.166622
11760490_ST3GAL5	0.143091	0.095392	0.166622
11734193_FXYD3	-0.0987	0.095411	0.16665
11721359_ITIH1	-0.1923	0.095421	0.166662
11754558_FAM71E1	-0.20714	0.095431	0.166672
11740554_PHOSPHO1	-0.12203	0.095476	0.166735
11737938_RIPK1	-0.08706	0.095477	0.166735
11756423_ITIH1	-0.14	0.095479	0.166735
11752719_DHX34	-0.0902	0.09548	0.166735
11722624_CNOT3	-0.16651	0.095497	0.166758
11738922_OR5I1	0.177924	0.095523	0.166793
11735052_KIAA1211	0.12871	0.095524	0.166793
11755164_LEFTY1	0.200547	0.095528	0.166794
11748425_ZNF736	0.182776	0.095532	0.166796
11738968_KRTAP19-8	-0.17383	0.095546	0.166815
11727656_EPHB1	0.07434	0.095589	0.166883
11756823_CENPL	-0.10003	0.095608	0.166911
11742570_OR8J3	-0.13191	0.095636	0.166953
11717838_SRSF4	-0.06249	0.095647	0.166967
11747570_MGST1	-0.07304	0.095661	0.166985

11755026_PHF2	0.085829	0.095679	0.167007
11763177_KIAA0907	0.079678	0.095681	0.167007
11748231_UNC13B	0.098161	0.095701	0.167035
11753591_PRR13	-0.07726	0.095705	0.167035
11725699_XIAP	0.078617	0.095707	0.167035
11732598_CHRNB2	0.083096	0.095759	0.16712
11729523_NLRC5	0.134159	0.095784	0.167157
11742921_HSPA9	0.080464	0.095798	0.167176
11726062_PTGIR	-0.12136	0.095809	0.16719
11746012_EXD2	-0.0668	0.095819	0.167201
11722439_FLYWCH2	-0.0722	0.095838	0.167228
11722006_KLHL25	-0.0825	0.095871	0.167279
11760559_PHLDB3	0.187624	0.095888	0.167303
11762415_PLPP3	-0.07866	0.095936	0.167381
11757996_FKBPL	-0.12362	0.095952	0.167403
11753132_SLC50A1	0.098899	0.095956	0.167404
11721911_SOX8	-0.07659	0.095975	0.16743
11734344_TRIM10	-0.06499	0.095979	0.167433
11725497_RAP1GAP	-0.1762	0.095994	0.167452
11721368_CRLF1	-0.09499	0.096004	0.167464
11739393_RBPJ	-0.09063	0.096023	0.167492
11717069_DIP2B	-0.06679	0.096027	0.167493
11744910_NUP160	0.0924	0.096065	0.167553
11759920_PHC3	0.167254	0.096101	0.16761
11740597_FAM161B	-0.12278	0.096109	0.167613
11727971_ST7 /// S	0.076138	0.09611	0.167613
11738651_KRT35	-0.12748	0.096118	0.167622
11755714_GPX5	-0.081	0.096122	0.167622
11738258_CXorf49 /	-0.12753	0.096157	0.167676
11760079_ABCA1	-0.16128	0.096163	0.167682
11755220_C17orf97	0.099506	0.09617	0.167688
11729583_CA1	-0.12141	0.096183	0.167705
11751477_B4GALT6	-0.09306	0.096198	0.167715
11726664_ZMIZ2	0.151624	0.096203	0.167715
11742030_ZMIZ2	0.151624	0.096203	0.167715
11748570_FAXDC2	-0.1439	0.096205	0.167715
11731456_MFN1	0.071559	0.096206	0.167715
11740863_TPH1	-0.18419	0.096247	0.16778
11722057_SNRNP40	-0.0765	0.096263	0.167801
11722666_GABRB3	0.124796	0.096267	0.167803
11727098_NUMBL	-0.07819	0.096286	0.167827
11755097_KIF17	0.095858	0.096288	0.167827
11759244_LINC00477	-0.118	0.096306	0.167853
11759103_CYP4F3	0.164754	0.096311	0.167855
11735918_FBXL6	-0.10408	0.096318	0.167862
11729782_BOLL	0.218522	0.09633	0.167873
11729071_HS2ST1	0.118148	0.096331	0.167873
11758559_NUDT4 ///	-0.14702	0.09634	0.167881
11721070_DEF8	0.074758	0.096343	0.167881
11717933_DNAL4	-0.07981	0.096361	0.167907
11751087_SF1	0.068545	0.096422	0.168007

11754951_NCOA7	0.075714	0.096446	0.16804
11763206_SH3RF2	0.16545	0.096448	0.16804
11756219_FGL1	-0.14794	0.096455	0.168047
11762932_PIK3IP1	-0.1162	0.096499	0.168116
11736840_FAM198A	0.087472	0.096502	0.168116
11738712_OR52K2	-0.154	0.09652	0.168142
11724396_ZNF83	-0.09425	0.096585	0.168249
11734215_MPP7	-0.1237	0.096617	0.168298
11727330_C7orf61	0.102449	0.096632	0.168319
11750698_ZNF418	0.117069	0.096647	0.16834
11760641_SLC22A20	-0.11065	0.096662	0.16836
11715333_NXNL2	-0.1072	0.096667	0.168361
11761524_SLC2A6	-0.10268	0.096686	0.168388
11731177_BCL2L15	-0.11201	0.096696	0.1684
11751967_RAPGEF6	0.129407	0.096716	0.168429
11722123_CYP27B1	0.105849	0.096743	0.168471
11737091_MED12L	0.112827	0.09675	0.168477
11758718_ATP5E ///	0.115404	0.096758	0.168484
11727747_TMEM63C	-0.12167	0.096806	0.168563
11747269_ALB	0.112826	0.09681	0.168563
11735454_TBP	0.064669	0.096819	0.168572
11751176_PRUNE2	0.213238	0.096838	0.1686
11743735_CCNK	-0.0727	0.09686	0.168631
11737253_SLC16A11	-0.09124	0.096893	0.168681
11715345_ADAMTS15	-0.10673	0.096897	0.168681
11750606_CD101	-0.09026	0.096898	0.168681
11734381_RAD51B	0.169211	0.096918	0.168709
11722335_FNDC5	-0.08698	0.096932	0.168727
11730191_SEC14L2	-0.11903	0.096956	0.168762
11728791_THRSP	-0.08508	0.096961	0.168764
11727283_ECM2	0.124871	0.096963	0.168764
11759389_OR10A2	-0.09494	0.097006	0.168831
11731707_VRTN	-0.08114	0.097037	0.168877
11742895_DRG2	0.084833	0.097039	0.168877
11740463_SYCE1L	-0.07916	0.097046	0.168883
11763649_APOC1	-0.22681	0.097073	0.168915
11726355_ENPP1	-0.16206	0.097075	0.168915
11747344_ACADM	0.118398	0.097078	0.168915
11762826_PAH	-0.10488	0.09708	0.168915
11739119_CNPY3	-0.11016	0.097084	0.168915
11730785_TTC21A	0.128597	0.097084	0.168915
11762929_FAM66D //	-0.11126	0.097122	0.168972
11720150_C1orf116	-0.18154	0.097124	0.168972
11743364_EIF5B	0.092621	0.097135	0.168985
11745645_STMN1	-0.07276	0.097205	0.169101
11751913_FGD5	0.118387	0.097212	0.169107
11725769_MIER3	0.13445	0.09724	0.169149
11740065_IL17RA	0.07253	0.097319	0.16928
11762756_CHRNA3	-0.1335	0.097355	0.169338
11725903_ZNF502	0.16923	0.097361	0.169341
11763138_RBM15	0.090841	0.097374	0.169355

11750280_EFEMP2	-0.06655	0.097376	0.169355
11728265_LILRB2	-0.15385	0.097401	0.169392
11720108_THAP11	-0.07943	0.097406	0.169396
11734034_FPR2	-0.11648	0.097456	0.169477
11748531_CACFD1	-0.10062	0.097503	0.169551
11731558_WNT2	-0.12131	0.097506	0.169551
11761808_SFPQ	-0.14126	0.097515	0.169556
11723218_GPN1	0.073296	0.097515	0.169556
11721890_ADGRL1	-0.07807	0.097536	0.169585
11746871_GATAD2A	-0.0739	0.097539	0.169585
11730153_CCDC114	-0.11391	0.097569	0.16963
11730418_PRND	-0.11365	0.097586	0.169655
11755741_MED16	-0.06724	0.097605	0.169682
11731464_CPM	-0.07825	0.097619	0.169699
11751583_LOC100288	-0.12391	0.097624	0.169702
11751786_CRNN	-0.14135	0.097636	0.169712
11752567_TP63	-0.15739	0.097637	0.169712
11745466_CDADC1	0.124729	0.097647	0.169724
11728219_BCLAF1	-0.07635	0.097657	0.169735
11753866_CTLA4	-0.09157	0.097684	0.169776
11739521_ST8SIA4	0.075039	0.097724	0.169839
11753778_KCNMB2	-0.14144	0.097743	0.169866
11750474_CABP4	-0.09869	0.097762	0.169894
11733991_ANGEL2	0.093391	0.097776	0.169913
11749380_WDR74	-0.07353	0.097793	0.169936
11739701_B3GNT2	-0.09536	0.097806	0.169949
11759586_RAB18	-0.0911	0.097808	0.169949
11753236_GPER1	-0.07596	0.097811	0.169949
11724574_ECE2	-0.07519	0.09782	0.169959
11730348_TLDC1	-0.08935	0.097832	0.169973
11731656_VAV3	-0.11351	0.097838	0.169978
11760568_EPGN	0.142574	0.097896	0.170072
11716444_KEAP1	-0.06806	0.097915	0.1701
11716701_CCNDBP1	0.063751	0.097925	0.170104
11729814_ZADH2	-0.09419	0.097925	0.170104
11732558_TEX37	-0.07888	0.097967	0.170172
11715823_SYPL1	-0.07146	0.097973	0.170175
11753855_CD28	-0.13429	0.097989	0.170198
11762459_XRCC1	-0.09791	0.098025	0.17025
11748432_ARMC6	-0.07779	0.098026	0.17025
11754749_TEX35	-0.09996	0.098061	0.170299
11734592_SLC10A7	-0.16326	0.098061	0.170299
11742525_OR52B6	-0.10346	0.098073	0.170312
11731156_APAF1	-0.13869	0.098076	0.170312
11763118_C7orf34	-0.06576	0.098082	0.170317
11745777_TTC31	-0.08962	0.098108	0.170356
11724504_SPON1	-0.08886	0.098116	0.170364
11725936_CAMK2A	-0.08645	0.098127	0.170376
11754165_LRRK1	-0.12851	0.098142	0.170396
11733260_TNPO2	-0.07052	0.098164	0.170429
11760259_SRSF9	0.106659	0.098173	0.170439

11754126_CFLAR	0.088306	0.098262	0.170586
11725331_USP9X	0.07486	0.098275	0.170604
11726674_ASCL1	-0.11669	0.09835	0.170727
11748640_DLGAP1	-0.20047	0.098359	0.170737
11759965_ANKDD1A	0.131271	0.098387	0.17078
11732512_CDX1	-0.08659	0.0984	0.170788
11727758_SYCP3	0.097707	0.098401	0.170788
11761386_ZNF599	-0.16555	0.098402	0.170788
11763389_LINC00906	-0.15273	0.098408	0.170791
11734887_SEMA3G	-0.10119	0.098447	0.170853
11748183_PLEKHH1	0.139502	0.098457	0.170865
11744104_TMC5	0.188092	0.098474	0.170884
11724541_VWF	-0.13521	0.098475	0.170884
11731463_CPM	0.100637	0.0985	0.17092
11742916_LMTK2	0.067868	0.098539	0.170983
11735189_H2AFY	-0.07598	0.098567	0.171024
11763047_AMELY	-0.11525	0.098595	0.171066
11752749_SYCP2L	0.271624	0.098598	0.171066
11725011_CEP78	-0.06625	0.098632	0.17112
11723834_TAPT1	-0.13117	0.098654	0.171152
11719163_CLCN5	-0.12362	0.098664	0.171164
11753043_DBF4	-0.09035	0.098669	0.171165
11758853_ARPC5L	0.081655	0.09868	0.171179
11720023_FAM118A	0.150929	0.098691	0.171191
11761063_SPDYE2B	0.103566	0.098696	0.171191
11737359_MYOZ3	-0.12603	0.098697	0.171191
11760570_SNX14	-0.08937	0.098723	0.171228
11726993_GFAP	-0.11199	0.098734	0.171242
11763695_ABTB1	0.102936	0.098775	0.171308
11756496_SERPINA6	-0.08537	0.098797	0.171338
11738429_POU1F1	0.216603	0.098804	0.171346
11760235_CBR1	-0.11129	0.098811	0.171352
11736694_TRIM38	0.071923	0.098859	0.171428
200057_PMNONO	-0.06451	0.098867	0.171437
11750038_MECR	-0.08728	0.098906	0.171497
11731056_CTAGE5	0.116295	0.098964	0.171593
11737948_C5	-0.10424	0.098988	0.171626
11743124_TLN1	-0.08083	0.098997	0.171626
11751042_F5	-0.16166	0.099	0.171626
11734522_CHRDL2	-0.09126	0.099007	0.171626
11722480_FGR	-0.13945	0.099008	0.171626
11723822_ZNF862	0.125116	0.099009	0.171626
11727127_TYRP1	0.231519	0.09901	0.171626
11728015_TRIM66	-0.14119	0.099012	0.171626
11743999_SSBP4	-0.0752	0.099031	0.171653
11763900_SNORA80A	-0.0997	0.099045	0.171672
11722779_SLC6A19	-0.10835	0.099068	0.171705
11760005_DNAJA1	0.097404	0.099086	0.17173
11729117_PRKCG	0.107106	0.099103	0.171753
11751029_VIPR1	-0.06639	0.099146	0.171821
11720911_CAPN6	-0.15177	0.099149	0.171821

11727601_WFDC1	-0.15697	0.09917	0.171851
11760007_ROPN1 ///	-0.09631	0.099222	0.171935
11731163_VIPR2	0.075648	0.099252	0.171981
11756253_LGI4	-0.09867	0.099263	0.171995
11761945_ALPK1	-0.09071	0.099277	0.172012
11740414_TLE1	-0.10892	0.099291	0.172031
11758755_MPC2	-0.10689	0.099298	0.172037
11725612_ATP5J2	-0.06629	0.099328	0.172083
11741842_CST11	-0.10975	0.099334	0.172087
11739580_TCTN2	0.173429	0.099345	0.172099
11720887_PLXNA3	0.120127	0.099379	0.172153
11722055_PLSCR3 //	0.065542	0.099391	0.172159
11762677_ZCCHC2	-0.20721	0.099391	0.172159
11741689_SUMF2	0.075468	0.099393	0.172159
11721965_NOTCH4	-0.19444	0.0994	0.172165
11735160_KCNAB3	-0.12076	0.09945	0.172245
11726909_ZBED2	-0.14548	0.099475	0.172282
11737034_RBBP6	-0.07426	0.099487	0.172297
11725404_RPL13	0.06611	0.099509	0.17232
11753526_MED25	-0.08944	0.09951	0.17232
11742150_OR2B3	-0.09872	0.099516	0.17232
11741212_GPN1	0.067353	0.099517	0.17232
11750560_FAM168B	0.071696	0.099518	0.17232
11737018_AGAP2	-0.10063	0.099549	0.172368
11749040_PGM2	0.070165	0.099554	0.17237
11741834_KIR2DS4	-0.10365	0.0996	0.172444
11715372_MDH1	-0.07885	0.099616	0.172466
11733657_HMX1	-0.10485	0.099621	0.172469
11762421_FBXO11	0.090461	0.099626	0.172471
11761852_NVL	-0.12648	0.099633	0.172476
11757288_CST3	0.08557	0.099654	0.172506
11756851_SUN1	0.13124	0.099686	0.172556
11717772_OTUB1	-0.09322	0.099693	0.172562
11754262_CHD9	-0.0974	0.099696	0.172562
11758987_PF4	-0.11011	0.099703	0.172567
11760302_HEXA	-0.07891	0.099728	0.172604
11719931_ARGLU1	0.081066	0.099743	0.172624
11737679_KREMEN1	-0.16809	0.099754	0.172638
11755530_ZNF34	0.163265	0.099781	0.172677
11730384_GREM2	0.093892	0.099848	0.172787
11762304_CCND3	0.213131	0.099868	0.172816
11715525_ATP6V1G2-	-0.09701	0.099892	0.172851
11736411_NUP62	-0.08759	0.099955	0.172954
11748283_MAVS	-0.08018	0.099969	0.172972
11735984_OR2B6	-0.11796	0.099988	0.172998
11762253_LMO3	-0.18368	0.099991	0.172998
11730372_FYB	0.121153	0.100004	0.173015
11739207_TCAF1	-0.12067	0.100023	0.173042
11746993_PRSS8	-0.08347	0.100035	0.173057
11735645_MASP2	-0.15998	0.100063	0.173099
11741067_SUMF2	0.083343	0.100076	0.173114

11715542_THY1	0.073607	0.100089	0.173131
11735783_CLVS2	-0.09865	0.100116	0.173164
11746750_MAPKBP1	-0.14768	0.100119	0.173164
11718582_PSMA1	-0.06444	0.100119	0.173164
11752564_ADIPOQ	-0.12993	0.100143	0.173201
11739515_CA8	0.098465	0.100156	0.173215
11723729_KREMEN1	-0.26316	0.100159	0.173215
11758823_PINX1	-0.06562	0.100162	0.173215
11729518_C1QL2	0.171151	0.100179	0.173238
11747776_TOP3A	0.108306	0.100192	0.173254
11753121_CYP4Z1 //	-0.08532	0.100206	0.173273
11757731_RAB31	0.085906	0.100237	0.17332
11761919_PABPN1L	-0.1242	0.100252	0.17334
11717766_MGLL	-0.07841	0.100257	0.173342
11734832_GPR22	-0.13137	0.100262	0.173344
11738613_OR2J3	-0.12519	0.100267	0.173347
11741630_C3orf58	-0.10112	0.100276	0.173356
11725600_SOWAHA	-0.09091	0.100313	0.173415
11752857_GPT	-0.0929	0.10032	0.17342
11738858_CSF2RA	-0.10419	0.100341	0.173446
11720362_PHIP	-0.09208	0.100345	0.173446
11761408_RBM42	-0.29981	0.100345	0.173446
11741668_NONO	0.06166	0.100351	0.17345
11718983_CCL4 ///	0.076442	0.100356	0.173452
11746508_ZFYVE27	-0.127	0.100434	0.173581
11749149_ODF2	-0.09854	0.100441	0.173587
11737288_TIRAP	0.084943	0.100448	0.173592
11744553_ZFYVE28	0.130293	0.10047	0.173624
11719151_FAM83H	-0.23304	0.100479	0.173633
11720988_SEC22A	0.185511	0.100482	0.173633
11763709_ARL6IP4	-0.09049	0.100491	0.173641
11718499_MRPL54	-0.0763	0.100494	0.173642
11735275_IL18R1	0.247793	0.100499	0.173644
11753025_TNFRSF1B	-0.1449	0.100506	0.17365
11754380_SOSTDC1	0.086287	0.10051	0.173651
11720831_C1orf21	-0.12425	0.100518	0.173659
11740755_LYNX1	-0.16043	0.10053	0.173673
11726144_NUDT18	-0.0809	0.100536	0.173677
11745807_AMMECR1L	0.080925	0.100542	0.173682
11734429_DACT2	-0.14733	0.100552	0.173692
11731577_SLC22A1	0.099939	0.100574	0.173725
11753217_KCNJ3	-0.08808	0.100608	0.173777
11751462_SYT4	-0.12677	0.100625	0.173794
11719221_CD79B	0.094579	0.100625	0.173794
11762144_EPHA6	0.126828	0.100647	0.173826
11752440_IL12RB1	-0.08387	0.100662	0.173844
11735114_CNOT4	-0.13094	0.100665	0.173844
11752906_TELO2	-0.1169	0.100673	0.173853
11762884_HSP90AB6F	-0.14193	0.100684	0.173858
11757418_PMEL	-0.12211	0.100684	0.173858
11736895_SOCS4	0.072409	0.100773	0.174007

11739496_SLC29A4	-0.10194	0.1008	0.174047
11724233_HGSNAT	-0.13153	0.100806	0.174051
11763242_G0S2	0.168892	0.100836	0.174097
11748494_FAM230A /	-0.14684	0.100842	0.174101
11724825_DOLK	0.0935	0.100887	0.174172
11746941_FAM138A /	-0.11729	0.100894	0.174178
11735183_FFAR2	-0.17305	0.100906	0.174192
11727162_NFIX	-0.06806	0.10095	0.174263
11732244_APOL6	-0.09747	0.10096	0.174273
11756284_RNF43	0.076237	0.10099	0.174319
11731668_SGCA	-0.13433	0.101007	0.174344
11722058_ATL2	-0.06918	0.101014	0.174349
11748510_KIF13B	-0.09556	0.101055	0.174413
11752218_NPC1L1	-0.09066	0.101076	0.174443
11728737_PPP1R17	-0.10362	0.101095	0.174469
11741282_AKAP7	-0.13332	0.101106	0.174482
11747971_RBSN	0.080526	0.101112	0.174486
11753521_RAB5C	-0.10015	0.101138	0.174526
11740616_PARK2	-0.15289	0.101153	0.174545
11746907_PDE4D	0.170415	0.101186	0.174597
11743261_ANXA11	0.067253	0.101228	0.174663
11750274_TTC6	-0.07437	0.101232	0.174663
11730034_CDHR2	-0.15363	0.101235	0.174663
11740226_PRR18	-0.08792	0.101247	0.174674
11724624_CLEC1A	-0.15445	0.101249	0.174674
11720577_ARFGEF1	0.070839	0.101255	0.174677
11745881_GUCA1A	-0.10062	0.101271	0.1747
11723499_PCSK9	-0.14752	0.101285	0.174718
11738394_RAD54L2	-0.07796	0.101324	0.174778
11754805_SPIDR	-0.10504	0.101331	0.174784
11740357_MLPH	-0.1176	0.101334	0.174784
11753250_BMP1	0.233012	0.101345	0.174797
11755507_PICALM	0.071239	0.101349	0.174797
11757505_HDLBP	-0.0696	0.101353	0.174797
11716349_TMEM214	0.067963	0.101393	0.17486
11756681_CCDC86	0.078924	0.101408	0.17488
11736717_RAPGEF4	0.070172	0.101468	0.174975
11727095_TMEM19	-0.10912	0.10147	0.174975
11745448_SLC22A10	-0.12131	0.10148	0.174986
11734021_SHANK2	-0.08845	0.101507	0.175026
11742721_LRRC58	0.096262	0.101533	0.175065
11749596_LOC100287	-0.18933	0.101551	0.175089
11760542_GAS8	-0.10835	0.101568	0.175107
11740830_EPHA6	0.141957	0.101568	0.175107
11720692_DIRAS1	0.093909	0.101633	0.175213
11745948_CHEK1	-0.0975	0.101665	0.175262
11751290_BCL2L2	-0.12826	0.101681	0.175283
11721008_MED1	-0.08095	0.101687	0.175288
11763578_GLI2	0.131476	0.101698	0.1753
11724743_ZBTB17	0.118088	0.101723	0.175337
11723836_SPNS2	-0.09151	0.10173	0.175343

11755597_SLC25A47	-0.12723	0.101746	0.175364
11754641_SFTPC	-0.09636	0.101751	0.175366
11744042_MGAT4B	-0.06278	0.101755	0.175367
11763858_UTY	0.130677	0.10182	0.175473
11725777_PRDM2	-0.08593	0.101839	0.175496
11749945_CCDC162P	0.172294	0.10184	0.175496
11718021_SSFA2	0.075567	0.101845	0.175498
11728324_PPP3R2	0.068524	0.101875	0.175543
11717710_MTFP1	-0.0941	0.101904	0.175588
11749650_A2ML1	0.160967	0.101963	0.175683
11719228_CAMK1	-0.1256	0.102005	0.175749
11715272_UGT2B11	0.079157	0.102033	0.175792
11730761_CRIPT	0.071963	0.102127	0.175946
11729941_TMEM56	-0.16165	0.102157	0.175991
11745376_APOL6	0.087883	0.102165	0.175999
11731868_BICD2	0.080203	0.102173	0.176007
11733476_KCNJ6	-0.07826	0.102182	0.176011
11721954_RNF41	0.091519	0.102183	0.176011
11718766_PRSS23	-0.07305	0.102192	0.176021
11739575_CABLES1	0.136176	0.102201	0.17603
11743850_PDGFRA	0.120201	0.102215	0.176048
11719893_VASH1	-0.08829	0.102244	0.176092
11752787_TP73	-0.17502	0.102258	0.176106
11733286_CYP20A1	-0.10191	0.102259	0.176106
11717614_KIF13B	0.071077	0.102263	0.176107
11724518_GUF1	0.083029	0.102305	0.176171
11749272_PALM	-0.07549	0.102308	0.176171
11733124_PEX19	-0.07398	0.102356	0.176246
11725209_LETM1	-0.0814	0.102358	0.176246
11746308_LINC00494	-0.12701	0.102383	0.176279
11725230_CEP290	-0.06951	0.102385	0.176279
11730253_AHCTF1	-0.08436	0.102389	0.17628
11729142_KDM5C	0.065178	0.102401	0.176294
11722316_SNX13	-0.07593	0.102411	0.176306
11737691_ESRRG	0.168898	0.102424	0.176321
11749816_ULK3	0.079225	0.102428	0.176322
11731293_SLITRK1	0.104964	0.102435	0.176326
11741404_MAS1L	-0.09097	0.102442	0.176326
11754993_PXDN	-0.07687	0.102443	0.176326
11747118_BRINP1	-0.18087	0.102445	0.176326
11729459_BNIPL	-0.11217	0.102469	0.176362
11746011_METTL15	0.108086	0.102502	0.176412
11740739_ZC3H12D	-0.12198	0.10251	0.176419
11723779_CYP2U1	0.195361	0.102513	0.176419
11741987_TSGA13	-0.09858	0.102523	0.17643
11745089_NR2C2	0.124639	0.102534	0.176443
11737712_ABCC12	-0.09538	0.10254	0.176447
11744912_NFATC4	0.129251	0.102556	0.176468
11717576_SFN	-0.11033	0.10261	0.176555
11741837_KRTAP9-2	-0.13212	0.102617	0.17656
11735554_PPP1R42	0.157568	0.102652	0.176614

11747678_BZW2	0.063255	0.102658	0.176619
11759485_GPBP1	-0.13284	0.102664	0.176623
11745060_RIN1	-0.0784	0.102668	0.176623
11763892_SNORD17	0.115099	0.102717	0.176702
11755751_DEFB108B	-0.07389	0.102734	0.176719
11745269_CHD9	0.092203	0.102735	0.176719
11762572_MTHFD2L	-0.07253	0.102741	0.176725
11757848_CTSD	-0.06223	0.102755	0.176741
11727323_NAGS	-0.09671	0.102759	0.176743
11738599_KRTAP15-1	-0.12001	0.102768	0.176746
11745316_FAM25A	0.08919	0.102768	0.176746
11730900_MBOAT2	-0.06949	0.102776	0.176753
11751873_DEFB133	-0.10837	0.102786	0.176764
11757617_ZNF672	-0.12654	0.10279	0.176765
11755041_ARHGAP33	-0.12712	0.102804	0.176783
11762689_TRIM72	-0.09903	0.10282	0.176804
11753540_CHN2 ///	-0.09226	0.102872	0.176887
11744218_SNAI2	-0.12947	0.102877	0.17689
11719144_C2CD2	-0.17953	0.102931	0.176977
11751893_COL4A6	0.219616	0.102936	0.176979
11734623_CRYGN	0.131511	0.102952	0.176993
11740331_RTP3	-0.07642	0.102954	0.176993
11730459_C17orf74	-0.13243	0.102955	0.176993
11755320_ANKRD13D	0.101855	0.102967	0.177007
11715475_DDX17	-0.08728	0.102973	0.177011
11717831_EIF4E2	0.070801	0.102982	0.17702
11741093_GPD2	-0.08175	0.102989	0.177026
11750893_LMOD1	-0.0719	0.102995	0.17703
11737031_KLK13	0.107815	0.103021	0.177069
11748447_IL17RC	-0.10641	0.103042	0.177099
11760157_GUCA1A	-0.1446	0.103057	0.177118
11763114_IGHG1	0.113359	0.103075	0.177143
11751941_CNGB1	-0.09502	0.103112	0.177201
11719176_GPRC5B	-0.07707	0.103126	0.177215
11739358_ERG	-0.07872	0.103128	0.177215
11721679_RBM17	-0.08358	0.103152	0.17725
11731985_HGFAC	-0.08367	0.103158	0.177254
11744164_VRK3	0.086213	0.103225	0.177363
11725076_FAM126A	0.21615	0.103238	0.177375
11757974_POLK	0.12725	0.103239	0.177375
11748364_ANK2	-0.17199	0.103255	0.177396
11737026_IRX4	-0.1227	0.103305	0.177476
11716398_MRPL1	0.067117	0.103324	0.177502
11752998_NXF5	-0.14094	0.103354	0.177547
11762241_PTK6	0.074458	0.103358	0.177547
11723965_AGA	-0.12131	0.103369	0.17756
11746968_CCDC178	-0.14257	0.103383	0.177578
11749160_SPHK2	-0.10921	0.103484	0.177746
11761567_FXYD5	-0.09419	0.103503	0.177771
11733740_HIST1H2BM	-0.18722	0.10352	0.177795
11738694_OR51G1	-0.13436	0.103535	0.177814

11716741_SLC6A8	0.07262	0.103549	0.177822
11743438_AP1AR	-0.11205	0.103549	0.177822
11752007_RECQL5	-0.0941	0.10355	0.177822
11718837_NDUFS2	-0.06601	0.103554	0.177822
11720869_SMG6	-0.09476	0.103564	0.177833
11763104_SKAP2	-0.17276	0.103573	0.177843
11716318_MAP2K2	-0.09174	0.103601	0.177885
11753552_KDM8	-0.11797	0.103607	0.177888
11738126_SARDH	-0.12158	0.103621	0.177907
11755954_ZBTB49	0.084041	0.103658	0.177963
11733493_IL17RE	-0.07385	0.103691	0.178013
11758824_NOL6	0.149279	0.103708	0.178037
11742236_SUPT20HL1	-0.10917	0.103723	0.178052
11761329_NME9	-0.13594	0.10373	0.178052
11753972_COL6A3	-0.10279	0.103731	0.178052
11735841_KRTAP3-2	-0.15539	0.103735	0.178052
11760792_ENPP3	-0.20913	0.103735	0.178052
11731584_FCRLA	0.066767	0.103745	0.178058
11732264_OLFM2	-0.08619	0.103746	0.178058
11719869_MYCN	-0.12087	0.103778	0.178106
11730996_AMICA1	-0.14577	0.103791	0.178123
11755692_MTMR8	0.104259	0.103812	0.178152
11749824_RUFY2	0.123347	0.10384	0.178195
11746566_RBM39	0.068204	0.10387	0.17824
11754872_E4F1	0.064507	0.10388	0.178245
11726194_RTN2	-0.06535	0.103883	0.178245
11750118_ADRBK2	-0.22772	0.103884	0.178245
11740149_CTXN3	-0.09456	0.103909	0.178281
11738290_KRTAP10-1	-0.08702	0.103921	0.178296
11761002_RHD	-0.08556	0.103962	0.178361
11763845_SPATA20	0.166975	0.104005	0.178428
11733793_STX7	-0.08664	0.104011	0.178431
11758711_YWHAZ	-0.06472	0.104143	0.178652
11759661_MYO10	0.11395	0.104154	0.178664
11725438_HBD /// H	-0.1398	0.104213	0.178757
11737254_ZCWPW2	0.143725	0.104219	0.178757
11745606_LRRC18	-0.11629	0.10422	0.178757
11763549_JRKL	-0.09291	0.104223	0.178757
11729695_MVD	-0.09553	0.104287	0.17886
11763651_GET4 ///	-0.16052	0.104293	0.178862
11736296_SLC5A1	0.127762	0.104297	0.178862
11754102_TMED3	-0.07266	0.104299	0.178862
11749104_HMHA1	-0.10548	0.104334	0.178917
11737870_FAM78A	-0.11203	0.104348	0.178931
11736991_ZNF846	0.108082	0.10435	0.178931
11716907_PITHD1	-0.07758	0.104363	0.178948
11730596_NR1I3	0.066234	0.104376	0.178962
11747066_EIF4B	0.068164	0.104385	0.178962
11716618_SS18L2	0.063109	0.104386	0.178962
11748031_ALPL	0.10229	0.104386	0.178962
11717024_LOC102724	0.0665	0.10446	0.179076

11735549_ASB15	-0.12868	0.10446	0.179076
11749491_PLOD1	-0.07095	0.104474	0.179093
11756115_SRP19	-0.06487	0.104492	0.179118
11736105_ZNF750	0.143421	0.104496	0.179118
11733301_GNGT1	0.11079	0.104503	0.179118
11751045_ADGRG5	-0.08686	0.104504	0.179118
11763470_ARHGAP8	-0.16181	0.104507	0.179118
11763621_OPRK1	-0.13592	0.10451	0.179118
11734208_CNTF ///	0.09615	0.104515	0.179119
11718369_GALNT14	0.064987	0.10455	0.179174
11757216_LOC100268	-0.17137	0.104565	0.179194
11734118_BTN3A3	-0.10788	0.104576	0.179205
11751664_KCNIP2	-0.10951	0.104625	0.179284
11735585_ZNF208	0.100764	0.104637	0.179298
11731794_CDAN1	-0.08672	0.104675	0.179357
11751961_MTR	0.086239	0.104697	0.179388
11745932_TMSB10	-0.06538	0.104729	0.179437
11738099_GPR25	-0.1227	0.104746	0.179458
11731590_ZNF576	-0.10907	0.104773	0.179499
11742768_FLRT3	0.092115	0.104808	0.179553
11737222_MAGIX	-0.11135	0.104822	0.17957
11732038_RDH16	-0.19674	0.104826	0.17957
11741444_RBMY1A1 /	-0.10682	0.104839	0.179585
11729198_MAP4	0.150371	0.104842	0.179585
11753844_NDUFA3	0.0645	0.104882	0.179648
11752383_COL6A1	0.083254	0.104891	0.179657
11749738_PIGS	-0.06096	0.104909	0.179681
11750469_FAM109A	-0.151	0.104944	0.179735
11734262_AK8	0.086447	0.105003	0.17983
11738362_ANKUB1	-0.10947	0.105018	0.179846
11731815_ISL2	0.139509	0.10502	0.179846
11734319_SLC17A4	-0.11429	0.105025	0.179848
200024_PMRPS5	0.061295	0.10505	0.179886
11741333_KIF18B	-0.08522	0.105072	0.179917
11749359_BBS1	0.077354	0.105082	0.179927
11745732_SLC20A2	0.084147	0.105104	0.179959
11718585_SAP30BP	0.074681	0.10512	0.17998
11746301_LTB4R2	0.088481	0.105129	0.179989
11740819_TCP10	-0.23891	0.105154	0.180019
11718462_ELMOD3	0.109355	0.105154	0.180019
11737858_ELMOD3	0.108848	0.105199	0.18009
11742381_OR5H1	-0.1387	0.105212	0.180106
11733449_UNC45B	-0.1084	0.105224	0.18012
11739881_GLIS3	0.075523	0.105248	0.180155
11760931_AQP3	-0.09341	0.105256	0.180163
11743049_DNAJC15	-0.11707	0.105284	0.180204
11722834_CCNY	-0.06654	0.105307	0.180237
11755991_CELF3	0.071204	0.105328	0.180267
11740557_TLX1	-0.11878	0.105373	0.180337
11721707_GPAM	-0.18521	0.105412	0.180397
11749256_RRP1	0.100433	0.105447	0.180451

11762172_ETV7	0.114184	0.105452	0.180453
11731791_SLC27A6	0.158213	0.105457	0.180454
11742439_ADAL	-0.20855	0.10546	0.180454
11729255_GIPC1	0.064339	0.105463	0.180454
11761305_FAXDC2	-0.17292	0.105467	0.180455
11742842_FAM217B	-0.07257	0.105481	0.180472
11761777_ITGAX	-0.10536	0.105508	0.180512
11758399_LPAR4	-0.11083	0.105527	0.180537
11727329_SOST	-0.06896	0.105577	0.180616
11734702_DKK3	-0.16901	0.105581	0.180617
11724398_RHCG	-0.08597	0.105621	0.180679
11715374_CALR	-0.06908	0.10563	0.180688
11733526_GABRR1	0.187822	0.105637	0.180694
11723249_EML2	-0.10157	0.105648	0.180702
11716999_RASA1	-0.07626	0.105649	0.180702
11752525_USP28	-0.1237	0.105656	0.180708
11751936_CDK12	0.063124	0.105692	0.180763
11722133_MGAT3	-0.10192	0.105729	0.180815
11728027_CCDC58	-0.079	0.10573	0.180815
11731912_GPR12	-0.09681	0.10574	0.180826
11749555_MAP2K1	-0.06485	0.105749	0.180835
11755470_G2E3	-0.06304	0.105775	0.180867
11753078_GLS2	-0.11517	0.105782	0.180867
11720371_RTCA	0.070146	0.105782	0.180867
11751405_MCHR2	-0.15408	0.105782	0.180867
11730939_ZBTB8A	-0.08708	0.105798	0.180888
11723877_KCNK5	-0.1771	0.105842	0.180957
11733548_TEC /// T	0.099141	0.105956	0.181145
11736499_TMEM38A	-0.10682	0.105992	0.181201
11716919_PDIA5	0.078825	0.105997	0.181202
11761995_IL1RAP	-0.10473	0.106003	0.181205
11719946_PKNOX1	-0.11428	0.106006	0.181205
11738411_C1QTNF7	-0.1239	0.106027	0.181234
11747621_LIMK1	-0.07289	0.10607	0.181302
11742088_ANKK1	0.127473	0.10608	0.181312
11738293_KCNK10	-0.13325	0.106086	0.181316
11755697_LGR6	0.131215	0.106095	0.181325
11720506_RAB6B	0.072715	0.106127	0.181373
11749547_TMEM129	-0.06626	0.106156	0.181417
11731370_NODAL	-0.10427	0.106169	0.181432
11720270_IBTK	0.068911	0.106175	0.181436
11748019_ZNF346	0.145012	0.106206	0.181484
11760373_RBBP5	0.07753	0.106225	0.181509
11755672_GRIK5	-0.07743	0.106244	0.181536
11755327_LOC154761	-0.11367	0.10627	0.181574
11760862_OLA1	-0.22137	0.106324	0.181659
11738332_B3GALT5	0.128076	0.106352	0.181701
11747169_MOG	0.065301	0.106367	0.18172
11735188_TMEM74	-0.10612	0.106414	0.181795
11752547_IGF2-AS	-0.10485	0.106433	0.181814
11720851_NFIA	-0.12016	0.106433	0.181814

11740466_TCP11L1	0.072472	0.10646	0.181854
11762793_YWHAE	-0.12069	0.106577	0.182041
11735736_CREB3L3	-0.07855	0.106577	0.182041
11739480_NINL	-0.12693	0.106593	0.182062
11750700_ACSL1	0.084091	0.106636	0.182128
11757324_LSM12	0.069665	0.106687	0.18221
11725421_ARHGAP35	0.067081	0.106745	0.182303
11739053_SLCO2B1	-0.13423	0.106754	0.182311
11732372_CLIC5	-0.21732	0.106757	0.182311
11732929_KBTBD12	0.207288	0.106767	0.18232
11752893_SHISA9	-0.08005	0.106784	0.182343
11755840_CLCN1	-0.07642	0.106793	0.182352
11750365_AGER	-0.1532	0.106854	0.18245
11734147_SLC6A4	-0.11725	0.1069	0.182523
11729810_WFIKKN2	-0.11386	0.106912	0.182536
11738908_OR2F1	-0.1069	0.106922	0.182547
11757363_RPS15A	0.061028	0.106936	0.182563
11715583_HLA-DPA1	-0.07343	0.106939	0.182563
11718937_WDYHV1	0.068989	0.106968	0.182607
11735934_WNT9A	-0.12606	0.107082	0.182796
11745698_HAND2	-0.14853	0.107089	0.182801
11762734_GCKR	-0.1552	0.107094	0.182803
11731796_FAM47B	0.114869	0.107104	0.182813
11757961_MAGI2	0.122364	0.107116	0.182828
11725522_MESP1	-0.08305	0.107125	0.182837
11747360_TSSK1B	-0.07643	0.107137	0.182851
11752706_SEC14L4	-0.08101	0.107151	0.182868
11740212_PIK3CG	0.102837	0.107159	0.182875
11753347_NMUR1	-0.1269	0.107181	0.182907
11733171_FBXO45	0.110731	0.107196	0.182924
11738615_KRTAP10-2	-0.10044	0.107199	0.182924
11746335_RAB11FIP3	-0.07463	0.107211	0.182938
11758999_UBE2H	-0.10161	0.107253	0.183004
11749795_NFIX	-0.06675	0.107326	0.183121
11729411_AKR7A3	-0.13831	0.107337	0.183134
11757979_DEGS1	0.09443	0.107347	0.183144
11738955_XAGE5	-0.1237	0.107382	0.183197
11744971_PSORS1C3	-0.17322	0.107386	0.183197
11719074_REEP1	-0.10795	0.107389	0.183197
11734074_RBFOX1	0.128081	0.107433	0.183266
11741481_GDF2	-0.09919	0.107444	0.183279
11754912_GAPDH	-0.06215	0.107458	0.183297
11758270_DBNDD1	0.060535	0.107499	0.18336
11726051_FMO3	0.079514	0.107522	0.183393
11753200_SEC24C	-0.07826	0.107537	0.183411
11742034_ERG	-0.10892	0.107541	0.183411
11759795_SLC22A2	-0.13693	0.107576	0.183465
11734791_SEPT6	-0.11157	0.107628	0.183548
11738733_OR2T10	-0.09638	0.107632	0.183549
11734088_CBFA2T3	-0.07787	0.107674	0.183613
11733561_ARHGAP28	0.131833	0.10772	0.183684

11731575_CTBS	0.225882	0.107726	0.183689
11763902_SNORA60	0.1323	0.107739	0.183705
11739835_GLTSCR1L	-0.14888	0.107766	0.183745
11738178_IL1RL1	0.098857	0.107777	0.183757
11733589_RAX	-0.09287	0.107813	0.183811
11715253_KRTAP10-1	-0.108	0.107817	0.183812
11724312_SH3BP2	-0.08624	0.107891	0.183932
11729921_KMT5C	-0.14307	0.107905	0.183949
11756006_LCK	-0.14694	0.107925	0.183971
11758479_ANGPTL1	0.118771	0.107925	0.183971
11730125_ADAMTSL2	-0.10477	0.107931	0.183974
11724827_BORCS6	0.080479	0.107938	0.18398
11744127_CXCL2	0.106915	0.107972	0.184031
11749377_CECR1	0.105047	0.107978	0.184036
11751888_COL4A6	0.207205	0.108004	0.184066
11717157_ACP1	-0.05952	0.108004	0.184066
11752511_HTR1E	-0.14247	0.108048	0.184135
11728976_ZNF792	0.20398	0.10806	0.184141
11741001_FAM13A	-0.09892	0.108061	0.184141
11740810_OR7E24	-0.07333	0.108063	0.184141
11744958_CPSF6	-0.09496	0.108067	0.184142
11727390_STEAP2	-0.21554	0.108084	0.184165
11754280_TSC22D4	-0.09887	0.10809	0.184165
200084_PMC11orf58	0.064741	0.108092	0.184165
11762407_IL18BP	-0.11279	0.108104	0.184173
11747020_HMHA1	-0.11575	0.108104	0.184173
11760299_SPR	-0.14092	0.108138	0.184224
11760748_PRDM1	0.125929	0.108148	0.184234
11738648_AIRE	-0.09446	0.108155	0.184241
11732881_TBX1	-0.20931	0.108205	0.184319
11740545_ADAM22	0.138482	0.108218	0.184335
11739048_ACPP	0.1842	0.108244	0.18436
11735072_PCDHB9	-0.13776	0.108244	0.18436
11738734_OR51A7	-0.11737	0.108244	0.18436
11747641_USB1	-0.11279	0.108278	0.184411
11731539_SESTD1	-0.07851	0.108305	0.184451
11746877_MXD3	-0.072	0.10831	0.184452
11752078_AP2A1	-0.0799	0.108358	0.184528
11715434_PDIA6	-0.06463	0.108369	0.18454
11726177_LOC105369	0.088686	0.108381	0.184554
11748110_LGMN	-0.06015	0.108428	0.184628
11738883_TNFSF14	-0.09451	0.108445	0.18465
11731414_GALM	-0.11529	0.108459	0.184668
11763158_MKNK2	-0.09098	0.108472	0.184682
11741041_DPP7	-0.1012	0.108474	0.184682
11744642_TRMU	0.107833	0.10852	0.184753
11739084_FKBP5	-0.07968	0.108531	0.184764
11715417_SKP1 ///	-0.07337	0.108536	0.184768
11734170_LINC00632	0.098426	0.108573	0.184824
11741768_VNN2	-0.21114	0.108583	0.184835
11746781_SNCAIP	-0.14931	0.108591	0.184841

11719948_GCC1	-0.07063	0.108603	0.184856
11755193_CP	0.102453	0.10861	0.184859
11759371_PCDHGA12	0.117616	0.108612	0.184859
11754981_TIGD6	0.111044	0.108634	0.18489
11748749_GTPBP4	-0.07046	0.108642	0.184896
11740355_GPR137C	-0.13188	0.108666	0.184931
11761747_ZNF527	0.20196	0.108673	0.184935
11744238_SERPINF2	-0.0746	0.108718	0.185006
11719499_MAOB	-0.11324	0.108751	0.185056
11743678_PCDH17	0.084745	0.108765	0.185073
11737903_AR	-0.19149	0.108774	0.185082
11721122_SPNS1	-0.08005	0.108799	0.185117
11729537_PEX11G	-0.08821	0.108829	0.185163
11751169_PGAP3	-0.09922	0.108839	0.185174
11757249_INGX	-0.16045	0.108849	0.185184
11730671_OPHN1	0.07836	0.108856	0.185189
11717502_NUB1	0.102037	0.108866	0.185199
11742604_OR6C70	-0.13625	0.108871	0.185202
11748342_TPP1	0.102483	0.108876	0.185204
11726197_SYN2	-0.07839	0.108892	0.185224
11745568_NPAS3	-0.09295	0.108904	0.185238
11722373_GGA2	0.07723	0.108927	0.185271
11752713_CEP128	-0.30122	0.10897	0.185338
11756758_SPECC1	0.091137	0.108979	0.18534
11736851_TMEM107	-0.17852	0.108981	0.18534
11738539_DISC1 ///	0.16009	0.108983	0.18534
11741361_ZNF26	0.099148	0.109009	0.185379
11752761_EMSY	-0.09969	0.10904	0.185425
11739294_SLC29A3	0.065725	0.109051	0.185438
11740555_POU5F2	-0.09954	0.109067	0.185453
11730976_NSUN7	0.082807	0.109068	0.185453
11728596_FAM180A	-0.09309	0.109086	0.185475
11730137_NXF2 ///	-0.15387	0.109088	0.185475
11763936_KRT17P5	-0.14641	0.109114	0.185512
11716663_GDF15	-0.07243	0.109147	0.185561
11740590_RPS24	0.090222	0.10918	0.185612
11731512_DYRK4	0.094908	0.109225	0.185681
11728940_KCNIP1	-0.14946	0.109244	0.185703
11725855_XAGE1B //	0.061686	0.109245	0.185703
11730006_C19orf18	-0.10631	0.109251	0.185706
11762628_CTCFL	0.079389	0.109277	0.185744
11751565_RASSF3	-0.09121	0.109282	0.185746
11763815_ZNF551	0.156291	0.10931	0.185788
11725938_DIRAS2	-0.10252	0.10934	0.185831
11722200_ADAM12	-0.13586	0.109365	0.185868
11736855_ATP6V1C2	-0.11402	0.109407	0.185932
11733341_CLEC4M	-0.09058	0.109411	0.185933
11753883_IL2RG	0.074416	0.109419	0.185941
11745787_MUC12	0.115803	0.10945	0.185986
11719562_SOX4	0.09501	0.109478	0.186028
11752057_VPS35	-0.1082	0.10949	0.186036

11736500_TMEM38A	-0.14655	0.109491	0.186036
11744680_SLC7A11	0.076923	0.109552	0.186134
11736251_MUC21	0.130178	0.109592	0.186195
11721330_RNGTT	0.111704	0.109622	0.186239
11756198_SEC11A	-0.06161	0.10964	0.186264
11715247_DEPDC5	-0.06747	0.109645	0.186266
11744716_PFN2	-0.06677	0.10966	0.186285
11755367_DNAAF3	-0.07687	0.109674	0.186302
11718145_DGCR14	-0.06366	0.109688	0.186319
11720552_IFFO1	-0.10673	0.109697	0.186326
11715319_MAGEA1	-0.07224	0.1097	0.186326
11756636_PCED1A	0.095957	0.10974	0.186388
11759909_GATS	-0.09695	0.109767	0.186427
11732229_NEUROD6	-0.11522	0.109789	0.186458
11741707_PCDHGC3	-0.1158	0.109826	0.186515
11737083_ZNF490 //	0.113109	0.109846	0.186542
11754220_PWP1	0.066563	0.109873	0.186581
11762236_LOC100287	-0.13546	0.109943	0.186689
11757136_SNORA2C	-0.10355	0.109944	0.186689
11720455_CAPRIN1	0.061456	0.109948	0.18669
11716749_CD99L2	-0.08249	0.109979	0.186735
11726021_EEPD1	-0.12233	0.109991	0.186746
11747832_FBXO21	-0.06322	0.109994	0.186746
11739617_CD44	0.072451	0.109997	0.186746
11730326_LOC105375	-0.10363	0.110007	0.186758
11748383_PAPD7	-0.1088	0.11002	0.186773
11725720_CDC42	-0.06719	0.11003	0.186783
11760372_PRKACA	-0.07079	0.110044	0.1868
11758917_TMEM204	0.093041	0.110071	0.186839
11752539_PSG1	-0.09695	0.110121	0.186919
11745519_ABI1	0.097975	0.110143	0.186949
11744271_ULK1	-0.09119	0.110154	0.186962
11746627_RIC3	-0.1605	0.110163	0.186969
11730722_NCAM1	0.110505	0.110175	0.186984
11762915_ESAM	-0.12555	0.11019	0.187002
11733723_HMP19	0.111861	0.110222	0.187051
11754623_CHPT1	-0.06482	0.110237	0.187067
11716271_POLR2E	-0.13109	0.110239	0.187067
11752576_ANKLE1	-0.09472	0.110248	0.187075
200013_PMRPL24	-0.05983	0.11029	0.187134
11753542_FLT4	-0.14983	0.110291	0.187134
11746245_IL17RC	-0.07631	0.110294	0.187134
11762648_CGB /// C	-0.09951	0.110307	0.18715
11759877_MYO5C	-0.16549	0.110325	0.187174
11724864_GJB1	-0.18912	0.110373	0.187249
11761805_CALCB	-0.09547	0.110383	0.18726
11746702_NKAIN3	-0.09116	0.110443	0.187354
11732546_DEFA4	-0.1357	0.110478	0.187408
11750956_GABRR1	-0.10672	0.110526	0.187482
11748216_STAU2	-0.06699	0.110536	0.187491
11718732_MAPT	-0.08565	0.110539	0.187491

11762305_ERG	-0.14828	0.110561	0.187522
11760420_GALNT16	-0.11448	0.110607	0.187589
11727419_CNBP	0.074016	0.110608	0.187589
11756515_EXOSC1	-0.07155	0.110612	0.18759
11754323_PIGT	-0.07022	0.110648	0.187644
11740549_ALOX12B	-0.12183	0.110657	0.187653
11752894_CCDC33	-0.09473	0.110676	0.187675
11751881_CACNA1E	0.15843	0.110678	0.187675
11754971_TARBP1	-0.06786	0.110708	0.187719
11753824_FXYD1	0.119138	0.110747	0.187779
11741729_LRTOMT	-0.15491	0.11076	0.187794
11723996_GLCCI1	-0.07108	0.110776	0.187815
11733993_TMEM150C	-0.19078	0.110783	0.187821
11730315_RGS7BP	0.147924	0.110814	0.187864
11758730_DUSP1	0.104607	0.110817	0.187864
11732864_TAS1R1	-0.09907	0.11083	0.187876
11729587_KLK2	-0.08167	0.110831	0.187876
11757094_LRRD1	0.122357	0.110865	0.187927
11733609_TRIM73 //	-0.0781	0.110885	0.187954
11722974_WDR90	-0.07235	0.110904	0.18798
11737340_DBF4B	-0.09456	0.110956	0.188056
11747281_FAM65B	0.08268	0.110958	0.188056
11749017_CARD14	-0.14896	0.110961	0.188056
11748951_CTNNB1	-0.0742	0.110971	0.188068
11724719_DEXI	0.070604	0.110981	0.188075
11756371_VSIG4	-0.19821	0.110983	0.188075
11717649_SMG9	-0.08633	0.11103	0.188142
11738546_KRT26	0.11781	0.11103	0.188142
11752946_MAPRE2	0.086855	0.111082	0.188209
11716202_PFKM	-0.07953	0.111083	0.188209
200007_PMSRP14	0.065988	0.111085	0.188209
11733722_MAX	0.076916	0.111086	0.188209
11757393_SI	-0.10301	0.111089	0.188209
11720525_HPS4	-0.10882	0.111107	0.188232
11736836_TPCN2	-0.08851	0.111153	0.1883
11729667_NOL3	-0.06833	0.111154	0.1883
11762117_DBT	-0.1659	0.111158	0.1883
11741343_DMTN	-0.12821	0.111166	0.188306
11763011_IGKC	0.136449	0.111173	0.188311
11754772_NR4A1	-0.08379	0.111183	0.188322
11725878_DEFB119	-0.12292	0.111188	0.188324
11754239_DYDC2	-0.16132	0.111215	0.188363
11760167_STK25	-0.12139	0.111226	0.188375
11740966_LOC391322	-0.10562	0.111244	0.188399
11741178_UPB1	-0.11085	0.111264	0.188427
11726937_ST18	0.117444	0.11128	0.188447
11729646_TM4SF4	-0.18304	0.111297	0.188469
11715343_MUC16	-0.08299	0.111325	0.18851
11761084_RABGAP1L	0.166384	0.111351	0.188548
11730096_BCL2A1	0.10373	0.111366	0.188567
11744941_AQP7	-0.15338	0.111373	0.188569

11739245_ANKFY1	-0.08457	0.111375	0.188569
11762670_DBIL5P2	-0.13912	0.111394	0.188595
11755380_WDR92	-0.11055	0.111433	0.188654
11757782_RPL15 ///	0.060505	0.111459	0.188691
11736087_FAM171A1	-0.06818	0.111469	0.188703
11758427_ENTHD1	-0.08547	0.111534	0.188806
11737493_DGKK	-0.07048	0.111568	0.188852
11744811_C14orf159	-0.13663	0.111569	0.188852
11741359_RALGDS	0.164678	0.111576	0.188856
11734115_SLC10A1	-0.08485	0.111606	0.188901
11715213_DUX1 ///	-0.11052	0.111636	0.188945
11733820_CHRNA2	0.093881	0.111654	0.188969
11760337_MAPK12	-0.0922	0.11166	0.188973
11744496_PCAT4	0.140016	0.11168	0.189
11737939_RIPK1	-0.07663	0.111687	0.189005
11746358_MTA1	-0.06906	0.111715	0.189046
11746918_L2HGDH	0.082737	0.111769	0.189131
11743726_RHOA	0.063549	0.111776	0.189137
11717440_EPS8	0.06484	0.111799	0.189169
11742139_BTNL2	-0.13912	0.111858	0.189261
11730718_PFKFB4 //	-0.11959	0.111875	0.189284
11741559_ANXA13	0.123375	0.111888	0.1893
11744438_SRI	-0.08409	0.111923	0.189353
11750219_NUP43	-0.0732	0.111929	0.189357
11742560_OR6N2	-0.08306	0.111938	0.189365
11741779_HSD11B1	0.151593	0.111945	0.189371
11733865_RPP30	-0.09431	0.111968	0.189402
11749488_PCDHB6	0.070685	0.112017	0.189478
11721336_LDOC1	0.098809	0.112041	0.189513
11760300_PNISR	0.106652	0.112096	0.189599
11754458_ACRBP	-0.12019	0.112114	0.189623
11725406_HERC3 ///	-0.12475	0.112131	0.189645
11732001_PDE11A	0.189701	0.112139	0.189652
11722867_CDCP1	-0.12037	0.112147	0.18966
11737638_ATP8B2	-0.09319	0.112158	0.189672
11758503_ZNF529	-0.09739	0.112185	0.18971
11737887_NRL	-0.14243	0.112214	0.189753
11748613_FNBP1L	0.11258	0.112235	0.189777
11764257_LOC102723	-0.12746	0.112236	0.189777
11741958_IL6R	0.104684	0.112243	0.189783
11741983_ESR2	-0.08812	0.112264	0.189811
11754230_DBIL5P	-0.13478	0.112328	0.189899
11761029_SLC11A1	-0.15881	0.11233	0.189899
11725055_HNMT	0.11459	0.112331	0.189899
11732966_MIA	-0.06316	0.112333	0.189899
11732107_CYP1A2	0.186117	0.112335	0.189899
11731766_SETD3	-0.09787	0.11234	0.1899
11733517_KCNK10	-0.11663	0.112356	0.189921
11751309_DNAJA3	0.070066	0.112389	0.189964
11716829_ABCB6 ///	0.062836	0.11239	0.189964
11727467_IL10RB	-0.10913	0.112401	0.189977

11742957_SMARCA4	0.071661	0.112411	0.189987
11722468_CACNG4	-0.14885	0.112423	0.19
11732041_MMD2 ///	-0.11925	0.112429	0.190005
11755619_D2HGDH	-0.08433	0.112457	0.190045
11759007_HAMP	-0.14185	0.112476	0.190068
11753202_SERPINB3	-0.11695	0.112478	0.190068
11737100_SLC5A7	-0.16165	0.112506	0.190108
11749800_BAIAP2	-0.06992	0.112535	0.190151
11737457_USP29	0.129759	0.112552	0.190173
11761536_TM2D3	0.418548	0.1126	0.190247
11740908_PMS2P9	-0.06925	0.112625	0.190283
11721224_MPPED2	-0.28164	0.112636	0.190294
11729032_RPGRIP1	-0.15715	0.112643	0.190294
11743908_CTPS1	-0.07687	0.112643	0.190294
11722301_ETS1	0.121828	0.112648	0.190296
11755354_RFNG	0.07182	0.112664	0.190313
11750381_ZNF423	-0.10194	0.112666	0.190313
11741205_GABRA1	0.139707	0.112723	0.190391
11737088_TLL1	0.111077	0.112723	0.190391
11734646_TBL1Y	-0.08987	0.112724	0.190391
11750964_CHRFAM7A	-0.11468	0.112744	0.190418
11735224_KLRG1	0.0907	0.112748	0.190418
11750581_FBXO3	0.085861	0.112752	0.190419
11723961_PTN	0.105063	0.112774	0.190443
11748287_ENDOU	-0.1315	0.112774	0.190443
11721328_RNGTT	-0.11217	0.112787	0.190455
11755012_CDC42SE2	0.07656	0.112789	0.190455
11759021_LNX1	-0.07175	0.112806	0.190465
11738265_SHH	-0.09722	0.112807	0.190465
11737413_MICALCL	-0.16446	0.112807	0.190465
11751680_UQCRB	0.084515	0.112816	0.190474
11757023_RPS12 ///	0.066119	0.112827	0.190486
11749603_HPN-AS1	-0.0922	0.112862	0.190539
11735668_SLC34A3	-0.07488	0.112885	0.190571
11759629_TRAC ///	-0.09595	0.11291	0.190607
11744806_PPY2P	-0.09488	0.112947	0.190662
11738635_SLCO6A1	-0.09404	0.113011	0.190764
11728869_ELAVL3	0.144044	0.113022	0.190775
11724467_KRT12	-0.14536	0.113058	0.19083
11746348_PNN	0.069823	0.113084	0.190868
11755943_VIM	-0.05867	0.113096	0.190881
11753536_LOC105372	-0.11387	0.113122	0.190918
11726383_OLFM1	-0.09479	0.113157	0.190971
11752191_SALL2	-0.10322	0.11317	0.190987
11718754_CAPN5	-0.12656	0.113228	0.191077
11735998_APOL1	-0.07731	0.113255	0.191116
11730832_RANBP10	0.080751	0.113279	0.19115
11734500_PABPC1L2B	-0.093	0.113327	0.191225
11747633_GLS2	-0.0822	0.113332	0.191226
11717970_FAM20B	0.061341	0.11335	0.191251
11722741_DDC	-0.07296	0.113402	0.191331

11758146_GORASP1	0.080781	0.113412	0.191332
11751126_ABCC6 ///	-0.07352	0.113413	0.191332
11747065_NUP88	0.067486	0.113414	0.191332
11760662_HLA-DOA	0.077477	0.11344	0.191368
11745276_SOAT1	0.078034	0.113446	0.191372
11744933_ASXL1	0.077511	0.11345	0.191372
AFFX-HSACACTB	0.058499	0.113456	0.191372
11739886_DSG1	-0.11884	0.113457	0.191372
11754938_CDK5	-0.1194	0.113469	0.191385
11744086_PRR15L	-0.10828	0.113505	0.191439
11742458_C2orf81	-0.11591	0.113509	0.191439
11731130_SPANXC //	0.118797	0.113575	0.191544
11727139_MYH7B	0.09295	0.11359	0.191556
11762805_UCKL1	-0.08104	0.113591	0.191556
11718864_SEC63	-0.07415	0.113594	0.191556
11733594_SLC26A1	-0.12435	0.113604	0.191567
11717135_PCSK1N	0.101884	0.113612	0.191573
11740307_SLC5A12	0.205449	0.113617	0.191575
11744031_MMP7	0.108382	0.113629	0.191589
11730683_TSHR	-0.14335	0.113637	0.191596
11733610_C9orf173	-0.14438	0.113644	0.1916
11736399_RFT1	-0.1054	0.113655	0.191613
11743596_PTPRE	0.074907	0.113692	0.191669
11733749_TNNI2	-0.16471	0.113702	0.191679
11761347_CLCN2	-0.14858	0.113715	0.191694
11722045_SLC52A3	-0.11009	0.113735	0.191717
11748168_FAM227A	-0.13925	0.113737	0.191717
11755884_SLC25A43	0.07442	0.11376	0.191749
11732424_ANKRD22	-0.16117	0.11378	0.191777
11746104_PLEKHM1 /	0.081944	0.113804	0.191809
11751705_NOTCH4	-0.1872	0.113807	0.191809
11750595_KIF5A	-0.12988	0.113827	0.191837
11761410_CD7	0.078006	0.113861	0.191886
11736650_CD5L	-0.12922	0.113872	0.191894
11739068_KIFAP3	-0.06939	0.113875	0.191894
11735150_NXPE3	-0.10322	0.113877	0.191894
11737729_ZNF705A /	-0.16799	0.113902	0.191929
11741626_MALT1	0.092492	0.113905	0.191929
11750310_SLC17A3	-0.08352	0.11394	0.19198
11747993_CALCRL	0.156892	0.113947	0.191983
11733713_DMP1	-0.21662	0.113951	0.191983
11761057_PDPK1	0.096177	0.113953	0.191983
11757872_PLSCR1	-0.16037	0.11396	0.191988
11734374_CPXCR1	-0.17504	0.113985	0.192024
11735656_ATOH7	-0.09647	0.114005	0.192051
11761461_FAM73B	0.094823	0.114021	0.19207
11760573_FOSL1	0.080474	0.114028	0.192075
11754698_PLAC8L1	-0.14226	0.114041	0.192091
11737656_LCE5A	-0.12001	0.114047	0.192095
11738486_DNASE1L2	-0.07763	0.114056	0.192099
11717525_RDX	0.059241	0.114057	0.192099

11756234_DTNB	-0.0671	0.114083	0.192132
11752711_TNR	0.130454	0.114085	0.192132
11746545_DAPK1	0.128153	0.114107	0.192162
11740370_RASGRP1	0.125783	0.114115	0.19217
11735255_RNF122	-0.13712	0.114142	0.192209
11749855_SH2B1	-0.11971	0.114179	0.192263
11746380_PHLDB1	0.171757	0.114183	0.192264
11759972_SYT9	-0.11672	0.114205	0.192291
11721363_IREB2	-0.0656	0.114207	0.192291
11722968_TRPV4	-0.10398	0.114295	0.192433
11734104_PNMAL2	-0.14505	0.114299	0.192433
11753909_BANP	0.066681	0.114305	0.192437
11763749_MLPH	-0.08274	0.11433	0.192459
11730678_NOXRED1	0.109674	0.114331	0.192459
11733747_MCHR2	0.111648	0.114331	0.192459
11735540_WEE2	-0.13873	0.114334	0.192459
11762221_ZNF333	-0.0816	0.114355	0.192487
11748739_NT5C1B	-0.08566	0.114401	0.192558
11719876_CKMT2	-0.08928	0.114408	0.192563
11718872_DHRS1	0.082304	0.114415	0.192568
11724618_MOCS1	-0.11116	0.114442	0.192606
11721047_SEPT8	-0.15812	0.114453	0.192615
11730806_BTN3A2	-0.06793	0.114455	0.192615
11760532_SLC34A1	-0.07918	0.114488	0.192665
11717526_RDX	0.060313	0.114506	0.192688
11733833_MAP2	-0.06672	0.11451	0.192688
11739742_B3GNT6	-0.10928	0.114532	0.192719
11743427_AHCYL1	-0.07148	0.114541	0.192728
11754557_PRKCA	0.080723	0.114556	0.192745
11762379_NOS1	-0.17611	0.114559	0.192745
11730079_SHF	-0.14273	0.114628	0.192853
11729433_BEND4	-0.17027	0.11464	0.192867
11733515_DGKB	-0.07255	0.114673	0.192916
11731117_PLEKHA8P1	-0.09159	0.114709	0.192971
11722536_CCNYL1	0.130209	0.11474	0.193016
11761962_KIF25-AS1	0.118057	0.114761	0.193045
11736593_LDB3	-0.10939	0.114783	0.193076
11751015_GLYATL1 /	-0.1352	0.114807	0.193108
11725684_PTPRO	0.124062	0.114824	0.19313
11719426_CHST14	-0.0627	0.114864	0.193191
11736301_HM13	0.084301	0.114914	0.193268
11740340_F2RL3	0.103105	0.114925	0.193281
11763584_MAP2K5	0.136793	0.11494	0.1933
11727527_TNNT2	-0.14404	0.114945	0.193301
11720924_TCAP	-0.07863	0.114961	0.19332
11760016_GALNT1	-0.19192	0.115006	0.19339
11755101_CEP76	-0.0761	0.115016	0.193399
11736442_SPINK7	0.15557	0.115019	0.193399
11726826_VPS37D	-0.07314	0.115031	0.193413
11753426_SLC25A45	-0.08877	0.115086	0.193498
11729463_H1FNT	-0.11515	0.115136	0.193575

11723700_OAS3	0.078121	0.115147	0.193588
11730930_IKBKB	-0.08982	0.115156	0.193593
11758257_SLC19A1	-0.0639	0.115159	0.193593
11755421_LYSMD4	0.068335	0.115164	0.193596
11763115_IGHG1	0.33959	0.115204	0.193657
11763891_SNORA78	-0.12963	0.11521	0.19366
11756151_B2M	0.067517	0.11522	0.19367
11753215_CCDC88C	-0.08766	0.115258	0.193727
11733763_ADAMTSL1	0.079824	0.115269	0.193738
11753201_SERPINB3	0.072592	0.115288	0.193764
11752345_CADM2	0.071059	0.115296	0.193771
11729543_MAGEC1	-0.13089	0.115342	0.193841
11750979_ADGRG3	-0.10251	0.115352	0.193853
11749877_MKNK2	-0.10462	0.115367	0.19387
11762966_OBP2B	-0.1154	0.115422	0.193956
11758954_ENPP5	0.131392	0.115477	0.194042
11739132_NOL10	-0.0752	0.115482	0.194043
11727902_EPB41L2	0.11212	0.115507	0.194079
11734934_FGD2	-0.15547	0.115542	0.194132
11742949_CNOT8	-0.08896	0.115582	0.194191
11752544_GPNMB	-0.05799	0.115616	0.194243
11723428_RPL13 ///	0.058989	0.115673	0.194331
11752772_KLHL3	-0.13103	0.115678	0.194334
11728062_SH3BP1	-0.07515	0.115698	0.194359
11763447_TRDC	-0.1215	0.115721	0.194386
11751617_ODF2L	0.070789	0.115722	0.194386
11749933_KIF26B	-0.13849	0.115734	0.1944
11747789_ZNF394	0.077017	0.115774	0.194461
11720559_FMO1	-0.11102	0.115781	0.194463
11722247_RASSF1	-0.08522	0.115783	0.194463
11726090_VPS53	-0.08063	0.115795	0.194475
11753322_DPH5	-0.09493	0.115824	0.194519
11757925_ZSCAN12	0.091826	0.115854	0.194562
11715190_FAM221A	-0.14236	0.115867	0.194577
11759058_MED6	-0.07727	0.115888	0.194606
11763550_IGK /// I	-0.21308	0.115914	0.194639
11753467_AMT	-0.13247	0.115917	0.194639
11733296_CYP4F2 //	0.092731	0.11592	0.194639
11760046_LOC338797	-0.09836	0.115944	0.194655
11744954_ANXA1	0.059817	0.115944	0.194655
11732262_FSCN3	-0.10675	0.115945	0.194655
11737263_HRK	-0.1711	0.115947	0.194655
11749080_ARSA	-0.1215	0.115949	0.194655
11733062_GABRA6	-0.14739	0.115962	0.19467
11727746_WDR93	-0.10167	0.115977	0.194682
11743646_C17orf105	-0.15971	0.115977	0.194682
11751108_MRC2	-0.09747	0.115986	0.19469
11753768_ZBTB4	-0.08249	0.115991	0.194691
11718202_BTD	-0.07496	0.115998	0.194697
11728149_NPM2	-0.07503	0.116017	0.194722
11763448_LINC01193	-0.14527	0.116056	0.19478

11732297_KIAA1715	-0.07488	0.11607	0.194797
11744814_PCM1	0.075085	0.116107	0.194853
11732452_MS4A3	0.137614	0.116161	0.194936
11748343_IFT22	0.073941	0.116176	0.194956
11736990_LPAL2	0.091793	0.116183	0.19496
11716401_SELENBP1	-0.09945	0.116188	0.194962
11743779_HERC2	-0.07743	0.116199	0.194973
11734581_CBLN2	-0.13538	0.116246	0.195046
11759415_SLC51B	-0.1243	0.116269	0.195077
11734152_ABRA	-0.10692	0.116289	0.195102
11737734_CAPS2	0.120238	0.116292	0.195102
11755431_ANKRD34C	-0.0872	0.116315	0.195134
11737259_FRMPD4	0.144744	0.116335	0.19516
11729652_CYP11A1	-0.14303	0.116338	0.19516
11739966_GPR39	-0.07252	0.11635	0.195173
11759400_PSMF1	0.098033	0.116401	0.195251
11726565_COL17A1	0.083949	0.116423	0.195282
11715529_ATP6V1G2-	-0.07137	0.11646	0.195338
11758003_MUC20	0.157419	0.116473	0.195352
11745477_TTYH1	-0.1061	0.116488	0.195371
11737584_ALX4	-0.09452	0.11654	0.195451
11751156_CABLES1	0.137993	0.116561	0.195481
11752907_DAOA	0.14435	0.116592	0.195525
11731001_AP5B1	0.100516	0.116601	0.19553
11739203_PLA2G6	-0.08485	0.116603	0.19553
11736094_DCTD	0.073472	0.116667	0.195626
11759474_NT5C1A	-0.1059	0.116668	0.195626
11737717_SLC26A1	-0.1466	0.116677	0.195634
11756302_CD37	-0.10028	0.116763	0.195772
11748844_BCCIP	-0.09271	0.116773	0.195778
11745016_C1orf43	0.066765	0.116774	0.195778
11730626_KLB	-0.11544	0.116783	0.195786
11753499_KRTAP1-1	-0.20139	0.116816	0.195834
11749982_PIP4K2B	0.070748	0.116828	0.195847
11749083_CRCP	0.068594	0.116837	0.195856
11719767_TPPP3	-0.17278	0.116894	0.195945
11727541_MPDZ	0.065586	0.1169	0.195948
11749850_RAB37	0.086913	0.11697	0.196058
11754578_C1orf101	-0.06913	0.116983	0.196073
11761383_SPATA9	-0.08566	0.117037	0.196158
11747275_SLC8B1	-0.19956	0.117075	0.196214
11730223_PNPLA2	-0.10174	0.117086	0.196223
11752881_RHCE ///	-0.19959	0.117088	0.196223
11743730_TNFSF10	0.116015	0.117133	0.196291
11735310_GIPC1	0.062537	0.117141	0.196298
11757255_FKSG29	-0.13504	0.117159	0.196322
11752143_ABCB11	-0.13899	0.117171	0.196334
11749734_JRKL	-0.11314	0.117174	0.196334
11750243_PPARGC1A	-0.20295	0.117188	0.196349
11728683_KRR1	0.066696	0.117201	0.196366
11728016_TRIM66	-0.11058	0.117211	0.196375

11758460_FAAH2	-0.11294	0.117215	0.196376
11736572_TIGD6	-0.09073	0.117226	0.196386
11742389_OR8G1	-0.08087	0.117262	0.19644
11734643_PLA2G4F	-0.07977	0.117283	0.196465
11749679_ELN	-0.10231	0.117285	0.196465
11737711_SAP30L	-0.08682	0.117293	0.196472
11738565_TMEM14EP	-0.08995	0.1173	0.196478
11736012_MGAT2	0.092482	0.117354	0.19656
11715376_RPS11	0.06912	0.117358	0.196561
11728158_EDA	-0.10304	0.117379	0.196579
11731908_C19orf71	-0.11831	0.11738	0.196579
11716052_LAMC1	0.066366	0.117381	0.196579
11716396_WBP1L	0.065725	0.117407	0.196616
11722282_LAMA4	-0.08833	0.117427	0.196643
11741684_RABL6	-0.09516	0.117446	0.196668
11730796_PSPH	-0.07796	0.117452	0.196671
11721258_KRT5	-0.08269	0.117458	0.196673
11719405_HIPK2	0.096267	0.117462	0.196673
11753298_BIRC7	-0.11281	0.117473	0.196685
11752158_MYBPC3	-0.12465	0.117514	0.196748
11749071_FAM172A	-0.08909	0.117524	0.196758
11756855_GCNT2	0.205911	0.117533	0.196764
11746456_GPR155	-0.14997	0.117536	0.196764
11735742_TEX101	-0.15517	0.117601	0.196866
11726381_TMEM41B	0.113189	0.117606	0.196868
11762057_MTHFD2L	-0.23472	0.117617	0.19688
11756422_RAPGEF1	0.081388	0.117667	0.196951
11721163_KHNYN	0.07686	0.117668	0.196951
11745502_GALNT9	-0.08859	0.117684	0.196971
11746667_BHMT2	-0.1244	0.117737	0.197048
11723182_CNOT6L	0.117373	0.117738	0.197048
11738463_FLJ33360	-0.08469	0.117774	0.197102
11759081_TCEB1	-0.21128	0.117796	0.197132
11758161_MAGEH1	-0.17128	0.11781	0.197145
11756562_PDCD10	-0.11046	0.117812	0.197145
11761476_ERG	-0.12212	0.117852	0.197205
11758497_NANP	0.131865	0.11786	0.197212
11733640_C6orf10	-0.1565	0.117902	0.197276
11751339_PSG9	0.077132	0.117977	0.197395
11763976_MRPS11	0.111634	0.117999	0.197425
11730588_GATA5	-0.12179	0.118012	0.19744
11728367_AARSD1 //	0.068288	0.118053	0.197501
11747611_TMCO6	-0.07652	0.118064	0.197513
11762701_WNT8A	0.105352	0.118101	0.197568
11757776_ARG2	-0.07596	0.118148	0.19764
11736034_EXOSC2	-0.07814	0.118183	0.197692
11734257_PRKAG3	-0.09765	0.118224	0.197753
11761698_TEKT1	0.134473	0.118245	0.197774
11727072_ENPP6	0.131558	0.118245	0.197774
11751012_RIPK3	-0.11884	0.118249	0.197774
11761878_PARD3B	0.151216	0.118254	0.197775

11738603_LCE3C	-0.09949	0.118257	0.197775
11736826_DHH	-0.09339	0.118303	0.197845
11752244_KIRREL3	-0.08644	0.118309	0.197849
11721251_MFAP1	-0.05958	0.118318	0.197857
11754776_CCDC74A	-0.07405	0.118323	0.197859
11742509_OR4C15	-0.09269	0.118333	0.197865
11729907_METTL15	0.078755	0.118335	0.197865
11732411_GPER1	-0.10109	0.118354	0.19789
11738051_MAGEB1	-0.08756	0.118366	0.197904
11757446_MINOS1 //	0.064045	0.118397	0.197948
11722054_CYP4X1	0.078965	0.118412	0.197966
11759120_LGR5	-0.14164	0.118433	0.197994
11725940_DIRAS2	-0.11563	0.11844	0.198
11737462_EPHA10	-0.13593	0.118449	0.198007
11718815_PVRL1	-0.181	0.11846	0.198019
11733639_CHM	0.101524	0.11851	0.19809
11740926_SATL1	0.101171	0.11851	0.19809
11739422_LOC102724	-0.11628	0.118554	0.19815
11729779_CDH17	-0.19109	0.118554	0.19815
11761079_PIGH	-0.07013	0.118581	0.198182
11736392_IRGC	-0.07762	0.118585	0.198182
11721975_TPSAB1 //	0.091708	0.118586	0.198182
11748769_POLL	-0.06146	0.11859	0.198182
11715489_PGRMC1	-0.08446	0.118613	0.198214
11735187_F9	-0.16136	0.118627	0.19823
11755549_SEMA6D	-0.09775	0.118649	0.198256
11735949_GPR142	-0.2511	0.11865	0.198256
11749239_ZNF672	-0.07822	0.11868	0.198295
11733485_CACNA1C	-0.08598	0.118682	0.198295
11746286_BTN3A1 //	-0.09109	0.118689	0.198301
11762909_LOC100127	-0.09587	0.118701	0.198313
11735049_PCDHB3	-0.21308	0.11871	0.198321
11719097_TAF10	0.065504	0.118715	0.198324
11749885_ACADS	-0.11644	0.118731	0.198344
11752553_FBXW12	-0.12071	0.118768	0.198397
11753465_CMTM5	-0.13096	0.118771	0.198397
11742911_IGSF6	0.121976	0.118803	0.198444
11733699_TEAD4	0.067749	0.118842	0.198496
11729849_MLLT10	0.108648	0.118843	0.198496
11762149_TMEM241	-0.08313	0.118879	0.198549
11762026_LRBA	-0.06271	0.118912	0.198598
11719883_APOLD1	0.139299	0.118957	0.198661
11738485_TBPL2	-0.12986	0.118959	0.198661
11725307_CALU	0.079441	0.118962	0.198661
11723948_SLC3A1	0.169602	0.118975	0.198677
11763948_CDRT15P1	-0.13237	0.119024	0.198751
11760135_LRCH3	0.074559	0.119034	0.198761
11728205_FAM120A	-0.06738	0.119044	0.198771
11732912_ODF4	-0.13992	0.119083	0.198829
11760904_FARP2	-0.07278	0.119091	0.198837
11744527_RNFT2	-0.07711	0.119101	0.198845

11719810_RRS1	0.057803	0.119146	0.198914
11760933_HSD17B7P2	-0.20276	0.119157	0.198926
11725437_SPRYD7	-0.08788	0.119199	0.19899
11724935_THAP7	-0.07645	0.11921	0.199001
11725487_ATG14	0.084527	0.119216	0.199004
11721034_PPP1R3B	0.112859	0.119271	0.199089
11737542_GOT1L1	0.120393	0.119335	0.199189
11759275_OR2M3	-0.14585	0.119375	0.199249
11761927_DPY19L3	0.19367	0.119381	0.199252
11730556_TRIM34 //	0.103024	0.119399	0.199275
11716847_SLC43A3	-0.06604	0.11942	0.199304
11746678_RBM11	-0.13005	0.11943	0.199313
11725547_OPCML	-0.1464	0.119478	0.199386
11718532_SIK3	-0.08718	0.119495	0.199408
11744437_ADH6	-0.1211	0.119504	0.199417
11720363_PHIP	0.065961	0.119509	0.199419
11747139_RASA1	0.074943	0.119532	0.19945
11737960_PDS5A	0.076596	0.119552	0.199477
11744613_SLC6A13	-0.12235	0.11965	0.199628
11716969_NRP2	0.072856	0.119652	0.199628
11762110_SH3GL2	-0.11903	0.119655	0.199628
11742628_OR2T6	-0.08963	0.119672	0.19965
11741598_SMR3A	-0.07521	0.119703	0.199694
11735372_GSG1L	-0.1297	0.119763	0.199788
11737460_ADARB2	-0.15022	0.119771	0.19979
11735740_RASA3	0.06841	0.119772	0.19979
11762748_TRA2A	-0.06116	0.119818	0.19986
11736912_CCDC33	0.067384	0.119839	0.199884
11753063_SIDT2	0.074637	0.119841	0.199884
11727502_ALDH3B2	-0.13567	0.119864	0.199915
11756767_PIFO	-0.165	0.119871	0.19992
11730747_PAIP2	0.071951	0.119883	0.199932
11727297_TGFB3	-0.1152	0.11989	0.199938
11749618_LILRA2	0.14413	0.119909	0.199962
11738196_SLC13A4	-0.11302	0.119926	0.199985
11750068_ZNF138	0.164158	0.119934	0.199991
11741000_GAP43	0.110103	0.119966	0.200034
11761672_SLC16A5	-0.13138	0.119968	0.200034
11761474_ULBP3	0.122151	0.119979	0.200045
11761297_TSPAN16	-0.10981	0.119991	0.200058
11737777_TNFSF8	-0.11213	0.120002	0.200067
11756484_UVSSA	-0.07538	0.120004	0.200067
11723932_PNMA3	-0.12716	0.120026	0.200097
11739170_G2E3	-0.06181	0.120057	0.200131
11747682_RAPGEFL1	-0.10796	0.12006	0.200131
11741066_SUMF2	0.073572	0.120065	0.200131
11726973_GH1	0.065921	0.120065	0.200131
11737097_DSC2	0.164255	0.120067	0.200131
11734187_GP6	-0.09792	0.120079	0.200143
11742908_SLF1	-0.07644	0.120099	0.20017
11740011_ZFP14	0.154638	0.120132	0.200207

11741397_DNAI2	-0.16287	0.120133	0.200207
11729957_KNG1	-0.06865	0.120133	0.200207
11760296_NTMT1	0.07527	0.120153	0.200233
11737962_AUTS2	0.105442	0.120186	0.200281
11732368_FGF14	-0.09287	0.120207	0.200309
11734302_MBL2	0.157561	0.120239	0.200357
11720953_PDCD5	-0.08023	0.120286	0.200428
11717102_SLC38A1	-0.10451	0.120301	0.200444
11739257_FAM63A	0.060943	0.120304	0.200444
11722393_TMEM126B	0.063208	0.120318	0.200461
11743411_RBM25	0.060215	0.120323	0.200462
11718205_TOE1	-0.06534	0.120345	0.200491
11741810_BIN1	0.113179	0.120356	0.200504
11742117_LYSMD1	-0.10311	0.120387	0.200549
11755813_SLC26A9	-0.13624	0.120429	0.20061
11762510_ABCB9	-0.12963	0.12048	0.200689
11747432_ADAM12	0.114908	0.12049	0.200698
11741809_HIST1H3J	-0.07632	0.120502	0.200713
11716533_PPT1	0.063692	0.120582	0.200838
11756378_RUVBL2	-0.06066	0.120598	0.200859
11756752_OSBPL5	-0.11278	0.120614	0.200877
11737094_SLC6A20	-0.20709	0.120639	0.200913
11742661_SCT	-0.0979	0.120647	0.200919
11719652_SPAG6	-0.06558	0.12066	0.200935
11730116_CACNA2D2	0.114773	0.120671	0.200946
11725824_ENTPD3	-0.07735	0.120678	0.20095
11719559_SOX4	-0.13719	0.120699	0.200979
11730771_NAPG	0.101189	0.12072	0.201007
11751465_GRB14	-0.13126	0.120731	0.201013
11760105_RPL7L1	-0.17612	0.120732	0.201013
11736103_SPECC1L	-0.07027	0.120747	0.201031
11752935_INTS4 ///	-0.06017	0.120812	0.201132
11734606_CXCR3	-0.11295	0.120822	0.201143
11731227_MATK	-0.1516	0.120838	0.201155
11753331_CLCNKA //	-0.10725	0.120838	0.201155
11744064_MUC5B	-0.09767	0.120872	0.201205
11737835_CD58	-0.14947	0.120885	0.201214
11753441_HILS1	-0.20766	0.120886	0.201214
11732766_ALOXE3	0.120478	0.120896	0.201224
11760000_INVS	-0.13071	0.120917	0.201252
11750896_F2	-0.07361	0.120938	0.201281
11750030_SULT1C2	-0.08001	0.121007	0.201389
11749261_TBCE	-0.06127	0.12102	0.201398
11761822_STXBP4	0.085764	0.121021	0.201398
11726310_GLRB	0.10116	0.121043	0.201424
11749733_JRKL	-0.12714	0.121045	0.201424
11716313_MRPS21	-0.07237	0.121073	0.201463
11731275_CLEC4G	-0.13518	0.121086	0.201479
11749576_FA2H	-0.06876	0.121115	0.20152
11758750_YWHAZ	-0.07063	0.121165	0.201597
11755099_WWTR1	0.060014	0.121205	0.201656

11740520_SERPINA11	-0.14285	0.121217	0.201669
11751199_MKNK2	-0.0907	0.121255	0.201725
11754310_HDHD3	-0.08102	0.121276	0.201753
11748932_CCDC150	-0.14381	0.121298	0.201783
11724787_KIF3A	0.104068	0.121324	0.20182
11730675_C17orf50	-0.09152	0.121333	0.201829
11747397_CCHCR1	-0.11879	0.121339	0.201832
11718919_ATP6V1B1	-0.14609	0.121388	0.201905
11727611_SCN2B	0.082676	0.121428	0.201966
11739962_B3GAT2	-0.16603	0.121449	0.20198
11743642_TFAP2A	0.099198	0.121449	0.20198
11749845_TBC1D22A	-0.09388	0.121449	0.20198
11735905_PNPLA1	-0.0751	0.121476	0.202018
11747031_DMPK	0.084185	0.121508	0.202058
11746729_LRMP	0.098776	0.121511	0.202058
11763199_PCBP2	0.101445	0.121512	0.202058
11744192_CASKIN1	-0.09465	0.12154	0.202097
11737538_NETO1	-0.13339	0.121558	0.202119
11763023_OR5G5P	-0.10412	0.12157	0.202132
11756333_CROCC	0.091101	0.121582	0.202146
11756710_MPP3	-0.0754	0.121595	0.202157
11759716_NPIPA5 //	0.107839	0.121597	0.202157
11757764_MSMO1	-0.07851	0.121611	0.202173
11728878_ARID1B	-0.12095	0.121621	0.202183
11759616_VASH1	-0.08589	0.121644	0.202215
11743233_C16orf96	-0.08656	0.121678	0.202264
11745697_CHN2 ///	-0.09597	0.121691	0.202278
11758153_FAM83H	-0.07258	0.121781	0.202422
11726575_CYP2A6 //	-0.07881	0.121793	0.202428
11739493_MGAT4A	-0.18349	0.121793	0.202428
11724923_PPP1R9A	-0.07694	0.1218	0.202432
11729065_SPPL2C	0.082147	0.121832	0.202479
11753751_SH3TC1	0.088162	0.121843	0.20248
11753727_RPL28	0.057356	0.121844	0.20248
11750569_IARS	0.058808	0.121845	0.20248
11734224_CTCFL	-0.12263	0.121855	0.202488
11750018_HIST2H2BF	0.098502	0.121858	0.202488
11738217_RELL1	0.136224	0.121873	0.202505
11759089_P2RX6	-0.10768	0.121902	0.202546
11737373_TCP11L2	-0.06415	0.121914	0.20256
11758880_RPS15A	-0.08745	0.12195	0.202614
11753395_CDKN2B-AS	-0.09373	0.121959	0.202621
11737842_SNX29P1 /	-0.07822	0.121994	0.202672
11722467_PAX6	-0.11199	0.122019	0.202707
11749203_CLEC4M	0.101632	0.122097	0.20283
11735185_F9	-0.10931	0.122138	0.20289
11736400_DTX3	0.1119	0.122145	0.202896
11728063_SH3BP1	-0.06773	0.122156	0.202906
11728252_NMNAT2	0.081028	0.122173	0.202928
11723079_C2orf47 /	-0.09619	0.12219	0.202949
11716498_ADRM1	-0.06924	0.122209	0.202974

11719669_SMYD5	0.08603	0.122225	0.202994
11720789_ASF1A	0.059659	0.122246	0.203022
11756487_TSN	-0.06322	0.122285	0.203079
11751727_TYW3	0.064161	0.122307	0.203104
11743702_THUMPD3	-0.07835	0.122308	0.203104
11760869_ACOT8	-0.09086	0.122342	0.203154
11748480_TTYH2	-0.09489	0.122408	0.203237
11729054_B3GNT8	-0.06422	0.122409	0.203237
11758680_UCHL1	0.115508	0.122414	0.203237
11734503_ALOX15	-0.11071	0.122416	0.203237
11733288_CXorf36	-0.08511	0.122416	0.203237
11717875_PPFIA1	0.101135	0.122417	0.203237
11729960_DHFRL1	-0.10746	0.122425	0.203244
11754970_EXO5	-0.09444	0.122459	0.203292
11739295_ICOSLG //	-0.10753	0.122465	0.203297
11749830_IDH3A	-0.06124	0.122471	0.2033
11742894_DRG2	0.061003	0.122481	0.203309
11725807_SNCAIP	-0.11059	0.122498	0.20333
11754410_APLNR	-0.10249	0.122503	0.203332
11736529_KCND2	0.160498	0.122511	0.203338
11739688_FAM107B	0.083215	0.122525	0.203354
11732339_BCL11A	0.199579	0.122549	0.203387
11740252_CWC27	-0.0743	0.122575	0.203419
11732061_RPA4	0.234125	0.122582	0.203419
11722327_PGBD5	0.063977	0.122583	0.203419
11721197_DCHS1	-0.10489	0.122585	0.203419
11762916_IZUMO1	-0.08992	0.122615	0.20346
11749505_PLOD3	-0.08435	0.122618	0.20346
11739523_ST8SIA4	0.09181	0.122623	0.203462
11732548_MYO1A	-0.11517	0.122629	0.203465
11738964_ANP32D	-0.11911	0.122637	0.203465
11716411_RPS4Y1	-0.09697	0.122638	0.203465
11727245_LGI3	-0.06922	0.122675	0.203521
11745677_LRIT2	0.109092	0.122718	0.203584
11723434_TBC1D22A	-0.10302	0.122791	0.203699
11743703_THUMPD3	0.086165	0.122873	0.203821
11735195_ZFP28	0.129172	0.122877	0.203821
11727711_C1QTNF2	-0.11429	0.122877	0.203821
11754167_ZNF148	-0.09314	0.122912	0.203872
11755116_ABHD11	-0.07317	0.122918	0.203874
11756547_CLU	0.059572	0.122963	0.203942
11750563_TRPM1	-0.07492	0.122974	0.203954
11741828_OR2A1 ///	-0.13363	0.122984	0.203957
11737513_GALNTL6	0.082547	0.122985	0.203957
11761128_YME1L1	-0.11833	0.123006	0.203987
11761060_TMEM175	-0.15953	0.123014	0.203991
11756967_SUCLG2	0.085509	0.123017	0.203991
11763807_MAP2K5	-0.2162	0.123089	0.204102
11757308_PPP1CB	0.070764	0.123115	0.204139
11752186_SLC27A1	-0.08855	0.123184	0.204246
11744505_TP53I11	-0.1683	0.123201	0.204268

11752929_SIDT1	-0.07702	0.123293	0.204413
11715896_SCARB2	-0.06254	0.123307	0.20443
11750248_SNX19	0.122548	0.123312	0.204431
11732878_CNTLN	0.07926	0.123322	0.204441
11748537_LMOD3	-0.13216	0.123339	0.20446
11754448_TDRD5	-0.12386	0.123342	0.20446
11761782_IRF1	-0.10158	0.123382	0.204516
11741876_IL9R	-0.08342	0.123384	0.204516
11729946_STX1B	-0.09953	0.123396	0.204529
11717152_APMAP	-0.09461	0.1234	0.204529
11749520_GPR21 ///	0.091902	0.123428	0.204569
11738680_OR56B4	-0.08516	0.123486	0.204657
11763146_HSP90AB5F	-0.06502	0.123499	0.204671
11749302_SRSF2	-0.07662	0.123515	0.20469
11732346_ZNF33A	-0.05876	0.123518	0.20469
11756663_NSUN2	0.080682	0.123526	0.204696
11746126_STAG3 ///	0.076795	0.123565	0.204753
11735954_MRGPRD	-0.10808	0.123598	0.204802
11755214_TMTC1	-0.06143	0.123608	0.20481
11729422_POPDC3	-0.06247	0.123632	0.204843
11720487_HEBP2	-0.06976	0.123648	0.204862
11720811_PAMR1	-0.10742	0.123652	0.204862
11729663_CHEK1	-0.07994	0.123667	0.204881
11723618_SNAP91	-0.12677	0.12368	0.204896
11734102_ZNF578	-0.09455	0.123689	0.204902
11754881_SIRPG	-0.08067	0.123708	0.204928
11720213_PPAN	-0.0727	0.123733	0.204952
11752987_EPN2	0.091305	0.123734	0.204952
11745092_MYO16	0.093082	0.123735	0.204952
11763465_CABLES1	-0.09665	0.123743	0.204958
11749001_ZGRF1	-0.11876	0.123789	0.205025
11731872_CD209	-0.09793	0.123792	0.205025
11759593_UROD	-0.13734	0.123806	0.205041
11715214_MARK4	-0.11054	0.123864	0.20513
11722774_DIDO1	0.100867	0.123888	0.205163
11716215_E2F4	0.073948	0.123916	0.205202
11731920_RSG1	-0.13736	0.123966	0.205278
11727665_HAO2	-0.11234	0.123991	0.205313
11742076_PROX2	-0.06741	0.124	0.205321
11749508_PAPPA	-0.10234	0.124026	0.205351
11720885_ACACB	-0.13756	0.124029	0.205351
11756950_CELF6	-0.06076	0.124033	0.205351
11747043_ADAMTSL4-	-0.17158	0.124035	0.205351
11716761_PGC	0.11073	0.124065	0.205394
11737257_ANKS4B	0.08956	0.124079	0.20541
11741390_NSFL1C	-0.07526	0.124096	0.205431
11749540_DLD	0.059847	0.124105	0.205439
11728260_DIEXF	-0.07643	0.124126	0.205466
11731813_GATA1	-0.11032	0.124156	0.205509
11744330_DNAJC8	-0.06142	0.124163	0.205509
11758037_MTMR12	-0.08501	0.124164	0.205509

11724763_DEPTOR	-0.0775	0.124185	0.205537
11716945_TWF1	-0.09225	0.124198	0.205552
11737694_LOC391343	0.12723	0.124216	0.205572
11723990_ALG6	-0.11683	0.124219	0.205572
11743966_CERCAM	-0.06562	0.124254	0.205623
11732935_CT83	-0.08792	0.124271	0.205644
11728664_HSD3B2	-0.19389	0.124305	0.205693
11731783_RASAL3	-0.08158	0.124315	0.205702
11741807_CMPK1	0.070885	0.124337	0.205732
11756120_IKBKG	-0.08942	0.124356	0.205757
11725400_TTLL1	-0.11887	0.124361	0.205757
11730351_ANKRD52	-0.08411	0.124367	0.205757
11749076_SLAMF1	0.129246	0.124368	0.205757
11761150_GALK1	-0.09823	0.124381	0.205771
11752682_CHRDL2	-0.1286	0.124385	0.205771
11715591_DYNC1H1	0.078967	0.124411	0.205806
11724866_SCGB3A1	-0.05756	0.124418	0.205811
11746904_RGL2	0.067788	0.124427	0.205818
11738636_PCDHGA3	-0.08807	0.124435	0.205825
11733508_RLN1	0.11424	0.124446	0.205836
11749503_RELN	0.082863	0.124465	0.205861
11715219_HIST1H2AI	0.189168	0.124491	0.205896
11737404_GREB1	0.105264	0.124564	0.206011
11746228_AHNAK2	0.089938	0.124572	0.206017
11759233_PLA2R1	0.210889	0.124612	0.206071
11758568_SLF1	-0.08078	0.124613	0.206071
11719239_PANX1	0.082449	0.12462	0.206075
11734219_MARVELD2	-0.14766	0.124698	0.206198
11742297_FBXO24	-0.12196	0.124708	0.206206
11737158_SLC9A2	-0.16416	0.124715	0.206212
11726312_TBL1X	0.090785	0.124734	0.206233
11715871_UFD1L	0.142968	0.124737	0.206233
11756916_ABCG5	-0.0774	0.124753	0.206253
11750071_ATG2A	-0.07917	0.124765	0.206267
11716820_PRSS1	0.096544	0.124783	0.206284
11750113_EP400	-0.11086	0.124784	0.206284
11738159_COX6B2	-0.10994	0.124815	0.206328
11720633_FLNC	-0.07394	0.12484	0.206355
11757483_VWA1	-0.10894	0.12484	0.206355
11746780_RASSF6	0.156583	0.124861	0.206383
11760614_SKIV2L	-0.10062	0.124869	0.20639
11737367_DYDC1	0.074661	0.124918	0.206464
11733850_GTF3A	0.061076	0.124955	0.206518
11762153_MRPL9	-0.06728	0.124963	0.206523
11716377_GRN	0.071398	0.124985	0.206553
11755545_EXD1	-0.07451	0.124991	0.206555
11724856_CEP63	-0.12481	0.125	0.206564
11761611_DDX11	0.128661	0.125005	0.206565
11741287_HTR3B	0.05636	0.12501	0.206566
11730727_OMG	-0.11784	0.125019	0.20657
11755411_IKBKB	-0.07012	0.125021	0.20657

11763126_IGKC	-0.11669	0.125035	0.206581
11760900_UBXN1	-0.09987	0.125036	0.206581
11758028_FOXQ1	-0.09058	0.12508	0.206641
11739133_NOL10	-0.15084	0.125081	0.206641
11760273_TRGV3	-0.14879	0.125085	0.206641
11734950_MTMR7	-0.11413	0.125115	0.206682
11719090_TAB1	-0.08795	0.125118	0.206682
11757305_RPSA ///	0.056183	0.12515	0.206728
11744964_MIR17HG /	0.105416	0.125216	0.206829
11743143_COX11	-0.06222	0.125232	0.206849
11737889_HRH4	-0.15514	0.125274	0.206912
11751120_OSBPL7	-0.11021	0.12529	0.206932
11715184_RPL38	-0.0785	0.125304	0.206947
11721834_GET4 ///	0.066507	0.125315	0.206958
11748099_PHACTR3	-0.17343	0.125322	0.206964
11721813_VWA1	-0.07212	0.12533	0.206969
11732709_SGCA	-0.12642	0.125336	0.206971
11733620_BCL11B	-0.19232	0.125388	0.207051
11722820_GGT7	-0.08371	0.12542	0.207097
11753107_MOGAT3	-0.10952	0.125425	0.207098
11745693_DFFB	-0.15245	0.125441	0.207113
11725263_PNCK	-0.13074	0.125442	0.207113
11754207_UBE3D	0.108105	0.125462	0.207139
11732615_TCEAL5	-0.10787	0.12549	0.207178
11751784_HCN3	-0.16727	0.125496	0.20718
11757460_ADAM12	-0.0965	0.125502	0.207183
11755990_CAPN12	-0.08514	0.125542	0.207242
11748998_SORCS2	-0.09132	0.125569	0.20728
11744760_TSLP	0.121059	0.125599	0.207323
11715822_SYPL1	-0.06895	0.125616	0.207343
11725091_RFX5	0.067456	0.12562	0.207343
11761699_MAD2L1	0.150419	0.125663	0.207406
11744972_MYO15A	-0.1356	0.12567	0.207411
11748886_FKBP7	0.076777	0.125692	0.207441
11746028_NMI	0.086507	0.125744	0.20752
11748026_PPFIA4	0.067711	0.12575	0.207523
11756724_PPT1	0.069703	0.125757	0.207527
11719905_ENGASE	-0.09312	0.125777	0.207552
11759011_PRPF4	-0.09427	0.1258	0.20758
200018_PMRPS13	0.057281	0.125802	0.20758
11749202_ME3	0.083279	0.125835	0.207626
11752082_CDH12	0.110305	0.125838	0.207626
11735637_LRRC23	-0.12995	0.125847	0.207634
11763754_RASSF1	-0.07576	0.125853	0.207636
11750781_CDH17	-0.14924	0.125858	0.207637
11759276_OR2M3	-0.15483	0.125862	0.207637
11761839_RFC3	-0.13173	0.125873	0.207649
11723366_SYCP1	-0.08969	0.125902	0.207688
11759940_SLC3A1	-0.21821	0.12591	0.207695
11729558_PRSS53	-0.1059	0.125917	0.2077
11756532_PIK3CD	0.07446	0.125944	0.207738

11744079_PRSS2 ///	-0.09871	0.125968	0.20777
11742970_KCTD7 ///	-0.06463	0.125978	0.20778
11719296_MAPKAP1	-0.07483	0.125985	0.207783
11746784_C9orf84	0.113933	0.126003	0.207807
11728178_SIRT4	0.062793	0.126028	0.207841
11732022_TTC25	0.143883	0.126041	0.207856
11729175_KLHL4	-0.12535	0.126051	0.207866
11725827_CD1D	0.112894	0.126102	0.207943
11724165_GPR146	-0.12354	0.126122	0.207968
11727594_KIAA2026	0.073789	0.126148	0.208004
11749728_KIAA1715	0.068936	0.126163	0.208022
11720989_SLC7A7	-0.11204	0.12622	0.208109
11729340_CRYGD	-0.13663	0.126237	0.208129
11761115_MTCH1	-0.10111	0.126249	0.208142
11749251_REXO4	-0.09038	0.126256	0.208147
11728140_SHISA2	-0.07156	0.126276	0.208173
11759933_C22orf15	-0.15553	0.126304	0.20821
11758541_CFAP47	-0.0785	0.126308	0.20821
11737361_CDRT1	0.105855	0.126312	0.20821
11729367_PRKAA2	0.143931	0.126317	0.208213
11761357_CELF4	-0.11898	0.126327	0.208221
11722662_HPSE	0.120455	0.126358	0.208265
11742204_MAPK14	-0.19898	0.126377	0.20829
11723640_C11orf58	0.095269	0.126429	0.208368
11726773_PRKCI	-0.08819	0.126461	0.208415
11739495_SLC29A4	0.095017	0.126541	0.208539
11739766_PNPLA3	-0.07761	0.126574	0.208586
11761545_FAM216A	-0.12383	0.126604	0.208629
11755018_PPP1CC	-0.06011	0.126661	0.208716
11731470_AKT2	-0.15844	0.126676	0.208734
11724242_TCF4	0.08518	0.126685	0.208742
11743665_SPEN	0.091073	0.126697	0.208754
11725312_TMED7	0.062878	0.126707	0.208764
11735051_KIAA1211	0.082477	0.126718	0.208775
11738891_KRTAP5-4	-0.08225	0.126728	0.208784
11764060_GJC2	-0.09386	0.126757	0.208825
11741539_DMKN	-0.10905	0.126763	0.208827
11721079_PNISR	0.058698	0.126769	0.20883
11741656_SLCO1C1	-0.06978	0.126791	0.208859
11753051_ZNF483	-0.12363	0.126813	0.208889
11745847_TACC1	0.074282	0.126823	0.208898
11754104_TMED3	-0.06608	0.126828	0.2089
11737176_C9orf72	-0.09798	0.126833	0.2089
11752618_MED24	-0.07444	0.126884	0.208977
11731546_SIK2	0.097125	0.12689	0.208977
11754796_SLCO3A1	0.081941	0.126892	0.208977
11744066_PRDM6	0.130832	0.126907	0.208994
11743234_NPBWR1	-0.11212	0.126961	0.20907
11746435_TANGO2	-0.07769	0.126962	0.20907
11743627_IRS1	0.0992	0.127028	0.209173
11732871_LCN6	0.098982	0.127038	0.209182

11738357_OR4N3P	-0.15164	0.127083	0.20925
11740898_NOVA1	-0.0971	0.127152	0.209355
11737183_EXOC3L2	-0.06525	0.12718	0.209394
11763457_RPL41 ///	0.069582	0.127204	0.209423
11730041_SMARCC2	-0.12882	0.127206	0.209423
11717018_ATP1A1	0.058734	0.127211	0.209425
11762860_CD300C	-0.13225	0.127231	0.209451
11716586_AXL	-0.06073	0.127246	0.209468
11749954_CLEC1A	-0.12301	0.127266	0.209494
11723021_BCKDHB	0.098752	0.12727	0.209494
11716343_JUP	-0.08127	0.127279	0.209501
11748487_RGL2	0.075613	0.127301	0.20953
11717760_GMFG	-0.10329	0.127311	0.20954
11716798_NDN	-0.06198	0.127352	0.2096
11755703_ZNF287	-0.07192	0.127356	0.2096
11753137_LDLRAD2	-0.11533	0.127439	0.209729
11740602_APH1B	0.103446	0.127443	0.209729
11729986_ROBO2	-0.14827	0.127451	0.209735
11716211_HIBADH	0.073159	0.127457	0.209738
11717692_ANKRD13A	0.105949	0.127476	0.209762
11729002_CDK13	0.08068	0.127486	0.209767
11749543_HABP2	-0.09825	0.12749	0.209767
11749785_CDKL3	0.173917	0.127492	0.209767
11763583_MAN1C1	-0.1283	0.127534	0.209828
11761958_TRAV12-3	-0.13826	0.127573	0.209887
11756325_CUTA	0.063391	0.127581	0.209891
11759977_LOC399886	-0.21583	0.1276	0.20991
11717632_KLHL9	0.070172	0.127604	0.20991
11715632_MAF1	-0.06401	0.127605	0.20991
11747639_NRCAM	0.269986	0.127629	0.209942
11728736_ZBTB38	-0.1555	0.127641	0.209956
11748165_LOC100507	0.073152	0.127648	0.209961
11755634_PNISR	0.062308	0.127679	0.209993
11760131_ACSM2A //	-0.1435	0.127679	0.209993
11755085_KIR3DL2	-0.09606	0.127681	0.209993
11739706_FETUB	-0.08544	0.127707	0.210025
11730161_TEX14	-0.06854	0.127709	0.210025
11755604_RPL7A	-0.05649	0.127758	0.210099
11721937_CHN2 ///	-0.07191	0.127793	0.210148
11724050_TRIM68	-0.11762	0.127864	0.210259
11754096_TAGLN	-0.12332	0.127881	0.210279
11753386_ST13 ///	0.065026	0.127978	0.210433
11717092_EIF4G2	0.063236	0.127983	0.210433
11761799_TMC6	-0.10883	0.127995	0.210445
11733569_C1QL4	-0.26797	0.128009	0.210458
11724508_FGG	-0.10386	0.128011	0.210458
11744356_TACC1	0.113943	0.128035	0.210491
11738527_TP73	-0.09297	0.128071	0.210543
11757408_RPS20	0.060468	0.128081	0.210552
11741760_SLA2	-0.05793	0.128093	0.210561
11762879_NARF	-0.07132	0.128095	0.210561

11758415_CLEC12B	0.154285	0.128122	0.210598
11753671_DSTN	-0.06268	0.128181	0.210685
11757615_MAP6	-0.10226	0.128183	0.210685
11723476_SLC17A5	0.064768	0.128198	0.210701
11746424_ZSCAN2	0.167995	0.128228	0.210743
11750097_CTNND2	0.138426	0.128234	0.210746
11738609_SYT10	-0.08918	0.128244	0.210756
11738266_GPR101	-0.05813	0.128251	0.21076
11717595_ELF3	-0.10874	0.128286	0.210811
11732671_CCDC107	0.1181	0.128313	0.210847
11736789_FBXO4	-0.09434	0.128316	0.210847
11754382_SMIM5	-0.08824	0.128365	0.210909
11748090_NPR1	-0.14459	0.128367	0.210909
11722819_GGT7	0.08519	0.128367	0.210909
11736972_KLRG2	-0.08973	0.128376	0.210916
11750388_HSF5	-0.07428	0.128464	0.21105
11730257_EPN3	-0.07679	0.128466	0.21105
11754406_LONP2	-0.05976	0.128548	0.211174
11750260_SYNJ2	0.1177	0.12855	0.211174
11748842_RAB3A	-0.09545	0.128555	0.211175
11760322_ZSCAN18	-0.11783	0.128564	0.211181
11721045_UNC50	0.0767	0.128569	0.211181
11759492_RPS27L	0.226678	0.128572	0.211181
11732472_DCAF4L2	-0.14904	0.12858	0.211188
11731911_GPR12	-0.11891	0.128598	0.211209
11756837_CLEC5A	-0.07684	0.128611	0.211218
11754930_ATP1A1	0.057554	0.128612	0.211218
11715635_TMEM14C	-0.06944	0.128631	0.211241
11747003_LRRC31	0.087761	0.128634	0.211241
11744963_SUFU	-0.12026	0.128641	0.211242
11731340_NOD2	0.084177	0.128646	0.211242
11734560_ADIPOQ	-0.11008	0.128648	0.211242
11734475_ZIM2	-0.11487	0.128665	0.211263
11741468_SOX6	-0.12902	0.12867	0.211264
11748437_SUSD4	0.138578	0.128676	0.211267
11748464_CORIN	0.129629	0.128684	0.211272
11756560_NDP	-0.17154	0.128696	0.211286
11722504_CYYR1	-0.09992	0.128713	0.211306
11719908_GBA /// G	0.105105	0.128725	0.211319
11737316_LOC102724	-0.10846	0.128808	0.211447
11716005_HYOU1	-0.0926	0.12883	0.211478
11752764_BCR	-0.08225	0.128854	0.21151
11731395_PPP1CC	-0.06171	0.128863	0.211517
11733567_L3MBTL1	0.128314	0.128869	0.211521
11738719_OR5K1 ///	-0.1248	0.12889	0.211547
11739950_CASC5	-0.06719	0.128902	0.21156
11760401_INADL	0.127789	0.128908	0.211562
11736742_C1orf168	-0.07392	0.128928	0.211589
11742413_HECTD4	-0.06087	0.12894	0.2116
11760906_GPR162	-0.17458	0.128944	0.2116
11745275_ITCH	0.069675	0.128983	0.211652

11735898_TOM1	0.077037	0.128984	0.211652
11739238_SNAPC3	-0.06796	0.129023	0.211709
11745393_APOC2	0.135381	0.129028	0.211709
11737625_PAPPA2	-0.10962	0.129058	0.211752
11748321_STARD4	0.112596	0.129065	0.211756
11757175_SNORA35	0.064571	0.12907	0.211758
11716537_SZRD1	0.130399	0.129091	0.211784
11738162_XCR1	-0.09599	0.129142	0.211862
11734644_PWWP2B	-0.1073	0.129156	0.211877
11761840_TFDP1	-0.08724	0.129171	0.211894
11730611_MCOLN2	-0.07601	0.129182	0.211906
11752771_CHST15	0.17589	0.129196	0.211921
11760392_SUGP2	-0.07337	0.129275	0.212044
11763243_G0S2	-0.09664	0.129295	0.21207
11747824_USP10	0.060999	0.129485	0.212374
11727261_ENTPD7	0.105095	0.129559	0.212489
11723695_HSF1	0.061901	0.129565	0.212491
11753006_BCR	-0.13605	0.129588	0.212516
11749279_ARHGAP9	-0.17764	0.129589	0.212516
11715533_LRP1	-0.06292	0.129606	0.212536
11729588_KLK2	-0.12955	0.12961	0.212536
11735723_RBCK1	0.088462	0.129615	0.212538
11758771_HLA-DPB1	-0.07589	0.129625	0.212546
11718413_C14orf166	-0.066	0.129633	0.212551
11717672_ENPP2	0.062095	0.129636	0.212551
11744908_SVBP	-0.05693	0.129691	0.212633
11759243_EPPIN-WFD	-0.11923	0.12971	0.212657
11754899_CEP89	0.082449	0.12973	0.212683
11760494_LINC01549	-0.18022	0.12976	0.212726
11728522_MYCL	-0.07134	0.129783	0.212756
11759270_IFNA5	-0.15009	0.129818	0.212806
11730892_ACTL7A	-0.10454	0.129831	0.212819
11746924_SHROOM4	-0.09865	0.129857	0.212854
11730779_ADH1C	-0.14791	0.129867	0.212854
11720021_RTEL1 ///	-0.12254	0.129868	0.212854
11741922_LCE2C	0.156912	0.129869	0.212854
11762786_COL9A1	-0.17751	0.129875	0.212856
11721185_MEGF8	-0.09268	0.129903	0.212895
11715907_FAM96B	-0.05758	0.129932	0.212936
11740064_SFR1	-0.10146	0.129949	0.212956
11743845_TMEM102	-0.05995	0.129987	0.213011
11757132_SNORA29 /	0.088517	0.130024	0.213065
11748495_OSR2	0.181055	0.130076	0.21313
11720050_PTPRN	-0.10913	0.130077	0.21313
11737919_GUCA1C	-0.0904	0.130077	0.21313
11721773_LOC339166	-0.13097	0.130086	0.213139
11741006_PARG	-0.09128	0.130115	0.213168
11732100_MRAP	-0.10137	0.130117	0.213168
11722059_ZG16	-0.08149	0.130117	0.213168
11725808_SNCAIP	-0.09401	0.130129	0.213176
11751957_HERC4	0.103539	0.130131	0.213176

11746900_PPEF2	-0.113	0.130153	0.213204
11736809_VAV2	-0.10074	0.130161	0.213211
11736481_TREML2	-0.0813	0.130205	0.213275
11760355_PMPCB	-0.08972	0.130209	0.213275
11730471_SCGB2A2	-0.09731	0.130221	0.213287
11758861_ZG16	-0.1443	0.130249	0.213326
11748176_RPS6KA4	-0.08564	0.130278	0.213366
11759526_MGAT4B	-0.10301	0.130328	0.213441
11732834_ZKSCAN7	0.085281	0.130354	0.213477
11723859_DDR2	-0.07128	0.130375	0.213504
11761700_RANBP10	-0.07837	0.13038	0.213505
11737643_OR10AD1	-0.08145	0.130387	0.213509
11733791_SLC25A12	-0.09731	0.130439	0.213586
11762568_TP63	-0.17925	0.130443	0.213586
11726495_ARMC12	-0.1163	0.130489	0.213655
11745618_RAB40A //	-0.12638	0.130495	0.213657
11756901_MASP1	-0.17253	0.1305	0.213657
11726109_HOMEZ	0.139323	0.130503	0.213657
11752034_ABCA8	-0.10501	0.130524	0.213684
11732686_PIWIL2	-0.08898	0.130537	0.213697
11761420_RGS11	-0.16219	0.130602	0.213794
11748745_SF1	-0.06697	0.130604	0.213794
11737529_RPTN	0.12543	0.130647	0.213856
11741869_PARK2	-0.09414	0.130671	0.213885
11734532_SGCZ	-0.06703	0.130673	0.213885
11726684_CDIP1	0.074929	0.130678	0.213886
11758426_B3GLCT	-0.1518	0.130701	0.213917
11755632_MYH14	-0.12555	0.130737	0.213967
11753079_DPF2	-0.07022	0.130748	0.213979
11750382_ACIN1	-0.09363	0.130764	0.213994
11738374_KIR3DL3	0.092802	0.130766	0.213994
11764025_LOC100507	-0.10488	0.130773	0.213998
11738535_WNT1	-0.08822	0.13078	0.214002
11718804_PDCD10	0.062661	0.130786	0.214004
11724806_CIR1	0.132963	0.130815	0.214045
11746645_AP2M1	-0.06624	0.130883	0.214147
11726598_PLPPR1	-0.16335	0.130886	0.214147
11736619_ALDH5A1	-0.07496	0.130922	0.214195
11760312_NAMPT	0.133022	0.130924	0.214195
11759179_TMEM38B	-0.05864	0.130945	0.214222
11751443_SH3BP5L	-0.13863	0.131012	0.214325
11763799_FAM71F1	0.247303	0.131053	0.214385
11739988_ZNF823	0.106075	0.131062	0.214392
11757409_TMSB10	-0.06051	0.131075	0.214405
11730235_MYADM	0.061526	0.131082	0.214405
11727416_CRYL1	0.101862	0.131083	0.214405
11748703_C1D	-0.08095	0.131133	0.21448
11724281_SUGT1	0.068205	0.131148	0.214496
11728913_C8B	-0.06829	0.131157	0.214504
11744520_PCGF1	-0.06829	0.131164	0.21451
11750207_HSD17B12	-0.06131	0.131171	0.214513

11757692_APC	-0.07175	0.13133	0.214765
11753026_PRLR	-0.14869	0.131352	0.214794
11740573_BEND7	0.154512	0.131377	0.214828
11751453_MRRF	0.074969	0.131385	0.214834
11760053_TIRAP	0.175075	0.131398	0.214848
11729807_COX6B1	-0.06016	0.131414	0.214867
11760959_RABGAP1L	0.138579	0.131425	0.214878
11763089_VEGFA	-0.07078	0.131448	0.214908
11746262_MAPK12	-0.06736	0.13147	0.214936
11730544_TEX38	-0.10043	0.131502	0.214983
11763541_SLC25A47	-0.09591	0.131507	0.214983
11732745_SYT3	-0.07434	0.131541	0.215032
11723053_SZT2	-0.0769	0.131552	0.215039
11742084_PCDHA1	-0.11336	0.131555	0.215039
11762604_GTF2IRD2B	0.159843	0.131559	0.215039
11740384_KSR1	0.108281	0.131625	0.21514
11718074_IL4R	0.112091	0.131644	0.215165
11734632_RNF180	0.175714	0.131685	0.215223
11752805_SERPINB3	-0.14347	0.131699	0.21524
11745616_TRIM15	-0.16082	0.131715	0.215258
11721921_SENP5	-0.06332	0.1318	0.215385
11753673_PLA2G2D	-0.12475	0.131801	0.215385
11743052_CHORDC1	0.111915	0.131808	0.21539
11721353_GALNT3	-0.08818	0.131824	0.215408
11743246_DEFB114	0.078742	0.13183	0.215411
11727435_AIPL1	-0.05958	0.131872	0.215464
11728778_RPAP2	0.142112	0.131872	0.215464
11735657_KCNC3	-0.09731	0.131884	0.215475
11737739_GPR27	-0.06643	0.131887	0.215475
11723193_LTF	0.10445	0.131909	0.215504
11745398_NF1	0.136602	0.131914	0.215504
11722833_CCNY	-0.06628	0.131953	0.21556
11719577_AMT	-0.08584	0.131958	0.215562
11758128_LOC101929	-0.08703	0.132006	0.215633
11755031_CENPP	0.083502	0.132013	0.215638
11721569_ANG	-0.19743	0.132044	0.215675
11747351_ZSCAN32	0.067015	0.132045	0.215675
11747331_AHCYL1	-0.07095	0.132049	0.215675
11761285_ASRGL1	-0.19884	0.132071	0.215703
11723444_CRTAC1	0.144574	0.13214	0.215809
11733708_CADPS	0.125546	0.13216	0.215834
11738465_TPH2	0.07992	0.132207	0.215904
11747774_SLC11A1	-0.08887	0.132212	0.215904
11757275_ARGLU1	0.065585	0.132221	0.215906
11718652_STAT2	-0.10815	0.132221	0.215906
11764019_NAB1	0.07768	0.132243	0.215934
11757116_SNORA26	-0.07447	0.132276	0.21598
11758703_MAFA	-0.08752	0.132297	0.216007
11743855_TFE3	-0.08726	0.132308	0.216019
11716276_CBX5	0.059688	0.132314	0.216021
11715560_PGA3 ///	-0.07558	0.13237	0.216105

11742107_PPT1	0.062947	0.13244	0.216211
11740403_SMCO3	0.129143	0.132483	0.216274
11721709_UBN1	-0.11604	0.132496	0.216288
11751340_KRT85	-0.1087	0.1325	0.216288
11734597_KCNG4	0.118441	0.132511	0.2163
11736863_EMC6	0.070594	0.13252	0.216307
11762606_LOC645261	0.15106	0.132527	0.216308
11716871_GAA	0.071719	0.13253	0.216308
11734926_ZDHHC15	-0.13779	0.132546	0.216328
11734313_OBSCN	-0.13821	0.132561	0.216342
11717779_DHX35	-0.08623	0.132564	0.216342
11745123_TMEM198B	-0.08185	0.132574	0.216351
11763397_TARID	-0.16	0.132596	0.21638
11749704_SLC9A1	0.074177	0.132603	0.216384
11755427_GUCY1A2	-0.15935	0.132607	0.216384
11749152_TBRG1	-0.10319	0.132619	0.216395
11751191_LIPG	-0.10301	0.132649	0.216437
11734845_TAF1B	-0.09132	0.13266	0.216447
11723628_FAM162B	-0.11571	0.132665	0.216447
11745489_GOLGA8CP	-0.11619	0.132668	0.216447
11763370_CHORDC1	-0.07364	0.132695	0.216484
11722607_UFSP2	0.112945	0.132823	0.216682
11761539_C20orf96	0.069691	0.132826	0.216682
11741569_SLC26A1	-0.09204	0.132895	0.216789
11723330_DNAJB12	-0.08711	0.13291	0.216806
11715302_NT5C1A	-0.11186	0.13292	0.216815
11754818_CATSPER2	-0.14637	0.132935	0.216833
11720873_MINK1	0.085003	0.132941	0.216834
11720857_INSR	-0.11482	0.132957	0.216853
11718784_TCEA2	-0.17844	0.132981	0.216886
11726946_PPM1A	0.087162	0.133017	0.216933
11734818_APBB2	-0.09167	0.133021	0.216933
11720949_PFDN4	0.113719	0.133024	0.216933
11762672_CNOT4	-0.23023	0.133059	0.216983
11757875_LIME1 ///	0.091325	0.133093	0.217032
11720101_SPIN1	0.06584	0.133099	0.217034
11732610_ZNF385D	0.100213	0.13312	0.217062
11733681_TMEM132D	-0.06268	0.13313	0.21707
11730680_MC3R	-0.14502	0.133169	0.217127
11725279_IHH	0.114982	0.133216	0.217197
11740189_UBAC2	-0.05815	0.13324	0.217228
11750558_ZNF271P	0.091153	0.133264	0.21726
11749005_CTNNA1	0.075009	0.133327	0.217355
11760717_RPS6KC1	0.190081	0.133335	0.217356
11726955_CASZ1	-0.06923	0.133337	0.217356
11729181_DHRSX	0.081677	0.133381	0.217422
11743902_PSMD1	-0.07066	0.133473	0.217565
11749165_GLT1D1	-0.12856	0.133496	0.217586
11730152_TMEM207	-0.1099	0.133496	0.217586
11732214_ZKSCAN1	0.064338	0.1335	0.217586
11735182_TTC7A	-0.05811	0.133545	0.217653

11758252_HSD3B7	-0.07047	0.133601	0.217737
11735071_PCDHB9	-0.1536	0.13361	0.217744
11737059_MMP26	-0.14137	0.133633	0.217773
11759775_SLC9B2	-0.10142	0.133649	0.217793
11724329_DGCR8	-0.10914	0.133665	0.217807
11719437_ZNF746	0.086602	0.133667	0.217807
11716212_HIBADH	0.058491	0.133679	0.21782
11729624_DCAF7	-0.08381	0.133691	0.217832
11740482_PAQR5	0.112646	0.133722	0.217876
11720853_NFIA	-0.13643	0.133751	0.217916
11715203_ATP13A5	-0.08789	0.133757	0.217917
11757583_TRIL	-0.13289	0.133761	0.217917
11757582_USP9X	0.072869	0.133798	0.217971
11736455_UGT2B10	-0.08506	0.133838	0.218028
11742915_LMTK2	0.144048	0.133857	0.218052
11761372_TNRC18	-0.10442	0.133872	0.218069
11737342_C20orf166	0.089521	0.133895	0.218099
11758835_SKIV2L2	-0.08686	0.133911	0.218112
11730026_PQBP1	0.073333	0.133912	0.218112
11742288_PTX4	-0.07127	0.133919	0.218112
11733625_MYO15A	0.132998	0.133921	0.218112
11716289_BLOC1S1	-0.06533	0.133945	0.218144
11744253_ODF3L1	-0.11019	0.133961	0.218158
11758894_PARD3	0.105906	0.133963	0.218158
11743705_ETAA1	-0.07649	0.133967	0.218158
11739600_AASS	-0.06091	0.133991	0.21819
11743225_C9orf172	-0.11415	0.134	0.218197
11725060_USP7	0.063597	0.134009	0.218205
11729880_ZNF445	0.225109	0.134068	0.218278
11751274_OLFML1	-0.16149	0.134069	0.218278
11726950_FAM131B	-0.14101	0.13407	0.218278
11731459_PRH2	-0.12303	0.134072	0.218278
11724867_GAB2	-0.07052	0.134103	0.218321
11722544_EYA2	0.102657	0.134147	0.218386
11740631_MYF5	0.083835	0.134171	0.218419
11749327_CD99L2	-0.11959	0.134204	0.218464
11730663_LRRC24	0.082006	0.13422	0.218483
11755534_ADCY7	-0.08505	0.134266	0.218551
11760873_TTC26	0.160882	0.134277	0.218561
11734484_GRIK1	-0.08765	0.134296	0.218583
11752691_STPG1	-0.15243	0.134299	0.218583
11759904_TIE1	-0.16348	0.134319	0.218607
11752910_ARL6IP1	-0.05947	0.134326	0.218612
11721044_ERI1	0.086055	0.134357	0.218655
11755068_BOD1L1	-0.06644	0.134407	0.218729
11725479_OVOL1	-0.07821	0.134415	0.218735
11754074_G0S2	-0.12885	0.134435	0.21876
11755684_CMPK1	0.060733	0.134441	0.218763
11752340_LMNA	-0.06235	0.134452	0.218773
11720057_POLR1D	-0.0696	0.13449	0.218827
11744722_RHCE ///	0.11646	0.134504	0.218843

11748072_HSPH1	0.058302	0.134531	0.21888
11730158_RBM20	-0.10547	0.13455	0.218904
11756514_TEKT5	-0.13913	0.134581	0.218946
11740018_INSR	0.138911	0.134622	0.219006
11743748_LAT	0.130611	0.13465	0.219044
11719818_PIP	-0.08581	0.134667	0.219065
11761335_TROAP	-0.10877	0.134675	0.219071
11760547_POLM	0.110426	0.134684	0.219077
11738451_ZCCHC16	-0.13587	0.134754	0.219185
11756293_UFSP2	0.058294	0.13476	0.219185
11744290_BRMS1	-0.07256	0.134763	0.219185
11718991_NDUFB7	-0.06409	0.134772	0.219192
11718434_NPNT	0.063014	0.134859	0.219326
11757278_ASS1	-0.08399	0.134886	0.219363
11724408_STAC2	-0.07258	0.134908	0.219392
11752697_PCED1A	0.064141	0.134938	0.219432
11753455_RETNLB	-0.11059	0.134988	0.219507
11734131_SLC2A4	-0.10079	0.134994	0.219509
11732516_CERS4	-0.09311	0.134999	0.21951
11732030_SPEM1	-0.08426	0.135018	0.219533
11724671_AMDHD1	-0.13573	0.135023	0.219533
11727030_MAP1A	-0.07753	0.13504	0.219554
11755568_SLC47A2	-0.06453	0.135044	0.219554
11730560_GCKR	-0.07867	0.135111	0.219651
11738709_OR56A1	-0.07453	0.135113	0.219651
11755878_TESK1	0.065434	0.135135	0.219679
11721168_EPB41L1	0.069439	0.135156	0.219704
11742226_AIPL1	-0.11145	0.135159	0.219704
11716209_VPS28	0.065625	0.135169	0.219712
11735386_DACT1	-0.08685	0.135299	0.219917
11717598_SMPD1	0.069252	0.135323	0.219949
11761528_SEPT10	-0.0594	0.135358	0.219998
11754801_PLPP1	0.073865	0.135402	0.220063
11730984_ZFYVE9	0.082005	0.135449	0.220132
11736516_SERAC1	0.190513	0.135461	0.220143
11744174_UBQLN4	-0.07633	0.135478	0.220164
11724694_ETV7	-0.11445	0.135512	0.220212
11723195_HLA-E	0.070289	0.13554	0.220243
11762294_TRBC1 ///	-0.07342	0.13554	0.220243
11762097_WNK4	-0.10681	0.13556	0.220268
11719854_TBCE	-0.06715	0.135585	0.220294
11747930_UBAP1	-0.07867	0.135586	0.220294
11751313_MANEAL	0.078862	0.135597	0.220294
11741595_TSHB	-0.0899	0.135599	0.220294
11731199_LHFPL3	0.120883	0.135602	0.220294
11749022_CTNNB1	-0.08408	0.135603	0.220294
11736070_C8orf33	-0.08452	0.135608	0.220295
11751119_OSBPL7	-0.09268	0.135629	0.22032
11763302_LOC100128	-0.1211	0.135633	0.22032
11751738_PPP1R3E	0.212461	0.135652	0.220344
11749265_AQP7	-0.08757	0.135678	0.220379

11721484_CCDC85B	0.067493	0.13574	0.220468
11734271_CDH18	-0.18303	0.135742	0.220468
11733660_CHIA	0.118509	0.13576	0.220491
11748300_LHX6	-0.14104	0.135765	0.220492
11719673_THBD	-0.09269	0.135802	0.220543
11717302_TACSTD2	-0.10925	0.135813	0.220555
11734052_ZMYM6	0.116994	0.135841	0.220588
11743945_ZNF839	0.065463	0.135842	0.220588
11738424_BMP10	-0.14262	0.135882	0.220645
11717015_CLDN7	-0.08385	0.135918	0.220696
11720596_CSRNP2	-0.07379	0.135947	0.220735
11758301_KRT8	0.067609	0.135968	0.220752
11728113_MBD5	0.124254	0.135969	0.220752
11731714_CACNB2	-0.10198	0.135971	0.220752
11738989_GANAB	-0.06516	0.135976	0.220753
11728490_NAA38	-0.06336	0.135991	0.220768
11717324_EZH1	0.075411	0.135994	0.220768
11724956_C14orf169	0.063098	0.136014	0.220793
11734760_HIP1	-0.1235	0.136071	0.220879
11763249_IGH /// I	-0.07494	0.136089	0.2209
11745270_RHBDL2	0.137861	0.136095	0.220903
11744983_NRG2	-0.09209	0.136105	0.220911
11717055_CORO1C	0.067703	0.136122	0.220931
11736349_ZNF366	0.121187	0.136147	0.220964
11741934_IL25	-0.07744	0.136171	0.220997
11755043_GSAP	0.090223	0.136182	0.221006
11761445_PATL1	-0.15388	0.136188	0.221007
11757196_TMEM51-AS	0.121314	0.136194	0.221007
11761976_PDS5B	0.059019	0.136196	0.221007
11726952_FAM131B	-0.09301	0.136203	0.221012
11741720_ADAR	-0.06981	0.136243	0.22107
11734542_TDRD12	-0.10026	0.136275	0.221114
11736620_CCR6	0.125641	0.136305	0.221156
11734460_RFPL2 ///	-0.06735	0.136383	0.221275
11738427_KRTAP4-1	-0.081	0.13641	0.22131
11722951_GAL3ST1	-0.21145	0.136442	0.221356
11715139_IFNA10	-0.07412	0.136459	0.221374
11735023_ZBTB7B	-0.08059	0.136463	0.221374
11744138_CAMK1D	-0.08799	0.1365	0.221427
11759116_VTI1B	0.099615	0.136504	0.221427
11724207_MAD1L1	-0.06334	0.136575	0.221535
11729571_FAM83A	-0.11076	0.136588	0.221549
11733651_FAM228B	-0.11684	0.136628	0.221601
11750666_VPS39	0.102828	0.136634	0.221601
11727129_MAGEB2	-0.08746	0.136634	0.221601
11754953_C1orf198	-0.08972	0.136642	0.221605
11730066_MAPK10	0.180354	0.136663	0.221633
11733682_ITSN2	-0.14328	0.136696	0.221678
11737488_PI4KB	0.063666	0.136751	0.221761
11759705_IGLV3-10	-0.1086	0.136779	0.221798
11747046_CAPZA2	0.077059	0.136786	0.221803

11746950_NUPL2	0.097551	0.136828	0.221863
11741253_MGAT4C	-0.10497	0.136877	0.221935
11760292_NAA10	-0.07683	0.136965	0.222071
11756965_RRP1	-0.07981	0.137003	0.222126
11734466_COL6A5	0.087927	0.137017	0.222134
11721324_TBC1D10B	-0.07716	0.137017	0.222134
11733586_NPFF	-0.07588	0.137057	0.222191
11762486_FUT6	-0.10107	0.137085	0.222229
11718507_PSMG2	-0.06142	0.137124	0.222285
11763042_LOC646813	0.146096	0.137147	0.222308
11754628_FBRS	0.118645	0.137148	0.222308
11729789_DOHH	-0.08097	0.137154	0.222311
11719623_GTPBP1	-0.06707	0.137181	0.222347
11755273_C16orf71	-0.086	0.137236	0.222429
11759711_RBM25	0.133212	0.137306	0.222533
11745324_NELL1	-0.09163	0.137309	0.222533
11729362_DIO1	-0.11431	0.137319	0.222542
11755452_HTRA2	-0.07633	0.137379	0.222632
11754354_PROM1	-0.1036	0.137408	0.222671
11719808_BLOC1S6	0.109784	0.137416	0.222671
11725934_HSPA5	-0.06378	0.137417	0.222671
11727954_PACS2	0.068158	0.137459	0.222732
11725504_GDF11	-0.06749	0.137474	0.222748
11743555_QTRTD1	0.07064	0.137543	0.222852
11741830_RFPL4A //	-0.09944	0.137585	0.222913
11732932_SAXO2	0.070876	0.137616	0.222957
11741881_DMKN	-0.12081	0.137709	0.2231
11721296_NDUFB1	0.059897	0.137714	0.2231
11728801_PRSS54	-0.13708	0.137736	0.223122
11742384_OR4E2	-0.08124	0.137736	0.223122
11758996_FAM120A	0.156579	0.137746	0.22313
11719579_TACO1	-0.06084	0.137765	0.223154
11738699_OR4D5	-0.06978	0.137781	0.223172
11745521_ABI1	0.071731	0.137807	0.223207
11753690_CBX7	-0.11932	0.137833	0.223232
11730578_ADM2	-0.11303	0.137835	0.223232
11743846_CFH /// C	0.175993	0.137836	0.223232
11746714_NUP43	-0.09237	0.137869	0.223278
11725904_ZNF502	-0.10366	0.137955	0.22341
11749413_ABLIM3	0.06094	0.137962	0.223414
11728808_CHAC2	-0.06381	0.137991	0.223454
11757057_PLA2G2A	-0.13568	0.138014	0.223483
11742862_ADGRF5	0.20392	0.13803	0.223501
11757811_CHURC1-FN	-0.08446	0.138044	0.223516
11742250_PDE11A	0.121854	0.138048	0.223516
11729513_CSF3	-0.06566	0.138058	0.223525
11723081_DVL1	-0.07374	0.138068	0.223533
11737556_TLX3	-0.17028	0.138079	0.223543
11750138_POGK	0.057681	0.138096	0.223564
11744894_FAM20A	0.086174	0.138101	0.223564
11744415_MFSD6	0.105589	0.13811	0.223566

11762166_ATAT1	0.098361	0.138111	0.223566
11750603_PLEKHG5	-0.06373	0.13813	0.223589
11756716_FBXL15	-0.06873	0.138134	0.223589
11754140_KCNIP2	-0.10877	0.13815	0.223607
11763413_ASAP1-IT2	0.093866	0.138175	0.22363
11725942_WNT7B	-0.07274	0.138175	0.22363
11757790_PCDH17	0.119159	0.138178	0.22363
11730109_NLRC3	0.093485	0.13819	0.223642
11762913_BAK1	-0.143	0.138197	0.223647
11738608_SYT10	0.148104	0.138204	0.22365
11719458_EXOC5	0.071719	0.138216	0.223661
11746960_ABCF3	-0.08943	0.138236	0.223687
11760266_ATPAF2	-0.09853	0.138277	0.223737
11735405_ANKS1B	-0.134	0.138277	0.223737
11760523_COL4A5	0.170104	0.138281	0.223737
11739161_IP6K1	-0.06637	0.138287	0.22374
11748829_TUBB4A	-0.08317	0.138304	0.223753
11756924_ATP6V1A	0.05908	0.138305	0.223753
11733756_VWC2L	-0.18336	0.138348	0.223813
11729573_KHDC1L	-0.07834	0.138351	0.223813
11752651_PPP1R12B	0.102532	0.138363	0.223823
11749557_APOL6	-0.10042	0.138366	0.223823
11738352_SCN1B	-0.07782	0.13844	0.223932
11759077_HIST1H3A	-0.08423	0.138443	0.223932
11739386_CDC73	-0.066	0.138459	0.223951
11748816_UGT2A3	0.072941	0.138466	0.223955
11736707_ZNF547	0.069231	0.138523	0.22404
11738726_OR2T2 ///	-0.11326	0.138559	0.224091
11759609_SLC18A2	-0.08166	0.138582	0.22412
11746893_MPP1	-0.09662	0.138607	0.224154
11725459_S100A7 //	-0.1244	0.138654	0.224221
11738693_OR13C3	0.083904	0.138668	0.224237
11735616_PRR35	-0.108	0.138693	0.224271
11748737_ARNT2	-0.06722	0.138705	0.224278
11762137_ACTG2	0.065551	0.138707	0.224278
11730601_INO80B	-0.05738	0.138716	0.224285
11737292_GLYCTK	-0.13165	0.138741	0.224319
11728828_F2RL1	0.128079	0.138762	0.224344
11753567_CKLF-CMTM	-0.08457	0.138782	0.22437
11734541_IL5RA	0.086727	0.138828	0.224436
11743833_RSBN1L	0.065159	0.13886	0.224481
11756947_CTSB	-0.0549	0.138866	0.224482
11751002_DLAT	-0.07251	0.138896	0.224522
11723404_PPARGC1A	0.212355	0.138899	0.224522
11747576_GGNBP2	-0.06279	0.138909	0.22453
11746385_GAPVD1	-0.07482	0.138923	0.224538
11746978_GAPVD1	-0.07482	0.138923	0.224538
11742958_SMARCA4	0.058954	0.138942	0.224562
11760698_AGBL2	-0.16735	0.138955	0.224574
11723301_RPS6KB1	0.086488	0.138984	0.224615
11757256_LINC00235	-0.11648	0.13899	0.224616

11719912_KDM1B	-0.10835	0.138994	0.224616
11741260_VSTM2A	-0.14159	0.139014	0.22464
11731693_HIST1H2AE	-0.0815	0.139018	0.22464
11732904_CLEC1B	-0.10453	0.139045	0.224676
11751205_DOK5	-0.08321	0.139054	0.224682
11750122_DNMT3B	-0.06883	0.139061	0.224686
11733015_DALRD3	-0.07891	0.139111	0.224761
11728900_ARHGAP6	-0.15568	0.139158	0.224829
11739640_DIP2A	0.05715	0.139188	0.224869
11744269_GIPC1	0.071079	0.139201	0.224871
11742237_KNCN	-0.0705	0.139206	0.224871
11748484_KRT6A	-0.10411	0.139206	0.224871
11738716_OR6C1	-0.06276	0.139207	0.224871
11715242_LCE3D	-0.11934	0.139212	0.224871
11725100_ESF1	0.106556	0.139239	0.224907
11740205_RPH3A	-0.12881	0.139256	0.224921
11749961_MDH1	-0.05745	0.139256	0.224921
11719886_IFI44	-0.06288	0.139293	0.224973
11722472_PEG3	-0.19084	0.139332	0.225029
11728436_BRWD1	0.106094	0.139356	0.225059
11723515_CAB39L	-0.1002	0.139411	0.22514
11750825_ADAMDEC1	-0.12931	0.139429	0.225162
11738837_NPAS1	0.057803	0.13948	0.225237
11762803_APP	0.157082	0.139504	0.225269
11754546_TMEM25	0.062287	0.13954	0.225319
11754291_ARHGEF2	0.060169	0.13955	0.225328
11750809_ARHGAP6	-0.07464	0.139579	0.225367
11730149_PAX1	-0.10559	0.139584	0.225367
11751848_HCG9	-0.10333	0.13965	0.225462
11734579_TAS2R14	0.1288	0.139652	0.225462
11764260_FAM124A	-0.10358	0.139691	0.225519
11735662_P2RY2	-0.10277	0.139713	0.225547
11741992_FAM170A	-0.11437	0.139722	0.225554
11731119_TEFM	-0.06568	0.139745	0.225583
11746611_KIFAP3	0.055058	0.139796	0.225659
11738289_KRTAP10-1	-0.0662	0.139804	0.225659
11719277_MRPL17	0.05758	0.139806	0.225659
11754050_CXADR	0.079972	0.139845	0.225715
11727720_BCAP29	0.081831	0.139851	0.225717
11749917_HEY2	-0.10349	0.139918	0.225818
11728583_RHOXF2 //	-0.07737	0.139975	0.225903
11761479_HSP90B2P	-0.09254	0.140027	0.225979
11728684_KRR1	-0.09373	0.14007	0.226041
11747737_CNN1	-0.08119	0.140129	0.226129
11751926_MAPKBP1	0.068384	0.14014	0.226138
11760584_TRDC	0.119052	0.14016	0.226163
11735383_LDLRAD4	-0.12946	0.140244	0.226289
11721177_PITX1	-0.09674	0.140247	0.226289
11718558_MKRN1	0.076987	0.140322	0.226403
11735974_NEU2	-0.09371	0.140363	0.226462
11759591_ATAT1	-0.16585	0.140369	0.226464

11738282_CCNK	-0.07069	0.140383	0.226479
11741896_RPLP1	0.056614	0.140397	0.226493
11760522_SHROOM3	-0.07659	0.140406	0.226501
11716619_TNPO1	-0.05876	0.140414	0.226506
11721725_PSKH1	-0.07029	0.140435	0.226532
11763258_CALM2	-0.05542	0.140484	0.226604
11757891_SCAND2P	0.161535	0.140505	0.22663
11739056_GPM6A	0.08802	0.140528	0.226659
11729078_SLC26A7	0.107519	0.140577	0.226732
11746156_IRF9	-0.08186	0.140641	0.226827
11752341_F8	-0.0766	0.140658	0.226847
11763188_SNX5	-0.08375	0.140695	0.226885
11729024_KCNA5	-0.1851	0.140696	0.226885
11735561_ZNF192P1	-0.15673	0.140696	0.226885
11753239_SCARA5	-0.07725	0.1407	0.226885
11745199_ANKRD9	-0.07346	0.140713	0.226898
11725734_NFYA	-0.11004	0.140728	0.226915
11746200_EHD2	0.062635	0.140734	0.226917
11721055_PRKX	0.099547	0.140762	0.226956
11719759_ARF1	-0.05656	0.140774	0.226967
11743829_PAM	0.065244	0.140791	0.226987
11734293_KCNJ11	0.078012	0.140844	0.227065
11731270_MGAM	-0.0878	0.140859	0.227081
11721962_SLC4A8	0.114091	0.140866	0.227084
11716002_FAF2	0.071876	0.14087	0.227084
11729042_DLEC1	-0.13571	0.1409	0.227125
11755441_MED15	-0.06243	0.14093	0.227166
11716922_CLEC11A	-0.06021	0.140939	0.227173
11751634_ACAD9	-0.06811	0.140951	0.227184
11763384_FBN2	0.106798	0.14096	0.227191
11736767_LOC101929	-0.08982	0.140995	0.227241
11737798_PROCR	0.065227	0.141031	0.227291
11760849_INADL	-0.1417	0.141099	0.227388
11725875_WDR66	-0.13133	0.1411	0.227388
11756596_PDPR	0.095719	0.141113	0.227401
11720497_MED4	-0.07297	0.141129	0.227419
11715912_EFNB1	-0.09394	0.141156	0.227455
11723204_NOL7	-0.05572	0.141172	0.227474
11735290_TNIP2	-0.0734	0.141208	0.227524
11740020_OTC	-0.11056	0.141263	0.227604
11737596_ACOXL	0.106749	0.141283	0.227629
11728154_FUT4	0.093211	0.141307	0.22766
11756617_TAPBP	0.070218	0.141324	0.227679
11746278_PLG	-0.15496	0.141328	0.227679
11739892_ARPC4-TTL	0.081266	0.141353	0.227705
11734272_RPS6KA6	-0.12154	0.141353	0.227705
11727830_SNAP29	-0.08117	0.141373	0.227729
11759783_CLASP2	-0.06411	0.1414	0.227765
11751127_SHISA5	-0.0638	0.14144	0.227819
11715209_SCAND1	0.071189	0.141444	0.227819
11752659_ANGPT4	0.072951	0.141448	0.227819

11754684_SRL	-0.1114	0.141459	0.227819
11734531_TNFSF11	-0.15477	0.141464	0.227819
11741855_ARGFX	-0.14084	0.141465	0.227819
11727952_CBX8	-0.07567	0.141466	0.227819
11744842_LINC00474	-0.15853	0.141477	0.22783
11730496_NUDT19	0.064652	0.141548	0.227936
11730101_CRYGA	-0.11288	0.141556	0.227943
11718583_CNPY2	-0.05649	0.141567	0.227947
11732917_DZIP1L	-0.09895	0.141569	0.227947
11752014_IKZF2	0.263286	0.141603	0.227991
11730894_UBA6	0.071584	0.141605	0.227991
11761309_PIH1D1	-0.06276	0.141612	0.227995
11762952_PMS1	-0.07572	0.141687	0.2281
11738865_CYP26C1	-0.09556	0.141692	0.2281
11732885_CCDC158	-0.17281	0.141692	0.2281
11753811_ENY2	-0.05808	0.141701	0.228108
11750179_ECHDC2	0.068429	0.141715	0.228117
11744577_EVC2	0.102339	0.141719	0.228117
11759684_IGKC	-0.135	0.14172	0.228117
11717913_CDO1	-0.07968	0.141755	0.228158
11736835_TPCN2	-0.09488	0.14176	0.228158
11735704_MT1A ///	-0.07796	0.14176	0.228158
11716972_NSF	-0.06302	0.141767	0.228161
11746284_NFATC4	-0.08131	0.14178	0.228175
11737179_SLC25A21	-0.09377	0.141789	0.228182
11723085_IL17RB	0.084355	0.141797	0.228186
11761278_FLT3LG	-0.16245	0.141812	0.228204
11757688_ANKHD1	-0.0584	0.141841	0.228243
11757559_MAML1	-0.06925	0.14186	0.228266
11719175_GPRC5B	-0.05483	0.141872	0.228278
11758695_C1QTNF7	0.076553	0.141908	0.228329
11717351_RPRD1A	0.083505	0.141935	0.228364
11740428_PSAPL1	-0.09228	0.141956	0.228391
11745192_EPB41L3	0.142267	0.142012	0.228473
11729757_RHAG	-0.1125	0.142027	0.228489
11736917_LYPD2	-0.0995	0.142041	0.228504
11744978_ORC4	-0.08356	0.142045	0.228504
11727531_PHF20L1	-0.05926	0.142063	0.228525
11734062_CD200R1	0.123396	0.14208	0.228544
11737194_UTS2B	0.147271	0.142094	0.228553
11763868_PDZD11	0.103919	0.142094	0.228553
11728366_LBP	-0.0864	0.142163	0.228655
11755487_KIF13A	0.180323	0.142195	0.228698
11745111_DLGAP1	-0.11503	0.142198	0.228698
11722897_REV1	0.089457	0.142212	0.228711
11725359_ACAN	-0.1257	0.142236	0.228743
11750767_SLC17A7	-0.09488	0.142249	0.228756
11731049_RIMKLA	-0.1263	0.142281	0.2288
11732369_FGF14	0.167964	0.142292	0.22881
11746561_ZFP36L1	-0.08798	0.142328	0.228861
11747569_CLCNKA //	-0.08701	0.142346	0.228883

11741187_APOL1	-0.11169	0.14236	0.228897
11722482_MFF	0.066231	0.142365	0.228898
11737833_IL12B	0.21471	0.142418	0.228976
11750718_RGS22	0.059747	0.14246	0.229035
11748315_PRNP	0.060363	0.142494	0.229082
11743897_KIAA1257	-0.11935	0.142511	0.229094
11751068_KIAA1257	-0.11935	0.142511	0.229094
11734210_TMPRSS11E	-0.06962	0.142558	0.229163
11754014_LOC100129	-0.09879	0.142582	0.229194
11716976_GLRX3	0.087152	0.142592	0.229202
11722841_TFB2M	0.055945	0.142611	0.229225
11728680_PAIP1	-0.08066	0.142682	0.229332
11753108_MOGAT3	0.059607	0.142731	0.229403
11718762_POMP	0.075632	0.142774	0.229462
11746188_RPL14 ///	0.055864	0.142777	0.229462
11744867_LYPD6	-0.06904	0.142797	0.229487
11763741_ACTR6	-0.07793	0.142843	0.229552
11730463_SYN3	-0.12564	0.142857	0.229568
11725173_MN1	-0.11277	0.142872	0.229585
11716383_CCT3	0.057064	0.142885	0.229598
11744548_RPL29	0.054451	0.142902	0.22961
11746168_SHROOM2	0.070945	0.142902	0.22961
11763882_LOC51145	-0.08998	0.14291	0.229616
11751606_SP140	0.085772	0.142915	0.229616
11745959_RGP1	0.113026	0.142951	0.229666
11754347_DBNDD1	0.093767	0.142966	0.229682
11723150_SUGCT	-0.05686	0.142987	0.22971
11757437_PODN	-0.07857	0.143002	0.229726
11728895_HES2	-0.07986	0.143035	0.229771
11756455_CLPS	-0.08584	0.143056	0.229797
11749534_ICK	0.31887	0.143071	0.229814
11757949_NINL	-0.10227	0.143096	0.229845
11760314_CLK4	0.119251	0.143101	0.229845
11754704_DNAJC11	0.062974	0.143105	0.229845
11746540_CAMK2D	-0.09361	0.143114	0.229852
11725604_HOXA9	-0.12054	0.143158	0.229916
11758732_STARD10	-0.06059	0.143181	0.229937
11748567_FGF11	-0.1284	0.143185	0.229937
11746801_IRF5	-0.07889	0.143186	0.229937
11755422_CD6	-0.10821	0.143208	0.229966
11761665_LINC00311	-0.236	0.143277	0.230069
11744291_BRMS1	-0.06279	0.143317	0.230125
11747513_GUSBP1	0.094473	0.143334	0.230145
11752579_TBXA2R	-0.07113	0.143346	0.230157
11757637_MUS81	0.086726	0.143363	0.230177
11751143_ABI1	0.067867	0.143372	0.230183
11741580_TOM1	0.112327	0.143384	0.230195
11736609_MSX1	0.068692	0.143391	0.230199
11754275_ST6GALNAC	0.063616	0.143473	0.230316
11716190_VWA9	-0.06176	0.143473	0.230316
11727515_TTC9	-0.15086	0.143496	0.230345

11751871_EPGN	-0.09037	0.143523	0.23038
11760774_GNL1	-0.09754	0.143528	0.230381
11727934_ACTR5	-0.0825	0.143608	0.230502
11738830_ADRA1A	-0.13986	0.143705	0.23065
11736248_AMIGO2	-0.12308	0.14374	0.230698
11736754_PCDHB13	0.075395	0.143757	0.230718
11741459_CYP4A11 /	-0.09379	0.143762	0.230719
11757761_LOC101929	-0.05309	0.14378	0.230737
11748242_CORO7 ///	-0.06284	0.143782	0.230737
11738798_PCDH15	-0.13738	0.143824	0.230796
11755820_KBTBD3	-0.07908	0.143843	0.230818
11755580_CYLC1	-0.09086	0.143891	0.230887
11729508_ATP8B3	-0.13124	0.143898	0.230892
11763154_THPO	-0.15258	0.143912	0.230906
11742258_CCT8L2	-0.06969	0.143925	0.230919
11718972_LPL	-0.10514	0.14393	0.230919
11721035_PPP1R3B	0.1244	0.143935	0.23092
11727912_ANKRD23 /	-0.07441	0.143942	0.230924
11734091_AATK	-0.06553	0.143965	0.230954
11757178_SNORD67	-0.22912	0.144091	0.231148
11736873_ZNF514	0.074291	0.14411	0.231171
11733801_CPPED1	0.084272	0.144117	0.231175
11732591_INSC	0.082125	0.144158	0.231225
11755455_GFAP	-0.10836	0.144158	0.231225
11732279_FOXP1	0.161116	0.144199	0.231284
11734084_SUSD3	-0.08799	0.144218	0.231306
11732141_COX16	-0.05993	0.144226	0.231311
11745057_KLF12	0.088179	0.144243	0.231331
11760927_LRCH3	0.081622	0.144274	0.231373
11733691_SIGLEC7	0.110425	0.144309	0.231422
11748240_NFATC4	-0.08058	0.144327	0.231442
11749182_NCSTN	0.072393	0.144332	0.231442
11746538_SPATA31C1	-0.12504	0.144336	0.231442
11755696_PRDM11	-0.11351	0.144374	0.231496
11717747_MCTS1 ///	-0.07047	0.144404	0.231536
11738665_GPX5	-0.14256	0.14444	0.231585
11741976_OR11A1	0.095206	0.144444	0.231585
11763623_FKBP9	-0.11715	0.144449	0.231586
11759508_PCDH17	0.071921	0.144493	0.231649
200066_PMIK	0.058482	0.144522	0.231688
11760204_CKMT1B	-0.12344	0.144528	0.23169
11759413_CDH11	-0.12903	0.14455	0.231718
11754237_TMEM53	-0.06403	0.144562	0.231728
11726557_GSDMB	0.06924	0.144594	0.231772
11737212_PLEKHA8	0.117342	0.144668	0.231883
11743793_ZNF280B	0.080342	0.144687	0.2319
11735912_WDFY3	-0.0885	0.144689	0.2319
11734462_CCDC105	-0.07253	0.144693	0.2319
11735814_RAD21L1	-0.07173	0.144716	0.231931
11742410_HIST2H3A	0.195143	0.144726	0.231932
11750448_SLC8A2	-0.10793	0.144729	0.231932

11738406_KRTAP1-1	-0.08805	0.144731	0.231932
11741736_CENPN	-0.13145	0.144749	0.231953
11732669_CORT	-0.10694	0.144776	0.231988
11718373_DHRS7B	-0.08372	0.144787	0.231999
11743401_CDC27	-0.05453	0.144794	0.232001
11739751_SLC25A16	0.110458	0.144858	0.232097
11741552_GOLGA6D	0.062751	0.144867	0.232103
11726874_IFT140	-0.06639	0.144916	0.232175
11760497_TBC1D8	-0.07388	0.144971	0.232255
11738816_CDH7	-0.15448	0.14501	0.23231
11746903_DNM1P46	-0.12579	0.14507	0.232398
11738973_DEFB115	-0.09885	0.145165	0.232542
11736122_GOLIM4	0.055743	0.145198	0.232587
11756375_ETV7	-0.07485	0.145219	0.232614
11757137_EIF4A2 //	-0.09161	0.145226	0.232618
11723137_CTXN1	0.05848	0.145254	0.232655
11718223_GSPT1	0.080574	0.14528	0.23269
11753701_MED24	-0.06705	0.145305	0.232721
11719874_PPP2R5B	-0.12155	0.145311	0.232724
11740231_CCNT1	0.061298	0.145395	0.232851
11761286_TMA16	-0.1549	0.145441	0.232917
11751316_EPS8L1	-0.06786	0.14547	0.232955
11741795_TECTA	-0.16783	0.145478	0.232961
11755854_KCNQ5	-0.08418	0.145557	0.233079
11759917_ZNF808	0.099674	0.14562	0.233172
11762667_AP5M1	-0.09527	0.145641	0.233198
11752084_KLHL6	-0.16258	0.145653	0.233209
11723111_EMILIN2	0.08928	0.145661	0.233216
11719166_DAB2IP	0.073413	0.145667	0.233218
11721401_NCOA3	0.121873	0.145731	0.233306
11720718_C22orf29	-0.08166	0.145732	0.233306
11745935_ITIH4	-0.0832	0.14574	0.233311
11762206_DRD5P2 //	-0.11433	0.145746	0.233312
11745681_FUT11	0.089179	0.145799	0.23339
11734432_CELF5	0.155485	0.14583	0.233429
11751228_ZNF76	-0.11891	0.145835	0.233429
11729359_RFTN2	0.095132	0.145838	0.233429
11737357_CNGA3	-0.14232	0.14588	0.233489
11716853_IMPAD1	-0.07098	0.145917	0.233541
11745643_F9	-0.1093	0.145951	0.233588
11733910_UTP15	0.076582	0.145992	0.233643
11758239_C9orf72	-0.06629	0.145995	0.233643
11745741_HBS1L	-0.07959	0.146005	0.23365
11718282_NDUFV2	-0.05594	0.146052	0.233718
11747205_PDK2	-0.08218	0.146088	0.233768
11739649_DLL4	-0.14469	0.146122	0.233815
11759367_KIF18B	-0.09342	0.146132	0.233817
11760511_KIF18B	-0.09342	0.146132	0.233817
11756319_ANO8	-0.10662	0.146138	0.233819
11750158_DTNA	-0.09	0.146163	0.233851
11726569_SPOCK3	0.122937	0.146176	0.233863

11737710_LFNG	-0.07513	0.146195	0.233886
11747718_SPINT1	-0.09768	0.14623	0.233928
11746635_LEF1	-0.15733	0.146231	0.233928
11725225_CHP2	-0.08923	0.146249	0.233942
11745484_PGC	-0.13098	0.146249	0.233942
11761712_FAH	0.103058	0.146268	0.233965
11761602_LILRB3	-0.18609	0.146284	0.233983
11760066_NPSR1-AS1	-0.07734	0.146312	0.23402
11735548_SLC36A2	-0.10177	0.14635	0.234073
11747159_NGFR	-0.06465	0.146419	0.234176
11715217_MOGAT2	0.081827	0.146426	0.23418
11722274_ZBED4	-0.11028	0.146434	0.234185
11743301_SRP72	-0.05778	0.146447	0.234197
11733402_CLEC7A	0.12244	0.146453	0.234199
11722598_ACKR3	-0.10247	0.146462	0.234201
11764048_AASDH	-0.0704	0.146464	0.234201
11737431_PAPL	0.104893	0.146504	0.234257
11741904_IZUMO1R	-0.09387	0.146531	0.234294
11716161_LBH	-0.12352	0.146538	0.234297
11728534_SPSB2	-0.07024	0.14656	0.234324
11744632_COX11	0.084633	0.146565	0.234324
11727564_GYG2	-0.06671	0.146597	0.234369
11759620_ATAT1	-0.06954	0.14662	0.234398
11741045_DGKZ	-0.08543	0.146656	0.234441
11763898_SNORA37	-0.08523	0.146657	0.234441
11760968_NTN5	-0.14272	0.146715	0.234527
11723376_GCOM1 ///	0.116657	0.146722	0.23453
11720144_CCSAP	-0.06815	0.14673	0.234535
11729988_ROBO2	-0.188	0.146802	0.234642
11721489_TRIP10	-0.10098	0.146845	0.234697
11726390_MZF1	-0.0589	0.146846	0.234697
11739691_LAGE3	-0.16552	0.146857	0.234707
11735612_GOLGA8CP	-0.07238	0.146864	0.234711
11748611_CARF	0.132047	0.146882	0.234732
11721166_APOM	0.069086	0.146887	0.234732
11716361_FAF1	-0.06883	0.146894	0.234736
11735629_ANKMY1	-0.10876	0.146924	0.234776
11741969_TRPV5	-0.12116	0.146944	0.2348
11740143_BEND6	-0.11994	0.146959	0.234816
11759053_ZNF333	-0.23168	0.146989	0.234857
11735625_UTY	-0.08588	0.147062	0.234966
11731917_KCNA3	-0.08156	0.147099	0.235016
11727555_GNB5	-0.06155	0.14712	0.235042
11721595_ARHGAP39	-0.11871	0.147138	0.235064
11727892_PRELP	-0.10652	0.147148	0.235068
11755967_KRT25	0.099439	0.14715	0.235068
11724163_HLA-DOB	-0.07915	0.147172	0.235094
11724005_FYB	0.08482	0.147176	0.235094
11739961_VPS36	0.083935	0.147195	0.235116
11745184_MFF	-0.06363	0.147202	0.235119
11750146_TKTL1	0.089852	0.147234	0.235164

11721165_KHNYN	0.074737	0.147254	0.235185
11739266_GPR61	-0.12056	0.147257	0.235185
11724074_SMARCAD1	-0.06734	0.147303	0.23525
11753723_PAX5	0.078312	0.147313	0.235258
11730450_NEURL2	-0.06656	0.147326	0.235272
11738619_SCN10A	-0.11014	0.147391	0.235369
11727193_MMP23A //	-0.0961	0.147526	0.235576
11753286_SETD3	0.083722	0.147615	0.235711
11763533_ZDHHC19	0.087876	0.147705	0.235846
11724877_AEBP2	0.100911	0.147724	0.235868
11734188_GP6	-0.07465	0.147728	0.235868
11733319_TRPC4	0.177084	0.147736	0.235872
11733667_PCBP3	-0.13341	0.147756	0.235898
11728723_VPS29	0.053135	0.147854	0.236046
11754459_FAM184A	-0.15613	0.147863	0.236049
11728123_KCTD13	0.058806	0.147866	0.236049
11762242_PTK6	0.062398	0.14792	0.236129
11762774_BLOC1S1-R	-0.11847	0.147926	0.236129
11730329_DIO3	-0.08552	0.147934	0.236136
11741370_ATP6V1E1	0.061801	0.147971	0.236186
11742495_SFTPA1 //	-0.09415	0.147986	0.236203
11761042_MBP	-0.08749	0.148003	0.236221
11720126_BCAR1	0.066038	0.148011	0.236227
11734453_C11orf86	-0.08765	0.148119	0.236392
11741389_NSFL1C	-0.07325	0.148152	0.236436
11754139_TNFRSF25	-0.09657	0.148232	0.236554
11735015_GPR137	-0.05717	0.148235	0.236554
11745320_ANKRD36	-0.1088	0.148241	0.236556
11740016_SLC6A11	-0.09268	0.148269	0.236593
11734198_LUZP2	-0.07068	0.148309	0.236648
11742356_OR4C11	0.057746	0.148379	0.236753
11762813_PABPN1L	0.153027	0.148415	0.2368
11715688_CTRB2	0.073952	0.148431	0.2368
11717574_PFN1	-0.05553	0.148434	0.2368
11761812_WDR86	0.093555	0.148436	0.2368
11764235_ZHX1-C8or	0.06578	0.148437	0.2368
11742342_OR4A16	-0.1124	0.148438	0.2368
11716413_MT1E	-0.08735	0.148461	0.236829
11747448_BTN3A2	-0.07518	0.148492	0.236871
11753136_ADGRB2	-0.09071	0.148508	0.236883
11749941_FCRL6	-0.09449	0.148509	0.236883
11762827_TAC3	-0.18081	0.148521	0.236893
11715936_NBEAL2	-0.10559	0.148528	0.236898
11753931_ABCC8	-0.11283	0.148538	0.236906
11751503_FGF17	0.097417	0.148559	0.236926
11762013_DHX58	-0.14531	0.14856	0.236926
11730330_DIO3	-0.09192	0.14861	0.236997
11730809_LRRTM2	-0.10091	0.148618	0.237002
11755708_SLC9B1	0.167259	0.148645	0.237038
11727551_ADAMTS10	-0.1018	0.148707	0.237128
11761293_EIF4E	0.054568	0.148807	0.23728

11750502_NXF3	-0.13292	0.148823	0.237297
11715628_FGB	-0.11504	0.148837	0.237313
11732090_IZUMO1	-0.18433	0.148851	0.237327
11718486_EI24	0.057893	0.148903	0.237402
11745522_UNC50	0.07385	0.148916	0.237415
11728927_DDX43	-0.08323	0.148952	0.237465
11725440_LPAR2	0.095076	0.148977	0.237496
11749326_OLFML2B	-0.12107	0.149034	0.237579
11729130_ANAPC16	-0.08757	0.149038	0.237579
11715681_CREG1	-0.08114	0.149077	0.237634
11736624_LRTOMT	-0.09522	0.149097	0.237658
11728099_NXPH3	-0.11439	0.149103	0.237659
11738050_RASGEF1C	-0.10666	0.149109	0.237659
11738038_KLHL38	-0.12753	0.149112	0.237659
11744260_CD63	0.061257	0.149126	0.237673
11751129_MAPK4	-0.05542	0.149141	0.237688
11748233_RBM25	0.060264	0.149149	0.237694
11723326_CA9	-0.11486	0.14916	0.237704
11740858_PKLR	-0.10532	0.149195	0.237751
11745226_SEPT4	-0.12567	0.149207	0.237762
11715707_MGEA5	-0.06842	0.149237	0.237803
11750613_GRIN1	0.080801	0.14929	0.237879
11734003_ACTN2	-0.17215	0.149328	0.237933
11741202_XAF1	0.160342	0.149369	0.23798
11756443_PROM2	-0.11436	0.149371	0.23798
11738355_NR1I3	0.131894	0.149372	0.23798
11755945_YPEL4	-0.13441	0.149437	0.238075
11734218_ZNF681	-0.09247	0.149461	0.238106
11731240_GRHL2	-0.08246	0.149474	0.238119
11724007_ENTPD1	-0.0942	0.149486	0.238131
11761143_TP53	-0.09197	0.149533	0.238196
11752275_SLC15A1	-0.11964	0.149543	0.238206
11725888_MCEMP1	0.084345	0.149592	0.238276
11760054_TIRAP	0.166975	0.149626	0.238321
11735643_RARRES1	-0.0977	0.14964	0.238336
11735604_OSBPL7	0.064023	0.149653	0.238349
11728472_PLPPR5	0.104981	0.149658	0.23835
11737642_RFPL2	-0.07275	0.149686	0.23838
11721191_CHST3	0.070539	0.149687	0.23838
11717785_CLUH	-0.08257	0.149702	0.238396
11744012_WIZ	-0.12026	0.149706	0.238396
11763616_TRBV10-2	-0.13147	0.149783	0.23851
11731555_SYNPO2	-0.1017	0.149837	0.238588
11750025_MYO15A	-0.05972	0.149863	0.238617
11729482_KRTCAP3	-0.07205	0.149865	0.238617
11756084_PHF8	0.076884	0.149873	0.238622
11736030_SLC16A3	0.079601	0.149913	0.238678
11736580_GCNT1	-0.16293	0.149921	0.238683
11755860_MME	-0.07127	0.149931	0.238692
11726338_ALOX5	-0.15898	0.14996	0.238729
11755529_ZNF322	-0.07813	0.149995	0.238778

11734322_BMP3	0.070369	0.150053	0.238862
11760939_SSX2IP	-0.08927	0.150117	0.238956
11738235_GCNT4	-0.13825	0.150129	0.238967
11735529_KLK14	-0.10005	0.150155	0.239002
11740298_LEPROTL1	0.061601	0.150187	0.239044
11724041_SPESP1	-0.08682	0.150217	0.239083
11760293_LOC100129	-0.08382	0.15029	0.239185
11731732_CD1B	-0.09997	0.15029	0.239185
11742593_OR14C36	-0.083	0.150373	0.239309
11763036_ZNF761	0.135156	0.150393	0.239334
11756017_LOC105377	0.107851	0.150404	0.239343
11761462_FABP7	-0.07537	0.150417	0.239355
11728761_LOC100506	-0.06426	0.150426	0.239362
11751602_SRSF7	0.060831	0.150447	0.23938
11739620_VSTM4	-0.14132	0.150447	0.23938
11725943_WNT7B	-0.0782	0.150498	0.239454
11743028_SPIRE2	-0.09532	0.150509	0.239463
11749960_PSMF1	-0.08785	0.150515	0.239465
11731251_EPHA8	-0.11346	0.150598	0.239589
11762668_NAIF1	0.08379	0.150604	0.23959
11754522_SUGT1	0.083772	0.150675	0.239693
11761397_TMEM138	0.061392	0.150678	0.239693
11739572_ZNF532	0.058983	0.150713	0.239735
11731210_C2orf57	0.078035	0.150715	0.239735
11750460_ZFYVE27	-0.06902	0.150727	0.239747
11724714_STK32B	0.070629	0.150733	0.239749
11724341_PRIMA1	-0.13251	0.150739	0.23975
11718822_DOK4	-0.08711	0.150756	0.23977
11734552_CX3CL1	-0.08712	0.150766	0.239778
11752592_CEP170	0.064921	0.15078	0.239792
11762887_DIO3OS	-0.06429	0.150797	0.239813
11732981_USP26	0.099409	0.150809	0.239822
11757834_AFF1	0.066946	0.150854	0.239886
11744633_BRAT1	-0.07112	0.150876	0.239914
11723619_SNAP91	0.107878	0.150885	0.239921
11732787_TXLNGY	-0.05523	0.15089	0.239921
11726036_TRNAU1AP	0.084421	0.150916	0.239954
11733608_LRRC34	-0.12097	0.150954	0.240006
11752778_TTC31	-0.06488	0.150989	0.240054
200081_PMRPS6	0.052764	0.151025	0.240104
11737569_YIPF7	-0.08811	0.151041	0.240121
11750919_ALPL	0.070216	0.151071	0.240162
11738505_KRTAP13-1	-0.13227	0.151099	0.240198
11738513_GADL1	0.098722	0.151117	0.24022
11760383_KIAA0513	0.17856	0.151142	0.24025
11761787_CD1E	-0.14537	0.151153	0.24026
11742583_OR10G3	-0.12256	0.151209	0.240342
11734487_C5orf52	-0.10387	0.151234	0.240372
11728157_EDA	-0.14902	0.151238	0.240372
11733299_CLDN5	0.153775	0.151263	0.240405
11748093_RIMS1	0.212075	0.151405	0.240623

11742357_OR10Q1	-0.09316	0.151528	0.240798
11719500_SMN1 ///	0.097909	0.151529	0.240798
11744803_MDK	-0.06058	0.151533	0.240798
11734216_MPP7	0.114093	0.151535	0.240798
11736744_C1orf168	0.125791	0.15155	0.240813
11731637_GAB1	-0.19282	0.151558	0.240813
11729594_PRPF4	-0.05765	0.15156	0.240813
11731695_CXXC4	-0.1155	0.151582	0.24084
11728409_TRIM31	-0.07705	0.151625	0.240901
11734391_FAM124A	-0.15116	0.151668	0.240962
11762951_MAGEC3	-0.09557	0.151683	0.240978
11750100_ST3GAL3	-0.13672	0.15169	0.240982
11755496_RITA1	-0.067	0.151702	0.240992
11729085_ZFY	-0.09101	0.15177	0.241093
11738172_DIP2A	-0.07121	0.151881	0.241251
11733216_USP53	0.089425	0.151886	0.241251
11733596_TMEM52B	-0.06085	0.151889	0.241251
11733661_C1orf105	-0.07284	0.15189	0.241251
11760898_CCDC40	0.150041	0.151962	0.241358
11731500_KRT19	-0.13155	0.152025	0.241443
11760211_CD48	-0.13415	0.152025	0.241443
11746828_SUMO1	-0.0569	0.152037	0.241453
11727304_TIGD3	0.130109	0.152065	0.241491
11746664_C16orf58	-0.06384	0.152074	0.241497
11727771_KIRREL2	0.105878	0.152091	0.241517
11759918_SPATA6	-0.09264	0.152195	0.241673
11724942_MAPK8IP2	0.10062	0.152201	0.241676
11740199_ARHGDIG	-0.10401	0.152241	0.24173
11748579_COMMD1	-0.08874	0.152271	0.24177
11749093_PAPLN	-0.11644	0.152303	0.241813
11734618_ALOX15B	0.074257	0.152308	0.241813
11763544_ZNF81	-0.10797	0.152313	0.241814
11745866_PSEN2	0.116595	0.152384	0.241918
11721899_FAM216A	-0.06764	0.152394	0.241922
11763501_RBM8A	-0.11694	0.152396	0.241922
11762749_NPSA	-0.10619	0.152457	0.242011
11719668_SMYD5	0.097135	0.152485	0.242045
11721694_WAS	-0.07525	0.152488	0.242045
11745823_ACAA1	-0.10399	0.152498	0.242052
11736151_PRMT7	0.065375	0.15257	0.242157
11753532_KCNIP3	0.134289	0.152574	0.242157
11760652_PNLIPRP1	-0.09781	0.152586	0.242168
11729926_ZNF510	0.083564	0.152606	0.242193
11752917_SLC27A1	-0.08166	0.152622	0.24221
11715893_IFI27	-0.18354	0.152677	0.24229
11741907_DRGX	-0.1666	0.152747	0.242392
11733641_KLC4	-0.07916	0.152752	0.242392
11753238_ANKRD36	-0.11181	0.152757	0.242392
11715539_RPLP1	0.05301	0.152772	0.242408
11722899_REV1	-0.06421	0.152811	0.242457
11761988_GALNTL6	-0.1051	0.152813	0.242457

11736004_PRH2	-0.19813	0.152869	0.242539
11757078_LOC101929	-0.11397	0.152889	0.242563
11761183_ZNF250	-0.13185	0.152895	0.242564
11743199_C8A	-0.0874	0.152947	0.24264
11756848_MCHR1	-0.09529	0.152972	0.24267
11742564_LOC101927	0.127085	0.152984	0.242683
11722878_TBC1D17	-0.08126	0.152998	0.242697
11744419_MFF	-0.08515	0.15301	0.242707
11733853_INTS9	-0.08614	0.153075	0.242803
11756031_KRTAP2-2	0.092241	0.153095	0.242823
11725184_KLF12	0.094443	0.153098	0.242823
11757806_PRDX6	0.055438	0.153167	0.242925
11721720_CELA2A	-0.11731	0.153225	0.24301
11758118_YARS	-0.05472	0.153231	0.24301
11731354_VCX	-0.0989	0.153279	0.243076
11726220_SYPL2	0.124953	0.153282	0.243076
11735365_OCM	-0.10303	0.15331	0.243103
11760756_AMBP	-0.07642	0.153312	0.243103
11745753_KLK3	-0.13197	0.153314	0.243103
11764033_RFC5	-0.07372	0.153337	0.243131
11751198_DIS3L	-0.10123	0.153347	0.24314
11755014_AP2A2	-0.06906	0.153355	0.243145
11753520_EPHB1	-0.12706	0.153371	0.243162
11718973_LPL	-0.06548	0.153379	0.243167
11760797_LOC100499	-0.12363	0.153403	0.243197
11747892_TOMM40L	0.084043	0.153415	0.243207
11731916_KCNA3	-0.15321	0.153481	0.243304
11753871_GNG7	-0.07957	0.153564	0.243428
11735007_FCHSD1	-0.10778	0.153592	0.243465
11760811_PSMC6	-0.17548	0.153611	0.243487
11758782_KLHL9	-0.10669	0.153617	0.243489
11754100_SEC16A	-0.08501	0.153651	0.243535
11752871_ACE	-0.10026	0.153673	0.243561
11762995_TRAV5	0.075055	0.153703	0.2436
11727619_EFNA3	-0.10574	0.153723	0.243624
11755074_PLA2G4F	-0.08789	0.153727	0.243624
11762662_SLC15A1	0.104729	0.153749	0.243651
11723663_HNRNPU	-0.05286	0.153761	0.243662
11741430_KCTD15	-0.09289	0.153863	0.243816
11736948_TEX26	0.079111	0.153883	0.24384
11721605_ZFYVE26	0.078799	0.153913	0.243878
11735626_UTY	-0.10763	0.153948	0.243927
11741861_AGER	-0.06615	0.153989	0.243984
11739901_SLC19A3	0.06777	0.154018	0.244022
11736242_ORMDL2	-0.06478	0.15404	0.244042
11739724_ATP2B2	-0.18424	0.154041	0.244042
11736692_TNFRSF18	-0.09575	0.154066	0.244071
11737544_TMEM202	-0.16513	0.154069	0.244071
11724636_ABCC10	0.063576	0.154078	0.244077
11737921_MILR1	0.080152	0.154083	0.244077
11752151_OR2C3	-0.0799	0.154089	0.244078

11762086_GTPBP8	0.154581	0.154098	0.244084
11720253_TOB1	-0.07231	0.154118	0.244103
11726468_C1orf106	-0.07043	0.154121	0.244103
11755550_POU2F3	0.078359	0.154125	0.244103
11754686_LRRC56	0.076779	0.154143	0.244121
11744508_CCDC146	-0.08383	0.154146	0.244121
11761171_SPHK2	-0.0763	0.154165	0.244138
11734038_ARFGAP2	-0.06016	0.154167	0.244138
11718010_SLC25A22	-0.05726	0.154172	0.244138
11730190_OLA1	-0.09879	0.154185	0.244152
11743317_ZFC3H1	0.059407	0.154241	0.244231
11762066_POLR3A	-0.15083	0.154245	0.244231
11721662_HDAC11	-0.10071	0.154271	0.244264
11762325_GLI2	-0.15439	0.15428	0.244269
11734952_MBNL3	0.095578	0.154306	0.244304
11747036_ZNF385B	-0.09236	0.154326	0.244324
11764236_ZFP41	0.15904	0.154329	0.244324
11726180_SOX6	-0.13965	0.154369	0.24438
11747010_VSIG4	-0.19751	0.154377	0.244384
11715505_IARS	0.056811	0.154426	0.244453
11730518_PLXNB3 //	-0.08894	0.154479	0.244529
11724692_CAPN13	-0.08206	0.154506	0.244564
11743638_DIS3L2	0.076581	0.154513	0.244567
11721374_BCL2	-0.11244	0.154535	0.244595
11747863_MED15	-0.08531	0.154597	0.244684
11737906_FOXO3	0.056284	0.154613	0.244702
11758465_ZBTB41	-0.05528	0.154623	0.24471
11738509_CPSF4L	-0.0776	0.154637	0.244714
11748022_TNNT3	0.113126	0.154638	0.244714
11733236_TAMM41	0.063384	0.154644	0.244714
11752641_PTK7	-0.1835	0.154655	0.244714
11724929_EVI5L	-0.07741	0.154655	0.244714
11751324_HARS	-0.08119	0.154656	0.244714
11760562_ASL	0.134004	0.15467	0.244729
11745973_DLG5	0.073424	0.154731	0.244817
11748145_HNRNPUL1	-0.06167	0.154751	0.244842
11746372_NUDT6	0.071427	0.154791	0.244895
11729010_CDH22	-0.07326	0.154797	0.244895
11762825_PLA2G4C	0.16284	0.1548	0.244895
11726489_SLC4A1	-0.11195	0.154839	0.244949
11750959_FAM129C	-0.08372	0.154851	0.244959
11747808_WIPI1	-0.09163	0.154894	0.245019
11719194_DFNA5	-0.06562	0.154914	0.245044
11740543_ADAMTSL5	0.08328	0.154963	0.245114
11747095_SNCAIP	-0.15915	0.154976	0.245125
11718418_TST	0.066938	0.154993	0.245144
11718842_C16orf62	-0.07024	0.155	0.245147
11748504_PPM1F	-0.06591	0.155019	0.245171
11755646_SIGLEC1	-0.11723	0.15503	0.245179
11715414_COL3A1	0.104903	0.155072	0.245239
11748401_TPM4	-0.06893	0.155098	0.245272

11741593_LENEP	-0.14088	0.155112	0.245286
11761841_HSF4	0.064339	0.155126	0.245299
11727825_TPMT	-0.08191	0.155147	0.24532
11715833_RUVBL1	-0.07528	0.155149	0.24532
11730511_MAMSTR	-0.10267	0.155171	0.245347
11764072_GPR17	0.145125	0.155185	0.245362
11755194_CCNL2	-0.14863	0.155194	0.245367
11759949_COBL	-0.17778	0.1552	0.245369
11748644_BCAR1	0.084225	0.155257	0.245451
11757188_TMEM254-A	-0.12959	0.155284	0.245486
11742296_POU1F1	-0.12626	0.155291	0.245489
11746290_SLC12A5	-0.10158	0.155306	0.245504
11734303_SPTBN4	-0.07974	0.155325	0.245527
11735994_USP14	0.054873	0.155403	0.245643
11734370_CTAGE1	-0.07265	0.155429	0.245675
11763068_BCL2L11	-0.1348	0.155451	0.245702
11737530_STOX1	-0.18214	0.155458	0.245705
11760571_LOC100506	-0.14942	0.155496	0.245757
11717131_AHNAK2	0.125149	0.155505	0.245764
11733592_LCA5	0.116839	0.155512	0.245764
11724795_ZG16B	-0.12406	0.155515	0.245764
11763085_RPS15	-0.13555	0.155523	0.245768
11742670_CXCL6	0.088793	0.155592	0.245862
11761375_TRMU	-0.10904	0.155595	0.245862
11751188_SYT4	-0.07599	0.155598	0.245862
11757678_EEF2	0.053126	0.155604	0.245865
11739676_PRR27	0.098041	0.155631	0.2459
11739102_SYF2	0.076347	0.155655	0.24593
11756367_SLC25A4	0.079477	0.155678	0.245957
11750247_GPER1	-0.18174	0.155701	0.245986
11746830_AGR2	0.074733	0.155711	0.245994
11719455_BBS1	0.069584	0.155719	0.245996
11739059_OCIAD1	0.057232	0.155723	0.245996
11732543_RSPH6A	-0.10438	0.155744	0.246021
11736168_CCDC34	-0.14549	0.155773	0.24606
11734275_HNF1A	-0.194	0.155809	0.246104
11758702_PSMD2	-0.0598	0.155815	0.246104
11754555_CEP68	-0.07452	0.155816	0.246104
11740795_SPTBN4	-0.08833	0.155821	0.246104
11736857_ODF1	-0.08851	0.155836	0.24612
11750470_EVX1	-0.06042	0.155856	0.246143
11716400_TP53INP2	-0.0764	0.155861	0.246143
11737227_PPP1R27	-0.09858	0.15587	0.246149
11717579_VPS26A	0.069982	0.155878	0.246154
11759393_KRTAP19-2	-0.17223	0.156058	0.246431
11757665_NDUFS5	-0.05723	0.156066	0.246435
11720142_ASTN1	-0.12008	0.156091	0.246461
11740599_GPR20	-0.11739	0.156092	0.246461
11759589_DSCR9	-0.07924	0.156101	0.246466
11741074_METTL18	0.081194	0.156109	0.24647
11719862_TREM1	-0.0874	0.156113	0.24647

11748109_FAM69A	-0.12744	0.156158	0.246532
11746341_CLTC	-0.07615	0.156163	0.246533
11735988_DEFB136	-0.13981	0.156193	0.246571
11747194_EVC	-0.13434	0.1562	0.246571
11731165_FGF19	-0.14509	0.156203	0.246571
11742624_OR5AC2	-0.10395	0.156219	0.246589
11742311_DUX1	-0.06961	0.156238	0.246609
11730633_MAK	-0.05815	0.156242	0.246609
11752533_DCST1	-0.06847	0.156248	0.246611
11761586_RHBG	-0.06702	0.156277	0.246649
11726844_PLXDC1	-0.10888	0.156295	0.246665
11725000_DUOXA2	-0.09794	0.156298	0.246665
11755589_DHRS2	0.07453	0.156357	0.246751
11738723_OR2Z1	-0.07651	0.156379	0.246771
11716047_C21orf33	-0.06636	0.15638	0.246771
11729999_B3GNT7	0.070535	0.156407	0.246806
11734598_SLC25A52	-0.08413	0.156457	0.246878
11762725_SIGLEC8	-0.11004	0.156493	0.246925
11759682_PDLIM5	-0.13138	0.156509	0.246938
11751489_TSPAN7	0.108908	0.156511	0.246938
11717176_SNX27	0.057532	0.156517	0.24694
11759321_MPZL3	0.082144	0.156535	0.24696
11736781_RAB11FIP1	0.07287	0.156551	0.246978
11737154_CRYGB	-0.16605	0.156582	0.247019
11754391_CCDC51	0.056327	0.156626	0.247072
11720438_ACVR1B	-0.06808	0.156626	0.247072
11762382_PER2	0.060764	0.156679	0.247148
11733114_MANBAL	0.052108	0.156691	0.247159
11735983_OR10H3	-0.06348	0.1567	0.247165
11757012_RHOA	0.069493	0.156727	0.247196
11752879_SMTN	-0.07961	0.156734	0.247196
11752925_NPY5R	-0.09065	0.156735	0.247196
11730519_ABCA9	-0.08035	0.156755	0.247219
11745688_AVPR2	-0.08548	0.156798	0.247279
11727974_FAM171A2	-0.06364	0.15683	0.247321
11735099_C2orf73	0.103649	0.15684	0.247325
11763225_IGLV1-41	0.155043	0.156842	0.247325
11733252_UACA	-0.08436	0.156906	0.247418
11748704_C1D	-0.07168	0.156944	0.247469
11753040_MECOM	0.116292	0.15695	0.247472
11743661_MBNL1	0.083768	0.156957	0.247474
11721670_RPS6KA4	-0.06281	0.156983	0.247507
11723827_TOP3B	0.061851	0.156992	0.247513
11719576_AMT	0.071831	0.156997	0.247513
11738757_OR2A25	-0.10689	0.157139	0.247729
11744732_ZNF610	0.16395	0.157155	0.247746
11758065_KCNIP4	-0.09674	0.157198	0.247804
11723780_DYRK1B	-0.08497	0.157202	0.247804
11751403_ABI2	-0.05432	0.15726	0.247884
11758549_POM121L2	-0.1159	0.157262	0.247884
11731553_SYNPO2	-0.07416	0.157292	0.247922

11751788_DBF4B	-0.08429	0.157343	0.247995
11728546_BTBD9	-0.1065	0.1574	0.248076
11724885_CLIC3	-0.11277	0.15747	0.24818
11754414_C1orf159	-0.07	0.157503	0.248215
11745712_ZNF365	0.153523	0.157503	0.248215
11749209_IKZF1	-0.10828	0.157516	0.24822
11732094_PYY	0.073441	0.157518	0.24822
11727075_PLAC4	-0.07139	0.157521	0.24822
11762841_CYP4Z2P	-0.151	0.157529	0.248223
11738296_CNGA2	-0.12447	0.157558	0.248261
11754692_AFG3L2	-0.07006	0.157576	0.248282
11718660_NENF	0.05801	0.157583	0.248285
11753783_CIB3	-0.09309	0.157659	0.248396
11731020_CCDC62	0.100179	0.157672	0.248409
11748346_FKBP9 ///	-0.13987	0.157701	0.248446
11725439_HBD /// H	-0.1021	0.157706	0.248446
11727728_DMRT2	-0.06531	0.157713	0.24845
11762639_DNAJA1P5	0.093768	0.157763	0.248521
11728585_FAM189A1	0.162876	0.1578	0.248571
11732475_C14orf180	-0.12188	0.157817	0.24859
11737727_XKR6	0.128003	0.157822	0.24859
11740153_ZNF384	0.118957	0.157853	0.24863
11729295_TANGO6	0.083476	0.157947	0.24877
11762374_COL25A1	0.141079	0.157981	0.248816
11749231_CAMKV	-0.08459	0.158072	0.248951
11735906_PNPLA1	-0.06588	0.158086	0.248965
11732235_GYPA	-0.07601	0.158094	0.248969
11724256_OAS1	-0.11174	0.158104	0.248977
11719745_ARHGAP27	0.074483	0.15813	0.249002
11731097_TTC16	-0.06137	0.158132	0.249002
11728201_LOX	0.063242	0.158135	0.249002
200031_PMRPS11	0.051913	0.158158	0.249031
11722545_EYA2	-0.13657	0.15817	0.249041
11762868_LOC100289	-0.08508	0.158183	0.249053
11759955_CNOT10	-0.12644	0.158238	0.249132
11726562_SAP18	-0.08491	0.158336	0.249279
11736890_PTPRD	0.12221	0.15835	0.249293
11752381_TRIM67	-0.08177	0.158376	0.249325
11715185_PRB1	-0.07291	0.158426	0.249382
11754450_MTCL1	0.083409	0.158431	0.249382
11760842_CTAGE3P	-0.08484	0.158432	0.249382
11761784_HPX	-0.13051	0.158432	0.249382
11758077_BHMT	-0.06149	0.158439	0.249384
11734053_ZMYM6	0.1248	0.158452	0.249396
11745237_B3GNT3	-0.09707	0.158465	0.249408
11730486_SERPINA9	-0.13954	0.158469	0.249408
11747644_LIMS1 ///	0.26139	0.158489	0.249431
11732358_ZNRF1	0.052718	0.158561	0.249536
11723728_CXorf57	-0.0851	0.15859	0.249575
11743384_PRPF40A	-0.13471	0.158682	0.24971
11745403_ZNF683	-0.08211	0.158711	0.249748

11752066_RAI1	-0.11668	0.158735	0.249778
11729325_RAMP2	-0.08741	0.158751	0.249795
11724114_UCKL1 ///	0.076138	0.158801	0.249866
11748760_CLEC4F	-0.10335	0.15886	0.249951
11728883_PRSS35	-0.06266	0.158901	0.250007
11742396_PRSS42	-0.08421	0.158924	0.250035
11739810_SIRT3	0.054032	0.158929	0.250035
11716672_PIGY ///	-0.07198	0.158955	0.250067
11729376_TAC3	-0.14535	0.158984	0.250106
11716523_S100A9	0.055313	0.159036	0.250179
11759418_CEMP1	-0.12347	0.159042	0.250179
11762582_GINS4	-0.17978	0.159046	0.250179
11724704_ST8SIA3	0.187064	0.159055	0.250186
11729467_RNF17	0.071493	0.159065	0.250193
11739302_FZD6	-0.05164	0.159124	0.250278
11752501_ACKR3	-0.08634	0.159145	0.250299
11747875_FNBP1L	0.07148	0.159148	0.250299
11720881_EHD4	-0.09048	0.159168	0.250307
11743027_DPCR1	0.121647	0.159174	0.250307
11741071_ANK1	0.228115	0.159176	0.250307
11728267_LILRB1 //	-0.11288	0.159176	0.250307
11734398_PRSS55	-0.12658	0.159179	0.250307
11746362_SPNS3	-0.07379	0.159194	0.250323
11736852_LRRC19	-0.08331	0.159216	0.25035
11757689_MIR7-3HG	0.073209	0.159234	0.250369
11721747_ANKRD12	0.07634	0.159251	0.250388
11740452_KLRD1	0.062844	0.159268	0.250406
11724764_TFCP2	0.071614	0.159272	0.250406
11741130_NLGN4X	-0.06205	0.15936	0.250533
11726707_IL34	0.060959	0.159363	0.250533
11727334_ALPPL2	-0.07768	0.159397	0.250578
11725859_APBA1 ///	-0.09898	0.159412	0.250594
11760865_MLH1	-0.15202	0.159431	0.250615
11726064_RPE	0.064406	0.159495	0.250708
11747574_C1orf109	0.067766	0.159512	0.250726
11733193_GTF2A1L /	0.094314	0.159591	0.250842
11730256_EPN3	-0.0802	0.159607	0.250859
11730654_C15orf62	0.085307	0.159614	0.250863
11752183_DNAJA3	0.052847	0.159649	0.250901
11724303_COG8 ///	0.07129	0.159649	0.250901
11738013_CCDC7	0.092746	0.159663	0.250915
11730715_CSAG1	0.065399	0.159714	0.250988
11756660_PROM1	-0.14942	0.159748	0.251033
11735322_MGAT5B	-0.12788	0.159802	0.25111
11727600_TNFRSF4	-0.06445	0.159859	0.251191
11738929_OR9G1 ///	-0.12641	0.159879	0.251215
11760848_ECE2	-0.15218	0.159898	0.251236
11755958_ZNF691	0.061328	0.159904	0.251238
11717461_NSUN2	-0.08084	0.15991	0.251239
11763459_CIDECP	0.10433	0.159918	0.251239
11744726_KLHL9	0.053493	0.15992	0.251239

11730454_GLYCTK	-0.19539	0.159932	0.251249
11746682_AACS	0.070088	0.159946	0.251259
11750227_RSAD1	-0.06535	0.159949	0.251259
11752512_AFF1	0.081125	0.159982	0.251303
11730060_LAG3	-0.13333	0.159999	0.251322
11728148_PRSS50	-0.12001	0.160054	0.251394
11735109_FBXO3	0.052975	0.160055	0.251394
11736066_HYPK ///	0.107456	0.160085	0.251434
11723303_PDZK1IP1	-0.08115	0.160094	0.251439
11743463_CHD4	-0.0625	0.160102	0.251444
11747756_KRT13	-0.11395	0.160109	0.251446
11719125_BRAT1	-0.07294	0.160122	0.251458
11761414_ORM1	-0.16305	0.160128	0.251459
11730619_ULBP1	-0.12245	0.160132	0.251459
11741078_NXPE3	-0.11658	0.160153	0.251482
11760893_SERGEF	-0.08158	0.160175	0.251501
11762155_CACNB2	-0.14008	0.160175	0.251501
11738359_LOC101805	-0.08994	0.160182	0.251504
11746596_NADK	-0.06644	0.1602	0.251525
11753573_EMC4	-0.05803	0.160269	0.251625
11763698_MBIP	-0.05756	0.160293	0.251655
11740131_KANSL1L	-0.07764	0.160315	0.251681
11732183_SLC35E4	0.065141	0.160351	0.25173
11738370_SGK2	-0.07409	0.160401	0.2518
11754841_AHRR	-0.08852	0.160474	0.251906
11748643_MYO15A	-0.14828	0.160503	0.251943
11726026_STAG3L4	0.09248	0.160508	0.251944
11759036_STT3B	-0.05662	0.160535	0.251977
11758681_ASCC1	0.060939	0.16066	0.252166
11740254_UBE2NL	-0.14501	0.160707	0.252231
11738340_LRRC74A	-0.08837	0.160732	0.252263
11722856_PDGFD	0.089398	0.160827	0.252404
11752406_SPATA13	-0.05989	0.160839	0.252405
11744120_RPL8	0.054188	0.160843	0.252405
11743220_NKX6-1	-0.10988	0.160844	0.252405
11758464_NAT16	-0.11641	0.16088	0.252454
11724455_ZDHHC1	-0.11864	0.16092	0.252509
11738983_MAP1LC3B	0.059848	0.160925	0.252509
11715106_CTAGE15 /	-0.13864	0.160967	0.252566
11762851_HLA-B	-0.0863	0.160988	0.252592
11759605_WFDC2	-0.07521	0.161045	0.252672
11730210_TRIM46	-0.07942	0.161072	0.252707
11729280_FAM175A	0.062645	0.161083	0.25271
11755162_ZNF620	0.072733	0.161084	0.25271
11745343_FAM53B	-0.10752	0.16109	0.252711
11729246_GGCX	0.078792	0.161151	0.252789
11760739_LOC100289	-0.10633	0.161154	0.252789
11740921_NKAIN3	-0.07163	0.161157	0.252789
11755271_ZNF626	0.084439	0.161161	0.252789
11742494_SFTPA1	-0.10945	0.161195	0.252836
11735151_NXPE3	-0.10368	0.161208	0.252848

11735264_CLEC4D	-0.11178	0.161225	0.252866
11752566_WWP2	0.068517	0.161245	0.252888
11735779_CD101	-0.10783	0.161296	0.252961
11752763_SLC25A35	-0.10729	0.161355	0.253046
11761315_ORC3	-0.06155	0.161365	0.25305
11738395_PCDH11X	0.081368	0.161368	0.25305
11730577_ZNF100	-0.06269	0.161376	0.253054
11737397_LRRC52	-0.12204	0.161392	0.253071
11749052_POU2F2	0.069285	0.161434	0.253128
11728980_KCNK12	-0.14686	0.161472	0.25318
11751341_PRAMEF1 /	-0.07766	0.161503	0.253221
11758913_PCSK5	-0.07542	0.161534	0.253253
11731323_AKAP14	0.101767	0.161534	0.253253
11737988_ECT2L	-0.1248	0.161554	0.25327
11740424_PPP4R4	-0.09398	0.161555	0.25327
11745499_IRF3	0.073119	0.161568	0.253281
11740425_PPP4R4	0.182189	0.161577	0.253288
11759863_PLB1	0.101591	0.1616	0.253316
11745583_BTBD18	-0.17193	0.161611	0.253325
11728351_EFEMP2	-0.056	0.161618	0.253327
11749290_SH3GL1	-0.07912	0.161666	0.253395
11717523_TMEM50B	-0.07721	0.161689	0.253423
11735000_SIPA1L3	-0.06928	0.161698	0.253429
11753110_SNX32	-0.06611	0.161714	0.253446
11731675_AGMAT	-0.08564	0.161737	0.253474
11717248_SARAF	0.063984	0.161749	0.25348
11729625_DCAF7	0.06008	0.161751	0.25348
11751162_TTYH1	-0.05937	0.161765	0.253494
11762124_GLS2	0.100883	0.161806	0.253549
11758743_RNF40	-0.08936	0.161835	0.253586
11738862_GJA10	0.108358	0.161856	0.253605
11762358_CHD2	-0.09294	0.161857	0.253605
11743545_QDPR	-0.05444	0.16187	0.253617
11735417_IL36RN	-0.11345	0.161916	0.25368
11758338_CRISPLD2	-0.12884	0.161935	0.253702
11723674_PSEN2	0.094403	0.161961	0.253735
11759791_FILIP1	0.096481	0.161996	0.253781
11757002_CHRDL2	-0.07286	0.162006	0.25379
11756075_BLK	-0.10218	0.162015	0.253795
11731691_TTC30B	0.117757	0.162022	0.253798
11759481_COPB1	-0.10076	0.162052	0.253837
11720883_UBALD1	-0.0581	0.162066	0.253851
11752933_FCRL2	-0.08677	0.162082	0.25386
11742579_OR9I1	-0.1703	0.162082	0.25386
11747923_LAMC2	-0.0987	0.1621	0.25388
11723558_MORF4L2	0.052846	0.16213	0.253919
11729724_ZBTB6	-0.05552	0.162148	0.253939
11727087_PTS	-0.06632	0.162193	0.254002
11722392_TMEM126B	0.062759	0.162234	0.254057
11747172_SPOCK3	-0.12225	0.162247	0.254069
11738031_FAM153C	-0.09899	0.16226	0.254081

11735969_NKPD1	-0.08635	0.162315	0.254159
11761598_MROH7	0.057589	0.162346	0.254199
11721102_SGSH	0.068092	0.162386	0.254255
11761650_CCDC129	0.161142	0.162421	0.254301
11731105_NR5A1	-0.0944	0.162431	0.254308
11722388_MTF1	-0.05909	0.162445	0.254323
11727080_NUFIP1	0.062867	0.162465	0.254345
11715637_UGP2	-0.05794	0.162482	0.254364
11755765_TUBGCP6	-0.06575	0.162491	0.25437
11735379_CEP162	0.075145	0.162527	0.254418
11758774_HACD2	-0.09555	0.162534	0.254421
11737236_IGLON5	-0.08298	0.162562	0.254456
11734701_DKK3	-0.07226	0.162673	0.254621
11715371_UBA1	0.057183	0.162695	0.254648
11737408_TIRAP	-0.08884	0.162702	0.254651
11739502_ABCA1	-0.08261	0.162728	0.254683
11750631_MAP3K9	-0.09476	0.162773	0.254746
11743422_BAG4	0.089491	0.162784	0.254754
11758553_DCT	-0.0944	0.162795	0.254764
11726434_CHL1	-0.10509	0.162814	0.254785
11740301_USP44	0.179929	0.162869	0.254859
11748065_LRRC49	0.081644	0.162872	0.254859
11745395_MYO5B	0.086203	0.162926	0.254918
11737520_KCNC1	-0.14585	0.162929	0.254918
11740009_CHRFAM7A	-0.06553	0.162931	0.254918
11732643_OR2C1	-0.08097	0.162939	0.254918
11752128_IARS	0.052166	0.162942	0.254918
11759581_HLA-B ///	0.117765	0.162945	0.254918
11761441_CEP78	-0.17317	0.162946	0.254918
11724947_MAGI2	-0.06164	0.162966	0.254942
11756845_LINC00176	-0.12879	0.163032	0.255037
11715676_NCSTN	0.054731	0.163069	0.255087
11740365_ZSWIM2	-0.0781	0.163081	0.255097
11736172_KIAA0355	0.107117	0.163196	0.255269
11754909_C2orf88	-0.0829	0.163246	0.255339
11730889_USF2	-0.06814	0.163268	0.255365
11734237_OXLD1	-0.06844	0.16328	0.255376
11738944_STARD6	0.126142	0.163308	0.255412
11718609_VPS72	0.05991	0.163455	0.255634
11739160_IP6K1	-0.09542	0.163513	0.255716
11721200_TRIM62	-0.07262	0.163549	0.255764
11743393_MATR3	-0.05698	0.163558	0.255767
11729301_GLYAT	0.093084	0.163562	0.255767
11731964_PODNL1	-0.11748	0.163573	0.255769
11727874_ZFYVE21	0.054099	0.163578	0.255769
11739453_ME2	0.062131	0.163579	0.255769
11734639_OR1A1	-0.11953	0.16361	0.255809
11756889_SNAP91	0.102271	0.16364	0.255849
11763662_FMNL1	-0.06598	0.16365	0.255856
11761996_DGKZ	-0.06293	0.163655	0.255856
11738649_KCNA10	-0.22297	0.163669	0.25587

11721689_ID4	-0.1077	0.163675	0.255871
11754848_ZNF169	-0.11378	0.163681	0.255872
11735136_TLL2	0.110538	0.163689	0.255876
11731653_IVL	0.065219	0.163755	0.255971
11750296_CLEC11A	-0.07336	0.163779	0.256
11741385_IP6K1	-0.05608	0.163784	0.256
11743126_EIF3G	0.05404	0.163824	0.256055
11732869_BTBD16	-0.14021	0.163841	0.256072
11759200_CNTN1	-0.08555	0.163884	0.256133
11744625_NABP2	0.060097	0.16394	0.256212
11741328_GAGE1 ///	-0.10386	0.163975	0.256257
11733257_CFAP36	0.077453	0.163985	0.256265
11732115_KRTAP19-5	-0.11437	0.164019	0.256311
11762811_FAM163A	-0.10266	0.164025	0.256311
11748170_TRPM2	-0.06839	0.164059	0.256356
11750455_ETV7	-0.06617	0.164092	0.2564
11760665_ZKSCAN1	-0.09279	0.164113	0.256411
11744102_ARSE	0.089839	0.164114	0.256411
11763418_C22orf29	-0.11344	0.164115	0.256411
11746003_POLDIP2	-0.05098	0.164168	0.256485
11752337_SLCO5A1	-0.07408	0.164173	0.256485
11750963_JPH2	-0.11743	0.164187	0.256499
11723087_GAS2L1	-0.07188	0.164205	0.256518
11734246_SLC5A11	-0.13375	0.164253	0.256582
11751470_PLEKHS1	-0.0751	0.164259	0.256582
11716222_RNASE1	-0.10649	0.16427	0.256582
11750153_GIMAP6	-0.26254	0.16427	0.256582
11736039_KIF1A	-0.07662	0.164272	0.256582
11739909_CDK15	-0.07429	0.164335	0.256668
11727801_POP4	0.091118	0.164337	0.256668
11734236_C16orf90	-0.07565	0.164349	0.256678
11735649_LOC284009	-0.15626	0.164356	0.256681
11730085_C10orf62	-0.05726	0.164412	0.256761
11738921_NFE4	-0.16662	0.164468	0.256839
11755654_PPP2R2C	-0.11889	0.164491	0.256866
11721303_CLEC2B	-0.06716	0.164499	0.256871
11749811_LUC7L3	-0.06057	0.164549	0.256941
11720974_ASB8	-0.09296	0.164578	0.256979
11729847_CCL7	-0.07949	0.164657	0.257093
11759819_ABCA6	0.170895	0.164677	0.257117
11725068_CAMK2B	-0.0756	0.164692	0.257132
11756748_LIPC	0.136623	0.164723	0.257171
11723550_ABHD6	0.067428	0.164774	0.257243
11727042_TTI2	0.056915	0.16479	0.257246
11758586_GGT6	-0.08519	0.164791	0.257246
11745002_FEZF1	-0.06392	0.164792	0.257246
11724828_CCL20	0.141345	0.164799	0.257247
11759055_TMEM72	-0.13426	0.164803	0.257247
11762923_COX3 ///	-0.0673	0.164826	0.257275
11725059_GNG3	-0.06546	0.164836	0.257282
11736614_AVPR1A	-0.08781	0.164854	0.257302

11732750_MEGF11	-0.08043	0.164868	0.257315
11744658_NRF1	-0.09112	0.16495	0.257428
11736298_LPAR5	-0.07171	0.16495	0.257428
11728771_CLCA4	0.080548	0.164961	0.257436
11724235_HGSNAT	0.066576	0.164983	0.257463
11742506_OR52R1	-0.07263	0.165035	0.257536
11733356_C5AR1	0.104976	0.165075	0.257577
11715981_VPS11	-0.06699	0.165075	0.257577
11737791_APOL1 ///	0.106387	0.165077	0.257577
11755488_AQP6	0.096276	0.165113	0.257624
11726490_SLC4A1	-0.20191	0.165135	0.257651
11733843_RBM17	-0.07843	0.165178	0.257707
11743236_PRSS48	0.115781	0.165182	0.257707
11722345_CELSR3	0.057319	0.165204	0.257733
11737013_APLF ///	-0.13496	0.165221	0.257752
11721531_AQP5	-0.09015	0.165231	0.257758
11742371_OR2AG1	0.064967	0.165253	0.257785
11758210_TRMT10C	-0.0573	0.165288	0.257831
11742474_CFAP157	-0.11446	0.165316	0.257862
11741858_NYX	-0.07266	0.165318	0.257862
11717349_RBP1	-0.1163	0.165338	0.257879
11760397_SARS2	-0.08605	0.165339	0.257879
11750653_SEC16B	-0.09481	0.165381	0.257936
11751649_EFHD1	-0.10834	0.165418	0.257985
11755916_OCRL	-0.05489	0.165424	0.257986
11748538_GUSBP2	0.105065	0.165434	0.257988
11732018_HMGCLL1	0.110653	0.165436	0.257988
11731720_LURAP1	-0.06759	0.165451	0.258003
11763192_GDPD1	-0.07047	0.165478	0.258037
11716394_ADH5	-0.06435	0.165501	0.258057
11738560_OR10W1	-0.11368	0.165501	0.258057
11762853_SPATA21	-0.18371	0.165523	0.258083
11755839_ARFGEF3	0.149758	0.16555	0.258117
11733244_DTNBP1	-0.08364	0.165568	0.258136
11748138_SEC31B	-0.06337	0.165577	0.258142
11717798_ZER1	-0.07587	0.165651	0.258249
11735915_TREML4	-0.09299	0.165707	0.258322
11725539_CACNA1H	-0.10171	0.165708	0.258322
11747649_GPR132	-0.11517	0.165731	0.258346
11762846_IYD	-0.09025	0.165734	0.258346
11734197_LUZP2	-0.14057	0.165771	0.258396
11748616_PLD2	-0.09418	0.16578	0.258402
11737951_ZNF623	-0.1025	0.165803	0.258428
11735412_RFESD	0.125751	0.165838	0.258476
11733583_PSORS1C2	0.143791	0.165864	0.258508
11728225_UBE2J1	-0.06938	0.165879	0.258523
11757912_ACADL	0.142398	0.1659	0.258539
11753456_PDDC1	0.072682	0.1659	0.258539
11720425_PENK	0.182721	0.165927	0.258572
11752236_SPATA5	-0.11309	0.165935	0.258577
11717938_SMIM3	-0.08177	0.165955	0.2586

11755124_SLCO3A1	-0.09374	0.165963	0.258603
11740017_INSR	-0.07898	0.165975	0.258614
11748478_TAB1	-0.06695	0.16599	0.258629
11749612_DVL2	-0.11637	0.166015	0.258652
11734296_P2RY14	0.117941	0.16602	0.258652
11728924_NFAM1	-0.06089	0.166023	0.258652
11730246_FMO2	-0.13375	0.166026	0.258652
11731271_MGAM	-0.11336	0.166044	0.258672
11718243_ITGA7	-0.09462	0.166068	0.258701
11738401_GLP1R	-0.06979	0.166111	0.258754
11738434_RNASE9	0.102874	0.166113	0.258754
11746916_SNX7	0.065152	0.166118	0.258754
11759648_CREB3L2	0.121043	0.166131	0.258767
11755794_CAPN14	0.265432	0.166145	0.25878
11715128_PCDHGB5	-0.08542	0.166155	0.258787
11741989_P2RX2	0.075783	0.166203	0.258853
11720074_EIF3B	0.057218	0.166209	0.258856
11753199_SMARCA2	0.079991	0.166264	0.258932
11759109_KRIT1	0.059812	0.166294	0.25897
11749561_LOC388882	-0.12941	0.166338	0.259031
11720984_NPTXR	-0.08356	0.16636	0.259058
11742666_RPS28	0.051263	0.166367	0.259059
11719682_NSA2	0.055632	0.166388	0.259084
11763509_CD9	-0.11216	0.166398	0.259091
11740605_TLX2	-0.05952	0.166451	0.259165
11735092_SLC16A6	0.098021	0.166456	0.259165
11726152_ZBTB21	0.10363	0.166499	0.259223
11761771_ALDH1L1	-0.05173	0.166518	0.259246
11715929_SH3GL1	-0.05739	0.166534	0.259256
11752249_MCTP2	0.182246	0.166536	0.259256
11743599_OTUD5	-0.07488	0.166585	0.259324
11735101_FAM228A	-0.0786	0.166622	0.259375
11732716_C1QTNF9B-	-0.09119	0.166662	0.259429
11754736_EIF4E1B	-0.09802	0.166694	0.259469
11736544_MACC1	-0.06388	0.166825	0.259659
11746499_GSTO2	-0.09281	0.166826	0.259659
11760374_CYP19A1	-0.10209	0.166839	0.259671
11717291_MAP2K1	-0.05264	0.166845	0.259671
11747974_MASP1	-0.11259	0.166873	0.259702
11741964_VEGFA	-0.05413	0.166875	0.259702
11753826_CTLA4	-0.08025	0.166889	0.259715
11751159_PER3	-0.16968	0.166939	0.259777
11763554_GLTSCR2	-0.08991	0.166948	0.259777
11715111_CGB /// C	-0.08253	0.16695	0.259777
11741590_ICAM4	-0.07313	0.16695	0.259777
11762000_CDHR1	-0.12134	0.166979	0.259814
11743433_PGLS	-0.05939	0.166988	0.259817
11726163_EPHA7	-0.08498	0.166991	0.259817
11747273_BAIAP3	-0.1445	0.167009	0.259837
11739924_ZNF37A	0.080941	0.167047	0.259887
11735126_N4BP3	-0.06094	0.167054	0.259889

11764016_TRAPPC2L	-0.10881	0.167076	0.259916
11753119_CMTM5	-0.10834	0.167146	0.260016
11741557_PCDHGA9	-0.09463	0.167175	0.260053
11732238_TMEM259	-0.06462	0.167191	0.260065
11744846_LCA5L	0.105545	0.167193	0.260065
11727723_DDN	0.104313	0.167219	0.260097
11760220_NUTF2	0.060672	0.167227	0.260101
11755237_PPM1M	-0.06816	0.167249	0.260126
11728010_PRTG	0.087549	0.167316	0.260223
11742241_IKZF3	-0.05528	0.167384	0.26032
11758095_FPR1	-0.11784	0.167392	0.260324
11747176_VWF	-0.08273	0.1674	0.260329
11748482_SRP54	0.057725	0.167426	0.26036
11763780_MBNL3	-0.09185	0.167433	0.260363
11730589_KCTD21	0.069879	0.167453	0.260386
11724981_PCDH7	0.107419	0.167521	0.260483
11749748_SNW1	0.05651	0.167535	0.260498
11741899_FCGR2B	-0.10242	0.167558	0.260525
11733190_ARSJ	-0.07189	0.167627	0.260623
11723384_PRR3	-0.09124	0.167637	0.260631
11716695_TSPAN6	0.053383	0.167684	0.260696
11738189_TP73	-0.10194	0.167704	0.260715
11758908_ZNF827	0.060022	0.167707	0.260715
11738898_CLEC4C	-0.10197	0.167741	0.260759
11726859_ZC3H12B	-0.07277	0.167819	0.260871
11749849_CTNS	-0.10818	0.167838	0.260894
11721058_ZNF189	-0.0579	0.167855	0.260896
11719245_LARGE	-0.1419	0.167855	0.260896
11742947_GTF2H3	0.05354	0.167856	0.260896
11728147_AQP11	0.076266	0.167914	0.260978
11749284_LOC100287	-0.15147	0.167922	0.260982
11758423_ATP13A5 /	0.049591	0.167928	0.260983
11726816_GPRIN1	-0.13401	0.16795	0.261009
11761196_SPNS1	-0.11917	0.167957	0.261012
11732552_ENKUR	-0.0849	0.167976	0.261033
11728096_STPG1	-0.09371	0.167993	0.261052
11751988_NFE2L1	-0.06346	0.168045	0.261122
11741932_KCNRG	-0.13772	0.168052	0.261122
11762274_HLA-DMB	-0.11314	0.168057	0.261122
11728191_CXCR4	-0.09307	0.16806	0.261122
11738341_MAGEA10-M	-0.14681	0.168067	0.261125
11761456_AKAP13	0.184272	0.168075	0.261129
11736460_RNF168	0.128536	0.168089	0.261142
11743365_EIF5B	0.094635	0.168114	0.261173
11749299_WDR77	0.05362	0.168165	0.261245
11756668_FAM213A	-0.05461	0.168177	0.261254
11745576_KIRREL3-A	-0.10301	0.16821	0.261296
11736006_MAPK1IP1L	-0.05627	0.168215	0.261296
11757193_JMJD7-PLA	-0.06843	0.168229	0.26131
11745093_LMOD2	-0.09891	0.168279	0.26138
11754925_SYK	-0.11671	0.168336	0.261459

11729891_ZFP3	-0.10613	0.16835	0.261474
11750191_PIGT	-0.06287	0.168395	0.261535
11726172_SCARA3	-0.10873	0.168429	0.261579
11762337_SNX32	-0.13868	0.168436	0.261581
11737857_DNAAF1	-0.11592	0.168448	0.261587
11760941_PLEKHM1P	0.123251	0.16845	0.261587
11752035_NOS2	-0.07388	0.168516	0.261679
11741383_STARD8	-0.05361	0.16852	0.261679
11761444_PSG9	-0.06126	0.168552	0.26172
11750283_OFD1P6Y /	-0.08323	0.168562	0.261727
11741391_KIFC3	-0.05038	0.168605	0.261785
11762736_BTNL9	-0.11913	0.168614	0.261792
11736872_TRPC6	-0.09226	0.168655	0.261841
11733391_AGTR2	0.109499	0.16866	0.261841
11730341_SLA2	-0.11598	0.168662	0.261841
11733787_HSDL1	0.080553	0.168677	0.261855
11733345_GTSF1L	-0.06489	0.168769	0.26199
11734037_GALC	0.142095	0.168804	0.262036
11734284_CSAG2 ///	0.051751	0.16883	0.262062
11761157_NDUFS8	-0.07234	0.168831	0.262062
11762207_PCOLCE-AS	-0.07628	0.168849	0.262075
11715997_NDUFC1	0.057356	0.16885	0.262075
11759253_HIST1H2AI	0.080577	0.168871	0.262099
11743354_PDCD4	-0.05607	0.168899	0.262134
11756486_EPHB6	-0.06904	0.169002	0.262285
11764219_LINC00380	-0.09458	0.169043	0.262341
11721945_MRVI1	-0.07749	0.169057	0.262348
11759544_PRMT2	0.071948	0.169059	0.262348
11761147_TYMP	-0.08093	0.169066	0.262351
11752460_NDST3	0.107357	0.169083	0.262369
11731180_PCDH11X /	-0.10049	0.169145	0.262457
11732855_CD300LG	-0.10536	0.169187	0.262514
11733176_ZNF507	-0.08032	0.169205	0.262534
11752283_ALOX15B	-0.11556	0.169213	0.262538
11744110_TMEM101	0.100121	0.169228	0.262545
11749464_SEMA5B	-0.08809	0.169228	0.262545
11759695_CNTN1	0.145847	0.169237	0.262551
11741421_SLC16A13	-0.05998	0.169244	0.262552
11731551_AP5S1	-0.07046	0.169267	0.262579
11759219_HIST1H2AK	0.143565	0.169278	0.262579
11749117_CD84	-0.0793	0.169281	0.262579
11719639_PLXNA1	0.078042	0.169282	0.262579
11736818_RPE65	-0.10283	0.169291	0.262583
11750235_YWHAEP7	-0.0893	0.169299	0.262588
11747363_CYB561A3	-0.07128	0.169324	0.262613
11761251_PMM2	0.09672	0.169326	0.262613
11755389_NTNG2	-0.08127	0.169416	0.262744
11759680_ATG3	-0.09316	0.169432	0.262756
11761832_PI16	-0.16534	0.169434	0.262756
11735337_CPLX4	-0.174	0.169465	0.262796
11738105_IGSF9B	-0.10526	0.169476	0.262805

11748079_NEB	0.11712	0.169482	0.262806
11733070_WDR5B	0.07958	0.1695	0.262825
11719029_PIK3AP1	0.079029	0.169526	0.262857
11744966_INSL3	-0.11855	0.169547	0.262881
11734149_PRRT3	-0.11515	0.16957	0.262909
11739692_LAGE3	-0.11019	0.169622	0.262981
11734411_SUMF2	0.061324	0.169702	0.263094
11720470_MED20	-0.06833	0.169706	0.263094
11755081_ZCCHC14	0.083753	0.169739	0.263136
11727750_NR5A2	0.193158	0.169786	0.263201
11737284_TAS2R20	-0.11216	0.16981	0.263231
11742164_MYEOV2	-0.07389	0.169864	0.263306
11716298_CLPTM1	0.058376	0.169874	0.263313
11760486_PHF11	0.130394	0.16995	0.263415
11726324_CRLF3	-0.05878	0.169951	0.263415
11727265_HAUS3	0.075584	0.169986	0.263462
11722132_MGAT3	-0.11356	0.170002	0.263477
11735834_WBP2NL	-0.0972	0.170045	0.263536
11728719_LTBP1	-0.10126	0.170078	0.263568
11734518_PAGE2 ///	0.08424	0.17008	0.263568
11751281_VWCE	-0.12993	0.170082	0.263568
11727933_VTI1A	0.112129	0.170118	0.263615
11747815_SUMF1	0.083572	0.170134	0.263633
11732759_GAPT	-0.15454	0.170182	0.263699
11749352_ABO	-0.07547	0.170196	0.263712
11747467_PRKCQ	-0.07806	0.170202	0.263712
11724147_RAB38	-0.0779	0.170291	0.263841
11716076_HSBP1	0.061701	0.170344	0.263915
11753183_LGI4	-0.05447	0.170349	0.263915
11753354_DAOA	-0.13154	0.17037	0.263939
11719171_AKR1C1	-0.0728	0.170385	0.263954
11733387_ZKSCAN3	0.122174	0.170409	0.263983
11741012_MSMO1	0.080671	0.170432	0.264011
11737372_NLRP10	0.07521	0.170467	0.264056
11743655_HPGD	-0.07771	0.170498	0.264096
11719907_BCL7A	-0.07642	0.170526	0.26413
11744282_PLBD1	-0.08635	0.170539	0.264137
11758272_SLC34A2	0.078857	0.170541	0.264137
11750405_LRIG1	-0.08453	0.170578	0.264187
11745985_SRRM1	0.051083	0.170625	0.264251
11751468_ABCB6 ///	-0.07237	0.170637	0.264261
11747848_STK11IP	0.117888	0.170657	0.264283
11722978_TAZ	-0.05764	0.170732	0.264391
11715192_FAM221A	-0.11596	0.170739	0.264393
11735499_GRM7	-0.06186	0.170765	0.264425
11763840_MEGF10	-0.05979	0.170803	0.264476
11730924_AICDA	-0.13376	0.17081	0.264478
11756580_SCP2	0.06939	0.170831	0.264495
11726339_MAGEA3 //	0.050914	0.170831	0.264495
11738295_ZNF804B	-0.142	0.170839	0.264498
11737637_CFAP77	0.118404	0.170865	0.26453

11733433_ODAM	-0.11929	0.170878	0.264542
11750490_BEND4	-0.12082	0.170939	0.264627
11737185_DUOX1	-0.12165	0.171014	0.264733
11723734_OTUD6B	0.082862	0.171021	0.264733
11761967_ECI2	-0.16296	0.171023	0.264733
11721924_PRKCB	-0.11041	0.171034	0.264742
11736715_TMPRSS11E	-0.07389	0.171085	0.264812
11755769_C1QTNF6	0.070659	0.171101	0.264828
11743194_TNFRSF14	0.068193	0.171174	0.264924
11723373_EPHA1	-0.12611	0.171174	0.264924
11761201_PMS1	0.104151	0.171263	0.26505
11730587_GATA5	-0.08611	0.171266	0.26505
11729572_CNRIP1	0.058955	0.171289	0.265077
11732597_CIB4	-0.09792	0.171296	0.26508
11738519_NBR1	-0.06978	0.171315	0.265102
11732123_PEX19	-0.06828	0.171406	0.265233
11742372_OR10P1	-0.09103	0.171448	0.265289
11719068_GPATCH3	-0.0782	0.171505	0.265369
11722613_KAT5	-0.06443	0.171538	0.265413
11718310_TMUB1	-0.05861	0.171551	0.265419
11735055_RAB3C	-0.16266	0.171554	0.265419
11736561_TSLP	0.084069	0.171659	0.265574
11736746_ADAM28	-0.11711	0.17172	0.26566
11751516_FAM71B	-0.10836	0.171727	0.26566
11732827_RNLS	-0.08825	0.171731	0.26566
11749884_TTC38	-0.0556	0.171811	0.26577
11758837_LOC100506	-0.0722	0.171815	0.26577
11739993_PDZD7	-0.10215	0.171819	0.26577
11734846_TAF1B	-0.07162	0.171826	0.26577
11726450_B3GAT1	-0.0847	0.171829	0.26577
11755044_GSAP	0.130331	0.171904	0.265877
11751768_SNX19	0.077948	0.171922	0.265896
11748952_CTNNB1	0.057887	0.171932	0.265896
11733867_HLA-DOA	-0.06146	0.171933	0.265896
11741788_FXYD3	0.081746	0.171937	0.265896
11719676_MAOA	0.094784	0.171944	0.265898
11763911_SNORA16B	0.094497	0.172013	0.265995
11733752_GPR50	-0.08831	0.172096	0.266115
11731729_P2RY13	-0.10235	0.172126	0.266154
11728458_STK32C	-0.17249	0.17214	0.266167
11721380_GNPTAB	0.060264	0.172145	0.266167
11729494_TRH	-0.22288	0.172182	0.266214
11757197_HNRNPU	-0.05802	0.172191	0.266221
11726574_BAIAP2	-0.09749	0.172221	0.266258
11751342_NDRG1	-0.1009	0.172284	0.266348
11716499_ADRM1	-0.0639	0.172333	0.266415
11747654_SGK2	-0.08199	0.172345	0.266417
11717671_SF3B6	0.055008	0.172345	0.266417
11758547_EML1	0.149635	0.172366	0.26644
11761529_MUC1	0.064504	0.172376	0.266448
11716406_LGALS9	-0.08089	0.172407	0.266485

11732118_USP10	0.05449	0.172411	0.266485
11715556_QSOX1	-0.05944	0.172438	0.266515
11742434_LRRC16B	-0.14041	0.172441	0.266515
11752175_DBF4	-0.05972	0.172473	0.266554
11731207_PTPRH	0.2208	0.172478	0.266554
11716872_ADCY3	-0.05777	0.172491	0.266565
11750431_BMPR1B	0.060416	0.172554	0.266656
11761770_KIAA0754	-0.07036	0.17256	0.266657
11729108_MBP	0.063769	0.172573	0.266659
11761715_CASC4	0.083386	0.172573	0.266659
11760554_MIER1	0.07839	0.172591	0.266678
11723924_NUP133	-0.0837	0.172629	0.266728
11737238_EOMES	-0.06565	0.172685	0.266807
11727071_ENPP6	-0.09302	0.172758	0.266911
11732687_UBXN2A	0.082454	0.172776	0.266931
11760736_RBKS	-0.0938	0.172789	0.266942
11737193_UTS2B	-0.09626	0.172813	0.266971
11758728_FGB	-0.12702	0.172821	0.266972
11753574_RAB9BP1	0.084097	0.172825	0.266972
11735572_SORBS2	0.064057	0.172872	0.267034
11745134_CCNY	-0.06338	0.172876	0.267034
11743048_ACO2	-0.06918	0.172909	0.267078
11726440_MALT1	0.065163	0.172965	0.267156
11716345_BACE2	0.067337	0.173041	0.267264
11736901_ANKRD45	0.08599	0.173047	0.267265
11729341_POU2F2	-0.06818	0.173053	0.267266
11753724_SSPN	-0.18223	0.173071	0.267284
11734183_CDH17	-0.14961	0.173078	0.267288
11748957_CORO1C	-0.04998	0.173087	0.267293
11745265_ZBTB46	-0.08078	0.173094	0.267295
11750192_CAPZA2	0.084891	0.173118	0.267324
11752125_SRRT	0.06324	0.173147	0.26736
11738236_PCSK6	-0.11004	0.173162	0.267374
11742331_EDA	-0.14514	0.17318	0.26739
11735812_FFAR4	-0.08854	0.173183	0.26739
11741415_IQCH	-0.11039	0.173223	0.267439
11744186_C16orf70	0.063958	0.173229	0.267439
11722483_FAM57B	-0.07743	0.173239	0.267439
11736360_LGI2	0.053089	0.173244	0.267439
11717581_CD52	-0.1116	0.173245	0.267439
11758356_HIST3H2A	0.070993	0.173248	0.267439
11727667_DNAH7	0.065933	0.173253	0.267439
11732618_SPATC1	-0.07677	0.173307	0.267514
11743858_IL33	0.123696	0.173321	0.267528
11721136_CMC2	-0.05288	0.173353	0.267568
11728887_GEMIN2	0.097112	0.173381	0.267603
11756316_CHI3L1	-0.08807	0.173407	0.267634
11738007_BEND2	-0.10224	0.173412	0.267634
11753711_DIO1	-0.12597	0.173428	0.26765
11720162_CXCL13	-0.1807	0.173434	0.26765
11718798_PPCDC	-0.09666	0.173447	0.267662

11731085_CAPN3	-0.10434	0.173471	0.267691
11734829_PRG4	0.109969	0.173537	0.267773
11760360_ADPGK	-0.11315	0.173539	0.267773
11746700_ITM2A	0.102652	0.17354	0.267773
11746623_NR3C2	-0.07053	0.173551	0.267776
11725381_RAPGEF3	-0.06899	0.173554	0.267776
11715317_CGB /// C	-0.07722	0.173579	0.267807
11726820_ARHGAP9	0.107339	0.173602	0.267834
11759056_FAM71F2	-0.05977	0.173616	0.267847
11753047_MTHFD2L	0.078429	0.173636	0.267866
11760136_GSTA1	-0.07408	0.173639	0.267866
11724331_NACAD	-0.07852	0.173666	0.267899
11722304_LRRFIP1	-0.05836	0.173693	0.267933
11761320_BEST1	0.125638	0.173741	0.267994
11715538_RPLP1	0.053147	0.173744	0.267994
11728056_CASP10	0.171003	0.173752	0.267998
11734436_BRSK1	0.070114	0.173758	0.267999
11733322_TRDN	0.132815	0.173768	0.268006
11733128_SLC25A26	-0.06876	0.173877	0.268159
11750326_LOC284912	-0.08389	0.173878	0.268159
11749624_TYW1B	-0.06989	0.173897	0.26818
11746062_PPY	-0.0623	0.173953	0.268257
11729161_RPF2	-0.05094	0.174019	0.26835
11746280_SYTL3	0.070454	0.174027	0.268355
11741874_SEPP1	0.154532	0.174073	0.268417
11754479_BEGAIN	-0.10951	0.174082	0.268423
11761956_ABCA11P /	-0.06408	0.174101	0.268443
11733127_SLC25A26	0.101166	0.174131	0.26848
11763735_FCRL3	0.12264	0.174169	0.268531
11747399_PLAT	0.068802	0.174184	0.268545
11721427_FZD5	-0.08657	0.174221	0.268594
11753412_C5orf60	-0.08656	0.174255	0.268638
11750926_EFCAB5	-0.06675	0.174346	0.26877
11723881_NFE2	0.12215	0.174375	0.268805
11733836_TRO	0.093345	0.174379	0.268805
11741871_FRG2 ///	-0.05852	0.174421	0.26886
11737770_APOL4	-0.16809	0.174455	0.268894
11732284_HSH2D	-0.16821	0.174458	0.268894
11717110_REPIN1	0.061523	0.174459	0.268894
11721556_ROM1	-0.0886	0.174476	0.268912
11760090_PPIAP30	-0.10816	0.174484	0.268914
11744245_MASP1	-0.12031	0.174528	0.268975
11762987_FGFR2	-0.05758	0.174539	0.268983
11732943_C7orf66	-0.11084	0.174563	0.26901
11739331_WDR82	0.079007	0.174587	0.269039
11729835_EIF5A2	0.196547	0.174616	0.269075
11732624_WFDC5	0.091178	0.174741	0.269255
11750886_CERCAM	0.055147	0.174748	0.269255
11756083_HLA-DQA1	0.094905	0.17475	0.269255
11737199_TMPRSS15	-0.08778	0.174754	0.269255
11753193_CIITA	-0.0891	0.174767	0.269261

11762574_CMPK2	-0.15703	0.174769	0.269261
11746971_NPY6R	-0.09383	0.174829	0.269345
11736961_ZNF365	0.060905	0.174849	0.269367
11717194_RAB5B	0.061318	0.174893	0.269418
11719051_CEP350	0.071851	0.174893	0.269418
11718863_SEC63	-0.07252	0.174899	0.269418
11718828_ITM2C	0.057227	0.174939	0.269464
11736690_CCDC89	0.065996	0.174939	0.269464
11764100_LOC105376	-0.09628	0.174948	0.269468
11727324_NAGS	0.05181	0.174977	0.269505
11762269_ACBD7	-0.08072	0.174986	0.26951
11729662_GLB1	-0.05296	0.175001	0.269525
11731869_AZU1	-0.0685	0.175014	0.269536
11718025_IL1R1	0.09826	0.175039	0.269567
11728943_ZNF404	0.063779	0.175091	0.269638
11744275_WDR12	0.053546	0.175126	0.269683
11733761_TMEM190	-0.07116	0.175147	0.269707
11760850_INADL	-0.15137	0.175163	0.269723
11731522_ZNF592	0.08829	0.175174	0.269732
11759220_HIST1H2AK	-0.07921	0.175195	0.269756
11725608_RTN4RL1	-0.10636	0.175201	0.269756
11729863_EPN1	-0.05305	0.175225	0.269784
11717180_OCRL	-0.0534	0.175232	0.269787
11752373_SNAP91	-0.0889	0.175247	0.269801
11729700_STXBP1	-0.09284	0.175296	0.269869
11735963_SPANXN4	0.0705	0.175359	0.269958
11751083_MLC1	-0.10213	0.175411	0.270028
11757677_UBE2F	0.066638	0.175485	0.270128
11750543_KLHL18	-0.05619	0.175487	0.270128
11757798_MAFB	-0.07362	0.175498	0.270137
11752263_FHAD1	-0.10921	0.175511	0.270148
11749513_NEK8	-0.06523	0.175538	0.270182
11738842_ADRA1A	-0.07427	0.17555	0.270192
11738167_CBX2	-0.12364	0.175559	0.270196
11724106_BATF2	-0.05098	0.175582	0.270223
11745682_SERINC5	0.075958	0.175593	0.270232
11738277_PLA2G4D	0.177002	0.175619	0.270263
11732336_SLC31A1	-0.11561	0.175649	0.270301
11745462_PRDX2	0.051479	0.175781	0.270496
11752500_MAN1A1	-0.13569	0.175853	0.27059
11747478_ZNF503	-0.069	0.175853	0.27059
11763738_AZIN2	-0.11507	0.175876	0.270608
11736941_CHRNB4	0.059675	0.175876	0.270608
11754665_LEFTY2	-0.06511	0.175922	0.270671
11741506_NREP	0.052408	0.175931	0.270675
11762877_TMEM9	-0.19335	0.175936	0.270675
11723627_ERP27	-0.10007	0.175955	0.270695
11759813_HOXB1	-0.14776	0.176009	0.27077
11751645_PBXIP1	-0.0692	0.176045	0.270816
11746482_PBRM1	-0.12685	0.176066	0.27084
11738468_MS4A10	-0.12587	0.176081	0.270855

11729178_POLH	0.0823	0.176201	0.271031
11735673_ARL13A	-0.1025	0.176225	0.27106
11744147_RPL18	0.054626	0.176231	0.27106
11732374_HSD17B7	-0.06618	0.176246	0.271075
11740136_T	0.113947	0.176296	0.271136
11763066_GAFA3	0.106447	0.176297	0.271136
11744030_MMP7	0.114688	0.176307	0.271143
11732226_C10orf54	0.051081	0.176341	0.271187
11761336_SEMA4F	-0.08763	0.176362	0.271211
11734862_TMLHE	0.076993	0.176407	0.271271
11753389_LOC439951	-0.05562	0.176433	0.271302
11728503_CD300C	-0.08785	0.176519	0.271427
11732051_C12orf77	-0.12581	0.176527	0.271429
11757740_CTDNEP1	-0.05393	0.176537	0.271436
11734311_CNN1	0.06378	0.176542	0.271436
11755342_NOP56	0.052656	0.176556	0.271449
11737692_PLEKHG4	-0.08317	0.176574	0.271469
11757159_DKC1 ///	-0.07314	0.176581	0.271471
11731273_KCNE1 ///	-0.15799	0.176599	0.27149
11731965_PODNL1	-0.06635	0.176646	0.27155
11756124_IKBKG	0.08637	0.176649	0.27155
11715172_DEPDC5	0.072195	0.17671	0.271633
11737822_APOLD1	-0.06759	0.176714	0.271633
11761040_UPP1	0.119365	0.176771	0.271711
11756750_TMEM30B	-0.13765	0.176795	0.27174
11762844_ITFG2	-0.077	0.176853	0.271821
11744354_TATDN2	0.093994	0.176866	0.271832
11746487_LINC00638	-0.09774	0.176878	0.271841
11762780_LOC102724	-0.13899	0.176935	0.271921
11719890_ 7-Mar	0.06274	0.177011	0.272026
11737104_ARL17A	0.074526	0.177015	0.272026
11762588_CYP3A4	0.110867	0.177036	0.272045
11750511_EFNA3	-0.1114	0.177038	0.272045
11719561_SOX4	-0.06775	0.177083	0.272105
11725458_KLHDC8A	-0.09477	0.177127	0.272163
11731768_ARSB	-0.05615	0.177141	0.272177
11745785_LOC148413	-0.10096	0.177152	0.272186
11725516_ABCG1	-0.15222	0.177174	0.27221
11758912_CLEC1A	0.167499	0.17719	0.272227
11734051_TRAT1	0.118696	0.177222	0.272268
11736423_POSTN	0.103824	0.177238	0.272277
11745366_POMGNT1	-0.05712	0.17724	0.272277
11720711_NEURL4	0.064122	0.177279	0.272321
11719558_SYS1	0.061317	0.177279	0.272321
11734233_TMEM253	0.055615	0.177308	0.272356
11743868_NGDN	0.069912	0.177338	0.272395
11740910_NELL2	0.053653	0.177354	0.272411
11726419_RNF112	-0.12537	0.177369	0.272425
11730402_WNK4	0.057514	0.177401	0.272465
11743297_CNOT6	-0.0676	0.177416	0.272475
11720119_GIGYF1	-0.06695	0.177418	0.272475

11755179_C3orf62	0.082759	0.177428	0.272481
11718683_AFP	0.157776	0.17745	0.272506
11737052_PLAGL1	-0.10384	0.177503	0.27258
11733913_HS3ST1	-0.07223	0.177509	0.27258
11753983_LINC00654	-0.09981	0.177551	0.272624
11719139_TNP1	-0.05312	0.177551	0.272624
11751285_MTF1	0.092599	0.177555	0.272624
11742460_CCDC154	-0.1036	0.177589	0.272668
11749224_TNFAIP1	-0.05053	0.177621	0.27271
11751554_GSTM1 ///	-0.06558	0.177643	0.272734
11751903_COL11A1	0.068993	0.177664	0.272758
11733116_CXCR5	-0.0656	0.177678	0.272768
11753168_MMP13	-0.05939	0.177681	0.272768
11760965_TTC3P1	-0.09625	0.177698	0.272784
11747366_CRTAM	0.083901	0.177739	0.272836
11723240_ARID4B //	0.053419	0.177743	0.272836
11715338_DCAF12L2	-0.07099	0.177757	0.27285
11763040_OFCC1	0.102348	0.177807	0.272917
11750570_PLCB2	-0.09653	0.177817	0.272924
11736587_SPATA6	0.052593	0.177856	0.272968
11731529_SLC17A6	-0.06479	0.177865	0.272968
11759901_CCPG1	0.097246	0.177866	0.272968
11762419_VASH2	-0.10221	0.177868	0.272968
11719311_CRY2	-0.06045	0.177892	0.272996
11732428_ADRB3	0.053349	0.177926	0.27304
11730869_PPY	-0.08395	0.177938	0.27305
11753273_ZNF584	0.082672	0.178017	0.273163
11740164_TOM1	0.054255	0.178042	0.273185
11754506_FASTK	-0.06966	0.178043	0.273185
11726940_SLC7A9	-0.13193	0.178087	0.273242
11725026_SUMF2	0.054246	0.178091	0.273242
11717087_RBM39	0.052713	0.178177	0.27336
11749524_GNAZ	-0.15287	0.178179	0.27336
11762062_HMGA2	-0.09333	0.178228	0.273426
11762997_YME1L1	0.092625	0.178248	0.273449
11747957_LOC100287	-0.05608	0.178254	0.27345
11751463_SYT4	-0.13094	0.178263	0.273454
11717509_IRF4	-0.06661	0.178289	0.273486
11746087_CD84	-0.12728	0.178315	0.273517
11729212_SUPT5H	0.052586	0.178334	0.273537
11738667_MLNR	-0.09912	0.178341	0.273539
11719781_EHD2	-0.07924	0.17839	0.2736
11715856_PRCP	0.080349	0.178396	0.2736
11715462_LYZ	0.075015	0.178397	0.2736
11763547_ANKRD36BP	-0.10613	0.178409	0.27361
11716278_CBX5	0.061968	0.17842	0.273618
11730943_OSCAR	-0.09159	0.178472	0.273688
11748937_TTLL4	-0.07519	0.178489	0.273707
11716559_TNS1	-0.11973	0.178514	0.273736
11729620_CLCN4	-0.09819	0.178521	0.273739
11739647_PRKAR2A	-0.0614	0.178542	0.273762

11742436_SLC6A15	0.083537	0.17858	0.273805
11764161_LOC105374	0.054322	0.178582	0.273805
11751990_LOC102723	-0.06142	0.178589	0.273808
11719357_FAM109B	-0.1093	0.1786	0.273816
11755880_SNX33	-0.07379	0.178697	0.273956
11749496_CUX1	0.058608	0.178705	0.27396
11747764_DDR1	-0.14424	0.178721	0.273977
11762132_AMICA1	-0.09206	0.178754	0.274019
11753057_HSPB7	0.069746	0.178822	0.274114
11752855_PIGA	0.08735	0.178845	0.27414
11750205_BCAS1	-0.06356	0.178898	0.274213
11762284_SSX8	0.13152	0.178931	0.274246
11762285_SSX8	0.13152	0.178931	0.274246
11733005_FCGR3B	0.086203	0.178936	0.274246
11740709_ACAD10	-0.06679	0.178967	0.274284
11741081_HTR1A	-0.05954	0.179028	0.27437
11742569_OR5M1 ///	-0.10767	0.179054	0.2744
11733908_CYP2D6 //	-0.06531	0.179078	0.274429
11728431_SCUBE3	-0.08966	0.179127	0.274487
11733770_DEFB104A	-0.19138	0.179128	0.274487
11738097_CATIP	-0.13308	0.17916	0.274528
11760705_NDUFS2	0.149302	0.179208	0.274594
11729536_SYCE2	0.074649	0.179219	0.274601
11726044_AGER	-0.08208	0.179254	0.274647
11727288_PROK1	-0.07131	0.179268	0.274659
11741933_RFPL1	-0.10344	0.179323	0.274736
11754166_ZNF148	-0.05745	0.179339	0.274748
11742535_FAM58BP	-0.07655	0.179343	0.274748
11738166_CBX2	0.088482	0.179402	0.27483
11738030_DLX4	-0.08553	0.179446	0.274889
11744187_SBK1	-0.0672	0.179504	0.274954
11731663_KIAA1217	0.140483	0.179505	0.274954
11736921_PCSK2	0.072025	0.17951	0.274954
11754528_TRAF7	-0.05982	0.179511	0.274954
11738001_C16orf89	-0.07626	0.17955	0.275005
11751518_CCDC148	0.106857	0.179566	0.275022
11729224_ZNF598	-0.05686	0.179614	0.275086
11738596_PF4V1	-0.06943	0.179637	0.275107
11734722_EDIL3	0.121548	0.179644	0.275107
11717191_TNNC2	0.113249	0.179645	0.275107
11763577_SLC25A23	-0.11253	0.179654	0.275107
11729917_RET	0.128145	0.179656	0.275107
11762613_TMEM234	0.087059	0.179751	0.275244
11753126_GJC1	0.056445	0.179757	0.275245
11718833_LOXL2	0.071419	0.179772	0.275259
11718701_ARFIP1	0.058675	0.179815	0.275309
11749286_USF2	-0.06984	0.179816	0.275309
11720676_CCS	-0.07113	0.179827	0.275317
11734664_DDX3Y	-0.06626	0.179872	0.275377
11735093_TOM1L2	0.067732	0.179885	0.275384
11746807_LOC100630	0.069984	0.179888	0.275384

11735959_FIGLA	-0.10255	0.179896	0.275389
11733034_C7orf73	-0.05871	0.179912	0.275404
11729004_ADRB1	-0.08102	0.179967	0.27548
11732913_SP140	-0.11739	0.180095	0.275658
11720249_FBXL15	-0.05493	0.180101	0.275658
11747456_GPT	0.129467	0.180104	0.275658
11756507_FAM188B /	0.059177	0.180106	0.275658
11738948_C11orf40	-0.22409	0.180118	0.275668
11741771_PSMD13	-0.06062	0.180126	0.275671
11762043_TOP1MT	-0.06194	0.180135	0.275676
11731283_KLK8	-0.05059	0.180208	0.27578
11751487_NAV3	0.056609	0.180251	0.275835
11735010_MEOX1	-0.10896	0.180255	0.275835
11731638_GAB1	0.101346	0.180285	0.275872
11715571_CLSTN1	0.053007	0.180292	0.275874
11750174_SVILP1	-0.19648	0.180332	0.275926
11748114_SYT12	-0.08305	0.180367	0.275971
11754708_TTC41P	0.14094	0.180402	0.276016
11730573_PLCB1	0.100935	0.180452	0.276084
11731473_TMEM70	-0.1569	0.180573	0.27626
11761239_ZDHHC20	-0.09379	0.180592	0.276281
11742131_FBLIM1	-0.0896	0.180614	0.276307
11742871_PIK3IP1	-0.05958	0.180679	0.276395
11736534_NELL1	0.058913	0.180684	0.276395
11716546_DRAP1	-0.07286	0.180699	0.27641
11732973_APLF ///	-0.10794	0.180727	0.276444
11729780_FAM127C	-0.06454	0.18075	0.276471
11742349_OR4A47	0.094173	0.180796	0.276533
11760905_FARP2	0.067307	0.180802	0.276534
11731702_CLEC2D	0.075867	0.180815	0.276544
11753639_C18orf12	-0.08647	0.180865	0.276607
11721877_MT1F	-0.08216	0.180868	0.276607
11734700_TBRG4	-0.06518	0.180873	0.276607
11730755_TXNDC12	0.050096	0.180895	0.276629
11747779_RPS6KC1	-0.07132	0.180899	0.276629
11718564_GJA4	-0.08753	0.180925	0.276661
11729975_PTPRN2	-0.08698	0.180947	0.276685
11724327_FASTKD3	0.059848	0.180957	0.276686
11758838_SPTLC2	-0.06272	0.180961	0.276686
11723853_BCL11A	-0.08842	0.180964	0.276686
11757135_SNORA49	0.079443	0.181005	0.27674
11758555_GPR183	-0.07586	0.181031	0.27677
11715288_CASP14	0.073162	0.181108	0.27688
11718625_DGUOK	0.049924	0.181142	0.276913
11742421_GRM5	0.167404	0.181146	0.276913
11740711_SLC38A4	0.164363	0.181146	0.276913
11756043_LETMD1	-0.05815	0.18121	0.277001
11757729_GDF15	-0.07254	0.18124	0.277032
11725358_ACAN	-0.05998	0.181241	0.277032
11763523_ATP6V0D1	-0.06713	0.18126	0.277051
11742293_OR6C68	-0.20694	0.181275	0.277066

11747274_SEPT9	-0.07978	0.181288	0.277076
11736993_DNAJB7	0.122525	0.181337	0.277143
11718687_WDTC1	0.053925	0.181361	0.277166
11761548_CLDN11	-0.09594	0.181367	0.277166
11754668_BAIAP2	-0.05762	0.181372	0.277166
11751121_PML	-0.05938	0.181375	0.277166
11742375_LILRA5	-0.11559	0.181395	0.277189
11759133_RAB40A	0.160125	0.181403	0.277191
11720194_CLPB	-0.13095	0.181489	0.277315
11733022_BTG1	-0.18765	0.181515	0.277346
11755767_RUSC2	0.08936	0.181533	0.277365
11715164_IGLL3P	0.150359	0.181593	0.277447
11760538_MRPL50	-0.12449	0.181638	0.277508
11741288_NXPH4	-0.0794	0.181647	0.277509
11743243_HOXD12	-0.0945	0.18165	0.277509
11715817_ZFP36L2	0.089684	0.181671	0.277532
11739650_DLL4	-0.11556	0.181687	0.277548
11758976_KIF5A	-0.07632	0.181695	0.277551
11753146_FAM83A-AS	-0.08747	0.181702	0.277554
11750958_FAM129C	-0.06798	0.181758	0.27763
11741737_SLC5A4	-0.11607	0.181783	0.277659
11728440_CHD5	-0.05759	0.181814	0.277698
11762726_CD55	0.078846	0.181864	0.277763
11746498_LZTS1	-0.13609	0.181868	0.277763
11723471_CHM	-0.05047	0.181876	0.277767
11749684_INTS9	-0.08077	0.181885	0.277767
11756216_PTP4A1	0.056038	0.181887	0.277767
11732806_ARHGAP24	0.101446	0.181926	0.277818
11749322_ALPK3	0.079635	0.181971	0.277878
11721322_UNC93B1	-0.05835	0.181979	0.277881
11752043_GRID1	-0.11281	0.182004	0.277911
11762327_METTL2B	-0.0783	0.182012	0.277915
11762925_TNIP3	-0.15551	0.18203	0.277932
11757034_SEMA5B	-0.05332	0.182045	0.277946
11744834_RPLP1	0.050507	0.182114	0.278043
11742482_ARPC4	-0.06305	0.182119	0.278043
11761656_FANCD2	-0.08928	0.182145	0.27807
11727612_SCN2B	-0.08192	0.182148	0.27807
11749171_UPB1	0.076251	0.182228	0.278182
11734726_PNPT1	-0.07419	0.182262	0.278222
11738093_CCDC172	0.089924	0.182265	0.278222
11737622_SLC6A6	0.12802	0.182304	0.278273
11763407_GTDC1	-0.06016	0.182323	0.278292
11719075_REEP1	-0.06732	0.182328	0.278292
11764291_LINC00570	-0.11084	0.182348	0.278315
11741738_C3orf33	0.129565	0.182373	0.278344
11761199_SIMC1	-0.09626	0.182424	0.278413
11762191_CA6	0.069381	0.182493	0.278509
11731690_PIK3C2G	-0.07859	0.182539	0.278567
11763910_SNORA20 /	-0.2178	0.182542	0.278567
11734920_PCYOX1L	0.062481	0.182558	0.278583

11720315_ATP6V0B	0.055386	0.182586	0.278617
11745294_OGFOD2	-0.07589	0.1826	0.278629
11739686_EXOC6B	0.073155	0.182703	0.278778
11726200_CTF1	-0.1081	0.182821	0.278949
11735496_SMCO2	0.160957	0.182868	0.279006
11751745_PRSS3	-0.11225	0.182873	0.279006
11755454_BRD7P3	0.122926	0.182883	0.279006
11734784_CLIC2	0.086879	0.182885	0.279006
11716522_C10orf10	0.06154	0.182887	0.279006
11735206_MMAA	0.069453	0.182921	0.279049
11725786_TMA16	-0.07973	0.182938	0.279066
11738286_CLEC6A	-0.13333	0.182946	0.27907
11728781_CD1C	-0.10803	0.182986	0.279122
11744004_PIK3R2	-0.06889	0.182998	0.279131
11717633_KLHL9	0.049851	0.183017	0.279152
11723637_C11orf58	-0.0735	0.183024	0.279154
11715180_CGB1 ///	0.127882	0.183153	0.279342
11748003_IFI44	-0.07911	0.183161	0.279346
11715670_IFITM1	0.050447	0.183173	0.279347
11739984_HAO1	0.130617	0.183174	0.279347
11730581_HECW2	0.097803	0.183239	0.279438
11742452_MAGED4 //	0.145147	0.183248	0.279444
11747247_RAP1B	0.076659	0.183341	0.279576
11736886_PAPOLB	-0.0663	0.183365	0.279604
11732464_WNT11	-0.12154	0.183382	0.279622
11731127_GLIPR1L1	0.140677	0.18339	0.279625
11755890_ANO7	0.072471	0.183476	0.279748
11725898_TAL1	-0.14614	0.18351	0.27979
11732219_TUBE1	0.055594	0.183523	0.279799
11761547_NOP16	0.10204	0.183527	0.279799
11728547_ZNF536	-0.11273	0.183573	0.27986
11736812_PDCD1LG2	0.121324	0.183579	0.279861
11734867_TPRG1	-0.15214	0.183617	0.27991
11723684_RPUSD2	-0.05795	0.183644	0.279943
11729082_GIPC3	-0.17583	0.183721	0.280051
11730208_TRIM46	-0.06744	0.183799	0.280161
11735095_LEP	0.076125	0.183812	0.280167
11744311_PLIN1	0.122355	0.183814	0.280167
11722136_GUCY1A3	-0.10684	0.183834	0.280185
11733607_LRRC34	-0.10056	0.183838	0.280185
11731112_GNMT	-0.09611	0.183844	0.280186
11715117_HIST1H2AJ	-0.11672	0.183857	0.280197
11756364_SLC22A2	-0.05324	0.183879	0.280222
11732903_TBX10	-0.11269	0.183897	0.28024
11740174_NRG3	0.155405	0.183912	0.280255
11759964_ZNF248	-0.15378	0.183931	0.280262
11724168_COLGALT2	0.110017	0.183932	0.280262
11743700_EHMT1	0.068867	0.183934	0.280262
11755277_PSD	-0.1256	0.183951	0.280279
11748229_IL15RA	0.081206	0.18402	0.280376
11759040_LILRB4	0.095602	0.184037	0.280393

11735308_BARHL1	0.068283	0.184095	0.280472
11715151_PRB3	0.064405	0.184148	0.280538
11719020_WDFY1	0.069236	0.184155	0.280538
11721124_MMP11	-0.06313	0.184156	0.280538
11735914_PLEKHG6	-0.07474	0.184167	0.280547
11755013_TNFRSF1B	-0.138	0.184183	0.280563
11758927_LANCL2	-0.09562	0.184216	0.280605
11745595_MRI1	0.068106	0.184266	0.280671
11715231_RPL36	-0.05532	0.184273	0.280674
11747782_CTBP2	-0.04995	0.184321	0.280737
11760639_GAPVD1	0.172791	0.184347	0.280768
11747770_ARMC5	-0.07576	0.184396	0.280835
11746253_LOC339166	-0.06219	0.184403	0.280836
11759817_MCPH1	-0.1067	0.184414	0.280845
11737102_RFX6	-0.13414	0.184434	0.280867
11747450_LOC102724	0.070195	0.184485	0.280936
11744196_SEC61A2	-0.06303	0.184492	0.280937
11762591_PPP2R3B	0.201897	0.184503	0.280946
11727947_MEGF6	0.124852	0.184516	0.280957
11757234_KCNIP4-IT	-0.09567	0.184542	0.280988
11720073_EIF3B	0.065548	0.184567	0.281016
11762179_UIMC1	0.119881	0.184661	0.281151
11738330_IL17REL	-0.10789	0.184693	0.281191
11761132_AGXT	-0.14068	0.184745	0.281262
11760772_AFMID	-0.05593	0.184758	0.281271
11752969_URAHP	-0.12198	0.184781	0.281298
11725782_ANXA2R	-0.10062	0.184839	0.281378
11761146_EIF2D	-0.08812	0.184851	0.281387
11733279_ACHE	0.127923	0.184866	0.281402
11733717_CALML6	0.089058	0.184894	0.281436
11744779_FAM13A	0.107257	0.184908	0.281444
11762899_APOBEC3G	-0.05473	0.184911	0.281444
11717299_HLA-DPB1	-0.1162	0.184944	0.281485
11733647_DCST1	0.128085	0.184976	0.281512
11747182_TMEM255B	-0.11065	0.184976	0.281512
11727300_FGFR1	0.069746	0.184979	0.281512
11719884_KAZN	0.074975	0.184988	0.281517
11719684_NTS	-0.10554	0.184995	0.281519
11718997_AHSP	-0.11939	0.185011	0.281534
11742169_FAM27L	-0.12612	0.185037	0.281565
11748095_PPFIBP2	0.085378	0.185061	0.281593
11751772_KLK2	-0.09103	0.185075	0.281606
11733199_CCDC14	-0.06053	0.185125	0.281667
11732659_LOC102723	-0.15583	0.185127	0.281667
11743125_CRYAA ///	-0.10232	0.185172	0.281727
11716316_CYTH2	-0.07509	0.185244	0.281827
11718709_TBC1D9B	-0.06845	0.18526	0.281844
11716804_PLEKHA2	-0.07388	0.185402	0.28205
11744455_UQCRH	-0.05177	0.185423	0.282074
11727950_DHX33	0.055254	0.185433	0.282074
11763880_CCNT2-AS1	-0.19108	0.185435	0.282074

11744899_FADS2	-0.15512	0.185458	0.282101
11715245_IGLL1	-0.08014	0.185513	0.282176
11740598_WDR31	-0.06022	0.185533	0.282197
11754820_RLTPR	-0.06591	0.185544	0.282205
11716910_NR2F6	0.07299	0.185571	0.282231
11724850_DIXDC1	0.091265	0.185573	0.282231
11759314_CRHR2	-0.13354	0.185602	0.282267
11722221_BFSP2	-0.11773	0.185617	0.282281
11753278_AKR1E2	-0.07146	0.185682	0.282371
11750508_PTPRN	-0.09966	0.18569	0.282375
11742106_C11orf21	-0.06518	0.18571	0.282396
11763734_UTY	-0.13995	0.185734	0.282423
11763453_METTL12	0.10764	0.185759	0.282453
11728206_FAM120A	-0.05246	0.185814	0.282528
11746955_SH3RF3-AS	0.126815	0.185821	0.282529
11745547_C5orf42	0.092548	0.185845	0.282557
11731754_CDX2	-0.15213	0.185875	0.282584
11762497_WISP3	0.123878	0.18588	0.282584
11763392_LOC100132	-0.13529	0.18588	0.282584
11750680_CTNNB1	0.061823	0.185955	0.28269
11731012_SYN2	0.199832	0.185976	0.282712
11753767_ZBTB4	-0.06427	0.185994	0.282732
11748890_CACFD1	0.05811	0.186008	0.282743
11734771_PHKG2	0.058014	0.186027	0.282764
11742067_LRRTM4	-0.10626	0.186063	0.282809
11743327_RBBP6	-0.06351	0.186083	0.282832
11724354_ATG16L2	-0.05713	0.18609	0.282833
11728530_BLNK	-0.11706	0.186104	0.282845
11728550_PIP5K1A	0.063046	0.186177	0.28294
11747204_GAS8	-0.07161	0.186178	0.28294
11737065_COL9A2	-0.0966	0.186206	0.282974
11733587_CR1	0.102558	0.186219	0.282985
11746683_NPR3	0.133427	0.186253	0.283029
11762837_ING5	0.067464	0.186309	0.283096
11738220_SLC50A1	0.098616	0.186313	0.283096
11758526_RELN	0.157082	0.186315	0.283096
11725692_VWA5A	-0.12408	0.186336	0.283119
11735734_CREB3L3	0.095128	0.186343	0.28312
11716079_CALCOCO1	-0.07792	0.186379	0.283167
11760580_TEKT4	-0.07205	0.186401	0.283192
11743708_SYCP2	-0.15957	0.186463	0.283261
11751194_TRIM54	-0.09742	0.186464	0.283261
11743357_ZBTB40	-0.06222	0.186469	0.283261
11747928_POSTN	0.117182	0.18647	0.283261
11737394_PATE2	0.18061	0.186494	0.283288
11721375_BCL2	-0.07987	0.186512	0.283307
11749410_DMKN	-0.12319	0.186523	0.283315
11735436_FHAD1	-0.08997	0.186533	0.283321
11741156_VWA7	-0.05464	0.186568	0.283365
11743690_PTCHD2	-0.08944	0.1866	0.283406
11731375_CAMKK2	-0.08163	0.18662	0.283427

11763986_APOL1	-0.06519	0.186638	0.283442
11745491_GOLGA8CP	-0.14337	0.186641	0.283442
11763548_JRKL	-0.16116	0.18667	0.283473
11740580_ASAH1	0.110266	0.186673	0.283473
11757191_AKR1C6P	-0.13801	0.186718	0.283528
11737220_GALNT8 //	0.159535	0.186721	0.283528
11732449_GTF2I ///	-0.04824	0.186733	0.283532
11743264_C14orf2	-0.04797	0.186736	0.283532
11715309_MLXIPL	-0.09022	0.186762	0.283563
11735916_TREML4	0.093783	0.186773	0.283564
11759064_AP5S1	-0.1182	0.186778	0.283564
11724687_PCSK5	0.13257	0.18678	0.283564
11749741_DMGDH	0.078656	0.186815	0.283609
11745565_MYH7B	-0.07559	0.18683	0.283615
11729889_ZFP3	-0.09423	0.186831	0.283615
11726764_AQP8	-0.1068	0.186861	0.283652
11727028_MAP1A	-0.0886	0.186886	0.283681
11756323_F12	-0.07365	0.186976	0.283804
11749381_ZNF343	0.096708	0.186978	0.283804
11762275_UQCRB	0.068644	0.186984	0.283804
11762935_HLA-DQB1	-0.11892	0.187007	0.28383
11721743_TMEM150A	0.055849	0.187036	0.283864
11752722_FAM122A	0.108517	0.187073	0.283913
11727229_OAZ3	0.080709	0.187109	0.283957
11724755_ALG2	0.1018	0.187124	0.283972
11727243_WNT4	-0.10612	0.187142	0.283991
11756034_RLN3	-0.0984	0.187163	0.284013
11731477_CHMP1B	-0.05246	0.187195	0.284053
11758373_NTF3	-0.07132	0.187269	0.284156
11734117_BTN3A3	0.092827	0.187293	0.284185
11762381_PER2	-0.16257	0.187331	0.284233
11732844_MICAL3	0.086784	0.187365	0.284276
11755788_KIF21A	-0.06942	0.187392	0.284308
11750135_MAOB	-0.08459	0.187421	0.284343
11741183_PROSER3	-0.13692	0.187472	0.284406
11759390_OR2A14	-0.0686	0.187481	0.284406
11750385_TMEM94	-0.07042	0.187485	0.284406
11762638_SSH2	0.194402	0.187486	0.284406
11759717_FNDC5	-0.0868	0.187505	0.284417
11757244_TFAMP1	-0.08217	0.18751	0.284417
11752790_TNRC6B	-0.13598	0.18751	0.284417
11715686_ARPC5	0.049914	0.187519	0.284421
11731694_HIST1H2AE	-0.12064	0.187556	0.284468
11720996_ADCY2	-0.10941	0.187587	0.284507
11744856_ABCA3	-0.07681	0.187605	0.284525
11756830_MAPKAP1	0.059155	0.187682	0.284632
11761795_ZNF138	-0.18708	0.187687	0.284632
11737811_NNT	-0.05093	0.187708	0.284654
11726779_IKBIP	0.05243	0.187733	0.284683
11734090_C5orf42	0.128607	0.187744	0.284692
11739838_KDM5A	-0.10389	0.187765	0.284714

11762018_DCLRE1C	0.087977	0.187803	0.284763
11759309_SNED1	-0.18043	0.187826	0.284784
11715316_HLA-C	0.082389	0.187831	0.284784
11729447_ARMS2	-0.06624	0.187834	0.284784
11735070_GNAL	-0.08648	0.187874	0.284836
11756129_GSK3A	-0.05018	0.187929	0.28491
11736951_RORB	-0.08201	0.187947	0.284929
11728588_MFSD13A	0.08053	0.187957	0.284936
11721220_ZNF318	0.082601	0.188015	0.285014
11746675_ZNF232	-0.10748	0.188026	0.28502
11761653_SEC16B	-0.15219	0.18803	0.28502
11727613_SCN2B	0.054484	0.18805	0.28504
11735441_ABCB5	-0.04924	0.188081	0.285079
11724494_SF3B4	-0.0517	0.188105	0.285106
11750650_PAMR1	0.060505	0.18816	0.285182
11740849_C19orf35	-0.05571	0.188176	0.285192
11743624_TIMM22	0.072115	0.188179	0.285192
11759468_LCE1A	-0.15249	0.188218	0.28524
11727673_CDH24	-0.07707	0.188223	0.28524
11716664_GDF15	-0.09196	0.188286	0.285325
11716748_AFF1	-0.06454	0.18829	0.285325
11737624_GAB2	-0.11096	0.188327	0.285366
11722554_NUP88	0.05431	0.188329	0.285366
11734792_SNAPC1	0.074428	0.188365	0.285412
11752177_SIAH1	0.061814	0.188379	0.285425
11715843_MAPK1	-0.05449	0.188392	0.285435
11758145_LIFR	-0.16763	0.188469	0.285542
11740476_NOX4	-0.0829	0.188477	0.285546
11750532_FAM215A	-0.05247	0.188485	0.285549
11740165_CSH1 ///	-0.06752	0.188507	0.285573
11736139_ITSN1	-0.07188	0.188534	0.285606
11721686_SPINK6	0.106889	0.188685	0.285826
11750801_DLAT	-0.05795	0.188691	0.285826
11745157_HELT	-0.07508	0.188734	0.285882
11758698_HBG1 ///	-0.09687	0.188746	0.285892
11735908_KRTAP4-12	0.119552	0.188773	0.285924
11726484_KRTDAP	-0.05332	0.188788	0.285937
11751981_ZMYM1	-0.04987	0.188821	0.285979
11735507_C14orf39	-0.11082	0.188858	0.286026
11761937_GPR17	0.125318	0.188869	0.286034
11728578_PRELID3A	0.047697	0.188918	0.286099
11761915_FFAR4	-0.09914	0.188949	0.286133
11723714_CPXM1	-0.085	0.188952	0.286133
11740115_CGN	0.092549	0.189003	0.286201
11749903_GORASP1	0.085526	0.189042	0.286252
11743560_PTPRC	0.129104	0.189074	0.286291
11727235_EPG5	-0.07938	0.189134	0.286372
11720572_JADE2	-0.07828	0.189166	0.286412
11720876_CORO2B	0.084894	0.189223	0.28649
11749396_DEXI ///	-0.06301	0.189231	0.286494
11754474_PLEK	-0.17135	0.18925	0.286514

11761731_SIRT6	0.067756	0.189263	0.286524
11740763_RGPD3 ///	0.093728	0.189314	0.286592
11735784_NPY2R	0.066965	0.189385	0.28669
11752918_FCRL6	-0.09845	0.189419	0.286734
11744537_PNPLA6	0.055849	0.189501	0.286839
11737868_PAPPA2	-0.09372	0.189501	0.286839
11734338_POU3F1	-0.06778	0.189648	0.287054
11763811_LHX1	-0.13264	0.189676	0.287088
11755196_CORO6	-0.08648	0.18969	0.287099
11737123_GPR82	-0.10504	0.189725	0.287143
11733926_CTNS	-0.0759	0.18974	0.287157
11737809_KCNH7	-0.08166	0.18976	0.287179
11744769_CDKN2A-AS	-0.17079	0.189804	0.287236
11739327_RAB1A	0.051334	0.1899	0.287367
11733215_STAT4	-0.06711	0.189902	0.287367
11740682_MC5R	-0.12202	0.189953	0.287435
11742901_CGGBP1	-0.05517	0.190008	0.287509
11747692_IQUB	-0.13818	0.190025	0.287526
11744676_COL11A2	-0.12526	0.190034	0.287531
11733325_FBXW8	0.093019	0.19004	0.287531
11717222_EXTL3	0.083978	0.190092	0.287601
11759292_FAM63A	-0.08343	0.190145	0.287672
11725450_TMEM45B	-0.06998	0.190197	0.287733
11726134_MYO7A	0.091308	0.190197	0.287733
11717345_FOSB	0.062593	0.190249	0.287802
11762190_CA6	0.128361	0.190285	0.287841
11717858_CD37	-0.11538	0.190286	0.287841
11749541_SUSD4	-0.0886	0.190301	0.287855
11729619_CLCN4	0.083103	0.190326	0.287883
11725494_RSPH9	0.112868	0.190356	0.28792
11724243_TCF4	-0.06824	0.190407	0.287988
11750550_NOD1	-0.06301	0.190427	0.28801
11759756_ERVK-4	0.184273	0.190457	0.288047
11754724_CCDC88B	-0.08165	0.190486	0.288081
11739643_TULP3	-0.06209	0.190525	0.288131
11759234_FABP12	0.071428	0.190532	0.288133
11744802_WFDC3	0.116229	0.190543	0.28814
11722320_EFNB3	0.066169	0.190559	0.288156
11747227_LINC00598	-0.13006	0.190572	0.288166
11746353_C1orf158	-0.05762	0.190588	0.288182
11749342_C20orf96	-0.0627	0.190619	0.28822
11716290_MRPL22	-0.17787	0.190662	0.288276
11761192_TDG	-0.08758	0.190681	0.288295
11749027_HERPUD2	0.055267	0.190705	0.288322
11724383_ST6GAL2	0.097305	0.19077	0.288412
11721982_PID1	0.08213	0.190822	0.288481
11756451_C1orf159	-0.06834	0.190848	0.288513
11735244_TRPV3	0.049279	0.190908	0.288592
11730542_ACAA1	0.067864	0.190913	0.288592
11751263_PCBP3	-0.1088	0.190919	0.288592
11763813_SAMD14	-0.09436	0.190933	0.288601

11763658_FMNL1	-0.12014	0.190936	0.288601
11750305_PPFIA2	-0.09549	0.190962	0.288631
11754865_RBM45	0.065554	0.190977	0.288645
11737234_USP6	-0.11127	0.191001	0.288673
11761327_NCAPG2	-0.11399	0.191024	0.288698
11748669_PDCD2	0.081288	0.191202	0.288952
11753003_DMBT1	-0.10725	0.191204	0.288952
11715293_TRPT1	-0.18875	0.19123	0.288982
11763179_UBE2F	-0.10152	0.191371	0.289181
11719689_TWSG1	-0.05966	0.191373	0.289181
11722603_NEFH	-0.14035	0.191398	0.28921
11730552_PRSS16	0.086037	0.191435	0.289255
11748164_VCAN	0.151365	0.191443	0.289255
11733687_STRA6	0.053348	0.191446	0.289255
11723522_PAEP	0.095994	0.191475	0.289291
11762954_KLRC2	-0.0821	0.191489	0.289302
11734141_GK5	-0.06685	0.191507	0.289321
11725113_SLC38A3	-0.07423	0.191534	0.289352
11737355_FXYD2	-0.09473	0.191549	0.289358
11725581_TNK1	-0.06547	0.19155	0.289358
11732614_CELF3	0.074811	0.191579	0.289394
11754738_PDE2A	0.10896	0.19159	0.289402
11762730_SUMO1	-0.13875	0.191664	0.289504
11758093_FBN1	0.123928	0.191709	0.289563
11750024_LMAN2L	0.066775	0.191748	0.289613
11753566_IL5RA	-0.097	0.191769	0.289636
11740389_MYOCD	-0.11609	0.191856	0.289758
11757334_UQCR11	0.046964	0.191925	0.289854
11734899_FAM71C	-0.08499	0.191932	0.289855
11734472_TEX11	0.151697	0.192022	0.289982
11720994_CCL3	0.054557	0.19203	0.289985
11752136_MCC	0.084149	0.192062	0.290024
11750318_ERC2-IT1	-0.09777	0.192117	0.290097
11726791_CNTN2	-0.14396	0.192122	0.290097
11715961_ACTR10	-0.05369	0.192158	0.290143
11754042_VPS29	0.057806	0.19218	0.290167
11738904_KRTAP4-4	0.089414	0.192198	0.290185
11739667_GALNT6	0.056523	0.192213	0.290199
11723624_PGM5	-0.08794	0.192257	0.29025
11748207_RXFP1	0.083467	0.192259	0.29025
11732412_GPER1	-0.10291	0.192283	0.290277
11741036_RPS6KA1	-0.06266	0.192412	0.290464
11738071_ZNF519	-0.12196	0.192452	0.290515
11735910_OR10H1 //	-0.12329	0.192481	0.29055
11722203_FBXO22	0.072168	0.192499	0.290558
11748598_LMOD1	0.123521	0.192499	0.290558
11715765_DUSP1	0.088161	0.192506	0.29056
11737714_CDY1 ///	0.092432	0.192548	0.290608
11727379_HDAC2	-0.16475	0.192555	0.290608
11723263_AMN1	-0.0704	0.192555	0.290608
11734076_TNRC6C	-0.06567	0.192565	0.290612

11756999_CYB561D2	-0.05273	0.19257	0.290612
11741403_ARHGEF38	-0.19867	0.192592	0.290637
11733321_CRTC1	-0.05522	0.192619	0.290668
11726817_GPRIN1	-0.07527	0.192625	0.290668
11755911_ADGRG7	-0.15358	0.192644	0.290688
11755789_HEATR1	-0.05363	0.192662	0.290706
11742753_FGF12	0.115567	0.192678	0.290719
11757336_LIMCH1	-0.0526	0.192682	0.290719
11748179_NEGR1	0.082797	0.192689	0.29072
11754662_ADAMTS1	0.082717	0.192718	0.290742
11729468_BZRAP1	-0.06816	0.19272	0.290742
11732429_ADRB3	-0.0752	0.192721	0.290742
11738358_PRR23C	-0.08894	0.192728	0.290744
11732033_NAT8B	0.05513	0.192756	0.290777
11748340_LAIR1	-0.07548	0.192769	0.290787
11733344_PTPRC	0.169127	0.192806	0.290834
11737936_RANBP3L	0.121805	0.192937	0.291017
11731931_KIAA1755	-0.07273	0.192939	0.291017
11715107_F8A1 ///	-0.07633	0.192971	0.291055
11717224_EXTL3	-0.0576	0.192984	0.291067
11726418_ABI1	0.062219	0.193009	0.291095
11720159_DENND3	0.059862	0.19305	0.291148
11720160_DENND3	-0.06992	0.193082	0.291187
11730163_SLC6A8	0.053856	0.193117	0.29123
11722353_LDB2	0.193138	0.19313	0.291241
11730333_DPYSL4	-0.06657	0.19315	0.291262
11761495_HERC3 ///	0.141573	0.193174	0.29129
11739291_EHF	0.139566	0.193187	0.291301
11721814_VWA1	-0.0913	0.193198	0.291308
11751630_HDAC11	-0.07476	0.193214	0.291324
11754103_TMED3	-0.05443	0.193264	0.291373
11722065_C12orf49	0.060898	0.193264	0.291373
11729827_FAM110B	0.140312	0.19327	0.291373
11746844_TMEM143	-0.09057	0.193271	0.291373
11717456_NLGN2	-0.10308	0.193338	0.291466
11719870_MYCN	-0.06723	0.193372	0.291507
11748334_RPAIN	-0.06256	0.193414	0.291561
11742523_OR5T1 ///	-0.13803	0.19344	0.291592
11742467_CDCA4	-0.07884	0.19346	0.291613
11747119_RGMA	0.079507	0.193518	0.291692
11756952_TKT	-0.05544	0.193532	0.291704
11751823_PTPRF	-0.13291	0.193568	0.291749
11736576_TMEM53	-0.06054	0.193585	0.291766
11734609_KRTAP5-7	-0.05907	0.193667	0.291881
11722656_STK17A	0.057956	0.193734	0.291973
11761162_LOC100507	0.06623	0.193766	0.292011
11745755_EPHB4	-0.07239	0.193836	0.292108
11718263_ERF	-0.05217	0.193855	0.292122
11762041_NCKAP5L	-0.11873	0.193858	0.292122
11760926_CENPT	0.082891	0.193872	0.292136
11717510_EXOSC10	-0.05504	0.19391	0.292175

11723165_SPI1	-0.09127	0.19391	0.292175
11745232_N6AMT1	0.073348	0.193917	0.292176
11715218_FAM127A	0.050708	0.193999	0.29229
11731545_SIK2	0.079416	0.194043	0.292347
11737232_ZNF696	-0.05963	0.194108	0.292435
11720174_SF1	-0.06208	0.194113	0.292435
11758530_KCNJ16	0.078986	0.194121	0.292438
11728574_TMEM151A	-0.0726	0.194163	0.29249
11729582_CA1	0.059693	0.194168	0.29249
11741583_LILRA4	-0.13527	0.194243	0.292594
11745494_ERCC8	0.062455	0.19427	0.292626
11734554_BCL7B	0.0564	0.194398	0.29281
11745691_SEMA4D	-0.08611	0.194443	0.292869
11748369_CARNS1	-0.06906	0.194478	0.292913
11730802_CRNN	-0.05576	0.194506	0.292946
11718734_POGK	0.049097	0.194512	0.292946
11758169_HSPA5	-0.05305	0.194524	0.292955
11736303_MIR22HG	0.065794	0.194621	0.293092
11732861_LYNX1	-0.07589	0.194632	0.2931
11734334_LINC01559	-0.06628	0.19465	0.293117
11735531_NPW	-0.08127	0.194664	0.29313
11752740_NIPAL4	-0.09989	0.194689	0.293157
11753425_SLC25A45	-0.07806	0.194698	0.293163
11758002_SPHKAP	-0.13221	0.194725	0.293194
11761430_PGAM5 ///	-0.07975	0.194756	0.293232
11733348_CNOT7	0.056922	0.194797	0.293284
11762831_TCHP	-0.10038	0.194823	0.293315
11757847_UQCRFS1	-0.05436	0.194845	0.293339
11723954_CLINT1	-0.05419	0.194874	0.293374
11758466_DCLK1	0.083914	0.194896	0.293397
11746528_ATP2B4	0.105759	0.194909	0.293408
11730704_OR51E2	-0.14493	0.194917	0.293411
11752808_EYA2	-0.06211	0.195009	0.293541
11715797_WDR45B	0.052757	0.195016	0.293542
11763003_NPHP3	-0.1443	0.195024	0.293545
11717872_IRF8	-0.14466	0.195047	0.293571
11763595_PTPRK	-0.14409	0.195082	0.293608
11761675_UTY	-0.08252	0.195084	0.293608
11760378_PER1	-0.12825	0.195093	0.293613
11742402_NKX2-6	-0.0665	0.195117	0.29364
11728849_RAB39B	0.100482	0.195149	0.293678
11753341_ICAM2	-0.07369	0.195235	0.293785
11761211_CYP4F12	-0.0661	0.195237	0.293785
11721974_SUOX	-0.05917	0.195238	0.293785
11730425_ZNF628	0.063907	0.195269	0.293824
11758429_MIR17HG /	0.080472	0.195312	0.29388
11741323_XRCC4	-0.07436	0.195321	0.293883
11757200_LOC285696	0.093411	0.195344	0.293901
11722983_NRK	-0.11545	0.195345	0.293901
11731476_MYL1	-0.10258	0.195417	0.294001
11753300_FCF1	-0.07613	0.19543	0.294011

11752657_DNMT3B	-0.08931	0.195443	0.294015
11761746_KRT17P2	0.085007	0.195449	0.294015
11740477_SP8	-0.09293	0.19545	0.294015
11720309_SERP1	0.050585	0.195477	0.294045
11745219_R3HDM1	-0.0675	0.195484	0.294048
11764069_STK3	-0.14742	0.195533	0.294112
11737274_CFAP44	0.09495	0.195542	0.294116
11738585_OR4D1	-0.13075	0.195562	0.294137
11720176_NOL6	-0.05833	0.195571	0.294142
11748692_IL9R	-0.07159	0.195585	0.294155
11721316_RCVRN	0.093738	0.195652	0.294246
11760555_HADHA	0.147675	0.19567	0.294263
11730859_SLC6A7	-0.08859	0.195684	0.294275
11724104_C8orf44-S	-0.09312	0.195741	0.294345
11750728_UGT2A1 //	0.150332	0.195742	0.294345
11749527_POSTN	-0.10018	0.195778	0.29439
11742175_TMEM88B	-0.06636	0.195791	0.294401
11762388_LOC145783	0.176769	0.195883	0.29453
11731926_TRPM3	-0.07576	0.195899	0.294542
11730279_SPATA16	-0.1417	0.195903	0.294542
11740464_PLCH1	-0.07449	0.19593	0.294573
11750953_CHRND	-0.06967	0.195948	0.294591
11739313_PWP1	-0.05233	0.195963	0.294605
11738378_IFNA2	0.113732	0.195974	0.294613
11741906_DRGX	-0.14127	0.195984	0.294618
11733579_C19orf47	0.066614	0.196054	0.294714
11719798_MBD4	-0.0654	0.1961	0.294775
11735920_MRGPRE	-0.06335	0.19611	0.29478
11731314_PAXBP1	0.055017	0.196132	0.294804
11742094_SCLY ///	0.079755	0.196143	0.294812
11733540_CNPY1	-0.05602	0.196161	0.294829
11740633_ABCA10	0.0876	0.196219	0.294908
11748961_MYOC	-0.17692	0.196235	0.294923
11726663_ZMIZ2	-0.06709	0.196339	0.295069
11723082_HEMK1	0.050997	0.196355	0.295085
11725218_TPSAB1 //	0.099858	0.196374	0.295102
11715275_SSX1	0.0996	0.196378	0.295102
11760340_CHRND	-0.14483	0.196391	0.295112
11729464_H1FNT	0.056009	0.196411	0.295133
11758740_EFTUD2	-0.16951	0.196454	0.295188
11719953_FKBP15	0.065327	0.196465	0.295196
11731982_LYPD4	-0.09575	0.196481	0.295211
11724348_ASTN2	-0.06744	0.19653	0.295276
11745317_ALDH1A3 /	-0.1253	0.196558	0.295308
11747501_SMAD1	0.100608	0.196577	0.295327
11724474_TXNDC15	0.091968	0.196647	0.295411
11740455_GNRHR	-0.07479	0.19665	0.295411
11757672_MRPS17	0.055105	0.196653	0.295411
11747198_LOC100288	0.060436	0.196657	0.295411
11742602_OR4P4	-0.14116	0.196712	0.295485
11759987_LARP1B	0.119841	0.196737	0.295514

11715457_SSR2	0.052282	0.196797	0.295595
11727905_IL13RA1	0.062002	0.196818	0.295616
11718173_PNKD	-0.11134	0.196867	0.295682
11732837_IL37	-0.0765	0.196874	0.295683
11745434_NRG3	0.149165	0.196941	0.295768
11720338_FIBIN	-0.09234	0.196943	0.295768
11756715_PACRGL	-0.09575	0.19697	0.295799
11750559_NIN	-0.0719	0.196982	0.295809
11730506_RD3	-0.11808	0.197	0.295825
11736608_TGM3	-0.16299	0.197007	0.295825
11729692_SERPINB3	-0.05247	0.197011	0.295825
11750144_CCT3	0.052722	0.197055	0.295882
11754768_SPATA13	-0.05494	0.197102	0.295941
11763504_NF1	-0.06754	0.197106	0.295941
11763656_VPS36	-0.12402	0.197129	0.295965
11747804_CLK2	-0.05333	0.197144	0.29598
11735503_BPIFA3	-0.10597	0.197211	0.296071
11717357_RTF1	-0.05562	0.197244	0.296112
11727732_LCN10	-0.09594	0.197288	0.296161
11731756_KLHDC7B	-0.11746	0.197289	0.296161
11733063_GABRA6	0.140274	0.197314	0.296189
11738005_NXNL1	-0.08158	0.197332	0.296207
11741085_APLP2	0.065914	0.197399	0.296285
11737438_BMF	-0.09708	0.197402	0.296285
11761161_EIF5A	0.202226	0.197402	0.296285
11729791_CARTPT	-0.07712	0.197427	0.296314
11730318_RGS7BP	-0.07132	0.197436	0.296317
11717683_MICU2	-0.05508	0.197469	0.296358
11736138_CTNNA2	-0.15178	0.197559	0.296484
11740516_FAM76A	-0.06747	0.197644	0.296602
11734583_ZDHHC14	0.049202	0.197681	0.296649
11759272_HIST1H2BI	-0.17984	0.19773	0.296714
11732333_NRIP2	0.058118	0.197748	0.296732
11721134_GFPT2	-0.06145	0.197775	0.296756
11731189_KIAA2022	-0.10285	0.197777	0.296756
11747817_ARFGAP1	0.066971	0.197813	0.296802
11759689_SIN3B	-0.15367	0.197822	0.296806
11753314_CEACAM21	-0.09403	0.197879	0.296882
11763391_COPE	-0.10651	0.197957	0.296989
11751424_TRAF3IP3	-0.06676	0.197963	0.296989
11763202_FAM65B	-0.08985	0.197968	0.296989
11753186_RBSN	-0.07234	0.197989	0.297011
11740650_SSH1	0.068344	0.197996	0.297013
11760185_ARRB1	0.148001	0.198005	0.297017
11724003_DPP6	0.109533	0.198042	0.297064
11743257_VAPA	-0.12655	0.198049	0.297065
11726169_FLT4	-0.09092	0.198146	0.297201
11762156_PRAMEF1 /	-0.06572	0.198182	0.297246
11763290_MRPS5	-0.14363	0.198196	0.297257
11720461_F11R	-0.05222	0.198261	0.297346
11715497_OXA1L	0.062441	0.198376	0.297509

11739110_MOCS1	-0.08349	0.198458	0.297621
11760432_GRTP1	-0.08925	0.198463	0.297621
11737421_SOWAHB	-0.12605	0.19848	0.297638
11738674_UTF1	-0.07808	0.198524	0.297695
11731601_SHCBP1L	0.105055	0.198533	0.297699
11743957_SERPINB6	-0.05663	0.198588	0.297773
11740488_DSC1	0.057793	0.198613	0.2978
11732890_DEFB132	-0.08753	0.19864	0.297832
11752072_ATP2C1	0.068203	0.198648	0.297835
11747552_TRIP10	-0.06269	0.198655	0.297837
11756341_RBM23	0.049332	0.198671	0.297851
11731846_SYTL3	-0.15418	0.198685	0.297854
11741173_PADI6	-0.14884	0.198685	0.297854
11725620_PDHB	0.047445	0.198719	0.297895
11761619_ERG	-0.07561	0.198764	0.297954
11726772_PRKCI	0.058425	0.198914	0.29817
11737968_CYP24A1	-0.09937	0.198937	0.298194
11753792_NEK3	0.07178	0.19897	0.298235
11744984_LENEP	-0.07436	0.198989	0.298253
11761587_PIGQ	-0.11804	0.198997	0.298253
11756249_CDC42	-0.05269	0.199	0.298253
11741314_OGDHL	-0.12037	0.199015	0.298266
11739551_ASB6	0.05972	0.199071	0.29834
11733300_ELOVL3	-0.0772	0.199092	0.298363
11738109_PIKFYVE	0.195805	0.199163	0.29846
11735279_LRRTM4	-0.10869	0.19919	0.298493
11741243_NGEF	-0.12	0.19924	0.298558
11750803_ALOX15B	-0.05765	0.199277	0.298602
11745319_ANKRD36 /	-0.07533	0.199282	0.298602
11738855_ACVR1B	0.065756	0.199368	0.298723
11746420_HORMAD1	-0.10945	0.199377	0.298726
11754118_CACNB2	-0.0572	0.199386	0.298731
11716732_CRYBA4	-0.07228	0.199401	0.298738
11751679_RASL12	-0.09141	0.199403	0.298738
11722426_FGL2	0.1389	0.199417	0.29875
11761575_NTHL1	-0.0933	0.1995	0.298864
11728023_TFR2	0.057339	0.199513	0.298875
11748377_IL12RB2	-0.11269	0.199542	0.29891
11729373_KCNIP2	-0.11949	0.199581	0.298959
11736042_DCAF4	0.058933	0.199617	0.299004
11755540_PUS7	0.058705	0.199671	0.299071
11755946_MED24	-0.05978	0.199674	0.299071
11761279_SIRT6	0.072558	0.19971	0.299116
11725744_SORBS1	-0.09558	0.199794	0.299232
11758630_FGL2	-0.1022	0.199834	0.299283
11752727_INPP5E	-0.07444	0.199842	0.299286
11736967_RAD51B	0.069769	0.199914	0.299384
11733346_UAP1L1	0.084828	0.199955	0.299436
11725377_LIG3	-0.05486	0.199978	0.299457
11751639_GTF2I	0.072594	0.199981	0.299457
11724356_TMEM177	-0.05097	0.200003	0.29948

11728999_GCNT2	0.1251	0.200025	0.299504
11763748_MLPH	-0.14639	0.200034	0.299509
11716302_ACSL1	-0.08134	0.200056	0.299532
11757349_FGA	-0.06569	0.200066	0.299538
11731038_FIBCD1	-0.06341	0.200084	0.299548
11753099_SORBS3	-0.09967	0.200085	0.299548
11721548_CMYA5	0.059336	0.20013	0.299606
11754784_KIAA0895L	0.051317	0.200217	0.299728
11726739_EARS2	-0.06483	0.200243	0.299758
11730807_BTN3A2	-0.07988	0.200281	0.299805
11756226_GRIPAP1	0.061735	0.2003	0.299825
11737171_CD80	-0.07308	0.200443	0.30003
11727654_SRRM4	-0.08391	0.200555	0.300187
11732845_PTCHD1	-0.05564	0.200587	0.300227
11738748_OR52K1	0.10712	0.200608	0.300248
11756933_LRRC25	-0.0624	0.200673	0.300337
11737249_C9orf43	0.049443	0.200686	0.300346
11729403_SLC30A3	-0.06348	0.20071	0.300373
11736845_CEP44	0.095175	0.200719	0.300377
11723157_INHA	-0.10852	0.200767	0.300441
11744385_C16orf91	-0.05579	0.200784	0.300457
11751048_CASC4	0.09188	0.200796	0.300465
11749215_LIMS2	-0.06512	0.200853	0.300541
11742720_LRRC58	0.056654	0.200866	0.300552
11750725_ZNF568	0.099822	0.200911	0.30061
11751322_PTCH1	-0.06229	0.200947	0.300647
11761871_LOC100131	0.10394	0.200948	0.300647
11739543_C19orf12	0.094838	0.200983	0.30069
11730953_PEX26	-0.07553	0.20099	0.300692
11759284_HIST2H2AB	0.102489	0.201035	0.300749
11740571_ZNF665	-0.09066	0.201063	0.300782
11761107_CYB561	-0.09252	0.201075	0.300787
11761600_SPRNP1	-0.07878	0.201079	0.300787
11728109_DNASE2B	-0.07537	0.201197	0.300955
11763473_PPP1R1C	0.13473	0.201216	0.300973
11741612_CSRP1	-0.07034	0.201256	0.301025
11727112_SIT1	-0.07534	0.201279	0.30105
11738854_CSF1	-0.06637	0.201306	0.30108
11761638_WRAP53	-0.10845	0.201349	0.301133
11724931_MERTK	-0.09682	0.201353	0.301133
11734217_ZNF681	0.079846	0.201372	0.301152
11756638_FBXO38	0.072066	0.201453	0.301264
11759878_PPP1R12B	-0.13559	0.201462	0.301269
11719186_N4BP2L2	-0.05246	0.201483	0.30129
11750663_SLC8B1	-0.10355	0.201523	0.301335
11722670_BAIAP2L1	-0.07069	0.201527	0.301335
11717946_LOC101928	0.073177	0.201532	0.301335
11745961_MTG2	0.108771	0.201588	0.301411
11752118_TBXA2R	-0.06887	0.201621	0.301449
11756204_TUBBP5	0.084859	0.20163	0.301449
11718741_FAM160B2	-0.08691	0.201632	0.301449

11739251_PSMD12	-0.05167	0.201663	0.301482
11747891_PDPR	-0.08322	0.201667	0.301482
11745548_CHRD	-0.08872	0.20169	0.301507
11735391_TH	-0.06461	0.201728	0.301555
11732974_OR1D2	-0.07241	0.201743	0.301569
11725460_S100A7	-0.10879	0.201762	0.301588
11731176_BCL2L15	-0.07261	0.201785	0.301612
11751323_CXCR1	-0.08439	0.201842	0.30169
11720006_TPRN	-0.07985	0.201855	0.301699
11745647_ASB16	-0.06475	0.201906	0.301766
11753651_MUC1	-0.06217	0.20199	0.301883
11735148_SOCS2	0.048906	0.202007	0.301898
11758397_RNF170	-0.12879	0.202019	0.301905
11742867_CDH6	-0.07013	0.202024	0.301905
11743589_SNX29	-0.04753	0.202039	0.301914
11738986_GABARAPL1	0.070967	0.202042	0.301914
11743003_CYP4B1	-0.08185	0.202057	0.301927
11741669_ESR2	-0.11232	0.202071	0.301939
11728839_TMCC3	-0.0537	0.202082	0.301947
11759294_KMT2A	0.048902	0.202091	0.30195
11750989_FLII	0.049612	0.202141	0.302016
11753203_LOC102723	-0.08034	0.202189	0.302079
11761158_LOC643276	-0.11178	0.202207	0.302096
11760003_ING5	-0.11287	0.202226	0.302115
11726411_ZBTB7C	-0.05118	0.202306	0.302226
11738020_RXFP4	-0.17258	0.202415	0.30238
11742585_NCR2	-0.09343	0.202506	0.3025
11732895_SLC7A13	-0.08199	0.202508	0.3025
11759500_TNPO3	-0.06545	0.202561	0.302559
11715397_MAGED2	0.05287	0.202564	0.302559
11729022_CNTN4	-0.05906	0.202567	0.302559
11744022_ZNF688	0.068457	0.20259	0.302585
11753657_BACE1	0.074235	0.202691	0.302726
11751385_MLC1	-0.07662	0.202773	0.30284
11715149_HIST2H2AB	-0.08716	0.202812	0.302889
11734312_NRN1L	-0.10179	0.202871	0.302967
11715153_PCDHGB1	0.087074	0.202887	0.302975
11729157_MFAP4	-0.05328	0.202888	0.302975
11744417_UBXN4	-0.04786	0.202916	0.303007
11761993_THRA	-0.0863	0.202981	0.303095
11761323_COMMD9	-0.12152	0.203033	0.303163
11738490_IL25	-0.07019	0.203042	0.303167
11731797_RGS13	0.154153	0.203125	0.303282
11753182_LGI4	-0.07575	0.203141	0.303297
11762150_HLA-A	-0.07875	0.203151	0.303302
11737726_CNTN5	-0.08168	0.203177	0.303326
11739024_ADAR	-0.05869	0.203179	0.303326
11744121_LGALS4	-0.08702	0.203217	0.303373
11719565_PNPLA6	0.056946	0.203231	0.30338
11718015_ZMAT5	-0.05991	0.203234	0.30338
11755638_PAX3	0.072376	0.203242	0.303383

11739860_ITGA6	-0.06021	0.203296	0.303454
11743632_PDLIM5	0.073868	0.203322	0.303483
11737142_LIPT2	0.093164	0.203355	0.303523
11763950_FKSG29	-0.06531	0.203376	0.303545
11761328_NCAPG2	-0.05188	0.203401	0.303573
11759714_ANGEL2	-0.05036	0.203486	0.303691
11719105_CPNE3	0.063518	0.203524	0.303738
11732323_FXN	0.089847	0.203565	0.30379
11726594_CTSZ	0.052366	0.203612	0.303851
11745668_BDP1	0.058631	0.203662	0.303917
11730095_KHDC3L	-0.08665	0.203679	0.303933
11743361_ADSS	-0.05626	0.203696	0.303949
11735375_MIXL1	0.047342	0.203737	0.304001
11748776_IGF2-AS	-0.06194	0.203747	0.304007
11754413_DENND1C	-0.11722	0.203789	0.304059
11762602_HOXD8	0.074895	0.203857	0.304151
11761607_ICK	-0.08499	0.203865	0.304154
11741917_OR12D3	-0.05517	0.203889	0.304181
11725537_ZNRF3	-0.06044	0.203907	0.304196
11760352_SAFB2	-0.14137	0.203912	0.304196
11761735_RXRB	-0.19214	0.203939	0.304228
11740250_SPTLC1	-0.06645	0.203949	0.304234
11750759_CES1P1	0.083609	0.20397	0.304256
11731065_GIMAP1	-0.07698	0.204069	0.304393
11756439_ADHFE1	-0.06492	0.204109	0.304444
11729490_ATP2B3	-0.06722	0.204128	0.304463
11731412_SLC8A2	-0.07951	0.204215	0.304584
11761925_FER1L6-AS	-0.10548	0.204252	0.304629
11753431_NDRG1	-0.0969	0.204327	0.304732
11715163_DEFB128	-0.1049	0.204398	0.304826
11763755_GNLY	-0.12882	0.204403	0.304826
11751515_ZNF330	0.048106	0.204427	0.304854
11752321_MIPOL1	0.121203	0.204471	0.304909
11726785_NKAPL	-0.09281	0.20453	0.304989
11731728_P2RY13	-0.12653	0.204543	0.304998
11723409_MKKS	0.049507	0.204678	0.30519
11717325_NUDT16L1	-0.04583	0.204704	0.305219
11743872_SOX17	-0.0851	0.204715	0.305224
11747842_MXRA8	-0.11024	0.204719	0.305224
11739439_RREB1	-0.0746	0.204752	0.305264
11728127_UCMA	0.108378	0.204783	0.3053
11761427_DOCK8	-0.08844	0.204844	0.305381
11738239_PURG	0.135918	0.20486	0.305396
11736136_OAS2	-0.08063	0.204877	0.305412
11733890_DNTT	-0.06638	0.204892	0.305426
11741950_GRM6	-0.11719	0.20494	0.305488
11761946_KIR2DL1	-0.11149	0.204967	0.305519
11753303_TMEM63A	0.087696	0.205003	0.305564
11726905_ARHGAP20	0.066755	0.205013	0.305569
11732893_CDKL3	0.084503	0.205051	0.305616
11730622_DSEL	0.079111	0.205072	0.305637

11738379_FGF23	-0.10793	0.205081	0.305638
11755699_ACCSL	-0.11662	0.205085	0.305638
11739887_DSG1	-0.08183	0.20514	0.305711
11723429_TPP2	0.051929	0.20518	0.305758
11762182_PPP5C	-0.18939	0.205184	0.305758
11761991_APOB	-0.1512	0.205191	0.30576
11722866_CDCP1	0.090655	0.205231	0.30581
11720779_KLHL22	-0.06914	0.205244	0.30582
11728797_LSM14A	-0.05359	0.205306	0.305903
11733209_ZNF480	0.047336	0.205329	0.305928
11748664_ZKSCAN8	-0.10256	0.205348	0.305946
11753139_ANXA8 ///	-0.14855	0.205358	0.305946
11750301_FAM92A1	-0.04772	0.20536	0.305946
11720469_TSTD1	-0.10741	0.205376	0.30596
11761842_PYCR1	-0.09264	0.205505	0.306143
11738691_OR2F2	-0.09436	0.205518	0.306153
11738805_NR1I2	-0.11602	0.205593	0.306255
11756812_ACBD4	-0.10106	0.205659	0.306345
11727242_WNT4	-0.09143	0.205675	0.306359
11744201_SNRNP200	0.053895	0.205684	0.306363
11751883_SPTBN4	-0.0743	0.2057	0.306369
11738606_KCTD16	-0.11484	0.2057	0.306369
11758409_RPL28	0.045621	0.205769	0.306461
11725597_SULT1C2	0.056482	0.205843	0.306564
11723826_C2orf88	-0.06407	0.205918	0.306666
11733297_CYP4F3	0.16854	0.205948	0.306701
11732398_ZNF326	0.048417	0.205967	0.306713
11719251_PHLPP1	-0.06081	0.205969	0.306713
11742556_OR13C9	0.052868	0.205993	0.306739
11749374_MSN	-0.06154	0.206027	0.306781
11725433_PBX2	-0.08638	0.20604	0.30679
11725895_RIN1	-0.07263	0.20605	0.306797
11762526_ZNF658	-0.08264	0.206117	0.306887
11727442_MPO	-0.08257	0.206136	0.306905
11763777_THEMIS2	-0.08717	0.206142	0.306905
11750261_RAB3GAP1	0.055874	0.20618	0.306953
11735296_RP1	0.12963	0.206201	0.306974
11730277_SULT2A1	0.123771	0.206223	0.306998
11728072_DEFB123	-0.10106	0.206314	0.307124
11743300_SRP72	-0.05099	0.206384	0.307218
11726361_ATXN3	-0.06984	0.206407	0.307243
11736015_ATP6V1G2-	-0.04925	0.206435	0.307276
11746407_TLR10	-0.13226	0.206445	0.307278
11760027_SART3	0.124497	0.206449	0.307278
11747557_RPRD2	-0.06494	0.206575	0.307456
11725285_ANKS6	-0.05289	0.206599	0.307477
11756144_EIF4B	0.046061	0.206601	0.307477
11736536_LEAP2	0.053552	0.206648	0.307537
11716731_SPSB3	-0.0476	0.206675	0.307562
11756834_PRKAG3	-0.08421	0.206678	0.307562
11729734_VIL1	-0.15379	0.206684	0.307562

11736545_MACC1	-0.10135	0.2067	0.307576
11720020_RTEL1-TNF	-0.06611	0.206763	0.307661
11749649_HIF1A	-0.04728	0.206785	0.307684
11719588_OAS1	-0.09005	0.206793	0.307687
11728518_HPR	-0.08935	0.206892	0.307825
11760224_MBD4	-0.07706	0.206928	0.307857
11729740_FAM151A	-0.09545	0.206933	0.307857
11739441_CCDC71L	0.084057	0.206935	0.307857
11721041_TRAPPC11	0.106906	0.206939	0.307857
11753528_FGFR4	-0.05911	0.206959	0.307878
11725617_HNRNPR	-0.05126	0.20704	0.307982
11740876_ZBTB12	-0.17056	0.207042	0.307982
11731129_ATPIF1	-0.04613	0.207143	0.308124
11755961_POLL	-0.05514	0.207157	0.308134
11728397_MT1M	-0.09041	0.207234	0.308241
11758051_LMO3	-0.08436	0.207279	0.308297
11730887_ACOT11	-0.07136	0.207294	0.308311
11756414_MRPS18A	-0.05717	0.207348	0.308381
11746662_ARMC3	-0.10769	0.207354	0.308381
11726585_PIPOX	-0.07008	0.207383	0.308414
11734266_PHOX2B	-0.07237	0.207469	0.308533
11757859_DLK1	-0.18217	0.207558	0.308655
11760063_CCL4L1 //	-0.09115	0.207579	0.308678
11763433_TTC39A	0.102944	0.207607	0.308711
11760666_ZKSCAN1	-0.07239	0.207665	0.308787
11733522_RUNDC3B	0.05394	0.20768	0.3088
11715227_PCDHA7	0.077894	0.207721	0.308852
11734265_PHOX2B	0.139837	0.207763	0.308904
11726848_SEMA5B	-0.06703	0.20777	0.308906
11728827_F2RL1	0.049952	0.207842	0.308998
11750772_PDGFRA	-0.05093	0.207845	0.308998
11722890_SLC17A7	-0.10126	0.207884	0.30904
11724800_LOC105369	-0.06554	0.207891	0.30904
11753755_ATP5J2	-0.05072	0.207892	0.30904
11734420_ACSM6	-0.14325	0.207911	0.309058
11737768_PIAS1	-0.05351	0.207917	0.309058
11738588_KCNK16	-0.11554	0.207926	0.309062
11751736_GPR12	-0.0929	0.20797	0.309112
11723825_PHYKPL	0.061851	0.207972	0.309112
11726842_PDS5A	0.047816	0.208056	0.309227
11729498_NMUR2	-0.09447	0.208087	0.309264
11744952_LOC79160	-0.0631	0.208099	0.309264
11735545_FAM177B	-0.05189	0.208101	0.309264
11741117_GRM1	-0.05924	0.20811	0.309264
11717855_TSSC4	-0.05278	0.208112	0.309264
11762622_FPR1	-0.12514	0.208128	0.309278
11729081_FAM155B	0.172857	0.208148	0.309291
11727975_ALDH8A1	0.080578	0.208149	0.309291
11759507_ZNF292	0.243775	0.208156	0.309292
11744820_LOC100130	0.072662	0.20819	0.309332
11723605_PCED1B	-0.06093	0.208202	0.309334

11738241_SNTG2	0.07475	0.208204	0.309334
11763699_CLASP2	-0.05873	0.20825	0.309392
11751099_GBAP1	0.066453	0.208256	0.309392
11754601_SPON2	-0.06501	0.208294	0.309439
11726033_CSNK1G3	0.077226	0.20836	0.309522
11758341_RPL37A	0.046569	0.208362	0.309522
11736751_HTR2A	-0.06712	0.208372	0.309528
11762194_AGER	-0.06634	0.208386	0.309539
11757897_SERPINB6	-0.06678	0.208414	0.309571
11727672_MICAL2	-0.10477	0.20853	0.309732
11751827_LRRC28	0.076908	0.208535	0.309732
11718281_PSG9	0.143448	0.208558	0.309756
11764106_ERGIC2	-0.08161	0.208566	0.30976
11752093_MORC1	0.069629	0.208616	0.309822
11722658_CXCL17	-0.07912	0.208621	0.309822
11731579_LYPD3	0.068014	0.208632	0.309828
11760069_SLC1A3	0.126764	0.208758	0.310007
11750060_BMF	-0.13008	0.208785	0.310037
11760747_LETMD1	-0.10044	0.208804	0.310056
11715528_ATP6V1G2-	-0.05617	0.208834	0.310091
11730004_SEMA4D	-0.07765	0.208886	0.31016
11735361_FCRL2	-0.12782	0.208946	0.310223
11732486_TRIM50	-0.07613	0.208953	0.310223
11740596_TGFB2	0.091268	0.208955	0.310223
11736773_ZIC3	0.06956	0.208958	0.310223
11743621_MSRA	0.081282	0.208966	0.310223
11763539_KLK8	-0.07752	0.208967	0.310223
11761903_DMD	-0.07737	0.209	0.310262
11739670_GRAP ///	-0.09929	0.209059	0.310335
11741449_CD99	0.049777	0.209061	0.310335
11738782_IGFL4	-0.05045	0.20911	0.310397
11760282_TRADD	0.080385	0.209179	0.310488
11726657_ADORA1	-0.06239	0.209184	0.310488
11745650_ADAMTSL1	-0.15445	0.209198	0.310499
11758042_ACTG1	0.045215	0.209273	0.310596
11717064_CPQ	0.070313	0.209275	0.310596
11728199_LOX	-0.07375	0.209294	0.310605
11717139_HARS	0.046005	0.209295	0.310605
11715168_POU3F3	0.078487	0.209335	0.310655
11739776_FNDC9	0.071719	0.209397	0.310739
11759781_TNS1	-0.05596	0.209405	0.310741
11727356_SLC6A3	-0.10442	0.209429	0.310767
11740902_PKD1L1	-0.11036	0.209449	0.310787
11737467_TMCO5A	-0.10004	0.209495	0.310846
11723878_ATP7B	-0.1138	0.209577	0.310958
11720850_RAB23	0.072487	0.209592	0.310971
11755257_TRIM31	-0.07634	0.209686	0.311102
11752634_KRT8	0.066903	0.209777	0.311226
11764205_LOC105377	-0.14753	0.20981	0.311266
11735299_GLP2R	-0.05909	0.209827	0.311281
11726184_ARSI	-0.08232	0.209848	0.311304

11721992_SLC6A6	0.11183	0.209927	0.311411
11750210_SYNPR	-0.1022	0.209936	0.311415
11752968_DHX9	0.051605	0.20999	0.311486
11744707_ANGPTL1	-0.1376	0.210017	0.311517
11718633_KIF1B	0.062389	0.21005	0.311556
11751400_TGFB3	0.057798	0.210107	0.311629
11744141_FOLR3	-0.07649	0.210119	0.311629
11732291_FAM186B	-0.09406	0.21012	0.311629
11738958_CARD17	-0.09938	0.210138	0.311629
11760091_PPIAP30	-0.09392	0.210141	0.311629
11751039_PRMT6	-0.0483	0.210142	0.311629
11732332_NRIP2	-0.07761	0.210144	0.311629
11729324_ASB5	0.138729	0.210201	0.311705
11753339_BNIPL	0.137486	0.210225	0.31173
11763086_C5orf17 /	0.092546	0.210231	0.31173
11715456_SSR2	0.050466	0.210251	0.31175
11725052_TOP1MT	0.053089	0.210263	0.311758
11729358_RFTN2	0.055416	0.210289	0.311786
11744647_PROM2	-0.11268	0.210294	0.311786
11730721_NCAM1	-0.07492	0.210351	0.311861
11758585_CEP44	-0.05264	0.21036	0.311865
11756028_GDPD1	0.125752	0.210404	0.31192
11756708_CAMTA1	-0.11023	0.210503	0.312057
11763454_METTL12	0.100711	0.210595	0.312184
11749095_ANKRD18A	-0.10136	0.210724	0.312366
11763421_ABCC9	-0.11491	0.210736	0.312375
11720979_ALKBH3	0.058559	0.210757	0.312397
11760116_TNFRSF14	0.060529	0.210791	0.312437
11732020_SPATA31D1	-0.06095	0.210819	0.312467
11724762_DEPTOR	-0.07721	0.210825	0.312467
11728502_CD300C	-0.10777	0.21083	0.312467
11737399_C1orf185	0.089811	0.210862	0.312504
11753488_OTUB2	-0.13211	0.210911	0.312567
11754046_SNX22	-0.07505	0.21105	0.312764
11738698_LILRA5	-0.07325	0.21113	0.312873
11760483_LY6D	-0.08422	0.211145	0.312886
11738647_FAM153A /	-0.07168	0.211167	0.312909
11736271_NANP	0.05323	0.211202	0.312952
11737388_MTMR11	0.05737	0.211214	0.31296
11733855_APOL1	-0.11377	0.211222	0.312963
11717570_MYH7	-0.08585	0.211267	0.313019
11753790_ST3GAL3	-0.12437	0.211282	0.313032
11737644_RNF183	-0.1003	0.211305	0.313049
11746209_SCYL1	-0.0697	0.211306	0.313049
11760132_ACSM2A	0.060291	0.211384	0.313146
11743216_OR10A6	-0.08993	0.211386	0.313146
11754213_PPIG	-0.05083	0.211391	0.313146
11762091_IGH /// I	-0.08289	0.211406	0.313159
11737997_LRRC3	-0.12739	0.21142	0.31317
11722255_RRAD	-0.06209	0.211427	0.313171
11732172_ABT1	-0.05838	0.211443	0.313185

11757170_SNORA71D	-0.07247	0.211474	0.313213
11756993_TIAL1	0.070822	0.211475	0.313213
11738469_MS4A10	-0.12659	0.211508	0.313246
11726799_SERPINA4	-0.07731	0.211509	0.313246
11753345_CUTC	0.054108	0.211533	0.313271
11733487_AFF2	-0.11751	0.21156	0.313301
11763783_LOC728613	0.060381	0.211598	0.313349
11746264_PLPP1	0.051733	0.211613	0.313361
11734366_ZFP42	-0.0881	0.211619	0.313361
11752401_POTEG ///	0.104852	0.211683	0.313446
11736232_ZNF362	0.057791	0.211723	0.313496
11732812_C11orf45	-0.08568	0.211733	0.313501
200004_PMEIF4G2	0.047473	0.211763	0.313536
11736848_B4GALNT3	-0.06556	0.211789	0.313565
11744195_FAT3	0.055168	0.211837	0.313617
11728318_FAM122A	0.107995	0.211838	0.313617
11716130_FSTL3	0.146818	0.211921	0.313729
11758561_MRAS	0.142327	0.211926	0.313729
11724700_ARHGEF37	0.094202	0.212005	0.313837
11735427_MRGPRX1	-0.11281	0.212035	0.313871
11750398_HTR2A	-0.11878	0.212041	0.313871
11752900_AZIN2	-0.04556	0.212057	0.313886
11728070_LHFPL4	-0.07813	0.212096	0.313933
11753190_KIR2DL2 /	-0.14325	0.212139	0.313987
11742021_UGGT1	0.059993	0.212173	0.314029
11715700_WDR6	-0.07069	0.212203	0.314064
11737310_RHOXF1	-0.10736	0.21223	0.314092
11738640_EFCAB12	-0.06464	0.212236	0.314092
11733566_L3MBTL1	0.216107	0.212242	0.314092
11722232_PCP4	-0.07376	0.212263	0.314114
11736681_SLC46A1	0.094019	0.212318	0.314186
11719824_ZAK	0.048732	0.212422	0.314331
11741671_CCDC36	-0.058	0.21251	0.314451
11751240_PHYHIP	-0.12681	0.212579	0.314543
11755647_DHX37	-0.06522	0.212704	0.314719
11746835_HSPA12B	-0.13875	0.212713	0.31472
11750699_ATP5J2-PT	-0.04835	0.212717	0.31472
11752226_SNRNP200	-0.05348	0.212737	0.314739
11729786_SOX13	-0.07711	0.212784	0.314799
11736584_TTC23	-0.06394	0.212858	0.314899
11720545_ANGEL1	-0.052	0.212879	0.31492
11752605_NRBP2	-0.07898	0.212885	0.31492
11726865_FOXO4	-0.07674	0.212935	0.314969
11759921_EEF1D	-0.08291	0.212937	0.314969
11753770_XRN2	0.050735	0.212938	0.314969
11763496_POLE3	0.067059	0.21297	0.315008
11741538_KLHL30	-0.09619	0.212992	0.315028
11739852_C17orf47	-0.05961	0.212996	0.315028
11736348_HFE	-0.15239	0.213031	0.315069
11741491_TMEM8B	-0.0995	0.213075	0.315125
11722469_ACSL6	-0.12088	0.213088	0.315134

11729038_DCDC2	0.102001	0.213169	0.315234
11761673_AP1S3	0.13384	0.213175	0.315234
11732544_GPR18	0.139737	0.213175	0.315234
11732211_ZKSCAN1	0.087069	0.213182	0.315236
11737568_YIPF7	-0.05328	0.213215	0.315274
11742366_OR10R2	-0.05995	0.213228	0.315284
11736856_RASL11A	-0.06338	0.213282	0.315354
11738533_KRT9	-0.07838	0.213297	0.315367
11729053_PPFIBP1	-0.05278	0.213316	0.315386
11743995_SYT5	-0.07301	0.21333	0.315397
11742170_CDRT15 //	0.076023	0.213399	0.31549
11761748_BCAS1	-0.06161	0.213442	0.315544
11733821_CHRNA2	-0.10561	0.213449	0.315544
11717873_IRF8	-0.07428	0.213462	0.315555
11747602_MAP3K8	-0.09795	0.213553	0.315678
11750275_CYTH4	-0.08127	0.213584	0.315711
11736588_CTNNA3	-0.10456	0.213587	0.315711
11747087_CFAP61	-0.15998	0.213609	0.315733
11723639_C11orf58	0.048172	0.21363	0.315755
11726931_TET1	0.13311	0.213752	0.315925
11734750_LIN52	-0.11986	0.213796	0.315981
11732991_DCHS2	-0.15119	0.213829	0.31602
11738476_CFAP47	-0.09134	0.213848	0.316038
11750479_PPARGC1A	-0.04919	0.213868	0.316058
11736560_ASIC2	-0.10836	0.213904	0.316102
11721193_CHST3	-0.08134	0.213914	0.316106
11728717_CXCL5	0.12954	0.21392	0.316106
11723067_FAM21A //	0.086001	0.213943	0.316126
11723223_CASQ2	-0.09303	0.213946	0.316126
11736687_FGF5	0.171343	0.213981	0.316165
11721583_LRMP	-0.08059	0.21399	0.316165
11761036_ANKRD44	-0.08127	0.213992	0.316165
11730438_FBP2	-0.06913	0.214007	0.316178
11747855_UQCRC1	-0.04869	0.214098	0.316303
11726943_ZKSCAN2	0.150305	0.214126	0.316335
11744545_GTF2IRD2	-0.05442	0.214133	0.316335
11759070_ZNF551	0.058825	0.214165	0.316373
11753849_COX7A2	-0.05148	0.214205	0.316421
11725580_BEND3	-0.08296	0.21421	0.316421
11753636_ZNF365	0.141115	0.214219	0.316424
11736965_FBXO40	-0.21722	0.214282	0.316499
11759652_IGKV1-12	0.103349	0.214283	0.316499
11719352_GCC2	-0.06768	0.214294	0.316506
11726613_SLC22A6	-0.10495	0.214315	0.316521
11752663_FKTN	0.106454	0.214317	0.316521
11748400_TPM4	-0.06053	0.214353	0.316555
11750294_LIG3	-0.06775	0.214353	0.316555
11750184_LOC100287	-0.06548	0.214409	0.316628
11754013_RPL15	0.044435	0.214518	0.316779
11717599_SLC20A2	0.083904	0.214534	0.316793
11732076_M1AP	0.066353	0.214578	0.316849

11745705_GSTM2P1	-0.08282	0.214594	0.316863
11725337_RLIM	0.047598	0.214605	0.316866
11730980_ZNF595	0.091901	0.214609	0.316866
11750343_MACC1	0.076783	0.214617	0.316869
11732953_SPINT3	-0.0567	0.214703	0.316979
11738959_CARD17	0.088804	0.214705	0.316979
11717227_CBFA2T2	0.055805	0.2148	0.317105
11734054_ZMYM6	0.111941	0.214809	0.317105
11748415_DDR1	-0.08615	0.21481	0.317105
11721514_NIT2	0.050385	0.214978	0.317335
11753745_CDKN2A	-0.05939	0.214979	0.317335
11761969_KIAA2012	-0.08109	0.215033	0.317406
11727164_ACVR2A	0.062596	0.215099	0.317494
11732055_CC2D2B	-0.11083	0.215119	0.317514
11737631_SLC26A7	0.199293	0.215147	0.317536
11748784_BBS4	0.078762	0.215147	0.317536
11724690_RFX4	0.099703	0.215281	0.317723
11750990_RXFP1	-0.1161	0.215291	0.317723
11722784_GRWD1	0.057983	0.215293	0.317723
11730159_RBM20	-0.12501	0.215301	0.317725
11736415_MFI2	-0.0915	0.215317	0.317739
11738222_RAET1L	0.100332	0.215377	0.317817
11750616_HYOU1	0.051847	0.215392	0.31783
11747564_HS6ST1	-0.10195	0.215445	0.317899
11729378_PFKFB2	0.064575	0.215461	0.3179
11718849_CORO1B //	-0.06237	0.21547	0.3179
11739373_SYT7	-0.08041	0.21547	0.3179
11719244_CNOT11	-0.06461	0.215472	0.3179
11742575_OR5B3	-0.17875	0.215496	0.317926
11759054_DEFB126	-0.15115	0.215505	0.317929
11733277_SLC2A2	-0.05343	0.215524	0.317948
11734783_CLIC2	0.142133	0.215578	0.31801
11719927_PSMD13	-0.05564	0.215579	0.31801
11756908_VPS50	0.059662	0.215601	0.318033
11720397_NBN	-0.05856	0.215608	0.318033
11729371_ZBTB16	-0.10976	0.215646	0.318081
11739326_PTPN2	0.142183	0.215664	0.318098
11746859_LPA	0.06319	0.215807	0.318292
11751912_CNTNAP2	0.104467	0.215809	0.318292
11751132_TAF7L	-0.07317	0.215826	0.318307
11736770_ADAM23	-0.12365	0.215924	0.318442
11759420_DOPEY1	0.084217	0.215961	0.318486
11741927_KCNIP4	0.082782	0.215976	0.3185
11734601_TAS2R43 /	0.093744	0.216004	0.318531
11742398_HOXC12	-0.14428	0.216045	0.318582
11724278_NCDN	-0.06054	0.21608	0.318624
11725592_SPATA2	0.066989	0.216094	0.318636
11762934_CD300LG	-0.15099	0.216102	0.318637
11734619_ALOX15B	-0.10645	0.216112	0.318642
11715794_ACTG2	0.051195	0.216127	0.318656
11743636_TNFAIP6	-0.13702	0.216206	0.318762

11758078_SLCO1A2	-0.08951	0.216238	0.318799
11744300_CHST15	0.074711	0.216268	0.318834
11758281_CTBS	-0.07662	0.2163	0.318872
11725242_PNOC	-0.12313	0.216341	0.318922
11720481_ABCD3	-0.05249	0.21638	0.318957
11734092_CCER1	0.107098	0.216381	0.318957
11736986_NUBPL	0.073618	0.216384	0.318957
11763056_SCAMPER	-0.122	0.216419	0.318998
11737277_HTR7	0.110258	0.216439	0.319019
200038_PMC18orf32	-0.04522	0.216455	0.319033
11753419_FAM172A	-0.1207	0.216469	0.319044
11726972_HRCT1	-0.0578	0.216508	0.319091
11738517_ZAN	-0.07116	0.216534	0.31912
11720003_NRARP	-0.07125	0.216563	0.319153
11753010_FHL1	0.084473	0.216648	0.319266
11762175_HSP90AB2F	-0.08108	0.216653	0.319266
11751721_ATP1A2	-0.1124	0.216693	0.319316
11737956_C15orf40	0.05728	0.216736	0.31937
11731039_IL15RA	0.064189	0.216775	0.319417
11761144_DDX39B	0.053323	0.216833	0.319493
11750338_KLK2	0.057476	0.216843	0.319498
11743103_PTCD3	0.050536	0.216859	0.319501
11759426_FAM127A	0.047303	0.216864	0.319501
11732692_TLDC2	0.118076	0.216864	0.319501
11742632_OR4X1	-0.09502	0.216902	0.319547
11761513_PLEKHM2	-0.08589	0.216974	0.319644
11753846_KRTAP21-3	-0.04839	0.217002	0.319676
11731419_UBE2G2	0.057996	0.217014	0.319682
11735826_VSX1	-0.09209	0.217084	0.319776
11745445_LMO3	0.055369	0.217103	0.319794
11762032_LOC441242	0.135656	0.217148	0.319852
11737604_DNM1L	-0.06982	0.217168	0.319872
11758068_WDFY3	0.11795	0.217209	0.319922
11731877_TAS2R5	-0.05222	0.217219	0.319922
11742310_OR1I1	-0.10394	0.217222	0.319922
11718444_MAPK8IP3	-0.06092	0.21725	0.319953
11746048_ADAL	0.077914	0.217322	0.32005
11745465_METTL7A	0.116664	0.217399	0.320154
11728592_IPO11 ///	-0.12012	0.217475	0.320256
11732985_FERD3L	-0.0779	0.217522	0.320315
11726129_HBZ	-0.11353	0.217556	0.320356
11728826_TRNP1	0.049308	0.217602	0.320414
11735967_PBOV1	-0.1413	0.217614	0.320422
11761635_CTSH	-0.17798	0.217634	0.320437
11761118_SH3GLB2	-0.08309	0.217641	0.320437
11727205_CBX4	0.125646	0.217644	0.320437
11746382_FBF1	-0.05704	0.217652	0.320439
11740940_HMX3	-0.09548	0.217687	0.320474
11716333_RPL28	0.044332	0.217689	0.320474
11761339_TCAF2	-0.09859	0.217704	0.320487
11762570_OR4F13P	0.050495	0.217724	0.320507

11758764_SNRPF	-0.0757	0.217798	0.320606
11745835_SF3B1	0.046135	0.217807	0.320609
11742852_RPN1	0.051134	0.217836	0.32064
11719816_BET1	0.060089	0.217841	0.32064
11759145_IL1RL1	-0.08609	0.217875	0.320676
11755035_MPDZ	0.050206	0.217879	0.320676
11715196_TMEM211	-0.04828	0.217946	0.320767
11748106_HIF3A	-0.06487	0.217965	0.320778
11743424_NSG1	-0.08584	0.217968	0.320778
11723027_ITLN1	-0.11041	0.218039	0.320874
11760934_HSD17B7P2	-0.11314	0.218047	0.320876
11752214_NCAN	0.061538	0.218054	0.320876
11721909_CRAMP1	0.052299	0.218097	0.32093
11722580_CHMP4A	0.053817	0.218239	0.321129
11735029_CLASP1	0.055127	0.218273	0.32117
11745394_PDZD2	-0.05378	0.218298	0.321196
11754582_RMND5B	0.067881	0.218308	0.321202
11748822_SH3BP5L	-0.10835	0.218323	0.321214
11755826_SELP	0.059903	0.218355	0.321251
11733082_UBXN1	0.07476	0.218375	0.321266
11763109_SYT8	-0.07377	0.218378	0.321266
11721845_MFAP5	0.09733	0.218419	0.321317
11748633_VHL	0.049067	0.218473	0.321386
11736159_MPPE1	0.053728	0.218483	0.321392
11760428_CCNG2	-0.12595	0.218534	0.321456
11723892_MTL5	-0.09141	0.218554	0.321477
11762912_POMT1	0.177015	0.218567	0.321484
11741804_BACH1	0.104793	0.218572	0.321484
11723480_SAAL1	0.047372	0.218639	0.321572
11734423_GPR26	-0.11843	0.218665	0.321595
11740751_ZNF688	-0.05434	0.218667	0.321595
11746491_PACRGL	-0.05445	0.218796	0.321774
11741439_VPS53	-0.06904	0.218848	0.321834
11762646_LRP11	-0.13243	0.21885	0.321834
11759273_HIST1H2BI	-0.12861	0.218879	0.321868
11739850_CHRFAM7A	-0.10346	0.218935	0.32194
11722060_PPP1R1A	-0.06114	0.219035	0.322075
11727176_ENTPD5	-0.06208	0.21904	0.322075
11740089_PIK3IP1	-0.05033	0.219089	0.322138
11738347_GPX6	-0.06973	0.219129	0.322183
11759736_BIRC5	-0.11858	0.219133	0.322183
11718311_RBM33	-0.06372	0.219143	0.322186
11759301_MAPRE2	-0.08674	0.219148	0.322186
11735498_ANKAR	0.145425	0.219239	0.322309
11738520_NFATC1	0.084705	0.219251	0.322315
11760290_NAA10	-0.10542	0.219256	0.322315
11720624_MYD88	0.045097	0.219272	0.322329
11727401_HOPX	0.097522	0.219285	0.322338
11735271_GCNT1	0.095201	0.219388	0.32248
11716246_WASF2	-0.07988	0.219409	0.322502
11756243_DAPP1	0.124387	0.219436	0.322527

11750918_HEPACAM2	-0.0638	0.219441	0.322527
11757407_FBLN1	-0.0537	0.219446	0.322527
11745503_TSPO	0.044662	0.219491	0.322584
11720161_CXCL13	0.133795	0.219515	0.322609
11743044_ALDH1A1	0.103977	0.219552	0.322653
11745538_MAP7D2	-0.10917	0.21958	0.322686
11736841_KLRAP1	0.070629	0.219594	0.322696
11756052_ADCYAP1R1	-0.0793	0.219665	0.322791
11749579_SCARB2	0.057274	0.219687	0.322813
11730468_NTN1	-0.06549	0.219705	0.322824
11729788_DOHH	-0.07687	0.219708	0.322824
11733305_TFAP2B	0.139171	0.219724	0.322838
11724615_LIMK1	-0.06291	0.219734	0.322844
11737846_FAM102A	-0.04581	0.219747	0.322853
11728457_STK32C	0.054581	0.219768	0.322874
11732016_TTC23L	0.152957	0.219783	0.322886
11737975_PAK6	-0.04516	0.2198	0.322901
11741787_ALG1L2	-0.05522	0.219917	0.323064
11760298_AKIP1	-0.10986	0.219934	0.323079
11750212_NOD2	-0.08665	0.219965	0.323106
11730040_GNG13	0.105991	0.219971	0.323106
11735404_ANKS1B	0.082381	0.219972	0.323106
11721048_N6AMT2	-0.05289	0.220009	0.323145
11737006_LRRC55	-0.09438	0.220012	0.323145
11740803_LHCGR	-0.08216	0.220076	0.323219
11716239_RAB6A ///	0.05577	0.220081	0.323219
11763793_LOC728613	-0.11309	0.220082	0.323219
11762187_CEP164	-0.06567	0.220095	0.323228
11760579_LETM2	0.087942	0.220143	0.323289
11735153_COL4A3	-0.05073	0.220153	0.323294
11756201_CHI3L2	-0.07087	0.220246	0.32342
11723385_PCSK6	-0.11296	0.220294	0.323481
11747949_DOK1	-0.1022	0.22033	0.323524
11724304_TEF	-0.06463	0.220402	0.32361
11735672_FAM183BP	-0.11879	0.220405	0.32361
11757732_GNAI2	-0.04537	0.220408	0.32361
11759639_CLEC16A	0.061313	0.220462	0.323667
11762818_LOC101927	-0.11367	0.220465	0.323667
11724125_HSPB9	-0.08687	0.220467	0.323667
11742279_KLK8 ///	-0.07054	0.220495	0.323698
11717025_CXCL5 ///	0.050732	0.220516	0.323719
11748620_SUCLA2	-0.04608	0.220551	0.323761
11729556_EYA2	0.113856	0.22056	0.323765
11752819_UXT-AS1	0.195914	0.220567	0.323765
11722013_CWC15	-0.06673	0.220634	0.323839
11756392_TRAPPC2L	0.052431	0.220637	0.323839
11750487_WISP1	-0.10473	0.220637	0.323839
11732620_MIR1-1HG	0.062161	0.220667	0.323873
11730666_FOXP1	-0.07833	0.220675	0.323876
11716279_CBX5	0.064237	0.220726	0.323941
11760812_CACUL1	0.096206	0.220742	0.323955

11755001_ARHGAP32	0.052318	0.220758	0.323967
11734232_CSAD	-0.09271	0.220795	0.324012
11734869_NADK	-0.08139	0.220862	0.3241
11738531_PCDHGA6	-0.07347	0.220909	0.32416
11754580_CCDC92	-0.05419	0.22092	0.324166
11739747_KLHL7	0.080743	0.22094	0.324187
11722621_AQP4	-0.09369	0.220959	0.324204
11739388_GGA3	-0.05542	0.220974	0.324217
11724174_KIF12	-0.05517	0.221053	0.324323
11731651_ARL2-SNX1	-0.04604	0.221194	0.32452
11763113_IGHG1	0.067837	0.221215	0.324531
11731428_CYP3A4	0.056339	0.221219	0.324531
11752729_LZTS1	-0.07467	0.221221	0.324531
11749937_ZFP36L1	-0.07853	0.22124	0.324548
11717242_YTHDF2	0.054367	0.221351	0.324693
11751372_TP73	-0.08859	0.221352	0.324693
11756277_YPEL1	-0.14115	0.221365	0.324703
11756686_ACCS	0.091525	0.221382	0.324718
11755460_CAPN3	0.103901	0.221393	0.324724
11736756_GAL3ST3	-0.06192	0.221419	0.324747
11745407_LOC441239	-0.07651	0.221422	0.324747
11754051_BTF3	0.043857	0.221429	0.324748
11756913_NOD1	-0.08466	0.221444	0.324758
11753140_LINC00205	-0.10673	0.221449	0.324758
11727352_RARA	0.074734	0.221489	0.324807
11752332_MEFV	-0.10615	0.221506	0.324822
11715147_MAATS1	0.11104	0.221533	0.324852
11752268_CERKL	0.076233	0.221551	0.324864
11742821_OGN	0.064366	0.221557	0.324864
11748679_CLCN2	-0.12021	0.221562	0.324864
11716068_VAMP2	0.074594	0.221591	0.324898
11720858_INSR	-0.07509	0.221605	0.324907
11728363_IL20RA	0.054566	0.221611	0.324907
11758849_NUP214	0.062509	0.221649	0.324953
11718470_GLIS2	-0.08706	0.221684	0.324995
11722821_COCH	0.10838	0.221716	0.325032
11750428_LOC100128	-0.05839	0.221744	0.325063
11729084_ZFY	-0.09277	0.221808	0.325147
11721049_CCL26	-0.13618	0.221819	0.325154
11732035_CFAP46	-0.09792	0.221877	0.325229
11753094_FAM86B3P	0.101265	0.221896	0.325247
11734238_GIP	0.056066	0.221902	0.325247
11730070_NWD2	0.117005	0.221914	0.325254
11724943_CLSTN2	-0.07442	0.221939	0.325281
11762821_LINC00856	-0.11956	0.221993	0.325351
11741645_ATXN7L1	0.088158	0.222018	0.325378
11744669_LINC00158	-0.08114	0.222043	0.325395
11750369_KRTAP5-2	-0.07473	0.222043	0.325395
11735930_PRDM7 ///	-0.06468	0.222092	0.325456
11761268_TACC1	-0.10335	0.222117	0.325483
11738042_TNIP3	-0.11584	0.22214	0.325507

11761796_CALCRL	-0.06233	0.222154	0.325518
11732589_ZNF467	0.047218	0.222355	0.325803
11748404_CAMSAP2	-0.05223	0.222368	0.325812
11754989_DMXL2	-0.0692	0.222401	0.325843
11763771_BRMS1	-0.13899	0.222402	0.325843
11722889_SLC17A7	0.074627	0.222419	0.325858
11736059_KIF5B	0.049887	0.222495	0.325959
11757153_SNHG12 //	-0.10253	0.222532	0.326004
11746257_DOCK9	0.102132	0.222545	0.326008
11759891_NAA15	-0.0803	0.222548	0.326008
11720489_ACTC1	-0.06781	0.222622	0.326107
11756181_YWHAZ	-0.04384	0.22265	0.326138
11760331_GPALPP1	-0.07557	0.222686	0.32618
11763753_KLK2	0.056262	0.222719	0.326219
11755551_MYO18B	-0.09753	0.222727	0.326221
11726818_TMEM201	-0.08246	0.222801	0.32632
11739046_MMRN2	-0.06241	0.222871	0.326403
11763038_SUPT5H	-0.11862	0.222872	0.326403
11741384_STARD8	-0.06292	0.22293	0.326479
11732046_KRT85	-0.04766	0.222943	0.326489
11761282_TUBGCP2	-0.06919	0.222988	0.326545
11751441_SLC25A44	0.055735	0.223	0.326552
11716133_MTOR	-0.06282	0.223019	0.326561
11761898_EPPIN-WFD	-0.12141	0.22302	0.326561
11758299_B2M	0.04758	0.223134	0.326719
11729967_CLYBL	0.118793	0.22321	0.32682
11729637_FAM134C	-0.08901	0.223276	0.326907
11737192_DTHD1	-0.14697	0.223282	0.326907
11753593_LETMD1	0.047356	0.223294	0.326911
11747760_POFUT2	0.04551	0.223305	0.326911
11724886_LRRN3	-0.10287	0.223305	0.326911
11752708_CRKL	0.058715	0.223329	0.326935
11759045_EBAG9	-0.12508	0.223365	0.326979
11732383_CDHR1	-0.07367	0.223442	0.327082
11742558_OR52M1	-0.09171	0.223463	0.327103
11745622_IL13	-0.0893	0.223495	0.32714
11730244_FMO2	-0.11221	0.223529	0.327179
11730020_ACSM2A	-0.10851	0.223541	0.327188
11737268_RHCE ///	-0.10664	0.223605	0.327271
11721129_HCFC1	-0.10353	0.223644	0.327319
11739648_PRKAR2A	0.05932	0.223695	0.327377
11748505_NCALD	0.124069	0.223697	0.327377
11760408_TSC2	-0.05296	0.22374	0.32743
11746083_SEC24D	0.06268	0.223756	0.327438
11735652_TMEM105	-0.06926	0.223759	0.327438
11761774_CRYL1	-0.09605	0.223846	0.327556
11737501_TRPC5	-0.07225	0.223856	0.32756
11745629_LOC149950	-0.0704	0.223871	0.327572
11742873_CDH11	-0.09468	0.223909	0.327618
11745046_TMEM52B	-0.10718	0.223944	0.32766
11740942_SALL3	-0.06057	0.223954	0.327664

11752168_LIN54	0.072484	0.223975	0.327685
11755351_TCF7	-0.05839	0.223983	0.327688
11762051_SNX22	-0.05992	0.223995	0.327695
11736355_MDN1	0.085074	0.224015	0.327714
11764035_CCDC92	-0.06087	0.224144	0.327894
11759476_KRT33A	-0.09548	0.2242	0.327965
11745312_SH3GLB2	-0.05169	0.224212	0.327973
11740521_ALDH1L2	-0.05574	0.224235	0.327997
11748750_GPNMB	-0.06365	0.224245	0.327998
11753641_TTTY6 ///	-0.09239	0.224249	0.327998
11733053_SCAMP1	0.050112	0.224265	0.328012
11726868_ERCC8	0.05286	0.224297	0.328048
11728137_XK	-0.10265	0.22431	0.328057
11756029_ASCC1	0.057803	0.224321	0.328064
11738367_GTSF1L	-0.08934	0.224385	0.328148
11753255_KLK7	-0.08124	0.224503	0.32831
11732679_ZNF471	0.072157	0.224546	0.328363
11721089_YLPM1	-0.05317	0.224587	0.328414
11733151_COX18	0.07369	0.224601	0.328425
11757898_GAS2	-0.11473	0.224685	0.328522
11732164_LMF1	-0.08077	0.224687	0.328522
11728937_GFRA2	0.075853	0.224694	0.328522
11743174_TSHZ3	-0.10666	0.224694	0.328522
11750936_HFM1	0.09919	0.224738	0.328568
11762717_PSTPIP1	-0.08258	0.22474	0.328568
11759741_COMMD3	0.188911	0.224834	0.328694
11728441_CHD5	-0.05432	0.224839	0.328694
11729621_CLCN4	-0.09609	0.22486	0.328714
11723551_SH2D3A	-0.06152	0.224888	0.328737
11731866_FCRLB	-0.05139	0.224889	0.328737
11734978_BTNL3	-0.06981	0.224896	0.328737
11761531_FCGRT	-0.05254	0.224907	0.328744
11750901_PGR	-0.10933	0.224994	0.328862
11757630_HERPUD2	-0.04716	0.225085	0.328985
11734264_CERS3	-0.12976	0.225164	0.32909
11763608_TVP23A	-0.09472	0.225273	0.329239
11758041_PSMD12	-0.0488	0.225297	0.329262
11742542_OR1S1 ///	-0.07723	0.225306	0.329262
11749571_ZNF692	-0.04356	0.225309	0.329262
11728562_DUSP13	-0.13288	0.225337	0.329294
11732916_KCNT2	0.10389	0.225392	0.329365
11744713_PITPNM2	-0.07972	0.225478	0.32948
11740623_KCNH5	0.109183	0.225486	0.329482
11760866_CALCOCO1	-0.07135	0.225567	0.32959
11723325_KCNQ1	-0.09965	0.225583	0.329604
11717418_DNAL1	0.100515	0.225593	0.329609
11727116_PLA1A	-0.04459	0.225601	0.32961
11744276_CAPG	-0.04909	0.225842	0.329948
11727074_PLAC4	-0.10742	0.225846	0.329948
11724927_PPP1R3D	0.061372	0.225853	0.329949
11721590_SLC17A9	0.045544	0.225868	0.329961

11761198_TMEM161B	-0.06707	0.225926	0.330036
11732225_ZNF664	-0.04294	0.225958	0.330063
11761532_SLC1A7	-0.08919	0.225961	0.330063
11715171_DEPDC5	-0.10415	0.225965	0.330063
11756197_ATPIF1	-0.04697	0.225977	0.330071
11746745_CHRM4	-0.06837	0.22601	0.330109
11732654_MCM9	-0.11877	0.226105	0.330239
11763160_OR6C4 ///	-0.12192	0.226171	0.330319
11742077_CHRNG	-0.1364	0.226174	0.330319
11761679_PBX1	-0.15312	0.226215	0.330368
11754094_LOC105369	-0.17047	0.226221	0.330368
11715781_EEF2	-0.04506	0.22624	0.330386
11760307_FAM216A	-0.05049	0.226265	0.330413
11731905_CRISP1	-0.08104	0.226287	0.330435
11741622_CYHR1	-0.046	0.226309	0.330458
11731032_MSH3	-0.04801	0.226336	0.330481
11748722_ZIK1	-0.10493	0.22634	0.330481
11742820_OGN	0.055771	0.226346	0.330481
11715121_HIST1H3A	-0.09764	0.226393	0.33054
11725109_FAM98C	-0.07695	0.226428	0.330581
11755258_CCDC148	-0.06935	0.226451	0.330605
11715699_SEC16A	0.047264	0.226537	0.330721
11749445_ARHGAP15	0.092837	0.226574	0.330757
11733794_STX7	0.048888	0.226575	0.330757
11715259_IFNA5	-0.14295	0.226593	0.330773
11727335_ACMSD	-0.12979	0.226608	0.330786
11723351_ABCA3	-0.08061	0.226645	0.330829
11758447_NUP50	0.055524	0.226736	0.330953
11757553_LTBP4	-0.06767	0.226877	0.331148
11756265_MTA2	-0.04595	0.226889	0.331152
11721478_GOLPH3L	0.096422	0.226895	0.331152
11725493_BEX5	-0.07036	0.2269	0.331152
11724796_DDIT4L	-0.07205	0.226921	0.331164
11737165_RBPJL	-0.07287	0.226922	0.331164
11720418_CUL2	-0.0494	0.22695	0.331195
11724659_RBFOX3	-0.06162	0.226956	0.331195
11749686_RFX4	-0.11688	0.226964	0.331196
11721121_SPNS1	-0.07126	0.227004	0.331237
11761521_DNMT3B	-0.08933	0.227006	0.331237
11755201_TRPV6	-0.05998	0.227017	0.331243
11736949_THEM5	-0.07906	0.227043	0.331272
11741077_ABR	-0.04358	0.227071	0.331302
11733737_IL22	0.087182	0.227125	0.331372
11724352_SMC6	-0.06119	0.227152	0.331401
11740228_GSDMA	-0.0831	0.227177	0.331418
11732165_LMF1	-0.11193	0.227177	0.331418
11724502_SPON1	-0.06447	0.227255	0.331521
11724760_CALM1 ///	-0.04461	0.227295	0.331565
11762538_ATP8A1	-0.06056	0.227301	0.331565
11736676_DPP10	-0.08444	0.227305	0.331565
11757480_IFI27	-0.04881	0.227314	0.331565

11719069_HSPA13	-0.05529	0.227319	0.331565
11745644_C8orf49	-0.0951	0.227453	0.331751
11737182_SLC22A13	-0.08741	0.227514	0.331829
11729461_CTNS	-0.09157	0.227529	0.331842
11729407_MYT1L	-0.06641	0.227538	0.331845
11742303_ASPH	-0.11527	0.227551	0.331853
11740668_KLRF1	-0.10937	0.227557	0.331853
11746060_TNFAIP2	-0.04837	0.227566	0.331856
11752462_ANKLE1	-0.05146	0.227597	0.331891
11762975_TMEM64	-0.12984	0.227638	0.331939
11742919_REG1B	0.097264	0.227643	0.331939
11733669_PAX3	-0.05234	0.227651	0.331941
11746661_KMT2B	0.070405	0.227663	0.331948
11763906_CCT6A ///	-0.06375	0.227671	0.33195
11745662_GNRHR2	-0.09868	0.227684	0.331959
11752415_BRSK2	-0.08554	0.227712	0.33199
11736280_SLC22A3	-0.07385	0.227885	0.332232
11737621_HEPN1	-0.06412	0.227964	0.332337
11728953_ADGRV1	0.063479	0.228003	0.332385
11731200_TLR5	-0.06032	0.228049	0.332441
11748197_MXRA8	-0.08434	0.228065	0.332455
11753538_ZNF573	0.070263	0.228088	0.332478
11759538_RPS28	0.066202	0.228177	0.332598
11751963_MBD5	0.080661	0.228192	0.332601
11753938_FAM166B	-0.07805	0.228192	0.332601
11750673_LZTR1	-0.12531	0.228209	0.332615
11734050_TRAT1	-0.13399	0.228248	0.332662
11743228_CR1L	-0.09542	0.228257	0.332666
11763832_FAM47E	-0.06848	0.228312	0.332735
11736875_ZNF514	0.063218	0.228333	0.332756
11717743_ACOT7	-0.04581	0.228392	0.332823
11723502_PRLR	0.049547	0.228394	0.332823
11758832_COL6A2	-0.14771	0.228399	0.332823
11727973_CENPN	0.045767	0.228435	0.332865
11735332_EXD3	-0.07571	0.228446	0.332871
11759469_ASB4	-0.13917	0.228455	0.332874
11728447_RAD54B	-0.04834	0.228495	0.332923
11723894_TEP1	-0.08626	0.228608	0.333077
11733524_PPP1R3A	0.101035	0.22863	0.3331
11715954_CNDP2	-0.07484	0.228649	0.333118
11719377_CAP1	0.049167	0.22867	0.333138
11753259_CAPN11	-0.0873	0.228729	0.333215
11747304_AKAP7	-0.10018	0.228781	0.333281
11722471_PEG3	-0.16222	0.228817	0.333321
11724259_COQ10B	-0.0539	0.228823	0.333321
11728681_PAIP1	-0.07647	0.228842	0.33334
11748330_PMM2	0.050917	0.228922	0.333446
11755328_IKBKG	-0.0547	0.228995	0.333543
11724578_L1CAM	-0.05395	0.229028	0.333568
11757590_ISY1-RAB4	-0.06378	0.229036	0.333568
11716496_CHMP4B	0.056264	0.229038	0.333568

11742533_OR10J3	-0.09312	0.22904	0.333568
11727678_MTMR14	-0.05303	0.229083	0.33362
11758363_SPTAN1	-0.05994	0.229133	0.333674
11742487_MAPK14	-0.07364	0.229143	0.333674
11763659_FMNL1	-0.10571	0.229146	0.333674
11762950_MAGEC3	-0.09813	0.229147	0.333674
11718748_C1QL1	0.070915	0.229159	0.333681
11737866_ITK	-0.11746	0.229206	0.333741
11725017_MAP9	-0.10212	0.229294	0.333851
11751376_LRP5L	-0.09125	0.229296	0.333851
11731857_MT1H	0.056374	0.229323	0.333874
11740665_TBC1D8B	-0.04712	0.229325	0.333874
11755655_MICAL3	-0.09429	0.229333	0.333876
11756667_C6orf223	-0.05713	0.229346	0.333884
11732425_ANKRD22	0.076387	0.229411	0.333969
11720207_IL2RG	-0.06664	0.229446	0.33401
11749597_UBAC2	-0.06112	0.229514	0.334099
11720776_RNF8	-0.14466	0.229523	0.334102
11733739_B3GNTL1	0.053664	0.229607	0.334214
11735536_ZNF660	-0.18536	0.229615	0.334217
11733019_PIK3R1	-0.05708	0.229623	0.334218
11729995_GDF10	-0.06275	0.229636	0.334227
11760534_NRG4	0.098337	0.229655	0.334244
11745631_NHS	0.141328	0.229712	0.334318
11750753_PRKCQ	-0.07966	0.229721	0.334321
11748934_DFNB59	-0.06358	0.229805	0.334433
11716123_NAT10	0.054136	0.229854	0.334495
11717061_PPP1R1B	-0.05498	0.229892	0.334539
11747016_PGAP3	-0.08611	0.230007	0.334696
11751688_GINM1	0.047457	0.230061	0.334765
11758546_VDR	-0.08534	0.230143	0.334875
11728122_ADGRG2	-0.0542	0.230162	0.334893
11762755_TM7SF2	0.074032	0.230244	0.335003
11751726_LOC155060	-0.05473	0.230273	0.335031
11716320_RPS3A	0.046426	0.230279	0.335031
11738879_PSG1 ///	-0.05457	0.230285	0.335031
11743827_LDLRAD4	-0.07372	0.230303	0.335048
11720730_CSDC2	-0.06858	0.230391	0.335166
11740255_UBE2NL	-0.09551	0.230409	0.335183
11729206_PTPN11	0.05654	0.230452	0.335234
11726561_SAP18	-0.04886	0.23046	0.335236
11715789_SLC40A1	-0.07441	0.230467	0.335237
11721411_BSPRY	-0.08029	0.230481	0.335247
11736939_ENDOV	0.075148	0.230522	0.335297
11715945_RALB	0.062179	0.230562	0.335345
11762259_HLA-G	-0.07221	0.230617	0.335412
11761178_SLC51A	-0.06747	0.230622	0.335412
11743581_ACTR2	-0.04775	0.230667	0.335467
11738390_DEFB121	-0.08928	0.230704	0.335506
11734526_PCSK6	0.072004	0.230707	0.335506
11746515_PDE7B	0.156032	0.230734	0.335535

11722064_REEP3	-0.09772	0.230744	0.33554
11757171_SNORA71D	-0.05595	0.230769	0.335565
11747749_SMARCA2	0.061817	0.230869	0.335701
11731276_CLEC4G	-0.12868	0.230921	0.335767
11737895_PCDHB11	0.076044	0.230955	0.335806
11751809_LINC00671	-0.08063	0.231001	0.335863
11759206_PON1	-0.12301	0.231008	0.335863
11745851_HSPD1	-0.0454	0.23102	0.335871
11727367_MED29	-0.05413	0.231043	0.335894
11720325_POLR2F	-0.07547	0.231072	0.335926
11747473_ITGB8	-0.05781	0.231083	0.335932
11763946_TAF1	-0.085	0.231089	0.335932
11736340_PCSK1	0.102331	0.23111	0.335952
11756896_COL6A6	-0.08115	0.231151	0.336002
11737233_ZNF696	-0.06726	0.231205	0.336069
11739412_MTR	-0.05853	0.231211	0.336069
11718155_THTPA	-0.09964	0.231223	0.336069
11750218_TSPAN6	-0.06013	0.231225	0.336069
11732501_TMOD1	0.04996	0.231236	0.336075
11735108_DCTN3	0.050672	0.231258	0.336096
11760603_HES2	-0.06215	0.231264	0.336096
11731021_MUCL1	-0.04967	0.231338	0.336193
11732177_MYH11	-0.13753	0.231509	0.336432
11754398_SMIM10	0.088034	0.231525	0.336441
11754955_P2RY11 //	-0.08679	0.231529	0.336441
11750617_MED24	-0.0543	0.231583	0.336498
11762962_TBATA	-0.12066	0.231586	0.336498
11740726_FAM9C	0.06515	0.231589	0.336498
11738048_ELOVL7	-0.12512	0.231615	0.336526
11732321_PLAT	-0.05128	0.231628	0.336535
11733155_COL9A1	-0.06781	0.231695	0.336622
11723345_TMUB2	-0.04789	0.231717	0.336644
11736306_GABRB1	-0.10439	0.231727	0.336648
11753365_DCSTAMP	-0.05867	0.23174	0.336658
11749106_PCDH1	-0.07368	0.231765	0.336684
11741080_SARDH	-0.06074	0.231779	0.336694
11750001_CLEC7A	-0.07466	0.231824	0.33675
11726919_SRD5A2	-0.07879	0.231844	0.33676
11760492_PRMT1	-0.08529	0.231845	0.33676
11720344_C3orf62 /	-0.07397	0.231927	0.336869
11757655_TIMM10B	0.049717	0.231964	0.33691
11724317_FGF1	0.06174	0.231969	0.33691
11722269_SASH3	0.109309	0.23199	0.336931
11733981_PDK1	-0.08706	0.232	0.336934
11754603_DUSP18	-0.06857	0.23201	0.33694
11755535_SYNPO2L	0.118806	0.232065	0.337
11731111_CLEC3A	0.091266	0.232066	0.337
11759095_GNAQ	0.061755	0.232089	0.337025
11744774_FAM135B	0.128421	0.232142	0.337091
11735775_HIST1H4K	0.11586	0.232166	0.337116
11718964_NUFIP2	0.093354	0.232205	0.337163

11726330_SLC23A1	0.103228	0.232249	0.337216
11733738_KRIT1	0.064629	0.232266	0.337231
11736372_MUC17	-0.06185	0.232332	0.337317
11752352_SLC9C2	0.095333	0.232353	0.337338
11727204_CBX4	-0.05701	0.232423	0.337429
11719514_ADNP2	-0.04494	0.232582	0.337649
11755557_HACE1	0.079183	0.232592	0.337654
11721027_GAK	-0.06653	0.23261	0.337671
11758884_AK3	-0.10274	0.232687	0.337772
11759451_TRPC7	-0.13642	0.232698	0.337778
11715294_HECTD2	-0.06064	0.232715	0.337792
11763556_EIF4A1	-0.05509	0.232738	0.337802
11729011_CDH22	0.055077	0.232739	0.337802
11762145_MAPK11	-0.07615	0.232742	0.337802
11754895_SNX31	-0.11033	0.232774	0.337838
11737970_PCDHGA7	0.064211	0.232783	0.337842
11735328_LMX1B	-0.09274	0.232855	0.337936
11726942_ZKSCAN2	0.080957	0.2329	0.337985
11725589_RAB5C	-0.06288	0.232902	0.337985
11719910_PICALM	0.059782	0.232916	0.337993
11723037_STX8	-0.05082	0.232948	0.338031
11759858_CTAGE5	-0.08781	0.233027	0.338135
11720008_STEAP4	-0.11141	0.233055	0.338157
11737066_GCSAML	-0.13618	0.233056	0.338157
11756701_ITGB7	-0.09927	0.23307	0.338167
11763471_CEP192	0.130497	0.2331	0.338199
11718524_NIPA2	0.044512	0.233106	0.338199
11742471_AR	-0.12129	0.233139	0.338237
11753306_EBF2	-0.08758	0.233214	0.338336
11715156_KRTAP6-1	-0.07604	0.233249	0.338377
11729107_HPCAL4	0.109189	0.233267	0.338392
11761773_PCOLCE	-0.05431	0.233281	0.338403
11732021_SMTN	-0.05897	0.233373	0.338523
11752089_MYO5C	-0.09878	0.233378	0.338523
11763304_TJP2	0.071523	0.233389	0.338529
11748978_FAM109A	-0.07632	0.2334	0.338535
11716827_RPS19BP1	-0.06055	0.233413	0.338544
11760765_POM121L9F	-0.09205	0.233436	0.338568
11720560_ZNF76	-0.04922	0.233493	0.33864
11757743_CAMSAP3	0.059508	0.2335	0.338641
11719549_RHOBTB1	0.091448	0.233579	0.338745
11730944_CCDC65	-0.10181	0.233605	0.338773
11742576_OR5B17	0.131869	0.233662	0.338846
11730502_ACTRT3	-0.14929	0.233692	0.338869
11716029_NDUFB9	-0.04511	0.233692	0.338869
11736960_ZNF365	-0.06462	0.233797	0.339011
11739195_PCGF3	-0.06456	0.233815	0.339027
11735735_CREB3L3	0.112387	0.233861	0.339083
11742036_DNMT3B	-0.06149	0.233902	0.339131
11757326_FTL	0.042347	0.233907	0.339131
11733289_CXorf36	-0.05917	0.233948	0.339174

11734124_FSTL4	-0.07259	0.233951	0.339174
11762053_AKNAD1 //	0.12859	0.233974	0.339197
11739374_SYT7	-0.05845	0.233995	0.339218
11728176_BMP8A	-0.08061	0.234004	0.33922
11759529_ZC3H15	-0.09682	0.234015	0.339227
11745065_RNF126	0.056775	0.234027	0.339234
11760612_RPL5 ///	0.078883	0.234066	0.33928
11744555_CYP2B7P	-0.08124	0.23409	0.339304
11736748_SLC22A9	-0.14254	0.234152	0.339381
11724579_L1CAM	0.04314	0.234156	0.339381
11716441_USO1	-0.04356	0.234166	0.339385
11763163_DNAJC9	0.085169	0.234177	0.33939
11726477_HS6ST3	0.046663	0.234188	0.339394
11727464_ZXDC	0.070367	0.234193	0.339394
11749253_CACNA2D4	0.066711	0.234202	0.339397
11721889_HSBP1L1	-0.07146	0.23422	0.339412
11750438_PGAM1	-0.04519	0.234233	0.339422
11728675_MED14	-0.05438	0.234258	0.339448
11741342_COL9A1	-0.04438	0.234287	0.339479
11730592_MCMDC2	0.099804	0.234311	0.339505
11756091_C11orf94	-0.07167	0.23434	0.339536
11734251_SEPT14	0.112424	0.234352	0.339544
11733724_HMP19	-0.07025	0.234435	0.339654
11737871_TMEFF2	0.085453	0.234493	0.339727
11727757_OSM	-0.07039	0.234539	0.33978
11731739_PROX1	0.078739	0.234545	0.33978
11756385_IKBKG	-0.06516	0.23455	0.33978
11731784_SNX9	-0.09537	0.234582	0.339812
11716109_PIGR	0.133415	0.234586	0.339812
11715265_FIG4	0.047666	0.234632	0.339868
11757948_TBL1X	0.056217	0.234656	0.339894
11746338_RAB35	-0.10022	0.234668	0.3399
11738310_SHANK2	0.080856	0.234688	0.339918
11727712_PLXNA4	-0.06312	0.234694	0.339918
11738354_BTG4	0.076812	0.23471	0.339931
11739318_FAM114A1	0.08995	0.234726	0.339945
11761669_ARHGAP17	-0.10836	0.234744	0.33996
11763295_COLGALT2	-0.08618	0.234768	0.339985
11735900_HS3ST4	-0.09045	0.234865	0.340115
11733759_LOC105369	-0.05747	0.234879	0.340126
11735506_C14orf39	-0.09413	0.23491	0.340161
11746558_LGALS9	-0.07125	0.235011	0.340292
11721490_TRIP10	-0.04485	0.235015	0.340292
11718495_SRP68	0.047494	0.235027	0.3403
11735528_IFT74	0.08187	0.235051	0.340324
11763784_MEI1	-0.056	0.235064	0.340333
11752496_GAS7	0.060587	0.235073	0.340334
11763894_SNORA58	0.067646	0.235078	0.340334
11748311_VCAN	0.056854	0.235114	0.340376
11746305_NOP14	0.044019	0.23516	0.340432
11747000_LTA4H	0.047665	0.235176	0.340441

11729547_AMH	-0.05342	0.235181	0.340441
11724661_PAX8	0.053294	0.235194	0.340443
11732649_C21orf62	-0.06164	0.235196	0.340443
11756610_CD226	-0.1269	0.235212	0.340456
11738399_RGMA	-0.07824	0.235266	0.340525
11718663_SRP14	0.057795	0.235305	0.340572
11758714_ITIH2	-0.08329	0.235343	0.340616
11750177_CEACAM8	0.058355	0.235381	0.340661
11726892_SLC51A	0.062351	0.235426	0.340716
11750649_SMCR5	-0.05377	0.235465	0.340762
11755463_TTC30A	0.084755	0.235475	0.340766
11749975_PGAP3	-0.07248	0.235492	0.340781
11721729_ADGRB2	-0.05771	0.23552	0.340811
11722794_GDE1	-0.04839	0.235552	0.340848
11740537_KRTAP19-3	-0.05756	0.235632	0.34095
11753691_RPL24	-0.04264	0.235642	0.34095
11721269_SCG3	0.075221	0.235644	0.34095
11747973_TBR1	0.157253	0.235662	0.340958
11728179_CYP46A1	-0.14139	0.235663	0.340958
11726810_SMIM24	-0.12884	0.235709	0.341015
11746295_UQCC1	0.060696	0.235773	0.341096
11735164_OSBPL5	-0.06782	0.235813	0.341145
11737969_PCDHGA7	0.059975	0.235822	0.341148
11735845_CCDC144A	0.080016	0.235847	0.341174
11757225_LOC389705	-0.12283	0.235858	0.341174
11733636_FAM92B	0.079492	0.235865	0.341174
11751482_BTN3A2	-0.05925	0.235868	0.341174
11724422_GFRA3	-0.1331	0.235938	0.341265
11739705_CRB1	-0.05368	0.235973	0.341306
11721910_CRAMP1	0.059287	0.235988	0.341317
11752024_PCDH15	-0.09903	0.236027	0.341364
11729855_CD3E	-0.10424	0.236051	0.341388
11752844_PTGER3	-0.06651	0.236077	0.341415
11728135_NIPAL4	-0.04956	0.236114	0.341453
11752688_RREB1	-0.05724	0.236117	0.341453
11753477_CCDC24	0.049916	0.236139	0.341465
11752963_SLC25A30	-0.04771	0.23614	0.341465
11761368_SLC6A9	-0.09978	0.236182	0.341517
11728868_ELAVL3	0.078191	0.236235	0.341583
11754649_ITK	-0.06458	0.236284	0.341644
11727788_PIGS	-0.04602	0.236352	0.341732
11745305_BIN3-IT1	-0.11022	0.236373	0.341753
11729832_CLDN8	0.124988	0.236412	0.341799
11753759_HINT1	0.04422	0.236473	0.341877
11748922_NSMF	-0.06755	0.236496	0.341899
11752484_TMEM209	0.069972	0.236559	0.341982
11763800_RSPH14	-0.05424	0.236581	0.342003
11738753_OR4X2	-0.05666	0.23661	0.342035
11721651_CDH5	0.0662	0.236637	0.342063
11749865_ABLIM2	-0.0595	0.236699	0.342142
11751662_IYD	-0.09509	0.236708	0.342145

11742470_SLC35F4	0.065507	0.236752	0.342199
11743247_DEFB114	-0.09186	0.236766	0.342209
11740285_EFCAB1	0.083836	0.236792	0.342237
11731061_ZNF516	-0.10203	0.236806	0.342247
11740223_LRRC26	-0.06876	0.236827	0.342267
11733350_ZNF287	0.12491	0.236835	0.342268
11735805_SPDYE4	-0.07953	0.236853	0.342284
11722635_IL2RB	-0.14396	0.236913	0.342361
11727384_ATP5J2-PT	-0.04342	0.236944	0.34239
11751082_MLC1	-0.11145	0.236947	0.34239
11755642_KIF1A	-0.05344	0.236971	0.342412
11749446_GFRA2	-0.10717	0.236981	0.342412
11748366_ASAP3	-0.11722	0.236983	0.342412
11720872_INPP5E	0.055905	0.237033	0.342465
11762383_TDRD1	-0.0987	0.237034	0.342465
11739563_ITPR1	-0.10384	0.237053	0.342469
11728613_TRIM10	-0.10187	0.237061	0.342469
11734925_ZDHHC15	-0.08942	0.237062	0.342469
11742194_LGSN	0.120418	0.237065	0.342469
11752378_ZNF114	-0.17028	0.237117	0.342524
11745177_TPD52L2	-0.07365	0.237123	0.342524
11719385_MAPRE3	0.109571	0.237124	0.342524
11754538_SLC9A5	-0.0519	0.237156	0.34256
11760780_ZAK	-0.06941	0.237249	0.342685
11729577_ATP1A3	-0.05285	0.237272	0.342698
11764247_WISP1	-0.0752	0.237277	0.342698
11763760_PLVAP	-0.0721	0.237279	0.342698
11760032_CRB1	-0.12274	0.237312	0.342735
11736078_NEK9	-0.06796	0.237369	0.342807
11732531_N4BP2L1	-0.1323	0.237447	0.342897
11738915_CD200R1	0.060647	0.237447	0.342897
11738645_SLC26A5	-0.08117	0.237452	0.342897
11731586_DOK3	0.078085	0.237477	0.342923
11742333_OR4M1 ///	0.049099	0.2375	0.342946
11741903_LOC286238	-0.14206	0.237511	0.342948
11730908_HRC	-0.08173	0.237516	0.342948
11746054_TSC22D4	0.047574	0.237563	0.343006
11734405_SCRT2	-0.07929	0.237662	0.343139
11761091_HEXDC	-0.10435	0.237677	0.343151
11751574_TMEM119	-0.05199	0.237738	0.343229
11730473_PDE3B	0.141644	0.23775	0.343235
11721101_SGSH	-0.05388	0.237818	0.343317
11759856_SNRPG	-0.12112	0.23782	0.343317
11740146_TRMT10A	-0.11154	0.237837	0.34333
11732711_TVP23C	-0.07407	0.237909	0.343425
11756960_ZC3HC1	0.052305	0.237918	0.343428
11739490_ATP10D	0.089102	0.237964	0.343482
11718350_NLRP2	-0.12209	0.23797	0.343482
11744044_PCYT2	-0.06896	0.237999	0.343514
11734477_SPATA9	-0.06593	0.238009	0.343519
11750905_MOV10L1	-0.0936	0.238088	0.343623

11736657_REST	-0.05509	0.238096	0.343624
11729045_TCERG1L	-0.12059	0.238111	0.343635
11748289_SLC23A1	-0.09437	0.238187	0.343724
11732565_SLC6A14	-0.09299	0.238187	0.343724
11724697_PHF24	-0.11296	0.238338	0.343932
11731325_CAPN8	-0.12018	0.238364	0.34396
11746976_GAPVD1	0.048281	0.238372	0.34396
11728968_AASDHPPT	0.058776	0.238459	0.344076
11730284_SMU1	0.113084	0.23847	0.344082
11762333_NDUFA13	-0.05181	0.238486	0.344095
11728247_TOP3A	-0.05014	0.238585	0.344228
11732948_IDO2	0.122747	0.238601	0.344241
11718456_SLC43A2	-0.07551	0.238615	0.34425
11730267_PPIG	0.043084	0.238631	0.344263
11760402_INADL	-0.06469	0.23864	0.344266
11762722_CPA6	-0.0838	0.238679	0.344312
11731523_ZNF592	0.056374	0.238741	0.344392
11732569_SLCO1B3	0.052721	0.238806	0.344458
11725422_ARHGAP35	0.046541	0.238808	0.344458
11754501_SMIM6	-0.0624	0.238808	0.344458
11748049_SUN2	-0.08683	0.238845	0.344501
11763681_PPARA	-0.09601	0.238857	0.344503
11735837_SPIC	-0.10539	0.23887	0.344503
11726784_LRFN5	0.103479	0.238873	0.344503
11738456_C20orf197	0.158678	0.238874	0.344503
11747317_RFWD3	-0.06476	0.238891	0.344516
11738388_IFNA1 ///	0.087361	0.239017	0.344688
11737407_GLT8D1	0.055929	0.239051	0.344722
11741843_ACRC	0.058956	0.239055	0.344722
11757555_UBE2D2	0.049379	0.239073	0.344738
11761885_PPP1R1C	0.127346	0.239096	0.344761
11755734_CCDC107	0.086206	0.239105	0.344764
11735792_THOC5	-0.05112	0.239135	0.344796
11762787_RPS3A	-0.06805	0.239141	0.344796
11731386_HYAL4	0.052888	0.239178	0.344838
11756940_ZSCAN21	-0.10305	0.239185	0.344838
11736362_MEDAG	-0.10109	0.239257	0.344932
11762555_ERCC4	-0.14219	0.239317	0.345
11731190_KIAA2022	-0.08097	0.239318	0.345
11735335_CPLX4	-0.20112	0.239338	0.345019
11728740_TBC1D20	0.053667	0.239348	0.345022
11747271_SAFB	-0.09233	0.239362	0.345033
11731704_ADAMTS8	-0.0843	0.239379	0.345037
11751785_CLDN22	-0.10746	0.239379	0.345037
11721253_MYLPF	-0.08883	0.239389	0.345041
11759264_KRTAP3-3	-0.07147	0.239407	0.345057
11758992_MAN2B2	-0.11624	0.239435	0.345087
11759698_STEAP1B	0.05131	0.239481	0.345135
11717081_VPS52	-0.07959	0.239482	0.345135
11724175_TSPAN11	-0.07731	0.239504	0.345156
11753845_CCL25	-0.05702	0.239551	0.345213

11751387_FAM69C	-0.06463	0.23956	0.345216
11733896_TADA2B	-0.05639	0.239583	0.345239
11763641_FMNL1	-0.09648	0.239599	0.345252
11719198_CCND2	-0.07148	0.239664	0.345335
11726517_WDR11	-0.04289	0.239705	0.345385
11732710_FAM46D	0.058058	0.239768	0.345455
11762708_POTEC	-0.09919	0.23977	0.345455
11717136_PCSK1N	0.08002	0.239775	0.345455
11760832_TBP	-0.08378	0.239816	0.345504
11741364_KCNJ1	-0.10016	0.239856	0.345545
11730995_ACTN3	-0.05652	0.239858	0.345545
11737278_CYP7A1	-0.11373	0.239924	0.345624
11759397_BTN2A3P	0.090877	0.239928	0.345624
11732187_PI4K2A	-0.05176	0.239983	0.345693
11717980_C7	-0.06392	0.240004	0.345713
11736969_TMEM213	-0.12216	0.240018	0.345723
11721252_MYLPF	-0.06499	0.240047	0.34575
11718851_SRPK1	0.047629	0.240051	0.34575
11715754_PRL	-0.07401	0.240064	0.345757
11750254_LINC00575	-0.106	0.24007	0.345757
11759169_CFAP74	-0.11845	0.240105	0.34579
11722674_HIF3A	-0.07944	0.240109	0.34579
11746375_NOVA1	-0.06928	0.240124	0.34579
11751933_ZNF793	0.074068	0.240127	0.34579
11730347_PDE6H	-0.09891	0.240128	0.34579
11722306_ARHGAP23	0.050389	0.240169	0.345839
11731046_RIMKLA	-0.05804	0.240192	0.34586
11746263_FECH	-0.05181	0.240198	0.34586
11741999_OR10J1	-0.08447	0.240219	0.345881
11724559_GNA15	0.05788	0.24023	0.345886
11725258_FZD10	0.070983	0.240241	0.345892
11721972_JADE3	0.055157	0.24037	0.346067
11761683_PYHIN1	-0.06179	0.240385	0.346079
11737037_TCEAL6	0.058479	0.240482	0.346209
11761601_LILRA6 //	-0.08416	0.240531	0.346269
11753501_PTCRA	-0.09509	0.240551	0.346287
11746671_NKAIN1	-0.14308	0.240559	0.346288
11758591_CXorf57	-0.11668	0.240595	0.34633
11731960_LOC401052	-0.05581	0.240646	0.346394
11750659_ABCG1	-0.06421	0.24067	0.346418
11730881_NEBL	-0.05987	0.240688	0.34643
11759154_STARD7-AS	0.083704	0.240693	0.34643
11727326_GFI1	-0.07604	0.240706	0.34643
11761722_CKMT1B	-0.12008	0.240707	0.34643
11746045_ELF2	0.077357	0.240717	0.346434
11738673_UTF1	-0.0985	0.24076	0.346486
11741854_CCDC140	-0.05777	0.240792	0.346522
11750768_OSBPL6	-0.06794	0.240809	0.346535
11744567_CD72	0.132702	0.240834	0.346561
11760840_KIAA2012	-0.07935	0.240877	0.346614
11748376_DAZL	-0.11148	0.240967	0.346733

11736970_TMEM213	-0.10674	0.240991	0.346757
11734040_ELK1	-0.04467	0.241005	0.346766
11758207_PKN2	-0.06205	0.241016	0.346773
11719081_CDC5L	0.044928	0.241075	0.346844
11737699_PCDHGB7	-0.11547	0.24108	0.346844
11751093_CHODL	0.061604	0.241115	0.346884
11727110_WSCD2	-0.05159	0.241155	0.346931
11727209_GLRX2	-0.05103	0.241241	0.347045
11739858_PACSIN1	-0.10343	0.241248	0.347045
11734156_PRDM14	-0.0676	0.241269	0.347065
11732292_MTMR2	-0.09045	0.241328	0.347141
11736425_STARD4	0.069847	0.241373	0.347195
11753803_CYCS	0.046906	0.241383	0.347198
11724575_ECE2	-0.06074	0.241436	0.347264
11726957_TCF12	0.057993	0.241468	0.347291
11759472_SP9	-0.07022	0.241468	0.347291
11744875_C11orf70	-0.0993	0.24148	0.347298
11761579_DMKN	0.087321	0.241501	0.347312
11762373_TMC3	-0.0763	0.241505	0.347312
11753281_RIC3	-0.0979	0.241514	0.347316
11715328_SPATA31D3	0.058716	0.241539	0.347342
11753733_PAOX	-0.11676	0.24162	0.347448
11731790_FGF11	-0.06305	0.241628	0.347448
11716855_COPG1	-0.04737	0.241682	0.347516
11750430_KBTBD11	-0.05682	0.241714	0.347551
11750519_ASMTL-AS1	0.064897	0.241752	0.347596
11723334_GMIP	-0.08716	0.241776	0.347613
11744937_EGFL7	0.086778	0.241778	0.347613
11755666_PDE10A	0.135802	0.241796	0.347617
11733429_HCN3	0.04796	0.241799	0.347617
11751382_P2RY12	-0.10901	0.241809	0.347617
11753124_ENDOU	-0.11924	0.241809	0.347617
11747029_SDR16C5	-0.0858	0.241842	0.347653
11746981_ARHGEF26	0.083748	0.241889	0.347709
11732269_CASD1	0.079433	0.241895	0.347709
11720242_TCN2	-0.08134	0.241902	0.34771
11740904_TMEM236	-0.09768	0.241933	0.347744
11715377_COX4I1	-0.04564	0.241952	0.347761
11722956_GLTPD2	-0.06682	0.241982	0.347784
11725305_CPSF6	-0.05817	0.241984	0.347784
11716983_CDC34	0.057074	0.241989	0.347784
11763959_RPS2 ///	0.078056	0.242046	0.347856
11761881_ZNF33A	-0.12682	0.242076	0.347888
11762914_RAP1B	-0.08156	0.242084	0.34789
11730862_CTTNBP2NL	0.084517	0.242213	0.348064
11730987_SBSPON	-0.08728	0.24222	0.348064
11762019_CENPI	-0.13214	0.242245	0.34809
11737496_CD200R1	0.110212	0.242252	0.34809
11752992_HSP90B3P	-0.09283	0.242279	0.348119
11763246_HLA-DQB1	-0.06628	0.242303	0.348143
11754266_E2F3	0.060355	0.242324	0.348162

11739031_ACTR2	-0.04956	0.242335	0.348166
11761208_PSMB7	-0.07142	0.242341	0.348166
11745590_UBXN1	0.045316	0.242367	0.348184
11748040_ATL2	0.063324	0.242373	0.348184
11729155_CEACAM6	-0.09051	0.242374	0.348184
11736108_ARL8A	-0.07272	0.2424	0.34821
11741900_KIR2DL1 /	-0.07225	0.242426	0.348219
11744719_C4BPA	-0.07918	0.242432	0.348219
11754701_SLC5A2	-0.06347	0.242435	0.348219
11731885_COX4I2	-0.11356	0.242435	0.348219
11736755_PCDHB13	0.078069	0.242442	0.348219
11731359_FAM19A4	0.088916	0.242478	0.34826
11717402_ARF6	-0.05595	0.242484	0.34826
11750267_INPP5J	-0.07076	0.242493	0.348262
11735607_C12orf56	0.058067	0.242529	0.348304
11742865_TJP3	-0.08573	0.242569	0.348332
11732667_RSPH10B /	0.114571	0.24257	0.348332
11751418_PSD4	-0.08271	0.242572	0.348332
11741994_SYNGR1	-0.08087	0.242578	0.348332
11716378_TOMM20	-0.09057	0.242642	0.348414
11763613_LINC00885	-0.05872	0.242708	0.348491
11733595_PLEKHG6	-0.06826	0.242709	0.348491
11737352_PNPLA5	-0.07288	0.242809	0.348616
11753377_IL17RE	-0.06385	0.242811	0.348616
11756018_C4orf22	-0.05607	0.24283	0.348633
11730783_ORC4	0.057223	0.242855	0.348659
11740775_SOX1	-0.07886	0.242867	0.348666
11740247_EXOSC3	-0.04312	0.242892	0.348691
11761891_VCX	-0.07591	0.242907	0.348703
11724554_PCGF5	0.054925	0.242933	0.34873
11736174_KIAA0355	-0.12334	0.242992	0.348804
11720794_SRPX	0.05436	0.243113	0.348968
11758357_RPL9	-0.04423	0.24312	0.348968
11755228_PTK6	-0.09079	0.243145	0.348989
11741028_NDST4	-0.06041	0.243149	0.348989
11730047_SLITRK5	-0.07975	0.243183	0.349028
11731858_ZNF529	-0.06496	0.243197	0.349038
11734534_MFSD6	-0.09842	0.243219	0.349049
11722496_RPTOR	-0.05617	0.243219	0.349049
11725203_ESCO1	0.049328	0.243255	0.34909
11761025_DMAP1	-0.08387	0.243331	0.349183
11750517_HPGD	-0.1159	0.243334	0.349183
11748569_IZUMO4	0.093319	0.243362	0.349213
11728112_SARM1	-0.06059	0.243377	0.349223
11715300_SSX3	0.151174	0.243407	0.349256
11715257_XAGE3	-0.10717	0.243438	0.349291
11724149_SKAP1	-0.14886	0.243453	0.349302
11737609_GRIN3B	0.090838	0.243475	0.349324
11757886_SRBD1	0.050115	0.243493	0.349338
11733121_DAZL	0.117877	0.243575	0.349447
11751256_STOML1	0.080915	0.24361	0.349486

11722330_MEX3A	-0.06172	0.243637	0.349513
11743188_CFAP97	0.086509	0.243643	0.349513
11725447_PLD6	-0.04195	0.243676	0.349551
11724625_SPATA7	0.093366	0.24372	0.349596
11737676_TSPEAR	-0.11218	0.243722	0.349596
11733238_RPGRIP1L	0.047517	0.243748	0.349623
11737470_PRSS57	-0.05089	0.24378	0.349658
11738661_C2orf27A	0.073842	0.2438	0.349677
11740460_C2orf78	-0.08	0.243858	0.349749
11734622_KRTAP1-3	0.139819	0.243869	0.349755
11744476_TRABD	-0.05914	0.243914	0.349809
11740931_SEZ6L2	-0.05867	0.243934	0.349827
11739855_ZFYVE16	-0.11247	0.243947	0.349836
11745273_ADGRL2	0.072724	0.244007	0.349912
11732839_ERC2	-0.09022	0.244045	0.349957
11758712_SELT	0.05227	0.244103	0.350014
11728339_CENPBD1	0.068667	0.244109	0.350014
11759286_RRP1	-0.06052	0.244115	0.350014
11735830_IL1RL2	-0.07387	0.244116	0.350014
11738479_GLRA1	-0.15903	0.244121	0.350014
11750848_TOMM70A	0.053945	0.244199	0.350116
11749915_SYNPR	-0.09708	0.244285	0.35022
11755785_SEC31B	0.131085	0.244286	0.35022
11726070_MAP4K4	-0.04849	0.2443	0.35023
11756998_GPR35	-0.04668	0.244372	0.350322
11741247_CPS1	0.090075	0.244399	0.350351
11763205_KRT16P1	-0.04492	0.244446	0.350406
11735510_CCDC149	-0.05498	0.244452	0.350406
11728364_IL20RA	-0.15097	0.244514	0.350485
11726508_CDKN2B	-0.07794	0.244531	0.350499
11740480_GALNT8 //	-0.10558	0.244544	0.350508
11735191_ADGRE2	-0.06112	0.244568	0.350524
11717604_TRIP4	0.049369	0.24457	0.350524
11734833_TAF9B	0.043515	0.244581	0.350529
200003_PM RPL28	0.041793	0.244594	0.350537
11725787_TMA16	-0.05945	0.244628	0.350576
11738148_PREX2	0.160097	0.244657	0.350608
11724401_HS3ST2	-0.05567	0.244692	0.350647
11729913_IBA57	-0.06696	0.244706	0.350658
11734558_ADIPOQ	0.065108	0.244753	0.350715
11740243_TOX	0.099501	0.244771	0.35073
11718904_FILIP1L	0.144927	0.244799	0.350759
11727794_EIF3E	0.042051	0.244834	0.350799
11724965_BLOC1S1-R	0.068579	0.244887	0.350865
11742403_CLLU1OS	-0.07582	0.244937	0.350919
11738718_OR5K2	-0.0686	0.244942	0.350919
11747313_OXA1L	0.0419	0.244946	0.350919
11755660_SCNN1D	0.059843	0.244987	0.350967
11746822_LOC100289	0.08858	0.245038	0.35103
11761263_LASP1	-0.06548	0.245079	0.351078
11756669_HPN	0.067217	0.245153	0.351174

11726651_GDAP1L1	-0.07149	0.245176	0.351197
11748548_P2RX4	-0.1188	0.245213	0.351225
11756339_SARDH	-0.06853	0.245216	0.351225
11761624_SSU72	-0.05822	0.245217	0.351225
11739518_FLRT3	0.061783	0.245228	0.351231
11729483_KLF8	-0.07482	0.245265	0.351273
11715181_KRTAP10-9	-0.0748	0.245283	0.351284
11715701_PFN2	-0.04157	0.245287	0.351284
11721266_TMPRSS4	-0.10112	0.245296	0.351287
11730413_CALCA	-0.13475	0.245308	0.351294
11722152_EIF2AK3	0.077906	0.245333	0.351319
11752047_FERMT1	-0.05387	0.245352	0.351335
11735755_PYHIN1	-0.06369	0.245417	0.351419
11734063_CD200R1	0.104042	0.245468	0.351474
11745117_NAPA	-0.04447	0.24547	0.351474
11733276_SLC2A2	-0.09539	0.245536	0.351558
11737112_ZNF396	-0.04693	0.245568	0.351593
11738068_SLC28A3	0.145942	0.245594	0.35162
11757969_JUND	-0.04697	0.24567	0.351719
11736882_FCRL1	-0.07797	0.245696	0.351737
11746579_ITIH1	-0.19525	0.245706	0.351737
11763856_HOMER3	-0.07965	0.245717	0.351737
11746996_CCDC7	-0.10436	0.245719	0.351737
11762468_ESR2	0.084614	0.245719	0.351737
11736861_PIH1D3	-0.0889	0.245777	0.35181
11743430_RBM27	-0.05211	0.245789	0.351818
11748048_SUN2	0.085543	0.245806	0.351831
11751052_STRAP	-0.04412	0.245879	0.351925
11759425_FAM127A	0.048449	0.245895	0.351937
11738091_ABTB1	0.05248	0.245972	0.352038
11735221_BTLA	0.126932	0.246005	0.352074
11754807_ZNF74	0.04954	0.246028	0.352098
11734635_KRTAP10-5	-0.05805	0.24604	0.352105
11762246_PGAM1P4	-0.07841	0.246047	0.352105
11729333_PADI1	0.092117	0.246084	0.352147
11752505_MAPK8IP1	0.068157	0.2461	0.352153
11734449_SOHLH1	-0.07166	0.246103	0.352153
11732644_SYT6	0.089871	0.246178	0.352251
11738267_C7orf33	-0.08062	0.246215	0.352284
11745247_PROB1	-0.08339	0.246216	0.352284
11735601_CHRNE	0.132343	0.246269	0.35235
11746552_TMEM106A	0.065092	0.246283	0.352352
11762277_CYP2F1	-0.07458	0.246285	0.352352
11722903_GSTT2 ///	-0.09802	0.246302	0.352365
11752812_TTYH1	-0.08951	0.246423	0.352528
11719675_VCAM1	0.067492	0.246478	0.352597
11759060_C22orf39	-0.12187	0.246499	0.352612
11731379_MED25	-0.05112	0.246503	0.352612
11715285_C15orf40	0.079141	0.246519	0.352625
11751303_GORASP2	0.062483	0.246537	0.35264
11740427_PSAPL1	-0.05982	0.246578	0.352688

11727882_BBS12	0.155806	0.246663	0.352797
11756212_ELP3	-0.05574	0.246668	0.352797
11722855_PDGFD	0.061573	0.246692	0.352821
11759338_ELAVL4	0.07913	0.246707	0.352822
11762740_ABHD6	-0.09429	0.246708	0.352822
11728077_PRKCQ	-0.09431	0.246732	0.352846
11751892_NWD1	0.055969	0.246748	0.352849
11746413_ANKS3	-0.06056	0.246748	0.352849
11731234_DEFB129	-0.08584	0.246796	0.352907
11745239_ATP9B	0.042897	0.246828	0.352935
11726864_FOXO4	-0.06325	0.24683	0.352935
11753102_MAP4K1	-0.05251	0.246863	0.352972
11754837_LRRC69	0.099536	0.246885	0.352993
11737046_RASSF8	-0.19287	0.24691	0.353018
11752295_SCN3A	0.079882	0.246947	0.353061
11739126_DNAJB2	0.064383	0.247078	0.353238
11763167_MARS	-0.05662	0.247113	0.353278
11724456_FBXW4	0.048575	0.247164	0.35334
11743255_VAPA	0.071318	0.247243	0.35343
11752909_FAM66B //	-0.10225	0.247244	0.35343
11725810_GUCA1B	-0.08904	0.247248	0.35343
11738825_PCDHA5	-0.07506	0.247283	0.353465
11726442_YBX2	-0.07251	0.247287	0.353465
11723097_VTCN1	-0.12335	0.247302	0.353476
11716030_NDUFB9	-0.04488	0.247348	0.353531
11760083_HYKK	-0.0873	0.247361	0.35354
11726953_ADAMTS14	-0.0657	0.247375	0.353548
11759209_AK9	-0.07771	0.247389	0.353554
11737086_TLL1	-0.114	0.247393	0.353554
11756762_GLMP	-0.04765	0.247416	0.353577
11716650_UBE2Z	0.048766	0.247445	0.353607
11730664_PDCL2	0.12445	0.24747	0.353626
11750482_LOXHD1	-0.0739	0.247472	0.353626
11746364_RNF207	-0.08621	0.2475	0.353655
11760636_NAGPA	-0.12937	0.247546	0.353707
11750564_TNRC6B	0.043915	0.247551	0.353707
11752279_TSPYL2	-0.07356	0.247665	0.353859
11760526_LIG3	-0.10229	0.247677	0.353866
11719237_INTS1	-0.14218	0.247701	0.35389
11743496_NRBP2	-0.08263	0.247733	0.353926
11748335_PRELID3B	0.047655	0.247746	0.353934
11736485_RASGEF1B	-0.07865	0.247766	0.353952
11734786_OLFM3	0.079379	0.24778	0.353962
11757862_VTN	-0.0495	0.247807	0.35399
11738324_USH2A	-0.10175	0.247815	0.353991
11759925_PRDM5	0.089841	0.247837	0.354013
11752807_EYA2	-0.05407	0.247927	0.35413
11749753_KIAA1328	-0.04333	0.247983	0.354197
11741482_CLCN5	-0.07488	0.247988	0.354197
11751209_LOC100129	-0.06993	0.248002	0.354207
11747242_EFS	0.07671	0.248018	0.354219

11760284_FOSL1	0.055614	0.248111	0.354334
11747131_REG4	0.094941	0.248118	0.354334
11763610_LOC101929	-0.08732	0.248133	0.354334
11733815_RPL22L1	-0.06241	0.248133	0.354334
11752554_VARS	0.057595	0.248134	0.354334
11741082_HTR1A	-0.09231	0.248154	0.354351
11745172_SYNPR	0.063238	0.248176	0.354373
11738804_CACNA1G	-0.05515	0.248259	0.354481
11751016_SOX7	-0.06689	0.248277	0.354495
11721491_FBP1	0.062395	0.248374	0.354624
11736911_TRIM14	0.056593	0.248391	0.354637
11759263_KRTAP3-3	-0.08924	0.248496	0.354778
11727228_FBXL8	-0.11306	0.248541	0.354832
11735766_C16orf54	-0.05253	0.248594	0.354897
11755045_ANKRD13A	-0.05663	0.248626	0.354932
11756389_HAPLN2	-0.05549	0.248638	0.354939
11737414_KIF6	-0.06096	0.248676	0.354975
11738244_AKR1C1 //	-0.07611	0.248678	0.354975
11740831_EPHA6	-0.09099	0.248736	0.355047
11728067_PTGDR2	0.053685	0.248762	0.355075
11736097_GGA1	0.055652	0.248771	0.355076
11764255_LOC105375	0.107278	0.248795	0.355082
11744251_ITGA8	-0.13154	0.248796	0.355082
11753080_DPF2	-0.05011	0.248796	0.355082
11722763_GATA2	-0.0497	0.248815	0.355091
11760008_BIRC5	-0.05529	0.248817	0.355091
11742774_CCDC112	-0.04582	0.248954	0.355275
11727945_ARHGAP8 /	-0.04639	0.249023	0.355364
11763942_MT1DP	-0.07869	0.249045	0.355385
11722450_POLR2D	0.069778	0.249054	0.355387
11753872_MT3	-0.06286	0.249068	0.355392
11718337_LMOD1	0.048442	0.249072	0.355392
11728541_MDGA1	0.060754	0.249082	0.355395
11745960_MTG2	0.049442	0.249121	0.355442
11718255_ABHD4	-0.05547	0.249182	0.355518
11757765_SGCD	-0.05642	0.249219	0.35556
11751267_ADAMDEC1	-0.04793	0.249406	0.355817
11722765_GIT2	-0.0462	0.249473	0.355902
11730036_SP5	-0.05634	0.249537	0.355983
11715295_CCL27	-0.08049	0.249553	0.355995
11740628_CARD14	-0.1145	0.24956	0.355996
11738977_KRTAP23-1	-0.07055	0.249613	0.35606
11749836_CSMD2	0.058631	0.249633	0.356079
11733917_OGDH	-0.06616	0.249728	0.356203
11728829_INHBB	0.099423	0.249778	0.356264
11746926_CCDC110	0.092014	0.249862	0.356372
11742322_OR2AK2	-0.0763	0.249868	0.356372
11757141_SNORA25 /	0.059032	0.24993	0.35645
11720654_BBX	-0.0511	0.250024	0.356573
11750922_AMT	0.072718	0.250205	0.356822
11719887_GIGYF2	-0.0571	0.25023	0.356847

11741988_MBOAT4	-0.06767	0.25024	0.35685
11719678_MED21	-0.06389	0.250248	0.356851
11735638_KRTAP5-2	-0.06503	0.250291	0.356902
11756682_INO80B-WE	-0.04598	0.250308	0.356913
11748765_CNOT6L	0.135592	0.250313	0.356913
11752966_ATF7	0.096587	0.250348	0.356952
11733280_ACHE	-0.05328	0.250372	0.356976
11722083_RUFY3	0.09095	0.250394	0.356998
11723211_KCNJ10	0.052537	0.250413	0.357013
11744206_PAH	-0.11123	0.250421	0.357014
11740609_RASSF6	0.079816	0.250436	0.357017
11747393_FRMD8	-0.06405	0.250437	0.357017
11746872_COL1A1	0.102759	0.25047	0.35705
11760951_RPS6KB2	-0.10668	0.250475	0.35705
11747129_SLC6A1	-0.08465	0.250515	0.357097
11731011_B3GALNT1	-0.12503	0.250547	0.357132
11752099_FOXP1	0.084201	0.250607	0.357206
11735013_GPR137	0.05679	0.250616	0.357209
11745032_ARSJ	-0.04581	0.250625	0.357212
11753584_MADCAM1	-0.06786	0.25066	0.357252
11757906_RPL10	0.040712	0.250715	0.357313
11740948_REXO1L1P	-0.07327	0.250726	0.357313
11750755_OLFM3	-0.06691	0.250726	0.357313
11758468_TSHZ2	-0.0471	0.250732	0.357313
11744568_STGC3	0.083055	0.250749	0.357326
11742502_PRR25	-0.08567	0.250767	0.357342
11749906_AQP9	-0.12538	0.250779	0.357348
11731292_STXBP5L	-0.06137	0.250812	0.357365
11716651_UBE2Z	-0.0508	0.250813	0.357365
11745884_UBE2Z	-0.0508	0.250813	0.357365
11717035_KIAA0141	-0.11213	0.250836	0.357387
11735470_LGALS14	-0.08006	0.250851	0.357398
11726729_KDM4A	-0.05243	0.250982	0.357575
11762983_MED25	-0.08097	0.251045	0.357654
11751190_DNMT3B	-0.06027	0.251058	0.357662
11755752_IL16	-0.04866	0.251075	0.357673
11724013_SOAT1	0.049092	0.25108	0.357673
11728871_ELAVL3	-0.06349	0.251116	0.357713
11761023_COQ9	0.100969	0.251124	0.357714
11721898_FAM3D	-0.09781	0.251165	0.357763
11727053_HOXB13	-0.07504	0.251179	0.357764
11763194_LOC100506	-0.17581	0.251181	0.357764
11733086_MAF	-0.05549	0.251194	0.357773
11717011_ATP6V1E1	0.047583	0.251223	0.357803
11739795_RHBDF2	-0.06742	0.251312	0.357912
11717067_GREM1	-0.11717	0.251314	0.357912
11745079_TNKS	0.0925	0.251396	0.358018
11738538_STMN1	0.091398	0.251461	0.3581
11742490_INCA1	-0.09717	0.25151	0.35816
11740697_MAGEB6	-0.07417	0.251566	0.358226
11736127_DPPA4	0.089854	0.251571	0.358226

11750171_TLE2	-0.05866	0.251603	0.358252
11742136_HTR3D	-0.04706	0.251604	0.358252
11735719_ALS2CR11	0.098283	0.251667	0.358331
11729311_SPX	0.086409	0.251679	0.358338
11719693_LARP7	-0.05409	0.251736	0.358403
11745763_PACSIN2	-0.05669	0.25174	0.358403
11722170_CORO7	-0.05573	0.251801	0.35848
11728629_PRR22	0.051925	0.25183	0.358504
11756292_CTSW	-0.05641	0.251832	0.358504
11745974_ZFAT	0.130266	0.25187	0.358536
11726344_GPA33	-0.06321	0.25187	0.358536
11746029_ZNF767P	0.06038	0.251877	0.358536
11761534_FANK1	-0.10287	0.251951	0.358622
11749236_CYP46A1	-0.06754	0.251952	0.358622
11731999_PDE11A	0.053022	0.251979	0.35865
11753642_TTTY6 ///	-0.08049	0.251993	0.35866
11728594_TDRD10	-0.05398	0.252022	0.35869
11738851_MMP16	0.050765	0.252029	0.35869
11718289_APBB3	0.061493	0.252051	0.358711
11726043_PCCB	0.047771	0.252099	0.358769
11749458_RAP1GAP2	0.088367	0.252208	0.358905
11762491_AIFM2	-0.06905	0.252209	0.358905
11756257_FLYWCH1	0.044769	0.252261	0.358968
11745441_TAF1A	0.050897	0.252352	0.359087
11744107_DLK1	-0.05521	0.25239	0.359131
11746730_TRIM29	-0.081	0.252414	0.359151
11752122_RALGDS	0.07455	0.252419	0.359151
11756825_SLC12A8	0.055112	0.252512	0.359273
11749720_OSBPL5	-0.06932	0.252584	0.359358
11736380_ZNF697	-0.09053	0.252587	0.359358
11720690_SLC35F6	-0.06368	0.252771	0.359609
11736377_TM2D3	-0.06641	0.252797	0.359636
11726843_MAPKBP1	0.086496	0.25286	0.35971
11748607_ARHGEF11	-0.05917	0.252864	0.35971
11745045_PERM1	-0.04995	0.252882	0.359723
11722250_MBD6	0.045203	0.252888	0.359723
11761381_SORBS2	-0.04817	0.252895	0.359723
11742956_ITFG2 ///	0.053774	0.252935	0.359761
11752030_SEMA6C	-0.05319	0.252936	0.359761
11755615_JAKMIP3	-0.08326	0.253014	0.359861
11757841_RANBP9	-0.04757	0.253031	0.359869
11748397_TULP3	0.053069	0.253034	0.359869
11761815_LRRC8C	-0.06706	0.253042	0.359869
11760245_CCDC163P	0.088223	0.253063	0.35989
11735252_SUPT3H	-0.05115	0.253142	0.359991
11743157_ETFDH	-0.09572	0.253164	0.360012
11718590_CHMP6	-0.0589	0.253217	0.360077
11722350_DCLK2	0.073552	0.253245	0.360104
11746376_IL16	0.05641	0.253254	0.360104
11725513_SMAD7	0.102484	0.253258	0.360104
11728653_IGFBP5	0.096881	0.25328	0.360125

11756412_NT5DC2	0.045045	0.253312	0.360151
11745386_ZNF638	-0.06522	0.253314	0.360151
11722038_FES	-0.05961	0.253465	0.360355
11762063_EPB41L5	0.173098	0.253488	0.360379
11763788_C14orf2	-0.11105	0.253498	0.360382
11715446_DNAJB1	-0.04448	0.253559	0.360458
11730904_APOBEC1	-0.07787	0.253571	0.360465
11728591_SHISA9	0.054736	0.253621	0.360525
11725919_SOGA1	-0.06471	0.253634	0.360533
11757772_RPLP0	0.040056	0.253674	0.36058
11737920_ZNF233	-0.07091	0.253694	0.360597
11719504_DHX36	-0.04462	0.253765	0.360684
11755833_GRM8	-0.08751	0.253769	0.360684
11750341_RGAG1	-0.08564	0.253792	0.360706
11747419_MOGS	-0.06737	0.25381	0.36072
11743207_DEFB108B	-0.07961	0.253828	0.360736
11736803_FGF9	-0.08879	0.253836	0.360736
11722848_PPP3CA	0.056743	0.253876	0.360781
11761342_SLC9A1	-0.05555	0.253882	0.360781
11759261_DCAF12L2	-0.06713	0.253924	0.36083
11750157_CD4	-0.07237	0.253984	0.360902
11762904_KRT17	-0.11605	0.253997	0.360902
11730214_DUOX2	-0.1094	0.253997	0.360902
11763776_THEMIS2	-0.06927	0.254008	0.360908
11716935_SOD1	-0.04364	0.254037	0.360939
11732777_PGLYRP4	0.057563	0.25405	0.360945
11732784_TRIM42	-0.10284	0.254074	0.360969
11734505_ASPDH	-0.07198	0.254098	0.360993
11740289_RGS9BP	-0.13019	0.254124	0.361014
11739357_ERG	-0.08775	0.254127	0.361014
11761418_IL23A	-0.09463	0.254157	0.361046
11731950_SAMD3	0.126292	0.254209	0.361103
11763905_MAGED4 //	0.09827	0.254212	0.361103
11750900_PGR	-0.07715	0.254255	0.361153
11741204_PTPDC1	-0.0847	0.254297	0.361202
11753708_CIDEC	-0.0575	0.254366	0.36129
11754452_RRBP1	-0.06119	0.254407	0.361337
11741025_PIP5K1A	0.079876	0.254416	0.36134
11743096_TMEM65	0.057188	0.254452	0.361375
11753260_HSP90B1	-0.06174	0.254455	0.361375
11758469_PPP1R17	-0.08757	0.254482	0.361403
11742459_ADGRF2	-0.10062	0.254562	0.361505
11741902_SHBG	-0.05781	0.254601	0.361551
11738084_SLC36A3	-0.06759	0.254646	0.361604
11731782_CT45A1 //	0.044574	0.254676	0.361635
11754679_AKT1S1	-0.05534	0.254712	0.361676
11715118_HIST1H2BF	0.07129	0.254809	0.361803
11725025_ISYNA1	0.054739	0.254874	0.361886
11745641_KDM4E	-0.09628	0.254918	0.361932
11740626_TULP4	0.066187	0.254922	0.361932
11763287_OBSL1	0.057822	0.254973	0.361985

11734879_TTLL2	-0.07815	0.254974	0.361985
11757501_POLR2I	-0.04424	0.254997	0.362008
11726471_SOCS7	0.085925	0.255057	0.362062
11727128_TYRP1	0.099657	0.255058	0.362062
11757262_SCARNA20	0.162121	0.255058	0.362062
11759929_NOX5	-0.13698	0.255095	0.3621
11761508_FGF7	-0.13492	0.2551	0.3621
11715501_IGFBP7	0.06971	0.255209	0.362245
11759537_RPS28	-0.06351	0.255246	0.362287
11762484_TTF2	-0.16271	0.25532	0.362379
11749471_MCF2	0.084391	0.255332	0.362379
11741496_PNMA5	-0.0634	0.255336	0.362379
11750940_MKL1	-0.05356	0.255342	0.362379
11733137_ETNPPL	-0.05469	0.255348	0.362379
11762784_SCN5A	-0.08954	0.255386	0.362422
11732852_ATP1B4	-0.10573	0.255417	0.362457
11760630_RNF43	-0.05634	0.255444	0.362484
11716685_MYO1D	0.181064	0.255498	0.36255
11743082_TAF3	0.054659	0.255549	0.362612
11726686_IRF5	-0.04862	0.255638	0.362727
11724946_MAGI2	0.068705	0.255677	0.362773
11745414_SCD5	-0.05548	0.255711	0.36281
11750352_KIAA0930	-0.05309	0.255729	0.362826
11741894_DPH3P1	-0.09054	0.255762	0.362861
11760720_SLC52A3	-0.0477	0.255808	0.362917
11730520_ABCA9	0.131904	0.255833	0.362941
11737064_MTL5	-0.07477	0.255864	0.362972
11742447_RNF222	-0.08196	0.25587	0.362972
11737849_AMBN	-0.06123	0.255891	0.362991
11757456_NACA	-0.0411	0.255942	0.363054
11723397_ZNF385B	-0.05785	0.255958	0.363066
11722178_DR1	-0.06589	0.25598	0.363086
11750335_NR1I3	-0.04819	0.256004	0.363101
11723982_ARHGAP28	0.07864	0.256005	0.363101
11739737_DCUN1D2	0.065156	0.256039	0.363138
11720173_SF1	0.046095	0.256058	0.363155
11755511_MTL5	-0.06789	0.256072	0.363161
11726867_ERCC8	0.06202	0.256077	0.363161
11744205_NPDC1	-0.04391	0.256084	0.363161
11734944_SLAMF6	-0.08053	0.256103	0.363163
11718750_SPG11	0.052527	0.256105	0.363163
11753349_NRG3	-0.08224	0.256108	0.363163
11738382_HMHB1	-0.08921	0.256168	0.363238
11756471_MFSD2A	0.095684	0.25624	0.363329
11716110_PIGR	0.091193	0.256271	0.363363
11734346_CEBPE	-0.06692	0.256345	0.363456
11733621_NCMAP	-0.05025	0.256382	0.363499
11754926_B3GALT5-A	0.101076	0.256491	0.363642
11746641_GRM5	-0.08924	0.256515	0.363666
11738143_SSX2IP	0.071454	0.256606	0.363784
11739252_HSD3B1	-0.05168	0.25669	0.363891

11748443_WNT7B	-0.04062	0.256696	0.363891
11758271_CASK	0.066891	0.256719	0.363913
11734498_SNX22	0.079357	0.256748	0.363943
11758393_DNAJC30	-0.06642	0.25677	0.363964
11722815_RANBP2	0.075492	0.256802	0.363999
11757245_TFAMP1	-0.08079	0.256838	0.364039
11720196_CIPC	-0.0634	0.256845	0.364039
11751410_CCDC64	0.066399	0.256908	0.364117
11722017_OLR1	-0.12258	0.256935	0.364133
11736134_OAS2	-0.06489	0.256938	0.364133
11743860_ANGPTL7	-0.10445	0.256942	0.364133
11728175_BMP8A	-0.0551	0.256954	0.364135
11740095_LRRC2	0.066951	0.256964	0.364135
11717467_TTC38	0.058924	0.256965	0.364135
11759956_ACTR3C	0.116269	0.257015	0.364187
11751839_MKL1	0.107862	0.257017	0.364187
11726771_XAF1	-0.04685	0.257028	0.364193
11722640_SSBP3	-0.06636	0.25706	0.364227
11724512_SULT4A1	-0.06046	0.257082	0.364248
11752530_VPS53	-0.04997	0.257095	0.364256
11739180_ST6GALNAC	-0.04909	0.257321	0.364566
11733342_IDI2	-0.05808	0.257357	0.364593
11725621_TRMT2A	-0.06489	0.257362	0.364593
11749266_PRRT2	-0.07521	0.257363	0.364593
11720033_TRIM29	-0.07485	0.257381	0.364608
11721557_ABCA8	-0.09941	0.257406	0.364633
11743885_ADPRHL1	-0.04459	0.257419	0.36464
11738316_GRK4	-0.06168	0.257443	0.364664
11733333_NRP2	-0.0853	0.257481	0.364708
11731269_KRT31	-0.12908	0.25749	0.36471
11716964_PPM1F	0.06198	0.257556	0.364793
11761256_RABEP2	-0.04394	0.257575	0.364809
11740713_ELAVL4	-0.11181	0.257585	0.364812
11759365_GPR157	0.072488	0.2576	0.364821
11736935_POU2F3	0.07066	0.257611	0.364821
11741399_CLUL1	-0.1076	0.257621	0.364821
11753823_S100A8	-0.08891	0.257624	0.364821
11732208_MON2	-0.09362	0.257632	0.364821
11738848_GFRAL	0.054169	0.257636	0.364821
11762143_RNH1	-0.08557	0.257655	0.364838
11760986_CCDC22	-0.07744	0.257676	0.364857
11719864_SMAGP	0.05533	0.257705	0.364877
11760335_EMG1	0.096005	0.257705	0.364877
11726153_POU2AF1	0.098352	0.257761	0.364946
11738131_RNF212	0.048487	0.257769	0.364946
11737392_PROKR2	0.057503	0.257817	0.365004
11754882_BRSK2	-0.05289	0.257929	0.365148
11757416_ILKAP	0.04496	0.257934	0.365148
11740734_RBFOX1	0.105608	0.258022	0.365263
11739049_ACPP	0.118272	0.258124	0.365396
11737271_ZNF805	-0.06602	0.258313	0.365653

11722854_HABP4	0.051784	0.25836	0.365708
11728624_PAX2	-0.07645	0.258378	0.365723
11734966_NFATC4	-0.05775	0.258426	0.36578
11755187_ERMP1	-0.06315	0.25851	0.36589
11750324_GAS7	-0.05719	0.258522	0.365896
11746836_TMEFF2	-0.07783	0.258559	0.36593
11739298_LRRFIP1	-0.04902	0.258562	0.36593
11733676_TEKT4	-0.06284	0.258568	0.36593
11731725_B4GALT7	0.060523	0.25861	0.365977
11718060_TTYH2	-0.06074	0.258624	0.365988
11741687_RHBDL2	0.061461	0.25866	0.366027
11728657_IGFBP5	-0.06392	0.258732	0.366118
11726687_IRF5	-0.10574	0.258874	0.366309
11758235_SRRM1	0.049301	0.258938	0.366389
11760864_TMEFF2	0.084193	0.258996	0.366461
11724251_SLC39A8	0.069904	0.25901	0.366469
11723174_FNDC1	0.070159	0.25902	0.366474
11761631_JAK3	-0.08055	0.259063	0.366523
11724891_POLRMT	-0.06038	0.259097	0.366561
11735670_LINC00336	-0.04191	0.259104	0.366561
11722707_MAP6	-0.09683	0.259139	0.366598
11737645_FAM150A	-0.07691	0.259145	0.366598
11755171_CAMSAP3	-0.07373	0.259208	0.366675
11731825_GPR83	0.07494	0.259233	0.366701
11729693_SERPINB3	-0.07104	0.259242	0.366703
11720891_USP20	0.063237	0.259346	0.366829
11754119_PLAU	0.059959	0.259347	0.366829
11734406_LRRC36	-0.144	0.259424	0.366928
11750130_MRVI1	-0.09439	0.259458	0.366966
11732742_ATP4B	-0.10333	0.259493	0.367004
11749911_SFTPB	-0.06001	0.259516	0.367026
11752408_R3HDM2	-0.05545	0.259605	0.367141
11744703_SYDE1	0.0638	0.259667	0.367219
11763110_DEAR	0.084061	0.259677	0.367222
11731530_DOK1	-0.09819	0.259702	0.367247
11763624_GADD45B	0.069952	0.259724	0.367267
11757536_ACADM	0.052142	0.259736	0.367273
11716478_LARP1	-0.04022	0.259808	0.367365
11760349_UQCC1	-0.07122	0.259826	0.367373
11747446_HLA-DOB	-0.09928	0.259835	0.367373
11734524_C6orf15	-0.10077	0.259837	0.367373
11749364_SAP30BP	-0.04804	0.259888	0.367435
11738536_WNT1	-0.13684	0.259901	0.367443
11723495_RNF166	-0.05932	0.259915	0.367449
11723317_FAM234B	-0.09725	0.25992	0.367449
11716479_F13A1	-0.13073	0.260002	0.367544
11754453_NUDT4 ///	-0.04481	0.260002	0.367544
11761053_ROBO3	-0.09558	0.260026	0.367566
11753784_RPL34	0.044285	0.260041	0.367578
11735307_C9orf139	0.06618	0.260111	0.367666
11750533_C1QL3	0.053701	0.260144	0.367696

11746562_ZFP36L1	-0.05592	0.260147	0.367696
11735613_ASIC4	-0.05849	0.260186	0.36774
11734654_ATP5F1	0.069134	0.26025	0.367819
11735807_SPATA32	0.099408	0.260261	0.367824
11761719_HPS1	0.042806	0.260287	0.36785
11735550_LMNTD2	-0.08495	0.260311	0.367869
11741573_ZNF219	-0.04623	0.260315	0.367869
11732789_OOEP	-0.04677	0.260325	0.367873
11731818_CD40	-0.05597	0.260384	0.367945
11739533_PPIE	-0.04665	0.260414	0.367973
11750691_ZMYM6	0.092792	0.260419	0.367973
11744009_LZTS3	-0.056	0.260434	0.367984
11744836_RPL35	-0.04189	0.260451	0.367997
11730951_TMEM151B	-0.05817	0.260464	0.368005
11730025_UPK3A	-0.09383	0.260476	0.368011
11752541_PPIL2	-0.07437	0.260496	0.368028
11747033_LOC162137	0.073037	0.260511	0.36804
11728853_TUB	-0.05512	0.260535	0.368063
11717458_NLGN2	-0.07906	0.260596	0.368137
11723745_SLC13A5	-0.08377	0.260603	0.368137
11750433_CCT3	0.042167	0.260662	0.368205
11727284_CUX2	-0.09367	0.260668	0.368205
11738980_CD4	-0.12501	0.260674	0.368205
11746811_KCTD3	0.046259	0.260819	0.368399
11739593_USH1C	0.048523	0.260833	0.368409
11761918_TRBC1 ///	0.061206	0.260842	0.368411
11757899_PWP1	0.041851	0.260851	0.368411
11724962_SRGAP3	-0.0642	0.260857	0.368411
11721801_ARL8B	0.052636	0.260883	0.368438
11734649_HNRNPCL2	-0.16722	0.260945	0.368514
11731266_NXPH2	-0.04859	0.261007	0.368592
11751044_FOXL2NB	-0.06584	0.261046	0.368635
11741693_SERHL	0.05812	0.261107	0.368711
11753698_IL20	0.167766	0.261164	0.368778
11736974_SMURF1	0.051561	0.26117	0.368778
11746063_IKBKB	-0.08584	0.261215	0.368831
11731670_PCDHB6	0.042445	0.261251	0.368872
11759600_PNISR	0.084418	0.261287	0.368912
11734886_SEMA3G	-0.0607	0.261306	0.368928
11750454_ETV7	0.054284	0.261329	0.36895
11719294_AP3B1	-0.04155	0.261429	0.369064
11754646_ERCC3	0.058197	0.261431	0.369064
11759351_RBP1	-0.0998	0.261433	0.369064
11720284_TRAK1	-0.04815	0.261489	0.369133
11738300_MCCD1	-0.07624	0.261508	0.36915
11756632_GNLY	0.057463	0.261518	0.369152
11737595_LINC01588	-0.06517	0.26158	0.369228
11751166_TRIM5	0.073945	0.261586	0.369228
11735149_ANGPTL6	-0.04698	0.261639	0.369292
11749853_DTNB	0.049121	0.26169	0.369353
11758419_TMEM106A	0.134198	0.261722	0.369383

11764233_C1orf56	0.097869	0.261727	0.369383
11736598_DLG3	-0.06589	0.261782	0.369451
11717825_STMN2	0.08255	0.261851	0.369538
11724588_ANKRD35	0.054094	0.261903	0.369601
11720606_SFRP2	0.092992	0.261946	0.36965
11763041_ANKRD18A	0.065797	0.262041	0.369773
11716706_TRIM44	-0.04136	0.262072	0.369795
11719303_EMC8	0.043063	0.262075	0.369795
11751541_PDCD1	0.072663	0.262079	0.369795
11746966_DNASE1L3	-0.08961	0.262096	0.369808
11760793_TBC1D26	-0.11277	0.262164	0.369894
11736196_DCX	-0.07795	0.262209	0.36994
11730591_MCMDC2	0.111235	0.262212	0.36994
11725742_SORBS1	-0.06229	0.26229	0.370039
11762174_DDB1	0.100579	0.262331	0.370083
11721620_FOXJ1	-0.04763	0.262336	0.370083
11759127_CD300E	-0.07405	0.262352	0.370095
11715680_C12orf57	0.045657	0.262368	0.370106
11742738_BBS9	0.052147	0.262385	0.370108
11731150_CBLN3	0.066347	0.262392	0.370108
11722308_TP63	-0.04974	0.262405	0.370108
11734528_ACPT	-0.06545	0.262405	0.370108
11761363_DLC1	0.073275	0.262407	0.370108
11716386_RAB11FIP5	-0.07289	0.262416	0.37011
11716040_RPS5	0.040175	0.262427	0.370115
11758129_CPLX3	0.07686	0.262461	0.370152
11754978_BMP1	0.065905	0.26247	0.370154
11733611_DOCK8	0.115271	0.262523	0.370218
11748836_PEX14	-0.05761	0.262532	0.37022
11733957_SPRR2F	-0.09713	0.262541	0.370223
11718817_MADD	0.053797	0.262557	0.370234
11758120_KCNB1	-0.04271	0.262588	0.37026
11729453_CYP2B6 //	-0.07659	0.26259	0.37026
11727373_PAPSS1	0.043134	0.262618	0.370289
11748394_FMO1	-0.05378	0.262634	0.3703
11735091_SLC16A6	0.070901	0.262646	0.370306
11731988_FAM19A3	-0.07176	0.262654	0.370307
11753522_NKX3-1	-0.0507	0.262742	0.370421
11760193_TMEM68	0.122313	0.262752	0.370424
11736391_ZNF662	0.134641	0.262774	0.370444
11737346_LINGO2	0.061553	0.262792	0.370459
11759455_TUBB7P //	-0.12383	0.262802	0.370462
11720017_CGNL1	-0.09273	0.262828	0.370488
11740503_APOBEC3D	-0.05841	0.262855	0.370516
11742599_OR6C76	-0.11295	0.262869	0.370524
11748866_RPH3AL	-0.12364	0.262903	0.370562
11727590_IL20RB	-0.05865	0.262939	0.370602
11738870_GLT6D1	0.095039	0.262967	0.370631
11724550_RSBN1	-0.05453	0.263008	0.370675
11723846_DEFB1	0.114562	0.263014	0.370675
11763697_SNHG9	-0.09935	0.263036	0.37069

11763130_IGKC	0.11474	0.263039	0.37069
11741703_HIST1H1T	-0.06771	0.263221	0.370936
11760148_ISCU	0.071592	0.263279	0.371006
11762544_DNAJC24	-0.07126	0.263452	0.371239
11760487_PDCL3	-0.05097	0.263588	0.371421
11716612_ABCF3	0.06069	0.263672	0.371516
11717505_TRAM2	-0.04243	0.263677	0.371516
11760152_LOC730098	-0.16312	0.263679	0.371516
11758504_FAM216B	0.068961	0.263719	0.371551
11752195_TAX1BP1	-0.04075	0.263719	0.371551
11721669_KLHDC4 //	0.064033	0.263807	0.371664
11735575_ZNF296	0.06205	0.263879	0.371755
11726752_C9orf91	-0.07148	0.263918	0.371789
11723490_GCLM	0.04515	0.263918	0.371789
11752717_CCNI	-0.04381	0.264047	0.37196
11731534_CCDC85C	-0.04661	0.264056	0.371962
11723447_MALL	-0.05178	0.264078	0.371982
11754744_WDR88	-0.10394	0.264116	0.372025
11738895_CAPN10	-0.08701	0.264158	0.372073
11738488_GPR148	-0.08144	0.264202	0.372125
11731909_KLK8	0.082055	0.264231	0.372154
11761627_WDR61	0.115237	0.264275	0.3722
11737994_LRRC3	-0.04478	0.264279	0.3722
11742597_OR8H2 ///	-0.08907	0.26429	0.372205
11736757_GAL3ST3	-0.05637	0.264314	0.372228
11736161_GBP6	-0.08129	0.264375	0.3723
11727875_DLX3	0.091921	0.26439	0.3723
11759444_BAX	0.048	0.26439	0.3723
11741278_ZFYVE27	0.087465	0.264395	0.3723
11760022_IFT172	-0.09682	0.264431	0.372339
11751562_C16orf45	-0.04275	0.264467	0.37238
11718140_EMP3	-0.04991	0.264483	0.372383
11719599_HOMER2	-0.05508	0.264491	0.372383
11725269_SLC12A3	-0.04485	0.264492	0.372383
11737558_PMS2P5	-0.04129	0.264519	0.372407
11762808_MPZL1	-0.05542	0.264525	0.372407
11726929_CACNA2D4	0.053919	0.264571	0.372462
11720001_SYNJ2BP	0.056423	0.264617	0.372515
11749629_ARHGAP33	-0.08189	0.264652	0.372554
11721346_MPEG1	-0.06509	0.264697	0.372602
11753870_ATOX1	-0.04223	0.264701	0.372602
11746621_RBM46	0.054948	0.264754	0.372666
11756437_RPS18	0.044832	0.264776	0.372686
11752973_DDX23	-0.05761	0.264836	0.37276
11718383_AIFM1	0.039502	0.264858	0.372763
11729931_ZNF496	-0.06446	0.264861	0.372763
11719341_TMEM41A	0.049031	0.264861	0.372763
11733533_CYP4F22	-0.17498	0.264924	0.372841
11735028_LRRC27	-0.06481	0.264933	0.372842
11763254_BMS1P20 /	-0.08735	0.264949	0.372855
11739473_MAGI1	0.085837	0.264964	0.372865

11727338_CARNS1	0.093822	0.264998	0.372901
11735714_OARD1	-0.05621	0.265128	0.373074
11763979_SRRT	0.06077	0.26526	0.373249
11718740_FAM160B2	0.067108	0.265314	0.373314
11720330_BRIX1	-0.0406	0.265329	0.373315
11725164_C7orf31	0.099594	0.26533	0.373315
11730018_PPP1R32	-0.05972	0.265367	0.373357
11738604_DEFB107A	0.076931	0.265447	0.373458
11743093_LIN7A	0.08181	0.265466	0.373466
11751644_PBXIP1	-0.042	0.265468	0.373466
11740777_FAM129C	0.057436	0.265482	0.373469
11763228_IGK /// I	-0.07553	0.265485	0.373469
11722264_MRPS10	0.050095	0.265521	0.373509
11759942_SLC2A13	-0.11591	0.265546	0.373533
11740889_ATP2A1	-0.07321	0.265601	0.3736
11762118_CHI3L2	-0.07369	0.265647	0.373641
11744394_EGFL7	0.061972	0.265653	0.373641
11749719_C9orf91	-0.06562	0.265655	0.373641
11721653_AGPAT4	-0.09152	0.265661	0.373641
11725918_SOGA1	-0.06417	0.265701	0.373687
11741387_ZNF530	0.086847	0.265721	0.373704
11742540_OR52E2	-0.10201	0.265809	0.373816
11729256_KANSL3	-0.05703	0.26585	0.373864
11738840_NUDT10	-0.05159	0.265862	0.373869
11722556_TMEM35	-0.11376	0.265894	0.373904
11737567_C1QTNF9B	-0.08116	0.265948	0.373958
11746035_GPATCH11	-0.05562	0.265952	0.373958
11757257_PISRT1	-0.04713	0.265955	0.373958
11723370_SHANK3	-0.06577	0.265968	0.373965
11735232_STARD7-AS	-0.05747	0.266015	0.37402
11722673_HIF3A	-0.07456	0.266025	0.374024
11724424_GPR68	0.077437	0.266058	0.374057
11733046_ABI1	0.050184	0.266064	0.374057
11747909_KDM4B	0.065891	0.266084	0.374074
11731239_GRHL2	-0.06617	0.266115	0.374107
11745147_ADCYAP1R1	-0.05388	0.266175	0.374172
11752046_HDAC7	-0.05092	0.266177	0.374172
11727734_CFAP126	-0.06425	0.266194	0.374186
11750857_SYNJ2	-0.06115	0.266212	0.3742
11727238_UST	-0.06153	0.266298	0.37431
11729567_LINC00922	-0.21071	0.266305	0.37431
11758340_HLA-DQA1	-0.04279	0.266357	0.374372
11731104_ATF7	-0.05326	0.266418	0.374446
11724829_STS	-0.08076	0.266445	0.374474
11763288_OBSL1	0.07742	0.266462	0.374486
11729230_KLF3	0.056243	0.266469	0.374486
11762393_VEPH1	0.072853	0.266481	0.374492
11739629_DROSHA	-0.05347	0.266493	0.374498
11727104_FGD5	0.06108	0.266511	0.374513
11732498_HCN2	0.095318	0.266522	0.374517
11760112_HCLS1	0.055821	0.266558	0.374557

11737266_RHD	0.153732	0.2666	0.374606
11728942_CCNDBP1	-0.10533	0.266629	0.374636
11738534_KRT9	-0.06675	0.266641	0.374643
11728590_MFSD13A	-0.04189	0.266777	0.374813
11739869_LRRN4	-0.08647	0.266778	0.374813
11734133_SLC2A4	-0.05628	0.266786	0.374813
11755764_PKD1L2	-0.07449	0.266798	0.374819
11715367_SEPT2	-0.04395	0.266835	0.37486
11740494_SLC6A12	-0.06867	0.26685	0.374871
11759528_MRPS31	0.166033	0.266965	0.375022
11746946_AGGF1	0.047811	0.267115	0.375222
11721071_DEF8	0.055576	0.267271	0.37543
11717045_C2	-0.09572	0.267289	0.375444
11722612_TUBGCP5	-0.05119	0.267305	0.375457
11733339_FANCE	0.068982	0.267335	0.375488
11752828_PXN	-0.06306	0.267406	0.375576
11761917_OLFM1	-0.07656	0.267436	0.375608
11721592_FANCC	0.046187	0.267459	0.37563
11732085_SLC13A2	-0.09117	0.267519	0.375703
11725370_VEGFA	-0.06199	0.267535	0.375714
11756042_ESR1	-0.06449	0.267545	0.375718
11718197_LHFP	0.046759	0.267583	0.37576
11755750_MOG	-0.04994	0.26761	0.375787
11750080_DDB2	0.042886	0.267644	0.375824
11734648_BPIFB3	-0.07693	0.267664	0.375841
11721211_SCRIB	-0.0502	0.267698	0.375879
11763940_LOC284441	0.049074	0.267712	0.375887
11743435_SEZ6L2	-0.08834	0.267751	0.375932
11735179_MORC1	-0.06254	0.267836	0.376039
11749591_GFAP	-0.07674	0.26787	0.376077
11761191_USB1	-0.05621	0.267917	0.376131
11749138_TRIM46	-0.06728	0.268003	0.376243
11742048_ITGB1	-0.08649	0.268025	0.376262
11750584_RNF17	-0.06049	0.268106	0.376365
11755658_ZNF594	0.077866	0.268147	0.376411
11752983_GPR68	-0.07936	0.268162	0.376414
11750067_SPIB	-0.08524	0.268169	0.376414
11747869_POLR2A	-0.05467	0.268172	0.376414
11734278_TSGA10	0.060073	0.268217	0.376466
11735858_RIIAD1	-0.06185	0.268228	0.376472
11730068_HGF	-0.0629	0.268241	0.376478
11736973_TBX5	-0.08615	0.268375	0.376648
11719209_NPTN	0.050073	0.268377	0.376648
11742614_OR4C16	-0.1488	0.268403	0.376669
11726889_ZFP36L1	-0.0551	0.268408	0.376669
11760058_SLC3A1	-0.08259	0.268429	0.376688
11749642_SLC4A10	0.068613	0.268454	0.376701
11745582_CFAP43	0.061066	0.268462	0.376701
11732866_MS4A4A	-0.11136	0.268467	0.376701
11730334_ZSWIM1	0.072128	0.268469	0.376701
11741259_GREB1L	0.083629	0.268485	0.376713

11754919_CAGE1	-0.06193	0.268501	0.376725
11745369_ZFYVE27	0.057231	0.268527	0.376748
11738864_ABHD12B	-0.12553	0.268533	0.376748
11723473_LYPLAL1	0.03942	0.268623	0.376863
11750850_FAM83F	0.069293	0.268675	0.376925
11746158_LINC00442	-0.07421	0.268689	0.376925
11746764_SCEL	0.117238	0.26869	0.376925
11723216_SFXN3	-0.0967	0.26871	0.376936
11754537_PHTF1	0.046123	0.268713	0.376936
11726296_ZNF500	-0.06541	0.268792	0.377035
11722622_TMEM8A	0.048267	0.268827	0.377063
11755626_PNLDC1	0.083166	0.268827	0.377063
11762188_SUGCT	-0.08119	0.268903	0.377145
11742256_SLC22A6	0.078792	0.268908	0.377145
11756702_CYP39A1	-0.05555	0.268909	0.377145
11738311_C15orf53	0.083165	0.268936	0.377172
11737737_GLYATL3	0.054572	0.268977	0.377219
11759271_IFNA5	-0.1757	0.269032	0.377286
11723852_BCL11A	-0.09518	0.269041	0.377287
11740569_ZNF665	0.166773	0.269049	0.377288
11761877_NDUFS4	0.063172	0.269073	0.37731
11748064_UBAP2	0.05287	0.269115	0.377359
11716737_PDRG1	-0.05894	0.269162	0.377414
11741847_C2orf83	0.07943	0.269188	0.37744
11739861_ITGA6	-0.04161	0.269208	0.377451
11719413_PBX1	-0.04395	0.269212	0.377451
11752972_RGS9	-0.10117	0.269246	0.377488
11741741_SLC25A41	0.084805	0.269324	0.377587
11736162_GBP6	-0.06262	0.269426	0.377719
11740925_FAM151B	0.102717	0.269437	0.377723
11746039_ZFHX2	-0.14023	0.269477	0.377768
11752402_CLDN18	-0.06857	0.26951	0.377804
11739982_ZBTB40	-0.05015	0.269562	0.377866
11740073_HTATIP2	0.056599	0.269584	0.377886
11745364_STAT5A	-0.08304	0.269733	0.378084
11736908_ZBTB3	0.068313	0.269839	0.378222
11731263_ZNF365	-0.0876	0.269852	0.37823
11748904_DBF4B	-0.11432	0.269877	0.378254
11741255_APOBEC3F	0.074872	0.269984	0.37839
11731809_FCRL4	-0.04839	0.26999	0.37839
11748966_JARID2	0.062276	0.270045	0.378456
11749439_WWC3	0.049326	0.270071	0.378481
11747191_SLC52A1	-0.08424	0.270092	0.378494
11751233_FIP1L1	-0.04594	0.270095	0.378494
11736778_TCL6	-0.09904	0.270105	0.378497
11749758_CYP4F2 //	0.074576	0.270172	0.378581
11736630_BTBD17	0.075407	0.270209	0.378621
11731160_ITGA2B	-0.0644	0.270217	0.378622
11760546_POLM	0.041751	0.270343	0.378788
11731162_VIPR2	0.047053	0.270385	0.378835
11759012_NCKAP1L	-0.04008	0.270419	0.378872

11733554_PAX7	-0.08091	0.270533	0.37902
11762065_TBXAS1	-0.13838	0.27057	0.379061
11764245_GPALPP1	0.078319	0.270622	0.379124
11750389_SLC26A7	0.084366	0.270666	0.379156
11757815_METTL7A	0.066898	0.270669	0.379156
11751708_FSCN3	-0.06299	0.270674	0.379156
11723999_NRAP	-0.042	0.270676	0.379156
11758305_OXCT2 ///	0.050607	0.270714	0.379198
11750749_BCL6	-0.04562	0.270734	0.379214
11735229_ZNF169	-0.04955	0.270741	0.379214
11752955_CEACAM22P	-0.10446	0.270799	0.379284
11740828_HIST1H2BL	-0.0805	0.270806	0.379284
11716382_CCT3	0.04454	0.270888	0.379378
11716328_GJA1	0.055293	0.270889	0.379378
11747536_SARDH	0.055599	0.270906	0.379392
11721939_ADORA2A /	-0.05777	0.270975	0.379476
11750948_MYCN	-0.05295	0.270991	0.379488
11746624_CPS1-IT1	-0.05014	0.271025	0.379526
11725387_HOMER1	0.044046	0.271037	0.379531
11738418_CES5A	-0.07481	0.271063	0.379556
11753620_TAC3	-0.07553	0.271073	0.379559
11746576_PPP4R3B	0.05236	0.271094	0.379577
11724736_PDPN	-0.05281	0.271104	0.379577
11729925_TET3	0.082507	0.271109	0.379577
11735521_LOC102723	-0.04339	0.271166	0.379647
11763571_LOC105379	-0.10676	0.271195	0.379676
11724880_ZNF217	-0.0733	0.27123	0.379715
11742351_OR1L8	-0.10371	0.271268	0.379757
11742636_INS-IGF2	-0.04967	0.271288	0.379773
11737977_APBB1	0.040231	0.271297	0.379775
11762663_SEL1L	-0.09339	0.271313	0.379787
11733965_SLC45A2	-0.11028	0.271348	0.379825
11754079_NDUFA5	-0.05276	0.271379	0.379857
11724363_FOXP4	-0.08105	0.2714	0.379874
11723346_LYL1	-0.04757	0.271406	0.379874
11753795_C1RL-AS1	-0.11561	0.271449	0.379923
11733358_PDE1A	-0.09843	0.271471	0.379942
11730692_TRIM7	-0.06338	0.271494	0.379964
11738790_DEFB110	-0.13114	0.27152	0.379989
11747144_BRF1	-0.09796	0.271572	0.380052
11739916_SERPINB11	-0.10589	0.271601	0.380079
11752234_NID1	0.042966	0.271613	0.380079
11738521_SLC14A2	-0.11157	0.271615	0.380079
11741305_ 8-Mar	0.086717	0.27175	0.380247
11734523_PPHLN1	-0.05861	0.271751	0.380247
11740068_CCDC8	0.09668	0.271777	0.380266
11735318_CACNB4	-0.11832	0.271784	0.380266
11727972_CENPN	-0.0459	0.271787	0.380266
11721723_GYLTL1B	0.107662	0.271807	0.380282
11748831_RFPL2	0.084534	0.272021	0.380571
11723731_GNPNAT1	0.05706	0.272118	0.380691

11725066_CAMK2B	0.080326	0.272122	0.380691
11723175_PLEKHA6	-0.07988	0.272156	0.380727
11746255_VPS29	0.040629	0.272195	0.380771
11762300_SLC35A1	0.116227	0.272244	0.380829
11739332_SSTR2	-0.15485	0.272285	0.380875
11728786_BRF1	-0.04528	0.272331	0.380924
11757246_MT1IP	0.04554	0.272336	0.380924
11756418_MEAF6	0.046869	0.272354	0.380939
11742015_PLET1	0.05321	0.272391	0.380979
11751135_CD86	-0.0662	0.272406	0.380989
11741355_THRB	0.070467	0.272491	0.381098
11749428_CCDC125	-0.07737	0.272512	0.381111
11725558_FBXL18	-0.04107	0.272516	0.381111
11717740_FUCA2	-0.06394	0.272581	0.38119
11745801_CAP1	0.039039	0.2726	0.381207
11746355_RAD9B	-0.07065	0.272613	0.38121
11735899_C12orf71	-0.12164	0.272618	0.38121
11744890_GTPBP4	-0.04433	0.272688	0.381293
11727581_CRYGC	-0.04068	0.272693	0.381293
11756900_CR2	-0.08138	0.272707	0.381301
200022_PMRPL18	0.043143	0.272742	0.381335
11754502_C21orf59	-0.03914	0.272747	0.381335
11734816_GSG1	-0.10954	0.272754	0.381335
11752210_GGCX	-0.08742	0.272772	0.381348
11750546_CHAT	-0.04693	0.27282	0.381404
11763769_MAN2A2	-0.06048	0.272875	0.38147
11737698_C6orf223	-0.05192	0.272892	0.381483
11726108_HOMEZ	0.077108	0.272905	0.381491
11738814_NFATC2	-0.08044	0.272934	0.381521
11745413_ICA1L	-0.07614	0.272958	0.381542
11737180_SLC25A21	-0.05502	0.272965	0.381542
11763928_FAM24B	0.139755	0.272993	0.38157
11745087_ABCC13	0.069132	0.273059	0.381652
11744756_LINC01561	-0.1264	0.273137	0.381749
11736922_PCSK2	0.050914	0.273275	0.381931
11736433_FBXO44	-0.05172	0.273294	0.381948
11762686_LOC100286	-0.08975	0.27333	0.381987
11735376_MSTN	-0.06918	0.273379	0.382044
11728893_TPPP	-0.07021	0.273388	0.382045
11715947_KCTD12	-0.14504	0.273527	0.382229
11736791_UNKL	-0.05157	0.273549	0.382249
11753486_PPP1R3A	0.066987	0.273562	0.382256
11723767_GFER	-0.07083	0.273582	0.382273
11715315_RABGEF1	0.091696	0.273609	0.382301
11748442_SART1	-0.07732	0.273636	0.382325
11755971_SEPT12	-0.09529	0.273642	0.382325
11759991_CFDP1	-0.07113	0.273654	0.38233
11740551_ASPG	0.058713	0.273697	0.382379
11752989_BCR	-0.11113	0.273741	0.38243
11761240_LSM14B	-0.11088	0.273813	0.38252
11743710_RGS22	-0.06628	0.273861	0.382576

11732900_LHFPL1	-0.09141	0.273879	0.38259
11738183_NLRP3	-0.05622	0.273936	0.382659
11723076_BCL9L	0.049501	0.273973	0.38268
11716795_IFI16	-0.0489	0.273974	0.38268
11722864_NETO2	-0.05241	0.273975	0.38268
11759554_PAPOLA	-0.08362	0.274003	0.382709
11740600_TRHR	0.119337	0.274086	0.382814
11741420_GPR75	0.051338	0.274284	0.383079
11754695_PLA2G5	-0.06705	0.2743	0.383083
11749132_SLCO2B1	0.04549	0.274303	0.383083
11735433_FER1L6	-0.08397	0.274336	0.383118
11763633_SLC12A4	-0.08144	0.274374	0.383156
11728064_GPER1	-0.07372	0.274379	0.383156
11718611_TP53INP1	0.057965	0.274386	0.383156
11731280_IGFN1	-0.09868	0.274401	0.383166
11723563_VWA5A	-0.0558	0.274462	0.38323
11726427_GABBR2	0.099319	0.274463	0.38323
11725088_MYH1	0.089564	0.274481	0.383245
11759404_TSPAN5	0.072335	0.274585	0.383374
11721576_C1orf131	-0.03989	0.274595	0.383374
11763657_DPP6	0.098904	0.2746	0.383374
11748078_SLC43A2	-0.06645	0.274622	0.383374
11756154_HRG	-0.13512	0.274625	0.383374
11753895_MPST	-0.06057	0.274626	0.383374
11747897_NUPL2	0.056627	0.274629	0.383374
11758510_PLAC4	0.068256	0.274735	0.383512
11735664_SFTPA2	0.055345	0.274772	0.38355
11736163_CDKN2B	0.112471	0.274778	0.38355
11729826_ZSCAN2	0.0517	0.274805	0.383576
11728553_MMP25	-0.05379	0.274814	0.383577
11741717_SOX6	0.065373	0.274821	0.383577
11755023_RER1	-0.04285	0.274856	0.383615
11760456_SRCIN1	-0.07748	0.2749	0.383665
11736579_GH2	-0.10374	0.274928	0.383694
11725778_PRDM2	-0.06164	0.274977	0.38375
11748070_WWP2	0.046122	0.274992	0.38375
11752425_URI1	0.051811	0.274992	0.38375
11764071_GCFC2	-0.04273	0.275027	0.383787
11750658_KANSL3	-0.04907	0.275146	0.383943
11720408_PPP6R2	-0.05984	0.275162	0.383955
11723127_NOTUM	-0.08779	0.275206	0.384003
11751849_PSORS1C1	0.076919	0.275213	0.384003
11759870_CCL4L1 //	-0.17647	0.275263	0.384048
11740993_PHAX	0.046181	0.275267	0.384048
11741782_TSPY1 ///	-0.08515	0.275269	0.384048
11743185_HLA-G	-0.06374	0.275309	0.384088
11752376_GHR	0.070383	0.275313	0.384088
11760385_MYEF2	-0.10236	0.275349	0.384128
11731267_NXPH2	0.135538	0.275397	0.384184
11746740_CTTN	-0.0485	0.275575	0.38442
11728171_PRDM10	-0.07557	0.275628	0.384475

11729557_EYA2	-0.05548	0.275634	0.384475
11754719_RAI2	-0.11379	0.275638	0.384475
11748811_AP2A1	-0.05711	0.275682	0.384516
11738566_ASTL	-0.05278	0.275682	0.384516
11752206_OTOF	-0.05537	0.275793	0.384658
11728054_DIAPH3	-0.06406	0.27582	0.384686
11761269_TMC6	-0.07892	0.275918	0.384811
11732259_SH2D4A	0.046389	0.275974	0.384878
11759210_AK9	-0.08552	0.276022	0.384934
11747951_RIPK3	-0.07537	0.276032	0.384938
11732162_ZCRB1	-0.0784	0.276076	0.384988
11733715_EXOC4	-0.04807	0.276131	0.385054
11726918_SRD5A2	0.119774	0.276149	0.385067
11738087_ZNF619	-0.0651	0.276167	0.385075
11736416_CCDC82	0.048079	0.27617	0.385075
11725646_CACNG3	-0.05217	0.276282	0.38522
11733677_C4orf50	-0.10024	0.276291	0.385221
11727368_MED29	-0.05076	0.276419	0.385389
11722270_SASH3	-0.08301	0.27643	0.385394
11751623_AP4M1	0.054373	0.276453	0.385404
11751631_AP4M1	0.054373	0.276453	0.385404
11745952_E4F1	-0.04686	0.276484	0.385436
11756061_RPS2	-0.03832	0.276518	0.385472
11726095_RBMXL1	-0.0546	0.27653	0.385478
11759143_TAF9B	-0.05117	0.276562	0.385502
11734880_TTLL2	-0.08743	0.276563	0.385502
11750077_SNAP25	0.118769	0.276571	0.385502
11742151_OR2AG2	-0.06126	0.276612	0.385548
11735666_C1orf100	-0.16476	0.276628	0.385555
11740295_ZNF30	0.045026	0.276633	0.385555
11737319_ZNF546	-0.05058	0.276741	0.385695
11726447_KCNK3	-0.0626	0.276764	0.385716
11726474_HES4	-0.06447	0.276779	0.385726
11741062_LOC101928	0.069415	0.276857	0.385824
11762931_ZMIZ1	0.131415	0.2769	0.385872
11755492_SUCLA2	0.047424	0.27691	0.385875
11744439_SRI	-0.0392	0.276924	0.385884
11716637_EMC7	0.044092	0.276943	0.385899
11761645_CASC5	-0.13287	0.277007	0.385978
11742590_OR2M5	0.077213	0.27703	0.385999
11742392_OR1L4 ///	-0.07774	0.277067	0.38604
11726509_SMPD3	0.056546	0.277078	0.386043
11745821_PLAGL1	-0.08781	0.277094	0.386055
11744124_SCYL1	-0.04231	0.277133	0.386084
11737436_TXNRD3 //	0.071162	0.277138	0.386084
11717832_MIIP	-0.04891	0.277139	0.386084
11726337_ALOX5	0.077087	0.277158	0.3861
11723088_GAS2L1	-0.06062	0.277182	0.386122
11724734_ABCB8	0.092163	0.277191	0.386123
11758759_GSTK1	0.074376	0.277224	0.386159
11741614_KLC2	0.053039	0.277263	0.386202

11729106_HPCAL4	-0.06104	0.277312	0.386259
11760394_SRGAP2	-0.07569	0.277324	0.386265
11719578_AMT	-0.06264	0.277357	0.3863
11752029_SGSM2	-0.06257	0.277426	0.38638
11758754_FAF1	-0.04767	0.27743	0.38638
11723369_SHANK3	-0.10916	0.277452	0.3864
11741728_NFATC3	0.062438	0.277519	0.386482
11715170_HNRNPCL1	-0.08648	0.277581	0.386558
11716018_RAB31	0.038586	0.277595	0.386565
11741233_CASC1	-0.07342	0.277615	0.386577
11718102_CD59	-0.08886	0.277619	0.386577
11738409_KRTAP3-2	-0.07155	0.277703	0.386682
11750629_C1QTNF8	-0.05098	0.277765	0.386758
11730997_LRRN1	-0.0505	0.277794	0.386787
11720576_ARFGEF1	0.043495	0.277828	0.386824
11735416_DCLRE1C	-0.04791	0.277854	0.386849
11758543_ARMCX6	-0.04342	0.277881	0.386869
11755879_C1QTNF5 /	-0.04976	0.277884	0.386869
11741981_CLEC7A	0.088698	0.277913	0.386898
11740413_VCX /// V	-0.08376	0.277922	0.386899
11749536_SLC6A9	-0.10411	0.277934	0.386905
11730568_PYDC1	0.065604	0.27795	0.386916
11760631_GIT2	-0.08635	0.277972	0.386929
11750952_CHRND	0.049571	0.277975	0.386929
11730728_UBA3	0.039373	0.278021	0.386983
11732891_CHRNA10	-0.05934	0.278079	0.387051
11755849_BAZ2A	-0.04103	0.27814	0.387126
11715140_PCDHA13	-0.11131	0.27817	0.387157
11735002_ST7L	0.146764	0.278245	0.38725
11721714_ATRX	0.053824	0.278295	0.387299
11761232_TRMU	-0.0899	0.278296	0.387299
11746047_KGFLP2	-0.08317	0.278335	0.387342
11740946_FAM153A /	-0.11122	0.278453	0.387495
11739014_UBAC2	-0.04251	0.278519	0.387576
11734298_ETV3	-0.05831	0.278553	0.387613
11732847_CABP7	-0.07522	0.278569	0.387623
11752473_MED24	-0.05808	0.278576	0.387623
11721673_GLRX5	0.046587	0.278597	0.387629
11759963_EFCAB13	-0.10346	0.278597	0.387629
11754900_SAMD14	0.058302	0.278659	0.387705
11735571_SORBS2	-0.044	0.278712	0.387767
11734276_HNF1A	-0.0927	0.278898	0.388015
11756242_PSMF1	-0.04356	0.278921	0.388036
11752353_NFATC4	-0.05737	0.278954	0.388071
11761510_KIN	0.071245	0.278975	0.38809
11727558_TK2	0.078698	0.278992	0.388101
11740958_CEP63	-0.05019	0.279036	0.388152
11748734_TBC1D25	-0.0596	0.279097	0.388226
11763775_LOC100130	-0.15511	0.279141	0.388272
11726917_KCNH3	-0.06306	0.279146	0.388272
11734434_TUBGCP5	-0.05096	0.279201	0.388337

11735685_LCE1E	-0.05413	0.279273	0.388426
11751409_RAB36	-0.07841	0.279283	0.388429
11722932_CHML	-0.05325	0.279325	0.388477
11764285_LOC102723	-0.10574	0.279365	0.38852
11757644_PASD1	0.091824	0.279382	0.388521
11730711_TEX13A	-0.09713	0.279384	0.388521
11738436_IFNA4	-0.06589	0.279388	0.388521
11764056_FMR1	0.078589	0.279398	0.388523
11739167_GIMAP6	-0.08561	0.279427	0.388541
11728495_GNRH1	-0.11169	0.279427	0.388541
11734171_PRTN3	-0.05202	0.279445	0.388556
11734048_PIH1D1	-0.03975	0.279503	0.388614
11756539_TCTEX1D1	-0.04511	0.279503	0.388614
11715157_KRTAP19-2	-0.09692	0.279525	0.38863
11758833_COL6A2	-0.12145	0.279531	0.38863
11736099_CCDC59	-0.04457	0.279611	0.38873
11731126_GXYLT2	-0.05223	0.279631	0.388746
11749569_CAPRIN1	0.039183	0.279638	0.388746
11727605_TMEM92	0.065534	0.27974	0.388876
11763493_KITLG	0.067383	0.279754	0.388885
11746544_IPO8	0.049277	0.27981	0.38895
11752639_PRICKLE3	-0.05095	0.279816	0.38895
11725530_DSC2	0.109722	0.279863	0.389003
11747791_TANGO2	-0.07165	0.279898	0.389033
11760040_NDUFB4	-0.07132	0.2799	0.389033
11731660_VWA3B	-0.11457	0.279914	0.38904
11760194_LRRIQ3	-0.15521	0.279973	0.389112
11754078_RAB11B	-0.07745	0.280009	0.389148
11745500_CLEC10A	-0.07452	0.280015	0.389148
11735922_AMELY	-0.04142	0.280162	0.389339
11756992_MARC2	-0.12257	0.280168	0.389339
11743944_ACSM2B	-0.06374	0.280196	0.389367
11719181_CLCN7	-0.05926	0.280215	0.389381
11741145_LMO3	-0.06348	0.280226	0.389386
11726412_ZBTB7C	0.043478	0.280235	0.389388
11724152_TNS4	-0.06287	0.280262	0.389415
11742598_OR8H3	-0.06567	0.280286	0.389434
11732316_SGCD	0.091158	0.280293	0.389434
11749712_PDE1B	-0.09749	0.28036	0.389516
11747177_AQP1	-0.09728	0.280388	0.389545
11732897_SLAMF9	-0.06267	0.280428	0.38959
11734426_CDH23	-0.07241	0.280449	0.389607
11738966_KRTAP25-1	0.047801	0.280468	0.389622
11760585_EMSY	0.088948	0.280519	0.389674
11759339_NTRK2	-0.07142	0.280521	0.389674
11753835_ECM1	-0.06757	0.28057	0.38973
11728424_PILRA	0.110245	0.280579	0.389732
11758729_MT2A	-0.05563	0.280618	0.389775
11721431_SYBU	0.058837	0.280664	0.389829
11752802_SP110	0.083187	0.280754	0.389937
11734661_CLSTN3	-0.04785	0.280758	0.389937

11726346_LAT2	0.043326	0.280792	0.38997
11736651_CD5L	-0.10237	0.280798	0.38997
11723324_HOXC10	0.067642	0.280811	0.389977
11740622_ADGRF4	-0.03916	0.280882	0.390064
11728677_CES2	0.043502	0.280902	0.390081
11751380_BEST1	-0.06509	0.280943	0.390128
11720448_SOX9	0.060113	0.280958	0.390137
11740742_ZNF645	0.067202	0.280967	0.390138
11745518_ZNF365	0.053999	0.280984	0.390151
11744821_CDH23	-0.0649	0.280995	0.390156
11754849_PAX6	-0.08631	0.281019	0.390178
11755188_BRD9	-0.05063	0.281142	0.390328
11754173_SNX3	-0.04402	0.281144	0.390328
11732781_NME8	-0.06442	0.281154	0.390332
11751781_APOL4	-0.08351	0.281218	0.390409
11723665_PLPP1	0.05956	0.281264	0.390457
11737780_B3GALNT1	0.082759	0.281268	0.390457
11723294_SP3	-0.06744	0.281289	0.390475
11721618_LRRC75B	-0.04705	0.28135	0.390547
11739511_SNED1	-0.06304	0.281362	0.390547
11741432_ZDHHC3	-0.06529	0.281365	0.390547
11755399_COL18A1	0.062009	0.281378	0.390554
11751757_LMO3	-0.06906	0.281477	0.39068
11756798_ZSCAN23	0.098952	0.2815	0.390691
11726284_IGHMBP2	-0.04078	0.281501	0.390691
11745371_LAMB4	0.080991	0.281528	0.390718
11736412_NHLH2	-0.09803	0.281539	0.390721
11750106_ANKAR	-0.10576	0.281547	0.390722
11749378_CLCF1	0.038822	0.281594	0.390775
11761400_NIFK	0.07359	0.281631	0.390816
11742347_FGF8	-0.05892	0.281642	0.39082
11726061_LRRC4	-0.13717	0.281654	0.390826
11721621_KATNAL1	0.051645	0.281671	0.390839
11733978_CD28	-0.12237	0.28172	0.390896
11752347_OS9	0.065144	0.281767	0.390949
11761356_PQBP1	0.064024	0.281789	0.390969
11736892_ZSCAN20	0.089846	0.281835	0.391022
11715829_PLS3	-0.03817	0.281935	0.391149
11738466_CLRN1	-0.08285	0.281944	0.391151
11762653_SARDH	-0.08141	0.281953	0.391152
11748896_ACKR4	-0.0956	0.282012	0.391222
11722567_NT5C2	-0.05234	0.282023	0.391226
11727161_NFIX	-0.05205	0.282062	0.39127
11761794_FAM122C	-0.04988	0.282123	0.391343
11739779_HFE2	-0.11889	0.282133	0.391346
11747671_CRIP2	-0.04884	0.282169	0.391385
11725391_CD6	-0.04899	0.282271	0.391494
11733168_TSSK3	-0.08108	0.282271	0.391494
11740873_MS4A7	0.044041	0.282272	0.391494
200083_PMUSP22	-0.04324	0.282532	0.391844
11752115_FMR1-AS1	-0.10866	0.282554	0.391864

11761403_ISCU	0.047689	0.282593	0.391906
11763966_TRIM71	-0.07163	0.282683	0.392002
11726219_DMTN	-0.04595	0.282684	0.392002
11718493_SLC1A2	-0.05943	0.282686	0.392002
11759894_GCLC	0.070691	0.282714	0.392021
11752982_CA9	-0.10007	0.282723	0.392021
11749631_APOB	-0.08644	0.282724	0.392021
11732635_HHIPL2	0.064832	0.282823	0.392137
11729293_SPPL2B	-0.04375	0.282824	0.392137
11721872_FAM110A	0.057192	0.282842	0.392152
11764215_WT1-AS	-0.09101	0.282938	0.392273
11736113_ARHGAP18	0.042717	0.282956	0.392287
11719523_TOR1B	-0.04959	0.283085	0.392455
11751478_TSC22D4	0.055041	0.283111	0.39248
11720248_FBXL15	-0.06919	0.283126	0.392489
11751420_ZCWPW1	-0.06521	0.283249	0.392649
11752856_RNF17	0.084315	0.283278	0.392678
11740556_CCDC141	-0.04722	0.283318	0.392723
11743391_VPS35	-0.04188	0.283418	0.39285
11752380_CEP83	-0.07224	0.283447	0.392878
11747538_MYD88	0.069916	0.2835	0.392942
11718628_BOK	-0.05946	0.283512	0.392947
11748137_SEC31B	-0.04828	0.283528	0.392957
11719586_TAGLN3	-0.04754	0.283557	0.392986
11745734_CAMKV	-0.07449	0.283576	0.393002
11744477_STK32C	-0.08954	0.283592	0.393009
11736625_BAAT	-0.06182	0.283597	0.393009
11730597_NR1I3	-0.04667	0.283621	0.393028
11757329_RPL26	0.045504	0.283627	0.393028
11717517_REXO2	0.042065	0.283635	0.393028
11734382_AQP10	-0.07313	0.283645	0.39303
11746307_LINC00494	0.118722	0.283675	0.393058
11727249_ZSCAN22	-0.08611	0.283681	0.393058
11729091_ZDHHC19	0.054224	0.283692	0.393063
11730713_KRT73	-0.05445	0.283728	0.393101
11732064_C2orf82	-0.05553	0.283761	0.393136
11731724_B4GALT7	0.071091	0.283782	0.393154
11720088_NUCB2	-0.05512	0.283803	0.39317
11733712_LMX1A	-0.10358	0.28381	0.39317
11762181_GTF2H4	-0.11214	0.283825	0.393179
11738548_KCTD17	-0.04893	0.28386	0.393217
11754597_DUS3L	-0.04378	0.283878	0.39323
11747750_CLCNKB	-0.04269	0.28389	0.393236
11758062_STK32B	0.039988	0.283933	0.393285
11756793_VNN2	0.084763	0.283948	0.393294
11753428_TATDN1	-0.05599	0.28396	0.3933
11757140_RPLP2 ///	-0.06122	0.283978	0.393314
11761961_MT1DP	-0.10956	0.284071	0.393431
11744558_VASH1	-0.09531	0.284082	0.393433
11722680_HCST	-0.04966	0.284088	0.393433
11724863_MEF2A	0.042402	0.284205	0.393583

11760899_UBXN1	-0.10738	0.2843	0.393703
11736711_TMIE	-0.07605	0.284314	0.393712
11736632_APOBEC3H	0.095208	0.284325	0.393716
11763590_ZNF213	0.042713	0.284346	0.393723
11763506_N4BP3	-0.11831	0.284346	0.393723
11722643_BRPF1	0.058334	0.28439	0.393773
11748817_SLC9A9	0.082537	0.284432	0.39382
11733805_AMN	-0.06284	0.284493	0.393893
11753634_MED24	-0.06861	0.284546	0.393956
11757673_RPL39	0.038242	0.284564	0.393969
11716134_MTOR	-0.06912	0.284582	0.393982
11762106_LOC100289	-0.07663	0.284649	0.394064
11750156_GABRG2	-0.09956	0.284664	0.394074
11763309_TCF4	-0.09863	0.284674	0.394077
11748036_ATP2A3	0.048128	0.28475	0.394171
11715232_RPL36	0.048822	0.284796	0.394223
11735371_GSG1L	0.064845	0.284921	0.394385
11742000_OR5K1	-0.04115	0.284943	0.394393
11744247_TSHZ2	-0.06701	0.284943	0.394393
11731808_CACNA1S	-0.07419	0.285085	0.394579
11729401_ZNF285	-0.08742	0.285145	0.39465
11756602_SYT4	-0.05509	0.285158	0.39465
11737036_TCEAL6	-0.11447	0.285161	0.39465
11763018_HSPD1	0.093686	0.285248	0.394759
11747738_USP39	-0.04467	0.285278	0.394789
11725860_APBA1 ///	0.11087	0.2853	0.394801
11757881_PRKACA	-0.04237	0.285302	0.394801
11743308_COPS4	0.039996	0.285359	0.394868
11740757_SIM2	-0.0504	0.285417	0.394933
11739637_CDK12	0.066474	0.285422	0.394933
11759158_ADAM7	-0.12227	0.285454	0.394967
11753728_MED25	-0.045	0.285516	0.395041
11737522_PDZD3	0.112389	0.285569	0.395103
11715114_FAM86C1	0.044353	0.285591	0.395122
11762440_LRRTM1	-0.09599	0.285704	0.395253
11757959_GOT2	-0.04564	0.285709	0.395253
11718017_ADARB1	-0.06519	0.28572	0.395253
11757000_STYXL1	-0.05875	0.285726	0.395253
11746386_SPIDR	-0.04465	0.285726	0.395253
11743375_FRYL	0.046229	0.285755	0.39528
11762896_LAS1L	-0.10283	0.285762	0.39528
11751566_FADS6	-0.06631	0.285786	0.395302
11762829_LOC100506	-0.07514	0.285804	0.395313
11760171_UQCC2	-0.05089	0.28581	0.395313
11734310_CNN1	-0.07193	0.285819	0.395315
11742675_LCE3B	-0.05033	0.285834	0.395315
11743884_ADPRHL1	-0.0469	0.285835	0.395315
11740812_VAMP1	-0.05663	0.285851	0.395325
11733250_ARHGAP31	0.094944	0.285871	0.395342
11746905_RGL2	0.042617	0.285918	0.395387
11731090_CACHD1	0.084357	0.28592	0.395387

11737781_ARMCX2	0.054861	0.285975	0.395451
11727949_DHX33	0.042235	0.286061	0.395559
11748710_GPATCH8	0.082949	0.286092	0.395592
11718573_SERBP1	-0.04561	0.286111	0.395597
11738074_PDE4A	-0.0475	0.286112	0.395597
11741125_HDGFL1	-0.09811	0.286147	0.395634
11760004_LOC100508	0.049417	0.28622	0.395724
11741510_HERPUD1	-0.04233	0.286242	0.395743
11718451_AXIN2	0.116756	0.286303	0.395809
11758292_PTGIR	-0.06659	0.286308	0.395809
11730562_GRIA4	0.102018	0.286314	0.395809
11751845_SERPING1	-0.148	0.286334	0.395819
11747216_LOC100130	-0.06072	0.286338	0.395819
11755117_FBXL22	0.071931	0.286529	0.396072
11725741_SMPDL3B	-0.06856	0.286541	0.396077
11734452_LEPR	-0.08837	0.286599	0.396147
11746617_CNOT6	-0.06698	0.286663	0.396223
11748812_SLC30A8	-0.06821	0.286685	0.396244
11736203_PLEKHB1	-0.06409	0.286694	0.396245
11762316_GLYATL1	-0.04869	0.286791	0.396364
11750150_ZNF815P /	-0.08424	0.286797	0.396364
11740720_IKZF3	-0.11532	0.286826	0.396394
11752590_CCR2	-0.1019	0.28688	0.396457
11723462_PHKB	0.08153	0.286933	0.396519
11716571_RPA1	-0.06637	0.286957	0.396536
11740868_LDLRAD1	-0.09083	0.286962	0.396536
11747068_CCT5	-0.03769	0.286978	0.396548
11759146_PPP2R2B	-0.0821	0.287009	0.396579
11734511_ASCL4	0.069619	0.287027	0.396593
11759727_C14orf159	0.065714	0.287108	0.396684
11735876_PCDHGA4	-0.07469	0.28711	0.396684
11748163_WDR81	-0.04481	0.287128	0.396699
11738679_NCR1	-0.08074	0.28729	0.396911
11748257_NPRL3	-0.06459	0.287315	0.396935
11743982_SH3GLB2	-0.05232	0.287344	0.396963
11720265_SLC8B1	-0.05159	0.287396	0.397013
11743171_RCSD1	0.076362	0.287396	0.397013
11762202_RARA	0.065058	0.287419	0.397033
11729725_ZBTB6	0.047922	0.287434	0.397043
11753020_CACNB1	-0.07154	0.287448	0.397051
11752623_PXK	-0.05932	0.287466	0.397064
11749854_MOG	-0.06078	0.287486	0.397081
11751361_CASQ2	0.103865	0.287521	0.397117
11732633_HOXA7	-0.1381	0.28753	0.397119
11762924_GUSBP1	0.160508	0.287539	0.39712
11752589_CH17-360D	0.060517	0.287572	0.397154
11727935_MGARP	0.044866	0.287631	0.397225
11734837_ZNF710	-0.05181	0.287656	0.397248
11725507_SPTBN2	-0.05537	0.287697	0.397293
11732975_OR1A2	-0.08731	0.287738	0.397339
11760936_CMTM5	0.061246	0.28776	0.397358

11738942_KRTAP27-1	-0.13945	0.287777	0.39737
11731425_CXCR2	0.047469	0.287869	0.397478
11727206_ZMAT4	-0.06998	0.287876	0.397478
11732528_TBX5	-0.11133	0.287889	0.397478
11759873_FAM175A	0.059946	0.287894	0.397478
11761906_MGAT4C	-0.09789	0.287896	0.397478
11761493_C14orf159	-0.08215	0.287918	0.397497
11762464_TARSL2	-0.07255	0.288075	0.397704
11734143_METTL8	-0.08699	0.288198	0.397862
11744498_TRO	0.061952	0.288257	0.397932
11726048_ASXL2	0.061433	0.288275	0.397945
11760056_VCAN	0.12203	0.2883	0.397968
11757060_RPL10	0.037233	0.28835	0.398027
11736032_VAMP1	-0.06754	0.288401	0.398085
11733427_GRIN1	-0.05363	0.288431	0.398116
11728919_LRRC73	0.069049	0.288473	0.398162
11742335_OR2D2	-0.09133	0.288543	0.398245
11748144_MED15	-0.05355	0.288549	0.398245
11756893_RAB11B	-0.04059	0.28858	0.398277
11739342_TNFRSF10C	-0.08471	0.288593	0.398283
11739658_LTB	-0.04933	0.288618	0.398307
11744008_LZTS3	-0.04402	0.288668	0.398356
11749648_HIF1A	-0.04261	0.28867	0.398356
11753326_GPR157	0.049396	0.288719	0.398412
11724415_DISP1	-0.05558	0.288815	0.398525
11733039_DPH3	-0.11851	0.288817	0.398525
11722979_F8	-0.04633	0.288833	0.398536
11738303_TMC3	-0.05298	0.288861	0.398556
11734746_CDKN2AIPN	-0.06584	0.288864	0.398556
11718993_CRKL	0.068106	0.288921	0.398624
11746155_IRF9	-0.04803	0.288996	0.398715
11761720_HPS1	0.047333	0.28912	0.398875
11736063_SEPT7	0.039565	0.289137	0.398887
11748479_RAPGEF1	-0.0479	0.289265	0.399053
11718658_CD34	-0.0809	0.289416	0.39925
11729708_CSMD1	-0.08917	0.289557	0.399434
11754128_BRMS1	-0.04581	0.289605	0.399488
11743989_VARS	0.041393	0.28962	0.399497
11728107_SLC23A3	-0.05042	0.289634	0.399506
11736150_LYRM2	0.096914	0.289646	0.399511
11752944_WDR27	0.042218	0.289665	0.399525
11750531_PYCARD	0.059994	0.289711	0.399579
11760757_SLC18B1	-0.05872	0.289728	0.39959
11731745_ARHGEF16	-0.07174	0.289767	0.399632
11716036_BST2	-0.06043	0.289906	0.399797
11742184_LAMA3	-0.07393	0.289909	0.399797
11744762_FAM53A	-0.06613	0.28991	0.399797
11753606_CDC42	-0.04097	0.289928	0.39981
11749959_TK2	-0.07178	0.289979	0.399868
11762918_CCDC159	0.039126	0.290007	0.399895
11735964_GNG8	-0.06847	0.29002	0.399902

11753335_TMEM177	0.054647	0.290082	0.399977
11731540_SESTD1	-0.08985	0.290125	0.400024
11723120_DSTYK	0.064459	0.290151	0.400047
11747884_TAF1C	-0.09928	0.290158	0.400047
11745346_TMEM266	0.06221	0.290263	0.400173
11762116_DDI2	-0.10359	0.290265	0.400173
11752560_SUSD4	-0.06275	0.290286	0.40019
11736716_TMPRSS11E	0.061695	0.290331	0.40024
11764065_LOC105375	-0.08549	0.290346	0.40024
11736494_TNRC6B	0.050882	0.290347	0.40024
11737548_PCDHAC1	-0.05271	0.290387	0.400285
11751934_PNPLA6	0.03987	0.290401	0.400293
11728124_BRICD5	-0.05512	0.29041	0.400294
11749575_LOC100287	-0.07938	0.29051	0.40042
11759315_RIMS3	-0.07881	0.29052	0.400423
11749185_RHBG	-0.09734	0.290539	0.400437
11740886_SMARCA4	0.041737	0.290559	0.400444
11730398_RUVBL2	-0.04565	0.29056	0.400444
11739682_PHF2	-0.03964	0.290591	0.400475
11758185_TWF1	0.03807	0.290719	0.40064
11763284_RPS28	0.05393	0.290772	0.400702
11740418_PLA2R1	0.102302	0.290792	0.400718
11731642_LOC102724	0.067405	0.290839	0.400772
11737242_TMEM233	-0.08054	0.290908	0.400855
11744701_DRD3	-0.11848	0.291003	0.400975
11749980_HOPX	0.099262	0.291023	0.400991
11744727_GPR137	0.047821	0.29104	0.401004
11747145_BRF1	-0.11088	0.291083	0.401051
11755131_PRR26	0.108818	0.291148	0.40113
11725966_ITGB7	-0.08421	0.291183	0.401166
11722227_DNAJC30	-0.05102	0.291234	0.401222
11741808_LTK	-0.04021	0.29124	0.401222
11747535_UQCC1	0.042756	0.29127	0.401253
11750499_ZNF705A /	0.067914	0.291302	0.401285
11751675_AK2	0.044041	0.291313	0.40129
11759188_ADCYAP1R1	-0.06347	0.291328	0.401299
11750136_ARHGAP36	-0.10638	0.2914	0.401387
11729176_KLHL4	0.103264	0.291517	0.401537
11737085_ZNF490	0.056576	0.291534	0.401548
11762836_ING5	-0.0382	0.291581	0.401602
11716763_NSMF	0.040179	0.291593	0.401604
11737148_CLEC7A	0.113375	0.291609	0.401604
11732915_KCNT2	-0.09411	0.291618	0.401604
11750991_UBQLN4	0.083975	0.291622	0.401604
11757350_FGA	0.099366	0.291623	0.401604
11716520_C10orf10	0.123887	0.291693	0.401688
11760625_SLC35B3	-0.05569	0.291787	0.401807
11735785_NPY2R	-0.07913	0.291797	0.401807
11724580_ZNF827	0.067372	0.291815	0.401807
11762595_FBN2	0.072184	0.291819	0.401807
11751160_LPAR4	-0.10443	0.29182	0.401807

11759173_KRTAP20-1	0.095062	0.291846	0.401831
11762481_LAMB4	0.041376	0.2919	0.401895
11724844_ABHD17A	0.046081	0.291915	0.401895
11755340_SPSB3	-0.04813	0.291924	0.401895
11744513_WFDC2	-0.07141	0.291925	0.401895
11740649_IRAK4	0.070143	0.292091	0.402111
11762079_NIT2	-0.05652	0.2921	0.402112
11740042_MTFMT	0.121057	0.292168	0.402186
11746074_OXNAD1	0.039691	0.292174	0.402186
11726866_FOXO4	-0.06853	0.292186	0.402186
11742914_GLOD5	-0.07434	0.292187	0.402186
11726226_CASR	-0.07677	0.292226	0.40223
11721542_ 9-Mar	-0.05443	0.292246	0.402246
11733412_ZC4H2	-0.06949	0.292296	0.402304
11730092_KCNK17	-0.11021	0.292354	0.402372
11751394_PIK3IP1	-0.04239	0.292385	0.402404
11746529_TNFRSF14	0.066539	0.292411	0.40242
11732970_C3orf36	-0.04925	0.292414	0.40242
11716921_CLEC11A	-0.04116	0.292495	0.402521
11733488_AFF2	-0.06133	0.292537	0.402555
11755920_SLC22A14	-0.05503	0.292537	0.402555
11738528_IL31RA	-0.08626	0.292559	0.402574
11717112_REPIN1	0.049364	0.292626	0.402647
11750721_ARHGEF15	-0.05639	0.292635	0.402647
11752769_PGBD5	-0.06571	0.292637	0.402647
11744988_HECTD4	0.062113	0.292649	0.402652
11730521_SLITRK2	-0.05439	0.292669	0.40267
11745607_FCRL2	-0.06789	0.29269	0.402683
11750954_PODNL1	-0.0839	0.292695	0.402683
11748508_NMUR1	-0.03886	0.292723	0.402709
11733132_OLFML1	0.074713	0.292732	0.402709
11728777_VDR	-0.0864	0.292739	0.402709
11749112_RB1	-0.05408	0.292766	0.402734
11752193_RGL4	-0.08062	0.292784	0.402748
11727845_HSPA14	-0.03936	0.292806	0.402767
11758733_RPL35A	-0.13162	0.292884	0.402863
11742990_EZR	0.042689	0.292915	0.402894
11751811_CCDC13	-0.04983	0.293089	0.403122
11750847_ANKRD44	0.078064	0.293155	0.403193
11753469_FLJ42393	0.095729	0.293157	0.403193
11739168_GIMAP6	-0.08589	0.293186	0.403219
11759030_MSX2	0.045101	0.293192	0.403219
11734289_DLGAP2	-0.08243	0.293235	0.403267
11733772_ASB11	-0.06719	0.293293	0.403335
11732772_NLRP11	-0.04974	0.29333	0.403374
11716555_PSMG3	-0.05162	0.293348	0.403388
11754711_C1orf123	-0.03959	0.293396	0.403442
11727955_ABCD1	-0.05579	0.293448	0.403502
11760217_PIK3CB	0.047691	0.293466	0.403516
11742397_ANP32C	0.047065	0.293501	0.403548
11735357_CXorf58	-0.05488	0.293506	0.403548

11743058_RHO	0.094036	0.293564	0.403613
11731974_SCNN1G	0.084095	0.293569	0.403613
11716998_RHBDD3	-0.05935	0.293687	0.403755
11735674_AKAP14	0.140446	0.293697	0.403755
11724322_CST6	-0.10997	0.293698	0.403755
11745660_POM121C	0.051203	0.293768	0.40384
11748860_APOBEC2	0.140054	0.293799	0.403872
11755379_SMPDL3B	-0.07139	0.293824	0.403895
11749125_FZD6	0.040565	0.293887	0.40397
11732825_SPATA19	-0.09779	0.293908	0.403987
11745304_PFN1P2	-0.09292	0.29395	0.404033
11737489_ODF3	0.048071	0.293958	0.404033
11734812_TMEM86A	-0.06739	0.29402	0.404107
11742963_ITPR3	0.075309	0.294064	0.404156
11749939_AIF1L	-0.07035	0.294113	0.404212
11752646_KCNJ5	-0.08668	0.294141	0.404239
11738314_SLC35G3	-0.06582	0.294168	0.404265
11756350_TADA2A	-0.04779	0.294268	0.404391
11737035_EBF2	0.057827	0.294286	0.404404
11750908_ESR2	-0.05629	0.294327	0.404449
11723839_FKBP1B	0.055729	0.294348	0.404466
11716473_PUM1	0.037731	0.294414	0.404546
11718892_FOXO3 ///	0.051968	0.294453	0.404582
11735490_EMX1	-0.05225	0.294457	0.404582
11736199_DCX	-0.10235	0.29447	0.404588
11740761_PRKG2	-0.06036	0.294481	0.404588
11740026_LOC200726	-0.07541	0.294486	0.404588
11747882_DEF8	-0.06346	0.294501	0.404589
11756269_ATXN7L2	0.044641	0.294511	0.404589
11752045_SH3TC2	0.053271	0.294512	0.404589
11762483_TIGD7	-0.07822	0.294546	0.404625
11721614_KIT	-0.07183	0.294567	0.404643
11726954_ADAMTS14	-0.10621	0.294618	0.404701
11736256_INPP4A	-0.06618	0.294639	0.404719
11716660_GRAMD4	-0.05297	0.294648	0.40472
11740733_SLC9C1	-0.08529	0.294657	0.404721
11733648_CCDC116	-0.0576	0.294704	0.404773
11728155_FUT4	-0.12239	0.294715	0.404773
11740058_TMPRSS11A	-0.04204	0.29472	0.404773
11715254_DEFB134 /	-0.06331	0.294787	0.40485
11730388_RYR1	-0.06845	0.294793	0.40485
11723653_MUT	-0.06231	0.29484	0.404892
11718070_NCOR2	-0.06309	0.294843	0.404892
11752826_RORC	-0.05016	0.294848	0.404892
11754152_MCF2	0.062686	0.294868	0.404908
11752515_ODC1	0.046396	0.294922	0.40497
11749101_UBAP2	0.041175	0.295049	0.405133
11751709_GNA15	-0.06001	0.295089	0.405177
11757119_RPSA ///	-0.05164	0.295098	0.405178
11761820_EDEM2	-0.13335	0.295129	0.405207
200002_PMRPL35	-0.03974	0.29514	0.405207

11725226_CHP2	-0.13231	0.295144	0.405207
11748151_LOC653712	-0.05769	0.295192	0.405262
11750410_UGT1A1 //	0.101398	0.295264	0.405339
11745544_TICAM2 //	0.052704	0.295265	0.405339
11726719_GEMIN7	0.049366	0.29538	0.405486
11761505_USB1	0.059524	0.295406	0.405508
11718335_LMOD1	0.077355	0.295413	0.405508
11721900_FCN1	0.075537	0.29546	0.40555
11717312_IKBKAP	0.131811	0.29546	0.40555
11718169_AZGP1	0.04522	0.295508	0.405605
11728136_XK	-0.07368	0.295582	0.405694
11753496_CSN1S2AP	0.050943	0.295602	0.405705
11752658_DNMT3B	0.042434	0.295606	0.405705
11748727_SIGLEC10	-0.08378	0.295633	0.405731
11750777_FAAP20	0.080125	0.295651	0.405744
11757589_NDUFA12	-0.03781	0.295709	0.405812
11762034_TTLL9	-0.09807	0.295805	0.405932
11726811_GPR143	0.097872	0.295828	0.405953
11745292_C22orf15	-0.06032	0.295876	0.406008
11744219_G0S2	-0.05488	0.295905	0.406036
11735105_CD40LG	-0.08679	0.295917	0.406038
11736524_DCSTAMP	-0.09331	0.295933	0.406038
11735671_OC90	-0.08139	0.295938	0.406038
11754904_ZFAND4	0.061602	0.29594	0.406038
11751657_C1orf159	-0.06297	0.295955	0.406047
11759048_ABCB1	-0.08381	0.296027	0.406135
11740994_GLUL	0.043509	0.29604	0.406141
11738233_ROS1	-0.1132	0.296061	0.406158
11757346_FTL	0.037993	0.296103	0.406205
11748141_CRIP2	-0.04265	0.296143	0.406249
11731716_ACKR2	0.147461	0.296157	0.406256
11735509_RTP5	-0.05892	0.296216	0.406325
11750391_IL17RE	-0.07314	0.29623	0.406334
11738098_MYBPHL	-0.10374	0.296271	0.406376
11740373_TMEM130	0.066141	0.296278	0.406376
11747412_ATF7IP	0.05703	0.296437	0.406583
11757129_RPS3 ///	0.047503	0.296463	0.406607
11753633_TTTY8 ///	-0.09213	0.296492	0.406635
11728582_C9orf50	-0.09158	0.296502	0.406638
11738160_NANOS2	-0.06049	0.296522	0.406654
11731863_NKX2-2	-0.07016	0.296558	0.406691
11737937_RANBP3L	0.088418	0.296664	0.406826
11752990_SERTM1	-0.18053	0.296682	0.406828
11718555_CREB3L2	0.044753	0.296683	0.406828
11742838_ARRB1	0.080709	0.296782	0.406953
11747151_GORASP2	0.040991	0.296861	0.407041
11722008_PRKAR1A	0.041362	0.296863	0.407041
11745605_GNB1L	-0.08387	0.296912	0.40709
11758918_TFCP2	-0.03809	0.296915	0.40709
11724090_EDN3	0.101399	0.296961	0.407142
11727355_CALN1	-0.10905	0.29698	0.407156

11735461_SPRR2B	-0.06688	0.297008	0.407181
11743595_C5orf49	-0.10394	0.297015	0.407181
11752751_MIF-AS1	0.043142	0.297035	0.407197
11742122_GABRG3	0.064468	0.297052	0.407207
11762357_RGPD4	-0.05892	0.297059	0.407207
11760055_KCNG4	0.133401	0.297153	0.407325
11743240_LY6G6D	-0.06942	0.2972	0.407377
11740715_ARIH2OS	-0.0634	0.297287	0.407478
11741849_KRTAP17-1	-0.03991	0.29729	0.407478
11739827_HTR3A	-0.07014	0.297322	0.4075
11743832_RALGPS1	-0.11146	0.297325	0.4075
11749861_SERPINF2	-0.08289	0.297331	0.4075
11741322_CCR3	-0.0632	0.297371	0.407544
11743572_ANKRD17	-0.04019	0.297403	0.407575
11755899_ANKRD36 /	-0.05477	0.297411	0.407575
11737444_GPR156	-0.08874	0.297425	0.407584
11763271_PEX11A	0.054662	0.297514	0.407694
11763300_RBM4	0.069129	0.297672	0.407888
11759457_LOC650293	-0.09245	0.297673	0.407888
11759827_ZNF154	0.088682	0.298028	0.408364
11723205_PGD	0.047962	0.298067	0.408406
11743531_USP27X	0.041958	0.298092	0.408421
11739660_PPCS	0.04344	0.298095	0.408421
11731968_CACNA1I	0.068083	0.298191	0.408541
11727961_ING1	0.047428	0.298247	0.408599
11721750_FZD7	-0.05504	0.298252	0.408599
11721327_DFNB31	0.077138	0.298258	0.408599
11719706_RNF113A	0.048559	0.298288	0.408628
11749913_HSPA13	0.044195	0.2983	0.408628
11762465_LOC101060	0.1247	0.298312	0.408628
11738312_C15orf53	-0.06397	0.298318	0.408628
11752286_DPP6	-0.06753	0.298322	0.408628
11729363_ZPBP	0.041168	0.29833	0.408628
11726011_KIAA1683	0.058684	0.298429	0.408753
11745442_ACTR2	-0.04646	0.298443	0.40876
11742793_SBDS	-0.04597	0.298503	0.408831
11721412_BSPRY	0.052555	0.29852	0.408844
11718294_NME1-NME2	-0.03877	0.298603	0.408946
11729095_KRTAP1-5	-0.0491	0.29863	0.408971
11720629_KIAA0196	-0.03966	0.29868	0.409028
11739777_FNDC9	0.048581	0.298735	0.409092
11727753_GRIK3	-0.06107	0.298746	0.409095
11743937_SLC7A14	-0.09025	0.298788	0.409141
11761798_TMC6	-0.03964	0.298878	0.409253
11741334_ERN1	-0.09575	0.299003	0.409413
11754574_C6orf52	-0.07572	0.299041	0.409454
11741683_ATG4A	0.050531	0.299065	0.409474
11755020_SLC46A3	-0.05322	0.299121	0.409541
11753432_FLT4	-0.11441	0.29916	0.409582
11758186_ENSA	0.070007	0.299175	0.409591
11742486_PRAMEF19	-0.07416	0.299202	0.409607

11749809_PLAT	0.040066	0.299203	0.409607
11747646_MSTO1 ///	-0.0646	0.299269	0.409685
11740315_FAM83G	-0.06323	0.299283	0.409687
11760947_POLK	0.09728	0.29929	0.409687
11723773_ABHD12	0.056551	0.299295	0.409687
11734369_CTAGE1	-0.08225	0.299316	0.409703
11748868_ACCS	0.042564	0.299361	0.409753
11750303_B3GALT5-A	0.12421	0.299375	0.409762
11737314_HIST1H4B	-0.0739	0.299453	0.409857
11761422_CCDC12	-0.0953	0.299524	0.409943
11724382_ST6GAL2	-0.11566	0.299566	0.409988
11745792_LOC100288	-0.04634	0.299602	0.410026
11749166_TTC23	-0.05012	0.29961	0.410026
11738617_MYO7A	-0.04288	0.299651	0.41007
11736957_TRIM72	-0.12305	0.299702	0.410128
11745454_CCL28	0.053541	0.299733	0.410159
11726742_OXNAD1	-0.06636	0.299799	0.410238
11741956_LRRIQ1	-0.08565	0.29983	0.41027
11721713_EID2	0.043182	0.299852	0.410289
11738867_GFOD1	0.072148	0.299866	0.410296
11729297_MMP13	0.100778	0.299891	0.410311
11737974_PLEKHG5	-0.08411	0.299894	0.410311
11731168_IGFL1	-0.10631	0.299908	0.410318
11761281_TUBGCP2	-0.04651	0.299921	0.410325
11719229_URI1	0.042572	0.299945	0.410347
11753581_PAOX	0.110562	0.300029	0.410443
11715671_IFITM1	0.038887	0.300033	0.410443
11718758_HIF1A	-0.06231	0.300063	0.410473
11759248_HIST1H2AL	-0.10186	0.300229	0.410689
11743657_HPGD	-0.09874	0.300278	0.410718
11757691_KAT7	0.055656	0.300278	0.410718
11744088_TYMSOS	0.04268	0.300283	0.410718
11760599_HIP1	-0.08041	0.300284	0.410718
11763486_MAPT	0.049098	0.300351	0.410799
11746430_SLC16A7	0.036625	0.300386	0.410834
11736569_PON1	0.112099	0.300417	0.410866
11762542_TMPRSS13	-0.05252	0.300472	0.41093
11740809_PRAMEF13	-0.04667	0.300492	0.410945
11758052_COMMD5	-0.05257	0.300539	0.410998
11743394_AIG1	0.049002	0.300598	0.411064
11753337_TFAP2A	0.055508	0.300604	0.411064
11729825_BMPER	0.063324	0.300685	0.411163
11760210_RGP1	-0.04632	0.300737	0.411222
11745064_GNPTAB	-0.04458	0.3008	0.411297
11763728_FKBP9	-0.04151	0.300808	0.411297
11715920_PGRMC2	0.044796	0.300838	0.411327
11745672_NFATC4	-0.06943	0.300873	0.411362
11734404_PABPC4L	0.051373	0.300885	0.411367
11747084_TRNAU1AP	0.040904	0.300907	0.411386
11759690_SLC11A2	-0.08047	0.300979	0.411472
11735811_FFAR4	0.071364	0.301066	0.41158

11760411_AIDA	-0.13099	0.301127	0.411653
11720025_FAM118A	0.05126	0.301263	0.411827
11735793_GPR132	-0.05756	0.301281	0.411839
11748009_RHBDL2	0.090315	0.301298	0.411852
11763867_POLE3	0.099034	0.301342	0.411887
11746534_TLR10	-0.05089	0.301344	0.411887
11762392_MYO9A	0.055586	0.30135	0.411887
11739444_DPF2	-0.04073	0.301382	0.41192
11744310_DIS3L	0.065992	0.301433	0.411963
11755713_CLEC17A	-0.05028	0.301436	0.411963
11736794_GNAT2	-0.06609	0.301439	0.411963
11745068_ZNF833P	-0.1249	0.301469	0.411993
11759020_RNF187	-0.0885	0.301509	0.412037
11741512_SEC14L3	-0.05652	0.301566	0.412097
11749707_HECTD3	-0.04338	0.30157	0.412097
11735351_EPO	-0.06686	0.301629	0.412165
11723765_KCTD7	-0.0867	0.301639	0.412167
11762386_MYO3B	-0.13005	0.301672	0.412201
11729781_FAM127C	-0.05067	0.301748	0.412293
11750513_OLR1	-0.0382	0.301782	0.412329
11732655_RAX2	-0.06176	0.301805	0.412346
11717034_KIAA0141	-0.05367	0.301816	0.412346
11718924_WWP2	-0.04082	0.30182	0.412346
11721600_BRPF3	0.051219	0.301844	0.412367
11732703_TAS2R1	-0.05592	0.301863	0.412381
11750714_CHD3	0.080154	0.30188	0.412388
11754069_CD79A	-0.06659	0.301884	0.412388
11747006_INSC	0.069954	0.301929	0.412437
11757164_SNORA32 /	-0.06133	0.301952	0.412457
11720164_FKBPL	-0.04948	0.301964	0.412462
11731980_FNDC7	-0.07914	0.301999	0.412499
11741670_ESR2	-0.10317	0.302117	0.412648
11723030_CRISPLD2	0.06855	0.302134	0.412659
11745924_ZEB1-AS1	0.055823	0.302148	0.412668
11742521_OR4K17	-0.08371	0.302202	0.412729
11761477_LOC105369	-0.04335	0.302254	0.412778
11731696_MDH1B	0.076946	0.302255	0.412778
11759958_GTF2IRD2	0.056012	0.302268	0.412785
11723143_NALCN	0.047104	0.30231	0.412823
11717774_PLEKHB1	-0.06925	0.302313	0.412823
11748396_MYADM	0.037659	0.302334	0.41284
11737831_FNBP1L	0.041909	0.302378	0.412889
11720394_RAB11FIP4	-0.09394	0.302396	0.412902
11736160_GBP6	-0.05232	0.302422	0.412919
11735556_AMZ1	0.060014	0.302426	0.412919
11761485_FAM162A	0.109694	0.302493	0.413
11747755_KRT13	-0.0926	0.302517	0.413021
11730369_EIF4E3	-0.10352	0.302559	0.413067
11743170_RCSD1	-0.11858	0.302647	0.413175
11761054_TBC1D16	-0.05413	0.302664	0.413186
11715169_PLEKHG2	-0.04479	0.302672	0.413186

11736351_TMEM220	-0.04411	0.302737	0.413263
11728304_SYNDIG1	-0.06667	0.302752	0.413273
11760826_GALNT3	0.125399	0.302807	0.413337
11744149_LPIN2	-0.03798	0.302882	0.413427
11751831_ELK1	0.05357	0.302907	0.413449
11738309_SHANK2	-0.07819	0.302965	0.413504
11740836_NUGGC	0.088454	0.302965	0.413504
11746629_KIF18B	-0.0591	0.302972	0.413504
11729283_MEF2B ///	-0.08105	0.302986	0.413512
11746410_SLC7A8	-0.05008	0.303015	0.41354
11732681_ZNF471	-0.06434	0.30303	0.413549
11725338_NOL12	0.036499	0.303052	0.413567
11725871_ZNF616	0.069492	0.303098	0.413617
11724583_HDAC10	-0.05056	0.303106	0.413618
11721405_NCOA3	0.044569	0.303176	0.413702
11732596_SIGLEC15	-0.05733	0.30323	0.413756
11736786_CATSPER2	0.109181	0.303233	0.413756
11747462_SNX32	-0.06602	0.303302	0.413839
11734072_RBFOX1	-0.09065	0.303423	0.413991
11735577_IL27	-0.05515	0.30351	0.414093
11742902_AP3S1	0.041636	0.303515	0.414093
11745591_NOS3	-0.05684	0.303522	0.414093
11730673_OPRK1	0.048303	0.303535	0.414098
11723740_WDR36	-0.04271	0.303587	0.414158
11734027_ZNF440	0.065698	0.303652	0.414235
11759003_SLC33A1	-0.05556	0.303686	0.414263
11746782_FKBP9 ///	-0.05004	0.30369	0.414263
11726297_ZNF500	-0.09685	0.303716	0.414288
11741803_FAM76A	-0.08995	0.303917	0.414551
11742626_OR14A16	0.090589	0.303996	0.414647
11716544_TNNI1	-0.07306	0.304022	0.414671
11740678_TBXAS1	-0.07265	0.304065	0.414715
11729522_CA5B	0.055463	0.304071	0.414715
11723539_KNOP1	0.047909	0.304081	0.414716
11724030_HIC2	0.04137	0.304123	0.414762
11745033_LINC00313	0.044061	0.304161	0.414802
11716587_AXL	0.03694	0.304224	0.414876
11735353_SLC37A2	-0.07465	0.304257	0.414909
11744600_TESPA1	-0.12796	0.304283	0.414933
11731674_AGMAT	-0.0746	0.304295	0.414938
11761664_RIN1	-0.03944	0.304309	0.414946
11752411_CCDC8	-0.08381	0.304382	0.415034
11728422_GRASP	-0.06719	0.304422	0.415078
11754549_AGTPBP1	0.065932	0.304447	0.4151
11742130_HTR4	-0.06198	0.304502	0.415163
11747035_IL7	-0.0563	0.304521	0.415177
11760683_SH3PXD2A	0.086691	0.304612	0.41529
11718662_NENF	0.045637	0.304632	0.415305
11725556_PRRX2	-0.05482	0.30465	0.415318
11720191_NDUFAF4	0.083433	0.304676	0.415337
11762531_MEGF9	-0.09572	0.304681	0.415337

11756751_UBE2F	0.039429	0.304699	0.415342
11721553_GABBR1	0.047095	0.304702	0.415342
11761274_ENPP3	0.107426	0.304716	0.41535
11727504_SOX7	-0.09293	0.304813	0.415462
11720916_HSPA8	-0.03802	0.304815	0.415462
11735838_MADCAM1	-0.09068	0.304869	0.415525
11729392_TRIM3	-0.09438	0.305005	0.415697
11754740_UMODL1	-0.11765	0.305081	0.415779
11746381_PHLDB1	0.071337	0.305081	0.415779
11752124_SRRT	0.041306	0.305124	0.415825
11716687_ABR	-0.03697	0.305133	0.415826
11747904_PIP5K1C	-0.06137	0.305179	0.415878
11752008_CDAN1	-0.04926	0.305197	0.41589
11729079_ESRRG	-0.16367	0.305265	0.415972
11730907_ABCB1	-0.0496	0.305313	0.416025
11729529_KRT86	0.07147	0.305332	0.416039
11720212_PPAN ///	-0.06205	0.305343	0.416042
11755739_SLC35A1	0.043221	0.305359	0.416053
11760203_IGFBP7	-0.0658	0.305369	0.416055
11750426_MPPED1	-0.08196	0.305383	0.416062
11755239_ELL2	0.045721	0.305564	0.416291
11753305_DLG4	-0.04448	0.305568	0.416291
11739804_RASAL2	-0.05917	0.305633	0.416369
11751789_UBASH3A	-0.04268	0.305651	0.416381
11724443_MEF2B ///	-0.10489	0.305678	0.416407
11745078_SYCP1	0.07547	0.305758	0.416494
11762783_RPL18A	0.085237	0.305759	0.416494
11730842_POLR3F	-0.04203	0.305769	0.416495
11737716_ICAM4	-0.09582	0.305803	0.41653
11744432_KANSL1	0.041065	0.305824	0.416546
11726787_TMEM100	-0.07429	0.305847	0.416567
11743505_NARS2	-0.04554	0.305931	0.41667
11715375_CALR	0.049478	0.305959	0.416692
11763828_DPEP1	-0.05955	0.305965	0.416692
11729973_PRSS36	-0.05971	0.30599	0.416714
11717005_POLDIP3	-0.04448	0.306013	0.416732
11724498_MFNG	0.048216	0.306019	0.416732
11728812_OTUD7B	-0.0907	0.306038	0.416745
11728272_ZNF330	0.043968	0.306053	0.416754
11721421_RAP1GAP2	-0.04795	0.306107	0.416815
11743390_VPS35	-0.03955	0.306114	0.416815
11740837_TCEANC	0.125756	0.306169	0.416878
11752824_PLSCR3 //	0.03778	0.306261	0.416991
11738171_C10orf111	-0.0868	0.306273	0.416995
11741266_UGT2B15	-0.07371	0.306363	0.417107
11749199_STAC	-0.05676	0.30642	0.417165
11741480_HFE	-0.0562	0.306427	0.417165
11728466_GLDC	0.113239	0.306433	0.417165
11723775_ZNF580	-0.0438	0.306442	0.417165
11721167_EPB41L1	-0.07707	0.306448	0.417165
11756826_ENTPD3	-0.11499	0.306475	0.417189

11731380_FIGNL2	0.051296	0.306484	0.417189
11742024_TBL1X	0.061519	0.306658	0.417415
11743083_TDRP	0.047725	0.30675	0.417529
11732299_KIAA1715	-0.06284	0.306821	0.417605
11735790_PTPN18	-0.06218	0.306823	0.417605
11735459_FBXO39	-0.03918	0.30685	0.417629
11731385_GJC2	0.041319	0.306887	0.417668
11727886_ZNF25	-0.08657	0.306895	0.417668
11762184_SLC25A42	0.047409	0.306907	0.417673
11763446_TRDC ///	0.06211	0.306919	0.417677
11745806_AMMECR1L	0.038198	0.306957	0.417717
11732748_DNAH6	-0.06066	0.307043	0.417823
11738622_XKR5	-0.08531	0.30709	0.417876
11734716_CBLL1	-0.0502	0.307218	0.418037
11732601_CEP135	0.063819	0.307354	0.418211
11731305_ABCC11	0.062651	0.307372	0.418222
11759952_TCFL5	-0.05055	0.307379	0.418222
11739382_GPR85	0.100581	0.307449	0.418306
11749168_KRT6A	-0.04776	0.307481	0.418337
11753180_CARF	0.046863	0.307529	0.418384
11740362_FOXN3	0.052396	0.307539	0.418384
11741565_C1QTNF7	0.060037	0.307541	0.418384
11728505_ZNF524	-0.05044	0.307555	0.418391
11762875_RPL4	0.041201	0.307578	0.41841
11737206_SPOCK2	-0.06165	0.307586	0.41841
11762814_PABPN1L	-0.0523	0.307602	0.41842
11748185_CCDC57	-0.04455	0.307618	0.418431
11749743_MST1R	-0.04471	0.307658	0.418473
11753440_GBA	0.051348	0.307688	0.418503
11754162_IL7	0.054699	0.307766	0.41859
11762750_ERG	-0.05419	0.307769	0.41859
11729785_SOX13	-0.07322	0.307821	0.418649
11723286_FIS1	0.038087	0.307831	0.418651
11759534_TP53I11	0.060216	0.307871	0.418693
11742594_OR8K3	-0.15048	0.307897	0.418716
11739615_CD3EAP	-0.04398	0.307905	0.418716
11760975_SP2	-0.09419	0.307976	0.418802
11747214_LOC105377	0.05957	0.308046	0.418884
11756929_CXXC1	-0.05956	0.308112	0.418962
11742329_OR51D1	0.040592	0.30813	0.418975
11745967_BCLAF1	-0.0472	0.30816	0.419005
11717724_RAB1A	0.037237	0.30818	0.41902
11763885_LINC01193	-0.07927	0.30821	0.419044
11731811_FCRL4	0.066764	0.308215	0.419044
11759985_TMEM161B	0.060746	0.308232	0.419056
11722619_AQP4	-0.06163	0.308243	0.419059
11721562_FAM189B	0.035828	0.308271	0.419075
11745051_GRIA3	0.055388	0.308272	0.419075
11740872_MS4A7	0.057744	0.308316	0.419123
11715178_OR2A14	-0.05485	0.308467	0.419307
11733307_TFAP2B	-0.0377	0.308468	0.419307

11732309_ZNF777	-0.04443	0.308516	0.41936
11761563_HEATR1	0.05919	0.308531	0.419369
11758576_CCDC59	0.119494	0.308571	0.419411
11745281_LOC100287	-0.08768	0.308612	0.419457
11740729_KRTAP5-8	0.074216	0.308625	0.419463
11754640_EPHX2	-0.08307	0.308645	0.419478
11734307_IL10	-0.04719	0.308715	0.419561
11728991_IGDCC4	0.106293	0.308736	0.419575
11738090_C5orf56	0.041883	0.308743	0.419575
11747431_SPSB2	-0.08004	0.308766	0.419595
11736201_GLUD2	-0.07092	0.308794	0.419622
11724547_RSBN1	0.061087	0.308827	0.419644
11754011_GPC3	-0.06152	0.308838	0.419644
11724655_SON	0.048875	0.308844	0.419644
11751271_PKNOX1	-0.04098	0.308845	0.419644
11742161_C22orf42	-0.08134	0.308864	0.419658
11735982_KRTAP9-9	-0.05614	0.308896	0.41969
11738002_SLC35E2	-0.06644	0.30891	0.419697
11752522_ST8SIA5	-0.0636	0.308936	0.41972
11719356_FAM109B	-0.0701	0.308944	0.41972
11753921_C7orf13	-0.05185	0.308965	0.419728
11733591_C1orf204	0.069061	0.30898	0.419728
11720980_PTK2B	-0.12766	0.308994	0.419728
11752107_FBF1	-0.05268	0.308999	0.419728
11751249_SPATA20	0.049132	0.309001	0.419728
11762448_FNDC3B	0.078512	0.309001	0.419728
11735712_CSNK1G2	-0.05075	0.30904	0.419769
11745711_ZNF365	-0.09241	0.30905	0.41977
11725235_ZBTB39	0.054018	0.309063	0.419777
11763497_POLE3	-0.04523	0.309077	0.419778
11740137_MCTP2	0.095538	0.309081	0.419778
11746655_ACAA1	0.04224	0.309099	0.419791
11755393_HHLA2	0.131979	0.30911	0.419794
11757636_ATP5L	-0.04083	0.309149	0.419835
11730443_CCNJ	0.043816	0.30924	0.419947
11763187_LOC105376	0.044789	0.309325	0.420052
11729538_LYPD8	-0.06938	0.309352	0.420077
11727722_MYB	-0.03668	0.309368	0.420079
11740892_KCNK7	0.065605	0.309391	0.420079
11751844_STMN2	-0.05097	0.309392	0.420079
11715205_PSG4	0.048549	0.309397	0.420079
11715206_PSG4	0.048549	0.309397	0.420079
11749952_CFAP46	0.099145	0.309481	0.420179
11758412_ALAS2	-0.05334	0.309488	0.420179
11752277_STON2	0.134453	0.309497	0.42018
11732432_GK	0.071909	0.309597	0.420303
11755987_ANKRD44	0.115256	0.309627	0.420331
11747373_DPF3	0.119852	0.309634	0.420331
11721267_TMPRSS4	0.079276	0.309705	0.420415
11720607_SFRP2	-0.06065	0.30972	0.420424
11751880_PIK3C2B	-0.0534	0.309824	0.420549

11752813_MSC-AS1	0.118524	0.309829	0.420549
11734194_UNC5D	-0.11247	0.309917	0.420645
11733556_CCDC67	0.13223	0.309921	0.420645
11733377_DCAF4L1	0.090627	0.309927	0.420645
11728406_DERL3	-0.04905	0.309934	0.420645
11740996_SPAG6	0.060493	0.309946	0.42065
11723886_NATD1	-0.07962	0.310034	0.420757
11749216_HAVCR1	-0.1582	0.310065	0.420787
11741819_TGM1	-0.0801	0.310087	0.420805
11763917_MED24 ///	-0.06492	0.310096	0.420805
11733627_FAM196A	0.050013	0.310181	0.42091
11732455_KCNJ15	0.054839	0.310233	0.420968
11763237_IGLV1-41	-0.0526	0.31028	0.42102
11740015_RPL28	-0.04057	0.310318	0.42106
11742767_PANK2	0.072131	0.310441	0.421215
11747809_PFKFB4	0.056059	0.310498	0.421281
11747077_EEF1A1 //	0.035863	0.310539	0.421325
11741963_GLRA3	-0.08008	0.310573	0.421359
11760883_FADD	0.089542	0.310583	0.421362
11763644_FMNL1	-0.11166	0.310627	0.421409
11732363_ZNF2	0.109133	0.310668	0.421454
11722187_AKT1	-0.04613	0.310683	0.421457
11721301_RAB4A	0.040807	0.310688	0.421457
11757867_BZW1	0.044931	0.310719	0.421488
11727289_LIM2	-0.09526	0.310749	0.421517
11731058_RASAL1	-0.04959	0.31077	0.421532
11723372_DOCK11	0.051929	0.310784	0.421532
11745976_ANXA11	-0.04609	0.31079	0.421532
11752725_GNB2L1	0.037194	0.310795	0.421532
11750472_PCK1	-0.09782	0.310814	0.421545
11741517_FCGR2B	-0.06055	0.31084	0.42157
11731312_WISP3	0.111798	0.31085	0.421571
11732069_GPR88	-0.06617	0.310892	0.421615
11734695_PPP4C	0.041792	0.3109	0.421615
11736386_LHX2	-0.0497	0.310949	0.421671
11762306_IRF5	-0.07736	0.311017	0.421751
11717057_CORO1C	0.046042	0.311033	0.421761
11717050_DES	-0.0496	0.311063	0.421791
11742383_OR5H1	-0.0538	0.311083	0.421805
11727664_STOX1	-0.13004	0.311102	0.421815
11739935_TSC1	0.05483	0.311107	0.421815
11718744_CXCL12	0.070737	0.311132	0.421837
11753801_LOC100128	-0.08964	0.311153	0.421854
11753613_LCN10	-0.07695	0.311197	0.421902
11726873_LIPE	-0.06076	0.311217	0.421917
11724730_YY1	-0.04365	0.311244	0.421942
11762362_TDRD6	-0.13428	0.311271	0.421967
11763564_ATP10B	-0.06027	0.311316	0.422016
11762797_SMARCB1	-0.04324	0.311338	0.422035
11726521_SPATA17	0.115527	0.31135	0.422038
11745306_STAM	0.037915	0.311379	0.422066

11753230_GATS	-0.04846	0.31145	0.42215
11718061_PVALB	0.099459	0.311553	0.422279
11742336_OR13D1	-0.09141	0.311563	0.422281
11749184_DTNA	0.096391	0.311661	0.422401
11756215_UBA52	0.03607	0.311733	0.422466
11730472_PDE3B	-0.0593	0.311737	0.422466
11737350_STAG3L2	0.053705	0.311739	0.422466
11718933_FKBP1A-SD	-0.04728	0.311747	0.422466
11755238_AFMID	0.057884	0.311752	0.422466
11733689_PANX3	-0.10358	0.311834	0.42256
11735879_BHLHE23	-0.04833	0.311838	0.42256
11762360_CACNA1I	-0.09884	0.311904	0.422637
11728621_FNDC8	0.053793	0.311958	0.422699
11761928_CPED1	0.096912	0.311984	0.422722
11760781_DCAF5	0.095174	0.312018	0.422757
11733523_PPP1R3A	0.057505	0.312254	0.423064
11760089_PMCHL1	0.071538	0.3124	0.423243
11740724_MOGAT3	0.048874	0.312403	0.423243
11751208_SLCO2A1	-0.05861	0.31257	0.423457
11743026_ 11-Mar	0.055525	0.31261	0.423499
11737351_ANO7	0.067303	0.312624	0.423499
11741164_CST5	-0.04987	0.312626	0.423499
11739598_ZNF398	0.055269	0.312656	0.423527
11750317_KCNS1	-0.05458	0.31278	0.423683
11730615_PLCD4	0.14411	0.312791	0.423687
11727342_ATG9B	0.051914	0.312838	0.423739
11730688_FOXP3	-0.08551	0.313026	0.42397
11742652_SPANXN1	0.100744	0.313026	0.42397
11754952_PLCD3	-0.05735	0.31304	0.423977
11749277_C14orf180	-0.05288	0.313133	0.424091
11741647_ESRRG	-0.06337	0.313169	0.424128
11760582_NOSIP	0.059362	0.313189	0.424144
11721107_TRABD	-0.04742	0.313202	0.42415
11761126_ZNF396	0.082365	0.313249	0.4242
11731193_UNC13C	-0.05233	0.313276	0.424226
11746007_SEC16A	0.041315	0.31335	0.424314
11761220_CRACR2B	-0.06669	0.31337	0.42432
11739504_NICN1	0.093613	0.313372	0.42432
11737214_LINC01465	-0.08375	0.31339	0.424327
11732568_MBP	-0.06529	0.313394	0.424327
11753943_MICAL2	-0.06925	0.313424	0.424356
11763100_MT1JP	0.10011	0.313468	0.424398
11722869_RAD1	-0.03919	0.313472	0.424398
11739127_DNAJB2	-0.08582	0.313485	0.424404
11732513_CDX1	-0.08062	0.313513	0.42443
11729109_MBP	0.068416	0.313604	0.42454
11753474_BRF1	0.060465	0.313624	0.424556
11759763_ODF3B	-0.06535	0.313642	0.424569
11736644_G6PC2	-0.09473	0.313681	0.42461
11718954_FBLIM1	0.055019	0.313697	0.424613
11729383_DMRTB1	-0.0493	0.313701	0.424613

11734935_ARID5A	0.053227	0.313713	0.424618
11759713_RAB6A	0.103186	0.313737	0.424638
11760985_EMC4	0.142491	0.313768	0.42466
11735021_ZNF462	-0.04199	0.313779	0.42466
11750660_TEP1	0.039234	0.313779	0.42466
11746912_TNIP3	-0.0965	0.313884	0.424791
11727241_WNT4	-0.07449	0.313922	0.42483
11725147_MCHR1	-0.0389	0.314	0.424924
11722740_DDC	0.087671	0.314022	0.424938
11746317_CFAP20	-0.04745	0.314028	0.424938
11722780_SLC6A19	0.052176	0.31407	0.424984
11719218_SOCS3	0.065749	0.314088	0.424996
11738806_DNMT3B	-0.04564	0.314102	0.425003
11727143_EPB41L3	0.127327	0.314153	0.42506
11750520_ASMTL-AS1	0.036654	0.314219	0.425138
11742674_LCE3B	-0.05048	0.314265	0.425189
11760868_ACOT8	-0.11778	0.31429	0.425199
11746334_SV2B	-0.062	0.314291	0.425199
11719058_FAM53C	0.063424	0.3143	0.425199
11744318_CCDC85A	0.081758	0.314311	0.425199
11738886_ASMT	0.0564	0.314317	0.425199
11753743_PAIP2	0.041338	0.314355	0.425239
11743400_CDC27	-0.03806	0.314369	0.425247
11750453_PARK2	-0.04895	0.314557	0.425489
11737914_GPR15	-0.06158	0.314605	0.425541
11738697_OR1N2	-0.05324	0.314613	0.425541
11746773_SLC27A1	-0.04853	0.314623	0.425542
11758470_ACTN2	-0.05787	0.314631	0.425542
11738786_LOC105369	0.043781	0.314728	0.425661
11740409_FGFR2	0.084576	0.314812	0.425764
11753037_ANKS1B	-0.04846	0.314869	0.425828
11726676_APOBEC2	0.067889	0.31494	0.425913
11753324_MYO5C	0.064176	0.315008	0.425993
11733506_MAGEB4	-0.10336	0.315026	0.426006
11744363_INPP5D	-0.04743	0.315037	0.426009
11725796_S1PR2	-0.03559	0.315074	0.426047
11720746_BCL6	-0.04397	0.315234	0.426251
11725957_TIMM17A	0.039191	0.315265	0.426279
11719031_PIP5K1C	0.043162	0.315272	0.426279
11748821_SORBS2	0.048699	0.315281	0.42628
11747716_PMFBP1	-0.06549	0.315359	0.426373
11750412_HAS3	-0.12413	0.315379	0.426388
11735396_ZNF70	-0.04636	0.315484	0.426518
11742353_OR11L1	-0.06897	0.315506	0.42653
11760281_H3F3A	-0.14658	0.31551	0.42653
11717359_RTF1	0.038681	0.31554	0.42656
11750464_CLEC4M	-0.07142	0.315593	0.426619
11739773_SNX20	-0.05418	0.315606	0.426625
11727967_MSMB	-0.04947	0.315667	0.426695
11720046_DNAJC10	0.045601	0.315734	0.426774
11716712_TCEAL8	-0.03708	0.315743	0.426775

11753707_CCDC17	-0.0725	0.315766	0.426784
11749126_HTR2C	-0.07096	0.315768	0.426784
11751611_TRAFD1	0.072683	0.315806	0.426824
11732356_ZNRF1	-0.05601	0.31586	0.426886
11748853_EFCAB1	0.177444	0.315956	0.427003
11733241_IRAK2	-0.07861	0.316063	0.427136
11731025_DAB1	-0.0982	0.316171	0.42727
11759608_EPB41L5	0.081299	0.316233	0.427342
200042_PMRTCB	-0.04416	0.316261	0.427368
11760707_EIF4A2	0.05224	0.316292	0.427399
11756165_GLDC	-0.09036	0.316305	0.427403
11725926_CLIC4	-0.0596	0.316328	0.427424
11724674_FAM149B1	-0.06672	0.316452	0.427572
11721349_LOC102725	-0.05305	0.316469	0.427572
11730963_LOC145783	0.092922	0.316472	0.427572
11754758_MKRN1	0.038638	0.316473	0.427572
11756372_APOOL	-0.05346	0.31649	0.427583
11727534_AKAP3	0.068871	0.316635	0.427767
11761621_TIPIN	-0.04821	0.316658	0.427787
11723968_PPCS	0.048487	0.316671	0.427792
11751033_ABHD8	-0.05531	0.31671	0.427832
11726520_RNF32	-0.04052	0.31673	0.427848
11763005_YME1L1	-0.09017	0.316741	0.427851
11740069_KRT222	0.090361	0.316767	0.427866
11746909_A1CF	-0.07706	0.31677	0.427866
11748008_CMAHP	0.073318	0.316822	0.427925
11749825_SEPT6	-0.04817	0.316858	0.427954
11732161_ZCRB1	-0.03801	0.316861	0.427954
11741851_INS /// I	-0.03936	0.31691	0.428008
11744334_RPS17	-0.0357	0.316941	0.428034
11754381_TMOD1	-0.0385	0.316946	0.428034
11748096_THUMPD3	0.04024	0.316966	0.428048
11742361_OR4C13	0.103588	0.316974	0.428048
11761797_C19orf45	-0.07669	0.317042	0.428128
11754775_H3F3B	0.035765	0.317078	0.428164
11731051_CEP126	-0.05334	0.317124	0.428207
11748310_PDDC1	-0.04176	0.317127	0.428207
11719815_BET1	-0.04601	0.317186	0.428274
11737705_LOC105370	0.083433	0.317274	0.428382
11727953_PACS2	-0.08791	0.317343	0.428463
11755729_CCDC163P	0.067654	0.317388	0.428506
11744328_RPL17 ///	-0.03501	0.317398	0.428506
11762527_LINC00315	-0.07878	0.317401	0.428506
11753059_UGGT2	0.0585	0.317418	0.428517
11763923_RPL32 ///	-0.04549	0.317451	0.42855
11725432_PBX2	0.053474	0.317508	0.428612
11728998_GCNT2	0.066041	0.317514	0.428612
11739764_KIAA0895L	-0.07578	0.317548	0.428631
11747293_CD1B	-0.08056	0.317553	0.428631
11732586_GRID1	-0.06404	0.317555	0.428631
11757870_NEURL1	-0.04892	0.317577	0.428641

11760328_MRPL9	-0.07056	0.31758	0.428641
11736153_XPR1	0.040056	0.317602	0.428659
11733729_LGMN	-0.0419	0.317627	0.428681
11725897_TUBB1	0.038698	0.317704	0.428772
11741483_CACNB4	0.062382	0.317712	0.428772
11759112_LOC100506	-0.07524	0.317755	0.428818
11722154_SGMS2	0.08523	0.317791	0.428854
11745323_GMIP	-0.06878	0.317811	0.42887
11742141_OR1C1	-0.07739	0.317905	0.428984
11722732_APPL1	-0.04179	0.317954	0.429039
11738168_BATF3	-0.06915	0.318057	0.429163
11722630_ICE2	-0.06468	0.318063	0.429163
200087_PM_TMED2	-0.0368	0.318075	0.429166
11716543_TNNI1	-0.05452	0.318214	0.429342
11758361_VNN1	-0.0728	0.318233	0.429351
11716506_CUL3	0.040022	0.318238	0.429351
11731929_DEFB127	-0.0634	0.318298	0.42942
11723164_LRRC15	-0.10485	0.318335	0.429458
11750096_CTNND2	0.117666	0.318364	0.429486
11745639_GRIK4	-0.08044	0.318411	0.429538
11735327_LMX1B	-0.05721	0.318433	0.429555
11760118_CNKSR2	-0.0879	0.318463	0.429582
11727693_SDR42E1	-0.13223	0.318471	0.429582
11724121_TRIM59	-0.06643	0.318492	0.429599
11727691_IL11	-0.05746	0.318548	0.429663
11729769_CD38	-0.05242	0.318558	0.429665
11733425_PMCH	-0.04764	0.318595	0.429702
11739510_SNED1	0.093165	0.318605	0.429704
11748444_PRTFDC1	-0.08799	0.31864	0.429739
11738745_OR1M1	-0.06259	0.318651	0.429742
11733555_PAX7	0.070552	0.318786	0.429908
11760346_HES1	-0.07488	0.318792	0.429908
11725012_KCNIP3	-0.07671	0.318815	0.429928
11745151_ARFGAP1	0.040466	0.318833	0.42994
11758607_VWA8	0.054112	0.318845	0.429945
11738700_OR51S1	-0.08704	0.318904	0.430012
11761838_AFF1	0.091034	0.318914	0.430013
11740890_ADAM28	-0.07845	0.31901	0.430131
11738039_PCDHAC2	0.044805	0.319061	0.430189
11764172_LOC102724	-0.11581	0.3191	0.430229
11721225_MTSS1L	-0.09334	0.319171	0.430313
11750894_TBX5	0.12183	0.319198	0.430338
11761509_KRT87P	-0.0872	0.319224	0.430358
11726096_RBMXL1	-0.05387	0.319231	0.430358
11732010_TNFAIP8L3	-0.07264	0.31925	0.430372
11715187_PRB1	-0.10732	0.319286	0.430408
11746526_PITRM1	0.054079	0.319385	0.430525
11717889_GNAI2	-0.04173	0.31939	0.430525
11717865_PSME4	0.045845	0.319427	0.430563
11721118_ANKHD1-EI	0.078372	0.319464	0.430601
11721979_SLC36A1	0.067959	0.319475	0.430604

11751853_MTAP	0.054095	0.319539	0.430678
11761862_SCN11A	-0.06683	0.31959	0.430736
11759752_GALNT5	0.106251	0.319623	0.43076
11759908_ITGBL1	0.043724	0.319626	0.43076
11742181_LCE3A	-0.04655	0.319648	0.430778
11727070_ORC5	0.038507	0.31968	0.430809
11742167_BSX	-0.07495	0.319896	0.431084
11761359_UBE2E1	-0.08179	0.319901	0.431084
11715474_DDX17	-0.0458	0.319932	0.431112
11725584_HAS3	0.058357	0.319941	0.431114
11738397_LHX6	-0.0842	0.319959	0.431116
11749976_DSE	0.052084	0.319961	0.431116
11753120_MADCAM1	-0.05108	0.319973	0.431121
11733769_RAB40A //	-0.07009	0.32	0.431143
11736218_GPR153	-0.11461	0.320011	0.431143
11758663_MATR3	-0.03484	0.320016	0.431143
11758985_ABHD15	0.074203	0.320053	0.431181
11753097_ACAD10	-0.07145	0.3201	0.431232
11760577_ZNF610	-0.07571	0.320152	0.43129
11751766_ECHDC2	-0.05488	0.320226	0.431378
11754862_RARRES2	-0.04291	0.320243	0.43139
11722340_PRR12	0.054105	0.320279	0.431426
11762601_SEC31B	0.081887	0.320307	0.431452
11752348_TBC1D30	-0.07713	0.320397	0.431561
11730067_GZMM	0.037288	0.320438	0.431605
11719197_CCND2	-0.0544	0.320518	0.431701
11727051_HOXB13	-0.08947	0.320609	0.431799
11737670_POLN	-0.05307	0.320609	0.431799
11751830_BCL2L11	-0.06067	0.32063	0.431816
11763952_PISRT1	-0.10002	0.320666	0.431851
11761639_HLA-DQB1	-0.06493	0.32073	0.431926
11718596_CCDC12	0.044905	0.320786	0.43199
11760980_PLA2G6	0.067016	0.320805	0.432003
11761398_TMEM138	0.039069	0.320818	0.432009
11742538_OR9G4	-0.08799	0.320871	0.432069
11742967_MTERF4	0.105629	0.320993	0.432221
11730167_GAST	-0.12991	0.321016	0.43224
11756759_IFT172	-0.0796	0.321189	0.43246
11752365_XRCC6P5	-0.09116	0.321295	0.432592
200036_PM RPL10A	0.039711	0.321327	0.432623
11750231_IGHG1 ///	-0.11193	0.3214	0.432704
11730291_ARHGAP5	-0.04058	0.321405	0.432704
11732515_BAK1	-0.14143	0.321427	0.432721
11761757_FXYD7	-0.07463	0.321437	0.432724
11726232_PRX	0.041106	0.321473	0.43276
11741083_EIF4G2	0.03793	0.321563	0.432869
11727635_SERPINI2	-0.08971	0.321659	0.432986
11723534_BRMS1L	-0.04309	0.321688	0.433013
11729958_SDK2	0.065437	0.321712	0.433028
11763006_TRAV21	-0.04508	0.321717	0.433028
11756802_RD3	-0.0637	0.321735	0.433041

11735552_FCRL6	-0.08514	0.321768	0.433073
11753869_AICDA	-0.08378	0.321809	0.433117
11760042_NPAS3	0.140575	0.321906	0.433235
11762458_SIGLEC6	0.069788	0.321972	0.433312
11726753_C9orf91	-0.06517	0.322032	0.433381
11741379_BACH2	-0.07828	0.32207	0.433421
11734456_SLC17A8	-0.10469	0.322099	0.433447
11724691_RIBC2	-0.06824	0.322115	0.433457
11733269_NAT8L	0.046168	0.322134	0.433471
200012_PM RPL21	0.035395	0.32215	0.43348
11758807_ECD	0.047697	0.322172	0.433497
11731898_ACTL10	-0.06859	0.322191	0.433503
11742179_HIGD1C	-0.07026	0.322194	0.433503
11728244_SLC12A1	0.105788	0.32223	0.43354
11741940_KRTAP11-1	-0.06529	0.322259	0.433567
11750920_CDC7	-0.07109	0.322298	0.433608
11727222_EVI5	-0.05477	0.32243	0.433773
11758410_SMCO3	-0.14427	0.322439	0.433774
11746073_TRPM2	-0.04879	0.322452	0.433779
11764140_ZDHHC11	0.045853	0.322472	0.433794
11749784_CDKL3	0.05527	0.322516	0.433842
11748001_SLC35F6	-0.05087	0.322598	0.43394
11720682_TIFA	0.054376	0.322622	0.433954
11761280_ACAT1	0.121105	0.322627	0.433954
11735641_HMSD	-0.05443	0.322645	0.433968
11761701_AP4B1	0.075526	0.322673	0.433993
11753796_C1RL-AS1	-0.08438	0.322684	0.433996
11730978_GAD2	0.101555	0.322737	0.434055
11734940_SLC16A9	-0.08656	0.322797	0.434124
11751706_CLN6	-0.03831	0.322888	0.434222
11753969_LILRA4	-0.06145	0.322888	0.434222
11743455_AP5M1	-0.05149	0.323071	0.434452
11751973_LZTS3	-0.04438	0.323076	0.434452
11722401_PAGE4	-0.04186	0.323134	0.434517
11725112_NDE1	-0.05671	0.323197	0.43459
11738261_ZNF493	0.050669	0.32324	0.434636
11731812_FCRL4	-0.12744	0.323254	0.434643
11736473_TCF7	0.057754	0.323277	0.434662
11751909_LAMC3	0.064498	0.323345	0.434741
11734656_MAG	0.057145	0.323368	0.43476
11753924_ATG3	0.051351	0.323393	0.434778
11726499_ANO5	-0.06355	0.323399	0.434778
11742489_ASB18	-0.07657	0.323533	0.434944
11738435_CD74	-0.04367	0.323541	0.434944
11734547_TGM4	-0.05872	0.323563	0.434962
11758394_PARM1	0.046913	0.323588	0.434984
11736317_STAP2	0.038853	0.323619	0.435014
11737063_MTL5	-0.05861	0.323722	0.43514
11751344_LEFTY2	0.047185	0.323748	0.435163
11747475_SPIDR	-0.04416	0.32378	0.435194
11744321_TLE6	-0.06735	0.323799	0.435209

11730538_ZSCAN10	-0.06335	0.323818	0.435214
11732005_ADAL	-0.05217	0.323824	0.435214
11750742_DHX58	-0.09189	0.323831	0.435214
11724043_PDZD4	0.051865	0.323922	0.435325
11738085_SLC36A3	-0.06088	0.324014	0.435437
11758302_LINC01001	-0.03521	0.324032	0.435449
11730117_TEX33	-0.06862	0.324041	0.435449
11752674_SPDEF	-0.09029	0.324072	0.435479
11724158_ECSCR	0.071289	0.324095	0.435498
11737455_FBXO47	0.074729	0.324128	0.435522
11750468_NUP35	0.046919	0.324142	0.435522
11730610_MCOLN2	-0.05026	0.324155	0.435522
11738796_PKHD1	0.053495	0.324156	0.435522
11718953_EPB41L5	-0.06509	0.324157	0.435522
11742097_SEC61A2	0.061139	0.324187	0.435547
11722718_BAIAP2	0.079484	0.324194	0.435547
11744191_SPNS3	0.044534	0.324228	0.435575
11755216_CDH16	-0.05047	0.324232	0.435575
11750820_MIPOL1	0.060223	0.324306	0.435657
11756281_CD7	-0.04312	0.324312	0.435657
11736615_AVPR1A	0.051256	0.324354	0.435702
11715721_GOLM1	0.069584	0.324433	0.435786
11720769_PPIF	0.055519	0.324435	0.435786
11724698_PHF24	0.048179	0.324465	0.435815
11759337_ATP10A	0.046222	0.324513	0.435857
11736343_OPA3	-0.05128	0.324513	0.435857
11732384_FBXO46	-0.05109	0.324869	0.436321
11717919_SRRT	-0.05732	0.324877	0.436321
11730495_TYR	-0.05023	0.324953	0.43641
11718301_PLAC9	-0.07599	0.324978	0.436432
11733174_ZNF507	0.047147	0.325032	0.436489
11732992_DCHS2	0.047006	0.325038	0.436489
11726631_TRIM8	0.061191	0.325112	0.436577
11736684_FGF5	0.107553	0.32514	0.436594
11763737_MBNL3	-0.07622	0.325143	0.436594
11724713_FAM212A	0.061497	0.325232	0.436679
11748981_NAT1	0.053162	0.325234	0.436679
11753029_TMEM178A	0.049328	0.325238	0.436679
11741652_PAX8	-0.04177	0.325242	0.436679
11735276_IL18R1	0.09801	0.325302	0.436739
11735356_HTR4	0.063317	0.325305	0.436739
11759349_F2RL3	-0.09821	0.325344	0.43678
11737661_B4GALT2	-0.03884	0.325358	0.436786
11757059_RPL36A //	0.035321	0.325436	0.43688
11741527_GAGE1 ///	0.150637	0.325461	0.436901
11729049_SLC10A2	0.061056	0.325506	0.43695
11755473_ZNF250	0.049651	0.325526	0.436965
11743993_KCTD5	-0.0403	0.325642	0.437097
11756563_RCBTB2	-0.04647	0.325643	0.437097
11758988_SYT17	-0.05089	0.325675	0.437128
11753881_FXYD2 ///	-0.05021	0.325691	0.437138

11749297_MAPK14	-0.04377	0.325737	0.437187
11751808_P3H3	-0.04995	0.325773	0.437224
11739178_ISM2	0.078338	0.325794	0.43724
11729428_ARHGAP36	0.101442	0.325819	0.437261
11719103_CPNE3	-0.03883	0.325962	0.437442
11738104_PPM1L	0.067449	0.325976	0.437449
11746415_ABCB5	-0.04485	0.325995	0.437462
11720981_PTK2B	0.061828	0.326032	0.4375
11734376_POU4F2	-0.08009	0.326078	0.437527
11758124_RCBTB2	-0.10647	0.326085	0.437527
11761733_KIF26B	0.109333	0.326085	0.437527
11746590_RGS8	-0.05408	0.326089	0.437527
11734260_TRIML1	-0.05644	0.326101	0.437532
11727404_GPC4	-0.07006	0.326168	0.437609
11722907_MIA3	0.041658	0.326195	0.437634
11725671_IPO7	0.035262	0.32621	0.437642
11716704_TRIM44	0.037984	0.326262	0.4377
11761277_LRRC37A4F	-0.05793	0.326313	0.437756
11745584_ZFP57	0.08092	0.326359	0.437806
11759350_METTL20	-0.12318	0.326403	0.437849
11738913_CD200R1	-0.05185	0.326409	0.437849
11759043_VKORC1L1	0.046841	0.326434	0.437871
11734414_CALCB	-0.06055	0.326457	0.43789
11727124_LRP2	-0.0724	0.326699	0.438195
11755378_WNK2	0.085501	0.326703	0.438195
11733380_SPZ1	0.047691	0.326722	0.438206
11763087_C5orf17 /	0.101207	0.326729	0.438206
11731764_CD3G	-0.08728	0.326824	0.438321
11737025_FAM83E	0.049995	0.326888	0.43838
11723133_RGS9	-0.07677	0.326892	0.43838
11715357_RPS21	0.034083	0.326894	0.43838
11761732_PILRA	-0.04496	0.326914	0.438394
11748553_UQCC1	0.068239	0.326928	0.438401
11732440_BPTF	0.040641	0.326954	0.438423
11737244_ALS2CR12	0.06666	0.326997	0.438469
11757150_EEF1B2 //	-0.06618	0.327037	0.438511
11743707_EXOC1	0.049956	0.327053	0.43852
11744240_SNTN	0.046302	0.327084	0.438549
11752311_CEP68	-0.04441	0.327146	0.438621
11749392_ATP5C1	-0.03691	0.327198	0.438679
11763147_BCL2L11	-0.12086	0.327231	0.43871
11737838_NSMF	-0.04585	0.32725	0.438724
11762039_ADAM17	-0.06498	0.327283	0.438757
11758545_RHAG	-0.05928	0.327332	0.43881
11734553_ANXA8 ///	-0.08993	0.327356	0.43883
11721038_SUFU	-0.04296	0.327394	0.438863
11726506_FUT9	-0.0963	0.327398	0.438863
11761129_ADIRF-AS1	-0.10483	0.32741	0.438866
11718575_SERBP1	-0.03936	0.327437	0.438885
11732632_HOXA7	-0.05845	0.327441	0.438885
11756754_PLSCR5	-0.04932	0.327487	0.438923

11748474_HOXB3	0.041338	0.327488	0.438923
11720495_YME1L1	0.039533	0.327656	0.439136
11737934_RUSC2	0.059546	0.327762	0.439266
11720521_ARHGEF17	-0.04453	0.327812	0.439321
11741413_FAM101A /	0.132079	0.327836	0.43934
11738510_NPPC	-0.06104	0.327869	0.439374
11753644_ADM2	0.049668	0.327889	0.439387
11748979_TIAL1	0.040891	0.327897	0.439387
11746125_NUP50	-0.03821	0.327921	0.439407
11719505_DHX36	0.04883	0.327935	0.439413
11739947_PFKFB2	-0.05486	0.327949	0.43942
11749654_BCAR1	0.050213	0.327965	0.43943
11746709_MAML1	-0.0524	0.327975	0.439431
11738751_OR13C2	-0.07122	0.328001	0.439449
11760078_NLN	0.056941	0.328007	0.439449
11717820_SLC4A4	-0.07301	0.328037	0.439478
11747211_PKD1L1	-0.05105	0.328107	0.439547
11717881_ATXN7L3	-0.04952	0.328111	0.439547
11747237_MCMDC2	0.119304	0.328116	0.439547
11754238_DYDC2	-0.05594	0.328206	0.439656
11760692_TRDMT1	0.075579	0.328328	0.439808
11720388_C1QC	-0.05475	0.328362	0.43984
11762162_LMNA	-0.05273	0.328473	0.439978
11736988_NT5C1B	-0.05632	0.328482	0.439978
11735617_SLC5A10	-0.04063	0.328506	0.439997
11755930_ZC3H7B	0.042714	0.328567	0.440068
11755034_REXO4	-0.05626	0.328641	0.440154
11729154_CEACAM6	-0.06399	0.328683	0.440198
11738690_OR52I1 //	-0.041	0.32876	0.44029
11725911_ERBB4	-0.10152	0.32877	0.44029
11764130_IGH /// I	0.085602	0.328841	0.440374
11738288_IFNW1	0.076451	0.328869	0.440399
11761388_COQ2	-0.1024	0.328914	0.440447
11734032_C15orf61	0.091678	0.328941	0.440472
11734105_PNMAL2	-0.11641	0.328951	0.440473
11719834_MPZL2	0.043591	0.329118	0.440681
11732522_CHST9	-0.08218	0.329124	0.440681
11723387_PTAR1	0.046285	0.329185	0.44075
11761755_DPPA4	0.058231	0.329208	0.440765
11717582_RGCC	0.12323	0.329214	0.440765
11731347_PRR32	-0.06746	0.32925	0.440801
11758194_DPP4	-0.05853	0.32934	0.44091
11744180_TCIRG1	0.049518	0.329409	0.44099
11760147_ISCU	0.055165	0.329448	0.44103
11760885_RXRB	0.076725	0.329514	0.441105
11723661_UBIAD1	0.051143	0.32953	0.441115
11759845_C17orf99	-0.05842	0.329582	0.441164
11750866_CORIN	-0.08874	0.329584	0.441164
11755842_RBBP6	-0.0494	0.329597	0.441166
11763380_FAAP20	0.054305	0.329604	0.441166
11740593_HOXD4	-0.04152	0.329696	0.441266

11756335_CARD19	0.04436	0.329702	0.441266
11744157_CHKB ///	-0.0434	0.329706	0.441266
11752752_ZNF793	0.074886	0.329715	0.441267
11752582_KIR3DL1	-0.05432	0.329775	0.441334
11724423_GPR68	0.058397	0.329837	0.441406
11751603_SRSF7	0.034293	0.329859	0.441422
11722140_GUCY1A3	0.130247	0.329873	0.441429
11730396_DDI1	-0.09013	0.329938	0.441505
11749049_TFAP2A	0.126251	0.329979	0.441547
11738024_CRACR2A	0.082196	0.329988	0.441547
11752834_BTNL8	0.061691	0.330043	0.441609
11749680_CASS4	-0.09361	0.330067	0.441628
11762141_LOC105372	0.046164	0.330077	0.44163
11727824_TPMT	0.05145	0.330089	0.441634
11755480_MYOCD	-0.0939	0.3301	0.441637
11742277_ASIC5	0.059271	0.330133	0.441669
11743219_GRID2	-0.06879	0.330156	0.441687
11745291_AFF2	-0.16643	0.330208	0.441744
11746075_ZNF511	-0.05397	0.330257	0.441798
11734914_UBQLNL	-0.05321	0.330336	0.441891
11738079_NR2E3	-0.07285	0.33035	0.441899
11761283_SMYD3	0.107548	0.330372	0.441916
11716051_LAMC1	0.036001	0.330418	0.441959
11759497_CHST11	0.058655	0.330422	0.441959
11734107_LSM11	-0.08574	0.330433	0.441961
11723933_PNMA3	0.043643	0.330461	0.441986
11715575_PPM1G	-0.03794	0.330475	0.441992
11757163_SNORA54	-0.07466	0.330484	0.441992
11736894_STAG3L1 /	0.040777	0.330501	0.442003
11753981_VEPH1	0.136548	0.330546	0.442051
11738337_SHISA6	-0.04352	0.33059	0.442093
11761561_REEP6	-0.09725	0.330595	0.442093
11756945_PIK3C2A	0.086602	0.330612	0.442104
11732521_CHST9	-0.05505	0.330638	0.442127
11738163_OR8A1	0.079937	0.330664	0.442149
11746569_SLC35C2	0.036269	0.3307	0.442185
11719670_RCAN2	-0.07569	0.330856	0.442372
11763660_FMNL1	-0.06671	0.330858	0.442372
11735253_NEK8	-0.0702	0.330898	0.442414
11747218_LOC442028	-0.12167	0.330917	0.442427
11756842_TMEM217	0.080639	0.330961	0.442474
11762565_ANKRD20A5	-0.05617	0.331033	0.442557
11735828_VSX1	-0.05917	0.33105	0.442568
11753769_SBNO2	0.070792	0.33108	0.442596
11728644_MLC1	-0.09727	0.331089	0.442597
11735314_TMPRSS12	-0.06429	0.331115	0.442613
11717907_FAM136A	0.03812	0.33112	0.442613
11741882_ARTN	-0.04741	0.331186	0.442678
11752891_TYR	-0.08353	0.331187	0.442678
11724855_RND2	0.047461	0.331215	0.442704
11728855_SCRT1	-0.07046	0.331228	0.442709

11715356_COL1A2	0.04635	0.331264	0.442746
11755005_ERBB2	-0.04259	0.331274	0.442747
11715313_LHB	-0.04858	0.331298	0.442766
11734130_SLC30A4	-0.06836	0.33131	0.442771
11753408_FUT6	0.086228	0.33134	0.442798
11734167_NT5C1B	0.071461	0.331362	0.442816
11754627_RAB37	0.076052	0.331435	0.442901
11729240_SLC35E3	-0.03404	0.331458	0.442911
11761617_SMS	-0.08252	0.331461	0.442911
11763253_IGKV2-28	0.059182	0.331518	0.442966
11740727_SYCE1	-0.04218	0.33152	0.442966
11747378_ATP6AP2	0.05156	0.331613	0.443078
11742872_PIK3IP1	-0.03965	0.331624	0.443081
11742212_VEGFA	-0.04735	0.331663	0.443121
11752513_DACH2	-0.06355	0.331754	0.44323
11750345_IKBKB	-0.07576	0.331763	0.44323
11744785_SCRIB	0.036745	0.331774	0.443233
11742536_OR5D16	-0.0676	0.331819	0.443281
11763912_KANSL2 //	0.085567	0.331857	0.44331
11734065_AMPD3	0.083057	0.331859	0.44331
11741796_SH3BP2	-0.03435	0.331869	0.443311
11715380_LAPTM4B	-0.03404	0.331887	0.443323
11743137_NID1	0.038314	0.331989	0.44344
11763625_ZNF384	0.039564	0.331992	0.44344
11762656_TXNDC11	-0.06498	0.33201	0.443451
11735333_EXD3	-0.07182	0.332067	0.443515
11740615_KANSL1	-0.04163	0.332162	0.443619
11732567_MBP	0.100424	0.332163	0.443619
11761175_SLC17A9	-0.06653	0.332196	0.443652
11742110_FBLN7	-0.07051	0.332224	0.443676
11734602_TAS2R43 /	0.130781	0.332252	0.443702
11763557_TRBC1 ///	-0.06017	0.332275	0.44372
11723031_CRISPLD2	-0.0851	0.33231	0.443754
11737666_HHLA1	0.093765	0.332331	0.443771
11760426_EPS15L1	-0.04839	0.332376	0.443819
11745832_NFKBIB	0.095497	0.332418	0.443862
11750461_NUBPL	-0.05016	0.332459	0.443905
11734730_MRPS12	-0.05008	0.33258	0.444054
11738376_GNAT3	0.058116	0.332589	0.444054
11737311_PAGE3	0.076253	0.332656	0.444133
11750687_ADAM28	-0.0624	0.332684	0.444158
11730702_LOC729966	-0.07767	0.332695	0.44416
11724909_TMPRSS2	-0.08456	0.332749	0.444216
11753015_NR2C1	0.047001	0.332755	0.444216
11742304_SSX5	-0.05602	0.332814	0.444282
11743309_ARMC7	0.067703	0.332838	0.444291
11749030_POLL	-0.06269	0.332839	0.444291
11716315_MRPL15	-0.0366	0.332876	0.444323
11760404_DYNLRB1	-0.05402	0.332881	0.444323
11718426_HSDL2	-0.03884	0.33293	0.444377
11753609_GPER1	-0.06584	0.33297	0.444417

11718380_DGKZ	-0.05188	0.333024	0.444478
11760695_CLDN15	0.071407	0.333081	0.444542
11734897_MATN1	0.057704	0.333105	0.444557
11747583_PNCK	-0.04458	0.333111	0.444557
11734508_FOXI2	-0.04	0.333171	0.444625
11751535_CA3	0.077663	0.333263	0.444736
11721046_SERINC2	-0.03823	0.333334	0.444808
11721050_TUBGCP6	0.034844	0.333342	0.444808
11752785_ZBTB7C	-0.06587	0.333345	0.444808
11721763_ADRBK2	-0.08264	0.333386	0.444851
11763985_LOC101928	-0.15464	0.333423	0.444889
11738687_OR5T1	0.068558	0.333453	0.444916
11728305_GULP1	-0.10156	0.333564	0.445053
11729753_ULK2	-0.07438	0.333605	0.445096
11741619_SCN7A	-0.03813	0.333699	0.4452
11750983_VDR	-0.04823	0.333702	0.4452
11729776_TMTC2	0.047048	0.333723	0.445216
11733665_NRP2	-0.06892	0.333736	0.445222
11717696_TYSND1	-0.06857	0.333781	0.44527
11729159_UCP2	-0.05979	0.333792	0.445272
11734301_MBL2	0.125136	0.333818	0.445295
11748198_NSG1	-0.04054	0.333864	0.445343
11724528_GCNT3	0.090978	0.333943	0.445437
11723545_PLD1	0.046144	0.334049	0.445566
11719674_REG3A	-0.05043	0.334074	0.445582
11725887_FAM167B	-0.04852	0.334103	0.445582
11715355_COL1A2	-0.08875	0.334107	0.445582
11761855_DPF1	-0.04058	0.334111	0.445582
11740634_FOXP2	-0.07576	0.334112	0.445582
11748872_PIANP	-0.0459	0.334116	0.445582
11751551_KRTAP5-5	0.046379	0.334173	0.445645
11715284_C15orf40	-0.03938	0.334224	0.445702
11734043_KIAA0408	-0.07873	0.334279	0.445759
11737709_ORC5	0.062412	0.334288	0.445759
11758191_MEX3C	-0.03591	0.334294	0.445759
11763778_MCM3AP-AS	-0.10228	0.33431	0.445768
11762394_AR	-0.06294	0.334401	0.445876
11726921_ARSG	0.070167	0.334446	0.445925
11747257_GRWD1	0.039554	0.33456	0.446065
11753562_LUZP2	-0.03525	0.334609	0.446111
11752895_SLC22A17	-0.05501	0.334613	0.446111
11736905_ROR2	-0.04978	0.33468	0.446189
11721784_SLC9A8	0.058269	0.334711	0.446217
11752941_PRKCG	-0.09008	0.334757	0.446267
11751023_ALG6	-0.10387	0.334773	0.446276
11748763_EEF2	-0.038	0.335011	0.446581
11752571_ARHGEF7	-0.06499	0.335066	0.446642
11752253_PLEKHN1	-0.03363	0.335095	0.446669
11737689_SPDYE2 //	0.067052	0.33512	0.44669
11731134_LTB4R	0.065957	0.335182	0.44676
11762222_GOLGA6L7F	0.061621	0.335327	0.446941

11727913_FMNL1	-0.03472	0.335396	0.44702
11739353_CHDH	-0.09935	0.335424	0.447046
11725281_PRDM8	-0.11197	0.335508	0.447145
11751860_HYAL1	-0.1	0.335552	0.447192
11750942_JAKMIP1	-0.12203	0.335561	0.447192
11738549_OTUD6A	-0.03597	0.33558	0.447203
11756466_PLEKHB1	-0.04571	0.335587	0.447203
11748619_SUCLA2	0.038336	0.335672	0.447304
11735154_COL4A3	0.093992	0.335729	0.447367
11755006_BCAR3	0.056824	0.335926	0.447618
11735508_AKR1D1	0.111251	0.335939	0.447622
11736697_ERN2	-0.06419	0.336136	0.447873
11761064_SPDYE2B	-0.03962	0.336148	0.447877
11725961_TCHH	-0.04861	0.336182	0.44791
11716247_TOP2B	0.034506	0.336221	0.447949
11749878_CNOT1	0.067191	0.336239	0.447961
11754337_NDRG1	0.041033	0.336301	0.448031
11756150_B2M	0.038198	0.33648	0.448258
11727423_CANX	0.038178	0.336546	0.448333
11740561_HTR1F	0.046353	0.336651	0.448461
11716897_PPIE	-0.04639	0.336669	0.448473
11722719_BAIAP2	0.049815	0.336693	0.448493
11715782_EEF2	0.039183	0.336704	0.448494
11740504_ZNF680	0.075517	0.336712	0.448494
11753443_SSTR1	-0.06337	0.336801	0.4486
11752521_F11	-0.10233	0.336843	0.448644
11756457_ARMC7	-0.05753	0.336931	0.448748
11734476_SPATA9	0.056056	0.336953	0.448765
11762453_CSNK1A1	-0.09059	0.336981	0.448791
11741870_PARK2	-0.0843	0.336994	0.448795
11751552_RNF223	-0.06977	0.337078	0.448895
11746821_ETNPPL	-0.06777	0.337163	0.448997
11727822_TPMT	0.044392	0.337194	0.449025
11755198_SNAI3	-0.05771	0.337254	0.449093
11752116_FGFR2	-0.0589	0.337266	0.449097
11739457_SYNC	0.052685	0.337329	0.449169
11731322_C22orf31	-0.06446	0.337343	0.449174
11733003_CXCR1	-0.08306	0.337358	0.449183
11736088_FAM171A1	0.04917	0.337405	0.449233
11721127_ENPEP	-0.06472	0.337462	0.449296
11721746_ANKRD12	-0.10114	0.337498	0.449323
11729225_SLC33A1	0.055312	0.3375	0.449323
11761008_TASP1	0.095685	0.337598	0.449442
11740167_HUNK	0.06241	0.337678	0.449535
11741621_LRIT1	-0.06957	0.337708	0.449563
11730694_GJB7	-0.07009	0.337766	0.449628
11733668_WDR78	0.077568	0.337778	0.449631
11742269_CLEC4M	-0.10099	0.337791	0.449633
11753041_GYS1	-0.05835	0.337798	0.449633
11716408_MET	0.037953	0.337828	0.449661
11717138_CDC42EP4	-0.06112	0.337858	0.449689

11743763_GTF3C3	0.036693	0.337888	0.449717
11740667_TPRX1	-0.04731	0.337952	0.44979
11742472_SLC9B1	0.055607	0.338021	0.44987
11762753_LETM2	0.082289	0.338032	0.449872
11763797_EPHB1	0.086245	0.338056	0.449892
11758166_ANOS1	-0.0897	0.338079	0.44991
11747071_UGT2B4	0.091463	0.338117	0.449948
11742360_OR5A2	-0.04645	0.338197	0.450042
11716810_SIN3B	0.042858	0.338235	0.45008
11762802_PRRT3	-0.06622	0.338295	0.450148
11748809_ARID3B	-0.03539	0.338328	0.450179
11729243_FBN2	0.093035	0.338358	0.450208
11730642_CT55	-0.08956	0.338443	0.450308
11757258_PRG1	-0.08616	0.338518	0.450396
11734653_ATP5F1	-0.03496	0.338533	0.450404
11732405_NOL9	0.053992	0.338655	0.450554
11763156_SHBG	-0.06724	0.338685	0.450563
11759642_HLA-DRB1	0.038403	0.338688	0.450563
11720580_TCF12	0.049552	0.33869	0.450563
11762479_EGFR	-0.08591	0.338854	0.450769
11762763_DUOXA2	-0.04631	0.33891	0.450832
11748305_TTC41P	0.055947	0.338946	0.45086
11718206_TOE1	-0.0409	0.33895	0.45086
11761215_BEST1	-0.04772	0.339001	0.450916
11739098_ARFIP2	-0.06696	0.339012	0.450919
11745332_KCNT2	-0.10088	0.339026	0.450925
11751422_CHDH	-0.08468	0.339038	0.450929
11727322_EMILIN3	-0.06943	0.339062	0.450947
11745128_CDH5	-0.06526	0.339103	0.450989
11746135_NOP56	0.036207	0.339168	0.451065
11723316_DUSP16	0.061971	0.339278	0.451199
11718671_C11orf49	-0.05311	0.339326	0.45125
11755391_H2AFY	-0.03821	0.33944	0.451377
11717453_SPOCK2	-0.09118	0.33944	0.451377
11738271_KCNIP2	0.045097	0.339498	0.451441
11756024_FAM19A3	-0.12068	0.339547	0.451484
11745656_PAX5	-0.0503	0.339549	0.451484
11743967_CERCAM	0.047314	0.339607	0.45155
11745528_GOLGA3	-0.08136	0.339642	0.451584
11725144_ZNF784	-0.05551	0.339672	0.451606
11734801_WIPI2	0.045104	0.339677	0.451606
11752654_GNPTAB	-0.05153	0.339715	0.451645
11752447_TBR1	-0.09816	0.339855	0.451818
11738838_IL5RA	-0.0466	0.339912	0.451873
11747028_HMCN2	-0.08022	0.339914	0.451873
11757048_EIF3B	0.045453	0.339948	0.451903
11756078_ARHGEF37	0.065007	0.339956	0.451903
11747563_PLOD3	-0.04497	0.340038	0.451999
11755716_RNF157	0.056176	0.340115	0.45209
11761760_LMF1	-0.04284	0.340163	0.452142
11739498_NPTX1	-0.05431	0.340196	0.452173

11736486_RASGEF1B	-0.07934	0.340226	0.452201
11750665_CHRNA2	-0.07166	0.340306	0.452294
11720166_CDC42EP5	-0.03616	0.340334	0.45232
11742089_KRT76	-0.06181	0.340355	0.45233
11752943_CATSPER2	-0.07605	0.34036	0.45233
11729796_SP2	0.040073	0.34043	0.45241
11751890_SCN5A	-0.04065	0.34044	0.452411
11740570_ZNF665	-0.06338	0.340454	0.452418
11731475_SFRP4	-0.08416	0.340468	0.452423
11738415_TBR1	-0.09001	0.340495	0.452448
11726171_SCARA3	-0.10246	0.340528	0.452479
11751497_MFSD13A	-0.03762	0.340614	0.452581
11735555_AMZ1	-0.04411	0.340656	0.452624
11748326_GORASP1	-0.03874	0.340691	0.452658
11764092_ACO1	-0.14465	0.340711	0.452672
11730674_MAP3K15	-0.08741	0.340736	0.452684
11751960_PAPLN	0.078386	0.340738	0.452684
11758890_FAM46A	0.079304	0.340795	0.452732
11733518_KCNK10	-0.08283	0.340799	0.452732
11728055_GHR	0.076772	0.340802	0.452732
11740577_VWA5B1	-0.1348	0.340933	0.452894
11757450_MSL1	-0.04476	0.340985	0.45295
11730698_FOXR1	-0.0731	0.340998	0.452955
11758150_ARSE	0.058775	0.341006	0.452955
11763460_GPRIN3	0.070511	0.341166	0.453154
11740566_ATXN3L	0.102139	0.341188	0.45317
11716632_FXR2	-0.06638	0.341244	0.453233
11745240_TIE1	-0.05962	0.34131	0.453308
11750761_PLXNB3	-0.03893	0.341335	0.453329
11736802_FGF9	-0.0535	0.341404	0.453409
11762769_ZNF215	-0.09697	0.341437	0.45344
11720024_FAM118A	0.077102	0.341475	0.453478
11717613_KLHDC8B	-0.05167	0.341528	0.453537
11745429_MYBBP1A	0.034839	0.341743	0.453801
11759776_GTDC1	0.060133	0.341746	0.453801
11721334_PLOD2	0.042648	0.341765	0.453814
11733813_GNAO1	-0.05615	0.341788	0.453832
11722309_TRPM4	0.036734	0.341804	0.453841
11718391_PREPL	-0.03732	0.341888	0.453941
11750832_SLC12A1	-0.06011	0.34191	0.453957
11760506_NCAM1-AS1	-0.08456	0.342047	0.454127
11726098_NKAIN1	0.065536	0.34208	0.454158
11747962_RFNG	0.055239	0.342211	0.45432
11720990_MED8	0.041723	0.34232	0.454452
11754756_FGD5	-0.07047	0.342331	0.454454
11739340_FAM46C	0.037362	0.342347	0.454464
11760419_JAM2	-0.0694	0.34237	0.454481
11729134_STK24	0.039292	0.342414	0.454528
11724316_LUZP1	-0.04307	0.342451	0.454558
11721981_SLC36A1	-0.10393	0.342456	0.454558
11732642_PGLYRP1	-0.08009	0.342466	0.454559

11746698_GNAS	-0.03306	0.34258	0.454698
11756755_CSH1 ///	0.070523	0.342598	0.45471
11724119_SAMD9L	0.035452	0.342631	0.454721
11763809_SFTPC	-0.05511	0.342652	0.454721
11732969_MYBPC1	0.046508	0.342655	0.454721
11755168_CENPL	-0.03529	0.34266	0.454721
11740960_DMD	0.135194	0.342663	0.454721
11730625_KLB	-0.04147	0.34267	0.454721
11739356_ERG	-0.07677	0.342671	0.454721
11743965_CERCAM	-0.03773	0.342761	0.454828
11744953_ANXA1	0.035726	0.342811	0.454881
11758471_SPATA2	-0.04229	0.342872	0.45495
11760686_OBSL1	-0.06043	0.342904	0.454976
11739816_DLG4	0.097809	0.342917	0.454976
11727802_GJA9-MYCB	0.038769	0.342919	0.454976
11756824_SLC8B1	-0.05559	0.342967	0.455027
11731196_PRR5L	0.051203	0.343033	0.455102
11758705_PTPDC1	0.050548	0.343056	0.45512
11731886_SCGB1D2	-0.1329	0.343086	0.455147
11761039_TMX1	0.057621	0.343109	0.455167
11762902_C7orf55	0.080683	0.343202	0.455277
11741746_LIN54	-0.07126	0.343261	0.455341
11733716_EXOC4	0.039623	0.343269	0.455341
11728780_ZAP70	-0.06223	0.343282	0.455347
11756528_GNAO1	-0.05486	0.343399	0.455489
11759992_DLEU7	0.048486	0.34346	0.455557
11723484_PDCL3	-0.03679	0.34347	0.455559
11745052_TMEM132B	-0.04736	0.343605	0.455724
11715166_DNAJC12	0.081773	0.343613	0.455724
11732045_KRT85	-0.08537	0.343638	0.455743
11739032_ACTR2	-0.03322	0.343687	0.455796
11744382_WDR74	-0.04045	0.343697	0.455797
11716980_SLC35A4	-0.04596	0.343734	0.455823
11730309_RIC3	0.081738	0.343735	0.455823
11764109_LOC105378	-0.17891	0.343761	0.455834
11724407_CCNE1	-0.03693	0.343762	0.455834
11745063_LRRC8B	-0.04547	0.343855	0.455945
11721169_PYCARD	0.052221	0.343894	0.455985
11741594_LST1	0.052312	0.343993	0.456103
11734737_FAR1	-0.04825	0.344051	0.456168
11747187_PTGES	0.042668	0.344096	0.456215
11717846_PLEKHJ1	0.041031	0.34412	0.456234
11747413_KANSL1	-0.04488	0.34414	0.456248
11723536_SPATA8	-0.09035	0.344168	0.456273
11744037_CMIP	-0.03662	0.344186	0.456285
11742229_SUPT20HL1	0.066636	0.344219	0.456309
11737044_FAM184B	0.044973	0.344228	0.456309
11763632_SLC12A4	0.0372	0.344232	0.456309
11736074_KCTD3	0.035091	0.344318	0.45641
11746399_PIGT	-0.03737	0.344365	0.45646
11751758_GPR85	0.053792	0.3444	0.456495

11723803_TREM2	0.057221	0.344434	0.456528
11761830_HERC2P4 /	0.056081	0.344479	0.456575
11751006_ATL2	-0.04634	0.344527	0.456616
11738386_ATOH1	-0.05056	0.344529	0.456616
11749600_PRG2 ///	-0.03528	0.344557	0.45664
11724872_CLDN2	-0.06119	0.344574	0.45664
11759386_OR5T2	-0.1021	0.344575	0.45664
11762103_ZNF274	0.084508	0.344587	0.456643
11722648_TNNI3	-0.06824	0.34461	0.456661
11743959_SLC16A3	-0.06153	0.344695	0.456761
11752325_NTRK3	-0.04719	0.344713	0.456771
11756413_ASTN2	0.081857	0.344721	0.456771
11758786_ARIH1	-0.10499	0.344794	0.45685
11760937_CYP19A1	0.111862	0.344799	0.45685
11747846_KHDRBS2	0.117161	0.344849	0.456903
11742531_OR6B3	0.064912	0.344858	0.456904
11715281_ASIP	0.100937	0.344923	0.456977
11718545_SGSM2	-0.04252	0.344988	0.457051
11734770_NEGR1	-0.11096	0.345043	0.457111
11723318_FAM234B	-0.07876	0.345061	0.457123
11725199_BTBD7	0.089291	0.345109	0.45717
11733886_FXR1	0.036302	0.345126	0.45717
11752538_GABRG1	-0.08225	0.345127	0.45717
11740827_HES3	-0.06871	0.345138	0.45717
11739829_CYS1	0.072932	0.345144	0.45717
11726054_MUC4	0.098046	0.345166	0.457172
11759410_KCNV2	0.088555	0.34517	0.457172
11763490_STK35	0.058711	0.345173	0.457172
11763841_LOC101930	-0.0952	0.345251	0.457262
11715510_NDFIP1	-0.03741	0.345276	0.457277
11737304_BRS3	-0.09066	0.34528	0.457277
11737837_NSMF	-0.03438	0.345356	0.457365
11754401_SHE	-0.05875	0.345448	0.457474
11745772_TNFRSF14	0.052379	0.345555	0.457603
11753814_IL2RG	0.064144	0.345586	0.457632
11732762_GRIN3A	-0.15018	0.345726	0.457805
11740129_CACNA1B	-0.08806	0.345802	0.457893
11755758_NLRC5	0.036249	0.345864	0.457963
11739720_GIMAP8	-0.1359	0.345873	0.457963
11751964_SRCIN1	-0.0639	0.345889	0.457972
11722646_MYL1	-0.07421	0.345928	0.45801
11760549_PPP1CB	-0.0405	0.346016	0.458114
11748268_ZNF282	0.056952	0.34609	0.4582
11731867_BICD2	-0.04172	0.346108	0.458205
11742228_SUPT20HL1	-0.06077	0.346112	0.458205
11734773_SFTPB	-0.04657	0.346228	0.458345
11740601_APH1B	-0.04439	0.346252	0.458365
11748412_FUT10	0.054358	0.34635	0.458478
11752441_GPR35	-0.07534	0.346356	0.458478
11752839_ZNF185	-0.06831	0.346385	0.458499
11728599_PHOSPHO2	-0.06179	0.346397	0.458499

11742629_OR4D2	-0.07126	0.3464	0.458499
11727779_GHITM	0.081873	0.34643	0.458526
11720279_FAM174B	-0.05071	0.346513	0.458623
11756483_CSHL1	-0.05592	0.346538	0.45863
11725613_PITRM1	0.038132	0.346542	0.45863
11737686_C2orf48	-0.05475	0.346546	0.45863
11745994_MAP7	-0.12839	0.34658	0.458663
11748292_GBA /// G	0.036543	0.346601	0.458678
11751331_DEPTOR	-0.06733	0.346614	0.458683
11726161_EPHA7	-0.04828	0.346628	0.458688
11732243_APOL6	-0.07834	0.346654	0.458711
11717235_RPS7 ///	-0.03353	0.346679	0.458731
11748279_TM7SF2	0.04233	0.346718	0.45877
11747539_MYD88	0.053505	0.346729	0.458772
11735331_RUNDC3B	0.062407	0.346799	0.458849
11733167_LRRN4CL	-0.04198	0.346805	0.458849
11745883_SEPT9	-0.04339	0.346877	0.458931
11751992_MOV10L1	-0.07033	0.34696	0.45902
11725609_DTNA	-0.05308	0.346963	0.45902
11742498_FAM86B1 /	0.094398	0.347014	0.459075
11729797_SP2	-0.04353	0.347038	0.459083
11740833_DST	0.06483	0.347039	0.459083
11760002_RNF212	0.047832	0.347057	0.459095
11763412_EPHA10	-0.0882	0.347122	0.459169
11761555_CFB	-0.09725	0.3472	0.459259
11740394_FGFR2	-0.05276	0.347216	0.459264
11723717_FTSJ2	0.037128	0.347223	0.459264
11759743_ZNF75A	0.057881	0.347302	0.459357
11738910_OR51B2	-0.08069	0.347408	0.459485
11751833_BCHE	-0.05044	0.347435	0.459508
11744948_SEMA3F	-0.06342	0.34747	0.459542
11746721_TREM1	0.057918	0.347545	0.459629
11738428_POU1F1	-0.06203	0.347573	0.459653
11723964_AGA	-0.04314	0.347613	0.459681
11762147_HMGA2	-0.0604	0.347618	0.459681
11750004_MAMLD1	-0.11409	0.347622	0.459681
11719187_MYOZ1	-0.05092	0.34764	0.459692
11725241_HOXD9	-0.06284	0.347654	0.459698
11729008_MYLK4	-0.077	0.34768	0.459715
11744481_OPN3	0.03764	0.347692	0.459715
11757071_STEAP1 //	-0.03686	0.347695	0.459715
11741102_PTPN22	0.086704	0.347783	0.459807
11731973_SCNN1G	0.080868	0.347783	0.459807
11728413_SERPINB8	0.057528	0.347794	0.459809
11723848_ZNF18	-0.06318	0.347812	0.459819
11724211_LMAN1	-0.03644	0.347861	0.459861
11763121_LOC100132	-0.05687	0.347867	0.459861
11725485_DIRC2	-0.11612	0.347871	0.459861
11756970_CHIT1	-0.06374	0.347909	0.459898
11748351_THBS3	0.048789	0.347951	0.459942
11741251_SIRPB2	-0.08469	0.348003	0.459997

11762575_LOC643733	-0.11674	0.348015	0.460001
11741217_EDARADD	-0.14038	0.348087	0.460083
11762263_FGD2	0.06097	0.348112	0.460104
11736210_MRPL57	0.035202	0.348139	0.460127
11733711_COL25A1	0.113886	0.348153	0.460134
11762764_DUOXA2	-0.04301	0.348186	0.460165
11751862_KDM1B	-0.10812	0.348196	0.460166
11737465_IL11RA	0.043228	0.348244	0.460217
11722972_FOXK1	-0.06339	0.348277	0.460247
11742214_DAZ1 ///	0.068561	0.348362	0.460348
11743402_CDC27	-0.05365	0.348431	0.460426
11717758_RALA	0.042362	0.34845	0.460431
11726004_LAYN	-0.06056	0.348453	0.460431
11723537_HMHA1	-0.11605	0.348495	0.460473
11738413_PUS10	0.072465	0.34858	0.460574
11724056_CAMTA2	-0.05563	0.348623	0.46061
11739832_RIN3	0.066487	0.348626	0.46061
11742104_CASP10	-0.03633	0.34879	0.460814
11720566_BCAT1	-0.03506	0.348819	0.46083
11757728_FTL	0.033008	0.348822	0.46083
11735517_TRIM60	0.109193	0.348833	0.460833
11718889_PIP5K1A	0.035299	0.348853	0.460847
11763877_LOC100128	-0.06117	0.348903	0.460901
11761081_PEF1	-0.06699	0.349076	0.461116
11716416_C1QA	-0.05176	0.349134	0.461181
11730784_ORC4	0.037017	0.349166	0.46121
11751863_PLAG1	-0.07556	0.349184	0.46121
11726410_GID4	-0.05075	0.349193	0.46121
11751499_ZNF846	0.142942	0.349194	0.46121
11762886_OR13H1	0.088928	0.349204	0.461211
11725150_HR	-0.05031	0.349233	0.46123
11753863_BCL2L11	-0.05109	0.349237	0.46123
11754260_NBR2	-0.04116	0.349276	0.461268
11726547_PITPNM3	-0.07071	0.349303	0.461292
11758978_ERGIC2	-0.06755	0.349318	0.4613
11731376_KRT27	0.054965	0.349397	0.461392
11761486_PSMD8	-0.05765	0.349455	0.461446
11715188_H3F3C	0.036127	0.349458	0.461446
11730009_MTUS2	0.053027	0.349528	0.461525
11748308_SGSM2	-0.05256	0.349543	0.461525
11752448_P4HB	-0.04411	0.349546	0.461525
11749979_GTF2H2 //	0.038157	0.349598	0.461582
11741151_ZNF154	0.08503	0.349687	0.461675
11757062_CDC26	-0.04168	0.349688	0.461675
11762107_CAST	0.115407	0.349727	0.461714
11739447_PAK7	-0.12434	0.349768	0.461756
11721748_INF2	-0.041	0.349875	0.461885
11736944_C2orf15	-0.09394	0.349885	0.461886
11715788_SLC40A1	-0.07131	0.349954	0.461964
11731197_PRR5L	0.097858	0.349992	0.462002
11728236_CD5	-0.10702	0.350022	0.462029

11731925_CRCT1	-0.07656	0.350041	0.462041
11748597_BCLAF1	0.044663	0.350197	0.462235
11763605_WNT11	-0.10738	0.350247	0.462289
11732379_DXO	0.03996	0.350315	0.462366
11730792_SVIP	0.048373	0.350408	0.462476
11758828_TOMM40L	0.129931	0.350435	0.462499
11729942_TMEM56	0.063344	0.350487	0.462556
11716617_MRPL36	-0.0362	0.350504	0.462556
11763921_NCL /// S	-0.04889	0.350506	0.462556
11747807_RNGTT	0.078155	0.350518	0.462559
11759927_ADAM22	0.106677	0.350589	0.462639
11735278_LRRTM4	-0.05514	0.350656	0.462717
11756847_CX3CR1	0.062345	0.35073	0.462801
11744753_LRRC28	0.071211	0.350793	0.462872
11734288_RBPMS	0.038988	0.350866	0.462956
11723610_HABP2	-0.08042	0.350913	0.463005
11724849_DIXDC1	0.079545	0.350962	0.463058
11759445_SNAPC5	-0.04834	0.351017	0.463098
11759470_LCE1F	-0.05269	0.351017	0.463098
11737680_GPR149	-0.07593	0.351021	0.463098
11750947_P2RX6	-0.04094	0.351032	0.4631
11750938_HIPK4	-0.03792	0.351146	0.463238
11759461_ZNF836	-0.06055	0.351169	0.463256
11719266_MLPH	0.107507	0.3512	0.463284
11720973_ASB8	-0.0555	0.351315	0.463423
11744857_MOV10L1	-0.04863	0.351382	0.463499
11734497_C6orf222	-0.04275	0.351406	0.463518
11742314_SLC35G6	-0.0858	0.35146	0.463575
11727925_PLD4	-0.0754	0.351469	0.463575
11715280_KRT17	0.068786	0.351534	0.46364
11756485_TXLNA	-0.03787	0.351537	0.46364
11761806_NDUFA3	0.048995	0.351608	0.463722
11744139_TPGS1	0.043362	0.351662	0.463781
11744608_PARP3	0.047304	0.35169	0.463793
11734766_THOC2	-0.041	0.351691	0.463793
11751650_GIGYF2	-0.05526	0.351704	0.463793
11723398_NGFR	-0.03615	0.35171	0.463793
11741543_ZNF577	-0.06666	0.351808	0.463911
11741891_EVX2	-0.05988	0.351918	0.464042
11736285_CHKA	0.045882	0.351947	0.464068
11737985_TENM1	0.118604	0.352017	0.46414
11747128_PHTF2	-0.04541	0.35202	0.46414
11738760_OR2M7	-0.05754	0.352049	0.464166
11739345_CLGN	-0.06726	0.352148	0.464279
11742955_KRT6A	-0.0907	0.352154	0.464279
11725988_MMP14	-0.04574	0.352204	0.464323
11729234_E2F6	0.051448	0.352206	0.464323
11738671_SZT2	-0.0538	0.352261	0.464374
11753065_LOC101928	-0.04114	0.352267	0.464374
11745080_RASGEF1B	0.07655	0.352273	0.464374
11724954_MRC1	0.062415	0.352308	0.464406

11727312_BHLHE22	-0.09503	0.352327	0.464419
11724145_SLC37A1	0.061056	0.352375	0.464471
11743848_SLC2A5	-0.07534	0.35239	0.464478
11757712_L3MBTL4	0.102264	0.352433	0.464521
11747991_TMEM106A	-0.04019	0.3526	0.464719
11746279_TAX1BP1	-0.03464	0.352602	0.464719
11730243_FMO2	-0.06589	0.352652	0.464772
11762231_BBS1	0.04469	0.35269	0.46481
11731233_NTF4	-0.06416	0.352782	0.464898
11735829_CHAT	0.04187	0.352785	0.464898
11762675_FCGR1A	0.050941	0.352786	0.464898
11726456_MYO5B	0.065496	0.352795	0.464898
11752424_SREK1IP1	0.051325	0.352872	0.464977
11738831_HSD17B13	0.09117	0.352873	0.464977
11759755_DMKN	0.054003	0.352898	0.464997
11761156_AKR1C3	-0.15418	0.352937	0.465036
11760318_CYP4V2	0.037864	0.352947	0.465036
11750055_CEP76	0.068992	0.352959	0.465039
11733483_RNF113B	-0.06219	0.352987	0.465064
11720725_ZYG11B	-0.06502	0.353004	0.465074
11737409_BTF3	-0.04016	0.353041	0.465109
11760953_UBXN11	0.060896	0.353127	0.465207
11741063_LOC101928	0.041077	0.353134	0.465207
11756973_RAD18	-0.04446	0.353144	0.465208
11748237_GLTSCR1L	-0.04642	0.353211	0.465276
11751238_HINFP	0.042303	0.353215	0.465276
11737324_LAMC2	-0.06066	0.353226	0.465278
11761189_APBB1	0.04513	0.353359	0.46544
11750685_GALNT6	0.055533	0.353369	0.465441
11721086_YLPM1	-0.0941	0.353381	0.465445
11740106_RGS3	-0.07743	0.353505	0.465595
11730620_IP6K3	0.072951	0.353518	0.465595
11746550_C17orf99	-0.05392	0.353523	0.465595
11722258_FAM174A	0.04082	0.353579	0.465654
11754921_TMEM168	0.064972	0.353588	0.465654
11731592_STAM2	0.04438	0.353628	0.465695
11721033_FAM107A	-0.06219	0.353687	0.46576
11758008_PNPLA4	-0.07755	0.353745	0.465823
11720748_LTBP4	-0.05751	0.353773	0.465848
11717749_MCTS1	-0.03694	0.353794	0.465863
11754650_ATP10B	0.081331	0.353872	0.465954
11759102_UBXN10	0.048377	0.353962	0.466059
11762917_CCDC159	0.034711	0.353994	0.466089
11735530_PIH1D2	0.073968	0.354076	0.466184
11727572_KLHL6	-0.06099	0.35413	0.466234
11723237_POC5	-0.04124	0.354133	0.466234
11735791_PTPN18	-0.04567	0.354158	0.466255
11718586_SAP30BP	-0.03447	0.354228	0.466335
11756622_HSPA8	0.034222	0.354267	0.466373
11717776_KIF1C	0.043116	0.354292	0.466381
11748803_RERG	0.083146	0.354293	0.466381

11743029_SPIRE2	-0.05564	0.354366	0.466465
11742938_ASCL2	-0.04743	0.354378	0.466469
11742149_KRTAP10-1	-0.06713	0.35439	0.466472
11757319_HINT1	0.033048	0.354406	0.46648
11729090_FAM178B	0.063196	0.354427	0.466496
11744220_C9orf114	-0.05915	0.354553	0.466644
11743244_DBX1	-0.05402	0.354559	0.466644
11757016_ZNF727 //	-0.05206	0.354601	0.466687
11729919_ADAMTS18	-0.07206	0.354623	0.466704
11762794_DAGLB	-0.09368	0.354666	0.466745
11743128_CD82	0.049145	0.354674	0.466745
11753876_POLR2L	-0.03913	0.354689	0.466752
11753315_CELF2	0.057561	0.354717	0.466777
11716264_LEPROTL1	0.034631	0.354783	0.46685
11760260_ROGDI	0.071476	0.354798	0.466854
11720703_MYL4	-0.05847	0.354804	0.466854
11753128_MIF-AS1	-0.07729	0.354821	0.466863
11742722_LRRC58	-0.0422	0.354853	0.466893
11754669_ARG1	-0.08481	0.354896	0.466937
11748642_AGAP1	-0.10415	0.354947	0.466991
11741049_TMEM100	0.073043	0.354986	0.46703
11727718_ZBTB42	-0.05476	0.355011	0.467051
11759160_IFT81	0.055623	0.355089	0.46714
11748623_SLC7A8	-0.05764	0.355099	0.46714
11752645_P4HB	0.039491	0.355162	0.467211
11744798_OGT	-0.04603	0.355202	0.46724
11741258_AGBL1	-0.07127	0.355203	0.46724
11736975_DNMT3B	-0.05578	0.355285	0.467335
11761998_HDAC4	-0.04371	0.355317	0.467355
11748906_NPM2	-0.07131	0.355319	0.467355
11763671_A4GALT	-0.06465	0.355343	0.467373
11750250_PPP1R13L	-0.04947	0.355495	0.467561
11748849_BAK1	-0.05144	0.355537	0.467587
11732138_TIMM13	-0.04928	0.35556	0.467587
11756188_PSMD13	-0.03252	0.355561	0.467587
11726155_FAM19A5	0.037318	0.355562	0.467587
11760417_DUOXA1	0.137314	0.355562	0.467587
11760609_RAPGEF3	-0.08588	0.355575	0.467587
11730811_LRRTM2	0.093152	0.35559	0.467587
11752612_TSPAN15	-0.07085	0.355591	0.467587
11753444_HLA-G	-0.03759	0.355605	0.467593
11741702_ABHD11	-0.04305	0.355708	0.467715
11719250_PHLPP1	-0.03821	0.355779	0.467792
11758947_CPNE4	-0.06722	0.355785	0.467792
11732639_NOVA2	-0.06322	0.35582	0.467825
11733477_SUCNR1	-0.05956	0.355842	0.467841
11735859_TAS2R7	-0.07975	0.355886	0.467886
11761616_CLCA4	0.045491	0.356057	0.468099
11715256_XAGE3	-0.07999	0.356128	0.468169
11757248_RPSAP9	0.051933	0.35613	0.468169
11722427_FGL2	0.117542	0.356298	0.468378

11731356_IP6K3	0.045032	0.356328	0.468405
11762331_ACP2	-0.04745	0.356365	0.46844
11762615_TRIM63	-0.13109	0.356512	0.468622
11761503_KCNA2	-0.11014	0.356561	0.468673
11741925_LOC101928	0.075317	0.356593	0.468703
11748115_PKNOX2	-0.06839	0.356657	0.468774
11759482_WWTR1	-0.03519	0.356692	0.468807
11733472_CSMD2	0.053919	0.356708	0.468816
11753635_MED24	-0.03826	0.356727	0.468829
11723464_CELF2	-0.04699	0.356739	0.468831
11744611_PEG3	-0.05368	0.356757	0.468843
11729110_ADAMDEC1	-0.0714	0.356846	0.468947
11753948_FBXW8	0.045668	0.356872	0.468969
11740474_PTPRO	0.08075	0.356913	0.46901
11720820_FUNDC2	0.058488	0.356928	0.469017
11761034_APBB1IP	0.085454	0.356953	0.469021
11715925_TRAM1	-0.05577	0.356959	0.469021
11747874_BTN2A1	-0.05239	0.356961	0.469021
11719428_BTBD7	0.058529	0.35697	0.469021
11751749_CUX1	0.045408	0.357009	0.46906
11755610_PGPEP1L	-0.04429	0.357097	0.469163
11728551_TULP1	0.047342	0.357112	0.46917
11735475_HBQ1	-0.06899	0.357196	0.469268
11749365_SAP30BP	-0.0415	0.357229	0.469299
11742505_CEACAM18	-0.06963	0.357356	0.469452
11753610_VTCN1	-0.06629	0.357378	0.46947
11758354_RNPC3	-0.04193	0.357388	0.46947
11729114_PSMD7	-0.03389	0.357465	0.469558
11758791_C14orf1	0.129561	0.357502	0.469585
11736799_PDE10A	0.084848	0.357504	0.469585
11728552_MMP25	-0.06655	0.357563	0.469649
11734463_QRFP	-0.09131	0.357595	0.469678
11762768_ZNF215	-0.08241	0.357638	0.469719
11719915_AIF1L	0.064837	0.357645	0.469719
11749745_SRP68	0.039674	0.357686	0.469761
11762710_PARK2	0.076695	0.357705	0.469772
11750833_GTPBP2	0.058656	0.357736	0.4698
11727315_EFCAB1	-0.04762	0.357798	0.469859
11738421_OR4C6	-0.0543	0.3578	0.469859
11719797_ASMTL	0.040347	0.35781	0.469859
11759764_ODF3B	-0.05229	0.357889	0.469951
11716894_TOX4	-0.03758	0.357899	0.469952
11738185_PDE8B	-0.05657	0.357961	0.470021
11732954_CCDC153	-0.05696	0.357997	0.470055
11728782_CD1C	-0.07486	0.358029	0.470084
11759171_OFCC1	0.096801	0.358091	0.470154
11756143_ADPRH	-0.04178	0.358152	0.470198
11735541_CCDC122	0.080756	0.358153	0.470198
11732801_SIRPB1	-0.06226	0.358161	0.470198
11755554_LRIT3	0.084446	0.358164	0.470198
11730462_OTX2	-0.08833	0.358198	0.470231

11761947_KIR2DL1	-0.06693	0.358254	0.470282
11739114_TSPYL1	0.078967	0.358257	0.470282
11756817_FCER2	-0.04918	0.358284	0.470306
11754842_MEI1	-0.04423	0.35832	0.470341
11735997_APOL1	-0.03447	0.358368	0.47039
11741318_KCNJ1	-0.03575	0.35849	0.470537
11736297_SLC5A1	-0.0521	0.358517	0.47056
11754281_TECPR2	0.053582	0.35855	0.470592
11755153_MKL1	0.060317	0.358622	0.470664
11740299_HOXA1	0.127307	0.358625	0.470664
11743337_PLPP6	0.038694	0.358652	0.470687
11735970_DNAJB13	0.04838	0.358706	0.470746
11740113_NIPSNAP3E	0.047916	0.358753	0.470795
11752260_TMC3	-0.05688	0.358797	0.470839
11732637_ANXA13	0.045681	0.358905	0.470969
11756809_EFHC2	0.074336	0.358965	0.471035
11756938_TMEM199	-0.03845	0.359085	0.47118
11759305_ZMYM2	0.039818	0.359137	0.471236
11759268_DEFB134 /	-0.05618	0.359172	0.471268
11747316_TRIM46	0.038928	0.359236	0.47134
11750140_TOP1MT	0.058483	0.359265	0.471351
11735186_F9	0.099011	0.359273	0.471351
11727014_PAPPA	-0.08722	0.35928	0.471351
11759226_BCL2L14	-0.0918	0.359283	0.471351
11719262_SMTN	-0.03546	0.359316	0.471381
11761592_KIN	0.064593	0.359378	0.471451
11762979_MDM4	-0.0374	0.359431	0.471507
11750826_ADAMDEC1	-0.09304	0.359502	0.471587
11751681_DUS3L	-0.04029	0.359643	0.471753
11752169_ALDH1L2	-0.07595	0.359647	0.471753
11731383_DFFB	-0.09979	0.359658	0.471754
11738130_ZFYVE9	-0.05435	0.359754	0.471867
11764242_GTSCR1	-0.0981	0.359778	0.471886
11735207_MDGA2	-0.05712	0.359869	0.471993
11718227_BCS1L	-0.036	0.359882	0.471997
11761758_PSMB9	0.081279	0.359912	0.472024
11732738_CECR2	0.060131	0.359953	0.472065
11741264_LTBP1	-0.07092	0.360133	0.472288
11731128_SLC25A51	-0.0599	0.360157	0.472308
11753271_MAGEB1	-0.08151	0.36024	0.472404
11741692_CDKL1	0.051688	0.360265	0.472424
11727760_CTXN3	0.103301	0.360445	0.472638
11741785_SBK2	-0.09544	0.360447	0.472638
11733525_C2orf71	-0.07924	0.360516	0.472715
11733534_ARHGAP27	0.067863	0.360566	0.472768
11751703_MCF2L2	0.062049	0.360633	0.472843
11750810_PTPN5	-0.06115	0.360684	0.472896
11736798_RUNX1T1	-0.06384	0.360693	0.472896
11761549_TMED3	-0.0437	0.360716	0.472912
11722173_UQCC1	-0.06001	0.360724	0.472912
11735689_DDX41	0.048726	0.360791	0.472987

11740057_TMPRSS11A	-0.09666	0.360825	0.473019
11720386_MRC2	-0.06855	0.360928	0.473136
11752391_RNF17	-0.09627	0.360934	0.473136
11737009_ABCD2	-0.07813	0.361015	0.473229
11725991_RMDN1	-0.03593	0.361104	0.473323
11715504_LASP1	0.032544	0.361106	0.473323
11727232_ZNF19	0.066656	0.361132	0.473343
11751471_LOC729966	-0.04811	0.36114	0.473343
11735144_YBEY	0.052526	0.361174	0.473375
11725482_MATN3	-0.05861	0.361223	0.473426
11739491_MGAT4A	0.093849	0.361257	0.473458
11736920_ZBTB10	0.067575	0.36134	0.473554
11749963_FGA	0.082422	0.361539	0.473802
11724737_PDPN	-0.03931	0.361637	0.473918
11724637_TEX40	-0.06298	0.36176	0.474055
11727966_MSMB	0.080401	0.361766	0.474055
11719488_EDNRA	-0.08816	0.361771	0.474055
11739253_SLC35E1	0.036617	0.36179	0.474067
11747372_SYBU	0.039319	0.361808	0.474078
11721802_ARL8B	0.037889	0.361832	0.474097
11725053_TOP1MT	0.049645	0.361917	0.474187
11763425_ARRDC3-AS	-0.13147	0.36192	0.474187
11729471_FAM181B	-0.05461	0.361992	0.474264
11718525_NIPA2	0.036033	0.361998	0.474264
11730308_RIC3	-0.0376	0.36202	0.47428
11753838_SNN	-0.06535	0.362099	0.474369
11744179_TM7SF2	-0.06009	0.362108	0.474369
11746547_SLC2A5	-0.06837	0.362225	0.47451
11732636_HHIPL2	-0.04727	0.36224	0.474517
11730765_CD177	-0.08117	0.36225	0.474518
11735157_CYSLTR1	-0.07352	0.362285	0.47455
11735600_AKR7L	-0.12739	0.36235	0.474623
11758997_SYNPO	0.03645	0.362388	0.474653
11741794_HIST4H4	0.099654	0.362392	0.474653
11755362_IFT172	-0.0489	0.36246	0.474728
11717247_EIF1B	0.04408	0.362476	0.474737
11739555_FAM171B	-0.04358	0.362488	0.47474
11732826_ESX1	-0.07214	0.362627	0.474909
11738677_OR8G1 ///	-0.05671	0.362637	0.474909
11721011_SMOC1	-0.05777	0.362646	0.474909
11716379_TOMM20	0.03479	0.362659	0.474909
11733507_MAGEB4	-0.04612	0.36267	0.474909
11760455_PPP2R4	-0.05941	0.362675	0.474909
11724964_SAMD10	-0.0505	0.362731	0.47496
11748924_KMT2D	0.074872	0.362743	0.47496
11758827_INTS3 ///	-0.07405	0.362743	0.47496
11718603_MTURN	0.078786	0.36282	0.475048
11752528_RASGRP1	-0.04975	0.362833	0.475051
11740088_PIK3IP1	0.082965	0.362906	0.475123
11735230_SPATA12	0.072437	0.362914	0.475123
11723696_FLI1	0.061464	0.362917	0.475123

11753008_IL36G	-0.05874	0.362937	0.475137
11735869_TRIM64 //	-0.06149	0.362954	0.47514
11731901_SESN3	0.124	0.362958	0.47514
11733099_ITGB4	-0.03221	0.363037	0.47523
11740695_C7orf71	0.038641	0.363056	0.475233
11752178_SEMA4D	-0.08849	0.363059	0.475233
11741196_SIGLEC14	0.06094	0.363098	0.475272
11747867_TFEB	0.102609	0.363112	0.475278
11723899_DHRS9	-0.11623	0.363128	0.475283
11745148_MID1	0.033029	0.363136	0.475283
11763607_MEI1	-0.06159	0.363152	0.475284
11727706_BBC3	-0.05545	0.363156	0.475284
11758830_SLA	0.040546	0.363196	0.475324
11721902_SERPINI1	0.103954	0.363214	0.475335
11733439_GBP5	-0.06744	0.363272	0.475397
11730981_ZNF595	-0.05183	0.363285	0.475401
11737118_PABPC5	-0.04055	0.363331	0.47545
11753963_LAMP1	-0.03377	0.363354	0.475467
11734340_FAM124B	-0.08882	0.363401	0.475515
11740275_LRRC25	-0.0454	0.36349	0.475619
11747189_BCL11A	-0.06597	0.363529	0.47565
11761736_HDAC8	-0.07646	0.363533	0.47565
11739464_SUSD4	-0.06347	0.363568	0.475683
11754685_RGS14	-0.03578	0.363636	0.475758
11740381_OR1G1	-0.05795	0.363673	0.475795
11753504_ACTA1	-0.05342	0.363686	0.475798
11752455_ARHGEF10L	0.033257	0.363695	0.475798
11763827_MS4A8	0.081612	0.363714	0.47581
11754715_STK31	0.058126	0.363828	0.475947
11750912_SP8	-0.07964	0.363895	0.476022
11726396_ANOS1	-0.09114	0.363936	0.476057
11763393_RNF207	-0.09654	0.363941	0.476057
11742129_CD82	0.048327	0.363986	0.476103
11734807_DTNA	0.119629	0.364067	0.476174
200058_PM SNRNP200	0.032957	0.364069	0.476174
11763851_CRHBP	0.053591	0.364073	0.476174
11722769_MMP24	0.050576	0.36408	0.476174
11734283_PDILT	0.067694	0.36411	0.476201
11761843_TTLL9	0.106624	0.364145	0.476234
11725815_SLC30A8	-0.06357	0.364356	0.476497
11737208_MACROD2	-0.07681	0.364419	0.476555
11760139_C9orf3	-0.0804	0.36442	0.476555
11733728_LGMN	-0.04769	0.364445	0.476576
11726202_BHLHB9	-0.05397	0.364462	0.476584
11746078_NARFL	-0.09948	0.364474	0.476588
11747559_PDZRN3	-0.0427	0.364566	0.476692
11723784_LIMCH1	-0.05874	0.364573	0.476692
11752699_ARHGEF4	0.038077	0.364611	0.476729
11738047_ELOVL7	-0.06327	0.364646	0.476762
11715946_RALB	0.039645	0.364988	0.477196
11745253_PITRM1	0.044343	0.365031	0.477239

11754394_SYNJ2	-0.03702	0.365088	0.477301
11734953_MBNL3	0.108186	0.365098	0.477301
11741274_KCNAB1	-0.0665	0.365173	0.477378
11747916_PI4KB	-0.03777	0.365176	0.477378
11735024_ZBTB7B	-0.04978	0.36529	0.477514
11721059_ZNF189	-0.05194	0.365326	0.477548
11735655_ATOH7	-0.05019	0.365405	0.477639
11755635_CDK11A //	0.039817	0.365418	0.477642
11747186_PTGES	0.045197	0.365435	0.477653
11737162_FAM83B	0.059689	0.365455	0.477666
11762590_OR4D2	-0.07463	0.365658	0.477916
11715410_PRDX2	0.033038	0.365666	0.477916
11763306_FCRL5	-0.05413	0.365686	0.477929
11729783_BOLL	-0.04404	0.3657	0.477935
11736273_TLR2	-0.06064	0.3659	0.478155
11734345_TRIM10	0.065881	0.365907	0.478155
11758919_TFCP2	-0.05176	0.365911	0.478155
11717918_SRRT	0.041218	0.365913	0.478155
11730015_NEUROD2	-0.07542	0.365917	0.478155
11729690_PGLYRP2	-0.06576	0.365968	0.478208
11741519_CD14	-0.05303	0.366001	0.478239
11716263_LEPROTL1	-0.03724	0.36604	0.47827
11727340_ZBTB2	0.052895	0.366045	0.47827
11748235_MMRN1	-0.06156	0.366063	0.478282
11758063_DCLK2	-0.03457	0.366095	0.47831
11759852_IGHG1 ///	-0.11352	0.366114	0.478322
11734357_ARHGEF33	-0.06489	0.366148	0.478354
11742343_OR4K5	-0.03658	0.366253	0.478478
11755374_HERC5	-0.0315	0.366266	0.478483
11741048_KCNK2	-0.06903	0.366322	0.478543
11740447_BTN3A1	0.075147	0.366455	0.478705
11733392_UBL4B	-0.06971	0.366472	0.478714
11724255_OAS1	-0.05944	0.366622	0.478897
11736616_AVPR1A	-0.09423	0.366645	0.478914
11740856_GYG2	0.090784	0.366681	0.478933
11740916_PCDHA9	0.033937	0.366687	0.478933
11716893_TOX4	0.03449	0.366689	0.478933
11758502_EHF	0.061913	0.366827	0.4791
11718994_CRKL	0.056187	0.36685	0.479118
11762005_ECE2	-0.10371	0.366909	0.479181
11717841_ETNK1	-0.05502	0.366922	0.479186
11742102_DPF1	-0.05666	0.366956	0.479218
11751432_ADAP1 ///	0.061122	0.367069	0.479346
11764028_LOC105375	-0.08338	0.367074	0.479346
11760184_KIZ	-0.06701	0.367083	0.479346
11763893_SNORA59A	0.076468	0.367097	0.47935
11757003_RPL6	-0.03759	0.36715	0.479407
11749468_TRIM24	0.034215	0.367204	0.479464
11736844_CEP44	-0.07398	0.367238	0.479489
11745920_CSH1 ///	0.092138	0.367242	0.479489
11745579_TTTY14	-0.05843	0.367296	0.479547

11730238_ABHD10	-0.03704	0.367362	0.47961
11747032_SLC52A3	-0.03792	0.367364	0.47961
11741467_LHX5	-0.0469	0.367422	0.47966
11725588_CBLC	-0.04458	0.36744	0.47966
11731106_NR5A1	-0.04414	0.367463	0.47966
11762226_CYTH4	0.064905	0.367468	0.47966
11763873_LOC100128	-0.06339	0.367475	0.47966
11723622_PHYHIP	-0.03417	0.367477	0.47966
11721042_NSMCE2	0.03582	0.367479	0.47966
11727149_CCDC87	-0.11145	0.367481	0.47966
11718934_C4A /// S	0.037	0.367491	0.47966
11747618_ADCK3	-0.06086	0.367503	0.479663
11735145_YBEY	-0.07598	0.367562	0.479727
11728618_SLC1A7	-0.06644	0.367607	0.479773
11731780_GSTA3	0.044144	0.367716	0.479899
11744101_NAPA	-0.04837	0.367723	0.479899
11718982_CCL4 ///	0.094405	0.367753	0.479926
11760844_RPN2	0.036398	0.367787	0.479957
11762543_LRP1	-0.08945	0.367865	0.480046
11762461_SEPT5-GP1	-0.05235	0.367978	0.480177
11745490_GOLGA8CP	-0.07164	0.367985	0.480177
11717904_STAU1	0.042205	0.368002	0.480185
11735087_SLFN13	0.051801	0.368057	0.480245
11736288_TAF8	0.058284	0.368139	0.480339
11726398_SPTB	-0.03469	0.36821	0.480419
11749305_ETNK2	-0.0398	0.368274	0.480489
11717725_RAB1A	0.034856	0.368373	0.480606
11755829_ATP13A4	0.07409	0.368485	0.480739
11738702_OR11H4	-0.06801	0.368549	0.48081
11740912_BANK1	-0.05663	0.368588	0.480847
11737931_STMN4	-0.03596	0.368603	0.480854
11759742_TSHZ2	-0.06576	0.368665	0.480915
11757130_RPS3 ///	0.044469	0.368669	0.480915
11751147_MOCS1	-0.05391	0.368692	0.480932
11728831_RAB3IP	-0.04302	0.36882	0.481087
11743385_PRPF40A	-0.03579	0.368842	0.481103
11719858_ARID1A	-0.03529	0.368898	0.481162
11723375_GLI3	0.049141	0.368982	0.481259
11750053_SLC37A2	0.040317	0.369058	0.481346
11755558_DRC7	-0.05347	0.36907	0.481348
11757744_FAM171A1	-0.04008	0.369182	0.481482
11740505_GPR62	-0.05105	0.3692	0.481492
11729050_SLC10A2	-0.05681	0.369277	0.481579
11761506_ME3	0.046221	0.369407	0.481736
11757028_RPS5	0.035483	0.369482	0.481811
11750481_PPBPP2	-0.05021	0.369484	0.481811
11761407_C6orf201	-0.04574	0.369553	0.481888
11756629_MTHFSD	-0.05556	0.369607	0.481945
11760775_RNF220	0.04195	0.369634	0.481968
11755905_ST8SIA5	-0.13961	0.369667	0.481998
11727631_APBA2	0.043277	0.369765	0.482113

11731030_CCDC64B	-0.06093	0.369842	0.4822
11728120_KHDC1	-0.06452	0.369934	0.482308
11752088_MYO5C	0.070309	0.369993	0.482372
11741230_TMEM169	0.100749	0.37001	0.482381
11760778_AP1S2	0.075037	0.370027	0.48239
11762586_IL17RE	-0.06355	0.370059	0.482419
11753330_NAP1L1	0.045018	0.370098	0.482449
11723549_UPF3B	0.037497	0.370102	0.482449
11760969_IP6K2	-0.05878	0.370159	0.482511
11743460_GPM6B	0.092637	0.370199	0.482551
11727422_CANX	-0.03501	0.370352	0.482736
11715409_PRDX2	0.035567	0.370367	0.482743
11720764_TOR1A	0.043914	0.370425	0.482807
11723943_PTTG1IP	0.044908	0.37046	0.482839
11763843_UACA	-0.03406	0.370619	0.483034
11747714_ABCA8	-0.08751	0.370643	0.483051
11717885_CCDC3	-0.06925	0.370704	0.483119
11761649_ACSL6	-0.09992	0.370828	0.483267
11740584_PACRGL	-0.03825	0.37085	0.483283
11732054_CLEC4F	-0.07933	0.37093	0.483375
11741639_DIO2	0.034568	0.371001	0.483454
11761729_ZNF765	-0.10923	0.371071	0.483526
11750932_EPHX2	-0.1072	0.371076	0.483526
11724020_C21orf2	-0.07596	0.371101	0.483546
11740723_LGALS9B /	-0.06906	0.37114	0.483575
11731300_TBX6	0.060519	0.371149	0.483575
11727106_BAD	-0.04859	0.371153	0.483575
11734882_ARID2	0.035398	0.37117	0.483584
11726658_ADORA1	-0.05409	0.37122	0.483636
11758522_CREM	0.051306	0.371282	0.483702
11757604_SAMM50	-0.03396	0.371306	0.483702
11761204_TM7SF2	-0.04998	0.371307	0.483702
11724670_LRTM1	-0.0494	0.37131	0.483702
11747822_KLHL21	-0.04185	0.371354	0.483744
11728496_GNRH1	-0.07549	0.371362	0.483744
11731345_UFSP1	-0.03833	0.37141	0.48378
11750813_RIPK4	-0.08622	0.37141	0.48378
11763614_FMN1	0.051313	0.371485	0.483866
11755021_MAP3K14-A	0.034307	0.371496	0.483867
11753959_PROCA1	-0.06653	0.371546	0.483919
11734041_ANKRD9	-0.05132	0.371573	0.483928
11749700_ZNF503	-0.05692	0.371573	0.483928
11745474_CTCFL	0.109454	0.371607	0.48396
11754639_GAS8	0.050956	0.371728	0.484105
11715124_CACNG8	-0.04354	0.371809	0.484196
11760309_SCYL1	0.047743	0.371819	0.484197
11741472_RAB11FIP3	-0.03487	0.371829	0.484197
11740269_TTLL11	-0.05373	0.371912	0.484292
11726439_MALT1	0.034398	0.371933	0.484307
11715986_YPEL5	0.040342	0.371951	0.484317
11764125_LINC00700	0.048075	0.371973	0.484333

11748798_PIK3AP1	0.083187	0.372038	0.484405
11754396_MLLT3	-0.06818	0.37207	0.484434
11745170_DIAPH1	-0.03407	0.37212	0.484463
11754123_HADHA	0.036088	0.372121	0.484463
11716568_PRRC2C	-0.03831	0.372122	0.484463
11728224_UBE2J1	0.046293	0.372152	0.484488
11750048_LILRB5	-0.07796	0.372271	0.484617
11734513_LRRC18	0.07594	0.372271	0.484617
11724591_TCP10L	0.066727	0.372323	0.484672
11736641_MECR	0.074889	0.37236	0.484708
11750273_ZPLD1	0.05856	0.372401	0.484718
11735768_C16orf54	-0.03693	0.372408	0.484718
11740595_EDDM3B	0.101376	0.372409	0.484718
11763398_LINC01013	0.092332	0.372411	0.484718
11732006_ADAL	0.058722	0.372417	0.484718
11754703_SLC22A16	-0.04242	0.372436	0.484729
11728856_SCRT1	-0.03891	0.372461	0.484749
11739683_PHF2	-0.04996	0.37253	0.484825
11739794_LRFN2	-0.04898	0.372555	0.484846
11738960_CARD17	-0.05924	0.372572	0.484852
11740141_EPB41L1	0.035389	0.37259	0.484852
11732715_SLC45A2	-0.03365	0.372591	0.484852
11729454_CYP2B6	-0.12963	0.3726	0.484852
11762314_C11orf74	0.051379	0.372659	0.484916
11750033_ITGAX	-0.03548	0.372671	0.484919
11731518_MFAP3	0.032159	0.372697	0.484939
11738422_PRAMEF2	-0.05054	0.372724	0.484962
11752138_WDR7	0.052578	0.372771	0.48501
11721268_TMPRSS4	0.048706	0.372825	0.485059
11762959_FAM198A /	-0.08301	0.372828	0.485059
11722390_MTF1	0.04863	0.372904	0.485136
11746810_KCTD3	-0.04547	0.372908	0.485136
11757610_MPZL1	-0.0752	0.372952	0.485182
11734977_PCDHB12	-0.07514	0.373054	0.485301
11759453_OR1E1	-0.07985	0.373074	0.485314
11745581_GRM7	0.059893	0.373091	0.485324
11760977_TYMP	0.049575	0.373201	0.485453
11726903_TRIL	-0.07463	0.373294	0.485551
11746030_SPATA33	-0.042	0.373296	0.485551
11741412_LHX9	0.095851	0.373368	0.485628
11733458_INTS2	0.03636	0.373375	0.485628
11724753_TMF1	0.041051	0.373456	0.485721
11763207_SH3RF2	-0.06258	0.373502	0.485767
11726519_TINAG	0.067699	0.373625	0.485914
11743559_PTPRC	0.10371	0.373683	0.485977
11761243_CARKD	-0.08259	0.373747	0.486047
11730567_MROH7	-0.08489	0.373798	0.486101
11750347_HOOK1	-0.06919	0.373819	0.486115
11748796_PXN	0.080875	0.373835	0.486123
11756065_APOD	0.045719	0.373944	0.486252
11744878_ITIH4	-0.0904	0.373976	0.48628

11740326_SORBS2	0.048253	0.37403	0.486331
11757222_HINT1	0.053988	0.374035	0.486331
11751712_ULBP3	0.069031	0.374058	0.486348
11740194_ATP6V0B	-0.03928	0.374149	0.486445
11750669_ZCCHC11	0.037563	0.374152	0.486445
11755438_PSG3	-0.08463	0.374227	0.486529
11729511_HOXB8	0.06555	0.374301	0.486613
11759896_OFD1	-0.12052	0.374323	0.486628
11719595_GMEB2	0.048444	0.374334	0.486629
11743818_CILP	-0.0534	0.374435	0.486747
11748772_POTEG ///	0.039188	0.374449	0.486753
11743223_SSTR5	0.050905	0.374513	0.486809
11715793_ACTG2	0.079172	0.374529	0.486809
11716259_RHEB	0.044401	0.374535	0.486809
11729115_PRKCG	-0.04496	0.374539	0.486809
11742417_ZNF737	0.058271	0.374542	0.486809
11753668_CRIP3	-0.05364	0.374563	0.486823
11754026_CXCL8	0.032369	0.374605	0.486861
11732952_TCTEX1D4	-0.04778	0.374612	0.486861
11724678_LY86	-0.0453	0.374743	0.487018
11747983_ICE2	-0.04322	0.374765	0.487022
11753742_PAIP2	0.038344	0.374766	0.487022
11762152_MRPL9	0.038425	0.374873	0.487148
11729612_LRTM2	0.072404	0.374884	0.48715
11716513_MOCS2	-0.04097	0.374995	0.487281
11752309_GRHL1	0.067858	0.375043	0.48733
11728926_NFAM1	0.10023	0.375061	0.487341
11759691_SLC11A2	-0.04969	0.375198	0.487498
11763665_CA6	-0.0621	0.375202	0.487498
11735082_GALNT13	0.137748	0.375224	0.487514
11762276_GINM1	-0.05516	0.375251	0.487536
11759117_FBXO42	-0.05206	0.375263	0.487538
11737685_OR13A1	-0.0621	0.375347	0.487635
11717569_HTN1	-0.07255	0.375369	0.48765
11730605_CNNM1	-0.10441	0.375412	0.487693
11716004_FAF2	0.057444	0.375536	0.487841
11734905_NOVA1	-0.06132	0.375556	0.487854
11748102_RUNX1T1	0.068703	0.375598	0.487885
11735693_SPRR2E	-0.05255	0.375599	0.487885
11721831_CHURC1	0.061855	0.375682	0.48798
11747899_CPLX2	-0.03531	0.375779	0.488092
11761088_RIPK3	-0.04451	0.375829	0.488141
11720691_DIRAS1	-0.04444	0.375837	0.488141
11738956_XAGE5	-0.0359	0.375874	0.488177
11733786_EVC	0.054785	0.375972	0.488291
11728860_RPL10L	-0.07546	0.375987	0.488298
11728437_BRWD1	-0.08434	0.376185	0.488542
11751148_STRN4	0.044351	0.376249	0.488612
11747715_SULT1C4	0.07047	0.376318	0.488688
11743307_COPS4	0.033656	0.376526	0.488945
11724826_BORCS6	-0.05831	0.376625	0.489061

11762852_FCRL2	0.054027	0.376702	0.489149
11736191_OGN	-0.05302	0.376741	0.489186
11754217_ACTG1	0.031037	0.376879	0.489352
11726221_SYPL2	-0.06983	0.377003	0.4895
11729533_REM2	0.045247	0.377021	0.4895
11746165_R3HDM2	-0.04264	0.377023	0.4895
11735747_PSG7	-0.07162	0.377059	0.489533
11734151_ABRA	0.080519	0.377082	0.489544
11716464_SIAE	0.033166	0.377087	0.489544
11735265_CLEC4D	-0.0813	0.377124	0.489571
11729019_SPRY3	-0.0591	0.377128	0.489571
11726812_TFF1	-0.07975	0.377216	0.489673
11757186_HK3	-0.04128	0.37728	0.489742
11742630_OR8K5	-0.08707	0.377334	0.489799
11715224_ACOXL	-0.04961	0.377364	0.489807
11742617_OR5H14	0.091596	0.37737	0.489807
11730985_SBSPON	-0.06141	0.377372	0.489807
11746088_IFI44	-0.05964	0.377379	0.489807
11738135_LRRC31	-0.10357	0.377419	0.489838
11729266_SPRR2D	-0.06376	0.377423	0.489838
11736189_ORMDL3	0.044682	0.377455	0.489866
11724496_NPRL3	-0.05102	0.377472	0.489875
11739906_CLDN16	0.059701	0.377503	0.489902
11759330_B3GAT2	0.089297	0.377549	0.489949
11755585_AZI2	-0.04511	0.377623	0.49003
11730317_RGS7BP	-0.08121	0.377632	0.49003
11752152_OR2C3	0.078538	0.377751	0.490166
11732109_FGF10	0.055582	0.377756	0.490166
11727857_NUP50	-0.03254	0.377801	0.49021
11741097_WBP1L	0.092686	0.377811	0.49021
11762027_CCDC40	0.097698	0.377821	0.49021
11751691_TMEM214	-0.03193	0.377858	0.490245
11737610_GRIN3B	0.060989	0.377962	0.490368
11760425_IL6	-0.04834	0.378007	0.490413
11747695_DLGAP1	-0.04016	0.378064	0.490465
11752774_RGS6	-0.08373	0.378067	0.490465
11745628_GPR6	-0.0866	0.378081	0.490469
11721183_PLPPR4	-0.06865	0.378109	0.490493
11736463_LILRA2	-0.05607	0.378174	0.490565
11759496_CHST11	0.093505	0.378236	0.490631
11749570_LINC01126	0.060063	0.378245	0.490631
11723048_CX3CR1	0.055651	0.378429	0.490855
11717784_THYN1	-0.04415	0.378504	0.49094
11762352_MT1IP	-0.08057	0.378555	0.490981
11761396_EIF4A1	0.037128	0.378556	0.490981
11744388_ARFRP1	-0.07706	0.378628	0.49106
11723307_ACACA	0.043198	0.378637	0.49106
11731256_HYPM	-0.07	0.378684	0.491108
11732559_RBM12B	-0.03371	0.378733	0.491159
11742697_ITM2A	0.050944	0.378902	0.491365
11747050_GPR137	-0.0375	0.378916	0.49137

11762659_MPP4	0.080517	0.379012	0.491482
11757015_ODF3L2	-0.07761	0.379027	0.491488
11758923_LMO2	-0.09147	0.379151	0.491636
11744039_TMEM8B	-0.06848	0.379195	0.49168
11730746_PAIP2	0.036213	0.379246	0.491733
11742433_ABCC9	-0.08602	0.379275	0.491757
11751975_SGIP1	-0.0446	0.379343	0.491832
11754143_EIF1	0.03287	0.379377	0.491863
11744076_OLAH	0.043855	0.379401	0.491875
11748879_TNFRSF13B	-0.0508	0.379407	0.491875
11755893_PIGN	0.040397	0.379416	0.491875
11752479_GPC3	-0.05565	0.379426	0.491875
11733726_SNAP25	0.031527	0.379442	0.491883
11763655_VPS36	0.091474	0.379611	0.492089
11720417_CAMSAP1	-0.10984	0.379675	0.492159
11724182_ATCAY	-0.05972	0.379724	0.492209
11738802_EYS	0.134221	0.379777	0.492265
11751386_RPE	-0.0384	0.379792	0.492272
11744509_RER1	0.032452	0.379876	0.492368
11737262_CABP5	-0.07964	0.379906	0.492393
11763127_IGKC	-0.04225	0.380011	0.492516
11741052_HMGCLL1	0.037098	0.380082	0.492593
11745945_CHPF	-0.04086	0.380091	0.492593
11715917_CD44	0.03786	0.380136	0.492638
11741886_FAM47A	-0.06996	0.380151	0.492645
11744659_CASC1	-0.10006	0.380205	0.492702
11742515_OR51T1	0.044291	0.380252	0.492737
11732349_LILRA6 //	-0.05275	0.380253	0.492737
11749084_VWCE	-0.06392	0.380388	0.492899
11730194_SEC14L2	0.035102	0.380439	0.492952
11761641_OGFOD2	-0.08703	0.38056	0.493096
11721980_SLC36A1	0.052253	0.380626	0.493169
11755732_SAMD3	-0.0893	0.380712	0.493268
11750971_CACTIN	0.069742	0.380776	0.493338
11761110_RBCK1	-0.04759	0.380791	0.493343
11739833_HSPE1-MOB	0.070195	0.380855	0.493413
11741767_DNAH12	0.042531	0.380916	0.493479
11748799_POU2F2	0.05691	0.380955	0.493517
11753973_MUC1	0.055784	0.381069	0.493651
11726466_C20orf141	0.069872	0.381084	0.493658
11737817_CDKL5	-0.07568	0.381096	0.49366
11725019_MST1R	0.047961	0.381244	0.493816
11761931_MTMR1	0.04639	0.381245	0.493816
11736300_ORMDL1	-0.07987	0.381246	0.493816
11760528_RDM1	-0.07535	0.381281	0.493847
11753887_TOMM5	0.030969	0.381312	0.493875
11734291_ADAMTS17	0.056092	0.381349	0.49391
11721009_MED1	0.03549	0.381366	0.493918
11756273_RBM12B	0.058658	0.381383	0.493928
11745955_CLSTN1	0.033114	0.381428	0.493972
11728042_PANX2	-0.04932	0.381479	0.494012

11742706_RAB8A	-0.03592	0.381484	0.494012
11751538_AGGF1	-0.0435	0.381489	0.494012
11741485_SLITRK3	-0.04776	0.381588	0.494127
11738559_ABHD12B	-0.03717	0.381621	0.494157
11735847_CPA5	-0.0662	0.381692	0.494224
11757606_ECHS1	-0.03321	0.381695	0.494224
11721836_TMEM14B	-0.03927	0.381703	0.494224
11749519_VPS45	-0.03779	0.381796	0.49432
11738151_OPRM1	0.059725	0.381798	0.49432
11763919_SNORA19	-0.0686	0.381817	0.494332
11761344_NDST3	0.068371	0.381905	0.494433
11759441_C16orf72	-0.07703	0.381937	0.494462
11742876_SGSM3	-0.04962	0.38199	0.494517
11741993_R3HDML	-0.0952	0.382005	0.494524
11759627_USP15	-0.05981	0.382066	0.494588
11739051_SLCO2B1	-0.05824	0.382119	0.494645
11746757_KY	-0.07804	0.38219	0.494724
11737007_LRRC55	0.04505	0.382362	0.494933
11760875_RARG	-0.05684	0.382418	0.494993
11727917_ACTL7B	0.054014	0.382531	0.49512
200005_PMEIF3D	-0.03303	0.382544	0.49512
11719119_NUPL2	0.034653	0.382547	0.49512
11738301_KRTAP3-1	-0.05688	0.382642	0.495229
11738323_OR5AP2	0.067021	0.382693	0.495283
11748181_SULT4A1	-0.06497	0.382716	0.495299
11759745_HLA-DQB1	-0.05289	0.382729	0.495303
11730008_RETN	-0.04505	0.38283	0.49542
11763338_BOD1L2	-0.0396	0.382914	0.495516
11727840_HPD	0.079206	0.382925	0.495518
11722456_C3orf58	0.043039	0.382949	0.495535
11746478_PRKAR2A	-0.06484	0.383026	0.495605
11739202_ATP5G3	-0.07339	0.383032	0.495605
11748117_FDXR	0.038791	0.383035	0.495605
11744684_CCNL2	0.047413	0.383044	0.495605
11740053_CENPP	-0.06255	0.383063	0.495617
11727067_SLIT3	-0.10391	0.38309	0.495639
11753069_PFKFB2	-0.03803	0.383128	0.495675
11729712_SPRR2A	-0.09522	0.383156	0.495698
11716175_P4HB	0.034309	0.383201	0.495743
11752892_LILRA2	-0.0622	0.383218	0.495752
11728785_BRF1	-0.03797	0.383295	0.495838
11719480_CSTA	0.03554	0.383389	0.495947
11737363_RPRML	-0.0843	0.383619	0.496231
11748044_SCEL	-0.05726	0.383655	0.496265
11737333_SIM1	-0.07905	0.383756	0.496382
11742427_STRC ///	-0.06214	0.383766	0.496382
11755537_HBG1 ///	-0.03608	0.383802	0.496415
11755776_C9orf84	0.051885	0.383836	0.496446
11752278_PABPC1	0.034033	0.383896	0.496511
11730397_DDI1	-0.07766	0.383964	0.496586
11721378_FBXL7	0.057508	0.384064	0.496702

11747604_WDR97	-0.03649	0.384082	0.496712
11746490_TRIM50	-0.04135	0.384114	0.49674
11727498_ZRANB1	-0.04655	0.384251	0.496904
11729339_ZNF771	-0.04382	0.384264	0.496908
11744748_FGFR1OP2	-0.03894	0.384315	0.49696
11723413_CYP17A1	-0.07489	0.384341	0.496981
11740217_LMOD3	0.08909	0.384375	0.497
11733489_ZNF704	0.039933	0.384376	0.497
11760771_PRLR	-0.06009	0.384543	0.497201
11747258_LOC100288	0.035103	0.384551	0.497201
11739626_ZNF740	-0.04634	0.384579	0.497223
11752952_GRAP2	-0.03635	0.384621	0.497264
11745791_LOC100288	0.076274	0.384672	0.497317
11749148_PEX5L	-0.10402	0.38469	0.497327
11717303_PDAP1	-0.0311	0.384746	0.497386
11753837_DEFB109P1	-0.03837	0.384789	0.497428
11737600_GDPD1	0.046954	0.384843	0.497486
11762324_RPL3	-0.06538	0.38488	0.49752
11756734_SPATA13	-0.05577	0.384917	0.497555
11756867_TNFSF13B	0.072958	0.384961	0.497599
11746922_TNNT3	0.063505	0.384976	0.497605
11739803_RASAL2	-0.05476	0.385105	0.497758
11749351_ABCA7	-0.05038	0.385274	0.497957
11752339_ADGRF4	-0.13461	0.385279	0.497957
11732696_DCST2	0.043033	0.385291	0.497959
11755108_LVRN	-0.07539	0.38532	0.49798
11720436_CLCN6	-0.08495	0.385331	0.49798
11762021_DYNC1I1	0.085273	0.385338	0.49798
11760889_LOC220729	-0.11281	0.385422	0.498076
11750909_SLC16A7	0.040485	0.385487	0.498146
11726751_MAP6D1	-0.04936	0.385502	0.498152
11728306_GULP1	-0.03571	0.385529	0.498174
11754893_KCTD19	0.058018	0.385539	0.498175
11725674_HINT3	0.032927	0.385583	0.498205
11749678_FAM120B	0.068834	0.385583	0.498205
11754750_NLGN4X	0.046286	0.385737	0.498391
11727677_BLOC1S3	0.038481	0.385777	0.498421
11732595_FAM47C	-0.06791	0.385781	0.498421
11715291_TBX5	-0.05505	0.385797	0.498424
11718667_ENY2	-0.03341	0.385804	0.498424
11757269_FTL	0.030524	0.385882	0.498511
11733224_ZNF71	0.047402	0.385891	0.498511
11749897_FMOD	-0.0828	0.385946	0.498569
11723818_PM20D2	0.058027	0.386077	0.498724
11760914_TAF1A	-0.04294	0.386157	0.498793
11729265_SPRR2D	0.051802	0.386169	0.498793
11741601_EDN3	-0.06715	0.386173	0.498793
11730700_C11orf49	0.051887	0.386181	0.498793
11761395_NINJ2	-0.03816	0.386187	0.498793
11723675_CASQ1	0.139029	0.386191	0.498793
11726896_IRAK3	0.0519	0.386233	0.498833

11747660_DMTN	-0.03752	0.386281	0.498882
11738029_SERINC4	-0.0504	0.386308	0.498904
11732028_AVP	-0.06925	0.386348	0.498943
11752526_GLRA2	-0.04408	0.386363	0.498948
11751486_CPNE6	-0.08601	0.386423	0.499013
11740845_ZNF662	-0.11589	0.386435	0.499015
11748384_TRIP10	-0.0381	0.386549	0.499149
11733678_C4orf50	-0.07154	0.386582	0.499178
11741853_P2RX2	0.054128	0.386594	0.499182
11759267_OR2L3	-0.12671	0.386631	0.499215
11726777_LAT	0.049613	0.386671	0.499254
11723492_ARHGEF3	-0.03442	0.386684	0.499258
11753592_EEF1G	0.030654	0.386699	0.499261
11736945_HIPK4	-0.04326	0.386707	0.499261
11744229_RASL11B	0.062314	0.386838	0.499417
11732217_BID	0.03798	0.38693	0.499523
11721475_GPM6B	0.054122	0.386947	0.499532
11760651_DLX5	-0.0608	0.386963	0.499539
11738998_ZDHHC9	0.062834	0.38701	0.499586
11744278_APBB1	-0.03679	0.387025	0.499593
11720080_NTRK2	0.038025	0.387098	0.499673
11725384_C15orf39	0.065579	0.387115	0.499681
11717890_GNAI2	-0.03079	0.387134	0.499681
11737432_PAPL	0.075378	0.387134	0.499681
11718242_PRUNE2	0.070837	0.38722	0.499778
11722395_RWDD4	-0.0356	0.387238	0.499788
11731389_DRICH1	-0.09548	0.387284	0.499834
11731817_RAB17	0.039971	0.387338	0.49989
11761244_CHRD	-0.06255	0.387391	0.499946
11755508_SFTPB	-0.03271	0.387476	0.500033
11731588_UGT2B4	-0.0696	0.387479	0.500033
11719805_ASPN	0.070191	0.387525	0.500079
11742178_KRTAP12-4	-0.04867	0.387695	0.500285
11731329_GNRH2	-0.03152	0.387807	0.500417
11760449_TXLNGY	-0.08768	0.387884	0.500504
11730979_AFM	0.095915	0.387928	0.500541
11746676_REXO1	0.033361	0.387934	0.500541
11760351_UBXN11	0.042954	0.38796	0.500562
[truncated: 369,922 more chars]
